# Reward sensitivity and internalizing symptoms during the transition to puberty: An examination of 9-and 10-year-olds in the ABCD Study

**DOI:** 10.1016/j.dcn.2022.101172

**Published:** 2022-10-31

**Authors:** Elizabeth A. McNeilly, Natalie M. Saragosa-Harris, Kathryn L. Mills, Ronald E. Dahl, Lucía Magis-Weinberg

**Affiliations:** a Department of Psychology, University of Oregon, USA; b Department of Psychology, University of California, Los Angeles, USA; c PROMENTA Research Center, Department of Psychology, University of Oslo, Norway; d Institute of Human Development, University of California, Berkeley, USA; e School of Public Health, University of California, Berkeley, USA; f Department of Psychology, University of Washington, USA

**Keywords:** Puberty, Internalizing, Reward, Split-half analysis, ABCD Study

## Abstract

Early pubertal timing has been linked to increased risk for internalizing psychopathology in adolescents. Work in older adolescents and adults suggests that heightened reward sensitivity may buffer risk for internalizing symptoms. However, few studies have investigated these associations during the early transition to puberty, a window of vulnerability to mental health risk. In this preregistered study, we investigated the associations among pubertal timing, internalizing symptoms, and reward sensitivity in a large, population-based sample of 11,224 9–10 year-olds from the ABCD Study®. Using split-half analysis, we tested for within-sample replications of hypothesized effects across two age- and sex-matched subsets of the sample. Early pubertal timing was associated with higher internalizing symptoms in female and male participants across samples, with 9–10 year-olds in the mid-pubertal stage at the highest risk for internalizing symptoms. Additionally, early pubertal timing was robustly associated with greater self-reported reward sensitivity in both female and male participants. We observed inconsistent evidence for a moderating role of reward sensitivity across measurement domains (self-report, behavioral, and fMRI data), several of which differed by sex, but none of these interactions replicated across samples. Together, these findings provide unique insights into early indicators of risk for internalizing psychopathology during the transition to puberty in a large, population-based, demographically diverse sample of youth.

## 1 Introduction

The onset of puberty is a hallmark of early adolescence accompanied by cascading changes in hormonal, neurobiological, and psychological processes (Pfeifer and Allen, 2021). Although the onset of puberty marks an expected milestone of development, it is also a stage characterized by heightened risk for internalizing disorders, including anxiety, depression, and non-suicidal self-injury (Patton et al., 2007, Patton et al., 2008, Pfeifer and Allen, 2021, Ullsperger and Nikolas, 2017). In particular, risk for future internalizing symptoms appears to be heightened in individuals who experience pubertal maturation earlier than their same-age and same-sex peers (i.e., early pubertal timing; Graber, 2013; Mendle and Ferrero, 2012; Mendle et al., 2007; Ullsperger and Nikolas, 2017). Despite this elevated risk, not all early maturing adolescents go on to develop internalizing disorders. It is therefore crucial to identify factors that modulate risk for internalizing psychopathology during this transitional stage (Mendle, 2014), especially amongst “early maturers” who are posited to be at highest risk (Ullsperger and Nikolas, 2017).

Existing research in older adolescents and adults points to neural and behavioral signatures of reward sensitivity as robust indicators of risk for internalizing disorders. Reduced self-reported and task-measured reward sensitivity predict heightened internalizing symptoms (Alloy et al., 2016, Taubitz et al., 2015, Whitton et al., 2015), patterns that are mirrored by neuroimaging data (Luking et al., 2016). In particular, heightened internalizing symptoms have been associated with attenuated activity within the ventral and dorsal striatum (i.e., nucleus accumbens, caudate, and putamen) during reward anticipation and feedback (Auerbach et al., 2021, Forbes, 2011, Forbes et al., 2009, Forbes et al., 2010, Luking et al., 2016, Morgan et al., 2013, Nielson et al., 2021, Pizzagalli et al., 2009, Rappaport et al., 2020, Zhang et al., 2013). Similarly, reward-related activity within the orbitofrontal cortex (OFC) has been linked to negative emotion biases evident in internalizing disorders (Dichter et al., 2012, Downar, 2019, McCabe et al., 2012, Rolls, 2016, Rolls, 2019, Ubl et al., 2015). Together, these findings suggest that reduced reward-related functional activity within orbitofrontal and striatal circuits may heighten risk for internalizing symptoms in older adolescents and adults (Luking et al., 2016). However, relatively little is known about the relationship between reward sensitivity and internalizing symptoms during the transition to puberty. Moreover, it is possible that this putative risk factor interacts with pubertal timing to heighten risk. That is, adolescents who experience both early pubertal maturation and lower reward sensitivity may be at highest risk, while those with greater reward sensitivity during this transitional stage may have reduced risk. However, to our knowledge, no prior study has examined how early pubertal maturation interacts with reward sensitivity in the association with internalizing symptoms.

When considering the potential modulating role of reward sensitivity in pubertal-related risk for internalizing, it is important to take into account the possibility that reward sensitivity varies with pubertal development (Steinberg, 2008). For instance, individual differences in concentrations of testosterone, a circulating gonadal hormone that increases in both female and male adolescents during puberty (Braams et al., 2015, Wierenga et al., 2018), have been shown to predict differences in neural signatures of reward processing (Forbes et al., 2010, Ladouceur et al., 2019, van Wingen et al., 2011). Although some studies have found that individuals with higher levels of testosterone demonstrate greater responses to reward within the nucleus accumbens (Braams et al., 2015, Op de Macks et al., 2011), not all research demonstrates a positive relation between pubertal maturation and striatal reactivity to rewards (Forbes et al., 2010), especially when pubertal maturation is assessed by measures other than sex hormones (Dai and Scherf, 2019). It is possible that group-level associations between pubertal maturation and reward sensitivity across studies are obscured by vast variability in reward sensitivity across adolescents. It is therefore important to examine how pubertal development — indexed via hormonal as well as non-hormonal measures — relates to reward sensitivity in a large sample of adolescents, and whether these two putative risk factors interact to predict internalizing risk during the crucial early stages of puberty.

Although a large body of work has linked early pubertal timing to heightened risk for internalizing symptoms, many studies have focused on single-sex, homogeneous samples — especially white, female samples of European descent (Cheng et al., 2021, Deardorff et al., 2019). Importantly, however, recent work highlights that early pubertal timing may be linked to risk for internalizing symptoms in male adolescents as well (Hamlat et al., 2019, Mendle and Ferrero, 2012, Ullsperger and Nikolas, 2017), although these results are less consistent (Mendle and Ferrero, 2012, Mendle et al., 2010). In addition to a predominant focus on homogeneous female samples, a notable portion of studies assessing psychosocial risk during puberty have centered on adolescents ages eleven and older (Galvao et al., 2014, Ullsperger and Nikolas, 2017). Given that the hormonal processes that unfold during puberty are typically initiated prior to age eleven (Dorn et al., 2006, Grumbach and Styne, 2003), it is important to incorporate younger samples in order to capture development of “early maturers'' who are thought to be at high risk for internalizing psychopathology (Ullsperger and Nikolas, 2017). Moreover, results from a recent study of 9–14 year-old female adolescents suggests that increases in internalizing symptoms are specifically linked to physical changes that emerge during early compared to later stages of puberty, highlighting the transition to puberty as a unique window of vulnerability (McGuire et al., 2019). While prior research has provided important insights into puberty-related risk for psychopathology, studies that include a larger, more diverse, and younger sample of youth — particularly those entering the early stages of puberty — are needed.

In the present study, we investigated the associations among puberty, reward sensitivity, and internalizing symptoms in a large, population-based sample of 9- and 10-year-olds from the Adolescent Brain Cognitive Development (ABCD) Study® (Jernigan et al., 2018). Baseline data from the ABCD Study are well-suited to address the aforementioned gaps in the literature in several critical ways. First, the narrow age range of the ABCD cohort enables examination of pubertal timing (i.e., pubertal maturation relative to adolescents within one’s age cohort) in particular (Barendse et al., 2021, Mendle et al., 2010). Investigating these associations within a cohort of 9- and 10-year-olds captures variability in the early transitional stages of puberty, a crucial phase for addressing psychosocial risk (McGuire et al., 2019). Second, the ABCD Study assessed internalizing symptoms — which encompass characteristics of both anxiety and depressive disorders — in a large sample of young adolescents, thereby capturing symptoms on a dimensional spectrum and enabling the identification of subclinical vulnerability that may precede a clinical threshold for diagnosis. Given that internalizing is a common higher-order factor across anxiety and depression (Kotov et al., 2017), understanding related vulnerabilities to internalizing symptoms provides a way to capture the full spectrum of risk, including sub-clinical risk, and could inform intervention that targets adolescents at early stages of risk. Third, the richness of the ABCD dataset allows for comprehensive, multimethod examinations of puberty via parent report and salivary hormonal assays, and of reward-related processes via questionnaires, behavioral data, and fMRI measures (Cheng et al., 2021). It is especially important to incorporate multimethod indicators of reward sensitivity given that different measures can have relatively low agreement (Radulescu et al., 2020) and to incorporate both hormonal assays and questionnaire data when investigating pubertal development (Shirtcliff et al., 2009). Lastly, the size of the ABCD sample enables the use of split-half analysis, a data-driven technique that tests for within-study replications across subsamples of the data (Karcher and Barch, 2021).

We tested two primary hypotheses: (1) early pubertal timing would be associated with higher internalizing symptoms in early adolescents, ages 9–10 years, and (2) this association would be heightened in adolescents with attenuated reward sensitivity, measured at multiple levels (i.e., self-report, behavioral, and neural). All hypotheses and analyses were preregistered on Open Science Framework (osf.io/3yrwh).

## 2 Methods

### 2.1 Data and code availability

Data were retrieved from the National Institute of Mental Health Data Archive (NDA) ABCD repository (ABCD Curated Annual Release 3.0, 2020; downloaded in March 2021). A list of all variables of interest, as well as their official (i.e., ABCD-provided) variable names, aliases, and definitions, are available on this study’s OSF page (osf.io/7u3jt). The original ABCD data analyzed in the present study can be accessed via the NDA ABCD repository.

The preregistration for this study is available on Open Science Framework (osf.io/3yrwh). Scripts for downloading, cleaning, splitting, analyzing, and plotting the data are available on GitHub (github.com/nsaragosaharris/abcd_transitiontopuberty_study).

### 2.2 Participants

Recruitment of the ABCD Study cohort took place in 21 catchment areas across the United States using a modified probability sampling method (Garavan et al., 2018). Special attention was dedicated to recruiting a sample that reflected the United States’ sociodemographic variation in terms of sex, race and ethnicity, socioeconomic status, and urbanicity. Informed consent was obtained from all participants and parents according to the ABCD Study protocol (Garavan et al., 2018). Further details on the recruitment methods for the ABCD Study can be found in Garavan et al. (2018).

The ABCD Curated Annual Release 3.0 included baseline data from 11,878 participants ages 8.9–11 years of age (Table 1). The sample was then divided into two subsamples to enable split-half analysis in order to test hypotheses in one half of the dataset (“Sample 1”) and test for replication in the other half of the dataset (“Sample 2”). Participants were randomly assigned to Sample 1 or Sample 2 using the function ‘block_ra’ from the R package randomizr (Uschner et al., 2018), which ensured that the two samples were matched in terms of sex. After excluding participants with missing parent-reported puberty data (see *Perceived Pubertal Development* section for details), Sample 1 included 5625 participants and Sample 2 included 5599 participants. T-tests of the two testing samples revealed no significant differences between the subsamples on the following variables of interest: age, race, CBCL internalizing factor score, testosterone, PDS score, and BIS/BAS reward responsiveness (see *Measures* for variable descriptions; see Supplement for t-test statistics). Descriptive statistics for the two samples can be found in Table 1.

**Table 1:** Descriptive statistics for sample demographics, pubertal development, and questionnaire-based measures for Sample 1 and Sample 2. *M=Mean, SD=Standard Deviation*.

	Female participants		Male participants	
	Sample 1	Sample 2	Sample 1	Sample 2
N	2701	2691	2924	2908
Age	M= 118.87 months (SD=7.51 months) Range= 107–132 months	M= 118.69 months (SD=7.45 months) Range= 107–132 months	M= 119.19 months (SD=7.48 months) Range= 107–133 months	M= 119.11 months (SD=7.53 months) Range= 107–132 months
Race (%)				
Asian	61 (2.3%)	74 (2.7%)	65 (2.2%)	61 (2.1%)
Black	406 (15%)	435 (16.2%)	422 (14.4%)	377 (13%)
Mixed	345 (12.8%)	320 (11.9%)	351 (12%)	347 (11.9%)
White	1723 (63.8%)	1689 (62.8%)	1915 (65.5%)	1932 (66.4%)
Other	130 (4.8%)	133 (4.9%)	139 (4.8%)	151 (5.2%)
Missing	36 (1.3%)	40 (1.5%)	32 (1.1%)	40 (1.4%)
Hispanic/Latina/o/x (%)	546 (20.2%)	525 (19.5%)	578 (19.8%)	595 (20.5%)
PDS Mean Score	M= 1.69 (SD=0.74) Range= 1–4	M= 1.67 (SD=0.73) Range= 1–4	M= 1.35 (SD=0.54) Range= 1–4	M= 1.34 (SD=0.56) Range= 1–4
PDS Stage (%)				
Pre-puberty	818 (30.3%)	850 (31.6%)	2043 (69.0%)	2059 (70.8%)
Early-puberty	654 (24.2%)	619 (23%)	722 (24.7%)	669 (23%)
Mid-puberty	1163 (43.6%)	1151 (42.8%)	145 (5%)	166 (5.7%)
Late-puberty	66 (2.4%)	71 (2.6%)	14 (0.5%)	14 (0.5%)
Testosterone (pg/mL) Average	M= 35.53 (SD=16.4) Range= 1.86–93.69	M= 34.93 (SD=16.35) Range= 3.31–88.16	M= 31.36 (SD=15.54) Range= 1.33–93.96	M= 30.91 (SD=14.8) Range= 0.72–87.87
Body Mass Index (BMI)	M= 18.88 (SD=4.09) Range = 12.03–34.64	M= 18.66 (SD=3.91) Range = 12.13–34.90	M= 18.58 (SD=3.71) Range = 12.11–34.50	M= 18.58 (SD=3.84) Range = 12.04–34.65
BIS/BAS Reward Responsivity (BAS-RR) Score	M= 8.7 (SD=2.34) Range= 2–12	M= 8.69 (SD=2.4) Range= 2–12	M= 8.98 (SD=2.3) Range= 2–12	M= 8.94 (SD=2.31) Range= 2–12
CBCL Internalizing Raw Score	M= 5.03 (SD=5.52) Range= 0–41	M= 5.0 (SD=5.43) Range= 0–51	M= 5.05 (SD=5.52) Range= 0–49	M= 5.16 (SD=5.6) Range= 0–42

## 3 Measures

### 3.1 Demographic variables

Age, sex, race, and ethnicity were assessed via parent report. Age was a continuous variable defined as the age in months at the time of the baseline visit. Questions within the Pubertal Development Scale (PDS), our primary predictor of interest, were administered based on sex at birth (Cheng et al., 2021). To align with this design, within our models, sex was defined as sex at birth (female or male; see *Discussion* for discussion of this limitation). We refer to participants as female participants or male participants throughout. Race was a categorical variable with five levels: Asian, Black, Mixed, White, and Other (Native Hawaiian and other Pacific Islander; American Indian and Alaskan Native). Ethnicity was a categorical variable based on yes/no responses to the question “Do you consider the child Hispanic/Latino/Latina?”. Descriptive statistics for Sample 1 and Sample 2 with respect to this set of demographic variables can be found in Table 1.

### 3.2 Pubertal development

#### 3.2.1 Perceived pubertal development

Perceived pubertal development was measured through the parental report of the Pubertal Development Scale (PDS) (Petersen et al., 1988), which has good internal consistency (Chronbach's *α* = 0.91–0.96) and test-retest reliability (ICC = 0.81–0.93). Only parent-report of perceived pubertal development was used, as parents may be more accurate reporters of their child’s pubertal development particularly at the early stages of pubertal development (Shirtcliff et al., 2009). For further considerations on measuring pubertal processes in the ABCD Study, see Cheng et al. (2021). PDS items include questions about physiological changes in height, body hair, and skin. The female version includes additional questions about breast development and menstruation while the male version includes additional questions regarding voice changes and facial hair growth. Each item has five response options (1 = not yet started, 2 = barely started, 3 = definitely started, 4 = seems complete, or I don’t know/Refuse to answer), except for the menstruation item (female participants only; 1 = no, 4 = yes, or I don’t know/Refuse to answer). Two scores are generated from the PDS: PDS stage and PDS score. PDS stage is determined based on the sum of items and is a categorical value with five levels (Carskadon and Acebo, 1993, Petersen et al., 1988): pre-pubertal, early pubertal, mid-pubertal, late pubertal, and post-pubertal. We used the pre-calculated values for PDS stage from the downloaded 3.0 release data. Individuals who were categorized as post-pubertal were excluded from analysis given the small number in each sample-sex bin (0.4%; *n* = 50 total; 27 female participants and 7 male participants in Sample 1; 15 female participants and 1 male in Sample 2). PDS mean score is a continuous value calculated by averaging across PDS items following Herting et al. (2021). Items on the PDS were considered missing if participants answered “I don’t know” or “refuse to answer”, if the response was left blank, or if the response value was outside of the expected range (1−4). Participants with two or more missing answers were excluded from the PDS score calculation, following recommendations from Herting et al. (2021); 5631 participants in Sample 1 and 5612 participants in Sample 2 met these inclusion criteria. Because pubertal timing can be inferred by pubertal maturation within a narrow age cohort (i.e., “stage-for-age”), wherein higher scores indicate earlier timing relative to one’s age-matched peers (Vijayakumar et al., 2018), here we conceptualized higher PDS scores as indicative of early timing in this young and narrow age group. To further delineate the effects of puberty above and beyond age on our outcomes of interest, we also controlled for age in our statistical analyses.

#### 3.2.2 Testosterone

Given existing work linking testosterone levels to both internalizing symptoms (Grannis et al., 2021, Ho et al., 2021, McHenry et al., 2014, Zarrouf et al., 2009) and reward circuitry (Forbes et al., 2010, Goddings et al., 2019, Hermans et al., 2010, Ho et al., 2021, Ladouceur et al., 2019, Op de Macks et al., 2011, Op de Macks et al., 2016, Peper et al., 2013, Wierenga et al., 2018), and because this was one of the two hormones collected in both male and female participants in the ABCD Study (Cheng et al., 2021), we chose to focus on testosterone as a hormonal indicator of pubertal development.

Testosterone was measured through hormonal assays from saliva samples collected between 7:00 a.m. and 7:00 p.m. and assayed using commercially available immunoassays. Details about the protocol for saliva sample collection, storage, and specifications are provided in Herting et al. (2021). We used the pre-calculated values for testosterone concentrations (in pg/mL) from the 3.0 release data. These values were cleaned following the procedures recommended by Herting et al. (2021). Specifically, for participants who had two values (i.e., two sample replicates of testosterone from saliva collection) within the range of 1–600 pg/mL, if neither value was endorsed as problematic in notes by ABCD research assistants, the two replicates were averaged into a single value. If only one replicate met these criteria, this was used as the final value. If one replicate was noted as too low for detection, its value was changed to zero before averaging with the other replicate value (see Herting et al., 2021, Fig. 1 for decision tree). After each participant had a single value for the testosterone measurement, the values were z-scored. Values within 3 standard deviations of the mean were included in the analyses, and values outside of this range were deemed erroneous and removed as recommended in Aguinis et al. (2013) and Pollet and van der Meij (2017) (after cleaning, >99% of values fell within 3 standard deviations of the mean).

### 3.3 Internalizing symptoms

Internalizing symptoms were measured through the higher-order Internalizing factor score from the Child Behavior Checklist (CBCL), a widely used measure of behavioral and emotional problems in youth that was administered to parents (Achenbach, 1991). The Internalizing factor score has been shown to have good internal consistency (Chronbach's *α* = 0.90) and high test-retest reliability (*r* = 0.91) (Achenbach and Verhulst, 2010). As recommended in Thurber and Sheehan (2012), rather than using the age- and sex-normed T-scores, we used the raw scores of the Internalizing factor in order to maximize variability, given that the age-normed T-scores are truncated (Thurber and Sheehan, 2012). Other research using the ABCD Study has also used CBCL raw scores to better assess sex- and age-related differences (Barch et al., 2021).

Internalizing symptoms encompass both depression- and anxiety-related symptoms. In order to determine whether any observed effects of pubertal timing on internalizing symptoms were specific to a specific symptom profile, we also tested the Anxious-Depressed, Withdrawn-Depressed, and DSM-5 Depressed subscales of the CBCL. Pre-calculated raw scores for the Internalizing factor, Anxious-Depressed, Withdrawn-Depressed, and DSM-5 Depressed subscales were included in the 3.0 release data. For the Internalizing factor and subscales, higher scores indicate greater symptom levels.

### 3.4 Reward sensitivity

#### 3.4.1 Self-reported reward sensitivity

Self-reported reward sensitivity was measured through the Reward Responsiveness subscale of the Behavioral Inhibition System/Behavioral Activation System (BIS/BAS) Scale (Carver and White, 1994). A modified version of the abridged BIS/BAS that adapts the scale for youth report and shortens the BAS Reward Responsiveness (BAS-RR) subscale (Pagliaccio et al., 2016) was administered to participants in the ABCD Study (Barch et al., 2018). Preliminary analyses from an initial sample of 1167 ABCD participants demonstrated that the Reward Responsiveness subscale from the modified BIS/BAS (BAS-RR) yielded an alpha of 0.62 (Barch et al., 2018). Following the methods outlined in Pagliaccio et al. (2016), BAS-RR scores were calculated by summing the four relevant items (i.e., “I feel excited and full of energy when I get something”; “When I am doing well at something, I like to keep doing this”; “It would excite me to win a contest”; “I get really excited when I see an opportunity to get something I like”). All four items were rated on a 4-point scale (0 = not true; 3 = very true) and had to be answered in order to compute the sum. Higher scores indicate greater reward sensitivity.

#### 3.4.2 Behavioral reward sensitivity

Behavioral and neural indicators of reward sensitivity were measured using the Monetary Incentive Delay (MID) Task (Knutson et al., 2000), a widely used task that measures anticipation and receipt of rewards and losses (Dichter et al., 2012, Oldham et al., 2018, Pizzagalli et al., 2009, van Hulst et al., 2015). On each trial, participants are shown a cue that corresponds to an amount of money, ranging from no reward (i.e., neutral) to small ($0.20) and large ($5) rewards, followed by a jittered anticipation event (1500–4000 ms). Next, a target appears (150–500 ms) during which participants are instructed to press a button as soon as they see the target on the screen in order to win the amount shown (including on neutral trials). Once the target disappears, participants received feedback on the reward trials as to whether the reward was received. Average reaction times in milliseconds by condition (i.e., large reward, small reward, or neutral trials) were provided in the 3.0 release data. A target accuracy rate was set at 60%, with the task difficulty adjusting to the participant’s performance after every third reward trial. In the analysis of behavioral data, only participants who met the ABCD-provided inclusion criteria for the MID task were included. Further details regarding the determination of inclusion for behavioral tasks in the ABCD Study can be found in Casey et al. (2018). Based on previous research using the MID task (Knutson et al., 2000, Pizzagalli et al., 2009, van Hulst et al., 2015), two difference scores were calculated as differential indicators of the magnitude of behavioral reward sensitivity: 1) difference in average reaction time on neutral trials versus large reward trials and 2) difference in average reaction time on small reward trials versus large reward trials (van Hulst et al., 2015). In this framework, higher values are equivalent to a greater difference in reaction time between conditions and indicative of greater sensitivity. Difference scores were z-scored, with greater z-scores reflecting greater reward sensitivity relative to the sample.

#### 3.4.3 Neural reward sensitivity

Neural reward sensitivity was operationalized as the blood-oxygen-level-dependent (BOLD) signal during reward anticipation and feedback stages of the MID task. Preliminary results evidencing the quality and age-appropriateness of the task for fMRI analyses in the ABCD sample are provided in Casey et al. (2018) and Chaarani et al. (2021). For analyses of fMRI data in the present study, we determined inclusion criteria based on ABCD-provided indicators of whether or not to include a participant in analyses of fMRI data from the MID task. For specification of quality control practices for neuroimaging data, see the ABCD MRI Quality Control and Recommended Image Inclusion Criteria guidance for the release 3.0 data (http://dx.doi.org/10.15154/1519007).

Five preregistered, *a priori* regions of interest (ROIs) were chosen based on meta-analyses of neural reward processing (Oldham et al., 2018, Sescousse et al., 2013, Silverman et al., 2015) and results from the original MID task studies (Knutson et al., 2000, Knutson et al., 2001). ROIs included subregions of the striatum (nucleus accumbens (NAcc), caudate, and putamen) and the medial and lateral orbitofrontal cortex (mOFC and lOFC). Given conflicting evidence regarding the role of reward anticipation versus reward feedback in predicting internalizing symptoms (Luking et al., 2016), we considered both stages via pre-calculated beta weights from each of the five ROIs. The ABCD Data Analysis, Informatics, and Resource Center (DAIRC) calculated average time courses within each ROI using FreeSurfer’s (Fischl, 2012) anatomically-defined cortical parcellations (“aparc”; Desikan et al., 2006; Destrieux et al., 2010) and subcortical segmentations (“aseg”; Fischl et al., 2002). The ABCD DAIRC estimated beta weights at the individual participant level using a general linear model that included motion estimates and their derivatives as nuisance regressors and averaged beta estimates across runs of the task. For a detailed overview of the imaging procedures, processing, quality control practices, ROI values extraction, and analysis methods used in the ABCD Study, see Casey et al. (2018) and Hagler et al. (2019).

*Reward anticipation* within a given ROI was operationalized as the contrast between average BOLD signal during reward trials compared to no reward trials during reward anticipation. *Reward feedback* within a given ROI was operationalized as the contrast between average BOLD signal in response to reward positive feedback (i.e., a reward was received) compared to reward negative feedback (i.e., a reward was not received) trials during reward feedback. These values were provided in the 3.0 release data. Because we did not have hemisphere-specific hypotheses, beta weights from the right and left hemispheres were averaged to create bilateral estimates for anticipation and feedback within each of the ROIs. These estimates were then z-scored so that greater values indicated greater contrast estimates within a bilateral ROI relative to the sample. Only beta weights from participants who met ABCD inclusion criteria were z-scored. Participants identified as outliers (those with a calculated z < −3 or z > 3) within a given ROI were excluded from analyses of that ROI given the high prevalence of artifacts that can introduce influential outliers in MRI data (Wager et al., 2005), particularly in developmental samples (Poldrack et al., 2002).

## 4 Statistical analysis

### 4.1 Split half analysis procedure

We tested our preregistered hypotheses independently across Samples 1 and 2. Results were considered robust if, across both samples, the effects were statistically significant (*p* < .05) and the coefficient estimates were in the same direction. While we present all results from Samples 1 and 2, we exercised caution in the interpretation of any non-robust findings, placing more emphasis on findings that replicated across the two samples.

### 4.2 Analysis by sex

Extant literature on pubertal development and risk for internalizing psychopathology in female and male participants support disaggregating analyses by sex. First, physiological indicators of pubertal development, such as menarche in female adolescents and a deepening of the voice in male adolescents, and the timing of their onset differ by sex, which convolutes cross-sex comparisons (Deardorff et al., 2019). Additionally, cross-sex comparisons in the current sample are limited given that questions within the Pubertal Development Scale (PDS), our primary predictor of interest, were administered based on sex at birth (i.e., female and male participants answered a different set of questions; Cheng et al., 2021). In addition, based on recent work (Hamlat et al., 2019, Ullsperger and Nikolas, 2017), we did not hypothesize sex differences in our effects, particularly in light of work suggesting that sex differences in risk for internalizing disorders do not emerge until age twelve or older (Angold et al., 1998; Ge et al., 2001; Hankin et al., 1998; Salk et al., 2017) and sex differences in reward processing during adolescence are equivocal (Goddings et al., 2019). Therefore, to aid interpretability, we chose to model male and female participants separately in all statistical analyses and did not treat sex as a moderating variable in our analyses.

### 4.3 Specification of multilevel models

We used a multilevel model approach to account for the non-independent nested structure of ABCD data (i.e., participants are nested within families, which are nested within sites). Mixed effects models were estimated in R 3.6.2 (R Core Team, 2019) using the ‘gamm4’ package version 0.2–6 (Wood and Scheipl, 2020) using restricted maximum likelihood estimation. Random-effect intercepts were selected for family and site. The maximum number of participants were included within each analysis (i.e., if a participant was excluded from imaging analyses based on imaging inclusion criteria, they could still be included in all statistical models that did not involve imaging data). Exact degrees of freedom for every analysis can be found in Supplements A-D, and for each reported statistic, we report the sample size (*N*) of participants included in the analysis. We report both unstandardized (b) and standardized (β) coefficients in the results and supplements. Standardized estimates are reported immediately following each model outlined in Supplements A-D.

### 4.4 Covariates

To examine the effects of pubertal timing above and beyond the effects of age, age was included as a fixed effect in all models that included PDS or testosterone as a predictor. Empirical findings suggest that race and ethnicity relate to pubertal timing (Biro et al., 2013, Chumlea et al., 2003) and risk for internalizing psychopathology (McLaughlin et al., 2007). As such, in statistical models that included measures of both pubertal timing and internalizing symptoms, race and ethnicity were included as fixed effects.

### 4.5 Outliers

Outliers were defined as values ≥ 3 standard deviations away from the mean (based on z-scores calculated after exclusion criteria were taken into account). Outliers were removed for BAS-RR, neuroimaging data, MID reaction time data (Pizzagalli et al., 2009), and testosterone scores (Herting et al., 2021). No outliers were excluded based on z-scores for CBCL because (1) variable-specific inclusion criteria were already taken into account when calculating scores for these variables and (2) even the more extreme values were deemed as within a plausible range for these variables.

### 4.6 Correlation matrix

Sample-specific correlation matrices for all continuous variables of interest are included at the end of Supplements A and B.

### 4.7 Deviations from preregistered analyses

Several details in our final analyses deviated from those in our initial, preregistered analysis plan. First, we used the ABCD 3.0 release in our analyses, rather than the 2.0 release detailed in the preregistration. Parental marital status, which we had previously planned to include in our models, was not included in our final analyses. Parent education and income were included as control variables in sensitivity analyses (rather than in our initial models) as they were not considered primary variables of interest but rather potential confounds (see *Sensitivity Analyses*). In addition to the preregistered neutral vs. large reward contrast in our analyses of MID reaction time, we added an analysis of the small vs. large reward contrast as well based on other studies (Knutson et al., 2001, Knutson et al., 2008, van Hulst et al., 2015). Further details about deviations from the preregistration can be found in the addendum file, included on the OSF page for the preregistration (osf.io/cx3m4/).

### 4.8 Sensitivity analyses

We performed sensitivity analyses to test whether observed associations change after adding several control variables. These variables were added in response to suggestions made during the peer-review process. As such, the sensitivity analyses and additional covariates were not preregistered. The complete output from every sensitivity analysis can be found in Supplements C and D. In our sensitivity analyses, we added the following variables to our original models to ensure that no findings differed after accounting for these covariates: 1) Body Mass Index (BMI) was added as a covariate to all models including PDS or testosterone as predictors. BMI was calculated according to the formula provided by the Center for Disease Control and Prevention growth charts for ages 2–20 years (Kuczmarski, 2002). BMI values outside of the possible range (i.e., 12–35) were removed; 2) Two variables indicating socioeconomic status, household income and highest parental education (referred to as “SES” hereinafter), were added as covariates to all models that included PDS or testosterone as predictors and CBCL internalizing symptoms as the outcome (i.e., all models in which race/ethnicity was already included as a covariate); 3) Minutes-since-midnight for the time of the saliva sample acquisition was added to any models with testosterone as a predictor; 4) The difference in minutes between the time of the saliva sample acquisition and the fMRI scan were added to any models that included both testosterone and neural indicators of reward processes. Unless otherwise stated, the reported results do not change as a result of the sensitivity analyses. In cases where they do differ between a given original model and its corresponding sensitivity analysis, we explicitly note these changes and refer the reader to the appropriate models in Supplements C and D. There were several findings that changed from non-significant to significant after adding control variables, but none of these replicated across samples (and were not preregistered), so we do not interpret them.

## 5 Results

### 5.1 Descriptive statistics

Descriptive statistics for Sample 1 and Sample 2 are displayed in Table 1.

### 5.2 Research question 1: Is pubertal development associated with internalizing symptoms?

**Fig. 1:**
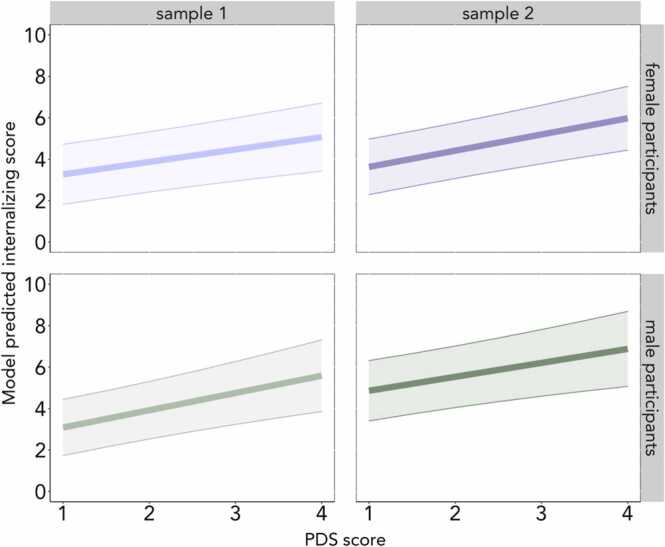
Higher PDS scores in female and male participants were associated with higher internalizing symptoms after adjusting for age, race, and ethnicity. For illustrative purposes, a limited range of model predicted CBCL scores are plotted here. (For the full range of values, see Table 1.) Female participants are plotted in purple and male participants are plotted in green. Light purple and green indicate Sample 1 and dark purple and green indicate Sample 2. (For interpretation of the references to colour in this figure legend, the reader is referred to the web version of this article.)

We first tested whether early pubertal timing, indicated by higher PDS mean scores after controlling for age, was associated with higher internalizing symptoms. As hypothesized, higher PDS mean scores in female and male participants were associated with higher internalizing symptoms in both Sample 1 (Female participants: b = 0.60, β = 0.08, *n*= 2640, SE = 0.16, *t* = 3.80, *p* < .001; Male participants: b = 0.83, β = 0.08, *n* = 2857, SE = 0.20, *t* = 4.21, *p* < .001) and Sample 2 (Female participants: b = 0.78, β = 0.11, *n* = 2620, SE = 0.16, *t* = 4.93, *p* < .001; Male participants: b = 0.67, β = 0.07, *n* = 2832, SE = 0.20, *t* = 3.39, *p* < .01) (Fig. 1; Supplements A & B Model 1.1). Notably, these associations remained in a sensitivity analysis including BMI and SES covariates (Supplements C & D Model 1.1). To address whether the observed association between early pubertal timing and internalizing symptoms was specific to a symptom profile, we next tested whether PDS mean score was associated with anxiety- or depression-specific CBCL subscales. We found that higher PDS mean scores in female and male participants were associated with higher Anxious-Depressed, higher Withdrawn-Depressed, and higher DSM-5 Depressed subscales in both Sample 1 and Sample 2, suggesting that the relationship between pubertal timing and internalizing symptoms exists broadly across internalizing symptom profiles (Supplements A & B Models 1.2–1.4). In a sensitivity analysis, the associations with specific symptom profiles did not replicate across samples after the addition of BMI and SES covariates (Supplements C & D Models 1.2–1.4). Contrary to our hypothesis, testosterone was not associated with internalizing symptoms or symptom subscales in female or male participants across either sample (Supplements A & B Models 1.9–1.12), with the exception of a positive association with the Withdrawn-Depressed subscale for female and male participants in Sample 2 (Supplement B Model 1.11), but this did not remain significant in female or male participants in the sensitivity analysis when BMI and SES were added to the model. Across both samples, the associations between PDS mean scores and internalizing symptoms in female and male participants held when testosterone was included in the model (Supplements A & B Models 1.13–1.20).

We next tested the association between internalizing symptoms and categorical PDS stages. Because we were interested in the transition to puberty, we tested CBCL internalizing scores by PDS stage (controlling for age), with the pre-pubertal stage as the reference level. (For detailed associations between categorical PDS stage and Anxious-Depressed, Withdrawn-Depressed, and DSM-5 Depressed subscales, see Supplements A-D, models 1.6–1.8; for detailed associations between testosterone and CBCL subscales, see models 1.10–1.12.) Compared to pre-puberty, mid-puberty was largely associated with internalizing symptoms across samples and sexes. For female participants in Sample 1, early, mid, and late pubertal stages were associated with higher internalizing symptoms (Supplement A Model 1.5). For female participants in Sample 2, only mid-puberty was associated with higher internalizing symptoms (Supplement B Model 1.5).

Across both samples, male participants in both early and mid-pubertal stages exhibited significantly higher internalizing symptoms than age-matched pre-pubertal male participants. The relatively low number of male participants in the late-pubertal stage (*n* = 28 male, compared with *n* = 137 female participants across samples), may have resulted in insufficient power to detect an effect in the late-pubertal stage. These findings were largely robust in the sensitivity analyses, with the exception that for male participants in Sample 1, only early pubertal stage (and not mid-pubertal stage) was significantly associated with CBCL internalizing symptoms after BMI and SES covariates were included in the model. Notably, the associations between pubertal stage and internalizing symptoms in female and male participants largely held when testosterone was included in the model, as well (Supplements A & B Models 1.13–1.20).

### 5.3 Research question 2: Does reward sensitivity moderate the association between pubertal development and internalizing symptoms?

We next examined whether self-report (i.e., reward responsiveness), behavioral (i.e., MID Task reaction time), or neural (i.e., activation of reward-related ROIs) measures of reward sensitivity moderated the observed association between pubertal timing and internalizing symptoms (controlling for age in all models). To further elucidate the role of reward sensitivity in the link between pubertal timing and internalizing symptoms, we also examined how these different measures of reward sensitivity related to these two constructs independently.

#### 5.3.1 Puberty and reward sensitivity

**Fig. 2:**
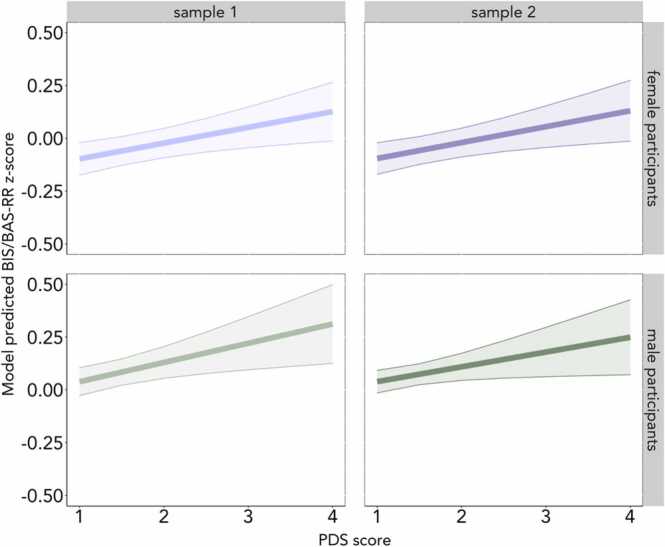
Early pubertal timing, indicated by higher PDS scores while controlling for age, was associated with higher BAS-RR scores across samples. (For the full range of values, see Table 1.) Female participants are plotted in purple and male participants are plotted in green. Light purple and green indicate Sample 1 and dark purple and green indicate Sample 2. Note that, for Sample 2, the association was no longer significant for female or male participants when BMI was added to the model.

We first tested whether early pubertal timing (i.e., higher PDS scores, controlling for age), related to self-reported, behavioral, or neural signatures of reward sensitivity. Early pubertal timing was associated with higher BIS/BAS reward responsiveness (BAS-RR) in female and male participants in Sample 1 (Female participants: b = 0.07, β = 0.06, *n* = 2690, SE = 0.02, *t* = 2.76, *p* < .01; Male participants: b = 0.09, β = 0.05, *n* = 2913, SE = 0.03, *t* = 2.69, *p* < .01) and Sample 2 (Female participants: b = 0.08, β = 0.05, *n* = 2683, SE = 0.03, *t* = 2.69, *p* < .01; Male participants: b = 0.07, β = 0.04, *n* = 2893, SE = 0.03, *t* = 2.14, *p* = .03) (Fig. 2; Supplements A & B Model 2.1). However, when BMI was added to the model in the sensitivity analysis, the association held for female and male participants in Sample 1 (Supplement C Model 2.1), but not in Sample 2 (Supplement D Model 2.1). When testing early pubertal timing and behavioral reward sensitivity (i.e., MID reaction time) we did not find robust evidence of an association (Supplements A & B Models 2.2 & 2.19).

When testing early pubertal timing and neural reward-related activity, we found a positive association between early pubertal timing and medial OFC activity during anticipation for male participants in Sample 1 (b = 0.07, β = 0.06, *n* = 2048, SE = 0.03, *t* = 2.57, *p* = .01); however, this finding did not replicate in Sample 2 (Supplements A & B Model 2.9). Conversely, early pubertal timing was negatively associated with putamen activity during reward anticipation for female participants in Sample 1 (b = −0.08, β = −0.06, *n* = 2041, SE = 0.03, *t* = −2.80, *p* =.01), medial OFC activity during reward anticipation for female participants in Sample 2 (b = −0.05, β = −0.06, *n* = 2059, SE = 0.02, *t* = −2.51, *p* = .01), and caudate activity during reward feedback in male participants in Sample 1 (b = −0.08, β = −0.05, *n* = 2058, SE = 0.04, *t* = −2.08, *p* = .04). However, these effects did not replicate across samples, and the association between caudate activity and reward feedback did not remain in the sensitivity analysis when BMI was added to the model. In separate models, we also tested whether testosterone levels, controlling for age, were associated with reward sensitivity. Testosterone was not robustly associated with self-report, behavioral, or neural reward sensitivity across both samples. While there was a positive association between testosterone and lateral OFC feedback for female participants in Sample 2 (b = 0.002, β = 0.05, *n* = 1900, SE = 0.001, *t* = 2.19, *p* = .03) this finding was not evident in Sample 1 or in male participants, and did not remain significant in the sensitivity analysis when BMI and collection timing variables were added to the model (Supplements C & D Model 2.18). There was also a positive association between testosterone and both caudate and putamen feedback for female participants in Sample 1 (Supplement A Models 2.14–2.15), but this finding was not evident in Sample 2, or in male participants.

#### 5.3.2 Reward sensitivity and internalizing symptoms

We did not observe any robust evidence for an association between self-report, behavioral, or neural measures of reward sensitivity and internalizing symptoms (Supplements A & B Section 3). Although NAcc activity during reward feedback was positively associated with internalizing symptoms in Sample 1 male participants (b = 0.32, β = 0.04, *n* = 2054, SE = 0.16, *t* = 1.97, *p* = .049), this effect did not replicate in Sample 2 (Supplements A & B Model 3.4). There were no other effects of reward sensitivity on internalizing symptoms in non-interaction (i.e., main effect) models.

#### 5.3.3 Reward sensitivity as a moderator

##### 5.3.3.1 Self-reported reward sensitivity

**Fig. 3:**
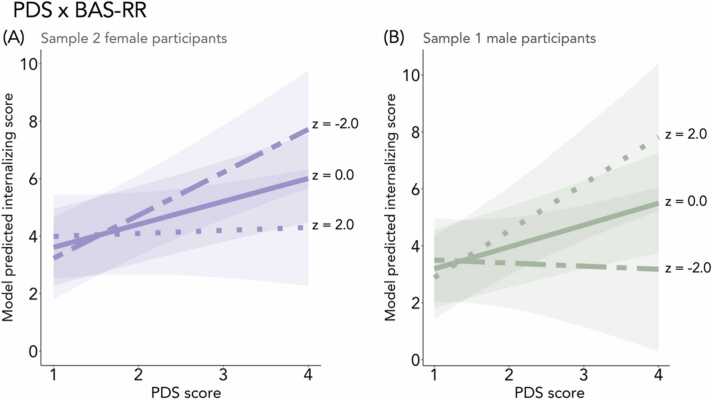
In Sample 2 female participants, the positive association between early pubertal timing and internalizing symptoms was heightened in participants with lower BAS-RR and attenuated in participants with higher BAS-RR (A). The opposite effect was observed in Sample 1 male participants (B), although this effect was not significant when BMI and SES were added as covariates. Two-dash line represents relatively low BAS-RR score (z-score = −2), solid line represents mean BAS-RR score (z-score = 0), and dotted line represents relatively high BAS-RR score (z-score = 2). Predicted lines are from models that adjust for age, race, and ethnicity.

We found evidence for a moderating role of self-reported reward sensitivity (BAS-RR) in female participants in Sample 2, such that, as hypothesized, the association between early pubertal timing and internalizing symptoms was heightened in female participants with lower BAS-RR and attenuated in female participants with higher BAS-RR (b = −0.15, β = −0.21, *n* = 2613, SE = 0.06, *t* = −2.48, *p* = .01; Fig. 3*A*). However, this finding was not evident for female participants in Sample 1 (b= −0.11, β = −0.16, *n* = 2629, SE = 0.06, *t* = −1.80, *p* = .07). Conversely for male participants, and in contrast to our hypothesis, the positive association between early pubertal timing and internalizing symptoms was heightened in male participants with higher BAS-RR in Sample 1 (b = 0.18, β = 0.21, *n* = 2841, SE = 0.08, *t* = 2.24, *p* = .03; Fig. 3*B*). This finding did not replicate in Sample 2, and did not remain significant in the sensitivity analysis when BMI and SES were added to the model (Supplement C Model 4.11). We next tested the interaction between testosterone and BAS-RR, but did not observe any moderation effects on internalizing symptoms (Supplements A & B Model 4.24).

##### 5.3.3.2 Behavioral reward sensitivity

**Fig. 4:**
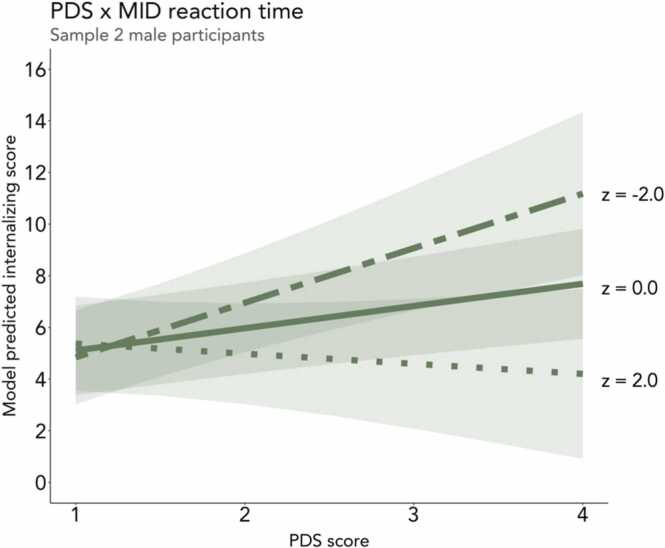
The positive association between early pubertal timing and internalizing symptoms was heightened in Sample 2 male participants who evidenced lower behavioral reward sensitivity (i.e., relatively small differences in reaction time for large reward vs. neutral trials on the MID task) and attenuated in those with higher behavioral reward sensitivity. Two-dash line represents relatively low difference in reaction time for large reward vs. neutral trials (z-score = −2), solid line represents mean difference in reaction time for large reward vs. neutral trials (z-score = 0), and dotted line represents relatively high difference in reaction time for large reward vs. neutral trials (z-score = 2). Predicted lines are from models that adjust for age, race, and ethnicity.

We next tested whether behavioral reward sensitivity (i.e., reaction time differences on large rewards versus neutral and small reward trials on the MID task) moderated the association between pubertal timing and internalizing symptoms. As hypothesized, early pubertal timing was associated with higher internalizing symptoms in Sample 2 male participants who evidenced lower behavioral reward sensitivity (i.e., more similar reaction times for large reward and neutral trials; b = −0.63, β = −0.15, *n* = 2248, SE = 0.23, *t* = −2.76, *p* < .01; Fig. 4). This finding was not evident in Sample 1 male participants or in female participants in either sample. We also tested behavioral reward sensitivity on large reward trials as compared to small reward trials, but did not observe any interactive effects with pubertal timing on internalizing symptoms for female or male participants in either sample (Supplements A & B Models 4.12–4.13). Additionally, we tested the interaction between behavioral reward sensitivity and testosterone, but did not observe an interaction effect on internalizing symptoms in female or male participants in either sample (Supplements A & B Models 4.25–4.26).

##### 5.3.3.3 Neural reward sensitivity: anticipation stage

**Fig. 5:**
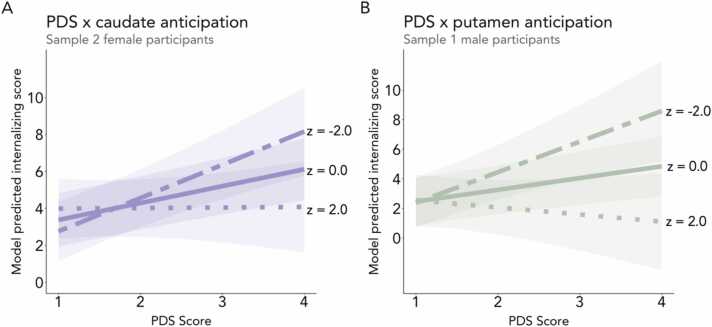
(A) The positive association between early pubertal timing and internalizing symptoms was heightened in Sample 2 female participants who evidenced relatively low caudate activation (i.e., low reward sensitivity) during reward anticipation and attenuated in those with greater caudate activation (i.e., higher reward sensitivity). (B) Similarly, in Sample 1 male participants, the association was heightened in individuals who exhibited relatively low putamen activation during reward anticipation and attenuated in those with greater putamen activation during reward anticipation. Two-dash line represents relatively low activation during reward anticipation (z-score = −2), solid line represents mean activation during reward anticipation (z-score = 0), and dotted line represents relatively high activation during reward anticipation (z-score = 2). Predicted lines are from models that adjust for age, race, and ethnicity.

We next tested whether activity in striatal subregions of interest (i.e., NAcc, caudate, and putamen), along with the medial and lateral OFC, during reward anticipation moderated the association between early pubertal timing and internalizing symptoms (Supplements A & B Section 4). We found two interaction effects in female participants in line with our hypothesis, but neither replicated across samples. First, as hypothesized, the positive association between PDS and internalizing symptoms was strengthened in Sample 2 female participants with attenuated caudate activity during reward anticipation (b = −0.44, β = −0.13, *n* = 2014, SE = 0.18, *t* = −2.42, *p* = .02; Fig. 5*A*). This finding did not replicate in Sample 1. Second, we observed an interaction between testosterone and NAcc reward anticipation, such that testosterone was positively associated with internalizing symptoms for female participants in Sample 2 (but not Sample 1) with attenuated NAcc activity during reward anticipation (b = −0.03, β = −0.14, *n* = 1850, SE = 0.01, *t* = −2.65, *p* < .01). We found one interaction effect in male participants in line with our hypothesis, such that the association between early pubertal timing and internalizing was strengthened for male participants in Sample 1 with attenuated putamen activity during reward anticipation (b = −0.64, β = −0.16, *n* = 2016, SE = 0.25, *t* = −2.59, *p* < .01; Fig. 5*B*). This finding did not replicate in Sample 2. We did not observe any moderating role of neural reward activity in the association between testosterone and internalizing symptoms for male participants in either sample (Supplements A & B Models 4.14–4.23). Additionally, medial and lateral OFC activity during reward anticipation did not play a moderating role for male or female participants in either sample (Supplements A & B Models 4.20–4.21).

##### 5.3.3.4 Neural reward sensitivity: feedback stage

We next tested whether activity in any of the five ROIs during reward feedback moderated the association between early pubertal timing and internalizing symptoms (Supplements A & B Models 4.6–4.10, 4.17–4.19, 4.22–4.23). We found no evidence for a moderating role of neural activity in any of the ROIs during reward feedback in either sample of female or male participants.

## 6 Discussion

We examined associations among pubertal timing, reward sensitivity, and internalizing symptoms in early adolescents from the ABCD Study, leveraging split-half analyses to test the robustness of observed effects across two halves of the data. By studying these associations during the transition to puberty, we captured a window of potential early vulnerability for internalizing symptoms (McGuire et al., 2019). To our knowledge, the association between pubertal timing and internalizing symptoms has yet to be examined in a population-based sample of young adolescents. Moreover, the potential interactive effects of pubertal timing and reward sensitivity — a potent predictor of internalizing risk later in adolescence — remain largely unexamined. Overall, we found robust evidence linking early pubertal timing to heightened internalizing symptoms in female and male participants, even at early stages of the transition into puberty, with non-robust evidence of interactions with reward processes.

### 6.1 Pubertal timing and internalizing symptoms

In line with our hypothesis, we found that female and male participants with early pubertal timing (indicated by greater PDS mean scores relative to age-matched peers) exhibited higher internalizing symptoms on average. This finding extends existing work by showcasing the association between early pubertal timing and internalizing symptoms during the early years of pubertal development. Notably, in this population-based sample of young adolescents, we found the association to be true for both female and male adolescents, which has important implications for the timing of clinical intervention. Sex differences in the effect of early pubertal timing on mental health has been an area of debate resulting from mixed findings, but increasingly, evidence suggests that the early maturing male adolescents are indeed at risk for internalizing disorders (Mendle and Ferrero, 2012, Ullsperger and Nikolas, 2017). Although the effect sizes for the observed associations were relatively small — whereby a one-unit difference in PDS mean score (ranging from 1–4) corresponded to an estimated 0.60–0.80 difference in internalizing scores (which ranged from 0–51 in our sample) — it is notable that this positive association replicated across samples and sexes. Moreover, these small effect sizes may more accurately reflect the association while still having clinical relevance for the early identification of adolescents who are most at risk (Dick et al., 2021). It is possible that as participants enter later stages of puberty, and internalizing rates increase (Costello et al., 2003, Costello et al., 2011, Pfeifer and Allen, 2021), these effects will grow in magnitude. The extent to which early pubertal timing can predict later trajectories of psychopathology is an important potential avenue for future research to explore with implications for early intervention.

Notably, robust associations between pubertal timing and internalizing symptoms were only evident when pubertal timing was based on perceived pubertal development, which captures observable physical changes associated with puberty, and not when it was based on testosterone levels. The physical changes that are perceived by others may be particularly relevant when considering potential psychosocial factors that heighten risk for psychopathology during puberty, such as differential treatment or assumptions made about one’s social and cognitive maturity based on observable physical changes (Barendse et al., 2021; Pfeifer and Allen, 2021; Rudolph, 2014). Previous research has similarly demonstrated an association between internalizing symptoms and psychosocial, but not hormonal, processes during early stages of pubertal development (Barendse et al., 2021). However, this interpretation is limited by the fact that, in the current study, physical changes were assessed exclusively using parent report. Although parent report of pubertal-related changes is more reliable than youth report during early stages of puberty (Cheng et al., 2021, Dorn et al., 1990), further investigation regarding how perceived pubertal development — especially perceptions by social agents other than parents — leads to differential treatment, and how this affects risk for mental illness, is warranted.

It is also important to note that while estradiol (female participants only) and DHEA were also collected in the ABCD Study (Karcher and Barch, 2021), we chose to limit our hormonal analyses to testosterone based on prior work linking this hormone to both internalizing symptoms (Grannis et al., 2021, Ho et al., 2021, McHenry et al., 2014, Zarrouf et al., 2009) and reward circuitry (Forbes et al., 2010, Goddings et al., 2019, Ho et al., 2021, Ladouceur et al., 2019, Op de Macks et al., 2011, Op de Macks et al., 2016, Peper et al., 2013, Wierenga et al., 2018). Moreover, although our sensitivity analysis controlled for saliva time collection, the wide range of sample timing during data collection may still have affected the reliability of the testosterone measurement (Cheng et al., 2021). Future work should incorporate a more comprehensive and controlled measure of hormonal processes (see Cheng et al., 2021 for suggestions) when examining psychosocial versus hormonal processes in determining risk for internalizing symptoms.

Across samples, internalizing symptoms were highest in female participants progressing through mid-puberty and in male participants progressing through early and mid-puberty. This finding aligns with prior work demonstrating that the highest risk for internalizing in female adolescents is experienced by those farthest along in puberty relative to peers during early adolescence (McGuire et al., 2019). Future work in samples with a greater number of adolescents in late stages of puberty is needed in order to determine whether these effects are indeed specific to early and middle stages of puberty. Together, these findings provide empirical support for theoretical models positing that puberty presents a window of vulnerability and opportunity, during which a milieu of developmental processes shape future health outcomes (Dorn et al., 2019, Piekarski et al., 2017).

### 6.2 The role of reward sensitivity in the association between pubertal timing and internalizing symptoms

Given prior work linking reward processes to internalizing risk (Alloy et al., 2016, Luking et al., 2016, Taubitz et al., 2015, Whitton et al., 2015), we tested whether self-reported, behavioral, or neural signatures of reward sensitivity moderated the association between early pubertal timing and internalizing symptoms. We hypothesized that, across domains, greater reward sensitivity would attenuate the association between early pubertal timing and internalizing symptoms.

#### 6.2.1 Self-reported reward sensitivity

Across both samples for female and male participants, early pubertal timing was associated with higher self-reported reward sensitivity (although it was not robust in the Sample 2 sensitivity analyses). This finding extends prior research demonstrating age-related increases in self-reported reward sensitivity across adolescence (Urošević, 2012) by demonstrating an association with pubertal development after accounting for age. Moreover, as hypothesized, the association between early pubertal timing and internalizing symptoms was attenuated in female participants (Sample 2 only) with greater self-reported reward sensitivity. This aligns with prior work suggesting that reward responsiveness, as indicated by the BAS-RR subscale, may play a uniquely protective role against internalizing symptoms (Taubitz al, 2015). However, we observed an opposite effect in male participants (Sample 1 only), such that the association between early pubertal timing and internalizing symptoms was heightened in male participants with greater self-reported reward sensitivity. This difference between female and male participants may indicate etiological differences in how the association between pubertal timing and internalizing symptoms unfolds (Hoyt et al., 2020, Mendle et al., 2010). Although we observed moderating effects of self-reported reward sensitivity, which differed by sex, neither of these findings replicated across samples. Therefore, we caution against strong interpretations of these non-robust associations. Moreover, in line with previous findings in adults (Radulescu et al., 2020) and adolescents (Demidenko et al., 2019), the self-reported measure of reward sensitivity demonstrated low convergence with behavioral and neural indicators of reward sensitivity (see correlation matrices in Supplements A and B), highlighting the importance of incorporating multimethod indicators of reward sensitivity as they do not appear to measure a single trait or process (Radulescu et al., 2020). Nonetheless, it is notable that the BAS-RR subscale is the only adolescent self-report measure used in the study. As such, the use of multiple informants across variables in the study — given the use of parent-reported PDS and internalizing symptoms — renders the moderating effect of self-reported reward sensitivity compelling.

#### 6.2.2 Behavioral reward sensitivity

In our analysis of behavioral reward sensitivity in the MID task, we observed additional evidence that reward sensitivity moderates risk for internalizing symptoms in early maturing adolescents. Specifically, the association between early pubertal timing and internalizing symptoms was heightened for male participants in Sample 2 with lower behavioral reward sensitivity, or smaller differences in reaction time for high reward versus neutral trials. However, this finding did not replicate for male participants in Sample 1 and was not observed in female participants. Notably, this contrasts the observed effect of self-reported reward sensitivity observed in Sample 2 male participants, wherein the association was heightened in those with greater self-reported reward sensitivity. The opposing direction of these two effects could reflect differences in the underlying reward construct being measured by self-report versus task performance (Radulescu et al., 2020). However, because neither of these effects replicated across samples, it is difficult to determine the extent to which self-reported versus task-based measures of reward sensitivity play an underlying role in risk for internalizing symptoms in early maturing male adolescents.

#### 6.2.3 Neural reward sensitivity

Based on research linking activity within striatal and orbitofrontal regions to reward processing in adolescence (van Hulst et al., 2015, Op de Macks et al., 2011, Silverman et al., 2015) and internalizing symptoms (Downar, 2019, Forbes et al., 2009, Rolls, 2019), we tested whether activation during reward anticipation and reward feedback within these regions moderated risk for internalizing symptoms. As with self-reported and behavioral signatures of reward sensitivity, we observed some interaction effects in the neural-based analyses; however, none of the effects replicated. Notably, although we found evidence in the hypothesized direction of a moderating role of caudate and NAcc activity in female participants and putamen activity in male participants — such that the association between early pubertal timing and heightened internalizing was heightened in individuals with lower striatal activity — these effects were only evident in one of the two samples and were specific to the reward anticipation, as opposed to feedback, stage of the task. Together, these findings lend support to the hypothesis that attenuated activity in response to rewards in the striatum may be a risk factor for developing internalizing symptoms (Forbes, 2020), particularly in youth who are already at risk due to early pubertal timing. Differences in neural activity during reward anticipation may therefore be an earlier indication of vulnerability. The role of neural reward circuitry in pubertal-related risk for internalizing is an important avenue for future longitudinal work to investigate, particularly as these systems undergo changes across later stages of adolescence (Galván, 2013). Although the current study focused on internalizing, a higher-order factor across anxiety and depression (Kotov et al., 2017), prior work suggests that patterns of striatal responses to reward may differ depending on the specific internalizing disorder studied (Guyer et al., 2012). It is therefore important for future work to examine how these neural signatures of risk may differ amongst specific internalizing disorders, particularly as they become more common in later stages of adolescence (Costello et al., 2003, Costello et al., 2011, Pfeifer and Allen, 2021).

Together, we observed evidence of a potential role of self-report, behavioral, and neural reward sensitivity in the association between pubertal timing and internalizing symptoms. Further, observed moderation effects were largely consistent with our hypotheses, such that risk was heightened in adolescents with lower reward sensitivity across these three domains of measurement. There are several potential reasons why the observed moderation effects of reward did not replicate across samples in the current study. First, both prior work (Galván, 2013, Steinberg, 2008) and our results herein suggest that reward sensitivity varies across pubertal stages. As such, individual differences in reward sensitivity — and their potential moderating role in pubertal-related risk for internalizing symptoms — may still be unfolding at this early age of adolescence. Notably, reward sensitivity has been shown to peak later than the age of the current sample, between the ages of 14–15 (Galván, 2013), suggesting that potential moderating effects may not emerge until later in the development of the reward system. It is therefore important for future work to examine these processes longitudinally to disentangle the interaction between two variables that are unfolding over time. In addition, it is likely that risk for internalizing during this stage of development is associated with an array of other factors that are not reflected in our reward sensitivity measures. For instance, recent models have emphasized the role of emotion regulation circuitry, including orbitofrontal-amygdala coupling, in linking pubertal maturation to internalizing risk (Spielberg et al., 2019). Future research interrogating these circuit-level mechanisms in early adolescence is warranted.

### 6.3 Strengths and limitations

The large sample size, diversity, and narrow age range of the ABCD cohort render this a highly unique sample. By focusing on a sample of 9–10 year-olds, the current study captures the transition into puberty, a developmental period posited to be particularly salient in regard to mental health risk (Costello et al., 2011, McGuire et al., 2019, Pfeifer and Allen, 2021). Additionally, this study leveraged multiple informants’ perspectives (i.e., participant self-report of reward responsivity, and parent-report of perceived pubertal development and internalizing symptoms), which contributes to the translational value of the findings. Moreover, by including three separate measurements of reward across levels of analysis (i.e., self-report, behavioral, neural), this study reflects heterogeneity in reward processing and its potential role in the association between pubertal timing and internalizing symptoms.

This study has several limitations to note. A limitation of this study was the use of a binary sex variable (i.e., male or female) rather than a more comprehensive and inclusive measure of gender and sex identity. Gender and sex identity may be particularly relevant to the psychosocial effects of early pubertal timing. Fortunately, more dimensional measures of gender and sex identity will be incorporated in future waves of the ABCD Study, offering opportunities for future work in this area (Barch et al., 2018). Additionally, future work should further investigate how the observed associations differ by race and ethnicity (Deardorff et al., 2021, Hayward et al., 1999), which were included in our models but not tested as moderators. Another limitation is that, given the cross-sectional nature of this study, we cannot make inferences about the effects of within-individual developmental processes. Thus, observed associations should be tested for replication in future releases of ABCD data that will have at least three waves of all measures in order to test how these processes unfold within individuals over time. It will also be important for future work to examine whether the observed effect sizes, which are currently relatively small, increase as adolescents mature. As detailed in Cheng et al. (2021), there are limitations regarding both parent-report and hormonal assessments of pubertal maturation in the ABCD Study. For instance, certain adrenal and gonadal processes that occur earlier in development cannot be captured given the age of participants at baseline. This may be particularly impactful for data collected in female participants, who begin these processes earlier on average (Brix et al., 2019), and early maturing adolescents (Cheng et al., 2021). Moreover, although we controlled for collection time in our sensitivity analyses, the diurnal rhythms of hormones present a significant challenge in accounting for between-subject variability in salivary measures when sample collection time is not experimentally controlled. This is especially important to consider given that these diurnal patterns differ across pubertal stages (Matchock et al., 2007; Norjavaara et al., 1996). As participants progress through puberty and a larger proportion of participants reach menarche, it will also be important to incorporate analyses of how menstruation affects hormonal estimates. In addition, while the CBCL is a comprehensive and normed measure of internalizing symptoms, it relies on the parent’s perspective. As such, future longitudinal work should also integrate self-report as adolescents mature (Barch et al., 2018).

Given the relatively narrow age range of the sample, it is difficult to differentiate potentially distinct effects of pubertal stage from timing on the risk for internalizing symptoms (i.e., it is possible that risk for internalizing symptoms is heightened when an individual transitions into puberty, regardless of age). Nonetheless, given that participants in this sample were only 9–10 years of age, advanced pubertal maturation at this young age is likely indicative of early pubertal timing. An additional avenue for future research using these longitudinal data will be to disentangle the effects of pubertal timing from pubertal tempo, or the rate at which an individual is developing through pubertal stages, which we were unable to do in the current study. While future longitudinal research in ABCD can attempt to address tempo, the differential effects of the rate of pubertal change may occur at faster rates than that by which data are collected (Vijayakumar et al., 2018). Nonetheless, the longitudinal nature of the ABCD study offers exciting opportunities to examine these dynamic processes over time and to further elucidate the unique effects of pubertal stage, timing, and tempo on mental health within this population-based sample of youth.

## 7 Conclusion

Our findings support the conclusion that early pubertal timing increases vulnerability for internalizing symptoms in female and male adolescents 9–10 years of age. Further research on the potential moderating role of reward sensitivity in vulnerability for internalizing symptoms is warranted. A greater understanding of individual differences in vulnerability to internalizing symptoms during the transition to puberty has important implications for early identification, prevention, and intervention efforts to improve adolescent mental health.

## CRediT authorship contribution statement

**EAM:** Methodology, Formal analysis, Visualization, Writing – original draft preparation. **NMSH:** Methodology, Formal analysis, Visualization, Writing – original draft preparation. **KLM:** Supervision, Writing – review & editing. RED: Supervision, Writing – review & editing. **LMW:** Supervision, Conceptualization, Methodology, Formal analysis, Writing – review & editing.

## Declaration of Competing Interest

The authors declare that they have no known competing financial interests or personal relationships that could have appeared to influence the work reported in this paper.

## Data Availability

Data used in the preparation of this article were obtained from the Adolescent Brain Cognitive Development (ABCD) Study (https://abcdstudy.org), held in the NIMH Data Archive (NDA).
