# Supplementary material for: Reward sensitivity and internalizing symptoms during the transition to puberty: An examination of 9-and 10-year-olds in the ABCD Study
Source: Dev Cogn Neurosci. 2022 Oct 31;58:101172. doi: 10.1016/j.dcn.2022.101172 (PMC9649995; doi:10.1016/j.dcn.2022.101172)
Supplement: Supplementary file 1 — Supplementary material [file mmc1.pdf]

Supplement A

Reward sensitivity and internalizing symptoms during the transition to puberty: An examination of 9- and 10-year-olds in the ABCD Study.

Contents

Results for Sample 1 9

1—Internalizing~Puberty— 9

1.1 Model: CBCL internalizing factor ~ PDS 9

Female participants 9

Male participants 9

1.2 Model: CBCL Anxious-Depressed ~ PDS 10

Female participants 10

Male participants 11

1.3 Model: CBCL Withdrawn-Depressed ~ PDS 12

Female participants 12

Male participants 12

1.4 Model: CBCL Depressed DSM-5 ~ PDS 13

Female participants 13

Male participants 14

1.5 Model: CBCL internalizing factor ~ Pubertal category 14

Female participants 14

Male participants 15

1.6 Model: CBCL Anxious-Depressed ~ Pubertal category 16

Female participants 16

Male participants 17

1.7 Model: CBCL Withdrawn-Depressed ~ Pubertal category 18

Female participants 18

Male participants 18

1.8 Model: CBCL Depressed DSM-5 ~ Pubertal category 19

Female participants 19

Male participants 20

1.9 Model: CBCL internalizing factor ~ Testosterone 21
Female participants 21
Male participants 22
1.10 Model: CBCL Anxious-Depressed ~ Testosterone 22
Female participants 22
Male participants 23
1.11 Model: CBCL Withdrawn-Depressed ~ Testosterone 24
Female participants 24
Male participants 24
1.12 Model: CBCL Depressed DSM-5 ~ Testosterone 25
Female participants 25
Male participants 26
1.13 Model: CBCL internalizing factor ~ Testosterone + PDS 27
Female participants 27
Male participants 27
1.14 Model: CBCL internalizing factor ~ Testosterone + Pubertal category 28
Female participants 28
Male participants 29
1.15 Model: CBCL Anxious-Depressed ~ Testosterone + PDS 30
Female participants 30
Male participants 30
1.16 Model: CBCL Anxious-Depressed ~ Testosterone + Pubertal category 31
Female participants 31
Male participants 32
1.17 Model: CBCL Withdrawn-Depressed ~ Testosterone + PDS 33
Female participants 33
Male participants 34
1.18 Model: CBCL Withdrawn-Depressed ~ Testosterone + Pubertal category 34
Female participants 34
Male participants 35
1.19 Model: CBCL Depressed DSM-5 ~ Testosterone + PDS 36
Female participants 36
Male participants 37
1.20 Model: CBCL Depressed DSM-5 ~ Testosterone + Pubertal category 38
Female participants 38
Male participants 38

2—Reward~Puberty— 40

2.1 Model: BIS-BAS-RR ~ PDS . . . 40

Female participants . . . 40

Male participants . . . 40

2.2 Model : Reaction Time ~ PDS . . . 41

Female participants . . . 41

Male participants . . . 42

2.3 Model: Caudate Anticipation ~ PDS . . . 42

Female participants . . . 42

Male participants . . . 43

2.4 Model B: Putamen Anticipation ~ PDS . . . 43

Female participants . . . 43

Male participants . . . 44

2.5 Model: Accumbens Anticipation ~ PDS . . . 44

Female participants . . . 44

Male participants . . . 45

2.6 Model: Caudate Feedback ~ PDS . . . 45

Female participants . . . 45

Male participants . . . 46

2.7 Model: Putamen Feedback ~ PDS . . . 46

Female participants . . . 46

Male participants . . . 47

2.8 Model: Accumbens Feedback ~ PDS . . . 47

Female participants . . . 47

Male participants . . . 48

2.9 Model: OFC Anticipation ~ PDS . . . 48

Female participants . . . 48

Male participants . . . 49

2.10 Model: OFC Feedback ~ PDS . . . 50

Female participants . . . 50

Male participants . . . 51

2.11 Model: Caudate Anticipation ~ Testosterone . . . 52

Female participants . . . 52

Male participants . . . 53

2.12 Model B: Putamen Anticipation ~ Testosterone . . . 53

Female participants . . . 53

Male participants 53
2.13 Model: Accumbens Anticipation ~ Testosterone 54
Female participants 54
Male participants 54
2.14 Model: Caudate Feedback ~ Testosterone 55
Female participants 55
Male participants 55
2.15 Model: Putamen Feedback ~ Testosterone 56
Female participants 56
Male participants 56
2.16 Model: Accumbens Feedback ~ Testosterone 57
Female participants 57
Male participants 57
2.17 Model: OFC Anticipation ~ Testosterone 58
Female participants 58
Male participants 59
2.18 Model: OFC Feedback ~ Testosterone 60
Female participants 60
Male participants 61
2.19 Model: MID Reaction Time ~ Testosterone 62
Female participants 62
Male participants 63
2.20 Model: BIS-BAS-RR ~ Testosterone 63
Female participants 63
Male participants 64

3—Internalizing~Reward— 65
3.1 Model: CBCL internalizing factor ~ Nucleus Accumbens activity (anticipation stage) 65
Female participants 65
Male participants 65
3.2 Model: CBCL internalizing factor ~ Caudate activity (anticipation stage) 66
Female participants 66
Male participants 66
3.3 Model: CBCL internalizing factor ~ Putamen activity (anticipation stage) 67
Female participants 67
Male participants 67
3.4 Model: CBCL internalizing factor ~ Accumbens activity (feedback stage) 68

Female participants 68
Male participants 68
3.5 Model: CBCL internalizing factor ~ Caudate activity (feedback stage) 69
Female participants 69
Male participants 69
3.6 Model: CBCL internalizing factor ~ Putamen activity (feedback stage) 70
Female participants 70
Male participants 70
3.7 Model: CBCL internalizing factor ~ OFC activity (anticipation stage) 71
Female participants 71
Male participants 72
3.8 Model: CBCL internalizing factor ~ OFC activity (feedback stage) 73
Female participants 73
Male participants 74
3.9 Model: CBCL internalizing factor ~ BIS-BAS-RR 75
Female participants 75
Male participants 75
3.10 Model: CBCL internalizing factor ~ MID Reaction Time 76
Female participants 76
Male participants 77

4—Internalizing~Puberty x Reward— 78
4.1 Model: CBCL internalizing factor ~ PDS x Accumbens activity (anticipation stage) 78
Female participants 78
Male participants 78
4.2 Model: CBCL internalizing factor ~ PDS x Caudate activity (anticipation stage) 79
Female participants 79
Male participants 80
4.3 Model: CBCL internalizing factor ~ PDS x Putamen activity (anticipation stage) 81
Female participants 81
Male participants 82
4.4 Model: CBCL internalizing factor ~ PDS x Lateral OFC activity (anticipation stage) 82
Female participants 82
Male participants 83
4.5 Model: CBCL internalizing factor ~ PDS x Medial OFC activity (anticipation stage) 84
Female participants 84
Male participants 85

4.6 Model: CBCL internalizing factor ~ PDS x Accumbens activity (feedback) 86
Female participants 86
Male participants 87
4.7 Model: CBCL internalizing factor ~ PDS x Caudate activity (feedback) 88
Female participants 88
Male participants 88
4.8 Model: CBCL internalizing factor ~ PDS x Putamen activity (feedback) 89
Female participants 89
Male participants 90
4.9 Model: CBCL internalizing factor ~ PDS x Lateral OFC activity (feedback stage) 91
Female participants 91
Male participants 92
4.10 Model: CBCL internalizing factor ~ PDS x Medial OFC activity (feedback stage) 92
Female participants 92
Male participants 93
4.11 Model: CBCL internalizing factor ~ PDS x BIS-BAS 94
Female participants 94
Male participants 95
4.12 Model: CBCL internalizing factor ~ PDS x MID reaction time (large reward vs. neutral) . . . 96
Female participants 96
Male participants 96
4.13 Model: CBCL internalizing factor ~ PDS x MID reaction time (large vs. small reward) 97
Female participants 97
Male participants 98
4.14 Model: CBCL internalizing factor ~ Testosterone x Accumbens activity (anticipation stage) + PDS 99
Female participants 99
Male participants 100
4.15 Model: CBCL internalizing factor ~ Testosterone x Caudate activity (anticipation stage) + PDS101
Female participants 101
Male participants 102
4.16 Model: CBCL internalizing factor ~ Testosterone x Putamen activity (anticipation stage) + PDS 103
Female participants 103
Male participants 104
4.17 Model: CBCL internalizing factor ~ Testosterone x Accumbens activity (feedback stage) + PDS105
Female participants 105

Male participants 106
4.18 Model: CBCL internalizing factor ~ Testosterone x Caudate activity (Feedback stage) + PDS 108
Female participants 108
Male participants 109
4.19 Model: CBCL internalizing factor ~ Testosterone x Putamen activity (Feedback stage) + PDS 110
Female participants 110
Male participants 111
4.20 Model: CBCL internalizing factor ~ Testosterone x Lateral OFC activity (anticipation stage) + PDS 112
Female participants 112
Male participants 113
4.21 Model: CBCL internalizing factor ~ Testosterone x Medial OFC activity (anticipation stage) + PDS 114
Female participants 114
Male participants 114
4.22 Model: CBCL internalizing factor ~ Testosterone x Lateral OFC activity (feedback stage) + PDS 115
Female participants 115
Male participants 116
4.23 Model: CBCL internalizing factor ~ Testosterone x Medial OFC activity (feedback stage) + PDS 117
Female participants 117
Male participants 119
4.24 Model: CBCL internalizing factor ~ Testosterone x BIS-BAS RR + PDS 120
Female participants 120
Male participants 121
4.25 Model: CBCL internalizing factor ~ Testosterone x MID Reaction Time + PDS (large reward vs. neutral) 122
Female participants 122
Male participants 123
4.26 Model: CBCL internalizing factor ~ Testosterone x MID Reaction Time + PDS (large vs. small reward) 124
Female participants 124
Male participants 125

5— Correlation Matrix — **128**
Female participants 128
Male participants 129

6— Compare Outliers to Non-Outliers on Demographic Variables — 130

Female participants 130

Male participants 130

Results for Sample 1

1—Internalizing~Puberty—

1.1 Model: CBCL internalizing factor ~ PDS

Female participants

```
##
## Family: gaussian
## Link function: identity
##
## Formula:
## cbcl_scr_syn_internal_r ~ PDS_score + race.ethnicity.5level +
##     interview_age + demo_race_hispanic
##
## Parametric coefficients:
##                           Estimate Std. Error t value Pr(>|t|)
## (Intercept)               3.364047   1.858610   1.810 0.070413 .
## PDS_score                 0.599362   0.157687   3.801 0.000147 ***
## race.ethnicity.5levelBlack  0.135086   0.792591   0.170 0.864681
## race.ethnicity.5levelMixed  1.837143   0.789510   2.327 0.020044 *
## race.ethnicity.5levelOther  2.439633   0.901292   2.707 0.006837 **
## race.ethnicity.5levelWhite  1.354995   0.742020   1.826 0.067950 .
## interview_age            -0.005834   0.014591  -0.400 0.689307
## demo_race_hispanic1       0.216061   0.316107   0.684 0.494348
## ---
## Signif. codes:  0 '***' 0.001 '**' 0.01 '*' 0.05 '.' 0.1 ' ' 1
##
##
## R-sq.(adj) =  0.0121
## lmer.REML =  16403  Scale est. = 13.201    n = 2640
```

```
##                                  stdcoef      stdse
## X(Intercept)                 0.000000000 0.00000000
## XPDS_score                   0.079678274 0.02096269
## Xrace.ethnicity.5levelBlack  0.008788022 0.05156209
## Xrace.ethnicity.5levelMixed  0.111225638 0.04779910
## Xrace.ethnicity.5levelOther  0.093887511 0.03468556
## Xrace.ethnicity.5levelWhite  0.116925862 0.06403075
## Xinterview_age              -0.007909539 0.01978176
## Xdemo_race_hispanic1         0.015489194 0.02266145
```

Male participants

```
##
## Family: gaussian
## Link function: identity
##
## Formula:
## cbcl_scr_syn_internal_r ~ PDS_score + race.ethnicity.5level +
##     interview_age + demo_race_hispanic
```

```
##
## Parametric coefficients:
##                             Estimate Std. Error t value Pr(>|t|)
## (Intercept)                2.2482183  1.7777137   1.265  0.20609
## PDS_score                  0.8330124  0.1980430   4.206 2.68e-05 ***
## race.ethnicity.5levelBlack 1.3769789  0.7417729   1.856  0.06351 .
## race.ethnicity.5levelMixed 2.0936563  0.7432047   2.817  0.00488 **
## race.ethnicity.5levelOther 1.9471613  0.8510452   2.288  0.02221 *
## race.ethnicity.5levelWhite 1.5406055  0.6957503   2.214  0.02689 *
## interview_age              0.0000239  0.0139517   0.002  0.99863
## demo_race_hispanic1        0.2444236  0.3000757   0.815  0.41540
## ---
## Signif. codes:  0 '***' 0.001 '**' 0.01 '*' 0.05 '.' 0.1 ' ' 1
##
##
## R-sq.(adj) =  0.00708
## lmer.REML =  17769  Scale est. = 15.936    n = 2857
```

```
##                                  stdcoef      stdse
## X(Intercept)                0.000000e+00 0.00000000
## XPDS_score                  8.290960e-02 0.01971119
## Xrace.ethnicity.5levelBlack 8.865125e-02 0.04775606
## Xrace.ethnicity.5levelMixed 1.244315e-01 0.04417061
## Xrace.ethnicity.5levelOther 7.605182e-02 0.03323995
## Xrace.ethnicity.5levelWhite 1.328003e-01 0.05997373
## Xinterview_age              3.256634e-05 0.01901086
## Xdemo_race_hispanic1        1.766394e-02 0.02168579
```

1.2 Model: CBCL Anxious-Depressed ~ PDS

Female participants

```
##
## Family: gaussian
## Link function: identity
##
## Formula:
## cbcl_scr_syn_anxdep_r ~ PDS_score + race.ethnicity.5level + interview_age +
##     demo_race_hispanic
##
## Parametric coefficients:
##                             Estimate Std. Error t value Pr(>|t|)
## (Intercept)                 1.738267   1.046826   1.661   0.0969 .
## PDS_score                   0.192989   0.088633   2.177   0.0295 *
## race.ethnicity.5levelBlack  0.034518   0.442769   0.078   0.9379
## race.ethnicity.5levelMixed  0.899818   0.441294   2.039   0.0415 *
## race.ethnicity.5levelOther  0.960117   0.504377   1.904   0.0571 .
## race.ethnicity.5levelWhite  0.798545   0.414637   1.926   0.0542 .
## interview_age              -0.002110   0.008232  -0.256   0.7977
## demo_race_hispanic1         0.024025   0.176180   0.136   0.8915
## ---
## Signif. codes:  0 '***' 0.001 '**' 0.01 '*' 0.05 '.' 0.1 ' ' 1
##
```

```
##
## R-sq.(adj) =  0.00724
## lmer.REML =  13376  Scale est. = 4.9862    n = 2640
```

```
##                                  stdcoef      stdse
## X(Intercept)                 0.000000000 0.00000000
## XPDS_score                   0.045939845 0.02109848
## Xrace.ethnicity.5levelBlack  0.004021024 0.05157799
## Xrace.ethnicity.5levelMixed  0.097548974 0.04784057
## Xrace.ethnicity.5levelOther  0.066162685 0.03475717
## Xrace.ethnicity.5levelWhite  0.123389443 0.06406881
## Xinterview_age              -0.005121910 0.01998372
## Xdemo_race_hispanic1         0.003084070 0.02261604
```

Male participants

```
##
## Family: gaussian
## Link function: identity
##
## Formula:
## cbcl_scr_syn_anxdep_r ~ PDS_score + race.ethnicity.5level + interview_age +
##     demo_race_hispanic
##
## Parametric coefficients:
##                              Estimate Std. Error t value Pr(>|t|)
## (Intercept)                  1.306805   0.993565   1.315 0.188526
## PDS_score                    0.415851   0.110411   3.766 0.000169 ***
## race.ethnicity.5levelBlack   0.620939   0.413367   1.502 0.133169
## race.ethnicity.5levelMixed   1.145179   0.414451   2.763 0.005762 **
## race.ethnicity.5levelOther   1.102178   0.473648   2.327 0.020035 *
## race.ethnicity.5levelWhite   1.048098   0.388072   2.701 0.006959 **
## interview_age               -0.003231   0.007798  -0.414 0.678696
## demo_race_hispanic1          0.097781   0.166098   0.589 0.556111
## ---
## Signif. codes:  0 '***' 0.001 '**' 0.01 '*' 0.05 '.' 0.1 ' ' 1
##
##
## R-sq.(adj) =  0.00662
## lmer.REML =  14455  Scale est. = 6.6672    n = 2857
```

```
##                                  stdcoef      stdse
## X(Intercept)                 0.000000000 0.00000000
## XPDS_score                   0.074413531 0.01975724
## Xrace.ethnicity.5levelBlack  0.071873260 0.04784694
## Xrace.ethnicity.5levelMixed  0.122365648 0.04428523
## Xrace.ethnicity.5levelOther  0.077396393 0.03326020
## Xrace.ethnicity.5levelWhite  0.162431737 0.06014250
## Xinterview_age              -0.007914842 0.01910478
## Xdemo_race_hispanic1         0.012704624 0.02158092
```

1.3 Model: CBCL Withdrawn-Depressed ~ PDS

Female participants

```
##
## Family: gaussian
## Link function: identity
##
## Formula:
## cbcl_scr_syn_withdep_r ~ PDS_score + race.ethnicity.5level +
##     interview_age + demo_race_hispanic
##
## Parametric coefficients:
##                            Estimate Std. Error t value Pr(>|t|)
## (Intercept)                0.560842   0.544323   1.030   0.3029
## PDS_score                  0.192902   0.046013   4.192 2.85e-05 ***
## race.ethnicity.5levelBlack  0.185794   0.228387   0.814   0.4160
## race.ethnicity.5levelMixed  0.401589   0.227843   1.763   0.0781 .
## race.ethnicity.5levelOther  0.569861   0.260772   2.185   0.0290 *
## race.ethnicity.5levelWhite  0.218364   0.213975   1.021   0.3076
## interview_age             -0.002093   0.004288  -0.488   0.6254
## demo_race_hispanic1        0.175618   0.090490   1.941   0.0524 .
## ---
## Signif. codes:  0 '***' 0.001 '**' 0.01 '*' 0.05 '.' 0.1 ' ' 1
##
##
## R-sq.(adj) =  0.0126
## lmer.REML = 9937.2  Scale est. = 1.6344    n = 2640
```

```
##                                  stdcoef      stdse
## X(Intercept)                 0.000000000 0.00000000
## XPDS_score                   0.088652509 0.02114652
## Xrace.ethnicity.5levelBlack  0.041784681 0.05136371
## Xrace.ethnicity.5levelMixed  0.084051987 0.04768728
## Xrace.ethnicity.5levelOther  0.075815285 0.03469358
## Xrace.ethnicity.5levelWhite  0.065141430 0.06383239
## Xinterview_age              -0.009811582 0.02009505
## Xdemo_race_hispanic1         0.043523846 0.02242636
```

Male participants

```
##
## Family: gaussian
## Link function: identity
##
## Formula:
## cbcl_scr_syn_withdep_r ~ PDS_score + race.ethnicity.5level +
##     interview_age + demo_race_hispanic
##
## Parametric coefficients:
##                             Estimate Std. Error t value Pr(>|t|)
## (Intercept)                0.4274730  0.5591341   0.765  0.44462
## PDS_score                  0.1832599  0.0624677   2.934  0.00338 **
```

```
## race.ethnicity.5levelBlack  0.5744953  0.2317862   2.479  0.01325 *
## race.ethnicity.5levelMixed  0.6118766  0.2336103   2.619  0.00886 **
## race.ethnicity.5levelOther  0.4630515  0.2673162   1.732  0.08334 .
## race.ethnicity.5levelWhite  0.3813838  0.2176813   1.752  0.07988 .
## interview_age             -0.0002625  0.0044008  -0.060  0.95244
## demo_race_hispanic1         0.0296570  0.0889215   0.334  0.73877
## ---
## Signif. codes:  0 '***' 0.001 '**' 0.01 '*' 0.05 '.' 0.1 ' ' 1
##
##
## R-sq.(adj) =  0.00628
## lmer.REML =  11223  Scale est. = 2.0769    n = 2857
```

```
##                                   stdcoef      stdse
## X(Intercept)                 0.000000000 0.00000000
## XPDS_score                   0.057881582 0.01973005
## Xrace.ethnicity.5levelBlack  0.117371769 0.04735488
## Xrace.ethnicity.5levelMixed  0.115400683 0.04405920
## Xrace.ethnicity.5levelOther  0.057392691 0.03313237
## Xrace.ethnicity.5levelWhite  0.104325279 0.05954541
## Xinterview_age              -0.001135110 0.01902957
## Xdemo_race_hispanic1         0.006801293 0.02039251
```

1.4 Model: CBCL Depressed DSM-5 ~ PDS

Female participants

```
##
## Family: gaussian
## Link function: identity
##
## Formula:
## cbcl_scr_dsm5_depress_r ~ PDS_score + race.ethnicity.5level +
##     interview_age + demo_race_hispanic
##
## Parametric coefficients:
##                             Estimate Std. Error t value Pr(>|t|)
## (Intercept)                 0.500757   0.632217   0.792 0.428394
## PDS_score                   0.191889   0.053684   3.574 0.000357 ***
## race.ethnicity.5levelBlack  0.220848   0.266590   0.828 0.407508
## race.ethnicity.5levelMixed  0.677402   0.266390   2.543 0.011051 *
## race.ethnicity.5levelOther  0.837469   0.304982   2.746 0.006075 **
## race.ethnicity.5levelWhite  0.519547   0.249759   2.080 0.037604 *
## interview_age              -0.001794   0.004979  -0.360 0.718640
## demo_race_hispanic1         0.107590   0.104881   1.026 0.305064
## ---
## Signif. codes:  0 '***' 0.001 '**' 0.01 '*' 0.05 '.' 0.1 ' ' 1
##
##
## R-sq.(adj) =  0.0102
## lmer.REML =  10738  Scale est. = 1.7625    n = 2640
```

```
##                                   stdcoef      stdse
```

```
## X(Intercept)                 0.000000000 0.00000000
## XPDS_score                   0.075266482 0.02105703
## Xrace.ethnicity.5levelBlack  0.042391288 0.05117131
## Xrace.ethnicity.5levelMixed  0.121006828 0.04758623
## Xrace.ethnicity.5levelOther  0.095094061 0.03463058
## Xrace.ethnicity.5levelWhite  0.132281618 0.06359093
## Xinterview_age              -0.007176567 0.01991746
## Xdemo_race_hispanic1         0.022757644 0.02218454
```

Male participants

```
##
## Family: gaussian
## Link function: identity
##
## Formula:
## cbcl_scr_dsm5_depress_r ~ PDS_score + race.ethnicity.5level +
##     interview_age + demo_race_hispanic
##
## Parametric coefficients:
##                             Estimate Std. Error t value Pr(>|t|)
## (Intercept)                0.4722037  0.6825704   0.692  0.48912
## PDS_score                  0.2264040  0.0760462   2.977  0.00293 **
## race.ethnicity.5levelBlack  0.4987411  0.2837433   1.758  0.07890 .
## race.ethnicity.5levelMixed  0.6664847  0.2849801   2.339  0.01942 *
## race.ethnicity.5levelOther  0.5841449  0.3259736   1.792  0.07324 .
## race.ethnicity.5levelWhite  0.5031305  0.2663922   1.889  0.05904 .
## interview_age              0.0007043  0.0053641   0.131  0.89555
## demo_race_hispanic1       -0.0466581  0.1125717  -0.414  0.67856
## ---
## Signif. codes:  0 '***' 0.001 '**' 0.01 '*' 0.05 '.' 0.1 ' ' 1
##
##
## R-sq.(adj) =  0.00279
## lmer.REML =  12329  Scale est. = 2.9436    n = 2857
```

```
##                                  stdcoef      stdse
## X(Intercept)                 0.000000000 0.00000000
## XPDS_score                   0.058929927 0.01979381
## Xrace.ethnicity.5levelBlack  0.083971353 0.04777291
## Xrace.ethnicity.5levelMixed  0.103588984 0.04429329
## Xrace.ethnicity.5levelOther  0.059665969 0.03329573
## Xrace.ethnicity.5levelWhite  0.113419240 0.06005202
## Xinterview_age               0.002509707 0.01911496
## Xdemo_race_hispanic1        -0.008818003 0.02127513
```

1.5 Model: CBCL internalizing factor ~ Pubertal category

Female participants

```
##
## Family: gaussian
```

```
## Link function: identity
##
## Formula:
## cbcl_scr_syn_internal_r ~ pds_p_ss_category + race.ethnicity.5level +
##     interview_age + demo_race_hispanic
##
## Parametric coefficients:
##                            Estimate Std. Error t value Pr(>|t|)
## (Intercept)                 4.19492    1.89422   2.215 0.026873 *
## pds_p_ss_categoryEarly      1.04585    0.28906   3.618 0.000302 ***
## pds_p_ss_categoryLate       1.70710    0.71494   2.388 0.017023 *
## pds_p_ss_categoryMid        1.20889    0.27421   4.409 1.08e-05 ***
## race.ethnicity.5levelBlack  0.19295    0.79221   0.244 0.807589
## race.ethnicity.5levelMixed  1.90499    0.78883   2.415 0.015805 *
## race.ethnicity.5levelOther  2.49651    0.89969   2.775 0.005562 **
## race.ethnicity.5levelWhite  1.42253    0.74138   1.919 0.055123 .
## interview_age              -0.01158    0.01481  -0.782 0.434254
## demo_race_hispanic1         0.14868    0.31697   0.469 0.639063
## ---
## Signif. codes:  0 '***' 0.001 '**' 0.01 '*' 0.05 '.' 0.1 ' ' 1
##
##
## R-sq.(adj) =  0.0143
## lmer.REML =  16394  Scale est. = 13.028    n = 2640
```

```
##                                  stdcoef      stdse
## X(Intercept)                  0.00000000 0.00000000
## Xpds_p_ss_categoryEarly       0.08060431 0.02227793
## Xpds_p_ss_categoryLate        0.04811159 0.02014948
## Xpds_p_ss_categoryMid         0.10804785 0.02450813
## Xrace.ethnicity.5levelBlack   0.01255255 0.05153739
## Xrace.ethnicity.5levelMixed   0.11533323 0.04775786
## Xrace.ethnicity.5levelOther   0.09607645 0.03462406
## Xrace.ethnicity.5levelWhite   0.12275348 0.06397543
## Xinterview_age               -0.01569732 0.02007197
## Xdemo_race_hispanic1          0.01065867 0.02272329
```

Male participants

```
##
## Family: gaussian
## Link function: identity
##
## Formula:
## cbcl_scr_syn_internal_r ~ pds_p_ss_category + race.ethnicity.5level +
##     interview_age + demo_race_hispanic
##
## Parametric coefficients:
##                            Estimate Std. Error t value Pr(>|t|)
## (Intercept)                2.759444   1.793520   1.539  0.12402
## pds_p_ss_categoryEarly     0.685912   0.246939   2.778  0.00551 **
## pds_p_ss_categoryLate      0.397484   1.460818   0.272  0.78557
## pds_p_ss_categoryMid       1.178202   0.495319   2.379  0.01744 *
```

```
## race.ethnicity.5levelBlack 1.455896   0.743026   1.959  0.05016 .
## race.ethnicity.5levelMixed 2.137193   0.744125   2.872  0.00411 **
## race.ethnicity.5levelOther 1.989141   0.852405   2.334  0.01969 *
## race.ethnicity.5levelWhite 1.578115   0.696638   2.265  0.02357 *
## interview_age              0.002922   0.013943   0.210  0.83403
## demo_race_hispanic1        0.226067   0.301238   0.750  0.45304
## ---
## Signif. codes:  0 '***' 0.001 '**' 0.01 '*' 0.05 '.' 0.1 ' ' 1
##
##
## R-sq.(adj) =  0.00484
## lmer.REML =  17772  Scale est. = 16.208    n = 2857
```

```
##                                 stdcoef      stdse
## X(Intercept)                0.000000000 0.00000000
## Xpds_p_ss_categoryEarly     0.053997924 0.01944007
## Xpds_p_ss_categoryLate      0.005056331 0.01858284
## Xpds_p_ss_categoryMid       0.046489820 0.01954444
## Xrace.ethnicity.5levelBlack 0.093732042 0.04783676
## Xrace.ethnicity.5levelMixed 0.127019025 0.04422529
## Xrace.ethnicity.5levelOther 0.077691440 0.03329308
## Xrace.ethnicity.5levelWhite 0.136033680 0.06005028
## Xinterview_age              0.003981226 0.01899876
## Xdemo_race_hispanic1        0.016337381 0.02176982
```

1.6 Model: CBCL Anxious-Depressed ~ Pubertal category

Female participants

```
##
## Family: gaussian
## Link function: identity
##
## Formula:
## cbcl_scr_syn_anxdep_r ~ pds_p_ss_category + race.ethnicity.5level +
##     interview_age + demo_race_hispanic
##
## Parametric coefficients:
##                             Estimate Std. Error t value Pr(>|t|)
## (Intercept)                 1.915933   1.067993   1.794  0.07293 .
## pds_p_ss_categoryEarly      0.483184   0.163247   2.960  0.00311 **
## pds_p_ss_categoryLate       0.412744   0.403926   1.022  0.30696
## pds_p_ss_categoryMid        0.404799   0.154202   2.625  0.00871 **
## race.ethnicity.5levelBlack  0.084441   0.442742   0.191  0.84876
## race.ethnicity.5levelMixed  0.937872   0.441088   2.126  0.03357 *
## race.ethnicity.5levelOther  0.990706   0.503685   1.967  0.04930 *
## race.ethnicity.5levelWhite  0.830010   0.414449   2.003  0.04531 *
## interview_age              -0.003648   0.008362  -0.436  0.66271
## demo_race_hispanic1         0.006521   0.176760   0.037  0.97058
## ---
## Signif. codes:  0 '***' 0.001 '**' 0.01 '*' 0.05 '.' 0.1 ' ' 1
##
##
```

```
## R-sq.(adj) =  0.00866
## lmer.REML =  13371  Scale est. = 4.9568    n = 2640
```

```
##                                  stdcoef      stdse
## X(Intercept)                  0.0000000000 0.00000000
## Xpds_p_ss_categoryEarly       0.0666821146 0.02252895
## Xpds_p_ss_categoryLate        0.0208294600 0.02038447
## Xpds_p_ss_categoryMid         0.0647849179 0.02467878
## Xrace.ethnicity.5levelBlack   0.0098365511 0.05157488
## Xrace.ethnicity.5levelMixed   0.1016743896 0.04781824
## Xrace.ethnicity.5levelOther   0.0682706259 0.03470949
## Xrace.ethnicity.5levelWhite   0.1282513981 0.06403985
## Xinterview_age               -0.0088550958 0.02029912
## Xdemo_race_hispanic1          0.0008370453 0.02269039
```

Male participants

```
##
## Family: gaussian
## Link function: identity
##
## Formula:
## cbcl_scr_syn_anxdep_r ~ pds_p_ss_category + race.ethnicity.5level +
##     interview_age + demo_race_hispanic
##
## Parametric coefficients:
##                              Estimate Std. Error t value Pr(>|t|)
## (Intercept)                  1.592050   1.001654   1.589  0.11208
## pds_p_ss_categoryEarly       0.436407   0.137843   3.166  0.00156 **
## pds_p_ss_categoryLate        0.345885   0.817733   0.423  0.67234
## pds_p_ss_categoryMid         0.435224   0.275427   1.580  0.11418
## race.ethnicity.5levelBlack   0.660100   0.413879   1.595  0.11084
## race.ethnicity.5levelMixed   1.171886   0.414745   2.826  0.00475 **
## race.ethnicity.5levelOther   1.135306   0.474168   2.394  0.01672 *
## race.ethnicity.5levelWhite   1.069142   0.388383   2.753  0.00595 **
## interview_age               -0.002196   0.007787  -0.282  0.77798
## demo_race_hispanic1          0.087381   0.166734   0.524  0.60027
## ---
## Signif. codes:  0 '***' 0.001 '**' 0.01 '*' 0.05 '.' 0.1 ' ' 1
##
##
## R-sq.(adj) =  0.00511
## lmer.REML =  14457  Scale est. = 6.7476    n = 2857
```

```
##                                 stdcoef      stdse
## X(Intercept)                  0.000000000 0.00000000
## Xpds_p_ss_categoryEarly       0.061767760 0.01950982
## Xpds_p_ss_categoryLate        0.007910588 0.01870203
## Xpds_p_ss_categoryMid         0.030875363 0.01953916
## Xrace.ethnicity.5levelBlack   0.076406127 0.04790630
## Xrace.ethnicity.5levelMixed   0.125219319 0.04431665
## Xrace.ethnicity.5levelOther   0.079722637 0.03329672
## Xrace.ethnicity.5levelWhite   0.165692943 0.06019062
```

```
## Xinterview_age              -0.005378974 0.01907577
## Xdemo_race_hispanic1         0.011353282 0.02166361
```

1.7 Model: CBCL Withdrawn-Depressed ~ Pubertal category

Female participants

```
##
## Family: gaussian
## Link function: identity
##
## Formula:
## cbcl_scr_syn_withdep_r ~ pds_p_ss_category + race.ethnicity.5level +
##     interview_age + demo_race_hispanic
##
## Parametric coefficients:
##                            Estimate Std. Error t value Pr(>|t|)
## (Intercept)                0.983908   0.554434   1.775  0.07608 .
## pds_p_ss_categoryEarly     0.254666   0.084977   2.997  0.00275 **
## pds_p_ss_categoryLate      0.905884   0.210356   4.306 1.72e-05 ***
## pds_p_ss_categoryMid       0.374417   0.079913   4.685 2.94e-06 ***
## race.ethnicity.5levelBlack  0.180349   0.227835   0.792  0.42868
## race.ethnicity.5levelMixed  0.414586   0.227277   1.824  0.06824 .
## race.ethnicity.5levelOther  0.568592   0.259949   2.187  0.02881 *
## race.ethnicity.5levelWhite  0.236442   0.213381   1.108  0.26793
## interview_age             -0.005017   0.004349  -1.154  0.24877
## demo_race_hispanic1        0.140478   0.090445   1.553  0.12050
## ---
## Signif. codes:  0 '***' 0.001 '**' 0.01 '*' 0.05 '.' 0.1 ' ' 1
##
##
## R-sq.(adj) =  0.0172
## lmer.REML =   9927  Scale est. = 1.6132    n = 2640
```

```
##                                stdcoef      stdse
## X(Intercept)                0.00000000 0.00000000
## Xpds_p_ss_categoryEarly     0.06785245 0.02264112
## Xpds_p_ss_categoryLate      0.08826088 0.02049517
## Xpds_p_ss_categoryMid       0.11568793 0.02469164
## Xrace.ethnicity.5levelBlack  0.04056024 0.05123969
## Xrace.ethnicity.5levelMixed  0.08677235 0.04756868
## Xrace.ethnicity.5levelOther  0.07564646 0.03458409
## Xrace.ethnicity.5levelWhite  0.07053444 0.06365511
## Xinterview_age             -0.02351395 0.02038300
## Xdemo_race_hispanic1        0.03481499 0.02241516
```

Male participants

```
##
## Family: gaussian
## Link function: identity
##
```

```
## Formula:
## cbcl_scr_syn_withdep_r ~ pds_p_ss_category + race.ethnicity.5level +
##     interview_age + demo_race_hispanic
##
## Parametric coefficients:
##                             Estimate Std. Error t value Pr(>|t|)
## (Intercept)                0.5480997  0.5635243   0.973  0.33082
## pds_p_ss_categoryEarly     0.1319028  0.0780810   1.689  0.09127 .
## pds_p_ss_categoryLate      0.0210301  0.4639902   0.045  0.96385
## pds_p_ss_categoryMid       0.3981541  0.1562028   2.549  0.01086 *
## race.ethnicity.5levelBlack 0.5803954  0.2319996   2.502  0.01242 *
## race.ethnicity.5levelMixed 0.6177300  0.2336883   2.643  0.00825 **
## race.ethnicity.5levelOther 0.4619416  0.2675335   1.727  0.08434 .
## race.ethnicity.5levelWhite 0.3885289  0.2177832   1.784  0.07453 .
## interview_age              0.0003246  0.0043919   0.074  0.94110
## demo_race_hispanic1        0.0223286  0.0893140   0.250  0.80260
## ---
## Signif. codes:  0 '***' 0.001 '**' 0.01 '*' 0.05 '.' 0.1 ' ' 1
##
##
## R-sq.(adj) =  0.00561
## lmer.REML =  11224  Scale est. = 2.0875    n = 2857
```

```
##                                  stdcoef      stdse
## X(Intercept)                0.0000000000 0.00000000
## Xpds_p_ss_categoryEarly     0.0329520473 0.01950624
## Xpds_p_ss_categoryLate      0.0008489385 0.01873028
## Xpds_p_ss_categoryMid       0.0498549997 0.01955899
## Xrace.ethnicity.5levelBlack 0.1185771728 0.04739848
## Xrace.ethnicity.5levelMixed 0.1165046408 0.04407390
## Xrace.ethnicity.5levelOther 0.0572551237 0.03315931
## Xrace.ethnicity.5levelWhite 0.1062797642 0.05957329
## Xinterview_age              0.0014034236 0.01899089
## Xdemo_race_hispanic1        0.0051206611 0.02048253
```

1.8 Model: CBCL Depressed DSM-5 ~ Pubertal category

Female participants

```
##
## Family: gaussian
## Link function: identity
##
## Formula:
## cbcl_scr_dsm5_depress_r ~ pds_p_ss_category + race.ethnicity.5level +
##     interview_age + demo_race_hispanic
##
## Parametric coefficients:
##                             Estimate Std. Error t value Pr(>|t|)
## (Intercept)                 0.861575   0.644696   1.336  0.18153
## pds_p_ss_categoryEarly      0.256133   0.098889   2.590  0.00965 **
## pds_p_ss_categoryLate       0.731980   0.244492   2.994  0.00278 **
## pds_p_ss_categoryMid        0.380329   0.093369   4.073 4.77e-05 ***
```

```
## race.ethnicity.5levelBlack  0.216245   0.266521   0.811  0.41723
## race.ethnicity.5levelMixed  0.687983   0.266189   2.585  0.00980 **
## race.ethnicity.5levelOther  0.840944   0.304487   2.762  0.00579 **
## race.ethnicity.5levelWhite  0.535046   0.249565   2.144  0.03213 *
## interview_age              -0.004198   0.005054  -0.831  0.40625
## demo_race_hispanic1         0.079854   0.105192   0.759  0.44785
## ---
## Signif. codes:  0 '***' 0.001 '**' 0.01 '*' 0.05 '.' 0.1 ' ' 1
##
##
## R-sq.(adj) =  0.0122
## lmer.REML =  10734  Scale est. = 1.7498    n = 2640
```

```
##                                  stdcoef      stdse
## X(Intercept)                  0.00000000 0.00000000
## Xpds_p_ss_categoryEarly       0.05824499 0.02248746
## Xpds_p_ss_categoryLate        0.06086848 0.02033099
## Xpds_p_ss_categoryMid         0.10029741 0.02462256
## Xrace.ethnicity.5levelBlack   0.04150767 0.05115809
## Xrace.ethnicity.5levelMixed   0.12289703 0.04755027
## Xrace.ethnicity.5levelOther   0.09548869 0.03457437
## Xrace.ethnicity.5levelWhite   0.13622777 0.06354166
## Xinterview_age               -0.01679345 0.02021751
## Xdemo_race_hispanic1          0.01689074 0.02225046
```

Male participants

```
##
## Family: gaussian
## Link function: identity
##
## Formula:
## cbcl_scr_dsm5_depress_r ~ pds_p_ss_category + race.ethnicity.5level +
##     interview_age + demo_race_hispanic
##
## Parametric coefficients:
##                             Estimate Std. Error t value Pr(>|t|)
## (Intercept)                 0.653071   0.687655   0.950   0.3423
## pds_p_ss_categoryEarly      0.220861   0.094888   2.328   0.0200 *
## pds_p_ss_categoryLate      -0.072883   0.562731  -0.130   0.8970
## pds_p_ss_categoryMid        0.474658   0.189822   2.501   0.0125 *
## race.ethnicity.5levelBlack  0.499664   0.283890   1.760   0.0785 .
## race.ethnicity.5levelMixed  0.674279   0.284982   2.366   0.0180 *
## race.ethnicity.5levelOther  0.586039   0.326133   1.797   0.0725 .
## race.ethnicity.5levelWhite  0.513277   0.266405   1.927   0.0541 .
## interview_age               0.001055   0.005352   0.197   0.8438
## demo_race_hispanic1        -0.058102   0.112922  -0.515   0.6069
## ---
## Signif. codes:  0 '***' 0.001 '**' 0.01 '*' 0.05 '.' 0.1 ' ' 1
##
##
## R-sq.(adj) =  0.00262
## lmer.REML =  12328  Scale est. = 2.9441    n = 2857
```

```
##                                   stdcoef      stdse
## X(Intercept)                  0.000000000 0.00000000
## Xpds_p_ss_categoryEarly       0.045470029 0.01953533
## Xpds_p_ss_categoryLate       -0.002424607 0.01872039
## Xpds_p_ss_categoryMid         0.048979790 0.01958767
## Xrace.ethnicity.5levelBlack   0.084126691 0.04779767
## Xrace.ethnicity.5levelMixed   0.104800481 0.04429357
## Xrace.ethnicity.5levelOther   0.059859395 0.03331198
## Xrace.ethnicity.5levelWhite   0.115706510 0.06005496
## Xinterview_age                0.003758767 0.01907250
## Xdemo_race_hispanic1         -0.010980840 0.02134141
```

1.9 Model: CBCL internalizing factor ~ Testosterone

Female participants

```
##
## Family: gaussian
## Link function: identity
##
## Formula:
## cbcl_scr_syn_internal_r ~ hormone_scr_ert_mean + race.ethnicity.5level +
##     interview_age + demo_race_hispanic
##
## Parametric coefficients:
##                             Estimate Std. Error t value Pr(>|t|)
## (Intercept)                 1.777154   1.897510   0.937  0.34907
## hormone_scr_ert_mean        0.005443   0.007058   0.771  0.44066
## race.ethnicity.5levelBlack  0.356154   0.793854   0.449  0.65373
## race.ethnicity.5levelMixed  1.827132   0.793913   2.301  0.02145 *
## race.ethnicity.5levelOther  2.642245   0.908951   2.907  0.00368 **
## race.ethnicity.5levelWhite  1.441831   0.745211   1.935  0.05313 .
## interview_age               0.013505   0.014891   0.907  0.36452
## demo_race_hispanic1         0.107062   0.326216   0.328  0.74279
## ---
## Signif. codes:  0 '***' 0.001 '**' 0.01 '*' 0.05 '.' 0.1 ' ' 1
##
##
## R-sq.(adj) =  0.00669
## lmer.REML =  15258  Scale est. = 13.026    n = 2453
```

```
##                                 stdcoef      stdse
## X(Intercept)                0.000000000 0.00000000
## Xhormone_scr_ert_mean       0.016033862 0.02079048
## Xrace.ethnicity.5levelBlack 0.022625361 0.05043105
## Xrace.ethnicity.5levelMixed 0.111704497 0.04853712
## Xrace.ethnicity.5levelOther 0.103316708 0.03554169
## Xrace.ethnicity.5levelWhite 0.124259018 0.06422335
## Xinterview_age              0.018455837 0.02034908
## Xdemo_race_hispanic1        0.007687489 0.02342355
```

Male participants

```
##
## Family: gaussian
## Link function: identity
##
## Formula:
## cbcl_scr_syn_internal_r ~ hormone_scr_ert_mean + race.ethnicity.5level +
##     interview_age + demo_race_hispanic
##
## Parametric coefficients:
##                            Estimate Std. Error t value Pr(>|t|)
## (Intercept)                2.157007   1.864225   1.157  0.24736
## hormone_scr_ert_mean       0.002743   0.007291   0.376  0.70680
## race.ethnicity.5levelBlack 1.728552   0.771557   2.240  0.02515 *
## race.ethnicity.5levelMixed 2.136824   0.774109   2.760  0.00581 **
## race.ethnicity.5levelOther 1.852717   0.891847   2.077  0.03786 *
## race.ethnicity.5levelWhite 1.578663   0.724376   2.179  0.02939 *
## interview_age              0.009071   0.014673   0.618  0.53647
## demo_race_hispanic1        0.379492   0.312391   1.215  0.22455
## ---
## Signif. codes:  0 '***' 0.001 '**' 0.01 '*' 0.05 '.' 0.1 ' ' 1
##
##
## R-sq.(adj) =  0.000908
## lmer.REML =  16587  Scale est. = 16.746    n = 2651
```

```
##                                stdcoef      stdse
## X(Intercept)                0.000000000 0.00000000
## Xhormone_scr_ert_mean       0.007636881 0.02030028
## Xrace.ethnicity.5levelBlack 0.109149150 0.04871981
## Xrace.ethnicity.5levelMixed 0.126176324 0.04570999
## Xrace.ethnicity.5levelOther 0.070878391 0.03411890
## Xrace.ethnicity.5levelWhite 0.134267496 0.06160918
## Xinterview_age              0.012240349 0.01979880
## Xdemo_race_hispanic1        0.027114435 0.02232008
```

1.10 Model: CBCL Anxious-Depressed ~ Testosterone

Female participants

```
##
## Family: gaussian
## Link function: identity
##
## Formula:
## cbcl_scr_syn_anxdep_r ~ hormone_scr_ert_mean + race.ethnicity.5level +
##     interview_age + demo_race_hispanic
##
## Parametric coefficients:
##                             Estimate Std. Error t value Pr(>|t|)
## (Intercept)                 1.183409   1.072832   1.103   0.2701
## hormone_scr_ert_mean        0.004823   0.003988   1.209   0.2266
```

```
## race.ethnicity.5levelBlack  0.034266   0.445232   0.077   0.9387
## race.ethnicity.5levelMixed  0.857746   0.445476   1.925   0.0543 .
## race.ethnicity.5levelOther  1.034142   0.510581   2.025   0.0429 *
## race.ethnicity.5levelWhite  0.850951   0.418116   2.035   0.0419 *
## interview_age               0.003577   0.008433   0.424   0.6714
## demo_race_hispanic1        -0.027670   0.182744  -0.151   0.8797
## ---
## Signif. codes:  0 '***' 0.001 '**' 0.01 '*' 0.05 '.' 0.1 ' ' 1
##
##
## R-sq.(adj) =  0.00676
## lmer.REML =  12461  Scale est. = 4.9188    n = 2453
```

```
##                                  stdcoef      stdse
## X(Intercept)                 0.000000000 0.00000000
## Xhormone_scr_ert_mean        0.025295012 0.02091515
## Xrace.ethnicity.5levelBlack  0.003875357 0.05035431
## Xrace.ethnicity.5levelMixed  0.093358200 0.04848619
## Xrace.ethnicity.5levelOther  0.071989735 0.03554307
## Xrace.ethnicity.5levelWhite  0.130560331 0.06415092
## Xinterview_age               0.008703330 0.02051598
## Xdemo_race_hispanic1        -0.003537101 0.02336061
```

Male participants

```
##
## Family: gaussian
## Link function: identity
##
## Formula:
## cbcl_scr_syn_anxdep_r ~ hormone_scr_ert_mean + race.ethnicity.5level +
##     interview_age + demo_race_hispanic
##
## Parametric coefficients:
##                              Estimate Std. Error t value Pr(>|t|)
## (Intercept)                 1.4058686  1.0408896   1.351  0.17693
## hormone_scr_ert_mean       -0.0003214  0.0040664  -0.079  0.93701
## race.ethnicity.5levelBlack  0.8143602  0.4297637   1.895  0.05821 .
## race.ethnicity.5levelMixed  1.1554458  0.4315880   2.677  0.00747 **
## race.ethnicity.5levelOther  1.0602400  0.4961530   2.137  0.03270 *
## race.ethnicity.5levelWhite  1.0355457  0.4039386   2.564  0.01041 *
## interview_age               0.0007097  0.0081887   0.087  0.93094
## demo_race_hispanic1         0.1603853  0.1727782   0.928  0.35335
## ---
## Signif. codes:  0 '***' 0.001 '**' 0.01 '*' 0.05 '.' 0.1 ' ' 1
##
##
## R-sq.(adj) =  0.00142
## lmer.REML =  13510  Scale est. = 7.1618    n = 2651
```

```
##                                  stdcoef      stdse
## X(Intercept)                 0.000000000 0.00000000
## Xhormone_scr_ert_mean        0.025295012 0.02091515
```

```
## Xrace.ethnicity.5levelBlack  0.003875357 0.05035431
## Xrace.ethnicity.5levelMixed  0.093358200 0.04848619
## Xrace.ethnicity.5levelOther  0.071989735 0.03554307
## Xrace.ethnicity.5levelWhite  0.130560331 0.06415092
## Xinterview_age               0.008703330 0.02051598
## Xdemo_race_hispanic1        -0.003537101 0.02336061
```

1.11 Model: CBCL Withdrawn-Depressed ~ Testosterone

Female participants

```
##
## Family: gaussian
## Link function: identity
##
## Formula:
## cbcl_scr_syn_withdep_r ~ hormone_scr_ert_mean + race.ethnicity.5level +
##     interview_age + demo_race_hispanic
##
## Parametric coefficients:
##                             Estimate Std. Error t value Pr(>|t|)
## (Intercept)                 0.133412   0.551030   0.242   0.8087
## hormone_scr_ert_mean        0.001354   0.002047   0.661   0.5084
## race.ethnicity.5levelBlack  0.276842   0.226348   1.223   0.2214
## race.ethnicity.5levelMixed  0.433104   0.226779   1.910   0.0563 .
## race.ethnicity.5levelOther  0.595871   0.260391   2.288   0.0222 *
## race.ethnicity.5levelWhite  0.254142   0.212744   1.195   0.2324
## interview_age               0.003477   0.004341   0.801   0.4232
## demo_race_hispanic1         0.143812   0.092509   1.555   0.1202
## ---
## Signif. codes:  0 '***' 0.001 '**' 0.01 '*' 0.05 '.' 0.1 ' ' 1
##
##
## R-sq.(adj) =  0.00431
## lmer.REML =   9205  Scale est. = 1.635     n = 2453
```

```
##                                 stdcoef      stdse
## X(Intercept)                 0.00000000 0.00000000
## Xhormone_scr_ert_mean        0.01394301 0.02108262
## Xrace.ethnicity.5levelBlack  0.06147413 0.05026165
## Xrace.ethnicity.5levelMixed  0.09255437 0.04846269
## Xrace.ethnicity.5levelOther  0.08144294 0.03558991
## Xrace.ethnicity.5levelWhite  0.07655856 0.06408774
## Xinterview_age               0.01661007 0.02073628
## Xdemo_race_hispanic1         0.03609479 0.02321844
```

Male participants

```
##
## Family: gaussian
## Link function: identity
##
```

```
## Formula:
## cbcl_scr_syn_withdep_r ~ hormone_scr_ert_mean + race.ethnicity.5level +
##     interview_age + demo_race_hispanic
##
## Parametric coefficients:
##                            Estimate Std. Error t value Pr(>|t|)
## (Intercept)                0.321946   0.577770   0.557  0.57742
## hormone_scr_ert_mean       0.001620   0.002244   0.722  0.47037
## race.ethnicity.5levelBlack 0.678586   0.237448   2.858  0.00430 **
## race.ethnicity.5levelMixed 0.653492   0.239863   2.724  0.00648 **
## race.ethnicity.5levelOther 0.449709   0.276074   1.629  0.10344
## race.ethnicity.5levelWhite 0.415977   0.223382   1.862  0.06269 .
## interview_age              0.001935   0.004558   0.425  0.67120
## demo_race_hispanic1        0.061942   0.091277   0.679  0.49744
## ---
## Signif. codes:  0 '***' 0.001 '**' 0.01 '*' 0.05 '.' 0.1 ' ' 1
##
##
## R-sq.(adj) =  0.00368
## lmer.REML =  10441  Scale est. = 2.2517    n = 2651
```

```
##                                  stdcoef      stdse
## X(Intercept)                0.000000000 0.00000000
## Xhormone_scr_ert_mean       0.014498371 0.02008132
## Xrace.ethnicity.5levelBlack 0.137702360 0.04818414
## Xrace.ethnicity.5levelMixed 0.124007428 0.04551675
## Xrace.ethnicity.5levelOther 0.055288500 0.03394135
## Xrace.ethnicity.5levelWhite 0.113697083 0.06105606
## Xinterview_age              0.008391835 0.01976630
## Xdemo_race_hispanic1        0.014222578 0.02095832
```

1.12 Model: CBCL Depressed DSM-5 ~ Testosterone

Female participants

```
##
## Family: gaussian
## Link function: identity
##
## Formula:
## cbcl_scr_dsm5_depress_r ~ hormone_scr_ert_mean + race.ethnicity.5level +
##     interview_age + demo_race_hispanic
##
## Parametric coefficients:
##                            Estimate Std. Error t value Pr(>|t|)
## (Intercept)                0.062803   0.644415   0.097  0.92237
## hormone_scr_ert_mean       0.001558   0.002399   0.650  0.51607
## race.ethnicity.5levelBlack 0.289625   0.265904   1.089  0.27617
## race.ethnicity.5levelMixed 0.689545   0.267077   2.582  0.00989 **
## race.ethnicity.5levelOther 0.886527   0.306968   2.888  0.00391 **
## race.ethnicity.5levelWhite 0.552485   0.249976   2.210  0.02719 *
## interview_age              0.003890   0.005076   0.766  0.44361
## demo_race_hispanic1        0.066325   0.107696   0.616  0.53805
```

```
## ---
## Signif. codes:  0 '***' 0.001 '**' 0.01 '*' 0.05 '.' 0.1 ' ' 1
##
##
## R-sq.(adj) =  0.00535
## lmer.REML =   9990  Scale est. = 1.7521    n = 2453
```

```
##                                stdcoef      stdse
## X(Intercept)                0.00000000 0.00000000
## Xhormone_scr_ert_mean       0.01354995 0.02086202
## Xrace.ethnicity.5levelBlack 0.05432347 0.04987408
## Xrace.ethnicity.5levelMixed 0.12446769 0.04820919
## Xrace.ethnicity.5levelOther 0.10234885 0.03543918
## Xrace.ethnicity.5levelWhite 0.14058123 0.06360714
## Xinterview_age              0.01569345 0.02048120
## Xdemo_race_hispanic1        0.01406111 0.02283175
```

Male participants

```
##
## Family: gaussian
## Link function: identity
##
## Formula:
## cbcl_scr_dsm5_depress_r ~ hormone_scr_ert_mean + race.ethnicity.5level +
##     interview_age + demo_race_hispanic
##
## Parametric coefficients:
##                              Estimate Std. Error t value Pr(>|t|)
## (Intercept)                 0.4369093  0.7141009   0.612   0.5407
## hormone_scr_ert_mean        0.0007399  0.0027866   0.266   0.7906
## race.ethnicity.5levelBlack  0.5968958  0.2944573   2.027   0.0428 *
## race.ethnicity.5levelMixed  0.7065006  0.2963343   2.384   0.0172 *
## race.ethnicity.5levelOther  0.5322299  0.3411650   1.560   0.1189
## race.ethnicity.5levelWhite  0.5149283  0.2767705   1.860   0.0629 .
## interview_age               0.0033258  0.0056274   0.591   0.5546
## demo_race_hispanic1        -0.0169056  0.1167966  -0.145   0.8849
## ---
## Signif. codes:  0 '***' 0.001 '**' 0.01 '*' 0.05 '.' 0.1 ' ' 1
##
##
## R-sq.(adj) =  -0.000239
## lmer.REML =  11531  Scale est. = 2.9567    n = 2651
```

```
##                                  stdcoef      stdse
## X(Intercept)                 0.000000000 0.00000000
## Xhormone_scr_ert_mean        0.005382587 0.02027209
## Xrace.ethnicity.5levelBlack  0.098482933 0.04858305
## Xrace.ethnicity.5levelMixed  0.109004982 0.04572099
## Xrace.ethnicity.5levelOther  0.053202093 0.03410311
## Xrace.ethnicity.5levelWhite  0.114433424 0.06150719
## Xinterview_age               0.011725524 0.01984050
## Xdemo_race_hispanic1        -0.003156124 0.02180481
```

1.13 Model: CBCL internalizing factor ~ Testosterone + PDS

Female participants

```
##
## Family: gaussian
## Link function: identity
##
## Formula:
## cbcl_scr_syn_internal_r ~ hormone_scr_ert_mean + PDS_score +
##     race.ethnicity.5level + interview_age + demo_race_hispanic
##
## Parametric coefficients:
##                              Estimate Std. Error t value Pr(>|t|)
## (Intercept)                 2.5126736  1.9039529   1.320 0.187054
## hormone_scr_ert_mean       -0.0007324  0.0072485  -0.101 0.919525
## PDS_score                   0.6083923  0.1697666   3.584 0.000345 ***
## race.ethnicity.5levelBlack -0.0375505  0.7994683  -0.047 0.962542
## race.ethnicity.5levelMixed  1.6444446  0.7935891   2.072 0.038355 *
## race.ethnicity.5levelOther  2.4066014  0.9091006   2.647 0.008167 **
## race.ethnicity.5levelWhite  1.3496082  0.7437925   1.814 0.069724 .
## interview_age               0.0018428  0.0152063   0.121 0.903555
## demo_race_hispanic1         0.0937585  0.3253881   0.288 0.773261
## ---
## Signif. codes:  0 '***' 0.001 '**' 0.01 '*' 0.05 '.' 0.1 ' ' 1
##
##
## R-sq.(adj) =  0.011
## lmer.REML =  15247  Scale est. = 12.976    n = 2453
```

```
##                                   stdcoef      stdse
## X(Intercept)                  0.000000000 0.00000000
## Xhormone_scr_ert_mean        -0.002157532 0.02135282
## XPDS_score                    0.079996978 0.02232246
## Xrace.ethnicity.5levelBlack -0.002385464 0.05078772
## Xrace.ethnicity.5levelMixed  0.100535650 0.04851729
## Xrace.ethnicity.5levelOther  0.094102604 0.03554753
## Xrace.ethnicity.5levelWhite  0.116311148 0.06410109
## Xinterview_age                0.002518249 0.02078038
## Xdemo_race_hispanic1          0.006732210 0.02336409
```

Male participants

```
##
## Family: gaussian
## Link function: identity
##
## Formula:
## cbcl_scr_syn_internal_r ~ hormone_scr_ert_mean + PDS_score +
##     race.ethnicity.5level + interview_age + demo_race_hispanic
##
## Parametric coefficients:
##                              Estimate Std. Error t value Pr(>|t|)
```

```
## (Intercept)                 2.3385395  1.8581774   1.259  0.20832
## hormone_scr_ert_mean       -0.0008243  0.0073102  -0.113  0.91023
## PDS_score                   0.9481165  0.2116215   4.480 7.77e-06 ***
## race.ethnicity.5levelBlack  1.3600344  0.7733580   1.759  0.07876 .
## race.ethnicity.5levelMixed  2.0456100  0.7716998   2.651  0.00808 **
## race.ethnicity.5levelOther  1.7250461  0.8892679   1.940  0.05250 .
## race.ethnicity.5levelWhite  1.5412692  0.7219657   2.135  0.03287 *
## interview_age              -0.0013724  0.0148083  -0.093  0.92617
## demo_race_hispanic1         0.3025942  0.3120542   0.970  0.33229
## ---
## Signif. codes:  0 '***' 0.001 '**' 0.01 '*' 0.05 '.' 0.1 ' ' 1
##
##
## R-sq.(adj) =  0.00746
## lmer.REML =  16568  Scale est. = 16.482    n = 2651
```

```
##                                  stdcoef      stdse
## X(Intercept)                 0.000000000 0.00000000
## Xhormone_scr_ert_mean       -0.002294870 0.02035276
## XPDS_score                   0.092394046 0.02062254
## Xrace.ethnicity.5levelBlack  0.085879143 0.04883356
## Xrace.ethnicity.5levelMixed  0.120790285 0.04556775
## Xrace.ethnicity.5levelOther  0.065994169 0.03402025
## Xrace.ethnicity.5levelWhite  0.131087101 0.06140419
## Xinterview_age              -0.001851802 0.01998125
## Xdemo_race_hispanic1         0.021620120 0.02229603
```

1.14 Model: CBCL internalizing factor ~ Testosterone + Pubertal category

Female participants

```
##
## Family: gaussian
## Link function: identity
##
## Formula:
## cbcl_scr_syn_internal_r ~ hormone_scr_ert_mean + pds_p_ss_category +
##     race.ethnicity.5level + interview_age + demo_race_hispanic
##
## Parametric coefficients:
##                             Estimate Std. Error t value Pr(>|t|)
## (Intercept)                 3.1911880  1.9388316   1.646  0.09991 .
## hormone_scr_ert_mean        0.0004012  0.0071781   0.056  0.95543
## pds_p_ss_categoryEarly      0.9264288  0.2982685   3.106  0.00192 **
## pds_p_ss_categoryLate       1.0992131  0.7741539   1.420  0.15577
## pds_p_ss_categoryMid        1.2225403  0.2890843   4.229 2.43e-05 ***
## race.ethnicity.5levelBlack  0.0056281  0.7994747   0.007  0.99438
## race.ethnicity.5levelMixed  1.6934486  0.7931990   2.135  0.03286 *
## race.ethnicity.5levelOther  2.4769969  0.9078018   2.729  0.00641 **
## race.ethnicity.5levelWhite  1.4036386  0.7433801   1.888  0.05912 .
## interview_age              -0.0024913  0.0154099  -0.162  0.87158
## demo_race_hispanic1         0.0477434  0.3264159   0.146  0.88372
## ---
```

```
## Signif. codes:  0 '***' 0.001 '**' 0.01 '*' 0.05 '.' 0.1 ' ' 1
##
##
## R-sq.(adj) =  0.0123
## lmer.REML =  15239  Scale est. = 12.821    n = 2453
```

```
##                                  stdcoef      stdse
## X(Intercept)                  0.0000000000 0.00000000
## Xhormone_scr_ert_mean         0.0011818672 0.02114545
## Xpds_p_ss_categoryEarly       0.0720675070 0.02320251
## Xpds_p_ss_categoryLate        0.0296449313 0.02087833
## Xpds_p_ss_categoryMid         0.1091599094 0.02581217
## Xrace.ethnicity.5levelBlack   0.0003575324 0.05078812
## Xrace.ethnicity.5levelMixed   0.1035315893 0.04849344
## Xrace.ethnicity.5levelOther   0.0968551970 0.03549674
## Xrace.ethnicity.5levelWhite   0.1209675645 0.06406555
## Xinterview_age               -0.0034045374 0.02105865
## Xdemo_race_hispanic1          0.0034281562 0.02343789
```

Male participants

```
##
## Family: gaussian
## Link function: identity
##
## Formula:
## cbcl_scr_syn_internal_r ~ hormone_scr_ert_mean + pds_p_ss_category +
##     race.ethnicity.5level + interview_age + demo_race_hispanic
##
## Parametric coefficients:
##                             Estimate Std. Error t value Pr(>|t|)
## (Intercept)                 2.966291   1.874389   1.583  0.11365
## hormone_scr_ert_mean        0.000713   0.007302   0.098  0.92222
## pds_p_ss_categoryEarly      0.814256   0.260188   3.129  0.00177 **
## pds_p_ss_categoryLate       0.840681   1.596758   0.526  0.59859
## pds_p_ss_categoryMid        1.274985   0.524943   2.429  0.01521 *
## race.ethnicity.5levelBlack 1.431202   0.775159   1.846  0.06496 .
## race.ethnicity.5levelMixed 2.091142   0.772971   2.705  0.00687 **
## race.ethnicity.5levelOther 1.786129   0.891038   2.005  0.04511 *
## race.ethnicity.5levelWhite 1.585169   0.723071   2.192  0.02845 *
## interview_age               0.001113   0.014814   0.075  0.94013
## demo_race_hispanic1         0.273079   0.313390   0.871  0.38363
## ---
## Signif. codes:  0 '***' 0.001 '**' 0.01 '*' 0.05 '.' 0.1 ' ' 1
##
##
## R-sq.(adj) =  0.00487
## lmer.REML =  16571  Scale est. = 16.785    n = 2651
```

```
##                                stdcoef      stdse
## X(Intercept)                 0.000000000 0.00000000
## Xhormone_scr_ert_mean        0.001985229 0.02032979
## Xpds_p_ss_categoryEarly      0.063275702 0.02021920
```

```
## Xpds_p_ss_categoryLate      0.010146829 0.01927251
## Xpds_p_ss_categoryMid       0.049505338 0.02038258
## Xrace.ethnicity.5levelBlack 0.090373022 0.04894729
## Xrace.ethnicity.5levelMixed 0.123478858 0.04564283
## Xrace.ethnicity.5levelOther 0.068330972 0.03408798
## Xrace.ethnicity.5levelWhite 0.134820812 0.06149817
## Xinterview_age              0.001501517 0.01998875
## Xdemo_race_hispanic1        0.019511277 0.02239146
```

1.15 Model: CBCL Anxious-Depressed ~ Testosterone + PDS

Female participants

```
##
## Family: gaussian
## Link function: identity
##
## Formula:
## cbcl_scr_syn_anxdep_r ~ hormone_scr_ert_mean + PDS_score + race.ethnicity.5level +
##     interview_age + demo_race_hispanic
##
## Parametric coefficients:
##                              Estimate Std. Error t value Pr(>|t|)
## (Intercept)                 1.3963972  1.0785158   1.295   0.1955
## hormone_scr_ert_mean        0.0030115  0.0041066   0.733   0.4634
## PDS_score                   0.1760253  0.0959901   1.834   0.0668 .
## race.ethnicity.5levelBlack -0.0801965  0.4493329  -0.178   0.8584
## race.ethnicity.5levelMixed  0.8045575  0.4461652   1.803   0.0715 .
## race.ethnicity.5levelOther  0.9650993  0.5116810   1.886   0.0594 .
## race.ethnicity.5levelWhite  0.8238669  0.4181297   1.970   0.0489 *
## interview_age               0.0002133  0.0086250   0.025   0.9803
## demo_race_hispanic1        -0.0314620  0.1826377  -0.172   0.8632
## ---
## Signif. codes:  0 '***' 0.001 '**' 0.01 '*' 0.05 '.' 0.1 ' ' 1
##
##
## R-sq.(adj) =  0.0077
## lmer.REML =  12460  Scale est. = 4.9271    n = 2453
```

```
##                                   stdcoef      stdse
## X(Intercept)                 0.0000000000 0.00000000
## Xhormone_scr_ert_mean        0.0157934393 0.02153673
## XPDS_score                   0.0412057495 0.02247032
## Xrace.ethnicity.5levelBlack -0.0090699635 0.05081812
## Xrace.ethnicity.5levelMixed  0.0875690910 0.04856120
## Xrace.ethnicity.5levelOther  0.0671834638 0.03561965
## Xrace.ethnicity.5levelWhite  0.1264048082 0.06415309
## Xinterview_age               0.0005188738 0.02098374
## Xdemo_race_hispanic1        -0.0040218594 0.02334697
```

Male participants

```
##
```

```
## Family: gaussian
## Link function: identity
##
## Formula:
## cbcl_scr_syn_anxdep_r ~ hormone_scr_ert_mean + PDS_score + race.ethnicity.5level +
##     interview_age + demo_race_hispanic
##
## Parametric coefficients:
##                             Estimate Std. Error t value Pr(>|t|)
## (Intercept)                 1.502920   1.038409   1.447   0.1479
## hormone_scr_ert_mean       -0.002132   0.004081  -0.523   0.6013
## PDS_score                   0.479811   0.118037   4.065 4.95e-05 ***
## race.ethnicity.5levelBlack  0.626666   0.431152   1.453   0.1462
## race.ethnicity.5levelMixed  1.107656   0.430574   2.573   0.0102 *
## race.ethnicity.5levelOther  0.996180   0.495062   2.012   0.0443 *
## race.ethnicity.5levelWhite  1.015140   0.402920   2.519   0.0118 *
## interview_age              -0.004613   0.008272  -0.558   0.5771
## demo_race_hispanic1         0.122108   0.172829   0.707   0.4799
## ---
## Signif. codes:  0 '***' 0.001 '**' 0.01 '*' 0.05 '.' 0.1 ' ' 1
##
##
## R-sq.(adj) =  0.0067
## lmer.REML =  13496  Scale est. = 7.0718    n = 2651
```

```
##                                 stdcoef      stdse
## X(Intercept)                 0.00000000 0.00000000
## Xhormone_scr_ert_mean       -0.01066931 0.02041700
## XPDS_score                   0.08402881 0.02067175
## Xrace.ethnicity.5levelBlack  0.07111311 0.04892653
## Xrace.ethnicity.5levelMixed  0.11754121 0.04569126
## Xrace.ethnicity.5levelOther  0.06848869 0.03403618
## Xrace.ethnicity.5levelWhite  0.15516135 0.06158525
## Xinterview_age              -0.01118530 0.02005850
## Xdemo_race_hispanic1         0.01567902 0.02219165
```

1.16 Model: CBCL Anxious-Depressed ~ Testosterone + Pubertal category

Female participants

```
##
## Family: gaussian
## Link function: identity
##
## Formula:
## cbcl_scr_syn_anxdep_r ~ hormone_scr_ert_mean + pds_p_ss_category +
##     race.ethnicity.5level + interview_age + demo_race_hispanic
##
## Parametric coefficients:
##                             Estimate Std. Error t value Pr(>|t|)
## (Intercept)                 1.508191   1.098965   1.372  0.17007
## hormone_scr_ert_mean        0.003622   0.004068   0.890  0.37332
## pds_p_ss_categoryEarly      0.453347   0.169177   2.680  0.00742 **
```

```
## pds_p_ss_categoryLate       0.098787   0.440211   0.224  0.82246
## pds_p_ss_categoryMid        0.398897   0.163385   2.441  0.01470 *
## race.ethnicity.5levelBlack -0.039410   0.449379  -0.088  0.93012
## race.ethnicity.5levelMixed  0.832990   0.445977   1.868  0.06191 .
## race.ethnicity.5levelOther  0.999761   0.510972   1.957  0.05051 .
## race.ethnicity.5levelWhite  0.849123   0.417941   2.032  0.04229 *
## interview_age              -0.001035   0.008746  -0.118  0.90579
## demo_race_hispanic1        -0.040163   0.183275  -0.219  0.82656
## ---
## Signif. codes:  0 '***' 0.001 '**' 0.01 '*' 0.05 '.' 0.1 ' ' 1
##
##
## R-sq.(adj) =  0.00886
## lmer.REML =  12456  Scale est. = 4.8884    n = 2453
```

```
##                                  stdcoef      stdse
## X(Intercept)                 0.000000000 0.00000000
## Xhormone_scr_ert_mean        0.018994922 0.02133217
## Xpds_p_ss_categoryEarly      0.062784333 0.02342944
## Xpds_p_ss_categoryLate       0.004743106 0.02113600
## Xpds_p_ss_categoryMid        0.063409386 0.02597197
## Xrace.ethnicity.5levelBlack -0.004457115 0.05082334
## Xrace.ethnicity.5levelMixed  0.090663707 0.04854067
## Xrace.ethnicity.5levelOther  0.069596400 0.03557028
## Xrace.ethnicity.5levelWhite  0.130279855 0.06412419
## Xinterview_age              -0.002518605 0.02127777
## Xdemo_race_hispanic1        -0.005134106 0.02342844
```

Male participants

```
##
## Family: gaussian
## Link function: identity
##
## Formula:
## cbcl_scr_syn_anxdep_r ~ hormone_scr_ert_mean + pds_p_ss_category +
##     race.ethnicity.5level + interview_age + demo_race_hispanic
##
## Parametric coefficients:
##                             Estimate Std. Error t value Pr(>|t|)
## (Intercept)                 1.831438   1.046851   1.749  0.08033 .
## hormone_scr_ert_mean       -0.001314   0.004074  -0.322  0.74715
## pds_p_ss_categoryEarly      0.482188   0.145339   3.318  0.00092 ***
## pds_p_ss_categoryLate       0.593810   0.895441   0.663  0.50729
## pds_p_ss_categoryMid        0.489201   0.291920   1.676  0.09389 .
## race.ethnicity.5levelBlack  0.670774   0.432012   1.553  0.12062
## race.ethnicity.5levelMixed  1.139091   0.431100   2.642  0.00828 **
## race.ethnicity.5levelOther  1.041831   0.495854   2.101  0.03573 *
## race.ethnicity.5levelWhite  1.042752   0.403392   2.585  0.00979 **
## interview_age              -0.003598   0.008269  -0.435  0.66348
## demo_race_hispanic1         0.106813   0.173601   0.615  0.53842
## ---
## Signif. codes:  0 '***' 0.001 '**' 0.01 '*' 0.05 '.' 0.1 ' ' 1
```

```
##
##
## R-sq.(adj) =  0.00467
## lmer.REML =  13499  Scale est. = 7.1632    n = 2651
```

```
##                                  stdcoef      stdse
## X(Intercept)                 0.000000000 0.00000000
## Xhormone_scr_ert_mean       -0.006572939 0.02038550
## Xpds_p_ss_categoryEarly      0.067339351 0.02029710
## Xpds_p_ss_categoryLate       0.012880212 0.01942282
## Xpds_p_ss_categoryMid        0.034135803 0.02036979
## Xrace.ethnicity.5levelBlack  0.076118506 0.04902415
## Xrace.ethnicity.5levelMixed  0.120876947 0.04574704
## Xrace.ethnicity.5levelOther  0.071627242 0.03409059
## Xrace.ethnicity.5levelWhite  0.159381710 0.06165740
## Xinterview_age              -0.008725106 0.02005045
## Xdemo_race_hispanic1         0.013715075 0.02229085
```

1.17 Model: CBCL Withdrawn-Depressed ~ Testosterone + PDS

Female participants

```
##
## Family: gaussian
## Link function: identity
##
## Formula:
## cbcl_scr_syn_withdep_r ~ hormone_scr_ert_mean + PDS_score + race.ethnicity.5level +
##     interview_age + demo_race_hispanic
##
## Parametric coefficients:
##                              Estimate Std. Error t value Pr(>|t|)
## (Intercept)                 3.556e-01  5.525e-01   0.644 0.519837
## hormone_scr_ert_mean       -5.752e-04  2.105e-03  -0.273 0.784659
## PDS_score                   1.833e-01  4.913e-02   3.731 0.000195 ***
## race.ethnicity.5levelBlack  1.587e-01  2.279e-01   0.696 0.486274
## race.ethnicity.5levelMixed  3.788e-01  2.266e-01   1.672 0.094735 .
## race.ethnicity.5levelOther  5.243e-01  2.604e-01   2.013 0.044193 *
## race.ethnicity.5levelWhite  2.268e-01  2.122e-01   1.069 0.285173
## interview_age              -1.613e-05  4.427e-03  -0.004 0.997094
## demo_race_hispanic1         1.384e-01  9.207e-02   1.503 0.132878
## ---
## Signif. codes:  0 '***' 0.001 '**' 0.01 '*' 0.05 '.' 0.1 ' ' 1
##
##
## R-sq.(adj) =  0.00942
## lmer.REML = 9195.4  Scale est. = 1.6114    n = 2453
```

```
##                                    stdcoef      stdse
## X(Intercept)                 0.000000e+00 0.00000000
## Xhormone_scr_ert_mean       -5.922691e-03 0.02167236
## XPDS_score                   8.424189e-02 0.02257935
## Xrace.ethnicity.5levelBlack  3.523631e-02 0.05060126
```

```
## Xrace.ethnicity.5levelMixed  8.093960e-02 0.04842092
## Xrace.ethnicity.5levelOther  7.165441e-02 0.03559062
## Xrace.ethnicity.5levelWhite  6.832264e-02 0.06391230
## Xinterview_age              -7.702846e-05 0.02114834
## Xdemo_race_hispanic1         3.473852e-02 0.02310743
```

Male participants

```
##
## Family: gaussian
## Link function: identity
##
## Formula:
## cbcl_scr_syn_withdep_r ~ hormone_scr_ert_mean + PDS_score + race.ethnicity.5level +
##     interview_age + demo_race_hispanic
##
## Parametric coefficients:
##                              Estimate Std. Error t value Pr(>|t|)
## (Intercept)                 0.3727478  0.5767911   0.646   0.5182
## hormone_scr_ert_mean        0.0007553  0.0022548   0.335   0.7377
## PDS_score                   0.2171594  0.0659443   3.293   0.0010 **
## race.ethnicity.5levelBlack  0.5881850  0.2385540   2.466   0.0137 *
## race.ethnicity.5levelMixed  0.6307270  0.2394854   2.634   0.0085 **
## race.ethnicity.5levelOther  0.4227457  0.2756505   1.534   0.1252
## race.ethnicity.5levelWhite  0.4077449  0.2229245   1.829   0.0675 .
## interview_age              -0.0005025  0.0046086  -0.109   0.9132
## demo_race_hispanic1         0.0411906  0.0911900   0.452   0.6515
## ---
## Signif. codes:  0 '***' 0.001 '**' 0.01 '*' 0.05 '.' 0.1 ' ' 1
##
##
## R-sq.(adj) =  0.00735
## lmer.REML =  10433  Scale est. = 2.241     n = 2651
```

```
##                                  stdcoef      stdse
## X(Intercept)                 0.000000000 0.00000000
## Xhormone_scr_ert_mean        0.006757970 0.02017453
## XPDS_score                   0.068007915 0.02065180
## Xrace.ethnicity.5levelBlack  0.119357650 0.04840866
## Xrace.ethnicity.5levelMixed  0.119687573 0.04544506
## Xrace.ethnicity.5levelOther  0.051973569 0.03388927
## Xrace.ethnicity.5levelWhite  0.111447014 0.06093092
## Xinterview_age              -0.002178819 0.01998401
## Xdemo_race_hispanic1         0.009457890 0.02093838
```

1.18 Model: CBCL Withdrawn-Depressed ~ Testosterone + Pubertal category

Female participants

```
##
## Family: gaussian
## Link function: identity
```

```
##
## Formula:
## cbcl_scr_syn_withdep_r ~ hormone_scr_ert_mean + pds_p_ss_category +
##     race.ethnicity.5level + interview_age + demo_race_hispanic
##
## Parametric coefficients:
##                            Estimate Std. Error t value Pr(>|t|)
## (Intercept)                0.686454   0.562795   1.220  0.22269
## hormone_scr_ert_mean      -0.000543   0.002084  -0.261  0.79440
## pds_p_ss_categoryEarly     0.223344   0.086901   2.570  0.01023 *
## pds_p_ss_categoryLate      0.686797   0.226687   3.030  0.00247 **
## pds_p_ss_categoryMid       0.353466   0.083518   4.232  2.4e-05 ***
## race.ethnicity.5levelBlack  0.155767   0.227619   0.684  0.49383
## race.ethnicity.5levelMixed  0.389973   0.226276   1.723  0.08494 .
## race.ethnicity.5levelOther  0.529753   0.259864   2.039  0.04160 *
## race.ethnicity.5levelWhite  0.241722   0.211807   1.141  0.25388
## interview_age             -0.002133   0.004488  -0.475  0.63468
## demo_race_hispanic1        0.112749   0.092161   1.223  0.22130
## ---
## Signif. codes:  0 '***' 0.001 '**' 0.01 '*' 0.05 '.' 0.1 ' ' 1
##
##
## R-sq.(adj) =  0.0118
## lmer.REML = 9191.5  Scale est. = 1.5977    n = 2453
```

```
##                                    stdcoef      stdse
## X(Intercept)                  0.000000000 0.00000000
## Xhormone_scr_ert_mean        -0.005591769 0.02145476
## Xpds_p_ss_categoryEarly       0.060730167 0.02362957
## Xpds_p_ss_categoryLate        0.064744089 0.02136966
## Xpds_p_ss_categoryMid         0.110319132 0.02606658
## Xrace.ethnicity.5levelBlack  0.034588750 0.05054392
## Xrace.ethnicity.5levelMixed  0.083337296 0.04835525
## Xrace.ethnicity.5levelOther  0.072405969 0.03551784
## Xrace.ethnicity.5levelWhite  0.072816912 0.06380542
## Xinterview_age               -0.010188454 0.02144014
## Xdemo_race_hispanic1          0.028298439 0.02313107
```

Male participants

```
##
## Family: gaussian
## Link function: identity
##
## Formula:
## cbcl_scr_syn_withdep_r ~ hormone_scr_ert_mean + pds_p_ss_category +
##     race.ethnicity.5level + interview_age + demo_race_hispanic
##
## Parametric coefficients:
##                            Estimate Std. Error t value Pr(>|t|)
## (Intercept)               0.5150833  0.5811894   0.886  0.37556
## hormone_scr_ert_mean      0.0010364  0.0022494   0.461  0.64501
## pds_p_ss_categoryEarly    0.1520206  0.0812850   1.870  0.06156 .
```

```
## pds_p_ss_categoryLate      0.0544412  0.5016980   0.109  0.91360
## pds_p_ss_categoryMid       0.4611184  0.1634376   2.821  0.00482 **
## race.ethnicity.5levelBlack 0.5902341  0.2389072   2.471  0.01355 *
## race.ethnicity.5levelMixed 0.6337494  0.2396387   2.645  0.00823 **
## race.ethnicity.5levelOther 0.4217610  0.2759530   1.528  0.12654
## race.ethnicity.5levelWhite 0.4137111  0.2230677   1.855  0.06376 .
## interview_age              0.0001616  0.0046033   0.035  0.97200
## demo_race_hispanic1        0.0322896  0.0916169   0.352  0.72454
## ---
## Signif. codes:  0 '***' 0.001 '**' 0.01 '*' 0.05 '.' 0.1 ' ' 1
##
##
## R-sq.(adj) =  0.00641
## lmer.REML =  10435  Scale est. = 2.2583    n = 2651
```

```
##                                  stdcoef      stdse
## X(Intercept)                0.0000000000 0.00000000
## Xhormone_scr_ert_mean       0.0092731138 0.02012577
## Xpds_p_ss_categoryEarly     0.0379644485 0.02029949
## Xpds_p_ss_categoryLate      0.0021116679 0.01945987
## Xpds_p_ss_categoryMid       0.0575384257 0.02039376
## Xrace.ethnicity.5levelBlack 0.1197734586 0.04848034
## Xrace.ethnicity.5levelMixed 0.1202611086 0.04547416
## Xrace.ethnicity.5levelOther 0.0518525047 0.03392646
## Xrace.ethnicity.5levelWhite 0.1130777430 0.06097005
## Xinterview_age              0.0007007875 0.01996094
## Xdemo_race_hispanic1        0.0074141019 0.02103640
```

1.19 Model: CBCL Depressed DSM-5 ~ Testosterone + PDS

Female participants

```
##
## Family: gaussian
## Link function: identity
##
## Formula:
## cbcl_scr_dsm5_depress_r ~ hormone_scr_ert_mean + PDS_score +
##     race.ethnicity.5level + interview_age + demo_race_hispanic
##
## Parametric coefficients:
##                              Estimate Std. Error t value Pr(>|t|)
## (Intercept)                 0.2843730  0.6470054   0.440  0.66032
## hormone_scr_ert_mean       -0.0003191  0.0024663  -0.129  0.89707
## PDS_score                   0.1832904  0.0577771   3.172  0.00153 **
## race.ethnicity.5levelBlack  0.1673917  0.2681722   0.624  0.53256
## race.ethnicity.5levelMixed  0.6327813  0.2671638   2.369  0.01794 *
## race.ethnicity.5levelOther  0.8149754  0.3072112   2.653  0.00803 **
## race.ethnicity.5levelWhite  0.5234749  0.2496598   2.097  0.03612 *
## interview_age               0.0003987  0.0051849   0.077  0.93870
## demo_race_hispanic1         0.0621108  0.1074870   0.578  0.56342
## ---
## Signif. codes:  0 '***' 0.001 '**' 0.01 '*' 0.05 '.' 0.1 ' ' 1
```

```
##
##
## R-sq.(adj) =  0.00875
## lmer.REML = 9983.8  Scale est. = 1.7485    n = 2453
```

```
##                                 stdcoef      stdse
## X(Intercept)                 0.000000000 0.00000000
## Xhormone_scr_ert_mean       -0.002775311 0.02145079
## XPDS_score                   0.071157860 0.02243049
## Xrace.ethnicity.5levelBlack  0.031396751 0.05029959
## Xrace.ethnicity.5levelMixed  0.114221486 0.04822494
## Xrace.ethnicity.5levelOther  0.094088220 0.03546727
## Xrace.ethnicity.5levelWhite  0.133199563 0.06352658
## Xinterview_age               0.001608878 0.02092015
## Xdemo_race_hispanic1         0.013167622 0.02278749
```

Male participants

```
##
## Family: gaussian
## Link function: identity
##
## Formula:
## cbcl_scr_dsm5_depress_r ~ hormone_scr_ert_mean + PDS_score +
##     race.ethnicity.5level + interview_age + demo_race_hispanic
##
## Parametric coefficients:
##                              Estimate Std. Error t value Pr(>|t|)
## (Intercept)                 0.4909365  0.7131053   0.688 0.491231
## hormone_scr_ert_mean       -0.0002916  0.0027999  -0.104 0.917070
## PDS_score                   0.2681471  0.0813222   3.297 0.000989 ***
## race.ethnicity.5levelBlack  0.4893579  0.2957950   1.654 0.098169 .
## race.ethnicity.5levelMixed  0.6794781  0.2959397   2.296 0.021754 *
## race.ethnicity.5levelOther  0.4969081  0.3407400   1.458 0.144871
## race.ethnicity.5levelWhite  0.5039662  0.2763480   1.824 0.068316 .
## interview_age               0.0003636  0.0056900   0.064 0.949058
## demo_race_hispanic1        -0.0390134  0.1169369  -0.334 0.738687
## ---
## Signif. codes:  0 '***' 0.001 '**' 0.01 '*' 0.05 '.' 0.1 ' ' 1
##
##
## R-sq.(adj) =  0.00329
## lmer.REML =  11523  Scale est. = 2.9467    n = 2651
```

```
##                                 stdcoef      stdse
## X(Intercept)                 0.000000000 0.00000000
## Xhormone_scr_ert_mean       -0.002121083 0.02036850
## XPDS_score                   0.068277888 0.02070695
## Xrace.ethnicity.5levelBlack  0.080740050 0.04880376
## Xrace.ethnicity.5levelMixed  0.104835714 0.04566012
## Xrace.ethnicity.5levelOther  0.049671305 0.03406063
## Xrace.ethnicity.5levelWhite  0.111997288 0.06141329
## Xinterview_age               0.001281818 0.02006094
## Xdemo_race_hispanic1        -0.007283433 0.02183101
```

1.20 Model: CBCL Depressed DSM-5 ~ Testosterone + Pubertal category

Female participants

```
##
## Family: gaussian
## Link function: identity
##
## Formula:
## cbcl_scr_dsm5_depress_r ~ hormone_scr_ert_mean + pds_p_ss_category +
##     race.ethnicity.5level + interview_age + demo_race_hispanic
##
## Parametric coefficients:
##                              Estimate Std. Error t value Pr(>|t|)
## (Intercept)                 0.5842775  0.6591968   0.886  0.37552
## hormone_scr_ert_mean       -0.0002682  0.0024428  -0.110  0.91259
## pds_p_ss_categoryEarly      0.2207987  0.1019876   2.165  0.03049 *
## pds_p_ss_categoryLate       0.4844084  0.2650177   1.828  0.06770 .
## pds_p_ss_categoryMid        0.3871347  0.0983396   3.937 8.49e-05 ***
## race.ethnicity.5levelBlack  0.1527621  0.2682545   0.569  0.56909
## race.ethnicity.5levelMixed  0.6344235  0.2670693   2.376  0.01760 *
## race.ethnicity.5levelOther  0.8202047  0.3068257   2.673  0.00756 **
## race.ethnicity.5levelWhite  0.5327023  0.2495551   2.135  0.03289 *
## interview_age              -0.0015013  0.0052556  -0.286  0.77516
## demo_race_hispanic1         0.0432026  0.1078722   0.400  0.68882
## ---
## Signif. codes:  0 '***' 0.001 '**' 0.01 '*' 0.05 '.' 0.1 ' ' 1
##
##
## R-sq.(adj) =  0.0101
## lmer.REML = 9980.8  Scale est. = 1.7325    n = 2453
```

```
##                                  stdcoef      stdse
## X(Intercept)                 0.000000000 0.00000000
## Xhormone_scr_ert_mean       -0.002332619 0.02124622
## Xpds_p_ss_categoryEarly      0.050712757 0.02342438
## Xpds_p_ss_categoryLate       0.038572117 0.02110263
## Xpds_p_ss_categoryMid        0.102059963 0.02592519
## Xrace.ethnicity.5levelBlack  0.028652756 0.05031503
## Xrace.ethnicity.5levelMixed  0.114517906 0.04820789
## Xrace.ethnicity.5levelOther  0.094691946 0.03542276
## Xrace.ethnicity.5levelWhite  0.135547491 0.06349994
## Xinterview_age              -0.006057651 0.02120532
## Xdemo_race_hispanic1         0.009159043 0.02286914
```

Male participants

```
##
## Family: gaussian
## Link function: identity
##
## Formula:
## cbcl_scr_dsm5_depress_r ~ hormone_scr_ert_mean + pds_p_ss_category +
```

```
##     race.ethnicity.5level + interview_age + demo_race_hispanic
##
## Parametric coefficients:
##                             Estimate Std. Error t value Pr(>|t|)
## (Intercept)                7.018e-01  7.183e-01   0.977  0.32867
## hormone_scr_ert_mean       1.921e-05  2.793e-03   0.007  0.99451
## pds_p_ss_categoryEarly     2.543e-01  1.000e-01   2.542  0.01108 *
## pds_p_ss_categoryLate      5.955e-02  6.154e-01   0.097  0.92292
## pds_p_ss_categoryMid       5.284e-01  2.014e-01   2.624  0.00875 **
## race.ethnicity.5levelBlack  4.865e-01  2.961e-01   1.643  0.10052
## race.ethnicity.5levelMixed  6.853e-01  2.961e-01   2.315  0.02069 *
## race.ethnicity.5levelOther  5.026e-01  3.410e-01   1.474  0.14071
## race.ethnicity.5levelWhite  5.145e-01  2.764e-01   1.861  0.06284 .
## interview_age              7.681e-04  5.683e-03   0.135  0.89251
## demo_race_hispanic1       -5.352e-02  1.174e-01  -0.456  0.64841
## ---
## Signif. codes:  0 '***' 0.001 '**' 0.01 '*' 0.05 '.' 0.1 ' ' 1
##
##
## R-sq.(adj) =  0.00269
## lmer.REML =  11523  Scale est. = 2.9327    n = 2651
```

```
##                                  stdcoef      stdse
## X(Intercept)                 0.0000000000 0.00000000
## Xhormone_scr_ert_mean        0.0001397591 0.02031649
## Xpds_p_ss_categoryEarly      0.0516292897 0.02031036
## Xpds_p_ss_categoryLate       0.0018780272 0.01940858
## Xpds_p_ss_categoryMid        0.0536036605 0.02043070
## Xrace.ethnicity.5levelBlack  0.0802724647 0.04885966
## Xrace.ethnicity.5levelMixed  0.1057399707 0.04567799
## Xrace.ethnicity.5levelOther  0.0502374363 0.03409148
## Xrace.ethnicity.5levelWhite  0.1143273575 0.06142974
## Xinterview_age               0.0027079279 0.02003763
## Xdemo_race_hispanic1        -0.0099909238 0.02190857
```

2—Reward~Puberty—

2.1 Model: BIS-BAS-RR ~ PDS

Female participants

```
##
## Family: gaussian
## Link function: identity
##
## Formula:
## bisbas_ss_basm_rr_z ~ PDS_score + interview_age
##
## Parametric coefficients:
##                Estimate Std. Error t value Pr(>|t|)
## (Intercept)    0.395174   0.306057   1.291  0.19675
## PDS_score      0.074580   0.027050   2.757  0.00587 **
## interview_age -0.004766   0.002626  -1.814  0.06972 .
## ---
## Signif. codes:  0 '***' 0.001 '**' 0.01 '*' 0.05 '.' 0.1 ' ' 1
##
##
## R-sq.(adj) =  0.00449
## lmer.REML = 7544.9  Scale est. = 0.75244   n = 2690
```

```
##                    stdcoef      stdse
## X(Intercept)    0.00000000 0.00000000
## XPDS_score      0.05558130 0.02015902
## Xinterview_age -0.03622943 0.01996716
```

Male participants

```
##
## Family: gaussian
## Link function: identity
##
## Formula:
## bisbas_ss_basm_rr_z ~ PDS_score + interview_age
##
## Parametric coefficients:
##                Estimate Std. Error t value Pr(>|t|)
## (Intercept)    0.164447   0.289455   0.568  0.56999
## PDS_score      0.091023   0.033987   2.678  0.00744 **
## interview_age -0.001822   0.002450  -0.743  0.45724
## ---
## Signif. codes:  0 '***' 0.001 '**' 0.01 '*' 0.05 '.' 0.1 ' ' 1
##
##
## R-sq.(adj) =  0.00265
## lmer.REML = 8052.9  Scale est. = 0.7399    n = 2907
```

```
##                    stdcoef      stdse
```

```
## X(Intercept)    0.00000000 0.00000000
## XPDS_score      0.05105448 0.01906309
## Xinterview_age -0.01407487 0.01893078
```

2.2 Model : Reaction Time ~ PDS

Female participants

```
##
## Family: gaussian
## Link function: identity
##
## Formula:
## rt_diff_large_neutral_z ~ PDS_score + interview_age
##
## Parametric coefficients:
##                Estimate Std. Error t value Pr(>|t|)
## (Intercept)   -0.572358   0.316549  -1.808   0.0707 .
## PDS_score     -0.020889   0.028535  -0.732   0.4642
## interview_age  0.005457   0.002728   2.000   0.0456 *
## ---
## Signif. codes:  0 '***' 0.001 '**' 0.01 '*' 0.05 '.' 0.1 ' ' 1
##
##
## R-sq.(adj) =  0.00104
## lmer.REML =   5938  Scale est. = 0.67938   n = 2201
```

```
##                    stdcoef      stdse
## X(Intercept)    0.00000000 0.00000000
## XPDS_score     -0.01620865 0.02214117
## Xinterview_age  0.04407552 0.02203886
```

```
##
## Family: gaussian
## Link function: identity
##
## Formula:
## rt_diff_large_small_z ~ PDS_score + interview_age
##
## Parametric coefficients:
##                Estimate Std. Error t value Pr(>|t|)
## (Intercept)   -0.349121   0.318183  -1.097    0.273
## PDS_score     -0.026958   0.028604  -0.942    0.346
## interview_age  0.003428   0.002742   1.250    0.211
##
##
## R-sq.(adj) =  0.000134
## lmer.REML = 5963.2  Scale est. = 0.77188   n = 2201
```

```
##                    stdcoef      stdse
## X(Intercept)    0.00000000 0.00000000
## XPDS_score     -0.02082256 0.02209392
## Xinterview_age  0.02756625 0.02204520
```

Male participants

```
##
## Family: gaussian
## Link function: identity
##
## Formula:
## rt_diff_large_neutral_z ~ PDS_score + interview_age
##
## Parametric coefficients:
##                 Estimate Std. Error t value Pr(>|t|)
## (Intercept)    0.1111430  0.2929755   0.379   0.7045
## PDS_score     -0.0632857  0.0355353  -1.781   0.0751 .
## interview_age -0.0003222  0.0024828  -0.130   0.8968
## ---
## Signif. codes:  0 '***' 0.001 '**' 0.01 '*' 0.05 '.' 0.1 ' ' 1
##
##
## R-sq.(adj) =  0.000741
## lmer.REML = 5939.1  Scale est. = 0.71204   n = 2297
```

```
##                     stdcoef      stdse
## X(Intercept)    0.000000000 0.00000000
## XPDS_score     -0.037980395 0.02132623
## Xinterview_age -0.002759926 0.02126704
```

```
##
## Family: gaussian
## Link function: identity
##
## Formula:
## rt_diff_large_small_z ~ PDS_score + interview_age
##
## Parametric coefficients:
##                 Estimate Std. Error t value Pr(>|t|)
## (Intercept)    0.0232816  0.2965386   0.079    0.937
## PDS_score     -0.0239408  0.0358885  -0.667    0.505
## interview_age  0.0001343  0.0025139   0.053    0.957
##
##
## R-sq.(adj) =  -0.000678
## lmer.REML =   6016  Scale est. = 0.79698   n = 2297
```

```
##                     stdcoef      stdse
## X(Intercept)    0.000000000 0.00000000
## XPDS_score     -0.014143995 0.02120258
## Xinterview_age  0.001132671 0.02119757
```

2.3 Model: Caudate Anticipation ~ PDS

Female participants

```
##
```

```
## Family: gaussian
## Link function: identity
##
## Formula:
## caudate_rvsn_ant_z ~ PDS_score + interview_age
##
## Parametric coefficients:
##                Estimate Std. Error t value Pr(>|t|)
## (Intercept)   -0.483420   0.318838  -1.516   0.1296
## PDS_score     -0.049471   0.028595  -1.730   0.0838 .
## interview_age  0.004869   0.002743   1.775   0.0760 .
## ---
## Signif. codes:  0 '***' 0.001 '**' 0.01 '*' 0.05 '.' 0.1 ' ' 1
##
##
## R-sq.(adj) =  0.00193
## lmer.REML = 5350.3  Scale est. = 0.77536   n = 2044
```

```
##                    stdcoef      stdse
## X(Intercept)    0.00000000 0.00000000
## XPDS_score     -0.03986291 0.02304081
## Xinterview_age  0.04069642 0.02292625
```

Male participants

```
##
## Family: gaussian
## Link function: identity
##
## Formula:
## caudate_rvsn_ant_z ~ PDS_score + interview_age
##
## Parametric coefficients:
##                Estimate Std. Error t value Pr(>|t|)
## (Intercept)   -0.186131   0.341694  -0.545    0.586
## PDS_score     -0.008219   0.041635  -0.197    0.844
## interview_age  0.001612   0.002897   0.556    0.578
##
##
## R-sq.(adj) =  -0.000776
## lmer.REML = 5730.1  Scale est. = 0.78555   n = 2060
```

```
##                     stdcoef      stdse
## X(Intercept)    0.000000000 0.00000000
## XPDS_score     -0.004462425 0.02260454
## Xinterview_age  0.012518023 0.02249980
```

2.4 Model B: Putamen Anticipation ~ PDS

Female participants

```
##
```

```
## Family: gaussian
## Link function: identity
##
## Formula:
## putamen_rvsn_ant_z ~ PDS_score + interview_age
##
## Parametric coefficients:
##                Estimate Std. Error t value Pr(>|t|)
## (Intercept)   -0.372726   0.310423  -1.201  0.23001
## PDS_score     -0.077949   0.027849  -2.799  0.00517 **
## interview_age  0.004245   0.002670   1.590  0.11202
## ---
## Signif. codes:  0 '***' 0.001 '**' 0.01 '*' 0.05 '.' 0.1 ' ' 1
##
##
## R-sq.(adj) =  0.00378
## lmer.REML = 5233.6  Scale est. = 0.73005   n = 2041
```

```
##                    stdcoef      stdse
## X(Intercept)    0.00000000 0.00000000
## XPDS_score     -0.06444037 0.02302285
## Xinterview_age  0.03643015 0.02291392
```

Male participants

```
##
## Family: gaussian
## Link function: identity
##
## Formula:
## putamen_rvsn_ant_z ~ PDS_score + interview_age
##
## Parametric coefficients:
##                Estimate Std. Error t value Pr(>|t|)
## (Intercept)   -0.422958   0.329720  -1.283    0.200
## PDS_score      0.005677   0.040377   0.141    0.888
## interview_age  0.003507   0.002797   1.254    0.210
##
##
## R-sq.(adj) =  -9.18e-05
## lmer.REML = 5571.2  Scale est. = 0.85641   n = 2057
```

```
##                    stdcoef      stdse
## X(Intercept)   0.000000000 0.00000000
## XPDS_score     0.003177134 0.02259754
## Xinterview_age 0.028240686 0.02252802
```

2.5 Model: Accumbens Anticipation ~ PDS

Female participants

```
##
```

```
## Family: gaussian
## Link function: identity
##
## Formula:
## accumbens_rvsn_ant_z ~ PDS_score + interview_age
##
## Parametric coefficients:
##                 Estimate Std. Error t value Pr(>|t|)
## (Intercept)   -0.0968160  0.2446591  -0.396    0.692
## PDS_score     -0.0008552  0.0219117  -0.039    0.969
## interview_age  0.0009134  0.0021051   0.434    0.664
##
##
## R-sq.(adj) =  -0.000795
## lmer.REML = 4276.4  Scale est. = 0.44122   n = 2044
```

```
##                      stdcoef      stdse
## X(Intercept)    0.0000000000 0.00000000
## XPDS_score     -0.0009005018 0.02307352
## Xinterview_age  0.0099580823 0.02295055
```

Male participants

```
##
## Family: gaussian
## Link function: identity
##
## Formula:
## accumbens_rvsn_ant_z ~ PDS_score + interview_age
##
## Parametric coefficients:
##                Estimate Std. Error t value Pr(>|t|)
## (Intercept)    0.324185   0.256773   1.263    0.207
## PDS_score      0.004833   0.031187   0.155    0.877
## interview_age -0.002717   0.002178  -1.247    0.212
##
##
## R-sq.(adj) =  -0.000206
## lmer.REML = 4574.9  Scale est. = 0.51375   n = 2059
```

```
##                    stdcoef      stdse
## X(Intercept)    0.00000000 0.00000000
## XPDS_score      0.00347335 0.02241537
## Xinterview_age -0.02794604 0.02240798
```

2.6 Model: Caudate Feedback ~ PDS

Female participants

```
##
## Family: gaussian
## Link function: identity
```

```
##
## Formula:
## caudate_posvsneg_feedback_z ~ PDS_score + interview_age
##
## Parametric coefficients:
##                Estimate Std. Error t value Pr(>|t|)
## (Intercept)    0.879700   0.304751   2.887  0.00394 **
## PDS_score     -0.021140   0.027194  -0.777  0.43703
## interview_age -0.007289   0.002625  -2.777  0.00553 **
## ---
## Signif. codes:  0 '***' 0.001 '**' 0.01 '*' 0.05 '.' 0.1 ' ' 1
##
##
## R-sq.(adj) =  0.00389
## lmer.REML = 5192.5  Scale est. = 0.73778   n = 2042
```

```
##                    stdcoef      stdse
## X(Intercept)    0.00000000 0.00000000
## XPDS_score     -0.01771233 0.02278498
## Xinterview_age -0.06327810 0.02278498
```

Male participants

```
##
## Family: gaussian
## Link function: identity
##
## Formula:
## caudate_posvsneg_feedback_z ~ PDS_score + interview_age
##
## Parametric coefficients:
##                Estimate Std. Error t value Pr(>|t|)
## (Intercept)   -0.035148   0.308762  -0.114   0.9094
## PDS_score     -0.077997   0.037515  -2.079   0.0377 *
## interview_age  0.001494   0.002619   0.570   0.5686
## ---
## Signif. codes:  0 '***' 0.001 '**' 0.01 '*' 0.05 '.' 0.1 ' ' 1
##
##
## R-sq.(adj) =  0.00119
## lmer.REML = 5321.7  Scale est. = 0.77007   n = 2058
```

```
##                    stdcoef      stdse
## X(Intercept)    0.00000000 0.00000000
## XPDS_score     -0.04660337 0.02241512
## Xinterview_age  0.01277557 0.02240242
```

2.7 Model: Putamen Feedback ~ PDS

Female participants

```
##
```

```
## Family: gaussian
## Link function: identity
##
## Formula:
## putamen_posvsneg_feedback_z ~ PDS_score + interview_age
##
## Parametric coefficients:
##                Estimate Std. Error t value Pr(>|t|)
## (Intercept)    0.553835   0.291446   1.900   0.0575 .
## PDS_score      0.005590   0.026008   0.215   0.8298
## interview_age -0.005130   0.002509  -2.044   0.0410 *
## ---
## Signif. codes:  0 '***' 0.001 '**' 0.01 '*' 0.05 '.' 0.1 ' ' 1
##
##
## R-sq.(adj) =  0.00101
## lmer.REML = 5000.7  Scale est. = 0.67013   n = 2042
```

```
##                     stdcoef      stdse
## X(Intercept)    0.000000000 0.00000000
## XPDS_score      0.004926153 0.02291867
## Xinterview_age -0.046761914 0.02287379
```

Male participants

```
##
## Family: gaussian
## Link function: identity
##
## Formula:
## putamen_posvsneg_feedback_z ~ PDS_score + interview_age
##
## Parametric coefficients:
##                 Estimate Std. Error t value Pr(>|t|)
## (Intercept)    0.2523532  0.3084372   0.818    0.413
## PDS_score     -0.0586858  0.0374986  -1.565    0.118
## interview_age -0.0007996  0.0026095  -0.306    0.759
##
##
## R-sq.(adj) =  0.000261
## lmer.REML = 5293.1  Scale est. = 0.75008   n = 2061
```

```
##                     stdcoef      stdse
## X(Intercept)    0.000000000 0.00000000
## XPDS_score     -0.035327960 0.02257361
## Xinterview_age -0.006884135 0.02246763
```

2.8 Model: Accumbens Feedback ~ PDS

Female participants

```
##
```

```
## Family: gaussian
## Link function: identity
##
## Formula:
## accumbens_posvsneg_feedback_z ~ PDS_score + interview_age
##
## Parametric coefficients:
##                Estimate Std. Error t value Pr(>|t|)
## (Intercept)    0.457768   0.230801   1.983   0.0475 *
## PDS_score     -0.001013   0.020566  -0.049   0.9607
## interview_age -0.003938   0.001988  -1.981   0.0477 *
## ---
## Signif. codes:  0 '***' 0.001 '**' 0.01 '*' 0.05 '.' 0.1 ' ' 1
##
##
## R-sq.(adj) =  0.00106
## lmer.REML = 4078.6  Scale est. = 0.42369   n = 2050
```

```
##                     stdcoef      stdse
## X(Intercept)    0.000000000 0.00000000
## XPDS_score     -0.001122932 0.02280433
## Xinterview_age -0.045142102 0.02278977
```

Male participants

```
##
## Family: gaussian
## Link function: identity
##
## Formula:
## accumbens_posvsneg_feedback_z ~ PDS_score + interview_age
##
## Parametric coefficients:
##                Estimate Std. Error t value Pr(>|t|)
## (Intercept)   -0.068876   0.248955  -0.277    0.782
## PDS_score     -0.040808   0.030313  -1.346    0.178
## interview_age  0.001428   0.002110   0.677    0.499
##
##
## R-sq.(adj) =  -4.31e-05
## lmer.REML = 4395.9  Scale est. = 0.42192   n = 2054
```

```
##                    stdcoef      stdse
## X(Intercept)    0.00000000 0.00000000
## XPDS_score     -0.03050319 0.02265835
## Xinterview_age  0.01525776 0.02254534
```

2.9 Model: OFC Anticipation ~ PDS

Female participants

```
##
```

```
## Family: gaussian
## Link function: identity
##
## Formula:
## lOFC_rvsn_ant_z ~ PDS_score + interview_age
##
## Parametric coefficients:
##                 Estimate Std. Error t value Pr(>|t|)
## (Intercept)    0.0590386  0.2040969   0.289    0.772
## PDS_score      0.0037308  0.0182241   0.205    0.838
## interview_age -0.0004418  0.0017592  -0.251    0.802
##
##
## R-sq.(adj) =  -0.000933
## lmer.REML = 3536.8  Scale est. = 0.29608   n = 2038

##                     stdcoef      stdse
## X(Intercept)    0.000000000 0.00000000
## XPDS_score      0.004699434 0.02295576
## Xinterview_age -0.005755180 0.02291520

##
## Family: gaussian
## Link function: identity
##
## Formula:
## mOFC_rvsn_ant_z ~ PDS_score + interview_age
##
## Parametric coefficients:
##                Estimate Std. Error t value Pr(>|t|)
## (Intercept)    0.039678   0.234446   0.169    0.866
## PDS_score      0.008097   0.020912   0.387    0.699
## interview_age -0.000431   0.002020  -0.213    0.831
##
##
## R-sq.(adj) =  -0.000901
## lmer.REML = 4110.5  Scale est. = 0.43526   n = 2039

##                     stdcoef      stdse
## X(Intercept)    0.000000000 0.00000000
## XPDS_score      0.008856036 0.02287269
## Xinterview_age -0.004880368 0.02287269
```

Male participants

```
##
## Family: gaussian
## Link function: identity
##
## Formula:
## lOFC_rvsn_ant_z ~ PDS_score + interview_age
##
## Parametric coefficients:
```

```
##                Estimate Std. Error t value Pr(>|t|)
## (Intercept)   -0.222499   0.216526  -1.028    0.304
## PDS_score      0.027361   0.026581   1.029    0.303
## interview_age  0.001621   0.001839   0.882    0.378
##
##
## R-sq.(adj) =  0.000144
## lmer.REML = 3835.2  Scale est. = 0.34288   n = 2053
```

```
##                   stdcoef      stdse
## X(Intercept)   0.00000000 0.00000000
## XPDS_score     0.02328767 0.02262399
## Xinterview_age 0.01987765 0.02254881
```

```
##
## Family: gaussian
## Link function: identity
##
## Formula:
## mOFC_rvsn_ant_z ~ PDS_score + interview_age
##
## Parametric coefficients:
##                 Estimate Std. Error t value Pr(>|t|)
## (Intercept)   -1.019e-01  2.360e-01  -0.432   0.6658
## PDS_score      7.386e-02  2.880e-02   2.565   0.0104 *
## interview_age  2.244e-05  2.005e-03   0.011   0.9911
## ---
## Signif. codes:  0 '***' 0.001 '**' 0.01 '*' 0.05 '.' 0.1 ' ' 1
##
##
## R-sq.(adj) =  0.00234
## lmer.REML = 4188.7  Scale est. = 0.40149   n = 2048
```

```
##                     stdcoef      stdse
## X(Intercept)   0.0000000000 0.00000000
## XPDS_score     0.0577094026 0.02250055
## Xinterview_age 0.0002516722 0.02248097
```

2.10 Model: OFC Feedback ~ PDS

Female participants

```
##
## Family: gaussian
## Link function: identity
##
## Formula:
## lOFC_posvsneg_feedback_z ~ PDS_score + interview_age
##
## Parametric coefficients:
##                Estimate Std. Error t value Pr(>|t|)
## (Intercept)    0.250497   0.179292   1.397    0.163
```

```
## PDS_score      0.009944   0.016003   0.621    0.534
## interview_age -0.002448   0.001545  -1.585    0.113
##
##
## R-sq.(adj) =  0.000286
## lmer.REML = 3018.9  Scale est. = 0.22332   n = 2039
```

```
##                    stdcoef      stdse
## X(Intercept)    0.00000000 0.00000000
## XPDS_score      0.01423897 0.02291448
## Xinterview_age -0.03622689 0.02286299
```

```
##
## Family: gaussian
## Link function: identity
##
## Formula:
## mOFC_posvsneg_feedback_z ~ PDS_score + interview_age
##
## Parametric coefficients:
##                 Estimate Std. Error t value Pr(>|t|)
## (Intercept)    0.0649137  0.2204114   0.295    0.768
## PDS_score      0.0101010  0.0197079   0.513    0.608
## interview_age -0.0007488  0.0018984  -0.394    0.693
##
##
## R-sq.(adj) =  -0.000837
## lmer.REML = 3842.5  Scale est. = 0.34392   n = 2040
```

```
##                     stdcoef      stdse
## X(Intercept)    0.000000000 0.00000000
## XPDS_score      0.011820358 0.02306259
## Xinterview_age -0.009053689 0.02295243
```

Male participants

```
##
## Family: gaussian
## Link function: identity
##
## Formula:
## lOFC_posvsneg_feedback_z ~ PDS_score + interview_age
##
## Parametric coefficients:
##                Estimate Std. Error t value Pr(>|t|)
## (Intercept)   -0.255747   0.195270  -1.310    0.190
## PDS_score      0.008412   0.023793   0.354    0.724
## interview_age  0.002314   0.001657   1.396    0.163
##
##
## R-sq.(adj) =  0.000154
## lmer.REML = 3464.9  Scale est. = 0.30926   n = 2063
```

```
##                    stdcoef      stdse
## X(Intercept)   0.000000000 0.00000000
## XPDS_score     0.007913114 0.02238156
## Xinterview_age 0.031243174 0.02238067
```

```
##
## Family: gaussian
## Link function: identity
##
## Formula:
## mOFC_posvsneg_feedback_z ~ PDS_score + interview_age
##
## Parametric coefficients:
##                 Estimate Std. Error t value Pr(>|t|)
## (Intercept)   -0.0388540  0.2246313  -0.173    0.863
## PDS_score      0.0051199  0.0274503   0.187    0.852
## interview_age  0.0005642  0.0019075   0.296    0.767
##
##
## R-sq.(adj) =  -0.000943
## lmer.REML = 4026.3  Scale est. = 0.31384   n = 2061
```

```
##                    stdcoef      stdse
## X(Intercept)   0.000000000 0.00000000
## XPDS_score     0.004195959 0.02249664
## Xinterview_age 0.006638855 0.02244611
```

2.11 Model: Caudate Anticipation ~ Testosterone

Female participants

```
##
## Family: gaussian
## Link function: identity
##
## Formula:
## caudate_rvsn_ant_z ~ hormone_scr_ert_mean + interview_age
##
## Parametric coefficients:
##                       Estimate Std. Error t value Pr(>|t|)
## (Intercept)          -0.531423   0.328131  -1.620   0.1055
## hormone_scr_ert_mean -0.001464   0.001304  -1.122   0.2618
## interview_age         0.004989   0.002807   1.777   0.0757 .
## ---
## Signif. codes:  0 '***' 0.001 '**' 0.01 '*' 0.05 '.' 0.1 ' ' 1
##
##
## R-sq.(adj) =  0.0012
## lmer.REML = 5021.6  Scale est. = 0.79211   n = 1912
```

```
##                           stdcoef      stdse
## X(Intercept)           0.00000000 0.00000000
## Xhormone_scr_ert_mean -0.02656944 0.02367206
## Xinterview_age         0.04181201 0.02352831
```

Male participants

```
##
## Family: gaussian
## Link function: identity
##
## Formula:
## caudate_rvsn_ant_z ~ hormone_scr_ert_mean + interview_age
##
## Parametric coefficients:
##                       Estimate Std. Error t value Pr(>|t|)
## (Intercept)          -0.0749252  0.3460507  -0.217    0.829
## hormone_scr_ert_mean  0.0005271  0.0014419   0.366    0.715
## interview_age         0.0004707  0.0029241   0.161    0.872
##
##
## R-sq.(adj) =  -0.00095
## lmer.REML =   5192  Scale est. = 0.68188   n = 1902
```

```
##                           stdcoef      stdse
## X(Intercept)          0.000000000 0.00000000
## Xhormone_scr_ert_mean 0.008604649 0.02353898
## Xinterview_age        0.003757341 0.02333946
```

2.12 Model B: Putamen Anticipation ~ Testosterone

Female participants

```
##
## Family: gaussian
## Link function: identity
##
## Formula:
## putamen_rvsn_ant_z ~ hormone_scr_ert_mean + interview_age
##
## Parametric coefficients:
##                       Estimate Std. Error t value Pr(>|t|)
## (Intercept)          -0.3478527  0.3189046  -1.091    0.276
## hormone_scr_ert_mean -0.0009987  0.0012676  -0.788    0.431
## interview_age         0.0031484  0.0027281   1.154    0.249
##
##
## R-sq.(adj) =  -5.42e-05
## lmer.REML = 4908.4  Scale est. = 0.74226   n = 1910
```

```
##                           stdcoef      stdse
## X(Intercept)           0.00000000 0.00000000
## Xhormone_scr_ert_mean -0.01866909 0.02369479
## Xinterview_age         0.02718732 0.02355821
```

Male participants

```
##
```

```
## Family: gaussian
## Link function: identity
##
## Formula:
## putamen_rvsn_ant_z ~ hormone_scr_ert_mean + interview_age
##
## Parametric coefficients:
##                       Estimate Std. Error t value Pr(>|t|)
## (Intercept)          -0.363244   0.342533  -1.060    0.289
## hormone_scr_ert_mean  0.002011   0.001432   1.405    0.160
## interview_age         0.002573   0.002891   0.890    0.374
##
##
## R-sq.(adj) =  0.000718
## lmer.REML =   5149  Scale est. = 0.75484   n = 1902
```

```
##                          stdcoef      stdse
## X(Intercept)          0.00000000 0.00000000
## Xhormone_scr_ert_mean 0.03316219 0.02361121
## Xinterview_age        0.02075429 0.02332550
```

2.13 Model: Accumbens Anticipation ~ Testosterone

Female participants

```
##
## Family: gaussian
## Link function: identity
##
## Formula:
## accumbens_rvsn_ant_z ~ hormone_scr_ert_mean + interview_age
##
## Parametric coefficients:
##                        Estimate Std. Error t value Pr(>|t|)
## (Intercept)          -0.1829273  0.2500379  -0.732   0.4645
## hormone_scr_ert_mean -0.0016855  0.0009949  -1.694   0.0904 .
## interview_age         0.0021275  0.0021417   0.993   0.3207
## ---
## Signif. codes:  0 '***' 0.001 '**' 0.01 '*' 0.05 '.' 0.1 ' ' 1
##
##
## R-sq.(adj) =  0.000853
## lmer.REML = 3998.8  Scale est. = 0.43208   n = 1913
```

```
##                           stdcoef      stdse
## X(Intercept)           0.00000000 0.00000000
## Xhormone_scr_ert_mean -0.04006650 0.02364976
## Xinterview_age         0.02337654 0.02353300
```

Male participants

```
##
```

```
## Family: gaussian
## Link function: identity
##
## Formula:
## accumbens_rvsn_ant_z ~ hormone_scr_ert_mean + interview_age
##
## Parametric coefficients:
##                        Estimate Std. Error t value Pr(>|t|)
## (Intercept)           3.226e-01  2.699e-01   1.195    0.232
## hormone_scr_ert_mean -8.252e-05  1.113e-03  -0.074    0.941
## interview_age        -2.616e-03  2.280e-03  -1.148    0.251
##
##
## R-sq.(adj) =  -0.000323
## lmer.REML = 4277.2  Scale est. = 0.50131   n = 1905
```

```
##                            stdcoef      stdse
## X(Intercept)           0.000000000 0.00000000
## Xhormone_scr_ert_mean -0.001723489 0.02325383
## Xinterview_age        -0.026653729 0.02322433
```

2.14 Model: Caudate Feedback ~ Testosterone

Female participants

```
##
## Family: gaussian
## Link function: identity
##
## Formula:
## caudate_posvsneg_feedback_z ~ hormone_scr_ert_mean + interview_age
##
## Parametric coefficients:
##                       Estimate Std. Error t value Pr(>|t|)
## (Intercept)           0.964423   0.313893   3.072 0.002153 **
## hormone_scr_ert_mean  0.002594   0.001242   2.089 0.036870 *
## interview_age        -0.009086   0.002692  -3.375 0.000753 ***
## ---
## Signif. codes:  0 '***' 0.001 '**' 0.01 '*' 0.05 '.' 0.1 ' ' 1
##
##
## R-sq.(adj) =  0.00588
## lmer.REML =   4870  Scale est. = 0.74202   n = 1908
```

```
##                           stdcoef      stdse
## X(Intercept)           0.00000000 0.00000000
## Xhormone_scr_ert_mean  0.04890655 0.02341514
## Xinterview_age        -0.07902547 0.02341514
```

Male participants

```
##
```

```
## Family: gaussian
## Link function: identity
##
## Formula:
## caudate_posvsneg_feedback_z ~ hormone_scr_ert_mean + interview_age
##
## Parametric coefficients:
##                       Estimate Std. Error t value Pr(>|t|)
## (Intercept)           0.0717921  0.3203480   0.224    0.823
## hormone_scr_ert_mean  0.0016157  0.0013266   1.218    0.223
## interview_age        -0.0006652  0.0027075  -0.246    0.806
##
##
## R-sq.(adj) =  -0.000306
## lmer.REML = 4914.7  Scale est. = 0.76342   n = 1903
```

```
##                            stdcoef      stdse
## X(Intercept)           0.000000000 0.00000000
## Xhormone_scr_ert_mean  0.028461130 0.02336805
## Xinterview_age        -0.005716953 0.02326983
```

2.15 Model: Putamen Feedback ~ Testosterone

Female participants

```
##
## Family: gaussian
## Link function: identity
##
## Formula:
## putamen_posvsneg_feedback_z ~ hormone_scr_ert_mean + interview_age
##
## Parametric coefficients:
##                       Estimate Std. Error t value Pr(>|t|)
## (Intercept)           0.612850   0.297927   2.057  0.03982 *
## hormone_scr_ert_mean  0.003420   0.001185   2.887  0.00393 **
## interview_age        -0.006594   0.002555  -2.581  0.00992 **
## ---
## Signif. codes:  0 '***' 0.001 '**' 0.01 '*' 0.05 '.' 0.1 ' ' 1
##
##
## R-sq.(adj) =  0.00534
## lmer.REML = 4670.6  Scale est. = 0.66657   n = 1909
```

```
##                           stdcoef      stdse
## X(Intercept)           0.00000000 0.00000000
## Xhormone_scr_ert_mean  0.06777610 0.02347543
## Xinterview_age        -0.06051217 0.02344254
```

Male participants

```
##
```

```
## Family: gaussian
## Link function: identity
##
## Formula:
## putamen_posvsneg_feedback_z ~ hormone_scr_ert_mean + interview_age
##
## Parametric coefficients:
##                       Estimate Std. Error t value Pr(>|t|)
## (Intercept)           0.400731   0.318183   1.259    0.208
## hormone_scr_ert_mean  0.001473   0.001325   1.112    0.266
## interview_age        -0.003093   0.002680  -1.154    0.248
##
##
## R-sq.(adj) =  -0.00011
## lmer.REML = 4880.1  Scale est. = 0.73977   n = 1907
```

```
##                           stdcoef      stdse
## X(Intercept)           0.00000000 0.00000000
## Xhormone_scr_ert_mean  0.02618158 0.02355376
## Xinterview_age        -0.02681733 0.02322933
```

2.16 Model: Accumbens Feedback ~ Testosterone

Female participants

```
##
## Family: gaussian
## Link function: identity
##
## Formula:
## accumbens_posvsneg_feedback_z ~ hormone_scr_ert_mean + interview_age
##
## Parametric coefficients:
##                        Estimate Std. Error t value Pr(>|t|)
## (Intercept)           0.4856058  0.2310580   2.102   0.0357 *
## hormone_scr_ert_mean  0.0003708  0.0009171   0.404   0.6860
## interview_age        -0.0043599  0.0019815  -2.200   0.0279 *
## ---
## Signif. codes:  0 '***' 0.001 '**' 0.01 '*' 0.05 '.' 0.1 ' ' 1
##
##
## R-sq.(adj) =  0.00144
## lmer.REML =   3720  Scale est. = 0.40205   n = 1916
```

```
##                            stdcoef      stdse
## X(Intercept)           0.000000000 0.00000000
## Xhormone_scr_ert_mean  0.009488918 0.02346939
## Xinterview_age        -0.051581945 0.02344264
```

Male participants

```
##
```

```
## Family: gaussian
## Link function: identity
##
## Formula:
## accumbens_posvsneg_feedback_z ~ hormone_scr_ert_mean + interview_age
##
## Parametric coefficients:
##                        Estimate Std. Error t value Pr(>|t|)
## (Intercept)           0.0489839  0.2610065   0.188    0.851
## hormone_scr_ert_mean  0.0014432  0.0010875   1.327    0.185
## interview_age        -0.0003314  0.0022052  -0.150    0.881
##
##
## R-sq.(adj) =  8.13e-05
## lmer.REML = 4104.6  Scale est. = 0.43257   n = 1899
```

```
##                            stdcoef      stdse
## X(Intercept)           0.000000000 0.00000000
## Xhormone_scr_ert_mean  0.031321924 0.02360226
## Xinterview_age        -0.003511598 0.02336325
```

2.17 Model: OFC Anticipation ~ Testosterone

Female participants

```
##
## Family: gaussian
## Link function: identity
##
## Formula:
## lOFC_rvsn_ant_z ~ hormone_scr_ert_mean + interview_age
##
## Parametric coefficients:
##                        Estimate Std. Error t value Pr(>|t|)
## (Intercept)           0.0032153  0.2104991   0.015    0.988
## hormone_scr_ert_mean -0.0006606  0.0008361  -0.790    0.430
## interview_age         0.0002883  0.0018063   0.160    0.873
##
##
## R-sq.(adj) =  -0.0007
## lmer.REML = 3333.4  Scale est. = 0.30459   n = 1906
```

```
##                            stdcoef      stdse
## X(Intercept)           0.000000000 0.00000000
## Xhormone_scr_ert_mean -0.018620645 0.02356633
## Xinterview_age         0.003757456 0.02354156
```

```
##
## Family: gaussian
## Link function: identity
##
## Formula:
```

```
## mOFC_rvsn_ant_z ~ hormone_scr_ert_mean + interview_age
##
## Parametric coefficients:
##                       Estimate Std. Error t value Pr(>|t|)
## (Intercept)           0.0682507  0.2410390   0.283    0.777
## hormone_scr_ert_mean  0.0001874  0.0009567   0.196    0.845
## interview_age        -0.0006112  0.0020683  -0.295    0.768
##
##
## R-sq.(adj) =  -0.000996
## lmer.REML = 3854.2  Scale est. = 0.43627   n = 1906
```

```
##                           stdcoef      stdse
## X(Intercept)           0.000000000 0.00000000
## Xhormone_scr_ert_mean  0.004606623 0.02352115
## Xinterview_age        -0.006950428 0.02352115
```

Male participants

```
##
## Family: gaussian
## Link function: identity
##
## Formula:
## lOFC_rvsn_ant_z ~ hormone_scr_ert_mean + interview_age
##
## Parametric coefficients:
##                       Estimate Std. Error t value Pr(>|t|)
## (Intercept)          -0.2768395  0.2264293  -1.223   0.2216
## hormone_scr_ert_mean -0.0015935  0.0009445  -1.687   0.0917 .
## interview_age         0.0028287  0.0019125   1.479   0.1393
## ---
## Signif. codes:  0 '***' 0.001 '**' 0.01 '*' 0.05 '.' 0.1 ' ' 1
##
##
## R-sq.(adj) =  0.0012
## lmer.REML = 3579.8  Scale est. = 0.33303   n = 1899
```

```
##                          stdcoef      stdse
## X(Intercept)           0.00000000 0.00000000
## Xhormone_scr_ert_mean -0.03950549 0.02341628
## Xinterview_age         0.03445050 0.02329201
```

```
##
## Family: gaussian
## Link function: identity
##
## Formula:
## mOFC_rvsn_ant_z ~ hormone_scr_ert_mean + interview_age
##
## Parametric coefficients:
##                      Estimate Std. Error t value Pr(>|t|)
## (Intercept)          -0.204876   0.247282  -0.829    0.407
```

```
## hormone_scr_ert_mean -0.001011   0.001023  -0.988    0.323
## interview_age         0.002031   0.002091   0.972    0.331
##
##
## R-sq.(adj) =  -0.000234
## lmer.REML = 3910.2  Scale est. = 0.40564   n = 1895
```

```
##                           stdcoef      stdse
## X(Intercept)           0.00000000 0.00000000
## Xhormone_scr_ert_mean -0.02309696 0.02337852
## Xinterview_age         0.02266259 0.02332311
```

2.18 Model: OFC Feedback ~ Testosterone

Female participants

```
##
## Family: gaussian
## Link function: identity
##
## Formula:
## lOFC_posvsneg_feedback_z ~ hormone_scr_ert_mean + interview_age
##
## Parametric coefficients:
##                        Estimate Std. Error t value Pr(>|t|)
## (Intercept)           0.2773309  0.1834713   1.512   0.1308
## hormone_scr_ert_mean  0.0012000  0.0007282   1.648   0.0995 .
## interview_age        -0.0029118  0.0015740  -1.850   0.0645 .
## ---
## Signif. codes:  0 '***' 0.001 '**' 0.01 '*' 0.05 '.' 0.1 ' ' 1
##
##
## R-sq.(adj) =  0.0017
## lmer.REML = 2821.7  Scale est. = 0.21617   n = 1908
```

```
##                           stdcoef      stdse
## X(Intercept)           0.00000000 0.00000000
## Xhormone_scr_ert_mean  0.03876310 0.02352205
## Xinterview_age        -0.04343638 0.02348011
```

```
##
## Family: gaussian
## Link function: identity
##
## Formula:
## mOFC_posvsneg_feedback_z ~ hormone_scr_ert_mean + interview_age
##
## Parametric coefficients:
##                        Estimate Std. Error t value Pr(>|t|)
## (Intercept)           0.1195496  0.2235176   0.535    0.593
## hormone_scr_ert_mean  0.0007248  0.0008892   0.815    0.415
## interview_age        -0.0012938  0.0019163  -0.675    0.500
```

```
##
##
## R-sq.(adj) =  -0.000459
## lmer.REML = 3559.8  Scale est. = 0.32991   n = 1910
```

```
##                            stdcoef      stdse
## X(Intercept)            0.00000000 0.00000000
## Xhormone_scr_ert_mean  0.01931141 0.02369241
## Xinterview_age        -0.01591504 0.02357242
```

Male participants

```
##
## Family: gaussian
## Link function: identity
##
## Formula:
## lOFC_posvsneg_feedback_z ~ hormone_scr_ert_mean + interview_age
##
## Parametric coefficients:
##                         Estimate Std. Error t value Pr(>|t|)
## (Intercept)           -0.1175909  0.2030853  -0.579    0.563
## hormone_scr_ert_mean  0.0000585  0.0008427   0.069    0.945
## interview_age         0.0012925  0.0017167   0.753    0.452
##
##
## R-sq.(adj) =  -0.000733
## lmer.REML = 3216.5  Scale est. = 0.31125   n = 1909
```

```
##                            stdcoef      stdse
## X(Intercept)           0.000000000 0.00000000
## Xhormone_scr_ert_mean 0.001609679 0.02318664
## Xinterview_age        0.017456649 0.02318664
```

```
##
## Family: gaussian
## Link function: identity
##
## Formula:
## mOFC_posvsneg_feedback_z ~ hormone_scr_ert_mean + interview_age
##
## Parametric coefficients:
##                         Estimate Std. Error t value Pr(>|t|)
## (Intercept)            0.0374550  0.2352774   0.159    0.874
## hormone_scr_ert_mean  0.0007922  0.0009749   0.813    0.417
## interview_age        -0.0001889  0.0019895  -0.095    0.924
##
##
## R-sq.(adj) =  -0.000776
## lmer.REML = 3761.8  Scale est. = 0.32397   n = 1907
```

```
##                             stdcoef      stdse
```

```
## X(Intercept)           0.000000000 0.00000000
## Xhormone_scr_ert_mean  0.018919183 0.02328135
## Xinterview_age        -0.002207377 0.02324932
```

2.19 Model: MID Reaction Time ~ Testosterone

Female participants

```
##
## Family: gaussian
## Link function: identity
##
## Formula:
## rt_diff_large_neutral_z ~ hormone_scr_ert_mean + interview_age
##
## Parametric coefficients:
##                        Estimate Std. Error t value Pr(>|t|)
## (Intercept)          -0.633165   0.323079  -1.960   0.0502 .
## hormone_scr_ert_mean -0.001511   0.001287  -1.174   0.2406
## interview_age         0.006170   0.002767   2.230   0.0259 *
## ---
## Signif. codes:  0 '***' 0.001 '**' 0.01 '*' 0.05 '.' 0.1 ' ' 1
##
##
## R-sq.(adj) =  0.00188
## lmer.REML = 5547.5  Scale est. = 0.69017   n = 2060
```

```
##                           stdcoef      stdse
## X(Intercept)           0.00000000 0.00000000
## Xhormone_scr_ert_mean -0.02654532 0.02261336
## Xinterview_age         0.05031463 0.02256225
```

```
##
## Family: gaussian
## Link function: identity
##
## Formula:
## rt_diff_large_small_z ~ hormone_scr_ert_mean + interview_age
##
## Parametric coefficients:
##                         Estimate Std. Error t value Pr(>|t|)
## (Intercept)          -0.3696554  0.3262044  -1.133    0.257
## hormone_scr_ert_mean -0.0008419  0.0012986  -0.648    0.517
## interview_age         0.0034240  0.0027933   1.226    0.220
##
##
## R-sq.(adj) =  4.58e-06
## lmer.REML = 5587.6  Scale est. = 0.75055   n = 2060
```

```
##                           stdcoef      stdse
## X(Intercept)           0.00000000 0.00000000
## Xhormone_scr_ert_mean -0.01466609 0.02262339
## Xinterview_age         0.02768875 0.02258842
```

Male participants

```
##
## Family: gaussian
## Link function: identity
##
## Formula:
## rt_diff_large_neutral_z ~ hormone_scr_ert_mean + interview_age
##
## Parametric coefficients:
##                       Estimate Std. Error t value Pr(>|t|)
## (Intercept)           0.0837097  0.3030565   0.276    0.782
## hormone_scr_ert_mean -0.0006574  0.0012592  -0.522    0.602
## interview_age        -0.0006073  0.0025605  -0.237    0.813
##
##
## R-sq.(adj) =  -0.000719
## lmer.REML = 5514.9  Scale est. = 0.71939   n = 2133
```

```
##                           stdcoef      stdse
## X(Intercept)           0.00000000 0.00000000
## Xhormone_scr_ert_mean -0.01157785 0.02217728
## Xinterview_age        -0.00522356 0.02202364
```

```
##
## Family: gaussian
## Link function: identity
##
## Formula:
## rt_diff_large_small_z ~ hormone_scr_ert_mean + interview_age
##
## Parametric coefficients:
##                       Estimate Std. Error t value Pr(>|t|)
## (Intercept)           0.1149651  0.3045511   0.377    0.706
## hormone_scr_ert_mean -0.0013004  0.0012582  -1.034    0.301
## interview_age        -0.0004868  0.0025746  -0.189    0.850
##
##
## R-sq.(adj) =  -0.000388
## lmer.REML = 5554.6  Scale est. = 0.77615   n = 2133
```

```
##                            stdcoef      stdse
## X(Intercept)           0.000000000 0.00000000
## Xhormone_scr_ert_mean -0.022694149 0.02195786
## Xinterview_age        -0.004149015 0.02194322
```

2.20 Model: BIS-BAS-RR ~ Testosterone

Female participants

```
##
## Family: gaussian
```

```
## Link function: identity
##
## Formula:
## bisbas_ss_basm_rr_z ~ hormone_scr_ert_mean + interview_age
##
## Parametric coefficients:
##                       Estimate Std. Error t value Pr(>|t|)
## (Intercept)           0.346680   0.314572   1.102    0.271
## hormone_scr_ert_mean -0.001039   0.001247  -0.833    0.405
## interview_age        -0.002943   0.002683  -1.097    0.273
##
##
## R-sq.(adj) =  0.000485
## lmer.REML = 7026.6  Scale est. = 0.70697   n = 2502
```

```
##                           stdcoef      stdse
## X(Intercept)           0.00000000 0.00000000
## Xhormone_scr_ert_mean -0.01725639 0.02071182
## Xinterview_age        -0.02254169 0.02055015
```

Male participants

```
##
## Family: gaussian
## Link function: identity
##
## Formula:
## bisbas_ss_basm_rr_z ~ hormone_scr_ert_mean + interview_age
##
## Parametric coefficients:
##                       Estimate Std. Error t value Pr(>|t|)
## (Intercept)           0.198446   0.298864   0.664   0.5067
## hormone_scr_ert_mean  0.002152   0.001242   1.732   0.0834 .
## interview_age        -0.001610   0.002525  -0.638   0.5237
## ---
## Signif. codes:  0 '***' 0.001 '**' 0.01 '*' 0.05 '.' 0.1 ' ' 1
##
##
## R-sq.(adj) =  0.00103
## lmer.REML = 7464.6  Scale est. = 0.72311   n = 2697
```

```
##                           stdcoef      stdse
## X(Intercept)           0.00000000 0.00000000
## Xhormone_scr_ert_mean  0.03464661 0.02000401
## Xinterview_age        -0.01252359 0.01963926
```

3—Internalizing~Reward—

3.1 Model: CBCL internalizing factor ~ Nucleus Accumbens activity (anticipation stage)

Female participants

```
##
## Family: gaussian
## Link function: identity
##
## Formula:
## cbcl_scr_syn_internal_r ~ accumbens_rvsn_ant_z + interview_age
##
## Parametric coefficients:
##                      Estimate Std. Error t value Pr(>|t|)
## (Intercept)           6.03142    1.86213   3.239  0.00122 **
## accumbens_rvsn_ant_z -0.04281    0.16866  -0.254  0.79968
## interview_age        -0.00972    0.01557  -0.624  0.53257
## ---
## Signif. codes:  0 '***' 0.001 '**' 0.01 '*' 0.05 '.' 0.1 ' ' 1
##
##
## R-sq.(adj) =  -0.000632
## lmer.REML =  12626  Scale est. = 11.232    n = 2044
```

```
##                            stdcoef      stdse
## X(Intercept)           0.000000000 0.00000000
## Xaccumbens_rvsn_ant_z -0.005427053 0.02138326
## Xinterview_age        -0.013435302 0.02152431
```

Male participants

```
##
## Family: gaussian
## Link function: identity
##
## Formula:
## cbcl_scr_syn_internal_r ~ accumbens_rvsn_ant_z + interview_age
##
## Parametric coefficients:
##                      Estimate Std. Error t value Pr(>|t|)
## (Intercept)           3.25746    1.86102   1.750   0.0802 .
## accumbens_rvsn_ant_z -0.14197    0.15732  -0.902   0.3669
## interview_age         0.01252    0.01550   0.808   0.4195
## ---
## Signif. codes:  0 '***' 0.001 '**' 0.01 '*' 0.05 '.' 0.1 ' ' 1
##
##
## R-sq.(adj) =  -0.000586
## lmer.REML =  12672  Scale est. = 18.374    n = 2059
```

```
##                           stdcoef      stdse
## X(Intercept)           0.00000000 0.00000000
## Xaccumbens_rvsn_ant_z -0.01970131 0.02183160
## Xinterview_age         0.01786603 0.02212499
```

3.2 Model: CBCL internalizing factor ~ Caudate activity (anticipation stage)

Female participants

```
##
## Family: gaussian
## Link function: identity
##
## Formula:
## cbcl_scr_syn_internal_r ~ caudate_rvsn_ant_z + interview_age
##
## Parametric coefficients:
##                    Estimate Std. Error t value Pr(>|t|)
## (Intercept)         6.09957    1.86424   3.272  0.00109 **
## caudate_rvsn_ant_z -0.02686    0.12935  -0.208  0.83552
## interview_age      -0.01023    0.01559  -0.656  0.51174
## ---
## Signif. codes:  0 '***' 0.001 '**' 0.01 '*' 0.05 '.' 0.1 ' ' 1
##
##
## R-sq.(adj) =  -0.000647
## lmer.REML =  12629  Scale est. = 11.292    n = 2044
```

```
##                          stdcoef      stdse
## X(Intercept)         0.000000000 0.00000000
## Xcaudate_rvsn_ant_z -0.004436974 0.02136731
## Xinterview_age      -0.014126520 0.02152610
```

Male participants

```
##
## Family: gaussian
## Link function: identity
##
## Formula:
## cbcl_scr_syn_internal_r ~ caudate_rvsn_ant_z + interview_age
##
## Parametric coefficients:
##                    Estimate Std. Error t value Pr(>|t|)
## (Intercept)         3.49750    1.86404   1.876   0.0608 .
## caudate_rvsn_ant_z -0.10650    0.12325  -0.864   0.3876
## interview_age       0.01067    0.01552   0.687   0.4919
## ---
## Signif. codes:  0 '***' 0.001 '**' 0.01 '*' 0.05 '.' 0.1 ' ' 1
##
##
## R-sq.(adj) =  -0.000812
## lmer.REML =  12656  Scale est. = 18.614    n = 2056
```

```
##                          stdcoef      stdse
## X(Intercept)         0.00000000 0.00000000
## Xcaudate_rvsn_ant_z -0.01892928 0.02190619
## Xinterview_age       0.01521727 0.02213864
```

3.3 Model: CBCL internalizing factor ~ Putamen activity (anticipation stage)

Female participants

```
##
## Family: gaussian
## Link function: identity
##
## Formula:
## cbcl_scr_syn_internal_r ~ putamen_rvsn_ant_z + interview_age
##
## Parametric coefficients:
##                     Estimate Std. Error t value Pr(>|t|)
## (Intercept)         5.934388   1.857662   3.195  0.00142 **
## putamen_rvsn_ant_z -0.095198   0.132427  -0.719  0.47230
## interview_age      -0.008957   0.015537  -0.577  0.56434
## ---
## Signif. codes:  0 '***' 0.001 '**' 0.01 '*' 0.05 '.' 0.1 ' ' 1
##
##
## R-sq.(adj) =  -0.000598
## lmer.REML =  12595  Scale est. = 11.211    n = 2041
```

```
##                          stdcoef      stdse
## X(Intercept)         0.00000000 0.00000000
## Xputamen_rvsn_ant_z -0.01538262 0.02139824
## Xinterview_age      -0.01242203 0.02154710
```

Male participants

```
##
## Family: gaussian
## Link function: identity
##
## Formula:
## cbcl_scr_syn_internal_r ~ putamen_rvsn_ant_z + interview_age
##
## Parametric coefficients:
##                    Estimate Std. Error t value Pr(>|t|)
## (Intercept)         3.35048    1.85734   1.804   0.0714 .
## putamen_rvsn_ant_z -0.15563    0.12301  -1.265   0.2060
## interview_age       0.01181    0.01547   0.763   0.4453
## ---
## Signif. codes:  0 '***' 0.001 '**' 0.01 '*' 0.05 '.' 0.1 ' ' 1
##
##
## R-sq.(adj) =  -0.000471
## lmer.REML =  12647  Scale est. = 18.132    n = 2057
```

```
##                         stdcoef      stdse
## X(Intercept)         0.00000000 0.00000000
## Xputamen_rvsn_ant_z -0.02763637 0.02184387
## Xinterview_age       0.01688681 0.02212169
```

3.4 Model: CBCL internalizing factor ~ Accumbens activity (feedback stage)

Female participants

```
##
## Family: gaussian
## Link function: identity
##
## Formula:
## cbcl_scr_syn_internal_r ~ accumbens_posvsneg_feedback_z + interview_age
##
## Parametric coefficients:
##                                Estimate Std. Error t value Pr(>|t|)
## (Intercept)                    5.830691   1.856144   3.141  0.00171 **
## accumbens_posvsneg_feedback_z -0.050476   0.176282  -0.286  0.77465
## interview_age                 -0.008162   0.015527  -0.526  0.59916
## ---
## Signif. codes:  0 '***' 0.001 '**' 0.01 '*' 0.05 '.' 0.1 ' ' 1
##
##
## R-sq.(adj) =  -0.000578
## lmer.REML =  12646  Scale est. = 11.21     n = 2050
```

```
##                                    stdcoef      stdse
## X(Intercept)                    0.000000000 0.00000000
## Xaccumbens_posvsneg_feedback_z -0.006103001 0.02131392
## Xinterview_age                 -0.011313685 0.02152144
```

Male participants

```
##
## Family: gaussian
## Link function: identity
##
## Formula:
## cbcl_scr_syn_internal_r ~ accumbens_posvsneg_feedback_z + interview_age
##
## Parametric coefficients:
##                               Estimate Std. Error t value Pr(>|t|)
## (Intercept)                    3.20768    1.84860   1.735   0.0829 .
## accumbens_posvsneg_feedback_z  0.32112    0.16304   1.970   0.0490 *
## interview_age                  0.01259    0.01539   0.818   0.4137
## ---
## Signif. codes:  0 '***' 0.001 '**' 0.01 '*' 0.05 '.' 0.1 ' ' 1
##
##
## R-sq.(adj) =  0.000399
## lmer.REML =  12603  Scale est. = 18.899    n = 2054
```

```
##                                    stdcoef      stdse
## X(Intercept)                    0.00000000 0.00000000
## Xaccumbens_posvsneg_feedback_z 0.04319714 0.02193190
## Xinterview_age                  0.01808956 0.02212641
```

3.5 Model: CBCL internalizing factor ~ Caudate activity (feedback stage)

Female participants

```
##
## Family: gaussian
## Link function: identity
##
## Formula:
## cbcl_scr_syn_internal_r ~ caudate_posvsneg_feedback_z + interview_age
##
## Parametric coefficients:
##                             Estimate Std. Error t value Pr(>|t|)
## (Intercept)                  6.08504    1.86838   3.257  0.00115 **
## caudate_posvsneg_feedback_z -0.18760    0.13267  -1.414  0.15750
## interview_age               -0.01029    0.01562  -0.659  0.51010
## ---
## Signif. codes:  0 '***' 0.001 '**' 0.01 '*' 0.05 '.' 0.1 ' ' 1
##
##
## R-sq.(adj) =  0.000878
## lmer.REML =  12610  Scale est. = 11.326    n = 2042
```

```
##                                   stdcoef      stdse
## X(Intercept)                   0.00000000 0.00000000
## Xcaudate_posvsneg_feedback_z -0.02984372 0.02110522
## Xinterview_age               -0.01421366 0.02157502
```

Male participants

```
##
## Family: gaussian
## Link function: identity
##
## Formula:
## cbcl_scr_syn_internal_r ~ caudate_posvsneg_feedback_z + interview_age
##
## Parametric coefficients:
##                             Estimate Std. Error t value Pr(>|t|)
## (Intercept)                 3.781203   1.865165   2.027   0.0428 *
## caudate_posvsneg_feedback_z 0.141738   0.131057   1.082   0.2796
## interview_age               0.008144   0.015537   0.524   0.6002
## ---
## Signif. codes:  0 '***' 0.001 '**' 0.01 '*' 0.05 '.' 0.1 ' ' 1
##
##
## R-sq.(adj) =  -0.000857
## lmer.REML =  12670  Scale est. = 18.536    n = 2058
```

```
##                                  stdcoef      stdse
## X(Intercept)                  0.00000000 0.00000000
## Xcaudate_posvsneg_feedback_z 0.02360106 0.02182246
## Xinterview_age                0.01159805 0.02212483
```

3.6 Model: CBCL internalizing factor ~ Putamen activity (feedback stage)

Female participants

```
##
## Family: gaussian
## Link function: identity
##
## Formula:
## cbcl_scr_syn_internal_r ~ putamen_posvsneg_feedback_z + interview_age
##
## Parametric coefficients:
##                              Estimate Std. Error t value Pr(>|t|)
## (Intercept)                  5.870302   1.864372   3.149  0.00166 **
## putamen_posvsneg_feedback_z -0.099433   0.139287  -0.714  0.47539
## interview_age               -0.008449   0.015591  -0.542  0.58795
## ---
## Signif. codes:  0 '***' 0.001 '**' 0.01 '*' 0.05 '.' 0.1 ' ' 1
##
##
## R-sq.(adj) =  -0.000157
## lmer.REML =  12610  Scale est. = 11.281    n = 2042
```

```
##                                   stdcoef      stdse
## X(Intercept)                   0.00000000 0.00000000
## Xputamen_posvsneg_feedback_z -0.01507505 0.02111722
## Xinterview_age               -0.01167656 0.02154777
```

Male participants

```
##
## Family: gaussian
## Link function: identity
##
## Formula:
## cbcl_scr_syn_internal_r ~ putamen_posvsneg_feedback_z + interview_age
##
## Parametric coefficients:
##                             Estimate Std. Error t value Pr(>|t|)
## (Intercept)                  3.54548    1.86842   1.898   0.0579 .
## putamen_posvsneg_feedback_z  0.13679    0.13284   1.030   0.3032
## interview_age                0.01013    0.01556   0.651   0.5149
## ---
## Signif. codes:  0 '***' 0.001 '**' 0.01 '*' 0.05 '.' 0.1 ' ' 1
##
##
## R-sq.(adj) =  -0.00108
## lmer.REML =  12701  Scale est. = 18.877    n = 2061
```

```
##                                 stdcoef      stdse
## X(Intercept)                 0.00000000 0.00000000
## Xputamen_posvsneg_feedback_z 0.02257279 0.02192055
## Xinterview_age               0.01439570 0.02210408
```

3.7 Model: CBCL internalizing factor ~ OFC activity (anticipation stage)

Female participants

```
##
## Family: gaussian
## Link function: identity
##
## Formula:
## cbcl_scr_syn_internal_r ~ lOFC_rvsn_ant_z + interview_age
##
## Parametric coefficients:
##                  Estimate Std. Error t value Pr(>|t|)
## (Intercept)      5.886252   1.871858   3.145  0.00169 **
## lOFC_rvsn_ant_z  0.028463   0.202850   0.140  0.88843
## interview_age   -0.008492   0.015651  -0.543  0.58746
## ---
## Signif. codes:  0 '***' 0.001 '**' 0.01 '*' 0.05 '.' 0.1 ' ' 1
##
##
## R-sq.(adj) =  -0.000729
## lmer.REML =  12589  Scale est. = 11.525    n = 2038
```

```
##                       stdcoef      stdse
## X(Intercept)      0.000000000 0.00000000
## XlOFC_rvsn_ant_z  0.003020377 0.02152582
## Xinterview_age   -0.011738081 0.02163280
```

```
##
## Family: gaussian
## Link function: identity
##
## Formula:
## cbcl_scr_syn_internal_r ~ mOFC_rvsn_ant_z + interview_age
##
## Parametric coefficients:
##                  Estimate Std. Error t value Pr(>|t|)
## (Intercept)      5.859094   1.868796   3.135  0.00174 **
## mOFC_rvsn_ant_z  0.158153   0.173166   0.913  0.36119
## interview_age   -0.008187   0.015629  -0.524  0.60045
## ---
## Signif. codes:  0 '***' 0.001 '**' 0.01 '*' 0.05 '.' 0.1 ' ' 1
##
##
## R-sq.(adj) =  -0.000132
## lmer.REML =  12597  Scale est. = 11.352    n = 2039
```

```
##                      stdcoef      stdse
```

```
## X(Intercept)      0.00000000 0.00000000
## XmOFC_rvsn_ant_z  0.01926434 0.02109318
## Xinterview_age   -0.01129104 0.02155432
```

Male participants

```
##
## Family: gaussian
## Link function: identity
##
## Formula:
## cbcl_scr_syn_internal_r ~ lOFC_rvsn_ant_z + interview_age
##
## Parametric coefficients:
##                 Estimate Std. Error t value Pr(>|t|)
## (Intercept)      2.59775    1.84642   1.407    0.160
## lOFC_rvsn_ant_z  0.01023    0.18646   0.055    0.956
## interview_age    0.01775    0.01538   1.155    0.248
##
##
## R-sq.(adj) =  -0.000919
## lmer.REML =  12592  Scale est. = 18.062    n = 2053
```

```
##                      stdcoef      stdse
## X(Intercept)     0.000000000 0.00000000
## XlOFC_rvsn_ant_z 0.001201186 0.02190392
## Xinterview_age   0.025574945 0.02215096
```

```
##
## Family: gaussian
## Link function: identity
##
## Formula:
## cbcl_scr_syn_internal_r ~ mOFC_rvsn_ant_z + interview_age
##
## Parametric coefficients:
##                 Estimate Std. Error t value Pr(>|t|)
## (Intercept)      2.66849    1.85435   1.439    0.150
## mOFC_rvsn_ant_z  0.25414    0.17105   1.486    0.137
## interview_age    0.01724    0.01544   1.116    0.264
##
##
## R-sq.(adj) =  0.000328
## lmer.REML =  12576  Scale est. = 18.194    n = 2048
```

```
##                     stdcoef      stdse
## X(Intercept)     0.00000000 0.00000000
## XmOFC_rvsn_ant_z 0.03252081 0.02188820
## Xinterview_age   0.02474006 0.02216395
```

3.8 Model: CBCL internalizing factor ~ OFC activity (feedback stage)

Female participants

```
##
## Family: gaussian
## Link function: identity
##
## Formula:
## cbcl_scr_syn_internal_r ~ lOFC_posvsneg_feedback_z + interview_age
##
## Parametric coefficients:
##                           Estimate Std. Error t value Pr(>|t|)
## (Intercept)               5.888613   1.861224   3.164  0.00158 **
## lOFC_posvsneg_feedback_z -0.237566   0.228693  -1.039  0.29902
## interview_age            -0.008663   0.015572  -0.556  0.57803
## ---
## Signif. codes:  0 '***' 0.001 '**' 0.01 '*' 0.05 '.' 0.1 ' ' 1
##
##
## R-sq.(adj) =  4.13e-05
## lmer.REML =  12579  Scale est. = 11.213    n = 2039
```

```
##                               stdcoef      stdse
## X(Intercept)                0.00000000 0.00000000
## XlOFC_posvsneg_feedback_z -0.02224531 0.02141444
## Xinterview_age            -0.01200652 0.02158089
```

```
##
## Family: gaussian
## Link function: identity
##
## Formula:
## cbcl_scr_syn_internal_r ~ mOFC_posvsneg_feedback_z + interview_age
##
## Parametric coefficients:
##                           Estimate Std. Error t value Pr(>|t|)
## (Intercept)               5.973940   1.863262   3.206  0.00137 **
## mOFC_posvsneg_feedback_z -0.159334   0.188165  -0.847  0.39722
## interview_age            -0.009333   0.015591  -0.599  0.54951
## ---
## Signif. codes:  0 '***' 0.001 '**' 0.01 '*' 0.05 '.' 0.1 ' ' 1
##
##
## R-sq.(adj) =  0.000255
## lmer.REML =  12595  Scale est. = 11.397    n = 2040
```

```
##                               stdcoef      stdse
## X(Intercept)                0.00000000 0.00000000
## XmOFC_posvsneg_feedback_z -0.01821223 0.02150767
## Xinterview_age            -0.01289717 0.02154552
```

Male participants

```
##
## Family: gaussian
## Link function: identity
##
## Formula:
## cbcl_scr_syn_internal_r ~ lOFC_posvsneg_feedback_z + interview_age
##
## Parametric coefficients:
##                          Estimate Std. Error t value Pr(>|t|)
## (Intercept)               3.13068    1.84350   1.698   0.0896 .
## lOFC_posvsneg_feedback_z  0.06848    0.20435   0.335   0.7376
## interview_age             0.01345    0.01535   0.876   0.3810
## ---
## Signif. codes:  0 '***' 0.001 '**' 0.01 '*' 0.05 '.' 0.1 ' ' 1
##
##
## R-sq.(adj) =  -0.00105
## lmer.REML =  12663  Scale est. = 18.043    n = 2063
```

```
##                                stdcoef      stdse
## X(Intercept)              0.000000000 0.00000000
## XlOFC_posvsneg_feedback_z 0.007299579 0.02178333
## Xinterview_age            0.019366812 0.02210083
```

```
##
## Family: gaussian
## Link function: identity
##
## Formula:
## cbcl_scr_syn_internal_r ~ mOFC_posvsneg_feedback_z + interview_age
##
## Parametric coefficients:
##                          Estimate Std. Error t value Pr(>|t|)
## (Intercept)               3.17570    1.84275   1.723    0.085 .
## mOFC_posvsneg_feedback_z  0.26532    0.17873   1.484    0.138
## interview_age             0.01304    0.01535   0.850    0.396
## ---
## Signif. codes:  0 '***' 0.001 '**' 0.01 '*' 0.05 '.' 0.1 ' ' 1
##
##
## R-sq.(adj) =  -0.000177
## lmer.REML =  12652  Scale est. = 18.142    n = 2061
```

```
##                               stdcoef      stdse
## X(Intercept)              0.00000000 0.00000000
## XmOFC_posvsneg_feedback_z 0.03243102 0.02184718
## Xinterview_age            0.01875492 0.02207703
```

3.9 Model: CBCL internalizing factor ~ BIS-BAS-RR

Female participants

```
##
## Family: gaussian
## Link function: identity
##
## Formula:
## cbcl_scr_syn_internal_r ~ bisbas_ss_basm_rr + interview_age
##
## Parametric coefficients:
##                    Estimate Std. Error t value Pr(>|t|)
## (Intercept)        4.576754   1.717826   2.664  0.00776 **
## bisbas_ss_basm_rr -0.070300   0.044419  -1.583  0.11362
## interview_age      0.008269   0.013933   0.593  0.55293
## ---
## Signif. codes:  0 '***' 0.001 '**' 0.01 '*' 0.05 '.' 0.1 ' ' 1
##
##
## R-sq.(adj) =  -0.000292
## lmer.REML =  16721  Scale est. = 12.884    n = 2690
```

```
##                        stdcoef      stdse
## X(Intercept)        0.00000000 0.00000000
## Xbisbas_ss_basm_rr -0.02987863 0.01887900
## Xinterview_age      0.01127390 0.01899736
```

Male participants

```
##
## Family: gaussian
## Link function: identity
##
## Formula:
## cbcl_scr_syn_internal_r ~ bisbas_ss_basm_rr + interview_age
##
## Parametric coefficients:
##                   Estimate Std. Error t value Pr(>|t|)
## (Intercept)        3.60221    1.69192   2.129   0.0333 *
## bisbas_ss_basm_rr  0.01009    0.04429   0.228   0.8198
## interview_age      0.01112    0.01371   0.811   0.4176
## ---
## Signif. codes:  0 '***' 0.001 '**' 0.01 '*' 0.05 '.' 0.1 ' ' 1
##
##
## R-sq.(adj) =  -0.000755
## lmer.REML =  18144  Scale est. = 16.373    n = 2907
```

```
##                        stdcoef      stdse
## X(Intercept)       0.000000000 0.00000000
## Xbisbas_ss_basm_rr 0.004192092 0.01840355
## Xinterview_age     0.015062217 0.01857752
```

3.10 Model: CBCL internalizing factor ~ MID Reaction Time

Female participants

```
##
## Family: gaussian
## Link function: identity
##
## Formula:
## cbcl_scr_syn_internal_r ~ rt_diff_large_neutral_z + interview_age
##
## Parametric coefficients:
##                          Estimate Std. Error t value Pr(>|t|)
## (Intercept)               6.34845    1.78964   3.547 0.000397 ***
## rt_diff_large_neutral_z  0.13672    0.12031   1.136 0.255921
## interview_age           -0.01246    0.01495  -0.833 0.404751
## ---
## Signif. codes:  0 '***' 0.001 '**' 0.01 '*' 0.05 '.' 0.1 ' ' 1
##
##
## R-sq.(adj) =  -5.14e-06
## lmer.REML =  13581  Scale est. = 11.707    n = 2201
```

```
##                              stdcoef      stdse
## X(Intercept)              0.00000000 0.00000000
## Xrt_diff_large_neutral_z  0.02353331 0.02070898
## Xinterview_age           -0.01731784 0.02078157
```

```
##
## Family: gaussian
## Link function: identity
##
## Formula:
## cbcl_scr_syn_internal_r ~ rt_diff_large_small_z + interview_age
##
## Parametric coefficients:
##                        Estimate Std. Error t value Pr(>|t|)
## (Intercept)             6.25233    1.78792   3.497  0.00048 ***
## rt_diff_large_small_z -0.15739    0.11917  -1.321  0.18675
## interview_age         -0.01158    0.01493  -0.775  0.43813
## ---
## Signif. codes:  0 '***' 0.001 '**' 0.01 '*' 0.05 '.' 0.1 ' ' 1
##
##
## R-sq.(adj) =  -0.000251
## lmer.REML =  13580  Scale est. = 11.639    n = 2201
```

```
##                            stdcoef      stdse
## X(Intercept)            0.00000000 0.00000000
## Xrt_diff_large_small_z -0.02721487 0.02060706
## Xinterview_age         -0.01609858 0.02075907
```

Male participants

```
##
## Family: gaussian
## Link function: identity
##
## Formula:
## cbcl_scr_syn_internal_r ~ rt_diff_large_neutral_z + interview_age
##
## Parametric coefficients:
##                         Estimate Std. Error t value Pr(>|t|)
## (Intercept)              2.95337    1.77854   1.661   0.0969 .
## rt_diff_large_neutral_z  0.04500    0.12536   0.359   0.7197
## interview_age            0.01547    0.01482   1.044   0.2965
## ---
## Signif. codes:  0 '***' 0.001 '**' 0.01 '*' 0.05 '.' 0.1 ' ' 1
##
##
## R-sq.(adj) =  -0.000758
## lmer.REML =  14182  Scale est. = 17.631    n = 2297
```

```
##                              stdcoef      stdse
## X(Intercept)              0.00000000 0.00000000
## Xrt_diff_large_neutral_z 0.00742118 0.02067627
## Xinterview_age            0.02185601 0.02093084
```

```
##
## Family: gaussian
## Link function: identity
##
## Formula:
## cbcl_scr_syn_internal_r ~ rt_diff_large_small_z + interview_age
##
## Parametric coefficients:
##                       Estimate Std. Error t value Pr(>|t|)
## (Intercept)            2.96179    1.77843   1.665    0.096 .
## rt_diff_large_small_z -0.07562    0.12322  -0.614    0.539
## interview_age          0.01540    0.01481   1.040    0.299
## ---
## Signif. codes:  0 '***' 0.001 '**' 0.01 '*' 0.05 '.' 0.1 ' ' 1
##
##
## R-sq.(adj) =  -0.000672
## lmer.REML =  14181  Scale est. = 17.688    n = 2297
```

```
##                            stdcoef      stdse
## X(Intercept)            0.00000000 0.00000000
## Xrt_diff_large_small_z -0.01266907 0.02064481
## Xinterview_age          0.02175852 0.02092903
```

4—Internalizing~Puberty x Reward—

4.1 Model: CBCL internalizing factor ~ PDS x Accumbens activity (anticipation stage)

Female participants

```
##
## Family: gaussian
## Link function: identity
##
## Formula:
## cbcl_scr_syn_internal_r ~ PDS_score * accumbens_rvsn_ant_z +
##     race.ethnicity.5level + demo_race_hispanic + interview_age
##
## Parametric coefficients:
##                              Estimate Std. Error t value Pr(>|t|)
## (Intercept)                   4.93246    2.07818   2.373 0.017717 *
## PDS_score                     0.66789    0.17883   3.735 0.000193 ***
## accumbens_rvsn_ant_z         -0.74666    0.42905  -1.740 0.081967 .
## race.ethnicity.5levelBlack    0.54545    0.89101   0.612 0.540495
## race.ethnicity.5levelMixed    2.34273    0.87490   2.678 0.007473 **
## race.ethnicity.5levelOther    2.34680    0.99199   2.366 0.018089 *
## race.ethnicity.5levelWhite    1.35252    0.82265   1.644 0.100314
## demo_race_hispanic1           0.49442    0.34785   1.421 0.155373
## interview_age                -0.02243    0.01629  -1.377 0.168697
## PDS_score:accumbens_rvsn_ant_z  0.42529    0.23874   1.781 0.074993 .
## ---
## Signif. codes:  0 '***' 0.001 '**' 0.01 '*' 0.05 '.' 0.1 ' ' 1
##
##
## R-sq.(adj) =  0.0145
## lmer.REML =  12315  Scale est. = 11.173    n = 1999
```

```
##                                   stdcoef      stdse
## X(Intercept)                    0.00000000 0.00000000
## XPDS_score                      0.08899043 0.02382796
## Xaccumbens_rvsn_ant_z          -0.09396490 0.05399451
## Xrace.ethnicity.5levelBlack     0.03339004 0.05454371
## Xrace.ethnicity.5levelMixed     0.14413866 0.05382876
## Xrace.ethnicity.5levelOther     0.09479789 0.04007102
## Xrace.ethnicity.5levelWhite     0.11707848 0.07121143
## Xdemo_race_hispanic1            0.03675147 0.02585679
## Xinterview_age                 -0.03088277 0.02242913
## XPDS_score:accumbens_rvsn_ant_z  0.09588237 0.05382300
```

Male participants

```
##
## Family: gaussian
## Link function: identity
##
```

```
## Formula:
## cbcl_scr_syn_internal_r ~ PDS_score * accumbens_rvsn_ant_z +
##     race.ethnicity.5level + demo_race_hispanic + interview_age
##
## Parametric coefficients:
##                                Estimate Std. Error t value Pr(>|t|)
## (Intercept)                    1.105143   2.013195   0.549  0.58310
## PDS_score                      0.744167   0.231992   3.208  0.00136 **
## accumbens_rvsn_ant_z          -0.182527   0.432717  -0.422  0.67321
## race.ethnicity.5levelBlack     1.141776   0.870109   1.312  0.18960
## race.ethnicity.5levelMixed     2.816734   0.860474   3.273  0.00108 **
## race.ethnicity.5levelOther     2.802547   0.990415   2.830  0.00471 **
## race.ethnicity.5levelWhite     2.105762   0.809086   2.603  0.00932 **
## demo_race_hispanic1            0.032827   0.334966   0.098  0.92194
## interview_age                  0.004667   0.015736   0.297  0.76681
## PDS_score:accumbens_rvsn_ant_z 0.032985   0.298462   0.111  0.91201
## ---
## Signif. codes:  0 '***' 0.001 '**' 0.01 '*' 0.05 '.' 0.1 ' ' 1
##
##
## R-sq.(adj) =   0.01
## lmer.REML =  12349  Scale est. = 18.343    n = 2017
```

```
##                                     stdcoef      stdse
## X(Intercept)                    0.000000000 0.00000000
## XPDS_score                      0.075294613 0.02347290
## Xaccumbens_rvsn_ant_z          -0.025438342 0.06030683
## Xrace.ethnicity.5levelBlack     0.071353141 0.05437581
## Xrace.ethnicity.5levelMixed     0.178180769 0.05443179
## Xrace.ethnicity.5levelOther     0.110302206 0.03898061
## Xrace.ethnicity.5levelWhite     0.186781241 0.07176600
## Xdemo_race_hispanic1            0.002517770 0.02569164
## Xinterview_age                  0.006725127 0.02267481
## XPDS_score:accumbens_rvsn_ant_z 0.006671157 0.06036306
```

4.2 Model: CBCL internalizing factor ~ PDS x Caudate activity (anticipation stage)

Female participants

```
##
## Family: gaussian
## Link function: identity
##
## Formula:
## cbcl_scr_syn_internal_r ~ PDS_score * caudate_rvsn_ant_z + race.ethnicity.5level +
##     demo_race_hispanic + interview_age
##
## Parametric coefficients:
##                             Estimate Std. Error t value Pr(>|t|)
## (Intercept)                  5.16482    2.08805   2.474 0.013462 *
## PDS_score                    0.69019    0.17965   3.842 0.000126 ***
## caudate_rvsn_ant_z          -0.11993    0.32559  -0.368 0.712651
```

```
## race.ethnicity.5levelBlack    0.54705    0.89316   0.612 0.540289
## race.ethnicity.5levelMixed    2.29666    0.87576   2.622 0.008796 **
## race.ethnicity.5levelOther    2.30580    0.99182   2.325 0.020181 *
## race.ethnicity.5levelWhite    1.32860    0.82400   1.612 0.107037
## demo_race_hispanic1           0.49035    0.34786   1.410 0.158803
## interview_age                -0.02440    0.01638  -1.490 0.136466
## PDS_score:caudate_rvsn_ant_z  0.05174    0.18124   0.285 0.775296
## ---
## Signif. codes:  0 '***' 0.001 '**' 0.01 '*' 0.05 '.' 0.1 ' ' 1
##
##
## R-sq.(adj) =  0.0131
## lmer.REML =  12316  Scale est. = 11.35     n = 1998
```

```
##                                   stdcoef      stdse
## X(Intercept)                   0.00000000 0.00000000
## XPDS_score                     0.09168964 0.02386600
## Xcaudate_rvsn_ant_z           -0.01968152 0.05343153
## Xrace.ethnicity.5levelBlack    0.03330435 0.05437603
## Xrace.ethnicity.5levelMixed    0.14172435 0.05404239
## Xrace.ethnicity.5levelOther    0.09311051 0.04005049
## Xrace.ethnicity.5levelWhite    0.11491193 0.07126832
## Xdemo_race_hispanic1           0.03640186 0.02582355
## Xinterview_age                -0.03354718 0.02251966
## XPDS_score:caudate_rvsn_ant_z  0.01524638 0.05340356
```

Male participants

```
##
## Family: gaussian
## Link function: identity
##
## Formula:
## cbcl_scr_syn_internal_r ~ PDS_score * caudate_rvsn_ant_z + race.ethnicity.5level +
##     demo_race_hispanic + interview_age
##
## Parametric coefficients:
##                              Estimate Std. Error t value Pr(>|t|)
## (Intercept)                  1.335424   2.020919   0.661 0.508816
## PDS_score                    0.766072   0.231952   3.303 0.000974 ***
## caudate_rvsn_ant_z           0.282502   0.351648   0.803 0.421859
## race.ethnicity.5levelBlack   1.034625   0.889356   1.163 0.244829
## race.ethnicity.5levelMixed   2.698696   0.880718   3.064 0.002212 **
## race.ethnicity.5levelOther   2.779288   1.004888   2.766 0.005731 **
## race.ethnicity.5levelWhite   2.023427   0.830117   2.438 0.014875 *
## demo_race_hispanic1          0.055234   0.336414   0.164 0.869603
## interview_age                0.003298   0.015749   0.209 0.834160
## PDS_score:caudate_rvsn_ant_z -0.297277   0.244097  -1.218 0.223418
## ---
## Signif. codes:  0 '***' 0.001 '**' 0.01 '*' 0.05 '.' 0.1 ' ' 1
##
##
## R-sq.(adj) =  0.0104
```

```
## lmer.REML =  12338  Scale est. = 18.37     n = 2015
```

```
##                                  stdcoef      stdse
## X(Intercept)                  0.000000000 0.00000000
## XPDS_score                    0.077470881 0.02345668
## Xcaudate_rvsn_ant_z           0.050628423 0.06302045
## Xrace.ethnicity.5levelBlack   0.064762432 0.05566931
## Xrace.ethnicity.5levelMixed   0.170105930 0.05551395
## Xrace.ethnicity.5levelOther   0.110541978 0.03996791
## Xrace.ethnicity.5levelWhite   0.179347856 0.07357803
## Xdemo_race_hispanic1          0.004223753 0.02572579
## Xinterview_age                0.004747977 0.02267455
## XPDS_score:caudate_rvsn_ant_z -0.076813601 0.06307229
```

4.3 Model: CBCL internalizing factor ~ PDS x Putamen activity (anticipation stage)

Female participants

```
##
## Family: gaussian
## Link function: identity
##
## Formula:
## cbcl_scr_syn_internal_r ~ PDS_score * putamen_rvsn_ant_z + race.ethnicity.5level +
##     demo_race_hispanic + interview_age
##
## Parametric coefficients:
##                            Estimate Std. Error t value Pr(>|t|)
## (Intercept)                 4.97477    2.07632   2.396 0.016669 *
## PDS_score                   0.69413    0.17949   3.867 0.000114 ***
## putamen_rvsn_ant_z         -0.37583    0.32746  -1.148 0.251214
## race.ethnicity.5levelBlack  0.59070    0.88838   0.665 0.506182
## race.ethnicity.5levelMixed  2.32554    0.87167   2.668 0.007695 **
## race.ethnicity.5levelOther  2.29813    0.98931   2.323 0.020281 *
## race.ethnicity.5levelWhite  1.29941    0.81989   1.585 0.113158
## demo_race_hispanic1         0.50620    0.34697   1.459 0.144749
## interview_age              -0.02287    0.01628  -1.404 0.160369
## PDS_score:putamen_rvsn_ant_z  0.18597    0.18035   1.031 0.302586
## ---
## Signif. codes:  0 '***' 0.001 '**' 0.01 '*' 0.05 '.' 0.1 ' ' 1
##
##
## R-sq.(adj) =  0.014
## lmer.REML =  12281  Scale est. = 11.319    n = 1995
```

```
##                                 stdcoef      stdse
## X(Intercept)                  0.00000000 0.00000000
## XPDS_score                    0.09255025 0.02393170
## Xputamen_rvsn_ant_z          -0.06021333 0.05246248
## Xrace.ethnicity.5levelBlack   0.03618439 0.05441955
## Xrace.ethnicity.5levelMixed   0.14390731 0.05394014
## Xrace.ethnicity.5levelOther   0.09322116 0.04013015
```

```
## Xrace.ethnicity.5levelWhite    0.11286005 0.07121149
## Xdemo_race_hispanic1           0.03777751 0.02589438
## Xinterview_age                -0.03158244 0.02248890
## XPDS_score:putamen_rvsn_ant_z  0.05394806 0.05231720
```

Male participants

```
##
## Family: gaussian
## Link function: identity
##
## Formula:
## cbcl_scr_syn_internal_r ~ PDS_score * putamen_rvsn_ant_z + race.ethnicity.5level +
##     demo_race_hispanic + interview_age
##
## Parametric coefficients:
##                               Estimate Std. Error t value Pr(>|t|)
## (Intercept)                   1.097535   2.015001   0.545 0.586033
## PDS_score                     0.793779   0.232575   3.413 0.000655 ***
## putamen_rvsn_ant_z            0.681711   0.349735   1.949 0.051408 .
## race.ethnicity.5levelBlack    1.111177   0.886597   1.253 0.210240
## race.ethnicity.5levelMixed    2.732572   0.875784   3.120 0.001833 **
## race.ethnicity.5levelOther    2.662829   1.004894   2.650 0.008116 **
## race.ethnicity.5levelWhite    2.059703   0.827160   2.490 0.012851 *
## demo_race_hispanic1          -0.001105   0.335942  -0.003 0.997377
## interview_age                 0.004782   0.015710   0.304 0.760864
## PDS_score:putamen_rvsn_ant_z -0.637708   0.246660  -2.585 0.009797 **
## ---
## Signif. codes:  0 '***' 0.001 '**' 0.01 '*' 0.05 '.' 0.1 ' ' 1
##
##
## R-sq.(adj) =  0.0125
## lmer.REML =  12328  Scale est. = 17.583    n = 2016
```

```
##                                     stdcoef      stdse
## X(Intercept)                   0.0000000000 0.00000000
## XPDS_score                     0.0799458470 0.02342394
## Xputamen_rvsn_ant_z            0.1218344044 0.06250409
## Xrace.ethnicity.5levelBlack    0.0694445332 0.05540909
## Xrace.ethnicity.5levelMixed    0.1740559719 0.05578460
## Xrace.ethnicity.5levelOther    0.1049969322 0.03962356
## Xrace.ethnicity.5levelWhite    0.1830151498 0.07349741
## Xdemo_race_hispanic1          -0.0000845667 0.02571651
## Xinterview_age                 0.0068968450 0.02265804
## XPDS_score:putamen_rvsn_ant_z -0.1616275006 0.06251620
```

4.4 Model: CBCL internalizing factor ~ PDS x Lateral OFC activity (anticipation stage)

Female participants

```
##
```

```
## Family: gaussian
## Link function: identity
##
## Formula:
## cbcl_scr_syn_internal_r ~ PDS_score * lOFC_rvsn_ant_z + race.ethnicity.5level +
##     demo_race_hispanic + interview_age
##
## Parametric coefficients:
##                            Estimate Std. Error t value Pr(>|t|)
## (Intercept)                 4.90115    2.09807   2.336 0.019589 *
## PDS_score                   0.66515    0.17977   3.700 0.000222 ***
## lOFC_rvsn_ant_z             0.01527    0.52247   0.029 0.976689
## race.ethnicity.5levelBlack  0.52114    0.89989   0.579 0.562581
## race.ethnicity.5levelMixed  2.24412    0.88436   2.538 0.011239 *
## race.ethnicity.5levelOther  2.23647    1.00065   2.235 0.025527 *
## race.ethnicity.5levelWhite  1.24974    0.83231   1.502 0.133380
## demo_race_hispanic1         0.51354    0.34902   1.471 0.141342
## interview_age              -0.02142    0.01641  -1.305 0.192031
## PDS_score:lOFC_rvsn_ant_z   0.03931    0.28333   0.139 0.889677
## ---
## Signif. codes:  0 '***' 0.001 '**' 0.01 '*' 0.05 '.' 0.1 ' ' 1
##
##
## R-sq.(adj) =  0.0124
## lmer.REML =  12288  Scale est. = 11.562    n = 1994
```

```
##                                  stdcoef      stdse
## X(Intercept)                 0.000000000 0.00000000
## XPDS_score                   0.088727047 0.02398104
## XlOFC_rvsn_ant_z             0.001601394 0.05479781
## Xrace.ethnicity.5levelBlack  0.031889966 0.05506718
## Xrace.ethnicity.5levelMixed  0.138259645 0.05448509
## Xrace.ethnicity.5levelOther  0.091050210 0.04073797
## Xrace.ethnicity.5levelWhite  0.108336858 0.07215133
## Xdemo_race_hispanic1         0.038283997 0.02601878
## Xinterview_age              -0.029496615 0.02260208
## XPDS_score:lOFC_rvsn_ant_z   0.007601278 0.05479157
```

Male participants

```
##
## Family: gaussian
## Link function: identity
##
## Formula:
## cbcl_scr_syn_internal_r ~ PDS_score * lOFC_rvsn_ant_z + race.ethnicity.5level +
##     demo_race_hispanic + interview_age
##
## Parametric coefficients:
##                            Estimate Std. Error t value Pr(>|t|)
## (Intercept)                 0.43320    1.99732   0.217  0.82831
## PDS_score                   0.65010    0.23340   2.785  0.00540 **
## lOFC_rvsn_ant_z            -0.47040    0.51114  -0.920  0.35753
```

```
## race.ethnicity.5levelBlack  1.16389    0.86244   1.350  0.17732
## race.ethnicity.5levelMixed  2.77910    0.85230   3.261  0.00113 **
## race.ethnicity.5levelOther  2.81399    0.97983   2.872  0.00412 **
## race.ethnicity.5levelWhite  2.03614    0.80073   2.543  0.01107 *
## demo_race_hispanic1        -0.05505    0.33262  -0.166  0.86856
## interview_age               0.01162    0.01563   0.743  0.45750
## PDS_score:lOFC_rvsn_ant_z   0.33920    0.34826   0.974  0.33017
## ---
## Signif. codes:  0 '***' 0.001 '**' 0.01 '*' 0.05 '.' 0.1 ' ' 1
##
##
## R-sq.(adj) =  0.00873
## lmer.REML =  12290  Scale est. = 17.836    n = 2014
```

```
##                                  stdcoef      stdse
## X(Intercept)                 0.000000000 0.00000000
## XPDS_score                   0.065971761 0.02368487
## XlOFC_rvsn_ant_z            -0.055571732 0.06038459
## Xrace.ethnicity.5levelBlack  0.072989147 0.05408490
## Xrace.ethnicity.5levelMixed  0.177068361 0.05430378
## Xrace.ethnicity.5levelOther  0.112541004 0.03918660
## Xrace.ethnicity.5levelWhite  0.182090163 0.07160877
## Xdemo_race_hispanic1        -0.004259226 0.02573431
## Xinterview_age               0.016891116 0.02273038
## XPDS_score:lOFC_rvsn_ant_z   0.058918560 0.06049135
```

4.5 Model: CBCL internalizing factor ~ PDS x Medial OFC activity (anticipation stage)

Female participants

```
##
## Family: gaussian
## Link function: identity
##
## Formula:
## cbcl_scr_syn_internal_r ~ PDS_score * mOFC_rvsn_ant_z + race.ethnicity.5level +
##     demo_race_hispanic + interview_age
##
## Parametric coefficients:
##                             Estimate Std. Error t value Pr(>|t|)
## (Intercept)                  4.84149    2.09453   2.311 0.020908 *
## PDS_score                    0.66802    0.17956   3.720 0.000204 ***
## mOFC_rvsn_ant_z             -0.03687    0.43630  -0.085 0.932667
## race.ethnicity.5levelBlack   0.51760    0.90004   0.575 0.565296
## race.ethnicity.5levelMixed   2.25855    0.88469   2.553 0.010757 *
## race.ethnicity.5levelOther   2.31830    1.00294   2.312 0.020907 *
## race.ethnicity.5levelWhite   1.29551    0.83274   1.556 0.119937
## demo_race_hispanic1          0.49881    0.34895   1.429 0.153030
## interview_age               -0.02114    0.01638  -1.291 0.196961
## PDS_score:mOFC_rvsn_ant_z    0.13133    0.23937   0.549 0.583311
## ---
## Signif. codes:  0 '***' 0.001 '**' 0.01 '*' 0.05 '.' 0.1 ' ' 1
```

```
##
##
## R-sq.(adj) =  0.0134
## lmer.REML =  12296  Scale est. = 11.364    n = 1995
```

```
##                                  stdcoef      stdse
## X(Intercept)                 0.000000000 0.00000000
## XPDS_score                   0.088811347 0.02387143
## XmOFC_rvsn_ant_z            -0.004478575 0.05300058
## Xrace.ethnicity.5levelBlack  0.031642800 0.05502242
## Xrace.ethnicity.5levelMixed  0.138775931 0.05435980
## Xrace.ethnicity.5levelOther  0.093688857 0.04053143
## Xrace.ethnicity.5levelWhite  0.112013216 0.07200151
## Xdemo_race_hispanic1         0.037053451 0.02592132
## Xinterview_age              -0.029046868 0.02250487
## XPDS_score:mOFC_rvsn_ant_z   0.029045655 0.05294075
```

Male participants

```
##
## Family: gaussian
## Link function: identity
##
## Formula:
## cbcl_scr_syn_internal_r ~ PDS_score * mOFC_rvsn_ant_z + race.ethnicity.5level +
##     demo_race_hispanic + interview_age
##
## Parametric coefficients:
##                              Estimate Std. Error t value Pr(>|t|)
## (Intercept)                  0.622361   2.005242   0.310  0.75631
## PDS_score                    0.686285   0.232818   2.948  0.00324 **
## mOFC_rvsn_ant_z              0.217975   0.462027   0.472  0.63713
## race.ethnicity.5levelBlack   1.086841   0.866342   1.255  0.20980
## race.ethnicity.5levelMixed   2.697266   0.855655   3.152  0.00164 **
## race.ethnicity.5levelOther   2.781770   0.980968   2.836  0.00462 **
## race.ethnicity.5levelWhite   2.016291   0.803861   2.508  0.01221 *
## demo_race_hispanic1         -0.014152   0.332878  -0.043  0.96609
## interview_age                0.009891   0.015680   0.631  0.52825
## PDS_score:mOFC_rvsn_ant_z    0.018780   0.301677   0.062  0.95037
## ---
## Signif. codes:  0 '***' 0.001 '**' 0.01 '*' 0.05 '.' 0.1 ' ' 1
##
##
## R-sq.(adj) =  0.0102
## lmer.REML =  12260  Scale est. = 18.013    n = 2007
```

```
##                                  stdcoef      stdse
## X(Intercept)                 0.000000000 0.00000000
## XPDS_score                   0.069700171 0.02364538
## XmOFC_rvsn_ant_z             0.028062735 0.05948261
## Xrace.ethnicity.5levelBlack  0.067640207 0.05391732
## Xrace.ethnicity.5levelMixed  0.171479098 0.05439839
## Xrace.ethnicity.5levelOther  0.111613818 0.03935968
```

```
## Xrace.ethnicity.5levelWhite  0.179665859 0.07162969
## Xdemo_race_hispanic1        -0.001093349 0.02571684
## Xinterview_age               0.014339695 0.02273278
## XPDS_score:mOFC_rvsn_ant_z   0.003714531 0.05966866
```

4.6 Model: CBCL internalizing factor ~ PDS x Accumbens activity (feedback)

Female participants

```
##
## Family: gaussian
## Link function: identity
##
## Formula:
## cbcl_scr_syn_internal_r ~ PDS_score * accumbens_posvsneg_feedback_z +
##     race.ethnicity.5level + demo_race_hispanic + interview_age
##
## Parametric coefficients:
##                                     Estimate Std. Error t value Pr(>|t|)
## (Intercept)                          4.86589    2.07602   2.344  0.01918
## PDS_score                            0.68636    0.17813   3.853  0.00012
## accumbens_posvsneg_feedback_z       -0.39320    0.44400  -0.886  0.37595
## race.ethnicity.5levelBlack           0.54437    0.88849   0.613  0.54015
## race.ethnicity.5levelMixed           2.21939    0.87236   2.544  0.01103
## race.ethnicity.5levelOther           2.36260    0.98666   2.395  0.01673
## race.ethnicity.5levelWhite           1.34627    0.82071   1.640  0.10108
## demo_race_hispanic1                  0.42095    0.34808   1.209  0.22667
## interview_age                       -0.02196    0.01627  -1.349  0.17744
## PDS_score:accumbens_posvsneg_feedback_z  0.20945    0.24541   0.853  0.39350
##
## (Intercept)                         *
## PDS_score                           ***
## accumbens_posvsneg_feedback_z
## race.ethnicity.5levelBlack
## race.ethnicity.5levelMixed          *
## race.ethnicity.5levelOther          *
## race.ethnicity.5levelWhite
## demo_race_hispanic1
## interview_age
## PDS_score:accumbens_posvsneg_feedback_z
## ---
## Signif. codes:  0 '***' 0.001 '**' 0.01 '*' 0.05 '.' 0.1 ' ' 1
##
##
## R-sq.(adj) =  0.0121
## lmer.REML =  12340  Scale est. = 11.244    n = 2005
```

```
##                                         stdcoef      stdse
## X(Intercept)                         0.00000000 0.00000000
## XPDS_score                           0.09184209 0.02383526
## Xaccumbens_posvsneg_feedback_z      -0.04725519 0.05336031
## Xrace.ethnicity.5levelBlack          0.03353625 0.05473619
## Xrace.ethnicity.5levelMixed          0.13741419 0.05401235
```

```
## Xrace.ethnicity.5levelOther               0.09659968 0.04034147
## Xrace.ethnicity.5levelWhite               0.11721814 0.07145787
## Xdemo_race_hispanic1                      0.03133133 0.02590731
## Xinterview_age                           -0.03031982 0.02247321
## XPDS_score:accumbens_posvsneg_feedback_z  0.04536103 0.05314860
```

Male participants

```
##
## Family: gaussian
## Link function: identity
##
## Formula:
## cbcl_scr_syn_internal_r ~ PDS_score * accumbens_posvsneg_feedback_z +
##     race.ethnicity.5level + demo_race_hispanic + interview_age
##
## Parametric coefficients:
##                                          Estimate Std. Error t value Pr(>|t|)
## (Intercept)                             9.718e-01  1.998e+00   0.486 0.626804
## PDS_score                               7.123e-01  2.299e-01   3.098 0.001972
## accumbens_posvsneg_feedback_z           1.674e-02  4.468e-01   0.037 0.970122
## race.ethnicity.5levelBlack              1.244e+00  8.632e-01   1.441 0.149834
## race.ethnicity.5levelMixed              2.849e+00  8.519e-01   3.344 0.000842
## race.ethnicity.5levelOther              2.956e+00  9.815e-01   3.012 0.002628
## race.ethnicity.5levelWhite              2.101e+00  8.012e-01   2.623 0.008790
## demo_race_hispanic1                     7.092e-05  3.327e-01   0.000 0.999830
## interview_age                           5.707e-03  1.562e-02   0.365 0.714890
## PDS_score:accumbens_posvsneg_feedback_z 2.356e-01  3.046e-01   0.774 0.439232
##
## (Intercept)
## PDS_score                               **
## accumbens_posvsneg_feedback_z
## race.ethnicity.5levelBlack
## race.ethnicity.5levelMixed              ***
## race.ethnicity.5levelOther              **
## race.ethnicity.5levelWhite              **
## demo_race_hispanic1
## interview_age
## PDS_score:accumbens_posvsneg_feedback_z
## ---
## Signif. codes:  0 '***' 0.001 '**' 0.01 '*' 0.05 '.' 0.1 ' ' 1
##
##
## R-sq.(adj) =  0.0113
## lmer.REML =  12294  Scale est. = 18.671    n = 2014
```

```
##                                               stdcoef      stdse
## X(Intercept)                             0.000000e+00 0.00000000
## XPDS_score                               7.260536e-02 0.02343254
## Xaccumbens_posvsneg_feedback_z           2.270536e-03 0.06061325
## Xrace.ethnicity.5levelBlack              7.800362e-02 0.05414395
## Xrace.ethnicity.5levelMixed              1.815168e-01 0.05428687
## Xrace.ethnicity.5levelOther              1.174139e-01 0.03898166
```

```
## Xrace.ethnicity.5levelWhite              1.877074e-01 0.07157101
## Xdemo_race_hispanic1                     5.467682e-06 0.02565261
## Xinterview_age                           8.278766e-03 0.02265989
## XPDS_score:accumbens_posvsneg_feedback_z 4.694157e-02 0.06067585
```

4.7 Model: CBCL internalizing factor ~ PDS x Caudate activity (feedback)

Female participants

```
##
## Family: gaussian
## Link function: identity
##
## Formula:
## cbcl_scr_syn_internal_r ~ PDS_score * caudate_posvsneg_feedback_z +
##     race.ethnicity.5level + demo_race_hispanic + interview_age
##
## Parametric coefficients:
##                                      Estimate Std. Error t value Pr(>|t|)
## (Intercept)                           5.24064    2.08996   2.508   0.0122 *
## PDS_score                             0.70970    0.17965   3.951 8.07e-05 ***
## caudate_posvsneg_feedback_z          -0.42312    0.33771  -1.253   0.2104
## race.ethnicity.5levelBlack            0.54975    0.89324   0.615   0.5383
## race.ethnicity.5levelMixed            2.22349    0.87503   2.541   0.0111 *
## race.ethnicity.5levelOther            2.17874    0.99090   2.199   0.0280 *
## race.ethnicity.5levelWhite            1.27525    0.82348   1.549   0.1216
## demo_race_hispanic1                   0.49242    0.34972   1.408   0.1593
## interview_age                        -0.02506    0.01638  -1.530   0.1263
## PDS_score:caudate_posvsneg_feedback_z  0.15205    0.18800   0.809   0.4187
## ---
## Signif. codes:  0 '***' 0.001 '**' 0.01 '*' 0.05 '.' 0.1 ' ' 1
##
##
## R-sq.(adj) =  0.0138
## lmer.REML =  12303  Scale est. = 11.31     n = 1997
```

```
##                                           stdcoef      stdse
## X(Intercept)                           0.00000000 0.00000000
## XPDS_score                             0.09443322 0.02390384
## Xcaudate_posvsneg_feedback_z          -0.06710132 0.05355554
## Xrace.ethnicity.5levelBlack            0.03356088 0.05453082
## Xrace.ethnicity.5levelMixed            0.13757834 0.05414223
## Xrace.ethnicity.5levelOther            0.08848400 0.04024316
## Xrace.ethnicity.5levelWhite            0.11053134 0.07137481
## Xdemo_race_hispanic1                   0.03652502 0.02594028
## Xinterview_age                        -0.03447507 0.02253990
## XPDS_score:caudate_posvsneg_feedback_z  0.04368556 0.05401517
```

Male participants

```
##
## Family: gaussian
```

```
## Link function: identity
##
## Formula:
## cbcl_scr_syn_internal_r ~ PDS_score * caudate_posvsneg_feedback_z +
##     race.ethnicity.5level + demo_race_hispanic + interview_age
##
## Parametric coefficients:
##                                      Estimate Std. Error t value Pr(>|t|)
## (Intercept)                          1.660869   2.025202   0.820 0.412256
## PDS_score                            0.796861   0.232639   3.425 0.000626 ***
## caudate_posvsneg_feedback_z         -0.151916   0.356251  -0.426 0.669840
## race.ethnicity.5levelBlack           1.121802   0.878802   1.277 0.201922
## race.ethnicity.5levelMixed           2.796188   0.869009   3.218 0.001313 **
## race.ethnicity.5levelOther           2.892817   0.994158   2.910 0.003656 **
## race.ethnicity.5levelWhite           2.070243   0.818202   2.530 0.011475 *
## demo_race_hispanic1                  0.069761   0.335517   0.208 0.835313
## interview_age                       -0.000388   0.015764  -0.025 0.980366
## PDS_score:caudate_posvsneg_feedback_z  0.207350   0.238202   0.870 0.384142
## ---
## Signif. codes:  0 '***' 0.001 '**' 0.01 '*' 0.05 '.' 0.1 ' ' 1
##
##
## R-sq.(adj) =  0.0108
## lmer.REML =  12345  Scale est. = 18.427    n = 2016
```

```
##                                           stdcoef      stdse
## X(Intercept)                          0.000000000 0.00000000
## XPDS_score                            0.080420355 0.02347821
## Xcaudate_posvsneg_feedback_z         -0.025476897 0.05974461
## Xrace.ethnicity.5levelBlack           0.070193320 0.05498835
## Xrace.ethnicity.5levelMixed           0.176489565 0.05485002
## Xrace.ethnicity.5levelOther           0.115012321 0.03952564
## Xrace.ethnicity.5levelWhite           0.183604445 0.07256417
## Xdemo_race_hispanic1                  0.005342790 0.02569638
## Xinterview_age                       -0.000557737 0.02266052
## XPDS_score:caudate_posvsneg_feedback_z  0.052004275 0.05974202
```

4.8 Model: CBCL internalizing factor ~ PDS x Putamen activity (feedback)

Female participants

```
##
## Family: gaussian
## Link function: identity
##
## Formula:
## cbcl_scr_syn_internal_r ~ PDS_score * putamen_posvsneg_feedback_z +
##     race.ethnicity.5level + demo_race_hispanic + interview_age
##
## Parametric coefficients:
##                                     Estimate Std. Error t value Pr(>|t|)
## (Intercept)                          4.86580    2.08450   2.334 0.019680 *
## PDS_score                            0.67167    0.17939   3.744 0.000186 ***
```

```
## putamen_posvsneg_feedback_z            0.04503    0.35173   0.128 0.898143
## race.ethnicity.5levelBlack             0.63545    0.89359   0.711 0.477091
## race.ethnicity.5levelMixed             2.29627    0.87490   2.625 0.008742 **
## race.ethnicity.5levelOther             2.27889    0.99332   2.294 0.021882 *
## race.ethnicity.5levelWhite             1.32205    0.82379   1.605 0.108689
## demo_race_hispanic1                    0.51174    0.34948   1.464 0.143267
## interview_age                         -0.02187    0.01634  -1.338 0.181011
## PDS_score:putamen_posvsneg_feedback_z -0.08259    0.19310  -0.428 0.668914
## ---
## Signif. codes:  0 '***' 0.001 '**' 0.01 '*' 0.05 '.' 0.1 ' ' 1
##
##
## R-sq.(adj) =  0.0131
## lmer.REML =  12299  Scale est. = 11.345    n = 1996
```

```
##                                            stdcoef      stdse
## X(Intercept)                            0.00000000 0.00000000
## XPDS_score                              0.08956683 0.02392100
## Xputamen_posvsneg_feedback_z            0.00678795 0.05302087
## Xrace.ethnicity.5levelBlack             0.03880078 0.05456262
## Xrace.ethnicity.5levelMixed             0.14234404 0.05423465
## Xrace.ethnicity.5levelOther             0.09170833 0.03997370
## Xrace.ethnicity.5levelWhite             0.11455641 0.07138204
## Xdemo_race_hispanic1                    0.03792995 0.02590293
## Xinterview_age                         -0.03010194 0.02249573
## XPDS_score:putamen_posvsneg_feedback_z -0.02279752 0.05330223
```

Male participants

```
##
## Family: gaussian
## Link function: identity
##
## Formula:
## cbcl_scr_syn_internal_r ~ PDS_score * putamen_posvsneg_feedback_z +
##     race.ethnicity.5level + demo_race_hispanic + interview_age
##
## Parametric coefficients:
##                                        Estimate Std. Error t value Pr(>|t|)
## (Intercept)                            1.360166   2.020508   0.673 0.500910
## PDS_score                              0.762492   0.232428   3.281 0.001054 **
## putamen_posvsneg_feedback_z           -0.309543   0.362742  -0.853 0.393572
## race.ethnicity.5levelBlack             1.160402   0.872609   1.330 0.183733
## race.ethnicity.5levelMixed             2.857744   0.862177   3.315 0.000934 ***
## race.ethnicity.5levelOther             2.924746   0.990420   2.953 0.003183 **
## race.ethnicity.5levelWhite             2.152726   0.811334   2.653 0.008033 **
## demo_race_hispanic1                    0.006837   0.337189   0.020 0.983825
## interview_age                          0.002033   0.015773   0.129 0.897473
## PDS_score:putamen_posvsneg_feedback_z  0.332882   0.244131   1.364 0.172866
## ---
## Signif. codes:  0 '***' 0.001 '**' 0.01 '*' 0.05 '.' 0.1 ' ' 1
##
##
```

```
## R-sq.(adj) =  0.0108
## lmer.REML =  12387  Scale est. = 18.667    n = 2021

##                                             stdcoef      stdse
## X(Intercept)                           0.0000000000 0.00000000
## XPDS_score                             0.0768104463 0.02341388
## Xputamen_posvsneg_feedback_z          -0.0515747401 0.06043866
## Xrace.ethnicity.5levelBlack            0.0723038142 0.05437164
## Xrace.ethnicity.5levelMixed            0.1799263971 0.05428355
## Xrace.ethnicity.5levelOther            0.1157805167 0.03920727
## Xrace.ethnicity.5levelWhite            0.1903577875 0.07174331
## Xdemo_race_hispanic1                   0.0005209947 0.02569557
## Xinterview_age                         0.0029171056 0.02263603
## XPDS_score:putamen_posvsneg_feedback_z  0.0823487608 0.06039344
```

4.9 Model: CBCL internalizing factor ~ PDS x Lateral OFC activity (feedback stage)

Female participants

```
##
## Family: gaussian
## Link function: identity
##
## Formula:
## cbcl_scr_syn_internal_r ~ PDS_score * lOFC_posvsneg_feedback_z +
##     race.ethnicity.5level + demo_race_hispanic + interview_age
##
## Parametric coefficients:
##                                  Estimate Std. Error t value Pr(>|t|)
## (Intercept)                       4.92052    2.08082   2.365 0.018140 *
## PDS_score                         0.68518    0.17862   3.836 0.000129 ***
## lOFC_posvsneg_feedback_z         -0.67670    0.57385  -1.179 0.238452
## race.ethnicity.5levelBlack        0.54472    0.88932   0.613 0.540267
## race.ethnicity.5levelMixed        2.21866    0.87231   2.543 0.011052 *
## race.ethnicity.5levelOther        2.47787    0.99339   2.494 0.012699 *
## race.ethnicity.5levelWhite        1.30039    0.82024   1.585 0.113041
## demo_race_hispanic1               0.41769    0.34743   1.202 0.229419
## interview_age                    -0.02219    0.01632  -1.360 0.173911
## PDS_score:lOFC_posvsneg_feedback_z  0.26950    0.31121   0.866 0.386604
## ---
## Signif. codes:  0 '***' 0.001 '**' 0.01 '*' 0.05 '.' 0.1 ' ' 1
##
##
## R-sq.(adj) =  0.0134
## lmer.REML =  12272  Scale est. = 11.19     n = 1994

##                                         stdcoef      stdse
## X(Intercept)                         0.00000000 0.00000000
## XPDS_score                           0.09169905 0.02390497
## XlOFC_posvsneg_feedback_z           -0.06300960 0.05343331
## Xrace.ethnicity.5levelBlack          0.03338588 0.05450613
## Xrace.ethnicity.5levelMixed          0.13713763 0.05391830
```

```
## Xrace.ethnicity.5levelOther          0.09961996 0.03993810
## Xrace.ethnicity.5levelWhite          0.11286173 0.07118942
## Xdemo_race_hispanic1                 0.03110200 0.02587032
## Xinterview_age                      -0.03063505 0.02252191
## XPDS_score:lOFC_posvsneg_feedback_z  0.04618863 0.05333658
```

Male participants

```
##
## Family: gaussian
## Link function: identity
##
## Formula:
## cbcl_scr_syn_internal_r ~ PDS_score * lOFC_posvsneg_feedback_z +
##     race.ethnicity.5level + demo_race_hispanic + interview_age
##
## Parametric coefficients:
##                                      Estimate Std. Error t value Pr(>|t|)
## (Intercept)                          0.979028   1.996429   0.490 0.623912
## PDS_score                            0.708560   0.231559   3.060 0.002243 **
## lOFC_posvsneg_feedback_z            -0.261914   0.565936  -0.463 0.643560
## race.ethnicity.5levelBlack           1.172783   0.864706   1.356 0.175163
## race.ethnicity.5levelMixed           2.822926   0.853873   3.306 0.000963 ***
## race.ethnicity.5levelOther           2.744911   0.984921   2.787 0.005371 **
## race.ethnicity.5levelWhite           2.056512   0.803007   2.561 0.010509 *
## demo_race_hispanic1                 -0.003833   0.333298  -0.011 0.990826
## interview_age                        0.006351   0.015611   0.407 0.684169
## PDS_score:lOFC_posvsneg_feedback_z   0.220286   0.383386   0.575 0.565639
## ---
## Signif. codes:  0 '***' 0.001 '**' 0.01 '*' 0.05 '.' 0.1 ' ' 1
##
##
## R-sq.(adj) =  0.00927
## lmer.REML =  12349  Scale est. = 17.93     n = 2022

##                                          stdcoef      stdse
## X(Intercept)                         0.000000000 0.00000000
## XPDS_score                           0.072016982 0.02353526
## XlOFC_posvsneg_feedback_z           -0.027977210 0.06045240
## Xrace.ethnicity.5levelBlack          0.073335943 0.05407142
## Xrace.ethnicity.5levelMixed          0.179947249 0.05443009
## Xrace.ethnicity.5levelOther          0.108667074 0.03899162
## Xrace.ethnicity.5levelWhite          0.183412227 0.07161702
## Xdemo_race_hispanic1                -0.000295504 0.02569613
## Xinterview_age                       0.009221725 0.02266674
## XPDS_score:lOFC_posvsneg_feedback_z  0.034719661 0.06042611
```

4.10 Model: CBCL internalizing factor ~ PDS x Medial OFC activity (feedback stage)

Female participants

```
##
```

```
## Family: gaussian
## Link function: identity
##
## Formula:
## cbcl_scr_syn_internal_r ~ PDS_score * mOFC_posvsneg_feedback_z +
##     race.ethnicity.5level + demo_race_hispanic + interview_age
##
## Parametric coefficients:
##                                   Estimate Std. Error t value Pr(>|t|)
## (Intercept)                        5.04965    2.08298   2.424  0.01543 *
## PDS_score                          0.68950    0.17888   3.855  0.00012 ***
## mOFC_posvsneg_feedback_z          -0.69715    0.48406  -1.440  0.14996
## race.ethnicity.5levelBlack         0.53721    0.89106   0.603  0.54665
## race.ethnicity.5levelMixed         2.19349    0.87432   2.509  0.01219 *
## race.ethnicity.5levelOther         2.30312    0.98997   2.326  0.02009 *
## race.ethnicity.5levelWhite         1.28148    0.82167   1.560  0.11901
## demo_race_hispanic1                0.46194    0.34777   1.328  0.18423
## interview_age                     -0.02318    0.01634  -1.419  0.15610
## PDS_score:mOFC_posvsneg_feedback_z  0.30275    0.26566   1.140  0.25459
## ---
## Signif. codes:  0 '***' 0.001 '**' 0.01 '*' 0.05 '.' 0.1 ' ' 1
##
##
## R-sq.(adj) =  0.0142
## lmer.REML =  12281  Scale est. = 11.435    n = 1994
```

```
##                                         stdcoef      stdse
## X(Intercept)                         0.00000000 0.00000000
## XPDS_score                           0.09205752 0.02388247
## XmOFC_posvsneg_feedback_z           -0.07972625 0.05535673
## Xrace.ethnicity.5levelBlack          0.03274690 0.05431612
## Xrace.ethnicity.5levelMixed          0.13531019 0.05393443
## Xrace.ethnicity.5levelOther          0.09372436 0.04028628
## Xrace.ethnicity.5levelWhite          0.11104141 0.07119834
## Xdemo_race_hispanic1                 0.03439118 0.02589090
## Xinterview_age                      -0.03189948 0.02248257
## XPDS_score:mOFC_posvsneg_feedback_z  0.06357237 0.05578438
```

Male participants

```
##
## Family: gaussian
## Link function: identity
##
## Formula:
## cbcl_scr_syn_internal_r ~ PDS_score * mOFC_posvsneg_feedback_z +
##     race.ethnicity.5level + demo_race_hispanic + interview_age
##
## Parametric coefficients:
##                                     Estimate Std. Error t value Pr(>|t|)
## (Intercept)                         1.034110   1.995537   0.518 0.604368
## PDS_score                           0.710784   0.231505   3.070 0.002167 **
## mOFC_posvsneg_feedback_z           -0.063667   0.507003  -0.126 0.900081
```

```
## race.ethnicity.5levelBlack          1.166348   0.864096   1.350 0.177235
## race.ethnicity.5levelMixed          2.840113   0.853678   3.327 0.000894 ***
## race.ethnicity.5levelOther          2.804023   0.982024   2.855 0.004343 **
## race.ethnicity.5levelWhite          2.063742   0.802717   2.571 0.010213 *
## demo_race_hispanic1                -0.021981   0.332877  -0.066 0.947358
## interview_age                       0.005773   0.015597   0.370 0.711295
## PDS_score:mOFC_posvsneg_feedback_z  0.249767   0.350278   0.713 0.475896
## ---
## Signif. codes:  0 '***' 0.001 '**' 0.01 '*' 0.05 '.' 0.1 ' ' 1
##
##
## R-sq.(adj) =  0.0109
## lmer.REML =  12337  Scale est. = 18.041    n = 2020
```

```
##                                         stdcoef      stdse
## X(Intercept)                        0.000000000 0.00000000
## XPDS_score                          0.072171105 0.02350639
## XmOFC_posvsneg_feedback_z          -0.007832675 0.06237454
## Xrace.ethnicity.5levelBlack         0.073046046 0.05411659
## Xrace.ethnicity.5levelMixed         0.180997361 0.05440397
## Xrace.ethnicity.5levelOther         0.111577504 0.03907663
## Xrace.ethnicity.5levelWhite         0.184144957 0.07162534
## Xdemo_race_hispanic1               -0.001694137 0.02565583
## Xinterview_age                      0.008376781 0.02262955
## XPDS_score:mOFC_posvsneg_feedback_z  0.044479181 0.06237857
```

4.11 Model: CBCL internalizing factor ~ PDS x BIS-BAS

Female participants

```
##
## Family: gaussian
## Link function: identity
##
## Formula:
## cbcl_scr_syn_internal_r ~ PDS_score * bisbas_ss_basm_rr + race.ethnicity.5level +
##     demo_race_hispanic + interview_age
##
## Parametric coefficients:
##                             Estimate Std. Error t value Pr(>|t|)
## (Intercept)                 2.191763   2.099766   1.044  0.29667
## PDS_score                   1.574106   0.551603   2.854  0.00436 **
## bisbas_ss_basm_rr           0.114562   0.111110   1.031  0.30260
## race.ethnicity.5levelBlack  0.201260   0.791776   0.254  0.79937
## race.ethnicity.5levelMixed  1.868473   0.787599   2.372  0.01775 *
## race.ethnicity.5levelOther  2.513910   0.901229   2.789  0.00532 **
## race.ethnicity.5levelWhite  1.340999   0.740403   1.811  0.07023 .
## demo_race_hispanic1         0.164739   0.316995   0.520  0.60332
## interview_age              -0.004925   0.014590  -0.338  0.73572
## PDS_score:bisbas_ss_basm_rr -0.107740   0.059762  -1.803  0.07153 .
## ---
## Signif. codes:  0 '***' 0.001 '**' 0.01 '*' 0.05 '.' 0.1 ' ' 1
##
```

```
##
## R-sq.(adj) =  0.0132
## lmer.REML =  16324  Scale est. = 13.08     n = 2629
```

```
##                                   stdcoef      stdse
## X(Intercept)                  0.000000000 0.00000000
## XPDS_score                    0.209729583 0.07349406
## Xbisbas_ss_basm_rr            0.048498991 0.04703765
## Xrace.ethnicity.5levelBlack   0.013141938 0.05170153
## Xrace.ethnicity.5levelMixed   0.113552923 0.04786484
## Xrace.ethnicity.5levelOther   0.096398587 0.03455859
## Xrace.ethnicity.5levelWhite   0.116005473 0.06404984
## Xdemo_race_hispanic1          0.011826552 0.02275701
## Xinterview_age               -0.006689046 0.01981553
## XPDS_score:bisbas_ss_basm_rr -0.156103576 0.08658904
```

Male participants

```
##
## Family: gaussian
## Link function: identity
##
## Formula:
## cbcl_scr_syn_internal_r ~ PDS_score * bisbas_ss_basm_rr + race.ethnicity.5level +
##     demo_race_hispanic + interview_age
##
## Parametric coefficients:
##                               Estimate Std. Error t value Pr(>|t|)
## (Intercept)                  4.7006846  2.0841176   2.255  0.02418 *
## PDS_score                   -0.8611461  0.7896629  -1.091  0.27558
## bisbas_ss_basm_rr           -0.2500233  0.1188474  -2.104  0.03549 *
## race.ethnicity.5levelBlack   1.2630721  0.7537979   1.676  0.09392 .
## race.ethnicity.5levelMixed   1.9863925  0.7541973   2.634  0.00849 **
## race.ethnicity.5levelOther   1.8142217  0.8610122   2.107  0.03520 *
## race.ethnicity.5levelWhite   1.4422989  0.7072059   2.039  0.04150 *
## demo_race_hispanic1          0.2546559  0.3010106   0.846  0.39762
## interview_age               -0.0005912  0.0140177  -0.042  0.96636
## PDS_score:bisbas_ss_basm_rr  0.1848411  0.0826208   2.237  0.02535 *
## ---
## Signif. codes:  0 '***' 0.001 '**' 0.01 '*' 0.05 '.' 0.1 ' ' 1
##
##
## R-sq.(adj) =  0.00836
## lmer.REML =  17683  Scale est. = 16.106    n = 2841
```

```
##                                    stdcoef      stdse
## X(Intercept)                  0.0000000000 0.00000000
## XPDS_score                   -0.0855145875 0.07841607
## Xbisbas_ss_basm_rr           -0.1047253177 0.04978071
## Xrace.ethnicity.5levelBlack   0.0810395185 0.04836416
## Xrace.ethnicity.5levelMixed   0.1179860170 0.04479715
## Xrace.ethnicity.5levelOther   0.0709229131 0.03365933
## Xrace.ethnicity.5levelWhite   0.1240563284 0.06082884
```

```
## Xdemo_race_hispanic1          0.0183849765 0.02173157
## Xinterview_age               -0.0008048901 0.01908485
## XPDS_score:bisbas_ss_basm_rr  0.2076296018 0.09280685
```

4.12 Model: CBCL internalizing factor ~ PDS x MID reaction time (large reward vs. neutral)

Female participants

```
##
## Family: gaussian
## Link function: identity
##
## Formula:
## cbcl_scr_syn_internal_r ~ PDS_score * rt_diff_large_neutral_z +
##     race.ethnicity.5level + demo_race_hispanic + interview_age
##
## Parametric coefficients:
##                                   Estimate Std. Error t value Pr(>|t|)
## (Intercept)                       5.501431   1.992806   2.761  0.00582 **
## PDS_score                         0.641948   0.172915   3.713  0.00021 ***
## rt_diff_large_neutral_z           0.154591   0.311300   0.497  0.61952
## race.ethnicity.5levelBlack        0.560716   0.845384   0.663  0.50723
## race.ethnicity.5levelMixed        2.155255   0.833294   2.586  0.00976 **
## race.ethnicity.5levelOther        2.598824   0.947143   2.744  0.00612 **
## race.ethnicity.5levelWhite        1.320738   0.781113   1.691  0.09101 .
## demo_race_hispanic1               0.456433   0.341115   1.338  0.18102
## interview_age                    -0.026474   0.015690  -1.687  0.09170 .
## PDS_score:rt_diff_large_neutral_z -0.008308   0.171353  -0.048  0.96133
## ---
## Signif. codes:  0 '***' 0.001 '**' 0.01 '*' 0.05 '.' 0.1 ' ' 1
##
##
## R-sq.(adj) =  0.0132
## lmer.REML =  13258  Scale est. = 11.823    n = 2153
```

```
##                                        stdcoef      stdse
## X(Intercept)                       0.000000000 0.00000000
## XPDS_score                         0.085432620 0.02301209
## Xrt_diff_large_neutral_z           0.026457754 0.05327813
## Xrace.ethnicity.5levelBlack        0.035027385 0.05281032
## Xrace.ethnicity.5levelMixed        0.131975792 0.05102624
## Xrace.ethnicity.5levelOther        0.104261170 0.03799806
## Xrace.ethnicity.5levelWhite        0.114828644 0.06791216
## Xdemo_race_hispanic1               0.033597952 0.02510939
## Xinterview_age                    -0.036666351 0.02173111
## XPDS_score:rt_diff_large_neutral_z -0.002595203 0.05352500
```

Male participants

```
##
## Family: gaussian
```

```
## Link function: identity
##
## Formula:
## cbcl_scr_syn_internal_r ~ PDS_score * rt_diff_large_neutral_z +
##     race.ethnicity.5level + demo_race_hispanic + interview_age
##
## Parametric coefficients:
##                                  Estimate Std. Error t value Pr(>|t|)
## (Intercept)                      1.468491   1.936596   0.758  0.44836
## PDS_score                        0.622357   0.221670   2.808  0.00503 **
## rt_diff_large_neutral_z          0.615672   0.346277   1.778  0.07554 .
## race.ethnicity.5levelBlack       0.747830   0.844881   0.885  0.37618
## race.ethnicity.5levelMixed       2.159276   0.837486   2.578  0.00999 **
## race.ethnicity.5levelOther       1.993475   0.961808   2.073  0.03832 *
## race.ethnicity.5levelWhite       1.472352   0.790394   1.863  0.06262 .
## demo_race_hispanic1              0.097591   0.322937   0.302  0.76253
## interview_age                    0.008204   0.015067   0.544  0.58618
## PDS_score:rt_diff_large_neutral_z -0.391427   0.240096  -1.630  0.10318
## ---
## Signif. codes:  0 '***' 0.001 '**' 0.01 '*' 0.05 '.' 0.1 ' ' 1
##
##
## R-sq.(adj) =  0.00718
## lmer.REML =  13840  Scale est. = 17.748    n = 2251
```

```
##                                        stdcoef      stdse
## X(Intercept)                       0.000000000 0.00000000
## XPDS_score                         0.062424145 0.02223409
## Xrt_diff_large_neutral_z           0.102340818 0.05756040
## Xrace.ethnicity.5levelBlack        0.047305062 0.05344412
## Xrace.ethnicity.5levelMixed        0.136588098 0.05297635
## Xrace.ethnicity.5levelOther        0.078885051 0.03806030
## Xrace.ethnicity.5levelWhite        0.130288521 0.06994200
## Xdemo_race_hispanic1               0.007341529 0.02429389
## Xinterview_age                     0.011689322 0.02146953
## XPDS_score:rt_diff_large_neutral_z -0.093733703 0.05749496
```

4.13 Model: CBCL internalizing factor ~ PDS x MID reaction time (large vs. small reward)

Female participants

```
##
## Family: gaussian
## Link function: identity
##
## Formula:
## cbcl_scr_syn_internal_r ~ PDS_score * rt_diff_large_small_z +
##     race.ethnicity.5level + demo_race_hispanic + interview_age
##
## Parametric coefficients:
##                               Estimate Std. Error t value Pr(>|t|)
## (Intercept)                    5.41837    1.99084   2.722 0.006548 **
```

```
## PDS_score                        0.62952    0.17249   3.650 0.000269 ***
## rt_diff_large_small_z           -0.54788    0.29956  -1.829 0.067547 .
## race.ethnicity.5levelBlack       0.56487    0.84371   0.670 0.503246
## race.ethnicity.5levelMixed       2.14230    0.83251   2.573 0.010140 *
## race.ethnicity.5levelOther       2.55705    0.94673   2.701 0.006969 **
## race.ethnicity.5levelWhite       1.29990    0.78025   1.666 0.095859 .
## demo_race_hispanic1              0.45983    0.34088   1.349 0.177498
## interview_age                   -0.02539    0.01567  -1.621 0.105253
## PDS_score:rt_diff_large_small_z  0.25827    0.16476   1.568 0.117134
## ---
## Signif. codes:  0 '***' 0.001 '**' 0.01 '*' 0.05 '.' 0.1 ' ' 1
##
##
## R-sq.(adj) =  0.0143
## lmer.REML =  13256  Scale est. = 11.79     n = 2153
```

```
##                                      stdcoef      stdse
## X(Intercept)                      0.00000000 0.00000000
## XPDS_score                        0.08377894 0.02295527
## Xrt_diff_large_small_z           -0.09440033 0.05161478
## Xrace.ethnicity.5levelBlack       0.03528668 0.05270563
## Xrace.ethnicity.5levelMixed       0.13118268 0.05097799
## Xrace.ethnicity.5levelOther       0.10258523 0.03798156
## Xrace.ethnicity.5levelWhite       0.11301707 0.06783728
## Xdemo_race_hispanic1              0.03384796 0.02509224
## Xinterview_age                   -0.03516595 0.02169951
## XPDS_score:rt_diff_large_small_z  0.08112913 0.05175544
```

Male participants

```
##
## Family: gaussian
## Link function: identity
##
## Formula:
## cbcl_scr_syn_internal_r ~ PDS_score * rt_diff_large_small_z +
##     race.ethnicity.5level + demo_race_hispanic + interview_age
##
## Parametric coefficients:
##                                  Estimate Std. Error t value Pr(>|t|)
## (Intercept)                      1.495433   1.937391   0.772  0.44027
## PDS_score                        0.625956   0.221517   2.826  0.00476 **
## rt_diff_large_small_z            0.112502   0.346367   0.325  0.74536
## race.ethnicity.5levelBlack       0.702795   0.844996   0.832  0.40566
## race.ethnicity.5levelMixed       2.106456   0.837600   2.515  0.01198 *
## race.ethnicity.5levelOther       1.868262   0.960621   1.945  0.05192 .
## race.ethnicity.5levelWhite       1.410928   0.790353   1.785  0.07437 .
## demo_race_hispanic1              0.094464   0.323247   0.292  0.77013
## interview_age                    0.008503   0.015075   0.564  0.57278
## PDS_score:rt_diff_large_small_z -0.142787   0.241824  -0.590  0.55494
## ---
## Signif. codes:  0 '***' 0.001 '**' 0.01 '*' 0.05 '.' 0.1 ' ' 1
##
```

```
##
## R-sq.(adj) =  0.00581
## lmer.REML =  13842  Scale est. = 17.609    n = 2251
```

```
##                                      stdcoef      stdse
## X(Intercept)                      0.000000000 0.00000000
## XPDS_score                        0.062785201 0.02221882
## Xrt_diff_large_small_z            0.018914894 0.05823430
## Xrace.ethnicity.5levelBlack       0.044456297 0.05345140
## Xrace.ethnicity.5levelMixed       0.133246850 0.05298360
## Xrace.ethnicity.5levelOther       0.073930194 0.03801336
## Xrace.ethnicity.5levelWhite       0.124853059 0.06993836
## Xdemo_race_hispanic1              0.007106301 0.02431718
## Xinterview_age                    0.012115652 0.02148020
## XPDS_score:rt_diff_large_small_z -0.034405634 0.05826948
```

4.14 Model: CBCL internalizing factor ~ Testosterone x Accumbens activity (anticipation stage) + PDS

Female participants

```
##
## Family: gaussian
## Link function: identity
##
## Formula:
## cbcl_scr_syn_internal_r ~ PDS_score + hormone_scr_ert_mean *
##     accumbens_rvsn_ant_z + race.ethnicity.5level + demo_race_hispanic +
##     interview_age
##
## Parametric coefficients:
##                                       Estimate Std. Error t value Pr(>|t|)
## (Intercept)                           4.460740   2.119085   2.105 0.035422
## PDS_score                             0.665395   0.191155   3.481 0.000511
## hormone_scr_ert_mean                  0.002798   0.008101   0.345 0.729815
## accumbens_rvsn_ant_z                  0.182512   0.418879   0.436 0.663094
## race.ethnicity.5levelBlack            0.220171   0.899271   0.245 0.806613
## race.ethnicity.5levelMixed            2.173556   0.878765   2.473 0.013471
## race.ethnicity.5levelOther            2.237816   0.998934   2.240 0.025195
## race.ethnicity.5levelWhite            1.325853   0.823882   1.609 0.107726
## demo_race_hispanic1                   0.355402   0.358674   0.991 0.321874
## interview_age                        -0.018447   0.016899  -1.092 0.275143
## hormone_scr_ert_mean:accumbens_rvsn_ant_z -0.006376   0.011134  -0.573 0.566914
##
## (Intercept)                          *
## PDS_score                            ***
## hormone_scr_ert_mean
## accumbens_rvsn_ant_z
## race.ethnicity.5levelBlack
## race.ethnicity.5levelMixed           *
## race.ethnicity.5levelOther           *
## race.ethnicity.5levelWhite
## demo_race_hispanic1
```

```
## interview_age
## hormone_scr_ert_mean:accumbens_rvsn_ant_z
## ---
## Signif. codes:  0 '***' 0.001 '**' 0.01 '*' 0.05 '.' 0.1 ' ' 1
##
##
## R-sq.(adj) =  0.0115
## lmer.REML =  11518  Scale est. = 10.565    n = 1870
```

```
##                                                stdcoef      stdse
## X(Intercept)                               0.000000000 0.00000000
## XPDS_score                                 0.088048832 0.02529474
## Xhormone_scr_ert_mean                      0.008367511 0.02422393
## Xaccumbens_rvsn_ant_z                      0.022981740 0.05274494
## Xrace.ethnicity.5levelBlack                0.013159110 0.05374735
## Xrace.ethnicity.5levelMixed                0.135545710 0.05480091
## Xrace.ethnicity.5levelOther                0.091880743 0.04101443
## Xrace.ethnicity.5levelWhite                0.115032704 0.07148108
## Xdemo_race_hispanic1                       0.026450768 0.02669426
## Xinterview_age                            -0.025633451 0.02348207
## Xhormone_scr_ert_mean:accumbens_rvsn_ant_z -0.030085404 0.05253210
```

Male participants

```
##
## Family: gaussian
## Link function: identity
##
## Formula:
## cbcl_scr_syn_internal_r ~ PDS_score + hormone_scr_ert_mean *
##     accumbens_rvsn_ant_z + race.ethnicity.5level + demo_race_hispanic +
##     interview_age
##
## Parametric coefficients:
##                                            Estimate Std. Error t value Pr(>|t|)
## (Intercept)                                0.826356   2.106340   0.392  0.69487
## PDS_score                                  0.807336   0.248609   3.247  0.00119
## hormone_scr_ert_mean                      -0.001599   0.008291  -0.193  0.84712
## accumbens_rvsn_ant_z                      -0.226384   0.363241  -0.623  0.53321
## race.ethnicity.5levelBlack                 1.015589   0.917880   1.106  0.26868
## race.ethnicity.5levelMixed                 2.778456   0.902439   3.079  0.00211
## race.ethnicity.5levelOther                 2.730933   1.035962   2.636  0.00846
## race.ethnicity.5levelWhite                 2.072912   0.848688   2.442  0.01468
## demo_race_hispanic1                        0.093889   0.347187   0.270  0.78686
## interview_age                              0.007195   0.016674   0.431  0.66618
## hormone_scr_ert_mean:accumbens_rvsn_ant_z  0.003889   0.010802   0.360  0.71890
##
## (Intercept)
## PDS_score                                 **
## hormone_scr_ert_mean
## accumbens_rvsn_ant_z
## race.ethnicity.5levelBlack
## race.ethnicity.5levelMixed                **
```

```
## race.ethnicity.5levelOther               **
## race.ethnicity.5levelWhite               *
## demo_race_hispanic1
## interview_age
## hormone_scr_ert_mean:accumbens_rvsn_ant_z
## ---
## Signif. codes:  0 '***' 0.001 '**' 0.01 '*' 0.05 '.' 0.1 ' ' 1
##
##
## R-sq.(adj) =  0.00997
## lmer.REML =  11479  Scale est. = 18.675    n = 1866
```

```
##                                                 stdcoef      stdse
## X(Intercept)                                0.000000000 0.00000000
## XPDS_score                                  0.080086034 0.02466148
## Xhormone_scr_ert_mean                      -0.004656018 0.02414640
## Xaccumbens_rvsn_ant_z                      -0.031496666 0.05053762
## Xrace.ethnicity.5levelBlack                 0.061964362 0.05600285
## Xrace.ethnicity.5levelMixed                 0.174432269 0.05665535
## Xrace.ethnicity.5levelOther                 0.107339563 0.04071859
## Xrace.ethnicity.5levelWhite                 0.181614492 0.07435632
## Xdemo_race_hispanic1                        0.007153489 0.02645243
## Xinterview_age                              0.010256443 0.02377085
## Xhormone_scr_ert_mean:accumbens_rvsn_ant_z  0.018217382 0.05060633
```

4.15 Model: CBCL internalizing factor ~ Testosterone x Caudate activity (anticipation stage) + PDS

Female participants

```
##
## Family: gaussian
## Link function: identity
##
## Formula:
## cbcl_scr_syn_internal_r ~ PDS_score + hormone_scr_ert_mean *
##     caudate_rvsn_ant_z + race.ethnicity.5level + demo_race_hispanic +
##     interview_age
##
## Parametric coefficients:
##                                          Estimate Std. Error t value Pr(>|t|)
## (Intercept)                             4.5348882  2.1219447   2.137 0.032717
## PDS_score                               0.6845890  0.1914868   3.575 0.000359
## hormone_scr_ert_mean                    0.0028676  0.0081083   0.354 0.723629
## caudate_rvsn_ant_z                     -0.0035717  0.3299659  -0.011 0.991365
## race.ethnicity.5levelBlack              0.2633481  0.8995029   0.293 0.769730
## race.ethnicity.5levelMixed              2.1525889  0.8777123   2.452 0.014278
## race.ethnicity.5levelOther              2.2184539  0.9953510   2.229 0.025945
## race.ethnicity.5levelWhite              1.3332409  0.8235547   1.619 0.105642
## demo_race_hispanic1                     0.3429821  0.3576367   0.959 0.337672
## interview_age                          -0.0193397  0.0169371  -1.142 0.253662
## hormone_scr_ert_mean:caudate_rvsn_ant_z  0.0001989  0.0087533   0.023 0.981875
##
```

```
## (Intercept)                             *
## PDS_score                               ***
## hormone_scr_ert_mean
## caudate_rvsn_ant_z
## race.ethnicity.5levelBlack
## race.ethnicity.5levelMixed              *
## race.ethnicity.5levelOther              *
## race.ethnicity.5levelWhite
## demo_race_hispanic1
## interview_age
## hormone_scr_ert_mean:caudate_rvsn_ant_z
## ---
## Signif. codes:  0 '***' 0.001 '**' 0.01 '*' 0.05 '.' 0.1 ' ' 1
##
##
## R-sq.(adj) =  0.0113
## lmer.REML =  11506  Scale est. = 10.618    n = 1868
```

```
##                                              stdcoef      stdse
## X(Intercept)                             0.0000000000 0.00000000
## XPDS_score                               0.0904617420 0.02530311
## Xhormone_scr_ert_mean                    0.0085822122 0.02426639
## Xcaudate_rvsn_ant_z                     -0.0005902608 0.05453016
## Xrace.ethnicity.5levelBlack              0.0156609707 0.05349228
## Xrace.ethnicity.5levelMixed              0.1348256129 0.05497478
## Xrace.ethnicity.5levelOther              0.0916153870 0.04110496
## Xrace.ethnicity.5levelWhite              0.1157573234 0.07150432
## Xdemo_race_hispanic1                     0.0255479878 0.02663958
## Xinterview_age                          -0.0268625946 0.02352540
## Xhormone_scr_ert_mean:caudate_rvsn_ant_z  0.0012411632 0.05462655
```

Male participants

```
##
## Family: gaussian
## Link function: identity
##
## Formula:
## cbcl_scr_syn_internal_r ~ PDS_score + hormone_scr_ert_mean *
##     caudate_rvsn_ant_z + race.ethnicity.5level + demo_race_hispanic +
##     interview_age
##
## Parametric coefficients:
##                                          Estimate Std. Error t value Pr(>|t|)
## (Intercept)                              1.090318   2.116933   0.515 0.606582
## PDS_score                                0.820521   0.248605   3.300 0.000983
## hormone_scr_ert_mean                    -0.001400   0.008342  -0.168 0.866760
## caudate_rvsn_ant_z                       0.207941   0.288077   0.722 0.470495
## race.ethnicity.5levelBlack               0.905254   0.938982   0.964 0.335132
## race.ethnicity.5levelMixed               2.714709   0.924768   2.936 0.003371
## race.ethnicity.5levelOther               2.652355   1.053709   2.517 0.011914
## race.ethnicity.5levelWhite               1.977938   0.872654   2.267 0.023531
## demo_race_hispanic1                      0.094266   0.349284   0.270 0.787280
```

```
## interview_age                           0.005727   0.016720   0.343 0.732003
## hormone_scr_ert_mean:caudate_rvsn_ant_z -0.009114   0.008086  -1.127 0.259871
##
## (Intercept)
## PDS_score                               ***
## hormone_scr_ert_mean
## caudate_rvsn_ant_z
## race.ethnicity.5levelBlack
## race.ethnicity.5levelMixed              **
## race.ethnicity.5levelOther              *
## race.ethnicity.5levelWhite              *
## demo_race_hispanic1
## interview_age
## hormone_scr_ert_mean:caudate_rvsn_ant_z
## ---
## Signif. codes:  0 '***' 0.001 '**' 0.01 '*' 0.05 '.' 0.1 ' ' 1
##
##
## R-sq.(adj) =  0.0103
## lmer.REML =  11473  Scale est. = 18.944    n = 1864
```

```
##                                              stdcoef      stdse
## X(Intercept)                             0.000000000 0.00000000
## XPDS_score                               0.081249074 0.02461724
## Xhormone_scr_ert_mean                   -0.004063508 0.02421654
## Xcaudate_rvsn_ant_z                      0.037011233 0.05127471
## Xrace.ethnicity.5levelBlack              0.055283898 0.05734370
## Xrace.ethnicity.5levelMixed              0.169939493 0.05789003
## Xrace.ethnicity.5levelOther              0.104735783 0.04160869
## Xrace.ethnicity.5levelWhite              0.173000734 0.07632682
## Xdemo_race_hispanic1                     0.007145795 0.02647729
## Xinterview_age                           0.008150815 0.02379699
## Xhormone_scr_ert_mean:caudate_rvsn_ant_z -0.057746001 0.05123680
```

4.16 Model: CBCL internalizing factor ~ Testosterone x Putamen activity (anticipation stage) + PDS

Female participants

```
##
## Family: gaussian
## Link function: identity
##
## Formula:
## cbcl_scr_syn_internal_r ~ PDS_score + hormone_scr_ert_mean *
##     putamen_rvsn_ant_z + race.ethnicity.5level + demo_race_hispanic +
##     interview_age
##
## Parametric coefficients:
##                                         Estimate Std. Error t value Pr(>|t|)
## (Intercept)                             4.341820   2.117114   2.051 0.040425
## PDS_score                               0.685162   0.191490   3.578 0.000355
## hormone_scr_ert_mean                    0.001752   0.008077   0.217 0.828324
```

```
## putamen_rvsn_ant_z                      -0.194659   0.331833  -0.587 0.557532
## race.ethnicity.5levelBlack               0.318982   0.895454   0.356 0.721714
## race.ethnicity.5levelMixed               2.189852   0.874549   2.504 0.012366
## race.ethnicity.5levelOther               2.243043   0.994948   2.254 0.024285
## race.ethnicity.5levelWhite               1.316069   0.820336   1.604 0.108817
## demo_race_hispanic1                      0.361351   0.357142   1.012 0.311772
## interview_age                           -0.017509   0.016893  -1.036 0.300127
## hormone_scr_ert_mean:putamen_rvsn_ant_z  0.004550   0.008692   0.523 0.600715
##
## (Intercept)                             *
## PDS_score                               ***
## hormone_scr_ert_mean
## putamen_rvsn_ant_z
## race.ethnicity.5levelBlack
## race.ethnicity.5levelMixed              *
## race.ethnicity.5levelOther              *
## race.ethnicity.5levelWhite
## demo_race_hispanic1
## interview_age
## hormone_scr_ert_mean:putamen_rvsn_ant_z
## ---
## Signif. codes:  0 '***' 0.001 '**' 0.01 '*' 0.05 '.' 0.1 ' ' 1
##
##
## R-sq.(adj) =  0.0114
## lmer.REML =  11482  Scale est. = 10.569    n = 1866
```

```
##                                               stdcoef      stdse
## X(Intercept)                              0.000000000 0.00000000
## XPDS_score                                0.090800903 0.02537719
## Xhormone_scr_ert_mean                     0.005262216 0.02426301
## Xputamen_rvsn_ant_z                      -0.031273105 0.05331090
## Xrace.ethnicity.5levelBlack               0.019075586 0.05354945
## Xrace.ethnicity.5levelMixed               0.137414357 0.05487842
## Xrace.ethnicity.5levelOther               0.092518827 0.04103862
## Xrace.ethnicity.5levelWhite               0.114607647 0.07143754
## Xdemo_race_hispanic1                      0.027011425 0.02669677
## Xinterview_age                           -0.024416110 0.02355743
## Xhormone_scr_ert_mean:putamen_rvsn_ant_z  0.027821247 0.05314857
```

Male participants

```
##
## Family: gaussian
## Link function: identity
##
## Formula:
## cbcl_scr_syn_internal_r ~ PDS_score + hormone_scr_ert_mean *
##     putamen_rvsn_ant_z + race.ethnicity.5level + demo_race_hispanic +
##     interview_age
##
## Parametric coefficients:
##                                          Estimate Std. Error t value Pr(>|t|)
```

```
## (Intercept)                              0.950507   2.111716   0.450 0.652683
## PDS_score                                0.835710   0.249503   3.349 0.000826
## hormone_scr_ert_mean                    -0.001234   0.008330  -0.148 0.882222
## putamen_rvsn_ant_z                       0.358346   0.285470   1.255 0.209533
## race.ethnicity.5levelBlack               0.938358   0.937376   1.001 0.316935
## race.ethnicity.5levelMixed               2.714538   0.920851   2.948 0.003240
## race.ethnicity.5levelOther               2.456016   1.055552   2.327 0.020085
## race.ethnicity.5levelWhite               1.963689   0.870617   2.256 0.024217
## demo_race_hispanic1                      0.055634   0.348600   0.160 0.873220
## interview_age                            0.006910   0.016685   0.414 0.678820
## hormone_scr_ert_mean:putamen_rvsn_ant_z -0.014910   0.007825  -1.906 0.056869
##
## (Intercept)
## PDS_score                               ***
## hormone_scr_ert_mean
## putamen_rvsn_ant_z
## race.ethnicity.5levelBlack
## race.ethnicity.5levelMixed              **
## race.ethnicity.5levelOther              *
## race.ethnicity.5levelWhite              *
## demo_race_hispanic1
## interview_age
## hormone_scr_ert_mean:putamen_rvsn_ant_z .
## ---
## Signif. codes:  0 '***' 0.001 '**' 0.01 '*' 0.05 '.' 0.1 ' ' 1
##
##
## R-sq.(adj) =  0.0113
## lmer.REML =  11461  Scale est. = 18.242    n = 1864
```

```
##                                              stdcoef      stdse
## X(Intercept)                               0.000000000 0.00000000
## XPDS_score                                 0.082371181 0.02459214
## Xhormone_scr_ert_mean                     -0.003584990 0.02419450
## Xputamen_rvsn_ant_z                        0.063156589 0.05031251
## Xrace.ethnicity.5levelBlack                0.057199532 0.05713969
## Xrace.ethnicity.5levelMixed                0.171840499 0.05829332
## Xrace.ethnicity.5levelOther                0.096091091 0.04129823
## Xrace.ethnicity.5levelWhite                0.172199089 0.07634584
## Xdemo_race_hispanic1                       0.004221928 0.02645460
## Xinterview_age                             0.009853074 0.02379169
## Xhormone_scr_ert_mean:putamen_rvsn_ant_z -0.095896161 0.05032583
```

4.17 Model: CBCL internalizing factor ~ Testosterone x Accumbens activity (feedback stage) + PDS

Female participants

```
##
## Family: gaussian
## Link function: identity
##
## Formula:
```

```
## cbcl_scr_syn_internal_r ~ PDS_score + hormone_scr_ert_mean *
##     accumbens_posvsneg_feedback_z + race.ethnicity.5level + demo_race_hispanic +
##     interview_age
##
## Parametric coefficients:
##                                                   Estimate Std. Error t value
## (Intercept)                                       4.189255   2.110672   1.985
## PDS_score                                         0.677795   0.190161   3.564
## hormone_scr_ert_mean                              0.002331   0.008078   0.289
## accumbens_posvsneg_feedback_z                     0.319909   0.465170   0.688
## race.ethnicity.5levelBlack                        0.284555   0.894195   0.318
## race.ethnicity.5levelMixed                        2.101703   0.874015   2.405
## race.ethnicity.5levelOther                        2.296337   0.991434   2.316
## race.ethnicity.5levelWhite                        1.361645   0.820086   1.660
## demo_race_hispanic1                               0.271252   0.357796   0.758
## interview_age                                    -0.016437   0.016839  -0.976
## hormone_scr_ert_mean:accumbens_posvsneg_feedback_z -0.010360   0.012227  -0.847
##                                                  Pr(>|t|)
## (Intercept)                                      0.047314 *
## PDS_score                                        0.000374 ***
## hormone_scr_ert_mean                             0.772937
## accumbens_posvsneg_feedback_z                    0.491712
## race.ethnicity.5levelBlack                       0.750350
## race.ethnicity.5levelMixed                       0.016285 *
## race.ethnicity.5levelOther                       0.020657 *
## race.ethnicity.5levelWhite                       0.097009 .
## demo_race_hispanic1                              0.448475
## interview_age                                    0.329146
## hormone_scr_ert_mean:accumbens_posvsneg_feedback_z 0.396926
## ---
## Signif. codes:  0 '***' 0.001 '**' 0.01 '*' 0.05 '.' 0.1 ' ' 1
##
##
## R-sq.(adj) =  0.0107
## lmer.REML =  11518  Scale est. = 10.473    n = 1873
```

```
##                                                        stdcoef      stdse
## X(Intercept)                                       0.000000000 0.00000000
## XPDS_score                                         0.090167889 0.02529740
## Xhormone_scr_ert_mean                              0.006997497 0.02424821
## Xaccumbens_posvsneg_feedback_z                     0.037588859 0.05465681
## Xrace.ethnicity.5levelBlack                        0.017149749 0.05389191
## Xrace.ethnicity.5levelMixed                        0.132108077 0.05493854
## Xrace.ethnicity.5levelOther                        0.095615174 0.04128145
## Xrace.ethnicity.5levelWhite                        0.118997652 0.07166940
## Xdemo_race_hispanic1                               0.020239798 0.02669737
## Xinterview_age                                    -0.022931083 0.02349276
## Xhormone_scr_ert_mean:accumbens_posvsneg_feedback_z -0.046304621 0.05464837
```

Male participants

```
##
## Family: gaussian
```

```
## Link function: identity
##
## Formula:
## cbcl_scr_syn_internal_r ~ PDS_score + hormone_scr_ert_mean *
##     accumbens_posvsneg_feedback_z + race.ethnicity.5level + demo_race_hispanic +
##     interview_age
##
## Parametric coefficients:
##                                                Estimate Std. Error t value
## (Intercept)                                    0.658559   2.091251   0.315
## PDS_score                                      0.769057   0.246289   3.123
## hormone_scr_ert_mean                          -0.002729   0.008376  -0.326
## accumbens_posvsneg_feedback_z                  0.304347   0.375976   0.809
## race.ethnicity.5levelBlack                     1.124960   0.909945   1.236
## race.ethnicity.5levelMixed                     2.837777   0.892904   3.178
## race.ethnicity.5levelOther                     2.905780   1.025407   2.834
## race.ethnicity.5levelWhite                     2.089149   0.840017   2.487
## demo_race_hispanic1                            0.063400   0.345254   0.184
## interview_age                                  0.008688   0.016568   0.524
## hormone_scr_ert_mean:accumbens_posvsneg_feedback_z  0.001418   0.010581   0.134
##                                               Pr(>|t|)
## (Intercept)                                    0.75286
## PDS_score                                      0.00182 **
## hormone_scr_ert_mean                           0.74457
## accumbens_posvsneg_feedback_z                  0.41834
## race.ethnicity.5levelBlack                     0.21651
## race.ethnicity.5levelMixed                     0.00151 **
## race.ethnicity.5levelOther                     0.00465 **
## race.ethnicity.5levelWhite                     0.01297 *
## demo_race_hispanic1                            0.85432
## interview_age                                  0.60006
## hormone_scr_ert_mean:accumbens_posvsneg_feedback_z  0.89337
## ---
## Signif. codes:  0 '***' 0.001 '**' 0.01 '*' 0.05 '.' 0.1 ' ' 1
##
##
## R-sq.(adj) =  0.0114
## lmer.REML =  11419  Scale est. = 19.181    n = 1862
```

```
##                                                          stdcoef      stdse
## X(Intercept)                                         0.000000000 0.00000000
## XPDS_score                                           0.076885588 0.02462248
## Xhormone_scr_ert_mean                               -0.007995204 0.02453590
## Xaccumbens_posvsneg_feedback_z                       0.041184918 0.05087796
## Xrace.ethnicity.5levelBlack                          0.068884609 0.05571865
## Xrace.ethnicity.5levelMixed                          0.179539583 0.05649196
## Xrace.ethnicity.5levelOther                          0.115321880 0.04069538
## Xrace.ethnicity.5levelWhite                          0.184381166 0.07413704
## Xdemo_race_hispanic1                                 0.004856984 0.02644958
## Xinterview_age                                       0.012474363 0.02378765
## Xhormone_scr_ert_mean:accumbens_posvsneg_feedback_z  0.006843802 0.05105107
```

4.18 Model: CBCL internalizing factor ~ Testosterone x Caudate activity (Feedback stage) + PDS

Female participants

```
##
## Family: gaussian
## Link function: identity
##
## Formula:
## cbcl_scr_syn_internal_r ~ PDS_score + hormone_scr_ert_mean *
##     caudate_posvsneg_feedback_z + race.ethnicity.5level + demo_race_hispanic +
##     interview_age
##
## Parametric coefficients:
##                                              Estimate Std. Error t value
## (Intercept)                                 4.554e+00  2.125e+00   2.143
## PDS_score                                   7.006e-01  1.917e-01   3.656
## hormone_scr_ert_mean                        2.420e-03  8.112e-03   0.298
## caudate_posvsneg_feedback_z                -1.997e-01  3.265e-01  -0.612
## race.ethnicity.5levelBlack                  2.948e-01  8.991e-01   0.328
## race.ethnicity.5levelMixed                  2.101e+00  8.768e-01   2.396
## race.ethnicity.5levelOther                  2.103e+00  9.960e-01   2.112
## race.ethnicity.5levelWhite                  1.291e+00  8.229e-01   1.569
## demo_race_hispanic1                         3.428e-01  3.598e-01   0.953
## interview_age                              -1.950e-02  1.697e-02  -1.149
## hormone_scr_ert_mean:caudate_posvsneg_feedback_z -8.056e-06  8.468e-03  -0.001
##                                            Pr(>|t|)
## (Intercept)                                0.032269 *
## PDS_score                                  0.000264 ***
## hormone_scr_ert_mean                       0.765486
## caudate_posvsneg_feedback_z                0.540722
## race.ethnicity.5levelBlack                 0.743000
## race.ethnicity.5levelMixed                 0.016683 *
## race.ethnicity.5levelOther                 0.034831 *
## race.ethnicity.5levelWhite                 0.116767
## demo_race_hispanic1                        0.340801
## interview_age                              0.250598
## hormone_scr_ert_mean:caudate_posvsneg_feedback_z 0.999241
## ---
## Signif. codes:  0 '***' 0.001 '**' 0.01 '*' 0.05 '.' 0.1 ' ' 1
##
##
## R-sq.(adj) =  0.0122
## lmer.REML =  11483  Scale est. = 10.562    n = 1865
```

```
##                                                 stdcoef      stdse
## X(Intercept)                               0.000000e+00 0.00000000
## XPDS_score                                 9.263388e-02 0.02534069
## Xhormone_scr_ert_mean                      7.251566e-03 0.02430729
## Xcaudate_posvsneg_feedback_z              -3.193048e-02 0.05218801
## Xrace.ethnicity.5levelBlack                1.758754e-02 0.05363161
## Xrace.ethnicity.5levelMixed                1.319344e-01 0.05506927
## Xrace.ethnicity.5levelOther                8.696694e-02 0.04117999
```

```
## Xrace.ethnicity.5levelWhite                        1.122973e-01 0.07156246
## Xdemo_race_hispanic1                              2.548609e-02 0.02674784
## Xinterview_age                                   -2.709446e-02 0.02357562
## Xhormone_scr_ert_mean:caudate_posvsneg_feedback_z -4.981198e-05 0.05235506
```

Male participants

```
##
## Family: gaussian
## Link function: identity
##
## Formula:
## cbcl_scr_syn_internal_r ~ PDS_score + hormone_scr_ert_mean *
##     caudate_posvsneg_feedback_z + race.ethnicity.5level + demo_race_hispanic +
##     interview_age
##
## Parametric coefficients:
##                                                    Estimate Std. Error t value
## (Intercept)                                       1.3883336  2.1190745   0.655
## PDS_score                                         0.8436839  0.2489426   3.389
## hormone_scr_ert_mean                             -0.0004785  0.0083682  -0.057
## caudate_posvsneg_feedback_z                       0.0385882  0.3254904   0.119
## race.ethnicity.5levelBlack                        1.0010979  0.9272429   1.080
## race.ethnicity.5levelMixed                        2.7672791  0.9117289   3.035
## race.ethnicity.5levelOther                        2.7627825  1.0407302   2.655
## race.ethnicity.5levelWhite                        2.0558581  0.8586835   2.394
## demo_race_hispanic1                               0.1363458  0.3485672   0.391
## interview_age                                     0.0018276  0.0167146   0.109
## hormone_scr_ert_mean:caudate_posvsneg_feedback_z  0.0037076  0.0092531   0.401
##                                                  Pr(>|t|)
## (Intercept)                                      0.512446
## PDS_score                                        0.000716 ***
## hormone_scr_ert_mean                             0.954412
## caudate_posvsneg_feedback_z                      0.905642
## race.ethnicity.5levelBlack                       0.280439
## race.ethnicity.5levelMixed                       0.002437 **
## race.ethnicity.5levelOther                       0.008007 **
## race.ethnicity.5levelWhite                       0.016756 *
## demo_race_hispanic1                              0.695723
## interview_age                                    0.912943
## hormone_scr_ert_mean:caudate_posvsneg_feedback_z 0.688697
## ---
## Signif. codes:  0 '***' 0.001 '**' 0.01 '*' 0.05 '.' 0.1 ' ' 1
##
##
## R-sq.(adj) =  0.0104
## lmer.REML =  11469  Scale est. = 18.894    n = 1864
```

```
##                                                       stdcoef      stdse
## X(Intercept)                                      0.000000000 0.00000000
## XPDS_score                                        0.083449931 0.02462325
## Xhormone_scr_ert_mean                            -0.001390307 0.02431675
## Xcaudate_posvsneg_feedback_z                      0.006366573 0.05370187
```

```
## Xrace.ethnicity.5levelBlack                         0.061180869 0.05666731
## Xrace.ethnicity.5levelMixed                         0.173354353 0.05711465
## Xrace.ethnicity.5levelOther                         0.109174442 0.04112562
## Xrace.ethnicity.5levelWhite                         0.180030947 0.07519468
## Xdemo_race_hispanic1                                0.010363679 0.02649469
## Xinterview_age                                      0.002600489 0.02378302
## Xhormone_scr_ert_mean:caudate_posvsneg_feedback_z  0.021559629 0.05380679
```

4.19 Model: CBCL internalizing factor ~ Testosterone x Putamen activity (Feedback stage) + PDS

Female participants

```
##
## Family: gaussian
## Link function: identity
##
## Formula:
## cbcl_scr_syn_internal_r ~ PDS_score + hormone_scr_ert_mean *
##     putamen_posvsneg_feedback_z + race.ethnicity.5level + demo_race_hispanic +
##     interview_age
##
## Parametric coefficients:
##                                                   Estimate Std. Error t value
## (Intercept)                                        4.287337   2.120112   2.022
## PDS_score                                          0.674074   0.191044   3.528
## hormone_scr_ert_mean                               0.002856   0.008144   0.351
## putamen_posvsneg_feedback_z                       -0.057460   0.364195  -0.158
## race.ethnicity.5levelBlack                         0.353506   0.899655   0.393
## race.ethnicity.5levelMixed                         2.143254   0.876523   2.445
## race.ethnicity.5levelOther                         2.180848   0.997524   2.186
## race.ethnicity.5levelWhite                         1.325057   0.823209   1.610
## demo_race_hispanic1                                0.360481   0.359323   1.003
## interview_age                                     -0.017350   0.016925  -1.025
## hormone_scr_ert_mean:putamen_posvsneg_feedback_z -0.003165   0.009367  -0.338
##                                                   Pr(>|t|)
## (Intercept)                                       0.043297 *
## PDS_score                                         0.000428 ***
## hormone_scr_ert_mean                              0.725826
## putamen_posvsneg_feedback_z                       0.874653
## race.ethnicity.5levelBlack                        0.694413
## race.ethnicity.5levelMixed                        0.014571 *
## race.ethnicity.5levelOther                        0.028921 *
## race.ethnicity.5levelWhite                        0.107650
## demo_race_hispanic1                               0.315884
## interview_age                                     0.305453
## hormone_scr_ert_mean:putamen_posvsneg_feedback_z 0.735491
## ---
## Signif. codes:  0 '***' 0.001 '**' 0.01 '*' 0.05 '.' 0.1 ' ' 1
##
##
## R-sq.(adj) =  0.0116
## lmer.REML =  11483  Scale est. = 10.565    n = 1865
```

```
##                                                     stdcoef      stdse
## X(Intercept)                                     0.000000000 0.00000000
## XPDS_score                                       0.089424969 0.02534449
## Xhormone_scr_ert_mean                            0.008533937 0.02433147
## Xputamen_posvsneg_feedback_z                    -0.008689526 0.05507630
## Xrace.ethnicity.5levelBlack                      0.021092081 0.05367831
## Xrace.ethnicity.5levelMixed                      0.134877786 0.05516076
## Xrace.ethnicity.5levelOther                      0.089750821 0.04105219
## Xrace.ethnicity.5levelWhite                      0.115262984 0.07160866
## Xdemo_race_hispanic1                             0.026804630 0.02671849
## Xinterview_age                                  -0.024123053 0.02353253
## Xhormone_scr_ert_mean:putamen_posvsneg_feedback_z -0.018618912 0.05510494
```

Male participants

```
##
## Family: gaussian
## Link function: identity
##
## Formula:
## cbcl_scr_syn_internal_r ~ PDS_score + hormone_scr_ert_mean *
##     putamen_posvsneg_feedback_z + race.ethnicity.5level + demo_race_hispanic +
##     interview_age
##
## Parametric coefficients:
##                                                    Estimate Std. Error t value
## (Intercept)                                       0.9678945  2.1150864   0.458
## PDS_score                                         0.8206911  0.2491162   3.294
## hormone_scr_ert_mean                              0.0001559  0.0083903   0.019
## putamen_posvsneg_feedback_z                       0.3279395  0.3259022   1.006
## race.ethnicity.5levelBlack                        1.0351830  0.9199736   1.125
## race.ethnicity.5levelMixed                        2.8187987  0.9041222   3.118
## race.ethnicity.5levelOther                        2.8285173  1.0349923   2.733
## race.ethnicity.5levelWhite                        2.1215584  0.8509597   2.493
## demo_race_hispanic1                               0.0681918  0.3505186   0.195
## interview_age                                     0.0050743  0.0167195   0.303
## hormone_scr_ert_mean:putamen_posvsneg_feedback_z -0.0049790  0.0091813  -0.542
##                                                  Pr(>|t|)
## (Intercept)                                       0.64728
## PDS_score                                         0.00100 **
## hormone_scr_ert_mean                              0.98517
## putamen_posvsneg_feedback_z                       0.31443
## race.ethnicity.5levelBlack                        0.26064
## race.ethnicity.5levelMixed                        0.00185 **
## race.ethnicity.5levelOther                        0.00634 **
## race.ethnicity.5levelWhite                        0.01275 *
## demo_race_hispanic1                               0.84577
## interview_age                                     0.76155
## hormone_scr_ert_mean:putamen_posvsneg_feedback_z  0.58768
## ---
## Signif. codes:  0 '***' 0.001 '**' 0.01 '*' 0.05 '.' 0.1 ' ' 1
##
##
```

```
## R-sq.(adj) =  0.0102
## lmer.REML =  11517  Scale est. = 19.045    n = 1870
```

```
##                                                        stdcoef      stdse
## X(Intercept)                                      0.0000000000 0.00000000
## XPDS_score                                        0.0810367444 0.02459825
## Xhormone_scr_ert_mean                             0.0004519792 0.02431881
## Xputamen_posvsneg_feedback_z                      0.0536290875 0.05329591
## Xrace.ethnicity.5levelBlack                       0.0629716317 0.05596328
## Xrace.ethnicity.5levelMixed                       0.1760914006 0.05648085
## Xrace.ethnicity.5levelOther                       0.1118613343 0.04093156
## Xrace.ethnicity.5levelWhite                       0.1852841829 0.07431771
## Xdemo_race_hispanic1                              0.0051603085 0.02652497
## Xinterview_age                                    0.0072033788 0.02373469
## Xhormone_scr_ert_mean:putamen_posvsneg_feedback_z -0.0289168505 0.05332263
```

4.20 Model: CBCL internalizing factor ~ Testosterone x Lateral OFC activity (anticipation stage) + PDS

Female participants

```
##
## Family: gaussian
## Link function: identity
##
## Formula:
## cbcl_scr_syn_internal_r ~ PDS_score + hormone_scr_ert_mean *
##     lOFC_rvsn_ant_z + race.ethnicity.5level + demo_race_hispanic +
##     interview_age
##
## Parametric coefficients:
##                                       Estimate Std. Error t value Pr(>|t|)
## (Intercept)                           4.386035   2.139491   2.050 0.040501 *
## PDS_score                             0.658410   0.191838   3.432 0.000612 ***
## hormone_scr_ert_mean                  0.003086   0.008159   0.378 0.705274
## lOFC_rvsn_ant_z                       0.330589   0.491379   0.673 0.501172
## race.ethnicity.5levelBlack            0.227744   0.906920   0.251 0.801751
## race.ethnicity.5levelMixed            2.115228   0.887612   2.383 0.017270 *
## race.ethnicity.5levelOther            2.179843   1.007403   2.164 0.030605 *
## race.ethnicity.5levelWhite            1.276544   0.832855   1.533 0.125512
## demo_race_hispanic1                   0.358385   0.359093   0.998 0.318396
## interview_age                        -0.017460   0.017050  -1.024 0.305924
## hormone_scr_ert_mean:lOFC_rvsn_ant_z -0.007097   0.012725  -0.558 0.577086
## ---
## Signif. codes:  0 '***' 0.001 '**' 0.01 '*' 0.05 '.' 0.1 ' ' 1
##
##
## R-sq.(adj) =  0.0109
## lmer.REML =  11483  Scale est. = 10.83     n = 1864
```

```
##                                          stdcoef      stdse
## X(Intercept)                         0.000000000 0.00000000
## XPDS_score                           0.087218772 0.02541257
```

```
## Xhormone_scr_ert_mean                 0.009225083 0.02438763
## XlOFC_rvsn_ant_z                      0.034944355 0.05194040
## Xrace.ethnicity.5levelBlack           0.013630564 0.05427961
## Xrace.ethnicity.5levelMixed           0.132116587 0.05544004
## Xrace.ethnicity.5levelOther           0.090248343 0.04170778
## Xrace.ethnicity.5levelWhite           0.110969448 0.07239970
## Xdemo_race_hispanic1                  0.026757077 0.02680994
## Xinterview_age                       -0.024258921 0.02368814
## Xhormone_scr_ert_mean:lOFC_rvsn_ant_z -0.028954334 0.05191328
```

Male participants

```
##
## Family: gaussian
## Link function: identity
##
## Formula:
## cbcl_scr_syn_internal_r ~ PDS_score + hormone_scr_ert_mean *
##     lOFC_rvsn_ant_z + race.ethnicity.5level + demo_race_hispanic +
##     interview_age
##
## Parametric coefficients:
##                                       Estimate Std. Error t value Pr(>|t|)
## (Intercept)                           0.150908   2.087223   0.072  0.94237
## PDS_score                             0.735069   0.248610   2.957  0.00315 **
## hormone_scr_ert_mean                 -0.003581   0.008285  -0.432  0.66566
## lOFC_rvsn_ant_z                       0.318482   0.425928   0.748  0.45472
## race.ethnicity.5levelBlack            0.995449   0.908689   1.095  0.27345
## race.ethnicity.5levelMixed            2.714104   0.892622   3.041  0.00239 **
## race.ethnicity.5levelOther            2.660468   1.024339   2.597  0.00947 **
## race.ethnicity.5levelWhite            1.971262   0.838789   2.350  0.01887 *
## demo_race_hispanic1                  -0.018859   0.345314  -0.055  0.95645
## interview_age                         0.014731   0.016549   0.890  0.37349
## hormone_scr_ert_mean:lOFC_rvsn_ant_z -0.011844   0.012319  -0.962  0.33642
## ---
## Signif. codes:  0 '***' 0.001 '**' 0.01 '*' 0.05 '.' 0.1 ' ' 1
##
##
## R-sq.(adj) =  0.0088
## lmer.REML =  11420  Scale est. = 18.159    n = 1863
```

```
##                                           stdcoef      stdse
## X(Intercept)                          0.000000000 0.00000000
## XPDS_score                            0.073113645 0.02472805
## Xhormone_scr_ert_mean                -0.010454089 0.02418883
## XlOFC_rvsn_ant_z                      0.037501635 0.05015365
## Xrace.ethnicity.5levelBlack           0.061069392 0.05574683
## Xrace.ethnicity.5levelMixed           0.171722959 0.05647675
## Xrace.ethnicity.5levelOther           0.105783195 0.04072886
## Xrace.ethnicity.5levelWhite           0.174235445 0.07413867
## Xdemo_race_hispanic1                 -0.001449026 0.02653155
## Xinterview_age                        0.021210017 0.02382701
## Xhormone_scr_ert_mean:lOFC_rvsn_ant_z -0.048290370 0.05022375
```

4.21 Model: CBCL internalizing factor ~ Testosterone x Medial OFC activity (anticipation stage) + PDS

Female participants

```
##
## Family: gaussian
## Link function: identity
##
## Formula:
## cbcl_scr_syn_internal_r ~ PDS_score + hormone_scr_ert_mean *
##     mOFC_rvsn_ant_z + race.ethnicity.5level + demo_race_hispanic +
##     interview_age
##
## Parametric coefficients:
##                                     Estimate Std. Error t value Pr(>|t|)
## (Intercept)                         4.202280   2.132609   1.970 0.048931 *
## PDS_score                           0.676571   0.191614   3.531 0.000424 ***
## hormone_scr_ert_mean                0.002583   0.008127   0.318 0.750694
## mOFC_rvsn_ant_z                     0.063317   0.437193   0.145 0.884864
## race.ethnicity.5levelBlack          0.216029   0.906342   0.238 0.811634
## race.ethnicity.5levelMixed          2.115741   0.887454   2.384 0.017223 *
## race.ethnicity.5levelOther          2.218632   1.008524   2.200 0.027938 *
## race.ethnicity.5levelWhite          1.295692   0.832708   1.556 0.119879
## demo_race_hispanic1                 0.351711   0.358836   0.980 0.327142
## interview_age                      -0.016049   0.016989  -0.945 0.344951
## hormone_scr_ert_mean:mOFC_rvsn_ant_z  0.002991   0.011334   0.264 0.791898
## ---
## Signif. codes:  0 '***' 0.001 '**' 0.01 '*' 0.05 '.' 0.1 ' ' 1
##
##
## R-sq.(adj) =  0.0115
## lmer.REML =  11480  Scale est. = 10.572    n = 1864
```

```
##                                          stdcoef      stdse
## X(Intercept)                         0.000000000 0.00000000
## XPDS_score                           0.089377321 0.02531284
## Xhormone_scr_ert_mean                0.007704058 0.02424402
## XmOFC_rvsn_ant_z                     0.007732696 0.05339312
## Xrace.ethnicity.5levelBlack          0.012931400 0.05425325
## Xrace.ethnicity.5levelMixed          0.131909474 0.05532983
## Xrace.ethnicity.5levelOther          0.091244834 0.04147718
## Xrace.ethnicity.5levelWhite          0.112430130 0.07225597
## Xdemo_race_hispanic1                 0.026185470 0.02671589
## Xinterview_age                      -0.022263668 0.02356767
## Xhormone_scr_ert_mean:mOFC_rvsn_ant_z  0.014123809 0.05352261
```

Male participants

```
##
## Family: gaussian
## Link function: identity
##
```

```
## Formula:
## cbcl_scr_syn_internal_r ~ PDS_score + hormone_scr_ert_mean *
##     mOFC_rvsn_ant_z + race.ethnicity.5level + demo_race_hispanic +
##     interview_age
##
## Parametric coefficients:
##                                      Estimate Std. Error t value Pr(>|t|)
## (Intercept)                         0.3832360  2.0976904   0.183  0.85506
## PDS_score                           0.7451921  0.2484437   2.999  0.00274 **
## hormone_scr_ert_mean               -0.0048427  0.0082810  -0.585  0.55876
## mOFC_rvsn_ant_z                     0.2130617  0.3870717   0.550  0.58208
## race.ethnicity.5levelBlack          1.0194439  0.9127620   1.117  0.26419
## race.ethnicity.5levelMixed          2.6588881  0.8961882   2.967  0.00305 **
## race.ethnicity.5levelOther          2.6485499  1.0260213   2.581  0.00992 **
## race.ethnicity.5levelWhite          1.9814512  0.8424593   2.352  0.01878 *
## demo_race_hispanic1                 0.0445607  0.3461643   0.129  0.89759
## interview_age                       0.0129518  0.0166157   0.779  0.43579
## hormone_scr_ert_mean:mOFC_rvsn_ant_z -0.0001119  0.0109075  -0.010  0.99182
## ---
## Signif. codes:  0 '***' 0.001 '**' 0.01 '*' 0.05 '.' 0.1 ' ' 1
##
##
## R-sq.(adj) =  0.0096
## lmer.REML =  11397  Scale est. = 18.344    n = 1857
```

```
##                                          stdcoef      stdse
## X(Intercept)                          0.0000000000 0.00000000
## XPDS_score                            0.0741649416 0.02472626
## Xhormone_scr_ert_mean                -0.0141767364 0.02424212
## XmOFC_rvsn_ant_z                      0.0273278189 0.04964676
## Xrace.ethnicity.5levelBlack           0.0621167897 0.05561644
## Xrace.ethnicity.5levelMixed           0.1677608905 0.05654443
## Xrace.ethnicity.5levelOther           0.1056256681 0.04091831
## Xrace.ethnicity.5levelWhite           0.1744784954 0.07418353
## Xdemo_race_hispanic1                  0.0034169889 0.02654445
## Xinterview_age                        0.0185909553 0.02385014
## Xhormone_scr_ert_mean:mOFC_rvsn_ant_z -0.0005086038 0.04957859
```

4.22 Model: CBCL internalizing factor ~ Testosterone x Lateral OFC activity (feedback stage) + PDS

Female participants

```
##
## Family: gaussian
## Link function: identity
##
## Formula:
## cbcl_scr_syn_internal_r ~ PDS_score + hormone_scr_ert_mean *
##     lOFC_posvsneg_feedback_z + race.ethnicity.5level + demo_race_hispanic +
##     interview_age
##
## Parametric coefficients:
```

```
##                                              Estimate Std. Error t value
## (Intercept)                                  4.334290   2.117637   2.047
## PDS_score                                    0.673271   0.190513   3.534
## hormone_scr_ert_mean                         0.001130   0.008091   0.140
## lOFC_posvsneg_feedback_z                     0.550378   0.567460   0.970
## race.ethnicity.5levelBlack                   0.298496   0.894428   0.334
## race.ethnicity.5levelMixed                   2.147933   0.873735   2.458
## race.ethnicity.5levelOther                   2.515196   0.999082   2.518
## race.ethnicity.5levelWhite                   1.364823   0.819389   1.666
## demo_race_hispanic1                          0.238185   0.357406   0.666
## interview_age                               -0.017253   0.016901  -1.021
## hormone_scr_ert_mean:lOFC_posvsneg_feedback_z -0.019692   0.014952  -1.317
##                                             Pr(>|t|)
## (Intercept)                                 0.040823 *
## PDS_score                                   0.000419 ***
## hormone_scr_ert_mean                        0.888980
## lOFC_posvsneg_feedback_z                    0.332224
## race.ethnicity.5levelBlack                  0.738622
## race.ethnicity.5levelMixed                  0.014049 *
## race.ethnicity.5levelOther                  0.011903 *
## race.ethnicity.5levelWhite                  0.095950 .
## demo_race_hispanic1                         0.505222
## interview_age                               0.307481
## hormone_scr_ert_mean:lOFC_posvsneg_feedback_z 0.188010
## ---
## Signif. codes:  0 '***' 0.001 '**' 0.01 '*' 0.05 '.' 0.1 ' ' 1
##
##
## R-sq.(adj) =  0.0128
## lmer.REML =  11471  Scale est. = 10.543    n = 1865
```

```
##                                                   stdcoef      stdse
## X(Intercept)                                  0.000000000 0.00000000
## XPDS_score                                    0.089530985 0.02533420
## Xhormone_scr_ert_mean                         0.003392483 0.02429891
## XlOFC_posvsneg_feedback_z                     0.051315582 0.05290828
## Xrace.ethnicity.5levelBlack                   0.017930105 0.05372656
## Xrace.ethnicity.5levelMixed                   0.134618193 0.05475990
## Xrace.ethnicity.5levelOther                   0.102781970 0.04082687
## Xrace.ethnicity.5levelWhite                   0.118852302 0.07135454
## Xdemo_race_hispanic1                          0.017760349 0.02665016
## Xinterview_age                               -0.024044745 0.02355472
## Xhormone_scr_ert_mean:lOFC_posvsneg_feedback_z -0.069808143 0.05300648
```

Male participants

```
##
## Family: gaussian
## Link function: identity
##
## Formula:
## cbcl_scr_syn_internal_r ~ PDS_score + hormone_scr_ert_mean *
##     lOFC_posvsneg_feedback_z + race.ethnicity.5level + demo_race_hispanic +
```

```
##     interview_age
##
## Parametric coefficients:
##                                              Estimate Std. Error t value
## (Intercept)                                  0.714685   2.088512   0.342
## PDS_score                                    0.783974   0.246780   3.177
## hormone_scr_ert_mean                        -0.003902   0.008305  -0.470
## lOFC_posvsneg_feedback_z                     0.077362   0.470889   0.164
## race.ethnicity.5levelBlack                   1.077394   0.910723   1.183
## race.ethnicity.5levelMixed                   2.805075   0.893849   3.138
## race.ethnicity.5levelOther                   2.628287   1.029908   2.552
## race.ethnicity.5levelWhite                   2.036011   0.840929   2.421
## demo_race_hispanic1                          0.054593   0.346160   0.158
## interview_age                                0.009083   0.016537   0.549
## hormone_scr_ert_mean:lOFC_posvsneg_feedback_z  0.001431   0.013061   0.110
##                                             Pr(>|t|)
## (Intercept)                                  0.73224
## PDS_score                                    0.00151 **
## hormone_scr_ert_mean                         0.63848
## lOFC_posvsneg_feedback_z                     0.86952
## race.ethnicity.5levelBlack                   0.23696
## race.ethnicity.5levelMixed                   0.00173 **
## race.ethnicity.5levelOther                   0.01079 *
## race.ethnicity.5levelWhite                   0.01557 *
## demo_race_hispanic1                          0.87470
## interview_age                                0.58291
## hormone_scr_ert_mean:lOFC_posvsneg_feedback_z  0.91278
## ---
## Signif. codes:  0 '***' 0.001 '**' 0.01 '*' 0.05 '.' 0.1 ' ' 1
##
##
## R-sq.(adj) =  0.00925
## lmer.REML =  11479  Scale est. = 18.264    n = 1871
```

```
##                                                    stdcoef      stdse
## X(Intercept)                                   0.000000000 0.00000000
## XPDS_score                                     0.078132761 0.02459470
## Xhormone_scr_ert_mean                         -0.011391423 0.02424239
## XlOFC_posvsneg_feedback_z                      0.008171791 0.04974011
## Xrace.ethnicity.5levelBlack                    0.065904541 0.05570922
## Xrace.ethnicity.5levelMixed                    0.177587855 0.05658912
## Xrace.ethnicity.5levelOther                    0.103380522 0.04051021
## Xrace.ethnicity.5levelWhite                    0.179456733 0.07412056
## Xdemo_race_hispanic1                           0.004179204 0.02649933
## Xinterview_age                                 0.013060368 0.02377935
## Xhormone_scr_ert_mean:lOFC_posvsneg_feedback_z  0.005459776 0.04983746
```

4.23 Model: CBCL internalizing factor ~ Testosterone x Medial OFC activity (feedback stage) + PDS

Female participants

```
##
```

```
## Family: gaussian
## Link function: identity
##
## Formula:
## cbcl_scr_syn_internal_r ~ PDS_score + hormone_scr_ert_mean *
##     mOFC_posvsneg_feedback_z + race.ethnicity.5level + demo_race_hispanic +
##     interview_age
##
## Parametric coefficients:
##                                              Estimate Std. Error t value
## (Intercept)                                  4.369334   2.116941   2.064
## PDS_score                                    0.684020   0.190714   3.587
## hormone_scr_ert_mean                         0.002015   0.008095   0.249
## mOFC_posvsneg_feedback_z                     0.562287   0.484833   1.160
## race.ethnicity.5levelBlack                   0.271402   0.896065   0.303
## race.ethnicity.5levelMixed                   2.143308   0.874392   2.451
## race.ethnicity.5levelOther                   2.290652   0.993534   2.306
## race.ethnicity.5levelWhite                   1.335606   0.819977   1.629
## demo_race_hispanic1                          0.315052   0.357365   0.882
## interview_age                               -0.017840   0.016903  -1.055
## hormone_scr_ert_mean:mOFC_posvsneg_feedback_z -0.019533   0.012998  -1.503
##                                              Pr(>|t|)
## (Intercept)                                  0.039158 *
## PDS_score                                    0.000344 ***
## hormone_scr_ert_mean                         0.803458
## mOFC_posvsneg_feedback_z                     0.246298
## race.ethnicity.5levelBlack                   0.762013
## race.ethnicity.5levelMixed                   0.014330 *
## race.ethnicity.5levelOther                   0.021245 *
## race.ethnicity.5levelWhite                   0.103518
## demo_race_hispanic1                          0.378109
## interview_age                                0.291362
## hormone_scr_ert_mean:mOFC_posvsneg_feedback_z 0.133070
## ---
## Signif. codes:  0 '***' 0.001 '**' 0.01 '*' 0.05 '.' 0.1 ' ' 1
##
##
## R-sq.(adj) =  0.0133
## lmer.REML =  11481  Scale est. = 10.705    n = 1866
```

```
##                                                    stdcoef      stdse
## X(Intercept)                                   0.000000000 0.00000000
## XPDS_score                                     0.090846633 0.02532931
## Xhormone_scr_ert_mean                          0.006043042 0.02427799
## XmOFC_posvsneg_feedback_z                      0.063887955 0.05508752
## Xrace.ethnicity.5levelBlack                    0.016222048 0.05355889
## Xrace.ethnicity.5levelMixed                    0.134192510 0.05474567
## Xrace.ethnicity.5levelOther                    0.094893539 0.04115858
## Xrace.ethnicity.5levelWhite                    0.116250283 0.07137022
## Xdemo_race_hispanic1                           0.023515428 0.02667365
## Xinterview_age                                -0.024822076 0.02351827
## Xhormone_scr_ert_mean:mOFC_posvsneg_feedback_z -0.082792073 0.05509320
```

Male participants

```
##
## Family: gaussian
## Link function: identity
##
## Formula:
## cbcl_scr_syn_internal_r ~ PDS_score + hormone_scr_ert_mean *
##     mOFC_posvsneg_feedback_z + race.ethnicity.5level + demo_race_hispanic +
##     interview_age
##
## Parametric coefficients:
##                                              Estimate Std. Error t value
## (Intercept)                                  0.705120   2.085170   0.338
## PDS_score                                    0.790538   0.246908   3.202
## hormone_scr_ert_mean                        -0.003745   0.008306  -0.451
## mOFC_posvsneg_feedback_z                     0.539658   0.420064   1.285
## race.ethnicity.5levelBlack                   1.043013   0.910260   1.146
## race.ethnicity.5levelMixed                   2.833043   0.893757   3.170
## race.ethnicity.5levelOther                   2.681549   1.026752   2.612
## race.ethnicity.5levelWhite                   2.032990   0.840740   2.418
## demo_race_hispanic1                          0.026393   0.345459   0.076
## interview_age                                0.009054   0.016506   0.549
## hormone_scr_ert_mean:mOFC_posvsneg_feedback_z -0.006810   0.012039  -0.566
##                                             Pr(>|t|)
## (Intercept)                                  0.73528
## PDS_score                                    0.00139 **
## hormone_scr_ert_mean                         0.65210
## mOFC_posvsneg_feedback_z                     0.19906
## race.ethnicity.5levelBlack                   0.25201
## race.ethnicity.5levelMixed                   0.00155 **
## race.ethnicity.5levelOther                   0.00908 **
## race.ethnicity.5levelWhite                   0.01570 *
## demo_race_hispanic1                          0.93911
## interview_age                                0.58341
## hormone_scr_ert_mean:mOFC_posvsneg_feedback_z  0.57169
## ---
## Signif. codes:  0 '***' 0.001 '**' 0.01 '*' 0.05 '.' 0.1 ' ' 1
##
##
## R-sq.(adj) =  0.0111
## lmer.REML =  11466  Scale est. = 18.396    n = 1869
```

```
##                                                    stdcoef      stdse
## X(Intercept)                                   0.000000000 0.00000000
## XPDS_score                                     0.078701912 0.02458087
## Xhormone_scr_ert_mean                         -0.010959530 0.02430454
## XmOFC_posvsneg_feedback_z                      0.066266778 0.05158130
## Xrace.ethnicity.5levelBlack                    0.063912773 0.05577805
## Xrace.ethnicity.5levelMixed                    0.179312006 0.05656865
## Xrace.ethnicity.5levelOther                    0.106057369 0.04060885
## Xrace.ethnicity.5levelWhite                    0.179287802 0.07414423
## Xdemo_race_hispanic1                           0.002019782 0.02643724
## Xinterview_age                                 0.013009696 0.02371808
```

```
## Xhormone_scr_ert_mean:mOFC_posvsneg_feedback_z -0.029213472 0.05164401
```

4.24 Model: CBCL internalizing factor ~ Testosterone x BIS-BAS RR + PDS

Female participants

```
##
## Family: gaussian
## Link function: identity
##
## Formula:
## cbcl_scr_syn_internal_r ~ PDS_score + hormone_scr_ert_mean *
##     bisbas_ss_basm_rr + race.ethnicity.5level + demo_race_hispanic +
##     interview_age
##
## Parametric coefficients:
##                                     Estimate Std. Error t value Pr(>|t|)
## (Intercept)                         3.020293   2.129138   1.419 0.156158
## PDS_score                           0.626624   0.169756   3.691 0.000228
## hormone_scr_ert_mean               -0.009565   0.025287  -0.378 0.705274
## bisbas_ss_basm_rr                  -0.084205   0.110618  -0.761 0.446597
## race.ethnicity.5levelBlack         -0.041861   0.799020  -0.052 0.958222
## race.ethnicity.5levelMixed          1.640258   0.791942   2.071 0.038447
## race.ethnicity.5levelOther          2.486882   0.909634   2.734 0.006304
## race.ethnicity.5levelWhite          1.312543   0.742548   1.768 0.077250
## demo_race_hispanic1                 0.027915   0.326365   0.086 0.931844
## interview_age                       0.003614   0.015214   0.238 0.812240
## hormone_scr_ert_mean:bisbas_ss_basm_rr  0.001030   0.002812   0.366 0.714173
##
## (Intercept)
## PDS_score                          ***
## hormone_scr_ert_mean
## bisbas_ss_basm_rr
## race.ethnicity.5levelBlack
## race.ethnicity.5levelMixed         *
## race.ethnicity.5levelOther         **
## race.ethnicity.5levelWhite         .
## demo_race_hispanic1
## interview_age
## hormone_scr_ert_mean:bisbas_ss_basm_rr
## ---
## Signif. codes:  0 '***' 0.001 '**' 0.01 '*' 0.05 '.' 0.1 ' ' 1
##
##
## R-sq.(adj) =  0.011
## lmer.REML =  15183  Scale est. = 12.902    n = 2443
```

```
##                                           stdcoef      stdse
## X(Intercept)                          0.000000000 0.00000000
## XPDS_score                            0.082604510 0.02237807
## Xhormone_scr_ert_mean                -0.028244945 0.07467170
## Xbisbas_ss_basm_rr                   -0.035672492 0.04686200
## Xrace.ethnicity.5levelBlack          -0.002670114 0.05096549
```

```
## Xrace.ethnicity.5levelMixed             0.100690667 0.04861502
## Xrace.ethnicity.5levelOther             0.096891536 0.03544029
## Xrace.ethnicity.5levelWhite             0.113429746 0.06417085
## Xdemo_race_hispanic1                    0.002007520 0.02347058
## Xinterview_age                          0.004948478 0.02083018
## Xhormone_scr_ert_mean:bisbas_ss_basm_rr 0.031154003 0.08505036
```

Male participants

```
##
## Family: gaussian
## Link function: identity
##
## Formula:
## cbcl_scr_syn_internal_r ~ PDS_score + hormone_scr_ert_mean *
##     bisbas_ss_basm_rr + race.ethnicity.5level + demo_race_hispanic +
##     interview_age
##
## Parametric coefficients:
##                                       Estimate Std. Error t value Pr(>|t|)
## (Intercept)                          2.9592426  2.1028478   1.407   0.1595
## PDS_score                            0.9679803  0.2133487   4.537 5.96e-06
## hormone_scr_ert_mean                -0.0087963  0.0275527  -0.319   0.7496
## bisbas_ss_basm_rr                   -0.0425337  0.1025861  -0.415   0.6785
## race.ethnicity.5levelBlack           1.2248010  0.7870707   1.556   0.1198
## race.ethnicity.5levelMixed           1.9529549  0.7843751   2.490   0.0128
## race.ethnicity.5levelOther           1.6129400  0.9007965   1.791   0.0735
## race.ethnicity.5levelWhite           1.4416035  0.7352031   1.961   0.0500
## demo_race_hispanic1                  0.3094286  0.3135437   0.987   0.3238
## interview_age                       -0.0027965  0.0148992  -0.188   0.8511
## hormone_scr_ert_mean:bisbas_ss_basm_rr  0.0009086  0.0029456   0.308   0.7578
##
## (Intercept)
## PDS_score                           ***
## hormone_scr_ert_mean
## bisbas_ss_basm_rr
## race.ethnicity.5levelBlack
## race.ethnicity.5levelMixed          *
## race.ethnicity.5levelOther          .
## race.ethnicity.5levelWhite          .
## demo_race_hispanic1
## interview_age
## hormone_scr_ert_mean:bisbas_ss_basm_rr
## ---
## Signif. codes:  0 '***' 0.001 '**' 0.01 '*' 0.05 '.' 0.1 ' ' 1
##
##
## R-sq.(adj) =  0.00677
## lmer.REML =  16493  Scale est. = 16.687    n = 2635
```

```
##                                             stdcoef      stdse
## X(Intercept)                            0.000000000 0.00000000
## XPDS_score                              0.094090592 0.02073813
```

```
## Xhormone_scr_ert_mean                  -0.024455762 0.07660252
## Xbisbas_ss_basm_rr                     -0.017511467 0.04223552
## Xrace.ethnicity.5levelBlack             0.077047126 0.04951134
## Xrace.ethnicity.5levelMixed             0.115244212 0.04628611
## Xrace.ethnicity.5levelOther             0.061763665 0.03449384
## Xrace.ethnicity.5levelWhite             0.122320168 0.06238204
## Xdemo_race_hispanic1                    0.022084175 0.02237788
## Xinterview_age                         -0.003769998 0.02008560
## Xhormone_scr_ert_mean:bisbas_ss_basm_rr  0.026593228 0.08621131
```

4.25 Model: CBCL internalizing factor ~ Testosterone x MID Reaction Time + PDS (large reward vs. neutral)

Female participants

```
##
## Family: gaussian
## Link function: identity
##
## Formula:
## cbcl_scr_syn_internal_r ~ PDS_score + hormone_scr_ert_mean *
##     rt_diff_large_neutral_z + race.ethnicity.5level + demo_race_hispanic +
##     interview_age
##
## Parametric coefficients:
##                                           Estimate Std. Error t value
## (Intercept)                               4.907584   2.030917   2.416
## PDS_score                                 0.640866   0.184675   3.470
## hormone_scr_ert_mean                      0.002759   0.007808   0.353
## rt_diff_large_neutral_z                  -0.234714   0.298041  -0.788
## race.ethnicity.5levelBlack                0.234371   0.848919   0.276
## race.ethnicity.5levelMixed                2.018688   0.835051   2.417
## race.ethnicity.5levelOther                2.518939   0.951958   2.646
## race.ethnicity.5levelWhite                1.333646   0.780222   1.709
## demo_race_hispanic1                       0.310120   0.350440   0.885
## interview_age                            -0.021805   0.016262  -1.341
## hormone_scr_ert_mean:rt_diff_large_neutral_z  0.010525   0.007542   1.395
##                                          Pr(>|t|)
## (Intercept)                              0.015762 *
## PDS_score                                0.000531 ***
## hormone_scr_ert_mean                     0.723886
## rt_diff_large_neutral_z                  0.431069
## race.ethnicity.5levelBlack               0.782514
## race.ethnicity.5levelMixed               0.015719 *
## race.ethnicity.5levelOther               0.008207 **
## race.ethnicity.5levelWhite               0.087547 .
## demo_race_hispanic1                      0.376293
## interview_age                            0.180119
## hormone_scr_ert_mean:rt_diff_large_neutral_z 0.163025
## ---
## Signif. codes:  0 '***' 0.001 '**' 0.01 '*' 0.05 '.' 0.1 ' ' 1
##
##
```

```
## R-sq.(adj) =  0.0141
## lmer.REML =  12398  Scale est. = 11.344    n = 2014
```

```
##                                                  stdcoef      stdse
## X(Intercept)                                 0.000000000 0.00000000
## XPDS_score                                   0.084660536 0.02439626
## Xhormone_scr_ert_mean                        0.008240673 0.02332361
## Xrt_diff_large_neutral_z                    -0.040099945 0.05091906
## Xrace.ethnicity.5levelBlack                  0.014379534 0.05208437
## Xrace.ethnicity.5levelMixed                  0.125306101 0.05183418
## Xrace.ethnicity.5levelOther                  0.102723097 0.03882115
## Xrace.ethnicity.5levelWhite                  0.116282984 0.06802895
## Xdemo_race_hispanic1                         0.022839216 0.02580865
## Xinterview_age                              -0.030444450 0.02270520
## Xhormone_scr_ert_mean:rt_diff_large_neutral_z  0.070724384 0.05068090
```

Male participants

```
##
## Family: gaussian
## Link function: identity
##
## Formula:
## cbcl_scr_syn_internal_r ~ PDS_score + hormone_scr_ert_mean *
##     rt_diff_large_neutral_z + race.ethnicity.5level + demo_race_hispanic +
##     interview_age
##
## Parametric coefficients:
##                                              Estimate Std. Error t value
## (Intercept)                                 1.4502677  2.0225524   0.717
## PDS_score                                   0.7057552  0.2370283   2.978
## hormone_scr_ert_mean                       -0.0002715  0.0079566  -0.034
## rt_diff_large_neutral_z                     0.4957660  0.2919671   1.698
## race.ethnicity.5levelBlack                  0.6326718  0.8872742   0.713
## race.ethnicity.5levelMixed                  2.1038840  0.8757925   2.402
## race.ethnicity.5levelOther                  1.7323746  1.0030273   1.727
## race.ethnicity.5levelWhite                  1.3926335  0.8266765   1.685
## demo_race_hispanic1                         0.1495244  0.3362208   0.445
## interview_age                               0.0083807  0.0159449   0.526
## hormone_scr_ert_mean:rt_diff_large_neutral_z -0.0100928  0.0080219  -1.258
##                                             Pr(>|t|)
## (Intercept)                                  0.47342
## PDS_score                                    0.00294 **
## hormone_scr_ert_mean                         0.97278
## rt_diff_large_neutral_z                      0.08965 .
## race.ethnicity.5levelBlack                   0.47589
## race.ethnicity.5levelMixed                   0.01638 *
## race.ethnicity.5levelOther                   0.08429 .
## race.ethnicity.5levelWhite                   0.09221 .
## demo_race_hispanic1                          0.65657
## interview_age                                0.59922
## hormone_scr_ert_mean:rt_diff_large_neutral_z  0.20848
## ---
```

```
## Signif. codes:  0 '***' 0.001 '**' 0.01 '*' 0.05 '.' 0.1 ' ' 1
##
##
## R-sq.(adj) =  0.00659
## lmer.REML =  12919  Scale est. = 18.48     n = 2091
```

```
##                                                   stdcoef      stdse
## X(Intercept)                                 0.0000000000 0.00000000
## XPDS_score                                   0.0691194630 0.02321381
## Xhormone_scr_ert_mean                       -0.0007818428 0.02291272
## Xrt_diff_large_neutral_z                     0.0812335089 0.04784013
## Xrace.ethnicity.5levelBlack                  0.0392929450 0.05510537
## Xrace.ethnicity.5levelMixed                  0.1321383124 0.05500576
## Xrace.ethnicity.5levelOther                  0.0679076958 0.03931787
## Xrace.ethnicity.5levelWhite                  0.1218099598 0.07230720
## Xdemo_race_hispanic1                         0.0111392536 0.02504774
## Xinterview_age                               0.0118296878 0.02250680
## Xhormone_scr_ert_mean:rt_diff_large_neutral_z -0.0601402975 0.04780078
```

4.26 Model: CBCL internalizing factor ~ Testosterone x MID Reaction Time + PDS (large vs. small reward)

Female participants

```
##
## Family: gaussian
## Link function: identity
##
## Formula:
## cbcl_scr_syn_internal_r ~ PDS_score + hormone_scr_ert_mean *
##     rt_diff_large_small_z + race.ethnicity.5level + demo_race_hispanic +
##     interview_age
##
## Parametric coefficients:
##                                             Estimate Std. Error t value
## (Intercept)                                 4.830633   2.029793   2.380
## PDS_score                                   0.653073   0.184699   3.536
## hormone_scr_ert_mean                        0.001939   0.007810   0.248
## rt_diff_large_small_z                      -0.398245   0.292018  -1.364
## race.ethnicity.5levelBlack                  0.219698   0.848951   0.259
## race.ethnicity.5levelMixed                  2.006919   0.835252   2.403
## race.ethnicity.5levelOther                  2.487240   0.952491   2.611
## race.ethnicity.5levelWhite                  1.329151   0.780631   1.703
## demo_race_hispanic1                         0.281757   0.350482   0.804
## interview_age                              -0.020947   0.016244  -1.289
## hormone_scr_ert_mean:rt_diff_large_small_z  0.007626   0.007556   1.009
##                                            Pr(>|t|)
## (Intercept)                                0.017412 *
## PDS_score                                  0.000416 ***
## hormone_scr_ert_mean                       0.803898
## rt_diff_large_small_z                      0.172793
## race.ethnicity.5levelBlack                 0.795825
## race.ethnicity.5levelMixed                 0.016362 *
```

```
## race.ethnicity.5levelOther                0.009087 **
## race.ethnicity.5levelWhite                0.088786 .
## demo_race_hispanic1                       0.421543
## interview_age                             0.197374
## hormone_scr_ert_mean:rt_diff_large_small_z 0.312973
## ---
## Signif. codes:  0 '***' 0.001 '**' 0.01 '*' 0.05 '.' 0.1 ' ' 1
##
##
## R-sq.(adj) =  0.0132
## lmer.REML =  12399  Scale est. = 11.209    n = 2014
```

```
##                                                  stdcoef      stdse
## X(Intercept)                                 0.000000000 0.00000000
## XPDS_score                                   0.086273148 0.02439933
## Xhormone_scr_ert_mean                        0.005793587 0.02332941
## Xrt_diff_large_small_z                      -0.068777079 0.05043159
## Xrace.ethnicity.5levelBlack                  0.013479336 0.05208638
## Xrace.ethnicity.5levelMixed                  0.124575534 0.05184659
## Xrace.ethnicity.5levelOther                  0.101430408 0.03884285
## Xrace.ethnicity.5levelWhite                  0.115891113 0.06806462
## Xdemo_race_hispanic1                         0.020750382 0.02581176
## Xinterview_age                              -0.029245957 0.02268009
## Xhormone_scr_ert_mean:rt_diff_large_small_z  0.050925567 0.05045846
```

Male participants

```
##
## Family: gaussian
## Link function: identity
##
## Formula:
## cbcl_scr_syn_internal_r ~ PDS_score + hormone_scr_ert_mean *
##     rt_diff_large_small_z + race.ethnicity.5level + demo_race_hispanic +
##     interview_age
##
## Parametric coefficients:
##                                             Estimate Std. Error t value
## (Intercept)                                1.5105404  2.0253677   0.746
## PDS_score                                  0.6937632  0.2369733   2.928
## hormone_scr_ert_mean                      -0.0003462  0.0079643  -0.043
## rt_diff_large_small_z                     -0.0119908  0.2898251  -0.041
## race.ethnicity.5levelBlack                 0.6102166  0.8879027   0.687
## race.ethnicity.5levelMixed                 2.0562980  0.8760914   2.347
## race.ethnicity.5levelOther                 1.6642936  1.0033058   1.659
## race.ethnicity.5levelWhite                 1.3562003  0.8270570   1.640
## demo_race_hispanic1                        0.1405787  0.3363157   0.418
## interview_age                              0.0083766  0.0159693   0.525
## hormone_scr_ert_mean:rt_diff_large_small_z -0.0016357  0.0082670  -0.198
##                                           Pr(>|t|)
## (Intercept)                                0.45587
## PDS_score                                  0.00345 **
## hormone_scr_ert_mean                       0.96533
```

```
## rt_diff_large_small_z                      0.96700
## race.ethnicity.5levelBlack                 0.49200
## race.ethnicity.5levelMixed                 0.01901 *
## race.ethnicity.5levelOther                 0.09730 .
## race.ethnicity.5levelWhite                 0.10120
## demo_race_hispanic1                        0.67599
## interview_age                              0.59996
## hormone_scr_ert_mean:rt_diff_large_small_z  0.84317
## ---
## Signif. codes:  0 '***' 0.001 '**' 0.01 '*' 0.05 '.' 0.1 ' ' 1
##
##
## R-sq.(adj) =  0.00531
## lmer.REML =  12922  Scale est. = 18.52     n = 2091
```

```
##                                                    stdcoef      stdse
## X(Intercept)                                0.0000000000 0.00000000
## XPDS_score                                  0.0679450047 0.02320843
## Xhormone_scr_ert_mean                      -0.0009970233 0.02293495
## Xrt_diff_large_small_z                     -0.0019734573 0.04769957
## Xrace.ethnicity.5levelBlack                 0.0378983317 0.05514441
## Xrace.ethnicity.5levelMixed                 0.1291495884 0.05502454
## Xrace.ethnicity.5levelOther                 0.0652389737 0.03932878
## Xrace.ethnicity.5levelWhite                 0.1186232462 0.07234048
## Xdemo_race_hispanic1                        0.0104728182 0.02505481
## Xinterview_age                              0.0118238679 0.02254134
## Xhormone_scr_ert_mean:rt_diff_large_small_z -0.0094174136 0.04759658
```

5— Correlation Matrix —

Female participants

x1	x2	N	corr	p
bmi	interview_age	2675	0.0777979236	0.00005626170294
PDS_score	interview_age	2701	0.2397245643	0.00000000000000
PDS_score	bmi	2675	0.2883194569	0.00000000000000
cbcl_scr_syn_internal_r	interview_age	2701	0.0011506908	0.95233479027429
cbcl_scr_syn_internal_r	bmi	2675	0.0643351043	0.00087054703099
cbcl_scr_syn_internal_r	PDS_score	2701	0.0576397227	0.00272912600408
hormone_scr_ert_mean_z	interview_age	2514	0.2111922127	0.00000000000000
hormone_scr_ert_mean_z	bmi	2488	0.2011164615	0.00000000000000
hormone_scr_ert_mean_z	PDS_score	2514	0.3194091104	0.00000000000000
hormone_scr_ert_mean_z	cbcl_scr_syn_internal_r	2514	0.0149428266	0.45391772219595
bisbas_ss_basm_rr_z	interview_age	2690	-0.0324228218	0.09270914672107
bisbas_ss_basm_rr_z	bmi	2664	0.0518639980	0.00741845859611
bisbas_ss_basm_rr_z	PDS_score	2690	0.0567567189	0.00323263005375
bisbas_ss_basm_rr_z	cbcl_scr_syn_internal_r	2690	-0.0234766912	0.22351849161127
bisbas_ss_basm_rr_z	hormone_scr_ert_mean_z	2504	-0.0149523266	0.45453109921919
rt_diff_large_neutral_z	interview_age	2229	0.0444337653	0.03593351564518
rt_diff_large_neutral_z	bmi	2206	-0.0079826125	0.70786767361752
rt_diff_large_neutral_z	PDS_score	2229	-0.0029053436	0.89095865956824
rt_diff_large_neutral_z	cbcl_scr_syn_internal_r	2229	0.0166852282	0.43107074180790
rt_diff_large_neutral_z	hormone_scr_ert_mean_z	2088	-0.0216332136	0.32313034241508
rt_diff_large_neutral_z	bisbas_ss_basm_rr_z	2220	-0.0006405387	0.97593690877362
rt_diff_large_small_z	interview_age	2229	0.0219796326	0.29961846451327
rt_diff_large_small_z	bmi	2206	-0.0213846091	0.31540780939492
rt_diff_large_small_z	PDS_score	2229	-0.0170095374	0.42216737250534
rt_diff_large_small_z	cbcl_scr_syn_internal_r	2229	-0.0216931306	0.30596364272833
rt_diff_large_small_z	hormone_scr_ert_mean_z	2087	-0.0043284021	0.84334249311512
rt_diff_large_small_z	bisbas_ss_basm_rr_z	2220	-0.0231864210	0.27483168284004
rt_diff_large_small_z	rt_diff_large_neutral_z	2201	0.4179924701	0.00000000000000
accumbens_rvsn_ant_z	interview_age	2237	0.0114048219	0.58979774095617
accumbens_rvsn_ant_z	bmi	2214	-0.0481381380	0.02350742889809
accumbens_rvsn_ant_z	PDS_score	2237	-0.0022709471	0.91451215925587
accumbens_rvsn_ant_z	cbcl_scr_syn_internal_r	2237	-0.0011046109	0.95835696200010
accumbens_rvsn_ant_z	hormone_scr_ert_mean_z	2090	-0.0393773431	0.07188968575698
accumbens_rvsn_ant_z	bisbas_ss_basm_rr_z	2229	0.0080008151	0.70577908106748
accumbens_rvsn_ant_z	rt_diff_large_neutral_z	2084	0.0168178792	0.44287695646753
accumbens_rvsn_ant_z	rt_diff_large_small_z	2084	0.0278108340	0.20441541659964
caudate_rvsn_ant_z	interview_age	2236	0.0219258951	0.30004325750421
caudate_rvsn_ant_z	bmi	2213	-0.0383512800	0.07126491835157
caudate_rvsn_ant_z	PDS_score	2236	-0.0398572505	0.05951099252948
caudate_rvsn_ant_z	cbcl_scr_syn_internal_r	2236	-0.0001264905	0.99523032935205
caudate_rvsn_ant_z	hormone_scr_ert_mean_z	2088	-0.0281662362	0.19825798097841
caudate_rvsn_ant_z	bisbas_ss_basm_rr_z	2228	-0.0060577944	0.77504638139374
caudate_rvsn_ant_z	rt_diff_large_neutral_z	2080	0.0235573317	0.28287521125039
caudate_rvsn_ant_z	rt_diff_large_small_z	2079	0.0303883236	0.16602979724595
caudate_rvsn_ant_z	accumbens_rvsn_ant_z	2220	0.5792092513	0.00000000000000
putamen_rvsn_ant_z	interview_age	2232	0.0244479138	0.24827723868069
putamen_rvsn_ant_z	bmi	2209	-0.0416045359	0.05056502376294
putamen_rvsn_ant_z	PDS_score	2232	-0.0601145918	0.00449673509563
putamen_rvsn_ant_z	cbcl_scr_syn_internal_r	2232	-0.0111745491	0.59774112699753
putamen_rvsn_ant_z	hormone_scr_ert_mean_z	2085	-0.0240042818	0.27326293836512
putamen_rvsn_ant_z	bisbas_ss_basm_rr_z	2224	-0.0131102287	0.53660946224109
putamen_rvsn_ant_z	rt_diff_large_neutral_z	2077	0.0499082533	0.02293157373042
putamen_rvsn_ant_z	rt_diff_large_small_z	2076	0.0429202276	0.05054723967104

Male participants

x1	x2	N	corr	p
bmi	interview_age	2900	0.0920315763	0.0000006889469
PDS_score	interview_age	2924	0.1709640628	0.0000000000000
PDS_score	bmi	2900	0.1999796195	0.0000000000000
cbcl_scr_syn_internal_r	interview_age	2924	0.0048032993	0.7951536979285
cbcl_scr_syn_internal_r	bmi	2900	0.0671358414	0.0002970040156
cbcl_scr_syn_internal_r	PDS_score	2924	0.0736940209	0.0000664496826
hormone_scr_ert_mean_z	interview_age	2719	0.1651198590	0.0000000000000
hormone_scr_ert_mean_z	bmi	2696	0.1952656071	0.0000000000000
hormone_scr_ert_mean_z	PDS_score	2719	0.1818699124	0.0000000000000
hormone_scr_ert_mean_z	cbcl_scr_syn_internal_r	2719	0.0031107954	0.8711993730656
bisbas_ss_basm_rr_z	interview_age	2907	-0.0107959520	0.5606692971792
bisbas_ss_basm_rr_z	bmi	2883	0.0733349112	0.0000810704533
bisbas_ss_basm_rr_z	PDS_score	2907	0.0547830388	0.0031300989251
bisbas_ss_basm_rr_z	cbcl_scr_syn_internal_r	2907	0.0062881600	0.7346897684193
bisbas_ss_basm_rr_z	hormone_scr_ert_mean_z	2702	0.0394470083	0.0403317612151
rt_diff_large_neutral_z	interview_age	2317	-0.0125248599	0.5467846238585
rt_diff_large_neutral_z	bmi	2303	-0.0043610748	0.8343127747883
rt_diff_large_neutral_z	PDS_score	2317	-0.0435115439	0.0362332963381
rt_diff_large_neutral_z	cbcl_scr_syn_internal_r	2317	0.0030708133	0.8825520064098
rt_diff_large_neutral_z	hormone_scr_ert_mean_z	2153	-0.0151380839	0.4826505237757
rt_diff_large_neutral_z	bisbas_ss_basm_rr_z	2308	-0.0149974799	0.4714302803412
rt_diff_large_small_z	interview_age	2327	-0.0051579972	0.8036048138750
rt_diff_large_small_z	bmi	2313	0.0073378533	0.7242997169935
rt_diff_large_small_z	PDS_score	2327	-0.0174964117	0.3988816266765
rt_diff_large_small_z	cbcl_scr_syn_internal_r	2327	-0.0085058265	0.6817330193853
rt_diff_large_small_z	hormone_scr_ert_mean_z	2165	-0.0255918733	0.2339340575978
rt_diff_large_small_z	bisbas_ss_basm_rr_z	2318	-0.0046769321	0.8219376295062
rt_diff_large_small_z	rt_diff_large_neutral_z	2297	0.3765420553	0.0000000000000
accumbens_rvsn_ant_z	interview_age	2334	-0.0222598818	0.2823903366529
accumbens_rvsn_ant_z	bmi	2319	-0.0241546628	0.2449373017172
accumbens_rvsn_ant_z	PDS_score	2334	-0.0050367610	0.8078468897401
accumbens_rvsn_ant_z	cbcl_scr_syn_internal_r	2334	-0.0277065434	0.1808689939010
accumbens_rvsn_ant_z	hormone_scr_ert_mean_z	2163	-0.0085922236	0.6896102373768
accumbens_rvsn_ant_z	bisbas_ss_basm_rr_z	2322	-0.0310751306	0.1343993142486
accumbens_rvsn_ant_z	rt_diff_large_neutral_z	2136	-0.0271739486	0.2093365727565
accumbens_rvsn_ant_z	rt_diff_large_small_z	2145	-0.0148592024	0.4915610579548
caudate_rvsn_ant_z	interview_age	2335	0.0125379417	0.5448097811537
caudate_rvsn_ant_z	bmi	2321	-0.0235785001	0.2561735798566
caudate_rvsn_ant_z	PDS_score	2335	-0.0120627715	0.5601596680241
caudate_rvsn_ant_z	cbcl_scr_syn_internal_r	2335	-0.0094961720	0.6464947816140
caudate_rvsn_ant_z	hormone_scr_ert_mean_z	2163	-0.0009235512	0.9657591228348
caudate_rvsn_ant_z	bisbas_ss_basm_rr_z	2323	-0.0136291044	0.5114601245295
caudate_rvsn_ant_z	rt_diff_large_neutral_z	2135	0.0035885457	0.8683804663115
caudate_rvsn_ant_z	rt_diff_large_small_z	2143	0.0027205694	0.8998357470434
caudate_rvsn_ant_z	accumbens_rvsn_ant_z	2306	0.5963711143	0.0000000000000
putamen_rvsn_ant_z	interview_age	2336	0.0303725159	0.1422338747573
putamen_rvsn_ant_z	bmi	2321	-0.0378703566	0.0681305325944
putamen_rvsn_ant_z	PDS_score	2336	0.0084909641	0.6816778890089
putamen_rvsn_ant_z	cbcl_scr_syn_internal_r	2336	-0.0257950259	0.2126652870664
putamen_rvsn_ant_z	hormone_scr_ert_mean_z	2163	0.0172838342	0.4217244997837
putamen_rvsn_ant_z	bisbas_ss_basm_rr_z	2324	-0.0089881147	0.6649612903899
putamen_rvsn_ant_z	rt_diff_large_neutral_z	2134	0.0163507533	0.4502880759024
putamen_rvsn_ant_z	rt_diff_large_small_z	2143	-0.0017338906	0.9360627280707
putamen_rvsn_ant_z	accumbens_rvsn_ant_z	2309	0.5469812194	0.0000000000000
putamen_rvsn_ant_z	caudate_rvsn_ant_z	2318	0.7826410426	0.0000000000000
[illegible]	[illegible]	[illegible]	[illegible]	[illegible]

6— Compare Outliers to Non-Outliers on Demographic Variables —

Female participants

```
##        interview_age                  bmi race.ethnicity.5level
##         4.567958e-03         2.428145e-01          6.700028e-05
##     household.income            high.educ    demo_race_hispanic
##                  NaN         6.700028e-05          1.002051e-01
```

```
##
## --------Summary descriptives table by 'is_outlier_any'---------
##
## ___________________________________________________________
##                         not outlier    outlier   p.overall
##                           N=2492       N=209
## -----------------------------------------------------------
## interview_age            119 (7.52)  117 (7.26)    0.003
## bmi                     18.9 (4.05)  19.2 (4.56)   0.243
## race.ethnicity.5level:                            <0.001
##     Asian                59 (2.40%)   2 (0.98%)
##     Black               359 (14.6%)  47 (23.0%)
##     Mixed               309 (12.6%)  36 (17.6%)
##     Other               113 (4.59%)  17 (8.33%)
##     White               1621 (65.9%) 102 (50.0%)
## household.income:                                    .
##     [<5K]                86 (3.73%)   9 (4.64%)
##     [>=200K]            298 (12.9%)  16 (8.25%)
##     [100K-200K]         699 (30.3%)  45 (23.2%)
##     [12K-16K]            54 (2.34%)   7 (3.61%)
##     [16K-25K]            98 (4.25%)  13 (6.70%)
##     [25K-35K]           140 (6.07%)  18 (9.28%)
##     [35K-50K]           193 (8.37%)  22 (11.3%)
##     [50K-75K]           307 (13.3%)  29 (14.9%)
##     [5K-12K]             83 (3.60%)   7 (3.61%)
##     [75K-100K]          347 (15.1%)  28 (14.4%)
## high.educ:                                        <0.001
##     < HS Diploma        111 (4.46%)  11 (5.26%)
##     Bachelor            660 (26.5%)  41 (19.6%)
##     HS Diploma/GED      226 (9.07%)  29 (13.9%)
##     Post Graduate Degree 880 (35.3%)  51 (24.4%)
##     Some College        614 (24.6%)  77 (36.8%)
## demo_race_hispanic:                                0.080
##     0                   1970 (80.0%) 156 (74.6%)
##     1                   493 (20.0%)  53 (25.4%)
## ___________________________________________________________
```

Male participants

```
##        interview_age                  bmi race.ethnicity.5level
##           0.19394814           0.29925725            0.19394814
##     household.income            high.educ    demo_race_hispanic
```

```
##           0.05039636           0.05393549           0.61967474

##
## --------Summary descriptives table by 'is_outlier_any'---------
##
## ___________________________________________________________
##                          not outlier    outlier   p.overall
##                             N=2664       N=260
## ¯¯¯¯¯¯¯¯¯¯¯¯¯¯¯¯¯¯¯¯¯¯¯¯¯¯¯¯¯¯¯¯¯¯¯¯¯¯¯¯¯¯¯¯¯¯¯¯¯¯¯¯¯¯¯¯¯¯¯¯¯
## interview_age             119 (7.47)  119 (7.56)    0.129
## bmi                      18.6 (3.67)  18.9 (4.11)   0.249
## race.ethnicity.5level:                              0.098
##     Asian                 58 (2.20%)   7 (2.72%)
##     Black                376 (14.3%)  46 (17.9%)
##     Mixed                313 (11.9%)  38 (14.8%)
##     Other                123 (4.67%)  16 (6.23%)
##     White                1765 (67.0%) 150 (58.4%)
## household.income:                                   0.008
##     [<5K]                 83 (3.37%)  11 (4.60%)
##     [>=200K]             285 (11.6%)  30 (12.6%)
##     [100K-200K]          787 (31.9%)  76 (31.8%)
##     [12K-16K]             61 (2.47%)   4 (1.67%)
##     [16K-25K]            116 (4.70%)  22 (9.21%)
##     [25K-35K]            143 (5.80%)   9 (3.77%)
##     [35K-50K]            206 (8.35%)  15 (6.28%)
##     [50K-75K]            351 (14.2%)  36 (15.1%)
##     [5K-12K]              78 (3.16%)  14 (5.86%)
##     [75K-100K]           356 (14.4%)  22 (9.21%)
## high.educ:                                          0.018
##     < HS Diploma         102 (3.83%)  17 (6.54%)
##     Bachelor             734 (27.6%)  60 (23.1%)
##     HS Diploma/GED       232 (8.72%)  29 (11.2%)
##     Post Graduate Degree 904 (34.0%)  74 (28.5%)
##     Some College         688 (25.9%)  80 (30.8%)
## demo_race_hispanic:                                 0.620
##     0                    2109 (80.1%) 198 (78.6%)
##     1                    524 (19.9%)  54 (21.4%)
## ¯¯¯¯¯¯¯¯¯¯¯¯¯¯¯¯¯¯¯¯¯¯¯¯¯¯¯¯¯¯¯¯¯¯¯¯¯¯¯¯¯¯¯¯¯¯¯¯¯¯¯¯¯¯¯¯¯¯¯¯¯
```
