# Supplementary material for: Reward sensitivity and internalizing symptoms during the transition to puberty: An examination of 9-and 10-year-olds in the ABCD Study
Source: Dev Cogn Neurosci. 2022 Oct 31;58:101172. doi: 10.1016/j.dcn.2022.101172 (PMC9649995; doi:10.1016/j.dcn.2022.101172)
Supplement: Supplementary file 2 — Supplementary material [file mmc2.pdf]

Supplement B

Reward sensitivity and internalizing symptoms during the transition to puberty: An examination of 9- and 10-year-olds in the ABCD Study.

Contents

Results for Sample 2 **9**

1—Internalizing~Puberty— **9**

1.1 Model: CBCL internalizing factor ~ PDS 9

Female participants 9

Male participants 9

1.2 Model: CBCL Anxious-Depressed ~ PDS 10

Female participants 10

Male participants 11

1.3 Model: CBCL Withdrawn-Depressed ~ PDS 12

Female participants 12

Male participants 12

1.4 Model: CBCL Depressed DSM-5 ~ PDS 13

Female participants 13

Male participants 14

1.5 Model: CBCL internalizing factor ~ Pubertal category 14

Female participants 14

Male participants 15

1.6 Model: CBCL Anxious-Depressed ~ Pubertal category 16

Female participants 16

Male participants 17

1.7 Model: CBCL Withdrawn-Depressed ~ Pubertal category 18

Female participants 18

Male participants 18

1.8 Model: CBCL Depressed DSM-5 ~ Pubertal category 19

Female participants 19

Male participants 20

1.9 Model: CBCL internalizing factor ~ Testosterone 21
Female participants 21
Male participants 22
1.10 Model: CBCL Anxious-Depressed ~ Testosterone 22
Female participants 22
Male participants 23
1.11 Model: CBCL Withdrawn-Depressed ~ Testosterone 24
Female participants 24
Male participants 24
1.12 Model: CBCL Depressed DSM-5 ~ Testosterone 25
Female participants 25
Male participants 26
1.13 Model: CBCL internalizing factor ~ Testosterone + PDS 27
Female participants 27
Male participants 27
1.14 Model: CBCL internalizing factor ~ Testosterone + Pubertal category 28
Female participants 28
Male participants 29
1.15 Model: CBCL Anxious-Depressed ~ Testosterone + PDS 30
Female participants 30
Male participants 30
1.16 Model: CBCL Anxious-Depressed ~ Testosterone + Pubertal category 31
Female participants 31
Male participants 32
1.17 Model: CBCL Withdrawn-Depressed ~ Testosterone + PDS 33
Female participants 33
Male participants 34
1.18 Model: CBCL Withdrawn-Depressed ~ Testosterone + Pubertal category 34
Female participants 34
Male participants 35
1.19 Model: CBCL Depressed DSM-5 ~ Testosterone + PDS 36
Female participants 36
Male participants 37
1.20 Model: CBCL Depressed DSM-5 ~ Testosterone + Pubertal category 38
Female participants 38
Male participants 38

2—Reward~Puberty— 40

2.1 Model: BIS-BAS-RR ~ PDS 40

Female participants 40

Male participants 40

2.2 Model : Reaction Time ~ PDS 41

Female participants 41

Male participants 42

2.3 Model: Caudate Anticipation ~ PDS 42

Female participants 42

Male participants 43

2.4 Model B: Putamen Anticipation ~ PDS 43

Female participants 43

Male participants 44

2.5 Model: Accumbens Anticipation ~ PDS 44

Female participants 44

Male participants 45

2.6 Model: Caudate Feedback ~ PDS 45

Female participants 45

Male participants 46

2.7 Model: Putamen Feedback ~ PDS 46

Female participants 46

Male participants 47

2.8 Model: Accumbens Feedback ~ PDS 47

Female participants 47

Male participants 48

2.9 Model: OFC Anticipation ~ PDS 48

Female participants 48

Male participants 49

2.10 Model: OFC Feedback ~ PDS 50

Female participants 50

Male participants 51

2.11 Model: Caudate Anticipation ~ Testosterone 52

Female participants 52

Male participants 52

2.12 Model B: Putamen Anticipation ~ Testosterone 53

Female participants 53

Male participants 53
2.13 Model: Accumbens Anticipation ~ Testosterone 54
Female participants 54
Male participants 54
2.14 Model: Caudate Feedback ~ Testosterone 55
Female participants 55
Male participants 55
2.15 Model: Putamen Feedback ~ Testosterone 56
Female participants 56
Male participants 56
2.16 Model: Accumbens Feedback ~ Testosterone 57
Female participants 57
Male participants 57
2.17 Model: OFC Anticipation ~ Testosterone 58
Female participants 58
Male participants 59
2.18 Model: OFC Feedback ~ Testosterone 60
Female participants 60
Male participants 60
2.19 Model: MID Reaction Time ~ Testosterone 61
Female participants 61
Male participants 62
2.20 Model: BIS-BAS-RR ~ Testosterone 63
Female participants 63
Male participants 64

3—Internalizing~Reward— 65

3.1 Model: CBCL internalizing factor ~ Nucleus Accumbens activity (anticipation stage) 65
Female participants 65
Male participants 65
3.2 Model: CBCL internalizing factor ~ Caudate activity (anticipation stage) 66
Female participants 66
Male participants 66
3.3 Model: CBCL internalizing factor ~ Putamen activity (anticipation stage) 67
Female participants 67
Male participants 67
3.4 Model: CBCL internalizing factor ~ Accumbens activity (feedback stage) 68

Female participants 68
Male participants 68
3.5 Model: CBCL internalizing factor ~ Caudate activity (feedback stage) 69
Female participants 69
Male participants 69
3.6 Model: CBCL internalizing factor ~ Putamen activity (feedback stage) 70
Female participants 70
Male participants 70
3.7 Model: CBCL internalizing factor ~ OFC activity (anticipation stage) 71
Female participants 71
Male participants 72
3.8 Model: CBCL internalizing factor ~ OFC activity (feedback stage) 73
Female participants 73
Male participants 74
3.9 Model: CBCL internalizing factor ~ BIS-BAS-RR 74
Female participants 74
Male participants 75
3.10 Model: CBCL internalizing factor ~ MID Reaction Time 75
Female participants 75
Male participants 76

4—Internalizing~Puberty x Reward— 78
4.1 Model: CBCL internalizing factor ~ PDS x Accumbens activity (anticipation stage) 78
Female participants 78
Male participants 78
4.2 Model: CBCL internalizing factor ~ PDS x Caudate activity (anticipation stage) 79
Female participants 79
Male participants 80
4.3 Model: CBCL internalizing factor ~ PDS x Putamen activity (anticipation stage) 81
Female participants 81
Male participants 82
4.4 Model: CBCL internalizing factor ~ PDS x Lateral OFC activity (anticipation stage) 82
Female participants 82
Male participants 83
4.5 Model: CBCL internalizing factor ~ PDS x Medial OFC activity (anticipation stage) 84
Female participants 84
Male participants 85

4.6 Model: CBCL internalizing factor ~ PDS x Accumbens activity (feedback) 86
Female participants 86
Male participants 87
4.7 Model: CBCL internalizing factor ~ PDS x Caudate activity (feedback) 88
Female participants 88
Male participants 88
4.8 Model: CBCL internalizing factor ~ PDS x Putamen activity (feedback) 89
Female participants 89
Male participants 90
4.9 Model: CBCL internalizing factor ~ PDS x Lateral OFC activity (feedback stage) 91
Female participants 91
Male participants 92
4.10 Model: CBCL internalizing factor ~ PDS x Medial OFC activity (feedback stage) 92
Female participants 92
Male participants 93
4.11 Model: CBCL internalizing factor ~ PDS x BIS-BAS 94
Female participants 94
Male participants 95
4.12 Model: CBCL internalizing factor ~ PDS x MID reaction time (large reward vs. neutral) . . . 96
Female participants 96
Male participants 96
4.13 Model: CBCL internalizing factor ~ PDS x MID reaction time (large vs. small reward) 97
Female participants 97
Male participants 98
4.14 Model: CBCL internalizing factor ~ Testosterone x Accumbens activity (anticipation stage) + PDS 99
Female participants 99
Male participants 100
4.15 Model: CBCL internalizing factor ~ Testosterone x Caudate activity (anticipation stage) + PDS101
Female participants 101
Male participants 102
4.16 Model: CBCL internalizing factor ~ Testosterone x Putamen activity (anticipation stage) + PDS 103
Female participants 103
Male participants 104
4.17 Model: CBCL internalizing factor ~ Testosterone x Accumbens activity (feedback stage) + PDS105
Female participants 105

Male participants 106
4.18 Model: CBCL internalizing factor ~ Testosterone x Caudate activity (Feedback stage) + PDS 108
Female participants 108
Male participants 109
4.19 Model: CBCL internalizing factor ~ Testosterone x Putamen activity (Feedback stage) + PDS 110
Female participants 110
Male participants 111
4.20 Model: CBCL internalizing factor ~ Testosterone x Lateral OFC activity (anticipation stage) + PDS 112
Female participants 112
Male participants 113
4.21 Model: CBCL internalizing factor ~ Testosterone x Medial OFC activity (anticipation stage) + PDS 114
Female participants 114
Male participants 114
4.22 Model: CBCL internalizing factor ~ Testosterone x Lateral OFC activity (feedback stage) + PDS 115
Female participants 115
Male participants 116
4.23 Model: CBCL internalizing factor ~ Testosterone x Medial OFC activity (feedback stage) + PDS 117
Female participants 117
Male participants 119
4.24 Model: CBCL internalizing factor ~ Testosterone x BIS-BAS RR + PDS 120
Female participants 120
Male participants 121
4.25 Model: CBCL internalizing factor ~ Testosterone x MID Reaction Time + PDS (large reward vs. neutral) 122
Female participants 122
Male participants 123
4.26 Model: CBCL internalizing factor ~ Testosterone x MID Reaction Time + PDS (large vs. small reward) 124
Female participants 124
Male participants 125

5— Correlation Matrix — 128

Female participants 128
Male participants 129

6— Compare Outliers to Non-Outliers on Demographic Variables — 130

Female participants 130

Male participants 130

Results for Sample 2

1—Internalizing~Puberty—

1.1 Model: CBCL internalizing factor ~ PDS

Female participants

```
##
## Family: gaussian
## Link function: identity
##
## Formula:
## cbcl_scr_syn_internal_r ~ PDS_score + race.ethnicity.5level +
##     interview_age + demo_race_hispanic
##
## Parametric coefficients:
##                            Estimate Std. Error t value Pr(>|t|)
## (Intercept)                 1.00435    1.81456   0.553   0.5800
## PDS_score                   0.78180    0.15854   4.931 8.69e-07 ***
## race.ethnicity.5levelBlack -0.25319    0.71823  -0.353   0.7245
## race.ethnicity.5levelMixed  1.22839    0.71864   1.709   0.0875 .
## race.ethnicity.5levelOther  0.39856    0.82932   0.481   0.6309
## race.ethnicity.5levelWhite  0.99113    0.66629   1.488   0.1370
## interview_age               0.01562    0.01471   1.062   0.2885
## demo_race_hispanic1         0.25310    0.32178   0.787   0.4316
## ---
## Signif. codes:  0 '***' 0.001 '**' 0.01 '*' 0.05 '.' 0.1 ' ' 1
##
##
## R-sq.(adj) =  0.0128
## lmer.REML =  16187  Scale est. = 17.323    n = 2620
```

```
##                                  stdcoef      stdse
## X(Intercept)                 0.00000000 0.00000000
## XPDS_score                   0.10536577 0.02136725
## Xrace.ethnicity.5levelBlack -0.01733433 0.04917261
## Xrace.ethnicity.5levelMixed  0.07387880 0.04322130
## Xrace.ethnicity.5levelOther  0.01604117 0.03337861
## Xrace.ethnicity.5levelWhite  0.08799336 0.05915374
## Xinterview_age               0.02151197 0.02026243
## Xdemo_race_hispanic1         0.01832534 0.02329731
```

Male participants

```
##
## Family: gaussian
## Link function: identity
##
## Formula:
## cbcl_scr_syn_internal_r ~ PDS_score + race.ethnicity.5level +
##     interview_age + demo_race_hispanic
```

```
##
## Parametric coefficients:
##                            Estimate Std. Error t value Pr(>|t|)
## (Intercept)                3.058782   1.842397   1.660 0.096982 .
## PDS_score                  0.672276   0.198546   3.386 0.000719 ***
## race.ethnicity.5levelBlack -0.831114   0.792617  -1.049 0.294466
## race.ethnicity.5levelMixed  0.427434   0.788949   0.542 0.588015
## race.ethnicity.5levelOther -0.893416   0.887818  -1.006 0.314355
## race.ethnicity.5levelWhite -0.061289   0.738629  -0.083 0.933876
## interview_age              0.009474   0.014107   0.672 0.501897
## demo_race_hispanic1        0.621208   0.322430   1.927 0.054124 .
## ---
## Signif. codes:  0 '***' 0.001 '**' 0.01 '*' 0.05 '.' 0.1 ' ' 1
##
##
## R-sq.(adj) =  0.00579
## lmer.REML =  17729  Scale est. = 17.104    n = 2832
```

```
##                                stdcoef      stdse
## X(Intercept)                0.00000000 0.00000000
## XPDS_score                  0.06632796 0.01958892
## Xrace.ethnicity.5levelBlack -0.05010210 0.04778137
## Xrace.ethnicity.5levelMixed  0.02476863 0.04571739
## Xrace.ethnicity.5levelOther -0.03540337 0.03518153
## Xrace.ethnicity.5levelWhite -0.00511654 0.06166235
## Xinterview_age              0.01269154 0.01889762
## Xdemo_race_hispanic1        0.04420342 0.02294319
```

1.2 Model: CBCL Anxious-Depressed ~ PDS

Female participants

```
##
## Family: gaussian
## Link function: identity
##
## Formula:
## cbcl_scr_syn_anxdep_r ~ PDS_score + race.ethnicity.5level + interview_age +
##     demo_race_hispanic
##
## Parametric coefficients:
##                            Estimate Std. Error t value Pr(>|t|)
## (Intercept)                1.108103   1.010173   1.097 0.272767
## PDS_score                  0.327885   0.088132   3.720 0.000203 ***
## race.ethnicity.5levelBlack -0.296592   0.397328  -0.746 0.455452
## race.ethnicity.5levelMixed  0.756457   0.398018   1.901 0.057470 .
## race.ethnicity.5levelOther  0.204912   0.459613   0.446 0.655753
## race.ethnicity.5levelWhite  0.584367   0.368865   1.584 0.113262
## interview_age              0.003237   0.008208   0.394 0.693331
## demo_race_hispanic1        0.074772   0.177107   0.422 0.672924
## ---
## Signif. codes:  0 '***' 0.001 '**' 0.01 '*' 0.05 '.' 0.1 ' ' 1
##
```

```
##
## R-sq.(adj) =  0.0128
## lmer.REML =  13136  Scale est. = 6.6266    n = 2620
```

```
##                                  stdcoef      stdse
## X(Intercept)                 0.000000000 0.00000000
## XPDS_score                   0.079555971 0.02138383
## Xrace.ethnicity.5levelBlack -0.036556917 0.04897321
## Xrace.ethnicity.5levelMixed  0.081906281 0.04309585
## Xrace.ethnicity.5levelOther  0.014847825 0.03330340
## Xrace.ethnicity.5levelWhite  0.093401575 0.05895699
## Xinterview_age               0.008028059 0.02035606
## Xdemo_race_hispanic1         0.009746320 0.02308537
```

Male participants

```
##
## Family: gaussian
## Link function: identity
##
## Formula:
## cbcl_scr_syn_anxdep_r ~ PDS_score + race.ethnicity.5level + interview_age +
##     demo_race_hispanic
##
## Parametric coefficients:
##                              Estimate Std. Error t value Pr(>|t|)
## (Intercept)                  2.678399   1.026431   2.609  0.00912 **
## PDS_score                    0.287407   0.110522   2.600  0.00936 **
## race.ethnicity.5levelBlack -0.369943   0.438408  -0.844  0.39883
## race.ethnicity.5levelMixed  0.350066   0.436534   0.802  0.42267
## race.ethnicity.5levelOther -0.229707   0.492277  -0.467  0.64081
## race.ethnicity.5levelWhite  0.242988   0.408759   0.594  0.55226
## interview_age              -0.006203   0.007875  -0.788  0.43094
## demo_race_hispanic1          0.260989   0.177342   1.472  0.14122
## ---
## Signif. codes:  0 '***' 0.001 '**' 0.01 '*' 0.05 '.' 0.1 ' ' 1
##
##
## R-sq.(adj) =  0.0047
## lmer.REML =  14428  Scale est. = 7.0594    n = 2832
```

```
##                                 stdcoef      stdse
## X(Intercept)                 0.00000000 0.00000000
## XPDS_score                   0.05122276 0.01969765
## Xrace.ethnicity.5levelBlack -0.04028538 0.04774088
## Xrace.ethnicity.5levelMixed  0.03664369 0.04569493
## Xrace.ethnicity.5levelOther -0.01644307 0.03523852
## Xrace.ethnicity.5levelWhite  0.03664339 0.06164226
## Xinterview_age              -0.01501059 0.01905650
## Xdemo_race_hispanic1         0.03354727 0.02279537
```

1.3 Model: CBCL Withdrawn-Depressed ~ PDS

Female participants

```
##
## Family: gaussian
## Link function: identity
##
## Formula:
## cbcl_scr_syn_withdep_r ~ PDS_score + race.ethnicity.5level +
##     interview_age + demo_race_hispanic
##
## Parametric coefficients:
##                             Estimate Std. Error t value Pr(>|t|)
## (Intercept)                 0.264236   0.542461   0.487    0.626
## PDS_score                   0.222482   0.047112   4.722 2.45e-06 ***
## race.ethnicity.5levelBlack -0.192139   0.212400  -0.905    0.366
## race.ethnicity.5levelMixed -0.016699   0.212612  -0.079    0.937
## race.ethnicity.5levelOther -0.052069   0.245382  -0.212    0.832
## race.ethnicity.5levelWhite -0.104268   0.197265  -0.529    0.597
## interview_age               0.003147   0.004407   0.714    0.475
## demo_race_hispanic1         0.155883   0.094464   1.650    0.099 .
## ---
## Signif. codes:  0 '***' 0.001 '**' 0.01 '*' 0.05 '.' 0.1 ' ' 1
##
##
## R-sq.(adj) =   0.01
## lmer.REML = 9892.2  Scale est. = 2.4029    n = 2620
```

```
##                                   stdcoef      stdse
## X(Intercept)                  0.000000000 0.00000000
## XPDS_score                    0.100765118 0.02133740
## Xrace.ethnicity.5levelBlack -0.044206830 0.04886834
## Xrace.ethnicity.5levelMixed -0.003375126 0.04297178
## Xrace.ethnicity.5levelOther -0.007042721 0.03318966
## Xrace.ethnicity.5levelWhite -0.031108702 0.05885468
## Xinterview_age                0.014566340 0.02040275
## Xdemo_race_hispanic1          0.037928350 0.02298439
```

Male participants

```
##
## Family: gaussian
## Link function: identity
##
## Formula:
## cbcl_scr_syn_withdep_r ~ PDS_score + race.ethnicity.5level +
##     interview_age + demo_race_hispanic
##
## Parametric coefficients:
##                             Estimate Std. Error t value Pr(>|t|)
## (Intercept)                -0.063981   0.606166  -0.106   0.9159
## PDS_score                   0.186135   0.065463   2.843   0.0045 **
```

```
## race.ethnicity.5levelBlack -0.182782   0.259995  -0.703   0.4821
## race.ethnicity.5levelMixed -0.013421   0.259142  -0.052   0.9587
## race.ethnicity.5levelOther -0.212504   0.292036  -0.728   0.4669
## race.ethnicity.5levelWhite -0.237451   0.242533  -0.979   0.3276
## interview_age               0.009317   0.004651   2.003   0.0452 *
## demo_race_hispanic1         0.134507   0.104104   1.292   0.1964
## ---
## Signif. codes:  0 '***' 0.001 '**' 0.01 '*' 0.05 '.' 0.1 ' ' 1
##
##
## R-sq.(adj) =  0.00457
## lmer.REML =  11461  Scale est. = 2.0684    n = 2832
```

```
##                                  stdcoef      stdse
## X(Intercept)                 0.000000000 0.00000000
## XPDS_score                   0.056067558 0.01971859
## Xrace.ethnicity.5levelBlack -0.033640575 0.04785127
## Xrace.ethnicity.5levelMixed -0.002374361 0.04584625
## Xrace.ethnicity.5levelOther -0.025709394 0.03533144
## Xrace.ethnicity.5levelWhite -0.060520311 0.06181563
## Xinterview_age               0.038105692 0.01902161
## Xdemo_race_hispanic1         0.029221142 0.02261626
```

1.4 Model: CBCL Depressed DSM-5 ~ PDS

Female participants

```
##
## Family: gaussian
## Link function: identity
##
## Formula:
## cbcl_scr_dsm5_depress_r ~ PDS_score + race.ethnicity.5level +
##     interview_age + demo_race_hispanic
##
## Parametric coefficients:
##                             Estimate Std. Error t value Pr(>|t|)
## (Intercept)                 0.877508   0.632006   1.388  0.16512
## PDS_score                   0.192397   0.055287   3.480  0.00051 ***
## race.ethnicity.5levelBlack -0.076952   0.249336  -0.309  0.75763
## race.ethnicity.5levelMixed  0.219642   0.249929   0.879  0.37958
## race.ethnicity.5levelOther  0.024488   0.288648   0.085  0.93240
## race.ethnicity.5levelWhite  0.128716   0.231475   0.556  0.57821
## interview_age              -0.001469   0.005136  -0.286  0.77490
## demo_race_hispanic1         0.126429   0.111105   1.138  0.25525
## ---
## Signif. codes:  0 '***' 0.001 '**' 0.01 '*' 0.05 '.' 0.1 ' ' 1
##
##
## R-sq.(adj) =  0.0042
## lmer.REML =  10689  Scale est. = 2.2893    n = 2620
```

```
##                                  stdcoef      stdse
```

```
## X(Intercept)                 0.000000000 0.00000000
## XPDS_score                   0.075023445 0.02155878
## Xrace.ethnicity.5levelBlack -0.015243330 0.04939042
## Xrace.ethnicity.5levelMixed  0.038220433 0.04349083
## Xrace.ethnicity.5levelOther  0.002851615 0.03361339
## Xrace.ethnicity.5levelWhite  0.033063515 0.05945922
## Xinterview_age              -0.005854702 0.02047123
## Xdemo_race_hispanic1         0.026484767 0.02327452
```

Male participants

```
##
## Family: gaussian
## Link function: identity
##
## Formula:
## cbcl_scr_dsm5_depress_r ~ PDS_score + race.ethnicity.5level +
##     interview_age + demo_race_hispanic
##
## Parametric coefficients:
##                             Estimate Std. Error t value Pr(>|t|)
## (Intercept)                 0.291321   0.703319   0.414    0.679
## PDS_score                   0.156004   0.075919   2.055    0.040 *
## race.ethnicity.5levelBlack -0.050116   0.302313  -0.166    0.868
## race.ethnicity.5levelMixed  0.186032   0.301170   0.618    0.537
## race.ethnicity.5levelOther -0.269981   0.339141  -0.796    0.426
## race.ethnicity.5levelWhite -0.039717   0.281893  -0.141    0.888
## interview_age               0.007343   0.005392   1.362    0.173
## demo_race_hispanic1         0.151825   0.121806   1.246    0.213
## ---
## Signif. codes:  0 '***' 0.001 '**' 0.01 '*' 0.05 '.' 0.1 ' ' 1
##
##
## R-sq.(adj) =  0.00245
## lmer.REML =  12298  Scale est. = 2.5816    n = 2832
```

```
##                                  stdcoef      stdse
## X(Intercept)                 0.000000000 0.00000000
## XPDS_score                   0.040516619 0.01971730
## Xrace.ethnicity.5levelBlack -0.007952833 0.04797349
## Xrace.ethnicity.5levelMixed  0.028377226 0.04594033
## Xrace.ethnicity.5levelOther -0.028162680 0.03537701
## Xrace.ethnicity.5levelWhite -0.008728164 0.06194806
## Xinterview_age               0.025893531 0.01901349
## Xdemo_race_hispanic1         0.028438914 0.02281585
```

1.5 Model: CBCL internalizing factor ~ Pubertal category

Female participants

```
##
## Family: gaussian
```

```
## Link function: identity
##
## Formula:
## cbcl_scr_syn_internal_r ~ pds_p_ss_category + race.ethnicity.5level +
##     interview_age + demo_race_hispanic
##
## Parametric coefficients:
##                            Estimate Std. Error t value Pr(>|t|)
## (Intercept)                 1.29818    1.86143   0.697 0.485610
## pds_p_ss_categoryEarly      0.30134    0.28987   1.040 0.298645
## pds_p_ss_categoryLate       0.78997    0.69372   1.139 0.254914
## pds_p_ss_categoryMid        0.99238    0.26806   3.702 0.000218 ***
## race.ethnicity.5levelBlack -0.12030    0.71934  -0.167 0.867198
## race.ethnicity.5levelMixed  1.25918    0.72006   1.749 0.080456 .
## race.ethnicity.5levelOther  0.42346    0.83096   0.510 0.610371
## race.ethnicity.5levelWhite  1.00589    0.66757   1.507 0.131986
## interview_age               0.01957    0.01499   1.305 0.191958
## demo_race_hispanic1         0.19558    0.32298   0.606 0.544867
## ---
## Signif. codes:  0 '***' 0.001 '**' 0.01 '*' 0.05 '.' 0.1 ' ' 1
##
##
## R-sq.(adj) =  0.0091
## lmer.REML =  16196  Scale est. = 17.558    n = 2620
```

```
##                                    stdcoef      stdse
## X(Intercept)                 0.000000000 0.00000000
## Xpds_p_ss_categoryEarly      0.023342057 0.02245401
## Xpds_p_ss_categoryLate       0.023360730 0.02051446
## Xpds_p_ss_categoryMid        0.090723632 0.02450653
## Xrace.ethnicity.5levelBlack -0.008236061 0.04924855
## Xrace.ethnicity.5levelMixed  0.075730420 0.04330612
## Xrace.ethnicity.5levelOther  0.017043625 0.03344477
## Xrace.ethnicity.5levelWhite  0.089303475 0.05926738
## Xinterview_age               0.026952773 0.02065116
## Xdemo_race_hispanic1         0.014160288 0.02338424
```

Male participants

```
##
## Family: gaussian
## Link function: identity
##
## Formula:
## cbcl_scr_syn_internal_r ~ pds_p_ss_category + race.ethnicity.5level +
##     interview_age + demo_race_hispanic
##
## Parametric coefficients:
##                            Estimate Std. Error t value Pr(>|t|)
## (Intercept)                 3.31718    1.84808   1.795   0.0728 .
## pds_p_ss_categoryEarly      0.54750    0.25778   2.124   0.0338 *
## pds_p_ss_categoryLate       0.13707    1.51153   0.091   0.9278
## pds_p_ss_categoryMid        1.15909    0.47569   2.437   0.0149 *
```

```
## race.ethnicity.5levelBlack -0.72574    0.79246  -0.916   0.3599
## race.ethnicity.5levelMixed  0.50254    0.79001   0.636   0.5248
## race.ethnicity.5levelOther -0.86211    0.88860  -0.970   0.3320
## race.ethnicity.5levelWhite  0.03352    0.73983   0.045   0.9639
## interview_age               0.01255    0.01405   0.893   0.3718
## demo_race_hispanic1         0.59764    0.32348   1.848   0.0648 .
## ---
## Signif. codes:  0 '***' 0.001 '**' 0.01 '*' 0.05 '.' 0.1 ' ' 1
##
##
## R-sq.(adj) =  0.00411
## lmer.REML =  17728  Scale est. = 17.081    n = 2832
```

```
##                                  stdcoef      stdse
## X(Intercept)                 0.000000000 0.00000000
## Xpds_p_ss_categoryEarly      0.040995710 0.01930197
## Xpds_p_ss_categoryLate       0.001711736 0.01887643
## Xpds_p_ss_categoryMid        0.047789864 0.01961283
## Xrace.ethnicity.5levelBlack -0.043749661 0.04777213
## Xrace.ethnicity.5levelMixed  0.029120905 0.04577898
## Xrace.ethnicity.5levelOther -0.034162882 0.03521257
## Xrace.ethnicity.5levelWhite  0.002798263 0.06176302
## Xinterview_age               0.016806689 0.01881649
## Xdemo_race_hispanic1         0.042526084 0.02301773
```

1.6 Model: CBCL Anxious-Depressed ~ Pubertal category

Female participants

```
##
## Family: gaussian
## Link function: identity
##
## Formula:
## cbcl_scr_syn_anxdep_r ~ pds_p_ss_category + race.ethnicity.5level +
##     interview_age + demo_race_hispanic
##
## Parametric coefficients:
##                             Estimate Std. Error t value Pr(>|t|)
## (Intercept)                 1.119072   1.035379   1.081  0.27987
## pds_p_ss_categoryEarly      0.197344   0.161806   1.220  0.22272
## pds_p_ss_categoryLate       0.160095   0.387250   0.413  0.67934
## pds_p_ss_categoryMid        0.389693   0.149135   2.613  0.00903 **
## race.ethnicity.5levelBlack -0.212867   0.397768  -0.535  0.59259
## race.ethnicity.5levelMixed  0.774922   0.398616   1.944  0.05200 .
## race.ethnicity.5levelOther  0.218712   0.460309   0.475  0.63473
## race.ethnicity.5levelWhite  0.592456   0.369390   1.604  0.10886
## interview_age               0.005779   0.008357   0.692  0.48925
## demo_race_hispanic1         0.054532   0.177675   0.307  0.75893
## ---
## Signif. codes:  0 '***' 0.001 '**' 0.01 '*' 0.05 '.' 0.1 ' ' 1
##
##
```

```
## R-sq.(adj) =  0.0101
## lmer.REML =  13144  Scale est. = 6.6835    n = 2620
```

```
##                                  stdcoef      stdse
## X(Intercept)                 0.000000000 0.00000000
## Xpds_p_ss_categoryEarly      0.027520492 0.02256461
## Xpds_p_ss_categoryLate       0.008523245 0.02061669
## Xpds_p_ss_categoryMid        0.064138031 0.02454552
## Xrace.ethnicity.5levelBlack -0.026237284 0.04902741
## Xrace.ethnicity.5levelMixed  0.083905616 0.04316057
## Xrace.ethnicity.5levelOther  0.015847778 0.03335384
## Xrace.ethnicity.5levelWhite  0.094694419 0.05904098
## Xinterview_age               0.014332455 0.02072361
## Xdemo_race_hispanic1         0.007108148 0.02315944
```

Male participants

```
##
## Family: gaussian
## Link function: identity
##
## Formula:
## cbcl_scr_syn_anxdep_r ~ pds_p_ss_category + race.ethnicity.5level +
##     interview_age + demo_race_hispanic
##
## Parametric coefficients:
##                             Estimate Std. Error t value Pr(>|t|)
## (Intercept)                 2.764965   1.029599   2.685  0.00729 **
## pds_p_ss_categoryEarly      0.195421   0.143528   1.362  0.17345
## pds_p_ss_categoryLate      -0.136256   0.838804  -0.162  0.87097
## pds_p_ss_categoryMid        0.442853   0.264922   1.672  0.09471 .
## race.ethnicity.5levelBlack -0.309604   0.438351  -0.706  0.48006
## race.ethnicity.5levelMixed  0.380709   0.437135   0.871  0.38387
## race.ethnicity.5levelOther -0.214669   0.492713  -0.436  0.66310
## race.ethnicity.5levelWhite  0.279504   0.409448   0.683  0.49489
## interview_age              -0.004581   0.007842  -0.584  0.55915
## demo_race_hispanic1         0.255484   0.177953   1.436  0.15120
## ---
## Signif. codes:  0 '***' 0.001 '**' 0.01 '*' 0.05 '.' 0.1 ' ' 1
##
##
## R-sq.(adj) =  0.00298
## lmer.REML =  14429  Scale est. = 7.0618    n = 2832
```

```
##                                  stdcoef      stdse
## X(Intercept)                 0.000000000 0.00000000
## Xpds_p_ss_categoryEarly      0.026432601 0.01941367
## Xpds_p_ss_categoryLate      -0.003073796 0.01892258
## Xpds_p_ss_categoryMid        0.032983362 0.01973118
## Xrace.ethnicity.5levelBlack -0.033714706 0.04773467
## Xrace.ethnicity.5levelMixed  0.039851301 0.04575777
## Xrace.ethnicity.5levelOther -0.015366542 0.03526972
## Xrace.ethnicity.5levelWhite  0.042150174 0.06174615
```

```
## Xinterview_age              -0.011085451 0.01897652
## Xdemo_race_hispanic1         0.032839759 0.02287397
```

1.7 Model: CBCL Withdrawn-Depressed ~ Pubertal category

Female participants

```
##
## Family: gaussian
## Link function: identity
##
## Formula:
## cbcl_scr_syn_withdep_r ~ pds_p_ss_category + race.ethnicity.5level +
##     interview_age + demo_race_hispanic
##
## Parametric coefficients:
##                            Estimate Std. Error t value Pr(>|t|)
## (Intercept)                0.418975   0.555757   0.754 0.450988
## pds_p_ss_categoryEarly     0.032612   0.086804   0.376 0.707168
## pds_p_ss_categoryLate      0.459686   0.207547   2.215 0.026856 *
## pds_p_ss_categoryMid       0.273222   0.079746   3.426 0.000622 ***
## race.ethnicity.5levelBlack -0.169202   0.212645  -0.796 0.426277
## race.ethnicity.5levelMixed -0.008629   0.212914  -0.041 0.967677
## race.ethnicity.5levelOther -0.045437   0.245728  -0.185 0.853315
## race.ethnicity.5levelWhite -0.098009   0.197526  -0.496 0.619808
## interview_age              0.003771   0.004484   0.841 0.400526
## demo_race_hispanic1        0.136455   0.094750   1.440 0.149941
## ---
## Signif. codes:  0 '***' 0.001 '**' 0.01 '*' 0.05 '.' 0.1 ' ' 1
##
##
## R-sq.(adj) =  0.0074
## lmer.REML = 9902.1  Scale est. = 2.4201    n = 2620
```

```
##                                 stdcoef      stdse
## X(Intercept)                0.000000000 0.00000000
## Xpds_p_ss_categoryEarly     0.008489447 0.02259618
## Xpds_p_ss_categoryLate      0.045682796 0.02062564
## Xpds_p_ss_categoryMid       0.083940423 0.02450006
## Xrace.ethnicity.5levelBlack -0.038929485 0.04892469
## Xrace.ethnicity.5levelMixed -0.001743948 0.04303298
## Xrace.ethnicity.5levelOther -0.006145691 0.03323642
## Xrace.ethnicity.5levelWhite -0.029241264 0.05893250
## Xinterview_age              0.017454233 0.02075860
## Xdemo_race_hispanic1        0.033201312 0.02305382
```

Male participants

```
##
## Family: gaussian
## Link function: identity
##
```

```
## Formula:
## cbcl_scr_syn_withdep_r ~ pds_p_ss_category + race.ethnicity.5level +
##     interview_age + demo_race_hispanic
##
## Parametric coefficients:
##                             Estimate Std. Error t value Pr(>|t|)
## (Intercept)                 0.002449   0.607574   0.004  0.99678
## pds_p_ss_categoryEarly      0.132614   0.084929   1.561  0.11852
## pds_p_ss_categoryLate      -0.238035   0.497191  -0.479  0.63215
## pds_p_ss_categoryMid        0.424294   0.156786   2.706  0.00685 **
## race.ethnicity.5levelBlack -0.153780   0.259695  -0.592  0.55379
## race.ethnicity.5levelMixed  0.015170   0.259260   0.059  0.95334
## race.ethnicity.5levelOther -0.201211   0.292061  -0.689  0.49092
## race.ethnicity.5levelWhite -0.204065   0.242712  -0.841  0.40055
## interview_age               0.010167   0.004628   2.197  0.02812 *
## demo_race_hispanic1         0.124921   0.104271   1.198  0.23100
## ---
## Signif. codes:  0 '***' 0.001 '**' 0.01 '*' 0.05 '.' 0.1 ' ' 1
##
##
## R-sq.(adj) =  0.00455
## lmer.REML =  11462  Scale est. = 2.08      n = 2832
```

```
##                                  stdcoef      stdse
## X(Intercept)                 0.000000000 0.00000000
## Xpds_p_ss_categoryEarly      0.030316362 0.01941525
## Xpds_p_ss_categoryLate      -0.009075646 0.01895656
## Xpds_p_ss_categoryMid        0.053409545 0.01973607
## Xrace.ethnicity.5levelBlack -0.028302694 0.04779604
## Xrace.ethnicity.5levelMixed  0.002683858 0.04586724
## Xrace.ethnicity.5levelOther -0.024343057 0.03533440
## Xrace.ethnicity.5levelWhite -0.052011083 0.06186126
## Xinterview_age               0.041582389 0.01892964
## Xdemo_race_hispanic1         0.027138523 0.02265241
```

1.8 Model: CBCL Depressed DSM-5 ~ Pubertal category

Female participants

```
##
## Family: gaussian
## Link function: identity
##
## Formula:
## cbcl_scr_dsm5_depress_r ~ pds_p_ss_category + race.ethnicity.5level +
##     interview_age + demo_race_hispanic
##
## Parametric coefficients:
##                              Estimate Std. Error t value Pr(>|t|)
## (Intercept)                 0.9682718  0.6476525   1.495   0.1350
## pds_p_ss_categoryEarly      0.0097474  0.1012280   0.096   0.9233
## pds_p_ss_categoryLate       0.3158399  0.2422368   1.304   0.1924
## pds_p_ss_categoryMid        0.2210179  0.0934547   2.365   0.0181 *
```

```
## race.ethnicity.5levelBlack -0.0494567  0.2494354  -0.198   0.8428
## race.ethnicity.5levelMixed  0.2280100  0.2501254   0.912   0.3621
## race.ethnicity.5levelOther  0.0302076  0.2888797   0.105   0.9167
## race.ethnicity.5levelWhite  0.1336232  0.2316532   0.577   0.5641
## interview_age              -0.0004703  0.0052282  -0.090   0.9283
## demo_race_hispanic1         0.1124883  0.1114040   1.010   0.3127
## ---
## Signif. codes:  0 '***' 0.001 '**' 0.01 '*' 0.05 '.' 0.1 ' ' 1
##
##
## R-sq.(adj) =  0.00221
## lmer.REML =  10696  Scale est. = 2.3302    n = 2620
```

```
##                                  stdcoef      stdse
## X(Intercept)                 0.000000000 0.00000000
## Xpds_p_ss_categoryEarly      0.002184582 0.02268725
## Xpds_p_ss_categoryLate       0.027023568 0.02072602
## Xpds_p_ss_categoryMid        0.058461203 0.02471961
## Xrace.ethnicity.5levelBlack -0.009796763 0.04941006
## Xrace.ethnicity.5levelMixed  0.039676615 0.04352497
## Xrace.ethnicity.5levelOther  0.003517710 0.03364039
## Xrace.ethnicity.5levelWhite  0.034323985 0.05950510
## Xinterview_age              -0.001874298 0.02083728
## Xdemo_race_hispanic1         0.023564393 0.02333726
```

Male participants

```
##
## Family: gaussian
## Link function: identity
##
## Formula:
## cbcl_scr_dsm5_depress_r ~ pds_p_ss_category + race.ethnicity.5level +
##     interview_age + demo_race_hispanic
##
## Parametric coefficients:
##                             Estimate Std. Error t value Pr(>|t|)
## (Intercept)                 0.379548   0.705032   0.538   0.5904
## pds_p_ss_categoryEarly      0.203805   0.098498   2.069   0.0386 *
## pds_p_ss_categoryLate       0.094555   0.577287   0.164   0.8699
## pds_p_ss_categoryMid        0.227129   0.181808   1.249   0.2117
## race.ethnicity.5levelBlack -0.033577   0.302044  -0.111   0.9115
## race.ethnicity.5levelMixed  0.197296   0.301372   0.655   0.5127
## race.ethnicity.5levelOther -0.265054   0.339220  -0.781   0.4347
## race.ethnicity.5levelWhite -0.018670   0.282163  -0.066   0.9473
## interview_age               0.007708   0.005366   1.437   0.1509
## demo_race_hispanic1         0.145882   0.122109   1.195   0.2323
## ---
## Signif. codes:  0 '***' 0.001 '**' 0.01 '*' 0.05 '.' 0.1 ' ' 1
##
##
## R-sq.(adj) =  0.00193
## lmer.REML =  12297  Scale est. = 2.5799    n = 2832
```

```
##                                   stdcoef      stdse
## X(Intercept)                  0.000000000 0.00000000
## Xpds_p_ss_categoryEarly       0.040171335 0.01941470
## Xpds_p_ss_categoryLate        0.003108413 0.01897773
## Xpds_p_ss_categoryMid         0.024651380 0.01973246
## Xrace.ethnicity.5levelBlack -0.005328331 0.04793089
## Xrace.ethnicity.5levelMixed  0.030095476 0.04597119
## Xrace.ethnicity.5levelOther -0.027648749 0.03538519
## Xrace.ethnicity.5levelWhite -0.004102781 0.06200735
## Xinterview_age               0.027182214 0.01892126
## Xdemo_race_hispanic1         0.027325654 0.02287254
```

1.9 Model: CBCL internalizing factor ~ Testosterone

Female participants

```
##
## Family: gaussian
## Link function: identity
##
## Formula:
## cbcl_scr_syn_internal_r ~ hormone_scr_ert_mean + race.ethnicity.5level +
##     interview_age + demo_race_hispanic
##
## Parametric coefficients:
##                              Estimate Std. Error t value Pr(>|t|)
## (Intercept)                 -0.855385   1.866170  -0.458  0.64673
## hormone_scr_ert_mean         0.006203   0.007005   0.886  0.37597
## race.ethnicity.5levelBlack   0.064913   0.725607   0.089  0.92872
## race.ethnicity.5levelMixed   1.367192   0.731022   1.870  0.06157 .
## race.ethnicity.5levelOther   0.392609   0.848930   0.462  0.64378
## race.ethnicity.5levelWhite   1.077302   0.675310   1.595  0.11078
## interview_age                0.039194   0.015051   2.604  0.00927 **
## demo_race_hispanic1          0.175791   0.333089   0.528  0.59771
## ---
## Signif. codes:  0 '***' 0.001 '**' 0.01 '*' 0.05 '.' 0.1 ' ' 1
##
##
## R-sq.(adj) =  0.0054
## lmer.REML =  14907  Scale est. = 17.56     n = 2409
```

```
##                                 stdcoef      stdse
## X(Intercept)                0.000000000 0.00000000
## Xhormone_scr_ert_mean       0.018821998 0.02125552
## Xrace.ethnicity.5levelBlack 0.004344118 0.04855950
## Xrace.ethnicity.5levelMixed 0.082458636 0.04408970
## Xrace.ethnicity.5levelOther 0.015908467 0.03439852
## Xrace.ethnicity.5levelWhite 0.095357412 0.05977511
## Xinterview_age              0.054151770 0.02079507
## Xdemo_race_hispanic1        0.012768403 0.02419353
```

Male participants

```
##
## Family: gaussian
## Link function: identity
##
## Formula:
## cbcl_scr_syn_internal_r ~ hormone_scr_ert_mean + race.ethnicity.5level +
##     interview_age + demo_race_hispanic
##
## Parametric coefficients:
##                             Estimate Std. Error t value Pr(>|t|)
## (Intercept)                 3.648782   1.877186   1.944    0.052 .
## hormone_scr_ert_mean        0.011484   0.007590   1.513    0.130
## race.ethnicity.5levelBlack -0.615060   0.810973  -0.758    0.448
## race.ethnicity.5levelMixed  0.293452   0.809804   0.362    0.717
## race.ethnicity.5levelOther -0.738635   0.909430  -0.812    0.417
## race.ethnicity.5levelWhite -0.014412   0.757886  -0.019    0.985
## interview_age               0.008913   0.014392   0.619    0.536
## demo_race_hispanic1         0.495960   0.331348   1.497    0.135
## ---
## Signif. codes:  0 '***' 0.001 '**' 0.01 '*' 0.05 '.' 0.1 ' ' 1
##
##
## R-sq.(adj) =  0.000756
## lmer.REML =  16412  Scale est. = 15.7      n = 2628
```

```
##                                  stdcoef      stdse
## X(Intercept)                 0.000000000 0.00000000
## Xhormone_scr_ert_mean        0.030571712 0.02020498
## Xrace.ethnicity.5levelBlack -0.037009874 0.04879853
## Xrace.ethnicity.5levelMixed  0.017131855 0.04727670
## Xrace.ethnicity.5levelOther -0.029714884 0.03658585
## Xrace.ethnicity.5levelWhite -0.001211391 0.06370447
## Xinterview_age               0.012133185 0.01959314
## Xdemo_race_hispanic1         0.035693514 0.02384661
```

1.10 Model: CBCL Anxious-Depressed ~ Testosterone

Female participants

```
##
## Family: gaussian
## Link function: identity
##
## Formula:
## cbcl_scr_syn_anxdep_r ~ hormone_scr_ert_mean + race.ethnicity.5level +
##     interview_age + demo_race_hispanic
##
## Parametric coefficients:
##                             Estimate Std. Error t value Pr(>|t|)
## (Intercept)                 0.174384   1.042465   0.167   0.8672
## hormone_scr_ert_mean        0.001262   0.003915   0.322   0.7473
```

```
## race.ethnicity.5levelBlack -0.114074   0.402044  -0.284   0.7766
## race.ethnicity.5levelMixed  0.830810   0.405722   2.048   0.0407 *
## race.ethnicity.5levelOther  0.225983   0.471554   0.479   0.6318
## race.ethnicity.5levelWhite  0.606919   0.374555   1.620   0.1053
## interview_age               0.014736   0.008431   1.748   0.0806 .
## demo_race_hispanic1         0.043874   0.183674   0.239   0.8112
## ---
## Signif. codes:  0 '***' 0.001 '**' 0.01 '*' 0.05 '.' 0.1 ' ' 1
##
##
## R-sq.(adj) =  0.00894
## lmer.REML =  12117  Scale est. = 6.9666    n = 2409
```

```
##                                  stdcoef      stdse
## X(Intercept)                 0.000000000 0.00000000
## Xhormone_scr_ert_mean        0.006862752 0.02129425
## Xrace.ethnicity.5levelBlack -0.013682794 0.04822397
## Xrace.ethnicity.5levelMixed  0.089810070 0.04385830
## Xrace.ethnicity.5levelOther  0.016411942 0.03424651
## Xrace.ethnicity.5levelWhite  0.096286332 0.05942228
## Xinterview_age               0.036490707 0.02087801
## Xdemo_race_hispanic1         0.005711696 0.02391131
```

Male participants

```
##
## Family: gaussian
## Link function: identity
##
## Formula:
## cbcl_scr_syn_anxdep_r ~ hormone_scr_ert_mean + race.ethnicity.5level +
##     interview_age + demo_race_hispanic
##
## Parametric coefficients:
##                              Estimate Std. Error t value Pr(>|t|)
## (Intercept)                  3.012459   1.045526   2.881  0.00399 **
## hormone_scr_ert_mean         0.005946   0.004217   1.410  0.15861
## race.ethnicity.5levelBlack -0.287294   0.448509  -0.641  0.52187
## race.ethnicity.5levelMixed  0.269561   0.448124   0.602  0.54754
## race.ethnicity.5levelOther -0.135198   0.504418  -0.268  0.78870
## race.ethnicity.5levelWhite  0.289551   0.419374   0.690  0.48998
## interview_age              -0.007605   0.008037  -0.946  0.34411
## demo_race_hispanic1         0.197889   0.182078   1.087  0.27721
## ---
## Signif. codes:  0 '***' 0.001 '**' 0.01 '*' 0.05 '.' 0.1 ' ' 1
##
##
## R-sq.(adj) =  0.0027
## lmer.REML =  13345  Scale est. = 6.4254    n = 2628
```

```
##                                  stdcoef      stdse
## X(Intercept)                 0.000000000 0.00000000
## Xhormone_scr_ert_mean        0.006862752 0.02129425
```

```
## Xrace.ethnicity.5levelBlack -0.013682794 0.04822397
## Xrace.ethnicity.5levelMixed  0.089810070 0.04385830
## Xrace.ethnicity.5levelOther  0.016411942 0.03424651
## Xrace.ethnicity.5levelWhite  0.096286332 0.05942228
## Xinterview_age               0.036490707 0.02087801
## Xdemo_race_hispanic1         0.005711696 0.02391131
```

1.11 Model: CBCL Withdrawn-Depressed ~ Testosterone

Female participants

```
##
## Family: gaussian
## Link function: identity
##
## Formula:
## cbcl_scr_syn_withdep_r ~ hormone_scr_ert_mean + race.ethnicity.5level +
##     interview_age + demo_race_hispanic
##
## Parametric coefficients:
##                             Estimate Std. Error t value Pr(>|t|)
## (Intercept)                -0.049692   0.560922  -0.089   0.9294
## hormone_scr_ert_mean        0.004957   0.002102   2.358   0.0184 *
## race.ethnicity.5levelBlack -0.124909   0.215595  -0.579   0.5624
## race.ethnicity.5levelMixed  0.034579   0.217293   0.159   0.8736
## race.ethnicity.5levelOther -0.058102   0.252429  -0.230   0.8180
## race.ethnicity.5levelWhite -0.055997   0.200812  -0.279   0.7804
## interview_age               0.007040   0.004534   1.553   0.1207
## demo_race_hispanic1         0.159992   0.098448   1.625   0.1043
## ---
## Signif. codes:  0 '***' 0.001 '**' 0.01 '*' 0.05 '.' 0.1 ' ' 1
##
##
## R-sq.(adj) =  0.00293
## lmer.REML = 9138.4  Scale est. = 2.3918    n = 2409
```

```
##                                  stdcoef      stdse
## X(Intercept)                 0.000000000 0.00000000
## Xhormone_scr_ert_mean        0.050354659 0.02135181
## Xrace.ethnicity.5levelBlack -0.027984173 0.04830116
## Xrace.ethnicity.5levelMixed  0.006981761 0.04387311
## Xrace.ethnicity.5levelOther -0.007881459 0.03424158
## Xrace.ethnicity.5levelWhite -0.016592972 0.05950501
## Xinterview_age               0.032560927 0.02097216
## Xdemo_race_hispanic1         0.038903013 0.02393831
```

Male participants

```
##
## Family: gaussian
## Link function: identity
##
```

```
## Formula:
## cbcl_scr_syn_withdep_r ~ hormone_scr_ert_mean + race.ethnicity.5level +
##     interview_age + demo_race_hispanic
##
## Parametric coefficients:
##                             Estimate Std. Error t value Pr(>|t|)
## (Intercept)                -0.065422   0.623459  -0.105   0.9164
## hormone_scr_ert_mean        0.005191   0.002515   2.064   0.0391 *
## race.ethnicity.5levelBlack -0.140493   0.267964  -0.524   0.6001
## race.ethnicity.5levelMixed -0.023699   0.268053  -0.088   0.9296
## race.ethnicity.5levelOther -0.148274   0.301637  -0.492   0.6231
## race.ethnicity.5levelWhite -0.217465   0.250758  -0.867   0.3859
## interview_age               0.010067   0.004796   2.099   0.0359 *
## demo_race_hispanic1         0.074814   0.107609   0.695   0.4870
## ---
## Signif. codes:  0 '***' 0.001 '**' 0.01 '*' 0.05 '.' 0.1 ' ' 1
##
##
## R-sq.(adj) =  0.00253
## lmer.REML =  10645  Scale est. = 2.0927    n = 2628
```

```
##                                  stdcoef      stdse
## X(Intercept)                 0.000000000 0.00000000
## Xhormone_scr_ert_mean        0.041822222 0.02025883
## Xrace.ethnicity.5levelBlack -0.025584149 0.04879711
## Xrace.ethnicity.5levelMixed -0.004187139 0.04735916
## Xrace.ethnicity.5levelOther -0.018052062 0.03672361
## Xrace.ethnicity.5levelWhite -0.055318793 0.06378776
## Xinterview_age               0.041473358 0.01975713
## Xdemo_race_hispanic1         0.016294514 0.02343740
```

1.12 Model: CBCL Depressed DSM-5 ~ Testosterone

Female participants

```
##
## Family: gaussian
## Link function: identity
##
## Formula:
## cbcl_scr_dsm5_depress_r ~ hormone_scr_ert_mean + race.ethnicity.5level +
##     interview_age + demo_race_hispanic
##
## Parametric coefficients:
##                            Estimate Std. Error t value Pr(>|t|)
## (Intercept)                0.365180   0.652083   0.560    0.576
## hormone_scr_ert_mean       0.001599   0.002453   0.652    0.514
## race.ethnicity.5levelBlack 0.049492   0.252355   0.196    0.845
## race.ethnicity.5levelMixed 0.266956   0.254911   1.047    0.295
## race.ethnicity.5levelOther 0.068762   0.296312   0.232    0.817
## race.ethnicity.5levelWhite 0.187339   0.235156   0.797    0.426
## interview_age              0.004566   0.005274   0.866    0.387
## demo_race_hispanic1        0.105591   0.115240   0.916    0.360
```

```
##
##
## R-sq.(adj) =  -0.000791
## lmer.REML = 9868.9  Scale est. = 2.3706    n = 2409

##                                  stdcoef      stdse
## X(Intercept)                 0.000000000 0.00000000
## Xhormone_scr_ert_mean        0.013981172 0.02144398
## Xrace.ethnicity.5levelBlack 0.009544096 0.04866454
## Xrace.ethnicity.5levelMixed 0.046395261 0.04430183
## Xrace.ethnicity.5levelOther 0.008028701 0.03459752
## Xrace.ethnicity.5levelWhite 0.047783046 0.05997921
## Xinterview_age               0.018180288 0.02099824
## Xdemo_race_hispanic1         0.022099986 0.02411957
```

Male participants

```
##
## Family: gaussian
## Link function: identity
##
## Formula:
## cbcl_scr_dsm5_depress_r ~ hormone_scr_ert_mean + race.ethnicity.5level +
##     interview_age + demo_race_hispanic
##
## Parametric coefficients:
##                              Estimate Std. Error t value Pr(>|t|)
## (Intercept)                  0.584252   0.716371   0.816    0.415
## hormone_scr_ert_mean         0.003873   0.002894   1.338    0.181
## race.ethnicity.5levelBlack -0.015288   0.309033  -0.049    0.961
## race.ethnicity.5levelMixed  0.122503   0.308904   0.397    0.692
## race.ethnicity.5levelOther -0.211947   0.347170  -0.610    0.542
## race.ethnicity.5levelWhite -0.045848   0.289020  -0.159    0.874
## interview_age                0.005727   0.005502   1.041    0.298
## demo_race_hispanic1          0.104462   0.125069   0.835    0.404
##
##
## R-sq.(adj) =  -3.42e-06
## lmer.REML =  11370  Scale est. = 2.4163    n = 2628

##                                   stdcoef      stdse
## X(Intercept)                  0.000000000 0.00000000
## Xhormone_scr_ert_mean         0.027139986 0.02027954
## Xrace.ethnicity.5levelBlack -0.002421329 0.04894377
## Xrace.ethnicity.5levelMixed  0.018823713 0.04746596
## Xrace.ethnicity.5levelOther -0.022442058 0.03676017
## Xrace.ethnicity.5levelWhite -0.010143182 0.06394185
## Xinterview_age                0.020522186 0.01971316
## Xdemo_race_hispanic1          0.019787604 0.02369111
```

1.13 Model: CBCL internalizing factor ~ Testosterone + PDS

Female participants

```
##
## Family: gaussian
## Link function: identity
##
## Formula:
## cbcl_scr_syn_internal_r ~ hormone_scr_ert_mean + PDS_score +
##     race.ethnicity.5level + interview_age + demo_race_hispanic
##
## Parametric coefficients:
##                            Estimate Std. Error t value Pr(>|t|)
## (Intercept)                0.342413   1.871973   0.183    0.855
## hormone_scr_ert_mean      -0.001808   0.007142  -0.253    0.800
## PDS_score                  0.858415   0.169573   5.062 4.46e-07 ***
## race.ethnicity.5levelBlack -0.448141   0.728585  -0.615    0.539
## race.ethnicity.5levelMixed  1.191085   0.727651   1.637    0.102
## race.ethnicity.5levelOther  0.282552   0.844386   0.335    0.738
## race.ethnicity.5levelWhite  0.995086   0.671704   1.481    0.139
## interview_age              0.020778   0.015411   1.348    0.178
## demo_race_hispanic1        0.163060   0.331259   0.492    0.623
## ---
## Signif. codes:  0 '***' 0.001 '**' 0.01 '*' 0.05 '.' 0.1 ' ' 1
##
##
## R-sq.(adj) =  0.0147
## lmer.REML =  14884  Scale est. = 17.835    n = 2409
```

```
##                                  stdcoef      stdse
## X(Intercept)                 0.000000000 0.00000000
## Xhormone_scr_ert_mean       -0.005485074 0.02167094
## XPDS_score                   0.114295098 0.02257808
## Xrace.ethnicity.5levelBlack -0.029990779 0.04875878
## Xrace.ethnicity.5levelMixed  0.071837186 0.04388638
## Xrace.ethnicity.5levelOther  0.011448981 0.03421439
## Xrace.ethnicity.5levelWhite  0.088080090 0.05945593
## Xinterview_age               0.028708084 0.02129285
## Xdemo_race_hispanic1         0.011843695 0.02406062
```

Male participants

```
##
## Family: gaussian
## Link function: identity
##
## Formula:
## cbcl_scr_syn_internal_r ~ hormone_scr_ert_mean + PDS_score +
##     race.ethnicity.5level + interview_age + demo_race_hispanic
##
## Parametric coefficients:
##                            Estimate Std. Error t value Pr(>|t|)
```

```
## (Intercept)                 3.601551   1.873001   1.923 0.054604 .
## hormone_scr_ert_mean        0.008530   0.007621   1.119 0.263134
## PDS_score                   0.722592   0.208138   3.472 0.000526 ***
## race.ethnicity.5levelBlack -0.920920   0.814194  -1.131 0.258124
## race.ethnicity.5levelMixed  0.265435   0.808196   0.328 0.742613
## race.ethnicity.5levelOther -0.774644   0.907628  -0.853 0.393470
## race.ethnicity.5levelWhite -0.011174   0.756320  -0.015 0.988213
## interview_age               0.002384   0.014484   0.165 0.869270
## demo_race_hispanic1         0.455845   0.330651   1.379 0.168127
## ---
## Signif. codes:  0 '***' 0.001 '**' 0.01 '*' 0.05 '.' 0.1 ' ' 1
##
##
## R-sq.(adj) =  0.00487
## lmer.REML =  16401  Scale est. = 15.567    n = 2628
```

```
##                                  stdcoef      stdse
## X(Intercept)                 0.0000000000 0.00000000
## Xhormone_scr_ert_mean        0.0227072479 0.02028781
## XPDS_score                   0.0708975056 0.02042158
## Xrace.ethnicity.5levelBlack -0.0554143164 0.04899231
## Xrace.ethnicity.5levelMixed  0.0154962302 0.04718284
## Xrace.ethnicity.5levelOther -0.0311634891 0.03651335
## Xrace.ethnicity.5levelWhite -0.0009392334 0.06357280
## Xinterview_age               0.0032456437 0.01971814
## Xdemo_race_hispanic1         0.0328064855 0.02379648
```

1.14 Model: CBCL internalizing factor ~ Testosterone + Pubertal category

Female participants

```
##
## Family: gaussian
## Link function: identity
##
## Formula:
## cbcl_scr_syn_internal_r ~ hormone_scr_ert_mean + pds_p_ss_category +
##     race.ethnicity.5level + interview_age + demo_race_hispanic
##
## Parametric coefficients:
##                             Estimate Std. Error t value Pr(>|t|)
## (Intercept)                 0.8557681  1.9228693   0.445   0.6563
## hormone_scr_ert_mean        0.0003425  0.0071332   0.048   0.9617
## pds_p_ss_categoryEarly      0.3933989  0.3005232   1.309   0.1906
## pds_p_ss_categoryLate       0.8687929  0.7326478   1.186   0.2358
## pds_p_ss_categoryMid        1.1166251  0.2832008   3.943 8.28e-05 ***
## race.ethnicity.5levelBlack -0.3133195  0.7296392  -0.429   0.6677
## race.ethnicity.5levelMixed  1.2366700  0.7289531   1.697   0.0899 .
## race.ethnicity.5levelOther  0.3206729  0.8459695   0.379   0.7047
## race.ethnicity.5levelWhite  1.0239192  0.6728981   1.522   0.1282
## interview_age               0.0225179  0.0157527   1.429   0.1530
## demo_race_hispanic1         0.1015637  0.3326217   0.305   0.7601
## ---
```

```
## Signif. codes:  0 '***' 0.001 '**' 0.01 '*' 0.05 '.' 0.1 ' ' 1
##
##
## R-sq.(adj) =  0.0109
## lmer.REML =  14892  Scale est. = 18.205    n = 2409
```

```
##                                  stdcoef      stdse
## X(Intercept)                 0.000000000 0.00000000
## Xhormone_scr_ert_mean        0.001039366 0.02164450
## Xpds_p_ss_categoryEarly      0.030669604 0.02342896
## Xpds_p_ss_categoryLate       0.025422800 0.02143889
## Xpds_p_ss_categoryMid        0.101942809 0.02585495
## Xrace.ethnicity.5levelBlack -0.020968151 0.04882934
## Xrace.ethnicity.5levelMixed  0.074586528 0.04396491
## Xrace.ethnicity.5levelOther  0.012993614 0.03427855
## Xrace.ethnicity.5levelWhite  0.090632263 0.05956161
## Xinterview_age               0.031111461 0.02176435
## Xdemo_race_hispanic1         0.007376971 0.02415962
```

Male participants

```
##
## Family: gaussian
## Link function: identity
##
## Formula:
## cbcl_scr_syn_internal_r ~ hormone_scr_ert_mean + pds_p_ss_category +
##     race.ethnicity.5level + interview_age + demo_race_hispanic
##
## Parametric coefficients:
##                            Estimate Std. Error t value Pr(>|t|)
## (Intercept)                3.948940   1.878350   2.102   0.0356 *
## hormone_scr_ert_mean       0.009780   0.007605   1.286   0.1986
## pds_p_ss_categoryEarly     0.584451   0.266802   2.191   0.0286 *
## pds_p_ss_categoryLate      1.751036   1.766389   0.991   0.3216
## pds_p_ss_categoryMid       1.087830   0.488954   2.225   0.0262 *
## race.ethnicity.5levelBlack -0.846385   0.814467  -1.039   0.2988
## race.ethnicity.5levelMixed  0.331370   0.809462   0.409   0.6823
## race.ethnicity.5levelOther -0.750826   0.908673  -0.826   0.4087
## race.ethnicity.5levelWhite  0.074787   0.757914   0.099   0.9214
## interview_age              0.004933   0.014448   0.341   0.7328
## demo_race_hispanic1        0.435394   0.331672   1.313   0.1894
## ---
## Signif. codes:  0 '***' 0.001 '**' 0.01 '*' 0.05 '.' 0.1 ' ' 1
##
##
## R-sq.(adj) =  0.00286
## lmer.REML =  16401  Scale est. = 15.602    n = 2628
```

```
##                                  stdcoef      stdse
## X(Intercept)                 0.000000000 0.00000000
## Xhormone_scr_ert_mean        0.026036280 0.02024654
## Xpds_p_ss_categoryEarly      0.043938067 0.02005777
```

```
## Xpds_p_ss_categoryLate       0.019377781 0.01954768
## Xpds_p_ss_categoryMid        0.045360899 0.02038863
## Xrace.ethnicity.5levelBlack -0.050929352 0.04900877
## Xrace.ethnicity.5levelMixed  0.019345540 0.04725674
## Xrace.ethnicity.5levelOther -0.030205319 0.03655539
## Xrace.ethnicity.5levelWhite  0.006286281 0.06370685
## Xinterview_age               0.006715086 0.01966883
## Xdemo_race_hispanic1         0.031334668 0.02386994
```

1.15 Model: CBCL Anxious-Depressed ~ Testosterone + PDS

Female participants

```
##
## Family: gaussian
## Link function: identity
##
## Formula:
## cbcl_scr_syn_anxdep_r ~ hormone_scr_ert_mean + PDS_score + race.ethnicity.5level +
##     interview_age + demo_race_hispanic
##
## Parametric coefficients:
##                              Estimate Std. Error t value Pr(>|t|)
## (Intercept)                  0.676640   1.047992   0.646 0.518566
## hormone_scr_ert_mean        -0.002159   0.004003  -0.539 0.589733
## PDS_score                    0.362799   0.094795   3.827 0.000133 ***
## race.ethnicity.5levelBlack -0.330779   0.404838  -0.817 0.413973
## race.ethnicity.5levelMixed  0.756457   0.404897   1.868 0.061846 .
## race.ethnicity.5levelOther  0.179155   0.470212   0.381 0.703230
## race.ethnicity.5levelWhite  0.571167   0.373553   1.529 0.126393
## interview_age                0.007008   0.008648   0.810 0.417827
## demo_race_hispanic1          0.037785   0.183185   0.206 0.836600
## ---
## Signif. codes:  0 '***' 0.001 '**' 0.01 '*' 0.05 '.' 0.1 ' ' 1
##
##
## R-sq.(adj) =  0.0135
## lmer.REML =  12105  Scale est. = 7.04      n = 2409
```

```
##                                   stdcoef      stdse
## X(Intercept)                 0.000000000 0.00000000
## Xhormone_scr_ert_mean       -0.011740483 0.02177005
## XPDS_score                   0.086579092 0.02262213
## Xrace.ethnicity.5levelBlack -0.039675953 0.04855914
## Xrace.ethnicity.5levelMixed  0.081772589 0.04376916
## Xrace.ethnicity.5levelOther  0.013011072 0.03414898
## Xrace.ethnicity.5levelWhite  0.090614354 0.05926332
## Xinterview_age               0.017353886 0.02141566
## Xdemo_race_hispanic1         0.004918995 0.02384771
```

Male participants

```
##
```

```
## Family: gaussian
## Link function: identity
##
## Formula:
## cbcl_scr_syn_anxdep_r ~ hormone_scr_ert_mean + PDS_score + race.ethnicity.5level +
##     interview_age + demo_race_hispanic
##
## Parametric coefficients:
##                             Estimate Std. Error t value Pr(>|t|)
## (Intercept)                 2.990092   1.044140   2.864  0.00422 **
## hormone_scr_ert_mean        0.004654   0.004237   1.098  0.27211
## PDS_score                   0.318842   0.115988   2.749  0.00602 **
## race.ethnicity.5levelBlack -0.420398   0.450649  -0.933  0.35097
## race.ethnicity.5levelMixed  0.259189   0.447607   0.579  0.56260
## race.ethnicity.5levelOther -0.149350   0.503841  -0.296  0.76693
## race.ethnicity.5levelWhite  0.292767   0.418862   0.699  0.48464
## interview_age              -0.010489   0.008095  -1.296  0.19518
## demo_race_hispanic1         0.180108   0.181839   0.990  0.32203
## ---
## Signif. codes:  0 '***' 0.001 '**' 0.01 '*' 0.05 '.' 0.1 ' ' 1
##
##
## R-sq.(adj) =  0.00514
## lmer.REML =  13340  Scale est. = 6.3863    n = 2628
```

```
##                                 stdcoef      stdse
## X(Intercept)                 0.00000000 0.00000000
## Xhormone_scr_ert_mean        0.02238912 0.02038267
## XPDS_score                   0.05652857 0.02056393
## Xrace.ethnicity.5levelBlack -0.04571053 0.04899974
## Xrace.ethnicity.5levelMixed  0.02734255 0.04721928
## Xrace.ethnicity.5levelOther -0.01085685 0.03662631
## Xrace.ethnicity.5levelWhite  0.04446756 0.06361982
## Xinterview_age              -0.02580229 0.01991299
## Xdemo_race_hispanic1         0.02342232 0.02364749
```

1.16 Model: CBCL Anxious-Depressed ~ Testosterone + Pubertal category

Female participants

```
##
## Family: gaussian
## Link function: identity
##
## Formula:
## cbcl_scr_syn_anxdep_r ~ hormone_scr_ert_mean + pds_p_ss_category +
##     race.ethnicity.5level + interview_age + demo_race_hispanic
##
## Parametric coefficients:
##                              Estimate Std. Error t value Pr(>|t|)
## (Intercept)                 0.7791112  1.0755659   0.724   0.4689
## hormone_scr_ert_mean       -0.0008758  0.0039961  -0.219   0.8266
## pds_p_ss_categoryEarly      0.2297358  0.1685985   1.363   0.1731
```

```
## pds_p_ss_categoryLate       0.1807508  0.4105624   0.440   0.6598
## pds_p_ss_categoryMid        0.4386130  0.1584967   2.767   0.0057 **
## race.ethnicity.5levelBlack -0.2448288  0.4053385  -0.604   0.5459
## race.ethnicity.5levelMixed  0.7824490  0.4055146   1.930   0.0538 .
## race.ethnicity.5levelOther  0.1977192  0.4709500   0.420   0.6746
## race.ethnicity.5levelWhite  0.5875329  0.3741000   1.571   0.1164
## interview_age               0.0085612  0.0088298   0.970   0.3324
## demo_race_hispanic1         0.0180341  0.1839019   0.098   0.9219
## ---
## Signif. codes:  0 '***' 0.001 '**' 0.01 '*' 0.05 '.' 0.1 ' ' 1
##
##
## R-sq.(adj) =  0.0106
## lmer.REML =  12113  Scale est. = 7.0992    n = 2409
```

```
##                                  stdcoef      stdse
## X(Intercept)                 0.000000000 0.00000000
## Xhormone_scr_ert_mean       -0.004762800 0.02173286
## Xpds_p_ss_categoryEarly      0.032101135 0.02355838
## Xpds_p_ss_categoryLate       0.009479907 0.02153293
## Xpds_p_ss_categoryMid        0.071770738 0.02593500
## Xrace.ethnicity.5levelBlack -0.029366487 0.04861914
## Xrace.ethnicity.5levelMixed  0.084582275 0.04383589
## Xrace.ethnicity.5levelOther  0.014359298 0.03420261
## Xrace.ethnicity.5levelWhite  0.093210770 0.05935012
## Xinterview_age               0.021200376 0.02186544
## Xdemo_race_hispanic1         0.002347750 0.02394101
```

Male participants

```
##
## Family: gaussian
## Link function: identity
##
## Formula:
## cbcl_scr_syn_anxdep_r ~ hormone_scr_ert_mean + pds_p_ss_category +
##     race.ethnicity.5level + interview_age + demo_race_hispanic
##
## Parametric coefficients:
##                             Estimate Std. Error t value Pr(>|t|)
## (Intercept)                 3.123291   1.047126   2.983  0.00288 **
## hormone_scr_ert_mean        0.005308   0.004229   1.255  0.20961
## pds_p_ss_categoryEarly      0.221117   0.148706   1.487  0.13715
## pds_p_ss_categoryLate       0.533178   0.980631   0.544  0.58669
## pds_p_ss_categoryMid        0.420430   0.272652   1.542  0.12319
## race.ethnicity.5levelBlack -0.371849   0.450855  -0.825  0.40958
## race.ethnicity.5levelMixed  0.285879   0.448350   0.638  0.52377
## race.ethnicity.5levelOther -0.137199   0.504456  -0.272  0.78566
## race.ethnicity.5levelWhite  0.325158   0.419795   0.775  0.43867
## interview_age              -0.009104   0.008075  -1.127  0.25966
## demo_race_hispanic1         0.174477   0.182433   0.956  0.33896
## ---
## Signif. codes:  0 '***' 0.001 '**' 0.01 '*' 0.05 '.' 0.1 ' ' 1
```

```
##
##
## R-sq.(adj) =  0.00299
## lmer.REML =  13342  Scale est. = 6.3973    n = 2628
```

```
##                                 stdcoef      stdse
## X(Intercept)                 0.000000000 0.00000000
## Xhormone_scr_ert_mean        0.025532655 0.02034563
## Xpds_p_ss_categoryEarly      0.030037945 0.02020120
## Xpds_p_ss_categoryLate       0.010661950 0.01960967
## Xpds_p_ss_categoryMid        0.031678892 0.02054398
## Xrace.ethnicity.5levelBlack -0.040431698 0.04902215
## Xrace.ethnicity.5levelMixed  0.030158117 0.04729767
## Xrace.ethnicity.5levelOther -0.009973597 0.03667097
## Xrace.ethnicity.5levelWhite  0.049387357 0.06376149
## Xinterview_age              -0.022395419 0.01986399
## Xdemo_race_hispanic1         0.022690107 0.02372475
```

1.17 Model: CBCL Withdrawn-Depressed ~ Testosterone + PDS

Female participants

```
##
## Family: gaussian
## Link function: identity
##
## Formula:
## cbcl_scr_syn_withdep_r ~ hormone_scr_ert_mean + PDS_score + race.ethnicity.5level +
##     interview_age + demo_race_hispanic
##
## Parametric coefficients:
##                             Estimate Std. Error t value Pr(>|t|)
## (Intercept)                 0.264555   0.563083   0.470    0.639
## hormone_scr_ert_mean        0.002833   0.002147   1.320    0.187
## PDS_score                   0.224695   0.050753   4.427 9.97e-06 ***
## race.ethnicity.5levelBlack -0.256632   0.216690  -1.184    0.236
## race.ethnicity.5levelMixed -0.008861   0.216562  -0.041    0.967
## race.ethnicity.5levelOther -0.085080   0.251403  -0.338    0.735
## race.ethnicity.5levelWhite -0.076689   0.199982  -0.383    0.701
## interview_age               0.002215   0.004646   0.477    0.634
## demo_race_hispanic1         0.157482   0.097957   1.608    0.108
## ---
## Signif. codes:  0 '***' 0.001 '**' 0.01 '*' 0.05 '.' 0.1 ' ' 1
##
##
## R-sq.(adj) =  0.0106
## lmer.REML =   9123  Scale est. = 2.4126    n = 2409
```

```
##                                stdcoef      stdse
## X(Intercept)                 0.00000000 0.00000000
## Xhormone_scr_ert_mean        0.02877887 0.02180940
## XPDS_score                   0.10015423 0.02262238
## Xrace.ethnicity.5levelBlack -0.05749500 0.04854638
```

```
## Xrace.ethnicity.5levelMixed -0.00178920 0.04372541
## Xrace.ethnicity.5levelOther -0.01154097 0.03410240
## Xrace.ethnicity.5levelWhite -0.02272450 0.05925907
## Xinterview_age               0.01024400 0.02148717
## Xdemo_race_hispanic1         0.03829283 0.02381879
```

Male participants

```
##
## Family: gaussian
## Link function: identity
##
## Formula:
## cbcl_scr_syn_withdep_r ~ hormone_scr_ert_mean + PDS_score + race.ethnicity.5level +
##     interview_age + demo_race_hispanic
##
## Parametric coefficients:
##                            Estimate Std. Error t value Pr(>|t|)
## (Intercept)               -0.079207   0.622787  -0.127   0.8988
## hormone_scr_ert_mean       0.004486   0.002528   1.775   0.0760 .
## PDS_score                  0.173144   0.069352   2.497   0.0126 *
## race.ethnicity.5levelBlack -0.213651   0.269371  -0.793   0.4278
## race.ethnicity.5levelMixed -0.030230   0.267826  -0.113   0.9101
## race.ethnicity.5levelOther -0.156291   0.301378  -0.519   0.6041
## race.ethnicity.5levelWhite -0.216493   0.250526  -0.864   0.3876
## interview_age              0.008521   0.004831   1.764   0.0779 .
## demo_race_hispanic1        0.064799   0.107499   0.603   0.5467
## ---
## Signif. codes:  0 '***' 0.001 '**' 0.01 '*' 0.05 '.' 0.1 ' ' 1
##
##
## R-sq.(adj) =  0.00437
## lmer.REML =  10642  Scale est. = 2.0749    n = 2628
```

```
##                                  stdcoef      stdse
## X(Intercept)                 0.000000000 0.00000000
## Xhormone_scr_ert_mean        0.036143616 0.02036342
## XPDS_score                   0.051411554 0.02059277
## Xrace.ethnicity.5levelBlack -0.038906454 0.04905326
## Xrace.ethnicity.5levelMixed -0.005341048 0.04731920
## Xrace.ethnicity.5levelOther -0.019028074 0.03669204
## Xrace.ethnicity.5levelWhite -0.055071465 0.06372869
## Xinterview_age               0.035105272 0.01990177
## Xdemo_race_hispanic1         0.014113375 0.02341326
```

1.18 Model: CBCL Withdrawn-Depressed ~ Testosterone + Pubertal category

Female participants

```
##
## Family: gaussian
## Link function: identity
```

```
##
## Formula:
## cbcl_scr_syn_withdep_r ~ hormone_scr_ert_mean + pds_p_ss_category +
##     race.ethnicity.5level + interview_age + demo_race_hispanic
##
## Parametric coefficients:
##                             Estimate Std. Error t value Pr(>|t|)
## (Intercept)                 0.435598   0.577770   0.754  0.45097
## hormone_scr_ert_mean        0.003353   0.002144   1.564  0.11788
## pds_p_ss_categoryEarly      0.047294   0.090456   0.523  0.60113
## pds_p_ss_categoryLate       0.402736   0.219935   1.831  0.06720 .
## pds_p_ss_categoryMid        0.275000   0.084897   3.239  0.00122 **
## race.ethnicity.5levelBlack -0.231080   0.216989  -1.065  0.28701
## race.ethnicity.5levelMixed  0.003088   0.216906   0.014  0.98864
## race.ethnicity.5levelOther -0.073623   0.251809  -0.292  0.77002
## race.ethnicity.5levelWhite -0.067910   0.200279  -0.339  0.73458
## interview_age               0.002517   0.004742   0.531  0.59564
## demo_race_hispanic1         0.138990   0.098341   1.413  0.15768
## ---
## Signif. codes:  0 '***' 0.001 '**' 0.01 '*' 0.05 '.' 0.1 ' ' 1
##
##
## R-sq.(adj) =  0.00763
## lmer.REML =   9133  Scale est. = 2.4228    n = 2409
```

```
##                                  stdcoef      stdse
## X(Intercept)                 0.0000000000 0.00000000
## Xhormone_scr_ert_mean        0.0340613151 0.02177411
## Xpds_p_ss_categoryEarly      0.0123432124 0.02360803
## Xpds_p_ss_categoryLate       0.0394524490 0.02154508
## Xpds_p_ss_categoryMid        0.0840480748 0.02594712
## Xrace.ethnicity.5levelBlack -0.0517703614 0.04861336
## Xrace.ethnicity.5levelMixed  0.0006235848 0.04379505
## Xrace.ethnicity.5levelOther -0.0099868739 0.03415742
## Xrace.ethnicity.5levelWhite -0.0201231413 0.05934696
## Xinterview_age               0.0116403786 0.02193215
## Xdemo_race_hispanic1         0.0337962441 0.02391227
```

Male participants

```
##
## Family: gaussian
## Link function: identity
##
## Formula:
## cbcl_scr_syn_withdep_r ~ hormone_scr_ert_mean + pds_p_ss_category +
##     race.ethnicity.5level + interview_age + demo_race_hispanic
##
## Parametric coefficients:
##                             Estimate Std. Error t value Pr(>|t|)
## (Intercept)                 0.002986   0.624066   0.005   0.9962
## hormone_scr_ert_mean        0.004787   0.002521   1.899   0.0576 .
## pds_p_ss_categoryEarly      0.127215   0.088825   1.432   0.1522
```

```
## pds_p_ss_categoryLate       0.118407   0.586546   0.202   0.8400
## pds_p_ss_categoryMid        0.372296   0.162909   2.285   0.0224 *
## race.ethnicity.5levelBlack -0.198817   0.269245  -0.738   0.4603
## race.ethnicity.5levelMixed -0.006389   0.268040  -0.024   0.9810
## race.ethnicity.5levelOther -0.145859   0.301509  -0.484   0.6286
## race.ethnicity.5levelWhite -0.187780   0.250862  -0.749   0.4542
## interview_age               0.009077   0.004816   1.885   0.0596 .
## demo_race_hispanic1         0.055606   0.107682   0.516   0.6056
## ---
## Signif. codes:  0 '***' 0.001 '**' 0.01 '*' 0.05 '.' 0.1 ' ' 1
##
##
## R-sq.(adj) =  0.00392
## lmer.REML =  10642  Scale est. = 2.0842    n = 2628
```

```
##                                  stdcoef      stdse
## X(Intercept)                 0.000000000 0.00000000
## Xhormone_scr_ert_mean        0.038570552 0.02030809
## Xpds_p_ss_categoryEarly      0.028943372 0.02020909
## Xpds_p_ss_categoryLate       0.003965544 0.01964390
## Xpds_p_ss_categoryMid        0.046981426 0.02055801
## Xrace.ethnicity.5levelBlack -0.036205086 0.04903038
## Xrace.ethnicity.5levelMixed -0.001128826 0.04735691
## Xrace.ethnicity.5levelOther -0.017757995 0.03670804
## Xrace.ethnicity.5levelWhite -0.047767508 0.06381431
## Xinterview_age               0.037394131 0.01983951
## Xdemo_race_hispanic1         0.012110972 0.02345319
```

1.19 Model: CBCL Depressed DSM-5 ~ Testosterone + PDS

Female participants

```
##
## Family: gaussian
## Link function: identity
##
## Formula:
## cbcl_scr_dsm5_depress_r ~ hormone_scr_ert_mean + PDS_score +
##     race.ethnicity.5level + interview_age + demo_race_hispanic
##
## Parametric coefficients:
##                              Estimate Std. Error t value Pr(>|t|)
## (Intercept)                 0.6460355  0.6558347   0.985 0.324695
## hormone_scr_ert_mean       -0.0002827  0.0025083  -0.113 0.910280
## PDS_score                   0.2031579  0.0595351   3.412 0.000655 ***
## race.ethnicity.5levelBlack -0.0729058  0.2543140  -0.287 0.774385
## race.ethnicity.5levelMixed  0.2254904  0.2546070   0.886 0.375900
## race.ethnicity.5levelOther  0.0420331  0.2957283   0.142 0.886986
## race.ethnicity.5levelWhite  0.1675795  0.2346831   0.714 0.475255
## interview_age               0.0002325  0.0054138   0.043 0.965748
## demo_race_hispanic1         0.1023451  0.1149749   0.890 0.373474
## ---
## Signif. codes:  0 '***' 0.001 '**' 0.01 '*' 0.05 '.' 0.1 ' ' 1
```

```
##
##
## R-sq.(adj) =  0.00332
## lmer.REML = 9861.1  Scale est. = 2.3708    n = 2409
```

```
##                                  stdcoef      stdse
## X(Intercept)                 0.0000000000 0.00000000
## Xhormone_scr_ert_mean       -0.0024716722 0.02193189
## XPDS_score                   0.0779455840 0.02284184
## Xrace.ethnicity.5levelBlack -0.0140592443 0.04904224
## Xrace.ethnicity.5levelMixed  0.0391888040 0.04424908
## Xrace.ethnicity.5levelOther  0.0049077939 0.03452931
## Xrace.ethnicity.5levelWhite  0.0427430404 0.05985856
## Xinterview_age               0.0009256356 0.02155368
## Xdemo_race_hispanic1         0.0214207060 0.02406411
```

Male participants

```
##
## Family: gaussian
## Link function: identity
##
## Formula:
## cbcl_scr_dsm5_depress_r ~ hormone_scr_ert_mean + PDS_score +
##     race.ethnicity.5level + interview_age + demo_race_hispanic
##
## Parametric coefficients:
##                             Estimate Std. Error t value Pr(>|t|)
## (Intercept)                 0.571436   0.715750   0.798    0.425
## hormone_scr_ert_mean        0.003161   0.002910   1.086    0.277
## PDS_score                   0.175141   0.079670   2.198    0.028 *
## race.ethnicity.5levelBlack -0.089073   0.310714  -0.287    0.774
## race.ethnicity.5levelMixed  0.115835   0.308718   0.375    0.708
## race.ethnicity.5levelOther -0.220547   0.346959  -0.636    0.525
## race.ethnicity.5levelWhite -0.045009   0.288819  -0.156    0.876
## interview_age               0.004156   0.005544   0.750    0.454
## demo_race_hispanic1         0.094639   0.124952   0.757    0.449
## ---
## Signif. codes:  0 '***' 0.001 '**' 0.01 '*' 0.05 '.' 0.1 ' ' 1
##
##
## R-sq.(adj) =  0.00146
## lmer.REML =  11369  Scale est. = 2.3989    n = 2628
```

```
##                                 stdcoef      stdse
## X(Intercept)                 0.000000000 0.00000000
## Xhormone_scr_ert_mean        0.022150446 0.02038962
## XPDS_score                   0.045229008 0.02057436
## Xrace.ethnicity.5levelBlack -0.014107184 0.04920999
## Xrace.ethnicity.5levelMixed  0.017799124 0.04743737
## Xrace.ethnicity.5levelOther -0.023352706 0.03673789
## Xrace.ethnicity.5levelWhite -0.009957701 0.06389749
## Xinterview_age               0.014890191 0.01986382
## Xdemo_race_hispanic1         0.017926843 0.02366887
```

1.20 Model: CBCL Depressed DSM-5 ~ Testosterone + Pubertal category

Female participants

```
##
## Family: gaussian
## Link function: identity
##
## Formula:
## cbcl_scr_dsm5_depress_r ~ hormone_scr_ert_mean + pds_p_ss_category +
##     race.ethnicity.5level + interview_age + demo_race_hispanic
##
## Parametric coefficients:
##                             Estimate Std. Error t value Pr(>|t|)
## (Intercept)                0.8106598  0.6728012   1.205  0.22836
## hormone_scr_ert_mean       0.0001381  0.0025021   0.055  0.95599
## pds_p_ss_categoryEarly     0.0492938  0.1055779   0.467  0.64062
## pds_p_ss_categoryLate      0.3461948  0.2572564   1.346  0.17852
## pds_p_ss_categoryMid       0.2601191  0.0993877   2.617  0.00892 **
## race.ethnicity.5levelBlack -0.0509616  0.2543593  -0.200  0.84122
## race.ethnicity.5levelMixed  0.2358067  0.2547330   0.926  0.35469
## race.ethnicity.5levelOther  0.0506706  0.2958991   0.171  0.86405
## race.ethnicity.5levelWhite  0.1747582  0.2347990   0.744  0.45677
## interview_age              0.0003926  0.0055252   0.071  0.94335
## demo_race_hispanic1        0.0859678  0.1153212   0.745  0.45606
## ---
## Signif. codes:  0 '***' 0.001 '**' 0.01 '*' 0.05 '.' 0.1 ' ' 1
##
##
## R-sq.(adj) =  0.00162
## lmer.REML = 9867.4  Scale est. = 2.4144    n = 2409
```

```
##                                   stdcoef      stdse
## X(Intercept)                  0.000000000 0.00000000
## Xhormone_scr_ert_mean         0.001207347 0.02187747
## Xpds_p_ss_categoryEarly       0.011073755 0.02371787
## Xpds_p_ss_categoryLate        0.029191408 0.02169205
## Xpds_p_ss_categoryMid         0.068430362 0.02614623
## Xrace.ethnicity.5levelBlack -0.009827497 0.04905098
## Xrace.ethnicity.5levelMixed  0.040981716 0.04427098
## Xrace.ethnicity.5levelOther  0.005916315 0.03454926
## Xrace.ethnicity.5levelWhite  0.044574051 0.05988814
## Xinterview_age                0.001563179 0.02199701
## Xdemo_race_hispanic1          0.017992972 0.02413660
```

Male participants

```
##
## Family: gaussian
## Link function: identity
##
## Formula:
## cbcl_scr_dsm5_depress_r ~ hormone_scr_ert_mean + pds_p_ss_category +
```

```
##     race.ethnicity.5level + interview_age + demo_race_hispanic
##
## Parametric coefficients:
##                            Estimate Std. Error t value Pr(>|t|)
## (Intercept)                0.689735   0.717064   0.962   0.3362
## hormone_scr_ert_mean       0.003294   0.002901   1.135   0.2563
## pds_p_ss_categoryEarly     0.237818   0.102015   2.331   0.0198 *
## pds_p_ss_categoryLate      0.714641   0.674953   1.059   0.2898
## pds_p_ss_categoryMid       0.170315   0.187023   0.911   0.3626
## race.ethnicity.5levelBlack -0.078464   0.310538  -0.253   0.8005
## race.ethnicity.5levelMixed  0.122465   0.308923   0.396   0.6918
## race.ethnicity.5levelOther -0.219424   0.347043  -0.632   0.5273
## race.ethnicity.5levelWhite -0.027166   0.289168  -0.094   0.9252
## interview_age              0.004416   0.005525   0.799   0.4242
## demo_race_hispanic1        0.091051   0.125217   0.727   0.4672
## ---
## Signif. codes:  0 '***' 0.001 '**' 0.01 '*' 0.05 '.' 0.1 ' ' 1
##
##
## R-sq.(adj) =  0.00109
## lmer.REML =  11367  Scale est. = 2.3933    n = 2628
```

```
##                                  stdcoef      stdse
## X(Intercept)                 0.000000000 0.00000000
## Xhormone_scr_ert_mean        0.023081528 0.02032942
## Xpds_p_ss_categoryEarly      0.047057475 0.02018586
## Xpds_p_ss_categoryLate       0.020815587 0.01965960
## Xpds_p_ss_categoryMid        0.018692446 0.02052616
## Xrace.ethnicity.5levelBlack -0.012426899 0.04918215
## Xrace.ethnicity.5levelMixed  0.018817879 0.04746887
## Xrace.ethnicity.5levelOther -0.023233793 0.03674676
## Xrace.ethnicity.5levelWhite -0.006010075 0.06397471
## Xinterview_age               0.015822566 0.01979495
## Xdemo_race_hispanic1         0.017247156 0.02371908
```

2—Reward~Puberty—

2.1 Model: BIS-BAS-RR ~ PDS

Female participants

```
##
## Family: gaussian
## Link function: identity
##
## Formula:
## bisbas_ss_basm_rr_z ~ PDS_score + interview_age
##
## Parametric coefficients:
##                Estimate Std. Error t value Pr(>|t|)
## (Intercept)    0.424883   0.314581   1.351  0.17693
## PDS_score      0.075275   0.028023   2.686  0.00727 **
## interview_age -0.005048   0.002715  -1.859  0.06310 .
## ---
## Signif. codes:  0 '***' 0.001 '**' 0.01 '*' 0.05 '.' 0.1 ' ' 1
##
##
## R-sq.(adj) =  0.00417
## lmer.REML = 7608.3  Scale est. = 0.75478   n = 2683
```

```
##                    stdcoef      stdse
## X(Intercept)    0.00000000 0.00000000
## XPDS_score      0.05471457 0.02036890
## Xinterview_age -0.03751186 0.02017537
```

Male participants

```
##
## Family: gaussian
## Link function: identity
##
## Formula:
## bisbas_ss_basm_rr_z ~ PDS_score + interview_age
##
## Parametric coefficients:
##                Estimate Std. Error t value Pr(>|t|)
## (Intercept)    0.113914   0.286158   0.398   0.6906
## PDS_score      0.069981   0.032783   2.135   0.0329 *
## interview_age -0.001217   0.002418  -0.503   0.6147
## ---
## Signif. codes:  0 '***' 0.001 '**' 0.01 '*' 0.05 '.' 0.1 ' ' 1
##
##
## R-sq.(adj) =  0.00134
## lmer.REML = 8001.9  Scale est. = 0.72946   n = 2893
```

```
##                     stdcoef      stdse
```

```
## X(Intercept)    0.000000000 0.00000000
## XPDS_score      0.040327184 0.01889153
## Xinterview_age -0.009502474 0.01887306
```

2.2 Model : Reaction Time ~ PDS

Female participants

```
##
## Family: gaussian
## Link function: identity
##
## Formula:
## rt_diff_large_neutral_z ~ PDS_score + interview_age
##
## Parametric coefficients:
##                Estimate Std. Error t value Pr(>|t|)
## (Intercept)   -0.632648   0.316922  -1.996   0.0460 *
## PDS_score      0.021223   0.028471   0.745   0.4561
## interview_age  0.005283   0.002744   1.925   0.0544 .
## ---
## Signif. codes:  0 '***' 0.001 '**' 0.01 '*' 0.05 '.' 0.1 ' ' 1
##
##
## R-sq.(adj) =  0.00148
## lmer.REML = 6029.4  Scale est. = 0.75407   n = 2239
```

```
##                   stdcoef      stdse
## X(Intercept)   0.00000000 0.00000000
## XPDS_score     0.01641790 0.02202495
## Xinterview_age 0.04232813 0.02198917
```

```
##
## Family: gaussian
## Link function: identity
##
## Formula:
## rt_diff_large_small_z ~ PDS_score + interview_age
##
## Parametric coefficients:
##                 Estimate Std. Error t value Pr(>|t|)
## (Intercept)   -5.279e-01  3.315e-01  -1.592    0.111
## PDS_score     -1.774e-06  2.985e-02   0.000    1.000
## interview_age  4.523e-03  2.868e-03   1.577    0.115
##
##
## R-sq.(adj) =  0.000326
## lmer.REML = 6207.2  Scale est. = 0.83565   n = 2239
```

```
##                      stdcoef      stdse
## X(Intercept)    0.000000e+00 0.00000000
## XPDS_score     -1.319351e-06 0.02220433
## Xinterview_age  3.484188e-02 0.02209669
```

Male participants

```
##
## Family: gaussian
## Link function: identity
##
## Formula:
## rt_diff_large_neutral_z ~ PDS_score + interview_age
##
## Parametric coefficients:
##                Estimate Std. Error t value Pr(>|t|)
## (Intercept)   -0.592862   0.301862  -1.964   0.0496 *
## PDS_score     -0.054721   0.035667  -1.534   0.1251
## interview_age  0.005403   0.002552   2.117   0.0344 *
## ---
## Signif. codes:  0 '***' 0.001 '**' 0.01 '*' 0.05 '.' 0.1 ' ' 1
##
##
## R-sq.(adj) =  0.00176
## lmer.REML = 6169.8  Scale est. = 0.84509   n = 2304
```

```
##                    stdcoef      stdse
## X(Intercept)    0.00000000 0.00000000
## XPDS_score     -0.03231221 0.02106137
## Xinterview_age  0.04458391 0.02106322
```

```
##
## Family: gaussian
## Link function: identity
##
## Formula:
## rt_diff_large_small_z ~ PDS_score + interview_age
##
## Parametric coefficients:
##                 Estimate Std. Error t value Pr(>|t|)
## (Intercept)    0.0325075  0.2987357   0.109    0.913
## PDS_score     -0.0140450  0.0353043  -0.398    0.691
## interview_age -0.0002824  0.0025262  -0.112    0.911
##
##
## R-sq.(adj) =  -0.000788
## lmer.REML = 6126.5  Scale est. = 0.82974   n = 2304
```

```
##                     stdcoef      stdse
## X(Intercept)    0.000000000 0.00000000
## XPDS_score     -0.008382513 0.02107077
## Xinterview_age -0.002355721 0.02107077
```

2.3 Model: Caudate Anticipation ~ PDS

Female participants

```
##
```

```
## Family: gaussian
## Link function: identity
##
## Formula:
## caudate_rvsn_ant_z ~ PDS_score + interview_age
##
## Parametric coefficients:
##                Estimate Std. Error t value Pr(>|t|)
## (Intercept)   -0.354322   0.315627  -1.123    0.262
## PDS_score     -0.027115   0.028254  -0.960    0.337
## interview_age  0.003233   0.002729   1.185    0.236
##
##
## R-sq.(adj) =  0.000154
## lmer.REML = 5413.7  Scale est. = 0.73298   n = 2071
```

```
##                    stdcoef      stdse
## X(Intercept)    0.00000000 0.00000000
## XPDS_score     -0.02214033 0.02306980
## Xinterview_age  0.02721126 0.02296476
```

Male participants

```
##
## Family: gaussian
## Link function: identity
##
## Formula:
## caudate_rvsn_ant_z ~ PDS_score + interview_age
##
## Parametric coefficients:
##                Estimate Std. Error t value Pr(>|t|)
## (Intercept)   -0.514465   0.321723  -1.599    0.110
## PDS_score      0.030375   0.039037   0.778    0.437
## interview_age  0.003963   0.002710   1.462    0.144
##
##
## R-sq.(adj) =  0.000119
## lmer.REML =   5482  Scale est. = 0.84259   n = 2045
```

```
##                   stdcoef      stdse
## X(Intercept)   0.00000000 0.00000000
## XPDS_score     0.01745211 0.02242884
## Xinterview_age 0.03279334 0.02242577
```

2.4 Model B: Putamen Anticipation ~ PDS

Female participants

```
##
## Family: gaussian
## Link function: identity
```

```
##
## Formula:
## putamen_rvsn_ant_z ~ PDS_score + interview_age
##
## Parametric coefficients:
##                Estimate Std. Error t value Pr(>|t|)
## (Intercept)   -0.492088   0.313014  -1.572    0.116
## PDS_score     -0.010024   0.027981  -0.358    0.720
## interview_age  0.004141   0.002706   1.531    0.126
##
##
## R-sq.(adj) =  0.000294
## lmer.REML = 5384.4  Scale est. = 0.70432   n = 2071
```

```
##                     stdcoef      stdse
## X(Intercept)    0.000000000 0.00000000
## XPDS_score     -0.008258293 0.02305309
## Xinterview_age  0.035115794 0.02294276
```

Male participants

```
##
## Family: gaussian
## Link function: identity
##
## Formula:
## putamen_rvsn_ant_z ~ PDS_score + interview_age
##
## Parametric coefficients:
##                Estimate Std. Error t value Pr(>|t|)
## (Intercept)   -0.358318   0.313849  -1.142    0.254
## PDS_score     -0.005580   0.038071  -0.147    0.883
## interview_age  0.003213   0.002644   1.215    0.224
##
##
## R-sq.(adj) =  -0.000341
## lmer.REML = 5394.6  Scale est. = 0.66477   n = 2050
```

```
##                     stdcoef      stdse
## X(Intercept)    0.000000000 0.00000000
## XPDS_score     -0.003287435 0.02242749
## Xinterview_age  0.027193302 0.02237501
```

2.5 Model: Accumbens Anticipation ~ PDS

Female participants

```
##
## Family: gaussian
## Link function: identity
##
## Formula:
```

```
## accumbens_rvsn_ant_z ~ PDS_score + interview_age
##
## Parametric coefficients:
##                 Estimate Std. Error t value Pr(>|t|)
## (Intercept)    0.1374282  0.2480263   0.554    0.580
## PDS_score     -0.0319156  0.0221177  -1.443    0.149
## interview_age -0.0006455  0.0021450  -0.301    0.763
##
##
## R-sq.(adj) =  0.000265
## lmer.REML = 4425.7  Scale est. = 0.48435   n = 2066
```

```
##                     stdcoef      stdse
## X(Intercept)    0.000000000 0.00000000
## XPDS_score     -0.033043238 0.02289918
## Xinterview_age -0.006884408 0.02287668
```

Male participants

```
##
## Family: gaussian
## Link function: identity
##
## Formula:
## accumbens_rvsn_ant_z ~ PDS_score + interview_age
##
## Parametric coefficients:
##                Estimate Std. Error t value Pr(>|t|)
## (Intercept)    0.166568   0.251739   0.662    0.508
## PDS_score      0.018581   0.030305   0.613    0.540
## interview_age -0.001568   0.002120  -0.739    0.460
##
##
## R-sq.(adj) =  -0.00061
## lmer.REML = 4487.9  Scale est. = 0.44126   n = 2046
```

```
##                    stdcoef      stdse
## X(Intercept)    0.00000000 0.00000000
## XPDS_score      0.01375500 0.02243394
## Xinterview_age -0.01655136 0.02238494
```

2.6 Model: Caudate Feedback ~ PDS

Female participants

```
##
## Family: gaussian
## Link function: identity
##
## Formula:
## caudate_posvsneg_feedback_z ~ PDS_score + interview_age
##
```

```
## Parametric coefficients:
##                Estimate Std. Error t value Pr(>|t|)
## (Intercept)   -0.010955   0.309581  -0.035    0.972
## PDS_score      0.037116   0.027726   1.339    0.181
## interview_age -0.000575   0.002675  -0.215    0.830
##
##
## R-sq.(adj) =  -0.000269
## lmer.REML = 5311.2  Scale est. = 0.61773   n = 2067
```

```
##                     stdcoef      stdse
## X(Intercept)    0.000000000 0.00000000
## XPDS_score      0.030988334 0.02314848
## Xinterview_age -0.004942956 0.02299573
```

Male participants

```
##
## Family: gaussian
## Link function: identity
##
## Formula:
## caudate_posvsneg_feedback_z ~ PDS_score + interview_age
##
## Parametric coefficients:
##                 Estimate Std. Error t value Pr(>|t|)
## (Intercept)   -0.0100644  0.3156855  -0.032    0.975
## PDS_score     -0.0023417  0.0381382  -0.061    0.951
## interview_age  0.0002062  0.0026623   0.077    0.938
##
##
## R-sq.(adj) =  -0.000971
## lmer.REML = 5442.1  Scale est. = 0.82381   n = 2051
```

```
##                     stdcoef      stdse
## X(Intercept)    0.000000000 0.00000000
## XPDS_score     -0.001370448 0.02231964
## Xinterview_age  0.001728585 0.02231775
```

2.7 Model: Putamen Feedback ~ PDS

Female participants

```
##
## Family: gaussian
## Link function: identity
##
## Formula:
## putamen_posvsneg_feedback_z ~ PDS_score + interview_age
##
## Parametric coefficients:
##                Estimate Std. Error t value Pr(>|t|)
```

```
## (Intercept)    0.126483   0.305477   0.414    0.679
## PDS_score      0.035447   0.027397   1.294    0.196
## interview_age -0.001628   0.002635  -0.618    0.537
##
##
## R-sq.(adj) =  -0.000225
## lmer.REML = 5245.6  Scale est. = 0.6817    n = 2067
```

```
##                    stdcoef      stdse
## X(Intercept)    0.00000000 0.00000000
## XPDS_score      0.02997135 0.02316538
## Xinterview_age -0.01421511 0.02301236
```

Male participants

```
##
## Family: gaussian
## Link function: identity
##
## Formula:
## putamen_posvsneg_feedback_z ~ PDS_score + interview_age
##
## Parametric coefficients:
##               Estimate Std. Error t value Pr(>|t|)
## (Intercept)    0.280037   0.313829   0.892    0.372
## PDS_score      0.028118   0.037766   0.745    0.457
## interview_age -0.002376   0.002641  -0.900    0.368
##
##
## R-sq.(adj) =  -0.000455
## lmer.REML = 5400.4  Scale est. = 0.79507   n = 2056
```

```
##                    stdcoef      stdse
## X(Intercept)    0.00000000 0.00000000
## XPDS_score      0.01666775 0.02238702
## Xinterview_age -0.02014259 0.02239142
```

2.8 Model: Accumbens Feedback ~ PDS

Female participants

```
##
## Family: gaussian
## Link function: identity
##
## Formula:
## accumbens_posvsneg_feedback_z ~ PDS_score + interview_age
##
## Parametric coefficients:
##               Estimate Std. Error t value Pr(>|t|)
## (Intercept)   -0.370594   0.241795  -1.533    0.126
## PDS_score      0.011952   0.021694   0.551    0.582
```

```
## interview_age  0.002769   0.002090   1.325    0.185
##
##
## R-sq.(adj) =  0.000574
## lmer.REML = 4288.8  Scale est. = 0.43394   n = 2066
```

```
##                   stdcoef      stdse
## X(Intercept)   0.00000000 0.00000000
## XPDS_score     0.01274990 0.02314187
## Xinterview_age 0.03048929 0.02301523
```

Male participants

```
##
## Family: gaussian
## Link function: identity
##
## Formula:
## accumbens_posvsneg_feedback_z ~ PDS_score + interview_age
##
## Parametric coefficients:
##                 Estimate Std. Error t value Pr(>|t|)
## (Intercept)    0.0057049  0.2578990   0.022    0.982
## PDS_score     -0.0306109  0.0310797  -0.985    0.325
## interview_age  0.0004227  0.0021715   0.195    0.846
##
##
## R-sq.(adj) =  -0.000345
## lmer.REML = 4573.9  Scale est. = 0.44906   n = 2049
```

```
##                     stdcoef      stdse
## X(Intercept)    0.000000000 0.00000000
## XPDS_score     -0.022114336 0.02245302
## Xinterview_age  0.004359055 0.02239593
```

2.9 Model: OFC Anticipation ~ PDS

Female participants

```
##
## Family: gaussian
## Link function: identity
##
## Formula:
## lOFC_rvsn_ant_z ~ PDS_score + interview_age
##
## Parametric coefficients:
##                 Estimate Std. Error t value Pr(>|t|)
## (Intercept)    0.0801892  0.2006050   0.400    0.689
## PDS_score     -0.0171588  0.0180734  -0.949    0.343
## interview_age -0.0004233  0.0017341  -0.244    0.807
##
```

```
##
## R-sq.(adj) =  -0.000415
## lmer.REML = 3499.7  Scale est. = 0.31577   n = 2058
```

```
##                      stdcoef      stdse
## X(Intercept)    0.000000000 0.00000000
## XPDS_score     -0.022009090 0.02318212
## Xinterview_age -0.005631825 0.02307328
```

```
##
## Family: gaussian
## Link function: identity
##
## Formula:
## mOFC_rvsn_ant_z ~ PDS_score + interview_age
##
## Parametric coefficients:
##                 Estimate Std. Error t value Pr(>|t|)
## (Intercept)    0.203967   0.231252   0.882   0.3779
## PDS_score     -0.052075   0.020727  -2.512   0.0121 *
## interview_age -0.001062   0.001998  -0.531   0.5952
## ---
## Signif. codes:  0 '***' 0.001 '**' 0.01 '*' 0.05 '.' 0.1 ' ' 1
##
##
## R-sq.(adj) =  0.00257
## lmer.REML = 4086.2  Scale est. = 0.41983   n = 2059
```

```
##                     stdcoef      stdse
## X(Intercept)    0.00000000 0.00000000
## XPDS_score     -0.05809832 0.02312399
## Xinterview_age -0.01222406 0.02300552
```

Male participants

```
##
## Family: gaussian
## Link function: identity
##
## Formula:
## lOFC_rvsn_ant_z ~ PDS_score + interview_age
##
## Parametric coefficients:
##                 Estimate Std. Error t value Pr(>|t|)
## (Intercept)   -0.428284   0.216354  -1.980   0.0479 *
## PDS_score      0.024197   0.026393   0.917   0.3594
## interview_age  0.003022   0.001822   1.659   0.0974 .
## ---
## Signif. codes:  0 '***' 0.001 '**' 0.01 '*' 0.05 '.' 0.1 ' ' 1
##
##
## R-sq.(adj) =  0.000838
## lmer.REML = 3831.4  Scale est. = 0.3788    n = 2036
```

```
##                    stdcoef      stdse
## X(Intercept)   0.00000000 0.00000000
## XPDS_score     0.02058862 0.02245720
## Xinterview_age 0.03726116 0.02246595
```

```
##
## Family: gaussian
## Link function: identity
##
## Formula:
## mOFC_rvsn_ant_z ~ PDS_score + interview_age
##
## Parametric coefficients:
##                 Estimate Std. Error t value Pr(>|t|)
## (Intercept)   -0.1786669  0.2468011  -0.724   0.4692
## PDS_score      0.0572368  0.0300034   1.908   0.0566 .
## interview_age  0.0006223  0.0020815   0.299   0.7650
## ---
## Signif. codes:  0 '***' 0.001 '**' 0.01 '*' 0.05 '.' 0.1 ' ' 1
##
##
## R-sq.(adj) =  0.000957
## lmer.REML =   4403  Scale est. = 0.50104   n = 2042
```

```
##                     stdcoef      stdse
## X(Intercept)   0.000000000 0.00000000
## XPDS_score     0.042597578 0.02232952
## Xinterview_age 0.006675486 0.02232952
```

2.10 Model: OFC Feedback ~ PDS

Female participants

```
##
## Family: gaussian
## Link function: identity
##
## Formula:
## lOFC_posvsneg_feedback_z ~ PDS_score + interview_age
##
## Parametric coefficients:
##                 Estimate Std. Error t value Pr(>|t|)
## (Intercept)    0.0625161  0.1799161   0.347    0.728
## PDS_score      0.0085022  0.0161609   0.526    0.599
## interview_age -0.0009009  0.0015555  -0.579    0.563
##
##
## R-sq.(adj) =  -0.000829
## lmer.REML = 3071.4  Scale est. = 0.25421   n = 2067
```

```
##                     stdcoef      stdse
## X(Intercept)    0.00000000 0.00000000
```

```
## XPDS_score      0.01217331 0.02313880
## Xinterview_age -0.01334089 0.02303385
```

```
##
## Family: gaussian
## Link function: identity
##
## Formula:
## mOFC_posvsneg_feedback_z ~ PDS_score + interview_age
##
## Parametric coefficients:
##                 Estimate Std. Error t value Pr(>|t|)
## (Intercept)   -0.0059040  0.2119441  -0.028    0.978
## PDS_score      0.0043212  0.0190101   0.227    0.820
## interview_age -0.0002242  0.0018349  -0.122    0.903
##
##
## R-sq.(adj) =  -0.000935
## lmer.REML = 3789.9  Scale est. = 0.33159   n = 2071
```

```
##                     stdcoef      stdse
## X(Intercept)    0.000000000 0.00000000
## XPDS_score      0.005208220 0.02291225
## Xinterview_age -0.002796687 0.02289108
```

Male participants

```
##
## Family: gaussian
## Link function: identity
##
## Formula:
## lOFC_posvsneg_feedback_z ~ PDS_score + interview_age
##
## Parametric coefficients:
##                Estimate Std. Error t value Pr(>|t|)
## (Intercept)   -0.151824   0.190749  -0.796    0.426
## PDS_score     -0.017865   0.023175  -0.771    0.441
## interview_age  0.001534   0.001608   0.954    0.340
##
##
## R-sq.(adj) =  -0.00028
## lmer.REML =   3332  Scale est. = 0.24825   n = 2035
```

```
##                    stdcoef      stdse
## X(Intercept)    0.00000000 0.00000000
## XPDS_score     -0.01731160 0.02245700
## Xinterview_age  0.02138588 0.02241312
```

```
##
## Family: gaussian
## Link function: identity
```

```
##
## Formula:
## mOFC_posvsneg_feedback_z ~ PDS_score + interview_age
##
## Parametric coefficients:
##                 Estimate Std. Error t value Pr(>|t|)
## (Intercept)   -0.0583309  0.2254788  -0.259    0.796
## PDS_score     -0.0215757  0.0274653  -0.786    0.432
## interview_age  0.0009864  0.0019008   0.519    0.604
##
##
## R-sq.(adj) =  -0.000549
## lmer.REML = 4043.1  Scale est. = 0.36715   n = 2044

##                    stdcoef      stdse
## X(Intercept)    0.00000000 0.00000000
## XPDS_score     -0.01758366 0.02238350
## Xinterview_age  0.01160070 0.02235347
```

2.11 Model: Caudate Anticipation ~ Testosterone

Female participants

```
##
## Family: gaussian
## Link function: identity
##
## Formula:
## caudate_rvsn_ant_z ~ hormone_scr_ert_mean + interview_age
##
## Parametric coefficients:
##                       Estimate Std. Error t value Pr(>|t|)
## (Intercept)          -0.331449   0.327022  -1.014    0.311
## hormone_scr_ert_mean -0.001533   0.001263  -1.213    0.225
## interview_age         0.003171   0.002795   1.135    0.257
##
##
## R-sq.(adj) =  0.000349
## lmer.REML = 4991.7  Scale est. = 0.75377   n = 1907

##                           stdcoef      stdse
## X(Intercept)           0.00000000 0.00000000
## Xhormone_scr_ert_mean -0.02858393 0.02355753
## Xinterview_age         0.02668023 0.02351705
```

Male participants

```
##
## Family: gaussian
## Link function: identity
##
## Formula:
```

```
## caudate_rvsn_ant_z ~ hormone_scr_ert_mean + interview_age
##
## Parametric coefficients:
##                      Estimate Std. Error t value Pr(>|t|)
## (Intercept)         -0.267557   0.328709  -0.814    0.416
## hormone_scr_ert_mean -0.001016   0.001469  -0.692    0.489
## interview_age        0.002560   0.002777   0.922    0.357
##
##
## R-sq.(adj) =  -0.000436
## lmer.REML = 5087.4  Scale est. = 0.82111   n = 1903
```

```
##                          stdcoef      stdse
## X(Intercept)           0.00000000 0.00000000
## Xhormone_scr_ert_mean -0.01617999 0.02339114
## Xinterview_age         0.02145159 0.02326813
```

2.12 Model B: Putamen Anticipation ~ Testosterone

Female participants

```
##
## Family: gaussian
## Link function: identity
##
## Formula:
## putamen_rvsn_ant_z ~ hormone_scr_ert_mean + interview_age
##
## Parametric coefficients:
##                      Estimate Std. Error t value Pr(>|t|)
## (Intercept)         -0.381268   0.321834  -1.185    0.236
## hormone_scr_ert_mean -0.000192   0.001245  -0.154    0.877
## interview_age        0.003094   0.002751   1.125    0.261
##
##
## R-sq.(adj) =  -0.000274
## lmer.REML = 4931.4  Scale est. = 0.67821   n = 1905
```

```
##                           stdcoef      stdse
## X(Intercept)           0.000000000 0.00000000
## Xhormone_scr_ert_mean -0.003633225 0.02356459
## Xinterview_age         0.026444121 0.02351027
```

Male participants

```
##
## Family: gaussian
## Link function: identity
##
## Formula:
## putamen_rvsn_ant_z ~ hormone_scr_ert_mean + interview_age
##
```

```
## Parametric coefficients:
##                      Estimate Std. Error t value Pr(>|t|)
## (Intercept)          -0.327363   0.324487  -1.009    0.313
## hormone_scr_ert_mean -0.001973   0.001451  -1.360    0.174
## interview_age         0.003410   0.002741   1.244    0.214
##
##
## R-sq.(adj) =  0.000567
## lmer.REML = 5034.6  Scale est. = 0.60689   n = 1903
```

```
##                          stdcoef      stdse
## X(Intercept)           0.00000000 0.00000000
## Xhormone_scr_ert_mean -0.03184640 0.02341146
## Xinterview_age         0.02888068 0.02321811
```

2.13 Model: Accumbens Anticipation ~ Testosterone

Female participants

```
##
## Family: gaussian
## Link function: identity
##
## Formula:
## accumbens_rvsn_ant_z ~ hormone_scr_ert_mean + interview_age
##
## Parametric coefficients:
##                       Estimate Std. Error t value Pr(>|t|)
## (Intercept)           0.0999811  0.2558527   0.391    0.696
## hormone_scr_ert_mean -0.0004158  0.0009888  -0.421    0.674
## interview_age        -0.0006720  0.0021883  -0.307    0.759
##
##
## R-sq.(adj) =  -0.000894
## lmer.REML = 4060.6  Scale est. = 0.4233    n = 1901
```

```
##                           stdcoef      stdse
## X(Intercept)           0.000000000 0.00000000
## Xhormone_scr_ert_mean -0.009877818 0.02348851
## Xinterview_age        -0.007205706 0.02346533
```

Male participants

```
##
## Family: gaussian
## Link function: identity
##
## Formula:
## accumbens_rvsn_ant_z ~ hormone_scr_ert_mean + interview_age
##
## Parametric coefficients:
##                       Estimate Std. Error t value Pr(>|t|)
```

```
## (Intercept)           2.128e-01  2.587e-01   0.823    0.411
## hormone_scr_ert_mean -7.013e-05  1.159e-03  -0.061    0.952
## interview_age        -1.700e-03  2.185e-03  -0.778    0.437
##
##
## R-sq.(adj) =  -0.000677
## lmer.REML = 4161.9  Scale est. = 0.43076   n = 1897
```

```
##                          stdcoef      stdse
## X(Intercept)           0.00000000 0.00000000
## Xhormone_scr_ert_mean -0.00141760 0.02342748
## Xinterview_age        -0.01811179 0.02327660
```

2.14 Model: Caudate Feedback ~ Testosterone

Female participants

```
##
## Family: gaussian
## Link function: identity
##
## Formula:
## caudate_posvsneg_feedback_z ~ hormone_scr_ert_mean + interview_age
##
## Parametric coefficients:
##                        Estimate Std. Error t value Pr(>|t|)
## (Intercept)          -0.0884340  0.3173849  -0.279    0.781
## hormone_scr_ert_mean  0.0003856  0.0012260   0.315    0.753
## interview_age         0.0004997  0.0027122   0.184    0.854
##
##
## R-sq.(adj) =  -0.000963
## lmer.REML =   4849  Scale est. = 0.59772   n = 1901
```

```
##                          stdcoef      stdse
## X(Intercept)          0.000000000 0.00000000
## Xhormone_scr_ert_mean 0.007444423 0.02366809
## Xinterview_age        0.004344444 0.02358197
```

Male participants

```
##
## Family: gaussian
## Link function: identity
##
## Formula:
## caudate_posvsneg_feedback_z ~ hormone_scr_ert_mean + interview_age
##
## Parametric coefficients:
##                       Estimate Std. Error t value Pr(>|t|)
## (Intercept)          -0.057292   0.325531  -0.176    0.860
## hormone_scr_ert_mean -0.002013   0.001445  -1.393    0.164
```

```
## interview_age         0.001059   0.002755   0.384    0.701
##
##
## R-sq.(adj) =  -1.49e-05
## lmer.REML = 5064.8  Scale est. = 0.82774   n = 1903
```

```
##                            stdcoef      stdse
## X(Intercept)           0.000000000 0.00000000
## Xhormone_scr_ert_mean -0.032305363 0.02319243
## Xinterview_age         0.008914469 0.02319243
```

2.15 Model: Putamen Feedback ~ Testosterone

Female participants

```
##
## Family: gaussian
## Link function: identity
##
## Formula:
## putamen_posvsneg_feedback_z ~ hormone_scr_ert_mean + interview_age
##
## Parametric coefficients:
##                       Estimate Std. Error t value Pr(>|t|)
## (Intercept)           0.186877   0.312380   0.598    0.550
## hormone_scr_ert_mean  0.000457   0.001207   0.379    0.705
## interview_age        -0.001756   0.002664  -0.659    0.510
##
##
## R-sq.(adj) =  -0.00103
## lmer.REML = 4789.3  Scale est. = 0.65976   n = 1904
```

```
##                            stdcoef      stdse
## X(Intercept)           0.000000000 0.00000000
## Xhormone_scr_ert_mean  0.008945327 0.02361755
## Xinterview_age        -0.015527475 0.02355634
```

Male participants

```
##
## Family: gaussian
## Link function: identity
##
## Formula:
## putamen_posvsneg_feedback_z ~ hormone_scr_ert_mean + interview_age
##
## Parametric coefficients:
##                       Estimate Std. Error t value Pr(>|t|)
## (Intercept)           0.263179   0.323216   0.814   0.4156
## hormone_scr_ert_mean -0.002500   0.001443  -1.732   0.0834 .
## interview_age        -0.001290   0.002722  -0.474   0.6355
## ---
```

```
## Signif. codes:  0 '***' 0.001 '**' 0.01 '*' 0.05 '.' 0.1 ' ' 1
##
##
## R-sq.(adj) =  0.000549
## lmer.REML = 5016.5  Scale est. = 0.7933    n = 1908
```

```
##                            stdcoef      stdse
## X(Intercept)            0.00000000 0.00000000
## Xhormone_scr_ert_mean -0.04066907 0.02347873
## Xinterview_age        -0.01101568 0.02323468
```

2.16 Model: Accumbens Feedback ~ Testosterone

Female participants

```
##
## Family: gaussian
## Link function: identity
##
## Formula:
## accumbens_posvsneg_feedback_z ~ hormone_scr_ert_mean + interview_age
##
## Parametric coefficients:
##                         Estimate Std. Error t value Pr(>|t|)
## (Intercept)           -3.609e-01  2.501e-01  -1.443    0.149
## hormone_scr_ert_mean  8.766e-06  9.658e-04   0.009    0.993
## interview_age         2.914e-03  2.136e-03   1.365    0.173
##
##
## R-sq.(adj) =  0.000316
## lmer.REML = 3943.2  Scale est. = 0.42807   n = 1900
```

```
##                              stdcoef      stdse
## X(Intercept)          0.0000000000 0.00000000
## Xhormone_scr_ert_mean 0.0002142457 0.02360565
## Xinterview_age        0.0321522608 0.02356008
```

Male participants

```
##
## Family: gaussian
## Link function: identity
##
## Formula:
## accumbens_posvsneg_feedback_z ~ hormone_scr_ert_mean + interview_age
##
## Parametric coefficients:
##                         Estimate Std. Error t value Pr(>|t|)
## (Intercept)            0.0216385  0.2648198   0.082    0.935
## hormone_scr_ert_mean -0.0014731  0.0011834  -1.245    0.213
## interview_age         0.0002912  0.0022317   0.130    0.896
##
```

```
##
## R-sq.(adj) =  -4.49e-05
## lmer.REML = 4241.3  Scale est. = 0.51321   n = 1903
```

```
##                            stdcoef      stdse
## X(Intercept)           0.000000000 0.00000000
## Xhormone_scr_ert_mean -0.029235035 0.02348611
## Xinterview_age         0.003034269 0.02325585
```

2.17 Model: OFC Anticipation ~ Testosterone

Female participants

```
##
## Family: gaussian
## Link function: identity
##
## Formula:
## lOFC_rvsn_ant_z ~ hormone_scr_ert_mean + interview_age
##
## Parametric coefficients:
##                        Estimate Std. Error t value Pr(>|t|)
## (Intercept)           0.1368392  0.2063384   0.663   0.5073
## hormone_scr_ert_mean  0.0015863  0.0007966   1.991   0.0466 *
## interview_age        -0.0015929  0.0017624  -0.904   0.3662
## ---
## Signif. codes:  0 '***' 0.001 '**' 0.01 '*' 0.05 '.' 0.1 ' ' 1
##
##
## R-sq.(adj) =  0.00096
## lmer.REML = 3200.9  Scale est. = 0.31099   n = 1894
```

```
##                           stdcoef      stdse
## X(Intercept)           0.00000000 0.00000000
## Xhormone_scr_ert_mean  0.04704085 0.02362244
## Xinterview_age        -0.02132550 0.02359493
```

```
##
## Family: gaussian
## Link function: identity
##
## Formula:
## mOFC_rvsn_ant_z ~ hormone_scr_ert_mean + interview_age
##
## Parametric coefficients:
##                        Estimate Std. Error t value Pr(>|t|)
## (Intercept)           0.3449714  0.2392035   1.442    0.149
## hormone_scr_ert_mean  0.0012793  0.0009213   1.389    0.165
## interview_age        -0.0033111  0.0020421  -1.621    0.105
##
##
## R-sq.(adj) =  0.000847
## lmer.REML = 3751.8  Scale est. = 0.41513   n = 1895
```

```
##                          stdcoef      stdse
## X(Intercept)           0.00000000 0.00000000
## Xhormone_scr_ert_mean  0.03282873 0.02364041
## Xinterview_age        -0.03827340 0.02360548
```

Male participants

```
##
## Family: gaussian
## Link function: identity
##
## Formula:
## lOFC_rvsn_ant_z ~ hormone_scr_ert_mean + interview_age
##
## Parametric coefficients:
##                        Estimate Std. Error t value Pr(>|t|)
## (Intercept)          -0.3857820  0.2220472  -1.737   0.0825 .
## hormone_scr_ert_mean -0.0012789  0.0009898  -1.292   0.1965
## interview_age         0.0032628  0.0018744   1.741   0.0819 .
## ---
## Signif. codes:  0 '***' 0.001 '**' 0.01 '*' 0.05 '.' 0.1 ' ' 1
##
##
## R-sq.(adj) =  0.00118
## lmer.REML =   3555  Scale est. = 0.37705   n = 1890
```

```
##                          stdcoef      stdse
## X(Intercept)           0.00000000 0.00000000
## Xhormone_scr_ert_mean -0.03032126 0.02346580
## Xinterview_age         0.04060635 0.02332744
```

```
##
## Family: gaussian
## Link function: identity
##
## Formula:
## mOFC_rvsn_ant_z ~ hormone_scr_ert_mean + interview_age
##
## Parametric coefficients:
##                        Estimate Std. Error t value Pr(>|t|)
## (Intercept)          -7.006e-02  2.529e-01  -0.277    0.782
## hormone_scr_ert_mean  1.755e-05  1.122e-03   0.016    0.988
## interview_age         3.285e-04  2.140e-03   0.154    0.878
##
##
## R-sq.(adj) =  -0.00104
## lmer.REML = 4071.1  Scale est. = 0.49562   n = 1894
```

```
##                            stdcoef      stdse
## X(Intercept)          0.0000000000 0.00000000
## Xhormone_scr_ert_mean 0.0003638866 0.02326086
## Xinterview_age        0.0035712162 0.02326086
```

2.18 Model: OFC Feedback ~ Testosterone

Female participants

```
##
## Family: gaussian
## Link function: identity
##
## Formula:
## lOFC_posvsneg_feedback_z ~ hormone_scr_ert_mean + interview_age
##
## Parametric coefficients:
##                        Estimate Std. Error t value Pr(>|t|)
## (Intercept)           0.0322220  0.1817395   0.177   0.8593
## hormone_scr_ert_mean  0.0015346  0.0007027   2.184   0.0291 *
## interview_age        -0.0009299  0.0015530  -0.599   0.5494
## ---
## Signif. codes:  0 '***' 0.001 '**' 0.01 '*' 0.05 '.' 0.1 ' ' 1
##
##
## R-sq.(adj) =  0.0016
## lmer.REML = 2743.8  Scale est. = 0.24369   n = 1900
```

```
##                           stdcoef      stdse
## X(Intercept)           0.00000000 0.00000000
## Xhormone_scr_ert_mean  0.05138086 0.02352729
## Xinterview_age        -0.01407796 0.02351004
```

```
##
## Family: gaussian
## Link function: identity
##
## Formula:
## mOFC_posvsneg_feedback_z ~ hormone_scr_ert_mean + interview_age
##
## Parametric coefficients:
##                        Estimate Std. Error t value Pr(>|t|)
## (Intercept)          -0.0399194  0.2172604  -0.184    0.854
## hormone_scr_ert_mean  0.0013479  0.0008409   1.603    0.109
## interview_age        -0.0002294  0.0018584  -0.123    0.902
##
##
## R-sq.(adj) =  0.000333
## lmer.REML =   3456  Scale est. = 0.34165   n = 1905
```

```
##                            stdcoef      stdse
## X(Intercept)           0.000000000 0.00000000
## Xhormone_scr_ert_mean  0.037510399 0.02339950
## Xinterview_age        -0.002888458 0.02339545
```

Male participants

```
##
```

```
## Family: gaussian
## Link function: identity
##
## Formula:
## lOFC_posvsneg_feedback_z ~ hormone_scr_ert_mean + interview_age
##
## Parametric coefficients:
##                        Estimate Std. Error t value Pr(>|t|)
## (Intercept)          -0.1192861  0.1968776  -0.606    0.545
## hormone_scr_ert_mean -0.0001786  0.0008749  -0.204    0.838
## interview_age         0.0010953  0.0016660   0.657    0.511
##
##
## R-sq.(adj) =  -0.000781
## lmer.REML = 3111.5  Scale est. = 0.2558    n = 1888
```

```
##                            stdcoef      stdse
## X(Intercept)           0.000000000 0.00000000
## Xhormone_scr_ert_mean -0.004771496 0.02337131
## Xinterview_age         0.015330283 0.02331736
```

```
##
## Family: gaussian
## Link function: identity
##
## Formula:
## mOFC_posvsneg_feedback_z ~ hormone_scr_ert_mean + interview_age
##
## Parametric coefficients:
##                        Estimate Std. Error t value Pr(>|t|)
## (Intercept)          -0.0309614  0.2311093  -0.134    0.893
## hormone_scr_ert_mean -0.0006028  0.0010264  -0.587    0.557
## interview_age         0.0006328  0.0019558   0.324    0.746
##
##
## R-sq.(adj) =  -0.000823
## lmer.REML = 3738.2  Scale est. = 0.3802    n = 1894
```

```
##                            stdcoef      stdse
## X(Intercept)           0.000000000 0.00000000
## Xhormone_scr_ert_mean -0.013675292 0.02328506
## Xinterview_age         0.007527073 0.02326509
```

2.19 Model: MID Reaction Time ~ Testosterone

Female participants

```
##
## Family: gaussian
## Link function: identity
##
## Formula:
```

```
## rt_diff_large_neutral_z ~ hormone_scr_ert_mean + interview_age
##
## Parametric coefficients:
##                      Estimate Std. Error t value Pr(>|t|)
## (Intercept)         -0.678680   0.329453  -2.060   0.0395 *
## hormone_scr_ert_mean -0.001352   0.001281  -1.055   0.2913
## interview_age         0.006348   0.002819   2.252   0.0244 *
## ---
## Signif. codes:  0 '***' 0.001 '**' 0.01 '*' 0.05 '.' 0.1 ' ' 1
##
##
## R-sq.(adj) =  0.00175
## lmer.REML =   5596  Scale est. = 0.76342   n = 2066
```

```
##                           stdcoef      stdse
## X(Intercept)           0.00000000 0.00000000
## Xhormone_scr_ert_mean -0.02372487 0.02247763
## Xinterview_age         0.05055583 0.02244872
```

```
##
## Family: gaussian
## Link function: identity
##
## Formula:
## rt_diff_large_small_z ~ hormone_scr_ert_mean + interview_age
##
## Parametric coefficients:
##                      Estimate Std. Error t value Pr(>|t|)
## (Intercept)         -0.505114   0.343770  -1.469    0.142
## hormone_scr_ert_mean -0.001082   0.001337  -0.809    0.419
## interview_age         0.004672   0.002940   1.589    0.112
##
##
## R-sq.(adj) =  0.000344
## lmer.REML = 5754.6  Scale est. = 0.86568   n = 2066
```

```
##                           stdcoef      stdse
## X(Intercept)           0.00000000 0.00000000
## Xhormone_scr_ert_mean -0.01828262 0.02259811
## Xinterview_age         0.03583781 0.02254702
```

Male participants

```
##
## Family: gaussian
## Link function: identity
##
## Formula:
## rt_diff_large_neutral_z ~ hormone_scr_ert_mean + interview_age
##
## Parametric coefficients:
##                       Estimate Std. Error t value Pr(>|t|)
## (Intercept)          -0.5366392  0.3111960  -1.724   0.0848 .
```

```
## hormone_scr_ert_mean -0.0008174  0.0013729  -0.595   0.5517
## interview_age         0.0045508  0.0026349   1.727   0.0843 .
## ---
## Signif. codes:  0 '***' 0.001 '**' 0.01 '*' 0.05 '.' 0.1 ' ' 1
##
##
## R-sq.(adj) =  0.000562
## lmer.REML = 5761.5  Scale est. = 0.84489   n = 2148
```

```
##                            stdcoef      stdse
## X(Intercept)            0.00000000 0.00000000
## Xhormone_scr_ert_mean -0.01307491 0.02196054
## Xinterview_age          0.03780163 0.02188705
```

```
##
## Family: gaussian
## Link function: identity
##
## Formula:
## rt_diff_large_small_z ~ hormone_scr_ert_mean + interview_age
##
## Parametric coefficients:
##                        Estimate Std. Error t value Pr(>|t|)
## (Intercept)          -0.0445913  0.3084921  -0.145    0.885
## hormone_scr_ert_mean -0.0012405  0.0013575  -0.914    0.361
## interview_age         0.0004983  0.0026142   0.191    0.849
##
##
## R-sq.(adj) =  -0.000542
## lmer.REML = 5736.4  Scale est. = 0.83637   n = 2148
```

```
##                             stdcoef      stdse
## X(Intercept)            0.000000000 0.00000000
## Xhormone_scr_ert_mean -0.019972637 0.02185602
## Xinterview_age          0.004166389 0.02185602
```

2.20 Model: BIS-BAS-RR ~ Testosterone

Female participants

```
##
## Family: gaussian
## Link function: identity
##
## Formula:
## bisbas_ss_basm_rr_z ~ hormone_scr_ert_mean + interview_age
##
## Parametric coefficients:
##                        Estimate Std. Error t value Pr(>|t|)
## (Intercept)           0.3737068  0.3253719   1.149    0.251
## hormone_scr_ert_mean  0.0006886  0.0012733   0.541    0.589
## interview_age        -0.0037300  0.0027769  -1.343    0.179
```

```
##
##
## R-sq.(adj) =  0.000906
## lmer.REML =   6997  Scale est. = 0.7909    n = 2467
```

```
##                          stdcoef      stdse
## X(Intercept)           0.00000000 0.00000000
## Xhormone_scr_ert_mean  0.01126782 0.02083541
## Xinterview_age        -0.02783589 0.02072306
```

Male participants

```
##
## Family: gaussian
## Link function: identity
##
## Formula:
## bisbas_ss_basm_rr_z ~ hormone_scr_ert_mean + interview_age
##
## Parametric coefficients:
##                       Estimate Std. Error t value Pr(>|t|)
## (Intercept)           0.017767   0.295379   0.060   0.9520
## hormone_scr_ert_mean -0.002133   0.001296  -1.646   0.0999 .
## interview_age         0.001037   0.002493   0.416   0.6774
## ---
## Signif. codes:  0 '***' 0.001 '**' 0.01 '*' 0.05 '.' 0.1 ' ' 1
##
##
## R-sq.(adj) =  -0.000457
## lmer.REML = 7435.8  Scale est. = 0.73815   n = 2686
```

```
##                           stdcoef      stdse
## X(Intercept)           0.000000000 0.00000000
## Xhormone_scr_ert_mean -0.032758198 0.01990263
## Xinterview_age         0.008154483 0.01959988
```

3—Internalizing~Reward—

3.1 Model: CBCL internalizing factor ~ Nucleus Accumbens activity (anticipation stage)

Female participants

```
##
## Family: gaussian
## Link function: identity
##
## Formula:
## cbcl_scr_syn_internal_r ~ accumbens_rvsn_ant_z + interview_age
##
## Parametric coefficients:
##                       Estimate Std. Error t value Pr(>|t|)
## (Intercept)            0.93807    1.89253   0.496   0.6202
## accumbens_rvsn_ant_z -0.25017    0.16587  -1.508   0.1317
## interview_age          0.03348    0.01579   2.120   0.0341 *
## ---
## Signif. codes:  0 '***' 0.001 '**' 0.01 '*' 0.05 '.' 0.1 ' ' 1
##
##
## R-sq.(adj) =  0.000946
## lmer.REML =  12780  Scale est. = 15.797    n = 2065
```

```
##                            stdcoef      stdse
## X(Intercept)            0.00000000 0.00000000
## Xaccumbens_rvsn_ant_z -0.03253184 0.02156971
## Xinterview_age          0.04644300 0.02190347
```

Male participants

```
##
## Family: gaussian
## Link function: identity
##
## Formula:
## cbcl_scr_syn_internal_r ~ accumbens_rvsn_ant_z + interview_age
##
## Parametric coefficients:
##                       Estimate Std. Error t value Pr(>|t|)
## (Intercept)            2.81159    1.92279   1.462    0.144
## accumbens_rvsn_ant_z  0.03617    0.16810   0.215    0.830
## interview_age          0.01815    0.01601   1.134    0.257
##
##
## R-sq.(adj) =  -0.00128
## lmer.REML =  12801  Scale est. = 14.906    n = 2046
```

```
##                            stdcoef      stdse
## X(Intercept)           0.000000000 0.00000000
```

```
## Xaccumbens_rvsn_ant_z 0.004671257 0.02171120
## Xinterview_age        0.024747561 0.02183056
```

3.2 Model: CBCL internalizing factor ~ Caudate activity (anticipation stage)

Female participants

```
##
## Family: gaussian
## Link function: identity
##
## Formula:
## cbcl_scr_syn_internal_r ~ caudate_rvsn_ant_z + interview_age
##
## Parametric coefficients:
##                    Estimate Std. Error t value Pr(>|t|)
## (Intercept)         1.23747    1.89544   0.653   0.5139
## caudate_rvsn_ant_z  0.03828    0.13153   0.291   0.7710
## interview_age       0.03104    0.01581   1.963   0.0498 *
## ---
## Signif. codes:  0 '***' 0.001 '**' 0.01 '*' 0.05 '.' 0.1 ' ' 1
##
##
## R-sq.(adj) =  -0.000229
## lmer.REML =  12819  Scale est. = 16.059    n = 2069
```

```
##                        stdcoef      stdse
## X(Intercept)        0.000000000 0.00000000
## Xcaudate_rvsn_ant_z 0.006289614 0.02160995
## Xinterview_age      0.042973215 0.02189105
```

Male participants

```
##
## Family: gaussian
## Link function: identity
##
## Formula:
## cbcl_scr_syn_internal_r ~ caudate_rvsn_ant_z + interview_age
##
## Parametric coefficients:
##                    Estimate Std. Error t value Pr(>|t|)
## (Intercept)         2.82781    1.92247   1.471    0.141
## caudate_rvsn_ant_z  0.17149    0.13191   1.300    0.194
## interview_age       0.01835    0.01602   1.145    0.252
##
##
## R-sq.(adj) =  -0.000683
## lmer.REML =  12841  Scale est. = 14.03     n = 2051
```

```
##                       stdcoef      stdse
## X(Intercept)        0.00000000 0.00000000
```

```
## Xcaudate_rvsn_ant_z 0.02799388 0.02153345
## Xinterview_age      0.02494894 0.02178028
```

3.3 Model: CBCL internalizing factor ~ Putamen activity (anticipation stage)

Female participants

```
##
## Family: gaussian
## Link function: identity
##
## Formula:
## cbcl_scr_syn_internal_r ~ putamen_rvsn_ant_z + interview_age
##
## Parametric coefficients:
##                    Estimate Std. Error t value Pr(>|t|)
## (Intercept)        1.055560   1.894179   0.557   0.5774
## putamen_rvsn_ant_z 0.002893   0.132368   0.022   0.9826
## interview_age      0.032556   0.015806   2.060   0.0395 *
## ---
## Signif. codes:  0 '***' 0.001 '**' 0.01 '*' 0.05 '.' 0.1 ' ' 1
##
##
## R-sq.(adj) =  -0.000189
## lmer.REML =  12818  Scale est. = 15.798    n = 2069
```

```
##                          stdcoef      stdse
## X(Intercept)        0.0000000000 0.00000000
## Xputamen_rvsn_ant_z 0.0004723149 0.02160736
## Xinterview_age      0.0450895285 0.02189021
```

Male participants

```
##
## Family: gaussian
## Link function: identity
##
## Formula:
## cbcl_scr_syn_internal_r ~ putamen_rvsn_ant_z + interview_age
##
## Parametric coefficients:
##                     Estimate Std. Error t value Pr(>|t|)
## (Intercept)         3.003713   1.926023   1.560    0.119
## putamen_rvsn_ant_z -0.002347   0.135141  -0.017    0.986
## interview_age       0.016796   0.016046   1.047    0.295
##
##
## R-sq.(adj) =  -0.00129
## lmer.REML =  12834  Scale est. = 14.129    n = 2050
```

```
##                           stdcoef      stdse
## X(Intercept)         0.0000000000 0.00000000
```

```
## Xputamen_rvsn_ant_z -0.0003768869 0.02170540
## Xinterview_age       0.0228301843 0.02181099
```

3.4 Model: CBCL internalizing factor ~ Accumbens activity (feedback stage)

Female participants

```
##
## Family: gaussian
## Link function: identity
##
## Formula:
## cbcl_scr_syn_internal_r ~ accumbens_posvsneg_feedback_z + interview_age
##
## Parametric coefficients:
##                               Estimate Std. Error t value Pr(>|t|)
## (Intercept)                    0.99988    1.89645   0.527   0.5981
## accumbens_posvsneg_feedback_z  0.19684    0.17181   1.146   0.2521
## interview_age                  0.03298    0.01582   2.085   0.0372 *
## ---
## Signif. codes:  0 '***' 0.001 '**' 0.01 '*' 0.05 '.' 0.1 ' ' 1
##
##
## R-sq.(adj) =  0.00028
## lmer.REML =  12781  Scale est. = 15.798    n = 2064
```

```
##                                   stdcoef      stdse
## X(Intercept)                   0.00000000 0.00000000
## Xaccumbens_posvsneg_feedback_z 0.02475519 0.02160702
## Xinterview_age                 0.04568771 0.02191529
```

Male participants

```
##
## Family: gaussian
## Link function: identity
##
## Formula:
## cbcl_scr_syn_internal_r ~ accumbens_posvsneg_feedback_z + interview_age
##
## Parametric coefficients:
##                               Estimate Std. Error t value Pr(>|t|)
## (Intercept)                    3.36478    1.93092   1.743   0.0816 .
## accumbens_posvsneg_feedback_z -0.22281    0.16571  -1.345   0.1789
## interview_age                  0.01399    0.01609   0.869   0.3847
## ---
## Signif. codes:  0 '***' 0.001 '**' 0.01 '*' 0.05 '.' 0.1 ' ' 1
##
##
## R-sq.(adj) =  -0.000388
## lmer.REML =  12833  Scale est. = 14.8      n = 2049
```

```
##                                   stdcoef      stdse
## X(Intercept)                    0.00000000 0.00000000
## Xaccumbens_posvsneg_feedback_z -0.02927669 0.02177379
## Xinterview_age                  0.01895670 0.02180197
```

3.5 Model: CBCL internalizing factor ~ Caudate activity (feedback stage)

Female participants

```
##
## Family: gaussian
## Link function: identity
##
## Formula:
## cbcl_scr_syn_internal_r ~ caudate_posvsneg_feedback_z + interview_age
##
## Parametric coefficients:
##                             Estimate Std. Error t value Pr(>|t|)
## (Intercept)                  0.99119    1.89617   0.523   0.6012
## caudate_posvsneg_feedback_z -0.03668    0.13461  -0.272   0.7853
## interview_age                0.03312    0.01582   2.094   0.0364 *
## ---
## Signif. codes:  0 '***' 0.001 '**' 0.01 '*' 0.05 '.' 0.1 ' ' 1
##
##
## R-sq.(adj) =  -0.000154
## lmer.REML =  12789  Scale est. = 15.852    n = 2065
```

```
##                                  stdcoef      stdse
## X(Intercept)                  0.000000000 0.00000000
## Xcaudate_posvsneg_feedback_z -0.005903269 0.02166702
## Xinterview_age                0.045857838 0.02190158
```

Male participants

```
##
## Family: gaussian
## Link function: identity
##
## Formula:
## cbcl_scr_syn_internal_r ~ caudate_posvsneg_feedback_z + interview_age
##
## Parametric coefficients:
##                             Estimate Std. Error t value Pr(>|t|)
## (Intercept)                  3.32207    1.92624   1.725   0.0847 .
## caudate_posvsneg_feedback_z -0.15618    0.13309  -1.174   0.2407
## interview_age                0.01417    0.01606   0.883   0.3775
## ---
## Signif. codes:  0 '***' 0.001 '**' 0.01 '*' 0.05 '.' 0.1 ' ' 1
##
##
## R-sq.(adj) =  -0.000217
## lmer.REML =  12843  Scale est. = 14.85     n = 2051
```

```
##                                  stdcoef      stdse
## X(Intercept)                  0.00000000 0.00000000
## Xcaudate_posvsneg_feedback_z -0.02531019 0.02156786
## Xinterview_age                0.01925548 0.02181229
```

3.6 Model: CBCL internalizing factor ~ Putamen activity (feedback stage)

Female participants

```
##
## Family: gaussian
## Link function: identity
##
## Formula:
## cbcl_scr_syn_internal_r ~ putamen_posvsneg_feedback_z + interview_age
##
## Parametric coefficients:
##                             Estimate Std. Error t value Pr(>|t|)
## (Intercept)                  1.13313    1.89586   0.598   0.5501
## putamen_posvsneg_feedback_z -0.11704    0.13669  -0.856   0.3919
## interview_age                0.03186    0.01582   2.014   0.0442 *
## ---
## Signif. codes:  0 '***' 0.001 '**' 0.01 '*' 0.05 '.' 0.1 ' ' 1
##
##
## R-sq.(adj) =  0.000199
## lmer.REML =  12792  Scale est. = 16.215    n = 2065
```

```
##                                  stdcoef      stdse
## X(Intercept)                  0.00000000 0.00000000
## Xputamen_posvsneg_feedback_z -0.01856874 0.02168511
## Xinterview_age                0.04414924 0.02192297
```

Male participants

```
##
## Family: gaussian
## Link function: identity
##
## Formula:
## cbcl_scr_syn_internal_r ~ putamen_posvsneg_feedback_z + interview_age
##
## Parametric coefficients:
##                             Estimate Std. Error t value Pr(>|t|)
## (Intercept)                  3.23873    1.92503   1.682   0.0926 .
## putamen_posvsneg_feedback_z -0.04213    0.13493  -0.312   0.7549
## interview_age                0.01493    0.01604   0.931   0.3521
## ---
## Signif. codes:  0 '***' 0.001 '**' 0.01 '*' 0.05 '.' 0.1 ' ' 1
##
##
## R-sq.(adj) =  -0.00116
## lmer.REML =  12878  Scale est. = 15.039    n = 2056
```

```
##                                   stdcoef     stdse
## X(Intercept)                  0.000000000 0.0000000
## Xputamen_posvsneg_feedback_z -0.006749898 0.0216173
## Xinterview_age                0.020279347 0.0217891
```

3.7 Model: CBCL internalizing factor ~ OFC activity (anticipation stage)

Female participants

```
##
## Family: gaussian
## Link function: identity
##
## Formula:
## cbcl_scr_syn_internal_r ~ lOFC_rvsn_ant_z + interview_age
##
## Parametric coefficients:
##                 Estimate Std. Error t value Pr(>|t|)
## (Intercept)      1.00430    1.90118   0.528   0.5974
## lOFC_rvsn_ant_z  0.05371    0.20796   0.258   0.7962
## interview_age    0.03302    0.01586   2.082   0.0374 *
## ---
## Signif. codes:  0 '***' 0.001 '**' 0.01 '*' 0.05 '.' 0.1 ' ' 1
##
##
## R-sq.(adj) =  -8.98e-05
## lmer.REML =  12736  Scale est. = 15.567    n = 2056

##                     stdcoef      stdse
## X(Intercept)     0.000000000 0.00000000
## XlOFC_rvsn_ant_z 0.005568829 0.02156341
## Xinterview_age   0.045736012 0.02196480

##
## Family: gaussian
## Link function: identity
##
## Formula:
## cbcl_scr_syn_internal_r ~ mOFC_rvsn_ant_z + interview_age
##
## Parametric coefficients:
##                 Estimate Std. Error t value Pr(>|t|)
## (Intercept)      0.82010    1.90148   0.431   0.6663
## mOFC_rvsn_ant_z  0.17691    0.17881   0.989   0.3226
## interview_age    0.03454    0.01587   2.177   0.0296 *
## ---
## Signif. codes:  0 '***' 0.001 '**' 0.01 '*' 0.05 '.' 0.1 ' ' 1
##
##
## R-sq.(adj) =  0.000336
## lmer.REML =  12741  Scale est. = 15.138    n = 2057

##                    stdcoef      stdse
```

```
## X(Intercept)     0.00000000 0.00000000
## XmOFC_rvsn_ant_z 0.02118601 0.02141320
## Xinterview_age   0.04776384 0.02194123
```

Male participants

```
##
## Family: gaussian
## Link function: identity
##
## Formula:
## cbcl_scr_syn_internal_r ~ lOFC_rvsn_ant_z + interview_age
##
## Parametric coefficients:
##                 Estimate Std. Error t value Pr(>|t|)
## (Intercept)      2.62529    1.91357   1.372    0.170
## lOFC_rvsn_ant_z -0.10772    0.19356  -0.557    0.578
## interview_age    0.01966    0.01592   1.235    0.217
##
##
## R-sq.(adj) =  -0.00122
## lmer.REML =  12706  Scale est. = 13.821    n = 2036
```

```
##                      stdcoef      stdse
## X(Intercept)      0.00000000 0.00000000
## XlOFC_rvsn_ant_z -0.01198528 0.02153614
## Xinterview_age    0.02697017 0.02184451
```

```
##
## Family: gaussian
## Link function: identity
##
## Formula:
## cbcl_scr_syn_internal_r ~ mOFC_rvsn_ant_z + interview_age
##
## Parametric coefficients:
##                 Estimate Std. Error t value Pr(>|t|)
## (Intercept)      2.51935    1.92180   1.311    0.190
## mOFC_rvsn_ant_z -0.12792    0.16826  -0.760    0.447
## interview_age    0.02059    0.01600   1.287    0.198
##
##
## R-sq.(adj) =  -0.0012
## lmer.REML =  12765  Scale est. = 13.816    n = 2042
```

```
##                      stdcoef      stdse
## X(Intercept)      0.00000000 0.00000000
## XmOFC_rvsn_ant_z -0.01623520 0.02135579
## Xinterview_age    0.02803578 0.02178658
```

3.8 Model: CBCL internalizing factor ~ OFC activity (feedback stage)

Female participants

```
##
## Family: gaussian
## Link function: identity
##
## Formula:
## cbcl_scr_syn_internal_r ~ lOFC_posvsneg_feedback_z + interview_age
##
## Parametric coefficients:
##                           Estimate Std. Error t value Pr(>|t|)
## (Intercept)                0.98560    1.89246   0.521   0.6026
## lOFC_posvsneg_feedback_z -0.04673    0.23076  -0.203   0.8395
## interview_age              0.03302    0.01579   2.091   0.0366 *
## ---
## Signif. codes:  0 '***' 0.001 '**' 0.01 '*' 0.05 '.' 0.1 ' ' 1
##
##
## R-sq.(adj) =  -0.000146
## lmer.REML =  12779  Scale est. = 16.099    n = 2065
```

```
##                                stdcoef      stdse
## X(Intercept)               0.000000000 0.00000000
## XlOFC_posvsneg_feedback_z -0.004375458 0.02160700
## Xinterview_age             0.045862853 0.02192932
```

```
##
## Family: gaussian
## Link function: identity
##
## Formula:
## cbcl_scr_syn_internal_r ~ mOFC_posvsneg_feedback_z + interview_age
##
## Parametric coefficients:
##                           Estimate Std. Error t value Pr(>|t|)
## (Intercept)                0.92801    1.89200   0.490   0.6238
## mOFC_posvsneg_feedback_z  0.20371    0.19485   1.046   0.2959
## interview_age              0.03360    0.01578   2.129   0.0334 *
## ---
## Signif. codes:  0 '***' 0.001 '**' 0.01 '*' 0.05 '.' 0.1 ' ' 1
##
##
## R-sq.(adj) =  0.000549
## lmer.REML =  12810  Scale est. = 15.903    n = 2069
```

```
##                              stdcoef      stdse
## X(Intercept)              0.00000000 0.00000000
## XmOFC_posvsneg_feedback_z 0.02252826 0.02154774
## Xinterview_age            0.04658350 0.02187988
```

Male participants

```
##
## Family: gaussian
## Link function: identity
##
## Formula:
## cbcl_scr_syn_internal_r ~ lOFC_posvsneg_feedback_z + interview_age
##
## Parametric coefficients:
##                          Estimate Std. Error t value Pr(>|t|)
## (Intercept)               3.12278    1.93031   1.618    0.106
## lOFC_posvsneg_feedback_z  0.15275    0.22303   0.685    0.493
## interview_age             0.01593    0.01608   0.991    0.322
##
##
## R-sq.(adj) =  -0.00122
## lmer.REML =  12729  Scale est. = 14.972    n = 2035
```

```
##                              stdcoef      stdse
## X(Intercept)              0.00000000 0.00000000
## XlOFC_posvsneg_feedback_z 0.01493689 0.02180927
## Xinterview_age            0.02171403 0.02191158
```

```
##
## Family: gaussian
## Link function: identity
##
## Formula:
## cbcl_scr_syn_internal_r ~ mOFC_posvsneg_feedback_z + interview_age
##
## Parametric coefficients:
##                          Estimate Std. Error t value Pr(>|t|)
## (Intercept)               3.07567    1.92215   1.600    0.110
## mOFC_posvsneg_feedback_z  0.06058    0.18722   0.324    0.746
## interview_age             0.01633    0.01601   1.020    0.308
##
##
## R-sq.(adj) =  -0.00121
## lmer.REML =  12780  Scale est. = 14.999    n = 2044
```

```
##                               stdcoef      stdse
## X(Intercept)              0.000000000 0.00000000
## XmOFC_posvsneg_feedback_z 0.007034218 0.02174054
## Xinterview_age            0.022296228 0.02186698
```

3.9 Model: CBCL internalizing factor ~ BIS-BAS-RR

Female participants

```
##
## Family: gaussian
```

```
## Link function: identity
##
## Formula:
## cbcl_scr_syn_internal_r ~ bisbas_ss_basm_rr + interview_age
##
## Parametric coefficients:
##                    Estimate Std. Error t value Pr(>|t|)
## (Intercept)         1.21645    1.72886   0.704   0.4817
## bisbas_ss_basm_rr -0.02712    0.04321  -0.628   0.5303
## interview_age      0.03358    0.01401   2.398   0.0166 *
## ---
## Signif. codes:  0 '***' 0.001 '**' 0.01 '*' 0.05 '.' 0.1 ' ' 1
##
##
## R-sq.(adj) =  -0.000264
## lmer.REML =  16599  Scale est. = 17.025    n = 2681
```

```
##                         stdcoef      stdse
## X(Intercept)        0.00000000 0.00000000
## Xbisbas_ss_basm_rr -0.01201656 0.01914694
## Xinterview_age      0.04625226 0.01929145
```

Male participants

```
##
## Family: gaussian
## Link function: identity
##
## Formula:
## cbcl_scr_syn_internal_r ~ bisbas_ss_basm_rr + interview_age
##
## Parametric coefficients:
##                    Estimate Std. Error t value Pr(>|t|)
## (Intercept)         3.11239    1.69534   1.836   0.0665 .
## bisbas_ss_basm_rr -0.06658    0.04448  -1.497   0.1346
## interview_age      0.02135    0.01374   1.554   0.1203
## ---
## Signif. codes:  0 '***' 0.001 '**' 0.01 '*' 0.05 '.' 0.1 ' ' 1
##
##
## R-sq.(adj) =  -0.000597
## lmer.REML =  18107  Scale est. = 16.736    n = 2893
```

```
##                         stdcoef      stdse
## X(Intercept)        0.00000000 0.00000000
## Xbisbas_ss_basm_rr -0.02744649 0.01833863
## Xinterview_age      0.02873301 0.01848803
```

3.10 Model: CBCL internalizing factor ~ MID Reaction Time

Female participants

```
##
```

```
## Family: gaussian
## Link function: identity
##
## Formula:
## cbcl_scr_syn_internal_r ~ rt_diff_large_neutral_z + interview_age
##
## Parametric coefficients:
##                         Estimate Std. Error t value Pr(>|t|)
## (Intercept)              0.65045    1.84166   0.353   0.7240
## rt_diff_large_neutral_z  0.10720    0.12129   0.884   0.3769
## interview_age            0.03602    0.01536   2.346   0.0191 *
## ---
## Signif. codes:  0 '***' 0.001 '**' 0.01 '*' 0.05 '.' 0.1 ' ' 1
##
##
## R-sq.(adj) =  0.000286
## lmer.REML =  13864  Scale est. = 16.79     n = 2237
```

```
##                              stdcoef      stdse
## X(Intercept)             0.00000000 0.00000000
## Xrt_diff_large_neutral_z 0.01831347 0.02072078
## Xinterview_age           0.04932191 0.02102775
```

```
##
## Family: gaussian
## Link function: identity
##
## Formula:
## cbcl_scr_syn_internal_r ~ rt_diff_large_small_z + interview_age
##
## Parametric coefficients:
##                       Estimate Std. Error t value Pr(>|t|)
## (Intercept)            0.65522    1.84051   0.356   0.7219
## rt_diff_large_small_z  0.14840    0.11684   1.270   0.2042
## interview_age          0.03601    0.01535   2.346   0.0191 *
## ---
## Signif. codes:  0 '***' 0.001 '**' 0.01 '*' 0.05 '.' 0.1 ' ' 1
##
##
## R-sq.(adj) =  0.000654
## lmer.REML =  13864  Scale est. = 16.809    n = 2237
```

```
##                            stdcoef      stdse
## X(Intercept)           0.00000000 0.00000000
## Xrt_diff_large_small_z 0.02637377 0.02076472
## Xinterview_age         0.04929782 0.02101228
```

Male participants

```
##
## Family: gaussian
## Link function: identity
##
```

```
## Formula:
## cbcl_scr_syn_internal_r ~ rt_diff_large_neutral_z + interview_age
##
## Parametric coefficients:
##                          Estimate Std. Error t value Pr(>|t|)
## (Intercept)               2.27829    1.81447   1.256    0.209
## rt_diff_large_neutral_z -0.09028    0.12278  -0.735    0.462
## interview_age             0.02284    0.01512   1.511    0.131
##
##
## R-sq.(adj) =  -0.00078
## lmer.REML =  14407  Scale est. = 13.459    n = 2304

##                              stdcoef      stdse
## X(Intercept)               0.00000000 0.00000000
## Xrt_diff_large_neutral_z -0.01487991 0.02023669
## Xinterview_age             0.03106944 0.02055981
```

```
##
## Family: gaussian
## Link function: identity
##
## Formula:
## cbcl_scr_syn_internal_r ~ rt_diff_large_small_z + interview_age
##
## Parametric coefficients:
##                        Estimate Std. Error t value Pr(>|t|)
## (Intercept)             2.32101    1.81284   1.280    0.201
## rt_diff_large_small_z -0.12010    0.12369  -0.971    0.332
## interview_age           0.02249    0.01510   1.489    0.137
##
##
## R-sq.(adj) =  -0.000713
## lmer.REML =  14407  Scale est. = 13.408    n = 2304

##                            stdcoef      stdse
## X(Intercept)             0.00000000 0.00000000
## Xrt_diff_large_small_z -0.01958396 0.02016891
## Xinterview_age           0.03058289 0.02054226
```

4—Internalizing~Puberty x Reward—

4.1 Model: CBCL internalizing factor ~ PDS x Accumbens activity (anticipation stage)

Female participants

```
##
## Family: gaussian
## Link function: identity
##
## Formula:
## cbcl_scr_syn_internal_r ~ PDS_score * accumbens_rvsn_ant_z +
##     race.ethnicity.5level + demo_race_hispanic + interview_age
##
## Parametric coefficients:
##                             Estimate Std. Error t value Pr(>|t|)
## (Intercept)                  0.77200    2.04095   0.378    0.705
## PDS_score                    0.93801    0.17923   5.234 1.84e-07 ***
## accumbens_rvsn_ant_z         0.13655    0.41196   0.331    0.740
## race.ethnicity.5levelBlack  -0.31095    0.79209  -0.393    0.695
## race.ethnicity.5levelMixed   0.99979    0.78464   1.274    0.203
## race.ethnicity.5levelOther   0.31596    0.92933   0.340    0.734
## race.ethnicity.5levelWhite   1.14295    0.72391   1.579    0.115
## demo_race_hispanic1          0.10693    0.35665   0.300    0.764
## interview_age                0.01433    0.01658   0.864    0.388
## PDS_score:accumbens_rvsn_ant_z -0.21652    0.22244  -0.973    0.330
## ---
## Signif. codes:  0 '***' 0.001 '**' 0.01 '*' 0.05 '.' 0.1 ' ' 1
##
##
## R-sq.(adj) =  0.0181
## lmer.REML =  12381  Scale est. = 15.847    n = 2010
```

```
##                                    stdcoef      stdse
## X(Intercept)                   0.000000000 0.00000000
## XPDS_score                     0.127119177 0.02428869
## Xaccumbens_rvsn_ant_z          0.017787600 0.05366165
## Xrace.ethnicity.5levelBlack   -0.020254759 0.05159550
## Xrace.ethnicity.5levelMixed    0.061024772 0.04789274
## Xrace.ethnicity.5levelOther    0.012402564 0.03648015
## Xrace.ethnicity.5levelWhite    0.100857757 0.06388025
## Xdemo_race_hispanic1           0.007846373 0.02616993
## Xinterview_age                 0.019981142 0.02312865
## XPDS_score:accumbens_rvsn_ant_z -0.052221566 0.05364977
```

Male participants

```
##
## Family: gaussian
## Link function: identity
##
```

```
## Formula:
## cbcl_scr_syn_internal_r ~ PDS_score * accumbens_rvsn_ant_z +
##     race.ethnicity.5level + demo_race_hispanic + interview_age
##
## Parametric coefficients:
##                                Estimate Std. Error t value Pr(>|t|)
## (Intercept)                    2.666877   2.176336   1.225 0.220570
## PDS_score                      0.891773   0.244024   3.654 0.000264 ***
## accumbens_rvsn_ant_z           0.346946   0.415334   0.835 0.403625
## race.ethnicity.5levelBlack    -0.441454   1.005029  -0.439 0.660532
## race.ethnicity.5levelMixed     0.547922   0.994986   0.551 0.581913
## race.ethnicity.5levelOther    -0.837758   1.109548  -0.755 0.450312
## race.ethnicity.5levelWhite     0.171422   0.936106   0.183 0.854721
## demo_race_hispanic1            0.910656   0.377863   2.410 0.016042 *
## interview_age                  0.007004   0.016364   0.428 0.668713
## PDS_score:accumbens_rvsn_ant_z -0.242806   0.278015  -0.873 0.382576
## ---
## Signif. codes:  0 '***' 0.001 '**' 0.01 '*' 0.05 '.' 0.1 ' ' 1
##
##
## R-sq.(adj) =  0.00704
## lmer.REML =  12495  Scale est. = 15.147    n = 2000
```

```
##                                     stdcoef      stdse
## X(Intercept)                     0.000000000 0.00000000
## XPDS_score                       0.084911659 0.02323514
## Xaccumbens_rvsn_ant_z            0.044937997 0.05379588
## Xrace.ethnicity.5levelBlack     -0.024688729 0.05620726
## Xrace.ethnicity.5levelMixed      0.031478540 0.05716269
## Xrace.ethnicity.5levelOther     -0.033246549 0.04403260
## Xrace.ethnicity.5levelWhite      0.014007340 0.07649165
## Xdemo_race_hispanic1             0.064614934 0.02681100
## Xinterview_age                   0.009527796 0.02226232
## XPDS_score:accumbens_rvsn_ant_z -0.047002510 0.05381839
```

4.2 Model: CBCL internalizing factor ~ PDS x Caudate activity (anticipation stage)

Female participants

```
##
## Family: gaussian
## Link function: identity
##
## Formula:
## cbcl_scr_syn_internal_r ~ PDS_score * caudate_rvsn_ant_z + race.ethnicity.5level +
##     demo_race_hispanic + interview_age
##
## Parametric coefficients:
##                             Estimate Std. Error t value Pr(>|t|)
## (Intercept)                  0.95469    2.03804   0.468   0.6395
## PDS_score                    0.91368    0.17921   5.098 3.75e-07 ***
## caudate_rvsn_ant_z           0.74877    0.33308   2.248   0.0247 *
```

```
## race.ethnicity.5levelBlack   -0.24603    0.79297  -0.310   0.7564
## race.ethnicity.5levelMixed    1.03899    0.78470   1.324   0.1856
## race.ethnicity.5levelOther    0.36634    0.93107   0.393   0.6940
## race.ethnicity.5levelWhite    1.21556    0.72461   1.678   0.0936 .
## demo_race_hispanic1           0.10129    0.35622   0.284   0.7762
## interview_age                 0.01267    0.01657   0.765   0.4444
## PDS_score:caudate_rvsn_ant_z -0.44212    0.18304  -2.415   0.0158 *
## ---
## Signif. codes:  0 '***' 0.001 '**' 0.01 '*' 0.05 '.' 0.1 ' ' 1
##
##
## R-sq.(adj) =  0.0188
## lmer.REML =  12417  Scale est. = 16.384    n = 2014
```

```
##                                    stdcoef      stdse
## X(Intercept)                    0.00000000 0.00000000
## XPDS_score                      0.12347381 0.02421871
## Xcaudate_rvsn_ant_z             0.12385070 0.05509295
## Xrace.ethnicity.5levelBlack    -0.01602007 0.05163382
## Xrace.ethnicity.5levelMixed     0.06375643 0.04815204
## Xrace.ethnicity.5levelOther     0.01425937 0.03624059
## Xrace.ethnicity.5levelWhite     0.10717664 0.06388924
## Xdemo_race_hispanic1            0.00743021 0.02613229
## Xinterview_age                  0.01763725 0.02305587
## XPDS_score:caudate_rvsn_ant_z -0.13340625 0.05523115
```

Male participants

```
##
## Family: gaussian
## Link function: identity
##
## Formula:
## cbcl_scr_syn_internal_r ~ PDS_score * caudate_rvsn_ant_z + race.ethnicity.5level +
##     demo_race_hispanic + interview_age
##
## Parametric coefficients:
##                               Estimate Std. Error t value Pr(>|t|)
## (Intercept)                   2.548908   2.176417   1.171 0.241679
## PDS_score                     0.892114   0.245847   3.629 0.000292 ***
## caudate_rvsn_ant_z           -0.134726   0.361758  -0.372 0.709620
## race.ethnicity.5levelBlack   -0.335689   1.006486  -0.334 0.738772
## race.ethnicity.5levelMixed    0.631809   0.997021   0.634 0.526351
## race.ethnicity.5levelOther   -0.922835   1.111439  -0.830 0.406465
## race.ethnicity.5levelWhite    0.241007   0.938854   0.257 0.797435
## demo_race_hispanic1           0.999729   0.381293   2.622 0.008809 **
## interview_age                 0.007682   0.016379   0.469 0.639090
## PDS_score:caudate_rvsn_ant_z  0.224536   0.253033   0.887 0.374981
## ---
## Signif. codes:  0 '***' 0.001 '**' 0.01 '*' 0.05 '.' 0.1 ' ' 1
##
##
## R-sq.(adj) =  0.00829
```

```
## lmer.REML =  12527  Scale est. = 14.127    n = 2004
```

```
##                                   stdcoef      stdse
## X(Intercept)                   0.00000000 0.00000000
## XPDS_score                     0.08396577 0.02313908
## Xcaudate_rvsn_ant_z           -0.02193078 0.05888723
## Xrace.ethnicity.5levelBlack   -0.01885254 0.05652497
## Xrace.ethnicity.5levelMixed    0.03629794 0.05727961
## Xrace.ethnicity.5levelOther   -0.03681492 0.04433896
## Xrace.ethnicity.5levelWhite    0.01970178 0.07674932
## Xdemo_race_hispanic1           0.07033827 0.02682675
## Xinterview_age                 0.01041830 0.02221182
## XPDS_score:caudate_rvsn_ant_z  0.05220388 0.05882923
```

4.3 Model: CBCL internalizing factor ~ PDS x Putamen activity (anticipation stage)

Female participants

```
##
## Family: gaussian
## Link function: identity
##
## Formula:
## cbcl_scr_syn_internal_r ~ PDS_score * putamen_rvsn_ant_z + race.ethnicity.5level +
##     demo_race_hispanic + interview_age
##
## Parametric coefficients:
##                             Estimate Std. Error t value Pr(>|t|)
## (Intercept)                  0.85569    2.03719   0.420   0.6745
## PDS_score                    0.92594    0.17910   5.170 2.57e-07 ***
## putamen_rvsn_ant_z           0.51517    0.33779   1.525   0.1274
## race.ethnicity.5levelBlack  -0.27463    0.79368  -0.346   0.7294
## race.ethnicity.5levelMixed   1.01544    0.78561   1.293   0.1963
## race.ethnicity.5levelOther   0.31711    0.93009   0.341   0.7332
## race.ethnicity.5levelWhite   1.21262    0.72506   1.672   0.0946 .
## demo_race_hispanic1          0.09662    0.35647   0.271   0.7864
## interview_age                0.01346    0.01656   0.813   0.4164
## PDS_score:putamen_rvsn_ant_z -0.33513    0.18396  -1.822   0.0686 .
## ---
## Signif. codes:  0 '***' 0.001 '**' 0.01 '*' 0.05 '.' 0.1 ' ' 1
##
##
## R-sq.(adj) =  0.018
## lmer.REML =  12418  Scale est. = 16.013    n = 2014
```

```
##                                    stdcoef      stdse
## X(Intercept)                   0.000000000 0.00000000
## XPDS_score                     0.125377667 0.02425075
## Xputamen_rvsn_ant_z            0.084458007 0.05537762
## Xrace.ethnicity.5levelBlack   -0.017858278 0.05161025
## Xrace.ethnicity.5levelMixed    0.062210629 0.04813059
## Xrace.ethnicity.5levelOther    0.012407145 0.03639053
```

```
## Xrace.ethnicity.5levelWhite    0.106891235 0.06391322
## Xdemo_race_hispanic1           0.007095542 0.02617838
## Xinterview_age                 0.018743826 0.02305931
## XPDS_score:putamen_rvsn_ant_z -0.100923737 0.05539811
```

Male participants

```
##
## Family: gaussian
## Link function: identity
##
## Formula:
## cbcl_scr_syn_internal_r ~ PDS_score * putamen_rvsn_ant_z + race.ethnicity.5level +
##     demo_race_hispanic + interview_age
##
## Parametric coefficients:
##                               Estimate Std. Error t value Pr(>|t|)
## (Intercept)                   2.772465   2.178555   1.273 0.203303
## PDS_score                     0.923913   0.246281   3.751 0.000181 ***
## putamen_rvsn_ant_z            0.263098   0.372731   0.706 0.480354
## race.ethnicity.5levelBlack   -0.414334   1.006452  -0.412 0.680620
## race.ethnicity.5levelMixed    0.631934   0.996600   0.634 0.526095
## race.ethnicity.5levelOther   -0.848368   1.112611  -0.763 0.445850
## race.ethnicity.5levelWhite    0.233564   0.938729   0.249 0.803535
## demo_race_hispanic1           0.939536   0.377969   2.486 0.013009 *
## interview_age                 0.005547   0.016391   0.338 0.735066
## PDS_score:putamen_rvsn_ant_z -0.212775   0.256544  -0.829 0.406984
## ---
## Signif. codes:  0 '***' 0.001 '**' 0.01 '*' 0.05 '.' 0.1 ' ' 1
##
##
## R-sq.(adj) =  0.00788
## lmer.REML =  12520  Scale est. = 14.196    n = 2003
```

```
##                                    stdcoef      stdse
## X(Intercept)                   0.000000000 0.00000000
## XPDS_score                     0.087043292 0.02320250
## Xputamen_rvsn_ant_z            0.042199778 0.05978439
## Xrace.ethnicity.5levelBlack   -0.023249208 0.05647435
## Xrace.ethnicity.5levelMixed    0.036411372 0.05742299
## Xrace.ethnicity.5levelOther   -0.033730505 0.04423659
## Xrace.ethnicity.5levelWhite    0.019103737 0.07678093
## Xdemo_race_hispanic1           0.066367952 0.02669942
## Xinterview_age                 0.007519015 0.02221661
## XPDS_score:putamen_rvsn_ant_z -0.049532787 0.05972202
```

4.4 Model: CBCL internalizing factor ~ PDS x Lateral OFC activity (anticipation stage)

Female participants

```
##
```

```
## Family: gaussian
## Link function: identity
##
## Formula:
## cbcl_scr_syn_internal_r ~ PDS_score * lOFC_rvsn_ant_z + race.ethnicity.5level +
##     demo_race_hispanic + interview_age
##
## Parametric coefficients:
##                           Estimate Std. Error t value Pr(>|t|)
## (Intercept)                0.54072    2.05077   0.264   0.7921
## PDS_score                  0.88175    0.18133   4.863 1.25e-06 ***
## lOFC_rvsn_ant_z            0.62047    0.49823   1.245   0.2132
## race.ethnicity.5levelBlack  0.03954    0.80302   0.049   0.9607
## race.ethnicity.5levelMixed  1.30077    0.79355   1.639   0.1013
## race.ethnicity.5levelOther  0.67842    0.93727   0.724   0.4693
## race.ethnicity.5levelWhite  1.46816    0.73318   2.002   0.0454 *
## demo_race_hispanic1         0.08453    0.35856   0.236   0.8136
## interview_age               0.01455    0.01665   0.874   0.3822
## PDS_score:lOFC_rvsn_ant_z  -0.36763    0.27263  -1.348   0.1777
## ---
## Signif. codes:  0 '***' 0.001 '**' 0.01 '*' 0.05 '.' 0.1 ' ' 1
##
##
## R-sq.(adj) =  0.0157
## lmer.REML =  12340  Scale est. = 15.727    n = 2001
```

```
##                                  stdcoef      stdse
## X(Intercept)                0.000000000 0.00000000
## XPDS_score                  0.118436912 0.02435617
## XlOFC_rvsn_ant_z            0.064859093 0.05208107
## Xrace.ethnicity.5levelBlack  0.002547559 0.05173774
## Xrace.ethnicity.5levelMixed  0.079484259 0.04849050
## Xrace.ethnicity.5levelOther  0.026619579 0.03677622
## Xrace.ethnicity.5levelWhite  0.129048318 0.06444503
## Xdemo_race_hispanic1        0.006197629 0.02628834
## Xinterview_age              0.020256001 0.02317621
## XPDS_score:lOFC_rvsn_ant_z  -0.070350335 0.05217059
```

Male participants

```
##
## Family: gaussian
## Link function: identity
##
## Formula:
## cbcl_scr_syn_internal_r ~ PDS_score * lOFC_rvsn_ant_z + race.ethnicity.5level +
##     demo_race_hispanic + interview_age
##
## Parametric coefficients:
##                           Estimate Std. Error t value Pr(>|t|)
## (Intercept)                2.42026    2.17280   1.114  0.26546
## PDS_score                  0.78810    0.24603   3.203  0.00138 **
## lOFC_rvsn_ant_z           -0.10417    0.52320  -0.199  0.84220
```

```
## race.ethnicity.5levelBlack -0.50068    1.01215  -0.495  0.62089
## race.ethnicity.5levelMixed  0.62900    1.00171   0.628  0.53012
## race.ethnicity.5levelOther -0.92145    1.11086  -0.829  0.40692
## race.ethnicity.5levelWhite  0.17931    0.94371   0.190  0.84932
## demo_race_hispanic1         0.95081    0.37923   2.507  0.01225 *
## interview_age               0.01009    0.01629   0.619  0.53589
## PDS_score:lOFC_rvsn_ant_z  -0.02032    0.36631  -0.055  0.95577
## ---
## Signif. codes:  0 '***' 0.001 '**' 0.01 '*' 0.05 '.' 0.1 ' ' 1
##
##
## R-sq.(adj) =  0.00612
## lmer.REML =  12395  Scale est. = 14.017    n = 1989
```

```
##                                  stdcoef      stdse
## X(Intercept)                 0.000000000 0.00000000
## XPDS_score                   0.074270145 0.02318588
## XlOFC_rvsn_ant_z            -0.011500392 0.05776100
## Xrace.ethnicity.5levelBlack -0.027904409 0.05641091
## Xrace.ethnicity.5levelMixed  0.036383272 0.05794174
## Xrace.ethnicity.5levelOther -0.037392582 0.04507855
## Xrace.ethnicity.5levelWhite  0.014731840 0.07753226
## Xdemo_race_hispanic1         0.068029704 0.02713368
## Xinterview_age               0.013801858 0.02229147
## XPDS_score:lOFC_rvsn_ant_z  -0.003189956 0.05750798
```

4.5 Model: CBCL internalizing factor ~ PDS x Medial OFC activity (anticipation stage)

Female participants

```
##
## Family: gaussian
## Link function: identity
##
## Formula:
## cbcl_scr_syn_internal_r ~ PDS_score * mOFC_rvsn_ant_z + race.ethnicity.5level +
##     demo_race_hispanic + interview_age
##
## Parametric coefficients:
##                             Estimate Std. Error t value Pr(>|t|)
## (Intercept)                  0.42146    2.04914   0.206   0.8371
## PDS_score                    0.88874    0.18142   4.899 1.04e-06 ***
## mOFC_rvsn_ant_z              0.71722    0.43302   1.656   0.0978 .
## race.ethnicity.5levelBlack   0.04807    0.80239   0.060   0.9522
## race.ethnicity.5levelMixed   1.30840    0.79473   1.646   0.0999 .
## race.ethnicity.5levelOther   0.69313    0.93547   0.741   0.4588
## race.ethnicity.5levelWhite   1.52006    0.73372   2.072   0.0384 *
## demo_race_hispanic1          0.07873    0.35818   0.220   0.8260
## interview_age                0.01508    0.01662   0.907   0.3645
## PDS_score:mOFC_rvsn_ant_z   -0.28976    0.23681  -1.224   0.2212
## ---
## Signif. codes:  0 '***' 0.001 '**' 0.01 '*' 0.05 '.' 0.1 ' ' 1
```

```
##
##
## R-sq.(adj) =  0.0171
## lmer.REML =  12345  Scale est. = 15.852    n = 2002
```

```
##                                   stdcoef      stdse
## X(Intercept)                  0.000000000 0.00000000
## XPDS_score                    0.119855558 0.02446675
## XmOFC_rvsn_ant_z              0.086273433 0.05208738
## Xrace.ethnicity.5levelBlack   0.003114671 0.05199474
## Xrace.ethnicity.5levelMixed   0.079798456 0.04847020
## Xrace.ethnicity.5levelOther   0.027328765 0.03688364
## Xrace.ethnicity.5levelWhite   0.133788558 0.06457867
## Xdemo_race_hispanic1          0.005777241 0.02628198
## Xinterview_age                0.020961492 0.02310778
## XPDS_score:mOFC_rvsn_ant_z   -0.063775183 0.05211967
```

Male participants

```
##
## Family: gaussian
## Link function: identity
##
## Formula:
## cbcl_scr_syn_internal_r ~ PDS_score * mOFC_rvsn_ant_z + race.ethnicity.5level +
##     demo_race_hispanic + interview_age
##
## Parametric coefficients:
##                             Estimate Std. Error t value Pr(>|t|)
## (Intercept)                 2.395114   2.178907   1.099 0.271802
## PDS_score                   0.883907   0.246051   3.592 0.000336 ***
## mOFC_rvsn_ant_z             0.585278   0.429236   1.364 0.172869
## race.ethnicity.5levelBlack -0.401830   1.014840  -0.396 0.692181
## race.ethnicity.5levelMixed  0.565238   1.004735   0.563 0.573789
## race.ethnicity.5levelOther -0.911887   1.115171  -0.818 0.413621
## race.ethnicity.5levelWhite  0.141791   0.947167   0.150 0.881016
## demo_race_hispanic1         0.915149   0.378878   2.415 0.015807 *
## interview_age               0.009584   0.016346   0.586 0.557713
## PDS_score:mOFC_rvsn_ant_z  -0.569479   0.300359  -1.896 0.058106 .
## ---
## Signif. codes:  0 '***' 0.001 '**' 0.01 '*' 0.05 '.' 0.1 ' ' 1
##
##
## R-sq.(adj) =  0.00913
## lmer.REML =  12456  Scale est. = 14.066    n = 1996
```

```
##                                  stdcoef      stdse
## X(Intercept)                  0.00000000 0.00000000
## XPDS_score                    0.08311883 0.02313761
## XmOFC_rvsn_ant_z              0.07421407 0.05442772
## Xrace.ethnicity.5levelBlack -0.02239482 0.05655907
## Xrace.ethnicity.5levelMixed  0.03273597 0.05818964
## Xrace.ethnicity.5levelOther -0.03677357 0.04497139
```

```
## Xrace.ethnicity.5levelWhite  0.01162335 0.07764427
## Xdemo_race_hispanic1         0.06502862 0.02692229
## Xinterview_age               0.01302008 0.02220563
## XPDS_score:mOFC_rvsn_ant_z  -0.10403604 0.05487148
```

4.6 Model: CBCL internalizing factor ~ PDS x Accumbens activity (feedback)

Female participants

```
##
## Family: gaussian
## Link function: identity
##
## Formula:
## cbcl_scr_syn_internal_r ~ PDS_score * accumbens_posvsneg_feedback_z +
##     race.ethnicity.5level + demo_race_hispanic + interview_age
##
## Parametric coefficients:
##                                         Estimate Std. Error t value Pr(>|t|)
## (Intercept)                             0.732221   2.043733   0.358   0.7202
## PDS_score                               0.897986   0.180161   4.984 6.75e-07
## accumbens_posvsneg_feedback_z           0.258370   0.434089   0.595   0.5518
## race.ethnicity.5levelBlack             -0.009524   0.801237  -0.012   0.9905
## race.ethnicity.5levelMixed              1.305136   0.792490   1.647   0.0997
## race.ethnicity.5levelOther              0.607052   0.933486   0.650   0.5156
## race.ethnicity.5levelWhite              1.434905   0.731666   1.961   0.0500
## demo_race_hispanic1                     0.086535   0.358899   0.241   0.8095
## interview_age                           0.012923   0.016589   0.779   0.4361
## PDS_score:accumbens_posvsneg_feedback_z -0.034845   0.235193  -0.148   0.8822
##
## (Intercept)
## PDS_score                               ***
## accumbens_posvsneg_feedback_z
## race.ethnicity.5levelBlack
## race.ethnicity.5levelMixed              .
## race.ethnicity.5levelOther
## race.ethnicity.5levelWhite              .
## demo_race_hispanic1
## interview_age
## PDS_score:accumbens_posvsneg_feedback_z
## ---
## Signif. codes:  0 '***' 0.001 '**' 0.01 '*' 0.05 '.' 0.1 ' ' 1
##
##
## R-sq.(adj) =  0.0162
## lmer.REML =  12385  Scale est. = 15.95     n = 2009
```

```
##                                                 stdcoef      stdse
## X(Intercept)                               0.0000000000 0.00000000
## XPDS_score                                 0.1212657324 0.02432935
## Xaccumbens_posvsneg_feedback_z             0.0326603430 0.05487280
## Xrace.ethnicity.5levelBlack               -0.0006177801 0.05197459
## Xrace.ethnicity.5levelMixed                0.0798392540 0.04847910
```

```
## Xrace.ethnicity.5levelOther               0.0239201109 0.03678284
## Xrace.ethnicity.5levelWhite               0.1264516366 0.06447838
## Xdemo_race_hispanic1                      0.0063478867 0.02632745
## Xinterview_age                            0.0179954633 0.02310042
## XPDS_score:accumbens_posvsneg_feedback_z -0.0081468891 0.05498938
```

Male participants

```
##
## Family: gaussian
## Link function: identity
##
## Formula:
## cbcl_scr_syn_internal_r ~ PDS_score * accumbens_posvsneg_feedback_z +
##     race.ethnicity.5level + demo_race_hispanic + interview_age
##
## Parametric coefficients:
##                                           Estimate Std. Error t value Pr(>|t|)
## (Intercept)                               3.088756   2.190077   1.410 0.158595
## PDS_score                                 0.911899   0.244935   3.723 0.000202
## accumbens_posvsneg_feedback_z             0.272356   0.470768   0.579 0.562968
## race.ethnicity.5levelBlack               -0.500608   1.018583  -0.491 0.623145
## race.ethnicity.5levelMixed                0.623315   1.009298   0.618 0.536927
## race.ethnicity.5levelOther               -0.916791   1.122424  -0.817 0.414143
## race.ethnicity.5levelWhite                0.213376   0.951979   0.224 0.822671
## demo_race_hispanic1                       0.921473   0.378062   2.437 0.014882
## interview_age                             0.003495   0.016450   0.212 0.831784
## PDS_score:accumbens_posvsneg_feedback_z -0.381878   0.337199  -1.133 0.257560
##
## (Intercept)
## PDS_score                                ***
## accumbens_posvsneg_feedback_z
## race.ethnicity.5levelBlack
## race.ethnicity.5levelMixed
## race.ethnicity.5levelOther
## race.ethnicity.5levelWhite
## demo_race_hispanic1                      *
## interview_age
## PDS_score:accumbens_posvsneg_feedback_z
## ---
## Signif. codes:  0 '***' 0.001 '**' 0.01 '*' 0.05 '.' 0.1 ' ' 1
##
##
## R-sq.(adj) =  0.00903
## lmer.REML =  12526  Scale est. = 15.01     n = 2003
```

```
##                                               stdcoef      stdse
## X(Intercept)                               0.000000000 0.00000000
## XPDS_score                                 0.086151713 0.02314028
## Xaccumbens_posvsneg_feedback_z             0.035609316 0.06155072
## Xrace.ethnicity.5levelBlack               -0.028032881 0.05703832
## Xrace.ethnicity.5levelMixed                0.035841382 0.05803587
## Xrace.ethnicity.5levelOther               -0.036539695 0.04473539
```

```
## Xrace.ethnicity.5levelWhite               0.017416930 0.07770561
## Xdemo_race_hispanic1                      0.065210346 0.02675449
## Xinterview_age                            0.004721555 0.02222510
## XPDS_score:accumbens_posvsneg_feedback_z -0.069839189 0.06166814
```

4.7 Model: CBCL internalizing factor ~ PDS x Caudate activity (feedback)

Female participants

```
##
## Family: gaussian
## Link function: identity
##
## Formula:
## cbcl_scr_syn_internal_r ~ PDS_score * caudate_posvsneg_feedback_z +
##     race.ethnicity.5level + demo_race_hispanic + interview_age
##
## Parametric coefficients:
##                                      Estimate Std. Error t value Pr(>|t|)
## (Intercept)                           0.79441    2.04022   0.389   0.6970
## PDS_score                             0.90585    0.17932   5.052 4.78e-07 ***
## caudate_posvsneg_feedback_z          -0.56189    0.33878  -1.659   0.0974 .
## race.ethnicity.5levelBlack           -0.08439    0.80103  -0.105   0.9161
## race.ethnicity.5levelMixed            1.21615    0.79183   1.536   0.1247
## race.ethnicity.5levelOther            0.51918    0.93338   0.556   0.5781
## race.ethnicity.5levelWhite            1.43132    0.73161   1.956   0.0506 .
## demo_race_hispanic1                   0.15113    0.35791   0.422   0.6729
## interview_age                         0.01242    0.01655   0.751   0.4529
## PDS_score:caudate_posvsneg_feedback_z  0.29937    0.18504   1.618   0.1059
## ---
## Signif. codes:  0 '***' 0.001 '**' 0.01 '*' 0.05 '.' 0.1 ' ' 1
##
##
## R-sq.(adj) =  0.0173
## lmer.REML =  12390  Scale est. = 15.498    n = 2010
```

```
##                                          stdcoef      stdse
## X(Intercept)                          0.00000000 0.00000000
## XPDS_score                            0.12258013 0.02426561
## Xcaudate_posvsneg_feedback_z         -0.09080247 0.05474774
## Xrace.ethnicity.5levelBlack          -0.00547223 0.05193948
## Xrace.ethnicity.5levelMixed           0.07461985 0.04858489
## Xrace.ethnicity.5levelOther           0.02044871 0.03676229
## Xrace.ethnicity.5levelWhite           0.12618144 0.06449706
## Xdemo_race_hispanic1                  0.01111437 0.02632123
## Xinterview_age                        0.01729377 0.02303798
## XPDS_score:caudate_posvsneg_feedback_z  0.08862193 0.05477777
```

Male participants

```
##
## Family: gaussian
```

```
## Link function: identity
##
## Formula:
## cbcl_scr_syn_internal_r ~ PDS_score * caudate_posvsneg_feedback_z +
##     race.ethnicity.5level + demo_race_hispanic + interview_age
##
## Parametric coefficients:
##                                       Estimate Std. Error t value Pr(>|t|)
## (Intercept)                           3.149418   2.184828   1.441 0.149602
## PDS_score                             0.882852   0.245486   3.596 0.000331 ***
## caudate_posvsneg_feedback_z           0.218534   0.382566   0.571 0.567906
## race.ethnicity.5levelBlack           -0.476884   1.011376  -0.472 0.637321
## race.ethnicity.5levelMixed            0.581684   1.000476   0.581 0.561032
## race.ethnicity.5levelOther           -0.953144   1.114209  -0.855 0.392408
## race.ethnicity.5levelWhite            0.154671   0.943292   0.164 0.869772
## demo_race_hispanic1                   0.914551   0.376759   2.427 0.015295 *
## interview_age                         0.003504   0.016424   0.213 0.831063
## PDS_score:caudate_posvsneg_feedback_z -0.248130   0.270533  -0.917 0.359155
## ---
## Signif. codes:  0 '***' 0.001 '**' 0.01 '*' 0.05 '.' 0.1 ' ' 1
##
##
## R-sq.(adj) =  0.00736
## lmer.REML =  12526  Scale est. = 14.813    n = 2003
```

```
##                                            stdcoef      stdse
## X(Intercept)                           0.000000000 0.00000000
## XPDS_score                             0.083372880 0.02318265
## Xcaudate_posvsneg_feedback_z           0.034851101 0.06101036
## Xrace.ethnicity.5levelBlack           -0.026696869 0.05661868
## Xrace.ethnicity.5levelMixed            0.033625908 0.05783541
## Xrace.ethnicity.5levelOther           -0.038052581 0.04448280
## Xrace.ethnicity.5levelWhite            0.012652317 0.07716272
## Xdemo_race_hispanic1                   0.064891593 0.02673277
## Xinterview_age                         0.004747137 0.02224881
## XPDS_score:caudate_posvsneg_feedback_z -0.056128205 0.06119594
```

4.8 Model: CBCL internalizing factor ~ PDS x Putamen activity (feedback)

Female participants

```
##
## Family: gaussian
## Link function: identity
##
## Formula:
## cbcl_scr_syn_internal_r ~ PDS_score * putamen_posvsneg_feedback_z +
##     race.ethnicity.5level + demo_race_hispanic + interview_age
##
## Parametric coefficients:
##                                      Estimate Std. Error t value Pr(>|t|)
## (Intercept)                           1.16888    2.03565   0.574    0.566
## PDS_score                             0.95437    0.17954   5.316 1.18e-07 ***
```

```
## putamen_posvsneg_feedback_z           -0.56731    0.34726  -1.634    0.102
## race.ethnicity.5levelBlack            -0.32326    0.79406  -0.407    0.684
## race.ethnicity.5levelMixed             1.04020    0.78627   1.323    0.186
## race.ethnicity.5levelOther             0.28592    0.93012   0.307    0.759
## race.ethnicity.5levelWhite             1.21883    0.72519   1.681    0.093 .
## demo_race_hispanic1                    0.11739    0.35894   0.327    0.744
## interview_age                          0.01033    0.01654   0.624    0.533
## PDS_score:putamen_posvsneg_feedback_z  0.28452    0.18772   1.516    0.130
## ---
## Signif. codes:  0 '***' 0.001 '**' 0.01 '*' 0.05 '.' 0.1 ' ' 1
##
##
## R-sq.(adj) =  0.0176
## lmer.REML =  12393  Scale est. = 15.793    n = 2010
```

```
##                                            stdcoef      stdse
## X(Intercept)                            0.000000000 0.00000000
## XPDS_score                              0.128848608 0.02423901
## Xputamen_posvsneg_feedback_z           -0.090822656 0.05559326
## Xrace.ethnicity.5levelBlack            -0.020952420 0.05146770
## Xrace.ethnicity.5levelMixed             0.063580939 0.04805953
## Xrace.ethnicity.5levelOther             0.011200420 0.03643609
## Xrace.ethnicity.5levelWhite             0.107333389 0.06386231
## Xdemo_race_hispanic1                    0.008596085 0.02628434
## Xinterview_age                          0.014384024 0.02304566
## XPDS_score:putamen_posvsneg_feedback_z  0.084127939 0.05550767
```

Male participants

```
##
## Family: gaussian
## Link function: identity
##
## Formula:
## cbcl_scr_syn_internal_r ~ PDS_score * putamen_posvsneg_feedback_z +
##     race.ethnicity.5level + demo_race_hispanic + interview_age
##
## Parametric coefficients:
##                                        Estimate Std. Error t value Pr(>|t|)
## (Intercept)                            3.114722   2.185689   1.425 0.154298
## PDS_score                              0.869838   0.246327   3.531 0.000423 ***
## putamen_posvsneg_feedback_z            0.116507   0.375657   0.310 0.756484
## race.ethnicity.5levelBlack            -0.423730   1.012755  -0.418 0.675705
## race.ethnicity.5levelMixed             0.605647   1.001732   0.605 0.545514
## race.ethnicity.5levelOther            -0.932281   1.115438  -0.836 0.403369
## race.ethnicity.5levelWhite             0.180709   0.944051   0.191 0.848217
## demo_race_hispanic1                    0.864330   0.377408   2.290 0.022115 *
## interview_age                          0.003863   0.016427   0.235 0.814113
## PDS_score:putamen_posvsneg_feedback_z -0.099337   0.261995  -0.379 0.704611
## ---
## Signif. codes:  0 '***' 0.001 '**' 0.01 '*' 0.05 '.' 0.1 ' ' 1
##
##
```

```
## R-sq.(adj) =  0.00676
## lmer.REML =  12562  Scale est. = 15.264    n = 2008
```

```
##                                            stdcoef      stdse
## X(Intercept)                           0.000000000 0.00000000
## XPDS_score                             0.082262516 0.02329571
## Xputamen_posvsneg_feedback_z           0.018483930 0.05959801
## Xrace.ethnicity.5levelBlack           -0.023630623 0.05647947
## Xrace.ethnicity.5levelMixed            0.034946992 0.05780188
## Xrace.ethnicity.5levelOther           -0.037147754 0.04444586
## Xrace.ethnicity.5levelWhite            0.014753571 0.07707503
## Xdemo_race_hispanic1                   0.061165569 0.02670784
## Xinterview_age                         0.005232681 0.02225210
## XPDS_score:putamen_posvsneg_feedback_z -0.022644964 0.05972447
```

4.9 Model: CBCL internalizing factor ~ PDS x Lateral OFC activity (feedback stage)

Female participants

```
##
## Family: gaussian
## Link function: identity
##
## Formula:
## cbcl_scr_syn_internal_r ~ PDS_score * lOFC_posvsneg_feedback_z +
##     race.ethnicity.5level + demo_race_hispanic + interview_age
##
## Parametric coefficients:
##                                Estimate Std. Error t value Pr(>|t|)
## (Intercept)                     0.92701    2.03846   0.455    0.649
## PDS_score                       0.86852    0.18074   4.805 1.66e-06 ***
## lOFC_posvsneg_feedback_z       -0.31213    0.56443  -0.553    0.580
## race.ethnicity.5levelBlack     -0.19693    0.79360  -0.248    0.804
## race.ethnicity.5levelMixed      0.98903    0.78500   1.260    0.208
## race.ethnicity.5levelOther      0.28542    0.92850   0.307    0.759
## race.ethnicity.5levelWhite      1.18368    0.72395   1.635    0.102
## demo_race_hispanic1             0.12639    0.35805   0.353    0.724
## interview_age                   0.01364    0.01659   0.822    0.411
## PDS_score:lOFC_posvsneg_feedback_z  0.17292    0.30096   0.575    0.566
## ---
## Signif. codes:  0 '***' 0.001 '**' 0.01 '*' 0.05 '.' 0.1 ' ' 1
##
##
## R-sq.(adj) =  0.0137
## lmer.REML =  12386  Scale est. = 16.122    n = 2010
```

```
##                                        stdcoef      stdse
## X(Intercept)                       0.000000000 0.00000000
## XPDS_score                         0.117356547 0.02442173
## XlOFC_posvsneg_feedback_z         -0.029449881 0.05325517
## Xrace.ethnicity.5levelBlack       -0.012806405 0.05160925
## Xrace.ethnicity.5levelMixed        0.060654456 0.04814192
```

```
## Xrace.ethnicity.5levelOther          0.011218070 0.03649347
## Xrace.ethnicity.5levelWhite          0.104584521 0.06396463
## Xdemo_race_hispanic1                 0.009285686 0.02630585
## Xinterview_age                       0.019041564 0.02316785
## XPDS_score:lOFC_posvsneg_feedback_z  0.030636295 0.05332136
```

Male participants

```
##
## Family: gaussian
## Link function: identity
##
## Formula:
## cbcl_scr_syn_internal_r ~ PDS_score * lOFC_posvsneg_feedback_z +
##     race.ethnicity.5level + demo_race_hispanic + interview_age
##
## Parametric coefficients:
##                                     Estimate Std. Error t value Pr(>|t|)
## (Intercept)                         3.010802   2.186445   1.377 0.168658
## PDS_score                           0.840726   0.246796   3.407 0.000671 ***
## lOFC_posvsneg_feedback_z           -0.081849   0.568744  -0.144 0.885585
## race.ethnicity.5levelBlack         -0.454122   1.017440  -0.446 0.655402
## race.ethnicity.5levelMixed          0.588660   1.007080   0.585 0.558936
## race.ethnicity.5levelOther         -0.967371   1.117777  -0.865 0.386901
## race.ethnicity.5levelWhite          0.188614   0.948079   0.199 0.842328
## demo_race_hispanic1                 0.925754   0.379740   2.438 0.014862 *
## interview_age                       0.004985   0.016427   0.303 0.761556
## PDS_score:lOFC_posvsneg_feedback_z  0.202684   0.383118   0.529 0.596839
## ---
## Signif. codes:  0 '***' 0.001 '**' 0.01 '*' 0.05 '.' 0.1 ' ' 1
##
##
## R-sq.(adj) =  0.00696
## lmer.REML =  12423  Scale est. = 15.194    n = 1989
```

```
##                                          stdcoef      stdse
## X(Intercept)                         0.000000000 0.00000000
## XPDS_score                           0.079298130 0.02327800
## XlOFC_posvsneg_feedback_z           -0.007950519 0.05524589
## Xrace.ethnicity.5levelBlack         -0.025168324 0.05638851
## Xrace.ethnicity.5levelMixed          0.033796367 0.05781887
## Xrace.ethnicity.5levelOther         -0.038863949 0.04490651
## Xrace.ethnicity.5levelWhite          0.015394216 0.07737991
## Xdemo_race_hispanic1                 0.065739758 0.02696611
## Xinterview_age                       0.006778793 0.02233682
## XPDS_score:lOFC_posvsneg_feedback_z  0.029282421 0.05535043
```

4.10 Model: CBCL internalizing factor ~ PDS x Medial OFC activity (feedback stage)

Female participants

```
##
```

```
## Family: gaussian
## Link function: identity
##
## Formula:
## cbcl_scr_syn_internal_r ~ PDS_score * mOFC_posvsneg_feedback_z +
##     race.ethnicity.5level + demo_race_hispanic + interview_age
##
## Parametric coefficients:
##                                    Estimate Std. Error t value Pr(>|t|)
## (Intercept)                          0.74223    2.03903   0.364   0.7159
## PDS_score                            0.89080    0.18011   4.946 8.21e-07 ***
## mOFC_posvsneg_feedback_z            -0.49262    0.48540  -1.015   0.3103
## race.ethnicity.5levelBlack           0.05135    0.80109   0.064   0.9489
## race.ethnicity.5levelMixed           1.27777    0.79211   1.613   0.1069
## race.ethnicity.5levelOther           0.52956    0.93373   0.567   0.5707
## race.ethnicity.5levelWhite           1.44839    0.73158   1.980   0.0479 *
## demo_race_hispanic1                  0.09621    0.35804   0.269   0.7882
## interview_age                        0.01285    0.01655   0.776   0.4377
## PDS_score:mOFC_posvsneg_feedback_z   0.44003    0.25859   1.702   0.0890 .
## ---
## Signif. codes:  0 '***' 0.001 '**' 0.01 '*' 0.05 '.' 0.1 ' ' 1
##
##
## R-sq.(adj) =  0.0172
## lmer.REML =  12411  Scale est. = 15.619    n = 2014
```

```
##                                          stdcoef      stdse
## X(Intercept)                          0.000000000 0.00000000
## XPDS_score                            0.120039416 0.02427068
## XmOFC_posvsneg_feedback_z            -0.054628055 0.05382738
## Xrace.ethnicity.5levelBlack           0.003329440 0.05193869
## Xrace.ethnicity.5levelMixed           0.078391173 0.04859585
## Xrace.ethnicity.5levelOther           0.020748351 0.03658377
## Xrace.ethnicity.5levelWhite           0.127674847 0.06448833
## Xdemo_race_hispanic1                  0.007061548 0.02627893
## Xinterview_age                        0.017907568 0.02306809
## XPDS_score:mOFC_posvsneg_feedback_z   0.091385095 0.05370431
```

Male participants

```
##
## Family: gaussian
## Link function: identity
##
## Formula:
## cbcl_scr_syn_internal_r ~ PDS_score * mOFC_posvsneg_feedback_z +
##     race.ethnicity.5level + demo_race_hispanic + interview_age
##
## Parametric coefficients:
##                                     Estimate Std. Error t value Pr(>|t|)
## (Intercept)                         2.949915   2.179857   1.353 0.176126
## PDS_score                           0.844548   0.245622   3.438 0.000597 ***
## mOFC_posvsneg_feedback_z           -0.111886   0.488217  -0.229 0.818758
```

```
## race.ethnicity.5levelBlack         -0.473855   1.015260  -0.467 0.640742
## race.ethnicity.5levelMixed          0.629701   1.004520   0.627 0.530818
## race.ethnicity.5levelOther         -0.930313   1.114845  -0.834 0.404112
## race.ethnicity.5levelWhite          0.198803   0.946430   0.210 0.833646
## demo_race_hispanic1                 0.918843   0.376808   2.438 0.014836 *
## interview_age                       0.005365   0.016377   0.328 0.743249
## PDS_score:mOFC_posvsneg_feedback_z  0.145290   0.335872   0.433 0.665371
## ---
## Signif. codes:  0 '***' 0.001 '**' 0.01 '*' 0.05 '.' 0.1 ' ' 1
##
##
## R-sq.(adj) =  0.00706
## lmer.REML =  12474  Scale est. = 15.237    n = 1998
```

```
##                                          stdcoef      stdse
## X(Intercept)                         0.000000000 0.00000000
## XPDS_score                           0.079648310 0.02316428
## XmOFC_posvsneg_feedback_z           -0.012994356 0.05670094
## Xrace.ethnicity.5levelBlack         -0.026360187 0.05647815
## Xrace.ethnicity.5levelMixed          0.036342689 0.05797510
## Xrace.ethnicity.5levelOther         -0.037522282 0.04496501
## Xrace.ethnicity.5levelWhite          0.016278813 0.07749751
## Xdemo_race_hispanic1                 0.065495915 0.02685919
## Xinterview_age                       0.007307066 0.02230488
## XPDS_score:mOFC_posvsneg_feedback_z  0.024572161 0.05680454
```

4.11 Model: CBCL internalizing factor ~ PDS x BIS-BAS

Female participants

```
##
## Family: gaussian
## Link function: identity
##
## Formula:
## cbcl_scr_syn_internal_r ~ PDS_score * bisbas_ss_basm_rr + race.ethnicity.5level +
##     demo_race_hispanic + interview_age
##
## Parametric coefficients:
##                              Estimate Std. Error t value Pr(>|t|)
## (Intercept)                  -0.82007    2.00463  -0.409  0.68251
## PDS_score                     2.07922    0.54268   3.831  0.00013 ***
## bisbas_ss_basm_rr             0.22482    0.10746   2.092  0.03653 *
## race.ethnicity.5levelBlack   -0.20759    0.71808  -0.289  0.77253
## race.ethnicity.5levelMixed    1.23262    0.71707   1.719  0.08574 .
## race.ethnicity.5levelOther    0.33424    0.82728   0.404  0.68623
## race.ethnicity.5levelWhite    0.98373    0.66399   1.482  0.13858
## demo_race_hispanic1           0.25986    0.32109   0.809  0.41842
## interview_age                 0.01402    0.01471   0.953  0.34071
## PDS_score:bisbas_ss_basm_rr  -0.14543    0.05856  -2.484  0.01307 *
## ---
## Signif. codes:  0 '***' 0.001 '**' 0.01 '*' 0.05 '.' 0.1 ' ' 1
##
```

```
##
## R-sq.(adj) =  0.0143
## lmer.REML =  16130  Scale est. = 17.079    n = 2613
```

```
##                                 stdcoef      stdse
## X(Intercept)                 0.00000000 0.00000000
## XPDS_score                   0.28111483 0.07337140
## Xbisbas_ss_basm_rr           0.09975998 0.04768455
## Xrace.ethnicity.5levelBlack  -0.01425601 0.04931247
## Xrace.ethnicity.5levelMixed   0.07412920 0.04312407
## Xrace.ethnicity.5levelOther   0.01345996 0.03331444
## Xrace.ethnicity.5levelWhite   0.08754146 0.05908767
## Xdemo_race_hispanic1          0.01887378 0.02332109
## Xinterview_age                0.01935850 0.02031466
## XPDS_score:bisbas_ss_basm_rr -0.21498159 0.08656035
```

Male participants

```
##
## Family: gaussian
## Link function: identity
##
## Formula:
## cbcl_scr_syn_internal_r ~ PDS_score * bisbas_ss_basm_rr + race.ethnicity.5level +
##     demo_race_hispanic + interview_age
##
## Parametric coefficients:
##                             Estimate Std. Error t value Pr(>|t|)
## (Intercept)                  2.64736    2.12801   1.244   0.2136
## PDS_score                    1.30648    0.79354   1.646   0.0998 .
## bisbas_ss_basm_rr            0.02156    0.12021   0.179   0.8576
## race.ethnicity.5levelBlack  -0.90753    0.79294  -1.145   0.2525
## race.ethnicity.5levelMixed   0.41465    0.78773   0.526   0.5987
## race.ethnicity.5levelOther  -0.88728    0.88639  -1.001   0.3169
## race.ethnicity.5levelWhite  -0.06893    0.73750  -0.093   0.9255
## demo_race_hispanic1          0.57431    0.32411   1.772   0.0765 .
## interview_age                0.01074    0.01411   0.761   0.4467
## PDS_score:bisbas_ss_basm_rr -0.06485    0.08372  -0.775   0.4387
## ---
## Signif. codes:  0 '***' 0.001 '**' 0.01 '*' 0.05 '.' 0.1 ' ' 1
##
##
## R-sq.(adj) =  0.00583
## lmer.REML =  17629  Scale est. = 17.075    n = 2817
```

```
##                                  stdcoef      stdse
## X(Intercept)                 0.000000000 0.00000000
## XPDS_score                   0.129216578 0.07848416
## Xbisbas_ss_basm_rr           0.008865546 0.04942009
## Xrace.ethnicity.5levelBlack  -0.054536450 0.04765043
## Xrace.ethnicity.5levelMixed   0.024087437 0.04576047
## Xrace.ethnicity.5levelOther  -0.035299988 0.03526451
## Xrace.ethnicity.5levelWhite  -0.005759135 0.06162124
```

```
## Xdemo_race_hispanic1          0.040898100 0.02308067
## Xinterview_age                0.014411219 0.01893509
## XPDS_score:bisbas_ss_basm_rr -0.071488593 0.09229758
```

4.12 Model: CBCL internalizing factor ~ PDS x MID reaction time (large reward vs. neutral)

Female participants

```
##
## Family: gaussian
## Link function: identity
##
## Formula:
## cbcl_scr_syn_internal_r ~ PDS_score * rt_diff_large_neutral_z +
##     race.ethnicity.5level + demo_race_hispanic + interview_age
##
## Parametric coefficients:
##                               Estimate Std. Error t value Pr(>|t|)
## (Intercept)                    0.95585    1.97223   0.485    0.628
## PDS_score                      1.00003    0.17508   5.712 1.27e-08 ***
## rt_diff_large_neutral_z        0.12550    0.31240   0.402    0.688
## race.ethnicity.5levelBlack    -0.42868    0.76687  -0.559    0.576
## race.ethnicity.5levelMixed     0.79246    0.76051   1.042    0.298
## race.ethnicity.5levelOther     0.28941    0.87767   0.330    0.742
## race.ethnicity.5levelWhite     0.94340    0.70278   1.342    0.180
## demo_race_hispanic1            0.25948    0.34763   0.746    0.455
## interview_age                  0.01344    0.01610   0.835    0.404
## PDS_score:rt_diff_large_neutral_z -0.03208    0.17169  -0.187    0.852
## ---
## Signif. codes:  0 '***' 0.001 '**' 0.01 '*' 0.05 '.' 0.1 ' ' 1
##
##
## R-sq.(adj) =  0.0172
## lmer.REML =  13443  Scale est. = 17.06     n = 2178
```

```
##                                        stdcoef      stdse
## X(Intercept)                       0.000000000 0.00000000
## XPDS_score                         0.132860230 0.02326017
## Xrt_diff_large_neutral_z           0.021452250 0.05340206
## Xrace.ethnicity.5levelBlack       -0.028019198 0.05012395
## Xrace.ethnicity.5levelMixed        0.048429992 0.04647723
## Xrace.ethnicity.5levelOther        0.011915370 0.03613498
## Xrace.ethnicity.5levelWhite        0.083350028 0.06209138
## Xdemo_race_hispanic1               0.019010003 0.02546788
## Xinterview_age                     0.018495409 0.02214367
## XPDS_score:rt_diff_large_neutral_z -0.009971141 0.05335881
```

Male participants

```
##
## Family: gaussian
```

```
## Link function: identity
##
## Formula:
## cbcl_scr_syn_internal_r ~ PDS_score * rt_diff_large_neutral_z +
##     race.ethnicity.5level + demo_race_hispanic + interview_age
##
## Parametric coefficients:
##                                   Estimate Std. Error t value Pr(>|t|)
## (Intercept)                        3.05494    2.06455   1.480  0.13909
## PDS_score                          0.86102    0.22570   3.815  0.00014 ***
## rt_diff_large_neutral_z            0.75812    0.32473   2.335  0.01965 *
## race.ethnicity.5levelBlack        -1.18271    0.94568  -1.251  0.21119
## race.ethnicity.5levelMixed        -0.18228    0.94053  -0.194  0.84634
## race.ethnicity.5levelOther        -1.45949    1.04063  -1.403  0.16090
## race.ethnicity.5levelWhite        -0.46556    0.88370  -0.527  0.59836
## demo_race_hispanic1                0.71219    0.35837   1.987  0.04701 *
## interview_age                      0.01001    0.01550   0.646  0.51833
## PDS_score:rt_diff_large_neutral_z -0.62565    0.22645  -2.763  0.00578 **
## ---
## Signif. codes:  0 '***' 0.001 '**' 0.01 '*' 0.05 '.' 0.1 ' ' 1
##
##
## R-sq.(adj) =  0.00916
## lmer.REML =  14034  Scale est. = 13.667    n = 2248
```

```
##                                        stdcoef      stdse
## X(Intercept)                        0.00000000 0.00000000
## XPDS_score                          0.08345122 0.02187509
## Xrt_diff_large_neutral_z            0.12485045 0.05347720
## Xrace.ethnicity.5levelBlack        -0.06789727 0.05428949
## Xrace.ethnicity.5levelMixed        -0.01048183 0.05408346
## Xrace.ethnicity.5levelOther        -0.05823251 0.04152043
## Xrace.ethnicity.5levelWhite        -0.03832250 0.07274152
## Xdemo_race_hispanic1                0.05051042 0.02541625
## Xinterview_age                      0.01358778 0.02103308
## XPDS_score:rt_diff_large_neutral_z -0.14827429 0.05366704
```

4.13 Model: CBCL internalizing factor ~ PDS x MID reaction time (large vs. small reward)

Female participants

```
##
## Family: gaussian
## Link function: identity
##
## Formula:
## cbcl_scr_syn_internal_r ~ PDS_score * rt_diff_large_small_z +
##     race.ethnicity.5level + demo_race_hispanic + interview_age
##
## Parametric coefficients:
##                                 Estimate Std. Error t value Pr(>|t|)
## (Intercept)                      0.95469    1.97005   0.485    0.628
```

```
## PDS_score                         0.99961    0.17502   5.711 1.28e-08 ***
## rt_diff_large_small_z             0.13182    0.29169   0.452    0.651
## race.ethnicity.5levelBlack       -0.39939    0.76684  -0.521    0.603
## race.ethnicity.5levelMixed        0.82152    0.76058   1.080    0.280
## race.ethnicity.5levelOther        0.31441    0.87735   0.358    0.720
## race.ethnicity.5levelWhite        0.96820    0.70249   1.378    0.168
## demo_race_hispanic1               0.25717    0.34745   0.740    0.459
## interview_age                     0.01327    0.01608   0.825    0.410
## PDS_score:rt_diff_large_small_z  0.00451    0.15941   0.028    0.977
## ---
## Signif. codes:  0 '***' 0.001 '**' 0.01 '*' 0.05 '.' 0.1 ' ' 1
##
##
## R-sq.(adj) =  0.0177
## lmer.REML =  13442  Scale est. = 17.021    n = 2178
```

```
##                                       stdcoef      stdse
## X(Intercept)                       0.000000000 0.00000000
## XPDS_score                         0.132804023 0.02325294
## Xrt_diff_large_small_z             0.023528010 0.05206012
## Xrace.ethnicity.5levelBlack       -0.026104901 0.05012214
## Xrace.ethnicity.5levelMixed        0.050205927 0.04648196
## Xrace.ethnicity.5levelOther        0.012944752 0.03612170
## Xrace.ethnicity.5levelWhite        0.085540857 0.06206517
## Xdemo_race_hispanic1               0.018840448 0.02545453
## Xinterview_age                     0.018251823 0.02212800
## XPDS_score:rt_diff_large_small_z  0.001474834 0.05212811
```

Male participants

```
##
## Family: gaussian
## Link function: identity
##
## Formula:
## cbcl_scr_syn_internal_r ~ PDS_score * rt_diff_large_small_z +
##     race.ethnicity.5level + demo_race_hispanic + interview_age
##
## Parametric coefficients:
##                                  Estimate Std. Error t value Pr(>|t|)
## (Intercept)                       3.257857   2.063983   1.578 0.114608
## PDS_score                         0.866678   0.226024   3.834 0.000129 ***
## rt_diff_large_small_z             0.308235   0.331032   0.931 0.351884
## race.ethnicity.5levelBlack       -1.143317   0.947228  -1.207 0.227555
## race.ethnicity.5levelMixed       -0.205018   0.941232  -0.218 0.827590
## race.ethnicity.5levelOther       -1.446505   1.041634  -1.389 0.165066
## race.ethnicity.5levelWhite       -0.500116   0.884459  -0.565 0.571826
## demo_race_hispanic1               0.734551   0.359205   2.045 0.040978 *
## interview_age                     0.008417   0.015495   0.543 0.587029
## PDS_score:rt_diff_large_small_z -0.346620   0.232225  -1.493 0.135682
## ---
## Signif. codes:  0 '***' 0.001 '**' 0.01 '*' 0.05 '.' 0.1 ' ' 1
##
```

```
##
## R-sq.(adj) =  0.00715
## lmer.REML =  14038  Scale est. = 13.663    n = 2248
```

```
##                                      stdcoef      stdse
## X(Intercept)                      0.00000000 0.00000000
## XPDS_score                        0.08399971 0.02190658
## Xrt_diff_large_small_z            0.05017715 0.05388819
## Xrace.ethnicity.5levelBlack      -0.06563570 0.05437863
## Xrace.ethnicity.5levelMixed      -0.01178916 0.05412380
## Xrace.ethnicity.5levelOther      -0.05771438 0.04156037
## Xrace.ethnicity.5levelWhite      -0.04116709 0.07280436
## Xdemo_race_hispanic1              0.05209626 0.02547577
## Xinterview_age                    0.01142007 0.02102270
## XPDS_score:rt_diff_large_small_z -0.08105480 0.05430429
```

4.14 Model: CBCL internalizing factor ~ Testosterone x Accumbens activity (anticipation stage) + PDS

Female participants

```
##
## Family: gaussian
## Link function: identity
##
## Formula:
## cbcl_scr_syn_internal_r ~ PDS_score + hormone_scr_ert_mean *
##     accumbens_rvsn_ant_z + race.ethnicity.5level + demo_race_hispanic +
##     interview_age
##
## Parametric coefficients:
##                                        Estimate Std. Error t value Pr(>|t|)
## (Intercept)                           -0.181291   2.081064  -0.087  0.93059
## PDS_score                              1.028257   0.190070   5.410 7.13e-08
## hormone_scr_ert_mean                  -0.002649   0.007808  -0.339  0.73446
## accumbens_rvsn_ant_z                   0.748352   0.395459   1.892  0.05860
## race.ethnicity.5levelBlack            -0.489731   0.799326  -0.613  0.54016
## race.ethnicity.5levelMixed             0.901860   0.791070   1.140  0.25441
## race.ethnicity.5levelOther             0.025106   0.942731   0.027  0.97876
## race.ethnicity.5levelWhite             1.193196   0.725804   1.644  0.10035
## demo_race_hispanic1                    0.059304   0.364861   0.163  0.87090
## interview_age                          0.021642   0.017153   1.262  0.20721
## hormone_scr_ert_mean:accumbens_rvsn_ant_z -0.025498   0.009632  -2.647  0.00818
##
## (Intercept)
## PDS_score                             ***
## hormone_scr_ert_mean
## accumbens_rvsn_ant_z                  .
## race.ethnicity.5levelBlack
## race.ethnicity.5levelMixed
## race.ethnicity.5levelOther
## race.ethnicity.5levelWhite
## demo_race_hispanic1
```

```
## interview_age
## hormone_scr_ert_mean:accumbens_rvsn_ant_z **
## ---
## Signif. codes:  0 '***' 0.001 '**' 0.01 '*' 0.05 '.' 0.1 ' ' 1
##
##
## R-sq.(adj) =  0.0241
## lmer.REML =  11374  Scale est. = 15.52     n = 1850
```

```
##                                                  stdcoef      stdse
## X(Intercept)                                0.0000000000 0.00000000
## XPDS_score                                  0.1389781901 0.02568966
## Xhormone_scr_ert_mean                      -0.0082652052 0.02436346
## Xaccumbens_rvsn_ant_z                       0.0977409074 0.05165021
## Xrace.ethnicity.5levelBlack                -0.0315021609 0.05141700
## Xrace.ethnicity.5levelMixed                 0.0552912412 0.04849888
## Xrace.ethnicity.5levelOther                 0.0009950354 0.03736316
## Xrace.ethnicity.5levelWhite                 0.1056549000 0.06426839
## Xdemo_race_hispanic1                        0.0043767488 0.02692761
## Xinterview_age                              0.0303985479 0.02409298
## Xhormone_scr_ert_mean:accumbens_rvsn_ant_z -0.1365559560 0.05158258
```

Male participants

```
##
## Family: gaussian
## Link function: identity
##
## Formula:
## cbcl_scr_syn_internal_r ~ PDS_score + hormone_scr_ert_mean *
##     accumbens_rvsn_ant_z + race.ethnicity.5level + demo_race_hispanic +
##     interview_age
##
## Parametric coefficients:
##                                             Estimate Std. Error t value Pr(>|t|)
## (Intercept)                                 2.971022   2.233067   1.330 0.183529
## PDS_score                                   0.941721   0.258284   3.646 0.000274
## hormone_scr_ert_mean                        0.011646   0.009135   1.275 0.202525
## accumbens_rvsn_ant_z                        0.064232   0.415192   0.155 0.877072
## race.ethnicity.5levelBlack                 -0.559369   1.048266  -0.534 0.593673
## race.ethnicity.5levelMixed                  0.379454   1.035112   0.367 0.713973
## race.ethnicity.5levelOther                 -0.814595   1.151470  -0.707 0.479383
## race.ethnicity.5levelWhite                  0.223149   0.974250   0.229 0.818858
## demo_race_hispanic1                         0.759191   0.392285   1.935 0.053105
## interview_age                               0.001740   0.016952   0.103 0.918262
## hormone_scr_ert_mean:accumbens_rvsn_ant_z -0.005147   0.012451  -0.413 0.679346
##
## (Intercept)
## PDS_score                                 ***
## hormone_scr_ert_mean
## accumbens_rvsn_ant_z
## race.ethnicity.5levelBlack
## race.ethnicity.5levelMixed
```

```
## race.ethnicity.5levelOther
## race.ethnicity.5levelWhite
## demo_race_hispanic1                       .
## interview_age
## hormone_scr_ert_mean:accumbens_rvsn_ant_z
## ---
## Signif. codes:  0 '***' 0.001 '**' 0.01 '*' 0.05 '.' 0.1 ' ' 1
##
##
## R-sq.(adj) =  0.00679
## lmer.REML =  11577  Scale est. = 14.337    n = 1853
```

```
##                                                stdcoef      stdse
## X(Intercept)                               0.000000000 0.00000000
## XPDS_score                                 0.088554592 0.02428767
## Xhormone_scr_ert_mean                      0.030435302 0.02387371
## Xaccumbens_rvsn_ant_z                      0.008316511 0.05375760
## Xrace.ethnicity.5levelBlack               -0.031221518 0.05850959
## Xrace.ethnicity.5levelMixed                0.021808009 0.05949013
## Xrace.ethnicity.5levelOther               -0.032493479 0.04593111
## Xrace.ethnicity.5levelWhite                0.018248418 0.07967120
## Xdemo_race_hispanic1                       0.053963970 0.02788392
## Xinterview_age                             0.002388339 0.02326968
## Xhormone_scr_ert_mean:accumbens_rvsn_ant_z -0.022138028 0.05354841
```

4.15 Model: CBCL internalizing factor ~ Testosterone x Caudate activity (anticipation stage) + PDS

Female participants

```
##
## Family: gaussian
## Link function: identity
##
## Formula:
## cbcl_scr_syn_internal_r ~ PDS_score + hormone_scr_ert_mean *
##     caudate_rvsn_ant_z + race.ethnicity.5level + demo_race_hispanic +
##     interview_age
##
## Parametric coefficients:
##                                         Estimate Std. Error t value Pr(>|t|)
## (Intercept)                             0.165775   2.091116   0.079   0.9368
## PDS_score                               0.995050   0.190494   5.224 1.95e-07
## hormone_scr_ert_mean                   -0.002227   0.007845  -0.284   0.7765
## caudate_rvsn_ant_z                      0.378963   0.306118   1.238   0.2159
## race.ethnicity.5levelBlack             -0.474215   0.802532  -0.591   0.5547
## race.ethnicity.5levelMixed              0.913794   0.793343   1.152   0.2495
## race.ethnicity.5levelOther              0.066373   0.947244   0.070   0.9441
## race.ethnicity.5levelWhite              1.221957   0.728462   1.677   0.0936
## demo_race_hispanic1                     0.023850   0.365429   0.065   0.9480
## interview_age                           0.019016   0.017253   1.102   0.2705
## hormone_scr_ert_mean:caudate_rvsn_ant_z -0.009497   0.007717  -1.231   0.2186
##
```

```
## (Intercept)
## PDS_score                              ***
## hormone_scr_ert_mean
## caudate_rvsn_ant_z
## race.ethnicity.5levelBlack
## race.ethnicity.5levelMixed
## race.ethnicity.5levelOther
## race.ethnicity.5levelWhite              .
## demo_race_hispanic1
## interview_age
## hormone_scr_ert_mean:caudate_rvsn_ant_z
## ---
## Signif. codes:  0 '***' 0.001 '**' 0.01 '*' 0.05 '.' 0.1 ' ' 1
##
##
## R-sq.(adj) =  0.0196
## lmer.REML =  11425  Scale est. = 16.042    n = 1855
```

```
##                                              stdcoef      stdse
## X(Intercept)                             0.000000000 0.00000000
## XPDS_score                               0.134085354 0.02566951
## Xhormone_scr_ert_mean                   -0.006933558 0.02442357
## Xcaudate_rvsn_ant_z                      0.063093058 0.05096514
## Xrace.ethnicity.5levelBlack             -0.030440818 0.05151619
## Xrace.ethnicity.5levelMixed              0.056339908 0.04891349
## Xrace.ethnicity.5levelOther              0.002605980 0.03719149
## Xrace.ethnicity.5levelWhite              0.108059514 0.06441898
## Xdemo_race_hispanic1                     0.001759428 0.02695781
## Xinterview_age                           0.026637580 0.02416899
## Xhormone_scr_ert_mean:caudate_rvsn_ant_z -0.062561015 0.05083950
```

Male participants

```
##
## Family: gaussian
## Link function: identity
##
## Formula:
## cbcl_scr_syn_internal_r ~ PDS_score + hormone_scr_ert_mean *
##     caudate_rvsn_ant_z + race.ethnicity.5level + demo_race_hispanic +
##     interview_age
##
## Parametric coefficients:
##                                          Estimate Std. Error t value Pr(>|t|)
## (Intercept)                              2.928668   2.237456   1.309 0.190722
## PDS_score                                0.959660   0.260826   3.679 0.000241
## hormone_scr_ert_mean                     0.011532   0.009148   1.261 0.207647
## caudate_rvsn_ant_z                       0.356827   0.323129   1.104 0.269612
## race.ethnicity.5levelBlack              -0.496693   1.049085  -0.473 0.635945
## race.ethnicity.5levelMixed               0.448062   1.035645   0.433 0.665326
## race.ethnicity.5levelOther              -0.875764   1.151663  -0.760 0.447092
## race.ethnicity.5levelWhite               0.281966   0.976133   0.289 0.772721
## demo_race_hispanic1                      0.829875   0.395562   2.098 0.036044
```

```
## interview_age                            0.001742   0.016998   0.103 0.918365
## hormone_scr_ert_mean:caudate_rvsn_ant_z -0.007925   0.009409  -0.842 0.399718
##
## (Intercept)
## PDS_score                              ***
## hormone_scr_ert_mean
## caudate_rvsn_ant_z
## race.ethnicity.5levelBlack
## race.ethnicity.5levelMixed
## race.ethnicity.5levelOther
## race.ethnicity.5levelWhite
## demo_race_hispanic1                    *
## interview_age
## hormone_scr_ert_mean:caudate_rvsn_ant_z
## ---
## Signif. codes:  0 '***' 0.001 '**' 0.01 '*' 0.05 '.' 0.1 ' ' 1
##
##
## R-sq.(adj) =  0.00784
## lmer.REML =  11617  Scale est. = 14.352    n = 1858
```

```
##                                              stdcoef      stdse
## X(Intercept)                             0.000000000 0.00000000
## XPDS_score                               0.089105606 0.02421797
## Xhormone_scr_ert_mean                    0.030080725 0.02386404
## Xcaudate_rvsn_ant_z                      0.058296260 0.05279085
## Xrace.ethnicity.5levelBlack             -0.027799742 0.05871691
## Xrace.ethnicity.5levelMixed              0.025861040 0.05977485
## Xrace.ethnicity.5levelOther             -0.035137985 0.04620780
## Xrace.ethnicity.5levelWhite              0.023090064 0.07993501
## Xdemo_race_hispanic1                     0.058656505 0.02795877
## Xinterview_age                           0.002383725 0.02325423
## Xhormone_scr_ert_mean:caudate_rvsn_ant_z -0.044409617 0.05272303
```

4.16 Model: CBCL internalizing factor ~ Testosterone x Putamen activity (anticipation stage) + PDS

Female participants

```
##
## Family: gaussian
## Link function: identity
##
## Formula:
## cbcl_scr_syn_internal_r ~ PDS_score + hormone_scr_ert_mean *
##     putamen_rvsn_ant_z + race.ethnicity.5level + demo_race_hispanic +
##     interview_age
##
## Parametric coefficients:
##                                         Estimate Std. Error t value Pr(>|t|)
## (Intercept)                             0.138727   2.090567   0.066   0.9471
## PDS_score                               1.010336   0.190694   5.298 1.31e-07
## hormone_scr_ert_mean                   -0.002053   0.007858  -0.261   0.7939
```

```
## putamen_rvsn_ant_z                      0.229449   0.311016   0.738   0.4608
## race.ethnicity.5levelBlack             -0.520634   0.802461  -0.649   0.5166
## race.ethnicity.5levelMixed              0.899355   0.793768   1.133   0.2574
## race.ethnicity.5levelOther              0.009675   0.945429   0.010   0.9918
## race.ethnicity.5levelWhite              1.227171   0.728365   1.685   0.0922
## demo_race_hispanic1                     0.017266   0.365130   0.047   0.9623
## interview_age                           0.019075   0.017255   1.105   0.2691
## hormone_scr_ert_mean:putamen_rvsn_ant_z -0.006497   0.007955  -0.817   0.4142
##
## (Intercept)
## PDS_score                              ***
## hormone_scr_ert_mean
## putamen_rvsn_ant_z
## race.ethnicity.5levelBlack
## race.ethnicity.5levelMixed
## race.ethnicity.5levelOther
## race.ethnicity.5levelWhite             .
## demo_race_hispanic1
## interview_age
## hormone_scr_ert_mean:putamen_rvsn_ant_z
## ---
## Signif. codes:  0 '***' 0.001 '**' 0.01 '*' 0.05 '.' 0.1 ' ' 1
##
##
## R-sq.(adj) =  0.0202
## lmer.REML =  11414  Scale est. = 16.009    n = 1853
```

```
##                                               stdcoef      stdse
## X(Intercept)                              0.0000000000 0.00000000
## XPDS_score                                0.1362493251 0.02571620
## Xhormone_scr_ert_mean                    -0.0063899970 0.02445485
## Xputamen_rvsn_ant_z                       0.0375247558 0.05086448
## Xrace.ethnicity.5levelBlack              -0.0333764669 0.05144360
## Xrace.ethnicity.5levelMixed               0.0553658299 0.04886572
## Xrace.ethnicity.5levelOther               0.0003821015 0.03733972
## Xrace.ethnicity.5levelWhite               0.1084946559 0.06439504
## Xdemo_race_hispanic1                      0.0012754771 0.02697221
## Xinterview_age                            0.0267353943 0.02418432
## Xhormone_scr_ert_mean:putamen_rvsn_ant_z -0.0415181775 0.05083685
```

Male participants

```
##
## Family: gaussian
## Link function: identity
##
## Formula:
## cbcl_scr_syn_internal_r ~ PDS_score + hormone_scr_ert_mean *
##     putamen_rvsn_ant_z + race.ethnicity.5level + demo_race_hispanic +
##     interview_age
##
## Parametric coefficients:
##                                           Estimate Std. Error t value Pr(>|t|)
```

```
## (Intercept)                              3.0632426  2.2387210   1.368 0.171385
## PDS_score                                0.9716892  0.2611534   3.721 0.000205
## hormone_scr_ert_mean                     0.0105792  0.0091274   1.159 0.246580
## putamen_rvsn_ant_z                       0.0169489  0.3346578   0.051 0.959613
## race.ethnicity.5levelBlack              -0.5776616  1.0494417  -0.550 0.582080
## race.ethnicity.5levelMixed               0.4255411  1.0362474   0.411 0.681373
## race.ethnicity.5levelOther              -0.8511298  1.1534642  -0.738 0.460675
## race.ethnicity.5levelWhite               0.2607401  0.9766309   0.267 0.789515
## demo_race_hispanic1                      0.7798854  0.3922592   1.988 0.046938
## interview_age                            0.0008853  0.0170100   0.052 0.958496
## hormone_scr_ert_mean:putamen_rvsn_ant_z -0.0037217  0.0098086  -0.379 0.704409
##
## (Intercept)
## PDS_score                               ***
## hormone_scr_ert_mean
## putamen_rvsn_ant_z
## race.ethnicity.5levelBlack
## race.ethnicity.5levelMixed
## race.ethnicity.5levelOther
## race.ethnicity.5levelWhite
## demo_race_hispanic1                     *
## interview_age
## hormone_scr_ert_mean:putamen_rvsn_ant_z
## ---
## Signif. codes:  0 '***' 0.001 '**' 0.01 '*' 0.05 '.' 0.1 ' ' 1
##
##
## R-sq.(adj) =  0.00749
## lmer.REML =  11617  Scale est. = 14.452    n = 1858
```

```
##                                              stdcoef      stdse
## X(Intercept)                             0.000000000 0.00000000
## XPDS_score                               0.090360023 0.02428536
## Xhormone_scr_ert_mean                    0.027668514 0.02387145
## Xputamen_rvsn_ant_z                      0.002744835 0.05419697
## Xrace.ethnicity.5levelBlack             -0.032297473 0.05867504
## Xrace.ethnicity.5levelMixed              0.024587933 0.05987478
## Xrace.ethnicity.5levelOther             -0.034023374 0.04610900
## Xrace.ethnicity.5levelWhite              0.021353195 0.07998076
## Xdemo_race_hispanic1                     0.055241195 0.02778468
## Xinterview_age                           0.001211161 0.02327025
## Xhormone_scr_ert_mean:putamen_rvsn_ant_z -0.020500789 0.05402989
```

4.17 Model: CBCL internalizing factor ~ Testosterone x Accumbens activity (feedback stage) + PDS

Female participants

```
##
## Family: gaussian
## Link function: identity
##
## Formula:
```

```
## cbcl_scr_syn_internal_r ~ PDS_score + hormone_scr_ert_mean *
##     accumbens_posvsneg_feedback_z + race.ethnicity.5level + demo_race_hispanic +
##     interview_age
##
## Parametric coefficients:
##                                               Estimate Std. Error t value
## (Intercept)                                  -0.190478   2.096649  -0.091
## PDS_score                                     0.960917   0.191780   5.011
## hormone_scr_ert_mean                         -0.001236   0.007857  -0.157
## accumbens_posvsneg_feedback_z                 0.168432   0.424783   0.397
## race.ethnicity.5levelBlack                   -0.222052   0.810009  -0.274
## race.ethnicity.5levelMixed                    1.183490   0.799554   1.480
## race.ethnicity.5levelOther                    0.317332   0.947940   0.335
## race.ethnicity.5levelWhite                    1.453078   0.734562   1.978
## demo_race_hispanic1                           0.014620   0.368046   0.040
## interview_age                                 0.020263   0.017274   1.173
## hormone_scr_ert_mean:accumbens_posvsneg_feedback_z  0.001104   0.010674   0.103
##                                              Pr(>|t|)
## (Intercept)                                    0.9276
## PDS_score                                    5.95e-07 ***
## hormone_scr_ert_mean                           0.8750
## accumbens_posvsneg_feedback_z                  0.6918
## race.ethnicity.5levelBlack                     0.7840
## race.ethnicity.5levelMixed                     0.1390
## race.ethnicity.5levelOther                     0.7378
## race.ethnicity.5levelWhite                     0.0481 *
## demo_race_hispanic1                            0.9683
## interview_age                                  0.2409
## hormone_scr_ert_mean:accumbens_posvsneg_feedback_z   0.9177
## ---
## Signif. codes:  0 '***' 0.001 '**' 0.01 '*' 0.05 '.' 0.1 ' ' 1
##
##
## R-sq.(adj) =  0.0194
## lmer.REML =  11378  Scale est. = 16.001    n = 1848
```

```
##                                                        stdcoef      stdse
## X(Intercept)                                       0.000000000 0.00000000
## XPDS_score                                         0.129194427 0.02578463
## Xhormone_scr_ert_mean                             -0.003852145 0.02448146
## Xaccumbens_posvsneg_feedback_z                     0.021435945 0.05406124
## Xrace.ethnicity.5levelBlack                       -0.014173104 0.05170109
## Xrace.ethnicity.5levelMixed                        0.072757083 0.04915399
## Xrace.ethnicity.5levelOther                        0.012632739 0.03773675
## Xrace.ethnicity.5levelWhite                        0.128406472 0.06491221
## Xdemo_race_hispanic1                               0.001078976 0.02716198
## Xinterview_age                                     0.028414943 0.02422368
## Xhormone_scr_ert_mean:accumbens_posvsneg_feedback_z  0.005569465 0.05386411
```

Male participants

```
##
## Family: gaussian
```

```
## Link function: identity
##
## Formula:
## cbcl_scr_syn_internal_r ~ PDS_score + hormone_scr_ert_mean *
##     accumbens_posvsneg_feedback_z + race.ethnicity.5level + demo_race_hispanic +
##     interview_age
##
## Parametric coefficients:
##                                                Estimate Std. Error t value
## (Intercept)                                    3.444893   2.246285   1.534
## PDS_score                                      0.969873   0.259187   3.742
## hormone_scr_ert_mean                           0.009416   0.009150   1.029
## accumbens_posvsneg_feedback_z                 -0.380898   0.411803  -0.925
## race.ethnicity.5levelBlack                    -0.637430   1.064034  -0.599
## race.ethnicity.5levelMixed                     0.420115   1.051165   0.400
## race.ethnicity.5levelOther                    -0.993047   1.164515  -0.853
## race.ethnicity.5levelWhite                     0.197282   0.992013   0.199
## demo_race_hispanic1                            0.800212   0.392623   2.038
## interview_age                                 -0.001290   0.017026  -0.076
## hormone_scr_ert_mean:accumbens_posvsneg_feedback_z  0.005856   0.012119   0.483
##                                               Pr(>|t|)
## (Intercept)                                   0.125300
## PDS_score                                     0.000188 ***
## hormone_scr_ert_mean                          0.303559
## accumbens_posvsneg_feedback_z                 0.355112
## race.ethnicity.5levelBlack                    0.549200
## race.ethnicity.5levelMixed                    0.689449
## race.ethnicity.5levelOther                    0.393905
## race.ethnicity.5levelWhite                    0.842386
## demo_race_hispanic1                           0.041680 *
## interview_age                                 0.939600
## hormone_scr_ert_mean:accumbens_posvsneg_feedback_z 0.629030
## ---
## Signif. codes:  0 '***' 0.001 '**' 0.01 '*' 0.05 '.' 0.1 ' ' 1
##
##
## R-sq.(adj) =  0.00789
## lmer.REML =  11627  Scale est. = 14.272    n = 1859
```

```
##                                                        stdcoef      stdse
## X(Intercept)                                        0.000000000 0.00000000
## XPDS_score                                          0.090490810 0.02418258
## Xhormone_scr_ert_mean                               0.024551517 0.02385684
## Xaccumbens_posvsneg_feedback_z                     -0.049725932 0.05376056
## Xrace.ethnicity.5levelBlack                        -0.035548230 0.05933907
## Xrace.ethnicity.5levelMixed                         0.024164801 0.06046249
## Xrace.ethnicity.5levelOther                        -0.039973665 0.04687586
## Xrace.ethnicity.5levelWhite                         0.016116429 0.08103974
## Xdemo_race_hispanic1                                0.056773617 0.02785589
## Xinterview_age                                     -0.001759647 0.02321967
## Xhormone_scr_ert_mean:accumbens_posvsneg_feedback_z  0.026069997 0.05395584
```

4.18 Model: CBCL internalizing factor ~ Testosterone x Caudate activity (Feedback stage) + PDS

Female participants

```
##
## Family: gaussian
## Link function: identity
##
## Formula:
## cbcl_scr_syn_internal_r ~ PDS_score + hormone_scr_ert_mean *
##     caudate_posvsneg_feedback_z + race.ethnicity.5level + demo_race_hispanic +
##     interview_age
##
## Parametric coefficients:
##                                               Estimate Std. Error t value
## (Intercept)                                 -0.2182413  2.0973786  -0.104
## PDS_score                                    0.9674477  0.1909243   5.067
## hormone_scr_ert_mean                        -0.0004754  0.0078346  -0.061
## caudate_posvsneg_feedback_z                 -0.1399113  0.3318573  -0.422
## race.ethnicity.5levelBlack                  -0.2926293  0.8113753  -0.361
## race.ethnicity.5levelMixed                   1.1277284  0.8007884   1.408
## race.ethnicity.5levelOther                   0.2609206  0.9475744   0.275
## race.ethnicity.5levelWhite                   1.4575625  0.7357842   1.981
## demo_race_hispanic1                          0.0668360  0.3667756   0.182
## interview_age                                0.0202858  0.0172731   1.174
## hormone_scr_ert_mean:caudate_posvsneg_feedback_z  0.0013410  0.0084975   0.158
##                                             Pr(>|t|)
## (Intercept)                                   0.9171
## PDS_score                                   4.44e-07 ***
## hormone_scr_ert_mean                          0.9516
## caudate_posvsneg_feedback_z                   0.6734
## race.ethnicity.5levelBlack                    0.7184
## race.ethnicity.5levelMixed                    0.1592
## race.ethnicity.5levelOther                    0.7831
## race.ethnicity.5levelWhite                    0.0477 *
## demo_race_hispanic1                           0.8554
## interview_age                                 0.2404
## hormone_scr_ert_mean:caudate_posvsneg_feedback_z   0.8746
## ---
## Signif. codes:  0 '***' 0.001 '**' 0.01 '*' 0.05 '.' 0.1 ' ' 1
##
##
## R-sq.(adj) =  0.0196
## lmer.REML =  11385  Scale est. = 16.086    n = 1849
```

```
##                                                  stdcoef      stdse
## X(Intercept)                                 0.000000000 0.00000000
## XPDS_score                                   0.130380989 0.02573048
## Xhormone_scr_ert_mean                       -0.001484189 0.02445936
## Xcaudate_posvsneg_feedback_z                -0.022550851 0.05348862
## Xrace.ethnicity.5levelBlack                 -0.018668983 0.05176361
## Xrace.ethnicity.5levelMixed                  0.069685574 0.04948301
## Xrace.ethnicity.5levelOther                  0.010381795 0.03770313
```

```
## Xrace.ethnicity.5levelWhite                        0.128905272 0.06507197
## Xdemo_race_hispanic1                               0.004945977 0.02714202
## Xinterview_age                                     0.028437833 0.02421436
## Xhormone_scr_ert_mean:caudate_posvsneg_feedback_z  0.008432340 0.05343465
```

Male participants

```
##
## Family: gaussian
## Link function: identity
##
## Formula:
## cbcl_scr_syn_internal_r ~ PDS_score + hormone_scr_ert_mean *
##     caudate_posvsneg_feedback_z + race.ethnicity.5level + demo_race_hispanic +
##     interview_age
##
## Parametric coefficients:
##                                                   Estimate Std. Error t value
## (Intercept)                                       3.465977   2.244693   1.544
## PDS_score                                         0.932193   0.259898   3.587
## hormone_scr_ert_mean                              0.009852   0.009139   1.078
## caudate_posvsneg_feedback_z                      -0.196700   0.327591  -0.600
## race.ethnicity.5levelBlack                       -0.601690   1.055714  -0.570
## race.ethnicity.5levelMixed                        0.417075   1.040924   0.401
## race.ethnicity.5levelOther                       -0.955471   1.156660  -0.826
## race.ethnicity.5levelWhite                        0.191056   0.982130   0.195
## demo_race_hispanic1                               0.774207   0.391508   1.977
## interview_age                                    -0.001332   0.017032  -0.078
## hormone_scr_ert_mean:caudate_posvsneg_feedback_z  0.004552   0.009460   0.481
##                                                  Pr(>|t|)
## (Intercept)                                      0.122741
## PDS_score                                        0.000344 ***
## hormone_scr_ert_mean                             0.281190
## caudate_posvsneg_feedback_z                      0.548285
## race.ethnicity.5levelBlack                       0.568790
## race.ethnicity.5levelMixed                       0.688704
## race.ethnicity.5levelOther                       0.408877
## race.ethnicity.5levelWhite                       0.845780
## demo_race_hispanic1                              0.048134 *
## interview_age                                    0.937662
## hormone_scr_ert_mean:caudate_posvsneg_feedback_z 0.630422
## ---
## Signif. codes:  0 '***' 0.001 '**' 0.01 '*' 0.05 '.' 0.1 ' ' 1
##
##
## R-sq.(adj) =  0.00627
## lmer.REML =  11616  Scale est. = 14.148    n = 1857
```

```
##                                                       stdcoef      stdse
## X(Intercept)                                       0.000000000 0.00000000
## XPDS_score                                         0.086892003 0.02422572
## Xhormone_scr_ert_mean                              0.025753964 0.02389128
## Xcaudate_posvsneg_feedback_z                      -0.031470577 0.05241234
```

```
## Xrace.ethnicity.5levelBlack                          -0.033545706 0.05885868
## Xrace.ethnicity.5levelMixed                           0.024129599 0.06022202
## Xrace.ethnicity.5levelOther                          -0.038352423 0.04642812
## Xrace.ethnicity.5levelWhite                           0.015634556 0.08036985
## Xdemo_race_hispanic1                                  0.055028143 0.02782714
## Xinterview_age                                       -0.001820504 0.02327427
## Xhormone_scr_ert_mean:caudate_posvsneg_feedback_z  0.025196283 0.05235997
```

4.19 Model: CBCL internalizing factor ~ Testosterone x Putamen activity (Feedback stage) + PDS

Female participants

```
##
## Family: gaussian
## Link function: identity
##
## Formula:
## cbcl_scr_syn_internal_r ~ PDS_score + hormone_scr_ert_mean *
##     putamen_posvsneg_feedback_z + race.ethnicity.5level + demo_race_hispanic +
##     interview_age
##
## Parametric coefficients:
##                                                  Estimate Std. Error t value
## (Intercept)                                      0.118158   2.089886   0.057
## PDS_score                                        1.024707   0.191144   5.361
## hormone_scr_ert_mean                            -0.002045   0.007853  -0.260
## putamen_posvsneg_feedback_z                      0.083296   0.337089   0.247
## race.ethnicity.5levelBlack                      -0.486371   0.803308  -0.605
## race.ethnicity.5levelMixed                       0.913244   0.793817   1.150
## race.ethnicity.5levelOther                      -0.045591   0.944110  -0.048
## race.ethnicity.5levelWhite                       1.224540   0.728436   1.681
## demo_race_hispanic1                              0.100062   0.366661   0.273
## interview_age                                    0.018886   0.017236   1.096
## hormone_scr_ert_mean:putamen_posvsneg_feedback_z -0.005448   0.008453  -0.645
##                                                 Pr(>|t|)
## (Intercept)                                       0.9549
## PDS_score                                       9.32e-08 ***
## hormone_scr_ert_mean                              0.7946
## putamen_posvsneg_feedback_z                       0.8049
## race.ethnicity.5levelBlack                        0.5449
## race.ethnicity.5levelMixed                        0.2501
## race.ethnicity.5levelOther                        0.9615
## race.ethnicity.5levelWhite                        0.0929 .
## demo_race_hispanic1                               0.7850
## interview_age                                     0.2734
## hormone_scr_ert_mean:putamen_posvsneg_feedback_z   0.5193
## ---
## Signif. codes:  0 '***' 0.001 '**' 0.01 '*' 0.05 '.' 0.1 ' ' 1
##
##
## R-sq.(adj) =  0.0207
## lmer.REML =  11406  Scale est. = 16.487    n = 1852
```

```
##                                                       stdcoef      stdse
## X(Intercept)                                     0.000000000 0.00000000
## XPDS_score                                       0.137664001 0.02567919
## Xhormone_scr_ert_mean                           -0.006366430 0.02445197
## Xputamen_posvsneg_feedback_z                     0.013264446 0.05367985
## Xrace.ethnicity.5levelBlack                     -0.031092619 0.05135368
## Xrace.ethnicity.5levelMixed                      0.056146235 0.04880387
## Xrace.ethnicity.5levelOther                     -0.001801638 0.03730880
## Xrace.ethnicity.5levelWhite                      0.108183726 0.06435470
## Xdemo_race_hispanic1                             0.007371908 0.02701319
## Xinterview_age                                   0.026492384 0.02417874
## Xhormone_scr_ert_mean:putamen_posvsneg_feedback_z -0.034536143 0.05358400
```

Male participants

```
##
## Family: gaussian
## Link function: identity
##
## Formula:
## cbcl_scr_syn_internal_r ~ PDS_score + hormone_scr_ert_mean *
##     putamen_posvsneg_feedback_z + race.ethnicity.5level + demo_race_hispanic +
##     interview_age
##
## Parametric coefficients:
##                                                    Estimate Std. Error t value
## (Intercept)                                       3.4212043  2.2432021   1.525
## PDS_score                                         0.9108598  0.2593916   3.512
## hormone_scr_ert_mean                              0.0101042  0.0091255   1.107
## putamen_posvsneg_feedback_z                       0.0652580  0.3318316   0.197
## race.ethnicity.5levelBlack                       -0.5384444  1.0569821  -0.509
## race.ethnicity.5levelMixed                        0.4621367  1.0422003   0.443
## race.ethnicity.5levelOther                       -0.9086726  1.1579139  -0.785
## race.ethnicity.5levelWhite                        0.2273192  0.9827489   0.231
## demo_race_hispanic1                               0.7256384  0.3922100   1.850
## interview_age                                    -0.0010137  0.0170283  -0.060
## hormone_scr_ert_mean:putamen_posvsneg_feedback_z -0.0002025  0.0098202  -0.021
##                                                  Pr(>|t|)
## (Intercept)                                      0.127394
## PDS_score                                        0.000456 ***
## hormone_scr_ert_mean                             0.268333
## putamen_posvsneg_feedback_z                      0.844115
## race.ethnicity.5levelBlack                       0.610521
## race.ethnicity.5levelMixed                       0.657511
## race.ethnicity.5levelOther                       0.432701
## race.ethnicity.5levelWhite                       0.817100
## demo_race_hispanic1                              0.064455 .
## interview_age                                    0.952537
## hormone_scr_ert_mean:putamen_posvsneg_feedback_z 0.983553
## ---
## Signif. codes:  0 '***' 0.001 '**' 0.01 '*' 0.05 '.' 0.1 ' ' 1
##
##
```

```
## R-sq.(adj) =  0.00599
## lmer.REML =  11650  Scale est. = 14.468    n = 1862
```

```
##                                                      stdcoef      stdse
## X(Intercept)                                      0.00000000 0.00000000
## XPDS_score                                        0.08504802 0.02421969
## Xhormone_scr_ert_mean                             0.02640010 0.02384309
## Xputamen_posvsneg_feedback_z                      0.01036207 0.05269024
## Xrace.ethnicity.5levelBlack                      -0.02989509 0.05868494
## Xrace.ethnicity.5levelMixed                       0.02668395 0.06017705
## Xrace.ethnicity.5levelOther                      -0.03639825 0.04638198
## Xrace.ethnicity.5levelWhite                       0.01856307 0.08025205
## Xdemo_race_hispanic1                              0.05142886 0.02779747
## Xinterview_age                                   -0.00138511 0.02326782
## Xhormone_scr_ert_mean:putamen_posvsneg_feedback_z -0.00108478 0.05261611
```

4.20 Model: CBCL internalizing factor ~ Testosterone x Lateral OFC activity (anticipation stage) + PDS

Female participants

```
##
## Family: gaussian
## Link function: identity
##
## Formula:
## cbcl_scr_syn_internal_r ~ PDS_score + hormone_scr_ert_mean *
##     lOFC_rvsn_ant_z + race.ethnicity.5level + demo_race_hispanic +
##     interview_age
##
## Parametric coefficients:
##                                     Estimate Std. Error t value Pr(>|t|)
## (Intercept)                        -0.268446   2.100526  -0.128   0.8983
## PDS_score                           0.953244   0.192624   4.949 8.15e-07 ***
## hormone_scr_ert_mean               -0.001902   0.007873  -0.242   0.8091
## lOFC_rvsn_ant_z                     0.501329   0.495476   1.012   0.3118
## race.ethnicity.5levelBlack         -0.166435   0.811719  -0.205   0.8376
## race.ethnicity.5levelMixed          1.195701   0.801185   1.492   0.1358
## race.ethnicity.5levelOther          0.403243   0.951844   0.424   0.6719
## race.ethnicity.5levelWhite          1.486237   0.735959   2.019   0.0436 *
## demo_race_hispanic1                 0.002524   0.367034   0.007   0.9945
## interview_age                       0.021066   0.017312   1.217   0.2238
## hormone_scr_ert_mean:lOFC_rvsn_ant_z -0.009990   0.012957  -0.771   0.4408
## ---
## Signif. codes:  0 '***' 0.001 '**' 0.01 '*' 0.05 '.' 0.1 ' ' 1
##
##
## R-sq.(adj) =  0.0189
## lmer.REML =  11345  Scale est. = 15.991    n = 1842
```

```
##                                           stdcoef      stdse
## X(Intercept)                         0.0000000000 0.00000000
## XPDS_score                           0.1278516846 0.02583520
```

```
## Xhormone_scr_ert_mean                 -0.0059243780 0.02451841
## XlOFC_rvsn_ant_z                       0.0524735728 0.05186100
## Xrace.ethnicity.5levelBlack           -0.0105915542 0.05165602
## Xrace.ethnicity.5levelMixed            0.0734021160 0.04918341
## Xrace.ethnicity.5levelOther            0.0159753798 0.03770947
## Xrace.ethnicity.5levelWhite            0.1311092043 0.06492304
## Xdemo_race_hispanic1                   0.0001861502 0.02706619
## Xinterview_age                         0.0295293385 0.02426700
## Xhormone_scr_ert_mean:lOFC_rvsn_ant_z -0.0400247798 0.05191245
```

Male participants

```
##
## Family: gaussian
## Link function: identity
##
## Formula:
## cbcl_scr_syn_internal_r ~ PDS_score + hormone_scr_ert_mean *
##     lOFC_rvsn_ant_z + race.ethnicity.5level + demo_race_hispanic +
##     interview_age
##
## Parametric coefficients:
##                                       Estimate Std. Error t value Pr(>|t|)
## (Intercept)                           2.891112   2.229670   1.297  0.19491
## PDS_score                             0.842111   0.260970   3.227  0.00127 **
## hormone_scr_ert_mean                  0.010887   0.009072   1.200  0.23027
## lOFC_rvsn_ant_z                       0.329895   0.470479   0.701  0.48327
## race.ethnicity.5levelBlack           -0.668096   1.054888  -0.633  0.52660
## race.ethnicity.5levelMixed            0.401557   1.040518   0.386  0.69960
## race.ethnicity.5levelOther           -0.923551   1.151088  -0.802  0.42247
## race.ethnicity.5levelWhite            0.184257   0.980977   0.188  0.85103
## demo_race_hispanic1                   0.768203   0.392387   1.958  0.05041 .
## interview_age                         0.003968   0.016886   0.235  0.81424
## hormone_scr_ert_mean:lOFC_rvsn_ant_z -0.015283   0.013037  -1.172  0.24122
## ---
## Signif. codes:  0 '***' 0.001 '**' 0.01 '*' 0.05 '.' 0.1 ' ' 1
##
##
## R-sq.(adj) =  0.00656
## lmer.REML =  11497  Scale est. = 14.294    n = 1845
```

```
##                                           stdcoef      stdse
## X(Intercept)                           0.000000000 0.00000000
## XPDS_score                             0.078260764 0.02425300
## Xhormone_scr_ert_mean                  0.028764952 0.02396965
## XlOFC_rvsn_ant_z                       0.036472965 0.05201584
## Xrace.ethnicity.5levelBlack           -0.037148298 0.05865522
## Xrace.ethnicity.5levelMixed            0.023343517 0.06048788
## Xrace.ethnicity.5levelOther           -0.037732930 0.04702927
## Xrace.ethnicity.5levelWhite            0.015177014 0.08080190
## Xdemo_race_hispanic1                   0.055165308 0.02817762
## Xinterview_age                         0.005484628 0.02333986
## Xhormone_scr_ert_mean:lOFC_rvsn_ant_z -0.060981212 0.05201763
```

4.21 Model: CBCL internalizing factor ~ Testosterone x Medial OFC activity (anticipation stage) + PDS

Female participants

```
##
## Family: gaussian
## Link function: identity
##
## Formula:
## cbcl_scr_syn_internal_r ~ PDS_score + hormone_scr_ert_mean *
##     mOFC_rvsn_ant_z + race.ethnicity.5level + demo_race_hispanic +
##     interview_age
##
## Parametric coefficients:
##                                     Estimate Std. Error t value Pr(>|t|)
## (Intercept)                        -0.335356   2.103623  -0.159    0.873
## PDS_score                           0.953065   0.192757   4.944 8.34e-07 ***
## hormone_scr_ert_mean               -0.001117   0.007860  -0.142    0.887
## mOFC_rvsn_ant_z                     0.415906   0.424412   0.980    0.327
## race.ethnicity.5levelBlack         -0.176200   0.811137  -0.217    0.828
## race.ethnicity.5levelMixed          1.170896   0.801983   1.460    0.144
## race.ethnicity.5levelOther          0.367373   0.949882   0.387    0.699
## race.ethnicity.5levelWhite          1.498526   0.736273   2.035    0.042 *
## demo_race_hispanic1                -0.004546   0.367034  -0.012    0.990
## interview_age                       0.021341   0.017329   1.232    0.218
## hormone_scr_ert_mean:mOFC_rvsn_ant_z -0.006879   0.010767  -0.639    0.523
## ---
## Signif. codes:  0 '***' 0.001 '**' 0.01 '*' 0.05 '.' 0.1 ' ' 1
##
##
## R-sq.(adj) =  0.019
## lmer.REML =  11351  Scale est. = 15.87     n = 1843
```

```
##                                           stdcoef      stdse
## X(Intercept)                         0.0000000000 0.00000000
## XPDS_score                           0.1279704547 0.02588203
## Xhormone_scr_ert_mean               -0.0034799949 0.02449159
## XmOFC_rvsn_ant_z                     0.0501705356 0.05119663
## Xrace.ethnicity.5levelBlack         -0.0112487431 0.05178357
## Xrace.ethnicity.5levelMixed          0.0718630435 0.04922126
## Xrace.ethnicity.5levelOther          0.0146298691 0.03782711
## Xrace.ethnicity.5levelWhite          0.1323339346 0.06501977
## Xdemo_race_hispanic1                -0.0003355565 0.02708967
## Xinterview_age                       0.0298629974 0.02424853
## Xhormone_scr_ert_mean:mOFC_rvsn_ant_z -0.0323540477 0.05064467
```

Male participants

```
##
## Family: gaussian
## Link function: identity
##
```

```
## Formula:
## cbcl_scr_syn_internal_r ~ PDS_score + hormone_scr_ert_mean *
##     mOFC_rvsn_ant_z + race.ethnicity.5level + demo_race_hispanic +
##     interview_age
##
## Parametric coefficients:
##                                      Estimate Std. Error t value Pr(>|t|)
## (Intercept)                          2.732943   2.241645   1.219 0.222937
## PDS_score                            0.918333   0.261391   3.513 0.000453 ***
## hormone_scr_ert_mean                 0.010503   0.009145   1.148 0.250927
## mOFC_rvsn_ant_z                     -0.080535   0.406891  -0.198 0.843123
## race.ethnicity.5levelBlack          -0.479505   1.059785  -0.452 0.650995
## race.ethnicity.5levelMixed           0.435798   1.045677   0.417 0.676902
## race.ethnicity.5levelOther          -0.853598   1.158486  -0.737 0.461325
## race.ethnicity.5levelWhite           0.220957   0.986898   0.224 0.822868
## demo_race_hispanic1                  0.725662   0.393610   1.844 0.065401 .
## interview_age                        0.004231   0.016984   0.249 0.803280
## hormone_scr_ert_mean:mOFC_rvsn_ant_z -0.002629   0.011546  -0.228 0.819926
## ---
## Signif. codes:  0 '***' 0.001 '**' 0.01 '*' 0.05 '.' 0.1 ' ' 1
##
##
## R-sq.(adj) =  0.00596
## lmer.REML =  11551  Scale est. = 14.216    n = 1850
```

```
##                                           stdcoef      stdse
## X(Intercept)                          0.000000000 0.00000000
## XPDS_score                            0.085168372 0.02424198
## Xhormone_scr_ert_mean                 0.027561145 0.02399827
## XmOFC_rvsn_ant_z                     -0.010173777 0.05140129
## Xrace.ethnicity.5levelBlack          -0.026667800 0.05894026
## Xrace.ethnicity.5levelMixed           0.025321201 0.06075709
## Xrace.ethnicity.5levelOther          -0.034647091 0.04702233
## Xrace.ethnicity.5levelWhite           0.018147146 0.08105381
## Xdemo_race_hispanic1                  0.051729398 0.02805882
## Xinterview_age                        0.005802362 0.02328953
## Xhormone_scr_ert_mean:mOFC_rvsn_ant_z -0.011738873 0.05156027
```

4.22 Model: CBCL internalizing factor ~ Testosterone x Lateral OFC activity (feedback stage) + PDS

Female participants

```
##
## Family: gaussian
## Link function: identity
##
## Formula:
## cbcl_scr_syn_internal_r ~ PDS_score + hormone_scr_ert_mean *
##     lOFC_posvsneg_feedback_z + race.ethnicity.5level + demo_race_hispanic +
##     interview_age
##
## Parametric coefficients:
```

```
##                                              Estimate Std. Error t value
## (Intercept)                                 -0.044937   2.092752  -0.021
## PDS_score                                    0.925586   0.192200   4.816
## hormone_scr_ert_mean                        -0.001409   0.007855  -0.179
## lOFC_posvsneg_feedback_z                     0.007051   0.551494   0.013
## race.ethnicity.5levelBlack                  -0.395454   0.802080  -0.493
## race.ethnicity.5levelMixed                   0.886869   0.792606   1.119
## race.ethnicity.5levelOther                  -0.015041   0.943164  -0.016
## race.ethnicity.5levelWhite                   1.204774   0.726589   1.658
## demo_race_hispanic1                          0.062441   0.366832   0.170
## interview_age                                0.021466   0.017291   1.241
## hormone_scr_ert_mean:lOFC_posvsneg_feedback_z -0.002902   0.014645  -0.198
##                                             Pr(>|t|)
## (Intercept)                                   0.9829
## PDS_score                                   1.59e-06 ***
## hormone_scr_ert_mean                          0.8576
## lOFC_posvsneg_feedback_z                      0.9898
## race.ethnicity.5levelBlack                    0.6220
## race.ethnicity.5levelMixed                    0.2633
## race.ethnicity.5levelOther                    0.9873
## race.ethnicity.5levelWhite                    0.0975 .
## demo_race_hispanic1                           0.8649
## interview_age                                 0.2146
## hormone_scr_ert_mean:lOFC_posvsneg_feedback_z   0.8430
## ---
## Signif. codes:  0 '***' 0.001 '**' 0.01 '*' 0.05 '.' 0.1 ' ' 1
##
##
## R-sq.(adj) =  0.0166
## lmer.REML =  11373  Scale est. = 16.334    n = 1848
```

```
##                                                     stdcoef      stdse
## X(Intercept)                                  0.0000000000 0.00000000
## XPDS_score                                    0.1244900328 0.02585069
## Xhormone_scr_ert_mean                        -0.0043951804 0.02449627
## XlOFC_posvsneg_feedback_z                     0.0006557489 0.05129109
## Xrace.ethnicity.5levelBlack                  -0.0253100900 0.05133526
## Xrace.ethnicity.5levelMixed                   0.0546712212 0.04886035
## Xrace.ethnicity.5levelOther                  -0.0005971492 0.03744558
## Xrace.ethnicity.5levelWhite                   0.1067558820 0.06438361
## Xdemo_race_hispanic1                          0.0046158893 0.02711757
## Xinterview_age                                0.0301766957 0.02430784
## Xhormone_scr_ert_mean:lOFC_posvsneg_feedback_z -0.0101490938 0.05121960
```

Male participants

```
##
## Family: gaussian
## Link function: identity
##
## Formula:
## cbcl_scr_syn_internal_r ~ PDS_score + hormone_scr_ert_mean *
##     lOFC_posvsneg_feedback_z + race.ethnicity.5level + demo_race_hispanic +
```

```
##     interview_age
##
## Parametric coefficients:
##                                              Estimate Std. Error t value
## (Intercept)                                 3.3821100  2.2425897   1.508
## PDS_score                                   0.9037521  0.2611570   3.461
## hormone_scr_ert_mean                        0.0106303  0.0091154   1.166
## lOFC_posvsneg_feedback_z                    0.0444634  0.5295193   0.084
## race.ethnicity.5levelBlack                 -0.6391895  1.0637349  -0.601
## race.ethnicity.5levelMixed                  0.4108271  1.0483152   0.392
## race.ethnicity.5levelOther                 -0.9633903  1.1604815  -0.830
## race.ethnicity.5levelWhite                  0.2085606  0.9879463   0.211
## demo_race_hispanic1                         0.7762967  0.3944475   1.968
## interview_age                              -0.0004695  0.0170015  -0.028
## hormone_scr_ert_mean:lOFC_posvsneg_feedback_z  0.0058190  0.0149572   0.389
##                                            Pr(>|t|)
## (Intercept)                                0.131694
## PDS_score                                  0.000551 ***
## hormone_scr_ert_mean                       0.243688
## lOFC_posvsneg_feedback_z                   0.933090
## race.ethnicity.5levelBlack                 0.547986
## race.ethnicity.5levelMixed                 0.695183
## race.ethnicity.5levelOther                 0.406554
## race.ethnicity.5levelWhite                 0.832829
## demo_race_hispanic1                        0.049212 *
## interview_age                              0.977971
## hormone_scr_ert_mean:lOFC_posvsneg_feedback_z 0.697287
## ---
## Signif. codes:  0 '***' 0.001 '**' 0.01 '*' 0.05 '.' 0.1 ' ' 1
##
##
## R-sq.(adj) =  0.00623
## lmer.REML =  11515  Scale est. = 14.231    n = 1844
```

```
##                                                    stdcoef      stdse
## X(Intercept)                                  0.0000000000 0.00000000
## XPDS_score                                    0.0841017340 0.02430286
## Xhormone_scr_ert_mean                         0.0279390510 0.02395747
## XlOFC_posvsneg_feedback_z                     0.0043392492 0.05167662
## Xrace.ethnicity.5levelBlack                  -0.0352509079 0.05866432
## Xrace.ethnicity.5levelMixed                   0.0236456850 0.06033713
## Xrace.ethnicity.5levelOther                  -0.0389431070 0.04691012
## Xrace.ethnicity.5levelWhite                   0.0170392495 0.08071449
## Xdemo_race_hispanic1                          0.0552414329 0.02806897
## Xinterview_age                               -0.0006446586 0.02334362
## Xhormone_scr_ert_mean:lOFC_posvsneg_feedback_z  0.0201473703 0.05178655
```

4.23 Model: CBCL internalizing factor ~ Testosterone x Medial OFC activity (feedback stage) + PDS

Female participants

```
##
```

```
## Family: gaussian
## Link function: identity
##
## Formula:
## cbcl_scr_syn_internal_r ~ PDS_score + hormone_scr_ert_mean *
##     mOFC_posvsneg_feedback_z + race.ethnicity.5level + demo_race_hispanic +
##     interview_age
##
## Parametric coefficients:
##                                             Estimate Std. Error t value
## (Intercept)                                -0.314760   2.093249  -0.150
## PDS_score                                   0.954451   0.191597   4.982
## hormone_scr_ert_mean                       -0.001444   0.007850  -0.184
## mOFC_posvsneg_feedback_z                    0.425476   0.479556   0.887
## race.ethnicity.5levelBlack                 -0.145210   0.809952  -0.179
## race.ethnicity.5levelMixed                  1.225959   0.799258   1.534
## race.ethnicity.5levelOther                  0.225314   0.948784   0.237
## race.ethnicity.5levelWhite                  1.475471   0.734587   2.009
## demo_race_hispanic1                         0.036169   0.366841   0.099
## interview_age                               0.021263   0.017250   1.233
## hormone_scr_ert_mean:mOFC_posvsneg_feedback_z -0.006730   0.012439  -0.541
##                                            Pr(>|t|)
## (Intercept)                                  0.8805
## PDS_score                                   6.9e-07 ***
## hormone_scr_ert_mean                         0.8540
## mOFC_posvsneg_feedback_z                     0.3751
## race.ethnicity.5levelBlack                   0.8577
## race.ethnicity.5levelMixed                   0.1252
## race.ethnicity.5levelOther                   0.8123
## race.ethnicity.5levelWhite                   0.0447 *
## demo_race_hispanic1                          0.9215
## interview_age                                0.2179
## hormone_scr_ert_mean:mOFC_posvsneg_feedback_z   0.5886
## ---
## Signif. codes:  0 '***' 0.001 '**' 0.01 '*' 0.05 '.' 0.1 ' ' 1
##
##
## R-sq.(adj) =  0.019
## lmer.REML =  11407  Scale est. = 16.118    n = 1853
```

```
##                                                  stdcoef      stdse
## X(Intercept)                                 0.000000000 0.00000000
## XPDS_score                                   0.128352481 0.02576556
## Xhormone_scr_ert_mean                       -0.004492190 0.02441558
## XmOFC_posvsneg_feedback_z                    0.047172633 0.05316858
## Xrace.ethnicity.5levelBlack                 -0.009278331 0.05175266
## Xrace.ethnicity.5levelMixed                  0.075602429 0.04928865
## Xrace.ethnicity.5levelOther                  0.008914153 0.03753696
## Xrace.ethnicity.5levelWhite                  0.130468821 0.06495598
## Xdemo_race_hispanic1                         0.002670764 0.02708800
## Xinterview_age                               0.029828940 0.02419954
## Xhormone_scr_ert_mean:mOFC_posvsneg_feedback_z -0.028800309 0.05323414
```

Male participants

```
##
## Family: gaussian
## Link function: identity
##
## Formula:
## cbcl_scr_syn_internal_r ~ PDS_score + hormone_scr_ert_mean *
##     mOFC_posvsneg_feedback_z + race.ethnicity.5level + demo_race_hispanic +
##     interview_age
##
## Parametric coefficients:
##                                              Estimate Std. Error t value
## (Intercept)                                 3.2404858  2.2355838   1.450
## PDS_score                                   0.9119659  0.2603544   3.503
## hormone_scr_ert_mean                        0.0119804  0.0090956   1.317
## mOFC_posvsneg_feedback_z                   -0.4701557  0.4399350  -1.069
## race.ethnicity.5levelBlack                 -0.7177073  1.0605834  -0.677
## race.ethnicity.5levelMixed                  0.4228034  1.0452461   0.405
## race.ethnicity.5levelOther                 -0.9602210  1.1572107  -0.830
## race.ethnicity.5levelWhite                  0.1925760  0.9856307   0.195
## demo_race_hispanic1                         0.7790182  0.3912546   1.991
## interview_age                               0.0004702  0.0169469   0.028
## hormone_scr_ert_mean:mOFC_posvsneg_feedback_z  0.0187015  0.0125052   1.496
##                                            Pr(>|t|)
## (Intercept)                                0.147368
## PDS_score                                  0.000471 ***
## hormone_scr_ert_mean                       0.187945
## mOFC_posvsneg_feedback_z                   0.285348
## race.ethnicity.5levelBlack                 0.498675
## race.ethnicity.5levelMixed                 0.685891
## race.ethnicity.5levelOther                 0.406775
## race.ethnicity.5levelWhite                 0.845114
## demo_race_hispanic1                        0.046620 *
## interview_age                              0.977867
## hormone_scr_ert_mean:mOFC_posvsneg_feedback_z 0.134955
## ---
## Signif. codes:  0 '***' 0.001 '**' 0.01 '*' 0.05 '.' 0.1 ' ' 1
##
##
## R-sq.(adj) =  0.00751
## lmer.REML =  11547  Scale est. = 14.247    n = 1850
```

```
##                                                    stdcoef      stdse
## X(Intercept)                                  0.0000000000 0.00000000
## XPDS_score                                    0.0848484940 0.02422315
## Xhormone_scr_ert_mean                         0.0315163343 0.02392728
## XmOFC_posvsneg_feedback_z                    -0.0544885170 0.05098610
## Xrace.ethnicity.5levelBlack                  -0.0397475842 0.05873652
## Xrace.ethnicity.5levelMixed                   0.0244261633 0.06038588
## Xrace.ethnicity.5levelOther                  -0.0389837909 0.04698133
## Xrace.ethnicity.5levelWhite                   0.0157783620 0.08075584
## Xdemo_race_hispanic1                          0.0556610101 0.02795522
## Xinterview_age                                0.0006462811 0.02329168
```

```
## Xhormone_scr_ert_mean:mOFC_posvsneg_feedback_z  0.0763436863 0.05104881
```

4.24 Model: CBCL internalizing factor ~ Testosterone x BIS-BAS RR + PDS

Female participants

```
##
## Family: gaussian
## Link function: identity
##
## Formula:
## cbcl_scr_syn_internal_r ~ PDS_score + hormone_scr_ert_mean *
##     bisbas_ss_basm_rr + race.ethnicity.5level + demo_race_hispanic +
##     interview_age
##
## Parametric coefficients:
##                                     Estimate Std. Error t value Pr(>|t|)
## (Intercept)                        -0.530598   2.090056  -0.254    0.800
## PDS_score                           0.864135   0.169223   5.106 3.54e-07
## hormone_scr_ert_mean                0.026192   0.025996   1.008    0.314
## bisbas_ss_basm_rr                   0.091454   0.107910   0.848    0.397
## race.ethnicity.5levelBlack         -0.424060   0.728587  -0.582    0.561
## race.ethnicity.5levelMixed          1.164004   0.726406   1.602    0.109
## race.ethnicity.5levelOther          0.225036   0.843315   0.267    0.790
## race.ethnicity.5levelWhite          0.991807   0.669711   1.481    0.139
## demo_race_hispanic1                 0.175401   0.330640   0.530    0.596
## interview_age                       0.021104   0.015400   1.370    0.171
## hormone_scr_ert_mean:bisbas_ss_basm_rr -0.003168   0.002824  -1.122    0.262
##
## (Intercept)
## PDS_score                          ***
## hormone_scr_ert_mean
## bisbas_ss_basm_rr
## race.ethnicity.5levelBlack
## race.ethnicity.5levelMixed
## race.ethnicity.5levelOther
## race.ethnicity.5levelWhite
## demo_race_hispanic1
## interview_age
## hormone_scr_ert_mean:bisbas_ss_basm_rr
## ---
## Signif. codes:  0 '***' 0.001 '**' 0.01 '*' 0.05 '.' 0.1 ' ' 1
##
##
## R-sq.(adj) =  0.0149
## lmer.REML =  14837  Scale est. = 17.701    n = 2402
```

```
##                                          stdcoef      stdse
## X(Intercept)                          0.00000000 0.00000000
## XPDS_score                            0.11545922 0.02261028
## Xhormone_scr_ert_mean                 0.07982666 0.07923060
## Xbisbas_ss_basm_rr                    0.04052063 0.04781192
## Xrace.ethnicity.5levelBlack          -0.02847250 0.04891917
```

```
## Xrace.ethnicity.5levelMixed              0.07020298 0.04381071
## Xrace.ethnicity.5levelOther              0.00912452 0.03419382
## Xrace.ethnicity.5levelWhite              0.08801248 0.05942978
## Xdemo_race_hispanic1                     0.01278432 0.02409909
## Xinterview_age                           0.02924195 0.02133847
## Xhormone_scr_ert_mean:bisbas_ss_basm_rr -0.10159039 0.09056734
```

Male participants

```
##
## Family: gaussian
## Link function: identity
##
## Formula:
## cbcl_scr_syn_internal_r ~ PDS_score + hormone_scr_ert_mean *
##     bisbas_ss_basm_rr + race.ethnicity.5level + demo_race_hispanic +
##     interview_age
##
## Parametric coefficients:
##                                      Estimate Std. Error t value Pr(>|t|)
## (Intercept)                          3.541810   2.130549   1.662  0.09655
## PDS_score                            0.770185   0.208179   3.700  0.00022
## hormone_scr_ert_mean                 0.018870   0.028738   0.657  0.51148
## bisbas_ss_basm_rr                   -0.026812   0.108613  -0.247  0.80504
## race.ethnicity.5levelBlack          -1.003452   0.815218  -1.231  0.21847
## race.ethnicity.5levelMixed           0.260081   0.807094   0.322  0.74729
## race.ethnicity.5levelOther          -0.736114   0.906751  -0.812  0.41697
## race.ethnicity.5levelWhite          -0.009121   0.755209  -0.012  0.99036
## demo_race_hispanic1                  0.410597   0.332495   1.235  0.21698
## interview_age                        0.004335   0.014488   0.299  0.76480
## hormone_scr_ert_mean:bisbas_ss_basm_rr -0.001179   0.003129  -0.377  0.70628
##
## (Intercept)                         .
## PDS_score                           ***
## hormone_scr_ert_mean
## bisbas_ss_basm_rr
## race.ethnicity.5levelBlack
## race.ethnicity.5levelMixed
## race.ethnicity.5levelOther
## race.ethnicity.5levelWhite
## demo_race_hispanic1
## interview_age
## hormone_scr_ert_mean:bisbas_ss_basm_rr
## ---
## Signif. codes:  0 '***' 0.001 '**' 0.01 '*' 0.05 '.' 0.1 ' ' 1
##
##
## R-sq.(adj) =  0.00473
## lmer.REML =  16313  Scale est. = 15.538    n = 2614
```

```
##                                               stdcoef      stdse
## X(Intercept)                             0.0000000000 0.00000000
## XPDS_score                               0.0757642639 0.02047886
```

```
## Xhormone_scr_ert_mean                    0.0503573544 0.07669158
## Xbisbas_ss_basm_rr                      -0.0111410122 0.04513166
## Xrace.ethnicity.5levelBlack             -0.0602361205 0.04893667
## Xrace.ethnicity.5levelMixed              0.0152234611 0.04724204
## Xrace.ethnicity.5levelOther             -0.0297382850 0.03663183
## Xrace.ethnicity.5levelWhite             -0.0007676174 0.06355737
## Xdemo_race_hispanic1                     0.0295746037 0.02394908
## Xinterview_age                           0.0059123612 0.01975949
## Xhormone_scr_ert_mean:bisbas_ss_basm_rr -0.0324415348 0.08607420
```

4.25 Model: CBCL internalizing factor ~ Testosterone x MID Reaction Time + PDS (large reward vs. neutral)

Female participants

```
##
## Family: gaussian
## Link function: identity
##
## Formula:
## cbcl_scr_syn_internal_r ~ PDS_score + hormone_scr_ert_mean *
##     rt_diff_large_neutral_z + race.ethnicity.5level + demo_race_hispanic +
##     interview_age
##
## Parametric coefficients:
##                                          Estimate Std. Error t value
## (Intercept)                              0.042720   2.026723   0.021
## PDS_score                                1.065186   0.186508   5.711
## hormone_scr_ert_mean                    -0.002569   0.007697  -0.334
## rt_diff_large_neutral_z                 -0.287147   0.292287  -0.982
## race.ethnicity.5levelBlack              -0.666256   0.777076  -0.857
## race.ethnicity.5levelMixed               0.643475   0.769832   0.836
## race.ethnicity.5levelOther               0.053665   0.891478   0.060
## race.ethnicity.5levelWhite               0.922364   0.707373   1.304
## demo_race_hispanic1                      0.207978   0.356637   0.583
## interview_age                            0.021349   0.016786   1.272
## hormone_scr_ert_mean:rt_diff_large_neutral_z  0.013061   0.007519   1.737
##                                         Pr(>|t|)
## (Intercept)                               0.9832
## PDS_score                               1.29e-08 ***
## hormone_scr_ert_mean                      0.7386
## rt_diff_large_neutral_z                   0.3260
## race.ethnicity.5levelBlack                0.3913
## race.ethnicity.5levelMixed                0.4033
## race.ethnicity.5levelOther                0.9520
## race.ethnicity.5levelWhite                0.1924
## demo_race_hispanic1                       0.5598
## interview_age                             0.2036
## hormone_scr_ert_mean:rt_diff_large_neutral_z   0.0826 .
## ---
## Signif. codes:  0 '***' 0.001 '**' 0.01 '*' 0.05 '.' 0.1 ' ' 1
##
##
```

```
## R-sq.(adj) =  0.0216
## lmer.REML =  12398  Scale est. = 16.797    n = 2010
```

```
##                                                    stdcoef      stdse
## X(Intercept)                                   0.000000000 0.00000000
## XPDS_score                                     0.140203722 0.02454889
## Xhormone_scr_ert_mean                         -0.007831063 0.02345907
## Xrt_diff_large_neutral_z                      -0.049511799 0.05039813
## Xrace.ethnicity.5levelBlack                   -0.042651042 0.04974531
## Xrace.ethnicity.5levelMixed                    0.039377712 0.04711012
## Xrace.ethnicity.5levelOther                    0.002224191 0.03694784
## Xrace.ethnicity.5levelWhite                    0.081403532 0.06242940
## Xdemo_race_hispanic1                           0.015280632 0.02620300
## Xinterview_age                                 0.029468446 0.02317054
## Xhormone_scr_ert_mean:rt_diff_large_neutral_z  0.087369050 0.05030100
```

Male participants

```
##
## Family: gaussian
## Link function: identity
##
## Formula:
## cbcl_scr_syn_internal_r ~ PDS_score + hormone_scr_ert_mean *
##     rt_diff_large_neutral_z + race.ethnicity.5level + demo_race_hispanic +
##     interview_age
##
## Parametric coefficients:
##                                               Estimate Std. Error t value
## (Intercept)                                  3.4300417  2.1051416   1.629
## PDS_score                                    0.9298927  0.2375120   3.915
## hormone_scr_ert_mean                         0.0138252  0.0084648   1.633
## rt_diff_large_neutral_z                     -0.0228206  0.3049842  -0.075
## race.ethnicity.5levelBlack                  -1.2181199  0.9857758  -1.236
## race.ethnicity.5levelMixed                  -0.2335701  0.9782657  -0.239
## race.ethnicity.5levelOther                  -1.3342474  1.0790027  -1.237
## race.ethnicity.5levelWhite                  -0.4363430  0.9211772  -0.474
## demo_race_hispanic1                          0.5510513  0.3683855   1.496
## interview_age                                0.0031154  0.0159261   0.196
## hormone_scr_ert_mean:rt_diff_large_neutral_z -0.0006389  0.0087567  -0.073
##                                              Pr(>|t|)
## (Intercept)                                     0.103
## PDS_score                                    9.33e-05 ***
## hormone_scr_ert_mean                            0.103
## rt_diff_large_neutral_z                         0.940
## race.ethnicity.5levelBlack                      0.217
## race.ethnicity.5levelMixed                      0.811
## race.ethnicity.5levelOther                      0.216
## race.ethnicity.5levelWhite                      0.636
## demo_race_hispanic1                             0.135
## interview_age                                   0.845
## hormone_scr_ert_mean:rt_diff_large_neutral_z    0.942
## ---
```

```
## Signif. codes:  0 '***' 0.001 '**' 0.01 '*' 0.05 '.' 0.1 ' ' 1
##
##
## R-sq.(adj) =  0.00634
## lmer.REML =  13051  Scale est. = 12.671    n = 2094
```

```
##                                                      stdcoef      stdse
## X(Intercept)                                     0.000000000 0.00000000
## XPDS_score                                       0.089299930 0.02280887
## Xhormone_scr_ert_mean                            0.036813723 0.02253999
## Xrt_diff_large_neutral_z                        -0.003787871 0.05062271
## Xrace.ethnicity.5levelBlack                     -0.070136835 0.05675894
## Xrace.ethnicity.5levelMixed                     -0.013544986 0.05673071
## Xrace.ethnicity.5levelOther                     -0.053704843 0.04343098
## Xrace.ethnicity.5levelWhite                     -0.036154708 0.07632733
## Xdemo_race_hispanic1                             0.039378652 0.02632518
## Xinterview_age                                   0.004289967 0.02193076
## Xhormone_scr_ert_mean:rt_diff_large_neutral_z -0.003701739 0.05073272
```

4.26 Model: CBCL internalizing factor ~ Testosterone x MID Reaction Time + PDS (large vs. small reward)

Female participants

```
##
## Family: gaussian
## Link function: identity
##
## Formula:
## cbcl_scr_syn_internal_r ~ PDS_score + hormone_scr_ert_mean *
##     rt_diff_large_small_z + race.ethnicity.5level + demo_race_hispanic +
##     interview_age
##
## Parametric coefficients:
##                                              Estimate Std. Error t value
## (Intercept)                                 -0.063616   2.023539  -0.031
## PDS_score                                    1.067081   0.186445   5.723
## hormone_scr_ert_mean                        -0.001765   0.007686  -0.230
## rt_diff_large_small_z                       -0.013276   0.277035  -0.048
## race.ethnicity.5levelBlack                  -0.616689   0.777151  -0.794
## race.ethnicity.5levelMixed                   0.685774   0.770222   0.890
## race.ethnicity.5levelOther                   0.084138   0.891335   0.094
## race.ethnicity.5levelWhite                   0.959070   0.707328   1.356
## demo_race_hispanic1                          0.185129   0.356411   0.519
## interview_age                                0.021724   0.016773   1.295
## hormone_scr_ert_mean:rt_diff_large_small_z  0.008115   0.007345   1.105
##                                             Pr(>|t|)
## (Intercept)                                    0.975
## PDS_score                                    1.2e-08 ***
## hormone_scr_ert_mean                           0.818
## rt_diff_large_small_z                          0.962
## race.ethnicity.5levelBlack                     0.428
## race.ethnicity.5levelMixed                     0.373
```

```
## race.ethnicity.5levelOther                     0.925
## race.ethnicity.5levelWhite                     0.175
## demo_race_hispanic1                            0.604
## interview_age                                  0.195
## hormone_scr_ert_mean:rt_diff_large_small_z    0.269
## ---
## Signif. codes:  0 '***' 0.001 '**' 0.01 '*' 0.05 '.' 0.1 ' ' 1
##
##
## R-sq.(adj) =  0.0227
## lmer.REML =  12397  Scale est. = 16.842    n = 2010
```

```
##                                                 stdcoef      stdse
## X(Intercept)                                0.000000000 0.00000000
## XPDS_score                                  0.140453190 0.02454059
## Xhormone_scr_ert_mean                      -0.005380816 0.02342617
## Xrt_diff_large_small_z                     -0.002385291 0.04977576
## Xrace.ethnicity.5levelBlack                -0.039477974 0.04975010
## Xrace.ethnicity.5levelMixed                 0.041966213 0.04713404
## Xrace.ethnicity.5levelOther                 0.003487131 0.03694191
## Xrace.ethnicity.5levelWhite                 0.084643049 0.06242543
## Xdemo_race_hispanic1                        0.013601884 0.02618636
## Xinterview_age                              0.029985943 0.02315189
## Xhormone_scr_ert_mean:rt_diff_large_small_z  0.055036536 0.04981308
```

Male participants

```
##
## Family: gaussian
## Link function: identity
##
## Formula:
## cbcl_scr_syn_internal_r ~ PDS_score + hormone_scr_ert_mean *
##     rt_diff_large_small_z + race.ethnicity.5level + demo_race_hispanic +
##     interview_age
##
## Parametric coefficients:
##                                             Estimate Std. Error t value
## (Intercept)                                 3.401014   2.103254   1.617
## PDS_score                                   0.937938   0.237736   3.945
## hormone_scr_ert_mean                        0.013567   0.008466   1.603
## rt_diff_large_small_z                       0.145048   0.305116   0.475
## race.ethnicity.5levelBlack                 -1.201490   0.985328  -1.219
## race.ethnicity.5levelMixed                 -0.243791   0.977497  -0.249
## race.ethnicity.5levelOther                 -1.327882   1.078007  -1.232
## race.ethnicity.5levelWhite                 -0.438065   0.920295  -0.476
## demo_race_hispanic1                         0.555928   0.368650   1.508
## interview_age                               0.003316   0.015908   0.208
## hormone_scr_ert_mean:rt_diff_large_small_z -0.007265   0.008915  -0.815
##                                            Pr(>|t|)
## (Intercept)                                   0.106
## PDS_score                                  8.23e-05 ***
## hormone_scr_ert_mean                          0.109
```

```
## rt_diff_large_small_z                          0.635
## race.ethnicity.5levelBlack                     0.223
## race.ethnicity.5levelMixed                     0.803
## race.ethnicity.5levelOther                     0.218
## race.ethnicity.5levelWhite                     0.634
## demo_race_hispanic1                            0.132
## interview_age                                  0.835
## hormone_scr_ert_mean:rt_diff_large_small_z    0.415
## ---
## Signif. codes:  0 '***' 0.001 '**' 0.01 '*' 0.05 '.' 0.1 ' ' 1
##
##
## R-sq.(adj) =  0.00666
## lmer.REML =  13050  Scale est. = 12.59     n = 2094
```

```
##                                                   stdcoef      stdse
## X(Intercept)                                  0.000000000 0.00000000
## XPDS_score                                    0.090072527 0.02283043
## Xhormone_scr_ert_mean                         0.036127311 0.02254277
## Xrt_diff_large_small_z                        0.023911333 0.05029860
## Xrace.ethnicity.5levelBlack                  -0.069179314 0.05673318
## Xrace.ethnicity.5levelMixed                  -0.014137705 0.05668611
## Xrace.ethnicity.5levelOther                  -0.053448628 0.04339089
## Xrace.ethnicity.5levelWhite                  -0.036297356 0.07625423
## Xdemo_race_hispanic1                          0.039727158 0.02634405
## Xinterview_age                                0.004566693 0.02190639
## Xhormone_scr_ert_mean:rt_diff_large_small_z -0.041011051 0.05032665
```

5— Correlation Matrix —

Female participants

x1	x2	N	corr	p
bmi	interview_age	2661	0.0697979271	0.000314298098
PDS_score	interview_age	2691	0.2677278740	0.000000000000
PDS_score	bmi	2661	0.2664128834	0.000000000000
cbcl_scr_syn_internal_r	interview_age	2689	0.0280218610	0.146307300053
cbcl_scr_syn_internal_r	bmi	2659	0.0167186288	0.388820050048
cbcl_scr_syn_internal_r	PDS_score	2689	0.0792744882	0.000038652507
hormone_scr_ert_mean_z	interview_age	2478	0.2047640705	0.000000000000
hormone_scr_ert_mean_z	bmi	2449	0.2012793974	0.000000000000
hormone_scr_ert_mean_z	PDS_score	2478	0.3093491991	0.000000000000
hormone_scr_ert_mean_z	cbcl_scr_syn_internal_r	2476	0.0043682310	0.828012020633
bisbas_ss_basm_rr_z	interview_age	2683	-0.0350871908	0.069194488613
bisbas_ss_basm_rr_z	bmi	2653	0.0877629223	0.000005981667
bisbas_ss_basm_rr_z	PDS_score	2683	0.0509531725	0.008296938217
bisbas_ss_basm_rr_z	cbcl_scr_syn_internal_r	2681	-0.0076173230	0.693408003127
bisbas_ss_basm_rr_z	hormone_scr_ert_mean_z	2470	0.0122127956	0.544063120481
rt_diff_large_neutral_z	interview_age	2258	0.0433000471	0.039650139681
rt_diff_large_neutral_z	bmi	2236	0.0064987983	0.758739908243
rt_diff_large_neutral_z	PDS_score	2258	0.0270868742	0.198216746294
rt_diff_large_neutral_z	cbcl_scr_syn_internal_r	2256	0.0170572357	0.418064501460
rt_diff_large_neutral_z	hormone_scr_ert_mean_z	2085	-0.0146554784	0.503603840224
rt_diff_large_neutral_z	bisbas_ss_basm_rr_z	2252	-0.0089581753	0.670920599276
rt_diff_large_small_z	interview_age	2265	0.0387203151	0.065409130890
rt_diff_large_small_z	bmi	2243	0.0191346959	0.365040078316
rt_diff_large_small_z	PDS_score	2265	0.0081123862	0.699586997350
rt_diff_large_small_z	cbcl_scr_syn_internal_r	2263	0.0246669300	0.240811584762
rt_diff_large_small_z	hormone_scr_ert_mean_z	2093	-0.0043207507	0.843393848595
rt_diff_large_small_z	bisbas_ss_basm_rr_z	2259	-0.0097351385	0.643754590861
rt_diff_large_small_z	rt_diff_large_neutral_z	2239	0.4153704338	0.000000000000
accumbens_rvsn_ant_z	interview_age	2256	-0.0176046681	0.403280509753
accumbens_rvsn_ant_z	bmi	2233	-0.0344483546	0.103648829348
accumbens_rvsn_ant_z	PDS_score	2256	-0.0297940098	0.157166213595
accumbens_rvsn_ant_z	cbcl_scr_syn_internal_r	2255	-0.0297104272	0.158426512633
accumbens_rvsn_ant_z	hormone_scr_ert_mean_z	2082	-0.0078304345	0.721027940906
accumbens_rvsn_ant_z	bisbas_ss_basm_rr_z	2249	0.0250223855	0.235551943639
accumbens_rvsn_ant_z	rt_diff_large_neutral_z	2118	0.0016282449	0.940301861162
accumbens_rvsn_ant_z	rt_diff_large_small_z	2125	0.0280878101	0.195569562859
caudate_rvsn_ant_z	interview_age	2265	0.0303692292	0.148494896103
caudate_rvsn_ant_z	bmi	2242	-0.0298576697	0.157573349804
caudate_rvsn_ant_z	PDS_score	2265	-0.0107774820	0.608193482802
caudate_rvsn_ant_z	cbcl_scr_syn_internal_r	2263	0.0135449263	0.519562969018
caudate_rvsn_ant_z	hormone_scr_ert_mean_z	2091	-0.0199724271	0.361330294647
caudate_rvsn_ant_z	bisbas_ss_basm_rr_z	2258	0.0065029299	0.757442405558
caudate_rvsn_ant_z	rt_diff_large_neutral_z	2126	-0.0063639367	0.769321588379
caudate_rvsn_ant_z	rt_diff_large_small_z	2133	0.0045931614	0.832099833472
caudate_rvsn_ant_z	accumbens_rvsn_ant_z	2244	0.5145685384	0.000000000000
putamen_rvsn_ant_z	interview_age	2266	0.0392854181	0.061515089681
putamen_rvsn_ant_z	bmi	2243	-0.0339508622	0.107946742297
putamen_rvsn_ant_z	PDS_score	2266	0.0030381797	0.885069694363
putamen_rvsn_ant_z	cbcl_scr_syn_internal_r	2264	0.0053204885	0.800253643605
putamen_rvsn_ant_z	hormone_scr_ert_mean_z	2090	0.0045604841	0.834943351642
putamen_rvsn_ant_z	bisbas_ss_basm_rr_z	2259	-0.0058706069	0.780343301389
putamen_rvsn_ant_z	rt_diff_large_neutral_z	2127	-0.0153184883	0.480122360551
putamen_rvsn_ant_z	rt_diff_large_small_z	2136	0.0125189606	0.563080983729

Male participants

x1	x2	N	corr	p
bmi	interview_age	2881	0.0966528288	0.000000201707735181
PDS_score	interview_age	2908	0.1396694054	0.00000000000000386357
PDS_score	bmi	2881	0.1960067350	0.0000000000000000000
cbcl_scr_syn_internal_r	interview_age	2908	0.0098049600	0.59713400918035297559
cbcl_scr_syn_internal_r	bmi	2881	0.0588245618	0.00158436194948330388
cbcl_scr_syn_internal_r	PDS_score	2908	0.0637146967	0.00058627251796972140
hormone_scr_ert_mean_z	interview_age	2701	0.1487784611	0.00000000000000777715
hormone_scr_ert_mean_z	bmi	2674	0.1433937770	0.00000000000000932587
hormone_scr_ert_mean_z	PDS_score	2701	0.1619303875	0.0000000000000000000
hormone_scr_ert_mean_z	cbcl_scr_syn_internal_r	2701	0.0281296607	0.1438664337380368696
bisbas_ss_basm_rr_z	interview_age	2893	-0.0079455258	0.6692445906073074546
bisbas_ss_basm_rr_z	bmi	2867	0.0124564412	0.5049590805262285098
bisbas_ss_basm_rr_z	PDS_score	2893	0.0433002340	0.0198559476401842793
bisbas_ss_basm_rr_z	cbcl_scr_syn_internal_r	2893	-0.0183855853	0.3228811129364856924
bisbas_ss_basm_rr_z	hormone_scr_ert_mean_z	2687	-0.0209221753	0.2783019400062161086
rt_diff_large_neutral_z	interview_age	2323	0.0373689790	0.0717420899860385929
rt_diff_large_neutral_z	bmi	2303	0.0024735094	0.9055618272087564424
rt_diff_large_neutral_z	PDS_score	2323	-0.0249753383	0.2288645369384461059
rt_diff_large_neutral_z	cbcl_scr_syn_internal_r	2323	-0.0159515540	0.4422137844954039387
rt_diff_large_neutral_z	hormone_scr_ert_mean_z	2166	-0.0066765724	0.7561398479829075647
rt_diff_large_neutral_z	bisbas_ss_basm_rr_z	2314	0.0181607093	0.3825520481546205164
rt_diff_large_small_z	interview_age	2324	-0.0060778257	0.7696412534858945164
rt_diff_large_small_z	bmi	2304	0.0087860458	0.6733830943449086170
rt_diff_large_small_z	PDS_score	2324	-0.0059343852	0.7749296259289932820
rt_diff_large_small_z	cbcl_scr_syn_internal_r	2324	-0.0228606158	0.2706299789261157329
rt_diff_large_small_z	hormone_scr_ert_mean_z	2166	-0.0186242256	0.3862971977215365892
rt_diff_large_small_z	bisbas_ss_basm_rr_z	2315	0.0173233510	0.4047794270526257065
rt_diff_large_small_z	rt_diff_large_neutral_z	2304	0.4305686936	0.0000000000000000000
accumbens_rvsn_ant_z	interview_age	2306	-0.0078626409	0.7058974327674916171
accumbens_rvsn_ant_z	bmi	2287	-0.0130347558	0.5332572877746026840
accumbens_rvsn_ant_z	PDS_score	2306	0.0114506167	0.5826020043465063302
accumbens_rvsn_ant_z	cbcl_scr_syn_internal_r	2306	0.0005122498	0.9803857240469753264
accumbens_rvsn_ant_z	hormone_scr_ert_mean_z	2146	0.0091296993	0.6725176876361220745
accumbens_rvsn_ant_z	bisbas_ss_basm_rr_z	2296	-0.0291293699	0.1629205146729537645
accumbens_rvsn_ant_z	rt_diff_large_neutral_z	2136	0.0003463538	0.9872359696310082366
accumbens_rvsn_ant_z	rt_diff_large_small_z	2139	-0.0072396845	0.7378972862804988874
caudate_rvsn_ant_z	interview_age	2317	0.0286907841	0.1674092652793606195
caudate_rvsn_ant_z	bmi	2297	-0.0334198270	0.1093130562505095770
caudate_rvsn_ant_z	PDS_score	2317	0.0079714580	0.7013439043759586155
caudate_rvsn_ant_z	cbcl_scr_syn_internal_r	2317	0.0119896629	0.5640513032392289183
caudate_rvsn_ant_z	hormone_scr_ert_mean_z	2158	-0.0046470795	0.8291804313783937008
caudate_rvsn_ant_z	bisbas_ss_basm_rr_z	2307	-0.0247957766	0.2338462150256703786
caudate_rvsn_ant_z	rt_diff_large_neutral_z	2148	0.0040716162	0.8504093235823517726
caudate_rvsn_ant_z	rt_diff_large_small_z	2149	-0.0327280850	0.1293400518926017817
caudate_rvsn_ant_z	accumbens_rvsn_ant_z	2283	0.5813610694	0.0000000000000000000
putamen_rvsn_ant_z	interview_age	2310	0.0248198000	0.2330892972307805344
putamen_rvsn_ant_z	bmi	2290	-0.0281733407	0.1777430238693766817
putamen_rvsn_ant_z	PDS_score	2310	-0.0070234550	0.7358259587091489084
putamen_rvsn_ant_z	cbcl_scr_syn_internal_r	2310	0.0047408986	0.8198505545923455706
putamen_rvsn_ant_z	hormone_scr_ert_mean_z	2154	-0.0233010527	0.2797198179940560969
putamen_rvsn_ant_z	bisbas_ss_basm_rr_z	2300	-0.0530016017	0.0110134216899611114
putamen_rvsn_ant_z	rt_diff_large_neutral_z	2142	0.0202584658	0.3486834436386410107
putamen_rvsn_ant_z	rt_diff_large_small_z	2144	-0.0215003876	0.3197000986782685405
putamen_rvsn_ant_z	accumbens_rvsn_ant_z	2276	0.5330442739	0.0000000000000000000
putamen_rvsn_ant_z	caudate_rvsn_ant_z	2293	0.7863880858	0.0000000000000000000
[illegible]	[illegible]	[illegible]	[illegible]	[illegible]

6— Compare Outliers to Non-Outliers on Demographic Variables —

Female participants

```
##        interview_age                 bmi race.ethnicity.5level
##         9.995608e-01        1.914984e-01          8.640229e-06
##     household.income           high.educ    demo_race_hispanic
##                  NaN        1.033884e-01          7.700048e-01
```

```
## 
## --------Summary descriptives table by 'is_outlier_any'---------
## 
## ___________________________________________________________
##                          not outlier    outlier   p.overall
##                             N=2511       N=180             
## ¯¯¯¯¯¯¯¯¯¯¯¯¯¯¯¯¯¯¯¯¯¯¯¯¯¯¯¯¯¯¯¯¯¯¯¯¯¯¯¯¯¯¯¯¯¯¯¯¯¯¯¯¯¯¯¯¯¯¯
## interview_age             119 (7.45)  119 (7.50)    1.000  
## bmi                      18.6 (3.90)  19.1 (3.96)   0.115  
## race.ethnicity.5level:                             <0.001  
##     Asian                 72 (2.91%)   2 (1.12%)           
##     Black                380 (15.4%)  55 (30.7%)           
##     Mixed                292 (11.8%)  28 (15.6%)           
##     Other                125 (5.06%)   8 (4.47%)           
##     White                1603 (64.8%) 86 (48.0%)           
## household.income:                                     .    
##     [<5K]                 63 (2.73%)   6 (3.59%)           
##     [>=200K]             264 (11.4%)  14 (8.38%)           
##     [100K-200K]          756 (32.7%)  37 (22.2%)           
##     [12K-16K]             55 (2.38%)   4 (2.40%)           
##     [16K-25K]             99 (4.29%)   9 (5.39%)           
##     [25K-35K]            142 (6.15%)  12 (7.19%)           
##     [35K-50K]            196 (8.48%)  21 (12.6%)           
##     [50K-75K]            309 (13.4%)  26 (15.6%)           
##     [5K-12K]              85 (3.68%)  14 (8.38%)           
##     [75K-100K]           341 (14.8%)  24 (14.4%)           
## high.educ:                                          0.041  
##     < HS Diploma         118 (4.70%)  12 (6.67%)           
##     Bachelor             648 (25.8%)  34 (18.9%)           
##     HS Diploma/GED       219 (8.73%)  21 (11.7%)           
##     Post Graduate Degree 907 (36.1%)  57 (31.7%)           
##     Some College         617 (24.6%)  56 (31.1%)           
## demo_race_hispanic:                                 0.616  
##     0                    1996 (80.4%) 139 (78.5%)          
##     1                    487 (19.6%)  38 (21.5%)           
## ¯¯¯¯¯¯¯¯¯¯¯¯¯¯¯¯¯¯¯¯¯¯¯¯¯¯¯¯¯¯¯¯¯¯¯¯¯¯¯¯¯¯¯¯¯¯¯¯¯¯¯¯¯¯¯¯¯¯¯
```

Male participants

```
##        interview_age                 bmi race.ethnicity.5level
##         2.168334e-01        1.209421e-02          2.105984e-05
##     household.income           high.educ    demo_race_hispanic
```

```
##                   NaN          8.937779e-02          2.196325e-01

##
## --------Summary descriptives table by 'is_outlier_any'---------
##
## ______________________________________________________________
##                          not outlier    outlier   p.overall
##                             N=2657       N=251
## ¯¯¯¯¯¯¯¯¯¯¯¯¯¯¯¯¯¯¯¯¯¯¯¯¯¯¯¯¯¯¯¯¯¯¯¯¯¯¯¯¯¯¯¯¯¯¯¯¯¯¯¯¯¯¯¯¯¯¯¯¯¯
## interview_age             119 (7.55)  119 (7.27)    0.173
## bmi                      18.5 (3.83)  19.2 (3.85)   0.005
## race.ethnicity.5level:                             <0.001
##     Asian                 57 (2.17%)   4 (1.65%)
##     Black                320 (12.2%)  57 (23.5%)
##     Mixed                311 (11.8%)  36 (14.8%)
##     Other                144 (5.49%)   7 (2.88%)
##     White                1793 (68.3%) 139 (57.2%)
## household.income:                                     .
##     [<5K]                 67 (2.76%)  17 (7.52%)
##     [>=200K]             299 (12.3%)  15 (6.64%)
##     [100K-200K]          768 (31.6%)  68 (30.1%)
##     [12K-16K]             49 (2.02%)   9 (3.98%)
##     [16K-25K]            111 (4.57%)  16 (7.08%)
##     [25K-35K]            131 (5.40%)   9 (3.98%)
##     [35K-50K]            207 (8.53%)  22 (9.73%)
##     [50K-75K]            345 (14.2%)  29 (12.8%)
##     [5K-12K]              85 (3.50%)  10 (4.42%)
##     [75K-100K]           366 (15.1%)  31 (13.7%)
## high.educ:                                          0.054
##     < HS Diploma         106 (3.99%)  13 (5.22%)
##     Bachelor             684 (25.8%)  58 (23.3%)
##     HS Diploma/GED       216 (8.14%)  28 (11.2%)
##     Post Graduate Degree 973 (36.7%)  74 (29.7%)
##     Some College         675 (25.4%)  76 (30.5%)
## demo_race_hispanic:                                 0.220
##     0                    2086 (79.6%) 187 (76.0%)
##     1                    536 (20.4%)  59 (24.0%)
## ¯¯¯¯¯¯¯¯¯¯¯¯¯¯¯¯¯¯¯¯¯¯¯¯¯¯¯¯¯¯¯¯¯¯¯¯¯¯¯¯¯¯¯¯¯¯¯¯¯¯¯¯¯¯¯¯¯¯¯¯¯¯
```
