# Supplementary material for: Reward sensitivity and internalizing symptoms during the transition to puberty: An examination of 9-and 10-year-olds in the ABCD Study
Source: Dev Cogn Neurosci. 2022 Oct 31;58:101172. doi: 10.1016/j.dcn.2022.101172 (PMC9649995; doi:10.1016/j.dcn.2022.101172)
Supplement: Supplementary file 3 — Supplementary material [file mmc3.pdf]

Supplement C

Reward sensitivity and internalizing symptoms during the transition to puberty: An examination of 9- and 10-year-olds in the ABCD Study.

Contents

Sensitivity Analysis Results for Sample 1 **8**

1—Internalizing~Puberty— **8**

1.1 Model: CBCL internalizing factor ~ PDS 8
Female participants 8
Male participants 9
1.2 Model: CBCL Anxious-Depressed ~ PDS 10
Female participants 10
Male participants 11
1.3 Model: CBCL Withdrawn-Depressed ~ PDS 13
Female participants 13
Male participants 14
1.4 Model: CBCL Depressed DSM-5 ~ PDS 15
Female participants 15
Male participants 16
1.5 Model: CBCL internalizing factor ~ Pubertal category 18
Female participants 18
Male participants 19
1.6 Model: CBCL Anxious-Depressed ~ Pubertal category 20
Female participants 20
Male participants 21
1.7 Model: CBCL Withdrawn-Depressed ~ Pubertal category 23
Female participants 23
Male participants 24
1.8 Model: CBCL Depressed DSM-5 ~ Pubertal category 25
Female participants 25
Male participants 27

1.9 Model: CBCL internalizing factor ~ Testosterone 28
Female participants 28
Male participants 29
1.10 Model: CBCL Anxious-Depressed ~ Testosterone 31
Female participants 31
Male participants 32
1.11 Model: CBCL Withdrawn-Depressed ~ Testosterone 33
Female participants 33
Male participants 35
1.12 Model: CBCL Depressed DSM-5 ~ Testosterone 36
Female participants 36
Male participants 37
1.13 Model: CBCL internalizing factor ~ Testosterone + PDS 38
Female participants 38
Male participants 40
1.14 Model: CBCL internalizing factor ~ Testosterone + Pubertal category 41
Female participants 41
Male participants 42
1.15 Model: CBCL Anxious-Depressed ~ Testosterone + PDS 44
Female participants 44
Male participants 45
1.16 Model: CBCL Anxious-Depressed ~ Testosterone + Pubertal category 47
Female participants 47
Male participants 48
1.17 Model: CBCL Withdrawn-Depressed ~ Testosterone + PDS 49
Female participants 49
Male participants 51
1.18 Model: CBCL Withdrawn-Depressed ~ Testosterone + Pubertal category 52
Female participants 52
Male participants 53
1.19 Model: CBCL Depressed DSM-5 ~ Testosterone + PDS 55
Female participants 55
Male participants 56
1.20 Model: CBCL Depressed DSM-5 ~ Testosterone + Pubertal category 58
Female participants 58
Male participants 59

2—Reward~Puberty— **61**
2.1 Model: BIS-BAS-RR ~ PDS . . . 61
Female participants . . . 61
Male participants . . . 61
2.2 Model : Reaction Time ~ PDS . . . 62
Female participants . . . 62
Male participants . . . 63
2.3 Model: Caudate Anticipation ~ PDS . . . 64
Female participants . . . 64
Male participants . . . 64
2.4 Model B: Putamen Anticipation ~ PDS . . . 65
Female participants . . . 65
Male participants . . . 65
2.5 Model: Accumbens Anticipation ~ PDS . . . 66
Female participants . . . 66
Male participants . . . 66
2.6 Model: Caudate Feedback ~ PDS . . . 67
Female participants . . . 67
Male participants . . . 67
2.7 Model: Putamen Feedback ~ PDS . . . 68
Female participants . . . 68
Male participants . . . 69
2.8 Model: Accumbens Feedback ~ PDS . . . 69
Female participants . . . 69
Male participants . . . 70
2.9 Model: OFC Anticipation ~ PDS . . . 70
Female participants . . . 70
Male participants . . . 71
2.10 Model: OFC Feedback ~ PDS . . . 72
Female participants . . . 72
Male participants . . . 73
2.11 Model: Caudate Anticipation ~ Testosterone . . . 74
Female participants . . . 74
Male participants . . . 75
2.12 Model B: Putamen Anticipation ~ Testosterone . . . 75
Female participants . . . 75

Male participants 76
2.13 Model: Accumbens Anticipation ~ Testosterone 77
Female participants 77
Male participants 78
2.14 Model: Caudate Feedback ~ Testosterone 78
Female participants 78
Male participants 79
2.15 Model: Putamen Feedback ~ Testosterone 80
Female participants 80
Male participants 80
2.16 Model: Accumbens Feedback ~ Testosterone 81
Female participants 81
Male participants 82
2.17 Model: OFC Anticipation ~ Testosterone 82
Female participants 82
Male participants 84
2.18 Model: OFC Feedback ~ Testosterone 85
Female participants 85
Male participants 86
2.19 Model: MID Reaction Time ~ Testosterone 88
Female participants 88
Male participants 89
2.20 Model: BIS-BAS-RR ~ Testosterone 90
Female participants 90
Male participants 90

3—Internalizing~Reward— **92**
3.1 Model: CBCL internalizing factor ~ Nucleus Accumbens activity (anticipation stage) 92
Female participants 92
Male participants 92
3.2 Model: CBCL internalizing factor ~ Caudate activity (anticipation stage) 93
Female participants 93
Male participants 93
3.3 Model: CBCL internalizing factor ~ Putamen activity (anticipation stage) 94
Female participants 94
Male participants 94
3.4 Model: CBCL internalizing factor ~ Accumbens activity (feedback stage) 95

Female participants 95
Male participants 95
3.5 Model: CBCL internalizing factor ~ Caudate activity (feedback stage) 96
Female participants 96
Male participants 96
3.6 Model: CBCL internalizing factor ~ Putamen activity (feedback stage) 97
Female participants 97
Male participants 97
3.7 Model: CBCL internalizing factor ~ OFC activity (anticipation stage) 98
Female participants 98
Male participants 99
3.8 Model: CBCL internalizing factor ~ OFC activity (feedback stage) 100
Female participants 100
Male participants 101
3.9 Model: CBCL internalizing factor ~ BIS-BAS-RR 102
Female participants 102
Male participants 102
3.10 Model: CBCL internalizing factor ~ MID Reaction Time 103
Female participants 103
Male participants 104

4—Internalizing~Puberty x Reward— 105

4.1 Model: CBCL internalizing factor ~ PDS x Accumbens activity (anticipation stage) 105
Female participants 105
Male participants 106
4.2 Model: CBCL internalizing factor ~ PDS x Caudate activity (anticipation stage) 107
Female participants 107
Male participants 109
4.3 Model: CBCL internalizing factor ~ PDS x Putamen activity (anticipation stage) 110
Female participants 110
Male participants 111
4.4 Model: CBCL internalizing factor ~ PDS x Lateral OFC activity (anticipation stage) 113
Female participants 113
Male participants 114
4.5 Model: CBCL internalizing factor ~ PDS x Medial OFC activity (anticipation stage) 115
Female participants 115
Male participants 117

4.6 Model: CBCL internalizing factor ~ PDS x Accumbens activity (feedback) 118
Female participants 118
Male participants 119
4.7 Model: CBCL internalizing factor ~ PDS x Caudate activity (feedback) 121
Female participants 121
Male participants 122
4.8 Model: CBCL internalizing factor ~ PDS x Putamen activity (feedback) 124
Female participants 124
Male participants 125
4.9 Model: CBCL internalizing factor ~ PDS x Lateral OFC activity (feedback stage) 127
Female participants 127
Male participants 128
4.10 Model: CBCL internalizing factor ~ PDS x Medial OFC activity (feedback stage) 129
Female participants 129
Male participants 131
4.11 Model: CBCL internalizing factor ~ PDS x BIS-BAS 132
Female participants 132
Male participants 133
4.12 Model: CBCL internalizing factor ~ PDS x MID reaction time (large reward vs. neutral) . . . 135
Female participants 135
Male participants 136
4.13 Model: CBCL internalizing factor ~ PDS x MID reaction time (large vs. small reward) 137
Female participants 137
Male participants 139
4.14 Model: CBCL internalizing factor ~ Testosterone x Accumbens activity (anticipation stage) + PDS 140
Female participants 140
Male participants 142
4.15 Model: CBCL internalizing factor ~ Testosterone x Caudate activity (anticipation stage) + PDS144
Female participants 144
Male participants 146
4.16 Model: CBCL internalizing factor ~ Testosterone x Putamen activity (anticipation stage) + PDS 148
Female participants 148
Male participants 150
4.17 Model: CBCL internalizing factor ~ Testosterone x Accumbens activity (feedback stage) + PDS152
Female participants 152

Male participants 154
4.18 Model: CBCL internalizing factor ~ Testosterone x Caudate activity (Feedback stage) + PDS 156
Female participants 156
Male participants 158
4.19 Model: CBCL internalizing factor ~ Testosterone x Putamen activity (Feedback stage) + PDS 160
Female participants 160
Male participants 162
4.20 Model: CBCL internalizing factor ~ Testosterone x Lateral OFC activity (anticipation stage) + PDS 165
Female participants 165
Male participants 166
4.21 Model: CBCL internalizing factor ~ Testosterone x Medial OFC activity (anticipation stage) + PDS 168
Female participants 168
Male participants 169
4.22 Model: CBCL internalizing factor ~ Testosterone x Lateral OFC activity (feedback stage) + PDS 171
Female participants 171
Male participants 173
4.23 Model: CBCL internalizing factor ~ Testosterone x Medial OFC activity (feedback stage) + PDS 175
Female participants 175
Male participants 177
4.24 Model: CBCL internalizing factor ~ Testosterone x BIS-BAS RR + PDS 179
Female participants 179
Male participants 181
4.25 Model: CBCL internalizing factor ~ Testosterone x MID Reaction Time + PDS (large reward vs. neutral) 183
Female participants 183
Male participants 185
4.26 Model: CBCL internalizing factor ~ Testosterone x MID Reaction Time + PDS (large vs. small reward) 187
Female participants 187
Male participants 188

Sensitivity Analysis Results for Sample 1

1—Internalizing~Puberty—

1.1 Model: CBCL internalizing factor ~ PDS

Female participants

```
##
## Family: gaussian
## Link function: identity
##
## Formula:
## cbcl_scr_syn_internal_r ~ PDS_score + race.ethnicity.5level +
##     interview_age + bmi + household.income + high.educ + demo_race_hispanic
##
## Parametric coefficients:
##                              Estimate Std. Error t value Pr(>|t|)
## (Intercept)                  4.459444   2.122551   2.101 0.035747 *
## PDS_score                    0.487526   0.168041   2.901 0.003751 **
## race.ethnicity.5levelBlack  -0.817758   0.867140  -0.943 0.345750
## race.ethnicity.5levelMixed   1.181182   0.847964   1.393 0.163760
## race.ethnicity.5levelOther   1.754101   0.961984   1.823 0.068364 .
## race.ethnicity.5levelWhite   1.265869   0.799230   1.584 0.113357
## interview_age               -0.009392   0.015097  -0.622 0.533939
## bmi                          0.064337   0.029572   2.176 0.029684 *
## household.income[>=200K]    -2.776896   0.770842  -3.602 0.000322 ***
## household.income[100K-200K] -2.207448   0.716007  -3.083 0.002073 **
## household.income[12K-16K]   -0.252511   0.955819  -0.264 0.791662
## household.income[16K-25K]    0.113765   0.797340   0.143 0.886554
## household.income[25K-35K]   -0.983116   0.753977  -1.304 0.192390
## household.income[35K-50K]   -1.167663   0.725683  -1.609 0.107736
## household.income[50K-75K]   -1.162286   0.721034  -1.612 0.107100
## household.income[5K-12K]    -0.641181   0.841959  -0.762 0.446412
## household.income[75K-100K]  -1.599332   0.725749  -2.204 0.027640 *
## high.educBachelor            0.190727   0.730984   0.261 0.794178
## high.educHS Diploma/GED     -0.501619   0.728623  -0.688 0.491237
## high.educPost Graduate Degree  0.367506   0.737471   0.498 0.618295
## high.educSome College        0.585969   0.687515   0.852 0.394133
## demo_race_hispanic1         -0.173681   0.342093  -0.508 0.611709
## ---
## Signif. codes:  0 '***' 0.001 '**' 0.01 '*' 0.05 '.' 0.1 ' ' 1
##
##
## R-sq.(adj) =   0.03
## lmer.REML =  14919  Scale est. = 13.539    n = 2420
```

```
##                                    stdcoef      stdse
## X(Intercept)                   0.000000000 0.00000000
## XPDS_score                     0.065395049 0.02254039
## Xrace.ethnicity.5levelBlack   -0.052083759 0.05522897
## Xrace.ethnicity.5levelMixed    0.072128844 0.05178091
## Xrace.ethnicity.5levelOther    0.068283490 0.03744803
```

```
## Xrace.ethnicity.5levelWhite     0.109687226 0.06925309
## Xinterview_age                 -0.012946301 0.02081082
## Xbmi                            0.047853725 0.02199586
## Xhousehold.income[>=200K]      -0.170748606 0.04739831
## Xhousehold.income[100K-200K]   -0.185936764 0.06031036
## Xhousehold.income[12K-16K]     -0.006790406 0.02570346
## Xhousehold.income[16K-25K]      0.004297033 0.03011635
## Xhousehold.income[25K-35K]     -0.043146177 0.03308989
## Xhousehold.income[35K-50K]     -0.060525130 0.03761533
## Xhousehold.income[50K-75K]     -0.072910362 0.04523055
## Xhousehold.income[5K-12K]      -0.021189349 0.02782451
## Xhousehold.income[75K-100K]    -0.105518924 0.04788265
## Xhigh.educBachelor              0.015592193 0.05975892
## Xhigh.educHS Diploma/GED       -0.024670574 0.03583504
## Xhigh.educPost Graduate Degree  0.032474269 0.06516583
## Xhigh.educSome College          0.046848632 0.05496729
## Xdemo_race_hispanic1           -0.012394386 0.02441271
```

Male participants

```
##
## Family: gaussian
## Link function: identity
##
## Formula:
## cbcl_scr_syn_internal_r ~ PDS_score + race.ethnicity.5level +
##     interview_age + bmi + household.income + high.educ + demo_race_hispanic
##
## Parametric coefficients:
##                                 Estimate Std. Error t value Pr(>|t|)
## (Intercept)                     3.785070   2.014579   1.879 0.060378 .
## PDS_score                       0.737717   0.207273   3.559 0.000379 ***
## race.ethnicity.5levelBlack      0.489925   0.774056   0.633 0.526834
## race.ethnicity.5levelMixed      1.948748   0.755376   2.580 0.009939 **
## race.ethnicity.5levelOther      1.531302   0.892338   1.716 0.086271 .
## race.ethnicity.5levelWhite      1.654851   0.705293   2.346 0.019034 *
## interview_age                  -0.009152   0.014150  -0.647 0.517833
## bmi                             0.064160   0.030113   2.131 0.033213 *
## household.income[>=200K]       -2.627871   0.751933  -3.495 0.000482 ***
## household.income[100K-200K]    -2.459072   0.695956  -3.533 0.000417 ***
## household.income[12K-16K]      -1.074351   0.929624  -1.156 0.247917
## household.income[16K-25K]      -0.431428   0.766687  -0.563 0.573675
## household.income[25K-35K]      -1.282458   0.753595  -1.702 0.088915 .
## household.income[35K-50K]      -0.950600   0.723559  -1.314 0.189035
## household.income[50K-75K]      -1.690631   0.691904  -2.443 0.014614 *
## household.income[5K-12K]        0.536524   0.817516   0.656 0.511698
## household.income[75K-100K]     -2.126198   0.707958  -3.003 0.002696 **
## high.educBachelor               0.559237   0.695096   0.805 0.421155
## high.educHS Diploma/GED        -0.368329   0.703659  -0.523 0.600707
## high.educPost Graduate Degree   0.478599   0.706571   0.677 0.498241
## high.educSome College           0.701271   0.661997   1.059 0.289549
## demo_race_hispanic1            -0.399618   0.321266  -1.244 0.213654
## ---
```

```
## Signif. codes:  0 '***' 0.001 '**' 0.01 '*' 0.05 '.' 0.1 ' ' 1
##
##
## R-sq.(adj) =  0.0245
## lmer.REML =  16171  Scale est. = 14.634    n = 2627
```

```
##                                    stdcoef      stdse
## X(Intercept)                    0.00000000 0.00000000
## XPDS_score                      0.07362395 0.02068578
## Xrace.ethnicity.5levelBlack     0.03142509 0.04965004
## Xrace.ethnicity.5levelMixed     0.11876720 0.04603671
## Xrace.ethnicity.5levelOther     0.05707653 0.03326028
## Xrace.ethnicity.5levelWhite     0.14420119 0.06145814
## Xinterview_age                 -0.01278526 0.01976762
## Xbmi                            0.04404831 0.02067381
## Xhousehold.income[>=200K]      -0.15842242 0.04533065
## Xhousehold.income[100K-200K]   -0.21388569 0.06053301
## Xhousehold.income[12K-16K]     -0.02862343 0.02476754
## Xhousehold.income[16K-25K]     -0.01734836 0.03082959
## Xhousehold.income[25K-35K]     -0.05520083 0.03243697
## Xhousehold.income[35K-50K]     -0.04786936 0.03643626
## Xhousehold.income[50K-75K]     -0.11066262 0.04528955
## Xhousehold.income[5K-12K]       0.01776640 0.02707115
## Xhousehold.income[75K-100K]    -0.13810005 0.04598306
## Xhigh.educBachelor              0.04683111 0.05820808
## Xhigh.educHS Diploma/GED       -0.01846662 0.03527881
## Xhigh.educPost Graduate Degree  0.04244276 0.06265952
## Xhigh.educSome College          0.05696582 0.05377547
## Xdemo_race_hispanic1           -0.02910291 0.02339678
```

1.2 Model: CBCL Anxious-Depressed ~ PDS

Female participants

```
##
## Family: gaussian
## Link function: identity
##
## Formula:
## cbcl_scr_syn_anxdep_r ~ PDS_score + race.ethnicity.5level + interview_age +
##     bmi + household.income + high.educ + demo_race_hispanic
##
## Parametric coefficients:
##                               Estimate Std. Error t value Pr(>|t|)
## (Intercept)                   2.111715   1.213530   1.740 0.081962 .
## PDS_score                     0.174618   0.096065   1.818 0.069235 .
## race.ethnicity.5levelBlack   -0.236175   0.493301  -0.479 0.632150
## race.ethnicity.5levelMixed    0.619583   0.482507   1.284 0.199234
## race.ethnicity.5levelOther    0.706582   0.547938   1.290 0.197339
## race.ethnicity.5levelWhite    0.760516   0.454699   1.673 0.094542 .
## interview_age                -0.003032   0.008651  -0.350 0.726007
## bmi                           0.011332   0.016912   0.670 0.502898
## household.income[>=200K]     -1.483952   0.438686  -3.383 0.000729 ***
```

```
## household.income[100K-200K]   -0.968914   0.407697  -2.377 0.017553 *
## household.income[12K-16K]     -0.177325   0.544682  -0.326 0.744788
## household.income[16K-25K]      0.088222   0.454470   0.194 0.846099
## household.income[25K-35K]     -0.420475   0.429723  -0.978 0.327936
## household.income[35K-50K]     -0.413257   0.413282  -1.000 0.317441
## household.income[50K-75K]     -0.422752   0.410589  -1.030 0.303290
## household.income[5K-12K]      -0.365691   0.479251  -0.763 0.445511
## household.income[75K-100K]    -0.701845   0.413218  -1.698 0.089545 .
## high.educBachelor              0.292754   0.416535   0.703 0.482229
## high.educHS Diploma/GED       -0.186536   0.415689  -0.449 0.653660
## high.educPost Graduate Degree  0.594945   0.420212   1.416 0.156958
## high.educSome College          0.495422   0.391857   1.264 0.206248
## demo_race_hispanic1           -0.066710   0.194367  -0.343 0.731464
## ---
## Signif. codes:  0 '***' 0.001 '**' 0.01 '*' 0.05 '.' 0.1 ' ' 1
##
##
## R-sq.(adj) =  0.0178
## lmer.REML =  12243  Scale est. = 5.202     n = 2420
```

```
##                                     stdcoef      stdse
## X(Intercept)                    0.000000000 0.00000000
## XPDS_score                      0.041276109 0.02270786
## Xrace.ethnicity.5levelBlack    -0.026507840 0.05536719
## Xrace.ethnicity.5levelMixed     0.066673610 0.05192281
## Xrace.ethnicity.5levelOther     0.048471444 0.03758851
## Xrace.ethnicity.5levelWhite     0.116128351 0.06943102
## Xinterview_age                 -0.007365033 0.02101399
## Xbmi                            0.014852735 0.02216702
## Xhousehold.income[>=200K]      -0.160797681 0.04753502
## Xhousehold.income[100K-200K]   -0.143821031 0.06051667
## Xhousehold.income[12K-16K]     -0.008403272 0.02581199
## Xhousehold.income[16K-25K]      0.005872134 0.03025009
## Xhousehold.income[25K-35K]     -0.032519204 0.03323442
## Xhousehold.income[35K-50K]     -0.037748564 0.03775089
## Xhousehold.income[50K-75K]     -0.046733089 0.04538850
## Xhousehold.income[5K-12K]      -0.021296728 0.02791012
## Xhousehold.income[75K-100K]    -0.081601002 0.04804330
## Xhigh.educBachelor              0.042175503 0.06000796
## Xhigh.educHS Diploma/GED       -0.016167058 0.03602767
## Xhigh.educPost Graduate Degree  0.092643328 0.06543441
## Xhigh.educSome College          0.069800702 0.05520925
## Xdemo_race_hispanic1           -0.008389327 0.02444313
```

Male participants

```
##
## Family: gaussian
## Link function: identity
##
## Formula:
## cbcl_scr_syn_anxdep_r ~ PDS_score + race.ethnicity.5level + interview_age +
##     bmi + household.income + high.educ + demo_race_hispanic
```

```
##
## Parametric coefficients:
##                              Estimate Std. Error t value Pr(>|t|)
## (Intercept)                   1.863189   1.135128   1.641 0.100837
## PDS_score                     0.427723   0.116663   3.666 0.000251 ***
## race.ethnicity.5levelBlack    0.443321   0.435410   1.018 0.308692
## race.ethnicity.5levelMixed    1.182962   0.425183   2.782 0.005437 **
## race.ethnicity.5levelOther    1.067078   0.501148   2.129 0.033326 *
## race.ethnicity.5levelWhite    1.169018   0.397179   2.943 0.003276 **
## interview_age                -0.007419   0.007980  -0.930 0.352613
## bmi                           0.016957   0.016941   1.001 0.316969
## household.income[>=200K]     -1.012245   0.420631  -2.406 0.016176 *
## household.income[100K-200K]  -0.917634   0.389528  -2.356 0.018559 *
## household.income[12K-16K]    -0.350618   0.521847  -0.672 0.501720
## household.income[16K-25K]     0.038471   0.428707   0.090 0.928502
## household.income[25K-35K]   -0.285208   0.421850  -0.676 0.499043
## household.income[35K-50K]   -0.225680   0.405042  -0.557 0.577454
## household.income[50K-75K]   -0.696180   0.387156  -1.798 0.072263 .
## household.income[5K-12K]      0.099463   0.458120   0.217 0.828140
## household.income[75K-100K]  -0.771405   0.396188  -1.947 0.051634 .
## high.educBachelor             0.321168   0.388761   0.826 0.408804
## high.educHS Diploma/GED      -0.298566   0.393624  -0.759 0.448217
## high.educPost Graduate Degree  0.314017   0.395162   0.795 0.426887
## high.educSome College         0.260739   0.370376   0.704 0.481505
## demo_race_hispanic1          -0.099263   0.179938  -0.552 0.581237
## ---
## Signif. codes:  0 '***' 0.001 '**' 0.01 '*' 0.05 '.' 0.1 ' ' 1
##
##
## R-sq.(adj) =  0.0134
## lmer.REML =  13187  Scale est. = 6.0677    n = 2627
```

```
##                                    stdcoef      stdse
## X(Intercept)                    0.000000000 0.00000000
## XPDS_score                      0.076457169 0.02085395
## Xrace.ethnicity.5levelBlack     0.050932175 0.05002329
## Xrace.ethnicity.5levelMixed     0.129133405 0.04641342
## Xrace.ethnicity.5levelOther     0.071239296 0.03345719
## Xrace.ethnicity.5levelWhite     0.182455907 0.06199016
## Xinterview_age                 -0.018564542 0.01996832
## Xbmi                            0.020851275 0.02083255
## Xhousehold.income[>=200K]      -0.109301273 0.04541930
## Xhousehold.income[100K-200K]   -0.142957445 0.06068424
## Xhousehold.income[12K-16K]     -0.016731574 0.02490266
## Xhousehold.income[16K-25K]      0.002770862 0.03087712
## Xhousehold.income[25K-35K]     -0.021988306 0.03252272
## Xhousehold.income[35K-50K]     -0.020355401 0.03653311
## Xhousehold.income[50K-75K]     -0.081620681 0.04539053
## Xhousehold.income[5K-12K]       0.005899250 0.02717169
## Xhousehold.income[75K-100K]    -0.089742745 0.04609121
## Xhigh.educBachelor              0.048172411 0.05831064
## Xhigh.educHS Diploma/GED       -0.026811351 0.03534763
## Xhigh.educPost Graduate Degree  0.049878319 0.06276729
## Xhigh.educSome College          0.037936851 0.05388874
```

```
## Xdemo_race_hispanic1          -0.012948032 0.02347154
```

1.3 Model: CBCL Withdrawn-Depressed ~ PDS

Female participants

```
##
## Family: gaussian
## Link function: identity
##
## Formula:
## cbcl_scr_syn_withdep_r ~ PDS_score + race.ethnicity.5level +
##     interview_age + bmi + household.income + high.educ + demo_race_hispanic
##
## Parametric coefficients:
##                                 Estimate Std. Error t value Pr(>|t|)
## (Intercept)                     1.095487   0.599256   1.828 0.067662 .
## PDS_score                       0.158433   0.047515   3.334 0.000868 ***
## race.ethnicity.5levelBlack     -0.129429   0.242278  -0.534 0.593242
## race.ethnicity.5levelMixed      0.211498   0.237210   0.892 0.372694
## race.ethnicity.5levelOther      0.388096   0.269925   1.438 0.150624
## race.ethnicity.5levelWhite      0.239030   0.223178   1.071 0.284263
## interview_age                  -0.004195   0.004282  -0.980 0.327328
## bmi                             0.020412   0.008365   2.440 0.014749 *
## household.income[>=200K]       -0.767334   0.216073  -3.551 0.000391 ***
## household.income[100K-200K]    -0.691186   0.201079  -3.437 0.000597 ***
## household.income[12K-16K]      -0.227585   0.269043  -0.846 0.397689
## household.income[16K-25K]      -0.140194   0.224589  -0.624 0.532539
## household.income[25K-35K]      -0.305108   0.212257  -1.437 0.150720
## household.income[35K-50K]     -0.491138   0.204030  -2.407 0.016151 *
## household.income[50K-75K]     -0.459631   0.202573  -2.269 0.023359 *
## household.income[5K-12K]        0.004470   0.236552   0.019 0.984926
## household.income[75K-100K]     -0.572401   0.203806  -2.809 0.005017 **
## high.educBachelor              -0.016307   0.205542  -0.079 0.936772
## high.educHS Diploma/GED        -0.090991   0.205417  -0.443 0.657836
## high.educPost Graduate Degree -0.044833   0.207345  -0.216 0.828833
## high.educSome College           0.086132   0.193435   0.445 0.656158
## demo_race_hispanic1             0.014752   0.094543   0.156 0.876016
## ---
## Signif. codes:  0 '***' 0.001 '**' 0.01 '*' 0.05 '.' 0.1 ' ' 1
##
##
## R-sq.(adj) =  0.0307
## lmer.REML = 8873.1  Scale est. = 1.4438    n = 2420
```

```
##                                      stdcoef      stdse
## X(Intercept)                    0.0000000000 0.00000000
## XPDS_score                      0.0756833576 0.02269785
## Xrace.ethnicity.5levelBlack    -0.0293572599 0.05495399
## Xrace.ethnicity.5levelMixed     0.0459943789 0.05158605
## Xrace.ethnicity.5levelOther     0.0538030834 0.03742063
## Xrace.ethnicity.5levelWhite     0.0737610269 0.06886914
## Xinterview_age                 -0.0205922477 0.02101870
```

```
## Xbmi                            0.0540694657 0.02215743
## Xhousehold.income[>=200K]      -0.1680306472 0.04731565
## Xhousehold.income[100K-200K]   -0.2073368677 0.06031816
## Xhousehold.income[12K-16K]     -0.0217954747 0.02576581
## Xhousehold.income[16K-25K]     -0.0188580061 0.03021024
## Xhousehold.income[25K-35K]     -0.0476868372 0.03317457
## Xhousehold.income[35K-50K]     -0.0906627303 0.03766328
## Xhousehold.income[50K-75K]     -0.1026816046 0.04525472
## Xhousehold.income[5K-12K]       0.0005260589 0.02784006
## Xhousehold.income[75K-100K]    -0.1344927651 0.04788685
## Xhigh.educBachelor             -0.0047475543 0.05984156
## Xhigh.educHS Diploma/GED       -0.0159371609 0.03597894
## Xhigh.educPost Graduate Degree -0.0141083050 0.06524931
## Xhigh.educSome College          0.0245242332 0.05507611
## Xdemo_race_hispanic1            0.0037491911 0.02402743
```

Male participants

```
##
## Family: gaussian
## Link function: identity
##
## Formula:
## cbcl_scr_syn_withdep_r ~ PDS_score + race.ethnicity.5level +
##     interview_age + bmi + household.income + high.educ + demo_race_hispanic
##
## Parametric coefficients:
##                                 Estimate Std. Error t value Pr(>|t|)
## (Intercept)                     1.083256   0.631366   1.716 0.086330 .
## PDS_score                       0.135831   0.065270   2.081 0.037526 *
## race.ethnicity.5levelBlack      0.103290   0.242292   0.426 0.669921
## race.ethnicity.5levelMixed      0.450675   0.237528   1.897 0.057892 .
## race.ethnicity.5levelOther      0.221089   0.279925   0.790 0.429708
## race.ethnicity.5levelWhite      0.334806   0.220703   1.517 0.129388
## interview_age                  -0.002392   0.004452  -0.537 0.591140
## bmi                             0.020795   0.009495   2.190 0.028613 *
## household.income[>=200K]       -0.900057   0.234748  -3.834 0.000129 ***
## household.income[100K-200K]    -0.859043   0.218132  -3.938 8.43e-05 ***
## household.income[12K-16K]      -0.349539   0.292628  -1.194 0.232398
## household.income[16K-25K]      -0.054727   0.240257  -0.228 0.819831
## household.income[25K-35K]      -0.387631   0.236799  -1.637 0.101759
## household.income[35K-50K]      -0.340271   0.227278  -1.497 0.134473
## household.income[50K-75K]      -0.477627   0.216854  -2.203 0.027715 *
## household.income[5K-12K]        0.320458   0.256846   1.248 0.212266
## household.income[75K-100K]     -0.730978   0.222071  -3.292 0.001009 **
## high.educBachelor               0.039784   0.216751   0.184 0.854384
## high.educHS Diploma/GED        -0.009461   0.219975  -0.043 0.965696
## high.educPost Graduate Degree   0.009379   0.220581   0.043 0.966089
## high.educSome College           0.126328   0.206881   0.611 0.541496
## demo_race_hispanic1            -0.291833   0.095693  -3.050 0.002314 **
## ---
## Signif. codes:  0 '***' 0.001 '**' 0.01 '*' 0.05 '.' 0.1 ' ' 1
##
```

```
##
## R-sq.(adj) =  0.0347
## lmer.REML =  10180  Scale est. = 2.0547    n = 2627
```

```
##                                     stdcoef      stdse
## X(Intercept)                     0.000000000 0.00000000
## XPDS_score                       0.042840148 0.02058573
## Xrace.ethnicity.5levelBlack      0.020937688 0.04911442
## Xrace.ethnicity.5levelMixed      0.086801575 0.04574874
## Xrace.ethnicity.5levelOther      0.026042776 0.03297327
## Xrace.ethnicity.5levelWhite      0.092199049 0.06077723
## Xinterview_age                  -0.010559957 0.01965551
## Xbmi                             0.045118044 0.02060184
## Xhousehold.income[>=200K]       -0.171476833 0.04472368
## Xhousehold.income[100K-200K]    -0.236128304 0.05995887
## Xhousehold.income[12K-16K]      -0.029430249 0.02463847
## Xhousehold.income[16K-25K]      -0.006954652 0.03053155
## Xhousehold.income[25K-35K]      -0.052728367 0.03221112
## Xhousehold.income[35K-50K]      -0.054151160 0.03616930
## Xhousehold.income[50K-75K]      -0.098801493 0.04485824
## Xhousehold.income[5K-12K]        0.033535418 0.02687855
## Xhousehold.income[75K-100K]     -0.150043507 0.04558329
## Xhigh.educBachelor               0.010528528 0.05736167
## Xhigh.educHS Diploma/GED        -0.001499081 0.03485361
## Xhigh.educPost Graduate Degree   0.002628400 0.06181904
## Xhigh.educSome College           0.032430389 0.05310954
## Xdemo_race_hispanic1            -0.067165915 0.02202397
```

1.4 Model: CBCL Depressed DSM-5 ~ PDS

Female participants

```
##
## Family: gaussian
## Link function: identity
##
## Formula:
## cbcl_scr_dsm5_depress_r ~ PDS_score + race.ethnicity.5level +
##     interview_age + bmi + household.income + high.educ + demo_race_hispanic
##
## Parametric coefficients:
##                               Estimate Std. Error t value Pr(>|t|)
## (Intercept)                   0.951849   0.712964   1.335  0.18198
## PDS_score                     0.163950   0.056585   2.897  0.00380 **
## race.ethnicity.5levelBlack   -0.063231   0.289565  -0.218  0.82716
## race.ethnicity.5levelMixed    0.475312   0.283540   1.676  0.09380 .
## race.ethnicity.5levelOther    0.682351   0.322480   2.116  0.03445 *
## race.ethnicity.5levelWhite    0.553199   0.266594   2.075  0.03809 *
## interview_age                -0.005394   0.005084  -1.061  0.28883
## bmi                           0.020409   0.009960   2.049  0.04056 *
## household.income[>=200K]     -0.824482   0.258495  -3.190  0.00144 **
## household.income[100K-200K]  -0.665538   0.240483  -2.768  0.00569 **
## household.income[12K-16K]    -0.058547   0.321515  -0.182  0.85552
```

```
## household.income[16K-25K]      0.007014   0.268398   0.026  0.97915
## household.income[25K-35K]     -0.234232   0.253629  -0.924  0.35583
## household.income[35K-50K]     -0.220153   0.244079  -0.902  0.36716
## household.income[50K-75K]     -0.307151   0.242287  -1.268  0.20502
## household.income[5K-12K]      -0.184737   0.283235  -0.652  0.51431
## household.income[75K-100K]    -0.443821   0.243788  -1.821  0.06880 .
## high.educBachelor              0.128556   0.245633   0.523  0.60077
## high.educHS Diploma/GED        0.027594   0.245196   0.113  0.91041
## high.educPost Graduate Degree  0.159235   0.247798   0.643  0.52054
## high.educSome College          0.208469   0.231117   0.902  0.36714
## demo_race_hispanic1           -0.015424   0.112626  -0.137  0.89108
## ---
## Signif. codes:  0 '***' 0.001 '**' 0.01 '*' 0.05 '.' 0.1 ' ' 1
##
##
## R-sq.(adj) =  0.0257
## lmer.REML = 9702.2  Scale est. = 1.6733    n = 2420
```

```
##                                      stdcoef      stdse
## X(Intercept)                    0.0000000000 0.00000000
## XPDS_score                      0.0656755527 0.02266716
## Xrace.ethnicity.5levelBlack    -0.0120268880 0.05507703
## Xrace.ethnicity.5levelMixed     0.0866796356 0.05170731
## Xrace.ethnicity.5levelOther     0.0793258839 0.03748948
## Xrace.ethnicity.5levelWhite     0.1431509619 0.06898630
## Xinterview_age                 -0.0222047671 0.02092973
## Xbmi                            0.0453345373 0.02212361
## Xhousehold.income[>=200K]      -0.1513995613 0.04746741
## Xhousehold.income[100K-200K]   -0.1674145894 0.06049299
## Xhousehold.income[12K-16K]     -0.0047018054 0.02582043
## Xhousehold.income[16K-25K]      0.0007912011 0.03027502
## Xhousehold.income[25K-35K]     -0.0306993965 0.03324161
## Xhousehold.income[35K-50K]     -0.0340791775 0.03778288
## Xhousehold.income[50K-75K]     -0.0575406001 0.04538926
## Xhousehold.income[5K-12K]      -0.0182321061 0.02795308
## Xhousehold.income[75K-100K]    -0.0874471067 0.04803407
## Xhigh.educBachelor              0.0313857202 0.05996913
## Xhigh.educHS Diploma/GED        0.0040529186 0.03601340
## Xhigh.educPost Graduate Degree  0.0420204104 0.06539116
## Xhigh.educSome College          0.0497747039 0.05518222
## Xdemo_race_hispanic1           -0.0032870789 0.02400240
```

Male participants

```
##
## Family: gaussian
## Link function: identity
##
## Formula:
## cbcl_scr_dsm5_depress_r ~ PDS_score + race.ethnicity.5level +
##     interview_age + bmi + household.income + high.educ + demo_race_hispanic
##
## Parametric coefficients:
```

```
##                               Estimate Std. Error t value Pr(>|t|)
## (Intercept)                  0.9756590  0.7766073   1.256  0.20912
## PDS_score                    0.2005080  0.0800175   2.506  0.01228 *
## race.ethnicity.5levelBlack   0.1839242  0.2979308   0.617  0.53707
## race.ethnicity.5levelMixed   0.6295363  0.2913306   2.161  0.03079 *
## race.ethnicity.5levelOther   0.4746904  0.3435015   1.382  0.16712
## race.ethnicity.5levelWhite   0.5457557  0.2716066   2.009  0.04460 *
## interview_age                0.0004869  0.0054666   0.089  0.92904
## bmi                         -0.0005122  0.0116261  -0.044  0.96486
## household.income[>=200K]    -0.8482320  0.2884279  -2.941  0.00330 **
## household.income[100K-200K] -0.8039332  0.2673921  -3.007  0.00267 **
## household.income[12K-16K]    0.0164045  0.3582129   0.046  0.96348
## household.income[16K-25K]    0.0978119  0.2944241   0.332  0.73975
## household.income[25K-35K]   -0.2008114  0.2898002  -0.693  0.48841
## household.income[35K-50K]   -0.1127505  0.2782281  -0.405  0.68533
## household.income[50K-75K]   -0.5281896  0.2658013  -1.987  0.04701 *
## household.income[5K-12K]    0.4128554  0.3145350   1.313  0.18944
## household.income[75K-100K]  -0.7107097  0.2720483  -2.612  0.00904 **
## high.educBachelor            0.2713006  0.2664893   1.018  0.30875
## high.educHS Diploma/GED     -0.0290162  0.2700281  -0.107  0.91444
## high.educPost Graduate Degree  0.1577469  0.2709935   0.582  0.56055
## high.educSome College        0.1140853  0.2540192   0.449  0.65338
## demo_race_hispanic1         -0.2982529  0.1213805  -2.457  0.01407 *
## ---
## Signif. codes:  0 '***' 0.001 '**' 0.01 '*' 0.05 '.' 0.1 ' ' 1
##
##
## R-sq.(adj) =  0.0184
## lmer.REML =  11224  Scale est. = 2.8078    n = 2627

##                                     stdcoef      stdse
## X(Intercept)                   0.0000000000 0.00000000
## XPDS_score                     0.0521012120 0.02079222
## Xrace.ethnicity.5levelBlack    0.0307165965 0.04975647
## Xrace.ethnicity.5levelMixed    0.0998961422 0.04622895
## Xrace.ethnicity.5levelOther    0.0460674461 0.03333591
## Xrace.ethnicity.5levelWhite    0.1238213030 0.06162222
## Xinterview_age                 0.0017708647 0.01988348
## Xbmi                          -0.0009156615 0.02078207
## Xhousehold.income[>=200K]     -0.1331417489 0.04527275
## Xhousehold.income[100K-200K]  -0.1820611983 0.06055445
## Xhousehold.income[12K-16K]     0.0011379590 0.02484871
## Xhousehold.income[16K-25K]     0.0102406698 0.03082550
## Xhousehold.income[25K-35K]    -0.0225049412 0.03247792
## Xhousehold.income[35K-50K]    -0.0147830986 0.03647943
## Xhousehold.income[50K-75K]    -0.0900179036 0.04529979
## Xhousehold.income[5K-12K]      0.0355955160 0.02711854
## Xhousehold.income[75K-100K]   -0.1201902875 0.04600691
## Xhigh.educBachelor             0.0591529555 0.05810393
## Xhigh.educHS Diploma/GED      -0.0037877342 0.03524906
## Xhigh.educPost Graduate Degree  0.0364233027 0.06257162
## Xhigh.educSome College         0.0241293270 0.05372570
## Xdemo_race_hispanic1          -0.0565539602 0.02301587
```

1.5 Model: CBCL internalizing factor ~ Pubertal category

Female participants

```
##
## Family: gaussian
## Link function: identity
##
## Formula:
## cbcl_scr_syn_internal_r ~ pds_p_ss_category + race.ethnicity.5level +
##     interview_age + bmi + household.income + high.educ + demo_race_hispanic
##
## Parametric coefficients:
##                              Estimate Std. Error t value Pr(>|t|)
## (Intercept)                   5.08076    2.18733   2.323 0.020273 *
## pds_p_ss_categoryEarly        0.96804    0.30158   3.210 0.001345 **
## pds_p_ss_categoryLate         1.03335    0.74541   1.386 0.165788
## pds_p_ss_categoryMid          0.83566    0.30015   2.784 0.005409 **
## race.ethnicity.5levelBlack   -0.65406    0.86623  -0.755 0.450285
## race.ethnicity.5levelMixed    1.27299    0.84780   1.502 0.133356
## race.ethnicity.5levelOther    1.87000    0.96091   1.946 0.051762 .
## race.ethnicity.5levelWhite    1.33650    0.79918   1.672 0.094586 .
## interview_age                -0.01103    0.01538  -0.718 0.473126
## bmi                           0.05221    0.03068   1.702 0.088885 .
## household.income[>=200K]     -2.80617    0.77050  -3.642 0.000276 ***
## household.income[100K-200K]  -2.30649    0.71539  -3.224 0.001281 **
## household.income[12K-16K]    -0.33950    0.95632  -0.355 0.722611
## household.income[16K-25K]    -0.01489    0.79787  -0.019 0.985110
## household.income[25K-35K]    -1.10245    0.75422  -1.462 0.143953
## household.income[35K-50K]    -1.30151    0.72580  -1.793 0.073065 .
## household.income[50K-75K]    -1.25475    0.72066  -1.741 0.081792 .
## household.income[5K-12K]     -0.66961    0.84279  -0.795 0.426975
## household.income[75K-100K]   -1.67574    0.72560  -2.309 0.021004 *
## high.educBachelor             0.18520    0.73167   0.253 0.800192
## high.educHS Diploma/GED      -0.44485    0.72877  -0.610 0.541642
## high.educPost Graduate Degree 0.37269    0.73817   0.505 0.613691
## high.educSome College         0.62253    0.68758   0.905 0.365348
## demo_race_hispanic1          -0.19907    0.34280  -0.581 0.561490
## ---
## Signif. codes:  0 '***' 0.001 '**' 0.01 '*' 0.05 '.' 0.1 ' ' 1
##
##
## R-sq.(adj) =  0.0305
## lmer.REML =  14914  Scale est. = 13.391    n = 2420
```

```
##                                    stdcoef      stdse
## X(Intercept)                    0.0000000000 0.00000000
## Xpds_p_ss_categoryEarly         0.0759719770 0.02366780
## Xpds_p_ss_categoryLate          0.0299980873 0.02163913
## Xpds_p_ss_categoryMid           0.0758280577 0.02723585
## Xrace.ethnicity.5levelBlack    -0.0416575372 0.05517094
## Xrace.ethnicity.5levelMixed     0.0777349016 0.05177118
## Xrace.ethnicity.5levelOther     0.0727952250 0.03740607
## Xrace.ethnicity.5levelWhite     0.1158078036 0.06924865
```

```
## Xinterview_age                 -0.0152084233 0.02119596
## Xbmi                            0.0388342160 0.02281682
## Xhousehold.income[>=200K]      -0.1725486950 0.04737722
## Xhousehold.income[100K-200K]   -0.1942794378 0.06025832
## Xhousehold.income[12K-16K]     -0.0091297954 0.02571682
## Xhousehold.income[16K-25K]     -0.0005625054 0.03013630
## Xhousehold.income[25K-35K]     -0.0483832471 0.03310055
## Xhousehold.income[35K-50K]     -0.0674630767 0.03762140
## Xhousehold.income[50K-75K]     -0.0787106874 0.04520715
## Xhousehold.income[5K-12K]      -0.0221287377 0.02785194
## Xhousehold.income[75K-100K]    -0.1105600978 0.04787314
## Xhigh.educBachelor              0.0151407507 0.05981491
## Xhigh.educHS Diploma/GED       -0.0218787787 0.03584223
## Xhigh.educPost Graduate Degree  0.0329320787 0.06522787
## Xhigh.educSome College          0.0497719993 0.05497279
## Xdemo_race_hispanic1           -0.0142061341 0.02446333
```

Male participants

```
##
## Family: gaussian
## Link function: identity
##
## Formula:
## cbcl_scr_syn_internal_r ~ pds_p_ss_category + race.ethnicity.5level +
##     interview_age + bmi + household.income + high.educ + demo_race_hispanic
##
## Parametric coefficients:
##                               Estimate Std. Error t value Pr(>|t|)
## (Intercept)                    4.18717    2.02779   2.065 0.039032 *
## pds_p_ss_categoryEarly         0.62456    0.25141   2.484 0.013045 *
## pds_p_ss_categoryLate          1.06167    1.67717   0.633 0.526781
## pds_p_ss_categoryMid           0.77091    0.52303   1.474 0.140619
## race.ethnicity.5levelBlack     0.53526    0.77514   0.691 0.489924
## race.ethnicity.5levelMixed     1.98449    0.75629   2.624 0.008742 **
## race.ethnicity.5levelOther     1.57327    0.89373   1.760 0.078467 .
## race.ethnicity.5levelWhite     1.68800    0.70621   2.390 0.016908 *
## interview_age                 -0.00713    0.01416  -0.504 0.614648
## bmi                            0.07273    0.02995   2.428 0.015250 *
## household.income[>=200K]      -2.71252    0.75213  -3.606 0.000316 ***
## household.income[100K-200K]   -2.52965    0.69653  -3.632 0.000287 ***
## household.income[12K-16K]     -1.08888    0.93092  -1.170 0.242235
## household.income[16K-25K]     -0.52745    0.76704  -0.688 0.491740
## household.income[25K-35K]     -1.34054    0.75436  -1.777 0.075676 .
## household.income[35K-50K]     -0.99624    0.72461  -1.375 0.169290
## household.income[50K-75K]     -1.75682    0.69229  -2.538 0.011216 *
## household.income[5K-12K]       0.43490    0.81790   0.532 0.594957
## household.income[75K-100K]    -2.19762    0.70843  -3.102 0.001942 **
## high.educBachelor              0.58260    0.69734   0.835 0.403533
## high.educHS Diploma/GED       -0.31060    0.70558  -0.440 0.659825
## high.educPost Graduate Degree  0.51877    0.70879   0.732 0.464287
## high.educSome College          0.74548    0.66425   1.122 0.261845
## demo_race_hispanic1           -0.42776    0.32190  -1.329 0.184014
```

```
## ---
## Signif. codes:  0 '***' 0.001 '**' 0.01 '*' 0.05 '.' 0.1 ' ' 1
##
##
## R-sq.(adj) =  0.0223
## lmer.REML =  16172  Scale est. = 14.855    n = 2627
```

```
##                                     stdcoef      stdse
## X(Intercept)                     0.000000000 0.00000000
## Xpds_p_ss_categoryEarly          0.050126756 0.02017789
## Xpds_p_ss_categoryLate           0.012166018 0.01921926
## Xpds_p_ss_categoryMid            0.030070790 0.02040165
## Xrace.ethnicity.5levelBlack      0.034332827 0.04971975
## Xrace.ethnicity.5levelMixed      0.120945215 0.04609259
## Xrace.ethnicity.5levelOther      0.058640903 0.03331223
## Xrace.ethnicity.5levelWhite      0.147089659 0.06153775
## Xinterview_age                  -0.009960785 0.01978270
## Xbmi                             0.049931241 0.02056476
## Xhousehold.income[>=200K]       -0.163525351 0.04534236
## Xhousehold.income[100K-200K]    -0.220024692 0.06058271
## Xhousehold.income[12K-16K]      -0.029010443 0.02480194
## Xhousehold.income[16K-25K]      -0.021209446 0.03084375
## Xhousehold.income[25K-35K]      -0.057700743 0.03246985
## Xhousehold.income[35K-50K]      -0.050167646 0.03648899
## Xhousehold.income[50K-75K]      -0.114995240 0.04531464
## Xhousehold.income[5K-12K]        0.014401302 0.02708378
## Xhousehold.income[75K-100K]     -0.142739319 0.04601398
## Xhigh.educBachelor               0.048787833 0.05839608
## Xhigh.educHS Diploma/GED        -0.015572341 0.03537513
## Xhigh.educPost Graduate Degree   0.046005233 0.06285612
## Xhigh.educSome College           0.060557047 0.05395877
## Xdemo_race_hispanic1            -0.031152317 0.02344309
```

1.6 Model: CBCL Anxious-Depressed ~ Pubertal category

Female participants

```
##
## Family: gaussian
## Link function: identity
##
## Formula:
## cbcl_scr_syn_anxdep_r ~ pds_p_ss_category + race.ethnicity.5level +
##     interview_age + bmi + household.income + high.educ + demo_race_hispanic
##
## Parametric coefficients:
##                               Estimate Std. Error t value Pr(>|t|)
## (Intercept)                   2.237706   1.250752   1.789 0.073727 .
## pds_p_ss_categoryEarly        0.457348   0.172985   2.644 0.008250 **
## pds_p_ss_categoryLate         0.273122   0.427555   0.639 0.523014
## pds_p_ss_categoryMid          0.279920   0.171581   1.631 0.102934
## race.ethnicity.5levelBlack   -0.152336   0.492589  -0.309 0.757153
## race.ethnicity.5levelMixed    0.663603   0.482253   1.376 0.168935
```

```
## race.ethnicity.5levelOther     0.756057   0.547164   1.382 0.167170
## race.ethnicity.5levelWhite     0.790522   0.454498   1.739 0.082105 .
## interview_age                 -0.003043   0.008814  -0.345 0.729933
## bmi                            0.007642   0.017541   0.436 0.663136
## household.income[>=200K]      -1.497082   0.438359  -3.415 0.000648 ***
## household.income[100K-200K]   -1.013073   0.407238  -2.488 0.012926 *
## household.income[12K-16K]     -0.220888   0.544847  -0.405 0.685210
## household.income[16K-25K]      0.030298   0.454677   0.067 0.946876
## household.income[25K-35K]     -0.470706   0.429776  -1.095 0.273523
## household.income[35K-50K]     -0.470186   0.413252  -1.138 0.255331
## household.income[50K-75K]     -0.462304   0.410272  -1.127 0.259931
## household.income[5K-12K]      -0.371009   0.479607  -0.774 0.439262
## household.income[75K-100K]    -0.732071   0.413027  -1.772 0.076446 .
## high.educBachelor              0.284008   0.416849   0.681 0.495734
## high.educHS Diploma/GED       -0.160654   0.415712  -0.386 0.699194
## high.educPost Graduate Degree  0.591259   0.420537   1.406 0.159865
## high.educSome College          0.512774   0.391815   1.309 0.190756
## demo_race_hispanic1           -0.074639   0.194659  -0.383 0.701432
## ---
## Signif. codes:  0 '***' 0.001 '**' 0.01 '*' 0.05 '.' 0.1 ' ' 1
##
##
## R-sq.(adj) =  0.0188
## lmer.REML =  12240  Scale est. = 5.1808    n = 2420
```

```
##                                      stdcoef      stdse
## X(Intercept)                     0.000000000 0.00000000
## Xpds_p_ss_categoryEarly          0.063251031 0.02392378
## Xpds_p_ss_categoryLate           0.013972252 0.02187264
## Xpds_p_ss_categoryMid            0.044760756 0.02743679
## Xrace.ethnicity.5levelBlack     -0.017097945 0.05528721
## Xrace.ethnicity.5levelMixed      0.071410707 0.05189549
## Xrace.ethnicity.5levelOther      0.051865438 0.03753541
## Xrace.ethnicity.5levelWhite      0.120710120 0.06940037
## Xinterview_age                  -0.007392315 0.02141120
## Xbmi                             0.010016014 0.02299138
## Xhousehold.income[>=200K]       -0.162220419 0.04749958
## Xhousehold.income[100K-200K]    -0.150375752 0.06044842
## Xhousehold.income[12K-16K]      -0.010467691 0.02581981
## Xhousehold.income[16K-25K]       0.002016703 0.03026382
## Xhousehold.income[25K-35K]      -0.036404017 0.03323851
## Xhousehold.income[35K-50K]      -0.042948697 0.03774816
## Xhousehold.income[50K-75K]      -0.051105307 0.04535350
## Xhousehold.income[5K-12K]       -0.021606452 0.02793085
## Xhousehold.income[75K-100K]     -0.085115261 0.04802116
## Xhigh.educBachelor               0.040915511 0.06005315
## Xhigh.educHS Diploma/GED        -0.013923846 0.03602964
## Xhigh.educPost Graduate Degree   0.092069373 0.06548499
## Xhigh.educSome College           0.072245475 0.05520336
## Xdemo_race_hispanic1            -0.009386370 0.02447978
```

Male participants

```
##
```

```
## Family: gaussian
## Link function: identity
##
## Formula:
## cbcl_scr_syn_anxdep_r ~ pds_p_ss_category + race.ethnicity.5level +
##     interview_age + bmi + household.income + high.educ + demo_race_hispanic
##
## Parametric coefficients:
##                              Estimate Std. Error t value Pr(>|t|)
## (Intercept)                  2.110998   1.142082   1.848  0.06466 .
## pds_p_ss_categoryEarly       0.416288   0.141696   2.938  0.00333 **
## pds_p_ss_categoryLate        0.683264   0.949778   0.719  0.47196
## pds_p_ss_categoryMid         0.324136   0.293717   1.104  0.26988
## race.ethnicity.5levelBlack   0.470661   0.435930   1.080  0.28039
## race.ethnicity.5levelMixed   1.206912   0.425611   2.836  0.00461 **
## race.ethnicity.5levelOther   1.099895   0.501834   2.192  0.02849 *
## race.ethnicity.5levelWhite   1.190217   0.397621   2.993  0.00279 **
## interview_age               -0.006418   0.007983  -0.804  0.42151
## bmi                          0.022004   0.016845   1.306  0.19158
## household.income[>=200K]    -1.063234   0.420692  -2.527  0.01155 *
## household.income[100K-200K] -0.962589   0.389812  -2.469  0.01360 *
## household.income[12K-16K]   -0.361584   0.522502  -0.692  0.48898
## household.income[16K-25K]   -0.024666   0.428828  -0.058  0.95414
## household.income[25K-35K]   -0.318766   0.422208  -0.755  0.45032
## household.income[35K-50K]   -0.256959   0.405576  -0.634  0.52642
## household.income[50K-75K]   -0.737522   0.387331  -1.904  0.05701 .
## household.income[5K-12K]     0.040392   0.458283   0.088  0.92977
## household.income[75K-100K]  -0.815930   0.396417  -2.058  0.03966 *
## high.educBachelor            0.331024   0.389948   0.849  0.39602
## high.educHS Diploma/GED     -0.261228   0.394645  -0.662  0.50807
## high.educPost Graduate Degree  0.333594   0.396336   0.842  0.40004
## high.educSome College        0.284240   0.371595   0.765  0.44439
## demo_race_hispanic1         -0.116018   0.180278  -0.644  0.51993
## ---
## Signif. codes:  0 '***' 0.001 '**' 0.01 '*' 0.05 '.' 0.1 ' ' 1
##
##
## R-sq.(adj) =  0.0113
## lmer.REML =  13190  Scale est. = 6.1496    n = 2627
```

```
##                                    stdcoef      stdse
## X(Intercept)                    0.000000000 0.00000000
## Xpds_p_ss_categoryEarly         0.059843171 0.02036936
## Xpds_p_ss_categoryLate          0.014024121 0.01949439
## Xpds_p_ss_categoryMid           0.022646264 0.02052096
## Xrace.ethnicity.5levelBlack     0.054073218 0.05008303
## Xrace.ethnicity.5levelMixed     0.131747832 0.04646016
## Xrace.ethnicity.5levelOther     0.073430229 0.03350299
## Xrace.ethnicity.5levelWhite     0.185764660 0.06205928
## Xinterview_age                 -0.016057745 0.01997420
## Xbmi                            0.027058410 0.02071457
## Xhousehold.income[>=200K]      -0.114807010 0.04542591
## Xhousehold.income[100K-200K]   -0.149960875 0.06072845
## Xhousehold.income[12K-16K]     -0.017254844 0.02493390
```

```
## Xhousehold.income[16K-25K]     -0.001776530 0.03088586
## Xhousehold.income[25K-35K]     -0.024575407 0.03255037
## Xhousehold.income[35K-50K]     -0.023176685 0.03658132
## Xhousehold.income[50K-75K]     -0.086467720 0.04541097
## Xhousehold.income[5K-12K]       0.002395695 0.02718135
## Xhousehold.income[75K-100K]    -0.094922626 0.04611783
## Xhigh.educBachelor              0.049650639 0.05848878
## Xhigh.educHS Diploma/GED       -0.023458391 0.03543933
## Xhigh.educPost Graduate Degree  0.052987942 0.06295375
## Xhigh.educSome College          0.041356220 0.05406619
## Xdemo_race_hispanic1           -0.015133594 0.02351590
```

1.7 Model: CBCL Withdrawn-Depressed ~ Pubertal category

Female participants

```
##
## Family: gaussian
## Link function: identity
##
## Formula:
## cbcl_scr_syn_withdep_r ~ pds_p_ss_category + race.ethnicity.5level +
##     interview_age + bmi + household.income + high.educ + demo_race_hispanic
##
## Parametric coefficients:
##                                 Estimate Std. Error t value Pr(>|t|)
## (Intercept)                     1.471416   0.617684   2.382 0.017289 *
## pds_p_ss_categoryEarly          0.222248   0.085782   2.591 0.009632 **
## pds_p_ss_categoryLate           0.648799   0.211977   3.061 0.002233 **
## pds_p_ss_categoryMid            0.278102   0.084841   3.278 0.001061 **
## race.ethnicity.5levelBlack     -0.099892   0.241939  -0.413 0.679730
## race.ethnicity.5levelMixed      0.232705   0.237107   0.981 0.326479
## race.ethnicity.5levelOther      0.410360   0.269564   1.522 0.128064
## race.ethnicity.5levelWhite      0.259925   0.223094   1.165 0.244098
## interview_age                  -0.005860   0.004362  -1.343 0.179250
## bmi                             0.015363   0.008674   1.771 0.076668 .
## household.income[>=200K]       -0.773713   0.215937  -3.583 0.000346 ***
## household.income[100K-200K]    -0.714600   0.200872  -3.557 0.000382 ***
## household.income[12K-16K]      -0.253332   0.269137  -0.941 0.346658
## household.income[16K-25K]      -0.168554   0.224701  -0.750 0.453252
## household.income[25K-35K]      -0.339785   0.212295  -1.601 0.109612
## household.income[35K-50K]      -0.527756   0.204039  -2.587 0.009753 **
## household.income[50K-75K]      -0.485478   0.202437  -2.398 0.016553 *
## household.income[5K-12K]       -0.020700   0.236764  -0.087 0.930337
## household.income[75K-100K]     -0.592643   0.203734  -2.909 0.003660 **
## high.educBachelor              -0.019808   0.205721  -0.096 0.923303
## high.educHS Diploma/GED        -0.081636   0.205436  -0.397 0.691123
## high.educPost Graduate Degree -0.045884   0.207531  -0.221 0.825037
## high.educSome College           0.088851   0.193428   0.459 0.646026
## demo_race_hispanic1            -0.003196   0.094670  -0.034 0.973075
## ---
## Signif. codes:  0 '***' 0.001 '**' 0.01 '*' 0.05 '.' 0.1 ' ' 1
##
```

```
##
## R-sq.(adj) =  0.0316
## lmer.REML = 8872.2  Scale est. = 1.4253    n = 2420
```

```
##                                      stdcoef      stdse
## X(Intercept)                       0.0000000000 0.00000000
## Xpds_p_ss_categoryEarly            0.0621159279 0.02397506
## Xpds_p_ss_categoryLate             0.0670754852 0.02191505
## Xpds_p_ss_categoryMid              0.0898692879 0.02741656
## Xrace.ethnicity.5levelBlack       -0.0226577291 0.05487701
## Xrace.ethnicity.5levelMixed        0.0506062847 0.05156372
## Xrace.ethnicity.5levelOther        0.0568895432 0.03737064
## Xrace.ethnicity.5levelWhite        0.0802087570 0.06884338
## Xinterview_age                    -0.0287697844 0.02141467
## Xbmi                               0.0406935273 0.02297618
## Xhousehold.income[>=200K]         -0.1694276970 0.04728588
## Xhousehold.income[100K-200K]      -0.2143603665 0.06025620
## Xhousehold.income[12K-16K]        -0.0242612648 0.02577487
## Xhousehold.income[16K-25K]        -0.0226728001 0.03022528
## Xhousehold.income[25K-35K]        -0.0531065962 0.03318057
## Xhousehold.income[35K-50K]        -0.0974221694 0.03766498
## Xhousehold.income[50K-75K]        -0.1084556402 0.04522430
## Xhousehold.income[5K-12K]         -0.0024362534 0.02786505
## Xhousehold.income[75K-100K]       -0.1392487418 0.04786972
## Xhigh.educBachelor                -0.0057668169 0.05989354
## Xhigh.educHS Diploma/GED          -0.0142986308 0.03598227
## Xhigh.educPost Graduate Degree -0.0144392532 0.06530763
## Xhigh.educSome College             0.0252982327 0.05507422
## Xdemo_race_hispanic1              -0.0008121388 0.02405972
```

Male participants

```
##
## Family: gaussian
## Link function: identity
##
## Formula:
## cbcl_scr_syn_withdep_r ~ pds_p_ss_category + race.ethnicity.5level +
##     interview_age + bmi + household.income + high.educ + demo_race_hispanic
##
## Parametric coefficients:
##                              Estimate Std. Error t value Pr(>|t|)
## (Intercept)                  1.172183   0.634957   1.846 0.064994 .
## pds_p_ss_categoryEarly       0.118748   0.079369   1.496 0.134739
## pds_p_ss_categoryLate        0.246636   0.533876   0.462 0.644139
## pds_p_ss_categoryMid         0.266967   0.164481   1.623 0.104691
## race.ethnicity.5levelBlack   0.102557   0.242445   0.423 0.672320
## race.ethnicity.5levelMixed   0.455863   0.237611   1.919 0.055154 .
## race.ethnicity.5levelOther   0.223129   0.280154   0.796 0.425842
## race.ethnicity.5levelWhite   0.341511   0.220823   1.547 0.122097
## interview_age               -0.002222   0.004449  -0.499 0.617540
## bmi                          0.022058   0.009434   2.338 0.019453 *
## household.income[>=200K]    -0.905391   0.234701  -3.858 0.000117 ***
```

```
## household.income[100K-200K]   -0.861608   0.218192  -3.949 8.06e-05 ***
## household.income[12K-16K]     -0.345477   0.292809  -1.180 0.238160
## household.income[16K-25K]     -0.060328   0.240228  -0.251 0.801736
## household.income[25K-35K]     -0.394042   0.236882  -1.663 0.096341 .
## household.income[35K-50K]     -0.338624   0.227467  -1.489 0.136695
## household.income[50K-75K]     -0.481118   0.216852  -2.219 0.026597 *
## household.income[5K-12K]       0.305266   0.256819   1.189 0.234689
## household.income[75K-100K]    -0.734006   0.222097  -3.305 0.000963 ***
## high.educBachelor              0.045557   0.217284   0.210 0.833947
## high.educHS Diploma/GED       -0.004101   0.220405  -0.019 0.985158
## high.educPost Graduate Degree  0.018882   0.221116   0.085 0.931956
## high.educSome College          0.133770   0.207440   0.645 0.519074
## demo_race_hispanic1           -0.299457   0.095931  -3.122 0.001819 **
## ---
## Signif. codes:  0 '***' 0.001 '**' 0.01 '*' 0.05 '.' 0.1 ' ' 1
##
##
## R-sq.(adj) =  0.034
## lmer.REML =  10181  Scale est. = 2.0597    n = 2627
```

```
##                                       stdcoef      stdse
## X(Intercept)                     0.0000000000 0.00000000
## Xpds_p_ss_categoryEarly          0.0301190887 0.02013122
## Xpds_p_ss_categoryLate           0.0089318202 0.01933408
## Xpds_p_ss_categoryMid            0.0329096169 0.02027586
## Xrace.ethnicity.5levelBlack      0.0207891645 0.04914535
## Xrace.ethnicity.5levelMixed      0.0878007598 0.04576474
## Xrace.ethnicity.5levelOther      0.0262830498 0.03300016
## Xrace.ethnicity.5levelWhite      0.0940454868 0.06081037
## Xinterview_age                  -0.0098094230 0.01964241
## Xbmi                             0.0478586646 0.02046830
## Xhousehold.income[>=200K]       -0.1724930595 0.04471463
## Xhousehold.income[100K-200K]    -0.2368333792 0.05997531
## Xhousehold.income[12K-16K]      -0.0290882138 0.02465376
## Xhousehold.income[16K-25K]      -0.0076663645 0.03052789
## Xhousehold.income[25K-35K]      -0.0536004438 0.03222232
## Xhousehold.income[35K-50K]      -0.0538889940 0.03619937
## Xhousehold.income[50K-75K]      -0.0995236893 0.04485791
## Xhousehold.income[5K-12K]        0.0319455930 0.02687568
## Xhousehold.income[75K-100K]     -0.1506650811 0.04558860
## Xhigh.educBachelor               0.0120562682 0.05750290
## Xhigh.educHS Diploma/GED        -0.0006497177 0.03492176
## Xhigh.educPost Graduate Degree   0.0052916774 0.06196902
## Xhigh.educSome College           0.0343408075 0.05325297
## Xdemo_race_hispanic1            -0.0689204977 0.02207866
```

1.8 Model: CBCL Depressed DSM-5 ~ Pubertal category

Female participants

```
##
## Family: gaussian
## Link function: identity
```

```
##
## Formula:
## cbcl_scr_dsm5_depress_r ~ pds_p_ss_category + race.ethnicity.5level +
##     interview_age + bmi + household.income + high.educ + demo_race_hispanic
##
## Parametric coefficients:
##                              Estimate Std. Error t value Pr(>|t|)
## (Intercept)                  1.247783   0.735176   1.697  0.08978 .
## pds_p_ss_categoryEarly       0.254143   0.101864   2.495  0.01267 *
## pds_p_ss_categoryLate        0.501426   0.251625   1.993  0.04640 *
## pds_p_ss_categoryMid         0.280387   0.101088   2.774  0.00559 **
## race.ethnicity.5levelBlack  -0.022299   0.289338  -0.077  0.93857
## race.ethnicity.5levelMixed   0.500412   0.283570   1.765  0.07774 .
## race.ethnicity.5levelOther   0.712650   0.322220   2.212  0.02708 *
## race.ethnicity.5levelWhite   0.575068   0.266667   2.157  0.03114 *
## interview_age               -0.006476   0.005181  -1.250  0.21142
## bmi                          0.015943   0.010334   1.543  0.12300
## household.income[>=200K]    -0.832805   0.258439  -3.222  0.00129 **
## household.income[100K-200K] -0.692663   0.240329  -2.882  0.00399 **
## household.income[12K-16K]   -0.083009   0.321749  -0.258  0.79643
## household.income[16K-25K]   -0.026447   0.268630  -0.098  0.92158
## household.income[25K-35K]   -0.269880   0.253768  -1.063  0.28767
## household.income[35K-50K]   -0.259486   0.244168  -1.063  0.28801
## household.income[50K-75K]   -0.334439   0.242212  -1.381  0.16748
## household.income[5K-12K]    -0.202605   0.283562  -0.714  0.47499
## household.income[75K-100K]  -0.466477   0.243790  -1.913  0.05581 .
## high.educBachelor            0.126548   0.245935   0.515  0.60691
## high.educHS Diploma/GED      0.040361   0.245317   0.165  0.86933
## high.educPost Graduate Degree  0.159981   0.248106   0.645  0.51911
## high.educSome College        0.215312   0.231201   0.931  0.35180
## demo_race_hispanic1         -0.028884   0.112909  -0.256  0.79811
## ---
## Signif. codes:  0 '***' 0.001 '**' 0.01 '*' 0.05 '.' 0.1 ' ' 1
##
##
## R-sq.(adj) =  0.0257
## lmer.REML = 9703.4  Scale est. = 1.6658    n = 2420
```

```
##                                    stdcoef      stdse
## X(Intercept)                   0.000000000 0.00000000
## Xpds_p_ss_categoryEarly        0.059563829 0.02387407
## Xpds_p_ss_categoryLate         0.043471021 0.02181453
## Xpds_p_ss_categoryMid          0.075981070 0.02739355
## Xrace.ethnicity.5levelBlack   -0.004241411 0.05503383
## Xrace.ethnicity.5levelMixed    0.091256958 0.05171280
## Xrace.ethnicity.5levelOther    0.082848265 0.03745930
## Xrace.ethnicity.5levelWhite    0.148810069 0.06900530
## Xinterview_age                -0.026660852 0.02132856
## Xbmi                           0.035413579 0.02295347
## Xhousehold.income[>=200K]     -0.152927913 0.04745711
## Xhousehold.income[100K-200K]  -0.174237960 0.06045437
## Xhousehold.income[12K-16K]    -0.006666319 0.02583920
## Xhousehold.income[16K-25K]    -0.002983161 0.03030120
## Xhousehold.income[25K-35K]    -0.035371507 0.03325987
```

```
## Xhousehold.income[35K-50K]     -0.040167845 0.03779666
## Xhousehold.income[50K-75K]     -0.062652657 0.04537508
## Xhousehold.income[5K-12K]      -0.019995551 0.02798540
## Xhousehold.income[75K-100K]    -0.091911081 0.04803449
## Xhigh.educBachelor              0.030895565 0.06004292
## Xhigh.educHS Diploma/GED        0.005928084 0.03603113
## Xhigh.educPost Graduate Degree  0.042217175 0.06547243
## Xhigh.educSome College          0.051408665 0.05520235
## Xdemo_race_hispanic1           -0.006155699 0.02406288
```

Male participants

```
##
## Family: gaussian
## Link function: identity
##
## Formula:
## cbcl_scr_dsm5_depress_r ~ pds_p_ss_category + race.ethnicity.5level +
##     interview_age + bmi + household.income + high.educ + demo_race_hispanic
##
## Parametric coefficients:
##                                 Estimate Std. Error t value Pr(>|t|)
## (Intercept)                    1.1079244  0.7810103   1.419  0.15614
## pds_p_ss_categoryEarly         0.1933279  0.0971636   1.990  0.04673 *
## pds_p_ss_categoryLate          0.1491069  0.6513543   0.229  0.81895
## pds_p_ss_categoryMid           0.3223268  0.2015104   1.600  0.10982
## race.ethnicity.5levelBlack     0.1868447  0.2981441   0.627  0.53092
## race.ethnicity.5levelMixed     0.6385088  0.2914657   2.191  0.02856 *
## race.ethnicity.5levelOther     0.4798801  0.3438282   1.396  0.16292
## race.ethnicity.5levelWhite     0.5561845  0.2717601   2.047  0.04080 *
## interview_age                  0.0007846  0.0054647   0.144  0.88584
## bmi                            0.0015652  0.0115543   0.135  0.89226
## household.income[>=200K]      -0.8628826  0.2884115  -2.992  0.00280 **
## household.income[100K-200K]   -0.8148384  0.2675157  -3.046  0.00234 **
## household.income[12K-16K]      0.0196608  0.3585018   0.055  0.95627
## household.income[16K-25K]      0.0815329  0.2944535   0.277  0.78188
## household.income[25K-35K]     -0.2150493  0.2899707  -0.742  0.45838
## household.income[35K-50K]     -0.1174305  0.2785200  -0.422  0.67333
## household.income[50K-75K]     -0.5385479  0.2658549  -2.026  0.04290 *
## household.income[5K-12K]       0.3861785  0.3145493   1.228  0.21966
## household.income[75K-100K]    -0.7216116  0.2721355  -2.652  0.00806 **
## high.educBachelor              0.2730222  0.2672366   1.022  0.30704
## high.educHS Diploma/GED       -0.0227443  0.2706505  -0.084  0.93303
## high.educPost Graduate Degree  0.1647385  0.2717346   0.606  0.54440
## high.educSome College          0.1192573  0.2547837   0.468  0.63977
## demo_race_hispanic1           -0.3083812  0.1216088  -2.536  0.01128 *
## ---
## Signif. codes:  0 '***' 0.001 '**' 0.01 '*' 0.05 '.' 0.1 ' ' 1
##
##
## R-sq.(adj) =  0.0174
## lmer.REML =  11225  Scale est. = 2.802     n = 2627
```

```
##                                     stdcoef      stdse
```

```
## X(Intercept)                      0.000000000 0.00000000
## Xpds_p_ss_categoryEarly           0.040399476 0.02030414
## Xpds_p_ss_categoryLate            0.004448824 0.01943412
## Xpds_p_ss_categoryMid             0.032735978 0.02046569
## Xrace.ethnicity.5levelBlack       0.031204331 0.04979208
## Xrace.ethnicity.5levelMixed       0.101319916 0.04625039
## Xrace.ethnicity.5levelOther       0.046571096 0.03336761
## Xrace.ethnicity.5levelWhite       0.126187390 0.06165706
## Xinterview_age                    0.002853917 0.01987647
## Xbmi                              0.002797796 0.02065364
## Xhousehold.income[>=200K]        -0.135441355 0.04527017
## Xhousehold.income[100K-200K]     -0.184530832 0.06058244
## Xhousehold.income[12K-16K]        0.001363838 0.02486874
## Xhousehold.income[16K-25K]        0.008536303 0.03082858
## Xhousehold.income[25K-35K]       -0.024100582 0.03249703
## Xhousehold.income[35K-50K]       -0.015396706 0.03651770
## Xhousehold.income[50K-75K]       -0.091783247 0.04530892
## Xhousehold.income[5K-12K]         0.033295492 0.02711977
## Xhousehold.income[75K-100K]      -0.122033945 0.04602166
## Xhigh.educBachelor                0.059528323 0.05826687
## Xhigh.educHS Diploma/GED         -0.002969000 0.03533031
## Xhigh.educPost Graduate Degree    0.038037644 0.06274274
## Xhigh.educSome College            0.025223220 0.05388741
## Xdemo_race_hispanic1             -0.058474463 0.02305916
```

1.9 Model: CBCL internalizing factor ~ Testosterone

Female participants

```
##
## Family: gaussian
## Link function: identity
##
## Formula:
## cbcl_scr_syn_internal_r ~ hormone_scr_ert_mean + hormone_sal_end_min_since_midnight +
##     race.ethnicity.5level + interview_age + bmi + household.income +
##     high.educ + demo_race_hispanic
##
## Parametric coefficients:
##                                     Estimate Std. Error t value Pr(>|t|)
## (Intercept)                        1.6588323  2.2615754   0.733  0.46334
## hormone_scr_ert_mean              -0.0026370  0.0073583  -0.358  0.72010
## hormone_sal_end_min_since_midnight -0.0001973  0.0006845  -0.288  0.77317
## race.ethnicity.5levelBlack        -0.6240199  0.8733463  -0.715  0.47498
## race.ethnicity.5levelMixed         1.0706228  0.8523047   1.256  0.20919
## race.ethnicity.5levelOther         1.9309965  0.9700818   1.991  0.04665 *
## race.ethnicity.5levelWhite         1.2930446  0.8021421   1.612  0.10711
## interview_age                      0.0115741  0.0153662   0.753  0.45140
## bmi                                0.0924760  0.0305522   3.027  0.00250 **
## household.income[>=200K]          -2.2497853  0.8144795  -2.762  0.00579 **
## household.income[100K-200K]       -1.6855646  0.7593305  -2.220  0.02653 *
## household.income[12K-16K]          0.2950437  1.0148288   0.291  0.77128
## household.income[16K-25K]          0.7862198  0.8515711   0.923  0.35597
```

```
## household.income[25K-35K]          -0.4137149  0.7977787  -0.519  0.60410
## household.income[35K-50K]          -0.2237425  0.7689633  -0.291  0.77110
## household.income[50K-75K]          -0.6171795  0.7663473  -0.805  0.42070
## household.income[5K-12K]            0.2368349  0.8995923   0.263  0.79237
## household.income[75K-100K]         -1.0669749  0.7689027  -1.388  0.16538
## high.educBachelor                   0.4579963  0.7578390   0.604  0.54568
## high.educHS Diploma/GED            -0.5167084  0.7602901  -0.680  0.49682
## high.educPost Graduate Degree       0.6709191  0.7651295   0.877  0.38065
## high.educSome College               0.7279549  0.7133271   1.021  0.30760
## demo_race_hispanic1                -0.2603928  0.3529664  -0.738  0.46076
## ---
## Signif. codes:  0 '***' 0.001 '**' 0.01 '*' 0.05 '.' 0.1 ' ' 1
##
##
## R-sq.(adj) =  0.0262
## lmer.REML =  13817  Scale est. = 13.585    n = 2239
```

```
##                                          stdcoef      stdse
## X(Intercept)                         0.000000000 0.00000000
## Xhormone_scr_ert_mean               -0.007874193 0.02197199
## Xhormone_sal_end_min_since_midnight -0.006468581 0.02243919
## Xrace.ethnicity.5levelBlack         -0.038559018 0.05396523
## Xrace.ethnicity.5levelMixed          0.066252569 0.05274255
## Xrace.ethnicity.5levelOther          0.076585374 0.03847448
## Xrace.ethnicity.5levelWhite          0.112019429 0.06949141
## Xinterview_age                       0.016125083 0.02140837
## Xbmi                                 0.068565752 0.02265276
## Xhousehold.income[>=200K]           -0.138400137 0.05010437
## Xhousehold.income[100K-200K]        -0.143028262 0.06443285
## Xhousehold.income[12K-16K]           0.007875706 0.02708918
## Xhousehold.income[16K-25K]           0.029206497 0.03163417
## Xhousehold.income[25K-35K]          -0.018211744 0.03511825
## Xhousehold.income[35K-50K]          -0.011680752 0.04014467
## Xhousehold.income[50K-75K]          -0.038581190 0.04790599
## Xhousehold.income[5K-12K]            0.007700212 0.02924844
## Xhousehold.income[75K-100K]         -0.071327852 0.05140156
## Xhigh.educBachelor                   0.037769617 0.06249676
## Xhigh.educHS Diploma/GED            -0.024949423 0.03671084
## Xhigh.educPost Graduate Degree       0.059646251 0.06802177
## Xhigh.educSome College               0.057857166 0.05669456
## Xdemo_race_hispanic1                -0.018682390 0.02532427
```

Male participants

```
##
## Family: gaussian
## Link function: identity
##
## Formula:
## cbcl_scr_syn_internal_r ~ hormone_scr_ert_mean + hormone_sal_end_min_since_midnight +
##     race.ethnicity.5level + interview_age + bmi + household.income +
##     high.educ + demo_race_hispanic
##
```

```
## Parametric coefficients:
##                                     Estimate Std. Error t value Pr(>|t|)
## (Intercept)                        2.8305430  2.1912802   1.292 0.196574
## hormone_scr_ert_mean              -0.0018477  0.0074522  -0.248 0.804206
## hormone_sal_end_min_since_midnight  0.0005432  0.0006555   0.829 0.407362
## race.ethnicity.5levelBlack         0.6692201  0.8050685   0.831 0.405910
## race.ethnicity.5levelMixed         1.9905022  0.7853397   2.535 0.011321 *
## race.ethnicity.5levelOther         1.4162368  0.9332018   1.518 0.129243
## race.ethnicity.5levelWhite         1.6828133  0.7322868   2.298 0.021645 *
## interview_age                     -0.0007187  0.0148280  -0.048 0.961344
## bmi                                0.0825284  0.0316322   2.609 0.009137 **
## household.income[>=200K]          -2.6430038  0.7954906  -3.322 0.000905 ***
## household.income[100K-200K]       -2.5172539  0.7392486  -3.405 0.000672 ***
## household.income[12K-16K]         -0.9400101  0.9979623  -0.942 0.346323
## household.income[16K-25K]         -0.4400764  0.8221541  -0.535 0.592511
## household.income[25K-35K]         -1.2313914  0.8015161  -1.536 0.124589
## household.income[35K-50K]         -0.9048572  0.7703956  -1.175 0.240296
## household.income[50K-75K]         -1.6204153  0.7373372  -2.198 0.028068 *
## household.income[5K-12K]           0.7221355  0.8621310   0.838 0.402329
## household.income[75K-100K]        -2.1646048  0.7514565  -2.881 0.004005 **
## high.educBachelor                  0.8448932  0.7358582   1.148 0.251010
## high.educHS Diploma/GED           -0.1420821  0.7430811  -0.191 0.848380
## high.educPost Graduate Degree      0.7162532  0.7464135   0.960 0.337356
## high.educSome College              0.9789514  0.7000419   1.398 0.162116
## demo_race_hispanic1               -0.4010861  0.3340153  -1.201 0.229946
## ---
## Signif. codes:  0 '***' 0.001 '**' 0.01 '*' 0.05 '.' 0.1 ' ' 1
##
##
## R-sq.(adj) =   0.02
## lmer.REML =  15105  Scale est. = 14.518    n = 2441
```

```
##                                          stdcoef      stdse
## X(Intercept)                        0.0000000000 0.00000000
## Xhormone_scr_ert_mean              -0.0052704207 0.02125725
## Xhormone_sal_end_min_since_midnight  0.0175811851 0.02121566
## Xrace.ethnicity.5levelBlack         0.0422108310 0.05077943
## Xrace.ethnicity.5levelMixed         0.1205736742 0.04757156
## Xrace.ethnicity.5levelOther         0.0522096503 0.03440254
## Xrace.ethnicity.5levelWhite         0.1450994752 0.06314095
## Xinterview_age                     -0.0009972119 0.02057299
## Xbmi                                0.0562379725 0.02155537
## Xhousehold.income[>=200K]          -0.1589487073 0.04784034
## Xhousehold.income[100K-200K]       -0.2169053457 0.06369917
## Xhousehold.income[12K-16K]         -0.0242890678 0.02578650
## Xhousehold.income[16K-25K]         -0.0171071796 0.03195976
## Xhousehold.income[25K-35K]         -0.0525721682 0.03421937
## Xhousehold.income[35K-50K]         -0.0454071523 0.03865966
## Xhousehold.income[50K-75K]         -0.1039994928 0.04732287
## Xhousehold.income[5K-12K]           0.0241117846 0.02878617
## Xhousehold.income[75K-100K]        -0.1402590616 0.04869184
## Xhigh.educBachelor                  0.0700304182 0.06099286
## Xhigh.educHS Diploma/GED           -0.0070801351 0.03702869
## Xhigh.educPost Graduate Degree      0.0628996212 0.06554822
```

```
## Xhigh.educSome College               0.0786738878 0.05625919
## Xdemo_race_hispanic1                -0.0289455101 0.02410516
```

1.10 Model: CBCL Anxious-Depressed ~ Testosterone

Female participants

```
##
## Family: gaussian
## Link function: identity
##
## Formula:
## cbcl_scr_syn_anxdep_r ~ hormone_scr_ert_mean + hormone_sal_end_min_since_midnight +
##     race.ethnicity.5level + interview_age + bmi + household.income +
##     high.educ + demo_race_hispanic
##
## Parametric coefficients:
##                                      Estimate Std. Error t value Pr(>|t|)
## (Intercept)                         1.1052703  1.3000119   0.850   0.3953
## hormone_scr_ert_mean                0.0017462  0.0042352   0.412   0.6801
## hormone_sal_end_min_since_midnight -0.0002491  0.0003927  -0.634   0.5260
## race.ethnicity.5levelBlack         -0.2348006  0.4996861  -0.470   0.6385
## race.ethnicity.5levelMixed          0.5457718  0.4877352   1.119   0.2633
## race.ethnicity.5levelOther          0.7722479  0.5555977   1.390   0.1647
## race.ethnicity.5levelWhite          0.7870124  0.4590129   1.715   0.0866 .
## interview_age                       0.0039389  0.0088512   0.445   0.6564
## bmi                                 0.0231365  0.0175594   1.318   0.1878
## household.income[>=200K]           -1.1410848  0.4661564  -2.448   0.0144 *
## household.income[100K-200K]        -0.6329692  0.4347897  -1.456   0.1456
## household.income[12K-16K]           0.1454448  0.5813603   0.250   0.8025
## household.income[16K-25K]           0.5762044  0.4879294   1.181   0.2378
## household.income[25K-35K]          -0.0194263  0.4570938  -0.042   0.9661
## household.income[35K-50K]           0.1106987  0.4403279   0.251   0.8015
## household.income[50K-75K]          -0.0555616  0.4388159  -0.127   0.8993
## household.income[5K-12K]            0.0655370  0.5148758   0.127   0.8987
## household.income[75K-100K]         -0.3659170  0.4402197  -0.831   0.4059
## high.educBachelor                   0.2991967  0.4341951   0.689   0.4908
## high.educHS Diploma/GED            -0.2848212  0.4360820  -0.653   0.5137
## high.educPost Graduate Degree       0.6423942  0.4383499   1.465   0.1429
## high.educSome College               0.4587277  0.4087598   1.122   0.2619
## demo_race_hispanic1                -0.1074454  0.2018505  -0.532   0.5946
## ---
## Signif. codes:  0 '***' 0.001 '**' 0.01 '*' 0.05 '.' 0.1 ' ' 1
##
##
## R-sq.(adj) =  0.019
## lmer.REML =  11364  Scale est. = 5.1815    n = 2239
```

```
##                                          stdcoef      stdse
## X(Intercept)                         0.000000000 0.00000000
## Xhormone_scr_ert_mean                0.009116347 0.02210978
## Xhormone_sal_end_min_since_midnight -0.014274908 0.02250652
## Xrace.ethnicity.5levelBlack         -0.025365894 0.05398192
```

```
## Xrace.ethnicity.5levelMixed          0.059047407 0.05276839
## Xrace.ethnicity.5levelOther          0.053548168 0.03852550
## Xrace.ethnicity.5levelWhite          0.119202384 0.06952296
## Xinterview_age                       0.009594252 0.02155974
## Xbmi                                 0.029991622 0.02276201
## Xhousehold.income[>=200K]           -0.122726087 0.05013611
## Xhousehold.income[100K-200K]        -0.093903672 0.06450290
## Xhousehold.income[12K-16K]           0.006787735 0.02713139
## Xhousehold.income[16K-25K]           0.037422741 0.03168955
## Xhousehold.income[25K-35K]          -0.001495077 0.03517865
## Xhousehold.income[35K-50K]           0.010103885 0.04019037
## Xhousehold.income[50K-75K]          -0.006072431 0.04795898
## Xhousehold.income[5K-12K]            0.003725348 0.02926733
## Xhousehold.income[75K-100K]         -0.042767217 0.05145149
## Xhigh.educBachelor                   0.043138083 0.06260211
## Xhigh.educHS Diploma/GED            -0.024044225 0.03681346
## Xhigh.educPost Graduate Degree       0.099847718 0.06813299
## Xhigh.educSome College               0.063742796 0.05679948
## Xdemo_race_hispanic1                -0.013477666 0.02531960
```

Male participants

```
##
## Family: gaussian
## Link function: identity
##
## Formula:
## cbcl_scr_syn_anxdep_r ~ hormone_scr_ert_mean + hormone_sal_end_min_since_midnight +
##     race.ethnicity.5level + interview_age + bmi + household.income +
##     high.educ + demo_race_hispanic
##
## Parametric coefficients:
##                                       Estimate Std. Error t value Pr(>|t|)
## (Intercept)                          1.798e+00  1.238e+00   1.453  0.14637
## hormone_scr_ert_mean                -1.504e-03  4.215e-03  -0.357  0.72118
## hormone_sal_end_min_since_midnight -2.791e-05  3.712e-04  -0.075  0.94008
## race.ethnicity.5levelBlack           5.635e-01  4.542e-01   1.241  0.21485
## race.ethnicity.5levelMixed           1.194e+00  4.434e-01   2.692  0.00716 **
## race.ethnicity.5levelOther           9.983e-01  5.253e-01   1.900  0.05750 .
## race.ethnicity.5levelWhite           1.147e+00  4.137e-01   2.773  0.00560 **
## interview_age                       -3.186e-03  8.381e-03  -0.380  0.70384
## bmi                                  2.554e-02  1.784e-02   1.432  0.15240
## household.income[>=200K]            -1.064e+00  4.455e-01  -2.387  0.01705 *
## household.income[100K-200K]         -9.860e-01  4.143e-01  -2.380  0.01738 *
## household.income[12K-16K]           -2.907e-01  5.613e-01  -0.518  0.60455
## household.income[16K-25K]           -2.666e-02  4.603e-01  -0.058  0.95382
## household.income[25K-35K]           -3.319e-01  4.493e-01  -0.739  0.46015
## household.income[35K-50K]           -2.302e-01  4.318e-01  -0.533  0.59405
## household.income[50K-75K]           -7.178e-01  4.131e-01  -1.738  0.08240 .
## household.income[5K-12K]            1.376e-01  4.839e-01   0.284  0.77610
## household.income[75K-100K]          -8.301e-01  4.211e-01  -1.972  0.04877 *
## high.educBachelor                    4.494e-01  4.121e-01   1.091  0.27555
## high.educHS Diploma/GED             -1.971e-01  4.162e-01  -0.474  0.63583
```

```
## high.educPost Graduate Degree       4.330e-01  4.180e-01   1.036  0.30030
## high.educSome College               3.733e-01  3.922e-01   0.952  0.34121
## demo_race_hispanic1                -7.894e-02  1.877e-01  -0.421  0.67406
## ---
## Signif. codes:  0 '***' 0.001 '**' 0.01 '*' 0.05 '.' 0.1 ' ' 1
##
##
## R-sq.(adj) =  0.00748
## lmer.REML =  12349  Scale est. = 6.403     n = 2441
```

```
##                                         stdcoef      stdse
## X(Intercept)                          0.000000000 0.00000000
## Xhormone_scr_ert_mean                -0.007671091 0.02149259
## Xhormone_sal_end_min_since_midnight -0.001614789 0.02147838
## Xrace.ethnicity.5levelBlack           0.063537004 0.05121164
## Xrace.ethnicity.5levelMixed           0.129257030 0.04802241
## Xrace.ethnicity.5levelOther           0.065792877 0.03462018
## Xrace.ethnicity.5levelWhite           0.176831174 0.06377566
## Xinterview_age                       -0.007903054 0.02078742
## Xbmi                                  0.031120209 0.02173874
## Xhousehold.income[>=200K]            -0.114354685 0.04790288
## Xhousehold.income[100K-200K]         -0.151897312 0.06381651
## Xhousehold.income[12K-16K]           -0.013429322 0.02592884
## Xhousehold.income[16K-25K]           -0.001852476 0.03198861
## Xhousehold.income[25K-35K]           -0.025331174 0.03429060
## Xhousehold.income[35K-50K]           -0.020648874 0.03873754
## Xhousehold.income[50K-75K]           -0.082360079 0.04739816
## Xhousehold.income[5K-12K]             0.008215611 0.02888375
## Xhousehold.income[75K-100K]          -0.096164093 0.04877562
## Xhigh.educBachelor                    0.066595037 0.06106137
## Xhigh.educHS Diploma/GED             -0.017559690 0.03707760
## Xhigh.educPost Graduate Degree        0.067984440 0.06562075
## Xhigh.educSome College                0.053640287 0.05634748
## Xdemo_race_hispanic1                 -0.010184792 0.02421330
```

1.11 Model: CBCL Withdrawn-Depressed ~ Testosterone

Female participants

```
##
## Family: gaussian
## Link function: identity
##
## Formula:
## cbcl_scr_syn_withdep_r ~ hormone_scr_ert_mean + hormone_sal_end_min_since_midnight +
##     race.ethnicity.5level + interview_age + bmi + household.income +
##     high.educ + demo_race_hispanic
##
## Parametric coefficients:
##                                      Estimate Std. Error t value Pr(>|t|)
## (Intercept)                         3.509e-01  6.286e-01   0.558 0.576788
## hormone_scr_ert_mean               -1.582e-03  2.052e-03  -0.771 0.440853
## hormone_sal_end_min_since_midnight  2.237e-05  1.864e-04   0.120 0.904519
```

```
## race.ethnicity.5levelBlack         -4.980e-02  2.396e-01  -0.208 0.835408
## race.ethnicity.5levelMixed          2.104e-01  2.342e-01   0.898 0.369229
## race.ethnicity.5levelOther          3.947e-01  2.677e-01   1.474 0.140625
## race.ethnicity.5levelWhite          2.634e-01  2.199e-01   1.198 0.231134
## interview_age                       1.825e-03  4.296e-03   0.425 0.670993
## bmi                                 2.913e-02  8.509e-03   3.423 0.000631 ***
## household.income[>=200K]          -6.952e-01  2.247e-01  -3.094 0.002000 **
## household.income[100K-200K]       -6.208e-01  2.100e-01  -2.957 0.003143 **
## household.income[12K-16K]         -3.749e-02  2.812e-01  -0.133 0.893967
## household.income[16K-25K]         -3.028e-02  2.362e-01  -0.128 0.897993
## household.income[25K-35K]         -2.001e-01  2.211e-01  -0.905 0.365449
## household.income[35K-50K]         -3.094e-01  2.129e-01  -1.453 0.146373
## household.income[50K-75K]         -3.972e-01  2.121e-01  -1.873 0.061205 .
## household.income[5K-12K]           2.209e-01  2.489e-01   0.887 0.374968
## household.income[75K-100K]        -5.158e-01  2.126e-01  -2.426 0.015345 *
## high.educBachelor                   5.996e-02  2.097e-01   0.286 0.775018
## high.educHS Diploma/GED            -1.251e-01  2.111e-01  -0.593 0.553555
## high.educPost Graduate Degree       3.723e-02  2.118e-01   0.176 0.860451
## high.educSome College               1.197e-01  1.975e-01   0.606 0.544582
## demo_race_hispanic1                -2.398e-02  9.562e-02  -0.251 0.801987
## ---
## Signif. codes:  0 '***' 0.001 '**' 0.01 '*' 0.05 '.' 0.1 ' ' 1
##
##
## R-sq.(adj) =  0.0245
## lmer.REML = 8164.3  Scale est. = 1.4664    n = 2239
```

```
##                                          stdcoef      stdse
## X(Intercept)                         0.000000000 0.00000000
## Xhormone_scr_ert_mean               -0.017099033 0.02218084
## Xhormone_sal_end_min_since_midnight  0.002653764 0.02212048
## Xrace.ethnicity.5levelBlack         -0.011136846 0.05359520
## Xrace.ethnicity.5levelMixed          0.047117600 0.05246355
## Xrace.ethnicity.5levelOther          0.056650882 0.03843361
## Xrace.ethnicity.5levelWhite          0.082598494 0.06896019
## Xinterview_age                       0.009202388 0.02166075
## Xbmi                                 0.078166085 0.02283535
## Xhousehold.income[>=200K]           -0.154779295 0.05002722
## Xhousehold.income[100K-200K]        -0.190671148 0.06448883
## Xhousehold.income[12K-16K]          -0.003621971 0.02717128
## Xhousehold.income[16K-25K]          -0.004071730 0.03175787
## Xhousehold.income[25K-35K]          -0.031888300 0.03522724
## Xhousehold.income[35K-50K]          -0.058453893 0.04023059
## Xhousehold.income[50K-75K]          -0.089866898 0.04798108
## Xhousehold.income[5K-12K]            0.025989233 0.02928742
## Xhousehold.income[75K-100K]         -0.124800142 0.05144236
## Xhigh.educBachelor                   0.017895422 0.06260369
## Xhigh.educHS Diploma/GED            -0.021863243 0.03689799
## Xhigh.educPost Graduate Degree       0.011979471 0.06813469
## Xhigh.educSome College               0.034434941 0.05682406
## Xdemo_race_hispanic1                -0.006227655 0.02483051
```

Male participants

```
##
## Family: gaussian
## Link function: identity
##
## Formula:
## cbcl_scr_syn_withdep_r ~ hormone_scr_ert_mean + hormone_sal_end_min_since_midnight +
##     race.ethnicity.5level + interview_age + bmi + household.income +
##     high.educ + demo_race_hispanic
##
## Parametric coefficients:
##                                     Estimate Std. Error t value Pr(>|t|)
## (Intercept)                        9.079e-01  6.768e-01   1.341 0.179905
## hormone_scr_ert_mean              -1.529e-04  2.291e-03  -0.067 0.946820
## hormone_sal_end_min_since_midnight -2.102e-05  1.948e-04  -0.108 0.914107
## race.ethnicity.5levelBlack         1.571e-01  2.482e-01   0.633 0.526786
## race.ethnicity.5levelMixed         4.722e-01  2.432e-01   1.942 0.052290 .
## race.ethnicity.5levelOther         2.252e-01  2.879e-01   0.782 0.434174
## race.ethnicity.5levelWhite         3.551e-01  2.258e-01   1.572 0.115975
## interview_age                     -6.192e-04  4.591e-03  -0.135 0.892720
## bmi                                2.529e-02  9.809e-03   2.578 0.009992 **
## household.income[>=200K]          -9.194e-01  2.439e-01  -3.769 0.000168 ***
## household.income[100K-200K]       -8.971e-01  2.274e-01  -3.945 8.22e-05 ***
## household.income[12K-16K]         -4.030e-01  3.091e-01  -1.304 0.192382
## household.income[16K-25K]         -1.638e-01  2.530e-01  -0.647 0.517383
## household.income[25K-35K]         -3.563e-01  2.473e-01  -1.441 0.149686
## household.income[35K-50K]         -3.405e-01  2.375e-01  -1.434 0.151810
## household.income[50K-75K]         -4.626e-01  2.268e-01  -2.039 0.041509 *
## household.income[5K-12K]           3.510e-01  2.662e-01   1.319 0.187398
## household.income[75K-100K]        -7.464e-01  2.314e-01  -3.226 0.001271 **
## high.educBachelor                  1.424e-01  2.251e-01   0.633 0.527103
## high.educHS Diploma/GED            3.734e-02  2.279e-01   0.164 0.869856
## high.educPost Graduate Degree      8.053e-02  2.286e-01   0.352 0.724685
## high.educSome College              2.261e-01  2.147e-01   1.053 0.292397
## demo_race_hispanic1               -2.817e-01  9.876e-02  -2.852 0.004378 **
## ---
## Signif. codes:  0 '***' 0.001 '**' 0.01 '*' 0.05 '.' 0.1 ' ' 1
##
##
## R-sq.(adj) =  0.0333
## lmer.REML = 9468.2  Scale est. = 2.1688    n = 2441
```

```
##                                         stdcoef      stdse
## X(Intercept)                         0.000000000 0.00000000
## Xhormone_scr_ert_mean               -0.001398718 0.02096795
## Xhormone_sal_end_min_since_midnight -0.002182305 0.02023077
## Xrace.ethnicity.5levelBlack          0.031788852 0.05021847
## Xrace.ethnicity.5levelMixed          0.091763986 0.04725978
## Xrace.ethnicity.5levelOther          0.026634995 0.03405140
## Xrace.ethnicity.5levelWhite          0.098231768 0.06247008
## Xinterview_age                      -0.002756175 0.02043472
## Xbmi                                 0.055285616 0.02144407
## Xhousehold.income[>=200K]           -0.177378100 0.04705747
```

```
## Xhousehold.income[100K-200K]        -0.247996531 0.06287142
## Xhousehold.income[12K-16K]          -0.033406071 0.02561957
## Xhousehold.income[16K-25K]          -0.020425760 0.03154655
## Xhousehold.income[25K-35K]          -0.048803408 0.03386541
## Xhousehold.income[35K-50K]          -0.054810531 0.03823227
## Xhousehold.income[50K-75K]          -0.095257441 0.04670633
## Xhousehold.income[5K-12K]            0.037597260 0.02851106
## Xhousehold.income[75K-100K]         -0.155161399 0.04809482
## Xhigh.educBachelor                   0.037866447 0.05986549
## Xhigh.educHS Diploma/GED             0.005968970 0.03642743
## Xhigh.educPost Graduate Degree       0.022688521 0.06441115
## Xhigh.educSome College               0.058288090 0.05534869
## Xdemo_race_hispanic1                -0.065218031 0.02286572
```

1.12 Model: CBCL Depressed DSM-5 ~ Testosterone

Female participants

```
##
## Family: gaussian
## Link function: identity
##
## Formula:
## cbcl_scr_dsm5_depress_r ~ hormone_scr_ert_mean + hormone_sal_end_min_since_midnight +
##     race.ethnicity.5level + interview_age + bmi + household.income +
##     high.educ + demo_race_hispanic
##
## Parametric coefficients:
##                                      Estimate Std. Error t value Pr(>|t|)
## (Intercept)                         3.522e-01  7.567e-01   0.465  0.64163
## hormone_scr_ert_mean               -1.427e-03  2.467e-03  -0.579  0.56289
## hormone_sal_end_min_since_midnight  3.798e-05  2.234e-04   0.170  0.86498
## race.ethnicity.5levelBlack         -3.087e-02  2.901e-01  -0.106  0.91527
## race.ethnicity.5levelMixed          4.476e-01  2.836e-01   1.578  0.11460
## race.ethnicity.5levelOther          7.004e-01  3.241e-01   2.161  0.03082 *
## race.ethnicity.5levelWhite          5.683e-01  2.660e-01   2.137  0.03273 *
## interview_age                       7.429e-04  5.161e-03   0.144  0.88555
## bmi                                 2.693e-02  1.026e-02   2.624  0.00875 **
## household.income[>=200K]           -7.676e-01  2.724e-01  -2.817  0.00488 **
## household.income[100K-200K]        -6.345e-01  2.545e-01  -2.493  0.01275 *
## household.income[12K-16K]           1.757e-03  3.407e-01   0.005  0.99589
## household.income[16K-25K]           8.674e-02  2.862e-01   0.303  0.76186
## household.income[25K-35K]          -1.500e-01  2.678e-01  -0.560  0.57549
## household.income[35K-50K]          -8.148e-02  2.582e-01  -0.315  0.75241
## household.income[50K-75K]          -2.602e-01  2.571e-01  -1.012  0.31165
## household.income[5K-12K]            1.290e-02  3.022e-01   0.043  0.96595
## household.income[75K-100K]         -3.974e-01  2.578e-01  -1.541  0.12337
## high.educBachelor                   1.109e-01  2.540e-01   0.437  0.66240
## high.educHS Diploma/GED            -4.673e-02  2.554e-01  -0.183  0.85483
## high.educPost Graduate Degree       1.566e-01  2.565e-01   0.611  0.54157
## high.educSome College               1.774e-01  2.392e-01   0.742  0.45846
## demo_race_hispanic1                -7.429e-02  1.151e-01  -0.645  0.51870
## ---
```

```
## Signif. codes:  0 '***' 0.001 '**' 0.01 '*' 0.05 '.' 0.1 ' ' 1
##
##
## R-sq.(adj) =  0.0227
## lmer.REML = 8987.3  Scale est. = 1.6772    n = 2239
```

```
##                                          stdcoef      stdse
## X(Intercept)                        0.0000000000 0.00000000
## Xhormone_scr_ert_mean              -0.0127467535 0.02202877
## Xhormone_sal_end_min_since_midnight  0.0037237527 0.02189679
## Xrace.ethnicity.5levelBlack        -0.0057036039 0.05360429
## Xrace.ethnicity.5levelMixed         0.0828432010 0.05248352
## Xrace.ethnicity.5levelOther         0.0830680289 0.03844284
## Xrace.ethnicity.5levelWhite         0.1472286648 0.06890575
## Xinterview_age                      0.0030951272 0.02150130
## Xbmi                                0.0597064131 0.02275467
## Xhousehold.income[>=200K]          -0.1412085706 0.05011889
## Xhousehold.income[100K-200K]       -0.1610040723 0.06458716
## Xhousehold.income[12K-16K]          0.0001402471 0.02719884
## Xhousehold.income[16K-25K]          0.0096359829 0.03179421
## Xhousehold.income[25K-35K]         -0.0197455459 0.03525538
## Xhousehold.income[35K-50K]         -0.0127205379 0.04031868
## Xhousehold.income[50K-75K]         -0.0486464679 0.04807018
## Xhousehold.income[5K-12K]           0.0012545580 0.02938546
## Xhousehold.income[75K-100K]        -0.0794430068 0.05154107
## Xhigh.educBachelor                  0.0273526377 0.06264096
## Xhigh.educHS Diploma/GED           -0.0067471398 0.03687383
## Xhigh.educPost Graduate Degree      0.0416286356 0.06818332
## Xhigh.educSome College              0.0421561834 0.05685095
## Xdemo_race_hispanic1               -0.0159401208 0.02469626
```

Male participants

```
##
## Family: gaussian
## Link function: identity
##
## Formula:
## cbcl_scr_dsm5_depress_r ~ hormone_scr_ert_mean + hormone_sal_end_min_since_midnight +
##     race.ethnicity.5level + interview_age + bmi + household.income +
##     high.educ + demo_race_hispanic
##
## Parametric coefficients:
##                                     Estimate Std. Error t value Pr(>|t|)
## (Intercept)                        0.7277812  0.8419088   0.864  0.38743
## hormone_scr_ert_mean               0.0004013  0.0028591   0.140  0.88840
## hormone_sal_end_min_since_midnight  0.0001355  0.0002475   0.548  0.58403
## race.ethnicity.5levelBlack         0.2320466  0.3088961   0.751  0.45260
## race.ethnicity.5levelMixed         0.6607383  0.3019878   2.188  0.02877 *
## race.ethnicity.5levelOther         0.4543809  0.3582533   1.268  0.20480
## race.ethnicity.5levelWhite         0.5523724  0.2810193   1.966  0.04946 *
## interview_age                      0.0020522  0.0057065   0.360  0.71915
## bmi                                0.0062779  0.0121787   0.515  0.60626
```

```
## household.income[>=200K]          -0.8510513  0.3045528  -2.794  0.00524 **
## household.income[100K-200K]       -0.8232642  0.2834486  -2.904  0.00371 **
## household.income[12K-16K]          0.0599005  0.3837280   0.156  0.87597
## household.income[16K-25K]          0.1174924  0.3152886   0.373  0.70944
## household.income[25K-35K]         -0.1814417  0.3077157  -0.590  0.55549
## household.income[35K-50K]         -0.0955917  0.2956817  -0.323  0.74650
## household.income[50K-75K]         -0.4746973  0.2827328  -1.679  0.09329 .
## household.income[5K-12K]           0.5004971  0.3310640   1.512  0.13072
## household.income[75K-100K]        -0.7334693  0.2882265  -2.545  0.01100 *
## high.educBachelor                  0.4021299  0.2814493   1.429  0.15320
## high.educHS Diploma/GED            0.0263121  0.2845031   0.092  0.92632
## high.educPost Graduate Degree      0.2478671  0.2856420   0.868  0.38561
## high.educSome College              0.2209309  0.2680104   0.824  0.40983
## demo_race_hispanic1               -0.3200236  0.1257430  -2.545  0.01099 *
## ---
## Signif. codes:  0 '***' 0.001 '**' 0.01 '*' 0.05 '.' 0.1 ' ' 1
##
##
## R-sq.(adj) =  0.0173
## lmer.REML =  10497  Scale est. = 2.6258    n = 2441
```

```
##                                          stdcoef      stdse
## X(Intercept)                         0.000000000 0.00000000
## Xhormone_scr_ert_mean                0.002981775 0.02124621
## Xhormone_sal_end_min_since_midnight  0.011428581 0.02087125
## Xrace.ethnicity.5levelBlack          0.038128954 0.05075654
## Xrace.ethnicity.5levelMixed          0.104266320 0.04765450
## Xrace.ethnicity.5levelOther          0.043637482 0.03440566
## Xrace.ethnicity.5levelWhite          0.124075665 0.06312345
## Xinterview_age                       0.007417661 0.02062553
## Xbmi                                 0.011144673 0.02161979
## Xhousehold.income[>=200K]           -0.133333626 0.04771408
## Xhousehold.income[100K-200K]        -0.184802213 0.06362713
## Xhousehold.income[12K-16K]           0.004032118 0.02583013
## Xhousehold.income[16K-25K]           0.011898298 0.03192885
## Xhousehold.income[25K-35K]          -0.020180018 0.03422427
## Xhousehold.income[35K-50K]          -0.012496524 0.03865390
## Xhousehold.income[50K-75K]          -0.079368167 0.04727219
## Xhousehold.income[5K-12K]            0.043534841 0.02879701
## Xhousehold.income[75K-100K]         -0.123810926 0.04865315
## Xhigh.educBachelor                   0.086831230 0.06077287
## Xhigh.educHS Diploma/GED             0.003415725 0.03693294
## Xhigh.educPost Graduate Degree       0.056705496 0.06534740
## Xhigh.educSome College               0.046254136 0.05611072
## Xdemo_race_hispanic1                -0.060165889 0.02364026
```

1.13 Model: CBCL internalizing factor ~ Testosterone + PDS

Female participants

```
##
## Family: gaussian
## Link function: identity
```

```
##
## Formula:
## cbcl_scr_syn_internal_r ~ hormone_scr_ert_mean + hormone_sal_end_min_since_midnight +
##     PDS_score + race.ethnicity.5level + interview_age + bmi +
##     household.income + high.educ + demo_race_hispanic
##
## Parametric coefficients:
##                                     Estimate Std. Error t value Pr(>|t|)
## (Intercept)                        2.4461488  2.2751447   1.075  0.28242
## hormone_scr_ert_mean              -0.0075078  0.0075461  -0.995  0.31988
## hormone_sal_end_min_since_midnight -0.0001712  0.0006840  -0.250  0.80241
## PDS_score                          0.5144749  0.1808484   2.845  0.00448 **
## race.ethnicity.5levelBlack        -0.8688962  0.8762787  -0.992  0.32151
## race.ethnicity.5levelMixed         0.9521369  0.8519837   1.118  0.26388
## race.ethnicity.5levelOther         1.7594951  0.9703720   1.813  0.06993 .
## race.ethnicity.5levelWhite         1.2221603  0.8013690   1.525  0.12738
## interview_age                      0.0020934  0.0157026   0.133  0.89396
## bmi                                0.0772399  0.0309647   2.494  0.01269 *
## household.income[>=200K]          -2.1872723  0.8133419  -2.689  0.00722 **
## household.income[100K-200K]       -1.6105881  0.7584153  -2.124  0.03381 *
## household.income[12K-16K]          0.3236496  1.0130354   0.319  0.74939
## household.income[16K-25K]          0.7771070  0.8500220   0.914  0.36070
## household.income[25K-35K]         -0.3955209  0.7963693  -0.497  0.61948
## household.income[35K-50K]         -0.2074727  0.7675679  -0.270  0.78696
## household.income[50K-75K]         -0.5760572  0.7650826  -0.753  0.45157
## household.income[5K-12K]           0.1821932  0.8981050   0.203  0.83926
## household.income[75K-100K]        -1.0448255  0.7675549  -1.361  0.17358
## high.educBachelor                  0.4338940  0.7565567   0.574  0.56636
## high.educHS Diploma/GED           -0.5711147  0.7591854  -0.752  0.45197
## high.educPost Graduate Degree      0.6605072  0.7637972   0.865  0.38726
## high.educSome College              0.6642026  0.7124090   0.932  0.35127
## demo_race_hispanic1               -0.2263130  0.3528082  -0.641  0.52129
## ---
## Signif. codes:  0 '***' 0.001 '**' 0.01 '*' 0.05 '.' 0.1 ' ' 1
##
##
## R-sq.(adj) =  0.029
## lmer.REML =  13811  Scale est. = 13.63     n = 2239
```

```
##                                          stdcoef      stdse
## X(Intercept)                         0.000000000 0.00000000
## Xhormone_scr_ert_mean               -0.022418502 0.02253260
## Xhormone_sal_end_min_since_midnight -0.005611828 0.02242386
## XPDS_score                           0.068193536 0.02397141
## Xrace.ethnicity.5levelBlack         -0.053690249 0.05414643
## Xrace.ethnicity.5levelMixed          0.058920391 0.05272269
## Xrace.ethnicity.5levelOther          0.069783449 0.03848599
## Xrace.ethnicity.5levelWhite          0.105878557 0.06942444
## Xinterview_age                       0.002916482 0.02187699
## Xbmi                                 0.057269037 0.02295861
## Xhousehold.income[>=200K]           -0.134554525 0.05003439
## Xhousehold.income[100K-200K]        -0.136666145 0.06435518
## Xhousehold.income[12K-16K]           0.008639292 0.02704131
## Xhousehold.income[16K-25K]           0.028867976 0.03157662
```

```
## Xhousehold.income[25K-35K]          -0.017410843 0.03505621
## Xhousehold.income[35K-50K]          -0.010831367 0.04007182
## Xhousehold.income[50K-75K]          -0.036010552 0.04782692
## Xhousehold.income[5K-12K]            0.005923647 0.02920009
## Xhousehold.income[75K-100K]         -0.069847150 0.05131146
## Xhigh.educBachelor                   0.035781964 0.06239101
## Xhigh.educHS Diploma/GED            -0.027576448 0.03665750
## Xhigh.educPost Graduate Degree       0.058720604 0.06790332
## Xhigh.educSome College               0.052790190 0.05662159
## Xdemo_race_hispanic1                -0.016237268 0.02531291
```

Male participants

```
##
## Family: gaussian
## Link function: identity
##
## Formula:
## cbcl_scr_syn_internal_r ~ hormone_scr_ert_mean + hormone_sal_end_min_since_midnight +
##     PDS_score + race.ethnicity.5level + interview_age + bmi +
##     household.income + high.educ + demo_race_hispanic
##
## Parametric coefficients:
##                                     Estimate Std. Error t value Pr(>|t|)
## (Intercept)                         3.0636351  2.1869470   1.401 0.161380
## hormone_scr_ert_mean               -0.0044763  0.0074680  -0.599 0.548966
## hormone_sal_end_min_since_midnight  0.0004987  0.0006553   0.761 0.446724
## PDS_score                           0.8357031  0.2200347   3.798 0.000149 ***
## race.ethnicity.5levelBlack          0.4523944  0.8053068   0.562 0.574327
## race.ethnicity.5levelMixed          1.9428955  0.7834697   2.480 0.013211 *
## race.ethnicity.5levelOther          1.3419921  0.9311275   1.441 0.149642
## race.ethnicity.5levelWhite          1.6711513  0.7306393   2.287 0.022268 *
## interview_age                      -0.0089172  0.0149493  -0.596 0.550900
## bmi                                 0.0663482  0.0318394   2.084 0.037280 *
## household.income[>=200K]           -2.4154203  0.7958525  -3.035 0.002431 **
## household.income[100K-200K]        -2.3219793  0.7391752  -3.141 0.001702 **
## household.income[12K-16K]          -0.7667771  0.9963472  -0.770 0.441619
## household.income[16K-25K]          -0.2099432  0.8223435  -0.255 0.798514
## household.income[25K-35K]          -1.1112127  0.8000305  -1.389 0.164972
## household.income[35K-50K]          -0.7260782  0.7698569  -0.943 0.345707
## household.income[50K-75K]          -1.4344393  0.7371329  -1.946 0.051774 .
## household.income[5K-12K]            0.8536562  0.8606169   0.992 0.321340
## household.income[75K-100K]         -1.9584540  0.7515481  -2.606 0.009220 **
## high.educBachelor                   0.7531701  0.7345240   1.025 0.305284
## high.educHS Diploma/GED            -0.3000409  0.7423657  -0.404 0.686125
## high.educPost Graduate Degree       0.6162290  0.7450707   0.827 0.408276
## high.educSome College               0.8536852  0.6990881   1.221 0.222152
## demo_race_hispanic1                -0.4194918  0.3339644  -1.256 0.209202
## ---
## Signif. codes:  0 '***' 0.001 '**' 0.01 '*' 0.05 '.' 0.1 ' ' 1
##
##
## R-sq.(adj) =  0.0248
```

```
## lmer.REML =  15092  Scale est. = 14.312    n = 2441

##                                        stdcoef      stdse
## X(Intercept)                         0.000000000 0.00000000
## Xhormone_scr_ert_mean               -0.012768474 0.02130228
## Xhormone_sal_end_min_since_midnight  0.016141241 0.02121024
## XPDS_score                           0.082101420 0.02161672
## Xrace.ethnicity.5levelBlack          0.028534622 0.05079446
## Xrace.ethnicity.5levelMixed          0.117689922 0.04745829
## Xrace.ethnicity.5levelOther          0.049472613 0.03432607
## Xrace.ethnicity.5levelWhite          0.144093935 0.06299890
## Xinterview_age                      -0.012372101 0.02074129
## Xbmi                                 0.045212119 0.02169655
## Xhousehold.income[>=200K]           -0.145261968 0.04786211
## Xhousehold.income[100K-200K]        -0.200079025 0.06369284
## Xhousehold.income[12K-16K]          -0.019812872 0.02574477
## Xhousehold.income[16K-25K]          -0.008161165 0.03196713
## Xhousehold.income[25K-35K]          -0.047441341 0.03415594
## Xhousehold.income[35K-50K]          -0.036435743 0.03863263
## Xhousehold.income[50K-75K]          -0.092063409 0.04730975
## Xhousehold.income[5K-12K]            0.028503201 0.02873562
## Xhousehold.income[75K-100K]         -0.126901184 0.04869777
## Xhigh.educBachelor                   0.062427787 0.06088228
## Xhigh.educHS Diploma/GED            -0.014951425 0.03699304
## Xhigh.educPost Graduate Degree       0.054115741 0.06543030
## Xhigh.educSome College               0.068606811 0.05618254
## Xdemo_race_hispanic1                -0.030273810 0.02410148
```

1.14 Model: CBCL internalizing factor ~ Testosterone + Pubertal category

Female participants

```
##
## Family: gaussian
## Link function: identity
##
## Formula:
## cbcl_scr_syn_internal_r ~ hormone_scr_ert_mean + hormone_sal_end_min_since_midnight +
##     pds_p_ss_category + race.ethnicity.5level + interview_age +
##     bmi + household.income + high.educ + demo_race_hispanic
##
## Parametric coefficients:
##                                      Estimate Std. Error t value Pr(>|t|)
## (Intercept)                         2.9343709  2.3437791   1.252  0.21071
## hormone_scr_ert_mean               -0.0045407  0.0074532  -0.609  0.54244
## hormone_sal_end_min_since_midnight -0.0002610  0.0006847  -0.381  0.70310
## pds_p_ss_categoryEarly              0.8508492  0.3109989   2.736  0.00627 **
## pds_p_ss_categoryLate               0.3787418  0.8060780   0.470  0.63850
## pds_p_ss_categoryMid                0.8417161  0.3159133   2.664  0.00777 **
## race.ethnicity.5levelBlack         -0.7118236  0.8757318  -0.813  0.41640
## race.ethnicity.5levelMixed          1.0356264  0.8522384   1.215  0.22443
## race.ethnicity.5levelOther          1.8996610  0.9695882   1.959  0.05021 .
## race.ethnicity.5levelWhite          1.2900693  0.8016640   1.609  0.10771
```

```
## interview_age                        0.0015350  0.0160024   0.096  0.92359
## bmi                                  0.0656637  0.0321391   2.043  0.04116 *
## household.income[>=200K]            -2.2265379  0.8138117  -2.736  0.00627 **
## household.income[100K-200K]         -1.7126966  0.7587557  -2.257  0.02409 *
## household.income[12K-16K]            0.2467046  1.0141088   0.243  0.80782
## household.income[16K-25K]            0.6676446  0.8512152   0.784  0.43292
## household.income[25K-35K]           -0.4990347  0.7971799  -0.626  0.53138
## household.income[35K-50K]           -0.3267396  0.7686434  -0.425  0.67082
## household.income[50K-75K]           -0.6555266  0.7655112  -0.856  0.39191
## household.income[5K-12K]             0.2039003  0.8987881   0.227  0.82055
## household.income[75K-100K]          -1.1196673  0.7682271  -1.457  0.14513
## high.educBachelor                    0.4671714  0.7573997   0.617  0.53742
## high.educHS Diploma/GED             -0.4859814  0.7593501  -0.640  0.52224
## high.educPost Graduate Degree        0.6997183  0.7647415   0.915  0.36031
## high.educSome College                0.7279044  0.7125354   1.022  0.30710
## demo_race_hispanic1                 -0.2293871  0.3539406  -0.648  0.51699
## ---
## Signif. codes:  0 '***' 0.001 '**' 0.01 '*' 0.05 '.' 0.1 ' ' 1
##
##
## R-sq.(adj) =  0.0282
## lmer.REML =  13807  Scale est. = 13.44     n = 2239
```

```
##                                          stdcoef      stdse
## X(Intercept)                         0.000000000 0.00000000
## Xhormone_scr_ert_mean               -0.013558450 0.02225536
## Xhormone_sal_end_min_since_midnight -0.008556562 0.02244703
## Xpds_p_ss_categoryEarly              0.067706670 0.02474786
## Xpds_p_ss_categoryLate               0.010513095 0.02237507
## Xpds_p_ss_categoryMid                0.076466756 0.02869954
## Xrace.ethnicity.5levelBlack         -0.043984525 0.05411263
## Xrace.ethnicity.5levelMixed          0.064086913 0.05273845
## Xrace.ethnicity.5levelOther          0.075342575 0.03845490
## Xrace.ethnicity.5levelWhite          0.111761664 0.06944999
## Xinterview_age                       0.002138538 0.02229470
## Xbmi                                 0.048685950 0.02382931
## Xhousehold.income[>=200K]           -0.136970026 0.05006329
## Xhousehold.income[100K-200K]        -0.145330539 0.06438407
## Xhousehold.income[12K-16K]           0.006585373 0.02706996
## Xhousehold.income[16K-25K]           0.024801666 0.03162095
## Xhousehold.income[25K-35K]          -0.021967525 0.03509189
## Xhousehold.income[35K-50K]          -0.017057843 0.04012797
## Xhousehold.income[50K-75K]          -0.040978348 0.04785372
## Xhousehold.income[5K-12K]            0.006629409 0.02922229
## Xhousehold.income[75K-100K]         -0.074850362 0.05135640
## Xhigh.educBachelor                   0.038526254 0.06246054
## Xhigh.educHS Diploma/GED            -0.023465761 0.03666545
## Xhigh.educPost Graduate Degree       0.062206561 0.06798728
## Xhigh.educSome College               0.057853147 0.05663164
## Xdemo_race_hispanic1                -0.016457827 0.02539416
```

Male participants

```
##
```

```
## Family: gaussian
## Link function: identity
##
## Formula:
## cbcl_scr_syn_internal_r ~ hormone_scr_ert_mean + hormone_sal_end_min_since_midnight +
##     pds_p_ss_category + race.ethnicity.5level + interview_age +
##     bmi + household.income + high.educ + demo_race_hispanic
##
## Parametric coefficients:
##                                      Estimate Std. Error t value Pr(>|t|)
## (Intercept)                         3.4992770  2.2000254   1.591  0.11184
## hormone_scr_ert_mean               -0.0033176  0.0074640  -0.444  0.65674
## hormone_sal_end_min_since_midnight  0.0005248  0.0006556   0.801  0.42347
## pds_p_ss_categoryEarly              0.7163539  0.2639980   2.713  0.00671 **
## pds_p_ss_categoryLate               1.7078214  1.7775241   0.961  0.33676
## pds_p_ss_categoryMid                0.8707815  0.5449396   1.598  0.11019
## race.ethnicity.5levelBlack          0.4933832  0.8068561   0.611  0.54093
## race.ethnicity.5levelMixed          1.9829017  0.7846283   2.527  0.01156 *
## race.ethnicity.5levelOther          1.4094483  0.9328849   1.511  0.13096
## race.ethnicity.5levelWhite          1.7129103  0.7317030   2.341  0.01931 *
## interview_age                      -0.0071998  0.0149660  -0.481  0.63050
## bmi                                 0.0758043  0.0316847   2.392  0.01681 *
## household.income[>=200K]           -2.5217285  0.7958802  -3.168  0.00155 **
## household.income[100K-200K]        -2.4089690  0.7397975  -3.256  0.00114 **
## household.income[12K-16K]          -0.8313515  0.9979709  -0.833  0.40490
## household.income[16K-25K]          -0.3135065  0.8230449  -0.381  0.70330
## household.income[25K-35K]          -1.1855573  0.8007384  -1.481  0.13885
## household.income[35K-50K]          -0.7957705  0.7709613  -1.032  0.30209
## household.income[50K-75K]          -1.5140901  0.7378099  -2.052  0.04026 *
## household.income[5K-12K]            0.7348528  0.8610535   0.853  0.39350
## household.income[75K-100K]         -2.0483107  0.7520507  -2.724  0.00650 **
## high.educBachelor                   0.8211136  0.7358735   1.116  0.26460
## high.educHS Diploma/GED            -0.1744831  0.7428657  -0.235  0.81432
## high.educPost Graduate Degree       0.7031304  0.7463564   0.942  0.34624
## high.educSome College               0.9465478  0.7001704   1.352  0.17654
## demo_race_hispanic1                -0.4633104  0.3346641  -1.384  0.16636
## ---
## Signif. codes:  0 '***' 0.001 '**' 0.01 '*' 0.05 '.' 0.1 ' ' 1
##
##
## R-sq.(adj) =  0.0225
## lmer.REML =  15093  Scale est. = 14.593    n = 2441
```

```
##                                          stdcoef      stdse
## X(Intercept)                         0.000000000 0.00000000
## Xhormone_scr_ert_mean               -0.009463222 0.02129086
## Xhormone_sal_end_min_since_midnight  0.016987315 0.02121972
## Xpds_p_ss_categoryEarly              0.056823747 0.02094126
## Xpds_p_ss_categoryLate               0.019069758 0.01984807
## Xpds_p_ss_categoryMid                0.033990900 0.02127168
## Xrace.ethnicity.5levelBlack          0.031119980 0.05089218
## Xrace.ethnicity.5levelMixed          0.120113279 0.04752847
## Xrace.ethnicity.5levelOther          0.051959389 0.03439085
## Xrace.ethnicity.5levelWhite          0.147694575 0.06309061
```

```
## Xinterview_age                          -0.009989352 0.02076438
## Xbmi                                     0.051655880 0.02159113
## Xhousehold.income[>=200K]               -0.151655282 0.04786377
## Xhousehold.income[100K-200K]            -0.207574711 0.06374646
## Xhousehold.income[12K-16K]              -0.021481422 0.02578673
## Xhousehold.income[16K-25K]              -0.012187003 0.03199439
## Xhousehold.income[25K-35K]              -0.050615359 0.03418617
## Xhousehold.income[35K-50K]              -0.039933011 0.03868805
## Xhousehold.income[50K-75K]              -0.097175463 0.04735320
## Xhousehold.income[5K-12K]                0.024536408 0.02875020
## Xhousehold.income[75K-100K]             -0.132723597 0.04873034
## Xhigh.educBachelor                       0.068059405 0.06099413
## Xhigh.educHS Diploma/GED                -0.008694719 0.03701795
## Xhigh.educPost Graduate Degree           0.061747209 0.06554321
## Xhigh.educSome College                   0.076069755 0.05626952
## Xdemo_race_hispanic1                    -0.033436100 0.02415198
```

1.15 Model: CBCL Anxious-Depressed ~ Testosterone + PDS

Female participants

```
##
## Family: gaussian
## Link function: identity
##
## Formula:
## cbcl_scr_syn_anxdep_r ~ hormone_scr_ert_mean + hormone_sal_end_min_since_midnight +
##     PDS_score + race.ethnicity.5level + interview_age + bmi +
##     household.income + high.educ + demo_race_hispanic
##
## Parametric coefficients:
##                                      Estimate Std. Error t value Pr(>|t|)
## (Intercept)                         1.3491718  1.3092276   1.031   0.3029
## hormone_scr_ert_mean                0.0002082  0.0043502   0.048   0.9618
## hormone_sal_end_min_since_midnight -0.0002412  0.0003927  -0.614   0.5391
## PDS_score                           0.1608238  0.1040954   1.545   0.1225
## race.ethnicity.5levelBlack         -0.3112513  0.5020279  -0.620   0.5353
## race.ethnicity.5levelMixed          0.5093285  0.4881697   1.043   0.2969
## race.ethnicity.5levelOther          0.7183732  0.5565194   1.291   0.1969
## race.ethnicity.5levelWhite          0.7652230  0.4591182   1.667   0.0957 .
## interview_age                       0.0009972  0.0090517   0.110   0.9123
## bmi                                 0.0183705  0.0178197   1.031   0.3027
## household.income[>=200K]           -1.1218565  0.4661361  -2.407   0.0162 *
## household.income[100K-200K]        -0.6098477  0.4348650  -1.402   0.1609
## household.income[12K-16K]           0.1542107  0.5811459   0.265   0.7908
## household.income[16K-25K]           0.5736075  0.4877287   1.176   0.2397
## household.income[25K-35K]          -0.0139938  0.4569201  -0.031   0.9756
## household.income[35K-50K]           0.1157897  0.4401511   0.263   0.7925
## household.income[50K-75K]          -0.0430719  0.4387043  -0.098   0.9218
## household.income[5K-12K]            0.0483078  0.5147670   0.094   0.9252
## household.income[75K-100K]         -0.3591740  0.4400595  -0.816   0.4145
## high.educBachelor                   0.2922563  0.4340477   0.673   0.5008
## high.educHS Diploma/GED            -0.3011563  0.4360365  -0.691   0.4898
```

```
## high.educPost Graduate Degree       0.6397212  0.4381818   1.460   0.1444
## high.educSome College               0.4392331  0.4087898   1.074   0.2827
## demo_race_hispanic1                -0.0970572  0.2019475  -0.481   0.6308
## ---
## Signif. codes:  0 '***' 0.001 '**' 0.01 '*' 0.05 '.' 0.1 ' ' 1
##
##
## R-sq.(adj) =  0.0195
## lmer.REML =  11365  Scale est. = 5.1945    n = 2239
```

```
##                                          stdcoef      stdse
## X(Intercept)                         0.000000000 0.00000000
## Xhormone_scr_ert_mean                0.001086665 0.02271019
## Xhormone_sal_end_min_since_midnight -0.013825024 0.02250906
## XPDS_score                           0.037269431 0.02412314
## Xrace.ethnicity.5levelBlack         -0.033624995 0.05423491
## Xrace.ethnicity.5levelMixed          0.055104578 0.05281540
## Xrace.ethnicity.5levelOther          0.049812463 0.03858941
## Xrace.ethnicity.5levelWhite          0.115902113 0.06953891
## Xinterview_age                       0.002428963 0.02204800
## Xbmi                                 0.023813524 0.02309948
## Xhousehold.income[>=200K]           -0.120658046 0.05013392
## Xhousehold.income[100K-200K]        -0.090473495 0.06451407
## Xhousehold.income[12K-16K]           0.007196828 0.02712138
## Xhousehold.income[16K-25K]           0.037254077 0.03167651
## Xhousehold.income[25K-35K]          -0.001076983 0.03516529
## Xhousehold.income[35K-50K]           0.010568557 0.04017424
## Xhousehold.income[50K-75K]          -0.004707409 0.04794679
## Xhousehold.income[5K-12K]            0.002745985 0.02926114
## Xhousehold.income[75K-100K]         -0.041979117 0.05143277
## Xhigh.educBachelor                   0.042137426 0.06258086
## Xhigh.educHS Diploma/GED            -0.025423214 0.03680962
## Xhigh.educPost Graduate Degree       0.099432249 0.06810686
## Xhigh.educSome College               0.061033914 0.05680364
## Xdemo_race_hispanic1                -0.012174602 0.02533177
```

Male participants

```
##
## Family: gaussian
## Link function: identity
##
## Formula:
## cbcl_scr_syn_anxdep_r ~ hormone_scr_ert_mean + hormone_sal_end_min_since_midnight +
##     PDS_score + race.ethnicity.5level + interview_age + bmi +
##     household.income + high.educ + demo_race_hispanic
##
## Parametric coefficients:
##                                      Estimate Std. Error t value Pr(>|t|)
## (Intercept)                         1.939e+00  1.235e+00   1.570  0.11656
## hormone_scr_ert_mean               -3.019e-03  4.223e-03  -0.715  0.47471
## hormone_sal_end_min_since_midnight -5.345e-05  3.711e-04  -0.144  0.88547
## PDS_score                           4.849e-01  1.242e-01   3.903 9.77e-05 ***
```

```
## race.ethnicity.5levelBlack          4.366e-01  4.542e-01   0.961  0.33653
## race.ethnicity.5levelMixed          1.165e+00  4.423e-01   2.633  0.00852 **
## race.ethnicity.5levelOther          9.550e-01  5.240e-01   1.822  0.06850 .
## race.ethnicity.5levelWhite          1.139e+00  4.127e-01   2.760  0.00582 **
## interview_age                      -7.991e-03  8.450e-03  -0.946  0.34439
## bmi                                 1.615e-02  1.796e-02   0.899  0.36856
## household.income[>=200K]           -9.312e-01  4.457e-01  -2.089  0.03678 *
## household.income[100K-200K]        -8.725e-01  4.141e-01  -2.107  0.03524 *
## household.income[12K-16K]          -1.844e-01  5.603e-01  -0.329  0.74207
## household.income[16K-25K]           1.080e-01  4.603e-01   0.235  0.81459
## household.income[25K-35K]          -2.616e-01  4.483e-01  -0.584  0.55958
## household.income[35K-50K]          -1.254e-01  4.314e-01  -0.291  0.77140
## household.income[50K-75K]          -6.104e-01  4.129e-01  -1.478  0.13944
## household.income[5K-12K]            2.124e-01  4.829e-01   0.440  0.66008
## household.income[75K-100K]         -7.112e-01  4.210e-01  -1.689  0.09132 .
## high.educBachelor                   3.957e-01  4.112e-01   0.962  0.33603
## high.educHS Diploma/GED            -2.879e-01  4.157e-01  -0.693  0.48865
## high.educPost Graduate Degree       3.745e-01  4.171e-01   0.898  0.36938
## high.educSome College               3.007e-01  3.916e-01   0.768  0.44260
## demo_race_hispanic1                -8.985e-02  1.876e-01  -0.479  0.63205
## ---
## Signif. codes:  0 '***' 0.001 '**' 0.01 '*' 0.05 '.' 0.1 ' ' 1
##
##
## R-sq.(adj) =  0.0126
## lmer.REML =  12336  Scale est. = 6.3224    n = 2441

##                                          stdcoef      stdse
## X(Intercept)                         0.000000000 0.00000000
## Xhormone_scr_ert_mean               -0.015396622 0.02153526
## Xhormone_sal_end_min_since_midnight -0.003092948 0.02147168
## XPDS_score                           0.085162657 0.02182069
## Xrace.ethnicity.5levelBlack          0.049234283 0.05121985
## Xrace.ethnicity.5levelMixed          0.126121295 0.04790079
## Xrace.ethnicity.5levelOther          0.062942740 0.03453654
## Xrace.ethnicity.5levelWhite          0.175613674 0.06362234
## Xinterview_age                      -0.019821500 0.02095919
## Xbmi                                 0.019676159 0.02187859
## Xhousehold.income[>=200K]           -0.100114558 0.04791703
## Xhousehold.income[100K-200K]        -0.134410532 0.06379880
## Xhousehold.income[12K-16K]          -0.008519946 0.02588434
## Xhousehold.income[16K-25K]           0.007503162 0.03199122
## Xhousehold.income[25K-35K]          -0.019969026 0.03422028
## Xhousehold.income[35K-50K]          -0.011246500 0.03870448
## Xhousehold.income[50K-75K]          -0.070036232 0.04737445
## Xhousehold.income[5K-12K]            0.012679252 0.02882569
## Xhousehold.income[75K-100K]         -0.082382103 0.04877113
## Xhigh.educBachelor                   0.058637711 0.06093888
## Xhigh.educHS Diploma/GED            -0.025648457 0.03703381
## Xhigh.educPost Graduate Degree       0.058796914 0.06548992
## Xhigh.educSome College               0.043203146 0.05625913
## Xdemo_race_hispanic1                -0.011592564 0.02420609
```

1.16 Model: CBCL Anxious-Depressed ~ Testosterone + Pubertal category

Female participants

```
##
## Family: gaussian
## Link function: identity
##
## Formula:
## cbcl_scr_syn_anxdep_r ~ hormone_scr_ert_mean + hormone_sal_end_min_since_midnight +
##     pds_p_ss_category + race.ethnicity.5level + interview_age +
##     bmi + household.income + high.educ + demo_race_hispanic
##
## Parametric coefficients:
##                                     Estimate Std. Error t value Pr(>|t|)
## (Intercept)                        1.4174326  1.3483710   1.051   0.2933
## hormone_scr_ert_mean               0.0015165  0.0042948   0.353   0.7240
## hormone_sal_end_min_since_midnight -0.0002695  0.0003929  -0.686   0.4929
## pds_p_ss_categoryEarly             0.4274732  0.1793089   2.384   0.0172 *
## pds_p_ss_categoryLate             -0.0561593  0.4653663  -0.121   0.9040
## pds_p_ss_categoryMid               0.2698784  0.1816560   1.486   0.1375
## race.ethnicity.5levelBlack        -0.2333945  0.5013054  -0.466   0.6416
## race.ethnicity.5levelMixed         0.5496745  0.4879409   1.127   0.2601
## race.ethnicity.5levelOther         0.7757835  0.5556627   1.396   0.1628
## race.ethnicity.5levelWhite         0.7940358  0.4589265   1.730   0.0837 .
## interview_age                      0.0011447  0.0092236   0.124   0.9012
## bmi                                0.0144347  0.0184864   0.781   0.4350
## household.income[>=200K]          -1.1406378  0.4660613  -2.447   0.0145 *
## household.income[100K-200K]       -0.6568266  0.4347502  -1.511   0.1310
## household.income[12K-16K]          0.1097854  0.5813576   0.189   0.8502
## household.income[16K-25K]          0.5208982  0.4880814   1.067   0.2860
## household.income[25K-35K]         -0.0582331  0.4570789  -0.127   0.8986
## household.income[35K-50K]          0.0623067  0.4404661   0.141   0.8875
## household.income[50K-75K]         -0.0790980  0.4386430  -0.180   0.8569
## household.income[5K-12K]           0.0604999  0.5147874   0.118   0.9065
## household.income[75K-100K]        -0.3910966  0.4401312  -0.889   0.3743
## high.educBachelor                  0.2971141  0.4342525   0.684   0.4939
## high.educHS Diploma/GED           -0.2650141  0.4358686  -0.608   0.5432
## high.educPost Graduate Degree      0.6486419  0.4384436   1.479   0.1392
## high.educSome College              0.4660503  0.4085898   1.141   0.2541
## demo_race_hispanic1               -0.0947171  0.2024227  -0.468   0.6399
## ---
## Signif. codes:  0 '***' 0.001 '**' 0.01 '*' 0.05 '.' 0.1 ' ' 1
##
##
## R-sq.(adj) =  0.0201
## lmer.REML =  11361  Scale est. = 5.1526    n = 2239
```

```
##                                          stdcoef      stdse
## X(Intercept)                         0.000000000 0.00000000
## Xhormone_scr_ert_mean                0.007916968 0.02242097
## Xhormone_sal_end_min_since_midnight -0.015444592 0.02251803
## Xpds_p_ss_categoryEarly              0.059471826 0.02494619
## Xpds_p_ss_categoryLate              -0.002725414 0.02258426
```

```
## Xpds_p_ss_categoryMid               0.042864595 0.02885230
## Xrace.ethnicity.5levelBlack        -0.025213999 0.05415685
## Xrace.ethnicity.5levelMixed         0.059469640 0.05279065
## Xrace.ethnicity.5levelOther         0.053793331 0.03853001
## Xrace.ethnicity.5levelWhite         0.120266150 0.06950987
## Xinterview_age                      0.002788334 0.02246684
## Xbmi                                0.018711515 0.02396367
## Xhousehold.income[>=200K]          -0.122678007 0.05012588
## Xhousehold.income[100K-200K]       -0.097443009 0.06449704
## Xhousehold.income[12K-16K]          0.005123551 0.02713126
## Xhousehold.income[16K-25K]          0.033830773 0.03169941
## Xhousehold.income[25K-35K]         -0.004481708 0.03517751
## Xhousehold.income[35K-50K]          0.005686967 0.04020298
## Xhousehold.income[50K-75K]         -0.008644768 0.04794009
## Xhousehold.income[5K-12K]           0.003439025 0.02926230
## Xhousehold.income[75K-100K]        -0.045710129 0.05144114
## Xhigh.educBachelor                  0.042837818 0.06261039
## Xhigh.educHS Diploma/GED           -0.022372138 0.03679545
## Xhigh.educPost Graduate Degree      0.100818793 0.06814755
## Xhigh.educSome College              0.064760313 0.05677586
## Xdemo_race_hispanic1               -0.011881064 0.02539137
```

Male participants

```
##
## Family: gaussian
## Link function: identity
##
## Formula:
## cbcl_scr_syn_anxdep_r ~ hormone_scr_ert_mean + hormone_sal_end_min_since_midnight +
##     pds_p_ss_category + race.ethnicity.5level + interview_age +
##     bmi + household.income + high.educ + demo_race_hispanic
##
## Parametric coefficients:
##                                     Estimate Std. Error t value Pr(>|t|)
## (Intercept)                        2.189e+00  1.242e+00   1.762  0.07821 .
## hormone_scr_ert_mean              -2.289e-03  4.220e-03  -0.542  0.58764
## hormone_sal_end_min_since_midnight -3.746e-05  3.712e-04  -0.101  0.91964
## pds_p_ss_categoryEarly             4.495e-01  1.493e-01   3.011  0.00263 **
## pds_p_ss_categoryLate              1.035e+00  1.012e+00   1.023  0.30663
## pds_p_ss_categoryMid               3.614e-01  3.068e-01   1.178  0.23892
## race.ethnicity.5levelBlack         4.704e-01  4.551e-01   1.034  0.30143
## race.ethnicity.5levelMixed         1.194e+00  4.430e-01   2.696  0.00706 **
## race.ethnicity.5levelOther         1.006e+00  5.250e-01   1.917  0.05535 .
## race.ethnicity.5levelWhite         1.168e+00  4.133e-01   2.826  0.00475 **
## interview_age                     -6.978e-03  8.456e-03  -0.825  0.40933
## bmi                                2.188e-02  1.787e-02   1.224  0.22091
## household.income[>=200K]          -1.001e+00  4.458e-01  -2.245  0.02483 *
## household.income[100K-200K]       -9.327e-01  4.146e-01  -2.250  0.02454 *
## household.income[12K-16K]         -2.325e-01  5.613e-01  -0.414  0.67881
## household.income[16K-25K]          3.449e-02  4.607e-01   0.075  0.94034
## household.income[25K-35K]         -3.101e-01  4.488e-01  -0.691  0.48965
## household.income[35K-50K]         -1.779e-01  4.321e-01  -0.412  0.68054
```

```
## household.income[50K-75K]          -6.662e-01  4.133e-01  -1.612  0.10717
## household.income[5K-12K]            1.389e-01  4.832e-01   0.287  0.77386
## household.income[75K-100K]         -7.726e-01  4.214e-01  -1.834  0.06683 .
## high.educBachelor                   4.301e-01  4.120e-01   1.044  0.29669
## high.educHS Diploma/GED            -2.118e-01  4.160e-01  -0.509  0.61071
## high.educPost Graduate Degree       4.198e-01  4.179e-01   1.004  0.31526
## high.educSome College               3.516e-01  3.922e-01   0.896  0.37013
## demo_race_hispanic1                -1.147e-01  1.880e-01  -0.610  0.54203
## ---
## Signif. codes:  0 '***' 0.001 '**' 0.01 '*' 0.05 '.' 0.1 ' ' 1
##
##
## R-sq.(adj) =  0.0101
## lmer.REML =  12340  Scale est. = 6.4195    n = 2441
```

```
##                                          stdcoef      stdse
## X(Intercept)                         0.000000000 0.00000000
## Xhormone_scr_ert_mean               -0.011671799 0.02152129
## Xhormone_sal_end_min_since_midnight -0.002167406 0.02148060
## Xpds_p_ss_categoryEarly              0.063746389 0.02117376
## Xpds_p_ss_categoryLate               0.020655419 0.02020040
## Xpds_p_ss_categoryMid                0.025219386 0.02140889
## Xrace.ethnicity.5levelBlack          0.053040525 0.05131663
## Xrace.ethnicity.5levelMixed          0.129342182 0.04796941
## Xrace.ethnicity.5levelOther          0.066333927 0.03460166
## Xrace.ethnicity.5levelWhite          0.180052052 0.06371455
## Xinterview_age                      -0.017309025 0.02097513
## Xbmi                                 0.026651667 0.02176660
## Xhousehold.income[>=200K]           -0.107616879 0.04792560
## Xhousehold.income[100K-200K]        -0.143686442 0.06386303
## Xhousehold.income[12K-16K]          -0.010738024 0.02592824
## Xhousehold.income[16K-25K]           0.002396719 0.03202035
## Xhousehold.income[25K-35K]          -0.023667771 0.03425276
## Xhousehold.income[35K-50K]          -0.015962753 0.03876590
## Xhousehold.income[50K-75K]          -0.076436131 0.04742708
## Xhousehold.income[5K-12K]            0.008288625 0.02884443
## Xhousehold.income[75K-100K]         -0.089501903 0.04881163
## Xhigh.educBachelor                   0.063728079 0.06105457
## Xhigh.educHS Diploma/GED            -0.018869390 0.03706222
## Xhigh.educPost Graduate Degree       0.065900382 0.06560748
## Xhigh.educSome College               0.050514655 0.05635283
## Xdemo_race_hispanic1                -0.014795822 0.02426217
```

1.17 Model: CBCL Withdrawn-Depressed ~ Testosterone + PDS

Female participants

```
##
## Family: gaussian
## Link function: identity
##
## Formula:
## cbcl_scr_syn_withdep_r ~ hormone_scr_ert_mean + hormone_sal_end_min_since_midnight +
```

```
##     PDS_score + race.ethnicity.5level + interview_age + bmi +
##     household.income + high.educ + demo_race_hispanic
##
## Parametric coefficients:
##                                    Estimate Std. Error t value Pr(>|t|)
## (Intercept)                       5.900e-01  6.322e-01   0.933  0.35079
## hormone_scr_ert_mean             -3.098e-03  2.106e-03  -1.471  0.14140
## hormone_sal_end_min_since_midnight  2.794e-05  1.863e-04   0.150  0.88077
## PDS_score                         1.571e-01  5.040e-02   3.117  0.00185 **
## race.ethnicity.5levelBlack       -1.272e-01  2.405e-01  -0.529  0.59689
## race.ethnicity.5levelMixed        1.737e-01  2.341e-01   0.742  0.45819
## race.ethnicity.5levelOther        3.417e-01  2.678e-01   1.276  0.20206
## race.ethnicity.5levelWhite        2.414e-01  2.197e-01   1.099  0.27196
## interview_age                    -1.015e-03  4.384e-03  -0.232  0.81686
## bmi                               2.444e-02  8.625e-03   2.834  0.00464 **
## household.income[>=200K]         -6.768e-01  2.244e-01  -3.017  0.00258 **
## household.income[100K-200K]      -5.990e-01  2.097e-01  -2.857  0.00432 **
## household.income[12K-16K]        -3.058e-02  2.807e-01  -0.109  0.91326
## household.income[16K-25K]        -3.312e-02  2.357e-01  -0.140  0.88829
## household.income[25K-35K]        -1.957e-01  2.207e-01  -0.887  0.37541
## household.income[35K-50K]        -3.046e-01  2.125e-01  -1.433  0.15196
## household.income[50K-75K]        -3.857e-01  2.117e-01  -1.822  0.06858 .
## household.income[5K-12K]          2.033e-01  2.485e-01   0.818  0.41323
## household.income[75K-100K]       -5.095e-01  2.122e-01  -2.401  0.01643 *
## high.educBachelor                 5.332e-02  2.094e-01   0.255  0.79898
## high.educHS Diploma/GED          -1.409e-01  2.108e-01  -0.669  0.50382
## high.educPost Graduate Degree     3.429e-02  2.114e-01   0.162  0.87115
## high.educSome College             1.004e-01  1.973e-01   0.509  0.61082
## demo_race_hispanic1              -1.373e-02  9.557e-02  -0.144  0.88581
## ---
## Signif. codes:  0 '***' 0.001 '**' 0.01 '*' 0.05 '.' 0.1 ' ' 1
##
##
## R-sq.(adj) =  0.0282
## lmer.REML = 8158.8  Scale est. = 1.4578    n = 2239
```

```
##                                         stdcoef      stdse
## X(Intercept)                        0.000000000 0.00000000
## Xhormone_scr_ert_mean              -0.033477147 0.02275636
## Xhormone_sal_end_min_since_midnight  0.003314838 0.02209792
## XPDS_score                          0.075375815 0.02417942
## Xrace.ethnicity.5levelBlack        -0.028455283 0.05379501
## Xrace.ethnicity.5levelMixed         0.038909230 0.05244136
## Xrace.ethnicity.5levelOther         0.049056634 0.03844342
## Xrace.ethnicity.5levelWhite         0.075696345 0.06888730
## Xinterview_age                     -0.005120291 0.02210661
## Xbmi                                0.065583874 0.02314480
## Xhousehold.income[>=200K]          -0.150693062 0.04995333
## Xhousehold.income[100K-200K]       -0.183973426 0.06440364
## Xhousehold.income[12K-16K]         -0.002954493 0.02711989
## Xhousehold.income[16K-25K]         -0.004452720 0.03169633
## Xhousehold.income[25K-35K]         -0.031172131 0.03516050
## Xhousehold.income[35K-50K]         -0.057546249 0.04015409
## Xhousehold.income[50K-75K]         -0.087270016 0.04789705
```

```
## Xhousehold.income[5K-12K]            0.023927959 0.02923822
## Xhousehold.income[75K-100K]         -0.123284405 0.05134784
## Xhigh.educBachelor                   0.015915981 0.06249027
## Xhigh.educHS Diploma/GED            -0.024629386 0.03683764
## Xhigh.educPost Graduate Degree       0.011032277 0.06800824
## Xhigh.educSome College               0.028881797 0.05674534
## Xdemo_race_hispanic1                -0.003564320 0.02481703
```

Male participants

```
##
## Family: gaussian
## Link function: identity
##
## Formula:
## cbcl_scr_syn_withdep_r ~ hormone_scr_ert_mean + hormone_sal_end_min_since_midnight +
##     PDS_score + race.ethnicity.5level + interview_age + bmi +
##     household.income + high.educ + demo_race_hispanic
##
## Parametric coefficients:
##                                      Estimate Std. Error t value Pr(>|t|)
## (Intercept)                         9.616e-01  6.765e-01   1.422 0.155274
## hormone_scr_ert_mean               -7.076e-04  2.301e-03  -0.308 0.758418
## hormone_sal_end_min_since_midnight -2.686e-05  1.946e-04  -0.138 0.890260
## PDS_score                           1.646e-01  6.835e-02   2.408 0.016121 *
## race.ethnicity.5levelBlack          1.102e-01  2.487e-01   0.443 0.657702
## race.ethnicity.5levelMixed          4.617e-01  2.430e-01   1.900 0.057546 .
## race.ethnicity.5levelOther          2.132e-01  2.877e-01   0.741 0.458746
## race.ethnicity.5levelWhite          3.529e-01  2.256e-01   1.564 0.117913
## interview_age                      -2.293e-03  4.638e-03  -0.494 0.621134
## bmi                                 2.201e-02  9.893e-03   2.225 0.026151 *
## household.income[>=200K]           -8.753e-01  2.443e-01  -3.582 0.000347 ***
## household.income[100K-200K]        -8.588e-01  2.278e-01  -3.771 0.000167 ***
## household.income[12K-16K]          -3.679e-01  3.091e-01  -1.190 0.234106
## household.income[16K-25K]          -1.187e-01  2.534e-01  -0.468 0.639583
## household.income[25K-35K]          -3.328e-01  2.472e-01  -1.346 0.178349
## household.income[35K-50K]          -3.049e-01  2.377e-01  -1.283 0.199757
## household.income[50K-75K]          -4.259e-01  2.271e-01  -1.875 0.060903 .
## household.income[5K-12K]            3.756e-01  2.661e-01   1.411 0.158256
## household.income[75K-100K]         -7.058e-01  2.317e-01  -3.046 0.002347 **
## high.educBachelor                   1.264e-01  2.250e-01   0.562 0.574360
## high.educHS Diploma/GED             7.909e-03  2.280e-01   0.035 0.972324
## high.educPost Graduate Degree       6.268e-02  2.285e-01   0.274 0.783869
## high.educSome College               2.033e-01  2.147e-01   0.947 0.343802
## demo_race_hispanic1                -2.884e-01  9.870e-02  -2.922 0.003509 **
## ---
## Signif. codes:  0 '***' 0.001 '**' 0.01 '*' 0.05 '.' 0.1 ' ' 1
##
##
## R-sq.(adj) =  0.0353
## lmer.REML =   9466  Scale est. = 2.1691    n = 2441
```

```
##                                          stdcoef      stdse
```

```
## X(Intercept)                          0.000000000 0.00000000
## Xhormone_scr_ert_mean                -0.006475343 0.02105164
## Xhormone_sal_end_min_since_midnight -0.002788968 0.02021146
## XPDS_score                            0.051868550 0.02154142
## Xrace.ethnicity.5levelBlack           0.022299964 0.05032227
## Xrace.ethnicity.5levelMixed           0.089720166 0.04721998
## Xrace.ethnicity.5levelOther           0.025211050 0.03402158
## Xrace.ethnicity.5levelWhite           0.097615618 0.06240801
## Xinterview_age                       -0.010205186 0.02064540
## Xbmi                                  0.048126560 0.02162664
## Xhousehold.income[>=200K]            -0.168874781 0.04713996
## Xhousehold.income[100K-200K]         -0.237404348 0.06295871
## Xhousehold.income[12K-16K]           -0.030493609 0.02562174
## Xhousehold.income[16K-25K]           -0.014799243 0.03159925
## Xhousehold.income[25K-35K]           -0.045578516 0.03385601
## Xhousehold.income[35K-50K]           -0.049079603 0.03826590
## Xhousehold.income[50K-75K]           -0.087683883 0.04676296
## Xhousehold.income[5K-12K]             0.040228222 0.02850237
## Xhousehold.income[75K-100K]          -0.146712266 0.04817164
## Xhigh.educBachelor                    0.033606500 0.05982798
## Xhigh.educHS Diploma/GED              0.001264395 0.03644115
## Xhigh.educPost Graduate Degree        0.017659220 0.06437659
## Xhigh.educSome College                0.052403391 0.05534408
## Xdemo_race_hispanic1                 -0.066770992 0.02285050
```

1.18 Model: CBCL Withdrawn-Depressed ~ Testosterone + Pubertal category

Female participants

```
##
## Family: gaussian
## Link function: identity
##
## Formula:
## cbcl_scr_syn_withdep_r ~ hormone_scr_ert_mean + hormone_sal_end_min_since_midnight +
##     pds_p_ss_category + race.ethnicity.5level + interview_age +
##     bmi + household.income + high.educ + demo_race_hispanic
##
## Parametric coefficients:
##                                       Estimate Std. Error t value Pr(>|t|)
## (Intercept)                          8.482e-01  6.521e-01   1.301  0.19351
## hormone_scr_ert_mean                -2.489e-03  2.081e-03  -1.196  0.23179
## hormone_sal_end_min_since_midnight  2.186e-06  1.866e-04   0.012  0.99065
## pds_p_ss_categoryEarly               1.858e-01  8.720e-02   2.131  0.03321 *
## pds_p_ss_categoryLate                3.959e-01  2.265e-01   1.748  0.08060 .
## pds_p_ss_categoryMid                 2.494e-01  8.798e-02   2.835  0.00462 **
## race.ethnicity.5levelBlack          -9.481e-02  2.405e-01  -0.394  0.69341
## race.ethnicity.5levelMixed           1.943e-01  2.343e-01   0.829  0.40699
## race.ethnicity.5levelOther           3.723e-01  2.677e-01   1.391  0.16451
## race.ethnicity.5levelWhite           2.609e-01  2.199e-01   1.186  0.23556
## interview_age                       -1.859e-03  4.473e-03  -0.416  0.67768
## bmi                                  2.071e-02  8.957e-03   2.312  0.02088 *
## household.income[>=200K]            -6.820e-01  2.246e-01  -3.037  0.00242 **
```

```
## household.income[100K-200K]        -6.190e-01  2.099e-01  -2.949  0.00322 **
## household.income[12K-16K]          -4.658e-02  2.811e-01  -0.166  0.86841
## household.income[16K-25K]          -5.411e-02  2.362e-01  -0.229  0.81882
## household.income[25K-35K]          -2.213e-01  2.210e-01  -1.001  0.31678
## household.income[35K-50K]          -3.320e-01  2.129e-01  -1.559  0.11907
## household.income[50K-75K]          -4.033e-01  2.119e-01  -1.903  0.05718 .
## household.income[5K-12K]            2.024e-01  2.488e-01   0.814  0.41597
## household.income[75K-100K]         -5.240e-01  2.125e-01  -2.466  0.01374 *
## high.educBachelor                   6.139e-02  2.097e-01   0.293  0.76977
## high.educHS Diploma/GED            -1.208e-01  2.110e-01  -0.573  0.56680
## high.educPost Graduate Degree       4.309e-02  2.118e-01   0.204  0.83876
## high.educSome College               1.143e-01  1.974e-01   0.579  0.56250
## demo_race_hispanic1                -2.331e-02  9.588e-02  -0.243  0.80791
## ---
## Signif. codes:  0 '***' 0.001 '**' 0.01 '*' 0.05 '.' 0.1 ' ' 1
##
##
## R-sq.(adj) =  0.0268
## lmer.REML = 8162.8  Scale est. = 1.4412    n = 2239
```

```
##                                          stdcoef      stdse
## X(Intercept)                        0.0000000000 0.00000000
## Xhormone_scr_ert_mean              -0.0269006826 0.02249058
## Xhormone_sal_end_min_since_midnight  0.0002593423 0.02213546
## Xpds_p_ss_categoryEarly             0.0535125911 0.02511320
## Xpds_p_ss_categoryLate              0.0397787103 0.02275645
## Xpds_p_ss_categoryMid               0.0820174233 0.02892643
## Xrace.ethnicity.5levelBlack        -0.0212027382 0.05377581
## Xrace.ethnicity.5levelMixed         0.0435237940 0.05247824
## Xrace.ethnicity.5levelOther         0.0534376216 0.03843030
## Xrace.ethnicity.5levelWhite         0.0817935474 0.06893782
## Xinterview_age                     -0.0093763539 0.02255634
## Xbmi                                0.0555666228 0.02403573
## Xhousehold.income[>=200K]          -0.1518511959 0.05000676
## Xhousehold.income[100K-200K]       -0.1900927970 0.06446537
## Xhousehold.income[12K-16K]         -0.0045005286 0.02716115
## Xhousehold.income[16K-25K]         -0.0072749249 0.03175566
## Xhousehold.income[25K-35K]         -0.0352612669 0.03521440
## Xhousehold.income[35K-50K]         -0.0627353072 0.04023270
## Xhousehold.income[50K-75K]         -0.0912422560 0.04794866
## Xhousehold.income[5K-12K]           0.0238180357 0.02927553
## Xhousehold.income[75K-100K]        -0.1267942339 0.05141875
## Xhigh.educBachelor                  0.0183235644 0.06259881
## Xhigh.educHS Diploma/GED           -0.0211193310 0.03686702
## Xhigh.educPost Graduate Degree      0.0138660188 0.06813503
## Xhigh.educSome College              0.0328915889 0.05678550
## Xdemo_race_hispanic1               -0.0060539612 0.02489808
```

Male participants

```
##
## Family: gaussian
## Link function: identity
```

```
##
## Formula:
## cbcl_scr_syn_withdep_r ~ hormone_scr_ert_mean + hormone_sal_end_min_since_midnight +
##     pds_p_ss_category + race.ethnicity.5level + interview_age +
##     bmi + household.income + high.educ + demo_race_hispanic
##
## Parametric coefficients:
##                                     Estimate Std. Error t value Pr(>|t|)
## (Intercept)                        1.053e+00  6.799e-01   1.548 0.121675
## hormone_scr_ert_mean              -5.717e-04  2.297e-03  -0.249 0.803440
## hormone_sal_end_min_since_midnight -2.282e-05  1.947e-04  -0.117 0.906724
## pds_p_ss_categoryEarly             1.313e-01  8.223e-02   1.597 0.110485
## pds_p_ss_categoryLate              3.450e-01  5.595e-01   0.617 0.537569
## pds_p_ss_categoryMid               3.344e-01  1.688e-01   1.981 0.047757 *
## race.ethnicity.5levelBlack         1.056e-01  2.490e-01   0.424 0.671522
## race.ethnicity.5levelMixed         4.644e-01  2.431e-01   1.910 0.056218 .
## race.ethnicity.5levelOther         2.154e-01  2.880e-01   0.748 0.454508
## race.ethnicity.5levelWhite         3.585e-01  2.258e-01   1.588 0.112419
## interview_age                     -2.060e-03  4.637e-03  -0.444 0.656819
## bmi                                2.369e-02  9.833e-03   2.409 0.016072 *
## household.income[>=200K]          -8.823e-01  2.442e-01  -3.612 0.000310 ***
## household.income[100K-200K]       -8.611e-01  2.278e-01  -3.779 0.000161 ***
## household.income[12K-16K]         -3.678e-01  3.094e-01  -1.189 0.234618
## household.income[16K-25K]         -1.229e-01  2.534e-01  -0.485 0.627848
## household.income[25K-35K]         -3.394e-01  2.472e-01  -1.373 0.169960
## household.income[35K-50K]         -3.034e-01  2.379e-01  -1.275 0.202274
## household.income[50K-75K]         -4.276e-01  2.272e-01  -1.882 0.059994 .
## household.income[5K-12K]           3.594e-01  2.661e-01   1.351 0.176865
## household.income[75K-100K]        -7.091e-01  2.318e-01  -3.059 0.002242 **
## high.educBachelor                  1.420e-01  2.253e-01   0.630 0.528573
## high.educHS Diploma/GED            2.429e-02  2.280e-01   0.107 0.915149
## high.educPost Graduate Degree      8.240e-02  2.287e-01   0.360 0.718712
## high.educSome College              2.201e-01  2.149e-01   1.024 0.305818
## demo_race_hispanic1               -2.986e-01  9.898e-02  -3.016 0.002585 **
## ---
## Signif. codes:  0 '***' 0.001 '**' 0.01 '*' 0.05 '.' 0.1 ' ' 1
##
##
## R-sq.(adj) =  0.0346
## lmer.REML = 9466.8  Scale est. = 2.1785    n = 2441

##                                          stdcoef      stdse
## X(Intercept)                         0.000000000 0.00000000
## Xhormone_scr_ert_mean               -0.005231451 0.02101614
## Xhormone_sal_end_min_since_midnight -0.002369436 0.02021970
## Xpds_p_ss_categoryEarly              0.033408574 0.02092479
## Xpds_p_ss_categoryLate               0.012356955 0.02004106
## Xpds_p_ss_categoryMid                0.041874111 0.02114286
## Xrace.ethnicity.5levelBlack          0.021366117 0.05037848
## Xrace.ethnicity.5levelMixed          0.090250788 0.04724536
## Xrace.ethnicity.5levelOther          0.025476821 0.03405802
## Xrace.ethnicity.5levelWhite          0.099172003 0.06245124
## Xinterview_age                      -0.009170387 0.02063707
## Xbmi                                 0.051780211 0.02149481
```

```
## Xhousehold.income[>=200K]          -0.170223244 0.04712264
## Xhousehold.income[100K-200K]       -0.238021698 0.06298129
## Xhousehold.income[12K-16K]         -0.030486383 0.02564371
## Xhousehold.income[16K-25K]         -0.015322867 0.03160529
## Xhousehold.income[25K-35K]         -0.046482133 0.03386080
## Xhousehold.income[35K-50K]         -0.048848161 0.03829888
## Xhousehold.income[50K-75K]         -0.088035482 0.04678455
## Xhousehold.income[5K-12K]           0.038498586 0.02849919
## Xhousehold.income[75K-100K]        -0.147399708 0.04817881
## Xhigh.educBachelor                  0.037750739 0.05989541
## Xhigh.educHS Diploma/GED            0.003883077 0.03644099
## Xhigh.educPost Graduate Degree      0.023214053 0.06444356
## Xhigh.educSome College              0.056737040 0.05539355
## Xdemo_race_hispanic1               -0.069123414 0.02291658
```

1.19 Model: CBCL Depressed DSM-5 ~ Testosterone + PDS

Female participants

```
##
## Family: gaussian
## Link function: identity
##
## Formula:
## cbcl_scr_dsm5_depress_r ~ hormone_scr_ert_mean + hormone_sal_end_min_since_midnight +
##     PDS_score + race.ethnicity.5level + interview_age + bmi +
##     household.income + high.educ + demo_race_hispanic
##
## Parametric coefficients:
##                                      Estimate Std. Error t value Pr(>|t|)
## (Intercept)                         6.077e-01  7.618e-01   0.798  0.42517
## hormone_scr_ert_mean               -3.013e-03  2.531e-03  -1.190  0.23402
## hormone_sal_end_min_since_midnight  4.308e-05  2.236e-04   0.193  0.84728
## PDS_score                           1.670e-01  6.077e-02   2.748  0.00605 **
## race.ethnicity.5levelBlack         -1.114e-01  2.914e-01  -0.382  0.70224
## race.ethnicity.5levelMixed          4.094e-01  2.837e-01   1.443  0.14912
## race.ethnicity.5levelOther          6.471e-01  3.244e-01   1.995  0.04615 *
## race.ethnicity.5levelWhite          5.455e-01  2.659e-01   2.051  0.04036 *
## interview_age                      -2.290e-03  5.274e-03  -0.434  0.66420
## bmi                                 2.195e-02  1.041e-02   2.109  0.03508 *
## household.income[>=200K]           -7.471e-01  2.722e-01  -2.745  0.00610 **
## household.income[100K-200K]        -6.105e-01  2.543e-01  -2.401  0.01644 *
## household.income[12K-16K]           9.782e-03  3.402e-01   0.029  0.97706
## household.income[16K-25K]           8.275e-02  2.857e-01   0.290  0.77213
## household.income[25K-35K]          -1.436e-01  2.674e-01  -0.537  0.59128
## household.income[35K-50K]          -7.616e-02  2.578e-01  -0.295  0.76772
## household.income[50K-75K]          -2.470e-01  2.568e-01  -0.962  0.33620
## household.income[5K-12K]           -5.264e-03  3.018e-01  -0.017  0.98608
## household.income[75K-100K]         -3.904e-01  2.574e-01  -1.517  0.12953
## high.educBachelor                   1.016e-01  2.537e-01   0.401  0.68866
## high.educHS Diploma/GED            -6.563e-02  2.550e-01  -0.257  0.79694
## high.educPost Graduate Degree       1.513e-01  2.561e-01   0.591  0.55480
## high.educSome College               1.552e-01  2.390e-01   0.649  0.51614
```

```
## demo_race_hispanic1                -6.311e-02  1.153e-01  -0.547  0.58414
## ---
## Signif. codes:  0 '***' 0.001 '**' 0.01 '*' 0.05 '.' 0.1 ' ' 1
##
##
## R-sq.(adj) =  0.0254
## lmer.REML = 8983.5  Scale est. = 1.6796    n = 2239
```

```
##                                          stdcoef      stdse
## X(Intercept)                          0.0000000000 0.00000000
## Xhormone_scr_ert_mean                -0.0269068861 0.02260357
## Xhormone_sal_end_min_since_midnight   0.0042230425 0.02192496
## XPDS_score                            0.0662015945 0.02409103
## Xrace.ethnicity.5levelBlack          -0.0205874543 0.05384519
## Xrace.ethnicity.5levelMixed           0.0757652562 0.05250046
## Xrace.ethnicity.5levelOther           0.0767559857 0.03847090
## Xrace.ethnicity.5levelWhite           0.1413258880 0.06889883
## Xinterview_age                       -0.0095411521 0.02197492
## Xbmi                                  0.0486650001 0.02307811
## Xhousehold.income[>=200K]            -0.1374398963 0.05006821
## Xhousehold.income[100K-200K]         -0.1549168255 0.06452679
## Xhousehold.income[12K-16K]            0.0007808487 0.02715600
## Xhousehold.income[16K-25K]            0.0091932548 0.03174126
## Xhousehold.income[25K-35K]           -0.0189048402 0.03520065
## Xhousehold.income[35K-50K]           -0.0118900399 0.04025218
## Xhousehold.income[50K-75K]           -0.0461724483 0.04800123
## Xhousehold.income[5K-12K]            -0.0005118614 0.02934136
## Xhousehold.income[75K-100K]          -0.0780462378 0.05146429
## Xhigh.educBachelor                    0.0250678713 0.06255631
## Xhigh.educHS Diploma/GED             -0.0094776428 0.03682851
## Xhigh.educPost Graduate Degree        0.0402174173 0.06808728
## Xhigh.educSome College                0.0368850060 0.05679700
## Xdemo_race_hispanic1                 -0.0135402488 0.02473444
```

Male participants

```
##
## Family: gaussian
## Link function: identity
##
## Formula:
## cbcl_scr_dsm5_depress_r ~ hormone_scr_ert_mean + hormone_sal_end_min_since_midnight +
##     PDS_score + race.ethnicity.5level + interview_age + bmi +
##     household.income + high.educ + demo_race_hispanic
##
## Parametric coefficients:
##                                     Estimate Std. Error t value Pr(>|t|)
## (Intercept)                        0.7966397  0.8414906   0.947  0.34389
## hormone_scr_ert_mean              -0.0003464  0.0028707  -0.121  0.90396
## hormone_sal_end_min_since_midnight  0.0001226  0.0002479   0.495  0.62090
## PDS_score                          0.2325044  0.0848805   2.739  0.00620 **
## race.ethnicity.5levelBlack         0.1696711  0.3094801   0.548  0.58357
## race.ethnicity.5levelMixed         0.6472280  0.3017022   2.145  0.03203 *
```

```
## race.ethnicity.5levelOther          0.4355901  0.3579175   1.217  0.22372
## race.ethnicity.5levelWhite          0.5492698  0.2807973   1.956  0.05057 .
## interview_age                      -0.0002317  0.0057630  -0.040  0.96793
## bmi                                 0.0017142  0.0122774   0.140  0.88897
## household.income[>=200K]           -0.7874366  0.3050497  -2.581  0.00990 **
## household.income[100K-200K]        -0.7686710  0.2837566  -2.709  0.00680 **
## household.income[12K-16K]           0.1087929  0.3836082   0.284  0.77674
## household.income[16K-25K]           0.1809445  0.3157108   0.573  0.56661
## household.income[25K-35K]          -0.1481556  0.3074970  -0.482  0.62998
## household.income[35K-50K]          -0.0455965  0.2958200  -0.154  0.87752
## household.income[50K-75K]          -0.4234338  0.2829844  -1.496  0.13470
## household.income[5K-12K]            0.5350011  0.3308581   1.617  0.10601
## household.income[75K-100K]         -0.6764892  0.2885918  -2.344  0.01915 *
## high.educBachelor                   0.3755064  0.2812672   1.335  0.18198
## high.educHS Diploma/GED            -0.0190531  0.2845675  -0.067  0.94662
## high.educPost Graduate Degree       0.2185827  0.2854614   0.766  0.44392
## high.educSome College               0.1852382  0.2679579   0.691  0.48945
## demo_race_hispanic1                -0.3256471  0.1259244  -2.586  0.00977 **
## ---
## Signif. codes:  0 '***' 0.001 '**' 0.01 '*' 0.05 '.' 0.1 ' ' 1
##
##
## R-sq.(adj) =  0.0198
## lmer.REML =  10493  Scale est. = 2.624     n = 2441
```

```
##                                          stdcoef      stdse
## X(Intercept)                         0.0000000000 0.00000000
## Xhormone_scr_ert_mean               -0.0025741563 0.02133183
## Xhormone_sal_end_min_since_midnight  0.0103404611 0.02090480
## XPDS_score                           0.0595051601 0.02172358
## Xrace.ethnicity.5levelBlack          0.0278796672 0.05085251
## Xrace.ethnicity.5levelMixed          0.1021343510 0.04760943
## Xrace.ethnicity.5levelOther          0.0418328708 0.03437341
## Xrace.ethnicity.5levelWhite          0.1233787448 0.06307360
## Xinterview_age                      -0.0008375659 0.02082988
## Xbmi                                 0.0030430027 0.02179497
## Xhousehold.income[>=200K]           -0.1233671475 0.04779193
## Xhousehold.income[100K-200K]        -0.1725474083 0.06369626
## Xhousehold.income[12K-16K]           0.0073232454 0.02582206
## Xhousehold.income[16K-25K]           0.0183240055 0.03197161
## Xhousehold.income[25K-35K]          -0.0164779256 0.03419994
## Xhousehold.income[35K-50K]          -0.0059607381 0.03867199
## Xhousehold.income[50K-75K]          -0.0707970410 0.04731427
## Xhousehold.income[5K-12K]            0.0465361088 0.02877910
## Xhousehold.income[75K-100K]         -0.1141925816 0.04871481
## Xhigh.educBachelor                   0.0810824635 0.06073355
## Xhigh.educHS Diploma/GED            -0.0024733851 0.03694130
## Xhigh.educPost Graduate Degree       0.0500059809 0.06530608
## Xhigh.educSome College               0.0387815151 0.05609973
## Xdemo_race_hispanic1                -0.0612231275 0.02367436
```

1.20 Model: CBCL Depressed DSM-5 ~ Testosterone + Pubertal category

Female participants

```
##
## Family: gaussian
## Link function: identity
##
## Formula:
## cbcl_scr_dsm5_depress_r ~ hormone_scr_ert_mean + hormone_sal_end_min_since_midnight +
##     pds_p_ss_category + race.ethnicity.5level + interview_age +
##     bmi + household.income + high.educ + demo_race_hispanic
##
## Parametric coefficients:
##                                     Estimate Std. Error t value Pr(>|t|)
## (Intercept)                        8.541e-01  7.856e-01   1.087  0.27708
## hormone_scr_ert_mean              -2.279e-03  2.501e-03  -0.911  0.36242
## hormone_sal_end_min_since_midnight  1.337e-05  2.242e-04   0.060  0.95247
## pds_p_ss_categoryEarly             2.317e-01  1.048e-01   2.210  0.02723 *
## pds_p_ss_categoryLate              2.386e-01  2.717e-01   0.878  0.37999
## pds_p_ss_categoryMid               2.952e-01  1.061e-01   2.782  0.00545 **
## race.ethnicity.5levelBlack        -7.418e-02  2.913e-01  -0.255  0.79903
## race.ethnicity.5levelMixed         4.308e-01  2.839e-01   1.517  0.12930
## race.ethnicity.5levelOther         6.850e-01  3.242e-01   2.113  0.03473 *
## race.ethnicity.5levelWhite         5.655e-01  2.662e-01   2.125  0.03371 *
## interview_age                     -3.037e-03  5.377e-03  -0.565  0.57229
## bmi                                1.748e-02  1.080e-02   1.618  0.10589
## household.income[>=200K]          -7.557e-01  2.724e-01  -2.774  0.00558 **
## household.income[100K-200K]       -6.368e-01  2.545e-01  -2.502  0.01241 *
## household.income[12K-16K]         -6.812e-03  3.406e-01  -0.020  0.98404
## household.income[16K-25K]          5.342e-02  2.862e-01   0.187  0.85193
## household.income[25K-35K]         -1.738e-01  2.677e-01  -0.649  0.51633
## household.income[35K-50K]         -1.108e-01  2.582e-01  -0.429  0.66788
## household.income[50K-75K]         -2.684e-01  2.570e-01  -1.045  0.29634
## household.income[5K-12K]          -2.340e-03  3.020e-01  -0.008  0.99382
## household.income[75K-100K]        -4.118e-01  2.577e-01  -1.598  0.11021
## high.educBachelor                  1.143e-01  2.540e-01   0.450  0.65274
## high.educHS Diploma/GED           -4.123e-02  2.552e-01  -0.162  0.87163
## high.educPost Graduate Degree      1.656e-01  2.565e-01   0.646  0.51852
## high.educSome College              1.727e-01  2.391e-01   0.722  0.47023
## demo_race_hispanic1               -6.657e-02  1.157e-01  -0.575  0.56525
## ---
## Signif. codes:  0 '***' 0.001 '**' 0.01 '*' 0.05 '.' 0.1 ' ' 1
##
##
## R-sq.(adj) =  0.0245
## lmer.REML = 8985.4  Scale est. = 1.659     n = 2239
```

```
##                                          stdcoef      stdse
## X(Intercept)                        0.0000000000 0.00000000
## Xhormone_scr_ert_mean              -0.0203466473 0.02233579
## Xhormone_sal_end_min_since_midnight  0.0013104027 0.02198039
## Xpds_p_ss_categoryEarly             0.0551307324 0.02494905
## Xpds_p_ss_categoryLate              0.0198038381 0.02255331
```

```
## Xpds_p_ss_categoryMid              0.0802107011 0.02883366
## Xrace.ethnicity.5levelBlack       -0.0137069628 0.05383053
## Xrace.ethnicity.5levelMixed        0.0797190484 0.05253584
## Xrace.ethnicity.5levelOther        0.0812479329 0.03845421
## Xrace.ethnicity.5levelWhite        0.1465200074 0.06895735
## Xinterview_age                    -0.0126534411 0.02240460
## Xbmi                               0.0387532895 0.02395701
## Xhousehold.income[>=200K]         -0.1390189306 0.05011136
## Xhousehold.income[100K-200K]      -0.1615862989 0.06457192
## Xhousehold.income[12K-16K]        -0.0005438026 0.02718883
## Xhousehold.income[16K-25K]         0.0059345685 0.03178949
## Xhousehold.income[25K-35K]        -0.0228767745 0.03524309
## Xhousehold.income[35K-50K]        -0.0172997829 0.04031550
## Xhousehold.income[50K-75K]        -0.0501773529 0.04803695
## Xhousehold.income[5K-12K]         -0.0002274952 0.02936731
## Xhousehold.income[75K-100K]       -0.0823225240 0.05152009
## Xhigh.educBachelor                 0.0281911314 0.06264487
## Xhigh.educHS Diploma/GED          -0.0059541802 0.03684472
## Xhigh.educPost Graduate Degree     0.0440333589 0.06819100
## Xhigh.educSome College             0.0410377178 0.05682092
## Xdemo_race_hispanic1              -0.0142827625 0.02483326
```

Male participants

```
##
## Family: gaussian
## Link function: identity
##
## Formula:
## cbcl_scr_dsm5_depress_r ~ hormone_scr_ert_mean + hormone_sal_end_min_since_midnight +
##     pds_p_ss_category + race.ethnicity.5level + interview_age +
##     bmi + household.income + high.educ + demo_race_hispanic
##
## Parametric coefficients:
##                                     Estimate Std. Error t value Pr(>|t|)
## (Intercept)                        9.322e-01  8.458e-01   1.102  0.27049
## hormone_scr_ert_mean              -1.287e-04  2.866e-03  -0.045  0.96418
## hormone_sal_end_min_since_midnight  1.280e-04  2.478e-04   0.517  0.60537
## pds_p_ss_categoryEarly             2.105e-01  1.019e-01   2.066  0.03897 *
## pds_p_ss_categoryLate              3.444e-01  6.892e-01   0.500  0.61730
## pds_p_ss_categoryMid               3.929e-01  2.098e-01   1.872  0.06129 .
## race.ethnicity.5levelBlack         1.675e-01  3.099e-01   0.541  0.58886
## race.ethnicity.5levelMixed         6.539e-01  3.019e-01   2.166  0.03040 *
## race.ethnicity.5levelOther         4.440e-01  3.584e-01   1.239  0.21552
## race.ethnicity.5levelWhite         5.598e-01  2.810e-01   1.992  0.04644 *
## interview_age                      5.579e-05  5.763e-03   0.010  0.99228
## bmi                                4.194e-03  1.221e-02   0.344  0.73120
## household.income[>=200K]          -8.044e-01  3.050e-01  -2.637  0.00841 **
## household.income[100K-200K]       -7.796e-01  2.839e-01  -2.745  0.00609 **
## household.income[12K-16K]          1.051e-01  3.840e-01   0.274  0.78446
## household.income[16K-25K]          1.680e-01  3.159e-01   0.532  0.59504
## household.income[25K-35K]         -1.631e-01  3.077e-01  -0.530  0.59605
## household.income[35K-50K]         -5.156e-02  2.962e-01  -0.174  0.86181
```

```
## household.income[50K-75K]          -4.318e-01  2.832e-01  -1.525  0.12748
## household.income[5K-12K]            5.076e-01  3.309e-01   1.534  0.12521
## household.income[75K-100K]         -6.879e-01  2.887e-01  -2.382  0.01728 *
## high.educBachelor                   3.937e-01  2.817e-01   1.398  0.16234
## high.educHS Diploma/GED             7.282e-03  2.847e-01   0.026  0.97960
## high.educPost Graduate Degree       2.426e-01  2.859e-01   0.849  0.39612
## high.educSome College               2.070e-01  2.683e-01   0.771  0.44050
## demo_race_hispanic1                -3.404e-01  1.262e-01  -2.698  0.00703 **
## ---
## Signif. codes:  0 '***' 0.001 '**' 0.01 '*' 0.05 '.' 0.1 ' ' 1
##
##
## R-sq.(adj) =  0.0186
## lmer.REML =  10494  Scale est. = 2.6077    n = 2441
```

```
##                                           stdcoef      stdse
## X(Intercept)                          0.0000000000 0.00000000
## Xhormone_scr_ert_mean                -0.0009564344 0.02129720
## Xhormone_sal_end_min_since_midnight   0.0107970707 0.02089387
## Xpds_p_ss_categoryEarly               0.0434984338 0.02105807
## Xpds_p_ss_categoryLate                0.0100191167 0.02004861
## Xpds_p_ss_categoryMid                 0.0399520215 0.02133919
## Xrace.ethnicity.5levelBlack           0.0275221321 0.05091455
## Xrace.ethnicity.5levelMixed           0.1031930017 0.04764112
## Xrace.ethnicity.5levelOther           0.0426397176 0.03441845
## Xrace.ethnicity.5levelWhite           0.1257481892 0.06311445
## Xinterview_age                        0.0002016645 0.02082943
## Xbmi                                  0.0074449630 0.02166958
## Xhousehold.income[>=200K]            -0.1260270049 0.04778730
## Xhousehold.income[100K-200K]         -0.1749924644 0.06373891
## Xhousehold.income[12K-16K]            0.0070714671 0.02585140
## Xhousehold.income[16K-25K]            0.0170083044 0.03199322
## Xhousehold.income[25K-35K]           -0.0181425772 0.03422120
## Xhousehold.income[35K-50K]           -0.0067406551 0.03871933
## Xhousehold.income[50K-75K]           -0.0721929647 0.04735086
## Xhousehold.income[5K-12K]             0.0441513192 0.02878551
## Xhousehold.income[75K-100K]          -0.1161155294 0.04873916
## Xhigh.educBachelor                    0.0850199282 0.06083031
## Xhigh.educHS Diploma/GED              0.0009453291 0.03695718
## Xhigh.educPost Graduate Degree        0.0555095267 0.06540393
## Xhigh.educSome College                0.0433360815 0.05617302
## Xdemo_race_hispanic1                 -0.0639889200 0.02372117
```

2—Reward~Puberty—

2.1 Model: BIS-BAS-RR ~ PDS

Female participants

```
##
## Family: gaussian
## Link function: identity
##
## Formula:
## bisbas_ss_basm_rr_z ~ PDS_score + interview_age + bmi
##
## Parametric coefficients:
##                Estimate Std. Error t value Pr(>|t|)
## (Intercept)    0.298321   0.316443   0.943   0.3459
## PDS_score      0.068436   0.028243   2.423   0.0155 *
## interview_age -0.004993   0.002643  -1.889   0.0590 .
## bmi            0.007032   0.004928   1.427   0.1537
## ---
## Signif. codes:  0 '***' 0.001 '**' 0.01 '*' 0.05 '.' 0.1 ' ' 1
##
##
## R-sq.(adj) =  0.00573
## lmer.REML = 7486.5  Scale est. = 0.75186   n = 2664
```

```
##                   stdcoef      stdse
## X(Intercept)    0.00000000 0.00000000
## XPDS_score      0.05073923 0.02093942
## Xinterview_age -0.03789419 0.02006129
## Xbmi            0.02908214 0.02037759
```

Male participants

```
##
## Family: gaussian
## Link function: identity
##
## Formula:
## bisbas_ss_basm_rr_z ~ PDS_score + interview_age + bmi
##
## Parametric coefficients:
##                 Estimate Std. Error t value Pr(>|t|)
## (Intercept)   -0.0005436  0.2967451  -0.002  0.99854
## PDS_score      0.0744553  0.0346846   2.147  0.03191 *
## interview_age -0.0025936  0.0024588  -1.055  0.29159
## bmi            0.0148169  0.0050138   2.955  0.00315 **
## ---
## Signif. codes:  0 '***' 0.001 '**' 0.01 '*' 0.05 '.' 0.1 ' ' 1
##
##
## R-sq.(adj) =  0.00649
## lmer.REML = 7986.3  Scale est. = 0.7387    n = 2883
```

```
##                   stdcoef      stdse
## X(Intercept)    0.00000000 0.00000000
## XPDS_score      0.04166627 0.01941001
## Xinterview_age -0.02006441 0.01902138
## Xbmi            0.05671888 0.01919280
```

2.2 Model : Reaction Time ~ PDS

Female participants

```
##
## Family: gaussian
## Link function: identity
##
## Formula:
## rt_diff_large_neutral_z ~ PDS_score + interview_age + bmi
##
## Parametric coefficients:
##                Estimate Std. Error t value Pr(>|t|)
## (Intercept)   -0.492319   0.326627  -1.507   0.1319
## PDS_score     -0.020354   0.029760  -0.684   0.4941
## interview_age  0.004998   0.002734   1.829   0.0676 .
## bmi           -0.001264   0.005203  -0.243   0.8081
## ---
## Signif. codes:  0 '***' 0.001 '**' 0.01 '*' 0.05 '.' 0.1 ' ' 1
##
##
## R-sq.(adj) =  0.000347
## lmer.REML = 5861.9  Scale est. = 0.67509   n = 2178
```

```
##                    stdcoef      stdse
## X(Intercept)    0.000000000 0.00000000
## XPDS_score     -0.015839921 0.02316012
## Xinterview_age  0.040615132 0.02221223
## Xbmi           -0.005465454 0.02250120
```

```
##
## Family: gaussian
## Link function: identity
##
## Formula:
## rt_diff_large_small_z ~ PDS_score + interview_age + bmi
##
## Parametric coefficients:
##                Estimate Std. Error t value Pr(>|t|)
## (Intercept)   -0.198499   0.328520  -0.604    0.546
## PDS_score     -0.016581   0.029856  -0.555    0.579
## interview_age  0.002938   0.002748   1.069    0.285
## bmi           -0.005648   0.005223  -1.081    0.280
##
##
## R-sq.(adj) =  -1.97e-05
## lmer.REML = 5896.6  Scale est. = 0.76102   n = 2178
```

```
##                    stdcoef      stdse
## X(Intercept)    0.00000000 0.00000000
## XPDS_score     -0.01281239 0.02307029
## Xinterview_age  0.02370299 0.02216791
## Xbmi           -0.02425320 0.02243013
```

Male participants

```
##
## Family: gaussian
## Link function: identity
##
## Formula:
## rt_diff_large_neutral_z ~ PDS_score + interview_age + bmi
##
## Parametric coefficients:
##                  Estimate Std. Error t value Pr(>|t|)
## (Intercept)    0.1217327  0.3014476   0.404   0.6864
## PDS_score     -0.0628579  0.0364862  -1.723   0.0851 .
## interview_age -0.0005185  0.0024951  -0.208   0.8354
## bmi            0.0006910  0.0051678   0.134   0.8936
## ---
## Signif. codes:  0 '***' 0.001 '**' 0.01 '*' 0.05 '.' 0.1 ' ' 1
##
##
## R-sq.(adj) =  0.000262
## lmer.REML =   5915  Scale est. = 0.71331   n = 2283
```

```
##                    stdcoef      stdse
## X(Intercept)    0.000000000 0.00000000
## XPDS_score     -0.037695689 0.02188064
## Xinterview_age -0.004442590 0.02137766
## Xbmi            0.002893587 0.02164127
```

```
##
## Family: gaussian
## Link function: identity
##
## Formula:
## rt_diff_large_small_z ~ PDS_score + interview_age + bmi
##
## Parametric coefficients:
##                  Estimate Std. Error t value Pr(>|t|)
## (Intercept)    0.0167726  0.3051802   0.055    0.956
## PDS_score     -0.0253887  0.0368668  -0.689    0.491
## interview_age -0.0003827  0.0025245  -0.152    0.880
## bmi            0.0038297  0.0052241   0.733    0.464
##
##
## R-sq.(adj) =  -0.000932
## lmer.REML = 5990.5  Scale est. = 0.79834   n = 2283
```

```
##                    stdcoef      stdse
```

```
## X(Intercept)    0.000000000 0.00000000
## XPDS_score     -0.014989099 0.02176561
## Xinterview_age -0.003228414 0.02129425
## Xbmi            0.015788393 0.02153720
```

2.3 Model: Caudate Anticipation ~ PDS

Female participants

```
##
## Family: gaussian
## Link function: identity
##
## Formula:
## caudate_rvsn_ant_z ~ PDS_score + interview_age + bmi
##
## Parametric coefficients:
##                Estimate Std. Error t value Pr(>|t|)
## (Intercept)   -0.380798   0.326697  -1.166   0.2439
## PDS_score     -0.038377   0.029682  -1.293   0.1962
## interview_age  0.004972   0.002747   1.810   0.0704 .
## bmi           -0.007080   0.005175  -1.368   0.1714
## ---
## Signif. codes:  0 '***' 0.001 '**' 0.01 '*' 0.05 '.' 0.1 ' ' 1
##
##
## R-sq.(adj) =  0.0024
## lmer.REML = 5292.9  Scale est. = 0.77206   n = 2024
```

```
##                   stdcoef      stdse
## X(Intercept)    0.00000000 0.00000000
## XPDS_score     -0.03094881 0.02393701
## Xinterview_age  0.04174111 0.02305947
## Xbmi           -0.03184258 0.02327484
```

Male participants

```
##
## Family: gaussian
## Link function: identity
##
## Formula:
## caudate_rvsn_ant_z ~ PDS_score + interview_age + bmi
##
## Parametric coefficients:
##                Estimate Std. Error t value Pr(>|t|)
## (Intercept)   -0.051331   0.350999  -0.146    0.884
## PDS_score      0.005881   0.042724   0.138    0.891
## interview_age  0.001868   0.002905   0.643    0.520
## bmi           -0.009917   0.006093  -1.627    0.104
##
##
```

```
## R-sq.(adj) =  -1.1e-05
## lmer.REML = 5707.4  Scale est. = 0.78025   n = 2051
```

```
##                    stdcoef      stdse
## X(Intercept)    0.000000000 0.00000000
## XPDS_score      0.003191922 0.02318964
## Xinterview_age  0.014526649 0.02259127
## Xbmi           -0.037291797 0.02291395
```

2.4 Model B: Putamen Anticipation ~ PDS

Female participants

```
##
## Family: gaussian
## Link function: identity
##
## Formula:
## putamen_rvsn_ant_z ~ PDS_score + interview_age + bmi
##
## Parametric coefficients:
##                Estimate Std. Error t value Pr(>|t|)
## (Intercept)   -0.290901   0.318558  -0.913   0.3613
## PDS_score     -0.068519   0.028963  -2.366   0.0181 *
## interview_age  0.004131   0.002677   1.543   0.1229
## bmi           -0.004351   0.005043  -0.863   0.3884
## ---
## Signif. codes:  0 '***' 0.001 '**' 0.01 '*' 0.05 '.' 0.1 ' ' 1
##
##
## R-sq.(adj) =  0.00336
## lmer.REML = 5181.5  Scale est. = 0.7261    n = 2021
```

```
##                   stdcoef      stdse
## X(Intercept)    0.00000000 0.00000000
## XPDS_score     -0.05665149 0.02394615
## Xinterview_age  0.03557893 0.02305580
## Xbmi           -0.02009704 0.02329329
```

Male participants

```
##
## Family: gaussian
## Link function: identity
##
## Formula:
## putamen_rvsn_ant_z ~ PDS_score + interview_age + bmi
##
## Parametric coefficients:
##                Estimate Std. Error t value Pr(>|t|)
## (Intercept)   -0.314442   0.338898  -0.928   0.3536
## PDS_score      0.016920   0.041379   0.409   0.6826
```

```
## interview_age  0.004024   0.002809   1.433   0.1521
## bmi           -0.010000   0.005902  -1.694   0.0903 .
## ---
## Signif. codes:  0 '***' 0.001 '**' 0.01 '*' 0.05 '.' 0.1 ' ' 1
##
##
## R-sq.(adj) =  0.000956
## lmer.REML = 5556.1  Scale est. = 0.86428   n = 2048
```

```
##                     stdcoef      stdse
## X(Intercept)    0.000000000 0.00000000
## XPDS_score      0.009450731 0.02311211
## Xinterview_age  0.032402338 0.02261670
## Xbmi           -0.038712530 0.02284670
```

2.5 Model: Accumbens Anticipation ~ PDS

Female participants

```
##
## Family: gaussian
## Link function: identity
##
## Formula:
## accumbens_rvsn_ant_z ~ PDS_score + interview_age + bmi
##
## Parametric coefficients:
##                 Estimate Std. Error t value Pr(>|t|)
## (Intercept)    0.0286509  0.2510732   0.114   0.9092
## PDS_score      0.0051654  0.0227507   0.227   0.8204
## interview_age  0.0008251  0.0021102   0.391   0.6958
## bmi           -0.0068322  0.0039745  -1.719   0.0858 .
## ---
## Signif. codes:  0 '***' 0.001 '**' 0.01 '*' 0.05 '.' 0.1 ' ' 1
##
##
## R-sq.(adj) =  0.000361
## lmer.REML = 4235.1  Scale est. = 0.44395   n = 2024
```

```
##                     stdcoef      stdse
## X(Intercept)    0.000000000 0.00000000
## XPDS_score      0.005435810 0.02394165
## Xinterview_age  0.009020845 0.02307031
## Xbmi           -0.040040812 0.02329293
```

Male participants

```
##
## Family: gaussian
## Link function: identity
##
## Formula:
```

```
## accumbens_rvsn_ant_z ~ PDS_score + interview_age + bmi
##
## Parametric coefficients:
##                Estimate Std. Error t value Pr(>|t|)
## (Intercept)    0.353219   0.264169   1.337    0.181
## PDS_score      0.015397   0.032094   0.480    0.631
## interview_age -0.002592   0.002186  -1.185    0.236
## bmi           -0.003171   0.004573  -0.693    0.488
##
##
## R-sq.(adj) =  -0.000477
## lmer.REML = 4563.7  Scale est. = 0.51688   n = 2050
```

```
##                    stdcoef      stdse
## X(Intercept)    0.00000000 0.00000000
## XPDS_score      0.01105455 0.02304184
## Xinterview_age -0.02667522 0.02250180
## Xbmi           -0.01579614 0.02278026
```

2.6 Model: Caudate Feedback ~ PDS

Female participants

```
##
## Family: gaussian
## Link function: identity
##
## Formula:
## caudate_posvsneg_feedback_z ~ PDS_score + interview_age + bmi
##
## Parametric coefficients:
##                Estimate Std. Error t value Pr(>|t|)
## (Intercept)    0.921257   0.313893   2.935  0.00337 **
## PDS_score     -0.015936   0.028439  -0.560  0.57530
## interview_age -0.007344   0.002634  -2.788  0.00535 **
## bmi           -0.002236   0.004976  -0.449  0.65320
## ---
## Signif. codes:  0 '***' 0.001 '**' 0.01 '*' 0.05 '.' 0.1 ' ' 1
##
##
## R-sq.(adj) =  0.00352
## lmer.REML = 5146.5  Scale est. = 0.73665   n = 2022
```

```
##                    stdcoef      stdse
## X(Intercept)    0.00000000 0.00000000
## XPDS_score     -0.01333577 0.02379859
## Xinterview_age -0.06389584 0.02291736
## Xbmi           -0.01041646 0.02317948
```

Male participants

```
##
```

```
## Family: gaussian
## Link function: identity
##
## Formula:
## caudate_posvsneg_feedback_z ~ PDS_score + interview_age + bmi
##
## Parametric coefficients:
##                Estimate Std. Error t value Pr(>|t|)
## (Intercept)   -0.073539   0.317520  -0.232    0.817
## PDS_score     -0.074984   0.038570  -1.944    0.052 .
## interview_age  0.001452   0.002626   0.553    0.580
## bmi            0.002145   0.005493   0.391    0.696
## ---
## Signif. codes:  0 '***' 0.001 '**' 0.01 '*' 0.05 '.' 0.1 ' ' 1
##
##
## R-sq.(adj) =  0.000463
## lmer.REML = 5301.3  Scale est. = 0.76814   n = 2049
```

```
##                     stdcoef      stdse
## X(Intercept)    0.000000000 0.00000000
## XPDS_score     -0.044823517 0.02305629
## Xinterview_age  0.012445945 0.02250737
## Xbmi            0.008899474 0.02278943
```

2.7 Model: Putamen Feedback ~ PDS

Female participants

```
##
## Family: gaussian
## Link function: identity
##
## Formula:
## putamen_posvsneg_feedback_z ~ PDS_score + interview_age + bmi
##
## Parametric coefficients:
##                Estimate Std. Error t value Pr(>|t|)
## (Intercept)    0.646281   0.300041   2.154   0.0314 *
## PDS_score      0.014996   0.027092   0.554   0.5800
## interview_age -0.005251   0.002518  -2.085   0.0372 *
## bmi           -0.005039   0.004753  -1.060   0.2892
## ---
## Signif. codes:  0 '***' 0.001 '**' 0.01 '*' 0.05 '.' 0.1 ' ' 1
##
##
## R-sq.(adj) =  0.00123
## lmer.REML = 4953.2  Scale est. = 0.66748   n = 2022
```

```
##                    stdcoef      stdse
## X(Intercept)    0.00000000 0.00000000
## XPDS_score      0.01320436 0.02385580
## Xinterview_age -0.04799661 0.02301781
## Xbmi           -0.02460305 0.02320754
```

Male participants

```
##
## Family: gaussian
## Link function: identity
##
## Formula:
## putamen_posvsneg_feedback_z ~ PDS_score + interview_age + bmi
##
## Parametric coefficients:
##                 Estimate Std. Error t value Pr(>|t|)
## (Intercept)    0.1455169  0.3164926   0.460   0.6457
## PDS_score     -0.0665416  0.0384073  -1.733   0.0833 .
## interview_age -0.0009454  0.0026163  -0.361   0.7179
## bmi            0.0072475  0.0054768   1.323   0.1859
## ---
## Signif. codes:  0 '***' 0.001 '**' 0.01 '*' 0.05 '.' 0.1 ' ' 1
##
##
## R-sq.(adj) =  0.000271
## lmer.REML = 5275.5  Scale est. = 0.74806   n = 2053
```

```
##                    stdcoef      stdse
## X(Intercept)    0.00000000 0.00000000
## XPDS_score     -0.04004270 0.02311235
## Xinterview_age -0.00815315 0.02256201
## Xbmi            0.03027470 0.02287807
```

2.8 Model: Accumbens Feedback ~ PDS

Female participants

```
##
## Family: gaussian
## Link function: identity
##
## Formula:
## accumbens_posvsneg_feedback_z ~ PDS_score + interview_age + bmi
##
## Parametric coefficients:
##                 Estimate Std. Error t value Pr(>|t|)
## (Intercept)    4.408e-01  2.379e-01   1.853   0.0640 .
## PDS_score      6.486e-05  2.152e-02   0.003   0.9976
## interview_age -4.008e-03  1.999e-03  -2.005   0.0451 *
## bmi            1.222e-03  3.765e-03   0.325   0.7454
## ---
## Signif. codes:  0 '***' 0.001 '**' 0.01 '*' 0.05 '.' 0.1 ' ' 1
##
##
## R-sq.(adj) =  0.000588
## lmer.REML = 4055.5  Scale est. = 0.42501   n = 2031
```

```
##                        stdcoef      stdse
```

```
## X(Intercept)    0.000000e+00 0.00000000
## XPDS_score      7.165354e-05 0.02377462
## Xinterview_age -4.595863e-02 0.02291827
## Xbmi            7.520182e-03 0.02315942
```

Male participants

```
##
## Family: gaussian
## Link function: identity
##
## Formula:
## accumbens_posvsneg_feedback_z ~ PDS_score + interview_age + bmi
##
## Parametric coefficients:
##                Estimate Std. Error t value Pr(>|t|)
## (Intercept)   -0.083528   0.254751  -0.328    0.743
## PDS_score     -0.037030   0.030969  -1.196    0.232
## interview_age  0.001101   0.002108   0.522    0.602
## bmi            0.002752   0.004405   0.625    0.532
##
##
## R-sq.(adj) =  -0.000632
## lmer.REML = 4371.1  Scale est. = 0.42105   n = 2046
```

```
##                    stdcoef      stdse
## X(Intercept)    0.00000000 0.00000000
## XPDS_score     -0.02776078 0.02321740
## Xinterview_age  0.01182846 0.02264852
## Xbmi            0.01433974 0.02294800
```

2.9 Model: OFC Anticipation ~ PDS

Female participants

```
##
## Family: gaussian
## Link function: identity
##
## Formula:
## lOFC_rvsn_ant_z ~ PDS_score + interview_age + bmi
##
## Parametric coefficients:
##                 Estimate Std. Error t value Pr(>|t|)
## (Intercept)    4.874e-02  2.099e-01   0.232    0.816
## PDS_score      8.897e-03  1.900e-02   0.468    0.640
## interview_age -7.427e-05  1.766e-03  -0.042    0.966
## bmi           -2.173e-03  3.330e-03  -0.653    0.514
##
##
## R-sq.(adj) =  -0.00121
## lmer.REML =   3505  Scale est. = 0.29594   n = 2018
```

```
##                    stdcoef      stdse
## X(Intercept)    0.000000000 0.00000000
## XPDS_score      0.011200572 0.02392156
## Xinterview_age -0.000970162 0.02306502
## Xbmi           -0.015198951 0.02329320
```

```
##
## Family: gaussian
## Link function: identity
##
## Formula:
## mOFC_rvsn_ant_z ~ PDS_score + interview_age + bmi
##
## Parametric coefficients:
##                 Estimate Std. Error t value Pr(>|t|)
## (Intercept)    0.0192469  0.2413048   0.080    0.936
## PDS_score      0.0017731  0.0218305   0.081    0.935
## interview_age -0.0003229  0.0020284  -0.159    0.874
## bmi            0.0008238  0.0038125   0.216    0.829
##
##
## R-sq.(adj) =  -0.00145
## lmer.REML = 4076.9  Scale est. = 0.43488   n = 2019
```

```
##                    stdcoef      stdse
## X(Intercept)    0.000000000 0.00000000
## XPDS_score      0.001936461 0.02384155
## Xinterview_age -0.003663281 0.02301131
## Xbmi            0.005015968 0.02321285
```

Male participants

```
##
## Family: gaussian
## Link function: identity
##
## Formula:
## lOFC_rvsn_ant_z ~ PDS_score + interview_age + bmi
##
## Parametric coefficients:
##                 Estimate Std. Error t value Pr(>|t|)
## (Intercept)   -0.1962797  0.2229717  -0.880    0.379
## PDS_score      0.0305165  0.0273309   1.117    0.264
## interview_age  0.0014567  0.0018474   0.788    0.431
## bmi           -0.0005914  0.0038763  -0.153    0.879
##
##
## R-sq.(adj) =  -0.000347
## lmer.REML = 3830.7  Scale est. = 0.34533   n = 2044
```

```
##                    stdcoef      stdse
## X(Intercept)    0.000000000 0.00000000
## XPDS_score      0.025925801 0.02321939
```

```
## Xinterview_age  0.017862169 0.02265410
## Xbmi           -0.003495261 0.02291033
```

```
##
## Family: gaussian
## Link function: identity
##
## Formula:
## mOFC_rvsn_ant_z ~ PDS_score + interview_age + bmi
##
## Parametric coefficients:
##                 Estimate Std. Error t value Pr(>|t|)
## (Intercept)   -0.1161914  0.2427040  -0.479  0.63218
## PDS_score      0.0767400  0.0296187   2.591  0.00964 **
## interview_age -0.0001415  0.0020102  -0.070  0.94389
## bmi            0.0016024  0.0042019   0.381  0.70297
## ---
## Signif. codes:  0 '***' 0.001 '**' 0.01 '*' 0.05 '.' 0.1 ' ' 1
##
##
## R-sq.(adj) =  0.00239
## lmer.REML = 4176.9  Scale est. = 0.40576   n = 2039
```

```
##                     stdcoef      stdse
## X(Intercept)    0.000000000 0.00000000
## XPDS_score      0.059917347 0.02312582
## Xinterview_age -0.001588685 0.02257011
## Xbmi            0.008707476 0.02283242
```

2.10 Model: OFC Feedback ~ PDS

Female participants

```
##
## Family: gaussian
## Link function: identity
##
## Formula:
## lOFC_posvsneg_feedback_z ~ PDS_score + interview_age + bmi
##
## Parametric coefficients:
##                 Estimate Std. Error t value Pr(>|t|)
## (Intercept)    0.2537734  0.1839272   1.380    0.168
## PDS_score      0.0126560  0.0166447   0.760    0.447
## interview_age -0.0024151  0.0015445  -1.564    0.118
## bmi           -0.0006546  0.0029147  -0.225    0.822
##
##
## R-sq.(adj) =  -0.00017
## lmer.REML =   2980  Scale est. = 0.22154   n = 2019
```

```
##                     stdcoef      stdse
```

```
## X(Intercept)    0.000000000 0.00000000
## XPDS_score      0.018169412 0.02389578
## Xinterview_age -0.035960513 0.02299817
## Xbmi           -0.005231914 0.02329458
```

```
##
## Family: gaussian
## Link function: identity
##
## Formula:
## mOFC_posvsneg_feedback_z ~ PDS_score + interview_age + bmi
##
## Parametric coefficients:
##                 Estimate Std. Error t value Pr(>|t|)
## (Intercept)    0.0070788  0.2264411   0.031    0.975
## PDS_score      0.0060376  0.0205133   0.294    0.769
## interview_age -0.0006626  0.0019026  -0.348    0.728
## bmi            0.0028691  0.0035897   0.799    0.424
##
##
## R-sq.(adj) =  -0.00111
## lmer.REML = 3805.3  Scale est. = 0.3422    n = 2020
```

```
##                    stdcoef      stdse
## X(Intercept)    0.000000000 0.00000000
## XPDS_score      0.007065521 0.02400584
## Xinterview_age -0.008038937 0.02308195
## Xbmi            0.018680512 0.02337226
```

Male participants

```
##
## Family: gaussian
## Link function: identity
##
## Formula:
## lOFC_posvsneg_feedback_z ~ PDS_score + interview_age + bmi
##
## Parametric coefficients:
##                Estimate Std. Error t value Pr(>|t|)
## (Intercept)   -0.169140   0.200981  -0.842   0.4001
## PDS_score      0.020621   0.024459   0.843   0.3993
## interview_age  0.002554   0.001663   1.536   0.1247
## bmi           -0.007087   0.003489  -2.031   0.0424 *
## ---
## Signif. codes:  0 '***' 0.001 '**' 0.01 '*' 0.05 '.' 0.1 ' ' 1
##
##
## R-sq.(adj) =  0.00176
## lmer.REML = 3457.8  Scale est. = 0.31012   n = 2054
```

```
##                   stdcoef      stdse
## X(Intercept)    0.00000000 0.00000000
```

```
## XPDS_score      0.01936435 0.02296797
## Xinterview_age  0.03448493 0.02245160
## Xbmi           -0.04609739 0.02269433
```

```
##
## Family: gaussian
## Link function: identity
##
## Formula:
## mOFC_posvsneg_feedback_z ~ PDS_score + interview_age + bmi
##
## Parametric coefficients:
##                  Estimate Std. Error t value Pr(>|t|)
## (Intercept)    0.0024664  0.2308330   0.011    0.991
## PDS_score      0.0128914  0.0281759   0.458    0.647
## interview_age  0.0005831  0.0019114   0.305    0.760
## bmi           -0.0028760  0.0040187  -0.716    0.474
##
##
## R-sq.(adj) =  -0.00111
## lmer.REML = 4011.3  Scale est. = 0.31302   n = 2052
```

```
##                    stdcoef      stdse
## X(Intercept)    0.000000000 0.00000000
## XPDS_score      0.010569214 0.02310056
## Xinterview_age  0.006876993 0.02254210
## Xbmi           -0.016337671 0.02282931
```

2.11 Model: Caudate Anticipation ~ Testosterone

Female participants

```
## Warning: Some predictor variables are on very different scales: consider
## rescaling
```

```
##
## Family: gaussian
## Link function: identity
##
## Formula:
## caudate_rvsn_ant_z ~ hormone_scr_ert_mean + hormone_sal_end_min_since_midnight +
##     interview_age + MRI_minus_hormone_date_time + bmi
##
## Parametric coefficients:
##                                      Estimate Std. Error t value Pr(>|t|)
## (Intercept)                        -4.131e-01  3.515e-01  -1.175   0.2401
## hormone_scr_ert_mean               -1.110e-03  1.338e-03  -0.830   0.4069
## hormone_sal_end_min_since_midnight  5.851e-06  1.269e-04   0.046   0.9632
## interview_age                       5.254e-03  2.825e-03   1.860   0.0631 .
## MRI_minus_hormone_date_time        -2.420e-06  2.642e-06  -0.916   0.3599
## bmi                                -8.360e-03  5.319e-03  -1.572   0.1161
## ---
```

```
## Signif. codes:  0 '***' 0.001 '**' 0.01 '*' 0.05 '.' 0.1 ' ' 1
##
##
## R-sq.(adj) =  0.00114
## lmer.REML = 4945.8  Scale est. = 0.78585   n = 1871

##                                          stdcoef      stdse
## X(Intercept)                         0.000000000 0.00000000
## Xhormone_scr_ert_mean               -0.020165853 0.02430817
## Xhormone_sal_end_min_since_midnight  0.001154729 0.02505497
## Xinterview_age                       0.044264345 0.02380244
## XMRI_minus_hormone_date_time        -0.021956586 0.02397539
## Xbmi                                -0.037336562 0.02375245
```

Male participants

```
## Warning: Some predictor variables are on very different scales: consider
## rescaling

##
## Family: gaussian
## Link function: identity
##
## Formula:
## caudate_rvsn_ant_z ~ hormone_scr_ert_mean + hormone_sal_end_min_since_midnight +
##     interview_age + MRI_minus_hormone_date_time + bmi
##
## Parametric coefficients:
##                                      Estimate Std. Error t value Pr(>|t|)
## (Intercept)                         2.834e-02  3.761e-01   0.075    0.940
## hormone_scr_ert_mean                6.787e-04  1.492e-03   0.455    0.649
## hormone_sal_end_min_since_midnight  1.764e-05  1.301e-04   0.136    0.892
## interview_age                       4.466e-04  2.956e-03   0.151    0.880
## MRI_minus_hormone_date_time         6.314e-07  2.640e-06   0.239    0.811
## bmi                                -6.543e-03  6.162e-03  -1.062    0.288
##
##
## R-sq.(adj) =  -0.00204
## lmer.REML = 5141.2  Scale est. = 0.69596   n = 1866

##                                          stdcoef      stdse
## X(Intercept)                         0.000000000 0.00000000
## Xhormone_scr_ert_mean                0.011040055 0.02427440
## Xhormone_sal_end_min_since_midnight  0.003318252 0.02447026
## Xinterview_age                       0.003572053 0.02364120
## XMRI_minus_hormone_date_time         0.005711641 0.02388667
## Xbmi                                -0.025401730 0.02392490
```

2.12 Model B: Putamen Anticipation ~ Testosterone

Female participants

```
## Warning: Some predictor variables are on very different scales: consider
```

```
## rescaling
```

```
##
## Family: gaussian
## Link function: identity
##
## Formula:
## putamen_rvsn_ant_z ~ hormone_scr_ert_mean + hormone_sal_end_min_since_midnight +
##     interview_age + MRI_minus_hormone_date_time + bmi
##
## Parametric coefficients:
##                                      Estimate Std. Error t value Pr(>|t|)
## (Intercept)                        -1.629e-01  3.418e-01  -0.477   0.6337
## hormone_scr_ert_mean               -5.084e-04  1.301e-03  -0.391   0.6960
## hormone_sal_end_min_since_midnight -2.539e-05  1.232e-04  -0.206   0.8368
## interview_age                       3.152e-03  2.744e-03   1.149   0.2508
## MRI_minus_hormone_date_time        -1.923e-06  2.611e-06  -0.737   0.4615
## bmi                                -9.126e-03  5.169e-03  -1.765   0.0777 .
## ---
## Signif. codes:  0 '***' 0.001 '**' 0.01 '*' 0.05 '.' 0.1 ' ' 1
##
##
## R-sq.(adj) =  0.000367
## lmer.REML = 4833.3  Scale est. = 0.72897   n = 1869
```

```
##                                          stdcoef      stdse
## X(Intercept)                         0.000000000 0.00000000
## Xhormone_scr_ert_mean               -0.009513765 0.02434281
## Xhormone_sal_end_min_since_midnight -0.005163063 0.02506278
## Xinterview_age                       0.027370889 0.02382766
## XMRI_minus_hormone_date_time        -0.017680473 0.02400558
## Xbmi                                -0.042014205 0.02379815
```

Male participants

```
## Warning: Some predictor variables are on very different scales: consider
## rescaling
```

```
##
## Family: gaussian
## Link function: identity
##
## Formula:
## putamen_rvsn_ant_z ~ hormone_scr_ert_mean + hormone_sal_end_min_since_midnight +
##     interview_age + MRI_minus_hormone_date_time + bmi
##
## Parametric coefficients:
##                                      Estimate Std. Error t value Pr(>|t|)
## (Intercept)                        -2.219e-01  3.713e-01  -0.598   0.5502
## hormone_scr_ert_mean                2.171e-03  1.476e-03   1.471   0.1414
## hormone_sal_end_min_since_midnight  8.367e-06  1.307e-04   0.064   0.9490
## interview_age                       3.066e-03  2.918e-03   1.051   0.2934
## MRI_minus_hormone_date_time         1.198e-06  2.554e-06   0.469   0.6392
```

```
## bmi                               -1.171e-02  6.139e-03  -1.908   0.0566 .
## ---
## Signif. codes:  0 '***' 0.001 '**' 0.01 '*' 0.05 '.' 0.1 ' ' 1
##
##
## R-sq.(adj) =  0.000936
## lmer.REML = 5092.2  Scale est. = 0.78837   n = 1866
```

```
##                                           stdcoef      stdse
## X(Intercept)                           0.000000000 0.00000000
## Xhormone_scr_ert_mean                  0.035720186 0.02427820
## Xhormone_sal_end_min_since_midnight  0.001591163 0.02485799
## Xinterview_age                         0.024817878 0.02361571
## XMRI_minus_hormone_date_time           0.011249966 0.02399131
## Xbmi                                  -0.045667131 0.02393893
```

2.13 Model: Accumbens Anticipation ~ Testosterone

Female participants

```
## Warning: Some predictor variables are on very different scales: consider
## rescaling
```

```
##
## Family: gaussian
## Link function: identity
##
## Formula:
## accumbens_rvsn_ant_z ~ hormone_scr_ert_mean + hormone_sal_end_min_since_midnight +
##     interview_age + MRI_minus_hormone_date_time + bmi
##
## Parametric coefficients:
##                                      Estimate Std. Error t value Pr(>|t|)
## (Intercept)                         -6.371e-02  2.674e-01  -0.238   0.8117
## hormone_scr_ert_mean                -1.753e-03  1.018e-03  -1.722   0.0852 .
## hormone_sal_end_min_since_midnight -4.486e-05  9.490e-05  -0.473   0.6365
## interview_age                        2.179e-03  2.148e-03   1.015   0.3104
## MRI_minus_hormone_date_time         -2.284e-06  2.047e-06  -1.116   0.2646
## bmi                                 -4.554e-03  4.048e-03  -1.125   0.2608
## ---
## Signif. codes:  0 '***' 0.001 '**' 0.01 '*' 0.05 '.' 0.1 ' ' 1
##
##
## R-sq.(adj) =  0.00168
## lmer.REML = 3934.2  Scale est. = 0.42606   n = 1872
```

```
##                                          stdcoef      stdse
## X(Intercept)                          0.00000000 0.00000000
## Xhormone_scr_ert_mean                -0.04182931 0.02428604
## Xhormone_sal_end_min_since_midnight -0.01162708 0.02459745
## Xinterview_age                        0.02414510 0.02379851
## XMRI_minus_hormone_date_time         -0.02665098 0.02388318
## Xbmi                                 -0.02675229 0.02378200
```

Male participants

```
## Warning: Some predictor variables are on very different scales: consider
## rescaling
```

```
##
## Family: gaussian
## Link function: identity
##
## Formula:
## accumbens_rvsn_ant_z ~ hormone_scr_ert_mean + hormone_sal_end_min_since_midnight +
##     interview_age + MRI_minus_hormone_date_time + bmi
##
## Parametric coefficients:
##                                     Estimate Std. Error t value Pr(>|t|)
## (Intercept)                        5.445e-01  2.946e-01   1.848   0.0647 .
## hormone_scr_ert_mean              -1.592e-04  1.152e-03  -0.138   0.8900
## hormone_sal_end_min_since_midnight -2.137e-04  9.906e-05  -2.158   0.0311 *
## interview_age                     -2.828e-03  2.311e-03  -1.224   0.2212
## MRI_minus_hormone_date_time        2.802e-06  2.015e-06   1.390   0.1646
## bmi                               -2.069e-03  4.806e-03  -0.430   0.6670
## ---
## Signif. codes:  0 '***' 0.001 '**' 0.01 '*' 0.05 '.' 0.1 ' ' 1
##
##
## R-sq.(adj) =  0.00124
## lmer.REML = 4253.1  Scale est. = 0.50728   n = 1869
```

```
##                                         stdcoef      stdse
## X(Intercept)                        0.000000000 0.00000000
## Xhormone_scr_ert_mean              -0.003310724 0.02394501
## Xhormone_sal_end_min_since_midnight -0.051031389 0.02365046
## Xinterview_age                     -0.028761585 0.02350046
## XMRI_minus_hormone_date_time        0.032768063 0.02356834
## Xbmi                               -0.010203115 0.02370620
```

2.14 Model: Caudate Feedback ~ Testosterone

Female participants

```
## Warning: Some predictor variables are on very different scales: consider
## rescaling
```

```
##
## Family: gaussian
## Link function: identity
##
## Formula:
## caudate_posvsneg_feedback_z ~ hormone_scr_ert_mean + hormone_sal_end_min_since_midnight +
##     interview_age + MRI_minus_hormone_date_time + bmi
##
## Parametric coefficients:
```

```
##                                     Estimate Std. Error t value Pr(>|t|)
## (Intercept)                        1.296e+00  3.374e-01   3.841 0.000127 ***
## hormone_scr_ert_mean               2.761e-03  1.274e-03   2.168 0.030314 *
## hormone_sal_end_min_since_midnight -2.376e-04  1.146e-04  -2.072 0.038379 *
## interview_age                      -9.490e-03  2.702e-03  -3.512 0.000455 ***
## MRI_minus_hormone_date_time        -2.304e-06  2.511e-06  -0.918 0.358905
## bmi                                -5.211e-03  5.077e-03  -1.026 0.304867
## ---
## Signif. codes:  0 '***' 0.001 '**' 0.01 '*' 0.05 '.' 0.1 ' ' 1
##
##
## R-sq.(adj) =  0.00871
## lmer.REML = 4788.9  Scale est. = 0.73183   n = 1868
```

```
##                                        stdcoef      stdse
## X(Intercept)                         0.00000000 0.00000000
## Xhormone_scr_ert_mean                0.05217795 0.02407149
## Xhormone_sal_end_min_since_midnight -0.04883571 0.02356651
## Xinterview_age                      -0.08309510 0.02365892
## XMRI_minus_hormone_date_time        -0.02158981 0.02352648
## Xbmi                                -0.02416453 0.02354451
```

Male participants

```
## Warning: Some predictor variables are on very different scales: consider
## rescaling
```

```
##
## Family: gaussian
## Link function: identity
##
## Formula:
## caudate_posvsneg_feedback_z ~ hormone_scr_ert_mean + hormone_sal_end_min_since_midnight +
##     interview_age + MRI_minus_hormone_date_time + bmi
##
## Parametric coefficients:
##                                     Estimate Std. Error t value Pr(>|t|)
## (Intercept)                        1.439e-01  3.485e-01   0.413    0.680
## hormone_scr_ert_mean               1.770e-03  1.369e-03   1.293    0.196
## hormone_sal_end_min_since_midnight -1.068e-04  1.190e-04  -0.898    0.369
## interview_age                      -4.641e-04  2.732e-03  -0.170    0.865
## MRI_minus_hormone_date_time         1.160e-06  2.382e-06   0.487    0.626
## bmi                                -1.163e-03  5.686e-03  -0.204    0.838
##
##
## R-sq.(adj) =  -0.00117
## lmer.REML = 4856.9  Scale est. = 0.76017   n = 1866
```

```
##                                         stdcoef      stdse
## X(Intercept)                         0.000000000 0.00000000
## Xhormone_scr_ert_mean                0.031144472 0.02408678
## Xhormone_sal_end_min_since_midnight -0.021626525 0.02408044
## Xinterview_age                      -0.004006366 0.02357831
```

```
## XMRI_minus_hormone_date_time         0.011565584 0.02375504
## Xbmi                                -0.004865033 0.02379009
```

2.15 Model: Putamen Feedback ~ Testosterone

Female participants

```
## Warning: Some predictor variables are on very different scales: consider
## rescaling
```

```
##
## Family: gaussian
## Link function: identity
##
## Formula:
## putamen_posvsneg_feedback_z ~ hormone_scr_ert_mean + hormone_sal_end_min_since_midnight +
##     interview_age + MRI_minus_hormone_date_time + bmi
##
## Parametric coefficients:
##                                     Estimate Std. Error t value Pr(>|t|)
## (Intercept)                        1.027e+00  3.206e-01   3.203  0.00138 **
## hormone_scr_ert_mean               3.559e-03  1.217e-03   2.924  0.00350 **
## hormone_sal_end_min_since_midnight -3.133e-04  1.115e-04  -2.810  0.00501 **
## interview_age                     -6.831e-03  2.571e-03  -2.657  0.00794 **
## MRI_minus_hormone_date_time       -9.577e-07  2.411e-06  -0.397  0.69121
## bmi                               -8.030e-03  4.844e-03  -1.658  0.09752 .
## ---
## Signif. codes:  0 '***' 0.001 '**' 0.01 '*' 0.05 '.' 0.1 ' ' 1
##
##
## R-sq.(adj) =  0.0102
## lmer.REML = 4604.2  Scale est. = 0.65935   n = 1870
```

```
##                                          stdcoef      stdse
## X(Intercept)                         0.000000000 0.00000000
## Xhormone_scr_ert_mean                0.070478249 0.02410291
## Xhormone_sal_end_min_since_midnight -0.067678751 0.02408893
## Xinterview_age                      -0.062898832 0.02366853
## XMRI_minus_hormone_date_time        -0.009404062 0.02367122
## Xbmi                                -0.039065659 0.02356425
```

Male participants

```
## Warning: Some predictor variables are on very different scales: consider
## rescaling
```

```
##
## Family: gaussian
## Link function: identity
##
## Formula:
## putamen_posvsneg_feedback_z ~ hormone_scr_ert_mean + hormone_sal_end_min_since_midnight +
```

```
##     interview_age + MRI_minus_hormone_date_time + bmi
##
## Parametric coefficients:
##                                      Estimate Std. Error t value Pr(>|t|)
## (Intercept)                         3.268e-01  3.456e-01   0.945    0.345
## hormone_scr_ert_mean                1.351e-03  1.368e-03   0.988    0.323
## hormone_sal_end_min_since_midnight  5.670e-05  1.241e-04   0.457    0.648
## interview_age                      -3.079e-03  2.711e-03  -1.136    0.256
## MRI_minus_hormone_date_time        -6.249e-07  2.404e-06  -0.260    0.795
## bmi                                 1.924e-03  5.672e-03   0.339    0.734
##
##
## R-sq.(adj) =  -0.00208
## lmer.REML = 4841.2  Scale est. = 0.72422   n = 1873
```

```
##                                          stdcoef      stdse
## X(Intercept)                         0.000000000 0.00000000
## Xhormone_scr_ert_mean                0.023972804 0.02426724
## Xhormone_sal_end_min_since_midnight  0.011552655 0.02527971
## Xinterview_age                      -0.026720647 0.02352598
## XMRI_minus_hormone_date_time        -0.006255488 0.02406265
## Xbmi                                 0.008108457 0.02389625
```

2.16 Model: Accumbens Feedback ~ Testosterone

Female participants

```
## Warning: Some predictor variables are on very different scales: consider
## rescaling
```

```
##
## Family: gaussian
## Link function: identity
##
## Formula:
## accumbens_posvsneg_feedback_z ~ hormone_scr_ert_mean + hormone_sal_end_min_since_midnight +
##     interview_age + MRI_minus_hormone_date_time + bmi
##
## Parametric coefficients:
##                                      Estimate Std. Error t value Pr(>|t|)
## (Intercept)                         6.492e-01  2.479e-01   2.619  0.00889 **
## hormone_scr_ert_mean                9.490e-05  9.387e-04   0.101  0.91948
## hormone_sal_end_min_since_midnight -1.564e-04  8.426e-05  -1.857  0.06351 .
## interview_age                      -4.647e-03  1.988e-03  -2.338  0.01950 *
## MRI_minus_hormone_date_time        -5.256e-06  1.845e-06  -2.849  0.00443 **
## bmi                                 9.609e-04  3.728e-03   0.258  0.79661
## ---
## Signif. codes:  0 '***' 0.001 '**' 0.01 '*' 0.05 '.' 0.1 ' ' 1
##
##
## R-sq.(adj) =  0.00765
## lmer.REML =   3671  Scale est. = 0.39826   n = 1876
```

```
##                                         stdcoef      stdse
## X(Intercept)                        0.000000000 0.00000000
## Xhormone_scr_ert_mean               0.002426367 0.02399983
## Xhormone_sal_end_min_since_midnight -0.043613946 0.02348969
## Xinterview_age                      -0.055202633 0.02361172
## XMRI_minus_hormone_date_time        -0.066897111 0.02348144
## Xbmi                                0.006057051 0.02349771
```

Male participants

```
## Warning: Some predictor variables are on very different scales: consider
## rescaling
```

```
##
## Family: gaussian
## Link function: identity
##
## Formula:
## accumbens_posvsneg_feedback_z ~ hormone_scr_ert_mean + hormone_sal_end_min_since_midnight +
##     interview_age + MRI_minus_hormone_date_time + bmi
##
## Parametric coefficients:
##                                      Estimate Std. Error t value Pr(>|t|)
## (Intercept)                         1.629e-01  2.839e-01   0.574    0.566
## hormone_scr_ert_mean                1.434e-03  1.120e-03   1.281    0.200
## hormone_sal_end_min_since_midnight -1.394e-04  9.824e-05  -1.419    0.156
## interview_age                      -2.851e-04  2.230e-03  -0.128    0.898
## MRI_minus_hormone_date_time         6.904e-07  1.941e-06   0.356    0.722
## bmi                                -6.762e-04  4.637e-03  -0.146    0.884
##
##
## R-sq.(adj) =  -6.14e-05
## lmer.REML = 4084.4  Scale est. = 0.43166   n = 1865
```

```
##                                         stdcoef      stdse
## X(Intercept)                        0.000000000 0.00000000
## Xhormone_scr_ert_mean               0.031050948 0.02424320
## Xhormone_sal_end_min_since_midnight -0.034750330 0.02448307
## Xinterview_age                      -0.003021811 0.02364248
## XMRI_minus_hormone_date_time        0.008493056 0.02388391
## Xbmi                                -0.003484422 0.02389012
```

2.17 Model: OFC Anticipation ~ Testosterone

Female participants

```
## Warning: Some predictor variables are on very different scales: consider
## rescaling
```

```
##
## Family: gaussian
## Link function: identity
```

```
##
## Formula:
## lOFC_rvsn_ant_z ~ hormone_scr_ert_mean + hormone_sal_end_min_since_midnight +
##     interview_age + MRI_minus_hormone_date_time + bmi
##
## Parametric coefficients:
##                                      Estimate Std. Error t value Pr(>|t|)
## (Intercept)                        -6.118e-02  2.256e-01  -0.271    0.786
## hormone_scr_ert_mean               -6.916e-04  8.561e-04  -0.808    0.419
## hormone_sal_end_min_since_midnight  5.984e-05  7.680e-05   0.779    0.436
## interview_age                       5.826e-04  1.813e-03   0.321    0.748
## MRI_minus_hormone_date_time         5.911e-08  1.678e-06   0.035    0.972
## bmi                                -9.242e-04  3.406e-03  -0.271    0.786
##
##
## R-sq.(adj) =  -0.00185
## lmer.REML = 3291.7  Scale est. = 0.3042    n = 1867

##                                           stdcoef      stdse
## X(Intercept)                         0.0000000000 0.00000000
## Xhormone_scr_ert_mean               -0.0195555832 0.02420616
## Xhormone_sal_end_min_since_midnight  0.0184728614 0.02371027
## Xinterview_age                       0.0076564336 0.02382798
## XMRI_minus_hormone_date_time         0.0008339757 0.02367074
## Xbmi                                -0.0064317078 0.02370743

## Warning: Some predictor variables are on very different scales: consider
## rescaling

##
## Family: gaussian
## Link function: identity
##
## Formula:
## mOFC_rvsn_ant_z ~ hormone_scr_ert_mean + hormone_sal_end_min_since_midnight +
##     interview_age + MRI_minus_hormone_date_time + bmi
##
## Parametric coefficients:
##                                      Estimate Std. Error t value Pr(>|t|)
## (Intercept)                        -1.179e-02  2.585e-01  -0.046    0.964
## hormone_scr_ert_mean               -1.737e-04  9.810e-04  -0.177    0.859
## hormone_sal_end_min_since_midnight  3.276e-06  8.775e-05   0.037    0.970
## interview_age                      -2.118e-04  2.077e-03  -0.102    0.919
## MRI_minus_hormone_date_time         1.985e-06  1.924e-06   1.032    0.302
## bmi                                 1.800e-03  3.888e-03   0.463    0.643
##
##
## R-sq.(adj) =  -0.00193
## lmer.REML = 3803.6  Scale est. = 0.43166   n = 1867

##                                           stdcoef      stdse
## X(Intercept)                         0.0000000000 0.00000000
## Xhormone_scr_ert_mean               -0.0042810095 0.02418097
```

```
## Xhormone_sal_end_min_since_midnight  0.0008833116 0.02366310
## Xinterview_age                      -0.0024282679 0.02380880
## XMRI_minus_hormone_date_time         0.0244069852 0.02365490
## Xbmi                                 0.0109494660 0.02365223
```

Male participants

```
## Warning: Some predictor variables are on very different scales: consider
## rescaling
```

```
##
## Family: gaussian
## Link function: identity
##
## Formula:
## lOFC_rvsn_ant_z ~ hormone_scr_ert_mean + hormone_sal_end_min_since_midnight +
##     interview_age + MRI_minus_hormone_date_time + bmi
##
## Parametric coefficients:
##                                      Estimate Std. Error t value Pr(>|t|)
## (Intercept)                        -2.952e-01  2.455e-01  -1.202   0.2294
## hormone_scr_ert_mean               -1.505e-03  9.730e-04  -1.547   0.1220
## hormone_sal_end_min_since_midnight  1.614e-05  8.437e-05   0.191   0.8483
## interview_age                       2.651e-03  1.931e-03   1.373   0.1699
## MRI_minus_hormone_date_time         3.029e-06  1.675e-06   1.808   0.0708 .
## bmi                                 9.285e-04  4.033e-03   0.230   0.8180
## ---
## Signif. codes:  0 '***' 0.001 '**' 0.01 '*' 0.05 '.' 0.1 ' ' 1
##
##
## R-sq.(adj) =  0.00138
## lmer.REML = 3554.1  Scale est. = 0.34152   n = 1863
```

```
##                                          stdcoef      stdse
## X(Intercept)                         0.000000000 0.00000000
## Xhormone_scr_ert_mean               -0.037327374 0.02412924
## Xhormone_sal_end_min_since_midnight  0.004635715 0.02422618
## Xinterview_age                       0.032394609 0.02359038
## XMRI_minus_hormone_date_time         0.043014155 0.02378963
## Xbmi                                 0.005488416 0.02384091
```

```
## Warning: Some predictor variables are on very different scales: consider
## rescaling
```

```
##
## Family: gaussian
## Link function: identity
##
## Formula:
## mOFC_rvsn_ant_z ~ hormone_scr_ert_mean + hormone_sal_end_min_since_midnight +
##     interview_age + MRI_minus_hormone_date_time + bmi
##
```

```
## Parametric coefficients:
##                                      Estimate Std. Error t value Pr(>|t|)
## (Intercept)                        -2.791e-01  2.691e-01  -1.037    0.300
## hormone_scr_ert_mean               -1.421e-03  1.058e-03  -1.343    0.180
## hormone_sal_end_min_since_midnight -1.999e-05  9.120e-05  -0.219    0.827
## interview_age                       1.886e-03  2.115e-03   0.892    0.373
## MRI_minus_hormone_date_time         2.427e-06  1.880e-06   1.291    0.197
## bmi                                 6.023e-03  4.392e-03   1.371    0.170
##
##
## R-sq.(adj) =  0.000297
## lmer.REML = 3891.6  Scale est. = 0.42825   n = 1860
```

```
##                                          stdcoef      stdse
## X(Intercept)                         0.000000000 0.00000000
## Xhormone_scr_ert_mean               -0.032297331 0.02405353
## Xhormone_sal_end_min_since_midnight -0.005234212 0.02388414
## Xinterview_age                       0.021033549 0.02359092
## XMRI_minus_hormone_date_time         0.030604893 0.02370390
## Xbmi                                 0.032619459 0.02378770
```

2.18 Model: OFC Feedback ~ Testosterone

Female participants

```
## Warning: Some predictor variables are on very different scales: consider
## rescaling
```

```
##
## Family: gaussian
## Link function: identity
##
## Formula:
## lOFC_posvsneg_feedback_z ~ hormone_scr_ert_mean + hormone_sal_end_min_since_midnight +
##     interview_age + MRI_minus_hormone_date_time + bmi
##
## Parametric coefficients:
##                                      Estimate Std. Error t value Pr(>|t|)
## (Intercept)                         4.104e-01  1.959e-01   2.096   0.0363 *
## hormone_scr_ert_mean                9.438e-04  7.423e-04   1.271   0.2037
## hormone_sal_end_min_since_midnight -1.112e-04  6.693e-05  -1.661   0.0969 .
## interview_age                      -3.061e-03  1.572e-03  -1.947   0.0517 .
## MRI_minus_hormone_date_time        -1.906e-06  1.455e-06  -1.310   0.1905
## bmi                                -8.271e-04  2.957e-03  -0.280   0.7797
## ---
## Signif. codes:  0 '***' 0.001 '**' 0.01 '*' 0.05 '.' 0.1 ' ' 1
##
##
## R-sq.(adj) =  0.00286
## lmer.REML = 2773.3  Scale est. = 0.21187   n = 1869
```

```
##                                          stdcoef      stdse
```

```
## X(Intercept)                          0.000000000 0.00000000
## Xhormone_scr_ert_mean                 0.030695473 0.02414145
## Xhormone_sal_end_min_since_midnight -0.039301721 0.02366452
## Xinterview_age                       -0.046216400 0.02373422
## XMRI_minus_hormone_date_time         -0.030898481 0.02359265
## Xbmi                                 -0.006625893 0.02368836
```

```
## Warning: Some predictor variables are on very different scales: consider
## rescaling
```

```
##
## Family: gaussian
## Link function: identity
##
## Formula:
## mOFC_posvsneg_feedback_z ~ hormone_scr_ert_mean + hormone_sal_end_min_since_midnight +
##     interview_age + MRI_minus_hormone_date_time + bmi
##
## Parametric coefficients:
##                                      Estimate Std. Error t value Pr(>|t|)
## (Intercept)                         2.387e-01  2.399e-01   0.995    0.320
## hormone_scr_ert_mean                4.352e-04  9.124e-04   0.477    0.633
## hormone_sal_end_min_since_midnight -1.574e-04  8.400e-05  -1.874    0.061 .
## interview_age                      -1.437e-03  1.928e-03  -0.746    0.456
## MRI_minus_hormone_date_time        -2.352e-06  1.793e-06  -1.312    0.190
## bmi                                 1.888e-03  3.632e-03   0.520    0.603
## ---
## Signif. codes:  0 '***' 0.001 '**' 0.01 '*' 0.05 '.' 0.1 ' ' 1
##
##
## R-sq.(adj) =  0.00157
## lmer.REML = 3524.1  Scale est. = 0.32039   n = 1871
```

```
##                                         stdcoef      stdse
## X(Intercept)                          0.00000000 0.00000000
## Xhormone_scr_ert_mean                 0.01158192 0.02427999
## Xhormone_sal_end_min_since_midnight -0.04565296 0.02435698
## Xinterview_age                      -0.01775186 0.02380678
## XMRI_minus_hormone_date_time        -0.03124780 0.02381339
## Xbmi                                  0.01236737 0.02379367
```

Male participants

```
## Warning: Some predictor variables are on very different scales: consider
## rescaling
```

```
##
## Family: gaussian
## Link function: identity
##
## Formula:
## lOFC_posvsneg_feedback_z ~ hormone_scr_ert_mean + hormone_sal_end_min_since_midnight +
```

```
##     interview_age + MRI_minus_hormone_date_time + bmi
##
## Parametric coefficients:
##                                     Estimate Std. Error t value Pr(>|t|)
## (Intercept)                        7.325e-03  2.203e-01   0.033   0.9735
## hormone_scr_ert_mean               2.564e-04  8.656e-04   0.296   0.7671
## hormone_sal_end_min_since_midnight -6.635e-05  7.414e-05  -0.895   0.3709
## interview_age                      1.773e-03  1.726e-03   1.027   0.3046
## MRI_minus_hormone_date_time        1.293e-06  1.497e-06   0.864   0.3879
## bmi                               -7.623e-03  3.600e-03  -2.117   0.0344 *
## ---
## Signif. codes:  0 '***' 0.001 '**' 0.01 '*' 0.05 '.' 0.1 ' ' 1
##
##
## R-sq.(adj) =  0.000999
## lmer.REML = 3181.6  Scale est. = 0.30739   n = 1873
```

```
##                                         stdcoef      stdse
## X(Intercept)                        0.000000000 0.00000000
## Xhormone_scr_ert_mean               0.007082771 0.02391501
## Xhormone_sal_end_min_since_midnight -0.021149325 0.02363006
## Xinterview_age                      0.024114046 0.02348010
## XMRI_minus_hormone_date_time        0.020335953 0.02354489
## Xbmi                               -0.050116822 0.02367017
```

```
## Warning: Some predictor variables are on very different scales: consider
## rescaling
```

```
##
## Family: gaussian
## Link function: identity
##
## Formula:
## mOFC_posvsneg_feedback_z ~ hormone_scr_ert_mean + hormone_sal_end_min_since_midnight +
##     interview_age + MRI_minus_hormone_date_time + bmi
##
## Parametric coefficients:
##                                     Estimate Std. Error t value Pr(>|t|)
## (Intercept)                        1.308e-01  2.563e-01   0.510    0.610
## hormone_scr_ert_mean               9.590e-04  1.007e-03   0.952    0.341
## hormone_sal_end_min_since_midnight -8.785e-05  8.646e-05  -1.016    0.310
## interview_age                      8.845e-05  2.012e-03   0.044    0.965
## MRI_minus_hormone_date_time        1.915e-06  1.741e-06   1.100    0.272
## bmi                               -3.633e-03  4.203e-03  -0.864    0.388
##
##
## R-sq.(adj) =  -0.000922
## lmer.REML = 3739.8  Scale est. = 0.34001   n = 1871
```

```
##                                         stdcoef      stdse
## X(Intercept)                        0.000000000 0.00000000
## Xhormone_scr_ert_mean               0.022874033 0.02403070
## Xhormone_sal_end_min_since_midnight -0.024101760 0.02372145
```

```
## Xinterview_age                        0.001035309 0.02355457
## XMRI_minus_hormone_date_time          0.025972845 0.02361983
## Xbmi                                 -0.020555470 0.02378329
```

2.19 Model: MID Reaction Time ~ Testosterone

Female participants

```
##
## Family: gaussian
## Link function: identity
##
## Formula:
## rt_diff_large_neutral_z ~ hormone_scr_ert_mean + hormone_sal_end_min_since_midnight +
##     interview_age + bmi
##
## Parametric coefficients:
##                                      Estimate Std. Error t value Pr(>|t|)
## (Intercept)                        -5.457e-01  3.479e-01  -1.569   0.1169
## hormone_scr_ert_mean               -1.281e-03  1.315e-03  -0.974   0.3303
## hormone_sal_end_min_since_midnight  4.443e-05  1.162e-04   0.382   0.7023
## interview_age                       5.703e-03  2.772e-03   2.057   0.0398 *
## bmi                                -3.898e-03  5.263e-03  -0.741   0.4590
## ---
## Signif. codes:  0 '***' 0.001 '**' 0.01 '*' 0.05 '.' 0.1 ' ' 1
##
##
## R-sq.(adj) =  0.000947
## lmer.REML = 5481.2  Scale est. = 0.68951   n = 2034
```

```
##                                          stdcoef      stdse
## X(Intercept)                          0.000000000 0.00000000
## Xhormone_scr_ert_mean                -0.022538378 0.02314437
## Xhormone_sal_end_min_since_midnight   0.008534295 0.02232871
## Xinterview_age                        0.046787863 0.02274280
## Xbmi                                 -0.016828938 0.02272304
```

```
##
## Family: gaussian
## Link function: identity
##
## Formula:
## rt_diff_large_small_z ~ hormone_scr_ert_mean + hormone_sal_end_min_since_midnight +
##     interview_age + bmi
##
## Parametric coefficients:
##                                      Estimate Std. Error t value Pr(>|t|)
## (Intercept)                        -1.701e-01  3.521e-01  -0.483   0.6291
## hormone_scr_ert_mean               -3.369e-04  1.330e-03  -0.253   0.8001
## hormone_sal_end_min_since_midnight  2.681e-06  1.175e-04   0.023   0.9818
## interview_age                       3.043e-03  2.805e-03   1.085   0.2782
## bmi                                -9.074e-03  5.321e-03  -1.705   0.0883 .
## ---
```

```
## Signif. codes:  0 '***' 0.001 '**' 0.01 '*' 0.05 '.' 0.1 ' ' 1
##
##
## R-sq.(adj) =  0.000203
## lmer.REML = 5530.1  Scale est. = 0.73904   n = 2034
```

```
##                                         stdcoef      stdse
## X(Intercept)                          0.0000000000 0.00000000
## Xhormone_scr_ert_mean                -0.0058619625 0.02314682
## Xhormone_sal_end_min_since_midnight   0.0005091501 0.02231943
## Xinterview_age                        0.0246812203 0.02275458
## Xbmi                                 -0.0387311422 0.02271408
```

Male participants

```
##
## Family: gaussian
## Link function: identity
##
## Formula:
## rt_diff_large_neutral_z ~ hormone_scr_ert_mean + hormone_sal_end_min_since_midnight +
##     interview_age + bmi
##
## Parametric coefficients:
##                                       Estimate Std. Error t value Pr(>|t|)
## (Intercept)                          1.511e-01  3.280e-01   0.461    0.645
## hormone_scr_ert_mean                -6.230e-04  1.287e-03  -0.484    0.629
## hormone_sal_end_min_since_midnight -2.515e-05  1.116e-04  -0.225    0.822
## interview_age                       -6.289e-04  2.580e-03  -0.244    0.807
## bmi                                 -2.519e-03  5.336e-03  -0.472    0.637
##
##
## R-sq.(adj) =  -0.00145
## lmer.REML = 5502.7  Scale est. = 0.70817   n = 2117
```

```
##                                        stdcoef      stdse
## X(Intercept)                          0.000000000 0.00000000
## Xhormone_scr_ert_mean                -0.010967953 0.02266533
## Xhormone_sal_end_min_since_midnight -0.005061990 0.02246235
## Xinterview_age                       -0.005409866 0.02219243
## Xbmi                                 -0.010554257 0.02235663
```

```
##
## Family: gaussian
## Link function: identity
##
## Formula:
## rt_diff_large_small_z ~ hormone_scr_ert_mean + hormone_sal_end_min_since_midnight +
##     interview_age + bmi
##
## Parametric coefficients:
##                                       Estimate Std. Error t value Pr(>|t|)
## (Intercept)                          1.111e-01  3.305e-01   0.336    0.737
```

```
## hormone_scr_ert_mean               -1.470e-03  1.287e-03  -1.142    0.253
## hormone_sal_end_min_since_midnight -9.374e-06  1.097e-04  -0.085    0.932
## interview_age                      -8.818e-04  2.596e-03  -0.340    0.734
## bmi                                 3.426e-03  5.357e-03   0.640    0.523
##
##
## R-sq.(adj) =  -0.00108
## lmer.REML = 5547.2  Scale est. = 0.78654   n = 2117
```

```
##                                        stdcoef      stdse
## X(Intercept)                         0.000000000 0.00000000
## Xhormone_scr_ert_mean               -0.025610298 0.02242158
## Xhormone_sal_end_min_since_midnight -0.001867229 0.02184855
## Xinterview_age                      -0.007507695 0.02210472
## Xbmi                                 0.014206864 0.02221422
```

2.20 Model: BIS-BAS-RR ~ Testosterone

Female participants

```
##
## Family: gaussian
## Link function: identity
##
## Formula:
## bisbas_ss_basm_rr_z ~ hormone_scr_ert_mean + hormone_sal_end_min_since_midnight +
##     interview_age + bmi
##
## Parametric coefficients:
##                                      Estimate Std. Error t value Pr(>|t|)
## (Intercept)                         2.064e-01  3.388e-01   0.609   0.5423
## hormone_scr_ert_mean               -1.325e-03  1.281e-03  -1.034   0.3011
## hormone_sal_end_min_since_midnight -1.526e-05  1.217e-04  -0.125   0.9002
## interview_age                      -3.198e-03  2.705e-03  -1.182   0.2372
## bmi                                 1.008e-02  5.067e-03   1.990   0.0468 *
## ---
## Signif. codes:  0 '***' 0.001 '**' 0.01 '*' 0.05 '.' 0.1 ' ' 1
##
##
## R-sq.(adj) =  0.00212
## lmer.REML = 6973.1  Scale est. = 0.70698   n = 2472
```

```
##                                        stdcoef      stdse
## X(Intercept)                         0.000000000 0.00000000
## Xhormone_scr_ert_mean               -0.021900218 0.02117234
## Xhormone_sal_end_min_since_midnight -0.002741078 0.02185022
## Xinterview_age                      -0.024451234 0.02067978
## Xbmi                                 0.041394319 0.02080619
```

Male participants

```
##
```

```
## Family: gaussian
## Link function: identity
##
## Formula:
## bisbas_ss_basm_rr_z ~ hormone_scr_ert_mean + hormone_sal_end_min_since_midnight +
##     interview_age + bmi
##
## Parametric coefficients:
##                                      Estimate Std. Error t value Pr(>|t|)
## (Intercept)                        -0.1642160  0.3202918  -0.513  0.60820
## hormone_scr_ert_mean                0.0013778  0.0012667   1.088  0.27681
## hormone_sal_end_min_since_midnight  0.0002448  0.0001142   2.145  0.03205 *
## interview_age                      -0.0023744  0.0025328  -0.937  0.34861
## bmi                                 0.0153514  0.0051832   2.962  0.00309 **
## ---
## Signif. codes:  0 '***' 0.001 '**' 0.01 '*' 0.05 '.' 0.1 ' ' 1
##
##
## R-sq.(adj) =  0.00678
## lmer.REML = 7395.5  Scale est. = 0.70481   n = 2670
```

```
##                                         stdcoef      stdse
## X(Intercept)                         0.00000000 0.00000000
## Xhormone_scr_ert_mean                0.02218857 0.02039898
## Xhormone_sal_end_min_since_midnight  0.04455039 0.02077038
## Xinterview_age                      -0.01852066 0.01975645
## Xbmi                                 0.05908547 0.01994935
```

3—Internalizing~Reward—

3.1 Model: CBCL internalizing factor ~ Nucleus Accumbens activity (anticipation stage)

Female participants

```
##
## Family: gaussian
## Link function: identity
##
## Formula:
## cbcl_scr_syn_internal_r ~ accumbens_rvsn_ant_z + interview_age
##
## Parametric coefficients:
##                      Estimate Std. Error t value Pr(>|t|)
## (Intercept)           6.03142    1.86213   3.239  0.00122 **
## accumbens_rvsn_ant_z -0.04281    0.16866  -0.254  0.79968
## interview_age        -0.00972    0.01557  -0.624  0.53257
## ---
## Signif. codes:  0 '***' 0.001 '**' 0.01 '*' 0.05 '.' 0.1 ' ' 1
##
##
## R-sq.(adj) =  -0.000632
## lmer.REML =  12626  Scale est. = 11.232    n = 2044
```

```
##                            stdcoef      stdse
## X(Intercept)           0.000000000 0.00000000
## Xaccumbens_rvsn_ant_z -0.005427053 0.02138326
## Xinterview_age        -0.013435302 0.02152431
```

Male participants

```
##
## Family: gaussian
## Link function: identity
##
## Formula:
## cbcl_scr_syn_internal_r ~ accumbens_rvsn_ant_z + interview_age
##
## Parametric coefficients:
##                      Estimate Std. Error t value Pr(>|t|)
## (Intercept)           3.25746    1.86102   1.750   0.0802 .
## accumbens_rvsn_ant_z -0.14197    0.15732  -0.902   0.3669
## interview_age         0.01252    0.01550   0.808   0.4195
## ---
## Signif. codes:  0 '***' 0.001 '**' 0.01 '*' 0.05 '.' 0.1 ' ' 1
##
##
## R-sq.(adj) =  -0.000586
## lmer.REML =  12672  Scale est. = 18.374    n = 2059
```

```
##                          stdcoef      stdse
## X(Intercept)           0.00000000 0.00000000
## Xaccumbens_rvsn_ant_z -0.01970131 0.02183160
## Xinterview_age         0.01786603 0.02212499
```

3.2 Model: CBCL internalizing factor ~ Caudate activity (anticipation stage)

Female participants

```
##
## Family: gaussian
## Link function: identity
##
## Formula:
## cbcl_scr_syn_internal_r ~ caudate_rvsn_ant_z + interview_age
##
## Parametric coefficients:
##                    Estimate Std. Error t value Pr(>|t|)
## (Intercept)         6.09957    1.86424   3.272  0.00109 **
## caudate_rvsn_ant_z -0.02686    0.12935  -0.208  0.83552
## interview_age      -0.01023    0.01559  -0.656  0.51174
## ---
## Signif. codes:  0 '***' 0.001 '**' 0.01 '*' 0.05 '.' 0.1 ' ' 1
##
##
## R-sq.(adj) =  -0.000647
## lmer.REML =  12629  Scale est. = 11.292    n = 2044
```

```
##                         stdcoef      stdse
## X(Intercept)         0.000000000 0.00000000
## Xcaudate_rvsn_ant_z -0.004436974 0.02136731
## Xinterview_age      -0.014126520 0.02152610
```

Male participants

```
##
## Family: gaussian
## Link function: identity
##
## Formula:
## cbcl_scr_syn_internal_r ~ caudate_rvsn_ant_z + interview_age
##
## Parametric coefficients:
##                    Estimate Std. Error t value Pr(>|t|)
## (Intercept)         3.49750    1.86404   1.876   0.0608 .
## caudate_rvsn_ant_z -0.10650    0.12325  -0.864   0.3876
## interview_age       0.01067    0.01552   0.687   0.4919
## ---
## Signif. codes:  0 '***' 0.001 '**' 0.01 '*' 0.05 '.' 0.1 ' ' 1
##
##
## R-sq.(adj) =  -0.000812
## lmer.REML =  12656  Scale est. = 18.614    n = 2056
```

```
##                           stdcoef      stdse
## X(Intercept)         0.00000000 0.00000000
## Xcaudate_rvsn_ant_z -0.01892928 0.02190619
## Xinterview_age       0.01521727 0.02213864
```

3.3 Model: CBCL internalizing factor ~ Putamen activity (anticipation stage)

Female participants

```
##
## Family: gaussian
## Link function: identity
##
## Formula:
## cbcl_scr_syn_internal_r ~ putamen_rvsn_ant_z + interview_age
##
## Parametric coefficients:
##                     Estimate Std. Error t value Pr(>|t|)
## (Intercept)         5.934388   1.857662   3.195  0.00142 **
## putamen_rvsn_ant_z -0.095198   0.132427  -0.719  0.47230
## interview_age      -0.008957   0.015537  -0.577  0.56434
## ---
## Signif. codes:  0 '***' 0.001 '**' 0.01 '*' 0.05 '.' 0.1 ' ' 1
##
##
## R-sq.(adj) =  -0.000598
## lmer.REML =  12595  Scale est. = 11.211    n = 2041
```

```
##                           stdcoef      stdse
## X(Intercept)         0.00000000 0.00000000
## Xputamen_rvsn_ant_z -0.01538262 0.02139824
## Xinterview_age      -0.01242203 0.02154710
```

Male participants

```
##
## Family: gaussian
## Link function: identity
##
## Formula:
## cbcl_scr_syn_internal_r ~ putamen_rvsn_ant_z + interview_age
##
## Parametric coefficients:
##                    Estimate Std. Error t value Pr(>|t|)
## (Intercept)         3.35048    1.85734   1.804   0.0714 .
## putamen_rvsn_ant_z -0.15563    0.12301  -1.265   0.2060
## interview_age       0.01181    0.01547   0.763   0.4453
## ---
## Signif. codes:  0 '***' 0.001 '**' 0.01 '*' 0.05 '.' 0.1 ' ' 1
##
##
## R-sq.(adj) =  -0.000471
## lmer.REML =  12647  Scale est. = 18.132    n = 2057
```

```
##                          stdcoef      stdse
## X(Intercept)         0.00000000 0.00000000
## Xputamen_rvsn_ant_z -0.02763637 0.02184387
## Xinterview_age       0.01688681 0.02212169
```

3.4 Model: CBCL internalizing factor ~ Accumbens activity (feedback stage)

Female participants

```
##
## Family: gaussian
## Link function: identity
##
## Formula:
## cbcl_scr_syn_internal_r ~ accumbens_posvsneg_feedback_z + interview_age
##
## Parametric coefficients:
##                                Estimate Std. Error t value Pr(>|t|)
## (Intercept)                    5.830691   1.856144   3.141  0.00171 **
## accumbens_posvsneg_feedback_z -0.050476   0.176282  -0.286  0.77465
## interview_age                 -0.008162   0.015527  -0.526  0.59916
## ---
## Signif. codes:  0 '***' 0.001 '**' 0.01 '*' 0.05 '.' 0.1 ' ' 1
##
##
## R-sq.(adj) =  -0.000578
## lmer.REML =  12646  Scale est. = 11.21     n = 2050
```

```
##                                     stdcoef      stdse
## X(Intercept)                    0.000000000 0.00000000
## Xaccumbens_posvsneg_feedback_z -0.006103001 0.02131392
## Xinterview_age                 -0.011313685 0.02152144
```

Male participants

```
##
## Family: gaussian
## Link function: identity
##
## Formula:
## cbcl_scr_syn_internal_r ~ accumbens_posvsneg_feedback_z + interview_age
##
## Parametric coefficients:
##                               Estimate Std. Error t value Pr(>|t|)
## (Intercept)                    3.20768    1.84860   1.735   0.0829 .
## accumbens_posvsneg_feedback_z  0.32112    0.16304   1.970   0.0490 *
## interview_age                  0.01259    0.01539   0.818   0.4137
## ---
## Signif. codes:  0 '***' 0.001 '**' 0.01 '*' 0.05 '.' 0.1 ' ' 1
##
##
## R-sq.(adj) =  0.000399
## lmer.REML =  12603  Scale est. = 18.899    n = 2054
```

```
##                                  stdcoef      stdse
## X(Intercept)                  0.00000000 0.00000000
## Xaccumbens_posvsneg_feedback_z 0.04319714 0.02193190
## Xinterview_age                0.01808956 0.02212641
```

3.5 Model: CBCL internalizing factor ~ Caudate activity (feedback stage)

Female participants

```
##
## Family: gaussian
## Link function: identity
##
## Formula:
## cbcl_scr_syn_internal_r ~ caudate_posvsneg_feedback_z + interview_age
##
## Parametric coefficients:
##                              Estimate Std. Error t value Pr(>|t|)
## (Intercept)                   6.08504    1.86838   3.257  0.00115 **
## caudate_posvsneg_feedback_z -0.18760    0.13267  -1.414  0.15750
## interview_age               -0.01029    0.01562  -0.659  0.51010
## ---
## Signif. codes:  0 '***' 0.001 '**' 0.01 '*' 0.05 '.' 0.1 ' ' 1
##
##
## R-sq.(adj) =  0.000878
## lmer.REML =  12610  Scale est. = 11.326    n = 2042
```

```
##                                 stdcoef      stdse
## X(Intercept)                  0.00000000 0.00000000
## Xcaudate_posvsneg_feedback_z -0.02984372 0.02110522
## Xinterview_age               -0.01421366 0.02157502
```

Male participants

```
##
## Family: gaussian
## Link function: identity
##
## Formula:
## cbcl_scr_syn_internal_r ~ caudate_posvsneg_feedback_z + interview_age
##
## Parametric coefficients:
##                              Estimate Std. Error t value Pr(>|t|)
## (Intercept)                 3.781203   1.865165   2.027   0.0428 *
## caudate_posvsneg_feedback_z 0.141738   0.131057   1.082   0.2796
## interview_age               0.008144   0.015537   0.524   0.6002
## ---
## Signif. codes:  0 '***' 0.001 '**' 0.01 '*' 0.05 '.' 0.1 ' ' 1
##
##
## R-sq.(adj) =  -0.000857
## lmer.REML =  12670  Scale est. = 18.536    n = 2058
```

```
##                                  stdcoef      stdse
## X(Intercept)                  0.00000000 0.00000000
## Xcaudate_posvsneg_feedback_z 0.02360106 0.02182246
## Xinterview_age               0.01159805 0.02212483
```

3.6 Model: CBCL internalizing factor ~ Putamen activity (feedback stage)

Female participants

```
##
## Family: gaussian
## Link function: identity
##
## Formula:
## cbcl_scr_syn_internal_r ~ putamen_posvsneg_feedback_z + interview_age
##
## Parametric coefficients:
##                              Estimate Std. Error t value Pr(>|t|)
## (Intercept)                  5.870302   1.864372   3.149  0.00166 **
## putamen_posvsneg_feedback_z -0.099433   0.139287  -0.714  0.47539
## interview_age               -0.008449   0.015591  -0.542  0.58795
## ---
## Signif. codes:  0 '***' 0.001 '**' 0.01 '*' 0.05 '.' 0.1 ' ' 1
##
##
## R-sq.(adj) =  -0.000157
## lmer.REML =  12610  Scale est. = 11.281    n = 2042
```

```
##                                   stdcoef      stdse
## X(Intercept)                   0.00000000 0.00000000
## Xputamen_posvsneg_feedback_z -0.01507505 0.02111722
## Xinterview_age               -0.01167656 0.02154777
```

Male participants

```
##
## Family: gaussian
## Link function: identity
##
## Formula:
## cbcl_scr_syn_internal_r ~ putamen_posvsneg_feedback_z + interview_age
##
## Parametric coefficients:
##                             Estimate Std. Error t value Pr(>|t|)
## (Intercept)                  3.54548    1.86842   1.898   0.0579 .
## putamen_posvsneg_feedback_z  0.13679    0.13284   1.030   0.3032
## interview_age                0.01013    0.01556   0.651   0.5149
## ---
## Signif. codes:  0 '***' 0.001 '**' 0.01 '*' 0.05 '.' 0.1 ' ' 1
##
##
## R-sq.(adj) =  -0.00108
## lmer.REML =  12701  Scale est. = 18.877    n = 2061
```

```
##                                 stdcoef      stdse
## X(Intercept)                 0.00000000 0.00000000
## Xputamen_posvsneg_feedback_z 0.02257279 0.02192055
## Xinterview_age               0.01439570 0.02210408
```

3.7 Model: CBCL internalizing factor ~ OFC activity (anticipation stage)

Female participants

```
##
## Family: gaussian
## Link function: identity
##
## Formula:
## cbcl_scr_syn_internal_r ~ lOFC_rvsn_ant_z + interview_age
##
## Parametric coefficients:
##                  Estimate Std. Error t value Pr(>|t|)
## (Intercept)      5.886252   1.871858   3.145  0.00169 **
## lOFC_rvsn_ant_z  0.028463   0.202850   0.140  0.88843
## interview_age   -0.008492   0.015651  -0.543  0.58746
## ---
## Signif. codes:  0 '***' 0.001 '**' 0.01 '*' 0.05 '.' 0.1 ' ' 1
##
##
## R-sq.(adj) =  -0.000729
## lmer.REML =  12589  Scale est. = 11.525    n = 2038
```

```
##                       stdcoef      stdse
## X(Intercept)      0.000000000 0.00000000
## XlOFC_rvsn_ant_z  0.003020377 0.02152582
## Xinterview_age   -0.011738081 0.02163280
```

```
##
## Family: gaussian
## Link function: identity
##
## Formula:
## cbcl_scr_syn_internal_r ~ mOFC_rvsn_ant_z + interview_age
##
## Parametric coefficients:
##                  Estimate Std. Error t value Pr(>|t|)
## (Intercept)      5.859094   1.868796   3.135  0.00174 **
## mOFC_rvsn_ant_z  0.158153   0.173166   0.913  0.36119
## interview_age   -0.008187   0.015629  -0.524  0.60045
## ---
## Signif. codes:  0 '***' 0.001 '**' 0.01 '*' 0.05 '.' 0.1 ' ' 1
##
##
## R-sq.(adj) =  -0.000132
## lmer.REML =  12597  Scale est. = 11.352    n = 2039
```

```
##                      stdcoef      stdse
```

```
## X(Intercept)      0.00000000 0.00000000
## XmOFC_rvsn_ant_z  0.01926434 0.02109318
## Xinterview_age   -0.01129104 0.02155432
```

Male participants

```
##
## Family: gaussian
## Link function: identity
##
## Formula:
## cbcl_scr_syn_internal_r ~ lOFC_rvsn_ant_z + interview_age
##
## Parametric coefficients:
##                  Estimate Std. Error t value Pr(>|t|)
## (Intercept)       2.59775    1.84642   1.407    0.160
## lOFC_rvsn_ant_z  0.01023    0.18646   0.055    0.956
## interview_age    0.01775    0.01538   1.155    0.248
##
##
## R-sq.(adj) =  -0.000919
## lmer.REML =  12592  Scale est. = 18.062    n = 2053
```

```
##                      stdcoef      stdse
## X(Intercept)     0.000000000 0.00000000
## XlOFC_rvsn_ant_z 0.001201186 0.02190392
## Xinterview_age   0.025574945 0.02215096
```

```
##
## Family: gaussian
## Link function: identity
##
## Formula:
## cbcl_scr_syn_internal_r ~ mOFC_rvsn_ant_z + interview_age
##
## Parametric coefficients:
##                  Estimate Std. Error t value Pr(>|t|)
## (Intercept)       2.66849    1.85435   1.439    0.150
## mOFC_rvsn_ant_z  0.25414    0.17105   1.486    0.137
## interview_age    0.01724    0.01544   1.116    0.264
##
##
## R-sq.(adj) =  0.000328
## lmer.REML =  12576  Scale est. = 18.194    n = 2048
```

```
##                     stdcoef      stdse
## X(Intercept)     0.00000000 0.00000000
## XmOFC_rvsn_ant_z 0.03252081 0.02188820
## Xinterview_age   0.02474006 0.02216395
```

3.8 Model: CBCL internalizing factor ~ OFC activity (feedback stage)

Female participants

```
##
## Family: gaussian
## Link function: identity
##
## Formula:
## cbcl_scr_syn_internal_r ~ lOFC_posvsneg_feedback_z + interview_age
##
## Parametric coefficients:
##                           Estimate Std. Error t value Pr(>|t|)
## (Intercept)               5.888613   1.861224   3.164  0.00158 **
## lOFC_posvsneg_feedback_z -0.237566   0.228693  -1.039  0.29902
## interview_age            -0.008663   0.015572  -0.556  0.57803
## ---
## Signif. codes:  0 '***' 0.001 '**' 0.01 '*' 0.05 '.' 0.1 ' ' 1
##
##
## R-sq.(adj) =  4.13e-05
## lmer.REML =  12579  Scale est. = 11.213    n = 2039
```

```
##                               stdcoef      stdse
## X(Intercept)                0.00000000 0.00000000
## XlOFC_posvsneg_feedback_z -0.02224531 0.02141444
## Xinterview_age            -0.01200652 0.02158089
```

```
##
## Family: gaussian
## Link function: identity
##
## Formula:
## cbcl_scr_syn_internal_r ~ mOFC_posvsneg_feedback_z + interview_age
##
## Parametric coefficients:
##                           Estimate Std. Error t value Pr(>|t|)
## (Intercept)               5.973940   1.863262   3.206  0.00137 **
## mOFC_posvsneg_feedback_z -0.159334   0.188165  -0.847  0.39722
## interview_age            -0.009333   0.015591  -0.599  0.54951
## ---
## Signif. codes:  0 '***' 0.001 '**' 0.01 '*' 0.05 '.' 0.1 ' ' 1
##
##
## R-sq.(adj) =  0.000255
## lmer.REML =  12595  Scale est. = 11.397    n = 2040
```

```
##                               stdcoef      stdse
## X(Intercept)                0.00000000 0.00000000
## XmOFC_posvsneg_feedback_z -0.01821223 0.02150767
## Xinterview_age            -0.01289717 0.02154552
```

Male participants

```
##
## Family: gaussian
## Link function: identity
##
## Formula:
## cbcl_scr_syn_internal_r ~ lOFC_posvsneg_feedback_z + interview_age
##
## Parametric coefficients:
##                          Estimate Std. Error t value Pr(>|t|)
## (Intercept)               3.13068    1.84350   1.698   0.0896 .
## lOFC_posvsneg_feedback_z  0.06848    0.20435   0.335   0.7376
## interview_age             0.01345    0.01535   0.876   0.3810
## ---
## Signif. codes:  0 '***' 0.001 '**' 0.01 '*' 0.05 '.' 0.1 ' ' 1
##
##
## R-sq.(adj) =  -0.00105
## lmer.REML =  12663  Scale est. = 18.043    n = 2063
```

```
##                               stdcoef      stdse
## X(Intercept)              0.000000000 0.00000000
## XlOFC_posvsneg_feedback_z 0.007299579 0.02178333
## Xinterview_age            0.019366812 0.02210083
```

```
##
## Family: gaussian
## Link function: identity
##
## Formula:
## cbcl_scr_syn_internal_r ~ mOFC_posvsneg_feedback_z + interview_age
##
## Parametric coefficients:
##                          Estimate Std. Error t value Pr(>|t|)
## (Intercept)               3.17570    1.84275   1.723    0.085 .
## mOFC_posvsneg_feedback_z  0.26532    0.17873   1.484    0.138
## interview_age             0.01304    0.01535   0.850    0.396
## ---
## Signif. codes:  0 '***' 0.001 '**' 0.01 '*' 0.05 '.' 0.1 ' ' 1
##
##
## R-sq.(adj) =  -0.000177
## lmer.REML =  12652  Scale est. = 18.142    n = 2061
```

```
##                              stdcoef      stdse
## X(Intercept)              0.00000000 0.00000000
## XmOFC_posvsneg_feedback_z 0.03243102 0.02184718
## Xinterview_age            0.01875492 0.02207703
```

3.9 Model: CBCL internalizing factor ~ BIS-BAS-RR

Female participants

```
##
## Family: gaussian
## Link function: identity
##
## Formula:
## cbcl_scr_syn_internal_r ~ bisbas_ss_basm_rr + interview_age
##
## Parametric coefficients:
##                    Estimate Std. Error t value Pr(>|t|)
## (Intercept)        4.576754   1.717826   2.664  0.00776 **
## bisbas_ss_basm_rr -0.070300   0.044419  -1.583  0.11362
## interview_age      0.008269   0.013933   0.593  0.55293
## ---
## Signif. codes:  0 '***' 0.001 '**' 0.01 '*' 0.05 '.' 0.1 ' ' 1
##
##
## R-sq.(adj) =  -0.000292
## lmer.REML =  16721  Scale est. = 12.884    n = 2690
```

```
##                        stdcoef      stdse
## X(Intercept)        0.00000000 0.00000000
## Xbisbas_ss_basm_rr -0.02987863 0.01887900
## Xinterview_age      0.01127390 0.01899736
```

Male participants

```
##
## Family: gaussian
## Link function: identity
##
## Formula:
## cbcl_scr_syn_internal_r ~ bisbas_ss_basm_rr + interview_age
##
## Parametric coefficients:
##                   Estimate Std. Error t value Pr(>|t|)
## (Intercept)        3.60221    1.69192   2.129   0.0333 *
## bisbas_ss_basm_rr  0.01009    0.04429   0.228   0.8198
## interview_age      0.01112    0.01371   0.811   0.4176
## ---
## Signif. codes:  0 '***' 0.001 '**' 0.01 '*' 0.05 '.' 0.1 ' ' 1
##
##
## R-sq.(adj) =  -0.000755
## lmer.REML =  18144  Scale est. = 16.373    n = 2907
```

```
##                        stdcoef      stdse
## X(Intercept)       0.000000000 0.00000000
## Xbisbas_ss_basm_rr 0.004192092 0.01840355
## Xinterview_age     0.015062217 0.01857752
```

3.10 Model: CBCL internalizing factor ~ MID Reaction Time

Female participants

```
##
## Family: gaussian
## Link function: identity
##
## Formula:
## cbcl_scr_syn_internal_r ~ rt_diff_large_neutral_z + interview_age
##
## Parametric coefficients:
##                         Estimate Std. Error t value Pr(>|t|)
## (Intercept)              6.34845    1.78964   3.547 0.000397 ***
## rt_diff_large_neutral_z  0.13672    0.12031   1.136 0.255921
## interview_age           -0.01246    0.01495  -0.833 0.404751
## ---
## Signif. codes:  0 '***' 0.001 '**' 0.01 '*' 0.05 '.' 0.1 ' ' 1
##
##
## R-sq.(adj) =  -5.14e-06
## lmer.REML =  13581  Scale est. = 11.707    n = 2201
```

```
##                              stdcoef      stdse
## X(Intercept)              0.00000000 0.00000000
## Xrt_diff_large_neutral_z  0.02353331 0.02070898
## Xinterview_age           -0.01731784 0.02078157
```

```
##
## Family: gaussian
## Link function: identity
##
## Formula:
## cbcl_scr_syn_internal_r ~ rt_diff_large_small_z + interview_age
##
## Parametric coefficients:
##                       Estimate Std. Error t value Pr(>|t|)
## (Intercept)            6.25233    1.78792   3.497  0.00048 ***
## rt_diff_large_small_z -0.15739    0.11917  -1.321  0.18675
## interview_age         -0.01158    0.01493  -0.775  0.43813
## ---
## Signif. codes:  0 '***' 0.001 '**' 0.01 '*' 0.05 '.' 0.1 ' ' 1
##
##
## R-sq.(adj) =  -0.000251
## lmer.REML =  13580  Scale est. = 11.639    n = 2201
```

```
##                            stdcoef      stdse
## X(Intercept)            0.00000000 0.00000000
## Xrt_diff_large_small_z -0.02721487 0.02060706
## Xinterview_age         -0.01609858 0.02075907
```

Male participants

```
##
## Family: gaussian
## Link function: identity
##
## Formula:
## cbcl_scr_syn_internal_r ~ rt_diff_large_neutral_z + interview_age
##
## Parametric coefficients:
##                         Estimate Std. Error t value Pr(>|t|)
## (Intercept)              2.95337    1.77854   1.661   0.0969 .
## rt_diff_large_neutral_z  0.04500    0.12536   0.359   0.7197
## interview_age            0.01547    0.01482   1.044   0.2965
## ---
## Signif. codes:  0 '***' 0.001 '**' 0.01 '*' 0.05 '.' 0.1 ' ' 1
##
##
## R-sq.(adj) =  -0.000758
## lmer.REML =  14182  Scale est. = 17.631    n = 2297
```

```
##                             stdcoef      stdse
## X(Intercept)             0.00000000 0.00000000
## Xrt_diff_large_neutral_z 0.00742118 0.02067627
## Xinterview_age           0.02185601 0.02093084
```

```
##
## Family: gaussian
## Link function: identity
##
## Formula:
## cbcl_scr_syn_internal_r ~ rt_diff_large_small_z + interview_age
##
## Parametric coefficients:
##                       Estimate Std. Error t value Pr(>|t|)
## (Intercept)            2.96179    1.77843   1.665    0.096 .
## rt_diff_large_small_z -0.07562    0.12322  -0.614    0.539
## interview_age          0.01540    0.01481   1.040    0.299
## ---
## Signif. codes:  0 '***' 0.001 '**' 0.01 '*' 0.05 '.' 0.1 ' ' 1
##
##
## R-sq.(adj) =  -0.000672
## lmer.REML =  14181  Scale est. = 17.688    n = 2297
```

```
##                            stdcoef      stdse
## X(Intercept)            0.00000000 0.00000000
## Xrt_diff_large_small_z -0.01266907 0.02064481
## Xinterview_age          0.02175852 0.02092903
```

4—Internalizing~Puberty x Reward—

4.1 Model: CBCL internalizing factor ~ PDS x Accumbens activity (anticipation stage)

Female participants

```
##
## Family: gaussian
## Link function: identity
##
## Formula:
## cbcl_scr_syn_internal_r ~ PDS_score * accumbens_rvsn_ant_z +
##     race.ethnicity.5level + demo_race_hispanic + interview_age +
##     bmi + household.income + high.educ
##
## Parametric coefficients:
##                               Estimate Std. Error t value Pr(>|t|)
## (Intercept)                    6.11905    2.40102   2.549  0.01090 *
## PDS_score                      0.56881    0.19031   2.989  0.00284 **
## accumbens_rvsn_ant_z          -0.69127    0.44437  -1.556  0.11997
## race.ethnicity.5levelBlack     0.05661    0.97047   0.058  0.95349
## race.ethnicity.5levelMixed     1.95031    0.93531   2.085  0.03719 *
## race.ethnicity.5levelOther     1.86410    1.05100   1.774  0.07629 .
## race.ethnicity.5levelWhite     1.50236    0.88078   1.706  0.08823 .
## demo_race_hispanic1            0.10997    0.37621   0.292  0.77008
## interview_age                 -0.02039    0.01679  -1.214  0.22484
## bmi                            0.05012    0.03333   1.504  0.13278
## household.income[>=200K]      -2.92893    0.91834  -3.189  0.00145 **
## household.income[100K-200K]   -2.45363    0.86409  -2.840  0.00457 **
## household.income[12K-16K]     -0.22604    1.10943  -0.204  0.83857
## household.income[16K-25K]     -0.48476    0.95778  -0.506  0.61283
## household.income[25K-35K]     -1.66374    0.91189  -1.824  0.06824 .
## household.income[35K-50K]     -1.06164    0.87487  -1.213  0.22510
## household.income[50K-75K]     -1.63166    0.87036  -1.875  0.06099 .
## household.income[5K-12K]      -0.90095    1.01115  -0.891  0.37304
## household.income[75K-100K]    -1.83395    0.87483  -2.096  0.03619 *
## high.educBachelor             -0.31676    0.82479  -0.384  0.70099
## high.educHS Diploma/GED       -1.01228    0.84052  -1.204  0.22861
## high.educPost Graduate Degree -0.12652    0.83344  -0.152  0.87936
## high.educSome College          0.08687    0.77796   0.112  0.91110
## PDS_score:accumbens_rvsn_ant_z  0.42314    0.24827   1.704  0.08848 .
## ---
## Signif. codes:  0 '***' 0.001 '**' 0.01 '*' 0.05 '.' 0.1 ' ' 1
##
##
## R-sq.(adj) =  0.0311
## lmer.REML =  11259  Scale est. = 11.173    n = 1844
```

```
##                                    stdcoef      stdse
## X(Intercept)                   0.000000000 0.00000000
## XPDS_score                     0.076633350 0.02563962
## Xaccumbens_rvsn_ant_z         -0.088329774 0.05678078
```

```
## Xrace.ethnicity.5levelBlack      0.003363031 0.05764810
## Xrace.ethnicity.5levelMixed      0.121484669 0.05826063
## Xrace.ethnicity.5levelOther      0.077152117 0.04349939
## Xrace.ethnicity.5levelWhite      0.130707659 0.07662918
## Xdemo_race_hispanic1             0.008185239 0.02800142
## Xinterview_age                  -0.028641556 0.02358949
## Xbmi                             0.037511702 0.02494275
## Xhousehold.income[>=200K]       -0.186749645 0.05855373
## Xhousehold.income[100K-200K]    -0.214082067 0.07539302
## Xhousehold.income[12K-16K]      -0.006271594 0.03078115
## Xhousehold.income[16K-25K]      -0.018246841 0.03605182
## Xhousehold.income[25K-35K]      -0.073193373 0.04011721
## Xhousehold.income[35K-50K]      -0.056551321 0.04660236
## Xhousehold.income[50K-75K]      -0.103854165 0.05539772
## Xhousehold.income[5K-12K]       -0.029590297 0.03320970
## Xhousehold.income[75K-100K]     -0.124387805 0.05933534
## Xhigh.educBachelor              -0.026898531 0.07003894
## Xhigh.educHS Diploma/GED        -0.047884057 0.03975905
## Xhigh.educPost Graduate Degree  -0.011527175 0.07593636
## Xhigh.educSome College           0.006984024 0.06254190
## XPDS_score:accumbens_rvsn_ant_z  0.096625908 0.05669338
```

Male participants

```
##
## Family: gaussian
## Link function: identity
##
## Formula:
## cbcl_scr_syn_internal_r ~ PDS_score * accumbens_rvsn_ant_z +
##     race.ethnicity.5level + demo_race_hispanic + interview_age +
##     bmi + household.income + high.educ
##
## Parametric coefficients:
##                                 Estimate Std. Error t value Pr(>|t|)
## (Intercept)                     3.117524   2.301765   1.354  0.17577
## PDS_score                       0.559132   0.241527   2.315  0.02072 *
## accumbens_rvsn_ant_z           -0.368486   0.441156  -0.835  0.40367
## race.ethnicity.5levelBlack      0.278699   0.898162   0.310  0.75637
## race.ethnicity.5levelMixed      2.407559   0.866909   2.777  0.00554 **
## race.ethnicity.5levelOther      1.985057   1.017121   1.952  0.05113 .
## race.ethnicity.5levelWhite      1.909716   0.812309   2.351  0.01883 *
## demo_race_hispanic1            -0.500052   0.358845  -1.394  0.16363
## interview_age                  -0.008402   0.015787  -0.532  0.59463
## bmi                             0.062725   0.034694   1.808  0.07078 .
## household.income[>=200K]       -2.103815   0.933603  -2.253  0.02435 *
## household.income[100K-200K]    -1.755222   0.877699  -2.000  0.04567 *
## household.income[12K-16K]      -1.079166   1.128151  -0.957  0.33890
## household.income[16K-25K]       0.336627   0.954785   0.353  0.72445
## household.income[25K-35K]      -0.546154   0.932203  -0.586  0.55803
## household.income[35K-50K]      -0.280224   0.902540  -0.310  0.75623
## household.income[50K-75K]      -1.203698   0.874783  -1.376  0.16899
## household.income[5K-12K]        0.837998   1.026971   0.816  0.41461
```

```
## household.income[75K-100K]     -1.489959   0.889095  -1.676  0.09394 .
## high.educBachelor               0.372567   0.801446   0.465  0.64208
## high.educHS Diploma/GED        -0.811606   0.827024  -0.981  0.32655
## high.educPost Graduate Degree   0.168145   0.812060   0.207  0.83599
## high.educSome College           0.623646   0.766586   0.814  0.41601
## PDS_score:accumbens_rvsn_ant_z  0.206892   0.304367   0.680  0.49675
## ---
## Signif. codes:  0 '***' 0.001 '**' 0.01 '*' 0.05 '.' 0.1 ' ' 1
##
##
## R-sq.(adj) =  0.0246
## lmer.REML =  11300  Scale est. = 16.293    n = 1871
```

```
##                                    stdcoef      stdse
## X(Intercept)                    0.00000000 0.00000000
## XPDS_score                      0.05744847 0.02481589
## Xaccumbens_rvsn_ant_z          -0.05233564 0.06265699
## Xrace.ethnicity.5levelBlack     0.01743433 0.05618542
## Xrace.ethnicity.5levelMixed     0.15630860 0.05628330
## Xrace.ethnicity.5levelOther     0.07733750 0.03962685
## Xrace.ethnicity.5levelWhite     0.17251213 0.07337904
## Xdemo_race_hispanic1           -0.03898454 0.02797589
## Xinterview_age                 -0.01250435 0.02349408
## Xbmi                            0.04446924 0.02459663
## Xhousehold.income[>=200K]      -0.13374283 0.05935060
## Xhousehold.income[100K-200K]   -0.16421092 0.08211370
## Xhousehold.income[12K-16K]     -0.03004272 0.03140641
## Xhousehold.income[16K-25K]      0.01375717 0.03901985
## Xhousehold.income[25K-35K]     -0.02480751 0.04234269
## Xhousehold.income[35K-50K]     -0.01501823 0.04837048
## Xhousehold.income[50K-75K]     -0.08283067 0.06019693
## Xhousehold.income[5K-12K]       0.02743820 0.03362567
## Xhousehold.income[75K-100K]    -0.10412026 0.06213106
## Xhigh.educBachelor              0.03309309 0.07118808
## Xhigh.educHS Diploma/GED       -0.04101662 0.04179583
## Xhigh.educPost Graduate Degree  0.01602987 0.07741659
## Xhigh.educSome College          0.05299792 0.06514501
## XPDS_score:accumbens_rvsn_ant_z  0.04261493 0.06269256
```

4.2 Model: CBCL internalizing factor ~ PDS x Caudate activity (anticipation stage)

Female participants

```
##
## Family: gaussian
## Link function: identity
##
## Formula:
## cbcl_scr_syn_internal_r ~ PDS_score * caudate_rvsn_ant_z + race.ethnicity.5level +
##     demo_race_hispanic + interview_age + bmi + household.income +
##     high.educ
##
```

```
## Parametric coefficients:
##                              Estimate Std. Error t value Pr(>|t|)
## (Intercept)                   6.20089    2.41763   2.565  0.01040 *
## PDS_score                     0.58077    0.19121   3.037  0.00242 **
## caudate_rvsn_ant_z           -0.14010    0.33814  -0.414  0.67868
## race.ethnicity.5levelBlack    0.08535    0.97324   0.088  0.93013
## race.ethnicity.5levelMixed    1.93044    0.93680   2.061  0.03948 *
## race.ethnicity.5levelOther    1.86084    1.05156   1.770  0.07696 .
## race.ethnicity.5levelWhite    1.49756    0.88263   1.697  0.08993 .
## demo_race_hispanic1           0.13130    0.37609   0.349  0.72705
## interview_age                -0.02300    0.01689  -1.362  0.17332
## bmi                           0.05510    0.03340   1.650  0.09914 .
## household.income[>=200K]     -2.85485    0.92181  -3.097  0.00198 **
## household.income[100K-200K]  -2.35873    0.86574  -2.725  0.00650 **
## household.income[12K-16K]    -0.22727    1.10464  -0.206  0.83702
## household.income[16K-25K]    -0.48754    0.95703  -0.509  0.61051
## household.income[25K-35K]    -1.54976    0.91506  -1.694  0.09051 .
## household.income[35K-50K]    -1.02675    0.87749  -1.170  0.24211
## household.income[50K-75K]    -1.51840    0.87269  -1.740  0.08204 .
## household.income[5K-12K]     -0.80578    1.01393  -0.795  0.42689
## household.income[75K-100K]   -1.75774    0.87749  -2.003  0.04531 *
## high.educBachelor            -0.28693    0.82785  -0.347  0.72894
## high.educHS Diploma/GED      -0.97129    0.84324  -1.152  0.24953
## high.educPost Graduate Degree -0.05426    0.83627  -0.065  0.94827
## high.educSome College         0.09622    0.78074   0.123  0.90193
## PDS_score:caudate_rvsn_ant_z  0.11331    0.19071   0.594  0.55249
## ---
## Signif. codes:  0 '***' 0.001 '**' 0.01 '*' 0.05 '.' 0.1 ' ' 1
##
##
## R-sq.(adj) =  0.0284
## lmer.REML =  11269  Scale est. = 11.324    n = 1844
```

```
##                                      stdcoef      stdse
## X(Intercept)                     0.000000000 0.00000000
## XPDS_score                       0.078197995 0.02574559
## Xcaudate_rvsn_ant_z             -0.023593773 0.05694375
## Xrace.ethnicity.5levelBlack      0.005045458 0.05753331
## Xrace.ethnicity.5levelMixed      0.120619213 0.05853407
## Xrace.ethnicity.5levelOther      0.076970947 0.04349633
## Xrace.ethnicity.5levelWhite      0.130211915 0.07674446
## Xdemo_race_hispanic1             0.009756317 0.02794607
## Xinterview_age                  -0.032266145 0.02368790
## Xbmi                             0.041170197 0.02495321
## Xhousehold.income[>=200K]       -0.181282770 0.05853468
## Xhousehold.income[100K-200K]    -0.205872155 0.07556275
## Xhousehold.income[12K-16K]      -0.006376440 0.03099255
## Xhousehold.income[16K-25K]      -0.018453833 0.03622456
## Xhousehold.income[25K-35K]      -0.067838713 0.04005563
## Xhousehold.income[35K-50K]      -0.054812320 0.04684414
## Xhousehold.income[50K-75K]      -0.096586882 0.05551267
## Xhousehold.income[5K-12K]       -0.026448800 0.03328108
## Xhousehold.income[75K-100K]     -0.118801513 0.05930754
## Xhigh.educBachelor              -0.024350438 0.07025642
```

```
## Xhigh.educHS Diploma/GED       -0.045917490 0.03986355
## Xhigh.educPost Graduate Degree -0.004939437 0.07612584
## Xhigh.educSome College          0.007736670 0.06277652
## XPDS_score:caudate_rvsn_ant_z   0.033877097 0.05701789
```

Male participants

```
##
## Family: gaussian
## Link function: identity
##
## Formula:
## cbcl_scr_syn_internal_r ~ PDS_score * caudate_rvsn_ant_z + race.ethnicity.5level +
##     demo_race_hispanic + interview_age + bmi + household.income +
##     high.educ
##
## Parametric coefficients:
##                                Estimate Std. Error t value Pr(>|t|)
## (Intercept)                    3.139328   2.305819   1.361  0.17353
## PDS_score                      0.603064   0.242005   2.492  0.01279 *
## caudate_rvsn_ant_z             0.341612   0.357929   0.954  0.34000
## race.ethnicity.5levelBlack     0.209785   0.918630   0.228  0.81939
## race.ethnicity.5levelMixed     2.335256   0.889971   2.624  0.00876 **
## race.ethnicity.5levelOther     1.974006   1.036313   1.905  0.05696 .
## race.ethnicity.5levelWhite     1.834745   0.836265   2.194  0.02836 *
## demo_race_hispanic1           -0.484359   0.360258  -1.344  0.17896
## interview_age                 -0.009867   0.015830  -0.623  0.53316
## bmi                            0.061409   0.034753   1.767  0.07740 .
## household.income[>=200K]      -1.860244   0.932271  -1.995  0.04615 *
## household.income[100K-200K]   -1.577514   0.875312  -1.802  0.07167 .
## household.income[12K-16K]     -0.970861   1.127323  -0.861  0.38923
## household.income[16K-25K]      0.392206   0.950354   0.413  0.67988
## household.income[25K-35K]     -0.372747   0.929644  -0.401  0.68850
## household.income[35K-50K]     -0.115792   0.900989  -0.129  0.89775
## household.income[50K-75K]     -1.045268   0.871695  -1.199  0.23063
## household.income[5K-12K]       1.070719   1.020260   1.049  0.29411
## household.income[75K-100K]    -1.276219   0.887261  -1.438  0.15050
## high.educBachelor              0.428282   0.798174   0.537  0.59162
## high.educHS Diploma/GED       -0.684071   0.824202  -0.830  0.40666
## high.educPost Graduate Degree  0.161066   0.809270   0.199  0.84226
## high.educSome College          0.630578   0.762282   0.827  0.40822
## PDS_score:caudate_rvsn_ant_z  -0.293166   0.248934  -1.178  0.23907
## ---
## Signif. codes:  0 '***' 0.001 '**' 0.01 '*' 0.05 '.' 0.1 ' ' 1
##
##
## R-sq.(adj) =  0.0244
## lmer.REML =  11297  Scale est. = 16.374    n = 1869
```

```
##                                    stdcoef      stdse
## X(Intercept)                   0.000000000 0.00000000
## XPDS_score                     0.061856638 0.02482255
## Xcaudate_rvsn_ant_z            0.062761641 0.06575938
```

```
## Xrace.ethnicity.5levelBlack     0.013132145 0.05750461
## Xrace.ethnicity.5levelMixed     0.151122760 0.05759319
## Xrace.ethnicity.5levelOther     0.077286727 0.04057395
## Xrace.ethnicity.5levelWhite     0.165400296 0.07538834
## Xdemo_race_hispanic1           -0.037587663 0.02795703
## Xinterview_age                 -0.014658582 0.02351762
## Xbmi                            0.043531682 0.02463611
## Xhousehold.income[>=200K]      -0.117394235 0.05883272
## Xhousehold.income[100K-200K]   -0.147239796 0.08169866
## Xhousehold.income[12K-16K]     -0.026991997 0.03134198
## Xhousehold.income[16K-25K]      0.016097662 0.03900626
## Xhousehold.income[25K-35K]     -0.016983796 0.04235825
## Xhousehold.income[35K-50K]     -0.006178408 0.04807497
## Xhousehold.income[50K-75K]     -0.071828156 0.05990066
## Xhousehold.income[5K-12K]       0.035330753 0.03366574
## Xhousehold.income[75K-100K]    -0.089058992 0.06191618
## Xhigh.educBachelor              0.037962378 0.07074913
## Xhigh.educHS Diploma/GED       -0.034403577 0.04145110
## Xhigh.educPost Graduate Degree  0.015307235 0.07691061
## Xhigh.educSome College          0.053663376 0.06487168
## XPDS_score:caudate_rvsn_ant_z  -0.077563289 0.06586088
```

4.3 Model: CBCL internalizing factor ~ PDS x Putamen activity (anticipation stage)

Female participants

```
##
## Family: gaussian
## Link function: identity
##
## Formula:
## cbcl_scr_syn_internal_r ~ PDS_score * putamen_rvsn_ant_z + race.ethnicity.5level +
##     demo_race_hispanic + interview_age + bmi + household.income +
##     high.educ
##
## Parametric coefficients:
##                              Estimate Std. Error t value Pr(>|t|)
## (Intercept)                   5.99889    2.39100   2.509  0.01220 *
## PDS_score                     0.59914    0.19096   3.138  0.00173 **
## putamen_rvsn_ant_z           -0.45726    0.33996  -1.345  0.17878
## race.ethnicity.5levelBlack    0.08926    0.96739   0.092  0.92650
## race.ethnicity.5levelMixed    1.97029    0.93130   2.116  0.03451 *
## race.ethnicity.5levelOther    1.84499    1.04737   1.762  0.07832 .
## race.ethnicity.5levelWhite    1.47228    0.87724   1.678  0.09346 .
## demo_race_hispanic1           0.16397    0.37473   0.438  0.66175
## interview_age                -0.02129    0.01676  -1.270  0.20423
## bmi                           0.05062    0.03323   1.523  0.12788
## household.income[>=200K]     -2.75584    0.91307  -3.018  0.00258 **
## household.income[100K-200K]  -2.31366    0.85687  -2.700  0.00700 **
## household.income[12K-16K]    -0.14369    1.10118  -0.130  0.89619
## household.income[16K-25K]    -0.54056    0.94947  -0.569  0.56921
## household.income[25K-35K]    -1.52466    0.90500  -1.685  0.09222 .
```

```
## household.income[35K-50K]     -0.98046    0.86790  -1.130  0.25875
## household.income[50K-75K]     -1.44783    0.86458  -1.675  0.09418 .
## household.income[5K-12K]      -0.77276    1.00435  -0.769  0.44175
## household.income[75K-100K]    -1.80298    0.86926  -2.074  0.03820 *
## high.educBachelor             -0.24602    0.81815  -0.301  0.76368
## high.educHS Diploma/GED       -0.94372    0.83287  -1.133  0.25732
## high.educPost Graduate Degree -0.08116    0.82642  -0.098  0.92178
## high.educSome College          0.12851    0.77104   0.167  0.86765
## PDS_score:putamen_rvsn_ant_z   0.31990    0.19028   1.681  0.09289 .
## ---
## Signif. codes:  0 '***' 0.001 '**' 0.01 '*' 0.05 '.' 0.1 ' ' 1
##
##
## R-sq.(adj) =  0.0298
## lmer.REML =  11225  Scale est. = 11.253    n = 1840
```

```
##                                    stdcoef      stdse
## X(Intercept)                    0.000000000 0.00000000
## XPDS_score                      0.081027053 0.02582528
## Xputamen_rvsn_ant_z            -0.074568247 0.05543988
## Xrace.ethnicity.5levelBlack     0.005306278 0.05751155
## Xrace.ethnicity.5levelMixed     0.123575770 0.05841079
## Xrace.ethnicity.5levelOther     0.076753205 0.04357167
## Xrace.ethnicity.5levelWhite     0.128630986 0.07664328
## Xdemo_race_hispanic1            0.012264432 0.02802887
## Xinterview_age                 -0.030029891 0.02364458
## Xbmi                            0.038068503 0.02499222
## Xhousehold.income[>=200K]      -0.176289523 0.05840853
## Xhousehold.income[100K-200K]   -0.202912742 0.07514898
## Xhousehold.income[12K-16K]     -0.004007414 0.03071012
## Xhousehold.income[16K-25K]     -0.020451777 0.03592279
## Xhousehold.income[25K-35K]     -0.067122206 0.03984235
## Xhousehold.income[35K-50K]     -0.052492899 0.04646650
## Xhousehold.income[50K-75K]     -0.092454981 0.05520982
## Xhousehold.income[5K-12K]      -0.025510986 0.03315642
## Xhousehold.income[75K-100K]    -0.122362756 0.05899371
## Xhigh.educBachelor             -0.020990584 0.06980608
## Xhigh.educHS Diploma/GED       -0.044869220 0.03959871
## Xhigh.educPost Graduate Degree -0.007425850 0.07561654
## Xhigh.educSome College          0.010349129 0.06209295
## XPDS_score:putamen_rvsn_ant_z   0.093124202 0.05539024
```

Male participants

```
##
## Family: gaussian
## Link function: identity
##
## Formula:
## cbcl_scr_syn_internal_r ~ PDS_score * putamen_rvsn_ant_z + race.ethnicity.5level +
##     demo_race_hispanic + interview_age + bmi + household.income +
##     high.educ
##
```

```
## Parametric coefficients:
##                               Estimate Std. Error t value Pr(>|t|)
## (Intercept)                   3.035922   2.306610   1.316  0.18828
## PDS_score                     0.643633   0.242613   2.653  0.00805 **
## putamen_rvsn_ant_z            0.659483   0.354021   1.863  0.06264 .
## race.ethnicity.5levelBlack    0.276774   0.916992   0.302  0.76282
## race.ethnicity.5levelMixed    2.383532   0.885719   2.691  0.00719 **
## race.ethnicity.5levelOther    2.025999   1.033877   1.960  0.05019 .
## race.ethnicity.5levelWhite    1.874622   0.834177   2.247  0.02474 *
## demo_race_hispanic1          -0.533612   0.359523  -1.484  0.13792
## interview_age                -0.009086   0.015814  -0.575  0.56565
## bmi                           0.063665   0.034884   1.825  0.06815 .
## household.income[>=200K]     -1.842993   0.934413  -1.972  0.04872 *
## household.income[100K-200K]  -1.604230   0.878787  -1.826  0.06809 .
## household.income[12K-16K]    -0.974947   1.128345  -0.864  0.38767
## household.income[16K-25K]     0.360639   0.953262   0.378  0.70524
## household.income[25K-35K]    -0.376623   0.931584  -0.404  0.68605
## household.income[35K-50K]    -0.105639   0.903977  -0.117  0.90698
## household.income[50K-75K]    -1.030987   0.875341  -1.178  0.23902
## household.income[5K-12K]      0.998059   1.022273   0.976  0.32904
## household.income[75K-100K]   -1.310451   0.889733  -1.473  0.14096
## high.educBachelor             0.289132   0.800577   0.361  0.71803
## high.educHS Diploma/GED      -0.781679   0.827308  -0.945  0.34486
## high.educPost Graduate Degree  0.090030   0.811549   0.111  0.91168
## high.educSome College         0.524744   0.765534   0.685  0.49314
## PDS_score:putamen_rvsn_ant_z  -0.562761   0.249537  -2.255  0.02424 *
## ---
## Signif. codes:  0 '***' 0.001 '**' 0.01 '*' 0.05 '.' 0.1 ' ' 1
##
##
## R-sq.(adj) =  0.0263
## lmer.REML =  11304  Scale est. = 15.673    n = 1872
```

```
##                                     stdcoef      stdse
## X(Intercept)                    0.000000000 0.00000000
## XPDS_score                      0.065611959 0.02473194
## Xputamen_rvsn_ant_z             0.121293724 0.06511234
## Xrace.ethnicity.5levelBlack     0.017257573 0.05717683
## Xrace.ethnicity.5levelMixed     0.155706937 0.05786059
## Xrace.ethnicity.5levelOther     0.079331479 0.04048324
## Xrace.ethnicity.5levelWhite     0.169326639 0.07534768
## Xdemo_race_hispanic1           -0.041421038 0.02790760
## Xinterview_age                 -0.013502900 0.02350096
## Xbmi                            0.044870562 0.02458584
## Xhousehold.income[>=200K]      -0.117252490 0.05944800
## Xhousehold.income[100K-200K]   -0.149864391 0.08209474
## Xhousehold.income[12K-16K]     -0.027108232 0.03137344
## Xhousehold.income[16K-25K]      0.014803794 0.03913024
## Xhousehold.income[25K-35K]     -0.017162641 0.04245210
## Xhousehold.income[35K-50K]     -0.005620217 0.04809340
## Xhousehold.income[50K-75K]     -0.070861871 0.06016401
## Xhousehold.income[5K-12K]       0.032936610 0.03373568
## Xhousehold.income[75K-100K]    -0.091467829 0.06210223
## Xhigh.educBachelor              0.025667919 0.07107187
```

```
## Xhigh.educHS Diploma/GED       -0.039318087 0.04161320
## Xhigh.educPost Graduate Degree  0.008573717 0.07728510
## Xhigh.educSome College          0.044574872 0.06502902
## XPDS_score:putamen_rvsn_ant_z  -0.146929637 0.06515075
```

4.4 Model: CBCL internalizing factor ~ PDS x Lateral OFC activity (anticipation stage)

Female participants

```
##
## Family: gaussian
## Link function: identity
##
## Formula:
## cbcl_scr_syn_internal_r ~ PDS_score * lOFC_rvsn_ant_z + race.ethnicity.5level +
##     demo_race_hispanic + interview_age + bmi + household.income +
##     high.educ
##
## Parametric coefficients:
##                               Estimate Std. Error t value Pr(>|t|)
## (Intercept)                    5.90537    2.41442   2.446  0.01454 *
## PDS_score                      0.56567    0.19115   2.959  0.00312 **
## lOFC_rvsn_ant_z                0.41960    0.53771   0.780  0.43529
## race.ethnicity.5levelBlack    -0.03386    0.98288  -0.034  0.97252
## race.ethnicity.5levelMixed     1.81158    0.94787   1.911  0.05613 .
## race.ethnicity.5levelOther     1.74425    1.06299   1.641  0.10099
## race.ethnicity.5levelWhite     1.38472    0.89322   1.550  0.12126
## demo_race_hispanic1            0.13510    0.37773   0.358  0.72065
## interview_age                 -0.02166    0.01693  -1.280  0.20085
## bmi                            0.05782    0.03355   1.723  0.08499 .
## household.income[>=200K]      -2.80214    0.91641  -3.058  0.00226 **
## household.income[100K-200K]   -2.29921    0.86017  -2.673  0.00759 **
## household.income[12K-16K]     -0.16492    1.09748  -0.150  0.88057
## household.income[16K-25K]     -0.38118    0.95058  -0.401  0.68847
## household.income[25K-35K]     -1.46350    0.90826  -1.611  0.10728
## household.income[35K-50K]     -0.86589    0.87326  -0.992  0.32154
## household.income[50K-75K]     -1.44208    0.86486  -1.667  0.09560 .
## household.income[5K-12K]      -0.68903    1.00797  -0.684  0.49433
## household.income[75K-100K]    -1.72254    0.87173  -1.976  0.04831 *
## high.educBachelor             -0.13652    0.82036  -0.166  0.86785
## high.educHS Diploma/GED       -0.84369    0.83387  -1.012  0.31178
## high.educPost Graduate Degree  0.07235    0.82870   0.087  0.93044
## high.educSome College          0.27419    0.77324   0.355  0.72293
## PDS_score:lOFC_rvsn_ant_z     -0.12883    0.29419  -0.438  0.66149
## ---
## Signif. codes:  0 '***' 0.001 '**' 0.01 '*' 0.05 '.' 0.1 ' ' 1
##
##
## R-sq.(adj) =  0.0293
## lmer.REML =  11242  Scale est. = 11.723    n = 1840
```

```
##                                         stdcoef      stdse
```

```
## X(Intercept)                      0.000000000 0.00000000
## XPDS_score                        0.076219676 0.02575657
## XlOFC_rvsn_ant_z                  0.045210225 0.05793605
## Xrace.ethnicity.5levelBlack      -0.002007279 0.05826730
## Xrace.ethnicity.5levelMixed       0.112881108 0.05906267
## Xrace.ethnicity.5levelOther       0.072720310 0.04431756
## Xrace.ethnicity.5levelWhite       0.120521981 0.07774355
## Xdemo_race_hispanic1              0.010076190 0.02817317
## Xinterview_age                   -0.030414646 0.02376908
## Xbmi                              0.043205914 0.02507069
## Xhousehold.income[>=200K]        -0.178744348 0.05845621
## Xhousehold.income[100K-200K]     -0.200980761 0.07519038
## Xhousehold.income[12K-16K]       -0.004640662 0.03088197
## Xhousehold.income[16K-25K]       -0.014470155 0.03608502
## Xhousehold.income[25K-35K]       -0.063962474 0.03969535
## Xhousehold.income[35K-50K]       -0.045969076 0.04636016
## Xhousehold.income[50K-75K]       -0.092148256 0.05526420
## Xhousehold.income[5K-12K]        -0.022492547 0.03290406
## Xhousehold.income[75K-100K]      -0.116572967 0.05899456
## Xhigh.educBachelor               -0.011595008 0.06967600
## Xhigh.educHS Diploma/GED         -0.039999780 0.03953408
## Xhigh.educPost Graduate Degree    0.006606509 0.07567538
## Xhigh.educSome College            0.022001061 0.06204575
## XPDS_score:lOFC_rvsn_ant_z       -0.025440754 0.05809366
```

Male participants

```
##
## Family: gaussian
## Link function: identity
##
## Formula:
## cbcl_scr_syn_internal_r ~ PDS_score * lOFC_rvsn_ant_z + race.ethnicity.5level +
##     demo_race_hispanic + interview_age + bmi + household.income +
##     high.educ
##
## Parametric coefficients:
##                               Estimate Std. Error t value Pr(>|t|)
## (Intercept)                  1.9531886  2.2909476   0.853  0.39401
## PDS_score                    0.4615541  0.2432095   1.898  0.05788 .
## lOFC_rvsn_ant_z             -0.5127591  0.5118239  -1.002  0.31656
## race.ethnicity.5levelBlack   0.3923116  0.8900997   0.441  0.65945
## race.ethnicity.5levelMixed   2.3845865  0.8584509   2.778  0.00553 **
## race.ethnicity.5levelOther   2.0889730  1.0058117   2.077  0.03795 *
## race.ethnicity.5levelWhite   1.8487644  0.8034487   2.301  0.02150 *
## demo_race_hispanic1         -0.5274017  0.3554138  -1.484  0.13800
## interview_age               -0.0002299  0.0156916  -0.015  0.98831
## bmi                          0.0590930  0.0346437   1.706  0.08823 .
## household.income[>=200K]    -2.2516095  0.9250924  -2.434  0.01503 *
## household.income[100K-200K] -1.9588985  0.8709089  -2.249  0.02461 *
## household.income[12K-16K]   -1.2476711  1.1174201  -1.117  0.26433
## household.income[16K-25K]    0.0505765  0.9488676   0.053  0.95750
## household.income[25K-35K]   -0.7652259  0.9251623  -0.827  0.40827
```

```
## household.income[35K-50K]     -0.5771132  0.8969768  -0.643  0.52005
## household.income[50K-75K]     -1.4121349  0.8673476  -1.628  0.10367
## household.income[5K-12K]       0.2643247  1.0229727   0.258  0.79614
## household.income[75K-100K]    -1.6594491  0.8822203  -1.881  0.06013 .
## high.educBachelor              1.0391944  0.8017404   1.296  0.19508
## high.educHS Diploma/GED       -0.1672633  0.8274419  -0.202  0.83983
## high.educPost Graduate Degree  0.7618793  0.8123774   0.938  0.34845
## high.educSome College          1.1981755  0.7673324   1.561  0.11858
## PDS_score:lOFC_rvsn_ant_z      0.3372186  0.3476121   0.970  0.33212
## ---
## Signif. codes:  0 '***' 0.001 '**' 0.01 '*' 0.05 '.' 0.1 ' ' 1
##
##
## R-sq.(adj) =  0.0212
## lmer.REML =  11243  Scale est. = 15.823    n = 1868
```

```
##                                     stdcoef      stdse
## X(Intercept)                   0.0000000000 0.00000000
## XPDS_score                     0.0475970227 0.02508058
## XlOFC_rvsn_ant_z              -0.0627634509 0.06264898
## Xrace.ethnicity.5levelBlack    0.0246378418 0.05589979
## Xrace.ethnicity.5levelMixed    0.1561370901 0.05620934
## Xrace.ethnicity.5levelOther    0.0829109252 0.03992047
## Xrace.ethnicity.5levelWhite    0.1685853877 0.07326499
## Xdemo_race_hispanic1          -0.0415316516 0.02798801
## Xinterview_age                -0.0003457246 0.02359671
## Xbmi                           0.0421220580 0.02469436
## Xhousehold.income[>=200K]     -0.1454689390 0.05976712
## Xhousehold.income[100K-200K]  -0.1854644201 0.08245584
## Xhousehold.income[12K-16K]    -0.0351644014 0.03149340
## Xhousehold.income[16K-25K]     0.0020686251 0.03880959
## Xhousehold.income[25K-35K]    -0.0351881369 0.04254265
## Xhousehold.income[35K-50K]    -0.0313115105 0.04866584
## Xhousehold.income[50K-75K]    -0.0980567234 0.06022743
## Xhousehold.income[5K-12K]      0.0086812666 0.03359769
## Xhousehold.income[75K-100K]   -0.1173880701 0.06240754
## Xhigh.educBachelor             0.0933689078 0.07203429
## Xhigh.educHS Diploma/GED      -0.0085575282 0.04233359
## Xhigh.educPost Graduate Degree  0.0734780283 0.07834823
## Xhigh.educSome College         0.1031344441 0.06604909
## XPDS_score:lOFC_rvsn_ant_z     0.0610047613 0.06288501
```

4.5 Model: CBCL internalizing factor ~ PDS x Medial OFC activity (anticipation stage)

Female participants

```
##
## Family: gaussian
## Link function: identity
##
## Formula:
## cbcl_scr_syn_internal_r ~ PDS_score * mOFC_rvsn_ant_z + race.ethnicity.5level +
```

```
##     demo_race_hispanic + interview_age + bmi + household.income +
##     high.educ
##
## Parametric coefficients:
##                              Estimate Std. Error t value Pr(>|t|)
## (Intercept)                   5.95284    2.41650   2.463  0.01385 *
## PDS_score                     0.57766    0.19133   3.019  0.00257 **
## mOFC_rvsn_ant_z               0.17795    0.44916   0.396  0.69201
## race.ethnicity.5levelBlack   -0.01316    0.98477  -0.013  0.98934
## race.ethnicity.5levelMixed    1.86190    0.94960   1.961  0.05006 .
## race.ethnicity.5levelOther    1.82582    1.06741   1.711  0.08734 .
## race.ethnicity.5levelWhite    1.44159    0.89498   1.611  0.10741
## demo_race_hispanic1           0.12548    0.37808   0.332  0.74001
## interview_age                -0.02118    0.01691  -1.252  0.21061
## bmi                           0.05110    0.03348   1.526  0.12711
## household.income[>=200K]     -2.79648    0.91690  -3.050  0.00232 **
## household.income[100K-200K]  -2.28025    0.86112  -2.648  0.00817 **
## household.income[12K-16K]    -0.14605    1.09907  -0.133  0.89430
## household.income[16K-25K]    -0.36027    0.95274  -0.378  0.70537
## household.income[25K-35K]    -1.39505    0.91116  -1.531  0.12592
## household.income[35K-50K]    -0.85522    0.87352  -0.979  0.32768
## household.income[50K-75K]    -1.47709    0.86620  -1.705  0.08832 .
## household.income[5K-12K]     -0.66847    1.01104  -0.661  0.50859
## household.income[75K-100K]   -1.70540    0.87233  -1.955  0.05074 .
## high.educBachelor            -0.18001    0.82748  -0.218  0.82781
## high.educHS Diploma/GED      -0.87661    0.83934  -1.044  0.29644
## high.educPost Graduate Degree  0.04142    0.83574   0.050  0.96048
## high.educSome College         0.19461    0.78039   0.249  0.80310
## PDS_score:mOFC_rvsn_ant_z    -0.01033    0.25076  -0.041  0.96714
## ---
## Signif. codes:  0 '***' 0.001 '**' 0.01 '*' 0.05 '.' 0.1 ' ' 1
##
##
## R-sq.(adj) =  0.0286
## lmer.REML =  11248  Scale est. = 11.415    n = 1840
```

```
##                                       stdcoef      stdse
## X(Intercept)                    0.0000000000 0.00000000
## XPDS_score                      0.0775121161 0.02567335
## XmOFC_rvsn_ant_z                0.0221964492 0.05602456
## Xrace.ethnicity.5levelBlack    -0.0007778966 0.05820090
## Xrace.ethnicity.5levelMixed     0.1156632109 0.05899016
## Xrace.ethnicity.5levelOther     0.0755102094 0.04414454
## Xrace.ethnicity.5levelWhite     0.1250281744 0.07762097
## Xdemo_race_hispanic1            0.0093206464 0.02808321
## Xinterview_age                 -0.0296556820 0.02368019
## Xbmi                            0.0381744729 0.02501090
## Xhousehold.income[>=200K]      -0.1775288583 0.05820750
## Xhousehold.income[100K-200K]   -0.1987160137 0.07504412
## Xhousehold.income[12K-16K]     -0.0040971350 0.03083229
## Xhousehold.income[16K-25K]     -0.0136346207 0.03605663
## Xhousehold.income[25K-35K]     -0.0605123063 0.03952263
## Xhousehold.income[35K-50K]     -0.0452641132 0.04623247
## Xhousehold.income[50K-75K]     -0.0942605751 0.05527686
```

```
## Xhousehold.income[5K-12K]      -0.0217548613 0.03290341
## Xhousehold.income[75K-100K]    -0.1152294175 0.05894128
## Xhigh.educBachelor             -0.0152422104 0.07006681
## Xhigh.educHS Diploma/GED       -0.0414338773 0.03967239
## Xhigh.educPost Graduate Degree  0.0037698450 0.07606431
## Xhigh.educSome College          0.0155922925 0.06252656
## XPDS_score:mOFC_rvsn_ant_z     -0.0023058252 0.05596138
```

Male participants

```
##
## Family: gaussian
## Link function: identity
##
## Formula:
## cbcl_scr_syn_internal_r ~ PDS_score * mOFC_rvsn_ant_z + race.ethnicity.5level +
##     demo_race_hispanic + interview_age + bmi + household.income +
##     high.educ
##
## Parametric coefficients:
##                               Estimate Std. Error t value Pr(>|t|)
## (Intercept)                   1.541980   2.294061   0.672  0.50157
## PDS_score                     0.481572   0.244298   1.971  0.04885 *
## mOFC_rvsn_ant_z              -0.069075   0.473349  -0.146  0.88399
## race.ethnicity.5levelBlack     0.364765   0.894574   0.408  0.68350
## race.ethnicity.5levelMixed     2.353313   0.862805   2.728  0.00644 **
## race.ethnicity.5levelOther     2.073530   1.010180   2.053  0.04025 *
## race.ethnicity.5levelWhite     1.868744   0.807566   2.314  0.02078 *
## demo_race_hispanic1          -0.483550   0.356420  -1.357  0.17505
## interview_age                -0.001482   0.015759  -0.094  0.92510
## bmi                           0.068437   0.034684   1.973  0.04862 *
## household.income[>=200K]     -2.059643   0.932007  -2.210  0.02724 *
## household.income[100K-200K]  -1.788792   0.877505  -2.038  0.04164 *
## household.income[12K-16K]    -1.018802   1.124441  -0.906  0.36503
## household.income[16K-25K]     0.247535   0.954102   0.259  0.79532
## household.income[25K-35K]    -0.505567   0.932893  -0.542  0.58793
## household.income[35K-50K]    -0.364108   0.901441  -0.404  0.68632
## household.income[50K-75K]    -1.155019   0.874845  -1.320  0.18691
## household.income[5K-12K]      0.627748   1.034822   0.607  0.54418
## household.income[75K-100K]   -1.490365   0.889194  -1.676  0.09389 .
## high.educBachelor             1.197439   0.801829   1.493  0.13551
## high.educHS Diploma/GED      -0.057118   0.827706  -0.069  0.94499
## high.educPost Graduate Degree  0.952177   0.812727   1.172  0.24152
## high.educSome College         1.305653   0.767917   1.700  0.08925 .
## PDS_score:mOFC_rvsn_ant_z      0.158711   0.312298   0.508  0.61137
## ---
## Signif. codes:  0 '***' 0.001 '**' 0.01 '*' 0.05 '.' 0.1 ' ' 1
##
##
## R-sq.(adj) =  0.0225
## lmer.REML =  11202  Scale est. = 15.934    n = 1859
```

```
##                                      stdcoef      stdse
```

```
## X(Intercept)                      0.000000000 0.00000000
## XPDS_score                        0.049698186 0.02521158
## XmOFC_rvsn_ant_z                 -0.009157360 0.06275262
## Xrace.ethnicity.5levelBlack       0.022712626 0.05570196
## Xrace.ethnicity.5levelMixed       0.153455935 0.05626218
## Xrace.ethnicity.5levelOther       0.082133256 0.04001361
## Xrace.ethnicity.5levelWhite       0.169554947 0.07327212
## Xdemo_race_hispanic1             -0.038024238 0.02802732
## Xinterview_age                   -0.002219935 0.02361036
## Xbmi                              0.048808290 0.02473576
## Xhousehold.income[>=200K]        -0.132511826 0.05996277
## Xhousehold.income[100K-200K]     -0.168570734 0.08269358
## Xhousehold.income[12K-16K]       -0.028658008 0.03162954
## Xhousehold.income[16K-25K]        0.010104093 0.03894528
## Xhousehold.income[25K-35K]       -0.023096359 0.04261835
## Xhousehold.income[35K-50K]       -0.019713169 0.04880490
## Xhousehold.income[50K-75K]       -0.080018972 0.06060869
## Xhousehold.income[5K-12K]         0.020188285 0.03327970
## Xhousehold.income[75K-100K]      -0.105024832 0.06266081
## Xhigh.educBachelor                0.107218821 0.07179583
## Xhigh.educHS Diploma/GED         -0.002895674 0.04196186
## Xhigh.educPost Graduate Degree    0.091498829 0.07809847
## Xhigh.educSome College            0.111590333 0.06563163
## XPDS_score:mOFC_rvsn_ant_z        0.032068853 0.06310229
```

4.6 Model: CBCL internalizing factor ~ PDS x Accumbens activity (feedback)

Female participants

```
##
## Family: gaussian
## Link function: identity
##
## Formula:
## cbcl_scr_syn_internal_r ~ PDS_score * accumbens_posvsneg_feedback_z +
##     race.ethnicity.5level + demo_race_hispanic + interview_age +
##     bmi + household.income + high.educ
##
## Parametric coefficients:
##                                      Estimate Std. Error t value Pr(>|t|)
## (Intercept)                           5.93116    2.39835   2.473  0.01349 *
## PDS_score                             0.57335    0.19027   3.013  0.00262 **
## accumbens_posvsneg_feedback_z        -0.27442    0.45265  -0.606  0.54443
## race.ethnicity.5levelBlack            0.03889    0.97222   0.040  0.96809
## race.ethnicity.5levelMixed            1.90379    0.93599   2.034  0.04210 *
## race.ethnicity.5levelOther            1.86117    1.04954   1.773  0.07634 .
## race.ethnicity.5levelWhite            1.50104    0.88205   1.702  0.08897 .
## demo_race_hispanic1                   0.11404    0.37699   0.302  0.76231
## interview_age                        -0.02168    0.01682  -1.289  0.19765
## bmi                                   0.05421    0.03328   1.629  0.10343
## household.income[>=200K]             -2.87558    0.91131  -3.155  0.00163 **
## household.income[100K-200K]          -2.31057    0.85440  -2.704  0.00691 **
## household.income[12K-16K]            -0.15670    1.09524  -0.143  0.88624
```

```
## household.income[16K-25K]              -0.41900    0.94660  -0.443  0.65808
## household.income[25K-35K]              -1.51328    0.90236  -1.677  0.09371 .
## household.income[35K-50K]              -0.94820    0.86520  -1.096  0.27325
## household.income[50K-75K]              -1.53267    0.86199  -1.778  0.07556 .
## household.income[5K-12K]               -0.72344    1.00300  -0.721  0.47083
## household.income[75K-100K]             -1.74104    0.86572  -2.011  0.04446 *
## high.educBachelor                      -0.17423    0.81983  -0.213  0.83172
## high.educHS Diploma/GED                -0.82917    0.83226  -0.996  0.31925
## high.educPost Graduate Degree           0.04705    0.82739   0.057  0.95466
## high.educSome College                   0.18218    0.77180   0.236  0.81342
## PDS_score:accumbens_posvsneg_feedback_z  0.16390    0.24965   0.657  0.51157
## ---
## Signif. codes:  0 '***' 0.001 '**' 0.01 '*' 0.05 '.' 0.1 ' ' 1
##
##
## R-sq.(adj) =  0.028
## lmer.REML =  11304  Scale est. = 11.196    n = 1851
```

```
##                                              stdcoef      stdse
## X(Intercept)                             0.000000000 0.00000000
## XPDS_score                               0.077286345 0.02564804
## Xaccumbens_posvsneg_feedback_z          -0.033587978 0.05540398
## Xrace.ethnicity.5levelBlack              0.002313521 0.05783117
## Xrace.ethnicity.5levelMixed              0.119159839 0.05858414
## Xrace.ethnicity.5levelOther              0.077733414 0.04383492
## Xrace.ethnicity.5levelWhite              0.130952319 0.07695112
## Xdemo_race_hispanic1                     0.008483225 0.02804471
## Xinterview_age                          -0.030451045 0.02362857
## Xbmi                                     0.040599084 0.02491870
## Xhousehold.income[>=200K]               -0.182582025 0.05786248
## Xhousehold.income[100K-200K]            -0.201766890 0.07460877
## Xhousehold.income[12K-16K]              -0.004395128 0.03071847
## Xhousehold.income[16K-25K]              -0.015854969 0.03581931
## Xhousehold.income[25K-35K]              -0.066224105 0.03948894
## Xhousehold.income[35K-50K]              -0.050609182 0.04617891
## Xhousehold.income[50K-75K]              -0.097825270 0.05501774
## Xhousehold.income[5K-12K]               -0.023738519 0.03291187
## Xhousehold.income[75K-100K]             -0.118181482 0.05876509
## Xhigh.educBachelor                      -0.014792084 0.06960189
## Xhigh.educHS Diploma/GED                -0.039189109 0.03933538
## Xhigh.educPost Graduate Degree           0.004290281 0.07544079
## Xhigh.educSome College                   0.014674236 0.06216643
## XPDS_score:accumbens_posvsneg_feedback_z  0.036169225 0.05509221
```

Male participants

```
##
## Family: gaussian
## Link function: identity
##
## Formula:
## cbcl_scr_syn_internal_r ~ PDS_score * accumbens_posvsneg_feedback_z +
##     race.ethnicity.5level + demo_race_hispanic + interview_age +
```

```
##     bmi + household.income + high.educ
##
## Parametric coefficients:
##                                         Estimate Std. Error t value Pr(>|t|)
## (Intercept)                             2.321104   2.284101   1.016  0.30967
## PDS_score                               0.541983   0.239283   2.265  0.02363
## accumbens_posvsneg_feedback_z           0.053979   0.458396   0.118  0.90627
## race.ethnicity.5levelBlack              0.477537   0.891891   0.535  0.59242
## race.ethnicity.5levelMixed              2.495980   0.859332   2.905  0.00372
## race.ethnicity.5levelOther              2.140700   1.010371   2.119  0.03425
## race.ethnicity.5levelWhite              1.926968   0.805110   2.393  0.01679
## demo_race_hispanic1                    -0.477850   0.356571  -1.340  0.18037
## interview_age                          -0.005793   0.015702  -0.369  0.71223
## bmi                                     0.062056   0.034427   1.803  0.07163
## household.income[>=200K]               -2.034562   0.922821  -2.205  0.02760
## household.income[100K-200K]            -1.761144   0.866159  -2.033  0.04217
## household.income[12K-16K]              -1.035130   1.116546  -0.927  0.35401
## household.income[16K-25K]               0.348823   0.942349   0.370  0.71130
## household.income[25K-35K]              -0.497740   0.919671  -0.541  0.58842
## household.income[35K-50K]              -0.355604   0.891213  -0.399  0.68993
## household.income[50K-75K]              -1.172384   0.863973  -1.357  0.17496
## household.income[5K-12K]                0.253480   1.013013   0.250  0.80244
## household.income[75K-100K]             -1.458671   0.877203  -1.663  0.09651
## high.educBachelor                       0.855083   0.798491   1.071  0.28437
## high.educHS Diploma/GED                -0.308663   0.823024  -0.375  0.70768
## high.educPost Graduate Degree           0.626620   0.810488   0.773  0.43954
## high.educSome College                   0.969506   0.762650   1.271  0.20381
## PDS_score:accumbens_posvsneg_feedback_z 0.184002   0.317322   0.580  0.56208
##
## (Intercept)
## PDS_score                              *
## accumbens_posvsneg_feedback_z
## race.ethnicity.5levelBlack
## race.ethnicity.5levelMixed             **
## race.ethnicity.5levelOther             *
## race.ethnicity.5levelWhite             *
## demo_race_hispanic1
## interview_age
## bmi                                    .
## household.income[>=200K]               *
## household.income[100K-200K]            *
## household.income[12K-16K]
## household.income[16K-25K]
## household.income[25K-35K]
## household.income[35K-50K]
## household.income[50K-75K]
## household.income[5K-12K]
## household.income[75K-100K]             .
## high.educBachelor
## high.educHS Diploma/GED
## high.educPost Graduate Degree
## high.educSome College
## PDS_score:accumbens_posvsneg_feedback_z
## ---
```

```
## Signif. codes:  0 '***' 0.001 '**' 0.01 '*' 0.05 '.' 0.1 ' ' 1
##
##
## R-sq.(adj) =  0.0225
## lmer.REML =  11240  Scale est. = 16.755    n = 1866
```

```
##                                              stdcoef      stdse
## X(Intercept)                             0.000000000 0.00000000
## XPDS_score                               0.056155814 0.02479252
## Xaccumbens_posvsneg_feedback_z           0.007541796 0.06404629
## Xrace.ethnicity.5levelBlack              0.030001006 0.05603249
## Xrace.ethnicity.5levelMixed              0.163146242 0.05616905
## Xrace.ethnicity.5levelOther              0.084291383 0.03978396
## Xrace.ethnicity.5levelWhite              0.175384100 0.07327754
## Xdemo_race_hispanic1                    -0.037522282 0.02799909
## Xinterview_age                          -0.008690114 0.02355590
## Xbmi                                     0.044416623 0.02464125
## Xhousehold.income[>=200K]               -0.130704673 0.05928404
## Xhousehold.income[100K-200K]            -0.166291440 0.08178483
## Xhousehold.income[12K-16K]              -0.029125176 0.03141596
## Xhousehold.income[16K-25K]               0.014325644 0.03870084
## Xhousehold.income[25K-35K]              -0.022849185 0.04221829
## Xhousehold.income[35K-50K]              -0.019201659 0.04812310
## Xhousehold.income[50K-75K]              -0.081265941 0.05988788
## Xhousehold.income[5K-12K]                0.008388296 0.03352324
## Xhousehold.income[75K-100K]             -0.103003714 0.06194347
## Xhigh.educBachelor                       0.076814548 0.07173067
## Xhigh.educHS Diploma/GED                -0.015709582 0.04188823
## Xhigh.educPost Graduate Degree           0.060259579 0.07794151
## Xhigh.educSome College                   0.083114848 0.06538122
## XPDS_score:accumbens_posvsneg_feedback_z  0.037188746 0.06413403
```

4.7 Model: CBCL internalizing factor ~ PDS x Caudate activity (feedback)

Female participants

```
##
## Family: gaussian
## Link function: identity
##
## Formula:
## cbcl_scr_syn_internal_r ~ PDS_score * caudate_posvsneg_feedback_z +
##     race.ethnicity.5level + demo_race_hispanic + interview_age +
##     bmi + household.income + high.educ
##
## Parametric coefficients:
##                                    Estimate Std. Error t value Pr(>|t|)
## (Intercept)                         5.95495    2.40525   2.476  0.01338 *
## PDS_score                           0.59398    0.19036   3.120  0.00183 **
## caudate_posvsneg_feedback_z        -0.38094    0.34707  -1.098  0.27252
## race.ethnicity.5levelBlack          0.04938    0.97223   0.051  0.95950
## race.ethnicity.5levelMixed          1.88251    0.93509   2.013  0.04424 *
## race.ethnicity.5levelOther          1.78700    1.04934   1.703  0.08874 .
```

```
## race.ethnicity.5levelWhite             1.46116    0.88123   1.658  0.09747 .
## demo_race_hispanic1                    0.14644    0.37830   0.387  0.69872
## interview_age                         -0.02327    0.01688  -1.379  0.16802
## bmi                                    0.05528    0.03327   1.661  0.09680 .
## household.income[>=200K]              -2.79696    0.90931  -3.076  0.00213 **
## household.income[100K-200K]           -2.31374    0.85389  -2.710  0.00680 **
## household.income[12K-16K]             -0.31789    1.10210  -0.288  0.77305
## household.income[16K-25K]             -0.39380    0.94903  -0.415  0.67823
## household.income[25K-35K]             -1.52636    0.90346  -1.689  0.09130 .
## household.income[35K-50K]             -0.95182    0.86454  -1.101  0.27106
## household.income[50K-75K]             -1.50619    0.86217  -1.747  0.08081 .
## household.income[5K-12K]              -0.76459    1.00742  -0.759  0.44798
## household.income[75K-100K]            -1.76200    0.86544  -2.036  0.04190 *
## high.educBachelor                     -0.04120    0.82261  -0.050  0.96007
## high.educHS Diploma/GED               -0.77545    0.83719  -0.926  0.35444
## high.educPost Graduate Degree          0.17832    0.83028   0.215  0.82997
## high.educSome College                  0.31797    0.77582   0.410  0.68197
## PDS_score:caudate_posvsneg_feedback_z  0.13666    0.19612   0.697  0.48602
## ---
## Signif. codes:  0 '***' 0.001 '**' 0.01 '*' 0.05 '.' 0.1 ' ' 1
##
##
## R-sq.(adj) =  0.0283
## lmer.REML =  11267  Scale est. = 11.217    n = 1845
```

```
##                                             stdcoef      stdse
## X(Intercept)                             0.000000000 0.00000000
## XPDS_score                               0.080127664 0.02567905
## Xcaudate_posvsneg_feedback_z            -0.061790493 0.05629539
## Xrace.ethnicity.5levelBlack              0.002936016 0.05780846
## Xrace.ethnicity.5levelMixed              0.118024950 0.05862625
## Xrace.ethnicity.5levelOther              0.074402336 0.04368979
## Xrace.ethnicity.5levelWhite              0.127501422 0.07689625
## Xdemo_race_hispanic1                     0.010875695 0.02809483
## Xinterview_age                          -0.032679751 0.02369566
## Xbmi                                     0.041450717 0.02494905
## Xhousehold.income[>=200K]               -0.178508485 0.05803430
## Xhousehold.income[100K-200K]            -0.202089467 0.07458134
## Xhousehold.income[12K-16K]              -0.008828063 0.03060656
## Xhousehold.income[16K-25K]              -0.014837076 0.03575607
## Xhousehold.income[25K-35K]              -0.066620456 0.03943286
## Xhousehold.income[35K-50K]              -0.051031883 0.04635266
## Xhousehold.income[50K-75K]              -0.095961462 0.05492964
## Xhousehold.income[5K-12K]               -0.024924730 0.03284088
## Xhousehold.income[75K-100K]             -0.119624475 0.05875606
## Xhigh.educBachelor                      -0.003495776 0.06980595
## Xhigh.educHS Diploma/GED                -0.036579485 0.03949183
## Xhigh.educPost Graduate Degree           0.016269242 0.07575316
## Xhigh.educSome College                   0.025647268 0.06257755
## XPDS_score:caudate_posvsneg_feedback_z   0.039508843 0.05670043
```

Male participants

```
##
```

```
## Family: gaussian
## Link function: identity
##
## Formula:
## cbcl_scr_syn_internal_r ~ PDS_score * caudate_posvsneg_feedback_z +
##     race.ethnicity.5level + demo_race_hispanic + interview_age +
##     bmi + household.income + high.educ
##
## Parametric coefficients:
##                                      Estimate Std. Error t value Pr(>|t|)
## (Intercept)                           3.53590    2.31130   1.530  0.12623
## PDS_score                             0.61267    0.24228   2.529  0.01153 *
## caudate_posvsneg_feedback_z           0.03180    0.35894   0.089  0.92941
## race.ethnicity.5levelBlack            0.30001    0.90680   0.331  0.74080
## race.ethnicity.5levelMixed            2.38657    0.87646   2.723  0.00653 **
## race.ethnicity.5levelOther            2.06988    1.02438   2.021  0.04346 *
## race.ethnicity.5levelWhite            1.87972    0.82230   2.286  0.02237 *
## demo_race_hispanic1                  -0.43626    0.35841  -1.217  0.22369
## interview_age                        -0.01461    0.01582  -0.924  0.35560
## bmi                                   0.06901    0.03478   1.984  0.04737 *
## household.income[>=200K]             -1.84592    0.93616  -1.972  0.04878 *
## household.income[100K-200K]          -1.57054    0.87890  -1.787  0.07411 .
## household.income[12K-16K]            -1.10084    1.13668  -0.968  0.33294
## household.income[16K-25K]             0.43391    0.95395   0.455  0.64927
## household.income[25K-35K]            -0.59678    0.93403  -0.639  0.52295
## household.income[35K-50K]            -0.14240    0.90392  -0.158  0.87484
## household.income[50K-75K]            -0.97524    0.87592  -1.113  0.26569
## household.income[5K-12K]              0.98571    1.02390   0.963  0.33583
## household.income[75K-100K]           -1.31514    0.89024  -1.477  0.13977
## high.educBachelor                     0.36080    0.80507   0.448  0.65409
## high.educHS Diploma/GED              -0.74726    0.83029  -0.900  0.36824
## high.educPost Graduate Degree         0.11236    0.81718   0.138  0.89065
## high.educSome College                 0.58216    0.76912   0.757  0.44919
## PDS_score:caudate_posvsneg_feedback_z  0.04556    0.24156   0.189  0.85042
## ---
## Signif. codes:  0 '***' 0.001 '**' 0.01 '*' 0.05 '.' 0.1 ' ' 1
##
##
## R-sq.(adj) =  0.0241
## lmer.REML =  11305  Scale est. = 16.437    n = 1871
```

```
##                                          stdcoef      stdse
## X(Intercept)                         0.000000000 0.00000000
## XPDS_score                           0.062807629 0.02483754
## Xcaudate_posvsneg_feedback_z         0.005493391 0.06200407
## Xrace.ethnicity.5levelBlack          0.018834118 0.05692760
## Xrace.ethnicity.5levelMixed          0.154573434 0.05676668
## Xrace.ethnicity.5levelOther          0.081104296 0.04013848
## Xrace.ethnicity.5levelWhite          0.169797230 0.07427952
## Xdemo_race_hispanic1                -0.033956662 0.02789781
## Xinterview_age                      -0.021719741 0.02350588
## Xbmi                                 0.048834394 0.02461003
## Xhousehold.income[>=200K]           -0.117284564 0.05948055
## Xhousehold.income[100K-200K]        -0.146741428 0.08211926
```

```
## Xhousehold.income[12K-16K]             -0.030231934 0.03121627
## Xhousehold.income[16K-25K]              0.017823386 0.03918496
## Xhousehold.income[25K-35K]             -0.026970661 0.04221202
## Xhousehold.income[35K-50K]             -0.007627371 0.04841793
## Xhousehold.income[50K-75K]             -0.067283930 0.06043166
## Xhousehold.income[5K-12K]               0.032551033 0.03381231
## Xhousehold.income[75K-100K]            -0.091853801 0.06217724
## Xhigh.educBachelor                      0.032012247 0.07142959
## Xhigh.educHS Diploma/GED               -0.037478496 0.04164322
## Xhigh.educPost Graduate Degree          0.010696765 0.07779336
## Xhigh.educSome College                  0.049661329 0.06560957
## XPDS_score:caudate_posvsneg_feedback_z  0.011702843 0.06204690
```

4.8 Model: CBCL internalizing factor ~ PDS x Putamen activity (feedback)

Female participants

```
##
## Family: gaussian
## Link function: identity
##
## Formula:
## cbcl_scr_syn_internal_r ~ PDS_score * putamen_posvsneg_feedback_z +
##     race.ethnicity.5level + demo_race_hispanic + interview_age +
##     bmi + household.income + high.educ
##
## Parametric coefficients:
##                                       Estimate Std. Error t value Pr(>|t|)
## (Intercept)                            5.70200    2.41205   2.364  0.01819 *
## PDS_score                              0.58110    0.19063   3.048  0.00233 **
## putamen_posvsneg_feedback_z           -0.15032    0.36813  -0.408  0.68308
## race.ethnicity.5levelBlack             0.11982    0.97377   0.123  0.90209
## race.ethnicity.5levelMixed             1.92004    0.93539   2.053  0.04025 *
## race.ethnicity.5levelOther             1.86909    1.05209   1.777  0.07581 .
## race.ethnicity.5levelWhite             1.48359    0.88187   1.682  0.09268 .
## demo_race_hispanic1                    0.15586    0.37857   0.412  0.68061
## interview_age                         -0.02120    0.01684  -1.258  0.20840
## bmi                                    0.05578    0.03336   1.672  0.09468 .
## household.income[>=200K]              -2.82159    0.91471  -3.085  0.00207 **
## household.income[100K-200K]           -2.32938    0.85988  -2.709  0.00681 **
## household.income[12K-16K]             -0.35123    1.10713  -0.317  0.75110
## household.income[16K-25K]             -0.44649    0.95195  -0.469  0.63911
## household.income[25K-35K]             -1.50449    0.90866  -1.656  0.09795 .
## household.income[35K-50K]             -0.97561    0.87048  -1.121  0.26254
## household.income[50K-75K]             -1.50875    0.86902  -1.736  0.08271 .
## household.income[5K-12K]              -0.81401    1.01548  -0.802  0.42289
## household.income[75K-100K]            -1.77382    0.87114  -2.036  0.04187 *
## high.educBachelor                     -0.04515    0.82669  -0.055  0.95645
## high.educHS Diploma/GED               -0.73742    0.84201  -0.876  0.38126
## high.educPost Graduate Degree          0.18641    0.83421   0.223  0.82320
## high.educSome College                  0.31740    0.78016   0.407  0.68418
## PDS_score:putamen_posvsneg_feedback_z  0.02997    0.20789   0.144  0.88540
## ---
```

```
## Signif. codes:  0 '***' 0.001 '**' 0.01 '*' 0.05 '.' 0.1 ' ' 1
##
##
## R-sq.(adj) =  0.0274
## lmer.REML =  11251  Scale est. = 11.22     n = 1842
```

```
##                                         stdcoef      stdse
## X(Intercept)                        0.000000000 0.00000000
## XPDS_score                          0.078367628 0.02570793
## Xputamen_posvsneg_feedback_z       -0.023140688 0.05667216
## Xrace.ethnicity.5levelBlack         0.007097139 0.05767964
## Xrace.ethnicity.5levelMixed         0.120647844 0.05877587
## Xrace.ethnicity.5levelOther         0.077078690 0.04338669
## Xrace.ethnicity.5levelWhite         0.129294460 0.07685459
## Xdemo_race_hispanic1                0.011566357 0.02809423
## Xinterview_age                     -0.029779802 0.02366429
## Xbmi                                0.041665145 0.02491818
## Xhousehold.income[>=200K]          -0.180154082 0.05840255
## Xhousehold.income[100K-200K]       -0.203296155 0.07504619
## Xhousehold.income[12K-16K]         -0.009759023 0.03076191
## Xhousehold.income[16K-25K]         -0.016934600 0.03610590
## Xhousehold.income[25K-35K]         -0.065697891 0.03967918
## Xhousehold.income[35K-50K]         -0.052331098 0.04669230
## Xhousehold.income[50K-75K]         -0.095995716 0.05529250
## Xhousehold.income[5K-12K]          -0.026549390 0.03312022
## Xhousehold.income[75K-100K]        -0.120473912 0.05916578
## Xhigh.educBachelor                 -0.003830144 0.07012637
## Xhigh.educHS Diploma/GED           -0.034671650 0.03958908
## Xhigh.educPost Graduate Degree      0.017003911 0.07609256
## Xhigh.educSome College              0.025627924 0.06299320
## XPDS_score:putamen_posvsneg_feedback_z  0.008231599 0.05710365
```

Male participants

```
##
## Family: gaussian
## Link function: identity
##
## Formula:
## cbcl_scr_syn_internal_r ~ PDS_score * putamen_posvsneg_feedback_z +
##     race.ethnicity.5level + demo_race_hispanic + interview_age +
##     bmi + household.income + high.educ
##
## Parametric coefficients:
##                                Estimate Std. Error t value Pr(>|t|)
## (Intercept)                     3.23860    2.30279   1.406  0.15978
## PDS_score                       0.58936    0.24186   2.437  0.01491 *
## putamen_posvsneg_feedback_z    -0.28224    0.36384  -0.776  0.43801
## race.ethnicity.5levelBlack      0.32769    0.90096   0.364  0.71611
## race.ethnicity.5levelMixed      2.44658    0.86903   2.815  0.00492 **
## race.ethnicity.5levelOther      2.06216    1.02080   2.020  0.04351 *
## race.ethnicity.5levelWhite      1.95265    0.81512   2.396  0.01670 *
## demo_race_hispanic1            -0.51329    0.36068  -1.423  0.15487
```

```
## interview_age                        -0.01210    0.01584  -0.764  0.44502
## bmi                                   0.07015    0.03482   2.015  0.04408 *
## household.income[>=200K]             -1.95651    0.93025  -2.103  0.03558 *
## household.income[100K-200K]          -1.63711    0.87487  -1.871  0.06147 .
## household.income[12K-16K]            -0.99974    1.12981  -0.885  0.37634
## household.income[16K-25K]             0.37793    0.94867   0.398  0.69039
## household.income[25K-35K]            -0.42574    0.93033  -0.458  0.64728
## household.income[35K-50K]            -0.25326    0.90112  -0.281  0.77870
## household.income[50K-75K]            -1.06940    0.87224  -1.226  0.22034
## household.income[5K-12K]              0.88544    1.01651   0.871  0.38384
## household.income[75K-100K]           -1.38891    0.88663  -1.567  0.11740
## high.educBachelor                     0.39446    0.79513   0.496  0.61989
## high.educHS Diploma/GED              -0.74481    0.82319  -0.905  0.36570
## high.educPost Graduate Degree         0.15626    0.80621   0.194  0.84634
## high.educSome College                 0.57082    0.75886   0.752  0.45202
## PDS_score:putamen_posvsneg_feedback_z  0.28037    0.24472   1.146  0.25206
## ---
## Signif. codes:  0 '***' 0.001 '**' 0.01 '*' 0.05 '.' 0.1 ' ' 1
##
##
## R-sq.(adj) =  0.0244
## lmer.REML =  11347  Scale est. = 16.606    n = 1876
```

```
##                                          stdcoef      stdse
## X(Intercept)                          0.00000000 0.00000000
## XPDS_score                            0.06025975 0.02472946
## Xputamen_posvsneg_feedback_z         -0.04824416 0.06219193
## Xrace.ethnicity.5levelBlack           0.02043040 0.05617131
## Xrace.ethnicity.5levelMixed           0.15827999 0.05622139
## Xrace.ethnicity.5levelOther           0.08039983 0.03979890
## Xrace.ethnicity.5levelWhite           0.17577701 0.07337725
## Xdemo_race_hispanic1                 -0.03972213 0.02791205
## Xinterview_age                       -0.01793129 0.02347316
## Xbmi                                  0.04951983 0.02457927
## Xhousehold.income[>=200K]            -0.12419310 0.05904915
## Xhousehold.income[100K-200K]         -0.15235433 0.08141771
## Xhousehold.income[12K-16K]           -0.02767713 0.03127823
## Xhousehold.income[16K-25K]            0.01553328 0.03899078
## Xhousehold.income[25K-35K]           -0.01923185 0.04202548
## Xhousehold.income[35K-50K]           -0.01345803 0.04788415
## Xhousehold.income[50K-75K]           -0.07331023 0.05979478
## Xhousehold.income[5K-12K]             0.02935385 0.03369913
## Xhousehold.income[75K-100K]          -0.09639430 0.06153449
## Xhigh.educBachelor                    0.03486034 0.07027004
## Xhigh.educHS Diploma/GED             -0.03717142 0.04108311
## Xhigh.educPost Graduate Degree        0.01481673 0.07644500
## Xhigh.educSome College                0.04850516 0.06448349
## XPDS_score:putamen_posvsneg_feedback_z  0.07120809 0.06215161
```

4.9 Model: CBCL internalizing factor ~ PDS x Lateral OFC activity (feedback stage)

Female participants

```
##
## Family: gaussian
## Link function: identity
##
## Formula:
## cbcl_scr_syn_internal_r ~ PDS_score * lOFC_posvsneg_feedback_z +
##     race.ethnicity.5level + demo_race_hispanic + interview_age +
##     bmi + household.income + high.educ
##
## Parametric coefficients:
##                                   Estimate Std. Error t value Pr(>|t|)
## (Intercept)                        5.60892    2.39866   2.338  0.01948 *
## PDS_score                          0.58998    0.19016   3.103  0.00195 **
## lOFC_posvsneg_feedback_z          -0.57997    0.58602  -0.990  0.32247
## race.ethnicity.5levelBlack         0.09947    0.97027   0.103  0.91836
## race.ethnicity.5levelMixed         1.85916    0.93345   1.992  0.04655 *
## race.ethnicity.5levelOther         2.07810    1.05341   1.973  0.04868 *
## race.ethnicity.5levelWhite         1.48751    0.87909   1.692  0.09080 .
## demo_race_hispanic1                0.03546    0.37627   0.094  0.92494
## interview_age                     -0.02108    0.01681  -1.254  0.21017
## bmi                                0.05356    0.03321   1.613  0.10695
## household.income[>=200K]          -2.49667    0.92153  -2.709  0.00681 **
## household.income[100K-200K]       -2.02343    0.86670  -2.335  0.01967 *
## household.income[12K-16K]          0.09671    1.10456   0.088  0.93024
## household.income[16K-25K]         -0.08282    0.96216  -0.086  0.93141
## household.income[25K-35K]         -1.16589    0.91780  -1.270  0.20414
## household.income[35K-50K]         -0.66086    0.87846  -0.752  0.45197
## household.income[50K-75K]         -1.16443    0.87515  -1.331  0.18351
## household.income[5K-12K]          -0.41258    1.01989  -0.405  0.68587
## household.income[75K-100K]        -1.41608    0.87860  -1.612  0.10719
## high.educBachelor                 -0.24887    0.82059  -0.303  0.76171
## high.educHS Diploma/GED           -1.08527    0.83624  -1.298  0.19452
## high.educPost Graduate Degree     -0.02498    0.82749  -0.030  0.97593
## high.educSome College              0.18198    0.77375   0.235  0.81408
## PDS_score:lOFC_posvsneg_feedback_z  0.21813    0.31889   0.684  0.49404
## ---
## Signif. codes:  0 '***' 0.001 '**' 0.01 '*' 0.05 '.' 0.1 ' ' 1
##
##
## R-sq.(adj) =  0.0291
## lmer.REML =  11223  Scale est. = 11.241    n = 1839
```

```
##                                          stdcoef      stdse
## X(Intercept)                         0.000000000 0.00000000
## XPDS_score                           0.079756342 0.02570690
## XlOFC_posvsneg_feedback_z           -0.054937454 0.05551104
## Xrace.ethnicity.5levelBlack          0.005894529 0.05749802
## Xrace.ethnicity.5levelMixed          0.116265146 0.05837485
## Xrace.ethnicity.5levelOther          0.085485365 0.04333331
```

```
## Xrace.ethnicity.5levelWhite          0.129558267 0.07656653
## Xdemo_race_hispanic1                 0.002640650 0.02802330
## Xinterview_age                      -0.029670010 0.02366884
## Xbmi                                 0.040236871 0.02494765
## Xhousehold.income[>=200K]           -0.159259378 0.05878356
## Xhousehold.income[100K-200K]        -0.177252834 0.07592301
## Xhousehold.income[12K-16K]           0.002726192 0.03113678
## Xhousehold.income[16K-25K]          -0.003130289 0.03636474
## Xhousehold.income[25K-35K]          -0.050816813 0.04000340
## Xhousehold.income[35K-50K]          -0.035442522 0.04711254
## Xhousehold.income[50K-75K]          -0.074407515 0.05592263
## Xhousehold.income[5K-12K]           -0.013492276 0.03335255
## Xhousehold.income[75K-100K]         -0.096281323 0.05973735
## Xhigh.educBachelor                  -0.021172970 0.06981336
## Xhigh.educHS Diploma/GED            -0.051160209 0.03942082
## Xhigh.educPost Graduate Degree      -0.002283122 0.07564540
## Xhigh.educSome College               0.014673180 0.06238723
## XPDS_score:lOFC_posvsneg_feedback_z  0.037883581 0.05538266
```

Male participants

```
##
## Family: gaussian
## Link function: identity
##
## Formula:
## cbcl_scr_syn_internal_r ~ PDS_score * lOFC_posvsneg_feedback_z +
##     race.ethnicity.5level + demo_race_hispanic + interview_age +
##     bmi + household.income + high.educ
##
## Parametric coefficients:
##                               Estimate Std. Error t value Pr(>|t|)
## (Intercept)                   2.080824   2.283191   0.911  0.36222
## PDS_score                     0.529966   0.240905   2.200  0.02794 *
## lOFC_posvsneg_feedback_z     -0.108625   0.563419  -0.193  0.84714
## race.ethnicity.5levelBlack    0.414574   0.893099   0.464  0.64256
## race.ethnicity.5levelMixed    2.481859   0.860378   2.885  0.00396 **
## race.ethnicity.5levelOther    2.069686   1.011429   2.046  0.04087 *
## race.ethnicity.5levelWhite    1.888120   0.806229   2.342  0.01929 *
## demo_race_hispanic1          -0.517870   0.356837  -1.451  0.14687
## interview_age                -0.006702   0.015670  -0.428  0.66890
## bmi                           0.075125   0.034658   2.168  0.03031 *
## household.income[>=200K]     -2.102524   0.922055  -2.280  0.02271 *
## household.income[100K-200K]  -1.837266   0.867773  -2.117  0.03437 *
## household.income[12K-16K]    -1.152235   1.118463  -1.030  0.30305
## household.income[16K-25K]     0.243963   0.949137   0.257  0.79718
## household.income[25K-35K]    -0.546735   0.923127  -0.592  0.55375
## household.income[35K-50K]    -0.424080   0.892141  -0.475  0.63459
## household.income[50K-75K]    -1.197564   0.864793  -1.385  0.16628
## household.income[5K-12K]      0.285937   1.011343   0.283  0.77742
## household.income[75K-100K]   -1.524302   0.878746  -1.735  0.08297 .
## high.educBachelor             1.150841   0.795300   1.447  0.14805
## high.educHS Diploma/GED      -0.030242   0.822387  -0.037  0.97067
```

```
## high.educPost Graduate Degree       0.881632   0.806089   1.094  0.27422
## high.educSome College               1.243346   0.760935   1.634  0.10243
## PDS_score:lOFC_posvsneg_feedback_z  0.082479   0.381557   0.216  0.82888
## ---
## Signif. codes:  0 '***' 0.001 '**' 0.01 '*' 0.05 '.' 0.1 ' ' 1
##
##
## R-sq.(adj) =  0.0218
## lmer.REML =  11298  Scale est. = 15.885    n = 1875
```

```
##                                         stdcoef      stdse
## X(Intercept)                         0.000000000 0.00000000
## XPDS_score                           0.054714194 0.02487126
## XlOFC_posvsneg_feedback_z           -0.012063629 0.06257204
## Xrace.ethnicity.5levelBlack          0.025948834 0.05590044
## Xrace.ethnicity.5levelMixed          0.162232933 0.05624075
## Xrace.ethnicity.5levelOther          0.081176808 0.03967004
## Xrace.ethnicity.5levelWhite          0.171511987 0.07323579
## Xdemo_race_hispanic1                -0.040610870 0.02798281
## Xinterview_age                      -0.010055779 0.02350976
## Xbmi                                 0.053387000 0.02462932
## Xhousehold.income[>=200K]           -0.135364113 0.05936348
## Xhousehold.income[100K-200K]        -0.173260901 0.08183416
## Xhousehold.income[12K-16K]          -0.032291830 0.03134535
## Xhousehold.income[16K-25K]           0.009922627 0.03860388
## Xhousehold.income[25K-35K]          -0.024889012 0.04202354
## Xhousehold.income[35K-50K]          -0.022881949 0.04813698
## Xhousehold.income[50K-75K]          -0.083104162 0.06001172
## Xhousehold.income[5K-12K]            0.009511024 0.03363995
## Xhousehold.income[75K-100K]         -0.107089559 0.06173612
## Xhigh.educBachelor                   0.103125757 0.07126609
## Xhigh.educHS Diploma/GED            -0.001533282 0.04169561
## Xhigh.educPost Graduate Degree       0.084638474 0.07738617
## Xhigh.educSome College               0.106636287 0.06526199
## XPDS_score:lOFC_posvsneg_feedback_z  0.013546621 0.06266848
```

4.10 Model: CBCL internalizing factor ~ PDS x Medial OFC activity (feedback stage)

Female participants

```
##
## Family: gaussian
## Link function: identity
##
## Formula:
## cbcl_scr_syn_internal_r ~ PDS_score * mOFC_posvsneg_feedback_z +
##     race.ethnicity.5level + demo_race_hispanic + interview_age +
##     bmi + household.income + high.educ
##
## Parametric coefficients:
##                                    Estimate Std. Error t value Pr(>|t|)
## (Intercept)                         6.00726    2.40169   2.501  0.01246 *
```

```
## PDS_score                          0.60093    0.19079   3.150  0.00166 **
## mOFC_posvsneg_feedback_z          -0.55843    0.50297  -1.110  0.26703
## race.ethnicity.5levelBlack         0.07234    0.97308   0.074  0.94075
## race.ethnicity.5levelMixed         1.85068    0.93641   1.976  0.04827 *
## race.ethnicity.5levelOther         1.91635    1.05082   1.824  0.06837 .
## race.ethnicity.5levelWhite         1.46991    0.88142   1.668  0.09555 .
## demo_race_hispanic1                0.09371    0.37709   0.249  0.80377
## interview_age                     -0.02328    0.01687  -1.380  0.16790
## bmi                                0.05185    0.03343   1.551  0.12106
## household.income[>=200K]          -2.76246    0.91490  -3.019  0.00257 **
## household.income[100K-200K]       -2.26204    0.85933  -2.632  0.00855 **
## household.income[12K-16K]         -0.39986    1.10498  -0.362  0.71749
## household.income[16K-25K]         -0.32530    0.95575  -0.340  0.73363
## household.income[25K-35K]         -1.34975    0.91223  -1.480  0.13915
## household.income[35K-50K]         -0.87015    0.87076  -0.999  0.31778
## household.income[50K-75K]         -1.45739    0.86729  -1.680  0.09305 .
## household.income[5K-12K]          -0.64304    1.01385  -0.634  0.52599
## household.income[75K-100K]        -1.67708    0.87056  -1.926  0.05421 .
## high.educBachelor                 -0.09457    0.82140  -0.115  0.90835
## high.educHS Diploma/GED           -0.83901    0.83421  -1.006  0.31467
## high.educPost Graduate Degree      0.13520    0.82795   0.163  0.87030
## high.educSome College              0.25943    0.77362   0.335  0.73740
## PDS_score:mOFC_posvsneg_feedback_z  0.19606    0.27841   0.704  0.48139
## ---
## Signif. codes:  0 '***' 0.001 '**' 0.01 '*' 0.05 '.' 0.1 ' ' 1
##
##
## R-sq.(adj) =  0.0286
## lmer.REML =  11233  Scale est. = 11.429    n = 1839
```

```
##                                          stdcoef      stdse
## X(Intercept)                         0.000000000 0.00000000
## XPDS_score                           0.081018690 0.02572313
## XmOFC_posvsneg_feedback_z           -0.064901395 0.05845494
## Xrace.ethnicity.5levelBlack          0.004258626 0.05728603
## Xrace.ethnicity.5levelMixed          0.115473393 0.05842740
## Xrace.ethnicity.5levelOther          0.079856825 0.04378922
## Xrace.ethnicity.5levelWhite          0.127799062 0.07663343
## Xdemo_race_hispanic1                 0.006978301 0.02808023
## Xinterview_age                      -0.032662694 0.02367669
## Xbmi                                 0.038825346 0.02503118
## Xhousehold.income[>=200K]           -0.175506983 0.05812622
## Xhousehold.income[100K-200K]        -0.197708175 0.07510717
## Xhousehold.income[12K-16K]          -0.011114550 0.03071455
## Xhousehold.income[16K-25K]          -0.012266786 0.03604089
## Xhousehold.income[25K-35K]          -0.058431746 0.03949119
## Xhousehold.income[35K-50K]          -0.046432321 0.04646472
## Xhousehold.income[50K-75K]          -0.092917765 0.05529475
## Xhousehold.income[5K-12K]           -0.020981379 0.03308005
## Xhousehold.income[75K-100K]         -0.114098825 0.05922801
## Xhigh.educBachelor                  -0.008032125 0.06976492
## Xhigh.educHS Diploma/GED            -0.039610562 0.03938409
## Xhigh.educPost Graduate Degree       0.012331474 0.07551664
## Xhigh.educSome College               0.020821955 0.06209104
```

```
## XPDS_score:mOFC_posvsneg_feedback_z  0.041442307 0.05884913
```

Male participants

```
##
## Family: gaussian
## Link function: identity
##
## Formula:
## cbcl_scr_syn_internal_r ~ PDS_score * mOFC_posvsneg_feedback_z +
##     race.ethnicity.5level + demo_race_hispanic + interview_age +
##     bmi + household.income + high.educ
##
## Parametric coefficients:
##                                   Estimate Std. Error t value Pr(>|t|)
## (Intercept)                       2.077601   2.281991   0.910   0.3627
## PDS_score                         0.524030   0.241049   2.174   0.0298 *
## mOFC_posvsneg_feedback_z         -0.032637   0.516562  -0.063   0.9496
## race.ethnicity.5levelBlack        0.382031   0.892778   0.428   0.6688
## race.ethnicity.5levelMixed        2.486176   0.860388   2.890   0.0039 **
## race.ethnicity.5levelOther        2.061880   1.011426   2.039   0.0416 *
## race.ethnicity.5levelWhite        1.881765   0.806204   2.334   0.0197 *
## demo_race_hispanic1              -0.518290   0.356959  -1.452   0.1467
## interview_age                    -0.006806   0.015672  -0.434   0.6641
## bmi                               0.075274   0.034645   2.173   0.0299 *
## household.income[>=200K]         -2.066003   0.921358  -2.242   0.0251 *
## household.income[100K-200K]      -1.807695   0.866439  -2.086   0.0371 *
## household.income[12K-16K]        -1.110884   1.117270  -0.994   0.3202
## household.income[16K-25K]         0.291152   0.947880   0.307   0.7588
## household.income[25K-35K]        -0.537687   0.922741  -0.583   0.5602
## household.income[35K-50K]        -0.406640   0.891388  -0.456   0.6483
## household.income[50K-75K]        -1.172514   0.863555  -1.358   0.1747
## household.income[5K-12K]          0.313643   1.010413   0.310   0.7563
## household.income[75K-100K]       -1.502330   0.877866  -1.711   0.0872 .
## high.educBachelor                 1.136318   0.794576   1.430   0.1529
## high.educHS Diploma/GED          -0.028502   0.821422  -0.035   0.9723
## high.educPost Graduate Degree     0.879156   0.805521   1.091   0.2752
## high.educSome College             1.238716   0.760052   1.630   0.1033
## PDS_score:mOFC_posvsneg_feedback_z  0.204529   0.358658   0.570   0.5686
## ---
## Signif. codes:  0 '***' 0.001 '**' 0.01 '*' 0.05 '.' 0.1 ' ' 1
##
##
## R-sq.(adj) =  0.0228
## lmer.REML =  11281  Scale est. = 15.919    n = 1872
```

```
##                                        stdcoef      stdse
## X(Intercept)                       0.000000000 0.00000000
## XPDS_score                         0.054055642 0.02486511
## XmOFC_posvsneg_feedback_z         -0.004094401 0.06480331
## Xrace.ethnicity.5levelBlack        0.023961152 0.05599552
## Xrace.ethnicity.5levelMixed        0.162508698 0.05623920
## Xrace.ethnicity.5levelOther        0.080873762 0.03967147
```

```
## Xrace.ethnicity.5levelWhite          0.170977978 0.07325204
## Xdemo_race_hispanic1                -0.040638993 0.02798911
## Xinterview_age                      -0.010203465 0.02349402
## Xbmi                                 0.053463672 0.02460702
## Xhousehold.income[>=200K]           -0.132490591 0.05908570
## Xhousehold.income[100K-200K]        -0.170285435 0.08161880
## Xhousehold.income[12K-16K]          -0.031134697 0.03131366
## Xhousehold.income[16K-25K]           0.011842332 0.03855415
## Xhousehold.income[25K-35K]          -0.024477756 0.04200704
## Xhousehold.income[35K-50K]          -0.021941005 0.04809650
## Xhousehold.income[50K-75K]          -0.081488380 0.06001608
## Xhousehold.income[5K-12K]            0.010433121 0.03361066
## Xhousehold.income[75K-100K]         -0.105539119 0.06167030
## Xhigh.educBachelor                   0.101683407 0.07110260
## Xhigh.educHS Diploma/GED            -0.001445073 0.04164719
## Xhigh.educPost Graduate Degree       0.084367250 0.07730090
## Xhigh.educSome College               0.106141851 0.06512653
## XPDS_score:mOFC_posvsneg_feedback_z  0.036990231 0.06486542
```

4.11 Model: CBCL internalizing factor ~ PDS x BIS-BAS

Female participants

```
##
## Family: gaussian
## Link function: identity
##
## Formula:
## cbcl_scr_syn_internal_r ~ PDS_score * bisbas_ss_basm_rr + race.ethnicity.5level +
##     demo_race_hispanic + interview_age + bmi + household.income +
##     high.educ
##
## Parametric coefficients:
##                               Estimate Std. Error t value Pr(>|t|)
## (Intercept)                   3.637801   2.358714   1.542 0.123138
## PDS_score                     1.334748   0.574777   2.322 0.020306 *
## bisbas_ss_basm_rr             0.057824   0.114880   0.503 0.614773
## race.ethnicity.5levelBlack   -0.712311   0.865482  -0.823 0.410577
## race.ethnicity.5levelMixed    1.216392   0.845298   1.439 0.150279
## race.ethnicity.5levelOther    1.831253   0.961528   1.905 0.056962 .
## race.ethnicity.5levelWhite    1.241210   0.796866   1.558 0.119458
## demo_race_hispanic1          -0.200009   0.342610  -0.584 0.559423
## interview_age                -0.008781   0.015087  -0.582 0.560618
## bmi                           0.068690   0.029543   2.325 0.020152 *
## household.income[>=200K]     -2.700557   0.775868  -3.481 0.000509 ***
## household.income[100K-200K]  -2.087601   0.721582  -2.893 0.003849 **
## household.income[12K-16K]    -0.149027   0.964289  -0.155 0.877192
## household.income[16K-25K]     0.225764   0.801051   0.282 0.778095
## household.income[25K-35K]    -0.912321   0.760485  -1.200 0.230392
## household.income[35K-50K]    -1.048597   0.730909  -1.435 0.151519
## household.income[50K-75K]    -1.082009   0.726901  -1.489 0.136745
## household.income[5K-12K]     -0.543702   0.844069  -0.644 0.519544
## household.income[75K-100K]   -1.490177   0.731175  -2.038 0.041654 *
```

```
## high.educBachelor              0.177135   0.732186   0.242 0.808858
## high.educHS Diploma/GED       -0.586376   0.730046  -0.803 0.421937
## high.educPost Graduate Degree  0.376779   0.738616   0.510 0.610018
## high.educSome College          0.552591   0.689514   0.801 0.422968
## PDS_score:bisbas_ss_basm_rr   -0.092385   0.062092  -1.488 0.136920
## ---
## Signif. codes:  0 '***' 0.001 '**' 0.01 '*' 0.05 '.' 0.1 ' ' 1
##
##
## R-sq.(adj) =  0.0314
## lmer.REML =  14840  Scale est. = 13.442    n = 2409
```

```
##                                    stdcoef      stdse
## X(Intercept)                    0.000000000 0.00000000
## XPDS_score                      0.179503789 0.07729904
## Xbisbas_ss_basm_rr              0.024912135 0.04949379
## Xrace.ethnicity.5levelBlack    -0.045562475 0.05535993
## Xrace.ethnicity.5levelMixed     0.074600696 0.05184170
## Xrace.ethnicity.5levelOther     0.071011218 0.03728555
## Xrace.ethnicity.5levelWhite     0.107862005 0.06924825
## Xdemo_race_hispanic1           -0.014295157 0.02448722
## Xinterview_age                 -0.012129691 0.02084118
## Xbmi                            0.051233186 0.02203510
## Xhousehold.income[>=200K]      -0.166772842 0.04791369
## Xhousehold.income[100K-200K]   -0.176281125 0.06093182
## Xhousehold.income[12K-16K]     -0.003988730 0.02580938
## Xhousehold.income[16K-25K]      0.008566186 0.03039440
## Xhousehold.income[25K-35K]     -0.039963063 0.03331209
## Xhousehold.income[35K-50K]     -0.054477829 0.03797297
## Xhousehold.income[50K-75K]     -0.067990077 0.04567616
## Xhousehold.income[5K-12K]      -0.018050271 0.02802212
## Xhousehold.income[75K-100K]    -0.098735273 0.04844575
## Xhigh.educBachelor              0.014515405 0.05999925
## Xhigh.educHS Diploma/GED       -0.028826462 0.03588932
## Xhigh.educPost Graduate Degree  0.033389120 0.06545407
## Xhigh.educSome College          0.044280099 0.05525197
## XPDS_score:bisbas_ss_basm_rr   -0.136013387 0.09141533
```

Male participants

```
##
## Family: gaussian
## Link function: identity
##
## Formula:
## cbcl_scr_syn_internal_r ~ PDS_score * bisbas_ss_basm_rr + race.ethnicity.5level +
##     demo_race_hispanic + interview_age + bmi + household.income +
##     high.educ
##
## Parametric coefficients:
##                               Estimate Std. Error t value Pr(>|t|)
## (Intercept)                   5.865014   2.303166   2.547 0.010938 *
## PDS_score                    -0.605716   0.811519  -0.746 0.455495
```

```
## bisbas_ss_basm_rr            -0.220969   0.122821  -1.799 0.072118 .
## race.ethnicity.5levelBlack     0.421737   0.786441   0.536 0.591825
## race.ethnicity.5levelMixed     1.865871   0.767303   2.432 0.015094 *
## race.ethnicity.5levelOther     1.424835   0.903016   1.578 0.114719
## race.ethnicity.5levelWhite     1.585856   0.717905   2.209 0.027262 *
## demo_race_hispanic1          -0.371595   0.322141  -1.154 0.248804
## interview_age                -0.009656   0.014212  -0.679 0.496925
## bmi                           0.065265   0.030339   2.151 0.031551 *
## household.income[>=200K]     -2.594882   0.753558  -3.444 0.000583 ***
## household.income[100K-200K]  -2.426943   0.697184  -3.481 0.000508 ***
## household.income[12K-16K]    -1.233492   0.936539  -1.317 0.187930
## household.income[16K-25K]    -0.443807   0.769274  -0.577 0.564046
## household.income[25K-35K]    -1.256360   0.754845  -1.664 0.096154 .
## household.income[35K-50K]    -0.937532   0.724947  -1.293 0.196042
## household.income[50K-75K]    -1.673369   0.693443  -2.413 0.015885 *
## household.income[5K-12K]      0.587305   0.820960   0.715 0.474433
## household.income[75K-100K]   -2.109108   0.709044  -2.975 0.002961 **
## high.educBachelor             0.562795   0.696298   0.808 0.419012
## high.educHS Diploma/GED      -0.319786   0.705764  -0.453 0.650510
## high.educPost Graduate Degree  0.480646   0.707904   0.679 0.497217
## high.educSome College         0.699610   0.663462   1.054 0.291760
## PDS_score:bisbas_ss_basm_rr    0.147564   0.085611   1.724 0.084890 .
## ---
## Signif. codes:  0 '***' 0.001 '**' 0.01 '*' 0.05 '.' 0.1 ' ' 1
##
##
## R-sq.(adj) =  0.0249
## lmer.REML =  16098  Scale est. = 14.709    n = 2613

##                                    stdcoef      stdse
## X(Intercept)                    0.00000000 0.00000000
## XPDS_score                     -0.06036174 0.08087072
## Xbisbas_ss_basm_rr             -0.09406723 0.05228542
## Xrace.ethnicity.5levelBlack     0.02697612 0.05030417
## Xrace.ethnicity.5levelMixed     0.11380337 0.04679943
## Xrace.ethnicity.5levelOther     0.05316247 0.03369270
## Xrace.ethnicity.5levelWhite     0.13795829 0.06245267
## Xdemo_race_hispanic1           -0.02703417 0.02343626
## Xinterview_age                 -0.01348278 0.01984417
## Xbmi                            0.04466200 0.02076121
## Xhousehold.income[>=200K]      -0.15633888 0.04540106
## Xhousehold.income[100K-200K]   -0.21067020 0.06051887
## Xhousehold.income[12K-16K]     -0.03260483 0.02475550
## Xhousehold.income[16K-25K]     -0.01779821 0.03085056
## Xhousehold.income[25K-35K]     -0.05413042 0.03252256
## Xhousehold.income[35K-50K]     -0.04715080 0.03645935
## Xhousehold.income[50K-75K]     -0.10924760 0.04527216
## Xhousehold.income[5K-12K]       0.01935868 0.02706035
## Xhousehold.income[75K-100K]    -0.13708533 0.04608564
## Xhigh.educBachelor              0.04711383 0.05828998
## Xhigh.educHS Diploma/GED       -0.01601198 0.03533822
## Xhigh.educPost Graduate Degree  0.04254683 0.06266372
## Xhigh.educSome College          0.05667664 0.05374824
## XPDS_score:bisbas_ss_basm_rr    0.16583847 0.09621328
```

4.12 Model: CBCL internalizing factor ~ PDS x MID reaction time (large reward vs. neutral)

Female participants

```
##
## Family: gaussian
## Link function: identity
##
## Formula:
## cbcl_scr_syn_internal_r ~ PDS_score * rt_diff_large_neutral_z +
##     race.ethnicity.5level + demo_race_hispanic + interview_age +
##     bmi + household.income + high.educ
##
## Parametric coefficients:
##                                  Estimate Std. Error t value Pr(>|t|)
## (Intercept)                       7.00246    2.30559   3.037  0.00242 **
## PDS_score                         0.60096    0.18439   3.259  0.00114 **
## rt_diff_large_neutral_z           0.25981    0.32290   0.805  0.42115
## race.ethnicity.5levelBlack        0.06072    0.92742   0.065  0.94781
## race.ethnicity.5levelMixed        1.67326    0.89776   1.864  0.06250 .
## race.ethnicity.5levelOther        2.11732    1.00913   2.098  0.03602 *
## race.ethnicity.5levelWhite        1.44877    0.84351   1.718  0.08604 .
## demo_race_hispanic1               0.13356    0.36730   0.364  0.71618
## interview_age                    -0.03391    0.01620  -2.093  0.03645 *
## bmi                               0.06141    0.03217   1.909  0.05644 .
## household.income[>=200K]         -2.33399    0.87062  -2.681  0.00741 **
## household.income[100K-200K]      -1.79326    0.81630  -2.197  0.02815 *
## household.income[12K-16K]        -0.52649    1.04709  -0.503  0.61515
## household.income[16K-25K]         0.28969    0.91306   0.317  0.75107
## household.income[25K-35K]        -0.98540    0.86444  -1.140  0.25446
## household.income[35K-50K]        -0.94460    0.82358  -1.147  0.25154
## household.income[50K-75K]        -1.13876    0.82599  -1.379  0.16816
## household.income[5K-12K]         -0.38560    0.96890  -0.398  0.69069
## household.income[75K-100K]       -1.33344    0.82533  -1.616  0.10633
## high.educBachelor                -0.22552    0.79915  -0.282  0.77782
## high.educHS Diploma/GED          -0.95627    0.81444  -1.174  0.24048
## high.educPost Graduate Degree    -0.13244    0.80540  -0.164  0.86941
## high.educSome College            -0.07357    0.75177  -0.098  0.92205
## PDS_score:rt_diff_large_neutral_z -0.09667    0.18020  -0.536  0.59172
## ---
## Signif. codes:  0 '***' 0.001 '**' 0.01 '*' 0.05 '.' 0.1 ' ' 1
##
##
## R-sq.(adj) =  0.0242
## lmer.REML =  12059  Scale est. = 11.823    n = 1977
```

```
##                                       stdcoef      stdse
## X(Intercept)                      0.000000000 0.00000000
## XPDS_score                        0.081250751 0.02493008
## Xrt_diff_large_neutral_z          0.045416205 0.05644574
## Xrace.ethnicity.5levelBlack       0.003700591 0.05652643
## Xrace.ethnicity.5levelMixed       0.104045545 0.05582375
## Xrace.ethnicity.5levelOther       0.087454751 0.04168161
```

```
## Xrace.ethnicity.5levelWhite         0.127128688 0.07401745
## Xdemo_race_hispanic1                0.009885902 0.02718730
## Xinterview_age                     -0.048148333 0.02300064
## Xbmi                                0.046055474 0.02412809
## Xhousehold.income[>=200K]          -0.150111970 0.05599411
## Xhousehold.income[100K-200K]       -0.157565785 0.07172436
## Xhousehold.income[12K-16K]         -0.015031285 0.02989410
## Xhousehold.income[16K-25K]          0.010810433 0.03407315
## Xhousehold.income[25K-35K]         -0.042959594 0.03768626
## Xhousehold.income[35K-50K]         -0.051333637 0.04475666
## Xhousehold.income[50K-75K]         -0.072285457 0.05243173
## Xhousehold.income[5K-12K]          -0.012527272 0.03147723
## Xhousehold.income[75K-100K]        -0.091093965 0.05638263
## Xhigh.educBachelor                 -0.019202358 0.06804479
## Xhigh.educHS Diploma/GED           -0.044401907 0.03781634
## Xhigh.educPost Graduate Degree     -0.012154167 0.07391455
## Xhigh.educSome College             -0.005999493 0.06130190
## XPDS_score:rt_diff_large_neutral_z -0.030469249 0.05679952
```

Male participants

```
##
## Family: gaussian
## Link function: identity
##
## Formula:
## cbcl_scr_syn_internal_r ~ PDS_score * rt_diff_large_neutral_z +
##     race.ethnicity.5level + demo_race_hispanic + interview_age +
##     bmi + household.income + high.educ
##
## Parametric coefficients:
##                                Estimate Std. Error t value Pr(>|t|)
## (Intercept)                    2.377634   2.216466   1.073  0.28353
## PDS_score                      0.480939   0.231172   2.080  0.03761 *
## rt_diff_large_neutral_z        0.647071   0.349244   1.853  0.06406 .
## race.ethnicity.5levelBlack     0.099216   0.883617   0.112  0.91061
## race.ethnicity.5levelMixed     2.032741   0.853305   2.382  0.01730 *
## race.ethnicity.5levelOther     1.519209   1.001915   1.516  0.12960
## race.ethnicity.5levelWhite     1.604974   0.803966   1.996  0.04603 *
## demo_race_hispanic1           -0.501886   0.344113  -1.458  0.14486
## interview_age                 -0.004826   0.015223  -0.317  0.75125
## bmi                            0.086967   0.033059   2.631  0.00859 **
## household.income[>=200K]      -2.234921   0.889364  -2.513  0.01205 *
## household.income[100K-200K]   -1.821515   0.833646  -2.185  0.02900 *
## household.income[12K-16K]     -1.405555   1.083403  -1.297  0.19466
## household.income[16K-25K]     -0.007106   0.905175  -0.008  0.99374
## household.income[25K-35K]     -0.468281   0.883812  -0.530  0.59628
## household.income[35K-50K]     -0.259682   0.858281  -0.303  0.76226
## household.income[50K-75K]     -1.338775   0.829944  -1.613  0.10688
## household.income[5K-12K]       0.965603   0.974547   0.991  0.32189
## household.income[75K-100K]    -1.662622   0.844582  -1.969  0.04914 *
## high.educBachelor              0.820661   0.755412   1.086  0.27744
## high.educHS Diploma/GED       -0.458995   0.783303  -0.586  0.55796
```

```
## high.educPost Graduate Degree      0.576719   0.768970   0.750  0.45335
## high.educSome College              0.989394   0.723144   1.368  0.17140
## PDS_score:rt_diff_large_neutral_z -0.379842   0.241675  -1.572  0.11617
## ---
## Signif. codes:  0 '***' 0.001 '**' 0.01 '*' 0.05 '.' 0.1 ' ' 1
##
##
## R-sq.(adj) =  0.0263
## lmer.REML =  12662  Scale est. = 16.299    n = 2084
```

```
##                                          stdcoef      stdse
## X(Intercept)                        0.0000000000 0.00000000
## XPDS_score                          0.0485079329 0.02331623
## Xrt_diff_large_neutral_z            0.1101107567 0.05943015
## Xrace.ethnicity.5levelBlack         0.0062385290 0.05556037
## Xrace.ethnicity.5levelMixed         0.1314931660 0.05519823
## Xrace.ethnicity.5levelOther         0.0582528045 0.03841757
## Xrace.ethnicity.5levelWhite         0.1436486215 0.07195667
## Xdemo_race_hispanic1               -0.0380960212 0.02612019
## Xinterview_age                     -0.0070558908 0.02225629
## Xbmi                                0.0613080794 0.02330521
## Xhousehold.income[>=200K]          -0.1390514847 0.05533414
## Xhousehold.income[100K-200K]       -0.1668346400 0.07635457
## Xhousehold.income[12K-16K]         -0.0382898444 0.02951385
## Xhousehold.income[16K-25K]         -0.0002902079 0.03696586
## Xhousehold.income[25K-35K]         -0.0213117728 0.04022284
## Xhousehold.income[35K-50K]         -0.0137049467 0.04529656
## Xhousehold.income[50K-75K]         -0.0912325379 0.05655761
## Xhousehold.income[5K-12K]           0.0315532926 0.03184558
## Xhousehold.income[75K-100K]        -0.1129878112 0.05739577
## Xhigh.educBachelor                  0.0720343374 0.06630706
## Xhigh.educHS Diploma/GED           -0.0227832099 0.03888091
## Xhigh.educPost Graduate Degree      0.0535656836 0.07142193
## Xhigh.educSome College              0.0832635907 0.06085700
## XPDS_score:rt_diff_large_neutral_z -0.0933214338 0.05937587
```

4.13 Model: CBCL internalizing factor ~ PDS x MID reaction time (large vs. small reward)

Female participants

```
##
## Family: gaussian
## Link function: identity
##
## Formula:
## cbcl_scr_syn_internal_r ~ PDS_score * rt_diff_large_small_z +
##     race.ethnicity.5level + demo_race_hispanic + interview_age +
##     bmi + household.income + high.educ
##
## Parametric coefficients:
##                                  Estimate Std. Error t value Pr(>|t|)
## (Intercept)                       6.98481    2.30398   3.032  0.00246 **
```

```
## PDS_score                      0.58229    0.18361   3.171  0.00154 **
## rt_diff_large_small_z         -0.53231    0.30554  -1.742  0.08163 .
## race.ethnicity.5levelBlack     0.07460    0.92575   0.081  0.93578
## race.ethnicity.5levelMixed     1.65014    0.89701   1.840  0.06598 .
## race.ethnicity.5levelOther     2.05364    1.00846   2.036  0.04184 *
## race.ethnicity.5levelWhite     1.42485    0.84282   1.691  0.09108 .
## demo_race_hispanic1            0.12354    0.36711   0.337  0.73652
## interview_age                 -0.03281    0.01618  -2.027  0.04278 *
## bmi                            0.05937    0.03216   1.846  0.06498 .
## household.income[>=200K]      -2.34575    0.87019  -2.696  0.00708 **
## household.income[100K-200K]   -1.80710    0.81608  -2.214  0.02692 *
## household.income[12K-16K]     -0.47048    1.04401  -0.451  0.65229
## household.income[16K-25K]      0.29011    0.91289   0.318  0.75068
## household.income[25K-35K]     -1.02569    0.86370  -1.188  0.23515
## household.income[35K-50K]     -0.96106    0.82356  -1.167  0.24337
## household.income[50K-75K]     -1.13576    0.82601  -1.375  0.16929
## household.income[5K-12K]      -0.37612    0.96780  -0.389  0.69759
## household.income[75K-100K]    -1.33375    0.82479  -1.617  0.10602
## high.educBachelor             -0.25051    0.79829  -0.314  0.75370
## high.educHS Diploma/GED       -0.95139    0.81449  -1.168  0.24292
## high.educPost Graduate Degree -0.14216    0.80484  -0.177  0.85982
## high.educSome College         -0.05981    0.75150  -0.080  0.93657
## PDS_score:rt_diff_large_small_z  0.22484    0.16977   1.324  0.18554
## ---
## Signif. codes:  0 '***' 0.001 '**' 0.01 '*' 0.05 '.' 0.1 ' ' 1
##
##
## R-sq.(adj) =  0.0257
## lmer.REML =  12057  Scale est. = 11.814    n = 1977
```

```
##                                      stdcoef      stdse
## X(Intercept)                     0.000000000 0.00000000
## XPDS_score                       0.078726486 0.02482382
## Xrt_diff_large_small_z          -0.094204960 0.05407299
## Xrace.ethnicity.5levelBlack      0.004546882 0.05642438
## Xrace.ethnicity.5levelMixed      0.102607950 0.05577739
## Xrace.ethnicity.5levelOther      0.084824767 0.04165383
## Xrace.ethnicity.5levelWhite      0.125030140 0.07395720
## Xdemo_race_hispanic1             0.009144216 0.02717321
## Xinterview_age                  -0.046585537 0.02297997
## Xbmi                             0.044524995 0.02411393
## Xhousehold.income[>=200K]       -0.150868200 0.05596657
## Xhousehold.income[100K-200K]    -0.158781628 0.07170558
## Xhousehold.income[12K-16K]      -0.013432196 0.02980614
## Xhousehold.income[16K-25K]       0.010826160 0.03406662
## Xhousehold.income[25K-35K]      -0.044716359 0.03765425
## Xhousehold.income[35K-50K]      -0.052228165 0.04475585
## Xhousehold.income[50K-75K]      -0.072094983 0.05243316
## Xhousehold.income[5K-12K]       -0.012219272 0.03144173
## Xhousehold.income[75K-100K]     -0.091115384 0.05634560
## Xhigh.educBachelor              -0.021330091 0.06797152
## Xhigh.educHS Diploma/GED        -0.044175269 0.03781860
## Xhigh.educPost Graduate Degree  -0.013046242 0.07386292
## Xhigh.educSome College          -0.004877169 0.06127966
```

```
## XPDS_score:rt_diff_large_small_z  0.071931385 0.05431425
```

Male participants

```
##
## Family: gaussian
## Link function: identity
##
## Formula:
## cbcl_scr_syn_internal_r ~ PDS_score * rt_diff_large_small_z +
##     race.ethnicity.5level + demo_race_hispanic + interview_age +
##     bmi + household.income + high.educ
##
## Parametric coefficients:
##                              Estimate Std. Error t value Pr(>|t|)
## (Intercept)                   2.42488    2.21794   1.093  0.27439
## PDS_score                     0.47778    0.23111   2.067  0.03883 *
## rt_diff_large_small_z         0.13667    0.35332   0.387  0.69893
## race.ethnicity.5levelBlack    0.03292    0.88371   0.037  0.97029
## race.ethnicity.5levelMixed    1.96919    0.85372   2.307  0.02118 *
## race.ethnicity.5levelOther    1.39258    1.00112   1.391  0.16437
## race.ethnicity.5levelWhite    1.53034    0.80408   1.903  0.05715 .
## demo_race_hispanic1          -0.50494    0.34452  -1.466  0.14290
## interview_age                -0.00437    0.01523  -0.287  0.77415
## bmi                           0.08877    0.03309   2.682  0.00737 **
## household.income[>=200K]     -2.25580    0.88974  -2.535  0.01131 *
## household.income[100K-200K]  -1.86806    0.83434  -2.239  0.02526 *
## household.income[12K-16K]    -1.39937    1.08426  -1.291  0.19698
## household.income[16K-25K]    -0.03999    0.90578  -0.044  0.96479
## household.income[25K-35K]    -0.54762    0.88374  -0.620  0.53555
## household.income[35K-50K]    -0.29123    0.85869  -0.339  0.73453
## household.income[50K-75K]    -1.37910    0.83070  -1.660  0.09704 .
## household.income[5K-12K]      0.87991    0.97510   0.902  0.36696
## household.income[75K-100K]   -1.69558    0.84549  -2.005  0.04505 *
## high.educBachelor             0.80947    0.75623   1.070  0.28457
## high.educHS Diploma/GED      -0.45401    0.78488  -0.578  0.56303
## high.educPost Graduate Degree  0.57067    0.76977   0.741  0.45856
## high.educSome College         0.96928    0.72294   1.341  0.18015
## PDS_score:rt_diff_large_small_z -0.17460    0.24738  -0.706  0.48039
## ---
## Signif. codes:  0 '***' 0.001 '**' 0.01 '*' 0.05 '.' 0.1 ' ' 1
##
##
## R-sq.(adj) =  0.0247
## lmer.REML =  12665  Scale est. = 16.136    n = 2084
```

```
##                                     stdcoef      stdse
## X(Intercept)                    0.000000000 0.00000000
## XPDS_score                      0.048189456 0.02331029
## Xrt_diff_large_small_z          0.023480229 0.06070028
## Xrace.ethnicity.5levelBlack     0.002070087 0.05556641
## Xrace.ethnicity.5levelMixed     0.127382303 0.05522480
## Xrace.ethnicity.5levelOther     0.053397150 0.03838708
```

```
## Xrace.ethnicity.5levelWhite       0.136968470 0.07196705
## Xdemo_race_hispanic1            -0.038328000 0.02615126
## Xinterview_age                  -0.006389517 0.02226420
## Xbmi                             0.062582093 0.02333104
## Xhousehold.income[>=200K]       -0.140350341 0.05535720
## Xhousehold.income[100K-200K]    -0.171097980 0.07641800
## Xhousehold.income[12K-16K]      -0.038121381 0.02953730
## Xhousehold.income[16K-25K]      -0.001633077 0.03699046
## Xhousehold.income[25K-35K]      -0.024922701 0.04021961
## Xhousehold.income[35K-50K]      -0.015369892 0.04531830
## Xhousehold.income[50K-75K]      -0.093980445 0.05660937
## Xhousehold.income[5K-12K]        0.028753197 0.03186367
## Xhousehold.income[75K-100K]     -0.115227805 0.05745749
## Xhigh.educBachelor               0.071051733 0.06637931
## Xhigh.educHS Diploma/GED        -0.022535805 0.03895931
## Xhigh.educPost Graduate Degree   0.053004239 0.07149642
## Xhigh.educSome College           0.081570827 0.06083994
## XPDS_score:rt_diff_large_small_z -0.042849866 0.06071014
```

4.14 Model: CBCL internalizing factor ~ Testosterone x Accumbens activity (anticipation stage) + PDS

Female participants

```
## Warning: Some predictor variables are on very different scales: consider
## rescaling
```

```
##
## Family: gaussian
## Link function: identity
##
## Formula:
## cbcl_scr_syn_internal_r ~ PDS_score + hormone_sal_end_min_since_midnight +
##     hormone_scr_ert_mean * accumbens_rvsn_ant_z + race.ethnicity.5level +
##     demo_race_hispanic + interview_age + MRI_minus_hormone_date_time +
##     bmi + household.income + high.educ
##
## Parametric coefficients:
##                                          Estimate Std. Error t value
## (Intercept)                             5.279e+00  2.556e+00   2.065
## PDS_score                               5.956e-01  2.049e-01   2.907
## hormone_sal_end_min_since_midnight     -9.280e-04  7.799e-04  -1.190
## hormone_scr_ert_mean                   -3.058e-03  8.509e-03  -0.359
## accumbens_rvsn_ant_z                   -8.694e-03  4.353e-01  -0.020
## race.ethnicity.5levelBlack             -1.827e-01  9.900e-01  -0.185
## race.ethnicity.5levelMixed              1.838e+00  9.455e-01   1.944
## race.ethnicity.5levelOther              1.737e+00  1.068e+00   1.626
## race.ethnicity.5levelWhite              1.427e+00  8.882e-01   1.606
## demo_race_hispanic1                     5.809e-02  3.919e-01   0.148
## interview_age                          -9.907e-03  1.750e-02  -0.566
## MRI_minus_hormone_date_time            -8.526e-06  1.650e-05  -0.517
## bmi                                     5.772e-02  3.493e-02   1.653
## household.income[>=200K]               -2.604e+00  9.644e-01  -2.700
```

```
## household.income[100K-200K]              -2.085e+00  9.092e-01  -2.293
## household.income[12K-16K]                -3.059e-02  1.183e+00  -0.026
## household.income[16K-25K]                -1.599e-01  1.015e+00  -0.158
## household.income[25K-35K]                -1.345e+00  9.604e-01  -1.400
## household.income[35K-50K]                -4.544e-01  9.183e-01  -0.495
## household.income[50K-75K]                -1.252e+00  9.197e-01  -1.361
## household.income[5K-12K]                 -1.912e-01  1.073e+00  -0.178
## household.income[75K-100K]               -1.626e+00  9.202e-01  -1.767
## high.educBachelor                        -3.254e-01  8.576e-01  -0.379
## high.educHS Diploma/GED                  -1.150e+00  8.773e-01  -1.311
## high.educPost Graduate Degree            -7.276e-03  8.659e-01  -0.008
## high.educSome College                    -1.040e-01  8.114e-01  -0.128
## hormone_scr_ert_mean:accumbens_rvsn_ant_z  1.553e-04  1.171e-02   0.013
##                                          Pr(>|t|)
## (Intercept)                               0.03904 *
## PDS_score                                 0.00370 **
## hormone_sal_end_min_since_midnight        0.23421
## hormone_scr_ert_mean                      0.71935
## accumbens_rvsn_ant_z                      0.98407
## race.ethnicity.5levelBlack                0.85363
## race.ethnicity.5levelMixed                0.05209 .
## race.ethnicity.5levelOther                0.10408
## race.ethnicity.5levelWhite                0.10844
## demo_race_hispanic1                       0.88218
## interview_age                             0.57136
## MRI_minus_hormone_date_time               0.60542
## bmi                                       0.09861 .
## household.income[>=200K]                  0.00701 **
## household.income[100K-200K]               0.02198 *
## household.income[12K-16K]                 0.97938
## household.income[16K-25K]                 0.87476
## household.income[25K-35K]                 0.16168
## household.income[35K-50K]                 0.62081
## household.income[50K-75K]                 0.17369
## household.income[5K-12K]                  0.85854
## household.income[75K-100K]                0.07733 .
## high.educBachelor                         0.70445
## high.educHS Diploma/GED                   0.19001
## high.educPost Graduate Degree             0.99330
## high.educSome College                     0.89803
## hormone_scr_ert_mean:accumbens_rvsn_ant_z  0.98943
## ---
## Signif. codes:  0 '***' 0.001 '**' 0.01 '*' 0.05 '.' 0.1 ' ' 1
##
##
## R-sq.(adj) =  0.0256
## lmer.REML =  10430  Scale est. = 10.943    n = 1703
```

```
##                                                  stdcoef      stdse
## X(Intercept)                                0.0000000000 0.00000000
## XPDS_score                                  0.0795657254 0.02737435
## Xhormone_sal_end_min_since_midnight        -0.0309274285 0.02598900
## Xhormone_scr_ert_mean                      -0.0092395076 0.02570930
## Xaccumbens_rvsn_ant_z                      -0.0011138135 0.05575992
```

```
## Xrace.ethnicity.5levelBlack              -0.0105121531 0.05697014
## Xrace.ethnicity.5levelMixed               0.1158372655 0.05959366
## Xrace.ethnicity.5levelOther               0.0726789802 0.04469054
## Xrace.ethnicity.5levelWhite               0.1242001259 0.07732940
## Xdemo_race_hispanic1                      0.0043302857 0.02921353
## Xinterview_age                           -0.0140496778 0.02481552
## XMRI_minus_hormone_date_time             -0.0129266927 0.02501683
## Xbmi                                      0.0432683037 0.02618305
## Xhousehold.income[>=200K]                -0.1660259048 0.06149896
## Xhousehold.income[100K-200K]             -0.1832932845 0.07994119
## Xhousehold.income[12K-16K]               -0.0008317845 0.03218066
## Xhousehold.income[16K-25K]               -0.0059192136 0.03755048
## Xhousehold.income[25K-35K]               -0.0589113049 0.04207750
## Xhousehold.income[35K-50K]               -0.0247047231 0.04992915
## Xhousehold.income[50K-75K]               -0.0792058549 0.05819642
## Xhousehold.income[5K-12K]                -0.0062180586 0.03488171
## Xhousehold.income[75K-100K]              -0.1119545254 0.06334202
## Xhigh.educBachelor                       -0.0278419470 0.07338639
## Xhigh.educHS Diploma/GED                 -0.0543376644 0.04144529
## Xhigh.educPost Graduate Degree           -0.0006673866 0.07942609
## Xhigh.educSome College                   -0.0083270445 0.06496818
## Xhormone_scr_ert_mean:accumbens_rvsn_ant_z  0.0007375426 0.05564323
```

Male participants

```
## Warning: Some predictor variables are on very different scales: consider
## rescaling


##
## Family: gaussian
## Link function: identity
##
## Formula:
## cbcl_scr_syn_internal_r ~ PDS_score + hormone_sal_end_min_since_midnight +
##     hormone_scr_ert_mean * accumbens_rvsn_ant_z + race.ethnicity.5level +
##     demo_race_hispanic + interview_age + MRI_minus_hormone_date_time +
##     bmi + household.income + high.educ
##
## Parametric coefficients:
##                                           Estimate Std. Error t value
## (Intercept)                              2.246e+00  2.550e+00   0.881
## PDS_score                                7.003e-01  2.610e-01   2.683
## hormone_sal_end_min_since_midnight       3.284e-04  7.587e-04   0.433
## hormone_scr_ert_mean                    -4.493e-03  8.505e-03  -0.528
## accumbens_rvsn_ant_z                    -9.117e-02  3.713e-01  -0.246
## race.ethnicity.5levelBlack               2.937e-02  9.778e-01   0.030
## race.ethnicity.5levelMixed               2.288e+00  9.397e-01   2.434
## race.ethnicity.5levelOther               1.824e+00  1.093e+00   1.669
## race.ethnicity.5levelWhite               1.718e+00  8.833e-01   1.945
## demo_race_hispanic1                     -4.716e-01  3.758e-01  -1.255
## interview_age                           -4.040e-03  1.686e-02  -0.240
## MRI_minus_hormone_date_time             -6.926e-06  1.443e-05  -0.480
## bmi                                      5.328e-02  3.714e-02   1.434
```

```
## household.income[>=200K]                 -1.351e+00  1.021e+00  -1.324
## household.income[100K-200K]              -8.882e-01  9.669e-01  -0.919
## household.income[12K-16K]                -2.975e-01  1.256e+00  -0.237
## household.income[16K-25K]                 1.169e+00  1.052e+00   1.112
## household.income[25K-35K]                 1.889e-01  1.024e+00   0.184
## household.income[35K-50K]                 6.472e-01  9.916e-01   0.653
## household.income[50K-75K]                -1.936e-01  9.652e-01  -0.201
## household.income[5K-12K]                  1.685e+00  1.103e+00   1.527
## household.income[75K-100K]               -6.211e-01  9.783e-01  -0.635
## high.educBachelor                         4.695e-02  8.519e-01   0.055
## high.educHS Diploma/GED                  -1.254e+00  8.740e-01  -1.435
## high.educPost Graduate Degree            -1.885e-01  8.624e-01  -0.219
## high.educSome College                     2.170e-01  8.139e-01   0.267
## hormone_scr_ert_mean:accumbens_rvsn_ant_z  5.962e-05  1.098e-02   0.005
##                                          Pr(>|t|)
## (Intercept)                               0.37864
## PDS_score                                 0.00737 **
## hormone_sal_end_min_since_midnight        0.66518
## hormone_scr_ert_mean                      0.59739
## accumbens_rvsn_ant_z                      0.80608
## race.ethnicity.5levelBlack                0.97604
## race.ethnicity.5levelMixed                0.01502 *
## race.ethnicity.5levelOther                0.09533 .
## race.ethnicity.5levelWhite                0.05197 .
## demo_race_hispanic1                       0.20968
## interview_age                             0.81069
## MRI_minus_hormone_date_time               0.63124
## bmi                                       0.15163
## household.income[>=200K]                  0.18583
## household.income[100K-200K]               0.35847
## household.income[12K-16K]                 0.81283
## household.income[16K-25K]                 0.26648
## household.income[25K-35K]                 0.85366
## household.income[35K-50K]                 0.51403
## household.income[50K-75K]                 0.84105
## household.income[5K-12K]                  0.12694
## household.income[75K-100K]                0.52561
## high.educBachelor                         0.95606
## high.educHS Diploma/GED                   0.15149
## high.educPost Graduate Degree             0.82704
## high.educSome College                     0.78980
## hormone_scr_ert_mean:accumbens_rvsn_ant_z  0.99567
## ---
## Signif. codes:  0 '***' 0.001 '**' 0.01 '*' 0.05 '.' 0.1 ' ' 1
##
##
## R-sq.(adj) =  0.024
## lmer.REML =  10431  Scale est. = 16.612    n = 1712
```

```
##                                                stdcoef      stdse
## X(Intercept)                              0.0000000000 0.00000000
## XPDS_score                                0.0703419378 0.02621760
## Xhormone_sal_end_min_since_midnight       0.0112561137 0.02600450
## Xhormone_scr_ert_mean                    -0.0134187569 0.02540190
```

```
## Xaccumbens_rvsn_ant_z                      -0.0129462957 0.05272856
## Xrace.ethnicity.5levelBlack                 0.0017959361 0.05978573
## Xrace.ethnicity.5levelMixed                 0.1466794510 0.06025560
## Xrace.ethnicity.5levelOther                 0.0712846068 0.04271414
## Xrace.ethnicity.5levelWhite                 0.1528386514 0.07859015
## Xdemo_race_hispanic1                       -0.0365007435 0.02908570
## Xinterview_age                             -0.0059306827 0.02475477
## XMRI_minus_hormone_date_time               -0.0119407136 0.02487253
## Xbmi                                        0.0374703508 0.02612180
## Xhousehold.income[>=200K]                  -0.0861012484 0.06505336
## Xhousehold.income[100K-200K]               -0.0818275079 0.08908445
## Xhousehold.income[12K-16K]                 -0.0078441339 0.03312427
## Xhousehold.income[16K-25K]                  0.0462818916 0.04163636
## Xhousehold.income[25K-35K]                  0.0084996614 0.04607488
## Xhousehold.income[35K-50K]                  0.0345238418 0.05289293
## Xhousehold.income[50K-75K]                 -0.0129689983 0.06465630
## Xhousehold.income[5K-12K]                   0.0562898958 0.03686180
## Xhousehold.income[75K-100K]                -0.0431847002 0.06802290
## Xhigh.educBachelor                          0.0041024861 0.07443982
## Xhigh.educHS Diploma/GED                   -0.0635129574 0.04426195
## Xhigh.educPost Graduate Degree             -0.0177148450 0.08106423
## Xhigh.educSome College                      0.0181901322 0.06822749
## Xhormone_scr_ert_mean:accumbens_rvsn_ant_z  0.0002857374 0.05261576
```

4.15 Model: CBCL internalizing factor ~ Testosterone x Caudate activity (anticipation stage) + PDS

Female participants

```
## Warning: Some predictor variables are on very different scales: consider
## rescaling
```

```
##
## Family: gaussian
## Link function: identity
##
## Formula:
## cbcl_scr_syn_internal_r ~ PDS_score + hormone_sal_end_min_since_midnight +
##     hormone_scr_ert_mean * caudate_rvsn_ant_z + race.ethnicity.5level +
##     demo_race_hispanic + interview_age + MRI_minus_hormone_date_time +
##     bmi + household.income + high.educ
##
## Parametric coefficients:
##                                     Estimate Std. Error t value Pr(>|t|)
## (Intercept)                        5.159e+00  2.556e+00   2.018  0.04372
## PDS_score                          6.056e-01  2.048e-01   2.956  0.00316
## hormone_sal_end_min_since_midnight -9.235e-04  7.788e-04  -1.186  0.23584
## hormone_scr_ert_mean              -3.121e-03  8.517e-03  -0.366  0.71410
## caudate_rvsn_ant_z                 1.692e-03  3.383e-01   0.005  0.99601
## race.ethnicity.5levelBlack        -1.365e-01  9.902e-01  -0.138  0.89041
## race.ethnicity.5levelMixed         1.805e+00  9.441e-01   1.912  0.05603
## race.ethnicity.5levelOther         1.719e+00  1.064e+00   1.616  0.10631
## race.ethnicity.5levelWhite         1.418e+00  8.876e-01   1.598  0.11022
```

```
## demo_race_hispanic1                      7.176e-02  3.904e-01   0.184  0.85419
## interview_age                           -1.092e-02  1.753e-02  -0.623  0.53353
## MRI_minus_hormone_date_time             -8.086e-06  1.610e-05  -0.502  0.61566
## bmi                                      5.899e-02  3.493e-02   1.689  0.09146
## household.income[>=200K]                -2.451e+00  9.602e-01  -2.552  0.01079
## household.income[100K-200K]             -1.923e+00  9.036e-01  -2.128  0.03349
## household.income[12K-16K]                9.423e-02  1.170e+00   0.081  0.93580
## household.income[16K-25K]               -8.698e-02  1.007e+00  -0.086  0.93118
## household.income[25K-35K]               -1.217e+00  9.556e-01  -1.274  0.20297
## household.income[35K-50K]               -3.272e-01  9.125e-01  -0.359  0.71994
## household.income[50K-75K]               -1.083e+00  9.143e-01  -1.185  0.23620
## household.income[5K-12K]                -8.135e-02  1.066e+00  -0.076  0.93920
## household.income[75K-100K]              -1.486e+00  9.153e-01  -1.623  0.10468
## high.educBachelor                       -2.666e-01  8.553e-01  -0.312  0.75528
## high.educHS Diploma/GED                 -1.074e+00  8.734e-01  -1.230  0.21880
## high.educPost Graduate Degree            5.179e-02  8.638e-01   0.060  0.95219
## high.educSome College                   -5.037e-02  8.082e-01  -0.062  0.95032
## hormone_scr_ert_mean:caudate_rvsn_ant_z  2.527e-03  9.086e-03   0.278  0.78098
##
## (Intercept)                             *
## PDS_score                               **
## hormone_sal_end_min_since_midnight
## hormone_scr_ert_mean
## caudate_rvsn_ant_z
## race.ethnicity.5levelBlack
## race.ethnicity.5levelMixed              .
## race.ethnicity.5levelOther
## race.ethnicity.5levelWhite
## demo_race_hispanic1
## interview_age
## MRI_minus_hormone_date_time
## bmi                                     .
## household.income[>=200K]                *
## household.income[100K-200K]             *
## household.income[12K-16K]
## household.income[16K-25K]
## household.income[25K-35K]
## household.income[35K-50K]
## household.income[50K-75K]
## household.income[5K-12K]
## household.income[75K-100K]
## high.educBachelor
## high.educHS Diploma/GED
## high.educPost Graduate Degree
## high.educSome College
## hormone_scr_ert_mean:caudate_rvsn_ant_z
## ---
## Signif. codes:  0 '***' 0.001 '**' 0.01 '*' 0.05 '.' 0.1 ' ' 1
##
##
## R-sq.(adj) =  0.0251
## lmer.REML =  10426  Scale est. = 11.089    n = 1702

##                                                stdcoef      stdse
```

```
## X(Intercept)                                 0.0000000000 0.00000000
## XPDS_score                                   0.0810109784 0.02740280
## Xhormone_sal_end_min_since_midnight         -0.0308411992 0.02600695
## Xhormone_scr_ert_mean                       -0.0094397354 0.02576217
## Xcaudate_rvsn_ant_z                          0.0002872754 0.05741463
## Xrace.ethnicity.5levelBlack                 -0.0078186645 0.05673688
## Xrace.ethnicity.5levelMixed                  0.1143225609 0.05978908
## Xrace.ethnicity.5levelOther                  0.0723733912 0.04478884
## Xrace.ethnicity.5levelWhite                  0.1236386817 0.07736937
## Xdemo_race_hispanic1                         0.0053531437 0.02912485
## Xinterview_age                              -0.0154766884 0.02485204
## XMRI_minus_hormone_date_time                -0.0125545581 0.02500417
## Xbmi                                         0.0442039138 0.02617579
## Xhousehold.income[>=200K]                   -0.1558190767 0.06104782
## Xhousehold.income[100K-200K]                -0.1693376059 0.07958080
## Xhousehold.income[12K-16K]                   0.0025991969 0.03226402
## Xhousehold.income[16K-25K]                  -0.0032447322 0.03756555
## Xhousehold.income[25K-35K]                  -0.0531057569 0.04169606
## Xhousehold.income[35K-50K]                  -0.0178065567 0.04965543
## Xhousehold.income[50K-75K]                  -0.0684821245 0.05779244
## Xhousehold.income[5K-12K]                   -0.0026478926 0.03471245
## Xhousehold.income[75K-100K]                 -0.1020551629 0.06286261
## Xhigh.educBachelor                          -0.0228334844 0.07324478
## Xhigh.educHS Diploma/GED                    -0.0508000220 0.04129472
## Xhigh.educPost Graduate Degree               0.0047481279 0.07918567
## Xhigh.educSome College                      -0.0040426815 0.06487278
## Xhormone_scr_ert_mean:caudate_rvsn_ant_z     0.0159581083 0.05738635
```

Male participants

```
## Warning: Some predictor variables are on very different scales: consider
## rescaling
```

```
##
## Family: gaussian
## Link function: identity
##
## Formula:
## cbcl_scr_syn_internal_r ~ PDS_score + hormone_sal_end_min_since_midnight +
##     hormone_scr_ert_mean * caudate_rvsn_ant_z + race.ethnicity.5level +
##     demo_race_hispanic + interview_age + MRI_minus_hormone_date_time +
##     bmi + household.income + high.educ
##
## Parametric coefficients:
##                                             Estimate Std. Error t value Pr(>|t|)
## (Intercept)                                2.407e+00  2.552e+00   0.943  0.34582
## PDS_score                                  7.188e-01  2.613e-01   2.751  0.00601
## hormone_sal_end_min_since_midnight         3.807e-04  7.589e-04   0.502  0.61592
## hormone_scr_ert_mean                      -4.521e-03  8.572e-03  -0.527  0.59799
## caudate_rvsn_ant_z                         3.459e-01  2.944e-01   1.175  0.24016
## race.ethnicity.5levelBlack                -5.380e-02  9.885e-01  -0.054  0.95661
## race.ethnicity.5levelMixed                 2.238e+00  9.524e-01   2.350  0.01887
## race.ethnicity.5levelOther                 1.745e+00  1.103e+00   1.582  0.11390
```

```
## race.ethnicity.5levelWhite              1.640e+00  8.962e-01   1.830  0.06746
## demo_race_hispanic1                    -4.736e-01  3.772e-01  -1.256  0.20946
## interview_age                          -5.929e-03  1.692e-02  -0.350  0.72601
## MRI_minus_hormone_date_time            -1.075e-05  1.473e-05  -0.730  0.46568
## bmi                                     5.195e-02  3.719e-02   1.397  0.16268
## household.income[>=200K]               -1.193e+00  1.016e+00  -1.174  0.24064
## household.income[100K-200K]            -7.736e-01  9.611e-01  -0.805  0.42103
## household.income[12K-16K]              -2.843e-01  1.253e+00  -0.227  0.82052
## household.income[16K-25K]               1.208e+00  1.044e+00   1.157  0.24743
## household.income[25K-35K]               3.991e-01  1.019e+00   0.392  0.69544
## household.income[35K-50K]               7.752e-01  9.871e-01   0.785  0.43237
## household.income[50K-75K]              -9.477e-02  9.598e-01  -0.099  0.92136
## household.income[5K-12K]                1.905e+00  1.093e+00   1.743  0.08147
## household.income[75K-100K]             -4.669e-01  9.730e-01  -0.480  0.63139
## high.educBachelor                       8.953e-02  8.491e-01   0.105  0.91604
## high.educHS Diploma/GED                -1.213e+00  8.724e-01  -1.390  0.16456
## high.educPost Graduate Degree          -2.224e-01  8.596e-01  -0.259  0.79583
## high.educSome College                   2.018e-01  8.098e-01   0.249  0.80329
## hormone_scr_ert_mean:caudate_rvsn_ant_z -1.150e-02  8.246e-03  -1.395  0.16316
##
## (Intercept)
## PDS_score                              **
## hormone_sal_end_min_since_midnight
## hormone_scr_ert_mean
## caudate_rvsn_ant_z
## race.ethnicity.5levelBlack
## race.ethnicity.5levelMixed             *
## race.ethnicity.5levelOther
## race.ethnicity.5levelWhite             .
## demo_race_hispanic1
## interview_age
## MRI_minus_hormone_date_time
## bmi
## household.income[>=200K]
## household.income[100K-200K]
## household.income[12K-16K]
## household.income[16K-25K]
## household.income[25K-35K]
## household.income[35K-50K]
## household.income[50K-75K]
## household.income[5K-12K]               .
## household.income[75K-100K]
## high.educBachelor
## high.educHS Diploma/GED
## high.educPost Graduate Degree
## high.educSome College
## hormone_scr_ert_mean:caudate_rvsn_ant_z
## ---
## Signif. codes:  0 '***' 0.001 '**' 0.01 '*' 0.05 '.' 0.1 ' ' 1
##
##
## R-sq.(adj) =  0.0251
## lmer.REML =  10425  Scale est. = 16.74     n = 1710
```

```
##                                           stdcoef      stdse
## X(Intercept)                          0.000000000 0.00000000
## XPDS_score                            0.072036081 0.02618950
## Xhormone_sal_end_min_since_midnight   0.013041637 0.02599327
## Xhormone_scr_ert_mean                -0.013417586 0.02544112
## Xcaudate_rvsn_ant_z                   0.063135976 0.05373314
## Xrace.ethnicity.5levelBlack          -0.003284597 0.06035527
## Xrace.ethnicity.5levelMixed           0.143026535 0.06085470
## Xrace.ethnicity.5levelOther           0.068089484 0.04304763
## Xrace.ethnicity.5levelWhite           0.145510841 0.07952427
## Xdemo_race_hispanic1                 -0.036481944 0.02905667
## Xinterview_age                       -0.008688864 0.02479032
## XMRI_minus_hormone_date_time         -0.018173685 0.02490607
## Xbmi                                  0.036490402 0.02612539
## Xhousehold.income[>=200K]            -0.075430502 0.06426170
## Xhousehold.income[100K-200K]         -0.071121199 0.08836772
## Xhousehold.income[12K-16K]           -0.007484246 0.03298323
## Xhousehold.income[16K-25K]            0.048074654 0.04155096
## Xhousehold.income[25K-35K]            0.018022628 0.04602829
## Xhousehold.income[35K-50K]            0.041159213 0.05241015
## Xhousehold.income[50K-75K]           -0.006339421 0.06420209
## Xhousehold.income[5K-12K]             0.064134129 0.03678992
## Xhousehold.income[75K-100K]          -0.032365388 0.06744837
## Xhigh.educBachelor                    0.007811159 0.07407878
## Xhigh.educHS Diploma/GED             -0.060889749 0.04379007
## Xhigh.educPost Graduate Degree       -0.020840536 0.08053068
## Xhigh.educSome College                0.016939731 0.06799665
## Xhormone_scr_ert_mean:caudate_rvsn_ant_z -0.074961849 0.05373090
```

4.16 Model: CBCL internalizing factor ~ Testosterone x Putamen activity (anticipation stage) + PDS

Female participants

```
## Warning: Some predictor variables are on very different scales: consider
## rescaling
```

```
##
## Family: gaussian
## Link function: identity
##
## Formula:
## cbcl_scr_syn_internal_r ~ PDS_score + hormone_sal_end_min_since_midnight +
##     hormone_scr_ert_mean * putamen_rvsn_ant_z + race.ethnicity.5level +
##     demo_race_hispanic + interview_age + MRI_minus_hormone_date_time +
##     bmi + household.income + high.educ
##
## Parametric coefficients:
##                                       Estimate Std. Error t value Pr(>|t|)
## (Intercept)                          4.932e+00  2.545e+00   1.938  0.05279
## PDS_score                            6.166e-01  2.048e-01   3.010  0.00265
## hormone_sal_end_min_since_midnight  -8.270e-04  7.747e-04  -1.068  0.28586
## hormone_scr_ert_mean                -4.339e-03  8.477e-03  -0.512  0.60881
```

```
## putamen_rvsn_ant_z                        -1.880e-01  3.407e-01  -0.552  0.58112
## race.ethnicity.5levelBlack                -1.232e-01  9.857e-01  -0.125  0.90054
## race.ethnicity.5levelMixed                 1.862e+00  9.400e-01   1.980  0.04781
## race.ethnicity.5levelOther                 1.760e+00  1.063e+00   1.656  0.09794
## race.ethnicity.5levelWhite                 1.421e+00  8.835e-01   1.609  0.10787
## demo_race_hispanic1                        9.851e-02  3.896e-01   0.253  0.80041
## interview_age                             -9.099e-03  1.747e-02  -0.521  0.60258
## MRI_minus_hormone_date_time               -6.753e-06  1.632e-05  -0.414  0.67905
## bmi                                        5.753e-02  3.482e-02   1.652  0.09867
## household.income[>=200K]                  -2.448e+00  9.575e-01  -2.557  0.01065
## household.income[100K-200K]               -1.944e+00  9.006e-01  -2.159  0.03100
## household.income[12K-16K]                  7.050e-02  1.174e+00   0.060  0.95213
## household.income[16K-25K]                 -1.732e-01  1.006e+00  -0.172  0.86337
## household.income[25K-35K]                 -1.267e+00  9.520e-01  -1.330  0.18354
## household.income[35K-50K]                 -3.538e-01  9.090e-01  -0.389  0.69720
## household.income[50K-75K]                 -1.063e+00  9.123e-01  -1.165  0.24431
## household.income[5K-12K]                  -9.828e-02  1.064e+00  -0.092  0.92643
## household.income[75K-100K]               -1.616e+00  9.131e-01  -1.770  0.07686
## high.educBachelor                         -2.591e-01  8.495e-01  -0.305  0.76041
## high.educHS Diploma/GED                   -1.087e+00  8.677e-01  -1.253  0.21038
## high.educPost Graduate Degree              2.023e-02  8.583e-01   0.024  0.98120
## high.educSome College                     -6.760e-02  8.029e-01  -0.084  0.93291
## hormone_scr_ert_mean:putamen_rvsn_ant_z  8.131e-03  9.010e-03   0.902  0.36694
##
## (Intercept)                               .
## PDS_score                                 **
## hormone_sal_end_min_since_midnight
## hormone_scr_ert_mean
## putamen_rvsn_ant_z
## race.ethnicity.5levelBlack
## race.ethnicity.5levelMixed                *
## race.ethnicity.5levelOther                .
## race.ethnicity.5levelWhite
## demo_race_hispanic1
## interview_age
## MRI_minus_hormone_date_time
## bmi                                       .
## household.income[>=200K]                  *
## household.income[100K-200K]               *
## household.income[12K-16K]
## household.income[16K-25K]
## household.income[25K-35K]
## household.income[35K-50K]
## household.income[50K-75K]
## household.income[5K-12K]
## household.income[75K-100K]                .
## high.educBachelor
## high.educHS Diploma/GED
## high.educPost Graduate Degree
## high.educSome College
## hormone_scr_ert_mean:putamen_rvsn_ant_z
## ---
## Signif. codes:  0 '***' 0.001 '**' 0.01 '*' 0.05 '.' 0.1 ' ' 1
##
```

```
##
## R-sq.(adj) =  0.0253
## lmer.REML =  10394  Scale est. = 11.009    n = 1699
```

```
##                                               stdcoef      stdse
## X(Intercept)                              0.000000000 0.00000000
## XPDS_score                                0.082779690 0.02749747
## Xhormone_sal_end_min_since_midnight      -0.027753321 0.02599609
## Xhormone_scr_ert_mean                    -0.013189100 0.02576636
## Xputamen_rvsn_ant_z                      -0.030832573 0.05587102
## Xrace.ethnicity.5levelBlack              -0.007093704 0.05675269
## Xrace.ethnicity.5levelMixed               0.118233807 0.05969967
## Xrace.ethnicity.5levelOther               0.074058991 0.04472511
## Xrace.ethnicity.5levelWhite               0.124313406 0.07727610
## Xdemo_race_hispanic1                      0.007383816 0.02920144
## Xinterview_age                           -0.012962625 0.02489004
## XMRI_minus_hormone_date_time             -0.010348424 0.02500622
## Xbmi                                      0.043344826 0.02623349
## Xhousehold.income[>=200K]                -0.156696560 0.06128123
## Xhousehold.income[100K-200K]             -0.172082218 0.07970849
## Xhousehold.income[12K-16K]                0.001927953 0.03211343
## Xhousehold.income[16K-25K]               -0.006447425 0.03745926
## Xhousehold.income[25K-35K]               -0.055537299 0.04174203
## Xhousehold.income[35K-50K]               -0.019286982 0.04955849
## Xhousehold.income[50K-75K]               -0.067350414 0.05782687
## Xhousehold.income[5K-12K]                -0.003214587 0.03480655
## Xhousehold.income[75K-100K]              -0.111372619 0.06291162
## Xhigh.educBachelor                       -0.022301265 0.07312096
## Xhigh.educHS Diploma/GED                 -0.051657420 0.04122688
## Xhigh.educPost Graduate Degree            0.001863550 0.07905498
## Xhigh.educSome College                   -0.005427194 0.06446074
## Xhormone_scr_ert_mean:putamen_rvsn_ant_z  0.050167016 0.05558893
```

Male participants

```
## Warning: Some predictor variables are on very different scales: consider
## rescaling
```

```
##
## Family: gaussian
## Link function: identity
##
## Formula:
## cbcl_scr_syn_internal_r ~ PDS_score + hormone_sal_end_min_since_midnight +
##     hormone_scr_ert_mean * putamen_rvsn_ant_z + race.ethnicity.5level +
##     demo_race_hispanic + interview_age + MRI_minus_hormone_date_time +
##     bmi + household.income + high.educ
##
## Parametric coefficients:
##                                           Estimate Std. Error t value Pr(>|t|)
## (Intercept)                              2.224e+00  2.556e+00   0.870  0.38441
## PDS_score                                7.447e-01  2.620e-01   2.842  0.00453
## hormone_sal_end_min_since_midnight       5.247e-04  7.571e-04   0.693  0.48843
```

```
## hormone_scr_ert_mean                    -4.803e-03  8.562e-03  -0.561  0.57493
## putamen_rvsn_ant_z                       4.834e-01  2.907e-01   1.663  0.09648
## race.ethnicity.5levelBlack              -2.945e-02  9.875e-01  -0.030  0.97621
## race.ethnicity.5levelMixed               2.262e+00  9.486e-01   2.384  0.01721
## race.ethnicity.5levelOther               1.720e+00  1.102e+00   1.560  0.11885
## race.ethnicity.5levelWhite               1.635e+00  8.947e-01   1.828  0.06778
## demo_race_hispanic1                     -5.141e-01  3.758e-01  -1.368  0.17152
## interview_age                           -5.179e-03  1.689e-02  -0.307  0.75915
## MRI_minus_hormone_date_time             -1.041e-05  1.432e-05  -0.727  0.46735
## bmi                                      5.644e-02  3.737e-02   1.510  0.13112
## household.income[>=200K]                -1.203e+00  1.021e+00  -1.179  0.23862
## household.income[100K-200K]             -8.041e-01  9.673e-01  -0.831  0.40595
## household.income[12K-16K]               -3.477e-01  1.255e+00  -0.277  0.78183
## household.income[16K-25K]                1.174e+00  1.050e+00   1.118  0.26354
## household.income[25K-35K]                3.950e-01  1.023e+00   0.386  0.69952
## household.income[35K-50K]                7.941e-01  9.929e-01   0.800  0.42395
## household.income[50K-75K]               -6.817e-02  9.663e-01  -0.071  0.94377
## household.income[5K-12K]                 1.811e+00  1.098e+00   1.649  0.09927
## household.income[75K-100K]              -5.047e-01  9.788e-01  -0.516  0.60622
## high.educBachelor                       -2.820e-02  8.521e-01  -0.033  0.97361
## high.educHS Diploma/GED                 -1.332e+00  8.768e-01  -1.519  0.12897
## high.educPost Graduate Degree           -2.814e-01  8.626e-01  -0.326  0.74432
## high.educSome College                    1.050e-01  8.138e-01   0.129  0.89738
## hormone_scr_ert_mean:putamen_rvsn_ant_z -1.706e-02  8.076e-03  -2.112  0.03481
##
## (Intercept)
## PDS_score                               **
## hormone_sal_end_min_since_midnight
## hormone_scr_ert_mean
## putamen_rvsn_ant_z                      .
## race.ethnicity.5levelBlack
## race.ethnicity.5levelMixed              *
## race.ethnicity.5levelOther
## race.ethnicity.5levelWhite              .
## demo_race_hispanic1
## interview_age
## MRI_minus_hormone_date_time
## bmi
## household.income[>=200K]
## household.income[100K-200K]
## household.income[12K-16K]
## household.income[16K-25K]
## household.income[25K-35K]
## household.income[35K-50K]
## household.income[50K-75K]
## household.income[5K-12K]                .
## household.income[75K-100K]
## high.educBachelor
## high.educHS Diploma/GED
## high.educPost Graduate Degree
## high.educSome College
## hormone_scr_ert_mean:putamen_rvsn_ant_z *
## ---
## Signif. codes:  0 '***' 0.001 '**' 0.01 '*' 0.05 '.' 0.1 ' ' 1
```

```
##
##
## R-sq.(adj) =  0.0271
## lmer.REML =  10429  Scale est. = 16.145    n = 1712
```

```
##                                              stdcoef      stdse
## X(Intercept)                              0.000000000 0.00000000
## XPDS_score                                0.074118738 0.02607661
## Xhormone_sal_end_min_since_midnight       0.017979517 0.02594577
## Xhormone_scr_ert_mean                    -0.014237346 0.02538230
## Xputamen_rvsn_ant_z                       0.087333771 0.05251296
## Xrace.ethnicity.5levelBlack              -0.001790197 0.06003164
## Xrace.ethnicity.5levelMixed               0.146025267 0.06124052
## Xrace.ethnicity.5levelOther               0.067123932 0.04301664
## Xrace.ethnicity.5levelWhite               0.145405755 0.07955867
## Xdemo_race_hispanic1                     -0.039607444 0.02895468
## Xinterview_age                           -0.007592938 0.02476113
## XMRI_minus_hormone_date_time             -0.018078032 0.02486770
## Xbmi                                      0.039396643 0.02608301
## Xhousehold.income[>=200K]                -0.076734752 0.06509257
## Xhousehold.income[100K-200K]             -0.073997633 0.08901930
## Xhousehold.income[12K-16K]               -0.009155976 0.03305676
## Xhousehold.income[16K-25K]                0.046711583 0.04176536
## Xhousehold.income[25K-35K]                0.017843493 0.04622193
## Xhousehold.income[35K-50K]                0.042033182 0.05255528
## Xhousehold.income[50K-75K]               -0.004553134 0.06454056
## Xhousehold.income[5K-12K]                 0.060995307 0.03698278
## Xhousehold.income[75K-100K]              -0.034992461 0.06787067
## Xhigh.educBachelor                       -0.002462678 0.07442012
## Xhigh.educHS Diploma/GED                 -0.066872502 0.04402606
## Xhigh.educPost Graduate Degree           -0.026419065 0.08099191
## Xhigh.educSome College                    0.008796876 0.06819589
## Xhormone_scr_ert_mean:putamen_rvsn_ant_z -0.111273652 0.05268080
```

4.17 Model: CBCL internalizing factor ~ Testosterone x Accumbens activity (feedback stage) + PDS

Female participants

```
## Warning: Some predictor variables are on very different scales: consider
## rescaling
```

```
##
## Family: gaussian
## Link function: identity
##
## Formula:
## cbcl_scr_syn_internal_r ~ PDS_score + hormone_sal_end_min_since_midnight +
##     hormone_scr_ert_mean * accumbens_posvsneg_feedback_z + race.ethnicity.5level +
##     demo_race_hispanic + interview_age + MRI_minus_hormone_date_time +
##     bmi + household.income + high.educ
##
## Parametric coefficients:
```

```
##                                                  Estimate Std. Error
## (Intercept)                                     4.994e+00  2.546e+00
## PDS_score                                       5.976e-01  2.045e-01
## hormone_sal_end_min_since_midnight             -9.425e-04  7.782e-04
## hormone_scr_ert_mean                           -1.890e-03  8.499e-03
## accumbens_posvsneg_feedback_z                  -1.059e-01  4.934e-01
## race.ethnicity.5levelBlack                     -1.750e-01  9.889e-01
## race.ethnicity.5levelMixed                      1.799e+00  9.435e-01
## race.ethnicity.5levelOther                      1.716e+00  1.063e+00
## race.ethnicity.5levelWhite                      1.426e+00  8.875e-01
## demo_race_hispanic1                             6.992e-02  3.914e-01
## interview_age                                  -1.016e-02  1.749e-02
## MRI_minus_hormone_date_time                    -1.032e-05  1.617e-05
## bmi                                             5.750e-02  3.491e-02
## household.income[>=200K]                       -2.467e+00  9.552e-01
## household.income[100K-200K]                    -1.885e+00  8.972e-01
## household.income[12K-16K]                       1.893e-01  1.164e+00
## household.income[16K-25K]                      -1.957e-02  1.001e+00
## household.income[25K-35K]                      -1.174e+00  9.482e-01
## household.income[35K-50K]                      -2.583e-01  9.050e-01
## household.income[50K-75K]                      -1.115e+00  9.076e-01
## household.income[5K-12K]                        8.564e-03  1.060e+00
## household.income[75K-100K]                     -1.461e+00  9.083e-01
## high.educBachelor                              -2.091e-01  8.512e-01
## high.educHS Diploma/GED                        -1.000e+00  8.664e-01
## high.educPost Graduate Degree                   1.044e-01  8.597e-01
## high.educSome College                          -2.553e-02  8.052e-01
## hormone_scr_ert_mean:accumbens_posvsneg_feedback_z  3.276e-03  1.306e-02
##                                                t value Pr(>|t|)
## (Intercept)                                      1.962  0.04997 *
## PDS_score                                        2.922  0.00352 **
## hormone_sal_end_min_since_midnight              -1.211  0.22603
## hormone_scr_ert_mean                            -0.222  0.82407
## accumbens_posvsneg_feedback_z                   -0.215  0.83009
## race.ethnicity.5levelBlack                      -0.177  0.85958
## race.ethnicity.5levelMixed                       1.907  0.05675 .
## race.ethnicity.5levelOther                       1.614  0.10673
## race.ethnicity.5levelWhite                       1.607  0.10820
## demo_race_hispanic1                              0.179  0.85823
## interview_age                                   -0.581  0.56152
## MRI_minus_hormone_date_time                     -0.639  0.52319
## bmi                                              1.647  0.09970 .
## household.income[>=200K]                        -2.582  0.00990 **
## household.income[100K-200K]                     -2.101  0.03577 *
## household.income[12K-16K]                        0.163  0.87091
## household.income[16K-25K]                       -0.020  0.98440
## household.income[25K-35K]                       -1.238  0.21599
## household.income[35K-50K]                       -0.285  0.77534
## household.income[50K-75K]                       -1.228  0.21943
## household.income[5K-12K]                         0.008  0.99355
## household.income[75K-100K]                      -1.608  0.10800
## high.educBachelor                               -0.246  0.80594
## high.educHS Diploma/GED                         -1.154  0.24850
## high.educPost Graduate Degree                    0.121  0.90339
```

```
## high.educSome College                               -0.032  0.97471
## hormone_scr_ert_mean:accumbens_posvsneg_feedback_z   0.251  0.80200
## ---
## Signif. codes:  0 '***' 0.001 '**' 0.01 '*' 0.05 '.' 0.1 ' ' 1
##
##
## R-sq.(adj) =  0.0249
## lmer.REML =  10450  Scale est. = 10.927    n = 1707
```

```
##                                                           stdcoef      stdse
## X(Intercept)                                         0.0000000000 0.00000000
## XPDS_score                                           0.0799411689 0.02735743
## Xhormone_sal_end_min_since_midnight                 -0.0315477903 0.02604921
## Xhormone_scr_ert_mean                               -0.0057105938 0.02568288
## Xaccumbens_posvsneg_feedback_z                      -0.0126344486 0.05887028
## Xrace.ethnicity.5levelBlack                         -0.0101006206 0.05708444
## Xrace.ethnicity.5levelMixed                          0.1141234916 0.05985908
## Xrace.ethnicity.5levelOther                          0.0726254886 0.04499910
## Xrace.ethnicity.5levelWhite                          0.1247055177 0.07759309
## Xdemo_race_hispanic1                                 0.0052162802 0.02919848
## Xinterview_age                                      -0.0144149190 0.02482322
## XMRI_minus_hormone_date_time                        -0.0159576453 0.02498961
## Xbmi                                                 0.0431776658 0.02621225
## Xhousehold.income[>=200K]                           -0.1568031199 0.06071964
## Xhousehold.income[100K-200K]                        -0.1660053688 0.07900514
## Xhousehold.income[12K-16K]                           0.0052182652 0.03210744
## Xhousehold.income[16K-25K]                          -0.0007298958 0.03731395
## Xhousehold.income[25K-35K]                          -0.0511935796 0.04136086
## Xhousehold.income[35K-50K]                          -0.0140547029 0.04923746
## Xhousehold.income[50K-75K]                          -0.0707374089 0.05758048
## Xhousehold.income[5K-12K]                            0.0002786871 0.03448910
## Xhousehold.income[75K-100K]                         -0.1007665639 0.06266248
## Xhigh.educBachelor                                  -0.0179136191 0.07290733
## Xhigh.educHS Diploma/GED                            -0.0472775463 0.04095438
## Xhigh.educPost Graduate Degree                       0.0095811684 0.07892292
## Xhigh.educSome College                              -0.0020510180 0.06469381
## Xhormone_scr_ert_mean:accumbens_posvsneg_feedback_z  0.0147779744 0.05892369
```

Male participants

```
## Warning: Some predictor variables are on very different scales: consider
## rescaling
```

```
##
## Family: gaussian
## Link function: identity
##
## Formula:
## cbcl_scr_syn_internal_r ~ PDS_score + hormone_sal_end_min_since_midnight +
##     hormone_scr_ert_mean * accumbens_posvsneg_feedback_z + race.ethnicity.5level +
##     demo_race_hispanic + interview_age + MRI_minus_hormone_date_time +
##     bmi + household.income + high.educ
##
```

```
## Parametric coefficients:
##                                                     Estimate Std. Error
## (Intercept)                                        1.150e+00  2.530e+00
## PDS_score                                          6.677e-01  2.588e-01
## hormone_sal_end_min_since_midnight                 4.719e-04  7.524e-04
## hormone_scr_ert_mean                              -5.815e-03  8.558e-03
## accumbens_posvsneg_feedback_z                      2.467e-01  3.786e-01
## race.ethnicity.5levelBlack                         2.690e-01  9.689e-01
## race.ethnicity.5levelMixed                         2.423e+00  9.304e-01
## race.ethnicity.5levelOther                         2.017e+00  1.084e+00
## race.ethnicity.5levelWhite                         1.762e+00  8.742e-01
## demo_race_hispanic1                               -4.332e-01  3.732e-01
## interview_age                                     -1.358e-04  1.679e-02
## MRI_minus_hormone_date_time                       -1.114e-05  1.423e-05
## bmi                                                5.472e-02  3.679e-02
## household.income[>=200K]                          -1.305e+00  1.006e+00
## household.income[100K-200K]                       -9.197e-01  9.507e-01
## household.income[12K-16K]                         -3.245e-01  1.240e+00
## household.income[16K-25K]                          1.185e+00  1.035e+00
## household.income[25K-35K]                          2.407e-01  1.007e+00
## household.income[35K-50K]                          5.440e-01  9.765e-01
## household.income[50K-75K]                         -1.820e-01  9.494e-01
## household.income[5K-12K]                           1.093e+00  1.085e+00
## household.income[75K-100K]                        -6.113e-01  9.621e-01
## high.educBachelor                                  5.938e-01  8.505e-01
## high.educHS Diploma/GED                           -7.018e-01  8.707e-01
## high.educPost Graduate Degree                      3.271e-01  8.616e-01
## high.educSome College                              6.116e-01  8.114e-01
## hormone_scr_ert_mean:accumbens_posvsneg_feedback_z 2.385e-03  1.069e-02
##                                                   t value Pr(>|t|)
## (Intercept)                                         0.455  0.64932
## PDS_score                                           2.580  0.00997 **
## hormone_sal_end_min_since_midnight                  0.627  0.53058
## hormone_scr_ert_mean                               -0.679  0.49696
## accumbens_posvsneg_feedback_z                       0.652  0.51472
## race.ethnicity.5levelBlack                          0.278  0.78132
## race.ethnicity.5levelMixed                          2.604  0.00930 **
## race.ethnicity.5levelOther                          1.861  0.06286 .
## race.ethnicity.5levelWhite                          2.016  0.04395 *
## demo_race_hispanic1                                -1.161  0.24591
## interview_age                                      -0.008  0.99355
## MRI_minus_hormone_date_time                        -0.783  0.43361
## bmi                                                 1.487  0.13716
## household.income[>=200K]                           -1.298  0.19457
## household.income[100K-200K]                        -0.967  0.33351
## household.income[12K-16K]                          -0.262  0.79354
## household.income[16K-25K]                           1.146  0.25213
## household.income[25K-35K]                           0.239  0.81108
## household.income[35K-50K]                           0.557  0.57757
## household.income[50K-75K]                          -0.192  0.84803
## household.income[5K-12K]                            1.007  0.31398
## household.income[75K-100K]                         -0.635  0.52526
## high.educBachelor                                   0.698  0.48514
## high.educHS Diploma/GED                            -0.806  0.42035
```

```
## high.educPost Graduate Degree                        0.380  0.70425
## high.educSome College                                0.754  0.45108
## hormone_scr_ert_mean:accumbens_posvsneg_feedback_z   0.223  0.82350
## ---
## Signif. codes:  0 '***' 0.001 '**' 0.01 '*' 0.05 '.' 0.1 ' ' 1
##
##
## R-sq.(adj) =  0.0225
## lmer.REML =  10377  Scale est. = 17.201    n = 1708
```

```
##                                                            stdcoef      stdse
## X(Intercept)                                          0.0000000000 0.00000000
## XPDS_score                                            0.0676577041 0.02622768
## Xhormone_sal_end_min_since_midnight                   0.0163370447 0.02604519
## Xhormone_scr_ert_mean                                -0.0174910266 0.02574376
## Xaccumbens_posvsneg_feedback_z                        0.0345240686 0.05297954
## Xrace.ethnicity.5levelBlack                           0.0165164096 0.05948789
## Xrace.ethnicity.5levelMixed                           0.1564224794 0.06007289
## Xrace.ethnicity.5levelOther                           0.0796885353 0.04281024
## Xrace.ethnicity.5levelWhite                           0.1580472347 0.07839296
## Xdemo_race_hispanic1                                 -0.0337855939 0.02910664
## Xinterview_age                                       -0.0002009955 0.02485034
## XMRI_minus_hormone_date_time                         -0.0195575441 0.02497075
## Xbmi                                                  0.0388718918 0.02613847
## Xhousehold.income[>=200K]                            -0.0840962854 0.06480444
## Xhousehold.income[100K-200K]                         -0.0855982893 0.08848843
## Xhousehold.income[12K-16K]                           -0.0086511329 0.03305084
## Xhousehold.income[16K-25K]                            0.0471403554 0.04114962
## Xhousehold.income[25K-35K]                            0.0109545934 0.04582197
## Xhousehold.income[35K-50K]                            0.0292436867 0.05249816
## Xhousehold.income[50K-75K]                           -0.0122808616 0.06407493
## Xhousehold.income[5K-12K]                             0.0369243464 0.03666018
## Xhousehold.income[75K-100K]                          -0.0429763509 0.06763695
## Xhigh.educBachelor                                    0.0525188537 0.07521959
## Xhigh.educHS Diploma/GED                             -0.0358059017 0.04442307
## Xhigh.educPost Graduate Degree                        0.0310452730 0.08177149
## Xhigh.educSome College                                0.0517440838 0.06864479
## Xhormone_scr_ert_mean:accumbens_posvsneg_feedback_z  0.0118506825 0.05312422
```

4.18 Model: CBCL internalizing factor ~ Testosterone x Caudate activity (Feedback stage) + PDS

Female participants

```
## Warning: Some predictor variables are on very different scales: consider
## rescaling
```

```
##
## Family: gaussian
## Link function: identity
##
## Formula:
## cbcl_scr_syn_internal_r ~ PDS_score + hormone_sal_end_min_since_midnight +
```

```
##     hormone_scr_ert_mean * caudate_posvsneg_feedback_z + race.ethnicity.5level +
##     demo_race_hispanic + interview_age + MRI_minus_hormone_date_time +
##     bmi + household.income + high.educ
##
## Parametric coefficients:
##                                               Estimate Std. Error t value
## (Intercept)                                  5.175e+00  2.559e+00   2.022
## PDS_score                                    6.181e-01  2.047e-01   3.020
## hormone_sal_end_min_since_midnight          -9.966e-04  7.788e-04  -1.280
## hormone_scr_ert_mean                        -3.057e-03  8.506e-03  -0.359
## caudate_posvsneg_feedback_z                 -3.740e-01  3.370e-01  -1.110
## race.ethnicity.5levelBlack                  -1.421e-01  9.891e-01  -0.144
## race.ethnicity.5levelMixed                   1.767e+00  9.429e-01   1.874
## race.ethnicity.5levelOther                   1.703e+00  1.062e+00   1.603
## race.ethnicity.5levelWhite                   1.387e+00  8.866e-01   1.564
## demo_race_hispanic1                          9.372e-02  3.936e-01   0.238
## interview_age                               -1.162e-02  1.756e-02  -0.661
## MRI_minus_hormone_date_time                 -9.672e-06  1.610e-05  -0.601
## bmi                                          5.878e-02  3.488e-02   1.685
## household.income[>=200K]                    -2.335e+00  9.547e-01  -2.446
## household.income[100K-200K]                 -1.841e+00  8.980e-01  -2.051
## household.income[12K-16K]                    2.154e-01  1.165e+00   0.185
## household.income[16K-25K]                    2.589e-02  1.005e+00   0.026
## household.income[25K-35K]                   -1.175e+00  9.508e-01  -1.236
## household.income[35K-50K]                   -2.233e-01  9.062e-01  -0.246
## household.income[50K-75K]                   -1.046e+00  9.097e-01  -1.150
## household.income[5K-12K]                    -1.899e-02  1.067e+00  -0.018
## household.income[75K-100K]                  -1.442e+00  9.092e-01  -1.585
## high.educBachelor                           -2.154e-01  8.499e-01  -0.253
## high.educHS Diploma/GED                     -1.091e+00  8.688e-01  -1.255
## high.educPost Graduate Degree                8.680e-02  8.588e-01   0.101
## high.educSome College                       -4.970e-02  8.040e-01  -0.062
## hormone_scr_ert_mean:caudate_posvsneg_feedback_z  5.414e-03  8.759e-03   0.618
##                                             Pr(>|t|)
## (Intercept)                                  0.04333 *
## PDS_score                                    0.00257 **
## hormone_sal_end_min_since_midnight           0.20086
## hormone_scr_ert_mean                         0.71939
## caudate_posvsneg_feedback_z                  0.26735
## race.ethnicity.5levelBlack                   0.88577
## race.ethnicity.5levelMixed                   0.06105 .
## race.ethnicity.5levelOther                   0.10904
## race.ethnicity.5levelWhite                   0.11804
## demo_race_hispanic1                          0.81185
## interview_age                                0.50845
## MRI_minus_hormone_date_time                  0.54794
## bmi                                          0.09215 .
## household.income[>=200K]                     0.01454 *
## household.income[100K-200K]                  0.04046 *
## household.income[12K-16K]                    0.85338
## household.income[16K-25K]                    0.97945
## household.income[25K-35K]                    0.21650
## household.income[35K-50K]                    0.80537
## household.income[50K-75K]                    0.25019
```

```
## household.income[5K-12K]                          0.98580
## household.income[75K-100K]                        0.11306
## high.educBachelor                                 0.79997
## high.educHS Diploma/GED                           0.20954
## high.educPost Graduate Degree                     0.91951
## high.educSome College                             0.95071
## hormone_scr_ert_mean:caudate_posvsneg_feedback_z  0.53657
## ---
## Signif. codes:  0 '***' 0.001 '**' 0.01 '*' 0.05 '.' 0.1 ' ' 1
##
##
## R-sq.(adj) =  0.0261
## lmer.REML =  10420  Scale est. = 10.986    n = 1702
```

```
##                                                          stdcoef      stdse
## X(Intercept)                                        0.0000000000 0.00000000
## XPDS_score                                          0.0827005848 0.02738493
## Xhormone_sal_end_min_since_midnight                -0.0333691150 0.02607764
## Xhormone_scr_ert_mean                              -0.0092590012 0.02576708
## Xcaudate_posvsneg_feedback_z                       -0.0608300790 0.05482437
## Xrace.ethnicity.5levelBlack                        -0.0081919844 0.05701360
## Xrace.ethnicity.5levelMixed                         0.1122391206 0.05987996
## Xrace.ethnicity.5levelOther                         0.0721271244 0.04498442
## Xrace.ethnicity.5levelWhite                         0.1212200604 0.07751292
## Xdemo_race_hispanic1                                0.0069809726 0.02932233
## Xinterview_age                                     -0.0164674479 0.02489832
## XMRI_minus_hormone_date_time                       -0.0150045269 0.02496737
## Xbmi                                                0.0441652435 0.02620865
## Xhousehold.income[>=200K]                          -0.1491670148 0.06098227
## Xhousehold.income[100K-200K]                       -0.1620761404 0.07903822
## Xhousehold.income[12K-16K]                          0.0059466288 0.03217161
## Xhousehold.income[16K-25K]                          0.0009599556 0.03726851
## Xhousehold.income[25K-35K]                         -0.0510832735 0.04131734
## Xhousehold.income[35K-50K]                         -0.0121977786 0.04949547
## Xhousehold.income[50K-75K]                         -0.0661992629 0.05755049
## Xhousehold.income[5K-12K]                          -0.0006130031 0.03442551
## Xhousehold.income[75K-100K]                        -0.0993911858 0.06268998
## Xhigh.educBachelor                                 -0.0184275074 0.07271308
## Xhigh.educHS Diploma/GED                           -0.0514028777 0.04094788
## Xhigh.educPost Graduate Degree                      0.0079685899 0.07884390
## Xhigh.educSome College                             -0.0039960926 0.06463989
## Xhormone_scr_ert_mean:caudate_posvsneg_feedback_z  0.0340791498 0.05513232
```

Male participants

```
## Warning: Some predictor variables are on very different scales: consider
## rescaling
```

```
##
## Family: gaussian
## Link function: identity
##
## Formula:
```

```
## cbcl_scr_syn_internal_r ~ PDS_score + hormone_sal_end_min_since_midnight +
##     hormone_scr_ert_mean * caudate_posvsneg_feedback_z + race.ethnicity.5level +
##     demo_race_hispanic + interview_age + MRI_minus_hormone_date_time +
##     bmi + household.income + high.educ
##
## Parametric coefficients:
##                                                    Estimate Std. Error t value
## (Intercept)                                       2.511e+00  2.565e+00   0.979
## PDS_score                                         7.429e-01  2.618e-01   2.837
## hormone_sal_end_min_since_midnight                5.355e-04  7.616e-04   0.703
## hormone_scr_ert_mean                             -3.514e-03  8.599e-03  -0.409
## caudate_posvsneg_feedback_z                       7.987e-02  3.282e-01   0.243
## race.ethnicity.5levelBlack                        5.869e-02  9.876e-01   0.059
## race.ethnicity.5levelMixed                        2.290e+00  9.508e-01   2.408
## race.ethnicity.5levelOther                        1.852e+00  1.102e+00   1.680
## race.ethnicity.5levelWhite                        1.712e+00  8.948e-01   1.913
## demo_race_hispanic1                              -4.036e-01  3.752e-01  -1.076
## interview_age                                    -1.028e-02  1.691e-02  -0.608
## MRI_minus_hormone_date_time                      -1.472e-05  1.443e-05  -1.020
## bmi                                               5.819e-02  3.725e-02   1.562
## household.income[>=200K]                         -1.108e+00  1.023e+00  -1.084
## household.income[100K-200K]                      -7.102e-01  9.670e-01  -0.734
## household.income[12K-16K]                        -3.456e-01  1.267e+00  -0.273
## household.income[16K-25K]                         1.298e+00  1.049e+00   1.237
## household.income[25K-35K]                         1.410e-01  1.025e+00   0.138
## household.income[35K-50K]                         7.902e-01  9.928e-01   0.796
## household.income[50K-75K]                         1.715e-02  9.654e-01   0.018
## household.income[5K-12K]                          1.851e+00  1.099e+00   1.684
## household.income[75K-100K]                       -4.598e-01  9.788e-01  -0.470
## high.educBachelor                                 5.870e-02  8.563e-01   0.069
## high.educHS Diploma/GED                          -1.225e+00  8.782e-01  -1.395
## high.educPost Graduate Degree                    -2.298e-01  8.679e-01  -0.265
## high.educSome College                             1.934e-01  8.165e-01   0.237
## hormone_scr_ert_mean:caudate_posvsneg_feedback_z  1.627e-03  9.335e-03   0.174
##                                                  Pr(>|t|)
## (Intercept)                                        0.3279
## PDS_score                                          0.0046 **
## hormone_sal_end_min_since_midnight                 0.4820
## hormone_scr_ert_mean                               0.6828
## caudate_posvsneg_feedback_z                        0.8078
## race.ethnicity.5levelBlack                         0.9526
## race.ethnicity.5levelMixed                         0.0161 *
## race.ethnicity.5levelOther                         0.0932 .
## race.ethnicity.5levelWhite                         0.0559 .
## demo_race_hispanic1                                0.2822
## interview_age                                      0.5433
## MRI_minus_hormone_date_time                        0.3077
## bmi                                                0.1184
## household.income[>=200K]                           0.2787
## household.income[100K-200K]                        0.4628
## household.income[12K-16K]                          0.7851
## household.income[16K-25K]                          0.2163
## household.income[25K-35K]                          0.8905
## household.income[35K-50K]                          0.4262
```

```
## household.income[50K-75K]                         0.9858  
## household.income[5K-12K]                          0.0924 .
## household.income[75K-100K]                        0.6386  
## high.educBachelor                                 0.9454  
## high.educHS Diploma/GED                           0.1632  
## high.educPost Graduate Degree                     0.7912  
## high.educSome College                             0.8128  
## hormone_scr_ert_mean:caudate_posvsneg_feedback_z   0.8617  
## ---
## Signif. codes:  0 '***' 0.001 '**' 0.01 '*' 0.05 '.' 0.1 ' ' 1
##
##
## R-sq.(adj) =  0.025   
## lmer.REML =  10421  Scale est. = 16.886    n = 1710
```

```
##                                                          stdcoef      stdse
## X(Intercept)                                         0.000000000 0.00000000
## XPDS_score                                           0.074409942 0.02622417
## Xhormone_sal_end_min_since_midnight                  0.018294050 0.02601530
## Xhormone_scr_ert_mean                               -0.010443696 0.02555439
## Xcaudate_posvsneg_feedback_z                         0.013518294 0.05554947
## Xrace.ethnicity.5levelBlack                          0.003595568 0.06050273
## Xrace.ethnicity.5levelMixed                          0.146452633 0.06081955
## Xrace.ethnicity.5levelOther                          0.072321567 0.04305672
## Xrace.ethnicity.5levelWhite                          0.152147723 0.07953081
## Xdemo_race_hispanic1                                -0.031156000 0.02896362
## Xinterview_age                                      -0.015074393 0.02479297
## XMRI_minus_hormone_date_time                        -0.025449646 0.02494369
## Xbmi                                                 0.040800857 0.02611877
## Xhousehold.income[>=200K]                           -0.070607496 0.06515843
## Xhousehold.income[100K-200K]                        -0.065341476 0.08896777
## Xhousehold.income[12K-16K]                          -0.008967432 0.03288197
## Xhousehold.income[16K-25K]                           0.051694324 0.04179612
## Xhousehold.income[25K-35K]                           0.006313095 0.04587025
## Xhousehold.income[35K-50K]                           0.042135623 0.05294064
## Xhousehold.income[50K-75K]                           0.001150452 0.06475874
## Xhousehold.income[5K-12K]                            0.062397366 0.03705283
## Xhousehold.income[75K-100K]                         -0.031908076 0.06792221
## Xhigh.educBachelor                                   0.005123814 0.07473975
## Xhigh.educHS Diploma/GED                            -0.061332766 0.04396685
## Xhigh.educPost Graduate Degree                      -0.021561492 0.08142821
## Xhigh.educSome College                               0.016278015 0.06873897
## Xhormone_scr_ert_mean:caudate_posvsneg_feedback_z  0.009711808 0.05573444
```

4.19 Model: CBCL internalizing factor ~ Testosterone x Putamen activity (Feedback stage) + PDS

Female participants

```
## Warning: Some predictor variables are on very different scales: consider
## rescaling
```

```
##
```

```
## Family: gaussian
## Link function: identity
##
## Formula:
## cbcl_scr_syn_internal_r ~ PDS_score + hormone_sal_end_min_since_midnight +
##     hormone_scr_ert_mean * putamen_posvsneg_feedback_z + race.ethnicity.5level +
##     demo_race_hispanic + interview_age + MRI_minus_hormone_date_time +
##     bmi + household.income + high.educ
##
## Parametric coefficients:
##                                                    Estimate Std. Error t value
## (Intercept)                                       4.916e+00  2.552e+00   1.926
## PDS_score                                         6.013e-01  2.045e-01   2.940
## hormone_sal_end_min_since_midnight               -1.044e-03  7.807e-04  -1.338
## hormone_scr_ert_mean                             -2.401e-03  8.539e-03  -0.281
## putamen_posvsneg_feedback_z                      -2.752e-01  3.756e-01  -0.733
## race.ethnicity.5levelBlack                       -6.006e-02  9.909e-01  -0.061
## race.ethnicity.5levelMixed                        1.811e+00  9.425e-01   1.922
## race.ethnicity.5levelOther                        1.776e+00  1.064e+00   1.669
## race.ethnicity.5levelWhite                        1.420e+00  8.867e-01   1.601
## demo_race_hispanic1                               1.089e-01  3.935e-01   0.277
## interview_age                                    -1.005e-02  1.752e-02  -0.574
## MRI_minus_hormone_date_time                      -7.194e-06  1.625e-05  -0.443
## bmi                                               5.874e-02  3.493e-02   1.682
## household.income[>=200K]                         -2.325e+00  9.550e-01  -2.434
## household.income[100K-200K]                     -1.825e+00  8.986e-01  -2.031
## household.income[12K-16K]                         2.221e-01  1.165e+00   0.191
## household.income[16K-25K]                         8.697e-03  1.002e+00   0.009
## household.income[25K-35K]                        -1.117e+00  9.505e-01  -1.175
## household.income[35K-50K]                        -2.129e-01  9.059e-01  -0.235
## household.income[50K-75K]                        -1.007e+00  9.111e-01  -1.105
## household.income[5K-12K]                         -4.703e-02  1.070e+00  -0.044
## household.income[75K-100K]                       -1.419e+00  9.097e-01  -1.559
## high.educBachelor                                -1.730e-01  8.509e-01  -0.203
## high.educHS Diploma/GED                          -1.003e+00  8.704e-01  -1.152
## high.educPost Graduate Degree                     1.353e-01  8.595e-01   0.157
## high.educSome College                             4.060e-03  8.044e-01   0.005
## hormone_scr_ert_mean:putamen_posvsneg_feedback_z  3.155e-03  9.716e-03   0.325
##                                                  Pr(>|t|)
## (Intercept)                                       0.05428 .
## PDS_score                                         0.00332 **
## hormone_sal_end_min_since_midnight                0.18117
## hormone_scr_ert_mean                              0.77857
## putamen_posvsneg_feedback_z                       0.46394
## race.ethnicity.5levelBlack                        0.95167
## race.ethnicity.5levelMixed                        0.05480 .
## race.ethnicity.5levelOther                        0.09524 .
## race.ethnicity.5levelWhite                        0.10955
## demo_race_hispanic1                               0.78203
## interview_age                                     0.56617
## MRI_minus_hormone_date_time                       0.65813
## bmi                                               0.09283 .
## household.income[>=200K]                          0.01502 *
## household.income[100K-200K]                       0.04240 *
```

```
## household.income[12K-16K]                        0.84883
## household.income[16K-25K]                        0.99308
## household.income[25K-35K]                        0.24013
## household.income[35K-50K]                        0.81423
## household.income[50K-75K]                        0.26923
## household.income[5K-12K]                         0.96493
## household.income[75K-100K]                       0.11907
## high.educBachelor                                0.83895
## high.educHS Diploma/GED                          0.24937
## high.educPost Graduate Degree                    0.87493
## high.educSome College                            0.99597
## hormone_scr_ert_mean:putamen_posvsneg_feedback_z  0.74540
## ---
## Signif. codes:  0 '***' 0.001 '**' 0.01 '*' 0.05 '.' 0.1 ' ' 1
##
##
## R-sq.(adj) =  0.0252
## lmer.REML =  10413  Scale est. = 10.985    n = 1701
```

```
##                                                        stdcoef      stdse
## X(Intercept)                                      0.0000000000 0.00000000
## XPDS_score                                        0.0805405392 0.02739218
## Xhormone_sal_end_min_since_midnight              -0.0349518651 0.02612776
## Xhormone_scr_ert_mean                            -0.0072524893 0.02578879
## Xputamen_posvsneg_feedback_z                     -0.0424290660 0.05792040
## Xrace.ethnicity.5levelBlack                      -0.0034463617 0.05685706
## Xrace.ethnicity.5levelMixed                       0.1153126311 0.06000211
## Xrace.ethnicity.5levelOther                       0.0748754692 0.04485441
## Xrace.ethnicity.5levelWhite                       0.1240398593 0.07747373
## Xdemo_race_hispanic1                              0.0081147240 0.02932536
## Xinterview_age                                   -0.0142638720 0.02485785
## XMRI_minus_hormone_date_time                     -0.0110670117 0.02500588
## Xbmi                                              0.0439766292 0.02615145
## Xhousehold.income[>=200K]                        -0.1485723569 0.06103158
## Xhousehold.income[100K-200K]                     -0.1606357006 0.07908800
## Xhousehold.income[12K-16K]                        0.0061356291 0.03218389
## Xhousehold.income[16K-25K]                        0.0003248905 0.03743301
## Xhousehold.income[25K-35K]                       -0.0485647133 0.04132863
## Xhousehold.income[35K-50K]                       -0.0116346158 0.04950732
## Xhousehold.income[50K-75K]                       -0.0636087779 0.05755418
## Xhousehold.income[5K-12K]                        -0.0015187492 0.03453670
## Xhousehold.income[75K-100K]                      -0.0978614183 0.06275297
## Xhigh.educBachelor                               -0.0148044794 0.07283195
## Xhigh.educHS Diploma/GED                         -0.0470958751 0.04087201
## Xhigh.educPost Graduate Degree                    0.0124226501 0.07891188
## Xhigh.educSome College                            0.0003268828 0.06476130
## Xhormone_scr_ert_mean:putamen_posvsneg_feedback_z  0.0188484325 0.05803620
```

Male participants

```
## Warning: Some predictor variables are on very different scales: consider
## rescaling
```

```
##
```

```
## Family: gaussian
## Link function: identity
##
## Formula:
## cbcl_scr_syn_internal_r ~ PDS_score + hormone_sal_end_min_since_midnight +
##     hormone_scr_ert_mean * putamen_posvsneg_feedback_z + race.ethnicity.5level +
##     demo_race_hispanic + interview_age + MRI_minus_hormone_date_time +
##     bmi + household.income + high.educ
##
## Parametric coefficients:
##                                                  Estimate Std. Error t value
## (Intercept)                                     2.011e+00  2.554e+00   0.788
## PDS_score                                       7.193e-01  2.615e-01   2.751
## hormone_sal_end_min_since_midnight              4.229e-04  7.612e-04   0.556
## hormone_scr_ert_mean                           -2.980e-03  8.574e-03  -0.348
## putamen_posvsneg_feedback_z                     3.189e-01  3.333e-01   0.957
## race.ethnicity.5levelBlack                      9.750e-02  9.792e-01   0.100
## race.ethnicity.5levelMixed                      2.348e+00  9.414e-01   2.494
## race.ethnicity.5levelOther                      1.901e+00  1.095e+00   1.736
## race.ethnicity.5levelWhite                      1.785e+00  8.856e-01   2.015
## demo_race_hispanic1                            -4.685e-01  3.779e-01  -1.240
## interview_age                                  -6.290e-03  1.693e-02  -0.372
## MRI_minus_hormone_date_time                    -1.174e-05  1.446e-05  -0.812
## bmi                                             6.231e-02  3.724e-02   1.673
## household.income[>=200K]                       -1.217e+00  1.016e+00  -1.198
## household.income[100K-200K]                    -8.017e-01  9.614e-01  -0.834
## household.income[12K-16K]                      -2.464e-01  1.256e+00  -0.196
## household.income[16K-25K]                       1.216e+00  1.043e+00   1.166
## household.income[25K-35K]                       3.284e-01  1.019e+00   0.322
## household.income[35K-50K]                       6.733e-01  9.889e-01   0.681
## household.income[50K-75K]                      -7.807e-02  9.600e-01  -0.081
## household.income[5K-12K]                        1.734e+00  1.091e+00   1.590
## household.income[75K-100K]                     -5.534e-01  9.732e-01  -0.569
## high.educBachelor                               1.378e-01  8.445e-01   0.163
## high.educHS Diploma/GED                        -1.176e+00  8.692e-01  -1.352
## high.educPost Graduate Degree                  -1.418e-01  8.550e-01  -0.166
## high.educSome College                           1.989e-01  8.051e-01   0.247
## hormone_scr_ert_mean:putamen_posvsneg_feedback_z -5.496e-03  9.405e-03  -0.584
##                                                Pr(>|t|)
## (Intercept)                                     0.43108
## PDS_score                                       0.00601 **
## hormone_sal_end_min_since_midnight              0.57856
## hormone_scr_ert_mean                            0.72819
## putamen_posvsneg_feedback_z                     0.33882
## race.ethnicity.5levelBlack                      0.92070
## race.ethnicity.5levelMixed                      0.01273 *
## race.ethnicity.5levelOther                      0.08276 .
## race.ethnicity.5levelWhite                      0.04406 *
## demo_race_hispanic1                             0.21524
## interview_age                                   0.71025
## MRI_minus_hormone_date_time                     0.41701
## bmi                                             0.09450 .
## household.income[>=200K]                        0.23104
## household.income[100K-200K]                     0.40445
```

```
## household.income[12K-16K]                        0.84456
## household.income[16K-25K]                        0.24375
## household.income[25K-35K]                        0.74735
## household.income[35K-50K]                        0.49605
## household.income[50K-75K]                        0.93519
## household.income[5K-12K]                         0.11205
## household.income[75K-100K]                       0.56966
## high.educBachelor                                0.87039
## high.educHS Diploma/GED                          0.17642
## high.educPost Graduate Degree                    0.86825
## high.educSome College                            0.80495
## hormone_scr_ert_mean:putamen_posvsneg_feedback_z  0.55907
## ---
## Signif. codes:  0 '***' 0.001 '**' 0.01 '*' 0.05 '.' 0.1 ' ' 1
##
##
## R-sq.(adj) =  0.0241
## lmer.REML =  10482  Scale est. = 17.07     n = 1718
```

```
##                                                        stdcoef      stdse
## X(Intercept)                                       0.000000000 0.00000000
## XPDS_score                                         0.071882136 0.02613404
## Xhormone_sal_end_min_since_midnight                0.014425522 0.02596421
## Xhormone_scr_ert_mean                             -0.008856081 0.02547843
## Xputamen_posvsneg_feedback_z                       0.053488991 0.05590595
## Xrace.ethnicity.5levelBlack                        0.005940750 0.05966475
## Xrace.ethnicity.5levelMixed                        0.149979792 0.06013620
## Xrace.ethnicity.5levelOther                        0.074336550 0.04282275
## Xrace.ethnicity.5levelWhite                        0.158217376 0.07851939
## Xdemo_race_hispanic1                              -0.035980201 0.02902206
## Xinterview_age                                    -0.009193892 0.02474295
## XMRI_minus_hormone_date_time                      -0.020191880 0.02487250
## Xbmi                                               0.043625585 0.02607531
## Xhousehold.income[>=200K]                         -0.077425129 0.06462190
## Xhousehold.income[100K-200K]                      -0.073486221 0.08812214
## Xhousehold.income[12K-16K]                        -0.006456663 0.03292534
## Xhousehold.income[16K-25K]                         0.048460078 0.04155805
## Xhousehold.income[25K-35K]                         0.014692871 0.04560322
## Xhousehold.income[35K-50K]                         0.035587730 0.05226860
## Xhousehold.income[50K-75K]                        -0.005209443 0.06405885
## Xhousehold.income[5K-12K]                          0.058640011 0.03688378
## Xhousehold.income[75K-100K]                       -0.038201183 0.06717678
## Xhigh.educBachelor                                 0.011977993 0.07340161
## Xhigh.educHS Diploma/GED                          -0.058747384 0.04343901
## Xhigh.educPost Graduate Degree                    -0.013263089 0.07994137
## Xhigh.educSome College                             0.016670800 0.06749645
## Xhormone_scr_ert_mean:putamen_posvsneg_feedback_z -0.032676123 0.05592001
```

4.20 Model: CBCL internalizing factor ~ Testosterone x Lateral OFC activity (anticipation stage) + PDS

Female participants

```
## Warning: Some predictor variables are on very different scales: consider
## rescaling


##
## Family: gaussian
## Link function: identity
##
## Formula:
## cbcl_scr_syn_internal_r ~ PDS_score + hormone_sal_end_min_since_midnight +
##     hormone_scr_ert_mean * lOFC_rvsn_ant_z + race.ethnicity.5level +
##     demo_race_hispanic + interview_age + MRI_minus_hormone_date_time +
##     bmi + household.income + high.educ
##
## Parametric coefficients:
##                                      Estimate Std. Error t value Pr(>|t|)
## (Intercept)                         5.131e+00  2.566e+00   2.000  0.04570 *
## PDS_score                           5.964e-01  2.054e-01   2.903  0.00374 **
## hormone_sal_end_min_since_midnight -8.785e-04  7.813e-04  -1.124  0.26099
## hormone_scr_ert_mean               -2.288e-03  8.571e-03  -0.267  0.78952
## lOFC_rvsn_ant_z                     1.243e-01  5.066e-01   0.245  0.80622
## race.ethnicity.5levelBlack         -2.778e-01  1.001e+00  -0.277  0.78149
## race.ethnicity.5levelMixed          1.697e+00  9.579e-01   1.771  0.07667 .
## race.ethnicity.5levelOther          1.621e+00  1.079e+00   1.502  0.13340
## race.ethnicity.5levelWhite          1.312e+00  9.004e-01   1.457  0.14524
## demo_race_hispanic1                 5.646e-02  3.924e-01   0.144  0.88560
## interview_age                      -1.172e-02  1.765e-02  -0.664  0.50671
## MRI_minus_hormone_date_time        -9.065e-06  1.623e-05  -0.559  0.57644
## bmi                                 5.995e-02  3.510e-02   1.708  0.08788 .
## household.income[>=200K]           -2.371e+00  9.569e-01  -2.478  0.01333 *
## household.income[100K-200K]        -1.840e+00  9.008e-01  -2.042  0.04129 *
## household.income[12K-16K]           1.688e-01  1.167e+00   0.145  0.88503
## household.income[16K-25K]           2.115e-02  1.004e+00   0.021  0.98320
## household.income[25K-35K]          -1.113e+00  9.547e-01  -1.165  0.24406
## household.income[35K-50K]          -1.623e-01  9.111e-01  -0.178  0.85864
## household.income[50K-75K]          -1.034e+00  9.106e-01  -1.136  0.25618
## household.income[5K-12K]            5.106e-02  1.066e+00   0.048  0.96181
## household.income[75K-100K]         -1.419e+00  9.115e-01  -1.557  0.11965
## high.educBachelor                  -1.806e-01  8.510e-01  -0.212  0.83201
## high.educHS Diploma/GED            -1.007e+00  8.677e-01  -1.160  0.24607
## high.educPost Graduate Degree       1.270e-01  8.596e-01   0.148  0.88261
## high.educSome College               4.265e-02  8.048e-01   0.053  0.95774
## hormone_scr_ert_mean:lOFC_rvsn_ant_z  2.043e-03  1.341e-02   0.152  0.87892
## ---
## Signif. codes:  0 '***' 0.001 '**' 0.01 '*' 0.05 '.' 0.1 ' ' 1
##
##
## R-sq.(adj) =  0.0256
## lmer.REML =  10410  Scale est. = 11.459    n = 1699
```

```
##                                              stdcoef      stdse
## X(Intercept)                            0.0000000000 0.00000000
## XPDS_score                              0.0796715192 0.02744070
## Xhormone_sal_end_min_since_midnight    -0.0293839010 0.02613219
## Xhormone_scr_ert_mean                  -0.0069003372 0.02584678
## XlOFC_rvsn_ant_z                        0.0134596424 0.05486028
## Xrace.ethnicity.5levelBlack            -0.0159822109 0.05761023
## Xrace.ethnicity.5levelMixed             0.1069805488 0.06039192
## Xrace.ethnicity.5levelOther             0.0683350504 0.04550991
## Xrace.ethnicity.5levelWhite             0.1143194467 0.07844929
## Xdemo_race_hispanic1                    0.0042228327 0.02934592
## Xinterview_age                         -0.0166100016 0.02501076
## XMRI_minus_hormone_date_time           -0.0140194973 0.02509314
## Xbmi                                    0.0448391071 0.02625696
## Xhousehold.income[>=200K]              -0.1512234431 0.06103705
## Xhousehold.income[100K-200K]           -0.1620354640 0.07934560
## Xhousehold.income[12K-16K]              0.0046619486 0.03223657
## Xhousehold.income[16K-25K]              0.0007901117 0.03750899
## Xhousehold.income[25K-35K]             -0.0483792683 0.04151587
## Xhousehold.income[35K-50K]             -0.0087926570 0.04935960
## Xhousehold.income[50K-75K]             -0.0657228083 0.05786156
## Xhousehold.income[5K-12K]               0.0016489633 0.03443511
## Xhousehold.income[75K-100K]            -0.0974585784 0.06259234
## Xhigh.educBachelor                     -0.0154527562 0.07283534
## Xhigh.educHS Diploma/GED               -0.0476789786 0.04108980
## Xhigh.educPost Graduate Degree          0.0116638645 0.07898083
## Xhigh.educSome College                  0.0034132324 0.06440337
## Xhormone_scr_ert_mean:lOFC_rvsn_ant_z  0.0083474453 0.05478884
```

Male participants

```
## Warning: Some predictor variables are on very different scales: consider
## rescaling
```

```
##
## Family: gaussian
## Link function: identity
##
## Formula:
## cbcl_scr_syn_internal_r ~ PDS_score + hormone_sal_end_min_since_midnight +
##     hormone_scr_ert_mean * lOFC_rvsn_ant_z + race.ethnicity.5level +
##     demo_race_hispanic + interview_age + MRI_minus_hormone_date_time +
##     bmi + household.income + high.educ
##
## Parametric coefficients:
##                                      Estimate Std. Error t value Pr(>|t|)
## (Intercept)                         7.193e-01  2.536e+00   0.284   0.7767
## PDS_score                           6.139e-01  2.610e-01   2.352   0.0188 *
## hormone_sal_end_min_since_midnight  4.380e-04  7.493e-04   0.585   0.5589
## hormone_scr_ert_mean               -6.270e-03  8.493e-03  -0.738   0.4604
## lOFC_rvsn_ant_z                     3.716e-01  4.289e-01   0.866   0.3864
## race.ethnicity.5levelBlack          1.358e-01  9.664e-01   0.140   0.8883
## race.ethnicity.5levelMixed          2.255e+00  9.288e-01   2.428   0.0153 *
```

```
## race.ethnicity.5levelOther            1.855e+00  1.080e+00   1.717   0.0862 .
## race.ethnicity.5levelWhite            1.636e+00  8.721e-01   1.876   0.0609 .
## demo_race_hispanic1                  -5.203e-01  3.716e-01  -1.400   0.1616
## interview_age                         5.256e-03  1.674e-02   0.314   0.7536
## MRI_minus_hormone_date_time          -7.494e-06  1.415e-05  -0.530   0.5964
## bmi                                   5.041e-02  3.694e-02   1.365   0.1725
## household.income[>=200K]             -1.447e+00  1.011e+00  -1.432   0.1524
## household.income[100K-200K]          -1.024e+00  9.588e-01  -1.068   0.2857
## household.income[12K-16K]            -4.503e-01  1.244e+00  -0.362   0.7174
## household.income[16K-25K]             9.799e-01  1.045e+00   0.938   0.3486
## household.income[25K-35K]             7.543e-02  1.015e+00   0.074   0.9407
## household.income[35K-50K]             4.464e-01  9.837e-01   0.454   0.6500
## household.income[50K-75K]            -3.592e-01  9.578e-01  -0.375   0.7077
## household.income[5K-12K]              1.204e+00  1.097e+00   1.098   0.2725
## household.income[75K-100K]           -6.957e-01  9.714e-01  -0.716   0.4740
## high.educBachelor                     8.458e-01  8.532e-01   0.991   0.3217
## high.educHS Diploma/GED              -5.380e-01  8.749e-01  -0.615   0.5386
## high.educPost Graduate Degree         5.312e-01  8.632e-01   0.615   0.5384
## high.educSome College                 9.231e-01  8.153e-01   1.132   0.2577
## hormone_scr_ert_mean:lOFC_rvsn_ant_z -1.403e-02  1.246e-02  -1.126   0.2602
## ---
## Signif. codes:  0 '***' 0.001 '**' 0.01 '*' 0.05 '.' 0.1 ' ' 1
##
##
## R-sq.(adj) =  0.0212
## lmer.REML =  10374  Scale est. = 16.214    n = 1709
```

```
##                                          stdcoef      stdse
## X(Intercept)                         0.000000000 0.00000000
## XPDS_score                           0.061866950 0.02630733
## Xhormone_sal_end_min_since_midnight  0.015208779 0.02601521
## Xhormone_scr_ert_mean               -0.018788895 0.02544880
## XlOFC_rvsn_ant_z                     0.045029394 0.05197030
## Xrace.ethnicity.5levelBlack          0.008352773 0.05946370
## Xrace.ethnicity.5levelMixed          0.145939140 0.06010199
## Xrace.ethnicity.5levelOther          0.073432370 0.04276820
## Xrace.ethnicity.5levelWhite          0.147030423 0.07838143
## Xdemo_race_hispanic1                -0.040666900 0.02904399
## Xinterview_age                       0.007805407 0.02485749
## XMRI_minus_hormone_date_time        -0.013209011 0.02494047
## Xbmi                                 0.035748733 0.02619653
## Xhousehold.income[>=200K]           -0.093827833 0.06553301
## Xhousehold.income[100K-200K]        -0.095565251 0.08948576
## Xhousehold.income[12K-16K]          -0.012031091 0.03323994
## Xhousehold.income[16K-25K]           0.038798644 0.04138188
## Xhousehold.income[25K-35K]           0.003440155 0.04627605
## Xhousehold.income[35K-50K]           0.024132433 0.05317139
## Xhousehold.income[50K-75K]          -0.024293339 0.06477917
## Xhousehold.income[5K-12K]            0.040384768 0.03679145
## Xhousehold.income[75K-100K]         -0.049014352 0.06843682
## Xhigh.educBachelor                   0.074876843 0.07553221
## Xhigh.educHS Diploma/GED            -0.027511146 0.04473457
## Xhigh.educPost Graduate Degree       0.050558151 0.08216817
## Xhigh.educSome College               0.078464770 0.06929495
```

```
## Xhormone_scr_ert_mean:lOFC_rvsn_ant_z -0.058804287 0.05221488
```

4.21 Model: CBCL internalizing factor ~ Testosterone x Medial OFC activity (anticipation stage) + PDS

Female participants

```
## Warning: Some predictor variables are on very different scales: consider
## rescaling
```

```
##
## Family: gaussian
## Link function: identity
##
## Formula:
## cbcl_scr_syn_internal_r ~ PDS_score + hormone_sal_end_min_since_midnight +
##     hormone_scr_ert_mean * mOFC_rvsn_ant_z + race.ethnicity.5level +
##     demo_race_hispanic + interview_age + MRI_minus_hormone_date_time +
##     bmi + household.income + high.educ
##
## Parametric coefficients:
##                                      Estimate Std. Error t value Pr(>|t|)
## (Intercept)                         5.172e+00  2.563e+00   2.018  0.04373 *
## PDS_score                           6.211e-01  2.055e-01   3.022  0.00255 **
## hormone_sal_end_min_since_midnight -9.234e-04  7.789e-04  -1.185  0.23600
## hormone_scr_ert_mean               -2.977e-03  8.547e-03  -0.348  0.72768
## mOFC_rvsn_ant_z                    -4.594e-02  4.442e-01  -0.103  0.91765
## race.ethnicity.5levelBlack         -2.799e-01  1.002e+00  -0.279  0.78003
## race.ethnicity.5levelMixed          1.723e+00  9.584e-01   1.798  0.07238 .
## race.ethnicity.5levelOther          1.613e+00  1.080e+00   1.493  0.13562
## race.ethnicity.5levelWhite          1.346e+00  9.010e-01   1.493  0.13552
## demo_race_hispanic1                 6.120e-02  3.926e-01   0.156  0.87615
## interview_age                      -1.035e-02  1.760e-02  -0.588  0.55655
## MRI_minus_hormone_date_time        -9.179e-06  1.622e-05  -0.566  0.57161
## bmi                                 5.397e-02  3.498e-02   1.543  0.12309
## household.income[>=200K]           -2.386e+00  9.588e-01  -2.489  0.01291 *
## household.income[100K-200K]        -1.875e+00  9.019e-01  -2.079  0.03777 *
## household.income[12K-16K]           1.706e-01  1.169e+00   0.146  0.88397
## household.income[16K-25K]          -2.987e-03  1.005e+00  -0.003  0.99763
## household.income[25K-35K]          -1.081e+00  9.568e-01  -1.130  0.25852
## household.income[35K-50K]         -1.945e-01  9.117e-01  -0.213  0.83110
## household.income[50K-75K]          -1.052e+00  9.115e-01  -1.154  0.24880
## household.income[5K-12K]            4.250e-02  1.068e+00   0.040  0.96826
## household.income[75K-100K]         -1.436e+00  9.129e-01  -1.573  0.11581
## high.educBachelor                  -2.383e-01  8.588e-01  -0.277  0.78144
## high.educHS Diploma/GED            -1.051e+00  8.732e-01  -1.203  0.22896
## high.educPost Graduate Degree       5.442e-02  8.675e-01   0.063  0.94998
## high.educSome College              -4.671e-02  8.121e-01  -0.058  0.95414
## hormone_scr_ert_mean:mOFC_rvsn_ant_z  3.782e-03  1.164e-02   0.325  0.74528
## ---
## Signif. codes:  0 '***' 0.001 '**' 0.01 '*' 0.05 '.' 0.1 ' ' 1
##
##
```

```
## R-sq.(adj) =  0.0252
## lmer.REML =  10405  Scale est. = 10.993    n = 1698

##                                             stdcoef      stdse
## X(Intercept)                           0.0000000000 0.00000000
## XPDS_score                             0.0826588179 0.02734791
## Xhormone_sal_end_min_since_midnight   -0.0308952379 0.02606165
## Xhormone_scr_ert_mean                 -0.0089591721 0.02572457
## XmOFC_rvsn_ant_z                      -0.0057367191 0.05547353
## Xrace.ethnicity.5levelBlack           -0.0160707147 0.05753420
## Xrace.ethnicity.5levelMixed            0.1084202698 0.06030456
## Xrace.ethnicity.5levelOther            0.0678690030 0.04545780
## Xrace.ethnicity.5levelWhite            0.1169941416 0.07834109
## Xdemo_race_hispanic1                   0.0045624301 0.02926852
## Xinterview_age                        -0.0146495894 0.02491023
## XMRI_minus_hormone_date_time          -0.0141667080 0.02503854
## Xbmi                                   0.0404112383 0.02619518
## Xhousehold.income[>=200K]             -0.1516285948 0.06092277
## Xhousehold.income[100K-200K]          -0.1648191191 0.07927691
## Xhousehold.income[12K-16K]             0.0047030232 0.03222076
## Xhousehold.income[16K-25K]            -0.0001113528 0.03748356
## Xhousehold.income[25K-35K]            -0.0467017375 0.04131828
## Xhousehold.income[35K-50K]            -0.0105157856 0.04929399
## Xhousehold.income[50K-75K]            -0.0666825004 0.05780081
## Xhousehold.income[5K-12K]              0.0013697058 0.03441689
## Xhousehold.income[75K-100K]           -0.0985855722 0.06265626
## Xhigh.educBachelor                    -0.0203528639 0.07334617
## Xhigh.educHS Diploma/GED              -0.0496616795 0.04126498
## Xhigh.educPost Graduate Degree         0.0049881032 0.07950733
## Xhigh.educSome College                -0.0037336428 0.06490809
## Xhormone_scr_ert_mean:mOFC_rvsn_ant_z  0.0180807607 0.05564728
```

Male participants

```
## Warning: Some predictor variables are on very different scales: consider
## rescaling

##
## Family: gaussian
## Link function: identity
##
## Formula:
## cbcl_scr_syn_internal_r ~ PDS_score + hormone_sal_end_min_since_midnight +
##     hormone_scr_ert_mean * mOFC_rvsn_ant_z + race.ethnicity.5level +
##     demo_race_hispanic + interview_age + MRI_minus_hormone_date_time +
##     bmi + household.income + high.educ
##
## Parametric coefficients:
##                                      Estimate Std. Error t value Pr(>|t|)
## (Intercept)                         6.122e-01  2.534e+00   0.242   0.8091
## PDS_score                           6.263e-01  2.617e-01   2.393   0.0168 *
## hormone_sal_end_min_since_midnight  3.834e-04  7.551e-04   0.508   0.6117
## hormone_scr_ert_mean               -8.359e-03  8.500e-03  -0.983   0.3256
```

```
## mOFC_rvsn_ant_z                     1.267e-01  3.908e-01   0.324   0.7458
## race.ethnicity.5levelBlack          1.651e-01  9.719e-01   0.170   0.8652
## race.ethnicity.5levelMixed          2.236e+00  9.330e-01   2.397   0.0167 *
## race.ethnicity.5levelOther          1.840e+00  1.085e+00   1.696   0.0900 .
## race.ethnicity.5levelWhite          1.683e+00  8.765e-01   1.921   0.0549 .
## demo_race_hispanic1                -4.497e-01  3.736e-01  -1.203   0.2290
## interview_age                       4.289e-03  1.682e-02   0.255   0.7987
## MRI_minus_hormone_date_time        -8.085e-06  1.462e-05  -0.553   0.5803
## bmi                                 6.185e-02  3.701e-02   1.671   0.0949 .
## household.income[>=200K]           -1.523e+00  1.009e+00  -1.510   0.1313
## household.income[100K-200K]        -1.133e+00  9.550e-01  -1.187   0.2355
## household.income[12K-16K]          -4.677e-01  1.243e+00  -0.376   0.7067
## household.income[16K-25K]           8.845e-01  1.040e+00   0.850   0.3952
## household.income[25K-35K]          4.721e-02  1.014e+00   0.047   0.9629
## household.income[35K-50K]           3.517e-01  9.798e-01   0.359   0.7197
## household.income[50K-75K]          -3.744e-01  9.546e-01  -0.392   0.6950
## household.income[5K-12K]            1.343e+00  1.103e+00   1.217   0.2236
## household.income[75K-100K]         -8.431e-01  9.684e-01  -0.871   0.3840
## high.educBachelor                   1.005e+00  8.526e-01   1.178   0.2389
## high.educHS Diploma/GED            -4.578e-01  8.765e-01  -0.522   0.6015
## high.educPost Graduate Degree       7.108e-01  8.632e-01   0.824   0.4103
## high.educSome College               1.031e+00  8.154e-01   1.264   0.2063
## hormone_scr_ert_mean:mOFC_rvsn_ant_z -2.661e-04  1.097e-02  -0.024   0.9807
## ---
## Signif. codes:  0 '***' 0.001 '**' 0.01 '*' 0.05 '.' 0.1 ' ' 1
##
##
## R-sq.(adj) =  0.022
## lmer.REML =  10345  Scale est. = 16.235    n = 1702
```

```
##                                         stdcoef      stdse
## X(Intercept)                        0.000000000 0.00000000
## XPDS_score                          0.063154279 0.02638969
## Xhormone_sal_end_min_since_midnight 0.013239124 0.02607461
## Xhormone_scr_ert_mean              -0.025070501 0.02549437
## XmOFC_rvsn_ant_z                    0.016719945 0.05156432
## Xrace.ethnicity.5levelBlack         0.010078532 0.05934564
## Xrace.ethnicity.5levelMixed         0.143972976 0.06007144
## Xrace.ethnicity.5levelOther         0.072649842 0.04283000
## Xrace.ethnicity.5levelWhite         0.150511851 0.07836152
## Xdemo_race_hispanic1               -0.035072765 0.02914303
## Xinterview_age                      0.006343975 0.02486915
## XMRI_minus_hormone_date_time       -0.013803967 0.02496192
## Xbmi                                0.043845945 0.02623557
## Xhousehold.income[>=200K]          -0.098288671 0.06509719
## Xhousehold.income[100K-200K]       -0.105252132 0.08868216
## Xhousehold.income[12K-16K]         -0.012460563 0.03311301
## Xhousehold.income[16K-25K]          0.035154383 0.04133626
## Xhousehold.income[25K-35K]          0.002136561 0.04590337
## Xhousehold.income[35K-50K]          0.018956740 0.05280810
## Xhousehold.income[50K-75K]         -0.025244465 0.06437372
## Xhousehold.income[5K-12K]           0.044045488 0.03618106
## Xhousehold.income[75K-100K]        -0.059031892 0.06779859
## Xhigh.educBachelor                  0.088567509 0.07517093
```

```
## Xhigh.educHS Diploma/GED              -0.023166950 0.04435229
## Xhigh.educPost Graduate Degree         0.067353808 0.08178579
## Xhigh.educSome College                 0.087124088 0.06890824
## Xhormone_scr_ert_mean:mOFC_rvsn_ant_z -0.001246187 0.05139691
```

4.22 Model: CBCL internalizing factor ~ Testosterone x Lateral OFC activity (feedback stage) + PDS

Female participants

```
## Warning: Some predictor variables are on very different scales: consider
## rescaling
```

```
##
## Family: gaussian
## Link function: identity
##
## Formula:
## cbcl_scr_syn_internal_r ~ PDS_score + hormone_sal_end_min_since_midnight +
##     hormone_scr_ert_mean * lOFC_posvsneg_feedback_z + race.ethnicity.5level +
##     demo_race_hispanic + interview_age + MRI_minus_hormone_date_time +
##     bmi + household.income + high.educ
##
## Parametric coefficients:
##                                              Estimate Std. Error t value
## (Intercept)                                 4.700e+00  2.550e+00   1.843
## PDS_score                                   6.250e-01  2.042e-01   3.061
## hormone_sal_end_min_since_midnight         -8.060e-04  7.769e-04  -1.037
## hormone_scr_ert_mean                       -3.921e-03  8.505e-03  -0.461
## lOFC_posvsneg_feedback_z                   -1.178e-01  6.282e-01  -0.187
## race.ethnicity.5levelBlack                 -1.230e-01  9.875e-01  -0.125
## race.ethnicity.5levelMixed                  1.767e+00  9.418e-01   1.876
## race.ethnicity.5levelOther                  1.986e+00  1.069e+00   1.857
## race.ethnicity.5levelWhite                  1.433e+00  8.849e-01   1.619
## demo_race_hispanic1                        -5.829e-02  3.910e-01  -0.149
## interview_age                              -1.062e-02  1.750e-02  -0.607
## MRI_minus_hormone_date_time                -9.137e-06  1.613e-05  -0.567
## bmi                                         5.760e-02  3.480e-02   1.655
## household.income[>=200K]                   -2.060e+00  9.693e-01  -2.126
## household.income[100K-200K]                -1.528e+00  9.137e-01  -1.672
## household.income[12K-16K]                  4.918e-01  1.178e+00   0.418
## household.income[16K-25K]                  3.301e-01  1.019e+00   0.324
## household.income[25K-35K]                  -7.743e-01  9.688e-01  -0.799
## household.income[35K-50K]                  7.026e-02  9.223e-01   0.076
## household.income[50K-75K]                  -7.315e-01  9.257e-01  -0.790
## household.income[5K-12K]                   3.934e-01  1.083e+00   0.363
## household.income[75K-100K]                 -1.091e+00  9.257e-01  -1.179
## high.educBachelor                          -3.065e-01  8.529e-01  -0.359
## high.educHS Diploma/GED                    -1.269e+00  8.721e-01  -1.455
## high.educPost Graduate Degree               2.273e-02  8.601e-01   0.026
## high.educSome College                      -6.160e-02  8.061e-01  -0.076
## hormone_scr_ert_mean:lOFC_posvsneg_feedback_z  4.257e-04  1.740e-02   0.024
##                                            Pr(>|t|)
```

```
## (Intercept)                                    0.06550 .
## PDS_score                                      0.00224 **
## hormone_sal_end_min_since_midnight             0.29968
## hormone_scr_ert_mean                           0.64483
## lOFC_posvsneg_feedback_z                       0.85130
## race.ethnicity.5levelBlack                     0.90088
## race.ethnicity.5levelMixed                     0.06077 .
## race.ethnicity.5levelOther                     0.06352 .
## race.ethnicity.5levelWhite                     0.10561
## demo_race_hispanic1                            0.88152
## interview_age                                  0.54414
## MRI_minus_hormone_date_time                    0.57107
## bmi                                            0.09805 .
## household.income[>=200K]                       0.03369 *
## household.income[100K-200K]                    0.09476 .
## household.income[12K-16K]                      0.67634
## household.income[16K-25K]                      0.74598
## household.income[25K-35K]                      0.42426
## household.income[35K-50K]                      0.93928
## household.income[50K-75K]                      0.42955
## household.income[5K-12K]                       0.71637
## household.income[75K-100K]                     0.23875
## high.educBachelor                              0.71939
## high.educHS Diploma/GED                        0.14595
## high.educPost Graduate Degree                  0.97892
## high.educSome College                          0.93909
## hormone_scr_ert_mean:lOFC_posvsneg_feedback_z  0.98048
## ---
## Signif. codes:  0 '***' 0.001 '**' 0.01 '*' 0.05 '.' 0.1 ' ' 1
##
##
## R-sq.(adj) =  0.0253
## lmer.REML =  10396  Scale est. = 10.994    n = 1699
```

```
##                                                     stdcoef      stdse
## X(Intercept)                                    0.000000000 0.00000000
## XPDS_score                                      0.083808201 0.02738026
## Xhormone_sal_end_min_since_midnight            -0.026966362 0.02599309
## Xhormone_scr_ert_mean                          -0.011886984 0.02578335
## XlOFC_posvsneg_feedback_z                      -0.011096695 0.05918505
## Xrace.ethnicity.5levelBlack                    -0.007090136 0.05691879
## Xrace.ethnicity.5levelMixed                     0.111848887 0.05960829
## Xrace.ethnicity.5levelOther                     0.082544932 0.04445553
## Xrace.ethnicity.5levelWhite                     0.125007008 0.07720684
## Xdemo_race_hispanic1                           -0.004352608 0.02919845
## Xinterview_age                                 -0.015095444 0.02488197
## XMRI_minus_hormone_date_time                   -0.014186810 0.02503922
## Xbmi                                            0.043335343 0.02617986
## Xhousehold.income[>=200K]                      -0.131421575 0.06182916
## Xhousehold.income[100K-200K]                   -0.134870744 0.08067594
## Xhousehold.income[12K-16K]                      0.013612067 0.03260072
## Xhousehold.income[16K-25K]                      0.012355358 0.03813346
## Xhousehold.income[25K-35K]                     -0.033566630 0.04199802
## Xhousehold.income[35K-50K]                      0.003847422 0.05050412
```

```
## Xhousehold.income[50K-75K]                       -0.046476196 0.05881894
## Xhousehold.income[5K-12K]                         0.012728142 0.03502732
## Xhousehold.income[75K-100K]                      -0.075291050 0.06388559
## Xhigh.educBachelor                               -0.026277135 0.07312835
## Xhigh.educHS Diploma/GED                         -0.059691685 0.04103506
## Xhigh.educPost Graduate Degree                    0.002091305 0.07912434
## Xhigh.educSome College                           -0.004960153 0.06490407
## Xhormone_scr_ert_mean:lOFC_posvsneg_feedback_z  0.001452810 0.05937458
```

Male participants

```
## Warning: Some predictor variables are on very different scales: consider
## rescaling
```

```
##
## Family: gaussian
## Link function: identity
##
## Formula:
## cbcl_scr_syn_internal_r ~ PDS_score + hormone_sal_end_min_since_midnight +
##     hormone_scr_ert_mean * lOFC_posvsneg_feedback_z + race.ethnicity.5level +
##     demo_race_hispanic + interview_age + MRI_minus_hormone_date_time +
##     bmi + household.income + high.educ
##
## Parametric coefficients:
##                                                  Estimate Std. Error t value
## (Intercept)                                     9.851e-01  2.526e+00   0.390
## PDS_score                                       6.633e-01  2.589e-01   2.562
## hormone_sal_end_min_since_midnight              4.118e-04  7.511e-04   0.548
## hormone_scr_ert_mean                           -7.542e-03  8.520e-03  -0.885
## lOFC_posvsneg_feedback_z                        1.339e-01  4.735e-01   0.283
## race.ethnicity.5levelBlack                      2.070e-01  9.697e-01   0.213
## race.ethnicity.5levelMixed                      2.401e+00  9.309e-01   2.579
## race.ethnicity.5levelOther                      1.865e+00  1.086e+00   1.716
## race.ethnicity.5levelWhite                      1.717e+00  8.751e-01   1.963
## demo_race_hispanic1                            -4.943e-01  3.735e-01  -1.323
## interview_age                                  -8.617e-04  1.675e-02  -0.051
## MRI_minus_hormone_date_time                    -8.214e-06  1.421e-05  -0.578
## bmi                                             6.854e-02  3.700e-02   1.852
## household.income[>=200K]                       -1.427e+00  1.005e+00  -1.421
## household.income[100K-200K]                    -1.042e+00  9.517e-01  -1.095
## household.income[12K-16K]                     -4.493e-01  1.241e+00  -0.362
## household.income[16K-25K]                      1.080e+00  1.042e+00   1.036
## household.income[25K-35K]                      1.614e-01  1.011e+00   0.160
## household.income[35K-50K]                      4.313e-01  9.774e-01   0.441
## household.income[50K-75K]                     -2.576e-01  9.506e-01  -0.271
## household.income[5K-12K]                       1.125e+00  1.084e+00   1.038
## household.income[75K-100K]                    -7.254e-01  9.634e-01  -0.753
## high.educBachelor                              9.198e-01  8.469e-01   1.086
## high.educHS Diploma/GED                       -4.429e-01  8.714e-01  -0.508
## high.educPost Graduate Degree                  6.107e-01  8.580e-01   0.712
## high.educSome College                          9.050e-01  8.103e-01   1.117
## hormone_scr_ert_mean:lOFC_posvsneg_feedback_z -5.769e-04  1.329e-02  -0.043
```

```
##                                               Pr(>|t|)
## (Intercept)                                    0.69655
## PDS_score                                      0.01050 *
## hormone_sal_end_min_since_midnight             0.58357
## hormone_scr_ert_mean                           0.37622
## lOFC_posvsneg_feedback_z                       0.77737
## race.ethnicity.5levelBlack                     0.83102
## race.ethnicity.5levelMixed                     0.00999 **
## race.ethnicity.5levelOther                     0.08628 .
## race.ethnicity.5levelWhite                     0.04985 *
## demo_race_hispanic1                            0.18593
## interview_age                                  0.95897
## MRI_minus_hormone_date_time                    0.56337
## bmi                                            0.06415 .
## household.income[>=200K]                       0.15559
## household.income[100K-200K]                    0.27360
## household.income[12K-16K]                      0.71748
## household.income[16K-25K]                      0.30054
## household.income[25K-35K]                      0.87312
## household.income[35K-50K]                      0.65911
## household.income[50K-75K]                      0.78642
## household.income[5K-12K]                       0.29931
## household.income[75K-100K]                     0.45157
## high.educBachelor                              0.27761
## high.educHS Diploma/GED                        0.61134
## high.educPost Graduate Degree                  0.47675
## high.educSome College                          0.26417
## hormone_scr_ert_mean:lOFC_posvsneg_feedback_z  0.96539
## ---
## Signif. codes:  0 '***' 0.001 '**' 0.01 '*' 0.05 '.' 0.1 ' ' 1
##
##
## R-sq.(adj) =  0.0216
## lmer.REML =  10428  Scale est. = 16.222    n = 1716
```

```
##                                                    stdcoef      stdse
## X(Intercept)                                   0.000000000 0.00000000
## XPDS_score                                     0.066954680 0.02613720
## Xhormone_sal_end_min_since_midnight            0.014231489 0.02595638
## Xhormone_scr_ert_mean                         -0.022587521 0.02551944
## XlOFC_posvsneg_feedback_z                      0.014580167 0.05155783
## Xrace.ethnicity.5levelBlack                    0.012691488 0.05946520
## Xrace.ethnicity.5levelMixed                    0.155106053 0.06013847
## Xrace.ethnicity.5levelOther                    0.072913837 0.04248204
## Xrace.ethnicity.5levelWhite                    0.153757225 0.07834019
## Xdemo_race_hispanic1                          -0.038462790 0.02906674
## Xinterview_age                                -0.001276803 0.02481747
## XMRI_minus_hormone_date_time                  -0.014385267 0.02488976
## Xbmi                                           0.048452474 0.02615767
## Xhousehold.income[>=200K]                     -0.092196804 0.06489617
## Xhousehold.income[100K-200K]                  -0.096881767 0.08846234
## Xhousehold.income[12K-16K]                    -0.011932722 0.03297315
## Xhousehold.income[16K-25K]                     0.042495153 0.04103427
## Xhousehold.income[25K-35K]                     0.007283698 0.04560172
```

```
## Xhousehold.income[35K-50K]                       0.023176774 0.05252829
## Xhousehold.income[50K-75K]                      -0.017419435 0.06427571
## Xhousehold.income[5K-12K]                        0.038211384 0.03680400
## Xhousehold.income[75K-100K]                     -0.050742759 0.06738998
## Xhigh.educBachelor                               0.081211788 0.07477716
## Xhigh.educHS Diploma/GED                        -0.022431274 0.04413369
## Xhigh.educPost Graduate Degree                   0.057847783 0.08128098
## Xhigh.educSome College                           0.076639017 0.06861326
## Xhormone_scr_ert_mean:lOFC_posvsneg_feedback_z -0.002248755 0.05182258
```

4.23 Model: CBCL internalizing factor ~ Testosterone x Medial OFC activity (feedback stage) + PDS

Female participants

```
## Warning: Some predictor variables are on very different scales: consider
## rescaling
```

```
##
## Family: gaussian
## Link function: identity
##
## Formula:
## cbcl_scr_syn_internal_r ~ PDS_score + hormone_sal_end_min_since_midnight +
##     hormone_scr_ert_mean * mOFC_posvsneg_feedback_z + race.ethnicity.5level +
##     demo_race_hispanic + interview_age + MRI_minus_hormone_date_time +
##     bmi + household.income + high.educ
##
## Parametric coefficients:
##                                               Estimate Std. Error t value
## (Intercept)                                  5.301e+00  2.548e+00   2.080
## PDS_score                                    6.422e-01  2.047e-01   3.137
## hormone_sal_end_min_since_midnight          -9.980e-04  7.776e-04  -1.283
## hormone_scr_ert_mean                        -3.441e-03  8.515e-03  -0.404
## mOFC_posvsneg_feedback_z                     1.454e-01  5.121e-01   0.284
## race.ethnicity.5levelBlack                  -1.715e-01  9.895e-01  -0.173
## race.ethnicity.5levelMixed                   1.776e+00  9.429e-01   1.884
## race.ethnicity.5levelOther                   1.807e+00  1.063e+00   1.699
## race.ethnicity.5levelWhite                   1.406e+00  8.861e-01   1.586
## demo_race_hispanic1                          2.899e-02  3.914e-01   0.074
## interview_age                               -1.308e-02  1.752e-02  -0.746
## MRI_minus_hormone_date_time                 -8.831e-06  1.613e-05  -0.548
## bmi                                          5.568e-02  3.493e-02   1.594
## household.income[>=200K]                    -2.382e+00  9.589e-01  -2.484
## household.income[100K-200K]                 -1.848e+00  9.028e-01  -2.047
## household.income[12K-16K]                   -1.297e-01  1.176e+00  -0.110
## household.income[16K-25K]                    1.151e-02  1.007e+00   0.011
## household.income[25K-35K]                   -1.042e+00  9.603e-01  -1.085
## household.income[35K-50K]                   -2.186e-01  9.105e-01  -0.240
## household.income[50K-75K]                   -1.090e+00  9.135e-01  -1.194
## household.income[5K-12K]                     8.755e-02  1.072e+00   0.082
## household.income[75K-100K]                  -1.422e+00  9.135e-01  -1.557
## high.educBachelor                           -1.252e-01  8.517e-01  -0.147
```

```
## high.educHS Diploma/GED                          -1.005e+00  8.666e-01  -1.160
## high.educPost Graduate Degree                     1.912e-01  8.586e-01   0.223
## high.educSome College                             4.857e-02  8.039e-01   0.060
## hormone_scr_ert_mean:mOFC_posvsneg_feedback_z -8.351e-03  1.401e-02  -0.596
##                                               Pr(>|t|)
## (Intercept)                                    0.03764 *
## PDS_score                                      0.00174 **
## hormone_sal_end_min_since_midnight             0.19951
## hormone_scr_ert_mean                           0.68618
## mOFC_posvsneg_feedback_z                       0.77647
## race.ethnicity.5levelBlack                     0.86243
## race.ethnicity.5levelMixed                     0.05979 .
## race.ethnicity.5levelOther                     0.08954 .
## race.ethnicity.5levelWhite                     0.11288
## demo_race_hispanic1                            0.94097
## interview_age                                  0.45555
## MRI_minus_hormone_date_time                    0.58406
## bmi                                            0.11108
## household.income[>=200K]                       0.01307 *
## household.income[100K-200K]                    0.04079 *
## household.income[12K-16K]                      0.91224
## household.income[16K-25K]                      0.99088
## household.income[25K-35K]                      0.27806
## household.income[35K-50K]                      0.81025
## household.income[50K-75K]                      0.23277
## household.income[5K-12K]                       0.93490
## household.income[75K-100K]                     0.11978
## high.educBachelor                              0.88317
## high.educHS Diploma/GED                        0.24626
## high.educPost Graduate Degree                  0.82383
## high.educSome College                          0.95182
## hormone_scr_ert_mean:mOFC_posvsneg_feedback_z  0.55111
## ---
## Signif. codes:  0 '***' 0.001 '**' 0.01 '*' 0.05 '.' 0.1 ' ' 1
##
##
## R-sq.(adj) =  0.025
## lmer.REML =  10407  Scale est. = 11.145    n = 1700
```

```
##                                                     stdcoef      stdse
## X(Intercept)                                  0.0000000000 0.00000000
## XPDS_score                                    0.0859927456 0.02741027
## Xhormone_sal_end_min_since_midnight          -0.0334293139 0.02604648
## Xhormone_scr_ert_mean                        -0.0104150420 0.02577218
## XmOFC_posvsneg_feedback_z                     0.0167734174 0.05906747
## Xrace.ethnicity.5levelBlack                  -0.0098217780 0.05667393
## Xrace.ethnicity.5levelMixed                   0.1122832577 0.05960967
## Xrace.ethnicity.5levelOther                   0.0762290813 0.04487256
## Xrace.ethnicity.5levelWhite                   0.1225643097 0.07726769
## Xdemo_race_hispanic1                          0.0021671461 0.02926292
## Xinterview_age                               -0.0185623859 0.02487011
## XMRI_minus_hormone_date_time                 -0.0137136681 0.02504488
## Xbmi                                          0.0417972110 0.02621809
## Xhousehold.income[>=200K]                    -0.1517801570 0.06109264
```

```
## Xhousehold.income[100K-200K]                  -0.1630067534 0.07962424
## Xhousehold.income[12K-16K]                    -0.0035371111 0.03208840
## Xhousehold.income[16K-25K]                     0.0004302306 0.03765028
## Xhousehold.income[25K-35K]                    -0.0448889650 0.04137115
## Xhousehold.income[35K-50K]                    -0.0119233251 0.04965153
## Xhousehold.income[50K-75K]                    -0.0692041605 0.05797463
## Xhousehold.income[5K-12K]                      0.0028295244 0.03463403
## Xhousehold.income[75K-100K]                   -0.0983142886 0.06316326
## Xhigh.educBachelor                            -0.0107284704 0.07299674
## Xhigh.educHS Diploma/GED                      -0.0474331911 0.04089466
## Xhigh.educPost Graduate Degree                 0.0175673105 0.07890051
## Xhigh.educSome College                         0.0039000986 0.06454497
## Xhormone_scr_ert_mean:mOFC_posvsneg_feedback_z -0.0352409074 0.05910727
```

Male participants

```
## Warning: Some predictor variables are on very different scales: consider
## rescaling
```

```
##
## Family: gaussian
## Link function: identity
##
## Formula:
## cbcl_scr_syn_internal_r ~ PDS_score + hormone_sal_end_min_since_midnight +
##     hormone_scr_ert_mean * mOFC_posvsneg_feedback_z + race.ethnicity.5level +
##     demo_race_hispanic + interview_age + MRI_minus_hormone_date_time +
##     bmi + household.income + high.educ
##
## Parametric coefficients:
##                                                 Estimate Std. Error t value
## (Intercept)                                    9.543e-01  2.526e+00   0.378
## PDS_score                                      6.685e-01  2.592e-01   2.579
## hormone_sal_end_min_since_midnight             4.188e-04  7.527e-04   0.556
## hormone_scr_ert_mean                          -7.142e-03  8.533e-03  -0.837
## mOFC_posvsneg_feedback_z                       4.783e-01  4.271e-01   1.120
## race.ethnicity.5levelBlack                     1.675e-01  9.696e-01   0.173
## race.ethnicity.5levelMixed                     2.424e+00  9.312e-01   2.603
## race.ethnicity.5levelOther                     1.850e+00  1.087e+00   1.702
## race.ethnicity.5levelWhite                     1.709e+00  8.752e-01   1.953
## demo_race_hispanic1                           -4.958e-01  3.733e-01  -1.328
## interview_age                                 -5.863e-04  1.673e-02  -0.035
## MRI_minus_hormone_date_time                   -9.130e-06  1.420e-05  -0.643
## bmi                                            6.825e-02  3.698e-02   1.846
## household.income[>=200K]                      -1.409e+00  1.005e+00  -1.402
## household.income[100K-200K]                   -1.027e+00  9.519e-01  -1.078
## household.income[12K-16K]                     -4.420e-01  1.243e+00  -0.356
## household.income[16K-25K]                      1.098e+00  1.042e+00   1.054
## household.income[25K-35K]                      1.487e-01  1.011e+00   0.147
## household.income[35K-50K]                      4.288e-01  9.775e-01   0.439
## household.income[50K-75K]                     -2.658e-01  9.506e-01  -0.280
## household.income[5K-12K]                       1.132e+00  1.083e+00   1.045
## household.income[75K-100K]                    -7.171e-01  9.636e-01  -0.744
```

```
## high.educBachelor                              8.890e-01  8.452e-01   1.052
## high.educHS Diploma/GED                        -4.673e-01  8.693e-01  -0.538
## high.educPost Graduate Degree                   5.914e-01  8.561e-01   0.691
## high.educSome College                           8.749e-01  8.084e-01   1.082
## hormone_scr_ert_mean:mOFC_posvsneg_feedback_z -5.922e-03  1.231e-02  -0.481
##                                               Pr(>|t|)
## (Intercept)                                    0.70563
## PDS_score                                      0.01000 *
## hormone_sal_end_min_since_midnight             0.57801
## hormone_scr_ert_mean                           0.40272
## mOFC_posvsneg_feedback_z                       0.26300
## race.ethnicity.5levelBlack                     0.86284
## race.ethnicity.5levelMixed                     0.00931 **
## race.ethnicity.5levelOther                     0.08900 .
## race.ethnicity.5levelWhite                     0.05096 .
## demo_race_hispanic1                            0.18435
## interview_age                                  0.97205
## MRI_minus_hormone_date_time                    0.52047
## bmi                                            0.06511 .
## household.income[>=200K]                       0.16114
## household.income[100K-200K]                    0.28102
## household.income[12K-16K]                      0.72214
## household.income[16K-25K]                      0.29226
## household.income[25K-35K]                      0.88307
## household.income[35K-50K]                      0.66095
## household.income[50K-75K]                      0.77980
## household.income[5K-12K]                       0.29604
## household.income[75K-100K]                     0.45690
## high.educBachelor                              0.29304
## high.educHS Diploma/GED                        0.59094
## high.educPost Graduate Degree                  0.48978
## high.educSome College                          0.27927
## hormone_scr_ert_mean:mOFC_posvsneg_feedback_z  0.63046
## ---
## Signif. codes:  0 '***' 0.001 '**' 0.01 '*' 0.05 '.' 0.1 ' ' 1
##
##
## R-sq.(adj) =  0.0228
## lmer.REML =  10411  Scale est. = 16.284    n = 1713
```

```
##                                                      stdcoef      stdse
## X(Intercept)                                    0.0000000000 0.00000000
## XPDS_score                                      0.0674163925 0.02614370
## Xhormone_sal_end_min_since_midnight             0.0144625764 0.02599282
## Xhormone_scr_ert_mean                          -0.0214474568 0.02562464
## XmOFC_posvsneg_feedback_z                       0.0598709797 0.05347029
## Xrace.ethnicity.5levelBlack                     0.0102972435 0.05959568
## Xrace.ethnicity.5levelMixed                     0.1566138004 0.06015517
## Xrace.ethnicity.5levelOther                     0.0723224661 0.04250093
## Xrace.ethnicity.5levelWhite                     0.1530806251 0.07837472
## Xdemo_race_hispanic1                           -0.0385796577 0.02905023
## Xinterview_age                                 -0.0008679922 0.02476582
## XMRI_minus_hormone_date_time                   -0.0160111845 0.02491031
## Xbmi                                            0.0482231460 0.02612686
```

```
## Xhousehold.income[>=200K]                    -0.0906393898 0.06465697
## Xhousehold.income[100K-200K]                 -0.0953001253 0.08837283
## Xhousehold.income[12K-16K]                   -0.0117392148 0.03300699
## Xhousehold.income[16K-25K]                    0.0432287455 0.04103300
## Xhousehold.income[25K-35K]                    0.0067084908 0.04560650
## Xhousehold.income[35K-50K]                    0.0230446532 0.05253188
## Xhousehold.income[50K-75K]                   -0.0180051797 0.06438961
## Xhousehold.income[5K-12K]                     0.0384657122 0.03679918
## Xhousehold.income[75K-100K]                  -0.0501537393 0.06739844
## Xhigh.educBachelor                            0.0783759190 0.07451543
## Xhigh.educHS Diploma/GED                     -0.0236673768 0.04402603
## Xhigh.educPost Graduate Degree                0.0559983749 0.08106230
## Xhigh.educSome College                        0.0740147032 0.06838630
## Xhormone_scr_ert_mean:mOFC_posvsneg_feedback_z -0.0258145553 0.05365001
```

4.24 Model: CBCL internalizing factor ~ Testosterone x BIS-BAS RR + PDS

Female participants

```
##
## Family: gaussian
## Link function: identity
##
## Formula:
## cbcl_scr_syn_internal_r ~ PDS_score + hormone_sal_end_min_since_midnight +
##     hormone_scr_ert_mean * bisbas_ss_basm_rr + race.ethnicity.5level +
##     demo_race_hispanic + interview_age + bmi + household.income +
##     high.educ
##
## Parametric coefficients:
##                                      Estimate Std. Error t value Pr(>|t|)
## (Intercept)                          2.5733616  2.4827380   1.037  0.30008
## PDS_score                            0.5419974  0.1807518   2.999  0.00274
## hormone_sal_end_min_since_midnight  -0.0001384  0.0006846  -0.202  0.83981
## hormone_scr_ert_mean                -0.0035476  0.0262828  -0.135  0.89264
## bisbas_ss_basm_rr                   -0.0625266  0.1139654  -0.549  0.58330
## race.ethnicity.5levelBlack          -0.7915837  0.8747433  -0.905  0.36560
## race.ethnicity.5levelMixed           0.9786799  0.8494547   1.152  0.24939
## race.ethnicity.5levelOther           1.8781297  0.9701226   1.936  0.05300
## race.ethnicity.5levelWhite           1.2030182  0.7992739   1.505  0.13243
## demo_race_hispanic1                 -0.2824106  0.3533951  -0.799  0.42430
## interview_age                        0.0033422  0.0157035   0.213  0.83148
## bmi                                  0.0813539  0.0309720   2.627  0.00868
## household.income[>=200K]            -2.0418215  0.8203184  -2.489  0.01288
## household.income[100K-200K]         -1.4328992  0.7659654  -1.871  0.06152
## household.income[12K-16K]            0.4741226  1.0246572   0.463  0.64362
## household.income[16K-25K]            0.9595029  0.8552890   1.122  0.26205
## household.income[25K-35K]           -0.3037771  0.8049072  -0.377  0.70591
## household.income[35K-50K]           -0.0097083  0.7744048  -0.013  0.99000
## household.income[50K-75K]           -0.4143656  0.7725939  -0.536  0.59178
## household.income[5K-12K]             0.3385735  0.9017917   0.375  0.70737
## household.income[75K-100K]          -0.8689576  0.7748511  -1.121  0.26222
## high.educBachelor                    0.3964730  0.7581573   0.523  0.60107
```

```
## high.educHS Diploma/GED                -0.6478445  0.7611177  -0.851  0.39476
## high.educPost Graduate Degree           0.6376835  0.7654442   0.833  0.40488
## high.educSome College                   0.5901610  0.7149730   0.825  0.40922
## hormone_scr_ert_mean:bisbas_ss_basm_rr -0.0004832  0.0029170  -0.166  0.86845
##
## (Intercept)
## PDS_score                              **
## hormone_sal_end_min_since_midnight
## hormone_scr_ert_mean
## bisbas_ss_basm_rr
## race.ethnicity.5levelBlack
## race.ethnicity.5levelMixed
## race.ethnicity.5levelOther             .
## race.ethnicity.5levelWhite
## demo_race_hispanic1
## interview_age
## bmi                                    **
## household.income[>=200K]               *
## household.income[100K-200K]            .
## household.income[12K-16K]
## household.income[16K-25K]
## household.income[25K-35K]
## household.income[35K-50K]
## household.income[50K-75K]
## household.income[5K-12K]
## household.income[75K-100K]
## high.educBachelor
## high.educHS Diploma/GED
## high.educPost Graduate Degree
## high.educSome College
## hormone_scr_ert_mean:bisbas_ss_basm_rr
## ---
## Signif. codes:  0 '***' 0.001 '**' 0.01 '*' 0.05 '.' 0.1 ' ' 1
##
##
## R-sq.(adj) =  0.0294
## lmer.REML =  13745  Scale est. = 13.519    n = 2229
```

```
##                                              stdcoef      stdse
## X(Intercept)                            0.0000000000 0.00000000
## XPDS_score                              0.0720555063 0.02402994
## Xhormone_sal_end_min_since_midnight    -0.0045472734 0.02249406
## Xhormone_scr_ert_mean                  -0.0106236418 0.07870553
## Xbisbas_ss_basm_rr                     -0.0270174152 0.04924382
## Xrace.ethnicity.5levelBlack            -0.0491437649 0.05430655
## Xrace.ethnicity.5levelMixed             0.0608485697 0.05281411
## Xrace.ethnicity.5levelOther             0.0742013763 0.03832772
## Xrace.ethnicity.5levelWhite             0.1045593401 0.06946823
## Xdemo_race_hispanic1                   -0.0202974397 0.02539925
## Xinterview_age                          0.0046670612 0.02192875
## Xbmi                                    0.0605015500 0.02303341
## Xhousehold.income[>=200K]              -0.1261999004 0.05070184
## Xhousehold.income[100K-200K]           -0.1219822049 0.06520636
## Xhousehold.income[12K-16K]              0.0125889296 0.02720675
```

```
## Xhousehold.income[16K-25K]               0.0358201274 0.03192962
## Xhousehold.income[25K-35K]              -0.0133450834 0.03535998
## Xhousehold.income[35K-50K]              -0.0005081138 0.04053093
## Xhousehold.income[50K-75K]              -0.0259504426 0.04838517
## Xhousehold.income[5K-12K]                0.0110628248 0.02946588
## Xhousehold.income[75K-100K]             -0.0583595638 0.05203933
## Xhigh.educBachelor                       0.0328002739 0.06272246
## Xhigh.educHS Diploma/GED                -0.0312589608 0.03672447
## Xhigh.educPost Graduate Degree           0.0568747629 0.06826969
## Xhigh.educSome College                   0.0470217714 0.05696632
## Xhormone_scr_ert_mean:bisbas_ss_basm_rr -0.0148476720 0.08963542
```

Male participants

```
##
## Family: gaussian
## Link function: identity
##
## Formula:
## cbcl_scr_syn_internal_r ~ PDS_score + hormone_sal_end_min_since_midnight +
##     hormone_scr_ert_mean * bisbas_ss_basm_rr + race.ethnicity.5level +
##     demo_race_hispanic + interview_age + bmi + household.income +
##     high.educ
##
## Parametric coefficients:
##                                          Estimate Std. Error t value Pr(>|t|)
## (Intercept)                             3.4157241  2.4037605   1.421  0.15545
## PDS_score                               0.8451464  0.2215717   3.814  0.00014
## hormone_sal_end_min_since_midnight      0.0005545  0.0006601   0.840  0.40096
## hormone_scr_ert_mean                   -0.0010417  0.0279147  -0.037  0.97024
## bisbas_ss_basm_rr                      -0.0340725  0.1042457  -0.327  0.74381
## race.ethnicity.5levelBlack              0.3496232  0.8189951   0.427  0.66950
## race.ethnicity.5levelMixed              1.8665517  0.7968386   2.342  0.01924
## race.ethnicity.5levelOther              1.2554938  0.9431208   1.331  0.18325
## race.ethnicity.5levelWhite              1.5980957  0.7448239   2.146  0.03200
## demo_race_hispanic1                    -0.4157467  0.3350506  -1.241  0.21478
## interview_age                          -0.0099159  0.0150292  -0.660  0.50946
## bmi                                     0.0696153  0.0320889   2.169  0.03015
## household.income[>=200K]               -2.4010204  0.7978384  -3.009  0.00264
## household.income[100K-200K]            -2.3047120  0.7407782  -3.111  0.00189
## household.income[12K-16K]              -0.8957783  1.0044135  -0.892  0.37257
## household.income[16K-25K]              -0.2313940  0.8254941  -0.280  0.77926
## household.income[25K-35K]              -1.0943380  0.8022721  -1.364  0.17268
## household.income[35K-50K]              -0.7046157  0.7719108  -0.913  0.36143
## household.income[50K-75K]              -1.4180418  0.7391606  -1.918  0.05517
## household.income[5K-12K]                0.9260322  0.8651056   1.070  0.28453
## household.income[75K-100K]             -1.9537490  0.7530364  -2.594  0.00953
## high.educBachelor                       0.7562883  0.7362945   1.027  0.30445
## high.educHS Diploma/GED                -0.2374542  0.7452505  -0.319  0.75004
## high.educPost Graduate Degree           0.6241826  0.7470882   0.835  0.40353
## high.educSome College                   0.8622771  0.7010440   1.230  0.21882
## hormone_scr_ert_mean:bisbas_ss_basm_rr -0.0003459  0.0029899  -0.116  0.90791
##
```

```
## (Intercept)
## PDS_score                             ***
## hormone_sal_end_min_since_midnight
## hormone_scr_ert_mean
## bisbas_ss_basm_rr
## race.ethnicity.5levelBlack
## race.ethnicity.5levelMixed            *
## race.ethnicity.5levelOther
## race.ethnicity.5levelWhite            *
## demo_race_hispanic1
## interview_age
## bmi                                   *
## household.income[>=200K]              **
## household.income[100K-200K]           **
## household.income[12K-16K]
## household.income[16K-25K]
## household.income[25K-35K]
## household.income[35K-50K]
## household.income[50K-75K]             .
## household.income[5K-12K]
## household.income[75K-100K]            **
## high.educBachelor
## high.educHS Diploma/GED
## high.educPost Graduate Degree
## high.educSome College
## hormone_scr_ert_mean:bisbas_ss_basm_rr
## ---
## Signif. codes:  0 '***' 0.001 '**' 0.01 '*' 0.05 '.' 0.1 ' ' 1
##
##
## R-sq.(adj) =  0.024
## lmer.REML =  15028  Scale est. = 14.409    n = 2427
```

```
##                                            stdcoef      stdse
## X(Intercept)                           0.000000000 0.00000000
## XPDS_score                             0.082894135 0.02173232
## Xhormone_sal_end_min_since_midnight    0.017898284 0.02130617
## Xhormone_scr_ert_mean                 -0.002967065 0.07950975
## Xbisbas_ss_basm_rr                    -0.014307018 0.04377268
## Xrace.ethnicity.5levelBlack            0.021984466 0.05149878
## Xrace.ethnicity.5levelMixed            0.113155962 0.04830674
## Xrace.ethnicity.5levelOther            0.046333882 0.03480579
## Xrace.ethnicity.5levelWhite            0.137543759 0.06410497
## Xdemo_race_hispanic1                  -0.029969706 0.02415261
## Xinterview_age                        -0.013750385 0.02084105
## Xbmi                                   0.047273037 0.02179034
## Xhousehold.income[>=200K]             -0.144302217 0.04795038
## Xhousehold.income[100K-200K]          -0.198162280 0.06369312
## Xhousehold.income[12K-16K]            -0.022939869 0.02572189
## Xhousehold.income[16K-25K]            -0.008966966 0.03198950
## Xhousehold.income[25K-35K]            -0.046769143 0.03428701
## Xhousehold.income[35K-50K]            -0.035310403 0.03868276
## Xhousehold.income[50K-75K]            -0.090746036 0.04730177
## Xhousehold.income[5K-12K]              0.030773505 0.02874882
```

```
## Xhousehold.income[75K-100K]             -0.126687285 0.04882927
## Xhigh.educBachelor                       0.062663249 0.06100664
## Xhigh.educHS Diploma/GED                -0.011816057 0.03708472
## Xhigh.educPost Graduate Degree           0.054706040 0.06547802
## Xhigh.educSome College                   0.069092822 0.05617348
## Xhormone_scr_ert_mean:bisbas_ss_basm_rr -0.010306438 0.08909063
```

4.25 Model: CBCL internalizing factor ~ Testosterone x MID Reaction Time + PDS (large reward vs. neutral)

Female participants

```
##
## Family: gaussian
## Link function: identity
##
## Formula:
## cbcl_scr_syn_internal_r ~ PDS_score + hormone_sal_end_min_since_midnight +
##     hormone_scr_ert_mean * rt_diff_large_neutral_z + race.ethnicity.5level +
##     demo_race_hispanic + interview_age + bmi + household.income +
##     high.educ
##
## Parametric coefficients:
##                                               Estimate Std. Error t value
## (Intercept)                                  6.5413632  2.4427387   2.678
## PDS_score                                    0.6396860  0.1967267   3.252
## hormone_sal_end_min_since_midnight          -0.0008488  0.0007362  -1.153
## hormone_scr_ert_mean                        -0.0060913  0.0081338  -0.749
## rt_diff_large_neutral_z                     -0.0604958  0.3090848  -0.196
## race.ethnicity.5levelBlack                  -0.1737481  0.9346733  -0.186
## race.ethnicity.5levelMixed                   1.4835060  0.9004032   1.648
## race.ethnicity.5levelOther                   2.0454507  1.0145698   2.016
## race.ethnicity.5levelWhite                   1.3912211  0.8439108   1.649
## demo_race_hispanic1                          0.0320287  0.3781033   0.085
## interview_age                               -0.0250939  0.0167438  -1.499
## bmi                                          0.0681206  0.0334878   2.034
## household.income[>=200K]                    -1.9388609  0.9064784  -2.139
## household.income[100K-200K]                 -1.3959074  0.8513444  -1.640
## household.income[12K-16K]                   -0.1381205  1.1020988  -0.125
## household.income[16K-25K]                    0.5341098  0.9564338   0.558
## household.income[25K-35K]                   -0.7422370  0.9005217  -0.824
## household.income[35K-50K]                   -0.2891681  0.8567313  -0.338
## household.income[50K-75K]                   -0.7458736  0.8628224  -0.864
## household.income[5K-12K]                     0.1412259  1.0154006   0.139
## household.income[75K-100K]                  -1.0553524  0.8596646  -1.228
## high.educBachelor                           -0.4078834  0.8222157  -0.496
## high.educHS Diploma/GED                     -1.3051980  0.8395918  -1.555
## high.educPost Graduate Degree               -0.2660409  0.8292069  -0.321
## high.educSome College                       -0.4513730  0.7743815  -0.583
## hormone_scr_ert_mean:rt_diff_large_neutral_z 0.0048676  0.0080199   0.607
##                                              Pr(>|t|)
## (Intercept)                                   0.00748 **
## PDS_score                                     0.00117 **
```

```
## hormone_sal_end_min_since_midnight           0.24913
## hormone_scr_ert_mean                         0.45402
## rt_diff_large_neutral_z                      0.84485
## race.ethnicity.5levelBlack                   0.85255
## race.ethnicity.5levelMixed                   0.09961 .
## race.ethnicity.5levelOther                   0.04394 *
## race.ethnicity.5levelWhite                   0.09941 .
## demo_race_hispanic1                          0.93250
## interview_age                                0.13413
## bmi                                          0.04208 *
## household.income[>=200K]                     0.03258 *
## household.income[100K-200K]                  0.10125
## household.income[12K-16K]                    0.90028
## household.income[16K-25K]                    0.57661
## household.income[25K-35K]                    0.40992
## household.income[35K-50K]                    0.73576
## household.income[50K-75K]                    0.38745
## household.income[5K-12K]                     0.88940
## household.income[75K-100K]                   0.21974
## high.educBachelor                            0.61990
## high.educHS Diploma/GED                      0.12022
## high.educPost Graduate Degree                0.74837
## high.educSome College                        0.56004
## hormone_scr_ert_mean:rt_diff_large_neutral_z  0.54397
## ---
## Signif. codes:  0 '***' 0.001 '**' 0.01 '*' 0.05 '.' 0.1 ' ' 1
##
##
## R-sq.(adj) =  0.0221
## lmer.REML =  11257  Scale est. = 11.55     n = 1845
```

```
##                                                    stdcoef      stdse
## X(Intercept)                                   0.000000000 0.00000000
## XPDS_score                                     0.085818983 0.02639246
## Xhormone_sal_end_min_since_midnight           -0.028585920 0.02479644
## Xhormone_scr_ert_mean                         -0.018528236 0.02474109
## Xrt_diff_large_neutral_z                      -0.010591032 0.05411166
## Xrace.ethnicity.5levelBlack                   -0.010354335 0.05570086
## Xrace.ethnicity.5levelMixed                    0.093846327 0.05695935
## Xrace.ethnicity.5levelOther                    0.086086951 0.04270023
## Xrace.ethnicity.5levelWhite                    0.122657007 0.07440340
## Xdemo_race_hispanic1                           0.002380926 0.02810715
## Xinterview_age                                -0.035998443 0.02401983
## Xbmi                                           0.051156458 0.02514829
## Xhousehold.income[>=200K]                     -0.124867248 0.05837936
## Xhousehold.income[100K-200K]                  -0.123761560 0.07548045
## Xhousehold.income[12K-16K]                    -0.003877357 0.03093843
## Xhousehold.income[16K-25K]                     0.019697844 0.03527305
## Xhousehold.income[25K-35K]                    -0.032304191 0.03919318
## Xhousehold.income[35K-50K]                    -0.015927576 0.04718935
## Xhousehold.income[50K-75K]                    -0.047183830 0.05458199
## Xhousehold.income[5K-12K]                      0.004533425 0.03259490
## Xhousehold.income[75K-100K]                   -0.073254954 0.05967172
## Xhigh.educBachelor                            -0.034987966 0.07052912
```

```
## Xhigh.educHS Diploma/GED                       -0.060817852 0.03912216
## Xhigh.educPost Graduate Degree                 -0.024611893 0.07671133
## Xhigh.educSome College                         -0.036689608 0.06294517
## Xhormone_scr_ert_mean:rt_diff_large_neutral_z  0.032757320 0.05397155
```

Male participants

```
##
## Family: gaussian
## Link function: identity
##
## Formula:
## cbcl_scr_syn_internal_r ~ PDS_score + hormone_sal_end_min_since_midnight +
##     hormone_scr_ert_mean * rt_diff_large_neutral_z + race.ethnicity.5level +
##     demo_race_hispanic + interview_age + bmi + household.income +
##     high.educ
##
## Parametric coefficients:
##                                                 Estimate Std. Error t value
## (Intercept)                                    2.1361936  2.4126705   0.885
## PDS_score                                      0.5962403  0.2475137   2.409
## hormone_sal_end_min_since_midnight             0.0003230  0.0007084   0.456
## hormone_scr_ert_mean                          -0.0045554  0.0080824  -0.564
## rt_diff_large_neutral_z                        0.5834987  0.2942373   1.983
## race.ethnicity.5levelBlack                     0.0395398  0.9301344   0.043
## race.ethnicity.5levelMixed                     2.0221029  0.8962546   2.256
## race.ethnicity.5levelOther                     1.3148952  1.0489371   1.254
## race.ethnicity.5levelWhite                     1.5175861  0.8441638   1.798
## demo_race_hispanic1                           -0.4751032  0.3593433  -1.322
## interview_age                                 -0.0022479  0.0160977  -0.140
## bmi                                            0.0855763  0.0351420   2.435
## household.income[>=200K]                      -2.2089607  0.9446375  -2.338
## household.income[100K-200K]                   -1.7367544  0.8890270  -1.954
## household.income[12K-16K]                     -1.3279664  1.1807770  -1.125
## household.income[16K-25K]                      0.0891287  0.9716020   0.092
## household.income[25K-35K]                     -0.5108687  0.9414259  -0.543
## household.income[35K-50K]                     -0.1069482  0.9152755  -0.117
## household.income[50K-75K]                     -1.2342727  0.8870770  -1.391
## household.income[5K-12K]                       1.0548150  1.0315982   1.023
## household.income[75K-100K]                    -1.5857704  0.9004481  -1.761
## high.educBachelor                              0.5975418  0.8000213   0.747
## high.educHS Diploma/GED                       -0.9192753  0.8293281  -1.108
## high.educPost Graduate Degree                  0.3095046  0.8131105   0.381
## high.educSome College                          0.7224167  0.7651848   0.944
## hormone_scr_ert_mean:rt_diff_large_neutral_z -0.0116613  0.0081555  -1.430
##                                               Pr(>|t|)
## (Intercept)                                     0.3760
## PDS_score                                       0.0161 *
## hormone_sal_end_min_since_midnight              0.6485
## hormone_scr_ert_mean                            0.5731
## rt_diff_large_neutral_z                         0.0475 *
## race.ethnicity.5levelBlack                      0.9661
## race.ethnicity.5levelMixed                      0.0242 *
```

```
## race.ethnicity.5levelOther                      0.2102
## race.ethnicity.5levelWhite                      0.0724 .
## demo_race_hispanic1                             0.1863
## interview_age                                   0.8890
## bmi                                             0.0150 *
## household.income[>=200K]                        0.0195 *
## household.income[100K-200K]                     0.0509 .
## household.income[12K-16K]                       0.2609
## household.income[16K-25K]                       0.9269
## household.income[25K-35K]                       0.5874
## household.income[35K-50K]                       0.9070
## household.income[50K-75K]                       0.1643
## household.income[5K-12K]                        0.3067
## household.income[75K-100K]                      0.0784 .
## high.educBachelor                               0.4552
## high.educHS Diploma/GED                         0.2678
## high.educPost Graduate Degree                   0.7035
## high.educSome College                           0.3452
## hormone_scr_ert_mean:rt_diff_large_neutral_z   0.1529
## ---
## Signif. codes:  0 '***' 0.001 '**' 0.01 '*' 0.05 '.' 0.1 ' ' 1
##
##
## R-sq.(adj) =  0.0262
## lmer.REML =  11865  Scale est. = 15.987    n = 1941
```

```
##                                                     stdcoef      stdse
## X(Intercept)                                    0.000000000 0.00000000
## XPDS_score                                      0.058742286 0.02438534
## Xhormone_sal_end_min_since_midnight             0.010896051 0.02389620
## Xhormone_scr_ert_mean                          -0.013430932 0.02382982
## Xrt_diff_large_neutral_z                        0.097485038 0.04915819
## Xrace.ethnicity.5levelBlack                     0.002441591 0.05743592
## Xrace.ethnicity.5levelMixed                     0.129218848 0.05727354
## Xrace.ethnicity.5levelOther                     0.050035426 0.03991498
## Xrace.ethnicity.5levelWhite                     0.134000337 0.07453827
## Xdemo_race_hispanic1                           -0.035648766 0.02696287
## Xinterview_age                                 -0.003248565 0.02326342
## Xbmi                                            0.059593883 0.02447227
## Xhousehold.income[>=200K]                      -0.137173839 0.05866087
## Xhousehold.income[100K-200K]                   -0.157198299 0.08046822
## Xhousehold.income[12K-16K]                     -0.034296840 0.03049544
## Xhousehold.income[16K-25K]                      0.003530210 0.03848320
## Xhousehold.income[25K-35K]                     -0.023087499 0.04254551
## Xhousehold.income[35K-50K]                     -0.005614283 0.04804769
## Xhousehold.income[50K-75K]                     -0.082140767 0.05903492
## Xhousehold.income[5K-12K]                       0.034664137 0.03390117
## Xhousehold.income[75K-100K]                    -0.106810954 0.06065047
## Xhigh.educBachelor                              0.051740691 0.06927324
## Xhigh.educHS Diploma/GED                       -0.045222157 0.04079736
## Xhigh.educPost Graduate Degree                  0.028386331 0.07457475
## Xhigh.educSome College                          0.060074178 0.06363065
## Xhormone_scr_ert_mean:rt_diff_large_neutral_z -0.070292740 0.04916042
```

4.26 Model: CBCL internalizing factor ~ Testosterone x MID Reaction Time + PDS (large vs. small reward)

Female participants

```
##
## Family: gaussian
## Link function: identity
##
## Formula:
## cbcl_scr_syn_internal_r ~ PDS_score + hormone_sal_end_min_since_midnight +
##     hormone_scr_ert_mean * rt_diff_large_small_z + race.ethnicity.5level +
##     demo_race_hispanic + interview_age + bmi + household.income +
##     high.educ
##
## Parametric coefficients:
##                                             Estimate Std. Error t value
## (Intercept)                                6.5136501  2.4416108   2.668
## PDS_score                                  0.6459282  0.1966569   3.285
## hormone_sal_end_min_since_midnight        -0.0008263  0.0007359  -1.123
## hormone_scr_ert_mean                      -0.0064726  0.0081312  -0.796
## rt_diff_large_small_z                     -0.2989034  0.3011891  -0.992
## race.ethnicity.5levelBlack                -0.1727935  0.9343065  -0.185
## race.ethnicity.5levelMixed                 1.4831680  0.9001886   1.648
## race.ethnicity.5levelOther                 2.0112018  1.0144435   1.983
## race.ethnicity.5levelWhite                 1.3910547  0.8438130   1.649
## demo_race_hispanic1                       -0.0017022  0.3781363  -0.005
## interview_age                             -0.0245902  0.0167264  -1.470
## bmi                                        0.0667401  0.0334825   1.993
## household.income[>=200K]                  -1.9178829  0.9066466  -2.115
## household.income[100K-200K]               -1.3697516  0.8518457  -1.608
## household.income[12K-16K]                 -0.1397710  1.1017579  -0.127
## household.income[16K-25K]                  0.5818334  0.9561320   0.609
## household.income[25K-35K]                 -0.7095187  0.9007144  -0.788
## household.income[35K-50K]                 -0.2580339  0.8569849  -0.301
## household.income[50K-75K]                 -0.7013218  0.8633823  -0.812
## household.income[5K-12K]                   0.1541572  1.0155816   0.152
## household.income[75K-100K]                -1.0437494  0.8600931  -1.214
## high.educBachelor                         -0.4476021  0.8217406  -0.545
## high.educHS Diploma/GED                   -1.3216781  0.8394559  -1.574
## high.educPost Graduate Degree             -0.3044644  0.8280769  -0.368
## high.educSome College                     -0.4763828  0.7742828  -0.615
## hormone_scr_ert_mean:rt_diff_large_small_z  0.0032307  0.0079265   0.408
##                                           Pr(>|t|)
## (Intercept)                                0.00770 **
## PDS_score                                  0.00104 **
## hormone_sal_end_min_since_midnight         0.26167
## hormone_scr_ert_mean                       0.42613
## rt_diff_large_small_z                      0.32113
## race.ethnicity.5levelBlack                 0.85329
## race.ethnicity.5levelMixed                 0.09960 .
## race.ethnicity.5levelOther                 0.04757 *
## race.ethnicity.5levelWhite                 0.09942 .
## demo_race_hispanic1                        0.99641
```

```
## interview_age                               0.14170
## bmi                                         0.04638 *
## household.income[>=200K]                    0.03453 *
## household.income[100K-200K]                 0.10801
## household.income[12K-16K]                   0.89906
## household.income[16K-25K]                   0.54291
## household.income[25K-35K]                   0.43096
## household.income[35K-50K]                   0.76338
## household.income[50K-75K]                   0.41673
## household.income[5K-12K]                    0.87937
## household.income[75K-100K]                  0.22508
## high.educBachelor                           0.58603
## high.educHS Diploma/GED                     0.11556
## high.educPost Graduate Degree               0.71316
## high.educSome College                       0.53846
## hormone_scr_ert_mean:rt_diff_large_small_z  0.68363
## ---
## Signif. codes:  0 '***' 0.001 '**' 0.01 '*' 0.05 '.' 0.1 ' ' 1
##
##
## R-sq.(adj) =  0.0225
## lmer.REML =  11256  Scale est. = 11.441    n = 1845
```

```
##                                                   stdcoef      stdse
## X(Intercept)                                0.0000000000 0.00000000
## XPDS_score                                  0.0866564279 0.02638309
## Xhormone_sal_end_min_since_midnight        -0.0278281861 0.02478444
## Xhormone_scr_ert_mean                      -0.0196878773 0.02473296
## Xrt_diff_large_small_z                     -0.0532040293 0.05361087
## Xrace.ethnicity.5levelBlack                -0.0102974466 0.05567900
## Xrace.ethnicity.5levelMixed                 0.0938249419 0.05694577
## Xrace.ethnicity.5levelOther                 0.0846455190 0.04269492
## Xrace.ethnicity.5levelWhite                 0.1226423385 0.07439477
## Xdemo_race_hispanic1                       -0.0001265352 0.02810960
## Xinterview_age                             -0.0352757967 0.02399486
## Xbmi                                        0.0501197247 0.02514434
## Xhousehold.income[>=200K]                  -0.1235162163 0.05839020
## Xhousehold.income[100K-200K]               -0.1214425754 0.07552489
## Xhousehold.income[12K-16K]                 -0.0039236911 0.03092886
## Xhousehold.income[16K-25K]                  0.0214578780 0.03526192
## Xhousehold.income[25K-35K]                 -0.0308802001 0.03920156
## Xhousehold.income[35K-50K]                 -0.0142126841 0.04720332
## Xhousehold.income[50K-75K]                 -0.0443654946 0.05461741
## Xhousehold.income[5K-12K]                   0.0049485274 0.03260070
## Xhousehold.income[75K-100K]                -0.0724495540 0.05970146
## Xhigh.educBachelor                         -0.0383950054 0.07048836
## Xhigh.educHS Diploma/GED                   -0.0615857667 0.03911583
## Xhigh.educPost Graduate Degree             -0.0281665149 0.07660680
## Xhigh.educSome College                     -0.0387225120 0.06293715
## Xhormone_scr_ert_mean:rt_diff_large_small_z  0.0218662258 0.05364978
```

Male participants

```
##
```

```
## Family: gaussian
## Link function: identity
##
## Formula:
## cbcl_scr_syn_internal_r ~ PDS_score + hormone_sal_end_min_since_midnight +
##     hormone_scr_ert_mean * rt_diff_large_small_z + race.ethnicity.5level +
##     demo_race_hispanic + interview_age + bmi + household.income +
##     high.educ
##
## Parametric coefficients:
##                                             Estimate Std. Error t value
## (Intercept)                                2.1312062  2.4161651   0.882
## PDS_score                                  0.5771526  0.2475086   2.332
## hormone_sal_end_min_since_midnight         0.0003167  0.0007085   0.447
## hormone_scr_ert_mean                      -0.0049959  0.0080963  -0.617
## rt_diff_large_small_z                      0.1189404  0.2904566   0.409
## race.ethnicity.5levelBlack                 0.0052800  0.9308585   0.006
## race.ethnicity.5levelMixed                 1.9592624  0.8968232   2.185
## race.ethnicity.5levelOther                 1.2386673  1.0493278   1.180
## race.ethnicity.5levelWhite                 1.4677433  0.8447909   1.737
## demo_race_hispanic1                       -0.4864069  0.3596077  -1.353
## interview_age                             -0.0016934  0.0161228  -0.105
## bmi                                        0.0876847  0.0352306   2.489
## household.income[>=200K]                  -2.1989955  0.9451083  -2.327
## household.income[100K-200K]               -1.7614038  0.8899890  -1.979
## household.income[12K-16K]                 -1.3614243  1.1810888  -1.153
## household.income[16K-25K]                  0.0730500  0.9723225   0.075
## household.income[25K-35K]                 -0.5382203  0.9425100  -0.571
## household.income[35K-50K]                 -0.1104774  0.9162691  -0.121
## household.income[50K-75K]                 -1.2238868  0.8879513  -1.378
## household.income[5K-12K]                   1.0069019  1.0323808   0.975
## household.income[75K-100K]                -1.6099755  0.9015575  -1.786
## high.educBachelor                          0.6053939  0.8013260   0.755
## high.educHS Diploma/GED                   -0.8691451  0.8313977  -1.045
## high.educPost Graduate Degree              0.3265201  0.8144361   0.401
## high.educSome College                      0.7235600  0.7647606   0.946
## hormone_scr_ert_mean:rt_diff_large_small_z -0.0057109  0.0082624  -0.691
##                                           Pr(>|t|)
## (Intercept)                                 0.3779
## PDS_score                                   0.0198 *
## hormone_sal_end_min_since_midnight          0.6549
## hormone_scr_ert_mean                        0.5373
## rt_diff_large_small_z                       0.6822
## race.ethnicity.5levelBlack                  0.9955
## race.ethnicity.5levelMixed                  0.0290 *
## race.ethnicity.5levelOther                  0.2380
## race.ethnicity.5levelWhite                  0.0825 .
## demo_race_hispanic1                         0.1763
## interview_age                               0.9164
## bmi                                         0.0129 *
## household.income[>=200K]                    0.0201 *
## household.income[100K-200K]                 0.0479 *
## household.income[12K-16K]                   0.2492
## household.income[16K-25K]                  0.9401
```

```
## household.income[25K-35K]                     0.5680
## household.income[35K-50K]                     0.9040
## household.income[50K-75K]                     0.1683
## household.income[5K-12K]                      0.3295
## household.income[75K-100K]                    0.0743 .
## high.educBachelor                             0.4500
## high.educHS Diploma/GED                       0.2960
## high.educPost Graduate Degree                 0.6885
## high.educSome College                         0.3442
## hormone_scr_ert_mean:rt_diff_large_small_z   0.4895
## ---
## Signif. codes:  0 '***' 0.001 '**' 0.01 '*' 0.05 '.' 0.1 ' ' 1
##
##
## R-sq.(adj) =  0.0245
## lmer.REML =  11869  Scale est. = 16.097    n = 1941
```

```
##                                                   stdcoef      stdse
## X(Intercept)                                 0.0000000000 0.00000000
## XPDS_score                                   0.0568617498 0.02438484
## Xhormone_sal_end_min_since_midnight          0.0106826638 0.02389816
## Xhormone_scr_ert_mean                       -0.0147297677 0.02387071
## Xrt_diff_large_small_z                       0.0199835464 0.04880050
## Xrace.ethnicity.5levelBlack                  0.0003260411 0.05748063
## Xrace.ethnicity.5levelMixed                  0.1252031396 0.05730987
## Xrace.ethnicity.5levelOther                  0.0471347415 0.03992985
## Xrace.ethnicity.5levelWhite                  0.1295993039 0.07459364
## Xdemo_race_hispanic1                        -0.0364969225 0.02698271
## Xinterview_age                              -0.0024471684 0.02329958
## Xbmi                                         0.0610621591 0.02453402
## Xhousehold.income[>=200K]                   -0.1365550116 0.05869011
## Xhousehold.income[100K-200K]                -0.1594293819 0.08055529
## Xhousehold.income[12K-16K]                  -0.0351609438 0.03050349
## Xhousehold.income[16K-25K]                   0.0028933628 0.03851174
## Xhousehold.income[25K-35K]                  -0.0243235907 0.04259450
## Xhousehold.income[35K-50K]                  -0.0057995475 0.04809985
## Xhousehold.income[50K-75K]                  -0.0814495892 0.05909310
## Xhousehold.income[5K-12K]                    0.0330895821 0.03392689
## Xhousehold.income[75K-100K]                 -0.1084413103 0.06072520
## Xhigh.educBachelor                           0.0524205957 0.06938621
## Xhigh.educHS Diploma/GED                    -0.0427560875 0.04089917
## Xhigh.educPost Graduate Degree               0.0299469195 0.07469632
## Xhigh.educSome College                       0.0601692439 0.06359537
## Xhormone_scr_ert_mean:rt_diff_large_small_z -0.0336212555 0.04864283
```
