# Supplementary material for: Reward sensitivity and internalizing symptoms during the transition to puberty: An examination of 9-and 10-year-olds in the ABCD Study
Source: Dev Cogn Neurosci. 2022 Oct 31;58:101172. doi: 10.1016/j.dcn.2022.101172 (PMC9649995; doi:10.1016/j.dcn.2022.101172)
Supplement: Supplementary file 4 — Supplementary material [file mmc4.pdf]

Supplement D

Reward sensitivity and internalizing symptoms during the transition to puberty: An examination of 9- and 10-year-olds in the ABCD Study.

Contents

Sensitivity Analysis Results for Sample 2 8

1—Internalizing~Puberty— 8

1.1 Model: CBCL internalizing factor ~ PDS 8

Female participants 8

Male participants 9

1.2 Model: CBCL Anxious-Depressed ~ PDS 10

Female participants 10

Male participants 11

1.3 Model: CBCL Withdrawn-Depressed ~ PDS 13

Female participants 13

Male participants 14

1.4 Model: CBCL Depressed DSM-5 ~ PDS 15

Female participants 15

Male participants 16

1.5 Model: CBCL internalizing factor ~ Pubertal category 18

Female participants 18

Male participants 19

1.6 Model: CBCL Anxious-Depressed ~ Pubertal category 20

Female participants 20

Male participants 21

1.7 Model: CBCL Withdrawn-Depressed ~ Pubertal category 23

Female participants 23

Male participants 24

1.8 Model: CBCL Depressed DSM-5 ~ Pubertal category 25

Female participants 25

Male participants 27

1.9 Model: CBCL internalizing factor ~ Testosterone 28
Female participants 28
Male participants 29
1.10 Model: CBCL Anxious-Depressed ~ Testosterone 31
Female participants 31
Male participants 32
1.11 Model: CBCL Withdrawn-Depressed ~ Testosterone 33
Female participants 33
Male participants 35
1.12 Model: CBCL Depressed DSM-5 ~ Testosterone 36
Female participants 36
Male participants 37
1.13 Model: CBCL internalizing factor ~ Testosterone + PDS 38
Female participants 38
Male participants 40
1.14 Model: CBCL internalizing factor ~ Testosterone + Pubertal category 41
Female participants 41
Male participants 42
1.15 Model: CBCL Anxious-Depressed ~ Testosterone + PDS 44
Female participants 44
Male participants 45
1.16 Model: CBCL Anxious-Depressed ~ Testosterone + Pubertal category 47
Female participants 47
Male participants 48
1.17 Model: CBCL Withdrawn-Depressed ~ Testosterone + PDS 49
Female participants 49
Male participants 51
1.18 Model: CBCL Withdrawn-Depressed ~ Testosterone + Pubertal category 52
Female participants 52
Male participants 53
1.19 Model: CBCL Depressed DSM-5 ~ Testosterone + PDS 55
Female participants 55
Male participants 56
1.20 Model: CBCL Depressed DSM-5 ~ Testosterone + Pubertal category 58
Female participants 58
Male participants 59

2—Reward~Puberty— **61**
2.1 Model: BIS-BAS-RR ~ PDS 61
Female participants 61
Male participants 61
2.2 Model : Reaction Time ~ PDS 62
Female participants 62
Male participants 63
2.3 Model: Caudate Anticipation ~ PDS 64
Female participants 64
Male participants 64
2.4 Model B: Putamen Anticipation ~ PDS 65
Female participants 65
Male participants 65
2.5 Model: Accumbens Anticipation ~ PDS 66
Female participants 66
Male participants 66
2.6 Model: Caudate Feedback ~ PDS 67
Female participants 67
Male participants 67
2.7 Model: Putamen Feedback ~ PDS 68
Female participants 68
Male participants 68
2.8 Model: Accumbens Feedback ~ PDS 69
Female participants 69
Male participants 69
2.9 Model: OFC Anticipation ~ PDS 70
Female participants 70
Male participants 71
2.10 Model: OFC Feedback ~ PDS 72
Female participants 72
Male participants 73
2.11 Model: Caudate Anticipation ~ Testosterone 74
Female participants 74
Male participants 75
2.12 Model B: Putamen Anticipation ~ Testosterone 75
Female participants 75

Male participants 76
2.13 Model: Accumbens Anticipation ~ Testosterone 77
Female participants 77
Male participants 77
2.14 Model: Caudate Feedback ~ Testosterone 78
Female participants 78
Male participants 79
2.15 Model: Putamen Feedback ~ Testosterone 80
Female participants 80
Male participants 80
2.16 Model: Accumbens Feedback ~ Testosterone 81
Female participants 81
Male participants 82
2.17 Model: OFC Anticipation ~ Testosterone 82
Female participants 82
Male participants 84
2.18 Model: OFC Feedback ~ Testosterone 85
Female participants 85
Male participants 86
2.19 Model: MID Reaction Time ~ Testosterone 88
Female participants 88
Male participants 89
2.20 Model: BIS-BAS-RR ~ Testosterone 90
Female participants 90
Male participants 90

3—Internalizing~Reward— **92**
3.1 Model: CBCL internalizing factor ~ Nucleus Accumbens activity (anticipation stage) 92
Female participants 92
Male participants 92
3.2 Model: CBCL internalizing factor ~ Caudate activity (anticipation stage) 93
Female participants 93
Male participants 93
3.3 Model: CBCL internalizing factor ~ Putamen activity (anticipation stage) 94
Female participants 94
Male participants 94
3.4 Model: CBCL internalizing factor ~ Accumbens activity (feedback stage) 95

Female participants 95
Male participants 95
3.5 Model: CBCL internalizing factor ~ Caudate activity (feedback stage) 96
Female participants 96
Male participants 96
3.6 Model: CBCL internalizing factor ~ Putamen activity (feedback stage) 97
Female participants 97
Male participants 97
3.7 Model: CBCL internalizing factor ~ OFC activity (anticipation stage) 98
Female participants 98
Male participants 99
3.8 Model: CBCL internalizing factor ~ OFC activity (feedback stage) 100
Female participants 100
Male participants 101
3.9 Model: CBCL internalizing factor ~ BIS-BAS-RR 101
Female participants 101
Male participants 102
3.10 Model: CBCL internalizing factor ~ MID Reaction Time 102
Female participants 102
Male participants 103

4—Internalizing~Puberty x Reward— **105**
4.1 Model: CBCL internalizing factor ~ PDS x Accumbens activity (anticipation stage) 105
Female participants 105
Male participants 106
4.2 Model: CBCL internalizing factor ~ PDS x Caudate activity (anticipation stage) 107
Female participants 107
Male participants 109
4.3 Model: CBCL internalizing factor ~ PDS x Putamen activity (anticipation stage) 110
Female participants 110
Male participants 111
4.4 Model: CBCL internalizing factor ~ PDS x Lateral OFC activity (anticipation stage) 113
Female participants 113
Male participants 114
4.5 Model: CBCL internalizing factor ~ PDS x Medial OFC activity (anticipation stage) 115
Female participants 115
Male participants 117

4.6 Model: CBCL internalizing factor ~ PDS x Accumbens activity (feedback) 118
Female participants 118
Male participants 120
4.7 Model: CBCL internalizing factor ~ PDS x Caudate activity (feedback) 122
Female participants 122
Male participants 123
4.8 Model: CBCL internalizing factor ~ PDS x Putamen activity (feedback) 124
Female participants 124
Male participants 126
4.9 Model: CBCL internalizing factor ~ PDS x Lateral OFC activity (feedback stage) 127
Female participants 127
Male participants 128
4.10 Model: CBCL internalizing factor ~ PDS x Medial OFC activity (feedback stage) 130
Female participants 130
Male participants 131
4.11 Model: CBCL internalizing factor ~ PDS x BIS-BAS 132
Female participants 132
Male participants 134
4.12 Model: CBCL internalizing factor ~ PDS x MID reaction time (large reward vs. neutral) . . . 135
Female participants 135
Male participants 136
4.13 Model: CBCL internalizing factor ~ PDS x MID reaction time (large vs. small reward) 138
Female participants 138
Male participants 139
4.14 Model: CBCL internalizing factor ~ Testosterone x Accumbens activity (anticipation stage) + PDS 140
Female participants 140
Male participants 142
4.15 Model: CBCL internalizing factor ~ Testosterone x Caudate activity (anticipation stage) + PDS145
Female participants 145
Male participants 147
4.16 Model: CBCL internalizing factor ~ Testosterone x Putamen activity (anticipation stage) + PDS 149
Female participants 149
Male participants 151
4.17 Model: CBCL internalizing factor ~ Testosterone x Accumbens activity (feedback stage) + PDS153
Female participants 153

Male participants 155
4.18 Model: CBCL internalizing factor ~ Testosterone x Caudate activity (Feedback stage) + PDS 157
Female participants 157
Male participants 159
4.19 Model: CBCL internalizing factor ~ Testosterone x Putamen activity (Feedback stage) + PDS 161
Female participants 161
Male participants 163
4.20 Model: CBCL internalizing factor ~ Testosterone x Lateral OFC activity (anticipation stage) + PDS 165
Female participants 165
Male participants 167
4.21 Model: CBCL internalizing factor ~ Testosterone x Medial OFC activity (anticipation stage) + PDS 168
Female participants 168
Male participants 170
4.22 Model: CBCL internalizing factor ~ Testosterone x Lateral OFC activity (feedback stage) + PDS 171
Female participants 171
Male participants 173
4.23 Model: CBCL internalizing factor ~ Testosterone x Medial OFC activity (feedback stage) + PDS 175
Female participants 175
Male participants 177
4.24 Model: CBCL internalizing factor ~ Testosterone x BIS-BAS RR + PDS 179
Female participants 179
Male participants 181
4.25 Model: CBCL internalizing factor ~ Testosterone x MID Reaction Time + PDS (large reward vs. neutral) 183
Female participants 183
Male participants 185
4.26 Model: CBCL internalizing factor ~ Testosterone x MID Reaction Time + PDS (large vs. small reward) 187
Female participants 187
Male participants 189

Sensitivity Analysis Results for Sample 2

1—Internalizing~Puberty—

1.1 Model: CBCL internalizing factor ~ PDS

Female participants

```
##
## Family: gaussian
## Link function: identity
##
## Formula:
## cbcl_scr_syn_internal_r ~ PDS_score + race.ethnicity.5level +
##     interview_age + bmi + household.income + high.educ + demo_race_hispanic
##
## Parametric coefficients:
##                              Estimate Std. Error t value Pr(>|t|)
## (Intercept)                   0.24585    2.14625   0.115 0.908813
## PDS_score                     0.63910    0.16994   3.761 0.000173 ***
## race.ethnicity.5levelBlack   -0.59950    0.80357  -0.746 0.455719
## race.ethnicity.5levelMixed    1.16125    0.78203   1.485 0.137698
## race.ethnicity.5levelOther   -0.07551    0.91532  -0.082 0.934261
## race.ethnicity.5levelWhite    1.16079    0.72966   1.591 0.111774
## interview_age                 0.02398    0.01551   1.546 0.122175
## bmi                           0.02183    0.03081   0.708 0.478737
## household.income[>=200K]     -2.48695    0.84306  -2.950 0.003210 **
## household.income[100K-200K]  -1.53646    0.78498  -1.957 0.050425 .
## household.income[12K-16K]    -0.16447    1.00678  -0.163 0.870247
## household.income[16K-25K]    -1.19402    0.86844  -1.375 0.169291
## household.income[25K-35K]     0.06806    0.82129   0.083 0.933964
## household.income[35K-50K]    -1.23125    0.79766  -1.544 0.122825
## household.income[50K-75K]    -1.17459    0.78183  -1.502 0.133139
## household.income[5K-12K]      0.01842    0.88108   0.021 0.983323
## household.income[75K-100K]   -1.20384    0.79552  -1.513 0.130345
## high.educBachelor             0.71480    0.72727   0.983 0.325782
## high.educHS Diploma/GED       0.57208    0.72972   0.784 0.433137
## high.educPost Graduate Degree 1.07092    0.74080   1.446 0.148410
## high.educSome College         0.98398    0.67857   1.450 0.147167
## demo_race_hispanic1           0.01775    0.35033   0.051 0.959598
## ---
## Signif. codes:  0 '***' 0.001 '**' 0.01 '*' 0.05 '.' 0.1 ' ' 1
##
##
## R-sq.(adj) =  0.0208
## lmer.REML =  14752  Scale est. = 17.681    n = 2393
```

```
##                                      stdcoef      stdse
## X(Intercept)                    0.0000000000 0.00000000
## XPDS_score                      0.0858969556 0.02283994
## Xrace.ethnicity.5levelBlack    -0.0398953423 0.05347634
## Xrace.ethnicity.5levelMixed     0.0704570266 0.04744831
## Xrace.ethnicity.5levelOther    -0.0029141215 0.03532548
```

```
## Xrace.ethnicity.5levelWhite     0.1021486553 0.06420956
## Xinterview_age                  0.0330686575 0.02138618
## Xbmi                            0.0154539404 0.02181381
## Xhousehold.income[>=200K]      -0.1461091791 0.04952977
## Xhousehold.income[100K-200K]   -0.1329916338 0.06794514
## Xhousehold.income[12K-16K]     -0.0045211969 0.02767591
## Xhousehold.income[16K-25K]     -0.0442252598 0.03216609
## Xhousehold.income[25K-35K]      0.0030151619 0.03638550
## Xhousehold.income[35K-50K]     -0.0646205500 0.04186418
## Xhousehold.income[50K-75K]     -0.0745794407 0.04964180
## Xhousehold.income[5K-12K]       0.0006416684 0.03069365
## Xhousehold.income[75K-100K]    -0.0795695287 0.05258123
## Xhigh.educBachelor              0.0583576388 0.05937596
## Xhigh.educHS Diploma/GED        0.0285592657 0.03642909
## Xhigh.educPost Graduate Degree  0.0959137805 0.06634693
## Xhigh.educSome College          0.0785885850 0.05419584
## Xdemo_race_hispanic1            0.0012658639 0.02498598
```

Male participants

```
##
## Family: gaussian
## Link function: identity
##
## Formula:
## cbcl_scr_syn_internal_r ~ PDS_score + race.ethnicity.5level +
##     interview_age + bmi + household.income + high.educ + demo_race_hispanic
##
## Parametric coefficients:
##                                Estimate Std. Error t value Pr(>|t|)
## (Intercept)                    3.153877   2.137007   1.476 0.140111
## PDS_score                      0.514573   0.213455   2.411 0.015993 *
## race.ethnicity.5levelBlack    -0.619868   0.876649  -0.707 0.479576
## race.ethnicity.5levelMixed     1.093012   0.857615   1.274 0.202610
## race.ethnicity.5levelOther     0.005758   0.975858   0.006 0.995293
## race.ethnicity.5levelWhite     0.836153   0.807256   1.036 0.300395
## interview_age                  0.007705   0.014707   0.524 0.600395
## bmi                            0.038066   0.030309   1.256 0.209254
## household.income[>=200K]      -3.161721   0.817944  -3.865 0.000114 ***
## household.income[100K-200K]   -2.502614   0.762334  -3.283 0.001042 **
## household.income[12K-16K]     -0.374511   0.979349  -0.382 0.702191
## household.income[16K-25K]      0.020226   0.819689   0.025 0.980316
## household.income[25K-35K]     -0.075257   0.821517  -0.092 0.927018
## household.income[35K-50K]     -1.121805   0.777926  -1.442 0.149413
## household.income[50K-75K]     -1.608016   0.755315  -2.129 0.033355 *
## household.income[5K-12K]     -0.081566   0.858887  -0.095 0.924349
## household.income[75K-100K]    -2.670305   0.777145  -3.436 0.000600 ***
## high.educBachelor              1.514557   0.769991   1.967 0.049294 *
## high.educHS Diploma/GED       -0.856803   0.763039  -1.123 0.261594
## high.educPost Graduate Degree  0.764688   0.773156   0.989 0.322734
## high.educSome College          0.988420   0.731778   1.351 0.176908
## demo_race_hispanic1            0.143689   0.348732   0.412 0.680350
## ---
```

```
## Signif. codes:  0 '***' 0.001 '**' 0.01 '*' 0.05 '.' 0.1 ' ' 1
##
##
## R-sq.(adj) =  0.0341
## lmer.REML =  15995  Scale est. = 17.372    n = 2569
```

```
##                                      stdcoef      stdse
## X(Intercept)                    0.0000000000 0.00000000
## XPDS_score                      0.0501268630 0.02079361
## Xrace.ethnicity.5levelBlack    -0.0362801371 0.05130919
## Xrace.ethnicity.5levelMixed     0.0637975146 0.05005773
## Xrace.ethnicity.5levelOther     0.0002159450 0.03659994
## Xrace.ethnicity.5levelWhite     0.0689975585 0.06661302
## Xinterview_age                  0.0103560104 0.01976723
## Xbmi                            0.0256333593 0.02040975
## Xhousehold.income[>=200K]      -0.1835245307 0.04747820
## Xhousehold.income[100K-200K]   -0.2080418970 0.06337267
## Xhousehold.income[12K-16K]     -0.0096730945 0.02529523
## Xhousehold.income[16K-25K]      0.0007585704 0.03074276
## Xhousehold.income[25K-35K]     -0.0029754039 0.03248017
## Xhousehold.income[35K-50K]     -0.0557839022 0.03868390
## Xhousehold.income[50K-75K]     -0.1000650516 0.04700242
## Xhousehold.income[5K-12K]      -0.0026327267 0.02772259
## Xhousehold.income[75K-100K]    -0.1700805605 0.04949893
## Xhigh.educBachelor              0.1188363857 0.06041566
## Xhigh.educHS Diploma/GED       -0.0404926101 0.03606134
## Xhigh.educPost Graduate Degree  0.0659720057 0.06670259
## Xhigh.educSome College          0.0773674151 0.05727903
## Xdemo_race_hispanic1            0.0100657488 0.02442952
```

1.2 Model: CBCL Anxious-Depressed ~ PDS

Female participants

```
##
## Family: gaussian
## Link function: identity
##
## Formula:
## cbcl_scr_syn_anxdep_r ~ PDS_score + race.ethnicity.5level + interview_age +
##     bmi + household.income + high.educ + demo_race_hispanic
##
## Parametric coefficients:
##                                Estimate Std. Error t value Pr(>|t|)
## (Intercept)                    0.571226   1.208616   0.473   0.6365
## PDS_score                      0.289623   0.095677   3.027   0.0025 **
## race.ethnicity.5levelBlack    -0.208973   0.450502  -0.464   0.6428
## race.ethnicity.5levelMixed     0.779587   0.438563   1.778   0.0756 .
## race.ethnicity.5levelOther     0.149325   0.513557   0.291   0.7713
## race.ethnicity.5levelWhite     0.701081   0.409125   1.714   0.0867 .
## interview_age                  0.009063   0.008759   1.035   0.3009
## bmi                           -0.010226   0.017333  -0.590   0.5552
## household.income[>=200K]      -0.915799   0.472779  -1.937   0.0529 .
```

```
## household.income[100K-200K]   -0.373934   0.440145  -0.850   0.3957
## household.income[12K-16K]     -0.026541   0.564057  -0.047   0.9625
## household.income[16K-25K]     -0.526282   0.487490  -1.080   0.2804
## household.income[25K-35K]      0.199691   0.460614   0.434   0.6647
## household.income[35K-50K]     -0.325213   0.447435  -0.727   0.4674
## household.income[50K-75K]     -0.226284   0.438393  -0.516   0.6058
## household.income[5K-12K]       0.123800   0.494970   0.250   0.8025
## household.income[75K-100K]    -0.200656   0.446142  -0.450   0.6529
## high.educBachelor              0.149894   0.407184   0.368   0.7128
## high.educHS Diploma/GED       -0.047246   0.408926  -0.116   0.9080
## high.educPost Graduate Degree  0.557669   0.414778   1.344   0.1789
## high.educSome College          0.379274   0.379997   0.998   0.3183
## demo_race_hispanic1            0.127401   0.195756   0.651   0.5152
## ---
## Signif. codes:  0 '***' 0.001 '**' 0.01 '*' 0.05 '.' 0.1 ' ' 1
##
##
## R-sq.(adj) =  0.0156
## lmer.REML =  12037  Scale est. = 6.6943    n = 2393
```

```
##                                    stdcoef      stdse
## X(Intercept)                    0.000000000 0.00000000
## XPDS_score                      0.069359931 0.02291317
## Xrace.ethnicity.5levelBlack    -0.024779475 0.05341929
## Xrace.ethnicity.5levelMixed     0.084280830 0.04741279
## Xrace.ethnicity.5levelOther     0.010268662 0.03531593
## Xrace.ethnicity.5levelWhite     0.109929192 0.06415064
## Xinterview_age                  0.022268642 0.02151971
## Xbmi                           -0.012900425 0.02186513
## Xhousehold.income[>=200K]      -0.095868389 0.04949182
## Xhousehold.income[100K-200K]   -0.057671691 0.06788344
## Xhousehold.income[12K-16K]     -0.001300012 0.02762839
## Xhousehold.income[16K-25K]     -0.034732893 0.03217275
## Xhousehold.income[25K-35K]      0.015763630 0.03636097
## Xhousehold.income[35K-50K]     -0.030412850 0.04184265
## Xhousehold.income[50K-75K]     -0.025600843 0.04959792
## Xhousehold.income[5K-12K]       0.007684532 0.03072389
## Xhousehold.income[75K-100K]    -0.023631730 0.05254320
## Xhigh.educBachelor              0.021805335 0.05923371
## Xhigh.educHS Diploma/GED       -0.004202619 0.03637494
## Xhigh.educPost Graduate Degree  0.088994823 0.06619178
## Xhigh.educSome College          0.053974901 0.05407771
## Xdemo_race_hispanic1            0.016190376 0.02487704
```

Male participants

```
##
## Family: gaussian
## Link function: identity
##
## Formula:
## cbcl_scr_syn_anxdep_r ~ PDS_score + race.ethnicity.5level + interview_age +
##     bmi + household.income + high.educ + demo_race_hispanic
```

```
##
## Parametric coefficients:
##                             Estimate Std. Error t value Pr(>|t|)
## (Intercept)                 2.333740   1.202275   1.941  0.05236 .
## PDS_score                   0.267563   0.120169   2.227  0.02606 *
## race.ethnicity.5levelBlack -0.176229   0.491482  -0.359  0.71995
## race.ethnicity.5levelMixed  0.580194   0.480731   1.207  0.22758
## race.ethnicity.5levelOther  0.238373   0.547966   0.435  0.66359
## race.ethnicity.5levelWhite  0.572274   0.452749   1.264  0.20635
## interview_age              -0.007035   0.008294  -0.848  0.39640
## bmi                         0.005614   0.017055   0.329  0.74206
## household.income[>=200K]   -1.282161   0.457120  -2.805  0.00507 **
## household.income[100K-200K] -0.936176  0.426220  -2.196  0.02815 *
## household.income[12K-16K]  -0.096356   0.547878  -0.176  0.86041
## household.income[16K-25K]  -0.012544   0.458713  -0.027  0.97819
## household.income[25K-35K]   0.060759   0.460008   0.132  0.89493
## household.income[35K-50K]  -0.271776   0.435075  -0.625  0.53225
## household.income[50K-75K]  -0.689185   0.422468  -1.631  0.10294
## household.income[5K-12K]    0.050973   0.480615   0.106  0.91555
## household.income[75K-100K] -0.953654   0.434445  -2.195  0.02825 *
## high.educBachelor           1.194694   0.431171   2.771  0.00563 **
## high.educHS Diploma/GED    -0.190006   0.427558  -0.444  0.65679
## high.educPost Graduate Degree  0.857192   0.432865   1.980  0.04778 *
## high.educSome College       0.730735   0.410062   1.782  0.07487 .
## demo_race_hispanic1         0.179448   0.195072   0.920  0.35771
## ---
## Signif. codes:  0 '***' 0.001 '**' 0.01 '*' 0.05 '.' 0.1 ' ' 1
##
##
## R-sq.(adj) =  0.0183
## lmer.REML =  13073  Scale est. = 7.132     n = 2569

##                                      stdcoef      stdse
## X(Intercept)                    0.0000000000 0.00000000
## XPDS_score                      0.0468631813 0.02104745
## Xrace.ethnicity.5levelBlack    -0.0185451581 0.05172010
## Xrace.ethnicity.5levelMixed     0.0608884820 0.05045026
## Xrace.ethnicity.5levelOther     0.0160743353 0.03695133
## Xrace.ethnicity.5levelWhite     0.0849052949 0.06717196
## Xinterview_age                 -0.0170015444 0.02004399
## Xbmi                            0.0067968208 0.02064873
## Xhousehold.income[>=200K]      -0.1338122873 0.04770717
## Xhousehold.income[100K-200K]   -0.1399256599 0.06370505
## Xhousehold.income[12K-16K]     -0.0044747020 0.02544297
## Xhousehold.income[16K-25K]     -0.0008458904 0.03093269
## Xhousehold.income[25K-35K]      0.0043191259 0.03270012
## Xhousehold.income[35K-50K]     -0.0242988139 0.03889901
## Xhousehold.income[50K-75K]     -0.0771100579 0.04726814
## Xhousehold.income[5K-12K]       0.0029581431 0.02789186
## Xhousehold.income[75K-100K]    -0.1092112870 0.04975207
## Xhigh.educBachelor              0.1685401813 0.06082698
## Xhigh.educHS Diploma/GED       -0.0161452782 0.03633062
## Xhigh.educPost Graduate Degree  0.1329648169 0.06714457
## Xhigh.educSome College          0.1028392442 0.05770974
```

```
## Xdemo_race_hispanic1           0.0226018647 0.02456973
```

1.3 Model: CBCL Withdrawn-Depressed ~ PDS

Female participants

```
##
## Family: gaussian
## Link function: identity
##
## Formula:
## cbcl_scr_syn_withdep_r ~ PDS_score + race.ethnicity.5level +
##     interview_age + bmi + household.income + high.educ + demo_race_hispanic
##
## Parametric coefficients:
##                               Estimate Std. Error t value Pr(>|t|)
## (Intercept)                   0.597114   0.631420   0.946 0.344413
## PDS_score                     0.167783   0.049930   3.360 0.000791 ***
## race.ethnicity.5levelBlack   -0.405523   0.234501  -1.729 0.083884 .
## race.ethnicity.5levelMixed   -0.037332   0.228166  -0.164 0.870046
## race.ethnicity.5levelOther   -0.296675   0.267095  -1.111 0.266789
## race.ethnicity.5levelWhite   -0.075008   0.213035  -0.352 0.724800
## interview_age                 0.003785   0.004588   0.825 0.409541
## bmi                           0.010756   0.009034   1.191 0.233957
## household.income[>=200K]     -0.790678   0.245498  -3.221 0.001296 **
## household.income[100K-200K]  -0.567993   0.228434  -2.486 0.012970 *
## household.income[12K-16K]    -0.250379   0.292350  -0.856 0.391845
## household.income[16K-25K]    -0.358819   0.253532  -1.415 0.157117
## household.income[25K-35K]    -0.008874   0.239098  -0.037 0.970398
## household.income[35K-50K]    -0.527085   0.232381  -2.268 0.023407 *
## household.income[50K-75K]    -0.477302   0.227514  -2.098 0.036019 *
## household.income[5K-12K]     -0.047157   0.257508  -0.183 0.854714
## household.income[75K-100K]   -0.483750   0.231611  -2.089 0.036848 *
## high.educBachelor            -0.025672   0.210861  -0.122 0.903110
## high.educHS Diploma/GED       0.204823   0.212147   0.965 0.334405
## high.educPost Graduate Degree -0.006554   0.214832  -0.031 0.975666
## high.educSome College        -0.004270   0.196842  -0.022 0.982694
## demo_race_hispanic1          -0.004556   0.101595  -0.045 0.964235
## ---
## Signif. codes:  0 '***' 0.001 '**' 0.01 '*' 0.05 '.' 0.1 ' ' 1
##
##
## R-sq.(adj) =  0.0224
## lmer.REML =   8972  Scale est. = 2.2943    n = 2393
```

```
##                                     stdcoef      stdse
## X(Intercept)                    0.000000000 0.00000000
## XPDS_score                      0.076640589 0.02280740
## Xrace.ethnicity.5levelBlack    -0.091717622 0.05303723
## Xrace.ethnicity.5levelMixed    -0.007698039 0.04704908
## Xrace.ethnicity.5levelOther    -0.038913410 0.03503350
## Xrace.ethnicity.5levelWhite    -0.022433095 0.06371354
## Xinterview_age                  0.017737127 0.02150353
```

```
## Xbmi                             0.025879703 0.02173793
## Xhousehold.income[>=200K]       -0.157874098 0.04901836
## Xhousehold.income[100K-200K]    -0.167088747 0.06719942
## Xhousehold.income[12K-16K]      -0.023391914 0.02731317
## Xhousehold.income[16K-25K]      -0.045168323 0.03191474
## Xhousehold.income[25K-35K]      -0.001336075 0.03600067
## Xhousehold.income[35K-50K]      -0.094016860 0.04145020
## Xhousehold.income[50K-75K]      -0.102998086 0.04909572
## Xhousehold.income[5K-12K]       -0.005583114 0.03048761
## Xhousehold.income[75K-100K]     -0.108667569 0.05202825
## Xhigh.educBachelor              -0.007123087 0.05850750
## Xhigh.educHS Diploma/GED         0.034751307 0.03599393
## Xhigh.educPost Graduate Degree -0.001994812 0.06539168
## Xhigh.educSome College          -0.001159097 0.05343082
## Xdemo_race_hispanic1            -0.001104318 0.02462576
```

Male participants

```
##
## Family: gaussian
## Link function: identity
##
## Formula:
## cbcl_scr_syn_withdep_r ~ PDS_score + race.ethnicity.5level +
##     interview_age + bmi + household.income + high.educ + demo_race_hispanic
##
## Parametric coefficients:
##                                 Estimate Std. Error t value Pr(>|t|)
## (Intercept)                     0.408095   0.694164   0.588 0.556655
## PDS_score                       0.128173   0.069601   1.842 0.065661 .
## race.ethnicity.5levelBlack     -0.168999   0.284411  -0.594 0.552427
## race.ethnicity.5levelMixed      0.249080   0.278540   0.894 0.371283
## race.ethnicity.5levelOther     -0.018398   0.317402  -0.058 0.953783
## race.ethnicity.5levelWhite      0.085257   0.261940   0.325 0.744841
## interview_age                   0.010816   0.004786   2.260 0.023929 *
## bmi                             0.001416   0.009881   0.143 0.886039
## household.income[>=200K]       -1.041422   0.265104  -3.928 8.78e-05 ***
## household.income[100K-200K]    -0.858382   0.247453  -3.469 0.000531 ***
## household.income[12K-16K]       0.046525   0.318434   0.146 0.883850
## household.income[16K-25K]       0.061961   0.266491   0.233 0.816164
## household.income[25K-35K]      -0.074734   0.267179  -0.280 0.779720
## household.income[35K-50K]      -0.455497   0.252901  -1.801 0.071808 .
## household.income[50K-75K]      -0.596437   0.245477  -2.430 0.015180 *
## household.income[5K-12K]       -0.029847   0.279416  -0.107 0.914942
## household.income[75K-100K]     -0.931872   0.252411  -3.692 0.000227 ***
## high.educBachelor               0.001744   0.250045   0.007 0.994435
## high.educHS Diploma/GED        -0.576591   0.247838  -2.326 0.020071 *
## high.educPost Graduate Degree -0.253336   0.251114  -1.009 0.313143
## high.educSome College          -0.109464   0.237591  -0.461 0.645034
## demo_race_hispanic1            -0.063744   0.110151  -0.579 0.562846
## ---
## Signif. codes:  0 '***' 0.001 '**' 0.01 '*' 0.05 '.' 0.1 ' ' 1
##
```

```
##
## R-sq.(adj) =  0.0369
## lmer.REML =  10286  Scale est. = 2.085     n = 2569
```

```
##                                     stdcoef      stdse
## X(Intercept)                     0.0000000000 0.00000000
## XPDS_score                       0.0384618415 0.02088585
## Xrace.ethnicity.5levelBlack     -0.0304694738 0.05127758
## Xrace.ethnicity.5levelMixed      0.0447845495 0.05008163
## Xrace.ethnicity.5levelOther     -0.0021255150 0.03667026
## Xrace.ethnicity.5levelWhite      0.0216715854 0.06658259
## Xinterview_age                   0.0447804938 0.01981775
## Xbmi                             0.0029377917 0.02049636
## Xhousehold.income[>=200K]       -0.1862123890 0.04740219
## Xhousehold.income[100K-200K]    -0.2198107481 0.06336677
## Xhousehold.income[12K-16K]       0.0037016688 0.02533564
## Xhousehold.income[16K-25K]       0.0071584658 0.03078838
## Xhousehold.income[25K-35K]      -0.0091018424 0.03253986
## Xhousehold.income[35K-50K]      -0.0697731763 0.03873949
## Xhousehold.income[50K-75K]      -0.1143320498 0.04705584
## Xhousehold.income[5K-12K]       -0.0029675861 0.02778183
## Xhousehold.income[75K-100K]     -0.1828359245 0.04952367
## Xhigh.educBachelor               0.0004215489 0.06043572
## Xhigh.educHS Diploma/GED        -0.0839409517 0.03608062
## Xhigh.educPost Graduate Degree -0.0673261868 0.06673571
## Xhigh.educSome College          -0.0263937134 0.05728717
## Xdemo_race_hispanic1            -0.0137553757 0.02376965
```

1.4 Model: CBCL Depressed DSM-5 ~ PDS

Female participants

```
##
## Family: gaussian
## Link function: identity
##
## Formula:
## cbcl_scr_dsm5_depress_r ~ PDS_score + race.ethnicity.5level +
##     interview_age + bmi + household.income + high.educ + demo_race_hispanic
##
## Parametric coefficients:
##                                Estimate Std. Error t value Pr(>|t|)
## (Intercept)                   1.2070529  0.7372460   1.637   0.1017
## PDS_score                     0.1130118  0.0584081   1.935   0.0531 .
## race.ethnicity.5levelBlack   -0.1890686  0.2748074  -0.688   0.4915
## race.ethnicity.5levelMixed    0.1951616  0.2676648   0.729   0.4660
## race.ethnicity.5levelOther   -0.2411360  0.3135123  -0.769   0.4419
## race.ethnicity.5levelWhite    0.2112751  0.2495603   0.847   0.3973
## interview_age                 0.0006124  0.0053434   0.115   0.9088
## bmi                           0.0034458  0.0105839   0.326   0.7448
## household.income[>=200K]     -0.7191737  0.2887547  -2.491   0.0128 *
## household.income[100K-200K]  -0.5574300  0.2688546  -2.073   0.0382 *
## household.income[12K-16K]     0.0074610  0.3445896   0.022   0.9827
```

```
## household.income[16K-25K]     -0.4500986  0.2977632  -1.512   0.1308
## household.income[25K-35K]     -0.0392555  0.2813844  -0.140   0.8891
## household.income[35K-50K]     -0.3336823  0.2732968  -1.221   0.2222
## household.income[50K-75K]     -0.4432217  0.2677925  -1.655   0.0980 .
## household.income[5K-12K]       0.1637256  0.3023181   0.542   0.5882
## household.income[75K-100K]    -0.4576971  0.2725190  -1.680   0.0932 .
## high.educBachelor             -0.2115200  0.2487655  -0.850   0.3953
## high.educHS Diploma/GED       -0.1026540  0.2497984  -0.411   0.6811
## high.educPost Graduate Degree -0.0302769  0.2533932  -0.119   0.9049
## high.educSome College         -0.0896484  0.2321673  -0.386   0.6994
## demo_race_hispanic1           -0.0097103  0.1192277  -0.081   0.9351
## ---
## Signif. codes:  0 '***' 0.001 '**' 0.01 '*' 0.05 '.' 0.1 ' ' 1
##
##
## R-sq.(adj) =  0.0104
## lmer.REML = 9694.8  Scale est. = 2.4385    n = 2393
```

```
##                                    stdcoef      stdse
## X(Intercept)                    0.0000000000 0.00000000
## XPDS_score                      0.0445728494 0.02303665
## Xrace.ethnicity.5levelBlack    -0.0369225047 0.05366610
## Xrace.ethnicity.5levelMixed     0.0347479419 0.04765692
## Xrace.ethnicity.5levelOther    -0.0273095536 0.03550644
## Xrace.ethnicity.5levelWhite     0.0545587163 0.06444531
## Xinterview_age                  0.0024778775 0.02162188
## Xbmi                            0.0071587457 0.02198857
## Xhousehold.income[>=200K]      -0.1239880580 0.04978231
## Xhousehold.income[100K-200K]   -0.1415887716 0.06828982
## Xhousehold.income[12K-16K]      0.0006018683 0.02779748
## Xhousehold.income[16K-25K]     -0.0489216348 0.03236416
## Xhousehold.income[25K-35K]     -0.0051035235 0.03658215
## Xhousehold.income[35K-50K]     -0.0513917052 0.04209150
## Xhousehold.income[50K-75K]     -0.0825831326 0.04989635
## Xhousehold.income[5K-12K]       0.0167372683 0.03090525
## Xhousehold.income[75K-100K]    -0.0887752885 0.05285800
## Xhigh.educBachelor             -0.0506758357 0.05959908
## Xhigh.educHS Diploma/GED       -0.0150384786 0.03659465
## Xhigh.educPost Graduate Degree -0.0079573691 0.06659683
## Xhigh.educSome College         -0.0210112257 0.05441392
## Xdemo_race_hispanic1           -0.0020322978 0.02495345
```

Male participants

```
##
## Family: gaussian
## Link function: identity
##
## Formula:
## cbcl_scr_dsm5_depress_r ~ PDS_score + race.ethnicity.5level +
##     interview_age + bmi + household.income + high.educ + demo_race_hispanic
##
## Parametric coefficients:
```

```
##                               Estimate Std. Error t value Pr(>|t|)
## (Intercept)                   0.851071   0.815348   1.044 0.296671
## PDS_score                     0.090309   0.081534   1.108 0.268129
## race.ethnicity.5levelBlack   -0.142114   0.334429  -0.425 0.670912
## race.ethnicity.5levelMixed    0.265217   0.327275   0.810 0.417796
## race.ethnicity.5levelOther   -0.017185   0.372525  -0.046 0.963208
## race.ethnicity.5levelWhite    0.166805   0.307970   0.542 0.588122
## interview_age                 0.007236   0.005614   1.289 0.197594
## bmi                           0.001601   0.011577   0.138 0.890035
## household.income[>=200K]     -1.154639   0.312000  -3.701 0.000220 ***
## household.income[100K-200K]  -1.060968   0.290907  -3.647 0.000271 ***
## household.income[12K-16K]     0.071333   0.373882   0.191 0.848706
## household.income[16K-25K]    -0.393897   0.312912  -1.259 0.208214
## household.income[25K-35K]    -0.314674   0.313637  -1.003 0.315807
## household.income[35K-50K]    -0.667159   0.296976  -2.247 0.024757 *
## household.income[50K-75K]    -0.744978   0.288320  -2.584 0.009825 **
## household.income[5K-12K]     -0.161025   0.327938  -0.491 0.623453
## household.income[75K-100K]   -0.973878   0.296607  -3.283 0.001040 **
## high.educBachelor             0.421055   0.293843   1.433 0.152002
## high.educHS Diploma/GED      -0.332825   0.291197  -1.143 0.253164
## high.educPost Graduate Degree  0.094430   0.295066   0.320 0.748971
## high.educSome College         0.381811   0.279236   1.367 0.171639
## demo_race_hispanic1          -0.056162   0.132099  -0.425 0.670762
## ---
## Signif. codes:  0 '***' 0.001 '**' 0.01 '*' 0.05 '.' 0.1 ' ' 1
##
##
## R-sq.(adj) =  0.0247
## lmer.REML =  11092  Scale est. = 2.5914    n = 2569

##                                    stdcoef      stdse
## X(Intercept)                    0.000000000 0.00000000
## XPDS_score                      0.023231845 0.02097449
## Xrace.ethnicity.5levelBlack    -0.021965279 0.05168951
## Xrace.ethnicity.5levelMixed     0.040879958 0.05044531
## Xrace.ethnicity.5levelOther    -0.001702091 0.03689594
## Xrace.ethnicity.5levelWhite     0.036348554 0.06710975
## Xinterview_age                  0.025682829 0.01992811
## Xbmi                            0.002846536 0.02058632
## Xhousehold.income[>=200K]      -0.176988993 0.04782494
## Xhousehold.income[100K-200K]   -0.232910161 0.06386166
## Xhousehold.income[12K-16K]      0.004865407 0.02550147
## Xhousehold.income[16K-25K]     -0.039012697 0.03099171
## Xhousehold.income[25K-35K]     -0.032854289 0.03274596
## Xhousehold.income[35K-50K]     -0.087609383 0.03899808
## Xhousehold.income[50K-75K]     -0.122423478 0.04738011
## Xhousehold.income[5K-12K]      -0.013725249 0.02795239
## Xhousehold.income[75K-100K]    -0.163805276 0.04988905
## Xhigh.educBachelor              0.087243276 0.06088474
## Xhigh.educHS Diploma/GED       -0.041537529 0.03634219
## Xhigh.educPost Graduate Degree  0.021513739 0.06722399
## Xhigh.educSome College          0.078921228 0.05771870
## Xdemo_race_hispanic1           -0.010389556 0.02443730
```

1.5 Model: CBCL internalizing factor ~ Pubertal category

Female participants

```
##
## Family: gaussian
## Link function: identity
##
## Formula:
## cbcl_scr_syn_internal_r ~ pds_p_ss_category + race.ethnicity.5level +
##     interview_age + bmi + household.income + high.educ + demo_race_hispanic
##
## Parametric coefficients:
##                               Estimate Std. Error t value Pr(>|t|)
## (Intercept)                   0.515354   2.220846   0.232  0.81652
## pds_p_ss_categoryEarly        0.181819   0.304480   0.597  0.55047
## pds_p_ss_categoryLate         0.690550   0.770801   0.896  0.37040
## pds_p_ss_categoryMid          0.756328   0.295630   2.558  0.01058 *
## race.ethnicity.5levelBlack   -0.489177   0.804046  -0.608  0.54298
## race.ethnicity.5levelMixed    1.186664   0.783467   1.515  0.13000
## race.ethnicity.5levelOther   -0.048921   0.916861  -0.053  0.95745
## race.ethnicity.5levelWhite    1.170397   0.730940   1.601  0.10946
## interview_age                 0.028234   0.015801   1.787  0.07409 .
## bmi                           0.018041   0.032035   0.563  0.57337
## household.income[>=200K]     -2.484994   0.845716  -2.938  0.00333 **
## household.income[100K-200K]  -1.530520   0.787420  -1.944  0.05205 .
## household.income[12K-16K]    -0.296461   1.008385  -0.294  0.76879
## household.income[16K-25K]    -1.203397   0.870334  -1.383  0.16689
## household.income[25K-35K]    -0.007737   0.823128  -0.009  0.99250
## household.income[35K-50K]    -1.280262   0.799148  -1.602  0.10928
## household.income[50K-75K]    -1.178881   0.783712  -1.504  0.13266
## household.income[5K-12K]      0.022507   0.883395   0.025  0.97968
## household.income[75K-100K]   -1.221179   0.797590  -1.531  0.12588
## high.educBachelor             0.660875   0.729065   0.906  0.36478
## high.educHS Diploma/GED       0.577993   0.731066   0.791  0.42925
## high.educPost Graduate Degree  1.021730   0.742951   1.375  0.16919
## high.educSome College         1.003400   0.680385   1.475  0.14041
## demo_race_hispanic1          -0.030365   0.350722  -0.087  0.93101
## ---
## Signif. codes:  0 '***' 0.001 '**' 0.01 '*' 0.05 '.' 0.1 ' ' 1
##
##
## R-sq.(adj) =  0.0177
## lmer.REML =  14757  Scale est. = 17.826    n = 2393
```

```
##                                      stdcoef      stdse
## X(Intercept)                    0.0000000000 0.00000000
## Xpds_p_ss_categoryEarly         0.0142308189 0.02383139
## Xpds_p_ss_categoryLate          0.0196565564 0.02194092
## Xpds_p_ss_categoryMid           0.0690944061 0.02700731
## Xrace.ethnicity.5levelBlack    -0.0325537778 0.05350772
## Xrace.ethnicity.5levelMixed     0.0719989748 0.04753565
## Xrace.ethnicity.5levelOther    -0.0018880452 0.03538506
## Xrace.ethnicity.5levelWhite     0.1029942426 0.06432230
```

```
## Xinterview_age                  0.0389323247 0.02178818
## Xbmi                            0.0127728554 0.02267986
## Xhousehold.income[>=200K]      -0.1459941203 0.04968608
## Xhousehold.income[100K-200K]   -0.1324772737 0.06815678
## Xhousehold.income[12K-16K]     -0.0081495718 0.02772002
## Xhousehold.income[16K-25K]     -0.0445724063 0.03223615
## Xhousehold.income[25K-35K]     -0.0003427720 0.03646705
## Xhousehold.income[35K-50K]     -0.0671927863 0.04194218
## Xhousehold.income[50K-75K]     -0.0748521380 0.04976120
## Xhousehold.income[5K-12K]       0.0007840619 0.03077421
## Xhousehold.income[75K-100K]    -0.0807155274 0.05271786
## Xhigh.educBachelor              0.0539551250 0.05952225
## Xhigh.educHS Diploma/GED        0.0288545448 0.03649626
## Xhigh.educPost Graduate Degree  0.0915079001 0.06654002
## Xhigh.educSome College          0.0801395833 0.05434097
## Xdemo_race_hispanic1           -0.0021656954 0.02501383
```

Male participants

```
##
## Family: gaussian
## Link function: identity
##
## Formula:
## cbcl_scr_syn_internal_r ~ pds_p_ss_category + race.ethnicity.5level +
##     interview_age + bmi + household.income + high.educ + demo_race_hispanic
##
## Parametric coefficients:
##                                 Estimate Std. Error t value Pr(>|t|)
## (Intercept)                     3.341790   2.141654   1.560 0.118795
## pds_p_ss_categoryEarly          0.336956   0.269998   1.248 0.212149
## pds_p_ss_categoryLate          -0.437416   1.620316  -0.270 0.787215
## pds_p_ss_categoryMid            1.315482   0.519604   2.532 0.011411 *
## race.ethnicity.5levelBlack     -0.546024   0.875812  -0.623 0.533046
## race.ethnicity.5levelMixed      1.173093   0.858244   1.367 0.171792
## race.ethnicity.5levelOther      0.057896   0.976330   0.059 0.952718
## race.ethnicity.5levelWhite      0.933042   0.807994   1.155 0.248296
## interview_age                   0.009916   0.014655   0.677 0.498723
## bmi                             0.041057   0.030212   1.359 0.174286
## household.income[>=200K]       -3.203710   0.819200  -3.911 9.44e-05 ***
## household.income[100K-200K]    -2.542325   0.763660  -3.329 0.000884 ***
## household.income[12K-16K]      -0.454010   0.981553  -0.463 0.643732
## household.income[16K-25K]      -0.012437   0.821120  -0.015 0.987917
## household.income[25K-35K]      -0.088994   0.824601  -0.108 0.914064
## household.income[35K-50K]      -1.155246   0.779254  -1.483 0.138330
## household.income[50K-75K]      -1.630575   0.756133  -2.156 0.031141 *
## household.income[5K-12K]       -0.114249   0.859707  -0.133 0.894289
## household.income[75K-100K]     -2.716858   0.778160  -3.491 0.000489 ***
## high.educBachelor               1.512679   0.770483   1.963 0.049722 *
## high.educHS Diploma/GED        -0.896291   0.764117  -1.173 0.240915
## high.educPost Graduate Degree   0.763686   0.773618   0.987 0.323657
## high.educSome College           0.962480   0.732667   1.314 0.189077
## demo_race_hispanic1             0.114078   0.349170   0.327 0.743913
```

```
## ---
## Signif. codes:  0 '***' 0.001 '**' 0.01 '*' 0.05 '.' 0.1 ' ' 1
##
##
## R-sq.(adj) =  0.034
## lmer.REML =  15990  Scale est. = 17.353    n = 2569
```

```
##                                     stdcoef      stdse
## X(Intercept)                     0.000000000 0.00000000
## Xpds_p_ss_categoryEarly          0.025214991 0.02020444
## Xpds_p_ss_categoryLate          -0.005322162 0.01971482
## Xpds_p_ss_categoryMid            0.052009967 0.02054348
## Xrace.ethnicity.5levelBlack     -0.031958093 0.05126022
## Xrace.ethnicity.5levelMixed      0.068471742 0.05009445
## Xrace.ethnicity.5levelOther      0.002171416 0.03661764
## Xrace.ethnicity.5levelWhite      0.076992637 0.06667397
## Xinterview_age                   0.013327499 0.01969782
## Xbmi                             0.027647472 0.02034488
## Xhousehold.income[>=200K]       -0.185961807 0.04755110
## Xhousehold.income[100K-200K]    -0.211343013 0.06348289
## Xhousehold.income[12K-16K]      -0.011726454 0.02535215
## Xhousehold.income[16K-25K]      -0.000466441 0.03079644
## Xhousehold.income[25K-35K]      -0.003518552 0.03260209
## Xhousehold.income[35K-50K]      -0.057446858 0.03874992
## Xhousehold.income[50K-75K]      -0.101468899 0.04705331
## Xhousehold.income[5K-12K]       -0.003687653 0.02774904
## Xhousehold.income[75K-100K]     -0.173045711 0.04956360
## Xhigh.educBachelor               0.118689023 0.06045423
## Xhigh.educHS Diploma/GED        -0.042358800 0.03611225
## Xhigh.educPost Graduate Degree   0.065885606 0.06674247
## Xhigh.educSome College           0.075336965 0.05734861
## Xdemo_race_hispanic1             0.007991434 0.02446023
```

1.6 Model: CBCL Anxious-Depressed ~ Pubertal category

Female participants

```
##
## Family: gaussian
## Link function: identity
##
## Formula:
## cbcl_scr_syn_anxdep_r ~ pds_p_ss_category + race.ethnicity.5level +
##     interview_age + bmi + household.income + high.educ + demo_race_hispanic
##
## Parametric coefficients:
##                              Estimate Std. Error t value Pr(>|t|)
## (Intercept)                  0.712308   1.249598   0.570   0.5687
## pds_p_ss_categoryEarly       0.205193   0.171892   1.194   0.2327
## pds_p_ss_categoryLate        0.262515   0.434926   0.604   0.5462
## pds_p_ss_categoryMid         0.375537   0.166525   2.255   0.0242 *
## race.ethnicity.5levelBlack  -0.154278   0.450501  -0.342   0.7320
## race.ethnicity.5levelMixed   0.789507   0.439111   1.798   0.0723 .
```

```
## race.ethnicity.5levelOther     0.159298   0.514129   0.310   0.7567
## race.ethnicity.5levelWhite     0.704038   0.409586   1.719   0.0858 .
## interview_age                  0.010598   0.008914   1.189   0.2346
## bmi                           -0.012694   0.018014  -0.705   0.4811
## household.income[>=200K]      -0.918478   0.473990  -1.938   0.0528 .
## household.income[100K-200K]   -0.374602   0.441262  -0.849   0.3960
## household.income[12K-16K]     -0.101662   0.564628  -0.180   0.8571
## household.income[16K-25K]     -0.521964   0.488287  -1.069   0.2852
## household.income[25K-35K]      0.161469   0.461399   0.350   0.7264
## household.income[35K-50K]     -0.344987   0.448011  -0.770   0.4414
## household.income[50K-75K]     -0.230054   0.439195  -0.524   0.6005
## household.income[5K-12K]       0.129438   0.495970   0.261   0.7941
## household.income[75K-100K]    -0.209103   0.447042  -0.468   0.6400
## high.educBachelor              0.128066   0.407970   0.314   0.7536
## high.educHS Diploma/GED       -0.040225   0.409457  -0.098   0.9218
## high.educPost Graduate Degree  0.537975   0.415769   1.294   0.1958
## high.educSome College          0.392179   0.380811   1.030   0.3032
## demo_race_hispanic1            0.103386   0.195826   0.528   0.5976
## ---
## Signif. codes:  0 '***' 0.001 '**' 0.01 '*' 0.05 '.' 0.1 ' ' 1
##
##
## R-sq.(adj) =  0.0136
## lmer.REML =  12042  Scale est. = 6.7337    n = 2393
```

```
##                                     stdcoef      stdse
## X(Intercept)                    0.000000000 0.00000000
## Xpds_p_ss_categoryEarly         0.028616651 0.02397244
## Xpds_p_ss_categoryLate          0.013314732 0.02205940
## Xpds_p_ss_categoryMid           0.061129570 0.02710673
## Xrace.ethnicity.5levelBlack    -0.018293806 0.05341911
## Xrace.ethnicity.5levelMixed     0.085353282 0.04747212
## Xrace.ethnicity.5levelOther     0.010954477 0.03535523
## Xrace.ethnicity.5levelWhite     0.110392964 0.06422297
## Xinterview_age                  0.026038142 0.02190216
## Xbmi                           -0.016012845 0.02272385
## Xhousehold.income[>=200K]      -0.096148806 0.04961856
## Xhousehold.income[100K-200K]   -0.057774653 0.06805564
## Xhousehold.income[12K-16K]     -0.004979540 0.02765638
## Xhousehold.income[16K-25K]     -0.034447946 0.03222537
## Xhousehold.income[25K-35K]      0.012746393 0.03642298
## Xhousehold.income[35K-50K]     -0.032262042 0.04189652
## Xhousehold.income[50K-75K]     -0.026027358 0.04968860
## Xhousehold.income[5K-12K]       0.008034517 0.03078597
## Xhousehold.income[75K-100K]    -0.024626499 0.05264923
## Xhigh.educBachelor              0.018630031 0.05934812
## Xhigh.educHS Diploma/GED       -0.003578076 0.03642216
## Xhigh.educPost Graduate Degree  0.085851921 0.06634991
## Xhigh.educSome College          0.055811391 0.05419360
## Xdemo_race_hispanic1            0.013138394 0.02488582
```

Male participants

```
##
```

```
## Family: gaussian
## Link function: identity
##
## Formula:
## cbcl_scr_syn_anxdep_r ~ pds_p_ss_category + race.ethnicity.5level +
##     interview_age + bmi + household.income + high.educ + demo_race_hispanic
##
## Parametric coefficients:
##                              Estimate Std. Error t value Pr(>|t|)
## (Intercept)                  2.422466   1.204786   2.011  0.04446 *
## pds_p_ss_categoryEarly       0.144110   0.151965   0.948  0.34306
## pds_p_ss_categoryLate       -0.071611   0.909260  -0.079  0.93723
## pds_p_ss_categoryMid         0.731536   0.292463   2.501  0.01244 *
## race.ethnicity.5levelBlack  -0.141292   0.491001  -0.288  0.77355
## race.ethnicity.5levelMixed   0.624893   0.481054   1.299  0.19406
## race.ethnicity.5levelOther   0.265797   0.548199   0.485  0.62782
## race.ethnicity.5levelWhite   0.623302   0.453141   1.376  0.16909
## interview_age               -0.005885   0.008265  -0.712  0.47649
## bmi                          0.007322   0.016999   0.431  0.66672
## household.income[>=200K]    -1.296193   0.457751  -2.832  0.00467 **
## household.income[100K-200K] -0.949878   0.426892  -2.225  0.02616 *
## household.income[12K-16K]   -0.132154   0.549044  -0.241  0.80981
## household.income[16K-25K]   -0.018957   0.459464  -0.041  0.96709
## household.income[25K-35K]    0.066068   0.461626   0.143  0.88621
## household.income[35K-50K]   -0.281288   0.435767  -0.645  0.51866
## household.income[50K-75K]   -0.695399   0.422871  -1.644  0.10020
## household.income[5K-12K]     0.038195   0.481025   0.079  0.93672
## household.income[75K-100K]  -0.970095   0.434952  -2.230  0.02581 *
## high.educBachelor            1.196853   0.431415   2.774  0.00557 **
## high.educHS Diploma/GED     -0.210520   0.428124  -0.492  0.62295
## high.educPost Graduate Degree  0.860020   0.433089   1.986  0.04716 *
## high.educSome College        0.719882   0.410528   1.754  0.07963 .
## demo_race_hispanic1          0.162565   0.195304   0.832  0.40528
## ---
## Signif. codes:  0 '***' 0.001 '**' 0.01 '*' 0.05 '.' 0.1 ' ' 1
##
##
## R-sq.(adj) =  0.0182
## lmer.REML =  13070  Scale est. = 7.1372    n = 2569

##                                    stdcoef      stdse
## X(Intercept)                   0.000000000 0.00000000
## Xpds_p_ss_categoryEarly        0.019389344 0.02044622
## Xpds_p_ss_categoryLate        -0.001566595 0.01989135
## Xpds_p_ss_categoryMid          0.052001981 0.02079003
## Xrace.ethnicity.5levelBlack   -0.014868597 0.05166952
## Xrace.ethnicity.5levelMixed    0.065579410 0.05048424
## Xrace.ethnicity.5levelOther    0.017923621 0.03696706
## Xrace.ethnicity.5levelWhite    0.092476000 0.06723015
## Xinterview_age                -0.014222067 0.01997299
## Xbmi                           0.008864696 0.02058159
## Xhousehold.income[>=200K]     -0.135276753 0.04777298
## Xhousehold.income[100K-200K]  -0.141973557 0.06380541
## Xhousehold.income[12K-16K]    -0.006137138 0.02549711
```

```
## Xhousehold.income[16K-25K]     -0.001278311 0.03098331
## Xhousehold.income[25K-35K]      0.004696506 0.03281518
## Xhousehold.income[35K-50K]     -0.025149264 0.03896092
## Xhousehold.income[50K-75K]     -0.077805250 0.04731328
## Xhousehold.income[5K-12K]       0.002216599 0.02791569
## Xhousehold.income[75K-100K]    -0.111094030 0.04981016
## Xhigh.educBachelor              0.168844765 0.06086141
## Xhigh.educHS Diploma/GED       -0.017888420 0.03637868
## Xhigh.educPost Graduate Degree  0.133403460 0.06717928
## Xhigh.educSome College          0.101311983 0.05777534
## Xdemo_race_hispanic1            0.020475393 0.02459902
```

1.7 Model: CBCL Withdrawn-Depressed ~ Pubertal category

Female participants

```
##
## Family: gaussian
## Link function: identity
##
## Formula:
## cbcl_scr_syn_withdep_r ~ pds_p_ss_category + race.ethnicity.5level +
##     interview_age + bmi + household.income + high.educ + demo_race_hispanic
##
## Parametric coefficients:
##                                 Estimate Std. Error t value Pr(>|t|)
## (Intercept)                     0.685287   0.652785   1.050  0.29392
## pds_p_ss_categoryEarly         -0.014723   0.089968  -0.164  0.87002
## pds_p_ss_categoryLate           0.348598   0.227289   1.534  0.12523
## pds_p_ss_categoryMid            0.171908   0.086948   1.977  0.04814 *
## race.ethnicity.5levelBlack     -0.379039   0.234582  -1.616  0.10627
## race.ethnicity.5levelMixed     -0.027092   0.228506  -0.119  0.90563
## race.ethnicity.5levelOther     -0.288256   0.267454  -1.078  0.28124
## race.ethnicity.5levelWhite     -0.069125   0.213328  -0.324  0.74594
## interview_age                   0.004899   0.004668   1.049  0.29413
## bmi                             0.009912   0.009392   1.055  0.29134
## household.income[>=200K]       -0.796185   0.246163  -3.234  0.00124 **
## household.income[100K-200K]    -0.570113   0.229054  -2.489  0.01288 *
## household.income[12K-16K]      -0.281132   0.292691  -0.961  0.33690
## household.income[16K-25K]      -0.368348   0.253986  -1.450  0.14712
## household.income[25K-35K]      -0.033463   0.239558  -0.140  0.88892
## household.income[35K-50K]      -0.545609   0.232715  -2.345  0.01913 *
## household.income[50K-75K]      -0.483238   0.227967  -2.120  0.03413 *
## household.income[5K-12K]       -0.054763   0.258043  -0.212  0.83195
## household.income[75K-100K]     -0.494470   0.232114  -2.130  0.03325 *
## high.educBachelor              -0.035306   0.211327  -0.167  0.86733
## high.educHS Diploma/GED         0.205500   0.212471   0.967  0.33355
## high.educPost Graduate Degree -0.012979   0.215409  -0.060  0.95196
## high.educSome College           0.004969   0.197313   0.025  0.97991
## demo_race_hispanic1            -0.018204   0.101669  -0.179  0.85791
## ---
## Signif. codes:  0 '***' 0.001 '**' 0.01 '*' 0.05 '.' 0.1 ' ' 1
##
```

```
##
## R-sq.(adj) =  0.0201
## lmer.REML = 8979.8  Scale est. = 2.2981    n = 2393
```

```
##                                      stdcoef      stdse
## X(Intercept)                      0.000000000 0.00000000
## Xpds_p_ss_categoryEarly          -0.003916490 0.02393205
## Xpds_p_ss_categoryLate            0.033723996 0.02198833
## Xpds_p_ss_categoryMid             0.053374111 0.02699566
## Xrace.ethnicity.5levelBlack      -0.085727616 0.05305563
## Xrace.ethnicity.5levelMixed      -0.005586584 0.04711925
## Xrace.ethnicity.5levelOther      -0.037809164 0.03508057
## Xrace.ethnicity.5levelWhite      -0.020673578 0.06380134
## Xinterview_age                    0.022957401 0.02187799
## Xbmi                              0.023849449 0.02259725
## Xhousehold.income[>=200K]        -0.158973738 0.04915123
## Xhousehold.income[100K-200K]     -0.167712165 0.06738156
## Xhousehold.income[12K-16K]       -0.026265116 0.02734501
## Xhousehold.income[16K-25K]       -0.046367869 0.03197196
## Xhousehold.income[25K-35K]       -0.005038501 0.03606987
## Xhousehold.income[35K-50K]       -0.097321128 0.04150962
## Xhousehold.income[50K-75K]       -0.104278902 0.04919356
## Xhousehold.income[5K-12K]        -0.006483617 0.03055096
## Xhousehold.income[75K-100K]      -0.111075830 0.05214124
## Xhigh.educBachelor               -0.009796329 0.05863681
## Xhigh.educHS Diploma/GED          0.034866193 0.03604901
## Xhigh.educPost Graduate Degree -0.003950723 0.06556730
## Xhigh.educSome College            0.001348863 0.05355879
## Xdemo_race_hispanic1             -0.004412581 0.02464367
```

Male participants

```
##
## Family: gaussian
## Link function: identity
##
## Formula:
## cbcl_scr_syn_withdep_r ~ pds_p_ss_category + race.ethnicity.5level +
##     interview_age + bmi + household.income + high.educ + demo_race_hispanic
##
## Parametric coefficients:
##                                 Estimate Std. Error t value Pr(>|t|)
## (Intercept)                    0.4451090  0.6951047   0.640 0.522004
## pds_p_ss_categoryEarly         0.0535955  0.0879299   0.610 0.542231
## pds_p_ss_categoryLate         -0.7354390  0.5269689  -1.396 0.162956
## pds_p_ss_categoryMid           0.4046924  0.1694073   2.389 0.016973 *
## race.ethnicity.5levelBlack    -0.1386539  0.2838751  -0.488 0.625285
## race.ethnicity.5levelMixed     0.2762690  0.2785028   0.992 0.321302
## race.ethnicity.5levelOther     0.0012882  0.3172956   0.004 0.996761
## race.ethnicity.5levelWhite     0.1160494  0.2619508   0.443 0.657789
## interview_age                  0.0116528  0.0047666   2.445 0.014565 *
## bmi                            0.0023750  0.0098428   0.241 0.809346
## household.income[>=200K]      -1.0696878  0.2652498  -4.033 5.67e-05 ***
```

```
## household.income[100K-200K]   -0.8863210  0.2476623  -3.579 0.000352 ***
## household.income[12K-16K]     -0.0005363  0.3188739  -0.002 0.998658
## household.income[16K-25K]      0.0415734  0.2667227   0.156 0.876150
## household.income[25K-35K]     -0.0910832  0.2679497  -0.340 0.733940
## household.income[35K-50K]     -0.4789527  0.2531100  -1.892 0.058568 .
## household.income[50K-75K]     -0.6138219  0.2455258  -2.500 0.012481 *
## household.income[5K-12K]      -0.0502801  0.2794385  -0.180 0.857220
## household.income[75K-100K]    -0.9602398  0.2525204  -3.803 0.000147 ***
## high.educBachelor             -0.0120874  0.2500124  -0.048 0.961443
## high.educHS Diploma/GED       -0.6043960  0.2479975  -2.437 0.014873 *
## high.educPost Graduate Degree -0.2652660  0.2510635  -1.057 0.290808
## high.educSome College         -0.1321018  0.2377060  -0.556 0.578440
## demo_race_hispanic1           -0.0726855  0.1101105  -0.660 0.509240
## ---
## Signif. codes:  0 '***' 0.001 '**' 0.01 '*' 0.05 '.' 0.1 ' ' 1
##
##
## R-sq.(adj) =  0.0383
## lmer.REML =  10282  Scale est. = 2.0956    n = 2569
```

```
##                                      stdcoef      stdse
## X(Intercept)                    0.000000e+00 0.00000000
## Xpds_p_ss_categoryEarly         1.235450e-02 0.02026907
## Xpds_p_ss_categoryLate         -2.756456e-02 0.01975101
## Xpds_p_ss_categoryMid           4.928762e-02 0.02063217
## Xrace.ethnicity.5levelBlack    -2.499842e-02 0.05118089
## Xrace.ethnicity.5levelMixed     4.967321e-02 0.05007486
## Xrace.ethnicity.5levelOther     1.488270e-04 0.03665802
## Xrace.ethnicity.5levelWhite     2.949866e-02 0.06658540
## Xinterview_age                  4.824700e-02 0.01973534
## Xbmi                            4.926626e-03 0.02041741
## Xhousehold.income[>=200K]      -1.912665e-01 0.04742824
## Xhousehold.income[100K-200K]   -2.269651e-01 0.06342026
## Xhousehold.income[12K-16K]     -4.267317e-05 0.02537064
## Xhousehold.income[16K-25K]      4.803083e-03 0.03081519
## Xhousehold.income[25K-35K]     -1.109306e-02 0.03263368
## Xhousehold.income[35K-50K]     -7.336613e-02 0.03877147
## Xhousehold.income[50K-75K]     -1.176646e-01 0.04706527
## Xhousehold.income[5K-12K]      -4.999254e-03 0.02778402
## Xhousehold.income[75K-100K]    -1.884017e-01 0.04954519
## Xhigh.educBachelor             -2.921513e-03 0.06042777
## Xhigh.educHS Diploma/GED       -8.798885e-02 0.03610384
## Xhigh.educPost Graduate Degree -7.049666e-02 0.06672221
## Xhigh.educSome College         -3.185198e-02 0.05731494
## Xdemo_race_hispanic1           -1.568491e-02 0.02376091
```

1.8 Model: CBCL Depressed DSM-5 ~ Pubertal category

Female participants

```
##
## Family: gaussian
## Link function: identity
```

```
##
## Formula:
## cbcl_scr_dsm5_depress_r ~ pds_p_ss_category + race.ethnicity.5level +
##     interview_age + bmi + household.income + high.educ + demo_race_hispanic
##
## Parametric coefficients:
##                              Estimate Std. Error t value Pr(>|t|)
## (Intercept)                  1.254271   0.761815   1.646   0.0998 .
## pds_p_ss_categoryEarly      -0.031469   0.104843  -0.300   0.7641
## pds_p_ss_categoryLate        0.127442   0.265291   0.480   0.6310
## pds_p_ss_categoryMid         0.127942   0.101585   1.259   0.2080
## race.ethnicity.5levelBlack  -0.175222   0.274631  -0.638   0.5235
## race.ethnicity.5levelMixed   0.198858   0.267809   0.743   0.4578
## race.ethnicity.5levelOther  -0.235300   0.313628  -0.750   0.4532
## race.ethnicity.5levelWhite   0.213269   0.249686   0.854   0.3931
## interview_age                0.001470   0.005435   0.271   0.7868
## bmi                          0.003036   0.010991   0.276   0.7824
## household.income[>=200K]    -0.713207   0.289257  -2.466   0.0137 *
## household.income[100K-200K] -0.551048   0.269311  -2.046   0.0409 *
## household.income[12K-16K]   -0.005753   0.344647  -0.017   0.9867
## household.income[16K-25K]   -0.453692   0.298004  -1.522   0.1280
## household.income[25K-35K]   -0.047835   0.281624  -0.170   0.8651
## household.income[35K-50K]   -0.341577   0.273423  -1.249   0.2117
## household.income[50K-75K]   -0.440780   0.268058  -1.644   0.1002
## household.income[5K-12K]     0.164917   0.302684   0.545   0.5859
## household.income[75K-100K]  -0.457504   0.272841  -1.677   0.0937 .
## high.educBachelor           -0.224131   0.249030  -0.900   0.3682
## high.educHS Diploma/GED     -0.104752   0.249911  -0.419   0.6751
## high.educPost Graduate Degree -0.042354   0.253781  -0.167   0.8675
## high.educSome College       -0.090303   0.232462  -0.388   0.6977
## demo_race_hispanic1         -0.016516   0.119227  -0.139   0.8898
## ---
## Signif. codes:  0 '***' 0.001 '**' 0.01 '*' 0.05 '.' 0.1 ' ' 1
##
##
## R-sq.(adj) =  0.00935
## lmer.REML = 9698.5  Scale est. = 2.4591    n = 2393
```

```
##                                     stdcoef      stdse
## X(Intercept)                   0.0000000000 0.00000000
## Xpds_p_ss_categoryEarly       -0.0072279099 0.02408050
## Xpds_p_ss_categoryLate         0.0106453589 0.02216007
## Xpds_p_ss_categoryMid          0.0342991655 0.02723315
## Xrace.ethnicity.5levelBlack   -0.0342184344 0.05363160
## Xrace.ethnicity.5levelMixed    0.0354061352 0.04768267
## Xrace.ethnicity.5levelOther   -0.0266486221 0.03551958
## Xrace.ethnicity.5levelWhite    0.0550736908 0.06447787
## Xinterview_age                 0.0059493874 0.02199277
## Xbmi                           0.0063081780 0.02283452
## Xhousehold.income[>=200K]     -0.1229593439 0.04986886
## Xhousehold.income[100K-200K]  -0.1399677838 0.06840581
## Xhousehold.income[12K-16K]    -0.0004641022 0.02780212
## Xhousehold.income[16K-25K]    -0.0493122113 0.03239028
## Xhousehold.income[25K-35K]    -0.0062189579 0.03661335
```

```
## Xhousehold.income[35K-50K]     -0.0526076176 0.04211086
## Xhousehold.income[50K-75K]     -0.0821282240 0.04994574
## Xhousehold.income[5K-12K]       0.0168590353 0.03094267
## Xhousehold.income[75K-100K]    -0.0887377464 0.05292043
## Xhigh.educBachelor             -0.0536971948 0.05966250
## Xhigh.educHS Diploma/GED       -0.0153458161 0.03661113
## Xhigh.educPost Graduate Degree -0.0111314677 0.06669864
## Xhigh.educSome College         -0.0211646044 0.05448293
## Xdemo_race_hispanic1           -0.0034566063 0.02495321
```

Male participants

```
##
## Family: gaussian
## Link function: identity
##
## Formula:
## cbcl_scr_dsm5_depress_r ~ pds_p_ss_category + race.ethnicity.5level +
##     interview_age + bmi + household.income + high.educ + demo_race_hispanic
##
## Parametric coefficients:
##                              Estimate Std. Error t value Pr(>|t|)
## (Intercept)                   0.919060   0.817124   1.125 0.260801
## pds_p_ss_categoryEarly        0.131314   0.103122   1.273 0.202997
## pds_p_ss_categoryLate        -0.050509   0.618679  -0.082 0.934939
## pds_p_ss_categoryMid          0.228391   0.198509   1.151 0.250033
## race.ethnicity.5levelBlack   -0.138135   0.334101  -0.413 0.679309
## race.ethnicity.5levelMixed    0.276470   0.327512   0.844 0.398664
## race.ethnicity.5levelOther   -0.008044   0.372706  -0.022 0.982783
## race.ethnicity.5levelWhite    0.185103   0.308249   0.601 0.548227
## interview_age                 0.007297   0.005595   1.304 0.192300
## bmi                           0.001438   0.011540   0.125 0.900848
## household.income[>=200K]     -1.156917   0.312470  -3.702 0.000218 ***
## household.income[100K-200K]  -1.061862   0.291411  -3.644 0.000274 ***
## household.income[12K-16K]     0.060032   0.374722   0.160 0.872733
## household.income[16K-25K]    -0.399162   0.313456  -1.273 0.202985
## household.income[25K-35K]    -0.318832   0.314814  -1.013 0.311269
## household.income[35K-50K]    -0.669739   0.297481  -2.251 0.024448 *
## household.income[50K-75K]    -0.744708   0.288630  -2.580 0.009931 **
## household.income[5K-12K]     -0.167979   0.328248  -0.512 0.608875
## household.income[75K-100K]   -0.978162   0.296995  -3.294 0.001003 **
## high.educBachelor             0.417229   0.294037   1.419 0.156031
## high.educHS Diploma/GED      -0.340316   0.291614  -1.167 0.243316
## high.educPost Graduate Degree  0.090867   0.295247   0.308 0.758285
## high.educSome College         0.372376   0.279584   1.332 0.183014
## demo_race_hispanic1          -0.061302   0.132246  -0.464 0.643013
## ---
## Signif. codes:  0 '***' 0.001 '**' 0.01 '*' 0.05 '.' 0.1 ' ' 1
##
##
## R-sq.(adj) =  0.0244
## lmer.REML =  11090  Scale est. = 2.5922    n = 2569

##                                      stdcoef      stdse
```

```
## X(Intercept)                      0.0000000000 0.00000000
## Xpds_p_ss_categoryEarly           0.0259494285 0.02037828
## Xpds_p_ss_categoryLate           -0.0016228984 0.01987868
## Xpds_p_ss_categoryMid             0.0238456814 0.02072579
## Xrace.ethnicity.5levelBlack      -0.0213502668 0.05163879
## Xrace.ethnicity.5levelMixed       0.0426144449 0.05048193
## Xrace.ethnicity.5levelOther      -0.0007966726 0.03691385
## Xrace.ethnicity.5levelWhite       0.0403358870 0.06717050
## Xinterview_age                    0.0258992541 0.01985907
## Xbmi                              0.0025570479 0.02052168
## Xhousehold.income[>=200K]        -0.1773381487 0.04789707
## Xhousehold.income[100K-200K]     -0.2331064804 0.06397243
## Xhousehold.income[12K-16K]        0.0040946171 0.02555875
## Xhousehold.income[16K-25K]       -0.0395341011 0.03104560
## Xhousehold.income[25K-35K]       -0.0332883855 0.03286885
## Xhousehold.income[35K-50K]       -0.0879480927 0.03906437
## Xhousehold.income[50K-75K]       -0.1223791109 0.04743111
## Xhousehold.income[5K-12K]        -0.0143179758 0.02797881
## Xhousehold.income[75K-100K]      -0.1645258568 0.04995420
## Xhigh.educBachelor                0.0864504615 0.06092491
## Xhigh.educHS Diploma/GED         -0.0424723861 0.03639425
## Xhigh.educPost Graduate Degree  0.0207019540 0.06726512
## Xhigh.educSome College            0.0769710225 0.05779063
## Xdemo_race_hispanic1             -0.0113403929 0.02446448
```

1.9 Model: CBCL internalizing factor ~ Testosterone

Female participants

```
##
## Family: gaussian
## Link function: identity
##
## Formula:
## cbcl_scr_syn_internal_r ~ hormone_scr_ert_mean + hormone_sal_end_min_since_midnight +
##     race.ethnicity.5level + interview_age + bmi + household.income +
##     high.educ + demo_race_hispanic
##
## Parametric coefficients:
##                                     Estimate Std. Error t value Pr(>|t|)
## (Intercept)                        -2.202149   2.280520  -0.966 0.334335
## hormone_scr_ert_mean                0.001591   0.007417   0.215 0.830155
## hormone_sal_end_min_since_midnight  0.000333   0.000704   0.473 0.636225
## race.ethnicity.5levelBlack         -0.564439   0.816203  -0.692 0.489299
## race.ethnicity.5levelMixed          1.216996   0.796092   1.529 0.126481
## race.ethnicity.5levelOther         -0.257284   0.939620  -0.274 0.784251
## race.ethnicity.5levelWhite          1.214960   0.740255   1.641 0.100886
## interview_age                       0.044621   0.015797   2.825 0.004778 **
## bmi                                 0.061797   0.032227   1.918 0.055302 .
## household.income[>=200K]           -2.985044   0.872121  -3.423 0.000631 ***
## household.income[100K-200K]        -2.097440   0.809473  -2.591 0.009630 **
## household.income[12K-16K]          -0.727069   1.048155  -0.694 0.487966
## household.income[16K-25K]          -1.411506   0.901101  -1.566 0.117395
```

```
## household.income[25K-35K]          -0.571138   0.846422  -0.675 0.499895
## household.income[35K-50K]          -1.576855   0.823333  -1.915 0.055597 .
## household.income[50K-75K]          -1.595383   0.809216  -1.972 0.048792 *
## household.income[5K-12K]           -0.556654   0.927818  -0.600 0.548595
## household.income[75K-100K]         -1.766294   0.820500  -2.153 0.031452 *
## high.educBachelor                   1.083622   0.760606   1.425 0.154393
## high.educHS Diploma/GED             1.135117   0.762582   1.489 0.136759
## high.educPost Graduate Degree       1.521768   0.774672   1.964 0.049611 *
## high.educSome College               1.495050   0.709347   2.108 0.035176 *
## demo_race_hispanic1                -0.097395   0.364022  -0.268 0.789069
## ---
## Signif. codes:  0 '***' 0.001 '**' 0.01 '*' 0.05 '.' 0.1 ' ' 1
##
##
## R-sq.(adj) =  0.0182
## lmer.REML =  13547  Scale est. = 17.516    n = 2194
```

```
##                                          stdcoef      stdse
## X(Intercept)                          0.000000000 0.00000000
## Xhormone_scr_ert_mean                 0.004840352 0.02256278
## Xhormone_sal_end_min_since_midnight  0.010829345 0.02289260
## Xrace.ethnicity.5levelBlack          -0.036638090 0.05298026
## Xrace.ethnicity.5levelMixed           0.073888624 0.04833387
## Xrace.ethnicity.5levelOther          -0.009980560 0.03644977
## Xrace.ethnicity.5levelWhite           0.106435657 0.06484950
## Xinterview_age                        0.061698398 0.02184361
## Xbmi                                  0.043354829 0.02260980
## Xhousehold.income[>=200K]            -0.173724052 0.05075584
## Xhousehold.income[100K-200K]         -0.181715502 0.07013011
## Xhousehold.income[12K-16K]           -0.019887697 0.02867044
## Xhousehold.income[16K-25K]           -0.051823166 0.03308374
## Xhousehold.income[25K-35K]           -0.025580886 0.03791066
## Xhousehold.income[35K-50K]           -0.083063963 0.04337066
## Xhousehold.income[50K-75K]           -0.100891812 0.05117474
## Xhousehold.income[5K-12K]            -0.018602294 0.03100586
## Xhousehold.income[75K-100K]          -0.117891092 0.05476419
## Xhigh.educBachelor                    0.089130809 0.06256192
## Xhigh.educHS Diploma/GED              0.056629670 0.03804432
## Xhigh.educPost Graduate Degree        0.136046561 0.06925596
## Xhigh.educSome College                0.118987462 0.05645521
## Xdemo_race_hispanic1                 -0.006961064 0.02601753
```

Male participants

```
##
## Family: gaussian
## Link function: identity
##
## Formula:
## cbcl_scr_syn_internal_r ~ hormone_scr_ert_mean + hormone_sal_end_min_since_midnight +
##     race.ethnicity.5level + interview_age + bmi + household.income +
##     high.educ + demo_race_hispanic
##
```

```
## Parametric coefficients:
##                                     Estimate Std. Error t value Pr(>|t|)    
## (Intercept)                        3.4702040  2.2481331   1.544 0.122821    
## hormone_scr_ert_mean               0.0046607  0.0078486   0.594 0.552682    
## hormone_sal_end_min_since_midnight  0.0011017  0.0006821   1.615 0.106444    
## race.ethnicity.5levelBlack        -0.4315199  0.8972575  -0.481 0.630609    
## race.ethnicity.5levelMixed         1.0298655  0.8773329   1.174 0.240570    
## race.ethnicity.5levelOther         0.0921606  0.9966106   0.092 0.926329    
## race.ethnicity.5levelWhite         0.9603325  0.8254114   1.163 0.244761    
## interview_age                      0.0070208  0.0149788   0.469 0.639316    
## bmi                                0.0301220  0.0311319   0.968 0.333363    
## household.income[>=200K]          -3.3042047  0.8501915  -3.886 0.000105 ***
## household.income[100K-200K]       -2.7538721  0.7955897  -3.461 0.000547 ***
## household.income[12K-16K]         -0.2693924  1.0311979  -0.261 0.793929    
## household.income[16K-25K]         -0.2937293  0.8548526  -0.344 0.731176    
## household.income[25K-35K]         -0.9334087  0.8540096  -1.093 0.274518    
## household.income[35K-50K]         -1.4377482  0.8114108  -1.772 0.076538 .  
## household.income[50K-75K]         -1.9120582  0.7880493  -2.426 0.015327 *  
## household.income[5K-12K]          -0.2427380  0.8845231  -0.274 0.783780    
## household.income[75K-100K]        -2.8687056  0.8109027  -3.538 0.000411 ***
## high.educBachelor                  1.2471681  0.7970956   1.565 0.117802    
## high.educHS Diploma/GED           -0.7415617  0.7910872  -0.937 0.348651    
## high.educPost Graduate Degree      0.4604767  0.8016954   0.574 0.565766    
## high.educSome College              0.8569055  0.7587888   1.129 0.258883    
## demo_race_hispanic1               -0.0396132  0.3573113  -0.111 0.911733    
## ---
## Signif. codes:  0 '***' 0.001 '**' 0.01 '*' 0.05 '.' 0.1 ' ' 1
##
##
## R-sq.(adj) =  0.0297
## lmer.REML =  14777  Scale est. = 16.018    n = 2379
```

```
##                                         stdcoef      stdse
## X(Intercept)                        0.000000000 0.00000000
## Xhormone_scr_ert_mean               0.012547944 0.02113050
## Xhormone_sal_end_min_since_midnight  0.035684718 0.02209578
## Xrace.ethnicity.5levelBlack        -0.025151973 0.05229840
## Xrace.ethnicity.5levelMixed         0.060850493 0.05183797
## Xrace.ethnicity.5levelOther         0.003525295 0.03812200
## Xrace.ethnicity.5levelWhite         0.079954464 0.06872133
## Xinterview_age                      0.009614454 0.02051232
## Xbmi                                0.020532669 0.02122106
## Xhousehold.income[>=200K]          -0.197242683 0.05075171
## Xhousehold.income[100K-200K]       -0.231486475 0.06687611
## Xhousehold.income[12K-16K]         -0.006909980 0.02645047
## Xhousehold.income[16K-25K]         -0.010994076 0.03199651
## Xhousehold.income[25K-35K]         -0.037325815 0.03415075
## Xhousehold.income[35K-50K]         -0.071558081 0.04038468
## Xhousehold.income[50K-75K]         -0.120706707 0.04974892
## Xhousehold.income[5K-12K]          -0.008090662 0.02948191
## Xhousehold.income[75K-100K]        -0.184809496 0.05224047
## Xhigh.educBachelor                  0.099482191 0.06358150
## Xhigh.educHS Diploma/GED           -0.035507148 0.03787851
## Xhigh.educPost Graduate Degree      0.040258808 0.07009106
```

```
## Xhigh.educSome College               0.067391134 0.05967477
## Xdemo_race_hispanic1                -0.002813609 0.02537876
```

1.10 Model: CBCL Anxious-Depressed ~ Testosterone

Female participants

```
##
## Family: gaussian
## Link function: identity
##
## Formula:
## cbcl_scr_syn_anxdep_r ~ hormone_scr_ert_mean + hormone_sal_end_min_since_midnight +
##     race.ethnicity.5level + interview_age + bmi + household.income +
##     high.educ + demo_race_hispanic
##
## Parametric coefficients:
##                                     Estimate Std. Error t value Pr(>|t|)
## (Intercept)                       -0.6772703  1.2914696  -0.524   0.6000
## hormone_scr_ert_mean               0.0004716  0.0042039   0.112   0.9107
## hormone_sal_end_min_since_midnight  0.0003276  0.0003970   0.825   0.4093
## race.ethnicity.5levelBlack        -0.1368519  0.4594753  -0.298   0.7659
## race.ethnicity.5levelMixed         0.8384900  0.4484098   1.870   0.0616 .
## race.ethnicity.5levelOther         0.1023564  0.5295352   0.193   0.8467
## race.ethnicity.5levelWhite         0.7245162  0.4167716   1.738   0.0823 .
## interview_age                      0.0192288  0.0089747   2.143   0.0323 *
## bmi                                0.0059966  0.0182163   0.329   0.7420
## household.income[>=200K]          -1.0963527  0.4912061  -2.232   0.0257 *
## household.income[100K-200K]       -0.5902143  0.4558312  -1.295   0.1955
## household.income[12K-16K]         -0.1953279  0.5895983  -0.331   0.7405
## household.income[16K-25K]         -0.6022378  0.5082887  -1.185   0.2362
## household.income[25K-35K]         -0.0412148  0.4767757  -0.086   0.9311
## household.income[35K-50K]         -0.4586758  0.4638686  -0.989   0.3229
## household.income[50K-75K]         -0.3492380  0.4556672  -0.766   0.4435
## household.income[5K-12K]          -0.1950754  0.5241195  -0.372   0.7098
## household.income[75K-100K]        -0.4164295  0.4621374  -0.901   0.3676
## high.educBachelor                  0.1977527  0.4281320   0.462   0.6442
## high.educHS Diploma/GED            0.1323975  0.4297721   0.308   0.7581
## high.educPost Graduate Degree      0.6574649  0.4361054   1.508   0.1318
## high.educSome College              0.4952835  0.3994622   1.240   0.2152
## demo_race_hispanic1                0.0680369  0.2042909   0.333   0.7391
## ---
## Signif. codes:  0 '***' 0.001 '**' 0.01 '*' 0.05 '.' 0.1 ' ' 1
##
##
## R-sq.(adj) =  0.0129
## lmer.REML =  11083  Scale est. = 6.8399    n = 2194
```

```
##                                         stdcoef      stdse
## X(Intercept)                        0.000000000 0.00000000
## Xhormone_scr_ert_mean               0.002544361 0.02268111
## Xhormone_sal_end_min_since_midnight  0.018894731 0.02289430
## Xrace.ethnicity.5levelBlack        -0.015755433 0.05289830
```

```
## Xrace.ethnicity.5levelMixed          0.090292208 0.04828670
## Xrace.ethnicity.5levelOther          0.007042411 0.03643351
## Xrace.ethnicity.5levelWhite          0.112573733 0.06475705
## Xinterview_age                       0.047157898 0.02201015
## Xbmi                                 0.007461794 0.02266703
## Xhousehold.income[>=200K]           -0.113167934 0.05070338
## Xhousehold.income[100K-200K]        -0.090693462 0.07004389
## Xhousehold.income[12K-16K]          -0.009476260 0.02860413
## Xhousehold.income[16K-25K]          -0.039216878 0.03309905
## Xhousehold.income[25K-35K]          -0.003274100 0.03787498
## Xhousehold.income[35K-50K]          -0.042853902 0.04333906
## Xhousehold.income[50K-75K]          -0.039172057 0.05110961
## Xhousehold.income[5K-12K]           -0.011562380 0.03106526
## Xhousehold.income[75K-100K]         -0.049297314 0.05470826
## Xhigh.educBachelor                   0.028849384 0.06245854
## Xhigh.educHS Diploma/GED             0.011715124 0.03802816
## Xhigh.educPost Graduate Degree       0.104249874 0.06915036
## Xhigh.educSome College               0.069913836 0.05638778
## Xdemo_race_hispanic1                 0.008624754 0.02589710
```

Male participants

```
##
## Family: gaussian
## Link function: identity
##
## Formula:
## cbcl_scr_syn_anxdep_r ~ hormone_scr_ert_mean + hormone_sal_end_min_since_midnight +
##     race.ethnicity.5level + interview_age + bmi + household.income +
##     high.educ + demo_race_hispanic
##
## Parametric coefficients:
##                                      Estimate Std. Error t value Pr(>|t|)
## (Intercept)                         2.7460088  1.2664351   2.168  0.03024 *
## hormone_scr_ert_mean                0.0034859  0.0044200   0.789  0.43039
## hormone_sal_end_min_since_midnight  0.0002436  0.0003841   0.634  0.52604
## race.ethnicity.5levelBlack         -0.1414372  0.5039069  -0.281  0.77898
## race.ethnicity.5levelMixed          0.4806227  0.4926632   0.976  0.32938
## race.ethnicity.5levelOther          0.2618683  0.5607106   0.467  0.64052
## race.ethnicity.5levelWhite          0.6198641  0.4636818   1.337  0.18141
## interview_age                      -0.0078450  0.0084611  -0.927  0.35392
## bmi                                 0.0016722  0.0175439   0.095  0.92407
## household.income[>=200K]           -1.3511839  0.4766146  -2.835  0.00462 **
## household.income[100K-200K]        -1.0675358  0.4462099  -2.392  0.01681 *
## household.income[12K-16K]           0.0284430  0.5779694   0.049  0.96075
## household.income[16K-25K]          -0.2406261  0.4797897  -0.502  0.61605
## household.income[25K-35K]          -0.3568352  0.4791715  -0.745  0.45653
## household.income[35K-50K]          -0.4482888  0.4550330  -0.985  0.32464
## household.income[50K-75K]          -0.8537161  0.4420421  -1.931  0.05357 .
## household.income[5K-12K]           -0.0012877  0.4960201  -0.003  0.99793
## household.income[75K-100K]         -1.0507535  0.4547320  -2.311  0.02093 *
## high.educBachelor                   1.0870212  0.4470939   2.431  0.01512 *
## high.educHS Diploma/GED            -0.0829352  0.4438869  -0.187  0.85180
```

```
## high.educPost Graduate Degree       0.7419754  0.4496310   1.650  0.09904 .
## high.educSome College               0.6922998  0.4257763   1.626  0.10409
## demo_race_hispanic1                 0.0974709  0.2003854   0.486  0.62672
## ---
## Signif. codes:  0 '***' 0.001 '**' 0.01 '*' 0.05 '.' 0.1 ' ' 1
##
##
## R-sq.(adj) =  0.0136
## lmer.REML =  12078  Scale est. = 6.4672    n = 2379
```

```
##                                          stdcoef      stdse
## X(Intercept)                        0.000000e+00 0.00000000
## Xhormone_scr_ert_mean               1.686789e-02 0.02138790
## Xhormone_sal_end_min_since_midnight  1.418051e-02 0.02236112
## Xrace.ethnicity.5levelBlack        -1.481686e-02 0.05278892
## Xrace.ethnicity.5levelMixed         5.103982e-02 0.05231846
## Xrace.ethnicity.5levelOther         1.800339e-02 0.03854874
## Xrace.ethnicity.5levelWhite         9.275532e-02 0.06938449
## Xinterview_age                     -1.930878e-02 0.02082506
## Xbmi                                2.048620e-03 0.02149363
## Xhousehold.income[>=200K]          -1.449672e-01 0.05113550
## Xhousehold.income[100K-200K]       -1.612819e-01 0.06741278
## Xhousehold.income[12K-16K]          1.311258e-03 0.02664510
## Xhousehold.income[16K-25K]         -1.618733e-02 0.03227629
## Xhousehold.income[25K-35K]         -2.564640e-02 0.03443893
## Xhousehold.income[35K-50K]         -4.010098e-02 0.04070427
## Xhousehold.income[50K-75K]         -9.686457e-02 0.05015510
## Xhousehold.income[5K-12K]          -7.713877e-05 0.02971439
## Xhousehold.income[75K-100K]        -1.216635e-01 0.05265202
## Xhigh.educBachelor                  1.558402e-01 0.06409739
## Xhigh.educHS Diploma/GED           -7.137211e-03 0.03819988
## Xhigh.educPost Graduate Degree      1.165907e-01 0.07065302
## Xhigh.educSome College              9.785551e-02 0.06018282
## Xdemo_race_hispanic1                1.244285e-02 0.02558061
```

1.11 Model: CBCL Withdrawn-Depressed ~ Testosterone

Female participants

```
##
## Family: gaussian
## Link function: identity
##
## Formula:
## cbcl_scr_syn_withdep_r ~ hormone_scr_ert_mean + hormone_sal_end_min_since_midnight +
##     race.ethnicity.5level + interview_age + bmi + household.income +
##     high.educ + demo_race_hispanic
##
## Parametric coefficients:
##                                      Estimate Std. Error t value Pr(>|t|)
## (Intercept)                         2.037e-01  6.726e-01   0.303  0.76200
## hormone_scr_ert_mean                4.124e-03  2.189e-03   1.884  0.05974 .
## hormone_sal_end_min_since_midnight -7.106e-05  2.069e-04  -0.343  0.73136
```

```
## race.ethnicity.5levelBlack         -4.212e-01  2.384e-01  -1.766  0.07745 .
## race.ethnicity.5levelMixed         -8.803e-04  2.326e-01  -0.004  0.99698
## race.ethnicity.5levelOther         -3.356e-01  2.745e-01  -1.222  0.22169
## race.ethnicity.5levelWhite         -2.897e-02  2.163e-01  -0.134  0.89345
## interview_age                       6.707e-03  4.685e-03   1.432  0.15241
## bmi                                 1.811e-02  9.463e-03   1.914  0.05579 .
## household.income[>=200K]           -9.507e-01  2.543e-01  -3.738  0.00019 ***
## household.income[100K-200K]        -7.053e-01  2.359e-01  -2.990  0.00282 **
## household.income[12K-16K]          -4.337e-01  3.047e-01  -1.423  0.15477
## household.income[16K-25K]          -3.490e-01  2.637e-01  -1.324  0.18571
## household.income[25K-35K]          -1.819e-01  2.468e-01  -0.737  0.46114
## household.income[35K-50K]          -6.173e-01  2.402e-01  -2.570  0.01024 *
## household.income[50K-75K]          -5.894e-01  2.358e-01  -2.500  0.01251 *
## household.income[5K-12K]           -1.436e-01  2.721e-01  -0.528  0.59789
## household.income[75K-100K]         -6.301e-01  2.392e-01  -2.634  0.00850 **
## high.educBachelor                   1.357e-01  2.215e-01   0.613  0.54025
## high.educHS Diploma/GED             4.185e-01  2.228e-01   1.878  0.06050 .
## high.educPost Graduate Degree       1.955e-01  2.257e-01   0.866  0.38656
## high.educSome College               2.308e-01  2.068e-01   1.116  0.26454
## demo_race_hispanic1                -1.692e-02  1.058e-01  -0.160  0.87297
## ---
## Signif. codes:  0 '***' 0.001 '**' 0.01 '*' 0.05 '.' 0.1 ' ' 1
##
##
## R-sq.(adj) =  0.0203
## lmer.REML = 8259.1  Scale est. = 2.2518    n = 2194
```

```
##                                          stdcoef      stdse
## X(Intercept)                         0.0000000000 0.00000000
## Xhormone_scr_ert_mean                0.0426125573 0.02262163
## Xhormone_sal_end_min_since_midnight -0.0078483593 0.02285727
## Xrace.ethnicity.5levelBlack         -0.0928636637 0.05256935
## Xrace.ethnicity.5levelMixed         -0.0001815482 0.04796267
## Xrace.ethnicity.5levelOther         -0.0442201714 0.03617525
## Xrace.ethnicity.5levelWhite         -0.0086210624 0.06436019
## Xinterview_age                       0.0315024732 0.02200576
## Xbmi                                 0.0431546353 0.02255065
## Xhousehold.income[>=200K]           -0.1879280523 0.05027772
## Xhousehold.income[100K-200K]        -0.2075481173 0.06942079
## Xhousehold.income[12K-16K]          -0.0402907825 0.02830635
## Xhousehold.income[16K-25K]          -0.0435275174 0.03288109
## Xhousehold.income[25K-35K]          -0.0276722498 0.03754205
## Xhousehold.income[35K-50K]          -0.1104534839 0.04298250
## Xhousehold.income[50K-75K]          -0.1265947324 0.05064665
## Xhousehold.income[5K-12K]           -0.0162952613 0.03089090
## Xhousehold.income[75K-100K]         -0.1428499416 0.05423376
## Xhigh.educBachelor                   0.0379140424 0.06189705
## Xhigh.educHS Diploma/GED             0.0709092319 0.03775538
## Xhigh.educPost Graduate Degree       0.0593634032 0.06854558
## Xhigh.educSome College               0.0623960102 0.05591010
## Xdemo_race_hispanic1                -0.0041073352 0.02568594
```

Male participants

```
##
## Family: gaussian
## Link function: identity
##
## Formula:
## cbcl_scr_syn_withdep_r ~ hormone_scr_ert_mean + hormone_sal_end_min_since_midnight +
##     race.ethnicity.5level + interview_age + bmi + household.income +
##     high.educ + demo_race_hispanic
##
## Parametric coefficients:
##                                     Estimate Std. Error t value Pr(>|t|)
## (Intercept)                        0.1749096  0.7338713   0.238  0.81164
## hormone_scr_ert_mean               0.0033801  0.0025531   1.324  0.18566
## hormone_sal_end_min_since_midnight  0.0004992  0.0002147   2.325  0.02015 *
## race.ethnicity.5levelBlack        -0.1281806  0.2924864  -0.438  0.66125
## race.ethnicity.5levelMixed         0.2745361  0.2863845   0.959  0.33784
## race.ethnicity.5levelOther         0.0361823  0.3261409   0.111  0.91167
## race.ethnicity.5levelWhite         0.1320389  0.2689971   0.491  0.62357
## interview_age                      0.0115222  0.0049116   2.346  0.01906 *
## bmi                               -0.0013690  0.0102158  -0.134  0.89341
## household.income[>=200K]          -1.1162297  0.2768150  -4.032  5.7e-05 ***
## household.income[100K-200K]       -0.9606560  0.2595893  -3.701  0.00022 ***
## household.income[12K-16K]         -0.0032637  0.3371566  -0.010  0.99228
## household.income[16K-25K]         -0.0013884  0.2795849  -0.005  0.99604
## household.income[25K-35K]         -0.3089656  0.2792356  -1.106  0.26864
## household.income[35K-50K]         -0.5523921  0.2653105  -2.082  0.03744 *
## household.income[50K-75K]         -0.7146659  0.2574872  -2.776  0.00555 **
## household.income[5K-12K]          -0.0756580  0.2894550  -0.261  0.79382
## household.income[75K-100K]        -1.0099203  0.2648045  -3.814  0.00014 ***
## high.educBachelor                 -0.0515743  0.2599490  -0.198  0.84275
## high.educHS Diploma/GED           -0.5481762  0.2580618  -2.124  0.03376 *
## high.educPost Graduate Degree     -0.3230344  0.2615604  -1.235  0.21694
## high.educSome College             -0.1263173  0.2473801  -0.511  0.60966
## demo_race_hispanic1               -0.1453466  0.1122593  -1.295  0.19554
## ---
## Signif. codes:  0 '***' 0.001 '**' 0.01 '*' 0.05 '.' 0.1 ' ' 1
##
##
## R-sq.(adj) =  0.0387
## lmer.REML = 9527.1  Scale est. = 2.0837    n = 2379
```

```
##                                          stdcoef      stdse
## X(Intercept)                         0.0000000000 0.00000000
## Xhormone_scr_ert_mean                0.0277994013 0.02099776
## Xhormone_sal_end_min_since_midnight  0.0493908784 0.02124256
## Xrace.ethnicity.5levelBlack         -0.0228230359 0.05207828
## Xrace.ethnicity.5levelMixed          0.0495521802 0.05169074
## Xrace.ethnicity.5levelOther          0.0042279077 0.03810966
## Xrace.ethnicity.5levelWhite          0.0335817038 0.06841454
## Xinterview_age                       0.0482005379 0.02054664
## Xbmi                                -0.0028506779 0.02127237
## Xhousehold.income[>=200K]           -0.2035481777 0.05047813
```

```
## Xhousehold.income[100K-200K]        -0.2466774492 0.06665741
## Xhousehold.income[12K-16K]          -0.0002557279 0.02641817
## Xhousehold.income[16K-25K]          -0.0001587499 0.03196721
## Xhousehold.income[25K-35K]          -0.0377422046 0.03411049
## Xhousehold.income[35K-50K]          -0.0839852427 0.04033760
## Xhousehold.income[50K-75K]          -0.1378202513 0.04965530
## Xhousehold.income[5K-12K]           -0.0077033702 0.02947183
## Xhousehold.income[75K-100K]         -0.1987490622 0.05211267
## Xhigh.educBachelor                  -0.0125670491 0.06334148
## Xhigh.educHS Diploma/GED            -0.0801804357 0.03774608
## Xhigh.educPost Graduate Degree      -0.0862743701 0.06985620
## Xhigh.educSome College              -0.0303467608 0.05943118
## Xdemo_race_hispanic1                -0.0315361096 0.02435710
```

1.12 Model: CBCL Depressed DSM-5 ~ Testosterone

Female participants

```
##
## Family: gaussian
## Link function: identity
##
## Formula:
## cbcl_scr_dsm5_depress_r ~ hormone_scr_ert_mean + hormone_sal_end_min_since_midnight +
##     race.ethnicity.5level + interview_age + bmi + household.income +
##     high.educ + demo_race_hispanic
##
## Parametric coefficients:
##                                     Estimate Std. Error t value Pr(>|t|)
## (Intercept)                         0.5448231  0.7855508   0.694  0.48804
## hormone_scr_ert_mean                0.0002492  0.0025575   0.097  0.92238
## hormone_sal_end_min_since_midnight  0.0001340  0.0002405   0.557  0.57748
## race.ethnicity.5levelBlack         -0.1485679  0.2793271  -0.532  0.59487
## race.ethnicity.5levelMixed          0.2228049  0.2728009   0.817  0.41417
## race.ethnicity.5levelOther         -0.2463580  0.3222510  -0.764  0.44466
## race.ethnicity.5levelWhite          0.2689667  0.2533752   1.062  0.28856
## interview_age                       0.0050960  0.0054599   0.933  0.35074
## bmi                                 0.0136328  0.0110909   1.229  0.21914
## household.income[>=200K]           -0.8399652  0.2990620  -2.809  0.00502 **
## household.income[100K-200K]        -0.6906508  0.2775604  -2.488  0.01291 *
## household.income[12K-16K]          -0.1248396  0.3590775  -0.348  0.72812
## household.income[16K-25K]          -0.4488379  0.3095188  -1.450  0.14717
## household.income[25K-35K]          -0.1706572  0.2903564  -0.588  0.55676
## household.income[35K-50K]          -0.4208837  0.2824487  -1.490  0.13634
## household.income[50K-75K]          -0.5418391  0.2774752  -1.953  0.05098 .
## household.income[5K-12K]           -0.0400520  0.3191595  -0.125  0.90015
## household.income[75K-100K]         -0.5672067  0.2814055  -2.016  0.04396 *
## high.educBachelor                  -0.1290761  0.2607057  -0.495  0.62058
## high.educHS Diploma/GED            -0.0003630  0.2616818  -0.001  0.99889
## high.educPost Graduate Degree       0.0669397  0.2655463   0.252  0.80100
## high.educSome College               0.0006746  0.2432671   0.003  0.99779
## demo_race_hispanic1                -0.0492478  0.1239892  -0.397  0.69126
## ---
```

```
## Signif. codes:  0 '***' 0.001 '**' 0.01 '*' 0.05 '.' 0.1 ' ' 1
##
##
## R-sq.(adj) =  0.0086
## lmer.REML =   8927  Scale est. = 2.504     n = 2194
```

```
##                                         stdcoef      stdse
## X(Intercept)                        0.000000e+00 0.00000000
## Xhormone_scr_ert_mean               2.220109e-03 0.02278263
## Xhormone_sal_end_min_since_midnight  1.276038e-02 0.02290299
## Xrace.ethnicity.5levelBlack        -2.824060e-02 0.05309604
## Xrace.ethnicity.5levelMixed         3.961381e-02 0.04850289
## Xrace.ethnicity.5levelOther        -2.798609e-02 0.03660749
## Xrace.ethnicity.5levelWhite         6.900126e-02 0.06500139
## Xinterview_age                      2.063498e-02 0.02210842
## Xbmi                                2.800840e-02 0.02278620
## Xhousehold.income[>=200K]          -1.431541e-01 0.05096874
## Xhousehold.income[100K-200K]       -1.752243e-01 0.07041955
## Xhousehold.income[12K-16K]         -9.999869e-03 0.02876274
## Xhousehold.income[16K-25K]         -4.825741e-02 0.03327833
## Xhousehold.income[25K-35K]         -2.238373e-02 0.03808370
## Xhousehold.income[35K-50K]         -6.492563e-02 0.04357061
## Xhousehold.income[50K-75K]         -1.003447e-01 0.05138643
## Xhousehold.income[5K-12K]          -3.919572e-03 0.03123359
## Xhousehold.income[75K-100K]        -1.108645e-01 0.05500268
## Xhigh.educBachelor                 -3.109061e-02 0.06279630
## Xhigh.educHS Diploma/GED           -5.303778e-05 0.03823051
## Xhigh.educPost Graduate Degree      1.752493e-02 0.06952047
## Xhigh.educSome College              1.572348e-04 0.05669726
## Xdemo_race_hispanic1               -1.030762e-02 0.02595108
```

Male participants

```
##
## Family: gaussian
## Link function: identity
##
## Formula:
## cbcl_scr_dsm5_depress_r ~ hormone_scr_ert_mean + hormone_sal_end_min_since_midnight +
##     race.ethnicity.5level + interview_age + bmi + household.income +
##     high.educ + demo_race_hispanic
##
## Parametric coefficients:
##                                      Estimate Std. Error t value Pr(>|t|)
## (Intercept)                         1.1583182  0.8551288   1.355 0.175690
## hormone_scr_ert_mean                0.0024320  0.0029842   0.815 0.415169
## hormone_sal_end_min_since_midnight  0.0004051  0.0002578   1.572 0.116179
## race.ethnicity.5levelBlack         -0.1207041  0.3411796  -0.354 0.723532
## race.ethnicity.5levelMixed          0.2177851  0.3337039   0.653 0.514059
## race.ethnicity.5levelOther          0.0377379  0.3792899   0.099 0.920753
## race.ethnicity.5levelWhite          0.1807367  0.3138576   0.576 0.564768
## interview_age                       0.0051344  0.0057044   0.900 0.368177
## bmi                                -0.0038901  0.0118563  -0.328 0.742863
```

```
## household.income[>=200K]          -1.2088173  0.3231958  -3.740 0.000188 ***
## household.income[100K-200K]       -1.1857935  0.3025695  -3.919 9.14e-05 ***
## household.income[12K-16K]          0.0721747  0.3923152   0.184 0.854052
## household.income[16K-25K]         -0.4517416  0.3252506  -1.389 0.164994
## household.income[25K-35K]         -0.6500384  0.3249181  -2.001 0.045548 *
## household.income[35K-50K]         -0.8092992  0.3087068  -2.622 0.008809 **
## household.income[50K-75K]         -0.8795666  0.2997802  -2.934 0.003378 **
## household.income[5K-12K]          -0.2344764  0.3365730  -0.697 0.486085
## household.income[75K-100K]        -1.0339160  0.3084391  -3.352 0.000815 ***
## high.educBachelor                  0.2846554  0.3031104   0.939 0.347768
## high.educHS Diploma/GED           -0.3710171  0.3008409  -1.233 0.217599
## high.educPost Graduate Degree     -0.0389068  0.3048837  -0.128 0.898467
## high.educSome College              0.2798248  0.2885268   0.970 0.332226
## demo_race_hispanic1               -0.1252888  0.1349469  -0.928 0.353280
## ---
## Signif. codes:  0 '***' 0.001 '**' 0.01 '*' 0.05 '.' 0.1 ' ' 1
##
##
## R-sq.(adj) =  0.025
## lmer.REML =  10227  Scale est. = 2.4284    n = 2379
```

```
##                                        stdcoef      stdse
## X(Intercept)                         0.000000000 0.00000000
## Xhormone_scr_ert_mean                0.017307166 0.02123644
## Xhormone_sal_end_min_since_midnight  0.034684002 0.02206929
## Xrace.ethnicity.5levelBlack         -0.018596333 0.05256400
## Xrace.ethnicity.5levelMixed          0.034013066 0.05211695
## Xrace.ethnicity.5levelOther          0.003815594 0.03834913
## Xrace.ethnicity.5levelWhite          0.039774209 0.06906974
## Xinterview_age                       0.018584945 0.02064840
## Xbmi                                -0.007009014 0.02136224
## Xhousehold.income[>=200K]           -0.190734274 0.05099572
## Xhousehold.income[100K-200K]        -0.263466270 0.06722660
## Xhousehold.income[12K-16K]           0.004893395 0.02659871
## Xhousehold.income[16K-25K]          -0.044692595 0.03217834
## Xhousehold.income[25K-35K]          -0.068708523 0.03434357
## Xhousehold.income[35K-50K]          -0.106467989 0.04061216
## Xhousehold.income[50K-75K]          -0.146768596 0.05002272
## Xhousehold.income[5K-12K]           -0.020657584 0.02965239
## Xhousehold.income[75K-100K]         -0.176058769 0.05252206
## Xhigh.educBachelor                   0.060016941 0.06390801
## Xhigh.educHS Diploma/GED            -0.046956582 0.03807497
## Xhigh.educPost Graduate Degree      -0.008991100 0.07045658
## Xhigh.educSome College               0.058168802 0.05997774
## Xdemo_race_hispanic1                -0.023521770 0.02533499
```

1.13 Model: CBCL internalizing factor ~ Testosterone + PDS

Female participants

```
##
## Family: gaussian
## Link function: identity
```

```
##
## Formula:
## cbcl_scr_syn_internal_r ~ hormone_scr_ert_mean + hormone_sal_end_min_since_midnight +
##     PDS_score + race.ethnicity.5level + interview_age + bmi +
##     household.income + high.educ + demo_race_hispanic
##
## Parametric coefficients:
##                                     Estimate Std. Error t value Pr(>|t|)
## (Intercept)                       -1.0235183  2.2937529  -0.446 0.655483
## hormone_scr_ert_mean              -0.0039075  0.0075274  -0.519 0.603738
## hormone_sal_end_min_since_midnight  0.0003904  0.0007025   0.556 0.578475
## PDS_score                          0.6968211  0.1813164   3.843 0.000125 ***
## race.ethnicity.5levelBlack        -0.8738375  0.8173791  -1.069 0.285156
## race.ethnicity.5levelMixed         1.0856218  0.7939893   1.367 0.171673
## race.ethnicity.5levelOther        -0.3238110  0.9364448  -0.346 0.729536
## race.ethnicity.5levelWhite         1.1398878  0.7379822   1.545 0.122589
## interview_age                      0.0293430  0.0162433   1.806 0.070984 .
## bmi                                0.0418235  0.0325327   1.286 0.198726
## household.income[>=200K]          -2.7947142  0.8702052  -3.212 0.001339 **
## household.income[100K-200K]       -1.9360962  0.8074842  -2.398 0.016583 *
## household.income[12K-16K]         -0.5222649  1.0454545  -0.500 0.617437
## household.income[16K-25K]         -1.3051584  0.8981967  -1.453 0.146344
## household.income[25K-35K]         -0.4354260  0.8439344  -0.516 0.605943
## household.income[35K-50K]         -1.4569271  0.8208460  -1.775 0.076053 .
## household.income[50K-75K]         -1.4951269  0.8065764  -1.854 0.063922 .
## household.income[5K-12K]          -0.4912888  0.9246786  -0.531 0.595260
## household.income[75K-100K]        -1.5973797  0.8185850  -1.951 0.051139 .
## high.educBachelor                  1.0797204  0.7577160   1.425 0.154310
## high.educHS Diploma/GED            1.0730662  0.7599078   1.412 0.158064
## high.educPost Graduate Degree      1.5024283  0.7717551   1.947 0.051691 .
## high.educSome College              1.3854747  0.7072093   1.959 0.050232 .
## demo_race_hispanic1               -0.0519846  0.3630728  -0.143 0.886162
## ---
## Signif. codes:  0 '***' 0.001 '**' 0.01 '*' 0.05 '.' 0.1 ' ' 1
##
##
## R-sq.(adj) =  0.0237
## lmer.REML =  13534  Scale est. = 17.697    n = 2194
```

```
##                                          stdcoef      stdse
## X(Intercept)                         0.000000000 0.00000000
## Xhormone_scr_ert_mean               -0.011886357 0.02289762
## Xhormone_sal_end_min_since_midnight  0.012693788 0.02284296
## XPDS_score                           0.092402199 0.02404352
## Xrace.ethnicity.5levelBlack         -0.056721329 0.05305658
## Xrace.ethnicity.5levelMixed          0.065912398 0.04820623
## Xrace.ethnicity.5levelOther         -0.012561281 0.03632658
## Xrace.ethnicity.5levelWhite          0.099859014 0.06465038
## Xinterview_age                       0.040573429 0.02246012
## Xbmi                                 0.029342108 0.02282400
## Xhousehold.income[>=200K]           -0.162647224 0.05064434
## Xhousehold.income[100K-200K]        -0.167737187 0.06995785
## Xhousehold.income[12K-16K]          -0.014285635 0.02859656
## Xhousehold.income[16K-25K]          -0.047918631 0.03297711
```

```
## Xhousehold.income[25K-35K]          -0.019502435 0.03779925
## Xhousehold.income[35K-50K]          -0.076746496 0.04323968
## Xhousehold.income[50K-75K]          -0.094551639 0.05100779
## Xhousehold.income[5K-12K]           -0.016417904 0.03090094
## Xhousehold.income[75K-100K]         -0.106616916 0.05463636
## Xhigh.educBachelor                   0.088809939 0.06232420
## Xhigh.educHS Diploma/GED             0.053534007 0.03791091
## Xhigh.educPost Graduate Degree       0.134317593 0.06899517
## Xhigh.educSome College               0.110266615 0.05628510
## Xdemo_race_hispanic1                -0.003715466 0.02594968
```

Male participants

```
##
## Family: gaussian
## Link function: identity
##
## Formula:
## cbcl_scr_syn_internal_r ~ hormone_scr_ert_mean + hormone_sal_end_min_since_midnight +
##     PDS_score + race.ethnicity.5level + interview_age + bmi +
##     household.income + high.educ + demo_race_hispanic
##
## Parametric coefficients:
##                                     Estimate Std. Error t value Pr(>|t|)
## (Intercept)                        3.4437338  2.2451820   1.534 0.125205
## hormone_scr_ert_mean               0.0028842  0.0078665   0.367 0.713921
## hormone_sal_end_min_since_midnight  0.0011284  0.0006811   1.657 0.097734 .
## PDS_score                          0.5969949  0.2241720   2.663 0.007795 **
## race.ethnicity.5levelBlack        -0.6231398  0.8990641  -0.693 0.488316
## race.ethnicity.5levelMixed         1.0254737  0.8762589   1.170 0.242004
## race.ethnicity.5levelOther         0.1110435  0.9953849   0.112 0.911183
## race.ethnicity.5levelWhite         0.9668760  0.8243843   1.173 0.240976
## interview_age                      0.0021672  0.0150709   0.144 0.885668
## bmi                                0.0185812  0.0313908   0.592 0.553952
## household.income[>=200K]          -3.1057810  0.8524625  -3.643 0.000275 ***
## household.income[100K-200K]       -2.5616659  0.7979366  -3.210 0.001344 **
## household.income[12K-16K]         -0.1753376  1.0306145  -0.170 0.864923
## household.income[16K-25K]         -0.1202592  0.8563328  -0.140 0.888328
## household.income[25K-35K]         -0.7302804  0.8564065  -0.853 0.393898
## household.income[35K-50K]         -1.2525154  0.8134669  -1.540 0.123762
## household.income[50K-75K]         -1.7627043  0.7891305  -2.234 0.025594 *
## household.income[5K-12K]          -0.2073932  0.8835927  -0.235 0.814450
## household.income[75K-100K]        -2.6771949  0.8131444  -3.292 0.001008 **
## high.educBachelor                  1.1555065  0.7968814   1.450 0.147182
## high.educHS Diploma/GED           -0.8403741  0.7910065  -1.062 0.288158
## high.educPost Graduate Degree      0.3738810  0.8013958   0.467 0.640874
## high.educSome College              0.7371071  0.7592100   0.971 0.331704
## demo_race_hispanic1               -0.0532883  0.3568215  -0.149 0.881297
## ---
## Signif. codes:  0 '***' 0.001 '**' 0.01 '*' 0.05 '.' 0.1 ' ' 1
##
##
## R-sq.(adj) =  0.0319
```

```
## lmer.REML =  14771  Scale est. = 15.906    n = 2379

##                                        stdcoef      stdse
## X(Intercept)                         0.000000000 0.00000000
## Xhormone_scr_ert_mean                0.007764948 0.02117874
## Xhormone_sal_end_min_since_midnight  0.036550456 0.02206353
## XPDS_score                           0.057753978 0.02168666
## Xrace.ethnicity.5levelBlack         -0.036320913 0.05240370
## Xrace.ethnicity.5levelMixed          0.060590997 0.05177452
## Xrace.ethnicity.5levelOther          0.004247596 0.03807511
## Xrace.ethnicity.5levelWhite          0.080499263 0.06863582
## Xinterview_age                       0.002967877 0.02063842
## Xbmi                                 0.012665912 0.02139756
## Xhousehold.income[>=200K]           -0.185397889 0.05088728
## Xhousehold.income[100K-200K]        -0.215329901 0.06707339
## Xhousehold.income[12K-16K]          -0.004497450 0.02643551
## Xhousehold.income[16K-25K]          -0.004501215 0.03205192
## Xhousehold.income[25K-35K]          -0.029202978 0.03424660
## Xhousehold.income[35K-50K]          -0.062338871 0.04048701
## Xhousehold.income[50K-75K]          -0.111278113 0.04981718
## Xhousehold.income[5K-12K]           -0.006912592 0.02945089
## Xhousehold.income[75K-100K]         -0.172471880 0.05238488
## Xhigh.educBachelor                   0.092170673 0.06356442
## Xhigh.educHS Diploma/GED            -0.040238440 0.03787465
## Xhigh.educPost Graduate Degree       0.032687877 0.07006487
## Xhigh.educSome College               0.057969620 0.05970790
## Xdemo_race_hispanic1                -0.003784907 0.02534397
```

1.14 Model: CBCL internalizing factor ~ Testosterone + Pubertal category

Female participants

```
##
## Family: gaussian
## Link function: identity
##
## Formula:
## cbcl_scr_syn_internal_r ~ hormone_scr_ert_mean + hormone_sal_end_min_since_midnight +
##     pds_p_ss_category + race.ethnicity.5level + interview_age +
##     bmi + household.income + high.educ + demo_race_hispanic
##
## Parametric coefficients:
##                                      Estimate Std. Error t value Pr(>|t|)
## (Intercept)                        -0.4791156  2.3737499  -0.202  0.84006
## hormone_scr_ert_mean               -0.0018296  0.0075063  -0.244  0.80745
## hormone_sal_end_min_since_midnight  0.0003195  0.0007049   0.453  0.65037
## pds_p_ss_categoryEarly              0.3199518  0.3157908   1.013  0.31109
## pds_p_ss_categoryLate               0.7413169  0.8198696   0.904  0.36600
## pds_p_ss_categoryMid                0.8521830  0.3109844   2.740  0.00619 **
## race.ethnicity.5levelBlack         -0.7575458  0.8179247  -0.926  0.35446
## race.ethnicity.5levelMixed          1.1289051  0.7953866   1.419  0.15595
## race.ethnicity.5levelOther         -0.2785297  0.9380016  -0.297  0.76654
## race.ethnicity.5levelWhite          1.1648902  0.7392110   1.576  0.11520
```

```
## interview_age                          0.0314119  0.0166281   1.889  0.05901 .
## bmi                                    0.0361033  0.0337279   1.070  0.28455
## household.income[>=200K]              -2.8229835  0.8727427  -3.235  0.00124 **
## household.income[100K-200K]           -1.9541412  0.8100575  -2.412  0.01593 *
## household.income[12K-16K]             -0.6780736  1.0467838  -0.648  0.51720
## household.income[16K-25K]             -1.3046423  0.9003999  -1.449  0.14749
## household.income[25K-35K]             -0.5202213  0.8459213  -0.615  0.53864
## household.income[35K-50K]             -1.5206784  0.8221872  -1.850  0.06451 .
## household.income[50K-75K]             -1.5254036  0.8084430  -1.887  0.05932 .
## household.income[5K-12K]              -0.5169751  0.9266703  -0.558  0.57698
## household.income[75K-100K]            -1.6452994  0.8204060  -2.005  0.04504 *
## high.educBachelor                      1.0535667  0.7602749   1.386  0.16596
## high.educHS Diploma/GED                1.0940361  0.7613975   1.437  0.15090
## high.educPost Graduate Degree          1.4814336  0.7745445   1.913  0.05592 .
## high.educSome College                  1.4376086  0.7093335   2.027  0.04281 *
## demo_race_hispanic1                   -0.1029159  0.3635832  -0.283  0.77716
## ---
## Signif. codes:  0 '***' 0.001 '**' 0.01 '*' 0.05 '.' 0.1 ' ' 1
##
##
## R-sq.(adj) =  0.0204
## lmer.REML =  13540  Scale est. = 17.955    n = 2194
```

```
##                                          stdcoef      stdse
## X(Intercept)                         0.000000000 0.00000000
## Xhormone_scr_ert_mean               -0.005565492 0.02283364
## Xhormone_sal_end_min_since_midnight  0.010389833 0.02292002
## Xpds_p_ss_categoryEarly              0.025202782 0.02487502
## Xpds_p_ss_categoryLate               0.020677457 0.02286852
## Xpds_p_ss_categoryMid                0.077669759 0.02834378
## Xrace.ethnicity.5levelBlack         -0.049172764 0.05309199
## Xrace.ethnicity.5levelMixed          0.068540298 0.04829107
## Xrace.ethnicity.5levelOther         -0.010804729 0.03638697
## Xrace.ethnicity.5levelWhite          0.102049329 0.06475802
## Xinterview_age                       0.043434253 0.02299212
## Xbmi                                 0.025329038 0.02366251
## Xhousehold.income[>=200K]           -0.164292446 0.05079202
## Xhousehold.income[100K-200K]        -0.169300541 0.07018079
## Xhousehold.income[12K-16K]          -0.018547508 0.02863293
## Xhousehold.income[16K-25K]          -0.047899680 0.03305800
## Xhousehold.income[25K-35K]          -0.023300360 0.03788824
## Xhousehold.income[35K-50K]          -0.080104722 0.04331032
## Xhousehold.income[50K-75K]          -0.096466329 0.05112584
## Xhousehold.income[5K-12K]           -0.017276288 0.03096750
## Xhousehold.income[75K-100K]         -0.109815308 0.05475790
## Xhigh.educBachelor                   0.086658727 0.06253468
## Xhigh.educHS Diploma/GED             0.054580174 0.03798523
## Xhigh.educPost Graduate Degree       0.132440660 0.06924454
## Xhigh.educSome College               0.114415826 0.05645416
## Xdemo_race_hispanic1                -0.007355643 0.02598615
```

Male participants

```
##
```

```
## Family: gaussian
## Link function: identity
##
## Formula:
## cbcl_scr_syn_internal_r ~ hormone_scr_ert_mean + hormone_sal_end_min_since_midnight +
##     pds_p_ss_category + race.ethnicity.5level + interview_age +
##     bmi + household.income + high.educ + demo_race_hispanic
##
## Parametric coefficients:
##                                     Estimate Std. Error t value Pr(>|t|)
## (Intercept)                        3.6689461  2.2514873   1.630 0.103327
## hormone_scr_ert_mean               0.0037245  0.0078567   0.474 0.635504
## hormone_sal_end_min_since_midnight  0.0011237  0.0006818   1.648 0.099446 .
## pds_p_ss_categoryEarly             0.4012560  0.2801908   1.432 0.152253
## pds_p_ss_categoryLate              1.5054973  1.9555287   0.770 0.441456
## pds_p_ss_categoryMid               1.1642074  0.5326569   2.186 0.028939 *
## race.ethnicity.5levelBlack        -0.5701081  0.8988333  -0.634 0.525963
## race.ethnicity.5levelMixed         1.0879670  0.8773825   1.240 0.215094
## race.ethnicity.5levelOther         0.1394460  0.9965038   0.140 0.888723
## race.ethnicity.5levelWhite         1.0481425  0.8257990   1.269 0.204479
## interview_age                      0.0039505  0.0150401   0.263 0.792834
## bmi                                0.0221825  0.0313074   0.709 0.478681
## household.income[>=200K]          -3.1120643  0.8542964  -3.643 0.000275 ***
## household.income[100K-200K]       -2.5644850  0.7998515  -3.206 0.001363 **
## household.income[12K-16K]         -0.1777668  1.0333340  -0.172 0.863427
## household.income[16K-25K]         -0.1266382  0.8583305  -0.148 0.882718
## household.income[25K-35K]         -0.7123444  0.8608932  -0.827 0.408067
## household.income[35K-50K]         -1.2576409  0.8157513  -1.542 0.123282
## household.income[50K-75K]         -1.7625261  0.7905309  -2.230 0.025872 *
## household.income[5K-12K]          -0.1966829  0.8854897  -0.222 0.824242
## household.income[75K-100K]        -2.6915757  0.8147083  -3.304 0.000968 ***
## high.educBachelor                  1.2082553  0.7974585   1.515 0.129873
## high.educHS Diploma/GED           -0.8177549  0.7922924  -1.032 0.302114
## high.educPost Graduate Degree      0.4255426  0.8020039   0.531 0.595747
## high.educSome College              0.7704997  0.7601576   1.014 0.310875
## demo_race_hispanic1               -0.0697534  0.3573940  -0.195 0.845275
## ---
## Signif. codes:  0 '***' 0.001 '**' 0.01 '*' 0.05 '.' 0.1 ' ' 1
##
##
## R-sq.(adj) =  0.0308
## lmer.REML =  14768  Scale est. = 15.928    n = 2379
```

```
##                                          stdcoef      stdse
## X(Intercept)                         0.000000000 0.00000000
## Xhormone_scr_ert_mean                0.010027471 0.02115251
## Xhormone_sal_end_min_since_midnight  0.036399292 0.02208436
## Xpds_p_ss_categoryEarly              0.030153890 0.02105599
## Xpds_p_ss_categoryLate               0.015740051 0.02044515
## Xpds_p_ss_categoryMid                0.046911323 0.02146322
## Xrace.ethnicity.5levelBlack         -0.033229858 0.05239024
## Xrace.ethnicity.5levelMixed          0.064283468 0.05184090
## Xrace.ethnicity.5levelOther          0.005334038 0.03811791
## Xrace.ethnicity.5levelWhite          0.087265268 0.06875360
```

```
## Xinterview_age                      0.005409874 0.02059629
## Xbmi                                0.015120703 0.02134070
## Xhousehold.income[>=200K]          -0.185772967 0.05099675
## Xhousehold.income[100K-200K]       -0.215566872 0.06723435
## Xhousehold.income[12K-16K]         -0.004559762 0.02650526
## Xhousehold.income[16K-25K]         -0.004739978 0.03212669
## Xhousehold.income[25K-35K]         -0.028485738 0.03442601
## Xhousehold.income[35K-50K]         -0.062593972 0.04060071
## Xhousehold.income[50K-75K]         -0.111266863 0.04990558
## Xhousehold.income[5K-12K]          -0.006555606 0.02951412
## Xhousehold.income[75K-100K]        -0.173398324 0.05248563
## Xhigh.educBachelor                  0.096378257 0.06361045
## Xhigh.educHS Diploma/GED           -0.039155399 0.03793621
## Xhigh.educPost Graduate Degree      0.037204568 0.07011804
## Xhigh.educSome College              0.060595773 0.05978242
## Xdemo_race_hispanic1               -0.004954374 0.02538463
```

1.15 Model: CBCL Anxious-Depressed ~ Testosterone + PDS

Female participants

```
##
## Family: gaussian
## Link function: identity
##
## Formula:
## cbcl_scr_syn_anxdep_r ~ hormone_scr_ert_mean + hormone_sal_end_min_since_midnight +
##     PDS_score + race.ethnicity.5level + interview_age + bmi +
##     household.income + high.educ + demo_race_hispanic
##
## Parametric coefficients:
##                                      Estimate Std. Error t value Pr(>|t|)
## (Intercept)                        -0.1474743  1.3006223  -0.113  0.90973
## hormone_scr_ert_mean               -0.0020267  0.0042730  -0.474  0.63532
## hormone_sal_end_min_since_midnight  0.0003520  0.0003967   0.887  0.37504
## PDS_score                           0.3139131  0.1027613   3.055  0.00228 **
## race.ethnicity.5levelBlack         -0.2742696  0.4607915  -0.595  0.55176
## race.ethnicity.5levelMixed          0.7800913  0.4478394   1.742  0.08167 .
## race.ethnicity.5levelOther          0.0738792  0.5284416   0.140  0.88883
## race.ethnicity.5levelWhite          0.6910203  0.4160889   1.661  0.09691 .
## interview_age                       0.0123825  0.0092363   1.341  0.18018
## bmi                                -0.0031050  0.0184162  -0.169  0.86613
## household.income[>=200K]           -1.0108430  0.4907583  -2.060  0.03954 *
## household.income[100K-200K]        -0.5181084  0.4552856  -1.138  0.25525
## household.income[12K-16K]          -0.1029896  0.5888390  -0.175  0.86117
## household.income[16K-25K]         -0.5560220  0.5073000  -1.096  0.27318
## household.income[25K-35K]           0.0186861  0.4759666   0.039  0.96869
## household.income[35K-50K]          -0.4055175  0.4630561  -0.876  0.38127
## household.income[50K-75K]          -0.3051537  0.4547577  -0.671  0.50228
## household.income[5K-12K]           -0.1680152  0.5229856  -0.321  0.74804
## household.income[75K-100K]         -0.3409762  0.4616468  -0.739  0.46022
## high.educBachelor                   0.1972908  0.4270731   0.462  0.64416
## high.educHS Diploma/GED             0.1063651  0.4288322   0.248  0.80413
```

```
## high.educPost Graduate Degree       0.6494771  0.4350427   1.493  0.13561
## high.educSome College               0.4468687  0.3987839   1.121  0.26259
## demo_race_hispanic1                 0.0883378  0.2040670   0.433  0.66514
## ---
## Signif. codes:  0 '***' 0.001 '**' 0.01 '*' 0.05 '.' 0.1 ' ' 1
##
##
## R-sq.(adj) =  0.0158
## lmer.REML =  11077  Scale est. = 6.9027    n = 2194
```

```
##                                         stdcoef      stdse
## X(Intercept)                        0.000000000 0.00000000
## Xhormone_scr_ert_mean              -0.010934720 0.02305375
## Xhormone_sal_end_min_since_midnight  0.020299057 0.02287848
## XPDS_score                          0.073830231 0.02416876
## Xrace.ethnicity.5levelBlack        -0.031576004 0.05304983
## Xrace.ethnicity.5levelMixed         0.084003581 0.04822527
## Xrace.ethnicity.5levelOther         0.005083095 0.03635827
## Xrace.ethnicity.5levelWhite         0.107369214 0.06465099
## Xinterview_age                      0.030367528 0.02265171
## Xbmi                               -0.003863601 0.02291580
## Xhousehold.income[>=200K]          -0.104341438 0.05065715
## Xhousehold.income[100K-200K]       -0.079613529 0.06996006
## Xhousehold.income[12K-16K]         -0.004996501 0.02856730
## Xhousehold.income[16K-25K]         -0.036207372 0.03303466
## Xhousehold.income[25K-35K]          0.001484422 0.03781071
## Xhousehold.income[35K-50K]         -0.037887343 0.04326316
## Xhousehold.income[50K-75K]         -0.034227365 0.05100760
## Xhousehold.income[5K-12K]          -0.009958486 0.03099805
## Xhousehold.income[75K-100K]        -0.040365089 0.05465018
## Xhigh.educBachelor                  0.028782003 0.06230405
## Xhigh.educHS Diploma/GED            0.009411660 0.03794499
## Xhigh.educPost Graduate Degree      0.102983306 0.06898185
## Xhigh.educSome College              0.063079648 0.05629203
## Xdemo_race_hispanic1                0.011198212 0.02586871
```

Male participants

```
##
## Family: gaussian
## Link function: identity
##
## Formula:
## cbcl_scr_syn_anxdep_r ~ hormone_scr_ert_mean + hormone_sal_end_min_since_midnight +
##     PDS_score + race.ethnicity.5level + interview_age + bmi +
##     household.income + high.educ + demo_race_hispanic
##
## Parametric coefficients:
##                                     Estimate Std. Error t value Pr(>|t|)
## (Intercept)                        2.7312371  1.2650644   2.159  0.03095 *
## hormone_scr_ert_mean               0.0025593  0.0044308   0.578  0.56358
## hormone_sal_end_min_since_midnight  0.0002587  0.0003837   0.674  0.50018
## PDS_score                          0.3148247  0.1264288   2.490  0.01284 *
```

```
## race.ethnicity.5levelBlack         -0.2418499  0.5050002  -0.479  0.63205
## race.ethnicity.5levelMixed          0.4790700  0.4921511   0.973  0.33044
## race.ethnicity.5levelOther          0.2721536  0.5601296   0.486  0.62710
## race.ethnicity.5levelWhite          0.6236600  0.4631967   1.346  0.17829
## interview_age                      -0.0104126  0.0085151  -1.223  0.22151
## bmi                                -0.0044218  0.0176953  -0.250  0.80270
## household.income[>=200K]           -1.2457309  0.4780094  -2.606  0.00922 **
## household.income[100K-200K]        -0.9651871  0.4476459  -2.156  0.03117 *
## household.income[12K-16K]           0.0793923  0.5777511   0.137  0.89071
## household.income[16K-25K]          -0.1493286  0.4807007  -0.311  0.75610
## household.income[25K-35K]          -0.2492141  0.4806234  -0.519  0.60414
## household.income[35K-50K]          -0.3512328  0.4562470  -0.770  0.44148
## household.income[50K-75K]          -0.7743932  0.4427403  -1.749  0.08041 .
## household.income[5K-12K]            0.0179599  0.4955765   0.036  0.97109
## household.income[75K-100K]         -0.9493947  0.4560868  -2.082  0.03749 *
## high.educBachelor                   1.0384365  0.4470564   2.323  0.02027 *
## high.educHS Diploma/GED            -0.1351437  0.4439215  -0.304  0.76083
## high.educPost Graduate Degree       0.6959693  0.4495474   1.548  0.12172
## high.educSome College               0.6287425  0.4260991   1.476  0.14019
## demo_race_hispanic1                 0.0907242  0.2001789   0.453  0.65044
## ---
## Signif. codes:  0 '***' 0.001 '**' 0.01 '*' 0.05 '.' 0.1 ' ' 1
##
##
## R-sq.(adj) =  0.0155
## lmer.REML =  12074  Scale est. = 6.4306    n = 2379

##                                          stdcoef      stdse
## X(Intercept)                         0.000000000 0.00000000
## Xhormone_scr_ert_mean                0.012383974 0.02144009
## Xhormone_sal_end_min_since_midnight  0.015062238 0.02233745
## XPDS_score                           0.054739570 0.02198257
## Xrace.ethnicity.5levelBlack         -0.025336016 0.05290346
## Xrace.ethnicity.5levelMixed          0.050874920 0.05226407
## Xrace.ethnicity.5levelOther          0.018710503 0.03850879
## Xrace.ethnicity.5levelWhite          0.093323317 0.06931190
## Xinterview_age                      -0.025628197 0.02095794
## Xbmi                                -0.005417284 0.02167907
## Xhousehold.income[>=200K]           -0.133653205 0.05128515
## Xhousehold.income[100K-200K]        -0.145819148 0.06762973
## Xhousehold.income[12K-16K]           0.003660082 0.02663504
## Xhousehold.income[16K-25K]          -0.010045594 0.03233757
## Xhousehold.income[25K-35K]          -0.017911474 0.03454328
## Xhousehold.income[35K-50K]          -0.031418986 0.04081287
## Xhousehold.income[50K-75K]          -0.087864414 0.05023433
## Xhousehold.income[5K-12K]            0.001075901 0.02968782
## Xhousehold.income[75K-100K]         -0.109927495 0.05280889
## Xhigh.educBachelor                   0.148874903 0.06409201
## Xhigh.educHS Diploma/GED            -0.011630151 0.03820286
## Xhigh.educPost Graduate Degree       0.109361528 0.07063988
## Xhigh.educSome College               0.088871775 0.06022845
## Xdemo_race_hispanic1                 0.011581578 0.02555425
```

1.16 Model: CBCL Anxious-Depressed ~ Testosterone + Pubertal category

Female participants

```
##
## Family: gaussian
## Link function: identity
##
## Formula:
## cbcl_scr_syn_anxdep_r ~ hormone_scr_ert_mean + hormone_sal_end_min_since_midnight +
##     pds_p_ss_category + race.ethnicity.5level + interview_age +
##     bmi + household.income + high.educ + demo_race_hispanic
##
## Parametric coefficients:
##                                     Estimate Std. Error t value Pr(>|t|)
## (Intercept)                        0.1130945  1.3447302   0.084   0.9330
## hormone_scr_ert_mean              -0.0010164  0.0042580  -0.239   0.8114
## hormone_sal_end_min_since_midnight  0.0003058  0.0003977   0.769   0.4420
## pds_p_ss_categoryEarly             0.2452367  0.1794051   1.367   0.1718
## pds_p_ss_categoryLate              0.2714538  0.4653101   0.583   0.5597
## pds_p_ss_categoryMid               0.4063780  0.1763814   2.304   0.0213 *
## race.ethnicity.5levelBlack        -0.2162464  0.4608365  -0.469   0.6389
## race.ethnicity.5levelMixed         0.7990085  0.4483636   1.782   0.0749 .
## race.ethnicity.5levelOther         0.0908101  0.5290057   0.172   0.8637
## race.ethnicity.5levelWhite         0.7024600  0.4165245   1.686   0.0918 .
## interview_age                      0.0130394  0.0094450   1.381   0.1676
## bmi                               -0.0060622  0.0190825  -0.318   0.7508
## household.income[>=200K]          -1.0276877  0.4918901  -2.089   0.0368 *
## household.income[100K-200K]       -0.5302331  0.4564655  -1.162   0.2455
## household.income[12K-16K]         -0.1829693  0.5892303  -0.311   0.7562
## household.income[16K-25K]         -0.5483277  0.5082410  -1.079   0.2808
## household.income[25K-35K]         -0.0221415  0.4768142  -0.046   0.9630
## household.income[35K-50K]         -0.4321851  0.4635323  -0.932   0.3512
## household.income[50K-75K]         -0.3207558  0.4555353  -0.704   0.4814
## household.income[5K-12K]          -0.1752306  0.5237732  -0.335   0.7380
## household.income[75K-100K]        -0.3633979  0.4623972  -0.786   0.4320
## high.educBachelor                  0.1876197  0.4283013   0.438   0.6614
## high.educHS Diploma/GED            0.1186534  0.4294391   0.276   0.7823
## high.educPost Graduate Degree      0.6420506  0.4364024   1.471   0.1414
## high.educSome College              0.4743118  0.3997786   1.186   0.2356
## demo_race_hispanic1                0.0647933  0.2042215   0.317   0.7511
## ---
## Signif. codes:  0 '***' 0.001 '**' 0.01 '*' 0.05 '.' 0.1 ' ' 1
##
##
## R-sq.(adj) =  0.0137
## lmer.REML =  11081  Scale est. = 6.96      n = 2194
```

```
##                                         stdcoef      stdse
## X(Intercept)                        0.000000000 0.00000000
## Xhormone_scr_ert_mean              -0.005483465 0.02297319
## Xhormone_sal_end_min_since_midnight  0.017637669 0.02293656
## Xpds_p_ss_categoryEarly             0.034262044 0.02506470
## Xpds_p_ss_categoryLate              0.013429285 0.02301969
```

```
## Xpds_p_ss_categoryMid               0.065692098 0.02851253
## Xrace.ethnicity.5levelBlack        -0.024895938 0.05305501
## Xrace.ethnicity.5levelMixed         0.086040668 0.04828172
## Xrace.ethnicity.5levelOther         0.006247987 0.03639708
## Xrace.ethnicity.5levelWhite         0.109146702 0.06471867
## Xinterview_age                      0.031978540 0.02316334
## Xbmi                               -0.007543393 0.02374494
## Xhousehold.income[>=200K]          -0.106080182 0.05077398
## Xhousehold.income[100K-200K]       -0.081476639 0.07014136
## Xhousehold.income[12K-16K]         -0.008876686 0.02858628
## Xhousehold.income[16K-25K]         -0.035706327 0.03309594
## Xhousehold.income[25K-35K]         -0.001758914 0.03787804
## Xhousehold.income[35K-50K]         -0.040378886 0.04330765
## Xhousehold.income[50K-75K]         -0.035977363 0.05109482
## Xhousehold.income[5K-12K]          -0.010386153 0.03104473
## Xhousehold.income[75K-100K]        -0.043019381 0.05473902
## Xhigh.educBachelor                  0.027371119 0.06248323
## Xhigh.educHS Diploma/GED            0.010498987 0.03799870
## Xhigh.educPost Graduate Degree      0.101805733 0.06919745
## Xhigh.educSome College              0.066953493 0.05643243
## Xdemo_race_hispanic1                0.008213569 0.02588830
```

Male participants

```
##
## Family: gaussian
## Link function: identity
##
## Formula:
## cbcl_scr_syn_anxdep_r ~ hormone_scr_ert_mean + hormone_sal_end_min_since_midnight +
##     pds_p_ss_category + race.ethnicity.5level + interview_age +
##     bmi + household.income + high.educ + demo_race_hispanic
##
## Parametric coefficients:
##                                     Estimate Std. Error t value Pr(>|t|)
## (Intercept)                         2.838562   1.268403   2.238  0.02532 *
## hormone_scr_ert_mean                0.003015   0.004425   0.681  0.49578
## hormone_sal_end_min_since_midnight  0.000257   0.000384   0.669  0.50336
## pds_p_ss_categoryEarly              0.187926   0.157966   1.190  0.23430
## pds_p_ss_categoryLate               0.814948   1.098624   0.742  0.45829
## pds_p_ss_categoryMid                0.671699   0.300350   2.236  0.02542 *
## race.ethnicity.5levelBlack         -0.216197   0.504815  -0.428  0.66849
## race.ethnicity.5levelMixed          0.515636   0.492719   1.047  0.29543
## race.ethnicity.5levelOther          0.288673   0.560685   0.515  0.60670
## race.ethnicity.5levelWhite          0.668739   0.463935   1.441  0.14959
## interview_age                      -0.009429   0.008496  -1.110  0.26721
## bmi                                -0.002472   0.017645  -0.140  0.88861
## household.income[>=200K]           -1.243759   0.478953  -2.597  0.00947 **
## household.income[100K-200K]        -0.961957   0.448636  -2.144  0.03212 *
## household.income[12K-16K]           0.079340   0.579178   0.137  0.89105
## household.income[16K-25K]          -0.144549   0.481751  -0.300  0.76417
## household.income[25K-35K]          -0.229036   0.483028  -0.474  0.63543
## household.income[35K-50K]          -0.347642   0.457452  -0.760  0.44736
```

```
## household.income[50K-75K]          -0.769647   0.443453  -1.736  0.08277 .
## household.income[5K-12K]            0.025933   0.496567   0.052  0.95835
## household.income[75K-100K]         -0.951304   0.456890  -2.082  0.03744 *
## high.educBachelor                   1.066659   0.447317   2.385  0.01718 *
## high.educHS Diploma/GED            -0.126485   0.444581  -0.285  0.77605
## high.educPost Graduate Degree       0.724044   0.449820   1.610  0.10761
## high.educSome College               0.645202   0.426566   1.513  0.13053
## demo_race_hispanic1                 0.080460   0.200474   0.401  0.68820
## ---
## Signif. codes:  0 '***' 0.001 '**' 0.01 '*' 0.05 '.' 0.1 ' ' 1
##
##
## R-sq.(adj) =  0.0146
## lmer.REML =  12072  Scale est. = 6.4397    n = 2379
```

```
##                                          stdcoef      stdse
## X(Intercept)                         0.000000000 0.00000000
## Xhormone_scr_ert_mean                0.014587245 0.02141269
## Xhormone_sal_end_min_since_midnight  0.014963554 0.02235634
## Xpds_p_ss_categoryEarly              0.025382267 0.02133572
## Xpds_p_ss_categoryLate               0.015313597 0.02064412
## Xpds_p_ss_categoryMid                0.048645594 0.02175182
## Xrace.ethnicity.5levelBlack         -0.022648588 0.05288408
## Xrace.ethnicity.5levelMixed          0.054757999 0.05232433
## Xrace.ethnicity.5levelOther          0.019846236 0.03854698
## Xrace.ethnicity.5levelWhite          0.100068830 0.06942237
## Xinterview_age                      -0.023207254 0.02091149
## Xbmi                                -0.003028270 0.02161754
## Xhousehold.income[>=200K]           -0.133441668 0.05138642
## Xhousehold.income[100K-200K]        -0.145331155 0.06777925
## Xhousehold.income[12K-16K]           0.003657676 0.02670083
## Xhousehold.income[16K-25K]          -0.009724045 0.03240820
## Xhousehold.income[25K-35K]          -0.016461208 0.03471612
## Xhousehold.income[35K-50K]          -0.031097783 0.04092061
## Xhousehold.income[50K-75K]          -0.087325948 0.05031523
## Xhousehold.income[5K-12K]            0.001553518 0.02974713
## Xhousehold.income[75K-100K]         -0.110148564 0.05290189
## Xhigh.educBachelor                   0.152921063 0.06412931
## Xhigh.educHS Diploma/GED            -0.010885013 0.03825964
## Xhigh.educPost Graduate Degree       0.113773145 0.07068265
## Xhigh.educSome College               0.091198303 0.06029441
## Xdemo_race_hispanic1                 0.010271323 0.02559198
```

1.17 Model: CBCL Withdrawn-Depressed ~ Testosterone + PDS

Female participants

```
##
## Family: gaussian
## Link function: identity
##
## Formula:
## cbcl_scr_syn_withdep_r ~ hormone_scr_ert_mean + hormone_sal_end_min_since_midnight +
```

```
##     PDS_score + race.ethnicity.5level + interview_age + bmi +
##     household.income + high.educ + demo_race_hispanic
##
## Parametric coefficients:
##                                    Estimate Std. Error t value Pr(>|t|)
## (Intercept)                       0.4749241  0.6774111   0.701 0.483324
## hormone_scr_ert_mean              0.0028502  0.0022259   1.280 0.200527
## hormone_sal_end_min_since_midnight -0.0000571  0.0002066  -0.276 0.782284
## PDS_score                         0.1591141  0.0534745   2.976 0.002957 **
## race.ethnicity.5levelBlack       -0.4903843  0.2390417  -2.051 0.040342 *
## race.ethnicity.5levelMixed       -0.0292756  0.2322633  -0.126 0.899708
## race.ethnicity.5levelOther       -0.3485297  0.2739778  -1.272 0.203471
## race.ethnicity.5levelWhite       -0.0453880  0.2158951  -0.210 0.833507
## interview_age                     0.0032131  0.0048217   0.666 0.505239
## bmi                               0.0134666  0.0095711   1.407 0.159568
## household.income[>=200K]         -0.9084967  0.2541282  -3.575 0.000358 ***
## household.income[100K-200K]      -0.6695469  0.2356401  -2.841 0.004534 **
## household.income[12K-16K]        -0.3862945  0.3043186  -1.269 0.204443
## household.income[16K-25K]        -0.3259204  0.2631970  -1.238 0.215734
## household.income[25K-35K]        -0.1524759  0.2463780  -0.619 0.536067
## household.income[35K-50K]        -0.5910347  0.2398287  -2.464 0.013801 *
## household.income[50K-75K]        -0.5671819  0.2353323  -2.410 0.016029 *
## household.income[5K-12K]         -0.1308079  0.2715993  -0.482 0.630124
## household.income[75K-100K]       -0.5921637  0.2389930  -2.478 0.013297 *
## high.educBachelor                 0.1356108  0.2210308   0.614 0.539585
## high.educHS Diploma/GED           0.4057324  0.2223492   1.825 0.068176 .
## high.educPost Graduate Degree     0.1914676  0.2252086   0.850 0.395319
## high.educSome College             0.2064858  0.2064999   1.000 0.317455
## demo_race_hispanic1              -0.0060614  0.1056212  -0.057 0.954241
## ---
## Signif. codes:  0 '***' 0.001 '**' 0.01 '*' 0.05 '.' 0.1 ' ' 1
##
##
## R-sq.(adj) =  0.0237
## lmer.REML = 8254.3  Scale est. = 2.269     n = 2194

##                                         stdcoef      stdse
## X(Intercept)                        0.000000000 0.00000000
## Xhormone_scr_ert_mean               0.029448697 0.02299885
## Xhormone_sal_end_min_since_midnight -0.006306394 0.02281798
## XPDS_score                          0.071667214 0.02408568
## Xrace.ethnicity.5levelBlack        -0.108119312 0.05270361
## Xrace.ethnicity.5levelMixed        -0.006037337 0.04789830
## Xrace.ethnicity.5levelOther        -0.045923296 0.03610012
## Xrace.ethnicity.5levelWhite        -0.013505701 0.06424197
## Xinterview_age                      0.015090769 0.02264590
## Xbmi                                0.032090664 0.02280774
## Xhousehold.income[>=200K]          -0.179590604 0.05023578
## Xhousehold.income[100K-200K]       -0.197030918 0.06934299
## Xhousehold.income[12K-16K]         -0.035890397 0.02827406
## Xhousehold.income[16K-25K]         -0.040644677 0.03282261
## Xhousehold.income[25K-35K]         -0.023196717 0.03748240
## Xhousehold.income[35K-50K]         -0.105751061 0.04291142
## Xhousehold.income[50K-75K]         -0.121832831 0.05055028
```

```
## Xhousehold.income[5K-12K]           -0.014847923 0.03082907
## Xhousehold.income[75K-100K]         -0.134248900 0.05418189
## Xhigh.educBachelor                   0.037887458 0.06175243
## Xhigh.educHS Diploma/GED             0.068753363 0.03767817
## Xhigh.educPost Graduate Degree       0.058141382 0.06838723
## Xhigh.educSome College               0.055819577 0.05582339
## Xdemo_race_hispanic1                -0.001471514 0.02564132
```

Male participants

```
##
## Family: gaussian
## Link function: identity
##
## Formula:
## cbcl_scr_syn_withdep_r ~ hormone_scr_ert_mean + hormone_sal_end_min_since_midnight +
##     PDS_score + race.ethnicity.5level + interview_age + bmi +
##     household.income + high.educ + demo_race_hispanic
##
## Parametric coefficients:
##                                     Estimate Std. Error t value Pr(>|t|)
## (Intercept)                         0.1662964  0.7336302   0.227 0.820695
## hormone_scr_ert_mean                0.0030111  0.0025615   1.176 0.239905
## hormone_sal_end_min_since_midnight  0.0005049  0.0002147   2.352 0.018749 *
## PDS_score                           0.1249157  0.0736918   1.695 0.090187 .
## race.ethnicity.5levelBlack         -0.1685618  0.2933680  -0.575 0.565634
## race.ethnicity.5levelMixed          0.2736702  0.2863042   0.956 0.339235
## race.ethnicity.5levelOther          0.0409310  0.3260456   0.126 0.900109
## race.ethnicity.5levelWhite          0.1336041  0.2689224   0.497 0.619367
## interview_age                       0.0105336  0.0049453   2.130 0.033272 *
## bmi                                -0.0037954  0.0103120  -0.368 0.712866
## household.income[>=200K]           -1.0743043  0.2778328  -3.867 0.000113 ***
## household.income[100K-200K]        -0.9198702  0.2606151  -3.530 0.000424 ***
## household.income[12K-16K]           0.0168451  0.3372803   0.050 0.960171
## household.income[16K-25K]           0.0351614  0.2803377   0.125 0.900198
## household.income[25K-35K]          -0.2660585  0.2802901  -0.949 0.342603
## household.income[35K-50K]          -0.5133837  0.2662264  -1.928 0.053929 .
## household.income[50K-75K]          -0.6828431  0.2580982  -2.646 0.008207 **
## household.income[5K-12K]           -0.0679823  0.2894138  -0.235 0.814309
## household.income[75K-100K]         -0.9692972  0.2658004  -3.647 0.000271 ***
## high.educBachelor                  -0.0710885  0.2601255  -0.273 0.784658
## high.educHS Diploma/GED            -0.5687296  0.2582706  -2.202 0.027757 *
## high.educPost Graduate Degree      -0.3417871  0.2617167  -1.306 0.191699
## high.educSome College              -0.1515277  0.2477497  -0.612 0.540851
## demo_race_hispanic1                -0.1496095  0.1122697  -1.333 0.182795
## ---
## Signif. codes:  0 '***' 0.001 '**' 0.01 '*' 0.05 '.' 0.1 ' ' 1
##
##
## R-sq.(adj) =  0.0393
## lmer.REML = 9527.6  Scale est. = 2.0699    n = 2379
```

```
##                                          stdcoef      stdse
```

```
## X(Intercept)                         0.000000000 0.00000000
## Xhormone_scr_ert_mean                0.024764079 0.02106645
## Xhormone_sal_end_min_since_midnight  0.049961021 0.02124085
## XPDS_score                           0.036915454 0.02177761
## Xrace.ethnicity.5levelBlack         -0.030013049 0.05223525
## Xrace.ethnicity.5levelMixed          0.049395895 0.05167625
## Xrace.ethnicity.5levelOther          0.004782801 0.03809853
## Xrace.ethnicity.5levelWhite          0.033979788 0.06839553
## Xinterview_age                       0.044064971 0.02068746
## Xbmi                                -0.007903102 0.02147270
## Xhousehold.income[>=200K]           -0.195902944 0.05066373
## Xhousehold.income[100K-200K]        -0.236204468 0.06692081
## Xhousehold.income[12K-16K]           0.001319914 0.02642786
## Xhousehold.income[16K-25K]           0.004020286 0.03205329
## Xhousehold.income[25K-35K]          -0.032500816 0.03423930
## Xhousehold.income[35K-50K]          -0.078054449 0.04047685
## Xhousehold.income[50K-75K]          -0.131683368 0.04977313
## Xhousehold.income[5K-12K]           -0.006921848 0.02946763
## Xhousehold.income[75K-100K]         -0.190754582 0.05230867
## Xhigh.educBachelor                  -0.017322063 0.06338448
## Xhigh.educHS Diploma/GED            -0.083186727 0.03777662
## Xhigh.educPost Graduate Degree      -0.091282724 0.06989793
## Xhigh.educSome College              -0.036403367 0.05951998
## Xdemo_race_hispanic1                -0.032461048 0.02435936
```

1.18 Model: CBCL Withdrawn-Depressed ~ Testosterone + Pubertal category

Female participants

```
##
## Family: gaussian
## Link function: identity
##
## Formula:
## cbcl_scr_syn_withdep_r ~ hormone_scr_ert_mean + hormone_sal_end_min_since_midnight +
##     pds_p_ss_category + race.ethnicity.5level + interview_age +
##     bmi + household.income + high.educ + demo_race_hispanic
##
## Parametric coefficients:
##                                      Estimate Std. Error t value Pr(>|t|)
## (Intercept)                         5.779e-01  7.004e-01   0.825 0.409458
## hormone_scr_ert_mean                3.360e-03  2.219e-03   1.515 0.130034
## hormone_sal_end_min_since_midnight -6.178e-05  2.072e-04  -0.298 0.765552
## pds_p_ss_categoryEarly              1.200e-02  9.357e-02   0.128 0.897927
## pds_p_ss_categoryLate               2.747e-01  2.422e-01   1.134 0.256893
## pds_p_ss_categoryMid                1.660e-01  9.186e-02   1.807 0.070923 .
## race.ethnicity.5levelBlack         -4.645e-01  2.392e-01  -1.942 0.052254 .
## race.ethnicity.5levelMixed         -1.629e-02  2.326e-01  -0.070 0.944172
## race.ethnicity.5levelOther         -3.355e-01  2.744e-01  -1.223 0.221597
## race.ethnicity.5levelWhite         -3.734e-02  2.162e-01  -0.173 0.862882
## interview_age                       3.902e-03  4.930e-03   0.791 0.428772
## bmi                                 1.259e-02  9.921e-03   1.269 0.204590
## household.income[>=200K]           -9.187e-01  2.548e-01  -3.605 0.000319 ***
```

```
## household.income[100K-200K]        -6.756e-01  2.364e-01  -2.859 0.004295 **
## household.income[12K-16K]          -4.199e-01  3.047e-01  -1.378 0.168289
## household.income[16K-25K]          -3.334e-01  2.638e-01  -1.264 0.206383
## household.income[25K-35K]          -1.749e-01  2.469e-01  -0.709 0.478708
## household.income[35K-50K]          -6.094e-01  2.402e-01  -2.538 0.011233 *
## household.income[50K-75K]          -5.772e-01  2.358e-01  -2.447 0.014465 *
## household.income[5K-12K]           -1.407e-01  2.721e-01  -0.517 0.605066
## household.income[75K-100K]         -6.070e-01  2.395e-01  -2.535 0.011325 *
## high.educBachelor                   1.326e-01  2.218e-01   0.598 0.550115
## high.educHS Diploma/GED             4.100e-01  2.228e-01   1.841 0.065802 .
## high.educPost Graduate Degree       1.908e-01  2.260e-01   0.844 0.398659
## high.educSome College               2.205e-01  2.071e-01   1.064 0.287263
## demo_race_hispanic1                -1.817e-02  1.058e-01  -0.172 0.863593
## ---
## Signif. codes:  0 '***' 0.001 '**' 0.01 '*' 0.05 '.' 0.1 ' ' 1
##
##
## R-sq.(adj) =  0.0211
## lmer.REML = 8261.8  Scale est. = 2.2679    n = 2194
```

```
##                                          stdcoef      stdse
## X(Intercept)                          0.000000000 0.00000000
## Xhormone_scr_ert_mean                 0.034719992 0.02292443
## Xhormone_sal_end_min_since_midnight -0.006824073 0.02288144
## Xpds_p_ss_categoryEarly               0.003211947 0.02503557
## Xpds_p_ss_categoryLate                0.026025022 0.02294847
## Xpds_p_ss_categoryMid                 0.051383481 0.02843800
## Xrace.ethnicity.5levelBlack          -0.102410574 0.05273183
## Xrace.ethnicity.5levelMixed          -0.003359807 0.04797293
## Xrace.ethnicity.5levelOther          -0.044202540 0.03615326
## Xrace.ethnicity.5levelWhite          -0.011111886 0.06433220
## Xinterview_age                        0.018325480 0.02315453
## Xbmi                                  0.029999534 0.02364098
## Xhousehold.income[>=200K]            -0.181606451 0.05037115
## Xhousehold.income[100K-200K]         -0.198826625 0.06955234
## Xhousehold.income[12K-16K]           -0.039009515 0.02830503
## Xhousehold.income[16K-25K]           -0.041575886 0.03289359
## Xhousehold.income[25K-35K]           -0.026615640 0.03756592
## Xhousehold.income[35K-50K]           -0.109041856 0.04297175
## Xhousehold.income[50K-75K]           -0.123983306 0.05065769
## Xhousehold.income[5K-12K]            -0.015973059 0.03088340
## Xhousehold.income[75K-100K]          -0.137611401 0.05429186
## Xhigh.educBachelor                    0.037033811 0.06196246
## Xhigh.educHS Diploma/GED              0.069480582 0.03774682
## Xhigh.educPost Graduate Degree        0.057942841 0.06863753
## Xhigh.educSome College                0.059595868 0.05598973
## Xdemo_race_hispanic1                 -0.004411155 0.02567280
```

Male participants

```
##
## Family: gaussian
## Link function: identity
```

```
##
## Formula:
## cbcl_scr_syn_withdep_r ~ hormone_scr_ert_mean + hormone_sal_end_min_since_midnight +
##     pds_p_ss_category + race.ethnicity.5level + interview_age +
##     bmi + household.income + high.educ + demo_race_hispanic
##
## Parametric coefficients:
##                                     Estimate Std. Error t value Pr(>|t|)
## (Intercept)                        0.2092646  0.7352380   0.285 0.775959
## hormone_scr_ert_mean               0.0032402  0.0025571   1.267 0.205222
## hormone_sal_end_min_since_midnight  0.0005022  0.0002147   2.339 0.019397 *
## pds_p_ss_categoryEarly             0.0572828  0.0919353   0.623 0.533294
## pds_p_ss_categoryLate             -0.4159963  0.6407029  -0.649 0.516220
## pds_p_ss_categoryMid               0.3362648  0.1751697   1.920 0.055023 .
## race.ethnicity.5levelBlack        -0.1480995  0.2930921  -0.505 0.613395
## race.ethnicity.5levelMixed         0.2942239  0.2864935   1.027 0.304534
## race.ethnicity.5levelOther         0.0574039  0.3262230   0.176 0.860336
## race.ethnicity.5levelWhite         0.1572625  0.2692283   0.584 0.559193
## interview_age                      0.0110921  0.0049327   2.249 0.024626 *
## bmi                               -0.0028786  0.0102788  -0.280 0.779461
## household.income[>=200K]          -1.0891504  0.2782600  -3.914 9.33e-05 ***
## household.income[100K-200K]       -0.9341644  0.2611014  -3.578 0.000354 ***
## household.income[12K-16K]         -0.0087476  0.3379642  -0.026 0.979353
## household.income[16K-25K]          0.0276251  0.2808236   0.098 0.921645
## household.income[25K-35K]         -0.2697189  0.2815953  -0.958 0.338250
## household.income[35K-50K]         -0.5280127  0.2668125  -1.979 0.047936 *
## household.income[50K-75K]         -0.6910410  0.2584062  -2.674 0.007542 **
## household.income[5K-12K]          -0.0814459  0.2898513  -0.281 0.778741
## household.income[75K-100K]        -0.9848292  0.2661698  -3.700 0.000221 ***
## high.educBachelor                 -0.0708398  0.2601759  -0.272 0.785433
## high.educHS Diploma/GED           -0.5822897  0.2585523  -2.252 0.024407 *
## high.educPost Graduate Degree     -0.3409967  0.2617654  -1.303 0.192811
## high.educSome College             -0.1596498  0.2479319  -0.644 0.519686
## demo_race_hispanic1               -0.1572059  0.1123687  -1.399 0.161939
## ---
## Signif. codes:  0 '***' 0.001 '**' 0.01 '*' 0.05 '.' 0.1 ' ' 1
##
##
## R-sq.(adj) =  0.0393
## lmer.REML = 9526.4  Scale est. = 2.0798    n = 2379
```

```
##                                           stdcoef      stdse
## X(Intercept)                         0.0000000000 0.00000000
## Xhormone_scr_ert_mean                0.0266486274 0.02103015
## Xhormone_sal_end_min_since_midnight  0.0496964810 0.02124304
## Xpds_p_ss_categoryEarly              0.0131500003 0.02110492
## Xpds_p_ss_categoryLate              -0.0132860337 0.02046268
## Xpds_p_ss_categoryMid                0.0413912347 0.02156185
## Xrace.ethnicity.5levelBlack         -0.0263696640 0.05218613
## Xrace.ethnicity.5levelMixed          0.0531057061 0.05171043
## Xrace.ethnicity.5levelOther          0.0067076625 0.03811926
## Xrace.ethnicity.5levelWhite          0.0399968783 0.06847335
## Xinterview_age                       0.0464015074 0.02063512
## Xbmi                                -0.0059941241 0.02140347
```

```
## Xhousehold.income[>=200K]          -0.1986101810 0.05074163
## Xhousehold.income[100K-200K]       -0.2398749355 0.06704567
## Xhousehold.income[12K-16K]         -0.0006854266 0.02648145
## Xhousehold.income[16K-25K]          0.0031586052 0.03210885
## Xhousehold.income[25K-35K]         -0.0329479638 0.03439875
## Xhousehold.income[35K-50K]         -0.0802786297 0.04056595
## Xhousehold.income[50K-75K]         -0.1332642944 0.04983253
## Xhousehold.income[5K-12K]          -0.0082926916 0.02951218
## Xhousehold.income[75K-100K]        -0.1938112199 0.05238137
## Xhigh.educBachelor                 -0.0172614478 0.06339677
## Xhigh.educHS Diploma/GED           -0.0851701313 0.03781783
## Xhigh.educPost Graduate Degree     -0.0910716279 0.06991094
## Xhigh.educSome College             -0.0383546327 0.05956374
## Xdemo_race_hispanic1               -0.0341092401 0.02438083
```

1.19 Model: CBCL Depressed DSM-5 ~ Testosterone + PDS

Female participants

```
##
## Family: gaussian
## Link function: identity
##
## Formula:
## cbcl_scr_dsm5_depress_r ~ hormone_scr_ert_mean + hormone_sal_end_min_since_midnight +
##     PDS_score + race.ethnicity.5level + interview_age + bmi +
##     household.income + high.educ + demo_race_hispanic
##
## Parametric coefficients:
##                                     Estimate Std. Error t value Pr(>|t|)
## (Intercept)                        0.7329447  0.7922449   0.925  0.35499
## hormone_scr_ert_mean              -0.0006339  0.0026033  -0.243  0.80764
## hormone_sal_end_min_since_midnight  0.0001436  0.0002405   0.597  0.55059
## PDS_score                          0.1121565  0.0626583   1.790  0.07360 .
## race.ethnicity.5levelBlack        -0.1981417  0.2805824  -0.706  0.48015
## race.ethnicity.5levelMixed         0.2021829  0.2729169   0.741  0.45888
## race.ethnicity.5levelOther        -0.2566456  0.3221469  -0.797  0.42573
## race.ethnicity.5levelWhite         0.2570199  0.2533572   1.014  0.31048
## interview_age                      0.0026545  0.0056264   0.472  0.63713
## bmi                                0.0103957  0.0112319   0.926  0.35478
## household.income[>=200K]         -0.8101511  0.2993534  -2.706  0.00686 **
## household.income[100K-200K]       -0.6652596  0.2777634  -2.395  0.01670 *
## household.income[12K-16K]         -0.0928040  0.3593345  -0.258  0.79623
## household.income[16K-25K]         -0.4328090  0.3094806  -1.399  0.16211
## household.income[25K-35K]         -0.1500203  0.2904260  -0.517  0.60552
## household.income[35K-50K]         -0.4022745  0.2824866  -1.424  0.15458
## household.income[50K-75K]         -0.5263118  0.2774590  -1.897  0.05797 .
## household.income[5K-12K]          -0.0307932  0.3190285  -0.097  0.92312
## household.income[75K-100K]        -0.5404168  0.2816470  -1.919  0.05514 .
## high.educBachelor                 -0.1294081  0.2605665  -0.497  0.61949
## high.educHS Diploma/GED           -0.0097230  0.2615916  -0.037  0.97035
## high.educPost Graduate Degree      0.0640450  0.2654093   0.241  0.80934
## high.educSome College             -0.0166985  0.2433245  -0.069  0.94529
```

```
## demo_race_hispanic1                -0.0419100  0.1240251  -0.338  0.73546
## ---
## Signif. codes:  0 '***' 0.001 '**' 0.01 '*' 0.05 '.' 0.1 ' ' 1
##
##
## R-sq.(adj) =  0.00929
## lmer.REML = 8927.5  Scale est. = 2.5026    n = 2194
```

```
##                                          stdcoef      stdse
## X(Intercept)                         0.000000000 0.00000000
## Xhormone_scr_ert_mean               -0.005646846 0.02319056
## Xhormone_sal_end_min_since_midnight  0.013674021 0.02290549
## XPDS_score                           0.043553078 0.02433174
## Xrace.ethnicity.5levelBlack         -0.037663875 0.05333466
## Xrace.ethnicity.5levelMixed          0.035947294 0.04852352
## Xrace.ethnicity.5levelOther         -0.029154766 0.03659567
## Xrace.ethnicity.5levelWhite          0.065936409 0.06499677
## Xinterview_age                       0.010748532 0.02278259
## Xbmi                                 0.021357943 0.02307579
## Xhousehold.income[>=200K]           -0.138072956 0.05101840
## Xhousehold.income[100K-200K]        -0.168782288 0.07047106
## Xhousehold.income[12K-16K]          -0.007433766 0.02878332
## Xhousehold.income[16K-25K]          -0.046534037 0.03327422
## Xhousehold.income[25K-35K]          -0.019676955 0.03809284
## Xhousehold.income[35K-50K]          -0.062054963 0.04357646
## Xhousehold.income[50K-75K]          -0.097469201 0.05138344
## Xhousehold.income[5K-12K]           -0.003013487 0.03122077
## Xhousehold.income[75K-100K]         -0.105628263 0.05504988
## Xhigh.educBachelor                  -0.031170584 0.06276277
## Xhigh.educHS Diploma/GED            -0.001420491 0.03821733
## Xhigh.educPost Graduate Degree       0.016767089 0.06948461
## Xhigh.educSome College              -0.003891861 0.05671065
## Xdemo_race_hispanic1                -0.008771805 0.02595859
```

Male participants

```
##
## Family: gaussian
## Link function: identity
##
## Formula:
## cbcl_scr_dsm5_depress_r ~ hormone_scr_ert_mean + hormone_sal_end_min_since_midnight +
##     PDS_score + race.ethnicity.5level + interview_age + bmi +
##     household.income + high.educ + demo_race_hispanic
##
## Parametric coefficients:
##                                      Estimate Std. Error t value Pr(>|t|)
## (Intercept)                         1.1512508  0.8548827   1.347 0.178214
## hormone_scr_ert_mean                0.0020613  0.0029940   0.688 0.491220
## hormone_sal_end_min_since_midnight  0.0004118  0.0002576   1.598 0.110066
## PDS_score                           0.1259513  0.0854774   1.474 0.140749
## race.ethnicity.5levelBlack         -0.1607423  0.3422252  -0.470 0.638615
## race.ethnicity.5levelMixed          0.2170055  0.3336456   0.650 0.515493
```

```
## race.ethnicity.5levelOther          0.0416674  0.3792214   0.110 0.912517
## race.ethnicity.5levelWhite          0.1822303  0.3137924   0.581 0.561474
## interview_age                       0.0041179  0.0057451   0.717 0.473590
## bmi                                -0.0063291  0.0119679  -0.529 0.596966
## household.income[>=200K]           -1.1670889  0.3244038  -3.598 0.000328 ***
## household.income[100K-200K]        -1.1453964  0.3037882  -3.770 0.000167 ***
## household.income[12K-16K]           0.0916379  0.3925174   0.233 0.815423
## household.income[16K-25K]          -0.4153483  0.3261648  -1.273 0.202991
## household.income[25K-35K]          -0.6071971  0.3261823  -1.862 0.062794 .
## household.income[35K-50K]          -0.7705732  0.3098218  -2.487 0.012946 *
## household.income[50K-75K]          -0.8482312  0.3005166  -2.823 0.004804 **
## household.income[5K-12K]           -0.2270973  0.3365819  -0.675 0.499922
## household.income[75K-100K]         -0.9936710  0.3096258  -3.209 0.001349 **
## high.educBachelor                   0.2652065  0.3033496   0.874 0.382066
## high.educHS Diploma/GED            -0.3918441  0.3011259  -1.301 0.193296
## high.educPost Graduate Degree      -0.0573529  0.3050946  -0.188 0.850905
## high.educSome College               0.2545760  0.2889903   0.881 0.378453
## demo_race_hispanic1                -0.1283901  0.1348766  -0.952 0.341242
## ---
## Signif. codes:  0 '***' 0.001 '**' 0.01 '*' 0.05 '.' 0.1 ' ' 1
##
##
## R-sq.(adj) =  0.0254
## lmer.REML =  10228  Scale est. = 2.4137    n = 2379
```

```
##                                          stdcoef      stdse
## X(Intercept)                         0.000000000 0.00000000
## Xhormone_scr_ert_mean                0.014668757 0.02130607
## Xhormone_sal_end_min_since_midnight  0.035254939 0.02205504
## XPDS_score                           0.032206845 0.02185732
## Xrace.ethnicity.5levelBlack         -0.024764840 0.05272509
## Xrace.ethnicity.5levelMixed          0.033891314 0.05210784
## Xrace.ethnicity.5levelOther          0.004212899 0.03834221
## Xrace.ethnicity.5levelWhite          0.040102908 0.06905539
## Xinterview_age                       0.014905581 0.02079562
## Xbmi                                -0.011403496 0.02156322
## Xhousehold.income[>=200K]           -0.184150113 0.05118633
## Xhousehold.income[100K-200K]        -0.254490625 0.06749738
## Xhousehold.income[12K-16K]           0.006212987 0.02661242
## Xhousehold.income[16K-25K]          -0.041092069 0.03226878
## Xhousehold.income[25K-35K]          -0.064180229 0.03447720
## Xhousehold.income[35K-50K]          -0.101373360 0.04075885
## Xhousehold.income[50K-75K]          -0.141539820 0.05014560
## Xhousehold.income[5K-12K]           -0.020007486 0.02965318
## Xhousehold.income[75K-100K]         -0.169205708 0.05272415
## Xhigh.educBachelor                   0.055916325 0.06395845
## Xhigh.educHS Diploma/GED            -0.049592493 0.03811104
## Xhigh.educPost Graduate Degree      -0.013253871 0.07050532
## Xhigh.educSome College               0.052920196 0.06007409
## Xdemo_race_hispanic1                -0.024104000 0.02532179
```

1.20 Model: CBCL Depressed DSM-5 ~ Testosterone + Pubertal category

Female participants

```
##
## Family: gaussian
## Link function: identity
##
## Formula:
## cbcl_scr_dsm5_depress_r ~ hormone_scr_ert_mean + hormone_sal_end_min_since_midnight +
##     pds_p_ss_category + race.ethnicity.5level + interview_age +
##     bmi + household.income + high.educ + demo_race_hispanic
##
## Parametric coefficients:
##                                     Estimate Std. Error t value Pr(>|t|)
## (Intercept)                        0.8478270  0.8185592   1.036  0.30043
## hormone_scr_ert_mean              -0.0004206  0.0025923  -0.162  0.87114
## hormone_sal_end_min_since_midnight  0.0001385  0.0002411   0.574  0.56578
## pds_p_ss_categoryEarly             0.0159055  0.1092878   0.146  0.88430
## pds_p_ss_categoryLate              0.1266151  0.2834960   0.447  0.65519
## pds_p_ss_categoryMid               0.1538762  0.1074603   1.432  0.15231
## race.ethnicity.5levelBlack        -0.1879642  0.2804143  -0.670  0.50273
## race.ethnicity.5levelMixed         0.2060143  0.2730257   0.755  0.45059
## race.ethnicity.5levelOther        -0.2489929  0.3222349  -0.773  0.43978
## race.ethnicity.5levelWhite         0.2596160  0.2534493   1.024  0.30579
## interview_age                      0.0028242  0.0057495   0.491  0.62333
## bmi                                0.0091407  0.0116286   0.786  0.43192
## household.income[>=200K]          -0.8059592  0.2997868  -2.688  0.00723 **
## household.income[100K-200K]       -0.6596126  0.2782410  -2.371  0.01784 *
## household.income[12K-16K]         -0.1107718  0.3592581  -0.308  0.75786
## household.income[16K-25K]         -0.4309941  0.3097888  -1.391  0.16429
## household.income[25K-35K]         -0.1582130  0.2906851  -0.544  0.58631
## household.income[35K-50K]        -0.4099075  0.2825349  -1.451  0.14697
## household.income[50K-75K]         -0.5262291  0.2776918  -1.895  0.05822 .
## household.income[5K-12K]          -0.0334329  0.3192385  -0.105  0.91660
## household.income[75K-100K]        -0.5419942  0.2818604  -1.923  0.05462 .
## high.educBachelor                 -0.1385506  0.2610855  -0.531  0.59570
## high.educHS Diploma/GED           -0.0103984  0.2617359  -0.040  0.96831
## high.educPost Graduate Degree      0.0553668  0.2660060   0.208  0.83514
## high.educSome College             -0.0144021  0.2437156  -0.059  0.95288
## demo_race_hispanic1               -0.0491056  0.1240621  -0.396  0.69228
## ---
## Signif. codes:  0 '***' 0.001 '**' 0.01 '*' 0.05 '.' 0.1 ' ' 1
##
##
## R-sq.(adj) =  0.00826
## lmer.REML = 8930.8  Scale est. = 2.5265    n = 2194
```

```
##                                          stdcoef      stdse
## X(Intercept)                         0.000000000 0.00000000
## Xhormone_scr_ert_mean               -0.003746282 0.02309240
## Xhormone_sal_end_min_since_midnight  0.013186416 0.02295798
## Xpds_p_ss_categoryEarly              0.003668969 0.02520976
## Xpds_p_ss_categoryLate               0.010342182 0.02315653
```

```
## Xpds_p_ss_categoryMid                  0.041069921 0.02868141
## Xrace.ethnicity.5levelBlack         -0.035729271 0.05330269
## Xrace.ethnicity.5levelMixed          0.036628516 0.04854287
## Xrace.ethnicity.5levelOther         -0.028285417 0.03660567
## Xrace.ethnicity.5levelWhite          0.066602398 0.06502039
## Xinterview_age                       0.011435636 0.02328088
## Xbmi                                 0.018779432 0.02389084
## Xhousehold.income[>=200K]           -0.137358538 0.05109225
## Xhousehold.income[100K-200K]        -0.167349595 0.07059221
## Xhousehold.income[12K-16K]          -0.008873018 0.02877720
## Xhousehold.income[16K-25K]          -0.046338906 0.03330736
## Xhousehold.income[25K-35K]          -0.020751522 0.03812681
## Xhousehold.income[35K-50K]          -0.063232443 0.04358390
## Xhousehold.income[50K-75K]          -0.097453891 0.05142655
## Xhousehold.income[5K-12K]           -0.003271809 0.03124132
## Xhousehold.income[75K-100K]         -0.105936571 0.05509159
## Xhigh.educBachelor                  -0.033372761 0.06288778
## Xhigh.educHS Diploma/GED            -0.001519158 0.03823841
## Xhigh.educPost Graduate Degree       0.014495119 0.06964082
## Xhigh.educSome College              -0.003356634 0.05680179
## Xdemo_race_hispanic1                -0.010277864 0.02596633
```

Male participants

```
##
## Family: gaussian
## Link function: identity
##
## Formula:
## cbcl_scr_dsm5_depress_r ~ hormone_scr_ert_mean + hormone_sal_end_min_since_midnight +
##     pds_p_ss_category + race.ethnicity.5level + interview_age +
##     bmi + household.income + high.educ + demo_race_hispanic
##
## Parametric coefficients:
##                                      Estimate Std. Error t value Pr(>|t|)
## (Intercept)                         1.2446890  0.8567825   1.453 0.146427
## hormone_scr_ert_mean                0.0020959  0.0029886   0.701 0.483189
## hormone_sal_end_min_since_midnight  0.0004113  0.0002576   1.596 0.110564
## pds_p_ss_categoryEarly              0.1816433  0.1067537   1.702 0.088978 .
## pds_p_ss_categoryLate               0.7119674  0.7446666   0.956 0.339126
## pds_p_ss_categoryMid                0.1698766  0.2030231   0.837 0.402826
## race.ethnicity.5levelBlack         -0.1613089  0.3419476  -0.472 0.637159
## race.ethnicity.5levelMixed          0.2226316  0.3338963   0.667 0.504985
## race.ethnicity.5levelOther          0.0429096  0.3794425   0.113 0.909972
## race.ethnicity.5levelWhite          0.1974357  0.3141618   0.628 0.529769
## interview_age                       0.0040662  0.0057301   0.710 0.478007
## bmi                                -0.0063638  0.0119295  -0.533 0.593772
## household.income[>=200K]           -1.1598034  0.3249337  -3.569 0.000365 ***
## household.income[100K-200K]        -1.1364740  0.3043665  -3.734 0.000193 ***
## household.income[12K-16K]          0.1053358  0.3933603   0.268 0.788889
## household.income[16K-25K]          -0.4188299  0.3267602  -1.282 0.200051
## household.income[25K-35K]          -0.6093998  0.3277276  -1.859 0.063085 .
## household.income[35K-50K]          -0.7657596  0.3105376  -2.466 0.013737 *
```

```
## household.income[50K-75K]          -0.8438526  0.3008998  -2.804 0.005082 **
## household.income[5K-12K]           -0.2196243  0.3371366  -0.651 0.514826
## household.income[75K-100K]         -0.9900089  0.3100697  -3.193 0.001427 **
## high.educBachelor                   0.2766757  0.3034185   0.912 0.361935
## high.educHS Diploma/GED            -0.3779824  0.3014643  -1.254 0.210031
## high.educPost Graduate Degree      -0.0463942  0.3051730  -0.152 0.879180
## high.educSome College               0.2641331  0.2892062   0.913 0.361176
## demo_race_hispanic1                -0.1289859  0.1349956  -0.955 0.339432
## ---
## Signif. codes:  0 '***' 0.001 '**' 0.01 '*' 0.05 '.' 0.1 ' ' 1
##
##
## R-sq.(adj) =  0.0251
## lmer.REML =  10226  Scale est. = 2.41      n = 2379
```

```
##                                          stdcoef      stdse
## X(Intercept)                         0.000000000 0.00000000
## Xhormone_scr_ert_mean                0.014914973 0.02126778
## Xhormone_sal_end_min_since_midnight  0.035211122 0.02205847
## Xpds_p_ss_categoryEarly              0.036080726 0.02120503
## Xpds_p_ss_categoryLate               0.019675253 0.02057890
## Xpds_p_ss_categoryMid                0.018093179 0.02162354
## Xrace.ethnicity.5levelBlack         -0.024852132 0.05268233
## Xrace.ethnicity.5levelMixed          0.034769989 0.05214699
## Xrace.ethnicity.5levelOther          0.004338487 0.03836456
## Xrace.ethnicity.5levelWhite          0.043449116 0.06913669
## Xinterview_age                       0.014718506 0.02074124
## Xbmi                                -0.011466051 0.02149412
## Xhousehold.income[>=200K]           -0.183000566 0.05126994
## Xhousehold.income[100K-200K]        -0.252508191 0.06762585
## Xhousehold.income[12K-16K]           0.007141696 0.02666957
## Xhousehold.income[16K-25K]          -0.041436518 0.03232769
## Xhousehold.income[25K-35K]          -0.064413052 0.03464053
## Xhousehold.income[35K-50K]          -0.100740106 0.04085302
## Xhousehold.income[50K-75K]          -0.140809191 0.05020954
## Xhousehold.income[5K-12K]           -0.019349102 0.02970205
## Xhousehold.income[75K-100K]         -0.168582123 0.05279973
## Xhigh.educBachelor                   0.058334497 0.06397297
## Xhigh.educHS Diploma/GED            -0.047838130 0.03815386
## Xhigh.educPost Graduate Degree      -0.010721387 0.07052344
## Xhigh.educSome College               0.054906873 0.06011897
## Xdemo_race_hispanic1                -0.024215872 0.02534413
```

2—Reward~Puberty—

2.1 Model: BIS-BAS-RR ~ PDS

Female participants

```
##
## Family: gaussian
## Link function: identity
##
## Formula:
## bisbas_ss_basm_rr_z ~ PDS_score + interview_age + bmi
##
## Parametric coefficients:
##                Estimate Std. Error t value Pr(>|t|)
## (Intercept)    0.133146   0.325594   0.409 0.682623
## PDS_score      0.044939   0.029001   1.550 0.121371
## interview_age -0.005092   0.002722  -1.870 0.061540 .
## bmi            0.018625   0.005168   3.604 0.000319 ***
## ---
## Signif. codes:  0 '***' 0.001 '**' 0.01 '*' 0.05 '.' 0.1 ' ' 1
##
##
## R-sq.(adj) =  0.00937
## lmer.REML = 7517.1  Scale est. = 0.7584    n = 2653
```

```
##                   stdcoef      stdse
## X(Intercept)    0.00000000 0.00000000
## XPDS_score      0.03260046 0.02103877
## Xinterview_age -0.03787395 0.02024920
## Xbmi            0.07275213 0.02018564
```

Male participants

```
##
## Family: gaussian
## Link function: identity
##
## Formula:
## bisbas_ss_basm_rr_z ~ PDS_score + interview_age + bmi
##
## Parametric coefficients:
##                 Estimate Std. Error t value Pr(>|t|)
## (Intercept)    0.1610747  0.2935925   0.549   0.5833
## PDS_score      0.0639298  0.0335433   1.906   0.0568 .
## interview_age -0.0015994  0.0024376  -0.656   0.5118
## bmi            0.0002617  0.0048322   0.054   0.9568
## ---
## Signif. codes:  0 '***' 0.001 '**' 0.01 '*' 0.05 '.' 0.1 ' ' 1
##
##
## R-sq.(adj) =  0.000769
## lmer.REML = 7939.1  Scale est. = 0.73357   n = 2867
```

```
##                     stdcoef      stdse
## X(Intercept)    0.00000000 0.00000000
## XPDS_score      0.03672356 0.01926849
## Xinterview_age -0.01248455 0.01902746
## Xbmi            0.00104335 0.01926316
```

2.2 Model : Reaction Time ~ PDS

Female participants

```
##
## Family: gaussian
## Link function: identity
##
## Formula:
## rt_diff_large_neutral_z ~ PDS_score + interview_age + bmi
##
## Parametric coefficients:
##                 Estimate Std. Error t value Pr(>|t|)
## (Intercept)   -0.6226269  0.3292373  -1.891   0.0587 .
## PDS_score      0.0210476  0.0296816   0.709   0.4783
## interview_age  0.0050633  0.0027659   1.831   0.0673 .
## bmi            0.0009066  0.0052428   0.173   0.8627
## ---
## Signif. codes:  0 '***' 0.001 '**' 0.01 '*' 0.05 '.' 0.1 ' ' 1
##
##
## R-sq.(adj) =  0.000899
## lmer.REML = 5989.8  Scale est. = 0.75476   n = 2217
```

```
##                     stdcoef      stdse
## X(Intercept)   0.000000000 0.00000000
## XPDS_score     0.016206938 0.02285525
## Xinterview_age 0.040472732 0.02210845
## Xbmi           0.003813751 0.02205340
```

```
##
## Family: gaussian
## Link function: identity
##
## Formula:
## rt_diff_large_small_z ~ PDS_score + interview_age + bmi
##
## Parametric coefficients:
##                Estimate Std. Error t value Pr(>|t|)
## (Intercept)   -0.569055   0.342373  -1.662   0.0966 .
## PDS_score     -0.001886   0.030923  -0.061   0.9514
## interview_age  0.004065   0.002877   1.413   0.1578
## bmi            0.005261   0.005438   0.967   0.3335
## ---
## Signif. codes:  0 '***' 0.001 '**' 0.01 '*' 0.05 '.' 0.1 ' ' 1
##
##
```

```
## R-sq.(adj) =  0.000148
## lmer.REML = 6142.5  Scale est. = 0.81938   n = 2217
```

```
##                     stdcoef      stdse
## X(Intercept)    0.000000000 0.00000000
## XPDS_score     -0.001403263 0.02300601
## Xinterview_age  0.031394536 0.02222092
## Xbmi            0.021381007 0.02210234
```

Male participants

```
##
## Family: gaussian
## Link function: identity
##
## Formula:
## rt_diff_large_neutral_z ~ PDS_score + interview_age + bmi
##
## Parametric coefficients:
##                Estimate Std. Error t value Pr(>|t|)
## (Intercept)   -0.634098   0.311502  -2.036   0.0419 *
## PDS_score     -0.054779   0.036735  -1.491   0.1361
## interview_age  0.005409   0.002576   2.100   0.0358 *
## bmi            0.002275   0.005262   0.432   0.6655
## ---
## Signif. codes:  0 '***' 0.001 '**' 0.01 '*' 0.05 '.' 0.1 ' ' 1
##
##
## R-sq.(adj) =  0.00135
## lmer.REML = 6133.6  Scale est. = 0.84844   n = 2284
```

```
##                     stdcoef      stdse
## X(Intercept)    0.000000000 0.00000000
## XPDS_score     -0.032175118 0.02157703
## Xinterview_age  0.044555302 0.02121420
## Xbmi            0.009273588 0.02144716
```

```
##
## Family: gaussian
## Link function: identity
##
## Formula:
## rt_diff_large_small_z ~ PDS_score + interview_age + bmi
##
## Parametric coefficients:
##                 Estimate Std. Error t value Pr(>|t|)
## (Intercept)    0.0139388  0.3073470   0.045    0.964
## PDS_score     -0.0289614  0.0362562  -0.799    0.424
## interview_age -0.0005069  0.0025405  -0.200    0.842
## bmi            0.0037755  0.0051909   0.727    0.467
##
##
## R-sq.(adj) =  -0.000858
## lmer.REML = 6078.1  Scale est. = 0.82862   n = 2284
```

```
##                   stdcoef      stdse
## X(Intercept)    0.000000000 0.00000000
## XPDS_score     -0.017238738 0.02158082
## Xinterview_age -0.004231179 0.02120524
## Xbmi            0.015592939 0.02143895
```

2.3 Model: Caudate Anticipation ~ PDS

Female participants

```
##
## Family: gaussian
## Link function: identity
##
## Formula:
## caudate_rvsn_ant_z ~ PDS_score + interview_age + bmi
##
## Parametric coefficients:
##                Estimate Std. Error t value Pr(>|t|)
## (Intercept)   -0.315320   0.325732  -0.968    0.333
## PDS_score     -0.015660   0.029322  -0.534    0.593
## interview_age  0.003899   0.002741   1.423    0.155
## bmi           -0.007257   0.005322  -1.364    0.173
##
##
## R-sq.(adj) =  0.000795
## lmer.REML = 5366.4  Scale est. = 0.73411   n = 2051
```

```
##                  stdcoef      stdse
## X(Intercept)    0.00000000 0.00000000
## XPDS_score     -0.01276227 0.02389702
## Xinterview_age  0.03283735 0.02308261
## Xbmi           -0.03139029 0.02302119
```

Male participants

```
##
## Family: gaussian
## Link function: identity
##
## Formula:
## caudate_rvsn_ant_z ~ PDS_score + interview_age + bmi
##
## Parametric coefficients:
##                Estimate Std. Error t value Pr(>|t|)
## (Intercept)   -0.340494   0.330330  -1.031   0.3028
## PDS_score      0.039484   0.039871   0.990   0.3221
## interview_age  0.004061   0.002723   1.491   0.1360
## bmi           -0.010783   0.005629  -1.916   0.0556 .
## ---
## Signif. codes:  0 '***' 0.001 '**' 0.01 '*' 0.05 '.' 0.1 ' ' 1
##
```

```
##
## R-sq.(adj) =  0.00121
## lmer.REML = 5432.7  Scale est. = 0.83878   n = 2027
```

```
##                    stdcoef      stdse
## X(Intercept)    0.00000000 0.00000000
## XPDS_score      0.02261445 0.02283559
## Xinterview_age  0.03366237 0.02257299
## Xbmi           -0.04360563 0.02276427
```

2.4 Model B: Putamen Anticipation ~ PDS

Female participants

```
##
## Family: gaussian
## Link function: identity
##
## Formula:
## putamen_rvsn_ant_z ~ PDS_score + interview_age + bmi
##
## Parametric coefficients:
##                 Estimate Std. Error t value Pr(>|t|)
## (Intercept)   -0.423647   0.322762  -1.313   0.1895
## PDS_score      0.005043   0.029045   0.174   0.8622
## interview_age  0.004721   0.002715   1.739   0.0822 .
## bmi           -0.008698   0.005310  -1.638   0.1016
## ---
## Signif. codes:  0 '***' 0.001 '**' 0.01 '*' 0.05 '.' 0.1 ' ' 1
##
##
## R-sq.(adj) =  0.00141
## lmer.REML = 5332.1  Scale est. = 0.69631   n = 2051
```

```
##                     stdcoef      stdse
## X(Intercept)    0.000000000 0.00000000
## XPDS_score      0.004151439 0.02390785
## Xinterview_age  0.040093886 0.02305580
## Xbmi           -0.037760930 0.02305285
```

Male participants

```
##
## Family: gaussian
## Link function: identity
##
## Formula:
## putamen_rvsn_ant_z ~ PDS_score + interview_age + bmi
##
## Parametric coefficients:
##                  Estimate Std. Error t value Pr(>|t|)
## (Intercept)   -0.2098831  0.3226638  -0.650   0.5155
```

```
## PDS_score      0.0009488  0.0389081   0.024   0.9805
## interview_age  0.0032872  0.0026568   1.237   0.2161
## bmi           -0.0090817  0.0054930  -1.653   0.0984 .
## ---
## Signif. codes:  0 '***' 0.001 '**' 0.01 '*' 0.05 '.' 0.1 ' ' 1
##
##
## R-sq.(adj) =  0.000503
## lmer.REML = 5344.5  Scale est. = 0.66471   n = 2031
```

```
##                      stdcoef      stdse
## X(Intercept)    0.0000000000 0.00000000
## XPDS_score      0.0005572441 0.02285242
## Xinterview_age  0.0278660679 0.02252240
## Xbmi           -0.0376742740 0.02278716
```

2.5 Model: Accumbens Anticipation ~ PDS

Female participants

```
##
## Family: gaussian
## Link function: identity
##
## Formula:
## accumbens_rvsn_ant_z ~ PDS_score + interview_age + bmi
##
## Parametric coefficients:
##                 Estimate Std. Error t value Pr(>|t|)
## (Intercept)    0.2093731  0.2557352   0.819    0.413
## PDS_score     -0.0195109  0.0229520  -0.850    0.395
## interview_age -0.0005292  0.0021487  -0.246    0.805
## bmi           -0.0057282  0.0042144  -1.359    0.174
##
##
## R-sq.(adj) =  0.000377
## lmer.REML = 4379.1  Scale est. = 0.47334   n = 2046
```

```
##                     stdcoef      stdse
## X(Intercept)    0.000000000 0.00000000
## XPDS_score     -0.020210714 0.02377521
## Xinterview_age -0.005660828 0.02298374
## Xbmi           -0.031273018 0.02300827
```

Male participants

```
##
## Family: gaussian
## Link function: identity
##
## Formula:
## accumbens_rvsn_ant_z ~ PDS_score + interview_age + bmi
```

```
##
## Parametric coefficients:
##                Estimate Std. Error t value Pr(>|t|)
## (Intercept)    0.194704   0.259591   0.750    0.453
## PDS_score      0.019946   0.031125   0.641    0.522
## interview_age -0.001337   0.002139  -0.625    0.532
## bmi           -0.003175   0.004420  -0.718    0.473
##
##
## R-sq.(adj) =  -0.000882
## lmer.REML = 4464.8  Scale est. = 0.44239   n = 2028
```

```
##                    stdcoef      stdse
## X(Intercept)    0.00000000 0.00000000
## XPDS_score      0.01467156 0.02289481
## Xinterview_age -0.01409280 0.02253948
## Xbmi           -0.01639603 0.02282584
```

2.6 Model: Caudate Feedback ~ PDS

Female participants

```
##
## Family: gaussian
## Link function: identity
##
## Formula:
## caudate_posvsneg_feedback_z ~ PDS_score + interview_age + bmi
##
## Parametric coefficients:
##                 Estimate Std. Error t value Pr(>|t|)
## (Intercept)   -4.561e-02  3.198e-01  -0.143    0.887
## PDS_score      3.641e-02  2.879e-02   1.264    0.206
## interview_age -2.853e-04  2.689e-03  -0.106    0.916
## bmi            5.477e-05  5.231e-03   0.010    0.992
##
##
## R-sq.(adj) =  -0.000764
## lmer.REML = 5269.5  Scale est. = 0.63123   n = 2048
```

```
##                      stdcoef      stdse
## X(Intercept)    0.0000000000 0.00000000
## XPDS_score      0.0303435214 0.02399714
## Xinterview_age -0.0024540261 0.02313015
## Xbmi            0.0002419529 0.02311001
```

Male participants

```
##
## Family: gaussian
## Link function: identity
##
```

```
## Formula:
## caudate_posvsneg_feedback_z ~ PDS_score + interview_age + bmi
##
## Parametric coefficients:
##                 Estimate Std. Error t value Pr(>|t|)
## (Intercept)   -4.379e-02  3.258e-01  -0.134    0.893
## PDS_score     -2.264e-03  3.913e-02  -0.058    0.954
## interview_age  2.117e-06  2.682e-03   0.001    0.999
## bmi            3.064e-03  5.523e-03   0.555    0.579
##
##
## R-sq.(adj) =  -0.00135
## lmer.REML = 5399.9  Scale est. = 0.82349   n = 2032
```

```
##                     stdcoef      stdse
## X(Intercept)    0.000000e+00 0.00000000
## XPDS_score     -1.319118e-03 0.02280107
## Xinterview_age  1.775226e-05 0.02248674
## Xbmi            1.258849e-02 0.02269318
```

2.7 Model: Putamen Feedback ~ PDS

Female participants

```
##
## Family: gaussian
## Link function: identity
##
## Formula:
## putamen_posvsneg_feedback_z ~ PDS_score + interview_age + bmi
##
## Parametric coefficients:
##                 Estimate Std. Error t value Pr(>|t|)
## (Intercept)    0.0988455  0.3149600   0.314    0.754
## PDS_score      0.0336585  0.0283956   1.185    0.236
## interview_age -0.0013557  0.0026478  -0.512    0.609
## bmi           -0.0001433  0.0051534  -0.028    0.978
##
##
## R-sq.(adj) =  -0.00083
## lmer.REML = 5203.2  Scale est. = 0.70883   n = 2048
```

```
##                     stdcoef      stdse
## X(Intercept)    0.0000000000 0.00000000
## XPDS_score      0.0284227111 0.02397843
## Xinterview_age -0.0118501992 0.02314501
## Xbmi           -0.0006406659 0.02304141
```

Male participants

```
##
## Family: gaussian
```

```
## Link function: identity
##
## Formula:
## putamen_posvsneg_feedback_z ~ PDS_score + interview_age + bmi
##
## Parametric coefficients:
##                Estimate Std. Error t value Pr(>|t|)
## (Intercept)    0.214649   0.323425   0.664    0.507
## PDS_score      0.024747   0.038699   0.639    0.523
## interview_age -0.002826   0.002663  -1.061    0.289
## bmi            0.006628   0.005479   1.210    0.227
##
##
## R-sq.(adj) =  -0.000494
## lmer.REML = 5363.4  Scale est. = 0.79657   n = 2037

##                    stdcoef      stdse
## X(Intercept)    0.00000000 0.00000000
## XPDS_score      0.01457976 0.02280021
## Xinterview_age -0.02392731 0.02254492
## Xbmi            0.02751314 0.02274540
```

2.8 Model: Accumbens Feedback ~ PDS

Female participants

```
##
## Family: gaussian
## Link function: identity
##
## Formula:
## accumbens_posvsneg_feedback_z ~ PDS_score + interview_age + bmi
##
## Parametric coefficients:
##                 Estimate Std. Error t value Pr(>|t|)
## (Intercept)   -0.3660146  0.2503784  -1.462    0.144
## PDS_score      0.0134280  0.0225898   0.594    0.552
## interview_age  0.0025873  0.0021072   1.228    0.220
## bmi            0.0008257  0.0040922   0.202    0.840
##
##
## R-sq.(adj) =  6.88e-05
## lmer.REML = 4268.4  Scale est. = 0.43456   n = 2047

##                    stdcoef      stdse
## X(Intercept)   0.000000000 0.00000000
## XPDS_score     0.014258065 0.02398625
## Xinterview_age 0.028418953 0.02314593
## Xbmi           0.004652283 0.02305739
```

Male participants

```
##
```

```
## Family: gaussian
## Link function: identity
##
## Formula:
## accumbens_posvsneg_feedback_z ~ PDS_score + interview_age + bmi
##
## Parametric coefficients:
##                 Estimate Std. Error t value Pr(>|t|)
## (Intercept)   -0.0873822  0.2649875  -0.330    0.742
## PDS_score     -0.0412568  0.0317171  -1.301    0.193
## interview_age  0.0003722  0.0021805   0.171    0.864
## bmi            0.0060185  0.0045187   1.332    0.183
##
##
## R-sq.(adj) =  -0.000298
## lmer.REML =   4527  Scale est. = 0.44187   n = 2030
```

```
##                    stdcoef      stdse
## X(Intercept)    0.000000000 0.00000000
## XPDS_score     -0.029745998 0.02286789
## Xinterview_age  0.003848071 0.02254625
## Xbmi            0.030395062 0.02282045
```

2.9 Model: OFC Anticipation ~ PDS

Female participants

```
##
## Family: gaussian
## Link function: identity
##
## Formula:
## lOFC_rvsn_ant_z ~ PDS_score + interview_age + bmi
##
## Parametric coefficients:
##                 Estimate Std. Error t value Pr(>|t|)
## (Intercept)    2.997e-02  2.044e-01   0.147    0.883
## PDS_score     -1.225e-02  1.851e-02  -0.662    0.508
## interview_age -1.434e-05  1.719e-03  -0.008    0.993
## bmi           -2.967e-04  3.336e-03  -0.089    0.929
##
##
## R-sq.(adj) =  -0.00118
## lmer.REML = 3424.1  Scale est. = 0.30859   n = 2038
```

```
##                     stdcoef      stdse
## X(Intercept)    0.0000000000 0.00000000
## XPDS_score     -0.0158778818 0.02399684
## Xinterview_age -0.0001933972 0.02318168
## Xbmi           -0.0020531943 0.02308835
```

```
##
```

```
## Family: gaussian
## Link function: identity
##
## Formula:
## mOFC_rvsn_ant_z ~ PDS_score + interview_age + bmi
##
## Parametric coefficients:
##                 Estimate Std. Error t value Pr(>|t|)
## (Intercept)    0.1633755  0.2378443   0.687   0.4922
## PDS_score     -0.0505856  0.0214306  -2.360   0.0183 *
## interview_age -0.0006933  0.0020002  -0.347   0.7289
## bmi           -0.0003504  0.0038820  -0.090   0.9281
## ---
## Signif. codes:  0 '***' 0.001 '**' 0.01 '*' 0.05 '.' 0.1 ' ' 1
##
##
## R-sq.(adj) =  0.00184
## lmer.REML = 4040.7  Scale est. = 0.41739   n = 2039
```

```
##                    stdcoef      stdse
## X(Intercept)    0.000000000 0.00000000
## XPDS_score     -0.056533996 0.02395062
## Xinterview_age -0.008015332 0.02312355
## Xbmi           -0.002080659 0.02305251
```

Male participants

```
##
## Family: gaussian
## Link function: identity
##
## Formula:
## lOFC_rvsn_ant_z ~ PDS_score + interview_age + bmi
##
## Parametric coefficients:
##                Estimate Std. Error t value Pr(>|t|)
## (Intercept)   -0.364481   0.223289  -1.632   0.1028
## PDS_score      0.028848   0.027119   1.064   0.2876
## interview_age  0.003064   0.001839   1.666   0.0959 .
## bmi           -0.004122   0.003792  -1.087   0.2772
## ---
## Signif. codes:  0 '***' 0.001 '**' 0.01 '*' 0.05 '.' 0.1 ' ' 1
##
##
## R-sq.(adj) =  0.000869
## lmer.REML = 3815.7  Scale est. = 0.38049   n = 2018
```

```
##                   stdcoef      stdse
## X(Intercept)    0.00000000 0.00000000
## XPDS_score      0.02436537 0.02290482
## Xinterview_age  0.03767736 0.02262065
## Xbmi           -0.02482405 0.02283966
```

```
##
## Family: gaussian
## Link function: identity
##
## Formula:
## mOFC_rvsn_ant_z ~ PDS_score + interview_age + bmi
##
## Parametric coefficients:
##                 Estimate Std. Error t value Pr(>|t|)
## (Intercept)   -0.1620470  0.2550107  -0.635   0.5252
## PDS_score      0.0556011  0.0308747   1.801   0.0719 .
## interview_age  0.0008762  0.0020992   0.417   0.6764
## bmi           -0.0023951  0.0043097  -0.556   0.5784
## ---
## Signif. codes:  0 '***' 0.001 '**' 0.01 '*' 0.05 '.' 0.1 ' ' 1
##
##
## R-sq.(adj) =  0.000338
## lmer.REML = 4381.8  Scale est. = 0.5034    n = 2024
```

```
##                     stdcoef      stdse
## X(Intercept)    0.000000000 0.00000000
## XPDS_score      0.041098168 0.02282133
## Xinterview_age  0.009380096 0.02247257
## Xbmi           -0.012611190 0.02269188
```

2.10 Model: OFC Feedback ~ PDS

Female participants

```
##
## Family: gaussian
## Link function: identity
##
## Formula:
## lOFC_posvsneg_feedback_z ~ PDS_score + interview_age + bmi
##
## Parametric coefficients:
##                 Estimate Std. Error t value Pr(>|t|)
## (Intercept)    5.939e-02  1.854e-01   0.320    0.749
## PDS_score      4.868e-03  1.674e-02   0.291    0.771
## interview_age -8.429e-04  1.561e-03  -0.540    0.589
## bmi            7.308e-05  3.024e-03   0.024    0.981
##
##
## R-sq.(adj) =  -0.00137
## lmer.REML = 3045.9  Scale est. = 0.2541    n = 2048
```

```
##                     stdcoef      stdse
## X(Intercept)    0.000000000 0.00000000
## XPDS_score      0.006971117 0.02397655
## Xinterview_age -0.012506084 0.02316288
## Xbmi            0.000556958 0.02304377
```

```
##
## Family: gaussian
## Link function: identity
##
## Formula:
## mOFC_posvsneg_feedback_z ~ PDS_score + interview_age + bmi
##
## Parametric coefficients:
##                Estimate Std. Error t value Pr(>|t|)
## (Intercept)    0.0262328  0.2188123   0.120    0.905
## PDS_score      0.0025914  0.0197740   0.131    0.896
## interview_age -0.0001621  0.0018415  -0.088    0.930
## bmi           -0.0020736  0.0035868  -0.578    0.563
##
##
## R-sq.(adj) =  -0.00129
## lmer.REML = 3754.4  Scale est. = 0.33097   n = 2051
```

```
##                     stdcoef      stdse
## X(Intercept)    0.000000000 0.00000000
## XPDS_score      0.003121964 0.02382272
## Xinterview_age -0.002026504 0.02302188
## Xbmi           -0.013310786 0.02302420
```

Male participants

```
##
## Family: gaussian
## Link function: identity
##
## Formula:
## lOFC_posvsneg_feedback_z ~ PDS_score + interview_age + bmi
##
## Parametric coefficients:
##                Estimate Std. Error t value Pr(>|t|)
## (Intercept)   -0.157779   0.196239  -0.804    0.421
## PDS_score     -0.020718   0.023717  -0.874    0.382
## interview_age  0.001151   0.001614   0.713    0.476
## bmi            0.002926   0.003342   0.876    0.381
##
##
## R-sq.(adj) =  -0.000642
## lmer.REML = 3299.1  Scale est. = 0.24695   n = 2016
```

```
##                    stdcoef      stdse
## X(Intercept)    0.00000000 0.00000000
## XPDS_score     -0.02004021 0.02294089
## Xinterview_age  0.01609524 0.02255933
## Xbmi            0.01999848 0.02284172
```

```
##
## Family: gaussian
## Link function: identity
```

```
##
## Formula:
## mOFC_posvsneg_feedback_z ~ PDS_score + interview_age + bmi
##
## Parametric coefficients:
##                 Estimate Std. Error t value Pr(>|t|)
## (Intercept)   -0.0698922  0.2328640  -0.300    0.764
## PDS_score     -0.0252354  0.0282122  -0.894    0.371
## interview_age  0.0007394  0.0019161   0.386    0.700
## bmi            0.0023849  0.0039657   0.601    0.548
##
##
## R-sq.(adj) =  -0.000915
## lmer.REML = 4014.8  Scale est. = 0.36025   n = 2025
```

```
##                     stdcoef      stdse
## X(Intercept)    0.000000000 0.00000000
## XPDS_score     -0.020467817 0.02288231
## Xinterview_age  0.008694975 0.02253299
## Xbmi            0.013711233 0.02279956
```

2.11 Model: Caudate Anticipation ~ Testosterone

Female participants

```
## Warning: Some predictor variables are on very different scales: consider
## rescaling
```

```
##
## Family: gaussian
## Link function: identity
##
## Formula:
## caudate_rvsn_ant_z ~ hormone_scr_ert_mean + hormone_sal_end_min_since_midnight +
##     interview_age + MRI_minus_hormone_date_time + bmi
##
## Parametric coefficients:
##                                      Estimate Std. Error t value Pr(>|t|)
## (Intercept)                        -2.508e-01  3.565e-01  -0.704    0.482
## hormone_scr_ert_mean               -1.281e-03  1.292e-03  -0.992    0.321
## hormone_sal_end_min_since_midnight -1.352e-04  1.239e-04  -1.091    0.275
## interview_age                       4.229e-03  2.828e-03   1.495    0.135
## MRI_minus_hormone_date_time         1.061e-06  2.635e-06   0.403    0.687
## bmi                                -5.840e-03  5.562e-03  -1.050    0.294
##
##
## R-sq.(adj) =  0.000508
## lmer.REML = 4903.7  Scale est. = 0.74968   n = 1859
```

```
##                                         stdcoef      stdse
## X(Intercept)                        0.000000000 0.00000000
## Xhormone_scr_ert_mean              -0.024011831 0.02421399
```

```
## Xhormone_sal_end_min_since_midnight -0.026693155 0.02446015
## Xinterview_age                       0.035730321 0.02389359
## XMRI_minus_hormone_date_time         0.009612325 0.02386402
## Xbmi                                -0.025000223 0.02380805
```

Male participants

```
## Warning: Some predictor variables are on very different scales: consider
## rescaling
```

```
##
## Family: gaussian
## Link function: identity
##
## Formula:
## caudate_rvsn_ant_z ~ hormone_scr_ert_mean + hormone_sal_end_min_since_midnight +
##     interview_age + MRI_minus_hormone_date_time + bmi
##
## Parametric coefficients:
##                                      Estimate Std. Error t value Pr(>|t|)
## (Intercept)                        -1.771e-01  3.551e-01  -0.499   0.6182
## hormone_scr_ert_mean               -2.009e-04  1.495e-03  -0.134   0.8931
## hormone_sal_end_min_since_midnight  1.413e-04  1.258e-04   1.123   0.2615
## interview_age                       2.394e-03  2.803e-03   0.854   0.3931
## MRI_minus_hormone_date_time         4.211e-07  2.890e-06   0.146   0.8842
## bmi                                -1.128e-02  5.722e-03  -1.971   0.0489 *
## ---
## Signif. codes:  0 '***' 0.001 '**' 0.01 '*' 0.05 '.' 0.1 ' ' 1
##
##
## R-sq.(adj) =  0.00076
## lmer.REML = 5003.1  Scale est. = 0.82633   n = 1861
```

```
##                                          stdcoef      stdse
## X(Intercept)                         0.000000000 0.00000000
## Xhormone_scr_ert_mean               -0.003222334 0.02397052
## Xhormone_sal_end_min_since_midnight  0.027777643 0.02472956
## Xinterview_age                       0.020144337 0.02358050
## XMRI_minus_hormone_date_time         0.003514959 0.02412369
## Xbmi                                -0.046611973 0.02365409
```

2.12 Model B: Putamen Anticipation ~ Testosterone

Female participants

```
## Warning: Some predictor variables are on very different scales: consider
## rescaling
```

```
##
## Family: gaussian
## Link function: identity
##
```

```
## Formula:
## putamen_rvsn_ant_z ~ hormone_scr_ert_mean + hormone_sal_end_min_since_midnight +
##     interview_age + MRI_minus_hormone_date_time + bmi
##
## Parametric coefficients:
##                                      Estimate Std. Error t value Pr(>|t|)
## (Intercept)                        -2.657e-01  3.506e-01  -0.758    0.449
## hormone_scr_ert_mean                1.005e-04  1.273e-03   0.079    0.937
## hormone_sal_end_min_since_midnight -1.937e-04  1.215e-04  -1.593    0.111
## interview_age                       4.141e-03  2.781e-03   1.489    0.137
## MRI_minus_hormone_date_time         1.282e-06  2.591e-06   0.495    0.621
## bmi                                -5.593e-03  5.504e-03  -1.016    0.310
##
##
## R-sq.(adj) =  0.000705
## lmer.REML = 4843.8  Scale est. = 0.64702   n = 1859
```

```
##                                          stdcoef      stdse
## X(Intercept)                         0.000000000 0.00000000
## Xhormone_scr_ert_mean                0.001912854 0.02422436
## Xhormone_sal_end_min_since_midnight -0.038932176 0.02443491
## Xinterview_age                       0.035546887 0.02387640
## XMRI_minus_hormone_date_time         0.011807851 0.02387406
## Xbmi                                -0.024240233 0.02385382
```

Male participants

```
## Warning: Some predictor variables are on very different scales: consider
## rescaling
```

```
##
## Family: gaussian
## Link function: identity
##
## Formula:
## putamen_rvsn_ant_z ~ hormone_scr_ert_mean + hormone_sal_end_min_since_midnight +
##     interview_age + MRI_minus_hormone_date_time + bmi
##
## Parametric coefficients:
##                                      Estimate Std. Error t value Pr(>|t|)
## (Intercept)                        -2.499e-01  3.510e-01  -0.712    0.477
## hormone_scr_ert_mean               -1.325e-03  1.479e-03  -0.896    0.370
## hormone_sal_end_min_since_midnight  1.920e-04  1.242e-04   1.546    0.122
## interview_age                       2.994e-03  2.775e-03   1.079    0.281
## MRI_minus_hormone_date_time         7.879e-07  2.866e-06   0.275    0.783
## bmi                                -1.073e-02  5.679e-03  -1.890    0.059 .
## ---
## Signif. codes:  0 '***' 0.001 '**' 0.01 '*' 0.05 '.' 0.1 ' ' 1
##
##
## R-sq.(adj) =  0.00224
## lmer.REML = 4964.9  Scale est. = 0.62557   n = 1861
```

```
##                                         stdcoef      stdse
## X(Intercept)                         0.000000000 0.00000000
## Xhormone_scr_ert_mean               -0.021466666 0.02395732
## Xhormone_sal_end_min_since_midnight  0.037998249 0.02457886
## Xinterview_age                       0.025375331 0.02352142
## XMRI_minus_hormone_date_time         0.006616824 0.02406615
## Xbmi                                -0.044735929 0.02367364
```

2.13 Model: Accumbens Anticipation ~ Testosterone

Female participants

```
## Warning: Some predictor variables are on very different scales: consider
## rescaling
```

```
##
## Family: gaussian
## Link function: identity
##
## Formula:
## accumbens_rvsn_ant_z ~ hormone_scr_ert_mean + hormone_sal_end_min_since_midnight +
##     interview_age + MRI_minus_hormone_date_time + bmi
##
## Parametric coefficients:
##                                      Estimate Std. Error t value Pr(>|t|)
## (Intercept)                         2.685e-01  2.796e-01   0.960   0.3369
## hormone_scr_ert_mean                1.317e-05  1.011e-03   0.013   0.9896
## hormone_sal_end_min_since_midnight -1.086e-04  9.423e-05  -1.152   0.2493
## interview_age                      -1.979e-04  2.214e-03  -0.089   0.9288
## MRI_minus_hormone_date_time        -2.295e-07  2.051e-06  -0.112   0.9109
## bmi                                -8.257e-03  4.398e-03  -1.878   0.0606 .
## ---
## Signif. codes:  0 '***' 0.001 '**' 0.01 '*' 0.05 '.' 0.1 ' ' 1
##
##
## R-sq.(adj) =  0.000136
## lmer.REML = 3998.4  Scale est. = 0.41352   n = 1853
```

```
##                                         stdcoef      stdse
## X(Intercept)                         0.0000000000 0.00000000
## Xhormone_scr_ert_mean                0.0003141855 0.02411402
## Xhormone_sal_end_min_since_midnight -0.0273686975 0.02374762
## Xinterview_age                      -0.0021284752 0.02381991
## XMRI_minus_hormone_date_time        -0.0026513982 0.02369330
## Xbmi                                -0.0446764288 0.02379433
```

Male participants

```
## Warning: Some predictor variables are on very different scales: consider
## rescaling
```

```
##
```

```
## Family: gaussian
## Link function: identity
##
## Formula:
## accumbens_rvsn_ant_z ~ hormone_scr_ert_mean + hormone_sal_end_min_since_midnight +
##     interview_age + MRI_minus_hormone_date_time + bmi
##
## Parametric coefficients:
##                                      Estimate Std. Error t value Pr(>|t|)
## (Intercept)                         2.093e-01  2.810e-01   0.745    0.457
## hormone_scr_ert_mean                9.080e-05  1.187e-03   0.076    0.939
## hormone_sal_end_min_since_midnight  7.517e-05  9.893e-05   0.760    0.447
## interview_age                      -1.597e-03  2.219e-03  -0.720    0.472
## MRI_minus_hormone_date_time        -3.321e-06  2.337e-06  -1.421    0.156
## bmi                                -3.338e-03  4.527e-03  -0.737    0.461
##
##
## R-sq.(adj) =  -0.000664
## lmer.REML =   4126  Scale est. = 0.44854   n = 1856
```

```
##                                          stdcoef      stdse
## X(Intercept)                         0.000000000 0.00000000
## Xhormone_scr_ert_mean                0.001837754 0.02402567
## Xhormone_sal_end_min_since_midnight  0.018667848 0.02456777
## Xinterview_age                      -0.016983118 0.02359567
## XMRI_minus_hormone_date_time        -0.034276041 0.02412604
## Xbmi                                -0.017497800 0.02373459
```

2.14 Model: Caudate Feedback ~ Testosterone

Female participants

```
## Warning: Some predictor variables are on very different scales: consider
## rescaling
```

```
##
## Family: gaussian
## Link function: identity
##
## Formula:
## caudate_posvsneg_feedback_z ~ hormone_scr_ert_mean + hormone_sal_end_min_since_midnight +
##     interview_age + MRI_minus_hormone_date_time + bmi
##
## Parametric coefficients:
##                                      Estimate Std. Error t value Pr(>|t|)
## (Intercept)                         6.533e-02  3.458e-01   0.189   0.8502
## hormone_scr_ert_mean               -3.724e-06  1.251e-03  -0.003   0.9976
## hormone_sal_end_min_since_midnight -2.922e-04  1.211e-04  -2.413   0.0159 *
## interview_age                       8.981e-04  2.742e-03   0.328   0.7433
## MRI_minus_hormone_date_time        -5.379e-07  2.551e-06  -0.211   0.8330
## bmi                                 2.408e-03  5.390e-03   0.447   0.6551
## ---
## Signif. codes:  0 '***' 0.001 '**' 0.01 '*' 0.05 '.' 0.1 ' ' 1
```

```
##
##
## R-sq.(adj) =  0.00178
## lmer.REML =   4764  Scale est. = 0.65794   n = 1854
```

```
##                                           stdcoef      stdse
## X(Intercept)                         0.000000e+00 0.00000000
## Xhormone_scr_ert_mean               -7.226421e-05 0.02428754
## Xhormone_sal_end_min_since_midnight -5.972708e-02 0.02475515
## Xinterview_age                       7.838678e-03 0.02393447
## XMRI_minus_hormone_date_time        -5.053207e-03 0.02396571
## Xbmi                                 1.065694e-02 0.02385575
```

Male participants

```
## Warning: Some predictor variables are on very different scales: consider
## rescaling
```

```
##
## Family: gaussian
## Link function: identity
##
## Formula:
## caudate_posvsneg_feedback_z ~ hormone_scr_ert_mean + hormone_sal_end_min_since_midnight +
##     interview_age + MRI_minus_hormone_date_time + bmi
##
## Parametric coefficients:
##                                      Estimate Std. Error t value Pr(>|t|)
## (Intercept)                         2.041e-01  3.529e-01   0.578   0.5631
## hormone_scr_ert_mean               -2.382e-03  1.475e-03  -1.615   0.1065
## hormone_sal_end_min_since_midnight -3.032e-04  1.211e-04  -2.504   0.0124 *
## interview_age                      -1.343e-04  2.786e-03  -0.048   0.9616
## MRI_minus_hormone_date_time         3.233e-06  2.852e-06   1.134   0.2571
## bmi                                 6.259e-03  5.663e-03   1.105   0.2692
## ---
## Signif. codes:  0 '***' 0.001 '**' 0.01 '*' 0.05 '.' 0.1 ' ' 1
##
##
## R-sq.(adj) =  0.00255
## lmer.REML = 4992.8  Scale est. = 0.82384   n = 1862
```

```
##                                         stdcoef      stdse
## X(Intercept)                         0.000000000 0.00000000
## Xhormone_scr_ert_mean               -0.038376921 0.02376535
## Xhormone_sal_end_min_since_midnight -0.059804205 0.02388165
## Xinterview_age                      -0.001131791 0.02347490
## XMRI_minus_hormone_date_time         0.027095786 0.02390339
## Xbmi                                 0.025996053 0.02351956
```

2.15 Model: Putamen Feedback ~ Testosterone

Female participants

```
## Warning: Some predictor variables are on very different scales: consider
## rescaling
```

```
##
## Family: gaussian
## Link function: identity
##
## Formula:
## putamen_posvsneg_feedback_z ~ hormone_scr_ert_mean + hormone_sal_end_min_since_midnight +
##     interview_age + MRI_minus_hormone_date_time + bmi
##
## Parametric coefficients:
##                                     Estimate Std. Error t value Pr(>|t|)
## (Intercept)                        3.559e-01  3.399e-01   1.047   0.2952
## hormone_scr_ert_mean               1.859e-04  1.233e-03   0.151   0.8802
## hormone_sal_end_min_since_midnight -3.255e-04  1.212e-04  -2.686   0.0073 **
## interview_age                     -1.146e-03  2.694e-03  -0.425   0.6705
## MRI_minus_hormone_date_time        3.695e-07  2.522e-06   0.146   0.8835
## bmi                                1.078e-03  5.311e-03   0.203   0.8391
## ---
## Signif. codes:  0 '***' 0.001 '**' 0.01 '*' 0.05 '.' 0.1 ' ' 1
##
##
## R-sq.(adj) =  0.00203
## lmer.REML = 4709.2  Scale est. = 0.70478   n = 1857
```

```
##                                          stdcoef      stdse
## X(Intercept)                         0.000000000 0.00000000
## Xhormone_scr_ert_mean                0.003654973 0.02423798
## Xhormone_sal_end_min_since_midnight -0.067547150 0.02515141
## Xinterview_age                      -0.010166339 0.02389360
## XMRI_minus_hormone_date_time         0.003516761 0.02400598
## Xbmi                                 0.004831829 0.02379334
```

Male participants

```
## Warning: Some predictor variables are on very different scales: consider
## rescaling
```

```
##
## Family: gaussian
## Link function: identity
##
## Formula:
## putamen_posvsneg_feedback_z ~ hormone_scr_ert_mean + hormone_sal_end_min_since_midnight +
##     interview_age + MRI_minus_hormone_date_time + bmi
##
## Parametric coefficients:
##                                     Estimate Std. Error t value Pr(>|t|)
```

```
## (Intercept)                         3.879e-01  3.483e-01   1.114   0.2656
## hormone_scr_ert_mean               -2.813e-03  1.469e-03  -1.915   0.0557 .
## hormone_sal_end_min_since_midnight -1.857e-04  1.279e-04  -1.452   0.1466
## interview_age                      -2.682e-03  2.751e-03  -0.975   0.3296
## MRI_minus_hormone_date_time         2.128e-06  2.844e-06   0.748   0.4543
## bmi                                 9.945e-03  5.597e-03   1.777   0.0758 .
## ---
## Signif. codes:  0 '***' 0.001 '**' 0.01 '*' 0.05 '.' 0.1 ' ' 1
##
##
## R-sq.(adj) =  0.00194
## lmer.REML = 4936.9  Scale est. = 0.78616   n = 1866
```

```
##                                        stdcoef      stdse
## X(Intercept)                         0.00000000 0.00000000
## Xhormone_scr_ert_mean               -0.04602278 0.02403324
## Xhormone_sal_end_min_since_midnight -0.03714332 0.02557454
## Xinterview_age                      -0.02295388 0.02353925
## XMRI_minus_hormone_date_time         0.01809190 0.02417495
## Xbmi                                 0.04197442 0.02362293
```

2.16 Model: Accumbens Feedback ~ Testosterone

Female participants

```
## Warning: Some predictor variables are on very different scales: consider
## rescaling
```

```
##
## Family: gaussian
## Link function: identity
##
## Formula:
## accumbens_posvsneg_feedback_z ~ hormone_scr_ert_mean + hormone_sal_end_min_since_midnight +
##     interview_age + MRI_minus_hormone_date_time + bmi
##
## Parametric coefficients:
##                                       Estimate Std. Error t value Pr(>|t|)
## (Intercept)                         -3.594e-01  2.744e-01  -1.310    0.190
## hormone_scr_ert_mean               -3.017e-04  9.935e-04  -0.304    0.761
## hormone_sal_end_min_since_midnight -8.185e-05  9.620e-05  -0.851    0.395
## interview_age                       2.820e-03  2.174e-03   1.297    0.195
## MRI_minus_hormone_date_time         6.440e-07  2.024e-06   0.318    0.750
## bmi                                 4.567e-03  4.284e-03   1.066    0.286
##
##
## R-sq.(adj) =  -0.000194
## lmer.REML = 3907.3  Scale est. = 0.43899   n = 1853
```

```
##                                         stdcoef      stdse
## X(Intercept)                         0.000000000 0.00000000
## Xhormone_scr_ert_mean               -0.007371565 0.02427177
```

```
## Xhormone_sal_end_min_since_midnight -0.021099616 0.02479750
## Xinterview_age                       0.031051368 0.02393974
## XMRI_minus_hormone_date_time         0.007631662 0.02398740
## Xbmi                                 0.025452793 0.02387189
```

Male participants

```
## Warning: Some predictor variables are on very different scales: consider
## rescaling
```

```
##
## Family: gaussian
## Link function: identity
##
## Formula:
## accumbens_posvsneg_feedback_z ~ hormone_scr_ert_mean + hormone_sal_end_min_since_midnight +
##     interview_age + MRI_minus_hormone_date_time + bmi
##
## Parametric coefficients:
##                                      Estimate Std. Error t value Pr(>|t|)
## (Intercept)                         3.484e-02  2.855e-01   0.122   0.9029
## hormone_scr_ert_mean               -1.970e-03  1.206e-03  -1.633   0.1026
## hormone_sal_end_min_since_midnight -1.261e-04  1.041e-04  -1.211   0.2259
## interview_age                      -1.692e-04  2.255e-03  -0.075   0.9402
## MRI_minus_hormone_date_time        -2.119e-06  2.389e-06  -0.887   0.3754
## bmi                                 8.528e-03  4.620e-03   1.846   0.0651 .
## ---
## Signif. codes:  0 '***' 0.001 '**' 0.01 '*' 0.05 '.' 0.1 ' ' 1
##
##
## R-sq.(adj) =  0.00181
## lmer.REML = 4180.1  Scale est. = 0.49593   n = 1861
```

```
##                                         stdcoef      stdse
## X(Intercept)                         0.00000000 0.00000000
## Xhormone_scr_ert_mean               -0.03930039 0.02406188
## Xhormone_sal_end_min_since_midnight -0.03083466 0.02545299
## Xinterview_age                      -0.00176689 0.02355255
## XMRI_minus_hormone_date_time        -0.02145408 0.02419669
## Xbmi                                 0.04371744 0.02368460
```

2.17 Model: OFC Anticipation ~ Testosterone

Female participants

```
## Warning: Some predictor variables are on very different scales: consider
## rescaling
```

```
##
## Family: gaussian
## Link function: identity
##
```

```
## Formula:
## lOFC_rvsn_ant_z ~ hormone_scr_ert_mean + hormone_sal_end_min_since_midnight +
##     interview_age + MRI_minus_hormone_date_time + bmi
##
## Parametric coefficients:
##                                      Estimate Std. Error t value Pr(>|t|)
## (Intercept)                         1.633e-01  2.223e-01   0.734   0.4628
## hormone_scr_ert_mean                1.662e-03  8.039e-04   2.067   0.0389 *
## hormone_sal_end_min_since_midnight -7.882e-05  7.681e-05  -1.026   0.3049
## interview_age                      -1.223e-03  1.762e-03  -0.695   0.4875
## MRI_minus_hormone_date_time        -1.728e-06  1.636e-06  -1.056   0.2909
## bmi                                -2.456e-04  3.464e-03  -0.071   0.9435
## ---
## Signif. codes:  0 '***' 0.001 '**' 0.01 '*' 0.05 '.' 0.1 ' ' 1
##
##
## R-sq.(adj) =  0.00119
## lmer.REML = 3120.1  Scale est. = 0.3033    n = 1846

##                                          stdcoef      stdse
## X(Intercept)                         0.000000000 0.00000000
## Xhormone_scr_ert_mean                0.050102372 0.02424050
## Xhormone_sal_end_min_since_midnight -0.025100771 0.02446024
## Xinterview_age                      -0.016634037 0.02395101
## XMRI_minus_hormone_date_time        -0.025260602 0.02391147
## Xbmi                                -0.001690531 0.02383915

## Warning: Some predictor variables are on very different scales: consider
## rescaling

##
## Family: gaussian
## Link function: identity
##
## Formula:
## mOFC_rvsn_ant_z ~ hormone_scr_ert_mean + hormone_sal_end_min_since_midnight +
##     interview_age + MRI_minus_hormone_date_time + bmi
##
## Parametric coefficients:
##                                      Estimate Std. Error t value Pr(>|t|)
## (Intercept)                         3.424e-01  2.608e-01   1.313    0.189
## hormone_scr_ert_mean                1.362e-03  9.417e-04   1.447    0.148
## hormone_sal_end_min_since_midnight -3.727e-05  9.118e-05  -0.409    0.683
## interview_age                      -2.918e-03  2.067e-03  -1.411    0.158
## MRI_minus_hormone_date_time         5.643e-07  1.920e-06   0.294    0.769
## bmi                                -1.155e-03  4.058e-03  -0.285    0.776
##
##
## R-sq.(adj) =  -0.000928
## lmer.REML =   3700  Scale est. = 0.41462   n = 1847

##                                          stdcoef      stdse
## X(Intercept)                         0.000000000 0.00000000
```

```
## Xhormone_scr_ert_mean                0.035151754 0.02430062
## Xhormone_sal_end_min_since_midnight -0.010139608 0.02480430
## Xinterview_age                      -0.033873347 0.02400020
## XMRI_minus_hormone_date_time         0.007060139 0.02402451
## Xbmi                                -0.006802320 0.02388850
```

Male participants

```
## Warning: Some predictor variables are on very different scales: consider
## rescaling
```

```
##
## Family: gaussian
## Link function: identity
##
## Formula:
## lOFC_rvsn_ant_z ~ hormone_scr_ert_mean + hormone_sal_end_min_since_midnight +
##     interview_age + MRI_minus_hormone_date_time + bmi
##
## Parametric coefficients:
##                                      Estimate Std. Error t value Pr(>|t|)
## (Intercept)                        -4.165e-01  2.415e-01  -1.725   0.0847 .
## hormone_scr_ert_mean               -1.104e-03  1.015e-03  -1.087   0.2772
## hormone_sal_end_min_since_midnight  1.147e-04  8.624e-05   1.330   0.1835
## interview_age                       3.145e-03  1.908e-03   1.648   0.0995 .
## MRI_minus_hormone_date_time        -1.608e-06  2.023e-06  -0.795   0.4269
## bmi                                -2.415e-03  3.888e-03  -0.621   0.5345
## ---
## Signif. codes:  0 '***' 0.001 '**' 0.01 '*' 0.05 '.' 0.1 ' ' 1
##
##
## R-sq.(adj) =  0.000887
## lmer.REML = 3541.2  Scale est. = 0.38017   n = 1849
```

```
##                                        stdcoef      stdse
## X(Intercept)                         0.00000000 0.00000000
## Xhormone_scr_ert_mean               -0.02613940 0.02404823
## Xhormone_sal_end_min_since_midnight  0.03313171 0.02490271
## Xinterview_age                       0.03897972 0.02364949
## XMRI_minus_hormone_date_time        -0.01924497 0.02421929
## Xbmi                                -0.01474407 0.02373317
```

```
## Warning: Some predictor variables are on very different scales: consider
## rescaling
```

```
##
## Family: gaussian
## Link function: identity
##
## Formula:
## mOFC_rvsn_ant_z ~ hormone_scr_ert_mean + hormone_sal_end_min_since_midnight +
##     interview_age + MRI_minus_hormone_date_time + bmi
```

```
##
## Parametric coefficients:
##                                      Estimate Std. Error t value Pr(>|t|)
## (Intercept)                        -1.552e-01  2.757e-01  -0.563    0.574
## hormone_scr_ert_mean               -8.129e-05  1.153e-03  -0.071    0.944
## hormone_sal_end_min_since_midnight  1.211e-04  9.495e-05   1.276    0.202
## interview_age                       5.885e-04  2.177e-03   0.270    0.787
## MRI_minus_hormone_date_time        -2.312e-06  2.286e-06  -1.011    0.312
## bmi                                -1.415e-03  4.413e-03  -0.321    0.748
##
##
## R-sq.(adj) =  -0.00146
## lmer.REML = 4044.7  Scale est. = 0.49948   n = 1853
```

```
##                                          stdcoef      stdse
## X(Intercept)                         0.000000000 0.00000000
## Xhormone_scr_ert_mean               -0.001684069 0.02388205
## Xhormone_sal_end_min_since_midnight  0.030657472 0.02403170
## Xinterview_age                       0.006372082 0.02357648
## XMRI_minus_hormone_date_time        -0.024329335 0.02405366
## Xbmi                                -0.007580023 0.02363578
```

2.18 Model: OFC Feedback ~ Testosterone

Female participants

```
## Warning: Some predictor variables are on very different scales: consider
## rescaling
```

```
##
## Family: gaussian
## Link function: identity
##
## Formula:
## lOFC_posvsneg_feedback_z ~ hormone_scr_ert_mean + hormone_sal_end_min_since_midnight +
##     interview_age + MRI_minus_hormone_date_time + bmi
##
## Parametric coefficients:
##                                      Estimate Std. Error t value Pr(>|t|)
## (Intercept)                         6.969e-02  1.983e-01   0.352   0.7252
## hormone_scr_ert_mean                1.333e-03  7.196e-04   1.853   0.0641 .
## hormone_sal_end_min_since_midnight -6.895e-05  6.801e-05  -1.014   0.3108
## interview_age                      -8.187e-04  1.574e-03  -0.520   0.6031
## MRI_minus_hormone_date_time        -2.228e-06  1.459e-06  -1.527   0.1269
## bmi                                 9.254e-04  3.094e-03   0.299   0.7649
## ---
## Signif. codes:  0 '***' 0.001 '**' 0.01 '*' 0.05 '.' 0.1 ' ' 1
##
##
## R-sq.(adj) =  0.00207
## lmer.REML = 2726.7  Scale est. = 0.24409   n = 1853
```

```
##                                          stdcoef      stdse
```

```
## X(Intercept)                            0.000000000 0.00000000
## Xhormone_scr_ert_mean                   0.044737037 0.02414910
## Xhormone_sal_end_min_since_midnight -0.024487829 0.02415402
## Xinterview_age                         -0.012409651 0.02385999
## XMRI_minus_hormone_date_time           -0.036315732 0.02378273
## Xbmi                                    0.007110948 0.02377586

## Warning: Some predictor variables are on very different scales: consider
## rescaling

##
## Family: gaussian
## Link function: identity
##
## Formula:
## mOFC_posvsneg_feedback_z ~ hormone_scr_ert_mean + hormone_sal_end_min_since_midnight +
##     interview_age + MRI_minus_hormone_date_time + bmi
##
## Parametric coefficients:
##                                       Estimate Std. Error t value Pr(>|t|)
## (Intercept)                         -8.624e-02  2.381e-01  -0.362    0.717
## hormone_scr_ert_mean                 1.328e-03  8.637e-04   1.538    0.124
## hormone_sal_end_min_since_midnight  1.808e-05  8.028e-05   0.225    0.822
## interview_age                        1.853e-04  1.889e-03   0.098    0.922
## MRI_minus_hormone_date_time         -1.567e-07  1.744e-06  -0.090    0.928
## bmi                                 -1.108e-03  3.718e-03  -0.298    0.766
##
##
## R-sq.(adj) =  -0.00132
## lmer.REML = 3423.2  Scale est. = 0.34969   n = 1857

##                                          stdcoef      stdse
## X(Intercept)                            0.000000000 0.00000000
## Xhormone_scr_ert_mean                   0.037044216 0.02408702
## Xhormone_sal_end_min_since_midnight  0.005335945 0.02369212
## Xinterview_age                          0.002335142 0.02380627
## XMRI_minus_hormone_date_time           -0.002123805 0.02364753
## Xbmi                                   -0.007076220 0.02374362
```

Male participants

```
## Warning: Some predictor variables are on very different scales: consider
## rescaling

##
## Family: gaussian
## Link function: identity
##
## Formula:
## lOFC_posvsneg_feedback_z ~ hormone_scr_ert_mean + hormone_sal_end_min_since_midnight +
##     interview_age + MRI_minus_hormone_date_time + bmi
##
```

```
## Parametric coefficients:
##                                      Estimate Std. Error t value Pr(>|t|)
## (Intercept)                         1.175e-02  2.134e-01   0.055    0.956
## hormone_scr_ert_mean               -2.512e-04  8.921e-04  -0.282    0.778
## hormone_sal_end_min_since_midnight -1.517e-04  7.382e-05  -2.055    0.040 *
## interview_age                       4.063e-04  1.685e-03   0.241    0.809
## MRI_minus_hormone_date_time         2.820e-06  1.730e-06   1.630    0.103
## bmi                                 3.248e-03  3.437e-03   0.945    0.345
## ---
## Signif. codes:  0 '***' 0.001 '**' 0.01 '*' 0.05 '.' 0.1 ' ' 1
##
##
## R-sq.(adj) =  0.00108
## lmer.REML = 3083.3  Scale est. = 0.26158   n = 1846
```

```
##                                          stdcoef      stdse
## X(Intercept)                         0.000000000 0.00000000
## Xhormone_scr_ert_mean               -0.006739477 0.02393281
## Xhormone_sal_end_min_since_midnight -0.049547081 0.02411355
## Xinterview_age                       0.005693600 0.02360541
## XMRI_minus_hormone_date_time         0.039237357 0.02407878
## Xbmi                                 0.022379157 0.02367673
```

```
## Warning: Some predictor variables are on very different scales: consider
## rescaling
```

```
##
## Family: gaussian
## Link function: identity
##
## Formula:
## mOFC_posvsneg_feedback_z ~ hormone_scr_ert_mean + hormone_sal_end_min_since_midnight +
##     interview_age + MRI_minus_hormone_date_time + bmi
##
## Parametric coefficients:
##                                      Estimate Std. Error t value Pr(>|t|)
## (Intercept)                         1.131e-01  2.510e-01   0.451   0.6522
## hormone_scr_ert_mean               -8.783e-04  1.051e-03  -0.836   0.4034
## hormone_sal_end_min_since_midnight -1.865e-04  8.712e-05  -2.140   0.0325 *
## interview_age                       2.904e-04  1.982e-03   0.147   0.8835
## MRI_minus_hormone_date_time         4.651e-07  2.092e-06   0.222   0.8241
## bmi                                 2.485e-03  4.044e-03   0.615   0.5389
## ---
## Signif. codes:  0 '***' 0.001 '**' 0.01 '*' 0.05 '.' 0.1 ' ' 1
##
##
## R-sq.(adj) =  0.000314
## lmer.REML = 3699.9  Scale est. = 0.37065   n = 1852
```

```
##                                          stdcoef      stdse
## X(Intercept)                         0.000000000 0.00000000
## Xhormone_scr_ert_mean               -0.019991357 0.02392200
## Xhormone_sal_end_min_since_midnight -0.051719729 0.02416670
```

```
## Xinterview_age                        0.003455419 0.02357937
## XMRI_minus_hormone_date_time         0.005350916 0.02407414
## Xbmi                                 0.014540207 0.02365941
```

2.19 Model: MID Reaction Time ~ Testosterone

Female participants

```
##
## Family: gaussian
## Link function: identity
##
## Formula:
## rt_diff_large_neutral_z ~ hormone_scr_ert_mean + hormone_sal_end_min_since_midnight +
##     interview_age + bmi
##
## Parametric coefficients:
##                                       Estimate Std. Error t value Pr(>|t|)
## (Intercept)                         -5.564e-01  3.587e-01  -1.551    0.121
## hormone_scr_ert_mean                -1.459e-03  1.316e-03  -1.109    0.267
## hormone_sal_end_min_since_midnight -9.983e-05  1.178e-04  -0.847    0.397
## interview_age                        5.704e-03  2.844e-03   2.006    0.045 *
## bmi                                  2.045e-03  5.483e-03   0.373    0.709
## ---
## Signif. codes:  0 '***' 0.001 '**' 0.01 '*' 0.05 '.' 0.1 ' ' 1
##
##
## R-sq.(adj) =  0.000767
## lmer.REML = 5555.2  Scale est. = 0.74256   n = 2042
```

```
##                                          stdcoef      stdse
## X(Intercept)                          0.000000000 0.00000000
## Xhormone_scr_ert_mean                -0.025625598 0.02309860
## Xhormone_sal_end_min_since_midnight -0.018906431 0.02231432
## Xinterview_age                        0.045434993 0.02265001
## Xbmi                                  0.008470944 0.02271608
```

```
##
## Family: gaussian
## Link function: identity
##
## Formula:
## rt_diff_large_small_z ~ hormone_scr_ert_mean + hormone_sal_end_min_since_midnight +
##     interview_age + bmi
##
## Parametric coefficients:
##                                       Estimate Std. Error t value Pr(>|t|)
## (Intercept)                         -5.932e-01  3.726e-01  -1.592    0.112
## hormone_scr_ert_mean                -1.456e-03  1.369e-03  -1.064    0.287
## hormone_sal_end_min_since_midnight  3.826e-05  1.251e-04   0.306    0.760
## interview_age                        4.125e-03  2.955e-03   1.396    0.163
## bmi                                  7.293e-03  5.690e-03   1.282    0.200
##
```

```
##
## R-sq.(adj) =  6.26e-05
## lmer.REML = 5698.5  Scale est. = 0.83135   n = 2042
```

```
##                                         stdcoef      stdse
## X(Intercept)                        0.000000000 0.00000000
## Xhormone_scr_ert_mean              -0.024701741 0.02321250
## Xhormone_sal_end_min_since_midnight  0.006999856 0.02289198
## Xinterview_age                      0.031737930 0.02273792
## Xbmi                                0.029186060 0.02277121
```

Male participants

```
##
## Family: gaussian
## Link function: identity
##
## Formula:
## rt_diff_large_neutral_z ~ hormone_scr_ert_mean + hormone_sal_end_min_since_midnight +
##     interview_age + bmi
##
## Parametric coefficients:
##                                       Estimate Std. Error t value Pr(>|t|)
## (Intercept)                         -0.6540210  0.3345588  -1.955   0.0507 .
## hormone_scr_ert_mean                -0.0007424  0.0014015  -0.530   0.5963
## hormone_sal_end_min_since_midnight  0.0001120  0.0001151   0.973   0.3305
## interview_age                        0.0046631  0.0026631   1.751   0.0801 .
## bmi                                  0.0008948  0.0053748   0.166   0.8678
## ---
## Signif. codes:  0 '***' 0.001 '**' 0.01 '*' 0.05 '.' 0.1 ' ' 1
##
##
## R-sq.(adj) =  1.81e-05
## lmer.REML = 5740.5  Scale est. = 0.8481    n = 2128
```

```
##                                         stdcoef      stdse
## X(Intercept)                        0.000000000 0.00000000
## Xhormone_scr_ert_mean              -0.011853868 0.02237617
## Xhormone_sal_end_min_since_midnight  0.021813886 0.02241059
## Xinterview_age                      0.038658848 0.02207794
## Xbmi                                0.003681314 0.02211377
```

```
##
## Family: gaussian
## Link function: identity
##
## Formula:
## rt_diff_large_small_z ~ hormone_scr_ert_mean + hormone_sal_end_min_since_midnight +
##     interview_age + bmi
##
## Parametric coefficients:
##                                       Estimate Std. Error t value Pr(>|t|)
## (Intercept)                         -4.621e-02  3.308e-01  -0.140    0.889
```

```
## hormone_scr_ert_mean               -1.401e-03  1.381e-03  -1.015    0.310
## hormone_sal_end_min_since_midnight -6.495e-06  1.110e-04  -0.059    0.953
## interview_age                       2.143e-04  2.633e-03   0.081    0.935
## bmi                                 2.723e-03  5.310e-03   0.513    0.608
##
##
## R-sq.(adj) =  -0.00133
## lmer.REML = 5704.9  Scale est. = 0.83586   n = 2128
```

```
##                                          stdcoef      stdse
## X(Intercept)                         0.000000000 0.00000000
## Xhormone_scr_ert_mean               -0.022576299 0.02225011
## Xhormone_sal_end_min_since_midnight -0.001276304 0.02181536
## Xinterview_age                       0.001793171 0.02203032
## Xbmi                                 0.011308848 0.02204872
```

2.20 Model: BIS-BAS-RR ~ Testosterone

Female participants

```
##
## Family: gaussian
## Link function: identity
##
## Formula:
## bisbas_ss_basm_rr_z ~ hormone_scr_ert_mean + hormone_sal_end_min_since_midnight +
##     interview_age + bmi
##
## Parametric coefficients:
##                                      Estimate Std. Error t value Pr(>|t|)
## (Intercept)                        -0.2620702  0.3517366  -0.745 0.456298
## hormone_scr_ert_mean               -0.0002111  0.0012958  -0.163 0.870628
## hormone_sal_end_min_since_midnight  0.0004106  0.0001222   3.359 0.000793 ***
## interview_age                      -0.0036414  0.0027825  -1.309 0.190754
## bmi                                 0.0180281  0.0053584   3.364 0.000779 ***
## ---
## Signif. codes:  0 '***' 0.001 '**' 0.01 '*' 0.05 '.' 0.1 ' ' 1
##
##
## R-sq.(adj) =  0.0126
## lmer.REML = 6907.2  Scale est. = 0.79616   n = 2435
```

```
##                                          stdcoef      stdse
## X(Intercept)                         0.000000000 0.00000000
## Xhormone_scr_ert_mean               -0.003462435 0.02125782
## Xhormone_sal_end_min_since_midnight  0.072805079 0.02167162
## Xinterview_age                      -0.027200344 0.02078397
## Xbmi                                 0.069923420 0.02078308
```

Male participants

```
##
```

```
## Family: gaussian
## Link function: identity
##
## Formula:
## bisbas_ss_basm_rr_z ~ hormone_scr_ert_mean + hormone_sal_end_min_since_midnight +
##     interview_age + bmi
##
## Parametric coefficients:
##                                     Estimate Std. Error t value Pr(>|t|)
## (Intercept)                       -0.0560894  0.3154876  -0.178   0.8589
## hormone_scr_ert_mean              -0.0022606  0.0013147  -1.719   0.0857 .
## hormone_sal_end_min_since_midnight  0.0001184  0.0001132   1.045   0.2960
## interview_age                      0.0004637  0.0025160   0.184   0.8538
## bmi                                0.0028392  0.0049884   0.569   0.5693
## ---
## Signif. codes:  0 '***' 0.001 '**' 0.01 '*' 0.05 '.' 0.1 ' ' 1
##
##
## R-sq.(adj) =  -0.000235
## lmer.REML = 7386.9  Scale est. = 0.74108   n = 2660
```

```
##                                          stdcoef      stdse
## X(Intercept)                         0.000000000 0.00000000
## Xhormone_scr_ert_mean               -0.034717343 0.02019126
## Xhormone_sal_end_min_since_midnight  0.022113435 0.02115513
## Xinterview_age                       0.003645394 0.01977897
## Xbmi                                 0.011321096 0.01989058
```

3—Internalizing~Reward—

3.1 Model: CBCL internalizing factor ~ Nucleus Accumbens activity (anticipation stage)

Female participants

```
##
## Family: gaussian
## Link function: identity
##
## Formula:
## cbcl_scr_syn_internal_r ~ accumbens_rvsn_ant_z + interview_age
##
## Parametric coefficients:
##                      Estimate Std. Error t value Pr(>|t|)
## (Intercept)           0.93807    1.89253   0.496   0.6202
## accumbens_rvsn_ant_z -0.25017    0.16587  -1.508   0.1317
## interview_age         0.03348    0.01579   2.120   0.0341 *
## ---
## Signif. codes:  0 '***' 0.001 '**' 0.01 '*' 0.05 '.' 0.1 ' ' 1
##
##
## R-sq.(adj) =  0.000946
## lmer.REML =  12780  Scale est. = 15.797    n = 2065
```

```
##                           stdcoef      stdse
## X(Intercept)           0.00000000 0.00000000
## Xaccumbens_rvsn_ant_z -0.03253184 0.02156971
## Xinterview_age         0.04644300 0.02190347
```

Male participants

```
##
## Family: gaussian
## Link function: identity
##
## Formula:
## cbcl_scr_syn_internal_r ~ accumbens_rvsn_ant_z + interview_age
##
## Parametric coefficients:
##                      Estimate Std. Error t value Pr(>|t|)
## (Intercept)           2.81159    1.92279   1.462    0.144
## accumbens_rvsn_ant_z  0.03617    0.16810   0.215    0.830
## interview_age         0.01815    0.01601   1.134    0.257
##
##
## R-sq.(adj) =  -0.00128
## lmer.REML =  12801  Scale est. = 14.906    n = 2046
```

```
##                           stdcoef      stdse
## X(Intercept)          0.000000000 0.00000000
```

```
## Xaccumbens_rvsn_ant_z 0.004671257 0.02171120
## Xinterview_age        0.024747561 0.02183056
```

3.2 Model: CBCL internalizing factor ~ Caudate activity (anticipation stage)

Female participants

```
##
## Family: gaussian
## Link function: identity
##
## Formula:
## cbcl_scr_syn_internal_r ~ caudate_rvsn_ant_z + interview_age
##
## Parametric coefficients:
##                    Estimate Std. Error t value Pr(>|t|)
## (Intercept)         1.23747    1.89544   0.653   0.5139
## caudate_rvsn_ant_z  0.03828    0.13153   0.291   0.7710
## interview_age       0.03104    0.01581   1.963   0.0498 *
## ---
## Signif. codes:  0 '***' 0.001 '**' 0.01 '*' 0.05 '.' 0.1 ' ' 1
##
##
## R-sq.(adj) =  -0.000229
## lmer.REML =  12819  Scale est. = 16.059    n = 2069
```

```
##                         stdcoef      stdse
## X(Intercept)        0.000000000 0.00000000
## Xcaudate_rvsn_ant_z 0.006289614 0.02160995
## Xinterview_age      0.042973215 0.02189105
```

Male participants

```
##
## Family: gaussian
## Link function: identity
##
## Formula:
## cbcl_scr_syn_internal_r ~ caudate_rvsn_ant_z + interview_age
##
## Parametric coefficients:
##                    Estimate Std. Error t value Pr(>|t|)
## (Intercept)         2.82781    1.92247   1.471    0.141
## caudate_rvsn_ant_z  0.17149    0.13191   1.300    0.194
## interview_age       0.01835    0.01602   1.145    0.252
##
##
## R-sq.(adj) =  -0.000683
## lmer.REML =  12841  Scale est. = 14.03     n = 2051
```

```
##                        stdcoef      stdse
## X(Intercept)        0.00000000 0.00000000
```

```
## Xcaudate_rvsn_ant_z 0.02799388 0.02153345
## Xinterview_age      0.02494894 0.02178028
```

3.3 Model: CBCL internalizing factor ~ Putamen activity (anticipation stage)

Female participants

```
##
## Family: gaussian
## Link function: identity
##
## Formula:
## cbcl_scr_syn_internal_r ~ putamen_rvsn_ant_z + interview_age
##
## Parametric coefficients:
##                    Estimate Std. Error t value Pr(>|t|)
## (Intercept)        1.055560   1.894179   0.557   0.5774
## putamen_rvsn_ant_z 0.002893   0.132368   0.022   0.9826
## interview_age      0.032556   0.015806   2.060   0.0395 *
## ---
## Signif. codes:  0 '***' 0.001 '**' 0.01 '*' 0.05 '.' 0.1 ' ' 1
##
##
## R-sq.(adj) =  -0.000189
## lmer.REML =  12818  Scale est. = 15.798    n = 2069
```

```
##                          stdcoef      stdse
## X(Intercept)        0.0000000000 0.00000000
## Xputamen_rvsn_ant_z 0.0004723149 0.02160736
## Xinterview_age      0.0450895285 0.02189021
```

Male participants

```
##
## Family: gaussian
## Link function: identity
##
## Formula:
## cbcl_scr_syn_internal_r ~ putamen_rvsn_ant_z + interview_age
##
## Parametric coefficients:
##                     Estimate Std. Error t value Pr(>|t|)
## (Intercept)         3.003713   1.926023   1.560    0.119
## putamen_rvsn_ant_z -0.002347   0.135141  -0.017    0.986
## interview_age       0.016796   0.016046   1.047    0.295
##
##
## R-sq.(adj) =  -0.00129
## lmer.REML =  12834  Scale est. = 14.129    n = 2050
```

```
##                           stdcoef      stdse
## X(Intercept)         0.0000000000 0.00000000
```

```
## Xputamen_rvsn_ant_z -0.0003768869 0.02170540
## Xinterview_age       0.0228301843 0.02181099
```

3.4 Model: CBCL internalizing factor ~ Accumbens activity (feedback stage)

Female participants

```
##
## Family: gaussian
## Link function: identity
##
## Formula:
## cbcl_scr_syn_internal_r ~ accumbens_posvsneg_feedback_z + interview_age
##
## Parametric coefficients:
##                               Estimate Std. Error t value Pr(>|t|)
## (Intercept)                    0.99988    1.89645   0.527   0.5981
## accumbens_posvsneg_feedback_z  0.19684    0.17181   1.146   0.2521
## interview_age                  0.03298    0.01582   2.085   0.0372 *
## ---
## Signif. codes:  0 '***' 0.001 '**' 0.01 '*' 0.05 '.' 0.1 ' ' 1
##
##
## R-sq.(adj) =  0.00028
## lmer.REML =  12781  Scale est. = 15.798    n = 2064
```

```
##                                     stdcoef      stdse
## X(Intercept)                    0.00000000 0.00000000
## Xaccumbens_posvsneg_feedback_z 0.02475519 0.02160702
## Xinterview_age                  0.04568771 0.02191529
```

Male participants

```
##
## Family: gaussian
## Link function: identity
##
## Formula:
## cbcl_scr_syn_internal_r ~ accumbens_posvsneg_feedback_z + interview_age
##
## Parametric coefficients:
##                               Estimate Std. Error t value Pr(>|t|)
## (Intercept)                    3.36478    1.93092   1.743   0.0816 .
## accumbens_posvsneg_feedback_z -0.22281    0.16571  -1.345   0.1789
## interview_age                  0.01399    0.01609   0.869   0.3847
## ---
## Signif. codes:  0 '***' 0.001 '**' 0.01 '*' 0.05 '.' 0.1 ' ' 1
##
##
## R-sq.(adj) =  -0.000388
## lmer.REML =  12833  Scale est. = 14.8      n = 2049
```

```
##                                    stdcoef      stdse
## X(Intercept)                    0.00000000 0.00000000
## Xaccumbens_posvsneg_feedback_z -0.02927669 0.02177379
## Xinterview_age                  0.01895670 0.02180197
```

3.5 Model: CBCL internalizing factor ~ Caudate activity (feedback stage)

Female participants

```
##
## Family: gaussian
## Link function: identity
##
## Formula:
## cbcl_scr_syn_internal_r ~ caudate_posvsneg_feedback_z + interview_age
##
## Parametric coefficients:
##                             Estimate Std. Error t value Pr(>|t|)
## (Intercept)                  0.99119    1.89617   0.523   0.6012
## caudate_posvsneg_feedback_z -0.03668    0.13461  -0.272   0.7853
## interview_age                0.03312    0.01582   2.094   0.0364 *
## ---
## Signif. codes:  0 '***' 0.001 '**' 0.01 '*' 0.05 '.' 0.1 ' ' 1
##
##
## R-sq.(adj) =  -0.000154
## lmer.REML =  12789  Scale est. = 15.852    n = 2065
```

```
##                                   stdcoef      stdse
## X(Intercept)                  0.000000000 0.00000000
## Xcaudate_posvsneg_feedback_z -0.005903269 0.02166702
## Xinterview_age                0.045857838 0.02190158
```

Male participants

```
##
## Family: gaussian
## Link function: identity
##
## Formula:
## cbcl_scr_syn_internal_r ~ caudate_posvsneg_feedback_z + interview_age
##
## Parametric coefficients:
##                             Estimate Std. Error t value Pr(>|t|)
## (Intercept)                  3.32207    1.92624   1.725   0.0847 .
## caudate_posvsneg_feedback_z -0.15618    0.13309  -1.174   0.2407
## interview_age                0.01417    0.01606   0.883   0.3775
## ---
## Signif. codes:  0 '***' 0.001 '**' 0.01 '*' 0.05 '.' 0.1 ' ' 1
##
##
## R-sq.(adj) =  -0.000217
## lmer.REML =  12843  Scale est. = 14.85     n = 2051
```

```
##                                   stdcoef      stdse
## X(Intercept)                   0.00000000 0.00000000
## Xcaudate_posvsneg_feedback_z -0.02531019 0.02156786
## Xinterview_age                 0.01925548 0.02181229
```

3.6 Model: CBCL internalizing factor ~ Putamen activity (feedback stage)

Female participants

```
##
## Family: gaussian
## Link function: identity
##
## Formula:
## cbcl_scr_syn_internal_r ~ putamen_posvsneg_feedback_z + interview_age
##
## Parametric coefficients:
##                             Estimate Std. Error t value Pr(>|t|)
## (Intercept)                  1.13313    1.89586   0.598   0.5501
## putamen_posvsneg_feedback_z -0.11704    0.13669  -0.856   0.3919
## interview_age                0.03186    0.01582   2.014   0.0442 *
## ---
## Signif. codes:  0 '***' 0.001 '**' 0.01 '*' 0.05 '.' 0.1 ' ' 1
##
##
## R-sq.(adj) =  0.000199
## lmer.REML =  12792  Scale est. = 16.215    n = 2065
```

```
##                                   stdcoef      stdse
## X(Intercept)                   0.00000000 0.00000000
## Xputamen_posvsneg_feedback_z -0.01856874 0.02168511
## Xinterview_age                 0.04414924 0.02192297
```

Male participants

```
##
## Family: gaussian
## Link function: identity
##
## Formula:
## cbcl_scr_syn_internal_r ~ putamen_posvsneg_feedback_z + interview_age
##
## Parametric coefficients:
##                             Estimate Std. Error t value Pr(>|t|)
## (Intercept)                  3.23873    1.92503   1.682   0.0926 .
## putamen_posvsneg_feedback_z -0.04213    0.13493  -0.312   0.7549
## interview_age                0.01493    0.01604   0.931   0.3521
## ---
## Signif. codes:  0 '***' 0.001 '**' 0.01 '*' 0.05 '.' 0.1 ' ' 1
##
##
## R-sq.(adj) =  -0.00116
## lmer.REML =  12878  Scale est. = 15.039    n = 2056
```

```
##                                   stdcoef     stdse
## X(Intercept)                 0.000000000 0.0000000
## Xputamen_posvsneg_feedback_z -0.006749898 0.0216173
## Xinterview_age               0.020279347 0.0217891
```

3.7 Model: CBCL internalizing factor ~ OFC activity (anticipation stage)

Female participants

```
##
## Family: gaussian
## Link function: identity
##
## Formula:
## cbcl_scr_syn_internal_r ~ lOFC_rvsn_ant_z + interview_age
##
## Parametric coefficients:
##                 Estimate Std. Error t value Pr(>|t|)
## (Intercept)      1.00430    1.90118   0.528   0.5974
## lOFC_rvsn_ant_z  0.05371    0.20796   0.258   0.7962
## interview_age    0.03302    0.01586   2.082   0.0374 *
## ---
## Signif. codes:  0 '***' 0.001 '**' 0.01 '*' 0.05 '.' 0.1 ' ' 1
##
##
## R-sq.(adj) =  -8.98e-05
## lmer.REML =  12736  Scale est. = 15.567    n = 2056
```

```
##                      stdcoef      stdse
## X(Intercept)     0.000000000 0.00000000
## XlOFC_rvsn_ant_z 0.005568829 0.02156341
## Xinterview_age   0.045736012 0.02196480
```

```
##
## Family: gaussian
## Link function: identity
##
## Formula:
## cbcl_scr_syn_internal_r ~ mOFC_rvsn_ant_z + interview_age
##
## Parametric coefficients:
##                 Estimate Std. Error t value Pr(>|t|)
## (Intercept)      0.82010    1.90148   0.431   0.6663
## mOFC_rvsn_ant_z  0.17691    0.17881   0.989   0.3226
## interview_age    0.03454    0.01587   2.177   0.0296 *
## ---
## Signif. codes:  0 '***' 0.001 '**' 0.01 '*' 0.05 '.' 0.1 ' ' 1
##
##
## R-sq.(adj) =  0.000336
## lmer.REML =  12741  Scale est. = 15.138    n = 2057
```

```
##                     stdcoef      stdse
```

```
## X(Intercept)     0.00000000 0.00000000
## XmOFC_rvsn_ant_z 0.02118601 0.02141320
## Xinterview_age   0.04776384 0.02194123
```

Male participants

```
##
## Family: gaussian
## Link function: identity
##
## Formula:
## cbcl_scr_syn_internal_r ~ lOFC_rvsn_ant_z + interview_age
##
## Parametric coefficients:
##                 Estimate Std. Error t value Pr(>|t|)
## (Intercept)      2.62529    1.91357   1.372    0.170
## lOFC_rvsn_ant_z -0.10772    0.19356  -0.557    0.578
## interview_age    0.01966    0.01592   1.235    0.217
##
##
## R-sq.(adj) =  -0.00122
## lmer.REML =  12706  Scale est. = 13.821    n = 2036
```

```
##                      stdcoef      stdse
## X(Intercept)      0.00000000 0.00000000
## XlOFC_rvsn_ant_z -0.01198528 0.02153614
## Xinterview_age    0.02697017 0.02184451
```

```
##
## Family: gaussian
## Link function: identity
##
## Formula:
## cbcl_scr_syn_internal_r ~ mOFC_rvsn_ant_z + interview_age
##
## Parametric coefficients:
##                 Estimate Std. Error t value Pr(>|t|)
## (Intercept)      2.51935    1.92180   1.311    0.190
## mOFC_rvsn_ant_z -0.12792    0.16826  -0.760    0.447
## interview_age    0.02059    0.01600   1.287    0.198
##
##
## R-sq.(adj) =  -0.0012
## lmer.REML =  12765  Scale est. = 13.816    n = 2042
```

```
##                      stdcoef      stdse
## X(Intercept)      0.00000000 0.00000000
## XmOFC_rvsn_ant_z -0.01623520 0.02135579
## Xinterview_age    0.02803578 0.02178658
```

3.8 Model: CBCL internalizing factor ~ OFC activity (feedback stage)

Female participants

```
##
## Family: gaussian
## Link function: identity
##
## Formula:
## cbcl_scr_syn_internal_r ~ lOFC_posvsneg_feedback_z + interview_age
##
## Parametric coefficients:
##                          Estimate Std. Error t value Pr(>|t|)
## (Intercept)               0.98560    1.89246   0.521   0.6026
## lOFC_posvsneg_feedback_z -0.04673    0.23076  -0.203   0.8395
## interview_age             0.03302    0.01579   2.091   0.0366 *
## ---
## Signif. codes:  0 '***' 0.001 '**' 0.01 '*' 0.05 '.' 0.1 ' ' 1
##
##
## R-sq.(adj) =  -0.000146
## lmer.REML =  12779  Scale est. = 16.099    n = 2065
```

```
##                                 stdcoef      stdse
## X(Intercept)               0.000000000 0.00000000
## XlOFC_posvsneg_feedback_z -0.004375458 0.02160700
## Xinterview_age             0.045862853 0.02192932
```

```
##
## Family: gaussian
## Link function: identity
##
## Formula:
## cbcl_scr_syn_internal_r ~ mOFC_posvsneg_feedback_z + interview_age
##
## Parametric coefficients:
##                          Estimate Std. Error t value Pr(>|t|)
## (Intercept)               0.92801    1.89200   0.490   0.6238
## mOFC_posvsneg_feedback_z  0.20371    0.19485   1.046   0.2959
## interview_age             0.03360    0.01578   2.129   0.0334 *
## ---
## Signif. codes:  0 '***' 0.001 '**' 0.01 '*' 0.05 '.' 0.1 ' ' 1
##
##
## R-sq.(adj) =  0.000549
## lmer.REML =  12810  Scale est. = 15.903    n = 2069
```

```
##                               stdcoef      stdse
## X(Intercept)              0.00000000 0.00000000
## XmOFC_posvsneg_feedback_z 0.02252826 0.02154774
## Xinterview_age            0.04658350 0.02187988
```

Male participants

```
##
## Family: gaussian
## Link function: identity
##
## Formula:
## cbcl_scr_syn_internal_r ~ lOFC_posvsneg_feedback_z + interview_age
##
## Parametric coefficients:
##                          Estimate Std. Error t value Pr(>|t|)
## (Intercept)               3.12278    1.93031   1.618    0.106
## lOFC_posvsneg_feedback_z  0.15275    0.22303   0.685    0.493
## interview_age             0.01593    0.01608   0.991    0.322
##
##
## R-sq.(adj) =  -0.00122
## lmer.REML =  12729  Scale est. = 14.972    n = 2035
```

```
##                              stdcoef      stdse
## X(Intercept)              0.00000000 0.00000000
## XlOFC_posvsneg_feedback_z 0.01493689 0.02180927
## Xinterview_age            0.02171403 0.02191158
```

```
##
## Family: gaussian
## Link function: identity
##
## Formula:
## cbcl_scr_syn_internal_r ~ mOFC_posvsneg_feedback_z + interview_age
##
## Parametric coefficients:
##                          Estimate Std. Error t value Pr(>|t|)
## (Intercept)               3.07567    1.92215   1.600    0.110
## mOFC_posvsneg_feedback_z  0.06058    0.18722   0.324    0.746
## interview_age             0.01633    0.01601   1.020    0.308
##
##
## R-sq.(adj) =  -0.00121
## lmer.REML =  12780  Scale est. = 14.999    n = 2044
```

```
##                               stdcoef      stdse
## X(Intercept)              0.000000000 0.00000000
## XmOFC_posvsneg_feedback_z 0.007034218 0.02174054
## Xinterview_age            0.022296228 0.02186698
```

3.9 Model: CBCL internalizing factor ~ BIS-BAS-RR

Female participants

```
##
## Family: gaussian
```

```
## Link function: identity
##
## Formula:
## cbcl_scr_syn_internal_r ~ bisbas_ss_basm_rr + interview_age
##
## Parametric coefficients:
##                   Estimate Std. Error t value Pr(>|t|)
## (Intercept)        1.21645    1.72886   0.704   0.4817
## bisbas_ss_basm_rr -0.02712    0.04321  -0.628   0.5303
## interview_age      0.03358    0.01401   2.398   0.0166 *
## ---
## Signif. codes:  0 '***' 0.001 '**' 0.01 '*' 0.05 '.' 0.1 ' ' 1
##
##
## R-sq.(adj) =  -0.000264
## lmer.REML =  16599  Scale est. = 17.025    n = 2681
```

```
##                        stdcoef      stdse
## X(Intercept)        0.00000000 0.00000000
## Xbisbas_ss_basm_rr -0.01201656 0.01914694
## Xinterview_age      0.04625226 0.01929145
```

Male participants

```
##
## Family: gaussian
## Link function: identity
##
## Formula:
## cbcl_scr_syn_internal_r ~ bisbas_ss_basm_rr + interview_age
##
## Parametric coefficients:
##                   Estimate Std. Error t value Pr(>|t|)
## (Intercept)        3.11239    1.69534   1.836   0.0665 .
## bisbas_ss_basm_rr -0.06658    0.04448  -1.497   0.1346
## interview_age      0.02135    0.01374   1.554   0.1203
## ---
## Signif. codes:  0 '***' 0.001 '**' 0.01 '*' 0.05 '.' 0.1 ' ' 1
##
##
## R-sq.(adj) =  -0.000597
## lmer.REML =  18107  Scale est. = 16.736    n = 2893
```

```
##                        stdcoef      stdse
## X(Intercept)        0.00000000 0.00000000
## Xbisbas_ss_basm_rr -0.02744649 0.01833863
## Xinterview_age      0.02873301 0.01848803
```

3.10 Model: CBCL internalizing factor ~ MID Reaction Time

Female participants

```
##
```

```
## Family: gaussian
## Link function: identity
##
## Formula:
## cbcl_scr_syn_internal_r ~ rt_diff_large_neutral_z + interview_age
##
## Parametric coefficients:
##                         Estimate Std. Error t value Pr(>|t|)
## (Intercept)              0.65045    1.84166   0.353   0.7240
## rt_diff_large_neutral_z  0.10720    0.12129   0.884   0.3769
## interview_age            0.03602    0.01536   2.346   0.0191 *
## ---
## Signif. codes:  0 '***' 0.001 '**' 0.01 '*' 0.05 '.' 0.1 ' ' 1
##
##
## R-sq.(adj) =  0.000286
## lmer.REML =  13864  Scale est. = 16.79     n = 2237
```

```
##                             stdcoef      stdse
## X(Intercept)             0.00000000 0.00000000
## Xrt_diff_large_neutral_z 0.01831347 0.02072078
## Xinterview_age           0.04932191 0.02102775
```

```
##
## Family: gaussian
## Link function: identity
##
## Formula:
## cbcl_scr_syn_internal_r ~ rt_diff_large_small_z + interview_age
##
## Parametric coefficients:
##                       Estimate Std. Error t value Pr(>|t|)
## (Intercept)            0.65522    1.84051   0.356   0.7219
## rt_diff_large_small_z  0.14840    0.11684   1.270   0.2042
## interview_age          0.03601    0.01535   2.346   0.0191 *
## ---
## Signif. codes:  0 '***' 0.001 '**' 0.01 '*' 0.05 '.' 0.1 ' ' 1
##
##
## R-sq.(adj) =  0.000654
## lmer.REML =  13864  Scale est. = 16.809    n = 2237
```

```
##                           stdcoef      stdse
## X(Intercept)           0.00000000 0.00000000
## Xrt_diff_large_small_z 0.02637377 0.02076472
## Xinterview_age         0.04929782 0.02101228
```

Male participants

```
##
## Family: gaussian
## Link function: identity
##
```

```
## Formula:
## cbcl_scr_syn_internal_r ~ rt_diff_large_neutral_z + interview_age
##
## Parametric coefficients:
##                         Estimate Std. Error t value Pr(>|t|)
## (Intercept)              2.27829    1.81447   1.256    0.209
## rt_diff_large_neutral_z -0.09028    0.12278  -0.735    0.462
## interview_age            0.02284    0.01512   1.511    0.131
##
##
## R-sq.(adj) =  -0.00078
## lmer.REML =  14407  Scale est. = 13.459    n = 2304
```

```
##                              stdcoef      stdse
## X(Intercept)              0.00000000 0.00000000
## Xrt_diff_large_neutral_z -0.01487991 0.02023669
## Xinterview_age            0.03106944 0.02055981
```

```
##
## Family: gaussian
## Link function: identity
##
## Formula:
## cbcl_scr_syn_internal_r ~ rt_diff_large_small_z + interview_age
##
## Parametric coefficients:
##                       Estimate Std. Error t value Pr(>|t|)
## (Intercept)            2.32101    1.81284   1.280    0.201
## rt_diff_large_small_z -0.12010    0.12369  -0.971    0.332
## interview_age          0.02249    0.01510   1.489    0.137
##
##
## R-sq.(adj) =  -0.000713
## lmer.REML =  14407  Scale est. = 13.408    n = 2304
```

```
##                            stdcoef      stdse
## X(Intercept)            0.00000000 0.00000000
## Xrt_diff_large_small_z -0.01958396 0.02016891
## Xinterview_age          0.03058289 0.02054226
```

4—Internalizing~Puberty x Reward—

4.1 Model: CBCL internalizing factor ~ PDS x Accumbens activity (anticipation stage)

Female participants

```
##
## Family: gaussian
## Link function: identity
##
## Formula:
## cbcl_scr_syn_internal_r ~ PDS_score * accumbens_rvsn_ant_z +
##     race.ethnicity.5level + demo_race_hispanic + interview_age +
##     bmi + household.income + high.educ
##
## Parametric coefficients:
##                                Estimate Std. Error t value Pr(>|t|)
## (Intercept)                     0.19987    2.43939   0.082   0.9347
## PDS_score                       0.79254    0.19269   4.113 4.08e-05 ***
## accumbens_rvsn_ant_z           -0.11644    0.43011  -0.271   0.7866
## race.ethnicity.5levelBlack     -0.54696    0.88663  -0.617   0.5374
## race.ethnicity.5levelMixed      1.03544    0.85146   1.216   0.2241
## race.ethnicity.5levelOther     -0.16088    1.02269  -0.157   0.8750
## race.ethnicity.5levelWhite      1.45279    0.79104   1.837   0.0664 .
## demo_race_hispanic1            -0.20403    0.38852  -0.525   0.5995
## interview_age                   0.02278    0.01742   1.307   0.1912
## bmi                             0.02269    0.03487   0.651   0.5153
## household.income[>=200K]       -1.88984    0.97221  -1.944   0.0521 .
## household.income[100K-200K]    -0.89020    0.90918  -0.979   0.3277
## household.income[12K-16K]       0.25208    1.12725   0.224   0.8231
## household.income[16K-25K]      -1.10913    1.03345  -1.073   0.2833
## household.income[25K-35K]       0.78057    0.95360   0.819   0.4131
## household.income[35K-50K]      -0.41598    0.92982  -0.447   0.6547
## household.income[50K-75K]      -0.73265    0.91346  -0.802   0.4226
## household.income[5K-12K]        0.55629    1.06287   0.523   0.6008
## household.income[75K-100K]     -0.62387    0.92037  -0.678   0.4980
## high.educBachelor              -0.31054    0.84267  -0.369   0.7125
## high.educHS Diploma/GED        -0.49667    0.85388  -0.582   0.5609
## high.educPost Graduate Degree   0.06275    0.85607   0.073   0.9416
## high.educSome College           0.36018    0.79327   0.454   0.6498
## PDS_score:accumbens_rvsn_ant_z -0.10649    0.23252  -0.458   0.6470
## ---
## Signif. codes:  0 '***' 0.001 '**' 0.01 '*' 0.05 '.' 0.1 ' ' 1
##
##
## R-sq.(adj) =  0.0285
## lmer.REML =  11325  Scale est. = 15.622    n = 1846
```

```
##                                      stdcoef      stdse
## X(Intercept)                     0.000000000 0.00000000
## XPDS_score                       0.107011992 0.02601736
## Xaccumbens_rvsn_ant_z           -0.014960479 0.05526203
```

```
## Xrace.ethnicity.5levelBlack     -0.034560375 0.05602296
## Xrace.ethnicity.5levelMixed      0.063797834 0.05246169
## Xrace.ethnicity.5levelOther     -0.006074813 0.03861621
## Xrace.ethnicity.5levelWhite      0.127058903 0.06918335
## Xdemo_race_hispanic1            -0.014771830 0.02812869
## Xinterview_age                   0.031799209 0.02432274
## Xbmi                             0.016025516 0.02462548
## Xhousehold.income[>=200K]       -0.113556047 0.05841779
## Xhousehold.income[100K-200K]    -0.078343337 0.08001394
## Xhousehold.income[12K-16K]       0.007252521 0.03243229
## Xhousehold.income[16K-25K]      -0.038975964 0.03631672
## Xhousehold.income[25K-35K]       0.035339503 0.04317302
## Xhousehold.income[35K-50K]      -0.021585969 0.04825047
## Xhousehold.income[50K-75K]      -0.045969663 0.05731450
## Xhousehold.income[5K-12K]        0.017645193 0.03371366
## Xhousehold.income[75K-100K]     -0.042175094 0.06221856
## Xhigh.educBachelor              -0.025565564 0.06937398
## Xhigh.educHS Diploma/GED        -0.024288047 0.04175622
## Xhigh.educPost Graduate Degree   0.005711212 0.07791975
## Xhigh.educSome College           0.028653464 0.06310652
## XPDS_score:accumbens_rvsn_ant_z -0.025316001 0.05528025
```

Male participants

```
##
## Family: gaussian
## Link function: identity
##
## Formula:
## cbcl_scr_syn_internal_r ~ PDS_score * accumbens_rvsn_ant_z +
##     race.ethnicity.5level + demo_race_hispanic + interview_age +
##     bmi + household.income + high.educ
##
## Parametric coefficients:
##                                 Estimate Std. Error t value Pr(>|t|)
## (Intercept)                     2.859246   2.569197   1.113 0.265901
## PDS_score                       0.746805   0.264678   2.822 0.004831 **
## accumbens_rvsn_ant_z            0.322388   0.441001   0.731 0.464851
## race.ethnicity.5levelBlack     -0.153525   1.087194  -0.141 0.887718
## race.ethnicity.5levelMixed      1.262630   1.059158   1.192 0.233375
## race.ethnicity.5levelOther      0.289358   1.196307   0.242 0.808904
## race.ethnicity.5levelWhite      1.163318   0.997636   1.166 0.243738
## demo_race_hispanic1             0.323762   0.408992   0.792 0.428693
## interview_age                   0.010786   0.017062   0.632 0.527358
## bmi                             0.012850   0.036989   0.347 0.728335
## household.income[>=200K]       -3.278609   0.990934  -3.309 0.000956 ***
## household.income[100K-200K]    -2.762172   0.934281  -2.956 0.003152 **
## household.income[12K-16K]      -1.157762   1.200897  -0.964 0.335134
## household.income[16K-25K]       0.001016   1.031449   0.001 0.999214
## household.income[25K-35K]      -0.651275   1.016740  -0.641 0.521895
## household.income[35K-50K]      -0.664395   0.975487  -0.681 0.495902
## household.income[50K-75K]      -2.268137   0.935495  -2.425 0.015425 *
## household.income[5K-12K]        0.169356   1.101038   0.154 0.877773
```

```
## household.income[75K-100K]     -2.902283   0.950458  -3.054 0.002294 **
## high.educBachelor               1.457488   0.961917   1.515 0.129898
## high.educHS Diploma/GED        -0.973618   0.987237  -0.986 0.324164
## high.educPost Graduate Degree   0.588792   0.961457   0.612 0.540353
## high.educSome College           0.873343   0.919136   0.950 0.342148
## PDS_score:accumbens_rvsn_ant_z -0.228251   0.300514  -0.760 0.447631
## ---
## Signif. codes:  0 '***' 0.001 '**' 0.01 '*' 0.05 '.' 0.1 ' ' 1
##
##
## R-sq.(adj) =  0.0365
## lmer.REML =  11401  Scale est. = 15.274    n = 1835
```

```
##                                      stdcoef      stdse
## X(Intercept)                    0.000000e+00 0.00000000
## XPDS_score                      6.874737e-02 0.02436500
## Xaccumbens_rvsn_ant_z           4.171684e-02 0.05706532
## Xrace.ethnicity.5levelBlack    -8.294041e-03 0.05873472
## Xrace.ethnicity.5levelMixed     7.251725e-02 0.06083113
## Xrace.ethnicity.5levelOther     1.086055e-02 0.04490136
## Xrace.ethnicity.5levelWhite     9.356175e-02 0.08023651
## Xdemo_race_hispanic1            2.251422e-02 0.02844112
## Xinterview_age                  1.462141e-02 0.02312906
## Xbmi                            8.340261e-03 0.02400803
## Xhousehold.income[>=200K]      -1.960010e-01 0.05923976
## Xhousehold.income[100K-200K]   -2.337609e-01 0.07906765
## Xhousehold.income[12K-16K]     -2.981900e-02 0.03092999
## Xhousehold.income[16K-25K]      3.662037e-05 0.03715945
## Xhousehold.income[25K-35K]     -2.498446e-02 0.03900457
## Xhousehold.income[35K-50K]     -3.128923e-02 0.04593996
## Xhousehold.income[50K-75K]     -1.387104e-01 0.05721120
## Xhousehold.income[5K-12K]       5.018424e-03 0.03262631
## Xhousehold.income[75K-100K]    -1.863685e-01 0.06103313
## Xhigh.educBachelor              1.152216e-01 0.07604422
## Xhigh.educHS Diploma/GED       -4.127320e-02 0.04185051
## Xhigh.educPost Graduate Degree  5.151005e-02 0.08411239
## Xhigh.educSome College          6.729733e-02 0.07082601
## XPDS_score:accumbens_rvsn_ant_z -4.337629e-02 0.05710894
```

4.2 Model: CBCL internalizing factor ~ PDS x Caudate activity (anticipation stage)

Female participants

```
##
## Family: gaussian
## Link function: identity
##
## Formula:
## cbcl_scr_syn_internal_r ~ PDS_score * caudate_rvsn_ant_z + race.ethnicity.5level +
##     demo_race_hispanic + interview_age + bmi + household.income +
##     high.educ
##
```

```
## Parametric coefficients:
##                              Estimate Std. Error t value Pr(>|t|)
## (Intercept)                   0.52927    2.43361   0.217   0.8279
## PDS_score                     0.76803    0.19272   3.985 7.01e-05 ***
## caudate_rvsn_ant_z            0.77370    0.34515   2.242   0.0251 *
## race.ethnicity.5levelBlack   -0.51173    0.88814  -0.576   0.5646
## race.ethnicity.5levelMixed    1.07930    0.85226   1.266   0.2055
## race.ethnicity.5levelOther   -0.07330    1.02515  -0.072   0.9430
## race.ethnicity.5levelWhite    1.52492    0.79237   1.925   0.0544 .
## demo_race_hispanic1          -0.22051    0.38852  -0.568   0.5704
## interview_age                 0.02131    0.01742   1.223   0.2213
## bmi                           0.03008    0.03466   0.868   0.3856
## household.income[>=200K]     -2.21288    0.96642  -2.290   0.0221 *
## household.income[100K-200K]  -1.20284    0.90240  -1.333   0.1827
## household.income[12K-16K]     0.06126    1.12824   0.054   0.9567
## household.income[16K-25K]    -1.36902    1.02749  -1.332   0.1829
## household.income[25K-35K]     0.43839    0.94424   0.464   0.6425
## household.income[35K-50K]    -0.68580    0.92095  -0.745   0.4566
## household.income[50K-75K]    -1.01690    0.90543  -1.123   0.2615
## household.income[5K-12K]      0.23352    1.05848   0.221   0.8254
## household.income[75K-100K]   -0.92123    0.91378  -1.008   0.3135
## high.educBachelor            -0.33766    0.83936  -0.402   0.6875
## high.educHS Diploma/GED      -0.49618    0.85416  -0.581   0.5614
## high.educPost Graduate Degree  0.09642    0.85272   0.113   0.9100
## high.educSome College         0.30288    0.79132   0.383   0.7019
## PDS_score:caudate_rvsn_ant_z -0.44649    0.19155  -2.331   0.0199 *
## ---
## Signif. codes:  0 '***' 0.001 '**' 0.01 '*' 0.05 '.' 0.1 ' ' 1
##
##
## R-sq.(adj) =  0.0296
## lmer.REML =  11351  Scale est. = 16.23     n = 1848
```

```
##                                    stdcoef      stdse
## X(Intercept)                     0.000000000 0.00000000
## XPDS_score                       0.103386830 0.02594317
## Xcaudate_rvsn_ant_z              0.127679675 0.05695820
## Xrace.ethnicity.5levelBlack     -0.032328048 0.05610742
## Xrace.ethnicity.5levelMixed      0.066748360 0.05270770
## Xrace.ethnicity.5levelOther     -0.002740822 0.03833191
## Xrace.ethnicity.5levelWhite      0.133189153 0.06920679
## Xdemo_race_hispanic1            -0.015925456 0.02805945
## Xinterview_age                   0.029680470 0.02425928
## Xbmi                             0.021343804 0.02459650
## Xhousehold.income[>=200K]       -0.132216431 0.05774189
## Xhousehold.income[100K-200K]    -0.105577357 0.07920656
## Xhousehold.income[12K-16K]       0.001736919 0.03198802
## Xhousehold.income[16K-25K]      -0.047930926 0.03597332
## Xhousehold.income[25K-35K]       0.019774365 0.04259193
## Xhousehold.income[35K-50K]      -0.035764538 0.04802750
## Xhousehold.income[50K-75K]      -0.063684416 0.05670397
## Xhousehold.income[5K-12K]        0.007379645 0.03344971
## Xhousehold.income[75K-100K]     -0.061785299 0.06128583
## Xhigh.educBachelor              -0.027717979 0.06890287
```

```
## Xhigh.educHS Diploma/GED       -0.024175106 0.04161622
## Xhigh.educPost Graduate Degree  0.008748282 0.07737202
## Xhigh.educSome College          0.024009078 0.06272739
## XPDS_score:caudate_rvsn_ant_z  -0.133227661 0.05715552
```

Male participants

```
##
## Family: gaussian
## Link function: identity
##
## Formula:
## cbcl_scr_syn_internal_r ~ PDS_score * caudate_rvsn_ant_z + race.ethnicity.5level +
##     demo_race_hispanic + interview_age + bmi + household.income +
##     high.educ
##
## Parametric coefficients:
##                               Estimate Std. Error t value Pr(>|t|)
## (Intercept)                    2.48555    2.56081   0.971  0.33187
## PDS_score                      0.72568    0.26626   2.725  0.00648 **
## caudate_rvsn_ant_z            -0.09282    0.38267  -0.243  0.80838
## race.ethnicity.5levelBlack    -0.05816    1.08640  -0.054  0.95731
## race.ethnicity.5levelMixed     1.34570    1.06052   1.269  0.20464
## race.ethnicity.5levelOther     0.23651    1.19759   0.197  0.84347
## race.ethnicity.5levelWhite     1.20280    0.99969   1.203  0.22907
## demo_race_hispanic1            0.42190    0.41215   1.024  0.30613
## interview_age                  0.01128    0.01707   0.661  0.50874
## bmi                            0.02058    0.03709   0.555  0.57903
## household.income[>=200K]      -3.18918    0.98250  -3.246  0.00119 **
## household.income[100K-200K]   -2.61262    0.92462  -2.826  0.00477 **
## household.income[12K-16K]     -0.77184    1.19761  -0.644  0.51934
## household.income[16K-25K]      0.16415    1.01899   0.161  0.87204
## household.income[25K-35K]     -0.46044    1.01018  -0.456  0.64859
## household.income[35K-50K]     -0.49419    0.96707  -0.511  0.60940
## household.income[50K-75K]     -2.00391    0.92521  -2.166  0.03045 *
## household.income[5K-12K]       0.43471    1.09766   0.396  0.69213
## household.income[75K-100K]    -2.72386    0.94042  -2.896  0.00382 **
## high.educBachelor              1.47288    0.94727   1.555  0.12015
## high.educHS Diploma/GED       -1.10369    0.97012  -1.138  0.25541
## high.educPost Graduate Degree  0.63371    0.94740   0.669  0.50365
## high.educSome College          0.87046    0.90511   0.962  0.33632
## PDS_score:caudate_rvsn_ant_z   0.18338    0.26974   0.680  0.49669
## ---
## Signif. codes:  0 '***' 0.001 '**' 0.01 '*' 0.05 '.' 0.1 ' ' 1
##
##
## R-sq.(adj) =  0.0376
## lmer.REML =  11428  Scale est. = 14.229    n = 1839
```

```
##                                     stdcoef      stdse
## X(Intercept)                    0.000000000 0.00000000
## XPDS_score                      0.066193606 0.02428756
## Xcaudate_rvsn_ant_z            -0.014992825 0.06181152
```

```
## Xrace.ethnicity.5levelBlack    -0.003170393 0.05921844
## Xrace.ethnicity.5levelMixed     0.077387769 0.06098759
## Xrace.ethnicity.5levelOther     0.008901371 0.04507310
## Xrace.ethnicity.5levelWhite     0.096946948 0.08057611
## Xdemo_race_hispanic1            0.029095825 0.02842299
## Xinterview_age                  0.015257237 0.02308444
## Xbmi                            0.013312345 0.02399022
## Xhousehold.income[>=200K]      -0.189449471 0.05836445
## Xhousehold.income[100K-200K]   -0.220849994 0.07815964
## Xhousehold.income[12K-16K]     -0.019572477 0.03036923
## Xhousehold.income[16K-25K]      0.005933171 0.03683043
## Xhousehold.income[25K-35K]     -0.017519520 0.03843717
## Xhousehold.income[35K-50K]     -0.022975644 0.04496025
## Xhousehold.income[50K-75K]     -0.123043257 0.05680924
## Xhousehold.income[5K-12K]       0.012724363 0.03212972
## Xhousehold.income[75K-100K]    -0.174953391 0.06040320
## Xhigh.educBachelor              0.116375237 0.07484562
## Xhigh.educHS Diploma/GED       -0.046656095 0.04100992
## Xhigh.educPost Graduate Degree  0.055358321 0.08276074
## Xhigh.educSome College          0.066707636 0.06936321
## XPDS_score:caudate_rvsn_ant_z   0.041986258 0.06175797
```

4.3 Model: CBCL internalizing factor ~ PDS x Putamen activity (anticipation stage)

Female participants

```
##
## Family: gaussian
## Link function: identity
##
## Formula:
## cbcl_scr_syn_internal_r ~ PDS_score * putamen_rvsn_ant_z + race.ethnicity.5level +
##     demo_race_hispanic + interview_age + bmi + household.income +
##     high.educ
##
## Parametric coefficients:
##                              Estimate Std. Error t value Pr(>|t|)
## (Intercept)                   0.18199    2.43846   0.075   0.9405
## PDS_score                     0.77946    0.19301   4.038  5.6e-05 ***
## putamen_rvsn_ant_z            0.51684    0.35098   1.473   0.1410
## race.ethnicity.5levelBlack   -0.51921    0.88894  -0.584   0.5592
## race.ethnicity.5levelMixed    1.06269    0.85367   1.245   0.2133
## race.ethnicity.5levelOther   -0.12951    1.02499  -0.126   0.8995
## race.ethnicity.5levelWhite    1.51865    0.79338   1.914   0.0558 .
## demo_race_hispanic1          -0.20874    0.38909  -0.536   0.5917
## interview_age                 0.02186    0.01743   1.254   0.2100
## bmi                           0.02891    0.03490   0.828   0.4075
## household.income[>=200K]     -1.97204    0.97578  -2.021   0.0434 *
## household.income[100K-200K]  -0.96416    0.91232  -1.057   0.2907
## household.income[12K-16K]     0.22577    1.13244   0.199   0.8420
## household.income[16K-25K]    -1.09675    1.03876  -1.056   0.2912
## household.income[25K-35K]     0.67425    0.95488   0.706   0.4802
```

```
## household.income[35K-50K]     -0.43173    0.93241  -0.463   0.6434
## household.income[50K-75K]     -0.79323    0.91494  -0.867   0.3861
## household.income[5K-12K]       0.49588    1.06790   0.464   0.6425
## household.income[75K-100K]    -0.69572    0.92325  -0.754   0.4512
## high.educBachelor             -0.26109    0.83684  -0.312   0.7551
## high.educHS Diploma/GED       -0.46064    0.85090  -0.541   0.5883
## high.educPost Graduate Degree  0.15474    0.85033   0.182   0.8556
## high.educSome College          0.38669    0.78792   0.491   0.6236
## PDS_score:putamen_rvsn_ant_z  -0.27967    0.19194  -1.457   0.1453
## ---
## Signif. codes:  0 '***' 0.001 '**' 0.01 '*' 0.05 '.' 0.1 ' ' 1
##
##
## R-sq.(adj) =  0.0276
## lmer.REML =  11353  Scale est. = 15.813    n = 1848
```

```
##                                     stdcoef      stdse
## X(Intercept)                    0.000000000 0.00000000
## XPDS_score                      0.105152168 0.02603766
## Xputamen_rvsn_ant_z             0.084077831 0.05709604
## Xrace.ethnicity.5levelBlack    -0.032747483 0.05606675
## Xrace.ethnicity.5levelMixed     0.065607816 0.05270353
## Xrace.ethnicity.5levelOther    -0.004872633 0.03856414
## Xrace.ethnicity.5levelWhite     0.132598789 0.06927303
## Xdemo_race_hispanic1           -0.015094407 0.02813535
## Xinterview_age                  0.030450451 0.02428342
## Xbmi                            0.020413623 0.02464068
## Xhousehold.income[>=200K]      -0.117838821 0.05830744
## Xhousehold.income[100K-200K]   -0.084704011 0.08014896
## Xhousehold.income[12K-16K]      0.006472136 0.03246424
## Xhousehold.income[16K-25K]     -0.038129703 0.03611347
## Xhousehold.income[25K-35K]      0.030293656 0.04290230
## Xhousehold.income[35K-50K]     -0.022323578 0.04821173
## Xhousehold.income[50K-75K]     -0.049856962 0.05750657
## Xhousehold.income[5K-12K]       0.015672282 0.03375099
## Xhousehold.income[75K-100K]    -0.046732573 0.06201619
## Xhigh.educBachelor             -0.021435037 0.06870257
## Xhigh.educHS Diploma/GED       -0.022445596 0.04146178
## Xhigh.educPost Graduate Degree  0.014035010 0.07712424
## Xhigh.educSome College          0.030679818 0.06251273
## XPDS_score:putamen_rvsn_ant_z  -0.083377790 0.05722210
```

Male participants

```
##
## Family: gaussian
## Link function: identity
##
## Formula:
## cbcl_scr_syn_internal_r ~ PDS_score * putamen_rvsn_ant_z + race.ethnicity.5level +
##     demo_race_hispanic + interview_age + bmi + household.income +
##     high.educ
##
```

```
## Parametric coefficients:
##                              Estimate Std. Error t value Pr(>|t|)
## (Intercept)                   2.71576    2.56820   1.057  0.29044
## PDS_score                     0.77404    0.26757   2.893  0.00386 **
## putamen_rvsn_ant_z            0.25804    0.39935   0.646  0.51826
## race.ethnicity.5levelBlack   -0.04199    1.08847  -0.039  0.96924
## race.ethnicity.5levelMixed    1.31702    1.06208   1.240  0.21512
## race.ethnicity.5levelOther    0.27435    1.19957   0.229  0.81912
## race.ethnicity.5levelWhite    1.16691    1.00147   1.165  0.24409
## demo_race_hispanic1           0.40045    0.41106   0.974  0.33009
## interview_age                 0.00832    0.01712   0.486  0.62702
## bmi                           0.01792    0.03719   0.482  0.63004
## household.income[>=200K]     -3.12119    0.98398  -3.172  0.00154 **
## household.income[100K-200K]  -2.59183    0.92584  -2.799  0.00517 **
## household.income[12K-16K]    -0.86593    1.19412  -0.725  0.46845
## household.income[16K-25K]     0.14300    1.01870   0.140  0.88838
## household.income[25K-35K]    -0.48889    1.01019  -0.484  0.62848
## household.income[35K-50K]    -0.46781    0.96956  -0.482  0.62952
## household.income[50K-75K]    -1.98913    0.92630  -2.147  0.03189 *
## household.income[5K-12K]      0.21180    1.09051   0.194  0.84603
## household.income[75K-100K]   -2.64598    0.94222  -2.808  0.00503 **
## high.educBachelor             1.59309    0.94154   1.692  0.09082 .
## high.educHS Diploma/GED      -1.04366    0.96527  -1.081  0.27975
## high.educPost Graduate Degree  0.74976    0.94224   0.796  0.42630
## high.educSome College         0.98208    0.89815   1.093  0.27434
## PDS_score:putamen_rvsn_ant_z -0.19638    0.27861  -0.705  0.48098
## ---
## Signif. codes:  0 '***' 0.001 '**' 0.01 '*' 0.05 '.' 0.1 ' ' 1
##
##
## R-sq.(adj) =  0.0356
## lmer.REML =  11435  Scale est. = 14.318    n = 1839
```

```
##                                      stdcoef      stdse
## X(Intercept)                     0.000000000 0.00000000
## XPDS_score                       0.070349821 0.02431875
## Xputamen_rvsn_ant_z              0.041192760 0.06375063
## Xrace.ethnicity.5levelBlack     -0.002287275 0.05929830
## Xrace.ethnicity.5levelMixed      0.075847633 0.06116563
## Xrace.ethnicity.5levelOther      0.010319796 0.04512266
## Xrace.ethnicity.5levelWhite      0.094055420 0.08072085
## Xdemo_race_hispanic1             0.027727711 0.02846230
## Xinterview_age                   0.011233894 0.02311466
## Xbmi                             0.011567033 0.02401003
## Xhousehold.income[>=200K]       -0.185308357 0.05841991
## Xhousehold.income[100K-200K]    -0.218802840 0.07815997
## Xhousehold.income[12K-16K]      -0.022227050 0.03065122
## Xhousehold.income[16K-25K]       0.005197051 0.03702160
## Xhousehold.income[25K-35K]      -0.018691926 0.03862319
## Xhousehold.income[35K-50K]      -0.021662539 0.04489732
## Xhousehold.income[50K-75K]      -0.121865818 0.05675036
## Xhousehold.income[5K-12K]        0.006312933 0.03250418
## Xhousehold.income[75K-100K]     -0.169610740 0.06039754
## Xhigh.educBachelor               0.125561341 0.07420882
```

```
## Xhigh.educHS Diploma/GED       -0.044281548 0.04095545
## Xhigh.educPost Graduate Degree  0.065400729 0.08219036
## Xhigh.educSome College          0.075500829 0.06904819
## XPDS_score:putamen_rvsn_ant_z  -0.044909045 0.06371277
```

4.4 Model: CBCL internalizing factor ~ PDS x Lateral OFC activity (anticipation stage)

Female participants

```
##
## Family: gaussian
## Link function: identity
##
## Formula:
## cbcl_scr_syn_internal_r ~ PDS_score * lOFC_rvsn_ant_z + race.ethnicity.5level +
##     demo_race_hispanic + interview_age + bmi + household.income +
##     high.educ
##
## Parametric coefficients:
##                                 Estimate Std. Error t value Pr(>|t|)
## (Intercept)                     0.509631   2.453955   0.208 0.835504
## PDS_score                       0.743493   0.195361   3.806 0.000146 ***
## lOFC_rvsn_ant_z                 0.371556   0.520662   0.714 0.475553
## race.ethnicity.5levelBlack     -0.509222   0.893439  -0.570 0.568777
## race.ethnicity.5levelMixed      1.085426   0.856802   1.267 0.205377
## race.ethnicity.5levelOther     -0.008328   1.028092  -0.008 0.993538
## race.ethnicity.5levelWhite      1.520267   0.795904   1.910 0.056276 .
## demo_race_hispanic1            -0.229686   0.391675  -0.586 0.557666
## interview_age                   0.022207   0.017531   1.267 0.205428
## bmi                             0.033037   0.034909   0.946 0.344094
## household.income[>=200K]      -2.349377   0.982168  -2.392 0.016857 *
## household.income[100K-200K]   -1.351875   0.918676  -1.472 0.141317
## household.income[12K-16K]     -0.067052   1.139912  -0.059 0.953100
## household.income[16K-25K]     -1.511751   1.041776  -1.451 0.146917
## household.income[25K-35K]      0.280808   0.961148   0.292 0.770199
## household.income[35K-50K]     -0.795571   0.938319  -0.848 0.396623
## household.income[50K-75K]     -1.151035   0.920871  -1.250 0.211482
## household.income[5K-12K]       0.107912   1.079860   0.100 0.920410
## household.income[75K-100K]    -1.073761   0.929025  -1.156 0.247918
## high.educBachelor              -0.285637   0.862130  -0.331 0.740444
## high.educHS Diploma/GED        -0.471556   0.874502  -0.539 0.589796
## high.educPost Graduate Degree   0.157297   0.875476   0.180 0.857431
## high.educSome College           0.349323   0.812297   0.430 0.667215
## PDS_score:lOFC_rvsn_ant_z      -0.164213   0.289256  -0.568 0.570304
## ---
## Signif. codes:  0 '***' 0.001 '**' 0.01 '*' 0.05 '.' 0.1 ' ' 1
##
##
## R-sq.(adj) =  0.0252
## lmer.REML =  11292  Scale est. = 15.515    n = 1837
```

```
##                                        stdcoef      stdse
```

```
## X(Intercept)                      0.0000000000 0.00000000
## XPDS_score                        0.0992864267 0.02608855
## XlOFC_rvsn_ant_z                  0.0386078794 0.05410134
## Xrace.ethnicity.5levelBlack      -0.0317648213 0.05573192
## Xrace.ethnicity.5levelMixed       0.0667265493 0.05267188
## Xrace.ethnicity.5levelOther      -0.0003138226 0.03874193
## Xrace.ethnicity.5levelWhite       0.1322072683 0.06921438
## Xdemo_race_hispanic1             -0.0165507281 0.02822332
## Xinterview_age                    0.0308949463 0.02439025
## Xbmi                              0.0233990477 0.02472547
## Xhousehold.income[>=200K]        -0.1405716100 0.05876658
## Xhousehold.income[100K-200K]     -0.1187419359 0.08069191
## Xhousehold.income[12K-16K]       -0.0019043810 0.03237532
## Xhousehold.income[16K-25K]       -0.0526417971 0.03627645
## Xhousehold.income[25K-35K]        0.0125326661 0.04289679
## Xhousehold.income[35K-50K]       -0.0409550973 0.04830357
## Xhousehold.income[50K-75K]       -0.0720564538 0.05764789
## Xhousehold.income[5K-12K]         0.0033244329 0.03326709
## Xhousehold.income[75K-100K]      -0.0723140891 0.06256662
## Xhigh.educBachelor               -0.0234651162 0.07082406
## Xhigh.educHS Diploma/GED         -0.0229340558 0.04253133
## Xhigh.educPost Graduate Degree    0.0142568755 0.07935018
## Xhigh.educSome College            0.0276502576 0.06429649
## XPDS_score:lOFC_rvsn_ant_z       -0.0307965833 0.05424722
```

Male participants

```
##
## Family: gaussian
## Link function: identity
##
## Formula:
## cbcl_scr_syn_internal_r ~ PDS_score * lOFC_rvsn_ant_z + race.ethnicity.5level +
##     demo_race_hispanic + interview_age + bmi + household.income +
##     high.educ
##
## Parametric coefficients:
##                             Estimate Std. Error t value Pr(>|t|)
## (Intercept)                  2.10197    2.57318   0.817   0.4141
## PDS_score                    0.66769    0.26742   2.497   0.0126 *
## lOFC_rvsn_ant_z             -0.20166    0.55535  -0.363   0.7166
## race.ethnicity.5levelBlack  -0.14132    1.09965  -0.129   0.8978
## race.ethnicity.5levelMixed   1.34572    1.06992   1.258   0.2086
## race.ethnicity.5levelOther   0.28138    1.20312   0.234   0.8151
## race.ethnicity.5levelWhite   1.20261    1.00894   1.192   0.2334
## demo_race_hispanic1          0.37432    0.41077   0.911   0.3623
## interview_age                0.01269    0.01701   0.746   0.4558
## bmi                          0.01546    0.03685   0.419   0.6749
## household.income[>=200K]    -2.70319    1.00470  -2.691   0.0072 **
## household.income[100K-200K] -2.20637    0.94944  -2.324   0.0202 *
## household.income[12K-16K]   -0.58643    1.21682  -0.482   0.6299
## household.income[16K-25K]    0.65737    1.04489   0.629   0.5293
## household.income[25K-35K]  -0.12516    1.02975  -0.122   0.9033
```

```
## household.income[35K-50K]     -0.07166    0.98848  -0.072   0.9422
## household.income[50K-75K]     -1.69955    0.95145  -1.786   0.0742 .
## household.income[5K-12K]       0.46359    1.11822   0.415   0.6785
## household.income[75K-100K]    -2.32741    0.96416  -2.414   0.0159 *
## high.educBachelor              1.46298    0.93815   1.559   0.1191
## high.educHS Diploma/GED       -0.91484    0.97359  -0.940   0.3475
## high.educPost Graduate Degree  0.60064    0.93775   0.641   0.5219
## high.educSome College          0.84112    0.89685   0.938   0.3484
## PDS_score:lOFC_rvsn_ant_z      0.04616    0.39036   0.118   0.9059
## ---
## Signif. codes:  0 '***' 0.001 '**' 0.01 '*' 0.05 '.' 0.1 ' ' 1
##
##
## R-sq.(adj) =  0.0345
## lmer.REML =  11318  Scale est. = 14.15     n = 1826
```

```
##                                      stdcoef      stdse
## X(Intercept)                    0.000000000 0.00000000
## XPDS_score                      0.060716176 0.02431744
## XlOFC_rvsn_ant_z               -0.022238347 0.06124179
## Xrace.ethnicity.5levelBlack    -0.007627576 0.05935342
## Xrace.ethnicity.5levelMixed     0.077644203 0.06173129
## Xrace.ethnicity.5levelOther     0.010773181 0.04606451
## Xrace.ethnicity.5levelWhite     0.097212629 0.08155745
## Xdemo_race_hispanic1            0.026219734 0.02877298
## Xinterview_age                  0.017308620 0.02320448
## Xbmi                            0.010086502 0.02404551
## Xhousehold.income[>=200K]      -0.162997084 0.06058133
## Xhousehold.income[100K-200K]   -0.187800311 0.08081359
## Xhousehold.income[12K-16K]     -0.015046547 0.03122130
## Xhousehold.income[16K-25K]      0.023746269 0.03774481
## Xhousehold.income[25K-35K]     -0.004844149 0.03985425
## Xhousehold.income[35K-50K]     -0.003393162 0.04680503
## Xhousehold.income[50K-75K]     -0.104833113 0.05868786
## Xhousehold.income[5K-12K]       0.013729750 0.03311778
## Xhousehold.income[75K-100K]    -0.151389930 0.06271514
## Xhigh.educBachelor              0.116289414 0.07457154
## Xhigh.educHS Diploma/GED       -0.038280631 0.04073908
## Xhigh.educPost Graduate Degree  0.052932683 0.08264072
## Xhigh.educSome College          0.065199940 0.06951984
## XPDS_score:lOFC_rvsn_ant_z      0.007193700 0.06082786
```

4.5 Model: CBCL internalizing factor ~ PDS x Medial OFC activity (anticipation stage)

Female participants

```
##
## Family: gaussian
## Link function: identity
##
## Formula:
## cbcl_scr_syn_internal_r ~ PDS_score * mOFC_rvsn_ant_z + race.ethnicity.5level +
```

```
##     demo_race_hispanic + interview_age + bmi + household.income +
##     high.educ
##
## Parametric coefficients:
##                             Estimate Std. Error t value Pr(>|t|)
## (Intercept)                  0.23125    2.44798   0.094 0.924750
## PDS_score                    0.74166    0.19569   3.790 0.000156 ***
## mOFC_rvsn_ant_z              0.68269    0.45307   1.507 0.132039
## race.ethnicity.5levelBlack  -0.49374    0.89222  -0.553 0.580068
## race.ethnicity.5levelMixed   1.06878    0.85750   1.246 0.212780
## race.ethnicity.5levelOther   0.01560    1.02588   0.015 0.987869
## race.ethnicity.5levelWhite   1.57030    0.79556   1.974 0.048552 *
## demo_race_hispanic1         -0.25419    0.39118  -0.650 0.515899
## interview_age                0.02340    0.01750   1.337 0.181485
## bmi                          0.03649    0.03489   1.046 0.295773
## household.income[>=200K]    -2.46751    0.98037  -2.517 0.011924 *
## household.income[100K-200K] -1.41492    0.91672  -1.543 0.122894
## household.income[12K-16K]   -0.22244    1.13312  -0.196 0.844391
## household.income[16K-25K]   -1.54100    1.04031  -1.481 0.138706
## household.income[25K-35K]    0.26413    0.95711   0.276 0.782602
## household.income[35K-50K]   -0.87687    0.93651  -0.936 0.349235
## household.income[50K-75K]   -1.21896    0.91802  -1.328 0.184408
## household.income[5K-12K]     0.04002    1.07524   0.037 0.970316
## household.income[75K-100K]  -1.14780    0.92676  -1.239 0.215686
## high.educBachelor           -0.15461    0.85496  -0.181 0.856513
## high.educHS Diploma/GED     -0.37719    0.86561  -0.436 0.663068
## high.educPost Graduate Degree  0.27185    0.86905   0.313 0.754462
## high.educSome College        0.45474    0.80486   0.565 0.572148
## PDS_score:mOFC_rvsn_ant_z   -0.23269    0.24885  -0.935 0.349888
## ---
## Signif. codes:  0 '***' 0.001 '**' 0.01 '*' 0.05 '.' 0.1 ' ' 1
##
##
## R-sq.(adj) =  0.028
## lmer.REML =  11289  Scale est. = 15.582    n = 1837
```

```
##                                   stdcoef      stdse
## X(Intercept)                  0.000000000 0.00000000
## XPDS_score                    0.099509736 0.02625529
## XmOFC_rvsn_ant_z              0.082381947 0.05467383
## Xrace.ethnicity.5levelBlack  -0.030963739 0.05595333
## Xrace.ethnicity.5levelMixed   0.065581331 0.05261681
## Xrace.ethnicity.5levelOther   0.000591398 0.03889171
## Xrace.ethnicity.5levelWhite   0.136736952 0.06927483
## Xdemo_race_hispanic1         -0.018337638 0.02822009
## Xinterview_age                0.032497620 0.02431162
## Xbmi                          0.025841144 0.02470844
## Xhousehold.income[>=200K]    -0.147641205 0.05865966
## Xhousehold.income[100K-200K] -0.124032850 0.08036032
## Xhousehold.income[12K-16K]   -0.006387366 0.03253742
## Xhousehold.income[16K-25K]   -0.053660688 0.03622589
## Xhousehold.income[25K-35K]    0.011885601 0.04306825
## Xhousehold.income[35K-50K]   -0.045140599 0.04821083
## Xhousehold.income[50K-75K]   -0.076444323 0.05757171
```

```
## Xhousehold.income[5K-12K]       0.001244252 0.03343280
## Xhousehold.income[75K-100K]    -0.077301204 0.06241443
## Xhigh.educBachelor             -0.012676501 0.07009793
## Xhigh.educHS Diploma/GED       -0.018468329 0.04238259
## Xhigh.educPost Graduate Degree  0.024620769 0.07870883
## Xhigh.educSome College          0.036079120 0.06385760
## XPDS_score:mOFC_rvsn_ant_z     -0.051230396 0.05478901
```

Male participants

```
##
## Family: gaussian
## Link function: identity
##
## Formula:
## cbcl_scr_syn_internal_r ~ PDS_score * mOFC_rvsn_ant_z + race.ethnicity.5level +
##     demo_race_hispanic + interview_age + bmi + household.income +
##     high.educ
##
## Parametric coefficients:
##                              Estimate Std. Error t value Pr(>|t|)
## (Intercept)                   2.54363    2.57390   0.988  0.32317
## PDS_score                     0.74359    0.26692   2.786  0.00540 **
## mOFC_rvsn_ant_z               0.61233    0.45000   1.361  0.17376
## race.ethnicity.5levelBlack   -0.09319    1.10302  -0.084  0.93268
## race.ethnicity.5levelMixed    1.23552    1.07249   1.152  0.24947
## race.ethnicity.5levelOther    0.28777    1.20757   0.238  0.81167
## race.ethnicity.5levelWhite    1.15398    1.01227   1.140  0.25444
## demo_race_hispanic1           0.36428    0.40994   0.889  0.37432
## interview_age                 0.01299    0.01705   0.762  0.44607
## bmi                           0.01669    0.03685   0.453  0.65060
## household.income[>=200K]     -3.25664    0.99570  -3.271  0.00109 **
## household.income[100K-200K]  -2.79416    0.93926  -2.975  0.00297 **
## household.income[12K-16K]    -1.23549    1.19344  -1.035  0.30070
## household.income[16K-25K]    -0.05577    1.03172  -0.054  0.95690
## household.income[25K-35K]    -0.82678    1.02134  -0.810  0.41833
## household.income[35K-50K]    -0.62499    0.97884  -0.638  0.52323
## household.income[50K-75K]    -2.26413    0.94042  -2.408  0.01616 *
## household.income[5K-12K]     -0.12935    1.11002  -0.117  0.90725
## household.income[75K-100K]   -2.86335    0.95452  -3.000  0.00274 **
## high.educBachelor             1.45568    0.93998   1.549  0.12165
## high.educHS Diploma/GED      -1.09945    0.97047  -1.133  0.25740
## high.educPost Graduate Degree  0.64010    0.93950   0.681  0.49576
## high.educSome College         0.89634    0.89649   1.000  0.31752
## PDS_score:mOFC_rvsn_ant_z    -0.62561    0.31555  -1.983  0.04757 *
## ---
## Signif. codes:  0 '***' 0.001 '**' 0.01 '*' 0.05 '.' 0.1 ' ' 1
##
##
## R-sq.(adj) =  0.0375
## lmer.REML =  11383  Scale est. = 14.183    n = 1834
```

```
##                                        stdcoef      stdse
```

```
## X(Intercept)                        0.000000000 0.00000000
## XPDS_score                          0.067482741 0.02422390
## XmOFC_rvsn_ant_z                    0.077096888 0.05665789
## Xrace.ethnicity.5levelBlack        -0.005032152 0.05955983
## Xrace.ethnicity.5levelMixed         0.071521144 0.06208380
## Xrace.ethnicity.5levelOther         0.010941783 0.04591504
## Xrace.ethnicity.5levelWhite         0.093140095 0.08170210
## Xdemo_race_hispanic1                0.025321377 0.02849497
## Xinterview_age                      0.017591386 0.02308134
## Xbmi                                0.010857514 0.02396748
## Xhousehold.income[>=200K]          -0.194699311 0.05952835
## Xhousehold.income[100K-200K]       -0.236886460 0.07962968
## Xhousehold.income[12K-16K]         -0.032278633 0.03117999
## Xhousehold.income[16K-25K]         -0.002025134 0.03746719
## Xhousehold.income[25K-35K]         -0.031607539 0.03904538
## Xhousehold.income[35K-50K]         -0.029291815 0.04587602
## Xhousehold.income[50K-75K]         -0.139192142 0.05781398
## Xhousehold.income[5K-12K]          -0.003804193 0.03264581
## Xhousehold.income[75K-100K]        -0.184479688 0.06149774
## Xhigh.educBachelor                  0.115286731 0.07444489
## Xhigh.educHS Diploma/GED           -0.046296195 0.04086477
## Xhigh.educPost Graduate Degree      0.056069579 0.08229600
## Xhigh.educSome College              0.069195269 0.06920672
## XPDS_score:mOFC_rvsn_ant_z         -0.113219309 0.05710713
```

4.6 Model: CBCL internalizing factor ~ PDS x Accumbens activity (feedback)

Female participants

```
##
## Family: gaussian
## Link function: identity
##
## Formula:
## cbcl_scr_syn_internal_r ~ PDS_score * accumbens_posvsneg_feedback_z +
##     race.ethnicity.5level + demo_race_hispanic + interview_age +
##     bmi + household.income + high.educ
##
## Parametric coefficients:
##                                        Estimate Std. Error t value Pr(>|t|)
## (Intercept)                             0.35307    2.43986   0.145   0.8850
## PDS_score                               0.76492    0.19403   3.942 8.37e-05
## accumbens_posvsneg_feedback_z           0.10595    0.44779   0.237   0.8130
## race.ethnicity.5levelBlack             -0.48032    0.89176  -0.539   0.5902
## race.ethnicity.5levelMixed              1.10492    0.85548   1.292   0.1967
## race.ethnicity.5levelOther             -0.05763    1.02403  -0.056   0.9551
## race.ethnicity.5levelWhite              1.51240    0.79409   1.905   0.0570
## demo_race_hispanic1                    -0.23063    0.39229  -0.588   0.5567
## interview_age                           0.02054    0.01746   1.176   0.2398
## bmi                                     0.03083    0.03480   0.886   0.3758
## household.income[>=200K]               -1.97151    0.97565  -2.021   0.0435
## household.income[100K-200K]            -0.93035    0.91144  -1.021   0.3075
## household.income[12K-16K]               0.23620    1.13195   0.209   0.8347
```

```
## household.income[16K-25K]              -1.17570    1.03619  -1.135   0.2567
## household.income[25K-35K]               0.67948    0.95356   0.713   0.4762
## household.income[35K-50K]              -0.44467    0.93169  -0.477   0.6332
## household.income[50K-75K]              -0.77898    0.91599  -0.850   0.3952
## household.income[5K-12K]                0.38165    1.07169   0.356   0.7218
## household.income[75K-100K]             -0.67134    0.92331  -0.727   0.4673
## high.educBachelor                      -0.30408    0.84731  -0.359   0.7197
## high.educHS Diploma/GED                -0.47572    0.85865  -0.554   0.5796
## high.educPost Graduate Degree           0.10898    0.86114   0.127   0.8993
## high.educSome College                   0.34659    0.79736   0.435   0.6639
## PDS_score:accumbens_posvsneg_feedback_z  0.05945    0.24407   0.244   0.8076
##
## (Intercept)
## PDS_score                              ***
## accumbens_posvsneg_feedback_z
## race.ethnicity.5levelBlack
## race.ethnicity.5levelMixed
## race.ethnicity.5levelOther
## race.ethnicity.5levelWhite             .
## demo_race_hispanic1
## interview_age
## bmi
## household.income[>=200K]               *
## household.income[100K-200K]
## household.income[12K-16K]
## household.income[16K-25K]
## household.income[25K-35K]
## household.income[35K-50K]
## household.income[50K-75K]
## household.income[5K-12K]
## household.income[75K-100K]
## high.educBachelor
## high.educHS Diploma/GED
## high.educPost Graduate Degree
## high.educSome College
## PDS_score:accumbens_posvsneg_feedback_z
## ---
## Signif. codes:  0 '***' 0.001 '**' 0.01 '*' 0.05 '.' 0.1 ' ' 1
##
##
## R-sq.(adj) =  0.0257
## lmer.REML =  11341  Scale est. = 15.659    n = 1846
```

```
##                                             stdcoef      stdse
## X(Intercept)                            0.000000000 0.00000000
## XPDS_score                              0.102813360 0.02607938
## Xaccumbens_posvsneg_feedback_z          0.013476231 0.05695898
## Xrace.ethnicity.5levelBlack            -0.030215838 0.05609839
## Xrace.ethnicity.5levelMixed             0.068022763 0.05266632
## Xrace.ethnicity.5levelOther            -0.002183297 0.03879566
## Xrace.ethnicity.5levelWhite             0.131974676 0.06929349
## Xdemo_race_hispanic1                   -0.016652635 0.02832608
## Xinterview_age                          0.028583582 0.02430622
## Xbmi                                    0.021839563 0.02465115
```

```
## Xhousehold.income[>=200K]               -0.117910084 0.05835076
## Xhousehold.income[100K-200K]            -0.081788592 0.08012622
## Xhousehold.income[12K-16K]               0.006777705 0.03248041
## Xhousehold.income[16K-25K]              -0.041204834 0.03631547
## Xhousehold.income[25K-35K]               0.030680126 0.04305584
## Xhousehold.income[35K-50K]              -0.023013018 0.04821807
## Xhousehold.income[50K-75K]              -0.048745550 0.05731900
## Xhousehold.income[5K-12K]                0.011966382 0.03360199
## Xhousehold.income[75K-100K]             -0.045133486 0.06207293
## Xhigh.educBachelor                      -0.025015949 0.06970521
## Xhigh.educHS Diploma/GED                -0.023201141 0.04187711
## Xhigh.educPost Graduate Degree           0.009893020 0.07817175
## Xhigh.educSome College                   0.027433570 0.06311406
## XPDS_score:accumbens_posvsneg_feedback_z  0.013917799 0.05714278
```

Male participants

```
##
## Family: gaussian
## Link function: identity
##
## Formula:
## cbcl_scr_syn_internal_r ~ PDS_score * accumbens_posvsneg_feedback_z +
##     race.ethnicity.5level + demo_race_hispanic + interview_age +
##     bmi + household.income + high.educ
##
## Parametric coefficients:
##                                          Estimate Std. Error t value Pr(>|t|)
## (Intercept)                               3.018972   2.585380   1.168 0.243077
## PDS_score                                 0.728786   0.265367   2.746 0.006086
## accumbens_posvsneg_feedback_z             0.509864   0.504454   1.011 0.312283
## race.ethnicity.5levelBlack               -0.211440   1.099040  -0.192 0.847461
## race.ethnicity.5levelMixed                1.322489   1.073039   1.232 0.217933
## race.ethnicity.5levelOther                0.253071   1.207146   0.210 0.833969
## race.ethnicity.5levelWhite                1.244603   1.014020   1.227 0.219833
## demo_race_hispanic1                       0.382408   0.407972   0.937 0.348709
## interview_age                             0.006769   0.017093   0.396 0.692123
## bmi                                       0.038407   0.037149   1.034 0.301342
## household.income[>=200K]                 -3.504046   1.000787  -3.501 0.000474
## household.income[100K-200K]              -3.073459   0.943729  -3.257 0.001148
## household.income[12K-16K]                -1.262883   1.196647  -1.055 0.291405
## household.income[16K-25K]                -0.332785   1.033838  -0.322 0.747571
## household.income[25K-35K]                -0.816356   1.029624  -0.793 0.427958
## household.income[35K-50K]                -0.977107   0.984886  -0.992 0.321280
## household.income[50K-75K]                -2.438422   0.945278  -2.580 0.009970
## household.income[5K-12K]                 -0.090017   1.119976  -0.080 0.935948
## household.income[75K-100K]               -3.128107   0.960304  -3.257 0.001145
## high.educBachelor                         1.569271   0.946051   1.659 0.097338
## high.educHS Diploma/GED                  -1.096906   0.971477  -1.129 0.259000
## high.educPost Graduate Degree             0.731940   0.946214   0.774 0.439300
## high.educSome College                     0.981834   0.903110   1.087 0.277106
## PDS_score:accumbens_posvsneg_feedback_z -0.606386   0.365947  -1.657 0.097685
##
```

```
## (Intercept)
## PDS_score                              **
## accumbens_posvsneg_feedback_z
## race.ethnicity.5levelBlack
## race.ethnicity.5levelMixed
## race.ethnicity.5levelOther
## race.ethnicity.5levelWhite
## demo_race_hispanic1
## interview_age
## bmi
## household.income[>=200K]               ***
## household.income[100K-200K]            **
## household.income[12K-16K]
## household.income[16K-25K]
## household.income[25K-35K]
## household.income[35K-50K]
## household.income[50K-75K]              **
## household.income[5K-12K]
## household.income[75K-100K]             **
## high.educBachelor                      .
## high.educHS Diploma/GED
## high.educPost Graduate Degree
## high.educSome College
## PDS_score:accumbens_posvsneg_feedback_z .
## ---
## Signif. codes:  0 '***' 0.001 '**' 0.01 '*' 0.05 '.' 0.1 ' ' 1
##
##
## R-sq.(adj) =  0.0401
## lmer.REML =  11414  Scale est. = 15.119    n = 1837

##                                             stdcoef      stdse
## X(Intercept)                            0.000000000 0.00000000
## XPDS_score                              0.066670253 0.02427612
## Xaccumbens_posvsneg_feedback_z          0.066928061 0.06621789
## Xrace.ethnicity.5levelBlack            -0.011471327 0.05962652
## Xrace.ethnicity.5levelMixed             0.076198452 0.06182576
## Xrace.ethnicity.5levelOther             0.009576849 0.04568155
## Xrace.ethnicity.5levelWhite             0.100236842 0.08166632
## Xdemo_race_hispanic1                    0.026579006 0.02835582
## Xinterview_age                          0.009138590 0.02307500
## Xbmi                                    0.024694044 0.02388541
## Xhousehold.income[>=200K]              -0.208135859 0.05944547
## Xhousehold.income[100K-200K]           -0.259730438 0.07975218
## Xhousehold.income[12K-16K]             -0.032837473 0.03111519
## Xhousehold.income[16K-25K]             -0.012100309 0.03759118
## Xhousehold.income[25K-35K]             -0.031061214 0.03917574
## Xhousehold.income[35K-50K]             -0.045886053 0.04625137
## Xhousehold.income[50K-75K]             -0.148960037 0.05774579
## Xhousehold.income[5K-12K]              -0.002609612 0.03246821
## Xhousehold.income[75K-100K]            -0.200605108 0.06158418
## Xhigh.educBachelor                      0.123404683 0.07439583
## Xhigh.educHS Diploma/GED               -0.046564372 0.04123984
## Xhigh.educPost Graduate Degree          0.063915807 0.08262696
```

```
## Xhigh.educSome College                    0.075453503 0.06940363
## XPDS_score:accumbens_posvsneg_feedback_z -0.109842974 0.06628886
```

4.7 Model: CBCL internalizing factor ~ PDS x Caudate activity (feedback)

Female participants

```
##
## Family: gaussian
## Link function: identity
##
## Formula:
## cbcl_scr_syn_internal_r ~ PDS_score * caudate_posvsneg_feedback_z +
##     race.ethnicity.5level + demo_race_hispanic + interview_age +
##     bmi + household.income + high.educ
##
## Parametric coefficients:
##                                       Estimate Std. Error t value Pr(>|t|)
## (Intercept)                            0.51190    2.42883   0.211   0.8331
## PDS_score                              0.76428    0.19286   3.963 7.69e-05 ***
## caudate_posvsneg_feedback_z           -0.71105    0.35018  -2.031   0.0424 *
## race.ethnicity.5levelBlack            -0.60318    0.89066  -0.677   0.4983
## race.ethnicity.5levelMixed             1.00450    0.85426   1.176   0.2398
## race.ethnicity.5levelOther            -0.17787    1.02347  -0.174   0.8620
## race.ethnicity.5levelWhite             1.49748    0.79346   1.887   0.0593 .
## demo_race_hispanic1                   -0.17104    0.38968  -0.439   0.6608
## interview_age                          0.02055    0.01739   1.181   0.2377
## bmi                                    0.03194    0.03473   0.920   0.3579
## household.income[>=200K]              -2.17768    0.96850  -2.249   0.0247 *
## household.income[100K-200K]           -1.20550    0.90382  -1.334   0.1824
## household.income[12K-16K]             -0.06817    1.12429  -0.061   0.9517
## household.income[16K-25K]             -1.38480    1.03248  -1.341   0.1800
## household.income[25K-35K]              0.40339    0.94577   0.427   0.6698
## household.income[35K-50K]             -0.71028    0.92465  -0.768   0.4425
## household.income[50K-75K]            -1.04628    0.90657  -1.154   0.2486
## household.income[5K-12K]               0.22101    1.05915   0.209   0.8347
## household.income[75K-100K]            -0.93949    0.91389  -1.028   0.3041
## high.educBachelor                     -0.16956    0.84517  -0.201   0.8410
## high.educHS Diploma/GED               -0.31013    0.85714  -0.362   0.7175
## high.educPost Graduate Degree          0.17402    0.85809   0.203   0.8393
## high.educSome College                  0.47665    0.79539   0.599   0.5491
## PDS_score:caudate_posvsneg_feedback_z  0.41089    0.19275   2.132   0.0332 *
## ---
## Signif. codes:  0 '***' 0.001 '**' 0.01 '*' 0.05 '.' 0.1 ' ' 1
##
##
## R-sq.(adj) =  0.0281
## lmer.REML =  11350  Scale est. = 15.053    n = 1848
```

```
##                                            stdcoef      stdse
## X(Intercept)                           0.000000000 0.00000000
## XPDS_score                             0.103068871 0.02600894
## Xcaudate_posvsneg_feedback_z          -0.114857614 0.05656456
```

```
## Xrace.ethnicity.5levelBlack            -0.037988010 0.05609318
## Xrace.ethnicity.5levelMixed             0.062031929 0.05275409
## Xrace.ethnicity.5levelOther            -0.006734470 0.03874923
## Xrace.ethnicity.5levelWhite             0.130786014 0.06929906
## Xdemo_race_hispanic1                   -0.012385072 0.02821749
## Xinterview_age                          0.028594080 0.02420649
## Xbmi                                    0.022645215 0.02462371
## Xhousehold.income[>=200K]              -0.129900278 0.05777143
## Xhousehold.income[100K-200K]           -0.105892839 0.07939283
## Xhousehold.income[12K-16K]             -0.001954850 0.03223936
## Xhousehold.income[16K-25K]             -0.048156973 0.03590489
## Xhousehold.income[25K-35K]              0.018202689 0.04267662
## Xhousehold.income[35K-50K]             -0.036736306 0.04782385
## Xhousehold.income[50K-75K]             -0.065549327 0.05679611
## Xhousehold.income[5K-12K]               0.006986897 0.03348368
## Xhousehold.income[75K-100K]            -0.063305138 0.06158001
## Xhigh.educBachelor                     -0.013906384 0.06931526
## Xhigh.educHS Diploma/GED               -0.015166467 0.04191708
## Xhigh.educPost Graduate Degree          0.015795715 0.07788825
## Xhigh.educSome College                  0.037827133 0.06312301
## XPDS_score:caudate_posvsneg_feedback_z  0.120673469 0.05660809
```

Male participants

```
##
## Family: gaussian
## Link function: identity
##
## Formula:
## cbcl_scr_syn_internal_r ~ PDS_score * caudate_posvsneg_feedback_z +
##     race.ethnicity.5level + demo_race_hispanic + interview_age +
##     bmi + household.income + high.educ
##
## Parametric coefficients:
##                                        Estimate Std. Error t value Pr(>|t|)
## (Intercept)                            3.139563   2.572936   1.220 0.222538
## PDS_score                              0.738174   0.265498   2.780 0.005486 **
## caudate_posvsneg_feedback_z            0.252663   0.410542   0.615 0.538343
## race.ethnicity.5levelBlack            -0.194140   1.092567  -0.178 0.858985
## race.ethnicity.5levelMixed             1.239086   1.064811   1.164 0.244712
## race.ethnicity.5levelOther             0.209688   1.201120   0.175 0.861431
## race.ethnicity.5levelWhite             1.105359   1.004990   1.100 0.271534
## demo_race_hispanic1                    0.377290   0.408111   0.924 0.355360
## interview_age                          0.007253   0.017107   0.424 0.671623
## bmi                                    0.021823   0.037046   0.589 0.555873
## household.income[>=200K]              -3.282110   0.987548  -3.323 0.000907 ***
## household.income[100K-200K]           -2.812670   0.931135  -3.021 0.002557 **
## household.income[12K-16K]             -1.127801   1.186812  -0.950 0.342098
## household.income[16K-25K]             -0.135907   1.028471  -0.132 0.894884
## household.income[25K-35K]             -0.556258   1.016070  -0.547 0.584130
## household.income[35K-50K]             -0.697369   0.972226  -0.717 0.473287
## household.income[50K-75K]             -2.190933   0.933407  -2.347 0.019020 *
## household.income[5K-12K]              -0.172457   1.104247  -0.156 0.875912
```

```
## household.income[75K-100K]            -2.876674   0.946853  -3.038 0.002414 **
## high.educBachelor                       1.569932   0.948042   1.656 0.097900 .
## high.educHS Diploma/GED                -1.057967   0.976312  -1.084 0.278670
## high.educPost Graduate Degree           0.699966   0.948189   0.738 0.460480
## high.educSome College                   0.932271   0.904269   1.031 0.302694
## PDS_score:caudate_posvsneg_feedback_z -0.313226   0.295090  -1.061 0.288622
## ---
## Signif. codes:  0 '***' 0.001 '**' 0.01 '*' 0.05 '.' 0.1 ' ' 1
##
##
## R-sq.(adj) =  0.0357
## lmer.REML =  11419  Scale est. = 14.952    n = 1837
```

```
##                                            stdcoef      stdse
## X(Intercept)                           0.000000000 0.00000000
## XPDS_score                             0.067638450 0.02432743
## Xcaudate_posvsneg_feedback_z           0.040339718 0.06554639
## Xrace.ethnicity.5levelBlack           -0.010525180 0.05923287
## Xrace.ethnicity.5levelMixed            0.071650281 0.06157281
## Xrace.ethnicity.5levelOther            0.007904438 0.04527764
## Xrace.ethnicity.5levelWhite            0.089115887 0.08102395
## Xdemo_race_hispanic1                   0.026295087 0.02844311
## Xinterview_age                         0.009815453 0.02315014
## Xbmi                                   0.014155034 0.02402856
## Xhousehold.income[>=200K]             -0.195979223 0.05896785
## Xhousehold.income[100K-200K]          -0.237893824 0.07875477
## Xhousehold.income[12K-16K]            -0.029372562 0.03090944
## Xhousehold.income[16K-25K]            -0.004920059 0.03723232
## Xhousehold.income[25K-35K]            -0.021199100 0.03872265
## Xhousehold.income[35K-50K]            -0.032582892 0.04542491
## Xhousehold.income[50K-75K]            -0.134283519 0.05720902
## Xhousehold.income[5K-12K]             -0.005056042 0.03237408
## Xhousehold.income[75K-100K]           -0.184509710 0.06073108
## Xhigh.educBachelor                     0.123737657 0.07472202
## Xhigh.educHS Diploma/GED              -0.044793998 0.04133675
## Xhigh.educPost Graduate Degree         0.061195134 0.08289621
## Xhigh.educSome College                 0.071866528 0.06970794
## XPDS_score:caudate_posvsneg_feedback_z -0.069804148 0.06576236
```

4.8 Model: CBCL internalizing factor ~ PDS x Putamen activity (feedback)

Female participants

```
##
## Family: gaussian
## Link function: identity
##
## Formula:
## cbcl_scr_syn_internal_r ~ PDS_score * putamen_posvsneg_feedback_z +
##     race.ethnicity.5level + demo_race_hispanic + interview_age +
##     bmi + household.income + high.educ
##
## Parametric coefficients:
```

```
##                                      Estimate Std. Error t value Pr(>|t|)
## (Intercept)                           0.57505    2.43611   0.236   0.8134
## PDS_score                             0.80088    0.19338   4.141 3.61e-05 ***
## putamen_posvsneg_feedback_z          -0.61980    0.36241  -1.710   0.0874 .
## race.ethnicity.5levelBlack           -0.59957    0.88903  -0.674   0.5001
## race.ethnicity.5levelMixed            1.06549    0.85352   1.248   0.2121
## race.ethnicity.5levelOther           -0.20223    1.02412  -0.197   0.8435
## race.ethnicity.5levelWhite            1.53343    0.79261   1.935   0.0532 .
## demo_race_hispanic1                  -0.21173    0.39216  -0.540   0.5893
## interview_age                         0.01948    0.01738   1.121   0.2625
## bmi                                   0.02802    0.03484   0.804   0.4214
## household.income[>=200K]             -2.01635    0.97301  -2.072   0.0384 *
## household.income[100K-200K]          -1.02319    0.90912  -1.125   0.2605
## household.income[12K-16K]             0.14503    1.13069   0.128   0.8980
## household.income[16K-25K]            -1.20627    1.03453  -1.166   0.2438
## household.income[25K-35K]             0.62129    0.95232   0.652   0.5142
## household.income[35K-50K]            -0.48021    0.93076  -0.516   0.6060
## household.income[50K-75K]            -0.83024    0.91322  -0.909   0.3634
## household.income[5K-12K]              0.54493    1.07020   0.509   0.6107
## household.income[75K-100K]           -0.81486    0.92045  -0.885   0.3761
## high.educBachelor                    -0.33667    0.84974  -0.396   0.6920
## high.educHS Diploma/GED              -0.53510    0.86179  -0.621   0.5347
## high.educPost Graduate Degree         0.05744    0.86134   0.067   0.9468
## high.educSome College                 0.33091    0.80000   0.414   0.6792
## PDS_score:putamen_posvsneg_feedback_z  0.31332    0.19814   1.581   0.1140
## ---
## Signif. codes:  0 '***' 0.001 '**' 0.01 '*' 0.05 '.' 0.1 ' ' 1
##
##
## R-sq.(adj) =  0.0286
## lmer.REML =  11330  Scale est. = 15.573    n = 1845
```

```
##                                          stdcoef      stdse
## X(Intercept)                         0.000000000 0.00000000
## XPDS_score                           0.107727068 0.02601181
## Xputamen_posvsneg_feedback_z        -0.098799050 0.05776922
## Xrace.ethnicity.5levelBlack         -0.037732325 0.05594899
## Xrace.ethnicity.5levelMixed          0.065621716 0.05256684
## Xrace.ethnicity.5levelOther         -0.007618943 0.03858338
## Xrace.ethnicity.5levelWhite          0.133794359 0.06915677
## Xdemo_race_hispanic1                -0.015259627 0.02826281
## Xinterview_age                       0.027164930 0.02423814
## Xbmi                                 0.019811728 0.02463622
## Xhousehold.income[>=200K]           -0.120640564 0.05821626
## Xhousehold.income[100K-200K]        -0.090012896 0.07997804
## Xhousehold.income[12K-16K]           0.004163389 0.03245828
## Xhousehold.income[16K-25K]          -0.042294234 0.03627298
## Xhousehold.income[25K-35K]           0.028064929 0.04301777
## Xhousehold.income[35K-50K]          -0.024790384 0.04804919
## Xhousehold.income[50K-75K]          -0.051789221 0.05696535
## Xhousehold.income[5K-12K]            0.017093151 0.03356980
## Xhousehold.income[75K-100K]         -0.054882048 0.06199378
## Xhigh.educBachelor                  -0.027615895 0.06970091
## Xhigh.educHS Diploma/GED            -0.026195673 0.04218874
```

```
## Xhigh.educPost Graduate Degree          0.005219198 0.07826839
## Xhigh.educSome College                  0.026222175 0.06339461
## XPDS_score:putamen_posvsneg_feedback_z  0.091244617 0.05770297
```

Male participants

```
##
## Family: gaussian
## Link function: identity
##
## Formula:
## cbcl_scr_syn_internal_r ~ PDS_score * putamen_posvsneg_feedback_z +
##     race.ethnicity.5level + demo_race_hispanic + interview_age +
##     bmi + household.income + high.educ
##
## Parametric coefficients:
##                                        Estimate Std. Error t value Pr(>|t|)
## (Intercept)                            3.219320   2.587687   1.244 0.213626
## PDS_score                              0.715741   0.266515   2.686 0.007307 **
## putamen_posvsneg_feedback_z            0.114113   0.401754   0.284 0.776414
## race.ethnicity.5levelBlack            -0.167483   1.094021  -0.153 0.878345
## race.ethnicity.5levelMixed             1.243634   1.066748   1.166 0.243841
## race.ethnicity.5levelOther             0.188140   1.203094   0.156 0.875751
## race.ethnicity.5levelWhite             1.119430   1.006209   1.113 0.266060
## demo_race_hispanic1                    0.337952   0.407576   0.829 0.407115
## interview_age                          0.006897   0.017112   0.403 0.686978
## bmi                                    0.027413   0.036981   0.741 0.458614
## household.income[>=200K]              -3.313477   0.993398  -3.335 0.000869 ***
## household.income[100K-200K]           -2.860442   0.936191  -3.055 0.002280 **
## household.income[12K-16K]             -1.158791   1.191781  -0.972 0.331021
## household.income[16K-25K]             -0.168135   1.037583  -0.162 0.871289
## household.income[25K-35K]             -0.610510   1.022563  -0.597 0.550555
## household.income[35K-50K]             -0.741169   0.977863  -0.758 0.448580
## household.income[50K-75K]             -2.179990   0.938724  -2.322 0.020327 *
## household.income[5K-12K]              -0.252260   1.111732  -0.227 0.820521
## household.income[75K-100K]            -2.901924   0.952440  -3.047 0.002346 **
## high.educBachelor                      1.460036   0.953666   1.531 0.125950
## high.educHS Diploma/GED               -1.132091   0.981399  -1.154 0.248837
## high.educPost Graduate Degree          0.631452   0.953740   0.662 0.508004
## high.educSome College                  0.869068   0.910466   0.955 0.339942
## PDS_score:putamen_posvsneg_feedback_z -0.118882   0.284791  -0.417 0.676410
## ---
## Signif. codes:  0 '***' 0.001 '**' 0.01 '*' 0.05 '.' 0.1 ' ' 1
##
##
## R-sq.(adj) =  0.0348
## lmer.REML =  11456  Scale est. = 15.398    n = 1842
```

```
##                                            stdcoef      stdse
## X(Intercept)                            0.000000000 0.00000000
## XPDS_score                              0.065524928 0.02439897
## Xputamen_posvsneg_feedback_z            0.018111216 0.06376336
## Xrace.ethnicity.5levelBlack            -0.009062302 0.05919609
```

```
## Xrace.ethnicity.5levelMixed            0.071775090 0.06156629
## Xrace.ethnicity.5levelOther            0.007077695 0.04525963
## Xrace.ethnicity.5levelWhite            0.090109243 0.08099541
## Xdemo_race_hispanic1                   0.023484946 0.02832329
## Xinterview_age                         0.009327087 0.02314279
## Xbmi                                   0.017766438 0.02396693
## Xhousehold.income[>=200K]             -0.197117740 0.05909694
## Xhousehold.income[100K-200K]          -0.241966114 0.07919287
## Xhousehold.income[12K-16K]            -0.030117037 0.03097445
## Xhousehold.income[16K-25K]            -0.006037459 0.03725798
## Xhousehold.income[25K-35K]            -0.023219297 0.03889072
## Xhousehold.income[35K-50K]            -0.034792980 0.04590417
## Xhousehold.income[50K-75K]            -0.133360239 0.05742616
## Xhousehold.income[5K-12K]             -0.007380435 0.03252616
## Xhousehold.income[75K-100K]           -0.186325678 0.06115391
## Xhigh.educBachelor                     0.115189515 0.07523943
## Xhigh.educHS Diploma/GED              -0.047835610 0.04146824
## Xhigh.educPost Graduate Degree         0.055163881 0.08331914
## Xhigh.educSome College                 0.066883311 0.07006929
## XPDS_score:putamen_posvsneg_feedback_z -0.026672989 0.06389751
```

4.9 Model: CBCL internalizing factor ~ PDS x Lateral OFC activity (feedback stage)

Female participants

```
##
## Family: gaussian
## Link function: identity
##
## Formula:
## cbcl_scr_syn_internal_r ~ PDS_score * lOFC_posvsneg_feedback_z +
##     race.ethnicity.5level + demo_race_hispanic + interview_age +
##     bmi + household.income + high.educ
##
## Parametric coefficients:
##                              Estimate Std. Error t value Pr(>|t|)
## (Intercept)                   0.30976    2.43644   0.127 0.898847
## PDS_score                     0.73791    0.19490   3.786 0.000158 ***
## lOFC_posvsneg_feedback_z     -0.47278    0.58504  -0.808 0.419125
## race.ethnicity.5levelBlack   -0.46824    0.89181  -0.525 0.599617
## race.ethnicity.5levelMixed    1.09005    0.85458   1.276 0.202283
## race.ethnicity.5levelOther   -0.18063    1.02502  -0.176 0.860140
## race.ethnicity.5levelWhite    1.50079    0.79359   1.891 0.058766 .
## demo_race_hispanic1          -0.22674    0.39222  -0.578 0.563272
## interview_age                 0.02290    0.01748   1.310 0.190270
## bmi                           0.02970    0.03483   0.853 0.393892
## household.income[>=200K]     -2.21091    0.96851  -2.283 0.022557 *
## household.income[100K-200K]  -1.20356    0.90400  -1.331 0.183231
## household.income[12K-16K]    -0.03615    1.12546  -0.032 0.974382
## household.income[16K-25K]    -1.37547    1.02896  -1.337 0.181471
## household.income[25K-35K]     0.51538    0.94793   0.544 0.586720
## household.income[35K-50K]    -0.60480    0.92575  -0.653 0.513641
```

```
## household.income[50K-75K]          -0.98708    0.90800  -1.087 0.277140
## household.income[5K-12K]            0.21756    1.06901   0.204 0.838755
## household.income[75K-100K]         -0.93897    0.91516  -1.026 0.305022
## high.educBachelor                  -0.22976    0.83796  -0.274 0.783968
## high.educHS Diploma/GED            -0.33738    0.85104  -0.396 0.691831
## high.educPost Graduate Degree       0.19193    0.85135   0.225 0.821665
## high.educSome College               0.38717    0.78861   0.491 0.623516
## PDS_score:lOFC_posvsneg_feedback_z  0.32573    0.31273   1.042 0.297747
## ---
## Signif. codes:  0 '***' 0.001 '**' 0.01 '*' 0.05 '.' 0.1 ' ' 1
##
##
## R-sq.(adj) =  0.0253
## lmer.REML =  11333  Scale est. = 15.886    n = 1845
```

```
##                                          stdcoef      stdse
## X(Intercept)                         0.000000000 0.00000000
## XPDS_score                           0.099092405 0.02617261
## XlOFC_posvsneg_feedback_z           -0.044539210 0.05511440
## Xrace.ethnicity.5levelBlack         -0.029432928 0.05605807
## Xrace.ethnicity.5levelMixed          0.067297449 0.05276021
## Xrace.ethnicity.5levelOther         -0.006809098 0.03863965
## Xrace.ethnicity.5levelWhite          0.131022486 0.06928267
## Xdemo_race_hispanic1                -0.016350398 0.02828328
## Xinterview_age                       0.031916936 0.02435909
## Xbmi                                 0.021094361 0.02473593
## Xhousehold.income[>=200K]           -0.132623810 0.05809721
## Xhousehold.income[100K-200K]        -0.105942582 0.07957357
## Xhousehold.income[12K-16K]          -0.001038266 0.03232710
## Xhousehold.income[16K-25K]          -0.048254901 0.03609869
## Xhousehold.income[25K-35K]           0.023105860 0.04249806
## Xhousehold.income[35K-50K]          -0.031056443 0.04753734
## Xhousehold.income[50K-75K]          -0.061828164 0.05687485
## Xhousehold.income[5K-12K]            0.006828368 0.03355201
## Xhousehold.income[75K-100K]         -0.063277426 0.06167321
## Xhigh.educBachelor                  -0.018882370 0.06886533
## Xhigh.educHS Diploma/GED            -0.016470914 0.04154764
## Xhigh.educPost Graduate Degree       0.017441471 0.07736771
## Xhigh.educSome College               0.030674759 0.06247953
## XPDS_score:lOFC_posvsneg_feedback_z  0.057541973 0.05524515
```

Male participants

```
##
## Family: gaussian
## Link function: identity
##
## Formula:
## cbcl_scr_syn_internal_r ~ PDS_score * lOFC_posvsneg_feedback_z +
##     race.ethnicity.5level + demo_race_hispanic + interview_age +
##     bmi + household.income + high.educ
##
## Parametric coefficients:
```

```
##                                    Estimate Std. Error t value Pr(>|t|)
## (Intercept)                         2.765864   2.575341   1.074  0.28298
## PDS_score                           0.680451   0.269303   2.527  0.01160 *
## lOFC_posvsneg_feedback_z           -0.114014   0.597849  -0.191  0.84878
## race.ethnicity.5levelBlack         -0.123147   1.097982  -0.112  0.91071
## race.ethnicity.5levelMixed          1.281752   1.072352   1.195  0.23214
## race.ethnicity.5levelOther          0.175437   1.205705   0.146  0.88433
## race.ethnicity.5levelWhite          1.183077   1.011434   1.170  0.24228
## demo_race_hispanic1                 0.405367   0.411223   0.986  0.32438
## interview_age                       0.007728   0.017112   0.452  0.65158
## bmi                                 0.026413   0.037148   0.711  0.47716
## household.income[>=200K]           -3.100859   1.027892  -3.017  0.00259 **
## household.income[100K-200K]        -2.631511   0.973503  -2.703  0.00693 **
## household.income[12K-16K]          -1.078124   1.228238  -0.878  0.38018
## household.income[16K-25K]           0.302518   1.076400   0.281  0.77871
## household.income[25K-35K]          -0.725125   1.058901  -0.685  0.49356
## household.income[35K-50K]          -0.561412   1.012343  -0.555  0.57926
## household.income[50K-75K]          -2.005423   0.976058  -2.055  0.04006 *
## household.income[5K-12K]            0.297411   1.133307   0.262  0.79302
## household.income[75K-100K]         -2.730079   0.989004  -2.760  0.00583 **
## high.educBachelor                   1.597982   0.950786   1.681  0.09300 .
## high.educHS Diploma/GED            -0.713699   0.980320  -0.728  0.46669
## high.educPost Graduate Degree       0.761277   0.950654   0.801  0.42336
## high.educSome College               1.022522   0.908486   1.126  0.26052
## PDS_score:lOFC_posvsneg_feedback_z  0.089986   0.404241   0.223  0.82387
## ---
## Signif. codes:  0 '***' 0.001 '**' 0.01 '*' 0.05 '.' 0.1 ' ' 1
##
##
## R-sq.(adj) =  0.0344
## lmer.REML =  11325  Scale est. = 15.384    n = 1824
```

```
##                                          stdcoef      stdse
## X(Intercept)                         0.000000000 0.00000000
## XPDS_score                           0.062061720 0.02456225
## XlOFC_posvsneg_feedback_z           -0.011042104 0.05790098
## Xrace.ethnicity.5levelBlack         -0.006625664 0.05907462
## Xrace.ethnicity.5levelMixed          0.073716986 0.06167380
## Xrace.ethnicity.5levelOther          0.006659198 0.04576580
## Xrace.ethnicity.5levelWhite          0.095257415 0.08143729
## Xdemo_race_hispanic1                 0.028238200 0.02864616
## Xinterview_age                       0.010498372 0.02324510
## Xbmi                                 0.017124772 0.02408464
## Xhousehold.income[>=200K]           -0.185701085 0.06155735
## Xhousehold.income[100K-200K]        -0.223397577 0.08264389
## Xhousehold.income[12K-16K]          -0.027928395 0.03181705
## Xhousehold.income[16K-25K]           0.010688379 0.03803071
## Xhousehold.income[25K-35K]          -0.027524095 0.04019347
## Xhousehold.income[35K-50K]          -0.026675643 0.04810174
## Xhousehold.income[50K-75K]          -0.123924861 0.06031536
## Xhousehold.income[5K-12K]            0.008863743 0.03377590
## Xhousehold.income[75K-100K]         -0.176753450 0.06403106
## Xhigh.educBachelor                   0.126680877 0.07537408
## Xhigh.educHS Diploma/GED            -0.029902354 0.04107316
```

```
## Xhigh.educPost Graduate Degree       0.066824424 0.08344789
## Xhigh.educSome College               0.078940212 0.07013646
## XPDS_score:lOFC_posvsneg_feedback_z  0.012944343 0.05814925
```

4.10 Model: CBCL internalizing factor ~ PDS x Medial OFC activity (feedback stage)

Female participants

```
##
## Family: gaussian
## Link function: identity
##
## Formula:
## cbcl_scr_syn_internal_r ~ PDS_score * mOFC_posvsneg_feedback_z +
##     race.ethnicity.5level + demo_race_hispanic + interview_age +
##     bmi + household.income + high.educ
##
## Parametric coefficients:
##                                    Estimate Std. Error t value Pr(>|t|)
## (Intercept)                         0.38712    2.43162   0.159   0.8735
## PDS_score                           0.76028    0.19404   3.918 9.25e-05 ***
## mOFC_posvsneg_feedback_z           -0.50792    0.50771  -1.000   0.3172
## race.ethnicity.5levelBlack         -0.44012    0.89170  -0.494   0.6217
## race.ethnicity.5levelMixed          1.10747    0.85501   1.295   0.1954
## race.ethnicity.5levelOther         -0.16579    1.02495  -0.162   0.8715
## race.ethnicity.5levelWhite          1.52923    0.79407   1.926   0.0543 .
## demo_race_hispanic1                -0.23353    0.39138  -0.597   0.5508
## interview_age                       0.02070    0.01742   1.188   0.2349
## bmi                                 0.03224    0.03473   0.928   0.3535
## household.income[>=200K]           -2.12483    0.96950  -2.192   0.0285 *
## household.income[100K-200K]        -1.09009    0.90505  -1.204   0.2286
## household.income[12K-16K]           0.02058    1.12426   0.018   0.9854
## household.income[16K-25K]          -1.25048    1.03215  -1.212   0.2258
## household.income[25K-35K]           0.53796    0.94739   0.568   0.5702
## household.income[35K-50K]          -0.57381    0.92545  -0.620   0.5353
## household.income[50K-75K]          -0.92159    0.90757  -1.015   0.3100
## household.income[5K-12K]            0.22099    1.07020   0.206   0.8364
## household.income[75K-100K]         -0.87021    0.91625  -0.950   0.3424
## high.educBachelor                  -0.22513    0.83778  -0.269   0.7882
## high.educHS Diploma/GED            -0.35398    0.85094  -0.416   0.6775
## high.educPost Graduate Degree       0.19295    0.85138   0.227   0.8207
## high.educSome College               0.40759    0.78890   0.517   0.6055
## PDS_score:mOFC_posvsneg_feedback_z  0.48424    0.27660   1.751   0.0802 .
## ---
## Signif. codes:  0 '***' 0.001 '**' 0.01 '*' 0.05 '.' 0.1 ' ' 1
##
##
## R-sq.(adj) =  0.0277
## lmer.REML =  11355  Scale est. = 15.46     n = 1849
```

```
##                                          stdcoef      stdse
## X(Intercept)                        0.0000000000 0.00000000
```

```
## XPDS_score                          0.1019066245 0.02600872
## XmOFC_posvsneg_feedback_z          -0.0562070018 0.05618424
## Xrace.ethnicity.5levelBlack        -0.0276202527 0.05595960
## Xrace.ethnicity.5levelMixed         0.0683864679 0.05279737
## Xrace.ethnicity.5levelOther        -0.0062387519 0.03856936
## Xrace.ethnicity.5levelWhite         0.1333923375 0.06926541
## Xdemo_race_hispanic1               -0.0168524509 0.02824311
## Xinterview_age                      0.0288307901 0.02426517
## Xbmi                                0.0228770706 0.02464973
## Xhousehold.income[>=200K]          -0.1272491656 0.05806013
## Xhousehold.income[100K-200K]       -0.0958717683 0.07959826
## Xhousehold.income[12K-16K]          0.0005899695 0.03223538
## Xhousehold.income[16K-25K]         -0.0437928490 0.03614674
## Xhousehold.income[25K-35K]          0.0241749399 0.04257404
## Xhousehold.income[35K-50K]         -0.0295892879 0.04772147
## Xhousehold.income[50K-75K]         -0.0578352250 0.05695545
## Xhousehold.income[5K-12K]           0.0068611262 0.03322729
## Xhousehold.income[75K-100K]        -0.0584662434 0.06155922
## Xhigh.educBachelor                 -0.0184994480 0.06884323
## Xhigh.educHS Diploma/GED           -0.0172518476 0.04147154
## Xhigh.educPost Graduate Degree      0.0175194719 0.07730452
## Xhigh.educSome College              0.0322706153 0.06246032
## XPDS_score:mOFC_posvsneg_feedback_z  0.0980655061 0.05601652
```

Male participants

```
##
## Family: gaussian
## Link function: identity
##
## Formula:
## cbcl_scr_syn_internal_r ~ PDS_score * mOFC_posvsneg_feedback_z +
##     race.ethnicity.5level + demo_race_hispanic + interview_age +
##     bmi + household.income + high.educ
##
## Parametric coefficients:
##                                Estimate Std. Error t value Pr(>|t|)
## (Intercept)                    2.705284   2.564134   1.055  0.29154
## PDS_score                      0.683700   0.267118   2.560  0.01056 *
## mOFC_posvsneg_feedback_z      -0.152095   0.513581  -0.296  0.76715
## race.ethnicity.5levelBlack    -0.155619   1.095354  -0.142  0.88704
## race.ethnicity.5levelMixed     1.314353   1.069340   1.229  0.21918
## race.ethnicity.5levelOther     0.206730   1.201419   0.172  0.86340
## race.ethnicity.5levelWhite     1.196304   1.009303   1.185  0.23606
## demo_race_hispanic1            0.386342   0.408376   0.946  0.34425
## interview_age                  0.008476   0.017047   0.497  0.61910
## bmi                            0.026992   0.036941   0.731  0.46506
## household.income[>=200K]      -3.116839   1.018219  -3.061  0.00224 **
## household.income[100K-200K]   -2.643988   0.963617  -2.744  0.00613 **
## household.income[12K-16K]     -1.006278   1.210510  -0.831  0.40592
## household.income[16K-25K]      0.258988   1.057312   0.245  0.80652
## household.income[25K-35K]     -0.734954   1.049556  -0.700  0.48386
## household.income[35K-50K]     -0.560274   1.002724  -0.559  0.57640
```

```
## household.income[50K-75K]         -2.041856   0.965917  -2.114  0.03466 *
## household.income[5K-12K]           0.270213   1.124088   0.240  0.81006
## household.income[75K-100K]        -2.739494   0.978903  -2.799  0.00519 **
## high.educBachelor                  1.547156   0.940430   1.645  0.10011
## high.educHS Diploma/GED           -0.744965   0.969281  -0.769  0.44225
## high.educPost Graduate Degree      0.731263   0.940295   0.778  0.43685
## high.educSome College              0.986081   0.896981   1.099  0.27177
## PDS_score:mOFC_posvsneg_feedback_z  0.006311   0.355274   0.018  0.98583
## ---
## Signif. codes:  0 '***' 0.001 '**' 0.01 '*' 0.05 '.' 0.1 ' ' 1
##
##
## R-sq.(adj) =  0.0344
## lmer.REML =  11375  Scale est. = 15.322    n = 1833
```

```
##                                          stdcoef      stdse
## X(Intercept)                         0.000000000 0.00000000
## XPDS_score                           0.062345059 0.02435787
## XmOFC_posvsneg_feedback_z           -0.017610891 0.05946685
## Xrace.ethnicity.5levelBlack         -0.008409815 0.05919395
## Xrace.ethnicity.5levelMixed          0.076024307 0.06185233
## Xrace.ethnicity.5levelOther          0.007885779 0.04582840
## Xrace.ethnicity.5levelWhite          0.096689985 0.08157578
## Xdemo_race_hispanic1                 0.027031556 0.02857319
## Xinterview_age                       0.011533747 0.02319698
## Xbmi                                 0.017539365 0.02400376
## Xhousehold.income[>=200K]           -0.186597126 0.06095815
## Xhousehold.income[100K-200K]        -0.224849269 0.08194762
## Xhousehold.income[12K-16K]          -0.026375242 0.03172831
## Xhousehold.income[16K-25K]           0.009320622 0.03805127
## Xhousehold.income[25K-35K]          -0.027881369 0.03981621
## Xhousehold.income[35K-50K]          -0.026608669 0.04762160
## Xhousehold.income[50K-75K]          -0.126139135 0.05967114
## Xhousehold.income[5K-12K]            0.008048181 0.03348054
## Xhousehold.income[75K-100K]         -0.177577488 0.06345371
## Xhigh.educBachelor                   0.122916317 0.07471400
## Xhigh.educHS Diploma/GED            -0.031333346 0.04076809
## Xhigh.educPost Graduate Degree       0.064251188 0.08261742
## Xhigh.educSome College               0.076362645 0.06946275
## XPDS_score:mOFC_posvsneg_feedback_z  0.001058222 0.05957656
```

4.11 Model: CBCL internalizing factor ~ PDS x BIS-BAS

Female participants

```
##
## Family: gaussian
## Link function: identity
##
## Formula:
## cbcl_scr_syn_internal_r ~ PDS_score * bisbas_ss_basm_rr + race.ethnicity.5level +
##     demo_race_hispanic + interview_age + bmi + household.income +
##     high.educ
```

```
##
## Parametric coefficients:
##                              Estimate Std. Error t value Pr(>|t|)
## (Intercept)                 -1.964742   2.316890  -0.848  0.39652
## PDS_score                    2.291122   0.572318   4.003 6.44e-05 ***
## bisbas_ss_basm_rr            0.270771   0.112787   2.401  0.01644 *
## race.ethnicity.5levelBlack  -0.480223   0.801906  -0.599  0.54933
## race.ethnicity.5levelMixed   1.182151   0.779342   1.517  0.12944
## race.ethnicity.5levelOther  -0.142460   0.912020  -0.156  0.87589
## race.ethnicity.5levelWhite   1.176660   0.726192   1.620  0.10530
## demo_race_hispanic1          0.041940   0.348870   0.120  0.90432
## interview_age                0.021133   0.015491   1.364  0.17265
## bmi                          0.022214   0.030695   0.724  0.46931
## household.income[>=200K]    -2.536212   0.839799  -3.020  0.00255 **
## household.income[100K-200K] -1.589044   0.781394  -2.034  0.04210 *
## household.income[12K-16K]   -0.175324   1.001862  -0.175  0.86110
## household.income[16K-25K]   -1.301729   0.865942  -1.503  0.13291
## household.income[25K-35K]    0.034045   0.817465   0.042  0.96678
## household.income[35K-50K]   -1.233934   0.793966  -1.554  0.12028
## household.income[50K-75K]   -1.240914   0.778372  -1.594  0.11102
## household.income[5K-12K]    -0.009487   0.878712  -0.011  0.99139
## household.income[75K-100K]  -1.305847   0.792143  -1.648  0.09938 .
## high.educBachelor            0.838193   0.726124   1.154  0.24848
## high.educHS Diploma/GED      0.673239   0.729377   0.923  0.35608
## high.educPost Graduate Degree  1.254149   0.740103   1.695  0.09029 .
## high.educSome College        1.121949   0.677781   1.655  0.09799 .
## PDS_score:bisbas_ss_basm_rr -0.185901   0.061830  -3.007  0.00267 **
## ---
## Signif. codes:  0 '***' 0.001 '**' 0.01 '*' 0.05 '.' 0.1 ' ' 1
##
##
## R-sq.(adj) =  0.0242
## lmer.REML =  14690  Scale est. = 17.387    n = 2386
```

```
##                                     stdcoef      stdse
## X(Intercept)                  0.0000000000 0.00000000
## XPDS_score                    0.3090182126 0.07719221
## Xbisbas_ss_basm_rr            0.1198830949 0.04993597
## Xrace.ethnicity.5levelBlack  -0.0320626833 0.05354020
## Xrace.ethnicity.5levelMixed   0.0717270701 0.04728661
## Xrace.ethnicity.5levelOther  -0.0054991619 0.03520531
## Xrace.ethnicity.5levelWhite   0.1037995012 0.06406128
## Xdemo_race_hispanic1          0.0030015000 0.02496745
## Xinterview_age                0.0292235831 0.02142233
## Xbmi                          0.0157925604 0.02182122
## Xhousehold.income[>=200K]    -0.1491902988 0.04940038
## Xhousehold.income[100K-200K] -0.1380421585 0.06788063
## Xhousehold.income[12K-16K]   -0.0048419230 0.02766841
## Xhousehold.income[16K-25K]   -0.0482043951 0.03206672
## Xhousehold.income[25K-35K]    0.0015151870 0.03638185
## Xhousehold.income[35K-50K]   -0.0650546439 0.04185893
## Xhousehold.income[50K-75K]   -0.0791406468 0.04964148
## Xhousehold.income[5K-12K]    -0.0003301899 0.03058376
## Xhousehold.income[75K-100K]  -0.0864933352 0.05246797
```

```
## Xhigh.educBachelor              0.0686455633 0.05946745
## Xhigh.educHS Diploma/GED        0.0336804238 0.03648884
## Xhigh.educPost Graduate Degree  0.1126989789 0.06650635
## Xhigh.educSome College          0.0899320017 0.05432890
## XPDS_score:bisbas_ss_basm_rr   -0.2721088169 0.09050284
```

Male participants

```
##
## Family: gaussian
## Link function: identity
##
## Formula:
## cbcl_scr_syn_internal_r ~ PDS_score * bisbas_ss_basm_rr + race.ethnicity.5level +
##     demo_race_hispanic + interview_age + bmi + household.income +
##     high.educ
##
## Parametric coefficients:
##                                Estimate Std. Error t value Pr(>|t|)
## (Intercept)                    2.437716   2.424760   1.005 0.314828
## PDS_score                      1.561503   0.850373   1.836 0.066437 .
## bisbas_ss_basm_rr              0.055658   0.127344   0.437 0.662101
## race.ethnicity.5levelBlack    -0.695545   0.876734  -0.793 0.427656
## race.ethnicity.5levelMixed     1.081876   0.856435   1.263 0.206622
## race.ethnicity.5levelOther    -0.048561   0.974318  -0.050 0.960253
## race.ethnicity.5levelWhite     0.853462   0.806188   1.059 0.289865
## demo_race_hispanic1            0.154129   0.349637   0.441 0.659377
## interview_age                  0.008173   0.014709   0.556 0.578502
## bmi                            0.038892   0.030298   1.284 0.199379
## household.income[>=200K]      -3.196990   0.817881  -3.909 9.52e-05 ***
## household.income[100K-200K]   -2.514059   0.762064  -3.299 0.000984 ***
## household.income[12K-16K]     -0.397658   0.983428  -0.404 0.685983
## household.income[16K-25K]      0.075379   0.819508   0.092 0.926721
## household.income[25K-35K]     -0.016679   0.821088  -0.020 0.983795
## household.income[35K-50K]     -1.118032   0.778295  -1.437 0.150979
## household.income[50K-75K]     -1.610669   0.754803  -2.134 0.032947 *
## household.income[5K-12K]       0.012167   0.858481   0.014 0.988693
## household.income[75K-100K]    -2.697662   0.776628  -3.474 0.000522 ***
## high.educBachelor              1.571194   0.769805   2.041 0.041351 *
## high.educHS Diploma/GED       -0.775980   0.762577  -1.018 0.308977
## high.educPost Graduate Degree  0.827979   0.772584   1.072 0.283956
## high.educSome College          1.034236   0.731760   1.413 0.157674
## PDS_score:bisbas_ss_basm_rr   -0.110327   0.089659  -1.231 0.218619
## ---
## Signif. codes:  0 '***' 0.001 '**' 0.01 '*' 0.05 '.' 0.1 ' ' 1
##
##
## R-sq.(adj) =  0.0362
## lmer.REML =  15928  Scale est. = 17.36     n = 2559
```

```
##                                      stdcoef      stdse
## X(Intercept)                    0.0000000000 0.00000000
## XPDS_score                      0.1522351501 0.08290513
```

```
## Xbisbas_ss_basm_rr              0.0227285829 0.05200276
## Xrace.ethnicity.5levelBlack    -0.0405680496 0.05113596
## Xrace.ethnicity.5levelMixed     0.0631882989 0.05002112
## Xrace.ethnicity.5levelOther    -0.0018252920 0.03662261
## Xrace.ethnicity.5levelWhite     0.0704034365 0.06650372
## Xdemo_race_hispanic1            0.0107999960 0.02449952
## Xinterview_age                  0.0109896647 0.01977819
## Xbmi                            0.0262234317 0.02042868
## Xhousehold.income[>=200K]      -0.1856883480 0.04750437
## Xhousehold.income[100K-200K]   -0.2091460066 0.06339649
## Xhousehold.income[12K-16K]     -0.0102021261 0.02523037
## Xhousehold.income[16K-25K]      0.0028333247 0.03080363
## Xhousehold.income[25K-35K]     -0.0006608686 0.03253429
## Xhousehold.income[35K-50K]     -0.0552475222 0.03845941
## Xhousehold.income[50K-75K]     -0.1003127513 0.04700923
## Xhousehold.income[5K-12K]       0.0003936045 0.02777120
## Xhousehold.income[75K-100K]    -0.1719741245 0.04950950
## Xhigh.educBachelor              0.1232406557 0.06038164
## Xhigh.educHS Diploma/GED       -0.0367512102 0.03611646
## Xhigh.educPost Graduate Degree  0.0714830729 0.06670058
## Xhigh.educSome College          0.0809261067 0.05725817
## XPDS_score:bisbas_ss_basm_rr   -0.1197052410 0.09728080
```

4.12 Model: CBCL internalizing factor ~ PDS x MID reaction time (large reward vs. neutral)

Female participants

```
##
## Family: gaussian
## Link function: identity
##
## Formula:
## cbcl_scr_syn_internal_r ~ PDS_score * rt_diff_large_neutral_z +
##     race.ethnicity.5level + demo_race_hispanic + interview_age +
##     bmi + household.income + high.educ
##
## Parametric coefficients:
##                               Estimate Std. Error t value Pr(>|t|)
## (Intercept)                    0.483412   2.328269   0.208   0.8355
## PDS_score                      0.855324   0.187590   4.560 5.44e-06 ***
## rt_diff_large_neutral_z        0.180991   0.322271   0.562   0.5744
## race.ethnicity.5levelBlack    -0.810916   0.856497  -0.947   0.3439
## race.ethnicity.5levelMixed     0.721284   0.825769   0.873   0.3825
## race.ethnicity.5levelOther    -0.385265   0.964903  -0.399   0.6897
## race.ethnicity.5levelWhite     1.173062   0.768815   1.526   0.1272
## demo_race_hispanic1           -0.091549   0.377055  -0.243   0.8082
## interview_age                  0.021268   0.016845   1.263   0.2069
## bmi                            0.021758   0.032986   0.660   0.5096
## household.income[>=200K]      -1.989396   0.923212  -2.155   0.0313 *
## household.income[100K-200K]   -1.121947   0.860960  -1.303   0.1927
## household.income[12K-16K]      0.006039   1.085815   0.006   0.9956
## household.income[16K-25K]     -1.085341   0.960839  -1.130   0.2588
```

```
## household.income[25K-35K]         0.687445   0.901306   0.763   0.4457
## household.income[35K-50K]        -0.594679   0.876094  -0.679   0.4974
## household.income[50K-75K]        -0.931958   0.860126  -1.084   0.2787
## household.income[5K-12K]          0.750464   1.011357   0.742   0.4582
## household.income[75K-100K]       -0.901655   0.873943  -1.032   0.3023
## high.educBachelor                -0.043493   0.791524  -0.055   0.9562
## high.educHS Diploma/GED          -0.136944   0.804438  -0.170   0.8648
## high.educPost Graduate Degree     0.369040   0.806417   0.458   0.6473
## high.educSome College             0.572753   0.743359   0.770   0.4411
## PDS_score:rt_diff_large_neutral_z -0.049630   0.177514  -0.280   0.7798
## ---
## Signif. codes:  0 '***' 0.001 '**' 0.01 '*' 0.05 '.' 0.1 ' ' 1
##
##
## R-sq.(adj) =  0.0274
## lmer.REML =  12297  Scale est. = 17.028    n = 2001
```

```
##                                        stdcoef      stdse
## X(Intercept)                       0.0000000000 0.00000000
## XPDS_score                         0.1134343877 0.02487851
## Xrt_diff_large_neutral_z           0.0312011385 0.05555635
## Xrace.ethnicity.5levelBlack       -0.0516711524 0.05457557
## Xrace.ethnicity.5levelMixed        0.0445684357 0.05102462
## Xrace.ethnicity.5levelOther       -0.0153085199 0.03834042
## Xrace.ethnicity.5levelWhite        0.1030428186 0.06753342
## Xdemo_race_hispanic1              -0.0066286652 0.02730090
## Xinterview_age                     0.0294057792 0.02329089
## Xbmi                               0.0155220774 0.02353166
## Xhousehold.income[>=200K]         -0.1186398849 0.05505675
## Xhousehold.income[100K-200K]      -0.0983707116 0.07548775
## Xhousehold.income[12K-16K]         0.0001682809 0.03025509
## Xhousehold.income[16K-25K]        -0.0397694456 0.03520742
## Xhousehold.income[25K-35K]         0.0306992988 0.04024974
## Xhousehold.income[35K-50K]        -0.0311542067 0.04589705
## Xhousehold.income[50K-75K]        -0.0588270667 0.05429289
## Xhousehold.income[5K-12K]          0.0236031988 0.03180865
## Xhousehold.income[75K-100K]       -0.0597648842 0.05792801
## Xhigh.educBachelor                -0.0035789618 0.06513260
## Xhigh.educHS Diploma/GED          -0.0066634878 0.03914277
## Xhigh.educPost Graduate Degree     0.0333594981 0.07289622
## Xhigh.educSome College             0.0455687147 0.05914231
## XPDS_score:rt_diff_large_neutral_z -0.0155201778 0.05551163
```

Male participants

```
##
## Family: gaussian
## Link function: identity
##
## Formula:
## cbcl_scr_syn_internal_r ~ PDS_score * rt_diff_large_neutral_z +
##     race.ethnicity.5level + demo_race_hispanic + interview_age +
##     bmi + household.income + high.educ
```

```
##
## Parametric coefficients:
##                                 Estimate Std. Error t value Pr(>|t|)
## (Intercept)                     1.750814   2.438546   0.718  0.47286
## PDS_score                       0.713788   0.244639   2.918  0.00356 **
## rt_diff_large_neutral_z         0.947685   0.354278   2.675  0.00753 **
## race.ethnicity.5levelBlack     -0.711053   1.051563  -0.676  0.49900
## race.ethnicity.5levelMixed      0.766583   1.030263   0.744  0.45692
## race.ethnicity.5levelOther     -0.182190   1.146920  -0.159  0.87380
## race.ethnicity.5levelWhite      0.799698   0.972742   0.822  0.41111
## demo_race_hispanic1             0.202015   0.389428   0.519  0.60399
## interview_age                   0.009153   0.016173   0.566  0.57147
## bmi                             0.040581   0.034733   1.168  0.24279
## household.income[>=200K]       -2.522586   0.940886  -2.681  0.00740 **
## household.income[100K-200K]    -2.071642   0.886966  -2.336  0.01961 *
## household.income[12K-16K]       0.345890   1.114246   0.310  0.75627
## household.income[16K-25K]       0.813649   0.971631   0.837  0.40246
## household.income[25K-35K]       0.194017   0.959768   0.202  0.83982
## household.income[35K-50K]      -0.080480   0.907634  -0.089  0.92935
## household.income[50K-75K]     -1.194339   0.883090  -1.352  0.17638
## household.income[5K-12K]        0.578763   1.025258   0.565  0.57247
## household.income[75K-100K]     -2.120489   0.902491  -2.350  0.01889 *
## high.educBachelor               1.923476   0.895775   2.147  0.03189 *
## high.educHS Diploma/GED        -0.059677   0.904250  -0.066  0.94739
## high.educPost Graduate Degree   1.152730   0.895154   1.288  0.19798
## high.educSome College           1.191013   0.851205   1.399  0.16190
## PDS_score:rt_diff_large_neutral_z -0.795264   0.254145  -3.129  0.00178 **
## ---
## Signif. codes:  0 '***' 0.001 '**' 0.01 '*' 0.05 '.' 0.1 ' ' 1
##
##
## R-sq.(adj) =  0.0373
## lmer.REML =  12711  Scale est. = 13.784    n = 2048
```

```
##                                       stdcoef       stdse
## X(Intercept)                      0.000000000 0.00000000
## XPDS_score                        0.067580199 0.02316200
## Xrt_diff_large_neutral_z          0.155727454 0.05821650
## Xrace.ethnicity.5levelBlack      -0.039348580 0.05819190
## Xrace.ethnicity.5levelMixed       0.044153226 0.05934049
## Xrace.ethnicity.5levelOther      -0.006896019 0.04341172
## Xrace.ethnicity.5levelWhite       0.064787225 0.07880631
## Xdemo_race_hispanic1              0.014091280 0.02716405
## Xinterview_age                    0.012421459 0.02194666
## Xbmi                              0.026518715 0.02269689
## Xhousehold.income[>=200K]        -0.149643755 0.05581486
## Xhousehold.income[100K-200K]     -0.174577460 0.07474473
## Xhousehold.income[12K-16K]        0.008980739 0.02893041
## Xhousehold.income[16K-25K]        0.029546432 0.03528327
## Xhousehold.income[25K-35K]        0.007487446 0.03703900
## Xhousehold.income[35K-50K]       -0.003976095 0.04484161
## Xhousehold.income[50K-75K]       -0.074133045 0.05481371
## Xhousehold.income[5K-12K]         0.017477638 0.03096103
## Xhousehold.income[75K-100K]      -0.135913743 0.05784560
```

```
## Xhigh.educBachelor                  0.150575723 0.07012409
## Xhigh.educHS Diploma/GED           -0.002624143 0.03976231
## Xhigh.educPost Graduate Degree      0.101100893 0.07851005
## Xhigh.educSome College              0.092591022 0.06617391
## XPDS_score:rt_diff_large_neutral_z -0.182637233 0.05836586
```

4.13 Model: CBCL internalizing factor ~ PDS x MID reaction time (large vs. small reward)

Female participants

```
##
## Family: gaussian
## Link function: identity
##
## Formula:
## cbcl_scr_syn_internal_r ~ PDS_score * rt_diff_large_small_z +
##     race.ethnicity.5level + demo_race_hispanic + interview_age +
##     bmi + household.income + high.educ
##
## Parametric coefficients:
##                                 Estimate Std. Error t value Pr(>|t|)
## (Intercept)                     0.465158   2.326515   0.200   0.8415
## PDS_score                       0.853732   0.187588   4.551 5.66e-06 ***
## rt_diff_large_small_z           0.256418   0.302976   0.846   0.3975
## race.ethnicity.5levelBlack     -0.795268   0.856419  -0.929   0.3532
## race.ethnicity.5levelMixed      0.737366   0.825822   0.893   0.3720
## race.ethnicity.5levelOther     -0.359183   0.964589  -0.372   0.7097
## race.ethnicity.5levelWhite      1.191243   0.768466   1.550   0.1213
## demo_race_hispanic1            -0.098777   0.376948  -0.262   0.7933
## interview_age                   0.021482   0.016833   1.276   0.2021
## bmi                             0.020974   0.032996   0.636   0.5251
## household.income[>=200K]       -1.993381   0.923763  -2.158   0.0311 *
## household.income[100K-200K]    -1.136227   0.860969  -1.320   0.1871
## household.income[12K-16K]      -0.002076   1.086360  -0.002   0.9985
## household.income[16K-25K]      -1.107996   0.961274  -1.153   0.2492
## household.income[25K-35K]       0.693545   0.902131   0.769   0.4421
## household.income[35K-50K]      -0.620326   0.875512  -0.709   0.4787
## household.income[50K-75K]      -0.940366   0.860516  -1.093   0.2746
## household.income[5K-12K]        0.748959   1.011491   0.740   0.4591
## household.income[75K-100K]     -0.911658   0.873901  -1.043   0.2970
## high.educBachelor              -0.039829   0.790783  -0.050   0.9598
## high.educHS Diploma/GED        -0.134607   0.803768  -0.167   0.8670
## high.educPost Graduate Degree   0.379282   0.805965   0.471   0.6380
## high.educSome College           0.591246   0.742842   0.796   0.4262
## PDS_score:rt_diff_large_small_z -0.085706   0.167808  -0.511   0.6096
## ---
## Signif. codes:  0 '***' 0.001 '**' 0.01 '*' 0.05 '.' 0.1 ' ' 1
##
##
## R-sq.(adj) =  0.0276
## lmer.REML =  12297  Scale est. = 16.964    n = 2001
```

```
##                                       stdcoef      stdse
## X(Intercept)                     0.000000e+00 0.00000000
## XPDS_score                       1.132232e-01 0.02487819
## Xrt_diff_large_small_z           4.601239e-02 0.05436674
## Xrace.ethnicity.5levelBlack     -5.067406e-02 0.05457058
## Xrace.ethnicity.5levelMixed      4.556215e-02 0.05102789
## Xrace.ethnicity.5levelOther     -1.427215e-02 0.03832794
## Xrace.ethnicity.5levelWhite      1.046399e-01 0.06750278
## Xdemo_race_hispanic1            -7.152033e-03 0.02729315
## Xinterview_age                   2.970139e-02 0.02327416
## Xbmi                             1.496306e-02 0.02353906
## Xhousehold.income[>=200K]       -1.188775e-01 0.05508961
## Xhousehold.income[100K-200K]    -9.962276e-02 0.07548858
## Xhousehold.income[12K-16K]      -5.785645e-05 0.03027028
## Xhousehold.income[16K-25K]      -4.059959e-02 0.03522336
## Xhousehold.income[25K-35K]       3.097171e-02 0.04028657
## Xhousehold.income[35K-50K]      -3.249784e-02 0.04586659
## Xhousehold.income[50K-75K]      -5.935781e-02 0.05431751
## Xhousehold.income[5K-12K]        2.355586e-02 0.03181288
## Xhousehold.income[75K-100K]     -6.042789e-02 0.05792527
## Xhigh.educBachelor              -3.277438e-03 0.06507160
## Xhigh.educHS Diploma/GED        -6.549760e-03 0.03911016
## Xhigh.educPost Graduate Degree   3.428531e-02 0.07285536
## Xhigh.educSome College           4.704006e-02 0.05910121
## XPDS_score:rt_diff_large_small_z -2.782374e-02 0.05447761
```

Male participants

```
##
## Family: gaussian
## Link function: identity
##
## Formula:
## cbcl_scr_syn_internal_r ~ PDS_score * rt_diff_large_small_z +
##     race.ethnicity.5level + demo_race_hispanic + interview_age +
##     bmi + household.income + high.educ
##
## Parametric coefficients:
##                               Estimate Std. Error t value Pr(>|t|)
## (Intercept)                   1.870093   2.442116   0.766  0.44390
## PDS_score                     0.706754   0.245997   2.873  0.00411 **
## rt_diff_large_small_z         0.350585   0.352886   0.993  0.32059
## race.ethnicity.5levelBlack   -0.755140   1.053667  -0.717  0.47366
## race.ethnicity.5levelMixed    0.640032   1.031413   0.621  0.53497
## race.ethnicity.5levelOther   -0.196229   1.149751  -0.171  0.86450
## race.ethnicity.5levelWhite    0.684948   0.974263   0.703  0.48211
## demo_race_hispanic1           0.220485   0.390379   0.565  0.57227
## interview_age                 0.008129   0.016188   0.502  0.61561
## bmi                           0.041499   0.034804   1.192  0.23326
## household.income[>=200K]     -2.452020   0.942582  -2.601  0.00935 **
## household.income[100K-200K]  -2.008295   0.888780  -2.260  0.02395 *
## household.income[12K-16K]     0.261321   1.116560   0.234  0.81498
## household.income[16K-25K]     0.925416   0.973383   0.951  0.34186
```

```
## household.income[25K-35K]        0.264859   0.962166   0.275  0.78313
## household.income[35K-50K]        0.023691   0.908774   0.026  0.97920
## household.income[50K-75K]       -1.114897   0.884676  -1.260  0.20773
## household.income[5K-12K]         0.723915   1.026440   0.705  0.48072
## household.income[75K-100K]      -2.037157   0.904216  -2.253  0.02437 *
## high.educBachelor                1.949119   0.897641   2.171  0.03002 *
## high.educHS Diploma/GED         -0.071330   0.906263  -0.079  0.93727
## high.educPost Graduate Degree    1.186178   0.896976   1.322  0.18618
## high.educSome College            1.216411   0.853046   1.426  0.15403
## PDS_score:rt_diff_large_small_z -0.332939   0.251496  -1.324  0.18571
## ---
## Signif. codes:  0 '***' 0.001 '**' 0.01 '*' 0.05 '.' 0.1 ' ' 1
##
##
## R-sq.(adj) =  0.0336
## lmer.REML =  12719  Scale est. = 13.792    n = 2048
```

```
##                                      stdcoef      stdse
## X(Intercept)                     0.000000000 0.00000000
## XPDS_score                       0.066914196 0.02329061
## Xrt_diff_large_small_z           0.056712021 0.05708419
## Xrace.ethnicity.5levelBlack     -0.041788288 0.05830833
## Xrace.ethnicity.5levelMixed      0.036864196 0.05940672
## Xrace.ethnicity.5levelOther     -0.007427417 0.04351889
## Xrace.ethnicity.5levelWhite      0.055490830 0.07892951
## Xdemo_race_hispanic1             0.015379634 0.02723037
## Xinterview_age                   0.011031075 0.02196707
## Xbmi                             0.027118168 0.02274338
## Xhousehold.income[>=200K]       -0.145457686 0.05591547
## Xhousehold.income[100K-200K]    -0.169239163 0.07489760
## Xhousehold.income[12K-16K]       0.006784967 0.02899049
## Xhousehold.income[16K-25K]       0.033605073 0.03534690
## Xhousehold.income[25K-35K]       0.010221318 0.03713152
## Xhousehold.income[35K-50K]       0.001170467 0.04489793
## Xhousehold.income[50K-75K]      -0.069202059 0.05491216
## Xhousehold.income[5K-12K]        0.021860983 0.03099672
## Xhousehold.income[75K-100K]     -0.130572555 0.05795614
## Xhigh.educBachelor               0.152583138 0.07027013
## Xhigh.educHS Diploma/GED        -0.003136557 0.03985083
## Xhigh.educPost Graduate Degree   0.104034481 0.07866989
## Xhigh.educSome College           0.094565569 0.06631704
## XPDS_score:rt_diff_large_small_z -0.076267999 0.05761143
```

4.14 Model: CBCL internalizing factor ~ Testosterone x Accumbens activity (anticipation stage) + PDS

Female participants

```
## Warning: Some predictor variables are on very different scales: consider
## rescaling
```

```
##
## Family: gaussian
```

```
## Link function: identity
##
## Formula:
## cbcl_scr_syn_internal_r ~ PDS_score + hormone_sal_end_min_since_midnight +
##     hormone_scr_ert_mean * accumbens_rvsn_ant_z + race.ethnicity.5level +
##     demo_race_hispanic + interview_age + MRI_minus_hormone_date_time +
##     bmi + household.income + high.educ
##
## Parametric coefficients:
##                                             Estimate Std. Error t value
## (Intercept)                               -9.452e-01  2.607e+00  -0.363
## PDS_score                                  8.551e-01  2.049e-01   4.173
## hormone_sal_end_min_since_midnight         8.284e-06  7.971e-04   0.010
## hormone_scr_ert_mean                      -3.709e-03  8.177e-03  -0.454
## accumbens_rvsn_ant_z                       5.906e-01  4.223e-01   1.398
## race.ethnicity.5levelBlack                -7.315e-01  9.032e-01  -0.810
## race.ethnicity.5levelMixed                 8.865e-01  8.682e-01   1.021
## race.ethnicity.5levelOther                -5.864e-01  1.052e+00  -0.558
## race.ethnicity.5levelWhite                 1.441e+00  8.030e-01   1.794
## demo_race_hispanic1                       -1.305e-01  4.041e-01  -0.323
## interview_age                              2.881e-02  1.812e-02   1.590
## MRI_minus_hormone_date_time                4.022e-05  1.635e-05   2.460
## bmi                                        4.328e-02  3.677e-02   1.177
## household.income[>=200K]                  -2.260e+00  9.943e-01  -2.273
## household.income[100K-200K]               -1.423e+00  9.238e-01  -1.540
## household.income[12K-16K]                 -2.295e-01  1.154e+00  -0.199
## household.income[16K-25K]                 -1.379e+00  1.061e+00  -1.300
## household.income[25K-35K]                  1.957e-01  9.656e-01   0.203
## household.income[35K-50K]                 -9.535e-01  9.437e-01  -1.010
## household.income[50K-75K]                 -1.266e+00  9.306e-01  -1.360
## household.income[5K-12K]                  -4.892e-01  1.124e+00  -0.435
## household.income[75K-100K]                -1.196e+00  9.340e-01  -1.281
## high.educBachelor                          1.943e-01  8.811e-01   0.221
## high.educHS Diploma/GED                   -9.843e-02  8.924e-01  -0.110
## high.educPost Graduate Degree              5.533e-01  8.958e-01   0.618
## high.educSome College                      7.772e-01  8.294e-01   0.937
## hormone_scr_ert_mean:accumbens_rvsn_ant_z -2.352e-02  1.019e-02  -2.307
##                                           Pr(>|t|)
## (Intercept)                                 0.7169
## PDS_score                                 3.17e-05 ***
## hormone_sal_end_min_since_midnight          0.9917
## hormone_scr_ert_mean                        0.6502
## accumbens_rvsn_ant_z                        0.1622
## race.ethnicity.5levelBlack                  0.4181
## race.ethnicity.5levelMixed                  0.3074
## race.ethnicity.5levelOther                  0.5772
## race.ethnicity.5levelWhite                  0.0730 .
## demo_race_hispanic1                         0.7467
## interview_age                               0.1120
## MRI_minus_hormone_date_time                 0.0140 *
## bmi                                         0.2394
## household.income[>=200K]                    0.0231 *
## household.income[100K-200K]                 0.1236
## household.income[12K-16K]                   0.8424
```

```
## household.income[16K-25K]                   0.1937
## household.income[25K-35K]                   0.8394
## household.income[35K-50K]                   0.3124
## household.income[50K-75K]                   0.1740
## household.income[5K-12K]                    0.6636
## household.income[75K-100K]                  0.2004
## high.educBachelor                           0.8255
## high.educHS Diploma/GED                     0.9122
## high.educPost Graduate Degree               0.5369
## high.educSome College                       0.3489
## hormone_scr_ert_mean:accumbens_rvsn_ant_z   0.0212 *
## ---
## Signif. codes:  0 '***' 0.001 '**' 0.01 '*' 0.05 '.' 0.1 ' ' 1
##
##
## R-sq.(adj) =  0.034
## lmer.REML =  10241  Scale est. = 15.403    n = 1669
```

```
##                                                 stdcoef      stdse
## X(Intercept)                                0.0000000000 0.00000000
## XPDS_score                                  0.1151112608 0.02758692
## Xhormone_sal_end_min_since_midnight         0.0002720458 0.02617698
## Xhormone_scr_ert_mean                      -0.0117064595 0.02580958
## Xaccumbens_rvsn_ant_z                       0.0762543368 0.05452845
## Xrace.ethnicity.5levelBlack                -0.0459208272 0.05669617
## Xrace.ethnicity.5levelMixed                 0.0547477692 0.05361575
## Xrace.ethnicity.5levelOther                -0.0224145625 0.04020419
## Xrace.ethnicity.5levelWhite                 0.1266115659 0.07057336
## Xdemo_race_hispanic1                       -0.0095142098 0.02945521
## Xinterview_age                              0.0406630141 0.02557239
## XMRI_minus_hormone_date_time                0.0616490564 0.02505686
## Xbmi                                        0.0304848054 0.02589956
## Xhousehold.income[>=200K]                  -0.1356843159 0.05968578
## Xhousehold.income[100K-200K]               -0.1259578935 0.08176756
## Xhousehold.income[12K-16K]                 -0.0066847311 0.03361981
## Xhousehold.income[16K-25K]                 -0.0483096403 0.03715413
## Xhousehold.income[25K-35K]                  0.0090970393 0.04488508
## Xhousehold.income[35K-50K]                 -0.0502119472 0.04969435
## Xhousehold.income[50K-75K]                 -0.0800874329 0.05888521
## Xhousehold.income[5K-12K]                  -0.0145800978 0.03351037
## Xhousehold.income[75K-100K]                -0.0827618842 0.06461114
## Xhigh.educBachelor                          0.0162566505 0.07371651
## Xhigh.educHS Diploma/GED                   -0.0048565056 0.04403192
## Xhigh.educPost Graduate Degree              0.0506997449 0.08208700
## Xhigh.educSome College                      0.0620921719 0.06626686
## Xhormone_scr_ert_mean:accumbens_rvsn_ant_z -0.1257196343 0.05449009
```

Male participants

```
## Warning: Some predictor variables are on very different scales: consider
## rescaling
```

```
##
```

```
## Family: gaussian
## Link function: identity
##
## Formula:
## cbcl_scr_syn_internal_r ~ PDS_score + hormone_sal_end_min_since_midnight +
##     hormone_scr_ert_mean * accumbens_rvsn_ant_z + race.ethnicity.5level +
##     demo_race_hispanic + interview_age + MRI_minus_hormone_date_time +
##     bmi + household.income + high.educ
##
## Parametric coefficients:
##                                             Estimate Std. Error t value
## (Intercept)                                2.795e+00  2.702e+00   1.035
## PDS_score                                  7.937e-01  2.814e-01   2.821
## hormone_sal_end_min_since_midnight         7.768e-04  8.132e-04   0.955
## hormone_scr_ert_mean                       7.709e-03  9.420e-03   0.818
## accumbens_rvsn_ant_z                      -4.280e-03  4.302e-01  -0.010
## race.ethnicity.5levelBlack                 3.156e-02  1.134e+00   0.028
## race.ethnicity.5levelMixed                 1.216e+00  1.103e+00   1.102
## race.ethnicity.5levelOther                 5.017e-01  1.240e+00   0.405
## race.ethnicity.5levelWhite                 1.403e+00  1.041e+00   1.348
## demo_race_hispanic1                        2.209e-03  4.247e-01   0.005
## interview_age                              5.106e-03  1.763e-02   0.290
## MRI_minus_hormone_date_time                2.211e-05  1.873e-05   1.180
## bmi                                        1.978e-03  3.803e-02   0.052
## household.income[>=200K]                  -3.043e+00  1.031e+00  -2.952
## household.income[100K-200K]               -2.609e+00  9.749e-01  -2.676
## household.income[12K-16K]                 -5.410e-01  1.259e+00  -0.430
## household.income[16K-25K]                 3.292e-01  1.073e+00   0.307
## household.income[25K-35K]                 -8.012e-01  1.054e+00  -0.760
## household.income[35K-50K]                 -6.506e-01  1.025e+00  -0.635
## household.income[50K-75K]                 -2.069e+00  9.719e-01  -2.129
## household.income[5K-12K]                  2.966e-01  1.120e+00   0.265
## household.income[75K-100K]                -2.703e+00  9.932e-01  -2.722
## high.educBachelor                          1.107e+00  9.827e-01   1.126
## high.educHS Diploma/GED                   -1.117e+00  1.012e+00  -1.104
## high.educPost Graduate Degree              2.399e-01  9.851e-01   0.244
## high.educSome College                      6.678e-01  9.392e-01   0.711
## hormone_scr_ert_mean:accumbens_rvsn_ant_z -2.688e-03  1.286e-02  -0.209
##                                           Pr(>|t|)
## (Intercept)                                0.30101
## PDS_score                                  0.00485 **
## hormone_sal_end_min_since_midnight         0.33956
## hormone_scr_ert_mean                       0.41330
## accumbens_rvsn_ant_z                       0.99206
## race.ethnicity.5levelBlack                 0.97780
## race.ethnicity.5levelMixed                 0.27047
## race.ethnicity.5levelOther                 0.68582
## race.ethnicity.5levelWhite                 0.17787
## demo_race_hispanic1                        0.99585
## interview_age                              0.77216
## MRI_minus_hormone_date_time                0.23811
## bmi                                        0.95851
## household.income[>=200K]                   0.00320 **
## household.income[100K-200K]                0.00753 **
```

```
## household.income[12K-16K]                0.66760
## household.income[16K-25K]                0.75905
## household.income[25K-35K]                0.44720
## household.income[35K-50K]                0.52567
## household.income[50K-75K]                0.03340 *
## household.income[5K-12K]                 0.79110
## household.income[75K-100K]               0.00656 **
## high.educBachelor                        0.26012
## high.educHS Diploma/GED                  0.26968
## high.educPost Graduate Degree            0.80760
## high.educSome College                    0.47714
## hormone_scr_ert_mean:accumbens_rvsn_ant_z  0.83447
## ---
## Signif. codes:  0 '***' 0.001 '**' 0.01 '*' 0.05 '.' 0.1 ' ' 1
##
##
## R-sq.(adj) =  0.0356
## lmer.REML =  10406  Scale est. = 13.535    n = 1674
```

```
##                                               stdcoef      stdse
## X(Intercept)                               0.0000000000 0.00000000
## XPDS_score                                 0.0723404749 0.02564502
## Xhormone_sal_end_min_since_midnight        0.0249720782 0.02614040
## Xhormone_scr_ert_mean                      0.0204931663 0.02504337
## Xaccumbens_rvsn_ant_z                     -0.0005567837 0.05596713
## Xrace.ethnicity.5levelBlack                0.0017185566 0.06173835
## Xrace.ethnicity.5levelMixed                0.0704817116 0.06393760
## Xrace.ethnicity.5levelOther                0.0192912758 0.04767845
## Xrace.ethnicity.5levelWhite                0.1139795398 0.08456004
## Xdemo_race_hispanic1                       0.0001548751 0.02977191
## Xinterview_age                             0.0070338937 0.02428848
## XMRI_minus_hormone_date_time               0.0294339933 0.02494075
## Xbmi                                       0.0013173751 0.02532139
## Xhousehold.income[>=200K]                 -0.1874760078 0.06349745
## Xhousehold.income[100K-200K]              -0.2221728964 0.08302499
## Xhousehold.income[12K-16K]                -0.0137555632 0.03202509
## Xhousehold.income[16K-25K]                 0.0118783970 0.03872016
## Xhousehold.income[25K-35K]                -0.0313507347 0.04123599
## Xhousehold.income[35K-50K]                -0.0299036175 0.04710972
## Xhousehold.income[50K-75K]                -0.1293417455 0.06075079
## Xhousehold.income[5K-12K]                  0.0091898337 0.03468816
## Xhousehold.income[75K-100K]               -0.1739574460 0.06391699
## Xhigh.educBachelor                         0.0891384651 0.07912945
## Xhigh.educHS Diploma/GED                  -0.0475163265 0.04303340
## Xhigh.educPost Graduate Degree             0.0211888078 0.08699726
## Xhigh.educSome College                     0.0513335740 0.07219249
## Xhormone_scr_ert_mean:accumbens_rvsn_ant_z -0.0116339789 0.05566242
```

4.15 Model: CBCL internalizing factor ~ Testosterone x Caudate activity (anticipation stage) + PDS

Female participants

```
## Warning: Some predictor variables are on very different scales: consider
## rescaling

##
## Family: gaussian
## Link function: identity
##
## Formula:
## cbcl_scr_syn_internal_r ~ PDS_score + hormone_sal_end_min_since_midnight +
##     hormone_scr_ert_mean * caudate_rvsn_ant_z + race.ethnicity.5level +
##     demo_race_hispanic + interview_age + MRI_minus_hormone_date_time +
##     bmi + household.income + high.educ
##
## Parametric coefficients:
##                                         Estimate Std. Error t value Pr(>|t|)
## (Intercept)                           -4.883e-01  2.616e+00  -0.187  0.85196
## PDS_score                              8.134e-01  2.054e-01   3.959 7.85e-05
## hormone_sal_end_min_since_midnight    -3.392e-05  8.014e-04  -0.042  0.96624
## hormone_scr_ert_mean                  -3.980e-03  8.231e-03  -0.484  0.62879
## caudate_rvsn_ant_z                     4.947e-01  3.215e-01   1.538  0.12412
## race.ethnicity.5levelBlack            -7.525e-01  9.076e-01  -0.829  0.40720
## race.ethnicity.5levelMixed             8.977e-01  8.715e-01   1.030  0.30316
## race.ethnicity.5levelOther            -5.283e-01  1.057e+00  -0.500  0.61721
## race.ethnicity.5levelWhite             1.477e+00  8.064e-01   1.832  0.06717
## demo_race_hispanic1                   -1.875e-01  4.050e-01  -0.463  0.64350
## interview_age                          2.639e-02  1.824e-02   1.447  0.14804
## MRI_minus_hormone_date_time            4.122e-05  1.646e-05   2.505  0.01234
## bmi                                    5.585e-02  3.659e-02   1.526  0.12715
## household.income[>=200K]              -2.629e+00  9.922e-01  -2.650  0.00813
## household.income[100K-200K]           -1.747e+00  9.209e-01  -1.897  0.05799
## household.income[12K-16K]             -4.319e-01  1.160e+00  -0.372  0.70965
## household.income[16K-25K]             -1.656e+00  1.059e+00  -1.563  0.11829
## household.income[25K-35K]             -1.877e-01  9.615e-01  -0.195  0.84522
## household.income[35K-50K]             -1.270e+00  9.391e-01  -1.352  0.17659
## household.income[50K-75K]             -1.558e+00  9.264e-01  -1.682  0.09275
## household.income[5K-12K]              -7.828e-01  1.124e+00  -0.697  0.48609
## household.income[75K-100K]            -1.543e+00  9.321e-01  -1.655  0.09809
## high.educBachelor                      1.966e-01  8.796e-01   0.223  0.82320
## high.educHS Diploma/GED               -7.450e-02  8.950e-01  -0.083  0.93367
## high.educPost Graduate Degree          6.087e-01  8.941e-01   0.681  0.49608
## high.educSome College                  7.508e-01  8.296e-01   0.905  0.36556
## hormone_scr_ert_mean:caudate_rvsn_ant_z -1.218e-02  8.165e-03  -1.491  0.13611
##
## (Intercept)
## PDS_score                             ***
## hormone_sal_end_min_since_midnight
## hormone_scr_ert_mean
## caudate_rvsn_ant_z
## race.ethnicity.5levelBlack
```

```
## race.ethnicity.5levelMixed
## race.ethnicity.5levelOther
## race.ethnicity.5levelWhite            .
## demo_race_hispanic1
## interview_age
## MRI_minus_hormone_date_time           *
## bmi
## household.income[>=200K]              **
## household.income[100K-200K]           .
## household.income[12K-16K]
## household.income[16K-25K]
## household.income[25K-35K]
## household.income[35K-50K]
## household.income[50K-75K]             .
## household.income[5K-12K]
## household.income[75K-100K]            .
## high.educBachelor
## high.educHS Diploma/GED
## high.educPost Graduate Degree
## high.educSome College
## hormone_scr_ert_mean:caudate_rvsn_ant_z
## ---
## Signif. codes:  0 '***' 0.001 '**' 0.01 '*' 0.05 '.' 0.1 ' ' 1
##
##
## R-sq.(adj) =  0.0304
## lmer.REML =  10279  Scale est. = 16.006    n = 1672
```

```
##                                              stdcoef      stdse
## X(Intercept)                             0.000000000 0.00000000
## XPDS_score                               0.109124827 0.02756336
## Xhormone_sal_end_min_since_midnight     -0.001107113 0.02615621
## Xhormone_scr_ert_mean                   -0.012524946 0.02590361
## Xcaudate_rvsn_ant_z                      0.082137502 0.05338872
## Xrace.ethnicity.5levelBlack             -0.047130510 0.05684949
## Xrace.ethnicity.5levelMixed              0.055659728 0.05403863
## Xrace.ethnicity.5levelOther             -0.019971414 0.03995091
## Xrace.ethnicity.5levelWhite              0.129552136 0.07072667
## Xdemo_race_hispanic1                    -0.013623992 0.02943230
## Xinterview_age                           0.037128261 0.02565552
## XMRI_minus_hormone_date_time             0.062860944 0.02509336
## Xbmi                                     0.039553515 0.02591629
## Xhousehold.income[>=200K]               -0.156788730 0.05917034
## Xhousehold.income[100K-200K]            -0.154173834 0.08126811
## Xhousehold.income[12K-16K]              -0.012374755 0.03323112
## Xhousehold.income[16K-25K]              -0.057737369 0.03694511
## Xhousehold.income[25K-35K]              -0.008688559 0.04450012
## Xhousehold.income[35K-50K]              -0.067192607 0.04970225
## Xhousehold.income[50K-75K]              -0.098561222 0.05859534
## Xhousehold.income[5K-12K]               -0.023230082 0.03334319
## Xhousehold.income[75K-100K]             -0.105794845 0.06391911
## Xhigh.educBachelor                       0.016389741 0.07334485
## Xhigh.educHS Diploma/GED                -0.003660007 0.04396884
## Xhigh.educPost Graduate Degree           0.055583918 0.08164207
```

```
## Xhigh.educSome College                    0.059793407 0.06606495
## Xhormone_scr_ert_mean:caudate_rvsn_ant_z -0.079458951 0.05328639
```

Male participants

```
## Warning: Some predictor variables are on very different scales: consider
## rescaling
```

```
##
## Family: gaussian
## Link function: identity
##
## Formula:
## cbcl_scr_syn_internal_r ~ PDS_score + hormone_sal_end_min_since_midnight +
##     hormone_scr_ert_mean * caudate_rvsn_ant_z + race.ethnicity.5level +
##     demo_race_hispanic + interview_age + MRI_minus_hormone_date_time +
##     bmi + household.income + high.educ
##
## Parametric coefficients:
##                                           Estimate Std. Error t value Pr(>|t|)
## (Intercept)                              2.530e+00  2.697e+00   0.938  0.34835
## PDS_score                                7.807e-01  2.836e-01   2.752  0.00598
## hormone_sal_end_min_since_midnight       7.234e-04  8.125e-04   0.890  0.37338
## hormone_scr_ert_mean                     6.546e-03  9.421e-03   0.695  0.48728
## caudate_rvsn_ant_z                       2.726e-01  3.353e-01   0.813  0.41639
## race.ethnicity.5levelBlack               6.818e-02  1.132e+00   0.060  0.95199
## race.ethnicity.5levelMixed               1.301e+00  1.103e+00   1.180  0.23825
## race.ethnicity.5levelOther               4.384e-01  1.239e+00   0.354  0.72360
## race.ethnicity.5levelWhite               1.441e+00  1.041e+00   1.383  0.16676
## demo_race_hispanic1                      8.548e-02  4.277e-01   0.200  0.84161
## interview_age                            5.520e-03  1.768e-02   0.312  0.75489
## MRI_minus_hormone_date_time              1.991e-05  1.827e-05   1.089  0.27618
## bmi                                      6.543e-03  3.816e-02   0.171  0.86389
## household.income[>=200K]                -2.904e+00  1.022e+00  -2.843  0.00453
## household.income[100K-200K]             -2.398e+00  9.648e-01  -2.485  0.01304
## household.income[12K-16K]                4.000e-02  1.262e+00   0.032  0.97471
## household.income[16K-25K]                6.496e-01  1.062e+00   0.612  0.54090
## household.income[25K-35K]               -5.434e-01  1.048e+00  -0.519  0.60409
## household.income[35K-50K]               -5.025e-01  1.016e+00  -0.495  0.62087
## household.income[50K-75K]               -1.779e+00  9.617e-01  -1.850  0.06450
## household.income[5K-12K]                 6.561e-01  1.115e+00   0.588  0.55637
## household.income[75K-100K]              -2.425e+00  9.814e-01  -2.471  0.01359
## high.educBachelor                        1.101e+00  9.674e-01   1.138  0.25543
## high.educHS Diploma/GED                 -1.185e+00  9.967e-01  -1.189  0.23476
## high.educPost Graduate Degree            2.577e-01  9.711e-01   0.265  0.79080
## high.educSome College                    6.339e-01  9.251e-01   0.685  0.49335
## hormone_scr_ert_mean:caudate_rvsn_ant_z -5.733e-03  9.681e-03  -0.592  0.55379
##
## (Intercept)
## PDS_score                               **
## hormone_sal_end_min_since_midnight
## hormone_scr_ert_mean
## caudate_rvsn_ant_z
```

```
## race.ethnicity.5levelBlack
## race.ethnicity.5levelMixed
## race.ethnicity.5levelOther
## race.ethnicity.5levelWhite
## demo_race_hispanic1
## interview_age
## MRI_minus_hormone_date_time
## bmi
## household.income[>=200K]                **
## household.income[100K-200K]             *
## household.income[12K-16K]
## household.income[16K-25K]
## household.income[25K-35K]
## household.income[35K-50K]
## household.income[50K-75K]               .
## household.income[5K-12K]
## household.income[75K-100K]              *
## high.educBachelor
## high.educHS Diploma/GED
## high.educPost Graduate Degree
## high.educSome College
## hormone_scr_ert_mean:caudate_rvsn_ant_z
## ---
## Signif. codes:  0 '***' 0.001 '**' 0.01 '*' 0.05 '.' 0.1 ' ' 1
##
##
## R-sq.(adj) =  0.0366
## lmer.REML =  10442  Scale est. = 13.537    n = 1679
```

```
##                                               stdcoef      stdse
## X(Intercept)                              0.0000000000 0.00000000
## XPDS_score                                0.0704299644 0.02558857
## Xhormone_sal_end_min_since_midnight       0.0232562277 0.02611879
## Xhormone_scr_ert_mean                     0.0173835179 0.02501940
## Xcaudate_rvsn_ant_z                       0.0444867628 0.05472613
## Xrace.ethnicity.5levelBlack               0.0037404396 0.06211397
## Xrace.ethnicity.5levelMixed               0.0758623810 0.06430135
## Xrace.ethnicity.5levelOther               0.0169095756 0.04780580
## Xrace.ethnicity.5levelWhite               0.1174457128 0.08490185
## Xdemo_race_hispanic1                      0.0059619618 0.02982979
## Xinterview_age                            0.0075844415 0.02428937
## XMRI_minus_hormone_date_time              0.0271249056 0.02490126
## Xbmi                                      0.0043397287 0.02531205
## Xhousehold.income[>=200K]                -0.1777161616 0.06251968
## Xhousehold.income[100K-200K]             -0.2040275017 0.08209479
## Xhousehold.income[12K-16K]                0.0009994828 0.03152755
## Xhousehold.income[16K-25K]                0.0233721478 0.03821576
## Xhousehold.income[25K-35K]               -0.0210816297 0.04064873
## Xhousehold.income[35K-50K]               -0.0228474132 0.04618342
## Xhousehold.income[50K-75K]               -0.1114931644 0.06026783
## Xhousehold.income[5K-12K]                 0.0200736746 0.03411816
## Xhousehold.income[75K-100K]              -0.1566335882 0.06340061
## Xhigh.educBachelor                        0.0885912758 0.07787159
## Xhigh.educHS Diploma/GED                 -0.0499924125 0.04205908
```

```
## Xhigh.educPost Graduate Degree           0.0227275673 0.08566148
## Xhigh.educSome College                   0.0485194919 0.07081591
## Xhormone_scr_ert_mean:caudate_rvsn_ant_z -0.0322742551 0.05449846
```

4.16 Model: CBCL internalizing factor ~ Testosterone x Putamen activity (anticipation stage) + PDS

Female participants

```
## Warning: Some predictor variables are on very different scales: consider
## rescaling
```

```
##
## Family: gaussian
## Link function: identity
##
## Formula:
## cbcl_scr_syn_internal_r ~ PDS_score + hormone_sal_end_min_since_midnight +
##     hormone_scr_ert_mean * putamen_rvsn_ant_z + race.ethnicity.5level +
##     demo_race_hispanic + interview_age + MRI_minus_hormone_date_time +
##     bmi + household.income + high.educ
##
## Parametric coefficients:
##                                           Estimate Std. Error t value Pr(>|t|)
## (Intercept)                             -7.804e-01  2.617e+00  -0.298   0.7656
## PDS_score                                8.319e-01  2.058e-01   4.042 5.54e-05
## hormone_sal_end_min_since_midnight       8.783e-05  7.995e-04   0.110   0.9125
## hormone_scr_ert_mean                    -3.805e-03  8.236e-03  -0.462   0.6441
## putamen_rvsn_ant_z                       4.369e-01  3.285e-01   1.330   0.1836
## race.ethnicity.5levelBlack              -7.620e-01  9.070e-01  -0.840   0.4010
## race.ethnicity.5levelMixed               9.132e-01  8.718e-01   1.048   0.2950
## race.ethnicity.5levelOther              -5.566e-01  1.055e+00  -0.527   0.5980
## race.ethnicity.5levelWhite               1.491e+00  8.063e-01   1.849   0.0647
## demo_race_hispanic1                     -1.718e-01  4.049e-01  -0.424   0.6714
## interview_age                            2.640e-02  1.824e-02   1.448   0.1479
## MRI_minus_hormone_date_time              4.011e-05  1.643e-05   2.442   0.0147
## bmi                                      5.184e-02  3.681e-02   1.408   0.1592
## household.income[>=200K]                -2.472e+00  9.995e-01  -2.473   0.0135
## household.income[100K-200K]             -1.563e+00  9.298e-01  -1.681   0.0929
## household.income[12K-16K]               -2.943e-01  1.162e+00  -0.253   0.8001
## household.income[16K-25K]               -1.397e+00  1.070e+00  -1.306   0.1918
## household.income[25K-35K]                6.128e-03  9.716e-01   0.006   0.9950
## household.income[35K-50K]               -1.034e+00  9.496e-01  -1.088   0.2766
## household.income[50K-75K]               -1.363e+00  9.351e-01  -1.458   0.1452
## household.income[5K-12K]                -5.567e-01  1.132e+00  -0.492   0.6228
## household.income[75K-100K]              -1.334e+00  9.404e-01  -1.419   0.1561
## high.educBachelor                        2.608e-01  8.755e-01   0.298   0.7658
## high.educHS Diploma/GED                 -7.912e-02  8.898e-01  -0.089   0.9292
## high.educPost Graduate Degree            6.494e-01  8.901e-01   0.730   0.4657
## high.educSome College                    7.941e-01  8.245e-01   0.963   0.3356
## hormone_scr_ert_mean:putamen_rvsn_ant_z -9.214e-03  8.488e-03  -1.085   0.2779
##
## (Intercept)
```

```
## PDS_score                              ***
## hormone_sal_end_min_since_midnight
## hormone_scr_ert_mean
## putamen_rvsn_ant_z
## race.ethnicity.5levelBlack
## race.ethnicity.5levelMixed
## race.ethnicity.5levelOther
## race.ethnicity.5levelWhite             .
## demo_race_hispanic1
## interview_age
## MRI_minus_hormone_date_time            *
## bmi
## household.income[>=200K]               *
## household.income[100K-200K]            .
## household.income[12K-16K]
## household.income[16K-25K]
## household.income[25K-35K]
## household.income[35K-50K]
## household.income[50K-75K]
## household.income[5K-12K]
## household.income[75K-100K]
## high.educBachelor
## high.educHS Diploma/GED
## high.educPost Graduate Degree
## high.educSome College
## hormone_scr_ert_mean:putamen_rvsn_ant_z
## ---
## Signif. codes:  0 '***' 0.001 '**' 0.01 '*' 0.05 '.' 0.1 ' ' 1
##
##
## R-sq.(adj) =  0.0305
## lmer.REML =  10280  Scale est. = 15.953    n = 1672
```

```
##                                             stdcoef      stdse
## X(Intercept)                            0.0000000000 0.00000000
## XPDS_score                              0.1116892733 0.02763220
## Xhormone_sal_end_min_since_midnight     0.0028744280 0.02616436
## Xhormone_scr_ert_mean                  -0.0119735534 0.02591645
## Xputamen_rvsn_ant_z                     0.0706986270 0.05314624
## Xrace.ethnicity.5levelBlack            -0.0476385230 0.05670529
## Xrace.ethnicity.5levelMixed             0.0565180228 0.05395366
## Xrace.ethnicity.5levelOther            -0.0211859141 0.04016680
## Xrace.ethnicity.5levelWhite             0.1307188423 0.07070305
## Xdemo_race_hispanic1                   -0.0125054555 0.02946845
## Xinterview_age                          0.0371558885 0.02566488
## XMRI_minus_hormone_date_time            0.0612620991 0.02508928
## Xbmi                                    0.0365728336 0.02596995
## Xhousehold.income[>=200K]              -0.1477967340 0.05975650
## Xhousehold.income[100K-200K]           -0.1380968182 0.08214396
## Xhousehold.income[12K-16K]             -0.0085354385 0.03370205
## Xhousehold.income[16K-25K]             -0.0483468536 0.03702476
## Xhousehold.income[25K-35K]              0.0002824595 0.04478637
## Xhousehold.income[35K-50K]             -0.0542033312 0.04979990
## Xhousehold.income[50K-75K]             -0.0863936015 0.05927286
```

```
## Xhousehold.income[5K-12K]                -0.0165255334 0.03359327
## Xhousehold.income[75K-100K]              -0.0916478869 0.06459562
## Xhigh.educBachelor                        0.0217480834 0.07301279
## Xhigh.educHS Diploma/GED                 -0.0038878874 0.04372003
## Xhigh.educPost Graduate Degree            0.0592963372 0.08127352
## Xhigh.educSome College                    0.0632509786 0.06567096
## Xhormone_scr_ert_mean:putamen_rvsn_ant_z -0.0576043126 0.05306916
```

Male participants

```
## Warning: Some predictor variables are on very different scales: consider
## rescaling
```

```
##
## Family: gaussian
## Link function: identity
##
## Formula:
## cbcl_scr_syn_internal_r ~ PDS_score + hormone_sal_end_min_since_midnight +
##     hormone_scr_ert_mean * putamen_rvsn_ant_z + race.ethnicity.5level +
##     demo_race_hispanic + interview_age + MRI_minus_hormone_date_time +
##     bmi + household.income + high.educ
##
## Parametric coefficients:
##                                           Estimate Std. Error t value Pr(>|t|)
## (Intercept)                              2.722e+00  2.702e+00   1.007  0.31399
## PDS_score                                8.062e-01  2.852e-01   2.827  0.00475
## hormone_sal_end_min_since_midnight       7.355e-04  8.151e-04   0.902  0.36705
## hormone_scr_ert_mean                     5.866e-03  9.413e-03   0.623  0.53325
## putamen_rvsn_ant_z                      -8.842e-02  3.461e-01  -0.255  0.79839
## race.ethnicity.5levelBlack               1.287e-01  1.135e+00   0.113  0.90970
## race.ethnicity.5levelMixed               1.267e+00  1.106e+00   1.146  0.25190
## race.ethnicity.5levelOther               4.548e-01  1.242e+00   0.366  0.71432
## race.ethnicity.5levelWhite               1.409e+00  1.044e+00   1.350  0.17733
## demo_race_hispanic1                      6.872e-02  4.266e-01   0.161  0.87204
## interview_age                            3.325e-03  1.773e-02   0.188  0.85122
## MRI_minus_hormone_date_time              2.232e-05  1.835e-05   1.216  0.22412
## bmi                                      5.396e-03  3.830e-02   0.141  0.88797
## household.income[>=200K]                -2.925e+00  1.021e+00  -2.863  0.00424
## household.income[100K-200K]             -2.474e+00  9.644e-01  -2.566  0.01039
## household.income[12K-16K]               -2.243e-01  1.253e+00  -0.179  0.85800
## household.income[16K-25K]                5.445e-01  1.060e+00   0.514  0.60751
## household.income[25K-35K]               -6.367e-01  1.046e+00  -0.609  0.54285
## household.income[35K-50K]               -5.702e-01  1.017e+00  -0.561  0.57495
## household.income[50K-75K]               -1.835e+00  9.615e-01  -1.908  0.05654
## household.income[5K-12K]                 3.469e-01  1.107e+00   0.313  0.75404
## household.income[75K-100K]              -2.464e+00  9.822e-01  -2.509  0.01220
## high.educBachelor                        1.259e+00  9.611e-01   1.310  0.19031
## high.educHS Diploma/GED                 -1.088e+00  9.906e-01  -1.099  0.27208
## high.educPost Graduate Degree            4.203e-01  9.650e-01   0.436  0.66322
## high.educSome College                    7.565e-01  9.173e-01   0.825  0.40962
## hormone_scr_ert_mean:putamen_rvsn_ant_z  3.176e-04  1.009e-02   0.031  0.97489
##
```

```
## (Intercept)
## PDS_score                                **
## hormone_sal_end_min_since_midnight
## hormone_scr_ert_mean
## putamen_rvsn_ant_z
## race.ethnicity.5levelBlack
## race.ethnicity.5levelMixed
## race.ethnicity.5levelOther
## race.ethnicity.5levelWhite
## demo_race_hispanic1
## interview_age
## MRI_minus_hormone_date_time
## bmi
## household.income[>=200K]                 **
## household.income[100K-200K]              *
## household.income[12K-16K]
## household.income[16K-25K]
## household.income[25K-35K]
## household.income[35K-50K]
## household.income[50K-75K]                .
## household.income[5K-12K]
## household.income[75K-100K]               *
## high.educBachelor
## high.educHS Diploma/GED
## high.educPost Graduate Degree
## high.educSome College
## hormone_scr_ert_mean:putamen_rvsn_ant_z
## ---
## Signif. codes:  0 '***' 0.001 '**' 0.01 '*' 0.05 '.' 0.1 ' ' 1
##
##
## R-sq.(adj) =  0.0345
## lmer.REML =  10455  Scale est. = 13.714    n = 1680
```

```
##                                              stdcoef      stdse
## X(Intercept)                             0.000000000 0.00000000
## XPDS_score                               0.072478644 0.02563621
## Xhormone_sal_end_min_since_midnight      0.023591496 0.02614688
## Xhormone_scr_ert_mean                    0.015596864 0.02502768
## Xputamen_rvsn_ant_z                     -0.014357397 0.05619919
## Xrace.ethnicity.5levelBlack              0.007057386 0.06221452
## Xrace.ethnicity.5levelMixed              0.073845438 0.06442873
## Xrace.ethnicity.5levelOther              0.017528207 0.04787541
## Xrace.ethnicity.5levelWhite              0.114815711 0.08507498
## Xdemo_race_hispanic1                     0.004802021 0.02980957
## Xinterview_age                           0.004562845 0.02432359
## XMRI_minus_hormone_date_time             0.030296006 0.02491218
## Xbmi                                     0.003569931 0.02533859
## Xhousehold.income[>=200K]               -0.178870890 0.06246895
## Xhousehold.income[100K-200K]            -0.210318131 0.08197442
## Xhousehold.income[12K-16K]              -0.005682002 0.03175282
## Xhousehold.income[16K-25K]               0.019709279 0.03836486
## Xhousehold.income[25K-35K]              -0.024824695 0.04078759
## Xhousehold.income[35K-50K]              -0.025903880 0.04618425
```

```
## Xhousehold.income[50K-75K]             -0.114909667 0.06021907
## Xhousehold.income[5K-12K]               0.010809443 0.03449472
## Xhousehold.income[75K-100K]            -0.158313587 0.06309977
## Xhigh.educBachelor                      0.101164659 0.07721316
## Xhigh.educHS Diploma/GED               -0.046112935 0.04197193
## Xhigh.educPost Graduate Degree          0.037027516 0.08501323
## Xhigh.educSome College                  0.058071036 0.07040864
## Xhormone_scr_ert_mean:putamen_rvsn_ant_z  0.001759602 0.05590481
```

4.17 Model: CBCL internalizing factor ~ Testosterone x Accumbens activity (feedback stage) + PDS

Female participants

```
## Warning: Some predictor variables are on very different scales: consider
## rescaling
```

```
##
## Family: gaussian
## Link function: identity
##
## Formula:
## cbcl_scr_syn_internal_r ~ PDS_score + hormone_sal_end_min_since_midnight +
##     hormone_scr_ert_mean * accumbens_posvsneg_feedback_z + race.ethnicity.5level +
##     demo_race_hispanic + interview_age + MRI_minus_hormone_date_time +
##     bmi + household.income + high.educ
##
## Parametric coefficients:
##                                                    Estimate Std. Error
## (Intercept)                                      -7.949e-01  2.625e+00
## PDS_score                                         8.086e-01  2.071e-01
## hormone_sal_end_min_since_midnight                9.388e-05  8.022e-04
## hormone_scr_ert_mean                             -3.682e-03  8.265e-03
## accumbens_posvsneg_feedback_z                     2.404e-01  4.472e-01
## race.ethnicity.5levelBlack                       -6.950e-01  9.106e-01
## race.ethnicity.5levelMixed                        9.445e-01  8.732e-01
## race.ethnicity.5levelOther                       -4.542e-01  1.054e+00
## race.ethnicity.5levelWhite                        1.482e+00  8.069e-01
## demo_race_hispanic1                              -2.111e-01  4.092e-01
## interview_age                                     2.648e-02  1.830e-02
## MRI_minus_hormone_date_time                       3.967e-05  1.643e-05
## bmi                                               5.208e-02  3.679e-02
## household.income[>=200K]                         -2.401e+00  1.001e+00
## household.income[100K-200K]                      -1.445e+00  9.304e-01
## household.income[12K-16K]                        -2.560e-01  1.162e+00
## household.income[16K-25K]                       -1.414e+00  1.069e+00
## household.income[25K-35K]                        7.553e-02  9.706e-01
## household.income[35K-50K]                       -9.574e-01  9.511e-01
## household.income[50K-75K]                       -1.244e+00  9.377e-01
## household.income[5K-12K]                        -6.201e-01  1.139e+00
## household.income[75K-100K]                      -1.218e+00  9.430e-01
## high.educBachelor                                 1.842e-01  8.873e-01
## high.educHS Diploma/GED                          -9.178e-02  8.989e-01
```

```
## high.educPost Graduate Degree                     5.942e-01  9.024e-01
## high.educSome College                             7.220e-01  8.347e-01
## hormone_scr_ert_mean:accumbens_posvsneg_feedback_z -4.005e-04  1.121e-02
##                                                   t value Pr(>|t|)
## (Intercept)                                        -0.303   0.7621
## PDS_score                                           3.905  9.8e-05 ***
## hormone_sal_end_min_since_midnight                  0.117   0.9068
## hormone_scr_ert_mean                               -0.446   0.6560
## accumbens_posvsneg_feedback_z                       0.538   0.5909
## race.ethnicity.5levelBlack                         -0.763   0.4454
## race.ethnicity.5levelMixed                          1.082   0.2796
## race.ethnicity.5levelOther                         -0.431   0.6665
## race.ethnicity.5levelWhite                          1.837   0.0664 .
## demo_race_hispanic1                                -0.516   0.6060
## interview_age                                       1.447   0.1480
## MRI_minus_hormone_date_time                         2.415   0.0159 *
## bmi                                                 1.415   0.1571
## household.income[>=200K]                           -2.400   0.0165 *
## household.income[100K-200K]                        -1.553   0.1207
## household.income[12K-16K]                          -0.220   0.8257
## household.income[16K-25K]                          -1.323   0.1860
## household.income[25K-35K]                           0.078   0.9380
## household.income[35K-50K]                          -1.007   0.3143
## household.income[50K-75K]                          -1.326   0.1849
## household.income[5K-12K]                           -0.544   0.5862
## household.income[75K-100K]                         -1.292   0.1965
## high.educBachelor                                   0.208   0.8356
## high.educHS Diploma/GED                            -0.102   0.9187
## high.educPost Graduate Degree                       0.659   0.5103
## high.educSome College                               0.865   0.3872
## hormone_scr_ert_mean:accumbens_posvsneg_feedback_z  -0.036   0.9715
## ---
## Signif. codes:  0 '***' 0.001 '**' 0.01 '*' 0.05 '.' 0.1 ' ' 1
##
##
## R-sq.(adj) =  0.0284
## lmer.REML =  10256  Scale est. = 15.925    n = 1668
```

```
##                                                         stdcoef      stdse
## X(Intercept)                                        0.000000000 0.00000000
## XPDS_score                                          0.108124221 0.02768792
## Xhormone_sal_end_min_since_midnight                 0.003071502 0.02624395
## Xhormone_scr_ert_mean                              -0.011591195 0.02601708
## Xaccumbens_posvsneg_feedback_z                      0.030805545 0.05730303
## Xrace.ethnicity.5levelBlack                        -0.043253988 0.05666949
## Xrace.ethnicity.5levelMixed                         0.058292677 0.05389254
## Xrace.ethnicity.5levelOther                        -0.017430997 0.04044054
## Xrace.ethnicity.5levelWhite                         0.129847516 0.07069123
## Xdemo_race_hispanic1                               -0.015345338 0.02974920
## Xinterview_age                                      0.037247389 0.02573694
## XMRI_minus_hormone_date_time                        0.060749051 0.02516001
## Xbmi                                                0.036836014 0.02602454
## Xhousehold.income[>=200K]                          -0.143765115 0.05990502
## Xhousehold.income[100K-200K]                       -0.127702405 0.08223726
```

```
## Xhousehold.income[12K-16K]                            -0.007435584 0.03376514
## Xhousehold.income[16K-25K]                            -0.049400563 0.03733671
## Xhousehold.income[25K-35K]                             0.003501769 0.04500085
## Xhousehold.income[35K-50K]                            -0.050281075 0.04994949
## Xhousehold.income[50K-75K]                            -0.078334681 0.05905846
## Xhousehold.income[5K-12K]                             -0.018225624 0.03347515
## Xhousehold.income[75K-100K]                           -0.083804874 0.06486592
## Xhigh.educBachelor                                     0.015400315 0.07418714
## Xhigh.educHS Diploma/GED                              -0.004499576 0.04407105
## Xhigh.educPost Graduate Degree                         0.054303132 0.08246194
## Xhigh.educSome College                                 0.057376268 0.06633482
## Xhormone_scr_ert_mean:accumbens_posvsneg_feedback_z -0.002037224 0.05704060
```

Male participants

```
## Warning: Some predictor variables are on very different scales: consider
## rescaling
```

```
##
## Family: gaussian
## Link function: identity
##
## Formula:
## cbcl_scr_syn_internal_r ~ PDS_score + hormone_sal_end_min_since_midnight +
##     hormone_scr_ert_mean * accumbens_posvsneg_feedback_z + race.ethnicity.5level +
##     demo_race_hispanic + interview_age + MRI_minus_hormone_date_time +
##     bmi + household.income + high.educ
##
## Parametric coefficients:
##                                                        Estimate Std. Error
## (Intercept)                                           3.184e+00  2.716e+00
## PDS_score                                             7.978e-01  2.821e-01
## hormone_sal_end_min_since_midnight                    6.255e-04  8.134e-04
## hormone_scr_ert_mean                                  3.908e-03  9.425e-03
## accumbens_posvsneg_feedback_z                        -2.177e-01  4.183e-01
## race.ethnicity.5levelBlack                           -4.490e-02  1.150e+00
## race.ethnicity.5levelMixed                            1.255e+00  1.120e+00
## race.ethnicity.5levelOther                            3.590e-01  1.254e+00
## race.ethnicity.5levelWhite                            1.420e+00  1.060e+00
## demo_race_hispanic1                                   7.689e-02  4.239e-01
## interview_age                                         1.170e-03  1.767e-02
## MRI_minus_hormone_date_time                           2.027e-05  1.876e-05
## bmi                                                   2.701e-02  3.819e-02
## household.income[>=200K]                             -3.333e+00  1.043e+00
## household.income[100K-200K]                          -2.967e+00  9.863e-01
## household.income[12K-16K]                            -6.930e-01  1.258e+00
## household.income[16K-25K]                             1.190e-01  1.081e+00
## household.income[25K-35K]                            -1.024e+00  1.068e+00
## household.income[35K-50K]                            -9.366e-01  1.036e+00
## household.income[50K-75K]                            -2.304e+00  9.844e-01
## household.income[5K-12K]                              3.558e-02  1.141e+00
## household.income[75K-100K]                           -2.955e+00  1.005e+00
## high.educBachelor                                     1.282e+00  9.661e-01
```

```
## high.educHS Diploma/GED                         -1.075e+00  9.964e-01
## high.educPost Graduate Degree                     4.644e-01  9.690e-01
## high.educSome College                             8.078e-01  9.222e-01
## hormone_scr_ert_mean:accumbens_posvsneg_feedback_z -7.150e-04  1.240e-02
##                                                  t value Pr(>|t|)
## (Intercept)                                        1.172  0.24127
## PDS_score                                          2.828  0.00475 **
## hormone_sal_end_min_since_midnight                 0.769  0.44206
## hormone_scr_ert_mean                               0.415  0.67850
## accumbens_posvsneg_feedback_z                     -0.520  0.60282
## race.ethnicity.5levelBlack                        -0.039  0.96885
## race.ethnicity.5levelMixed                         1.120  0.26267
## race.ethnicity.5levelOther                         0.286  0.77474
## race.ethnicity.5levelWhite                         1.340  0.18044
## demo_race_hispanic1                                0.181  0.85611
## interview_age                                      0.066  0.94724
## MRI_minus_hormone_date_time                        1.080  0.28013
## bmi                                                0.707  0.47952
## household.income[>=200K]                          -3.196  0.00142 **
## household.income[100K-200K]                       -3.008  0.00267 **
## household.income[12K-16K]                         -0.551  0.58191
## household.income[16K-25K]                          0.110  0.91235
## household.income[25K-35K]                         -0.959  0.33777
## household.income[35K-50K]                         -0.904  0.36601
## household.income[50K-75K]                         -2.341  0.01936 *
## household.income[5K-12K]                           0.031  0.97513
## household.income[75K-100K]                        -2.940  0.00333 **
## high.educBachelor                                  1.327  0.18459
## high.educHS Diploma/GED                           -1.079  0.28075
## high.educPost Graduate Degree                      0.479  0.63177
## high.educSome College                              0.876  0.38117
## hormone_scr_ert_mean:accumbens_posvsneg_feedback_z  -0.058  0.95404
## ---
## Signif. codes:  0 '***' 0.001 '**' 0.01 '*' 0.05 '.' 0.1 ' ' 1
##
##
## R-sq.(adj) =  0.0372
## lmer.REML =  10436  Scale est. = 13.379    n = 1678
```

```
##                                                        stdcoef      stdse
## X(Intercept)                                       0.000000000 0.00000000
## XPDS_score                                         0.072225268 0.02554224
## Xhormone_sal_end_min_since_midnight                0.020062230 0.02609202
## Xhormone_scr_ert_mean                              0.010380629 0.02503868
## Xaccumbens_posvsneg_feedback_z                    -0.028720748 0.05518435
## Xrace.ethnicity.5levelBlack                       -0.002449120 0.06270211
## Xrace.ethnicity.5levelMixed                        0.072994861 0.06514578
## Xrace.ethnicity.5levelOther                        0.013917023 0.04862301
## Xrace.ethnicity.5levelWhite                        0.115499936 0.08619632
## Xdemo_race_hispanic1                               0.005385960 0.02969832
## Xinterview_age                                     0.001604375 0.02424158
## XMRI_minus_hormone_date_time                       0.026852995 0.02485526
## Xbmi                                               0.017802167 0.02517135
## Xhousehold.income[>=200K]                         -0.203830778 0.06377801
```

```
## Xhousehold.income[100K-200K]                          -0.252350144 0.08389365
## Xhousehold.income[12K-16K]                            -0.017809719 0.03234002
## Xhousehold.income[16K-25K]                             0.004306450 0.03911837
## Xhousehold.income[25K-35K]                            -0.039706958 0.04141033
## Xhousehold.income[35K-50K]                            -0.042895933 0.04743985
## Xhousehold.income[50K-75K]                            -0.144057837 0.06154357
## Xhousehold.income[5K-12K]                              0.001077386 0.03455824
## Xhousehold.income[75K-100K]                           -0.190134030 0.06467996
## Xhigh.educBachelor                                     0.102732306 0.07739963
## Xhigh.educHS Diploma/GED                              -0.045555718 0.04222053
## Xhigh.educPost Graduate Degree                         0.040946224 0.08542421
## Xhigh.educSome College                                 0.061954031 0.07072573
## Xhormone_scr_ert_mean:accumbens_posvsneg_feedback_z -0.003201184 0.05553586
```

4.18 Model: CBCL internalizing factor ~ Testosterone x Caudate activity (Feedback stage) + PDS

Female participants

```
## Warning: Some predictor variables are on very different scales: consider
## rescaling
```

```
##
## Family: gaussian
## Link function: identity
##
## Formula:
## cbcl_scr_syn_internal_r ~ PDS_score + hormone_sal_end_min_since_midnight +
##     hormone_scr_ert_mean * caudate_posvsneg_feedback_z + race.ethnicity.5level +
##     demo_race_hispanic + interview_age + MRI_minus_hormone_date_time +
##     bmi + household.income + high.educ
##
## Parametric coefficients:
##                                                  Estimate Std. Error t value
## (Intercept)                                    -5.841e-01  2.615e+00  -0.223
## PDS_score                                       8.067e-01  2.058e-01   3.919
## hormone_sal_end_min_since_midnight             -2.040e-05  8.007e-04  -0.025
## hormone_scr_ert_mean                           -2.745e-03  8.232e-03  -0.333
## caudate_posvsneg_feedback_z                    -1.008e-01  3.496e-01  -0.288
## race.ethnicity.5levelBlack                     -8.449e-01  9.111e-01  -0.927
## race.ethnicity.5levelMixed                      8.695e-01  8.737e-01   0.995
## race.ethnicity.5levelOther                     -5.496e-01  1.053e+00  -0.522
## race.ethnicity.5levelWhite                      1.468e+00  8.079e-01   1.817
## demo_race_hispanic1                            -1.677e-01  4.058e-01  -0.413
## interview_age                                   2.722e-02  1.828e-02   1.489
## MRI_minus_hormone_date_time                     4.026e-05  1.642e-05   2.451
## bmi                                             5.339e-02  3.676e-02   1.452
## household.income[>=200K]                       -2.613e+00  9.933e-01  -2.630
## household.income[100K-200K]                    -1.747e+00  9.219e-01  -1.894
## household.income[12K-16K]                      -5.951e-01  1.154e+00  -0.516
## household.income[16K-25K]                      -1.659e+00  1.064e+00  -1.559
## household.income[25K-35K]                      -2.102e-01  9.625e-01  -0.218
## household.income[35K-50K]                     -1.273e+00  9.431e-01  -1.349
```

```
## household.income[50K-75K]                          -1.560e+00  9.273e-01  -1.682
## household.income[5K-12K]                           -7.702e-01  1.125e+00  -0.685
## household.income[75K-100K]                         -1.521e+00  9.322e-01  -1.632
## high.educBachelor                                   2.517e-01  8.831e-01   0.285
## high.educHS Diploma/GED                             4.783e-02  8.967e-01   0.053
## high.educPost Graduate Degree                       6.008e-01  8.976e-01   0.669
## high.educSome College                               8.141e-01  8.315e-01   0.979
## hormone_scr_ert_mean:caudate_posvsneg_feedback_z  8.726e-04  9.032e-03   0.097
##                                                    Pr(>|t|)
## (Intercept)                                         0.82329
## PDS_score                                          9.24e-05 ***
## hormone_sal_end_min_since_midnight                  0.97967
## hormone_scr_ert_mean                                0.73884
## caudate_posvsneg_feedback_z                         0.77321
## race.ethnicity.5levelBlack                          0.35389
## race.ethnicity.5levelMixed                          0.31978
## race.ethnicity.5levelOther                          0.60183
## race.ethnicity.5levelWhite                          0.06944 .
## demo_race_hispanic1                                 0.67949
## interview_age                                       0.13666
## MRI_minus_hormone_date_time                         0.01435 *
## bmi                                                 0.14658
## household.income[>=200K]                            0.00862 **
## household.income[100K-200K]                         0.05834 .
## household.income[12K-16K]                           0.60613
## household.income[16K-25K]                           0.11913
## household.income[25K-35K]                           0.82713
## household.income[35K-50K]                           0.17739
## household.income[50K-75K]                           0.09267 .
## household.income[5K-12K]                            0.49370
## household.income[75K-100K]                          0.10288
## high.educBachelor                                   0.77564
## high.educHS Diploma/GED                             0.95746
## high.educPost Graduate Degree                       0.50334
## high.educSome College                               0.32772
## hormone_scr_ert_mean:caudate_posvsneg_feedback_z  0.92305
## ---
## Signif. codes:  0 '***' 0.001 '**' 0.01 '*' 0.05 '.' 0.1 ' ' 1
##
##
## R-sq.(adj) =  0.029
## lmer.REML =  10268  Scale est. = 16.02     n = 1670
```

```
##                                                          stdcoef      stdse
## X(Intercept)                                        0.0000000000 0.00000000
## XPDS_score                                          0.1082912132 0.02763003
## Xhormone_sal_end_min_since_midnight                -0.0006679496 0.02621354
## Xhormone_scr_ert_mean                              -0.0086582437 0.02596560
## Xcaudate_posvsneg_feedback_z                       -0.0161919606 0.05617694
## Xrace.ethnicity.5levelBlack                        -0.0526499156 0.05677590
## Xrace.ethnicity.5levelMixed                         0.0539640395 0.05422408
## Xrace.ethnicity.5levelOther                        -0.0210756270 0.04038483
## Xrace.ethnicity.5levelWhite                         0.1287721020 0.07088108
## Xdemo_race_hispanic1                               -0.0122285144 0.02959242
```

```
## Xinterview_age                                        0.0382850335 0.02571057
## XMRI_minus_hormone_date_time                          0.0616199508 0.02514172
## Xbmi                                                  0.0378309070 0.02604700
## Xhousehold.income[>=200K]                            -0.1555979077 0.05916089
## Xhousehold.income[100K-200K]                         -0.1542441311 0.08141814
## Xhousehold.income[12K-16K]                           -0.0172744242 0.03349709
## Xhousehold.income[16K-25K]                           -0.0574574818 0.03684921
## Xhousehold.income[25K-35K]                           -0.0097398433 0.04459274
## Xhousehold.income[35K-50K]                           -0.0667890523 0.04949474
## Xhousehold.income[50K-75K]                           -0.0985869774 0.05859738
## Xhousehold.income[5K-12K]                            -0.0228804490 0.03342235
## Xhousehold.income[75K-100K]                          -0.1048883246 0.06427212
## Xhigh.educBachelor                                    0.0209760818 0.07358843
## Xhigh.educHS Diploma/GED                              0.0023520854 0.04409317
## Xhigh.educPost Graduate Degree                        0.0548830546 0.08199029
## Xhigh.educSome College                                0.0649413443 0.06633445
## Xhormone_scr_ert_mean:caudate_posvsneg_feedback_z  0.0054254362 0.05615769
```

Male participants

```
## Warning: Some predictor variables are on very different scales: consider
## rescaling
```

```
##
## Family: gaussian
## Link function: identity
##
## Formula:
## cbcl_scr_syn_internal_r ~ PDS_score + hormone_sal_end_min_since_midnight +
##     hormone_scr_ert_mean * caudate_posvsneg_feedback_z + race.ethnicity.5level +
##     demo_race_hispanic + interview_age + MRI_minus_hormone_date_time +
##     bmi + household.income + high.educ
##
## Parametric coefficients:
##                                                   Estimate Std. Error t value
## (Intercept)                                      2.974e+00  2.708e+00   1.098
## PDS_score                                        7.730e-01  2.815e-01   2.746
## hormone_sal_end_min_since_midnight               9.463e-04  8.106e-04   1.168
## hormone_scr_ert_mean                             5.350e-03  9.411e-03   0.569
## caudate_posvsneg_feedback_z                     -1.387e-01  3.377e-01  -0.411
## race.ethnicity.5levelBlack                       1.362e-02  1.140e+00   0.012
## race.ethnicity.5levelMixed                       1.204e+00  1.109e+00   1.086
## race.ethnicity.5levelOther                       3.628e-01  1.245e+00   0.291
## race.ethnicity.5levelWhite                       1.325e+00  1.048e+00   1.265
## demo_race_hispanic1                              6.919e-02  4.239e-01   0.163
## interview_age                                    1.577e-03  1.770e-02   0.089
## MRI_minus_hormone_date_time                      2.008e-05  1.824e-05   1.101
## bmi                                              8.278e-03  3.804e-02   0.218
## household.income[>=200K]                        -2.967e+00  1.027e+00  -2.889
## household.income[100K-200K]                     -2.557e+00  9.706e-01  -2.634
## household.income[12K-16K]                       -3.674e-01  1.249e+00  -0.294
## household.income[16K-25K]                        4.323e-01  1.069e+00   0.404
## household.income[25K-35K]                       -6.437e-01  1.053e+00  -0.611
```

```
## household.income[35K-50K]                        -5.709e-01  1.021e+00  -0.559
## household.income[50K-75K]                        -1.929e+00  9.682e-01  -1.993
## household.income[5K-12K]                          6.161e-02  1.125e+00   0.055
## household.income[75K-100K]                      -2.565e+00  9.883e-01  -2.595
## high.educBachelor                                 1.232e+00  9.676e-01   1.273
## high.educHS Diploma/GED                          -1.075e+00  1.001e+00  -1.073
## high.educPost Graduate Degree                     3.758e-01  9.705e-01   0.387
## high.educSome College                             7.203e-01  9.228e-01   0.781
## hormone_scr_ert_mean:caudate_posvsneg_feedback_z  1.835e-03  9.812e-03   0.187
##                                                  Pr(>|t|)
## (Intercept)                                       0.27220
## PDS_score                                         0.00610 **
## hormone_sal_end_min_since_midnight                0.24317
## hormone_scr_ert_mean                              0.56977
## caudate_posvsneg_feedback_z                       0.68134
## race.ethnicity.5levelBlack                        0.99047
## race.ethnicity.5levelMixed                        0.27761
## race.ethnicity.5levelOther                        0.77079
## race.ethnicity.5levelWhite                        0.20596
## demo_race_hispanic1                               0.87035
## interview_age                                     0.92901
## MRI_minus_hormone_date_time                       0.27108
## bmi                                               0.82775
## household.income[>=200K]                          0.00392 **
## household.income[100K-200K]                       0.00851 **
## household.income[12K-16K]                         0.76865
## household.income[16K-25K]                         0.68606
## household.income[25K-35K]                         0.54100
## household.income[35K-50K]                         0.57613
## household.income[50K-75K]                         0.04646 *
## household.income[5K-12K]                          0.95633
## household.income[75K-100K]                        0.00953 **
## high.educBachelor                                 0.20304
## high.educHS Diploma/GED                           0.28334
## high.educPost Graduate Degree                     0.69860
## high.educSome College                             0.43516
## hormone_scr_ert_mean:caudate_posvsneg_feedback_z  0.85164
## ---
## Signif. codes:  0 '***' 0.001 '**' 0.01 '*' 0.05 '.' 0.1 ' ' 1
##
##
## R-sq.(adj) =  0.0339
## lmer.REML =  10439  Scale est. = 13.289    n = 1678
```

```
##                                                          stdcoef      stdse
## X(Intercept)                                        0.0000000000 0.00000000
## XPDS_score                                          0.0702170181 0.02556938
## Xhormone_sal_end_min_since_midnight                 0.0304057990 0.02604320
## Xhormone_scr_ert_mean                               0.0142546163 0.02507400
## Xcaudate_posvsneg_feedback_z                       -0.0223386803 0.05439057
## Xrace.ethnicity.5levelBlack                         0.0007425218 0.06212783
## Xrace.ethnicity.5levelMixed                         0.0702751088 0.06470604
## Xrace.ethnicity.5levelOther                         0.0140082283 0.04807428
## Xrace.ethnicity.5levelWhite                         0.1079060774 0.08528526
```

```
## Xdemo_race_hispanic1                                0.0048611318 0.02978015
## Xinterview_age                                      0.0021677637 0.02432838
## XMRI_minus_hormone_date_time                        0.0274166854 0.02490266
## Xbmi                                                0.0055059413 0.02530029
## Xhousehold.income[>=200K]                          -0.1824957510 0.06317812
## Xhousehold.income[100K-200K]                       -0.2176891375 0.08263178
## Xhousehold.income[12K-16K]                         -0.0094579933 0.03214916
## Xhousehold.income[16K-25K]                          0.0155711492 0.03851552
## Xhousehold.income[25K-35K]                         -0.0249980349 0.04088435
## Xhousehold.income[35K-50K]                         -0.0259859379 0.04647321
## Xhousehold.income[50K-75K]                         -0.1210456297 0.06074430
## Xhousehold.income[5K-12K]                           0.0018869622 0.03445426
## Xhousehold.income[75K-100K]                        -0.1650743753 0.06360454
## Xhigh.educBachelor                                  0.0989503040 0.07770276
## Xhigh.educHS Diploma/GED                           -0.0454014070 0.04230484
## Xhigh.educPost Graduate Degree                      0.0331759384 0.08566449
## Xhigh.educSome College                              0.0554302229 0.07101075
## Xhormone_scr_ert_mean:caudate_posvsneg_feedback_z  0.0101522435 0.05427285
```

4.19 Model: CBCL internalizing factor ~ Testosterone x Putamen activity (Feedback stage) + PDS

Female participants

```
## Warning: Some predictor variables are on very different scales: consider
## rescaling
```

```
##
## Family: gaussian
## Link function: identity
##
## Formula:
## cbcl_scr_syn_internal_r ~ PDS_score + hormone_sal_end_min_since_midnight +
##     hormone_scr_ert_mean * putamen_posvsneg_feedback_z + race.ethnicity.5level +
##     demo_race_hispanic + interview_age + MRI_minus_hormone_date_time +
##     bmi + household.income + high.educ
##
## Parametric coefficients:
##                                                   Estimate Std. Error t value
## (Intercept)                                     -7.672e-01  2.616e+00  -0.293
## PDS_score                                        8.623e-01  2.064e-01   4.178
## hormone_sal_end_min_since_midnight               1.615e-05  8.024e-04   0.020
## hormone_scr_ert_mean                            -4.140e-03  8.260e-03  -0.501
## putamen_posvsneg_feedback_z                      1.294e-01  3.542e-01   0.365
## race.ethnicity.5levelBlack                      -7.112e-01  9.080e-01  -0.783
## race.ethnicity.5levelMixed                       9.082e-01  8.718e-01   1.042
## race.ethnicity.5levelOther                      -6.296e-01  1.054e+00  -0.597
## race.ethnicity.5levelWhite                       1.503e+00  8.064e-01   1.863
## demo_race_hispanic1                             -9.560e-02  4.067e-01  -0.235
## interview_age                                    2.668e-02  1.823e-02   1.463
## MRI_minus_hormone_date_time                      4.047e-05  1.641e-05   2.466
## bmi                                              4.551e-02  3.676e-02   1.238
## household.income[>=200K]                        -2.409e+00  9.972e-01  -2.416
```

```
## household.income[100K-200K]                      -1.508e+00  9.271e-01  -1.627
## household.income[12K-16K]                        -2.799e-01  1.160e+00  -0.241
## household.income[16K-25K]                        -1.362e+00  1.066e+00  -1.278
## household.income[25K-35K]                         9.007e-02  9.684e-01   0.093
## household.income[35K-50K]                        -9.729e-01  9.485e-01  -1.026
## household.income[50K-75K]                        -1.269e+00  9.346e-01  -1.358
## household.income[5K-12K]                         -4.818e-01  1.130e+00  -0.426
## household.income[75K-100K]                       -1.301e+00  9.379e-01  -1.387
## high.educBachelor                                 2.732e-01  8.825e-01   0.310
## high.educHS Diploma/GED                          -8.317e-02  8.955e-01  -0.093
## high.educPost Graduate Degree                     6.473e-01  8.958e-01   0.723
## high.educSome College                             7.895e-01  8.304e-01   0.951
## hormone_scr_ert_mean:putamen_posvsneg_feedback_z -7.443e-03  8.916e-03  -0.835
##                                                  Pr(>|t|)
## (Intercept)                                        0.7694
## PDS_score                                        3.09e-05 ***
## hormone_sal_end_min_since_midnight                 0.9839
## hormone_scr_ert_mean                               0.6163
## putamen_posvsneg_feedback_z                        0.7150
## race.ethnicity.5levelBlack                         0.4336
## race.ethnicity.5levelMixed                         0.2977
## race.ethnicity.5levelOther                         0.5504
## race.ethnicity.5levelWhite                         0.0626 .
## demo_race_hispanic1                                0.8142
## interview_age                                      0.1436
## MRI_minus_hormone_date_time                        0.0138 *
## bmi                                                0.2158
## household.income[>=200K]                           0.0158 *
## household.income[100K-200K]                        0.1040
## household.income[12K-16K]                          0.8093
## household.income[16K-25K]                          0.2013
## household.income[25K-35K]                          0.9259
## household.income[35K-50K]                          0.3052
## household.income[50K-75K]                          0.1746
## household.income[5K-12K]                           0.6699
## household.income[75K-100K]                         0.1655
## high.educBachelor                                  0.7569
## high.educHS Diploma/GED                            0.9260
## high.educPost Graduate Degree                      0.4701
## high.educSome College                              0.3419
## hormone_scr_ert_mean:putamen_posvsneg_feedback_z   0.4040
## ---
## Signif. codes:  0 '***' 0.001 '**' 0.01 '*' 0.05 '.' 0.1 ' ' 1
##
##
## R-sq.(adj) =  0.0304
## lmer.REML =  10266  Scale est. = 16.538    n = 1670
```

```
##                                                         stdcoef      stdse
## X(Intercept)                                        0.000000000 0.00000000
## XPDS_score                                          0.115330710 0.02760209
## Xhormone_sal_end_min_since_midnight                 0.000528730 0.02626373
## Xhormone_scr_ert_mean                              -0.013037101 0.02601144
## Xputamen_posvsneg_feedback_z                        0.020517649 0.05617195
```

```
## Xrace.ethnicity.5levelBlack                       -0.044411549 0.05670048
## Xrace.ethnicity.5levelMixed                        0.056141425 0.05388927
## Xrace.ethnicity.5levelOther                       -0.023988409 0.04015863
## Xrace.ethnicity.5levelWhite                        0.131708229 0.07068338
## Xdemo_race_hispanic1                              -0.006937489 0.02951559
## Xinterview_age                                     0.037587071 0.02568545
## XMRI_minus_hormone_date_time                       0.061872798 0.02509295
## Xbmi                                               0.032166607 0.02597890
## Xhousehold.income[>=200K]                         -0.143835966 0.05953462
## Xhousehold.income[100K-200K]                      -0.133332073 0.08195622
## Xhousehold.income[12K-16K]                        -0.008125934 0.03366959
## Xhousehold.income[16K-25K]                        -0.047556563 0.03720010
## Xhousehold.income[25K-35K]                         0.004173189 0.04486651
## Xhousehold.income[35K-50K]                        -0.051067343 0.04978478
## Xhousehold.income[50K-75K]                        -0.079750317 0.05871535
## Xhousehold.income[5K-12K]                         -0.014313446 0.03357519
## Xhousehold.income[75K-100K]                       -0.089722196 0.06467499
## Xhigh.educBachelor                                 0.022768379 0.07354601
## Xhigh.educHS Diploma/GED                          -0.004090378 0.04403856
## Xhigh.educPost Graduate Degree                     0.059164610 0.08188457
## Xhigh.educSome College                             0.062877813 0.06613379
## Xhormone_scr_ert_mean:putamen_posvsneg_feedback_z -0.046887713 0.05617029
```

Male participants

```
## Warning: Some predictor variables are on very different scales: consider
## rescaling
```

```
##
## Family: gaussian
## Link function: identity
##
## Formula:
## cbcl_scr_syn_internal_r ~ PDS_score + hormone_sal_end_min_since_midnight +
##     hormone_scr_ert_mean * putamen_posvsneg_feedback_z + race.ethnicity.5level +
##     demo_race_hispanic + interview_age + MRI_minus_hormone_date_time +
##     bmi + household.income + high.educ
##
## Parametric coefficients:
##                                                     Estimate Std. Error t value
## (Intercept)                                        3.032e+00  2.720e+00   1.114
## PDS_score                                          7.563e-01  2.821e-01   2.681
## hormone_sal_end_min_since_midnight                 8.772e-04  8.102e-04   1.083
## hormone_scr_ert_mean                               5.471e-03  9.391e-03   0.583
## putamen_posvsneg_feedback_z                        1.313e-01  3.403e-01   0.386
## race.ethnicity.5levelBlack                         4.954e-02  1.141e+00   0.043
## race.ethnicity.5levelMixed                         1.257e+00  1.111e+00   1.131
## race.ethnicity.5levelOther                         4.109e-01  1.247e+00   0.329
## race.ethnicity.5levelWhite                         1.383e+00  1.049e+00   1.319
## demo_race_hispanic1                                2.583e-02  4.233e-01   0.061
## interview_age                                      2.037e-03  1.770e-02   0.115
## MRI_minus_hormone_date_time                        1.932e-05  1.826e-05   1.058
## bmi                                                1.351e-02  3.797e-02   0.356
```

```
## household.income[>=200K]                         -3.061e+00  1.034e+00  -2.959
## household.income[100K-200K]                      -2.674e+00  9.775e-01  -2.735
## household.income[12K-16K]                        -4.539e-01  1.254e+00  -0.362
## household.income[16K-25K]                         3.263e-01  1.080e+00   0.302
## household.income[25K-35K]                        -7.394e-01  1.060e+00  -0.698
## household.income[35K-50K]                        -6.608e-01  1.027e+00  -0.643
## household.income[50K-75K]                        -1.991e+00  9.753e-01  -2.041
## household.income[5K-12K]                         -6.369e-02  1.132e+00  -0.056
## household.income[75K-100K]                       -2.645e+00  9.954e-01  -2.657
## high.educBachelor                                 1.113e+00  9.740e-01   1.143
## high.educHS Diploma/GED                          -1.187e+00  1.008e+00  -1.178
## high.educPost Graduate Degree                     3.073e-01  9.768e-01   0.315
## high.educSome College                             6.404e-01  9.298e-01   0.689
## hormone_scr_ert_mean:putamen_posvsneg_feedback_z -1.569e-03  1.013e-02  -0.155
##                                                  Pr(>|t|)
## (Intercept)                                       0.26525
## PDS_score                                         0.00742 **
## hormone_sal_end_min_since_midnight                0.27908
## hormone_scr_ert_mean                              0.56029
## putamen_posvsneg_feedback_z                       0.69971
## race.ethnicity.5levelBlack                        0.96539
## race.ethnicity.5levelMixed                        0.25814
## race.ethnicity.5levelOther                        0.74190
## race.ethnicity.5levelWhite                        0.18743
## demo_race_hispanic1                               0.95135
## interview_age                                     0.90837
## MRI_minus_hormone_date_time                       0.29025
## bmi                                               0.72197
## household.income[>=200K]                          0.00313 **
## household.income[100K-200K]                       0.00630 **
## household.income[12K-16K]                         0.71746
## household.income[16K-25K]                         0.76251
## household.income[25K-35K]                         0.48540
## household.income[35K-50K]                        0.52019
## household.income[50K-75K]                         0.04137 *
## household.income[5K-12K]                          0.95515
## household.income[75K-100K]                        0.00796 **
## high.educBachelor                                 0.25337
## high.educHS Diploma/GED                           0.23915
## high.educPost Graduate Degree                     0.75306
## high.educSome College                             0.49109
## hormone_scr_ert_mean:putamen_posvsneg_feedback_z  0.87697
## ---
## Signif. codes:  0 '***' 0.001 '**' 0.01 '*' 0.05 '.' 0.1 ' ' 1
##
##
## R-sq.(adj) =  0.0333
## lmer.REML =  10467  Scale est. = 13.594    n = 1682
```

```
##                                                       stdcoef      stdse
## X(Intercept)                                      0.000000000 0.00000000
## XPDS_score                                        0.068534018 0.02556388
## Xhormone_sal_end_min_since_midnight               0.028182902 0.02602935
## Xhormone_scr_ert_mean                             0.014567676 0.02500765
```

```
## Xputamen_posvsneg_feedback_z                            0.020857539 0.05406622
## Xrace.ethnicity.5levelBlack                             0.002694687 0.06208699
## Xrace.ethnicity.5levelMixed                             0.073203599 0.06471409
## Xrace.ethnicity.5levelOther                             0.015832572 0.04806535
## Xrace.ethnicity.5levelWhite                             0.112431933 0.08525560
## Xdemo_race_hispanic1                                    0.001809104 0.02964780
## Xinterview_age                                          0.002798632 0.02431281
## XMRI_minus_hormone_date_time                            0.026335395 0.02489377
## Xbmi                                                    0.008980047 0.02523287
## Xhousehold.income[>=200K]                              -0.187535979 0.06337130
## Xhousehold.income[100K-200K]                           -0.227561882 0.08319984
## Xhousehold.income[12K-16K]                             -0.011661282 0.03222053
## Xhousehold.income[16K-25K]                              0.011647426 0.03853811
## Xhousehold.income[25K-35K]                             -0.028656648 0.04106723
## Xhousehold.income[35K-50K]                             -0.030255036 0.04703856
## Xhousehold.income[50K-75K]                             -0.124677979 0.06107552
## Xhousehold.income[5K-12K]                              -0.001946748 0.03461195
## Xhousehold.income[75K-100K]                            -0.170460175 0.06415027
## Xhigh.educBachelor                                      0.089463373 0.07829837
## Xhigh.educHS Diploma/GED                               -0.050051657 0.04250562
## Xhigh.educPost Graduate Degree                          0.027100249 0.08612701
## Xhigh.educSome College                                  0.049194568 0.07142804
## Xhormone_scr_ert_mean:putamen_posvsneg_feedback_z -0.008358016 0.05397823
```

4.20 Model: CBCL internalizing factor ~ Testosterone x Lateral OFC activity (anticipation stage) + PDS

Female participants

```
## Warning: Some predictor variables are on very different scales: consider
## rescaling
```

```
##
## Family: gaussian
## Link function: identity
##
## Formula:
## cbcl_scr_syn_internal_r ~ PDS_score + hormone_sal_end_min_since_midnight +
##     hormone_scr_ert_mean * lOFC_rvsn_ant_z + race.ethnicity.5level +
##     demo_race_hispanic + interview_age + MRI_minus_hormone_date_time +
##     bmi + household.income + high.educ
##
## Parametric coefficients:
##                                      Estimate Std. Error t value Pr(>|t|)
## (Intercept)                        -3.576e-01  2.632e+00  -0.136 0.891931
## PDS_score                           7.860e-01  2.081e-01   3.777 0.000165 ***
## hormone_sal_end_min_since_midnight -1.128e-04  8.038e-04  -0.140 0.888419
## hormone_scr_ert_mean               -4.293e-03  8.278e-03  -0.519 0.604115
## lOFC_rvsn_ant_z                     6.104e-01  5.218e-01   1.170 0.242248
## race.ethnicity.5levelBlack         -7.362e-01  9.112e-01  -0.808 0.419225
## race.ethnicity.5levelMixed          9.211e-01  8.745e-01   1.053 0.292372
## race.ethnicity.5levelOther         -4.026e-01  1.057e+00  -0.381 0.703414
## race.ethnicity.5levelWhite          1.486e+00  8.081e-01   1.838 0.066195 .
```

```
## demo_race_hispanic1                 -2.115e-01  4.071e-01  -0.520 0.603430
## interview_age                        2.787e-02  1.833e-02   1.520 0.128701
## MRI_minus_hormone_date_time          4.028e-05  1.649e-05   2.443 0.014667 *
## bmi                                  5.615e-02  3.685e-02   1.524 0.127799
## household.income[>=200K]            -2.815e+00  1.006e+00  -2.797 0.005211 **
## household.income[100K-200K]         -1.934e+00  9.363e-01  -2.065 0.039040 *
## household.income[12K-16K]           -6.029e-01  1.170e+00  -0.515 0.606359
## household.income[16K-25K]           -1.797e+00  1.074e+00  -1.673 0.094437 .
## household.income[25K-35K]           -3.892e-01  9.778e-01  -0.398 0.690686
## household.income[35K-50K]           -1.382e+00  9.568e-01  -1.444 0.148919
## household.income[50K-75K]           -1.667e+00  9.413e-01  -1.771 0.076733 .
## household.income[5K-12K]            -8.864e-01  1.141e+00  -0.777 0.437233
## household.income[75K-100K]          -1.696e+00  9.467e-01  -1.791 0.073468 .
## high.educBachelor                    1.784e-01  8.990e-01   0.198 0.842708
## high.educHS Diploma/GED             -1.461e-01  9.125e-01  -0.160 0.872835
## high.educPost Graduate Degree        6.039e-01  9.139e-01   0.661 0.508835
## high.educSome College                7.079e-01  8.470e-01   0.836 0.403433
## hormone_scr_ert_mean:lOFC_rvsn_ant_z -9.843e-03  1.361e-02  -0.723 0.469561
## ---
## Signif. codes:  0 '***' 0.001 '**' 0.01 '*' 0.05 '.' 0.1 ' ' 1
##
##
## R-sq.(adj) =  0.0294
## lmer.REML =  10216  Scale est. = 15.996    n = 1661
```

```
##                                           stdcoef      stdse
## X(Intercept)                          0.000000000 0.00000000
## XPDS_score                            0.104746582 0.02773426
## Xhormone_sal_end_min_since_midnight  -0.003687643 0.02627883
## Xhormone_scr_ert_mean                -0.013494577 0.02602156
## XlOFC_rvsn_ant_z                      0.063432726 0.05422481
## Xrace.ethnicity.5levelBlack          -0.045639980 0.05648720
## Xrace.ethnicity.5levelMixed           0.056745812 0.05387572
## Xrace.ethnicity.5levelOther          -0.015356376 0.04032850
## Xrace.ethnicity.5levelWhite           0.129825103 0.07062113
## Xdemo_race_hispanic1                 -0.015339838 0.02952377
## Xinterview_age                        0.039176287 0.02577367
## XMRI_minus_hormone_date_time          0.061614882 0.02521989
## Xbmi                                  0.039691154 0.02605069
## Xhousehold.income[>=200K]            -0.168194580 0.06012462
## Xhousehold.income[100K-200K]         -0.170775785 0.08268316
## Xhousehold.income[12K-16K]           -0.017310467 0.03358796
## Xhousehold.income[16K-25K]           -0.062289719 0.03722310
## Xhousehold.income[25K-35K]           -0.017816369 0.04476538
## Xhousehold.income[35K-50K]           -0.072123932 0.04994592
## Xhousehold.income[50K-75K]           -0.105224325 0.05941246
## Xhousehold.income[5K-12K]            -0.026064076 0.03354171
## Xhousehold.income[75K-100K]          -0.116821517 0.06522467
## Xhigh.educBachelor                    0.014892711 0.07504037
## Xhigh.educHS Diploma/GED             -0.007164273 0.04475327
## Xhigh.educPost Graduate Degree        0.055078794 0.08335187
## Xhigh.educSome College                0.056297632 0.06736406
## Xhormone_scr_ert_mean:lOFC_rvsn_ant_z -0.039302819 0.05433350
```

Male participants

```
## Warning: Some predictor variables are on very different scales: consider
## rescaling

##
## Family: gaussian
## Link function: identity
##
## Formula:
## cbcl_scr_syn_internal_r ~ PDS_score + hormone_sal_end_min_since_midnight +
##     hormone_scr_ert_mean * lOFC_rvsn_ant_z + race.ethnicity.5level +
##     demo_race_hispanic + interview_age + MRI_minus_hormone_date_time +
##     bmi + household.income + high.educ
##
## Parametric coefficients:
##                                       Estimate Std. Error t value Pr(>|t|)
## (Intercept)                          2.338e+00  2.698e+00   0.867   0.3863
## PDS_score                            7.071e-01  2.849e-01   2.482   0.0132 *
## hormone_sal_end_min_since_midnight   6.298e-04  8.136e-04   0.774   0.4390
## hormone_scr_ert_mean                 7.931e-03  9.329e-03   0.850   0.3953
## lOFC_rvsn_ant_z                      3.156e-01  4.841e-01   0.652   0.5145
## race.ethnicity.5levelBlack          -1.390e-02  1.146e+00  -0.012   0.9903
## race.ethnicity.5levelMixed           1.250e+00  1.113e+00   1.124   0.2613
## race.ethnicity.5levelOther           4.802e-01  1.245e+00   0.386   0.6999
## race.ethnicity.5levelWhite           1.393e+00  1.051e+00   1.326   0.1850
## demo_race_hispanic1                  2.391e-02  4.254e-01   0.056   0.9552
## interview_age                        5.941e-03  1.758e-02   0.338   0.7354
## MRI_minus_hormone_date_time          2.295e-05  1.874e-05   1.224   0.2210
## bmi                                  3.536e-03  3.781e-02   0.093   0.9255
## household.income[>=200K]            -2.426e+00  1.044e+00  -2.323   0.0203 *
## household.income[100K-200K]         -2.000e+00  9.897e-01  -2.021   0.0435 *
## household.income[12K-16K]            1.615e-01  1.279e+00   0.126   0.8995
## household.income[16K-25K]            1.060e+00  1.087e+00   0.975   0.3295
## household.income[25K-35K]           -1.831e-01  1.067e+00  -0.172   0.8637
## household.income[35K-50K]           -7.989e-02  1.037e+00  -0.077   0.9386
## household.income[50K-75K]           -1.448e+00  9.880e-01  -1.465   0.1430
## household.income[5K-12K]             6.693e-01  1.137e+00   0.589   0.5560
## household.income[75K-100K]          -2.074e+00  1.007e+00  -2.059   0.0396 *
## high.educBachelor                    1.086e+00  9.544e-01   1.138   0.2554
## high.educHS Diploma/GED             -1.054e+00  9.944e-01  -1.060   0.2893
## high.educPost Graduate Degree        2.116e-01  9.571e-01   0.221   0.8251
## high.educSome College                5.627e-01  9.118e-01   0.617   0.5372
## hormone_scr_ert_mean:lOFC_rvsn_ant_z -1.418e-02  1.334e-02  -1.063   0.2881
## ---
## Signif. codes:  0 '***' 0.001 '**' 0.01 '*' 0.05 '.' 0.1 ' ' 1
##
##
## R-sq.(adj) =  0.0342
## lmer.REML =  10343  Scale est. = 13.518    n = 1668

##                                            stdcoef      stdse
## X(Intercept)                          0.0000000000 0.00000000
## XPDS_score                            0.0635357637 0.02559995
```

```
## Xhormone_sal_end_min_since_midnight    0.0203658800 0.02630882
## Xhormone_scr_ert_mean                  0.0213564043 0.02511965
## XlOFC_rvsn_ant_z                       0.0351253145 0.05387880
## Xrace.ethnicity.5levelBlack           -0.0007561635 0.06235132
## Xrace.ethnicity.5levelMixed            0.0731683973 0.06511948
## Xrace.ethnicity.5levelOther            0.0188659068 0.04893149
## Xrace.ethnicity.5levelWhite            0.1140575360 0.08600439
## Xdemo_race_hispanic1                   0.0016926342 0.03011215
## Xinterview_age                         0.0082468688 0.02440154
## XMRI_minus_hormone_date_time           0.0305979914 0.02498910
## Xbmi                                   0.0023694367 0.02534187
## Xhousehold.income[>=200K]             -0.1508702540 0.06494212
## Xhousehold.income[100K-200K]          -0.1716318367 0.08493992
## Xhousehold.income[12K-16K]             0.0040879983 0.03237153
## Xhousehold.income[16K-25K]             0.0383688925 0.03933353
## Xhousehold.income[25K-35K]            -0.0072369356 0.04215332
## Xhousehold.income[35K-50K]            -0.0037078429 0.04812370
## Xhousehold.income[50K-75K]            -0.0913805451 0.06235683
## Xhousehold.income[5K-12K]              0.0207416038 0.03521933
## Xhousehold.income[75K-100K]           -0.1353923860 0.06574764
## Xhigh.educBachelor                     0.0880595540 0.07740709
## Xhigh.educHS Diploma/GED              -0.0441724983 0.04167435
## Xhigh.educPost Graduate Degree         0.0188520680 0.08529288
## Xhigh.educSome College                 0.0435863506 0.07062695
## Xhormone_scr_ert_mean:lOFC_rvsn_ant_z -0.0571062415 0.05373913
```

4.21 Model: CBCL internalizing factor ~ Testosterone x Medial OFC activity (anticipation stage) + PDS

Female participants

```
## Warning: Some predictor variables are on very different scales: consider
## rescaling
```

```
##
## Family: gaussian
## Link function: identity
##
## Formula:
## cbcl_scr_syn_internal_r ~ PDS_score + hormone_sal_end_min_since_midnight +
##     hormone_scr_ert_mean * mOFC_rvsn_ant_z + race.ethnicity.5level +
##     demo_race_hispanic + interview_age + MRI_minus_hormone_date_time +
##     bmi + household.income + high.educ
##
## Parametric coefficients:
##                                      Estimate Std. Error t value Pr(>|t|)
## (Intercept)                         -5.152e-01  2.631e+00  -0.196 0.844796
## PDS_score                            7.833e-01  2.083e-01   3.760 0.000176 ***
## hormone_sal_end_min_since_midnight  -1.243e-04  8.037e-04  -0.155 0.877142
## hormone_scr_ert_mean                -3.450e-03  8.260e-03  -0.418 0.676204
## mOFC_rvsn_ant_z                      5.145e-01  4.421e-01   1.164 0.244700
## race.ethnicity.5levelBlack          -7.166e-01  9.106e-01  -0.787 0.431409
## race.ethnicity.5levelMixed           8.955e-01  8.751e-01   1.023 0.306322
```

```
## race.ethnicity.5levelOther           -4.112e-01  1.056e+00  -0.390 0.696916
## race.ethnicity.5levelWhite            1.512e+00  8.080e-01   1.871 0.061506 .
## demo_race_hispanic1                  -2.349e-01  4.073e-01  -0.577 0.564241
## interview_age                         2.814e-02  1.835e-02   1.533 0.125465
## MRI_minus_hormone_date_time           3.977e-05  1.648e-05   2.413 0.015912 *
## bmi                                   5.756e-02  3.685e-02   1.562 0.118485
## household.income[>=200K]             -2.924e+00  1.006e+00  -2.905 0.003719 **
## household.income[100K-200K]          -1.954e+00  9.363e-01  -2.087 0.037036 *
## household.income[12K-16K]            -7.172e-01  1.163e+00  -0.617 0.537600
## household.income[16K-25K]            -1.792e+00  1.073e+00  -1.670 0.095061 .
## household.income[25K-35K]            -3.777e-01  9.745e-01  -0.388 0.698376
## household.income[35K-50K]            -1.436e+00  9.568e-01  -1.501 0.133618
## household.income[50K-75K]            -1.705e+00  9.411e-01  -1.812 0.070152 .
## household.income[5K-12K]             -9.008e-01  1.141e+00  -0.790 0.429911
## household.income[75K-100K]           -1.735e+00  9.465e-01  -1.832 0.067062 .
## high.educBachelor                     3.065e-01  8.933e-01   0.343 0.731563
## high.educHS Diploma/GED              -2.041e-02  9.045e-01  -0.023 0.981997
## high.educPost Graduate Degree         7.162e-01  9.087e-01   0.788 0.430681
## high.educSome College                 7.948e-01  8.405e-01   0.946 0.344476
## hormone_scr_ert_mean:mOFC_rvsn_ant_z -7.698e-03  1.118e-02  -0.689 0.491147
## ---
## Signif. codes:  0 '***' 0.001 '**' 0.01 '*' 0.05 '.' 0.1 ' ' 1
##
##
## R-sq.(adj) =  0.0299
## lmer.REML =  10216  Scale est. = 15.823    n = 1661
```

```
##                                           stdcoef      stdse
## X(Intercept)                          0.000000000 0.00000000
## XPDS_score                            0.104543983 0.02780055
## Xhormone_sal_end_min_since_midnight  -0.004057655 0.02624318
## Xhormone_scr_ert_mean                -0.010853035 0.02598141
## XmOFC_rvsn_ant_z                      0.062329912 0.05356011
## Xrace.ethnicity.5levelBlack          -0.044514610 0.05656396
## Xrace.ethnicity.5levelMixed           0.055163530 0.05390795
## Xrace.ethnicity.5levelOther          -0.015787648 0.04052740
## Xrace.ethnicity.5levelWhite           0.132254229 0.07068147
## Xdemo_race_hispanic1                 -0.017054686 0.02957433
## Xinterview_age                        0.039474614 0.02574959
## XMRI_minus_hormone_date_time          0.060825339 0.02520265
## Xbmi                                  0.040700622 0.02605699
## Xhousehold.income[>=200K]            -0.174682968 0.06012713
## Xhousehold.income[100K-200K]         -0.172327460 0.08256871
## Xhousehold.income[12K-16K]           -0.020842081 0.03380292
## Xhousehold.income[16K-25K]           -0.062125378 0.03719522
## Xhousehold.income[25K-35K]           -0.017442051 0.04500217
## Xhousehold.income[35K-50K]           -0.074954807 0.04994552
## Xhousehold.income[50K-75K]           -0.107644831 0.05940291
## Xhousehold.income[5K-12K]            -0.026486124 0.03354625
## Xhousehold.income[75K-100K]          -0.119497517 0.06521077
## Xhigh.educBachelor                    0.025530327 0.07440945
## Xhigh.educHS Diploma/GED             -0.001004876 0.04452621
## Xhigh.educPost Graduate Degree        0.065301372 0.08284631
## Xhigh.educSome College                0.063318618 0.06695899
```

```
## Xhormone_scr_ert_mean:mOFC_rvsn_ant_z -0.036416732 0.05288203
```

Male participants

```
## Warning: Some predictor variables are on very different scales: consider
## rescaling

##
## Family: gaussian
## Link function: identity
##
## Formula:
## cbcl_scr_syn_internal_r ~ PDS_score + hormone_sal_end_min_since_midnight +
##     hormone_scr_ert_mean * mOFC_rvsn_ant_z + race.ethnicity.5level +
##     demo_race_hispanic + interview_age + MRI_minus_hormone_date_time +
##     bmi + household.income + high.educ
##
## Parametric coefficients:
##                                        Estimate Std. Error t value Pr(>|t|)
## (Intercept)                           2.703e+00  2.708e+00   0.998  0.31820
## PDS_score                             7.821e-01  2.850e-01   2.744  0.00613 **
## hormone_sal_end_min_since_midnight    4.927e-04  8.147e-04   0.605  0.54545
## hormone_scr_ert_mean                  7.250e-03  9.410e-03   0.770  0.44114
## mOFC_rvsn_ant_z                      -6.111e-02  4.207e-01  -0.145  0.88453
## race.ethnicity.5levelBlack            1.047e-01  1.152e+00   0.091  0.92760
## race.ethnicity.5levelMixed            1.222e+00  1.118e+00   1.093  0.27450
## race.ethnicity.5levelOther            5.220e-01  1.254e+00   0.416  0.67722
## race.ethnicity.5levelWhite            1.415e+00  1.057e+00   1.339  0.18070
## demo_race_hispanic1                   4.574e-03  4.264e-01   0.011  0.99144
## interview_age                         6.684e-03  1.767e-02   0.378  0.70523
## MRI_minus_hormone_date_time           2.166e-05  1.876e-05   1.154  0.24855
## bmi                                   5.221e-03  3.796e-02   0.138  0.89060
## household.income[>=200K]             -3.041e+00  1.036e+00  -2.935  0.00339 **
## household.income[100K-200K]          -2.658e+00  9.809e-01  -2.710  0.00680 **
## household.income[12K-16K]            -4.887e-01  1.255e+00  -0.390  0.69694
## household.income[16K-25K]             3.425e-01  1.077e+00   0.318  0.75050
## household.income[25K-35K]            -9.402e-01  1.060e+00  -0.887  0.37508
## household.income[35K-50K]            -6.699e-01  1.030e+00  -0.651  0.51534
## household.income[50K-75K]            -2.107e+00  9.775e-01  -2.155  0.03127 *
## household.income[5K-12K]              4.270e-02  1.131e+00   0.038  0.96988
## household.income[75K-100K]           -2.680e+00  9.984e-01  -2.685  0.00733 **
## high.educBachelor                     1.216e+00  9.582e-01   1.269  0.20461
## high.educHS Diploma/GED              -1.053e+00  9.958e-01  -1.057  0.29050
## high.educPost Graduate Degree         3.845e-01  9.609e-01   0.400  0.68912
## high.educSome College                 7.744e-01  9.137e-01   0.847  0.39685
## hormone_scr_ert_mean:mOFC_rvsn_ant_z -4.277e-03  1.189e-02  -0.360  0.71909
## ---
## Signif. codes:  0 '***' 0.001 '**' 0.01 '*' 0.05 '.' 0.1 ' ' 1
##
##
## R-sq.(adj) =  0.0332
## lmer.REML =  10402  Scale est. = 13.455    n = 1674

##                                            stdcoef      stdse
```

```
## X(Intercept)                          0.000000000 0.00000000
## XPDS_score                            0.070145617 0.02555960
## Xhormone_sal_end_min_since_midnight    0.015844699 0.02620180
## Xhormone_scr_ert_mean                 0.019369257 0.02514020
## XmOFC_rvsn_ant_z                     -0.007715799 0.05312078
## Xrace.ethnicity.5levelBlack           0.005701167 0.06273238
## Xrace.ethnicity.5levelMixed           0.071610801 0.06551051
## Xrace.ethnicity.5levelOther           0.020355788 0.04889339
## Xrace.ethnicity.5levelWhite           0.115576142 0.08630511
## Xdemo_race_hispanic1                  0.000321012 0.02992606
## Xinterview_age                        0.009201865 0.02432205
## XMRI_minus_hormone_date_time          0.028802919 0.02495284
## Xbmi                                  0.003484347 0.02532947
## Xhousehold.income[>=200K]            -0.187428629 0.06386806
## Xhousehold.income[100K-200K]         -0.226984391 0.08376461
## Xhousehold.income[12K-16K]           -0.012634421 0.03243547
## Xhousehold.income[16K-25K]            0.012388411 0.03895241
## Xhousehold.income[25K-35K]           -0.036666823 0.04132726
## Xhousehold.income[35K-50K]           -0.030737006 0.04723814
## Xhousehold.income[50K-75K]           -0.132480894 0.06146333
## Xhousehold.income[5K-12K]             0.001313206 0.03477240
## Xhousehold.income[75K-100K]          -0.173180258 0.06450498
## Xhigh.educBachelor                    0.098144370 0.07733752
## Xhigh.educHS Diploma/GED             -0.044231064 0.04183129
## Xhigh.educPost Graduate Degree        0.034025017 0.08503701
## Xhigh.educSome College                0.059716333 0.07046297
## Xhormone_scr_ert_mean:mOFC_rvsn_ant_z -0.019162481 0.05326820
```

4.22 Model: CBCL internalizing factor ~ Testosterone x Lateral OFC activity (feedback stage) + PDS

Female participants

```
## Warning: Some predictor variables are on very different scales: consider
## rescaling
```

```
##
## Family: gaussian
## Link function: identity
##
## Formula:
## cbcl_scr_syn_internal_r ~ PDS_score + hormone_sal_end_min_since_midnight +
##     hormone_scr_ert_mean * lOFC_posvsneg_feedback_z + race.ethnicity.5level +
##     demo_race_hispanic + interview_age + MRI_minus_hormone_date_time +
##     bmi + household.income + high.educ
##
## Parametric coefficients:
##                                             Estimate Std. Error t value
## (Intercept)                               -6.786e-01  2.622e+00  -0.259
## PDS_score                                  7.605e-01  2.078e-01   3.661
## hormone_sal_end_min_since_midnight        -1.261e-04  8.027e-04  -0.157
## hormone_scr_ert_mean                      -3.526e-03  8.277e-03  -0.426
## lOFC_posvsneg_feedback_z                   7.349e-02  5.803e-01   0.127
```

```
## race.ethnicity.5levelBlack                    -6.927e-01  9.109e-01  -0.760
## race.ethnicity.5levelMixed                     9.582e-01  8.729e-01   1.098
## race.ethnicity.5levelOther                    -6.081e-01  1.056e+00  -0.576
## race.ethnicity.5levelWhite                     1.471e+00  8.068e-01   1.824
## demo_race_hispanic1                           -2.006e-01  4.089e-01  -0.490
## interview_age                                  2.929e-02  1.833e-02   1.598
## MRI_minus_hormone_date_time                    4.161e-05  1.646e-05   2.528
## bmi                                            5.384e-02  3.675e-02   1.465
## household.income[>=200K]                      -2.683e+00  9.934e-01  -2.701
## household.income[100K-200K]                   -1.750e+00  9.228e-01  -1.897
## household.income[12K-16K]                     -5.499e-01  1.157e+00  -0.475
## household.income[16K-25K]                     -1.625e+00  1.061e+00  -1.531
## household.income[25K-35K]                    -1.229e-01  9.647e-01  -0.127
## household.income[35K-50K]                     -1.140e+00  9.448e-01  -1.206
## household.income[50K-75K]                     -1.490e+00  9.291e-01  -1.604
## household.income[5K-12K]                      -9.107e-01  1.131e+00  -0.805
## household.income[75K-100K]                    -1.536e+00  9.334e-01  -1.646
## high.educBachelor                              2.144e-01  8.760e-01   0.245
## high.educHS Diploma/GED                        3.681e-02  8.911e-01   0.041
## high.educPost Graduate Degree                  6.310e-01  8.909e-01   0.708
## high.educSome College                          7.253e-01  8.248e-01   0.879
## hormone_scr_ert_mean:lOFC_posvsneg_feedback_z -1.863e-03  1.539e-02  -0.121
##                                               Pr(>|t|)
## (Intercept)                                    0.79578
## PDS_score                                      0.00026 ***
## hormone_sal_end_min_since_midnight             0.87517
## hormone_scr_ert_mean                           0.67015
## lOFC_posvsneg_feedback_z                       0.89924
## race.ethnicity.5levelBlack                     0.44708
## race.ethnicity.5levelMixed                     0.27247
## race.ethnicity.5levelOther                     0.56467
## race.ethnicity.5levelWhite                     0.06837 .
## demo_race_hispanic1                            0.62386
## interview_age                                  0.11017
## MRI_minus_hormone_date_time                    0.01156 *
## bmi                                            0.14310
## household.income[>=200K]                       0.00699 **
## household.income[100K-200K]                    0.05806 .
## household.income[12K-16K]                      0.63451
## household.income[16K-25K]                      0.12592
## household.income[25K-35K]                      0.89867
## household.income[35K-50K]                      0.22790
## household.income[50K-75K]                      0.10884
## household.income[5K-12K]                       0.42097
## household.income[75K-100K]                     0.10004
## high.educBachelor                              0.80668
## high.educHS Diploma/GED                        0.96706
## high.educPost Graduate Degree                  0.47892
## high.educSome College                          0.37931
## hormone_scr_ert_mean:lOFC_posvsneg_feedback_z  0.90367
## ---
## Signif. codes:  0 '***' 0.001 '**' 0.01 '*' 0.05 '.' 0.1 ' ' 1
##
##
```

```
## R-sq.(adj) =  0.0273
## lmer.REML =  10243  Scale est. = 16.292    n = 1666
```

```
##                                                    stdcoef      stdse
## X(Intercept)                                   0.000000000 0.00000000
## XPDS_score                                     0.101559643 0.02774393
## Xhormone_sal_end_min_since_midnight           -0.004131081 0.02629279
## Xhormone_scr_ert_mean                         -0.011080969 0.02601086
## XlOFC_posvsneg_feedback_z                      0.006830691 0.05393776
## Xrace.ethnicity.5levelBlack                   -0.043068356 0.05663318
## Xrace.ethnicity.5levelMixed                    0.059325835 0.05404287
## Xrace.ethnicity.5levelOther                   -0.023209519 0.04029198
## Xrace.ethnicity.5levelWhite                    0.128952891 0.07070820
## Xdemo_race_hispanic1                          -0.014557893 0.02968077
## Xinterview_age                                 0.041240663 0.02580254
## XMRI_minus_hormone_date_time                   0.063719820 0.02520281
## Xbmi                                           0.038181333 0.02606162
## Xhousehold.income[>=200K]                     -0.160809143 0.05954018
## Xhousehold.income[100K-200K]                  -0.154911704 0.08167903
## Xhousehold.income[12K-16K]                    -0.015992434 0.03363468
## Xhousehold.income[16K-25K]                    -0.056816533 0.03710685
## Xhousehold.income[25K-35K]                    -0.005653660 0.04439061
## Xhousehold.income[35K-50K]                    -0.059348007 0.04920076
## Xhousehold.income[50K-75K]                    -0.093977319 0.05857871
## Xhousehold.income[5K-12K]                     -0.026795573 0.03328911
## Xhousehold.income[75K-100K]                   -0.105923894 0.06436915
## Xhigh.educBachelor                             0.017906419 0.07316054
## Xhigh.educHS Diploma/GED                       0.001806602 0.04373555
## Xhigh.educPost Graduate Degree                 0.057703472 0.08147807
## Xhigh.educSome College                         0.057694569 0.06560676
## Xhormone_scr_ert_mean:lOFC_posvsneg_feedback_z -0.006520132 0.05386592
```

Male participants

```
## Warning: Some predictor variables are on very different scales: consider
## rescaling
```

```
##
## Family: gaussian
## Link function: identity
##
## Formula:
## cbcl_scr_syn_internal_r ~ PDS_score + hormone_sal_end_min_since_midnight +
##     hormone_scr_ert_mean * lOFC_posvsneg_feedback_z + race.ethnicity.5level +
##     demo_race_hispanic + interview_age + MRI_minus_hormone_date_time +
##     bmi + household.income + high.educ
##
## Parametric coefficients:
##                                                 Estimate Std. Error t value
## (Intercept)                                    2.787e+00  2.705e+00   1.031
## PDS_score                                      7.408e-01  2.848e-01   2.601
## hormone_sal_end_min_since_midnight             7.373e-04  8.157e-04   0.904
## hormone_scr_ert_mean                           6.196e-03  9.377e-03   0.661
```

```
## lOFC_posvsneg_feedback_z                    1.096e-01  5.400e-01   0.203
## race.ethnicity.5levelBlack                  2.362e-02  1.148e+00   0.021
## race.ethnicity.5levelMixed                  1.273e+00  1.118e+00   1.139
## race.ethnicity.5levelOther                  3.950e-01  1.251e+00   0.316
## race.ethnicity.5levelWhite                  1.432e+00  1.056e+00   1.356
## demo_race_hispanic1                         7.979e-02  4.270e-01   0.187
## interview_age                               1.818e-03  1.766e-02   0.103
## MRI_minus_hormone_date_time                 2.230e-05  1.829e-05   1.219
## bmi                                         1.377e-02  3.807e-02   0.362
## household.income[>=200K]                   -2.829e+00  1.071e+00  -2.640
## household.income[100K-200K]                -2.430e+00  1.018e+00  -2.388
## household.income[12K-16K]                  -3.698e-01  1.290e+00  -0.287
## household.income[16K-25K]                   7.271e-01  1.122e+00   0.648
## household.income[25K-35K]                  -8.239e-01  1.100e+00  -0.749
## household.income[35K-50K]                  -5.168e-01  1.064e+00  -0.486
## household.income[50K-75K]                  -1.810e+00  1.017e+00  -1.780
## household.income[5K-12K]                    5.034e-01  1.155e+00   0.436
## household.income[75K-100K]                 -2.468e+00  1.035e+00  -2.385
## high.educBachelor                           1.219e+00  9.702e-01   1.257
## high.educHS Diploma/GED                    -8.273e-01  1.004e+00  -0.824
## high.educPost Graduate Degree               3.934e-01  9.726e-01   0.404
## high.educSome College                       7.829e-01  9.268e-01   0.845
## hormone_scr_ert_mean:lOFC_posvsneg_feedback_z -8.987e-04  1.521e-02  -0.059
##                                            Pr(>|t|)
## (Intercept)                                 0.30291
## PDS_score                                   0.00938 **
## hormone_sal_end_min_since_midnight          0.36621
## hormone_scr_ert_mean                        0.50882
## lOFC_posvsneg_feedback_z                    0.83921
## race.ethnicity.5levelBlack                  0.98358
## race.ethnicity.5levelMixed                  0.25490
## race.ethnicity.5levelOther                  0.75213
## race.ethnicity.5levelWhite                  0.17536
## demo_race_hispanic1                         0.85178
## interview_age                               0.91804
## MRI_minus_hormone_date_time                 0.22304
## bmi                                         0.71769
## household.income[>=200K]                    0.00836 **
## household.income[100K-200K]                 0.01706 *
## household.income[12K-16K]                   0.77444
## household.income[16K-25K]                   0.51695
## household.income[25K-35K]                   0.45396
## household.income[35K-50K]                   0.62731
## household.income[50K-75K]                   0.07525 .
## household.income[5K-12K]                    0.66291
## household.income[75K-100K]                  0.01720 *
## high.educBachelor                           0.20901
## high.educHS Diploma/GED                     0.40987
## high.educPost Graduate Degree               0.68593
## high.educSome College                       0.39840
## hormone_scr_ert_mean:lOFC_posvsneg_feedback_z  0.95288
## ---
## Signif. codes:  0 '***' 0.001 '**' 0.01 '*' 0.05 '.' 0.1 ' ' 1
##
```

```
##
## R-sq.(adj) =  0.0333
## lmer.REML =  10340  Scale est. = 13.487    n = 1665
```

```
##                                                    stdcoef      stdse
## X(Intercept)                                    0.000000000 0.00000000
## XPDS_score                                      0.066814104 0.02568905
## Xhormone_sal_end_min_since_midnight             0.023737089 0.02626255
## Xhormone_scr_ert_mean                           0.016612408 0.02513932
## XlOFC_posvsneg_feedback_z                       0.010798470 0.05321196
## Xrace.ethnicity.5levelBlack                     0.001277318 0.06205891
## Xrace.ethnicity.5levelMixed                     0.074080380 0.06504407
## Xrace.ethnicity.5levelOther                     0.015377485 0.04868102
## Xrace.ethnicity.5levelWhite                     0.116545600 0.08596318
## Xdemo_race_hispanic1                            0.005606457 0.03000074
## Xinterview_age                                  0.002511691 0.02440374
## XMRI_minus_hormone_date_time                    0.030527328 0.02504381
## Xbmi                                            0.009163431 0.02534108
## Xhousehold.income[>=200K]                      -0.174680268 0.06615635
## Xhousehold.income[100K-200K]                   -0.207851912 0.08704566
## Xhousehold.income[12K-16K]                     -0.009464088 0.03302026
## Xhousehold.income[16K-25K]                      0.025672491 0.03960632
## Xhousehold.income[25K-35K]                     -0.031883324 0.04256757
## Xhousehold.income[35K-50K]                     -0.023997428 0.04941662
## Xhousehold.income[50K-75K]                     -0.114431743 0.06428418
## Xhousehold.income[5K-12K]                       0.015695517 0.03600015
## Xhousehold.income[75K-100K]                    -0.160569272 0.06733177
## Xhigh.educBachelor                              0.098731927 0.07855870
## Xhigh.educHS Diploma/GED                       -0.034724034 0.04212367
## Xhigh.educPost Graduate Degree                  0.034890981 0.08626736
## Xhigh.educSome College                          0.060260926 0.07134014
## Xhormone_scr_ert_mean:lOFC_posvsneg_feedback_z -0.003150178 0.05330462
```

4.23 Model: CBCL internalizing factor ~ Testosterone x Medial OFC activity (feedback stage) + PDS

Female participants

```
## Warning: Some predictor variables are on very different scales: consider
## rescaling
```

```
##
## Family: gaussian
## Link function: identity
##
## Formula:
## cbcl_scr_syn_internal_r ~ PDS_score + hormone_sal_end_min_since_midnight +
##     hormone_scr_ert_mean * mOFC_posvsneg_feedback_z + race.ethnicity.5level +
##     demo_race_hispanic + interview_age + MRI_minus_hormone_date_time +
##     bmi + household.income + high.educ
##
## Parametric coefficients:
##                                                 Estimate Std. Error t value
```

```
## (Intercept)                                -6.142e-01  2.617e+00  -0.235
## PDS_score                                   7.911e-01  2.069e-01   3.824
## hormone_sal_end_min_since_midnight         -5.056e-05  8.017e-04  -0.063
## hormone_scr_ert_mean                       -3.903e-03  8.260e-03  -0.472
## mOFC_posvsneg_feedback_z                    3.936e-01  5.025e-01   0.783
## race.ethnicity.5levelBlack                 -6.661e-01  9.109e-01  -0.731
## race.ethnicity.5levelMixed                  9.851e-01  8.728e-01   1.129
## race.ethnicity.5levelOther                 -5.960e-01  1.056e+00  -0.564
## race.ethnicity.5levelWhite                  1.503e+00  8.072e-01   1.861
## demo_race_hispanic1                        -2.092e-01  4.078e-01  -0.513
## interview_age                               2.728e-02  1.827e-02   1.493
## MRI_minus_hormone_date_time                 4.078e-05  1.643e-05   2.482
## bmi                                         5.696e-02  3.668e-02   1.553
## household.income[>=200K]                   -2.661e+00  9.925e-01  -2.681
## household.income[100K-200K]                -1.704e+00  9.215e-01  -1.849
## household.income[12K-16K]                  -5.174e-01  1.154e+00  -0.448
## household.income[16K-25K]                  -1.642e+00  1.061e+00  -1.549
## household.income[25K-35K]                  -1.177e-01  9.637e-01  -0.122
## household.income[35K-50K]                  -1.178e+00  9.428e-01  -1.249
## household.income[50K-75K]                  -1.469e+00  9.280e-01  -1.583
## household.income[5K-12K]                   -9.146e-01  1.131e+00  -0.809
## household.income[75K-100K]                 -1.525e+00  9.331e-01  -1.635
## high.educBachelor                           1.956e-01  8.755e-01   0.223
## high.educHS Diploma/GED                     1.015e-02  8.910e-01   0.011
## high.educPost Graduate Degree               6.173e-01  8.906e-01   0.693
## high.educSome College                       7.275e-01  8.244e-01   0.883
## hormone_scr_ert_mean:mOFC_posvsneg_feedback_z -4.174e-03  1.327e-02  -0.315
##                                            Pr(>|t|)
## (Intercept)                                0.814439
## PDS_score                                  0.000136 ***
## hormone_sal_end_min_since_midnight         0.949727
## hormone_scr_ert_mean                       0.636645
## mOFC_posvsneg_feedback_z                   0.433582
## race.ethnicity.5levelBlack                 0.464763
## race.ethnicity.5levelMixed                 0.259182
## race.ethnicity.5levelOther                 0.572527
## race.ethnicity.5levelWhite                 0.062876 .
## demo_race_hispanic1                        0.607953
## interview_age                              0.135541
## MRI_minus_hormone_date_time                0.013168 *
## bmi                                        0.120659
## household.income[>=200K]                   0.007420 **
## household.income[100K-200K]                0.064593 .
## household.income[12K-16K]                  0.653978
## household.income[16K-25K]                  0.121655
## household.income[25K-35K]                  0.902848
## household.income[35K-50K]                  0.211811
## household.income[50K-75K]                  0.113684
## household.income[5K-12K]                   0.418873
## household.income[75K-100K]                 0.102275
## high.educBachelor                          0.823204
## high.educHS Diploma/GED                    0.990915
## high.educPost Graduate Degree              0.488311
## high.educSome College                      0.377611
```

```
## hormone_scr_ert_mean:mOFC_posvsneg_feedback_z 0.753118
## ---
## Signif. codes:  0 '***' 0.001 '**' 0.01 '*' 0.05 '.' 0.1 ' ' 1
##
##
## R-sq.(adj) =  0.029
## lmer.REML =  10273  Scale est. = 16.096    n = 1671
```

```
##                                                    stdcoef      stdse
## X(Intercept)                                    0.0000000000 0.00000000
## XPDS_score                                      0.1057678041 0.02765860
## Xhormone_sal_end_min_since_midnight            -0.0016544525 0.02623638
## Xhormone_scr_ert_mean                          -0.0122507765 0.02592853
## XmOFC_posvsneg_feedback_z                       0.0436755275 0.05576056
## Xrace.ethnicity.5levelBlack                    -0.0414175605 0.05664348
## Xrace.ethnicity.5levelMixed                     0.0610077576 0.05405059
## Xrace.ethnicity.5levelOther                    -0.0227023101 0.04022042
## Xrace.ethnicity.5levelWhite                     0.1316270698 0.07071680
## Xdemo_race_hispanic1                           -0.0151994818 0.02962310
## Xinterview_age                                  0.0383763109 0.02569836
## XMRI_minus_hormone_date_time                    0.0624253793 0.02515228
## Xbmi                                            0.0403650612 0.02599471
## Xhousehold.income[>=200K]                      -0.1591701204 0.05937602
## Xhousehold.income[100K-200K]                   -0.1506979211 0.08148892
## Xhousehold.income[12K-16K]                     -0.0150164570 0.03349457
## Xhousehold.income[16K-25K]                     -0.0573271540 0.03701720
## Xhousehold.income[25K-35K]                     -0.0054267195 0.04445075
## Xhousehold.income[35K-50K]                     -0.0616026015 0.04931805
## Xhousehold.income[50K-75K]                     -0.0927937212 0.05863038
## Xhousehold.income[5K-12K]                      -0.0268574568 0.03321550
## Xhousehold.income[75K-100K]                    -0.1048522710 0.06413621
## Xhigh.educBachelor                              0.0163372336 0.07310990
## Xhigh.educHS Diploma/GED                        0.0004970438 0.04364734
## Xhigh.educPost Graduate Degree                  0.0564086308 0.08137972
## Xhigh.educSome College                          0.0578838175 0.06558730
## Xhormone_scr_ert_mean:mOFC_posvsneg_feedback_z -0.0175650916 0.05583614
```

Male participants

```
## Warning: Some predictor variables are on very different scales: consider
## rescaling
```

```
##
## Family: gaussian
## Link function: identity
##
## Formula:
## cbcl_scr_syn_internal_r ~ PDS_score + hormone_sal_end_min_since_midnight +
##     hormone_scr_ert_mean * mOFC_posvsneg_feedback_z + race.ethnicity.5level +
##     demo_race_hispanic + interview_age + MRI_minus_hormone_date_time +
##     bmi + household.income + high.educ
##
## Parametric coefficients:
```

```
##                                             Estimate Std. Error t value
## (Intercept)                                2.674e+00  2.693e+00   0.993
## PDS_score                                  7.470e-01  2.837e-01   2.633
## hormone_sal_end_min_since_midnight         7.420e-04  8.123e-04   0.913
## hormone_scr_ert_mean                       7.391e-03  9.364e-03   0.789
## mOFC_posvsneg_feedback_z                  -5.372e-01  4.515e-01  -1.190
## race.ethnicity.5levelBlack                -7.376e-02  1.144e+00  -0.064
## race.ethnicity.5levelMixed                 1.274e+00  1.114e+00   1.144
## race.ethnicity.5levelOther                 3.890e-01  1.246e+00   0.312
## race.ethnicity.5levelWhite                 1.421e+00  1.053e+00   1.350
## demo_race_hispanic1                        6.035e-02  4.236e-01   0.142
## interview_age                              2.953e-03  1.760e-02   0.168
## MRI_minus_hormone_date_time                2.207e-05  1.876e-05   1.177
## bmi                                        1.444e-02  3.790e-02   0.381
## household.income[>=200K]                  -2.908e+00  1.062e+00  -2.739
## household.income[100K-200K]               -2.490e+00  1.008e+00  -2.471
## household.income[12K-16K]                 -3.400e-01  1.272e+00  -0.267
## household.income[16K-25K]                  6.533e-01  1.101e+00   0.594
## household.income[25K-35K]                 -8.746e-01  1.090e+00  -0.802
## household.income[35K-50K]                 -5.700e-01  1.055e+00  -0.540
## household.income[50K-75K]                 -1.847e+00  1.006e+00  -1.837
## household.income[5K-12K]                   4.606e-01  1.145e+00   0.402
## household.income[75K-100K]                -2.517e+00  1.025e+00  -2.456
## high.educBachelor                          1.207e+00  9.596e-01   1.258
## high.educHS Diploma/GED                   -8.384e-01  9.914e-01  -0.846
## high.educPost Graduate Degree              4.017e-01  9.621e-01   0.418
## high.educSome College                      7.868e-01  9.153e-01   0.860
## hormone_scr_ert_mean:mOFC_posvsneg_feedback_z  1.371e-02  1.276e-02   1.074
##                                           Pr(>|t|)
## (Intercept)                                0.32087
## PDS_score                                  0.00855 **
## hormone_sal_end_min_since_midnight         0.36116
## hormone_scr_ert_mean                       0.43006
## mOFC_posvsneg_feedback_z                   0.23431
## race.ethnicity.5levelBlack                 0.94860
## race.ethnicity.5levelMixed                 0.25294
## race.ethnicity.5levelOther                 0.75491
## race.ethnicity.5levelWhite                 0.17727
## demo_race_hispanic1                        0.88672
## interview_age                              0.86673
## MRI_minus_hormone_date_time                0.23953
## bmi                                        0.70329
## household.income[>=200K]                   0.00624 **
## household.income[100K-200K]                0.01358 *
## household.income[12K-16K]                  0.78923
## household.income[16K-25K]                  0.55285
## household.income[25K-35K]                  0.42257
## household.income[35K-50K]                  0.58895
## household.income[50K-75K]                  0.06646 .
## household.income[5K-12K]                   0.68756
## household.income[75K-100K]                 0.01413 *
## high.educBachelor                          0.20848
## high.educHS Diploma/GED                    0.39788
## high.educPost Graduate Degree              0.67634
```

```
## high.educSome College                        0.39016
## hormone_scr_ert_mean:mOFC_posvsneg_feedback_z  0.28284
## ---
## Signif. codes:  0 '***' 0.001 '**' 0.01 '*' 0.05 '.' 0.1 ' ' 1
##
##
## R-sq.(adj) =  0.0344
## lmer.REML =  10372  Scale est. = 13.478    n = 1671
```

```
##                                                   stdcoef      stdse
## X(Intercept)                                  0.000000000 0.00000000
## XPDS_score                                    0.067345545 0.02558020
## Xhormone_sal_end_min_since_midnight           0.023920122 0.02618752
## Xhormone_scr_ert_mean                         0.019833090 0.02512801
## XmOFC_posvsneg_feedback_z                    -0.062620980 0.05263249
## Xrace.ethnicity.5levelBlack                  -0.004008230 0.06216375
## Xrace.ethnicity.5levelMixed                   0.074435744 0.06508718
## Xrace.ethnicity.5levelOther                   0.015223826 0.04875839
## Xrace.ethnicity.5levelWhite                   0.116080203 0.08599897
## Xdemo_race_hispanic1                          0.004260754 0.02990523
## Xinterview_age                                0.004085447 0.02434111
## XMRI_minus_hormone_date_time                  0.029371361 0.02496337
## Xbmi                                          0.009625529 0.02526679
## Xhousehold.income[>=200K]                    -0.179506417 0.06554840
## Xhousehold.income[100K-200K]                 -0.213227326 0.08630051
## Xhousehold.income[12K-16K]                   -0.008821688 0.03299569
## Xhousehold.income[16K-25K]                    0.023550336 0.03967256
## Xhousehold.income[25K-35K]                   -0.033830005 0.04217300
## Xhousehold.income[35K-50K]                   -0.026458125 0.04895467
## Xhousehold.income[50K-75K]                   -0.116519316 0.06344580
## Xhousehold.income[5K-12K]                     0.014355743 0.03568948
## Xhousehold.income[75K-100K]                  -0.163743436 0.06665846
## Xhigh.educBachelor                            0.097775001 0.07770669
## Xhigh.educHS Diploma/GED                     -0.035346993 0.04179943
## Xhigh.educPost Graduate Degree                0.035652246 0.08538576
## Xhigh.educSome College                        0.060772750 0.07070320
## Xhormone_scr_ert_mean:mOFC_posvsneg_feedback_z  0.056631944 0.05271474
```

4.24 Model: CBCL internalizing factor ~ Testosterone x BIS-BAS RR + PDS

Female participants

```
##
## Family: gaussian
## Link function: identity
##
## Formula:
## cbcl_scr_syn_internal_r ~ PDS_score + hormone_sal_end_min_since_midnight +
##     hormone_scr_ert_mean * bisbas_ss_basm_rr + race.ethnicity.5level +
##     demo_race_hispanic + interview_age + bmi + household.income +
##     high.educ
##
## Parametric coefficients:
```

```
##                                         Estimate Std. Error t value Pr(>|t|)
## (Intercept)                           -2.2881141  2.4859836  -0.920 0.357463
## PDS_score                              0.6990737  0.1807471   3.868 0.000113
## hormone_sal_end_min_since_midnight     0.0005025  0.0007016   0.716 0.473926
## hormone_scr_ert_mean                   0.0355740  0.0271762   1.309 0.190669
## bisbas_ss_basm_rr                      0.1145067  0.1128078   1.015 0.310191
## race.ethnicity.5levelBlack            -0.8215660  0.8157206  -1.007 0.313968
## race.ethnicity.5levelMixed             1.0382209  0.7917347   1.311 0.189888
## race.ethnicity.5levelOther            -0.4027054  0.9344986  -0.431 0.666561
## race.ethnicity.5levelWhite             1.1360019  0.7349889   1.546 0.122347
## demo_race_hispanic1                   -0.0254809  0.3617656  -0.070 0.943854
## interview_age                          0.0293723  0.0162086   1.812 0.070103
## bmi                                    0.0467351  0.0324171   1.442 0.149537
## household.income[>=200K]              -2.8694103  0.8677004  -3.307 0.000959
## household.income[100K-200K]           -1.9970589  0.8044696  -2.482 0.013124
## household.income[12K-16K]             -0.5572779  1.0414608  -0.535 0.592641
## household.income[16K-25K]             -1.4534527  0.8964733  -1.621 0.105099
## household.income[25K-35K]             -0.4815663  0.8406688  -0.573 0.566815
## household.income[35K-50K]             -1.4876909  0.8175625  -1.820 0.068948
## household.income[50K-75K]             -1.5588882  0.8037028  -1.940 0.052554
## household.income[5K-12K]              -0.5648003  0.9240168  -0.611 0.541102
## household.income[75K-100K]            -1.7545814  0.8157066  -2.151 0.031587
## high.educBachelor                      1.2047441  0.7573489   1.591 0.111815
## high.educHS Diploma/GED                1.1642626  0.7608834   1.530 0.126127
## high.educPost Graduate Degree          1.6518817  0.7718419   2.140 0.032452
## high.educSome College                  1.5162082  0.7070525   2.144 0.032112
## hormone_scr_ert_mean:bisbas_ss_basm_rr -0.0045127  0.0029733  -1.518 0.129226
##
## (Intercept)
## PDS_score                             ***
## hormone_sal_end_min_since_midnight
## hormone_scr_ert_mean
## bisbas_ss_basm_rr
## race.ethnicity.5levelBlack
## race.ethnicity.5levelMixed
## race.ethnicity.5levelOther
## race.ethnicity.5levelWhite
## demo_race_hispanic1
## interview_age                         .
## bmi
## household.income[>=200K]              ***
## household.income[100K-200K]           *
## household.income[12K-16K]
## household.income[16K-25K]
## household.income[25K-35K]
## household.income[35K-50K]             .
## household.income[50K-75K]             .
## household.income[5K-12K]
## household.income[75K-100K]            *
## high.educBachelor
## high.educHS Diploma/GED
## high.educPost Graduate Degree         *
## high.educSome College                 *
## hormone_scr_ert_mean:bisbas_ss_basm_rr
```

```
## ---
## Signif. codes:  0 '***' 0.001 '**' 0.01 '*' 0.05 '.' 0.1 ' ' 1
##
##
## R-sq.(adj) =  0.0253
## lmer.REML =  13484  Scale est. = 17.482    n = 2187
```

```
##                                            stdcoef      stdse
## X(Intercept)                           0.000000000 0.00000000
## XPDS_score                             0.093059152 0.02406065
## Xhormone_sal_end_min_since_midnight    0.016408451 0.02290941
## Xhormone_scr_ert_mean                  0.108741152 0.08307109
## Xbisbas_ss_basm_rr                     0.050625992 0.04987486
## Xrace.ethnicity.5levelBlack           -0.053515989 0.05313522
## Xrace.ethnicity.5levelMixed            0.063037885 0.04807193
## Xrace.ethnicity.5levelOther           -0.015626297 0.03626163
## Xrace.ethnicity.5levelWhite            0.099781223 0.06455807
## Xdemo_race_hispanic1                  -0.001828068 0.02595403
## Xinterview_age                         0.040742011 0.02248282
## Xbmi                                   0.032937296 0.02284646
## Xhousehold.income[>=200K]             -0.167212031 0.05056438
## Xhousehold.income[100K-200K]          -0.173707144 0.06997396
## Xhousehold.income[12K-16K]            -0.015320787 0.02863203
## Xhousehold.income[16K-25K]            -0.053346512 0.03290353
## Xhousehold.income[25K-35K]            -0.021677075 0.03784160
## Xhousehold.income[35K-50K]            -0.078755712 0.04328030
## Xhousehold.income[50K-75K]            -0.099063571 0.05107336
## Xhousehold.income[5K-12K]             -0.018845871 0.03083196
## Xhousehold.income[75K-100K]           -0.117388027 0.05457381
## Xhigh.educBachelor                     0.099435944 0.06250929
## Xhigh.educHS Diploma/GED               0.058218374 0.03804760
## Xhigh.educPost Graduate Degree         0.148219715 0.06925568
## Xhigh.educSome College                 0.121149644 0.05649564
## Xhormone_scr_ert_mean:bisbas_ss_basm_rr -0.143813690 0.09475502
```

Male participants

```
##
## Family: gaussian
## Link function: identity
##
## Formula:
## cbcl_scr_syn_internal_r ~ PDS_score + hormone_sal_end_min_since_midnight +
##     hormone_scr_ert_mean * bisbas_ss_basm_rr + race.ethnicity.5level +
##     demo_race_hispanic + interview_age + bmi + household.income +
##     high.educ
##
## Parametric coefficients:
##                                       Estimate Std. Error t value Pr(>|t|)
## (Intercept)                          3.0700148  2.4497348   1.253 0.210257
## PDS_score                            0.6333701  0.2242306   2.825 0.004773
## hormone_sal_end_min_since_midnight   0.0012404  0.0006839   1.814 0.069827
## hormone_scr_ert_mean                 0.0310191  0.0295671   1.049 0.294236
```

```
## bisbas_ss_basm_rr                       0.0097928  0.1120956   0.087 0.930392
## race.ethnicity.5levelBlack             -0.6899456  0.8997476  -0.767 0.443265
## race.ethnicity.5levelMixed              1.0220473  0.8750018   1.168 0.242905
## race.ethnicity.5levelOther              0.1065064  0.9941366   0.107 0.914691
## race.ethnicity.5levelWhite              0.9929804  0.8232594   1.206 0.227879
## demo_race_hispanic1                    -0.0383609  0.3580065  -0.107 0.914678
## interview_age                           0.0033148  0.0150659   0.220 0.825873
## bmi                                     0.0209773  0.0313558   0.669 0.503556
## household.income[>=200K]               -3.2145911  0.8513929  -3.776 0.000164
## household.income[100K-200K]            -2.6523459  0.7969541  -3.328 0.000888
## household.income[12K-16K]              -0.2252179  1.0350648  -0.218 0.827769
## household.income[16K-25K]              -0.1541392  0.8546927  -0.180 0.856898
## household.income[25K-35K]              -0.7415642  0.8546364  -0.868 0.385650
## household.income[35K-50K]              -1.3023926  0.8133024  -1.601 0.109431
## household.income[50K-75K]              -1.8194922  0.7879161  -2.309 0.021017
## household.income[5K-12K]               -0.1888681  0.8817180  -0.214 0.830406
## household.income[75K-100K]             -2.7715519  0.8118699  -3.414 0.000652
## high.educBachelor                       1.1946798  0.7961832   1.501 0.133617
## high.educHS Diploma/GED                -0.8071015  0.7896952  -1.022 0.306867
## high.educPost Graduate Degree           0.4164022  0.8002683   0.520 0.602884
## high.educSome College                   0.7595378  0.7586181   1.001 0.316828
## hormone_scr_ert_mean:bisbas_ss_basm_rr -0.0032405  0.0032246  -1.005 0.315027
##
## (Intercept)
## PDS_score                              **
## hormone_sal_end_min_since_midnight     .
## hormone_scr_ert_mean
## bisbas_ss_basm_rr
## race.ethnicity.5levelBlack
## race.ethnicity.5levelMixed
## race.ethnicity.5levelOther
## race.ethnicity.5levelWhite
## demo_race_hispanic1
## interview_age
## bmi
## household.income[>=200K]               ***
## household.income[100K-200K]            ***
## household.income[12K-16K]
## household.income[16K-25K]
## household.income[25K-35K]
## household.income[35K-50K]
## household.income[50K-75K]              *
## household.income[5K-12K]
## household.income[75K-100K]             ***
## high.educBachelor
## high.educHS Diploma/GED
## high.educPost Graduate Degree
## high.educSome College
## hormone_scr_ert_mean:bisbas_ss_basm_rr
## ---
## Signif. codes:  0 '***' 0.001 '**' 0.01 '*' 0.05 '.' 0.1 ' ' 1
##
##
## R-sq.(adj) =  0.0341
```

```
## lmer.REML =  14711  Scale est. = 15.902    n = 2369

##                                            stdcoef      stdse
## X(Intercept)                            0.000000000 0.00000000
## XPDS_score                              0.061327058 0.02171148
## Xhormone_sal_end_min_since_midnight     0.040196153 0.02216053
## Xhormone_scr_ert_mean                   0.083633350 0.07971845
## Xbisbas_ss_basm_rr                      0.004054305 0.04640866
## Xrace.ethnicity.5levelBlack            -0.040056173 0.05223665
## Xrace.ethnicity.5levelMixed             0.060434571 0.05173964
## Xrace.ethnicity.5levelOther             0.004083985 0.03812016
## Xrace.ethnicity.5levelWhite             0.082646942 0.06852086
## Xdemo_race_hispanic1                   -0.002725647 0.02543733
## Xinterview_age                          0.004541811 0.02064249
## Xbmi                                    0.014320170 0.02140509
## Xhousehold.income[>=200K]              -0.192045925 0.05086387
## Xhousehold.income[100K-200K]           -0.223142445 0.06704792
## Xhousehold.income[12K-16K]             -0.005733171 0.02634872
## Xhousehold.income[16K-25K]             -0.005783425 0.03206875
## Xhousehold.income[25K-35K]             -0.029726246 0.03425884
## Xhousehold.income[35K-50K]             -0.064370055 0.04019703
## Xhousehold.income[50K-75K]             -0.114973396 0.04978828
## Xhousehold.income[5K-12K]              -0.006310667 0.02946092
## Xhousehold.income[75K-100K]            -0.178731369 0.05235573
## Xhigh.educBachelor                      0.095271849 0.06349304
## Xhigh.educHS Diploma/GED               -0.038736911 0.03790149
## Xhigh.educPost Graduate Degree          0.036435608 0.07002427
## Xhigh.educSome College                  0.059711999 0.05963969
## Xhormone_scr_ert_mean:bisbas_ss_basm_rr -0.089802562 0.08936072
```

4.25 Model: CBCL internalizing factor ~ Testosterone x MID Reaction Time + PDS (large reward vs. neutral)

Female participants

```
##
## Family: gaussian
## Link function: identity
##
## Formula:
## cbcl_scr_syn_internal_r ~ PDS_score + hormone_sal_end_min_since_midnight +
##     hormone_scr_ert_mean * rt_diff_large_neutral_z + race.ethnicity.5level +
##     demo_race_hispanic + interview_age + bmi + household.income +
##     high.educ
##
## Parametric coefficients:
##                                              Estimate Std. Error t value
## (Intercept)                                -5.772e-01  2.483e+00  -0.232
## PDS_score                                   8.920e-01  1.992e-01   4.478
## hormone_sal_end_min_since_midnight         -3.517e-05  7.567e-04  -0.046
## hormone_scr_ert_mean                       -5.736e-03  8.069e-03  -0.711
## rt_diff_large_neutral_z                    -1.598e-01  3.041e-01  -0.526
## race.ethnicity.5levelBlack                 -1.099e+00  8.703e-01  -1.263
```

```
## race.ethnicity.5levelMixed                       5.522e-01  8.380e-01   0.659
## race.ethnicity.5levelOther                      -7.529e-01  9.843e-01  -0.765
## race.ethnicity.5levelWhite                       1.139e+00  7.764e-01   1.467
## demo_race_hispanic1                             -1.130e-01  3.896e-01  -0.290
## interview_age                                    2.918e-02  1.758e-02   1.660
## bmi                                              4.115e-02  3.480e-02   1.183
## household.income[>=200K]                        -2.402e+00  9.466e-01  -2.538
## household.income[100K-200K]                     -1.540e+00  8.797e-01  -1.751
## household.income[12K-16K]                       -4.218e-01  1.126e+00  -0.375
## household.income[16K-25K]                      -1.289e+00  9.864e-01  -1.307
## household.income[25K-35K]                       1.575e-01  9.182e-01   0.171
## household.income[35K-50K]                      -9.507e-01  8.922e-01  -1.066
## household.income[50K-75K]                      -1.323e+00  8.807e-01  -1.503
## household.income[5K-12K]                       -1.639e-01  1.066e+00  -0.154
## household.income[75K-100K]                     -1.372e+00  8.934e-01  -1.536
## high.educBachelor                                3.206e-01  8.244e-01   0.389
## high.educHS Diploma/GED                          4.006e-01  8.373e-01   0.478
## high.educPost Graduate Degree                    8.279e-01  8.396e-01   0.986
## high.educSome College                            9.495e-01  7.742e-01   1.227
## hormone_scr_ert_mean:rt_diff_large_neutral_z  1.016e-02  7.768e-03   1.307
##                                                 Pr(>|t|)
## (Intercept)                                       0.8162
## PDS_score                                       8.01e-06 ***
## hormone_sal_end_min_since_midnight                0.9629
## hormone_scr_ert_mean                              0.4773
## rt_diff_large_neutral_z                           0.5993
## race.ethnicity.5levelBlack                        0.2068
## race.ethnicity.5levelMixed                        0.5100
## race.ethnicity.5levelOther                        0.4444
## race.ethnicity.5levelWhite                        0.1425
## demo_race_hispanic1                               0.7717
## interview_age                                     0.0971 .
## bmi                                               0.2371
## household.income[>=200K]                          0.0112 *
## household.income[100K-200K]                       0.0801 .
## household.income[12K-16K]                         0.7079
## household.income[16K-25K]                         0.1914
## household.income[25K-35K]                         0.8639
## household.income[35K-50K]                         0.2868
## household.income[50K-75K]                         0.1331
## household.income[5K-12K]                          0.8778
## household.income[75K-100K]                        0.1247
## high.educBachelor                                 0.6974
## high.educHS Diploma/GED                           0.6324
## high.educPost Graduate Degree                     0.3242
## high.educSome College                             0.2202
## hormone_scr_ert_mean:rt_diff_large_neutral_z   0.1912
## ---
## Signif. codes:  0 '***' 0.001 '**' 0.01 '*' 0.05 '.' 0.1 ' ' 1
##
##
## R-sq.(adj) =  0.031
## lmer.REML =  11329  Scale est. = 16.647    n = 1843
```

```
##                                                    stdcoef      stdse
## X(Intercept)                                   0.000000000 0.00000000
## XPDS_score                                     0.117077632 0.02614633
## Xhormone_sal_end_min_since_midnight           -0.001152505 0.02479984
## Xhormone_scr_ert_mean                         -0.017592135 0.02474815
## Xrt_diff_large_neutral_z                      -0.027686587 0.05268142
## Xrace.ethnicity.5levelBlack                   -0.068608582 0.05433203
## Xrace.ethnicity.5levelMixed                    0.034094540 0.05174338
## Xrace.ethnicity.5levelOther                   -0.030099097 0.03934931
## Xrace.ethnicity.5levelWhite                    0.099868864 0.06807508
## Xdemo_race_hispanic1                          -0.008204901 0.02827822
## Xinterview_age                                 0.040500830 0.02439684
## Xbmi                                           0.029125839 0.02462504
## Xhousehold.income[>=200K]                     -0.142935430 0.05631931
## Xhousehold.income[100K-200K]                  -0.135438021 0.07735628
## Xhousehold.income[12K-16K]                    -0.011600825 0.03096065
## Xhousehold.income[16K-25K]                    -0.046895388 0.03587792
## Xhousehold.income[25K-35K]                     0.007130854 0.04158204
## Xhousehold.income[35K-50K]                    -0.050479931 0.04737427
## Xhousehold.income[50K-75K]                    -0.083501059 0.05556474
## Xhousehold.income[5K-12K]                     -0.004869232 0.03166977
## Xhousehold.income[75K-100K]                   -0.091989757 0.05988925
## Xhigh.educBachelor                             0.026648234 0.06852335
## Xhigh.educHS Diploma/GED                       0.019529276 0.04082059
## Xhigh.educPost Graduate Degree                 0.074936092 0.07598699
## Xhigh.educSome College                         0.075545764 0.06159447
## Xhormone_scr_ert_mean:rt_diff_large_neutral_z  0.068770830 0.05260059
```

Male participants

```
##
## Family: gaussian
## Link function: identity
##
## Formula:
## cbcl_scr_syn_internal_r ~ PDS_score + hormone_sal_end_min_since_midnight +
##     hormone_scr_ert_mean * rt_diff_large_neutral_z + race.ethnicity.5level +
##     demo_race_hispanic + interview_age + bmi + household.income +
##     high.educ
##
## Parametric coefficients:
##                                                Estimate Std. Error t value
## (Intercept)                                   1.4491934  2.5474438   0.569
## PDS_score                                     0.8272036  0.2579718   3.207
## hormone_sal_end_min_since_midnight            0.0012260  0.0007532   1.628
## hormone_scr_ert_mean                          0.0071055  0.0087393   0.813
## rt_diff_large_neutral_z                      -0.0990673  0.3162520  -0.313
## race.ethnicity.5levelBlack                   -0.6455596  1.0930777  -0.591
## race.ethnicity.5levelMixed                    0.7692020  1.0685583   0.720
## race.ethnicity.5levelOther                   -0.0886980  1.1854223  -0.075
## race.ethnicity.5levelWhite                    0.8919913  1.0106820   0.883
## demo_race_hispanic1                          -0.0063952  0.3983118  -0.016
## interview_age                                 0.0036095  0.0165850   0.218
```

```
## bmi                                           0.0202513  0.0355351   0.570
## household.income[>=200K]                     -2.0381157  0.9758745  -2.089
## household.income[100K-200K]                  -1.6831721  0.9220691  -1.825
## household.income[12K-16K]                     0.9724012  1.1672111   0.833
## household.income[16K-25K]                     1.4242683  1.0083455   1.412
## household.income[25K-35K]                     0.2867102  0.9927782   0.289
## household.income[35K-50K]                     0.3022748  0.9461961   0.319
## household.income[50K-75K]                    -0.9179342  0.9157934  -1.002
## household.income[5K-12K]                      0.8969878  1.0434052   0.860
## household.income[75K-100K]                   -1.6312908  0.9381027  -1.739
## high.educBachelor                             1.4928792  0.9160442   1.630
## high.educHS Diploma/GED                      -0.0754513  0.9292278  -0.081
## high.educPost Graduate Degree                 0.7462380  0.9168771   0.814
## high.educSome College                         0.9084777  0.8715841   1.042
## hormone_scr_ert_mean:rt_diff_large_neutral_z  0.0013216  0.0090710   0.146
##                                              Pr(>|t|)
## (Intercept)                                   0.56950
## PDS_score                                     0.00137 **
## hormone_sal_end_min_since_midnight            0.10376
## hormone_scr_ert_mean                          0.41629
## rt_diff_large_neutral_z                       0.75412
## race.ethnicity.5levelBlack                    0.55487
## race.ethnicity.5levelMixed                    0.47171
## race.ethnicity.5levelOther                    0.94036
## race.ethnicity.5levelWhite                    0.37758
## demo_race_hispanic1                           0.98719
## interview_age                                 0.82773
## bmi                                           0.56882
## household.income[>=200K]                      0.03689 *
## household.income[100K-200K]                   0.06810 .
## household.income[12K-16K]                     0.40490
## household.income[16K-25K]                     0.15797
## household.income[25K-35K]                     0.77277
## household.income[35K-50K]                     0.74941
## household.income[50K-75K]                     0.31631
## household.income[5K-12K]                      0.39008
## household.income[75K-100K]                    0.08221 .
## high.educBachelor                             0.10333
## high.educHS Diploma/GED                       0.93529
## high.educPost Graduate Degree                 0.41581
## high.educSome College                         0.29739
## hormone_scr_ert_mean:rt_diff_large_neutral_z  0.88418
## ---
## Signif. codes:  0 '***' 0.001 '**' 0.01 '*' 0.05 '.' 0.1 ' ' 1
##
##
## R-sq.(adj) =  0.0337
## lmer.REML =  11805  Scale est. = 12.697    n = 1904

##                                                     stdcoef      stdse
## X(Intercept)                                   0.0000000000 0.00000000
## XPDS_score                                     0.0774135355 0.02414220
## Xhormone_sal_end_min_since_midnight            0.0396701359 0.02437190
## Xhormone_scr_ert_mean                          0.0191104178 0.02350451
```

```
## Xrt_diff_large_neutral_z                        -0.0164892275 0.05263847
## Xrace.ethnicity.5levelBlack                     -0.0357446050 0.06052366
## Xrace.ethnicity.5levelMixed                      0.0449409732 0.06243100
## Xrace.ethnicity.5levelOther                     -0.0034067416 0.04553008
## Xrace.ethnicity.5levelWhite                      0.0729314741 0.08263593
## Xdemo_race_hispanic1                            -0.0004503044 0.02804633
## Xinterview_age                                   0.0049831711 0.02289663
## Xbmi                                             0.0135409712 0.02376048
## Xhousehold.income[>=200K]                       -0.1239435790 0.05934569
## Xhousehold.income[100K-200K]                    -0.1431088750 0.07839738
## Xhousehold.income[12K-16K]                       0.0249288741 0.02992310
## Xhousehold.income[16K-25K]                       0.0514070005 0.03639484
## Xhousehold.income[25K-35K]                       0.0112416936 0.03892610
## Xhousehold.income[35K-50K]                       0.0147831436 0.04627496
## Xhousehold.income[50K-75K]                      -0.0578671407 0.05773219
## Xhousehold.income[5K-12K]                        0.0281317142 0.03272372
## Xhousehold.income[75K-100K]                     -0.1059380619 0.06092156
## Xhigh.educBachelor                               0.1188991321 0.07295758
## Xhigh.educHS Diploma/GED                        -0.0033313505 0.04102759
## Xhigh.educPost Graduate Degree                   0.0661862719 0.08132081
## Xhigh.educSome College                           0.0707945558 0.06791957
## Xhormone_scr_ert_mean:rt_diff_large_neutral_z  0.0076911529 0.05279068
```

4.26 Model: CBCL internalizing factor ~ Testosterone x MID Reaction Time + PDS (large vs. small reward)

Female participants

```
##
## Family: gaussian
## Link function: identity
##
## Formula:
## cbcl_scr_syn_internal_r ~ PDS_score + hormone_sal_end_min_since_midnight +
##     hormone_scr_ert_mean * rt_diff_large_small_z + race.ethnicity.5level +
##     demo_race_hispanic + interview_age + bmi + household.income +
##     high.educ
##
## Parametric coefficients:
##                                      Estimate Std. Error t value
## (Intercept)                        -5.450e-01  2.484e+00  -0.219
## PDS_score                           8.954e-01  1.992e-01   4.494
## hormone_sal_end_min_since_midnight -7.833e-05  7.564e-04  -0.104
## hormone_scr_ert_mean               -5.047e-03  8.059e-03  -0.626
## rt_diff_large_small_z               2.202e-02  2.861e-01   0.077
## race.ethnicity.5levelBlack         -1.075e+00  8.705e-01  -1.235
## race.ethnicity.5levelMixed          5.787e-01  8.384e-01   0.690
## race.ethnicity.5levelOther         -7.290e-01  9.845e-01  -0.740
## race.ethnicity.5levelWhite          1.165e+00  7.763e-01   1.501
## demo_race_hispanic1                -1.366e-01  3.898e-01  -0.350
## interview_age                       2.953e-02  1.757e-02   1.681
## bmi                                 3.902e-02  3.483e-02   1.120
## household.income[>=200K]           -2.452e+00  9.469e-01  -2.589
```

```
## household.income[100K-200K]              -1.601e+00  8.794e-01  -1.821
## household.income[12K-16K]                -4.366e-01  1.126e+00  -0.388
## household.income[16K-25K]                -1.353e+00  9.867e-01  -1.372
## household.income[25K-35K]                 1.457e-01  9.180e-01   0.159
## household.income[35K-50K]                -1.012e+00  8.924e-01  -1.134
## household.income[50K-75K]                -1.375e+00  8.809e-01  -1.561
## household.income[5K-12K]                 -1.671e-01  1.067e+00  -0.157
## household.income[75K-100K]               -1.433e+00  8.933e-01  -1.605
## high.educBachelor                         3.350e-01  8.243e-01   0.406
## high.educHS Diploma/GED                   3.851e-01  8.377e-01   0.460
## high.educPost Graduate Degree             8.283e-01  8.405e-01   0.986
## high.educSome College                     9.683e-01  7.745e-01   1.250
## hormone_scr_ert_mean:rt_diff_large_small_z  6.434e-03  7.626e-03   0.844
##                                          Pr(>|t|)
## (Intercept)                                0.8263
## PDS_score                                7.42e-06 ***
## hormone_sal_end_min_since_midnight         0.9175
## hormone_scr_ert_mean                       0.5312
## rt_diff_large_small_z                      0.9387
## race.ethnicity.5levelBlack                 0.2169
## race.ethnicity.5levelMixed                 0.4901
## race.ethnicity.5levelOther                 0.4591
## race.ethnicity.5levelWhite                 0.1336
## demo_race_hispanic1                        0.7261
## interview_age                              0.0929 .
## bmi                                        0.2627
## household.income[>=200K]                   0.0097 **
## household.income[100K-200K]                0.0688 .
## household.income[12K-16K]                  0.6981
## household.income[16K-25K]                  0.1703
## household.income[25K-35K]                  0.8739
## household.income[35K-50K]                  0.2569
## household.income[50K-75K]                  0.1187
## household.income[5K-12K]                   0.8755
## household.income[75K-100K]                 0.1088
## high.educBachelor                          0.6845
## high.educHS Diploma/GED                    0.6458
## high.educPost Graduate Degree              0.3245
## high.educSome College                      0.2114
## hormone_scr_ert_mean:rt_diff_large_small_z   0.3989
## ---
## Signif. codes:  0 '***' 0.001 '**' 0.01 '*' 0.05 '.' 0.1 ' ' 1
##
##
## R-sq.(adj) =  0.0315
## lmer.REML =  11329  Scale est. = 16.636    n = 1843

##                                              stdcoef      stdse
## X(Intercept)                              0.000000000 0.00000000
## XPDS_score                                0.117534000 0.02615223
## Xhormone_sal_end_min_since_midnight      -0.002567061 0.02478714
## Xhormone_scr_ert_mean                    -0.015480570 0.02471752
## Xrt_diff_large_small_z                    0.003976742 0.05167000
## Xrace.ethnicity.5levelBlack              -0.067117095 0.05433948
```

```
## Xrace.ethnicity.5levelMixed                 0.035731363 0.05176827
## Xrace.ethnicity.5levelOther                -0.029143515 0.03935950
## Xrace.ethnicity.5levelWhite                 0.102141185 0.06806825
## Xdemo_race_hispanic1                       -0.009913033 0.02829184
## Xinterview_age                              0.040989615 0.02438027
## Xbmi                                        0.027613401 0.02464817
## Xhousehold.income[>=200K]                  -0.145870296 0.05633914
## Xhousehold.income[100K-200K]               -0.140781047 0.07733092
## Xhousehold.income[12K-16K]                 -0.012008415 0.03095813
## Xhousehold.income[16K-25K]                 -0.049228650 0.03588798
## Xhousehold.income[25K-35K]                  0.006599697 0.04157680
## Xhousehold.income[35K-50K]                 -0.053738480 0.04738205
## Xhousehold.income[50K-75K]                 -0.086760785 0.05557737
## Xhousehold.income[5K-12K]                  -0.004964232 0.03168658
## Xhousehold.income[75K-100K]                -0.096086507 0.05988096
## Xhigh.educBachelor                          0.027845313 0.06851535
## Xhigh.educHS Diploma/GED                    0.018777254 0.04084244
## Xhigh.educPost Graduate Degree              0.074971309 0.07607300
## Xhigh.educSome College                      0.077037894 0.06162080
## Xhormone_scr_ert_mean:rt_diff_large_small_z  0.043645635 0.05172939
```

Male participants

```
##
## Family: gaussian
## Link function: identity
##
## Formula:
## cbcl_scr_syn_internal_r ~ PDS_score + hormone_sal_end_min_since_midnight +
##     hormone_scr_ert_mean * rt_diff_large_small_z + race.ethnicity.5level +
##     demo_race_hispanic + interview_age + bmi + household.income +
##     high.educ
##
## Parametric coefficients:
##                                              Estimate Std. Error t value
## (Intercept)                                 1.4989448  2.5458407   0.589
## PDS_score                                   0.8301503  0.2582905   3.214
## hormone_sal_end_min_since_midnight          0.0012165  0.0007536   1.614
## hormone_scr_ert_mean                        0.0070965  0.0087423   0.812
## rt_diff_large_small_z                       0.0172049  0.3187302   0.054
## race.ethnicity.5levelBlack                 -0.6407558  1.0929680  -0.586
## race.ethnicity.5levelMixed                  0.7657586  1.0678277   0.717
## race.ethnicity.5levelOther                 -0.0797751  1.1851105  -0.067
## race.ethnicity.5levelWhite                  0.8962683  1.0099723   0.887
## demo_race_hispanic1                        -0.0037019  0.3986543  -0.009
## interview_age                               0.0032499  0.0165670   0.196
## bmi                                         0.0200622  0.0355305   0.565
## household.income[>=200K]                   -2.0481496  0.9757093  -2.099
## household.income[100K-200K]                -1.6913308  0.9225889  -1.833
## household.income[12K-16K]                   0.9687013  1.1673428   0.830
## household.income[16K-25K]                   1.4190042  1.0099180   1.405
## household.income[25K-35K]                   0.2792207  0.9940798   0.281
## household.income[35K-50K]                   0.2925003  0.9458280   0.309
```

```
## household.income[50K-75K]                 -0.9274208  0.9154502  -1.013
## household.income[5K-12K]                   0.8966287  1.0435544   0.859
## household.income[75K-100K]                -1.6395871  0.9385216  -1.747
## high.educBachelor                          1.4946722  0.9154577   1.633
## high.educHS Diploma/GED                    -0.0670181  0.9280507  -0.072
## high.educPost Graduate Degree              0.7522253  0.9163482   0.821
## high.educSome College                      0.9148788  0.8705160   1.051
## hormone_scr_ert_mean:rt_diff_large_small_z -0.0006893  0.0093227  -0.074
##                                            Pr(>|t|)
## (Intercept)                                 0.55608
## PDS_score                                   0.00133 **
## hormone_sal_end_min_since_midnight          0.10662
## hormone_scr_ert_mean                        0.41705
## rt_diff_large_small_z                       0.95696
## race.ethnicity.5levelBlack                  0.55778
## race.ethnicity.5levelMixed                  0.47339
## race.ethnicity.5levelOther                  0.94634
## race.ethnicity.5levelWhite                  0.37497
## demo_race_hispanic1                         0.99259
## interview_age                               0.84450
## bmi                                         0.57238
## household.income[>=200K]                    0.03594 *
## household.income[100K-200K]                 0.06692 .
## household.income[12K-16K]                   0.40674
## household.income[16K-25K]                   0.16017
## household.income[25K-35K]                   0.77883
## household.income[35K-50K]                   0.75716
## household.income[50K-75K]                   0.31115
## household.income[5K-12K]                    0.39034
## household.income[75K-100K]                  0.08080 .
## high.educBachelor                           0.10270
## high.educHS Diploma/GED                     0.94244
## high.educPost Graduate Degree               0.41181
## high.educSome College                       0.29341
## hormone_scr_ert_mean:rt_diff_large_small_z  0.94107
## ---
## Signif. codes:  0 '***' 0.001 '**' 0.01 '*' 0.05 '.' 0.1 ' ' 1
##
##
## R-sq.(adj) =  0.0336
## lmer.REML =  11805  Scale est. = 12.694    n = 1904
```

```
##                                                  stdcoef      stdse
## X(Intercept)                              0.0000000000 0.00000000
## XPDS_score                                0.0776893021 0.02417202
## Xhormone_sal_end_min_since_midnight       0.0393629908 0.02438324
## Xhormone_scr_ert_mean                     0.0190860990 0.02351277
## Xrt_diff_large_small_z                    0.0028234074 0.05230519
## Xrace.ethnicity.5levelBlack              -0.0354786208 0.06051759
## Xrace.ethnicity.5levelMixed               0.0447397906 0.06238831
## Xrace.ethnicity.5levelOther              -0.0030640267 0.04551811
## Xrace.ethnicity.5levelWhite               0.0732811735 0.08257790
## Xdemo_race_hispanic1                     -0.0002606616 0.02807045
## Xinterview_age                            0.0044867315 0.02287172
```

```
## Xbmi                                         0.0134145728 0.02375740
## Xhousehold.income[>=200K]                   -0.1245537650 0.05933564
## Xhousehold.income[100K-200K]                -0.1438025608 0.07844157
## Xhousehold.income[12K-16K]                   0.0248340228 0.02992648
## Xhousehold.income[16K-25K]                   0.0512169981 0.03645160
## Xhousehold.income[25K-35K]                   0.0109480371 0.03897713
## Xhousehold.income[35K-50K]                   0.0143051115 0.04625695
## Xhousehold.income[50K-75K]                  -0.0584651857 0.05771055
## Xhousehold.income[5K-12K]                    0.0281204498 0.03272840
## Xhousehold.income[75K-100K]                 -0.1064768382 0.06094877
## Xhigh.educBachelor                           0.1190419305 0.07291087
## Xhigh.educHS Diploma/GED                    -0.0029590052 0.04097562
## Xhigh.educPost Graduate Degree               0.0667173065 0.08127390
## Xhigh.educSome College                       0.0712933728 0.06783633
## Xhormone_scr_ert_mean:rt_diff_large_small_z -0.0038713261 0.05235798
```
